# Effect of feeding raw potato starch on the composition dynamics of the piglet intestinal microbiome

**DOI:** 10.5713/ab.22.0045

**Published:** 2022-09-02

**Authors:** Seung-Won Yi, Han Gyu Lee, Kyoung-Min So, Eunju Kim, Young-Hun Jung, Minji Kim, Jin Young Jeong, Ki Hyun Kim, Jae-Ku Oem, Tai-Young Hur, Sang-Ik Oh

**Affiliations:** 1 Division of Animal Diseases & Health, National Institute of Animal Science, Rural Development Administration, Wanju 55365, Korea; 2 Laboratory of Veterinary Infectious Disease, College of Veterinary Medicine, Jeonbuk National University, Iksan 54596, Korea; 3 Animal Nutrition and Physiology Division, National Institute of Animal Science, Rural Development Administration, Wanju 55365, Korea; 4 Animal Welfare Research Team, National Institute of Animal Science, Rural Development Administration, Wanju 55365, Korea

**Keywords:** Feed, Metagenomics, Microbiome, Piglet, Resistant Starch, Short Chain Fatty Acids

## Abstract

**Objective:**

Raw potato starch (RPS) is resistant to digestion, escapes absorption, and is metabolized by intestinal microflora in the large intestine and acts as their energy source. In this study, we compared the effect of different concentrations of RPS on the intestinal bacterial community of weaned piglets.

**Methods:**

Male weaned piglets (25-days-old, 7.03±0.49 kg) were either fed a corn/soybean-based control diet (CON, n = 6) or two treatment diets supplemented with 5% RPS (RPS5, n = 4) or 10% RPS (RPS10, n = 4) for 20 days and their fecal samples were collected. The day 0 and 20 samples were analyzed using a 16S rRNA gene sequencing technology, followed by total genomic DNA extraction, library construction, and high-throughput sequencing. After statistical analysis, five phyla and 45 genera accounting for over 0.5% of the reads in any of the three groups were further analyzed. Furthermore, short-chain fatty acids (SCFAs) in the day 20 fecal samples were analyzed using gas chromatography.

**Results:**

Significant changes were not observed in the bacterial composition at the phylum level even after 20 d post feeding (dpf); however, the abundance of *Intestinimonas* and *Barnesiella* decreased in both RPS treatment groups compared to the CON group. Consumption of 5% RPS increased the abundance of *Roseburia* (p<0.05) and decreased the abundance of *Clostridium* (p<0.01) and *Mediterraneibacter* (p< 0.05). In contrast, consumption of 10% RPS increased the abundance of *Olsenella* (p<0.05) and decreased the abundance of *Campylobacter* (p<0.05), *Kineothrix* (p<0.05), *Paraprevotella* (p<0.05), and *Vallitalea* (p<0.05). Additionally, acetate (p<0.01), butyrate (p<0.05), valerate (p = 0.01), and total SCFAs (p = 0.01) were upregulated in the RPS5 treatment group

**Conclusion:**

Feeding 5% RPS altered bacterial community composition and promoted gut health in weaned piglets. Thus, resistant starch as a feed additive may prevent diarrhea in piglets during weaning.

## INTRODUCTION

The mammalian intestinal microbiota is a dynamic and complex ecosystem of bacterial communities that is closely associated with host homeostasis and health [1]. The initial microbiota of the neonate is acquired at birth when it escapes through the birth canal and is continuously affected by the surrounding environment [2]. Weaning is the most critical phase in pig production as the piglets are separated from the sow and subjected to a transition from milk-based diet to solid diet [3], and these dietary changes could lead to gastrointestinal changes and alterations in the intestinal microbiota composition [4]. Hence, weaning transition in piglets can cause intestinal immune dysfunction, poor growth performance, and post-weaning diarrhea, resulting in large economic losses to the swine industry [3,5]. Post-weaning diarrhea is the most important factor in global piglet production as it is strongly associated with increased morbidity, reduced average gain, and decreased animal welfare. Diarrhea in pigs under weaning stress can be caused by various pathogens, including enterotoxigenic *Escherichia coli*, *Salmonella* spp., porcine kobuvirus, and porcine parvovirus [6].

Over the last several decades, antimicrobials have been used to promote piglet development by preventing various post-weaning diseases [7]; however, its overuse is strongly associated with an increase in the number of antibiotic-resistant pathogens. Furthermore, the environmental pollution caused by the antimicrobials has raised important concerns regarding animal and human health [4]. For this reason, the use of antibiotics in feed additives has been banned in various countries. However, the elimination of antibiotics in feed may reduce weight gain and increase mortality due to the emergence of intestinal diseases in post-weaned pigs [8]. Therefore, to keep the swine industry profitable, there is a need to identify sustainable feed additives that can improve the gut microbiota in weaned piglets without the use of antibiotics.

Dietary fibers such as resistant starch (RS) containing starch and starch degradation products, are an important external factor that can improve the intestinal microbiota composition [9]. Digestion of RS is difficult in the small intestines; however, in the large intestines, it serves as an energy source to the gut microbiota, which metabolizes it to produce gases and short-chain fatty acids (SCFAs) via fermentation [10]. Therefore, RS is a representative source of microbiota-accessible carbohydrates that can improve intestinal health [7,11]. Several studies have reported that high RS diets lower nutrient digestibility and feed efficiency, which is not beneficial for pig farming [12,13]; nevertheless, RS is considered as a potential prebiotic and a functional dietary ingredient due to its high fermentability [13].

Raw potato starch (RPS) is a well-known component of RS that can enhance fermentation in the digestive tract and increase pro- and anti-inflammatory cytokine levels in animals [14]. We speculated that RPS could improve intestinal homeostasis and thereby support growth performance by preventing diarrhea in weaned and growing pigs. Recently developed metagenomics technologies yield high-quality and high-throughput sequencing results, which can facilitate the analysis of the composition, abundance, and function of porcine intestinal microbial communities [11,15], and have enabled the investigation of the effect of RPS on animal and human gut microbiota [11,14,16–18]. Therefore, the aim of the present study was to investigate the effects of two different concentrations of RPS feeds on growth performance, SCFA production, and the composition of intestinal microbial communities of weaned piglets. However, considering that RPS is expensive and that long-term RPS intake increases average daily feed intake, determination of the shortest and optimal feeding period is essential for economic reasons [19,11]. Consequently, we aimed to determine the optimal RS feeding period and concentration, in addition to monitoring the changes in the gut microbiota composition.

## MATERIALS AND METHODS

### Ethics statements

This experimental study followed the guidelines approved by the Animal Ethics Committee of the National Institute of Animal Science, Republic of Korea. The study was approved by the Institutional Animal Care and Use Committee of the National Institute of Animal Science, Republic of Korea (approval number: NIAS 2021-503).

### Animals and experimental design

In total, 14, (25-days-old), castrated male piglets (Landrace× Yorkshire), with an initial average weight of 7.03±0.49 kg, were obtained from the same herd from a commercial pig farm. The animals were carefully inspected by veterinarians before housing them in the experimental facility at the National Institute of Animal Science, Republic of Korea. All the piglets were transferred to the facility three days prior to the start of the experiment. The facilities were managed by the designated veterinarians, under a 12:12 h light-dark cycle at constant temperature (21°C±2°C) and relative humidity (55%±10%). During the adaptation period, all animals were sero-negative for major swine diseases: foot-and-mouth disease, classical swine fever, porcine respiratory and reproductive syndrome, and *Mycoplasma* spp. infection. In addition, *Escherichia coli* and *Salmonella* spp. were not isolated in the fecal samples of these pigs. At 28 d of age, the piglets were randomly divided into two treatment groups and one control group (CON, n = 6). The CON diet was formulated according to the nutritional requirements suggested by the Korean feeding standard for pigs (Table 1). Animals in treatment group 1 (RPS10, n = 4) and treatment group 2 (RPS5, n = 4) were fed the CON diet supplemented with 10% RPS and 5% RPS, respectively (Table 1). The pigs were provided 1 kg of feed twice a day (09:00 and 16:00) and had *ad libitum* access to water until the end of the experimental period (at 48 days of age). All the piglets were weighed and fecal samples were collected from their rectum at 0, 5, 10, 15, and 20 days post feeding (dpf). Approximately 10 g fresh feces were transported on ice and stored at −80°C.

### DNA sample preparation

In total, 70 fecal samples were collected from each pig at 0, 5, 10, 15, and 20 dpf and immediately transported to Macrogen Inc. (Seoul, Korea) for metagenomic analysis. DNA was extracted using a DNeasyPowerSoil Kit (Qiagen, Hilden, Germany) according to the manufacturer’s instructions. The extracted DNA was quantified using Quant-IT PicoGreen assay (Invitrogen, NY, USA).

### Library construction and sequencing

The sequencing libraries were prepared according to the Illumina 16S metagenomic sequencing library protocols to amplify the V3-V4 region. Genomic DNA (2 ng) was polymerase chain reaction (PCR)-amplified with 5× reaction buffer, 1 mM dNTP mix, 500 nM of universal F/R PCR primer each, and Herculase II fusion DNA polymerase (Agilent Technologies, Santa Clara, CA, USA). PCR conditions were set as follows: 3 min at 95°C, 25 cycles of 30 s at 95°C, 30 s at 55°C, and 30 s at 72°C, followed by a final extension of 5 min at 72°C. The universal primer pair with Illumina adapter overhang sequences used for the first amplification was as follows: V3-F: 5′-TCGTCGGCAGCGTCAGATGTGTATA AGAGACAGCCTACGGGNGGCWGCAG-3′ and V4-R: 5′-GTCTCGTGGGCTCGGAGATGTGTATAAGAGACA GGACTACHVGGGTATCTAATCC-3′. The PCR product was purified with AMPure beads (Agencourt Bioscience, Berverly, MA, USA). Thereafter, 2 μL of the PCR product was amplified for final library construction using NexteraXT Indexed Primer. The PCR was run for 10 cycles at the conditions mentioned above. Subsequently, the PCR product was purified with AMPure beads and quantified using qPCR according to the qPCR quantification protocol guide (KAPA Library Quantification kits for Illumina Sequencing platforms) and qualified using the TapeStation D1000 ScreenTape system (Agilent Technologies, Waldbronn, Germany). Paired-end (2×300 bp) sequencing was performed using the MiSeq platform (Illumina, San Diego, CA, USA).

### Bioinformatics

Bioinformatics analysis was performed using the quantitative insights into microbial ecology (QIIME) software version 2.0. The paired-end raw sequence data were multiplexed and formatted into FASTQ files. To ensure high sequence quality, the adapter and reads with low Phred score of 20 (Q20 <20%) were trimmed using Cutadapt. Thereafter, the trimmed reads were quality-filtered, denoised, and merged, and the chimeras were removed using Divisive Amplicon Denoising Algorithm (DADA) 2. Taxonomic assignments were determined for amplicon sequence variants (ASVs) using the q2-feature-classifier plugin and a classifier based on the SILVA database (http://www.arb-silva.de). Sequencing data were arranged according to the experimental groups (CON, RPS5, and RPS10) for analytical purposes.

### Microbial community and statistical analysis

Kruskal-Wallis test was performed to assess the alpha diversity of the three diet groups (CON, RPS5, and RPS10) and of the two dpf groups (D0 and D20). In addition, Wilcoxon signed rank test was performed to compare differences in alpha diversities between any two groups among the three diet groups (CON vs RPS5, CON vs RPS10, RPS5 vs RPS10) and two dpf groups (0 dpf vs 20 dpf). All the statistical analyses adopted a p-value of 0.05 as the cut-off value and linear discriminant analysis score of 2.0, using the QIIME software version 2.0. Beta diversity was measured using a weighted UniFrac distance matrix of the QIIME software. The clustering of bacterial communities according to RPS intake or experimental period was visualized using principal coordinate analysis (PCoA) of the EMPerore tool [20]. Variations in microbiota composition during the experimental period and within each group at the phylum and genus levels were assessed. The dominant taxa, which accounted for >0.5% of the ASV tables, were arranged and the data were visualized using GraphPad Prism 7.0 (GraphPad Software Inc., San Diego, CA, USA). Linear discriminant analysis (LDA) effect size (LEfSe) was used to determine the specific effect on the relative abundance of taxa between CON and the two RS-fed groups. Taxa with significance difference (p<0.05) between any two groups were used to build a LEfSe, and those with an LDA score >2.0 were considered to have a significantly altered taxa after RPS consumption compared consumption of control feed. Significant changes in SCFA concentrations between CON and RPS5 and CON and RPS10, were compared using Student’s t-test using SPSS software version 22.0 (IBM, Armonk, NY, USA).

### Measurement of SCFA concentrations

The fecal concentrations of SCFAs were determined using Agilent 6890 series gas chromatography (Agilent Technologies, Santa Clara, CA, USA) coupled with mass spectrometry, according to the method described by Zhang et al [21] with slight modifications. One gram of fecal sample was added and homogenized in 10 mL distilled water and centrifuged at 12,000×*g* for 10 min at 20°C. After the supernatant was filtered using a 0.22 μm syringe filter, 5 μL sample was injected into a 15.0 m×0.53 mm×0.5 μm NUKOL capillary column (Supelco, Bellefonte, PA, USA). Both the injector and detector were operated at 240°C with a split ratio of 50:1. Hydrogen was used as the carrier gas at the flow rate of 30 mL/min. The column temperature was programmed as follows: the initial column temperature was maintained at 100°C for 5 min, followed by an increase at 5°C/min to 150°C and 30°C/min to 240°C, and then held at 240°C for 30 min.

### Data availability

The sequencing data analyzed in this study has been deposited in the NCBI Sequencing Read Archive (SRA) database under the accession number PRJNA771504.

## RESULTS

### Growth traits during the experiment

**Figure 1:**
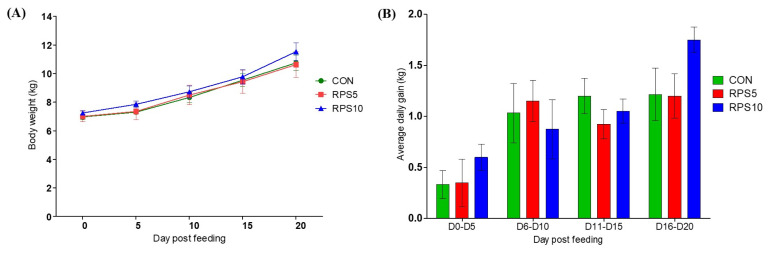
Comparison of (A) body weight change and (B) average daily gain in CON, RPS5, and RPS10 during the experimental period (0 to 20 dpf). RPS, raw potato starch; CON, piglets fed corn/soybean control formula diet; RPS5, piglets fed control formula diet supplemented with 5% RPS; RPS10, piglets fed control formula diet supplemented with 10% RPS; dpf, day post feeding.

Any obvious differences in health or behavior were not observed in any group after consumption of the corn/soybean-based control diet, which was partially replaced with 5% and 10% RPS. BW and average daily gain (ADG) were affected by the diet in all the groups, and they tended to be higher in RPS10 at 20 dpf than in CON and RPS5 (Figure 1).

### High throughput sequencing

**Figure 2:**
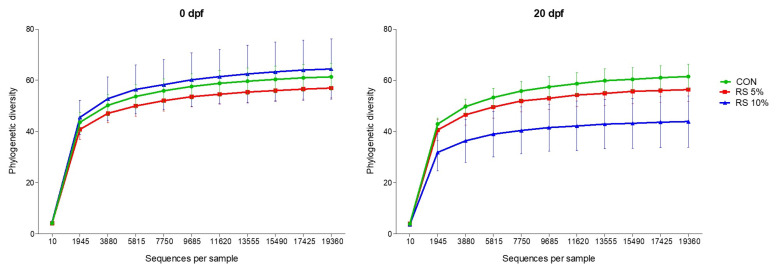
Rarefaction curves plotted based on the number of ASVs, showing richness of the intestinal microbiota observed in fecal samples from CON, RPS5, and RPS10 at 0 and 20 dpf. Comparison with phylogenetic diversity at 0 dpf. ASV, amplicon sequence variant; RPS, raw potato starch; CON, piglets fed corn/soybean control formula diet; RPS5, piglets fed control formula diet supplemented with 5% RPS; RPS10, piglets fed control formula diet supplemented with 10% RPS; dpf, days post feeding.

High-throughput sequencing was performed to compare the composition of bacterial communities in the fecal samples from pigs in the three treatment groups (CON, RPS5, and RPS10). Across all samples, 3,509,381 high-quality sequence reads were obtained after adapter and primer trimming (an average of 93,674±9,151 per sample), quality filtering (67,557±7,504), denoising (130,471±7,461), paired-end read merging (47,035±7,127), and chimera removal (29,408± 5,161). The average length of the quality sequences was 413±11.3 bp. The 2,621 ASVs, comprising archaea (n = 12) and unassigned taxa (n = 2,609), were removed from the dataset, resulting in 5,686 ASVs (Supplementary Table S1). The statistical estimates of richness for the 19,369 sequence subsets from each sample at a genetic distance of 3% are shown in Figure 2. The rarefaction curves were plotted based on the number of ASVs. In all the libraries, the curve tended to approach the saturation plateau, indicating that the libraries in this study were large enough to capture most of the ASVs in each sample.

### Effects of RPS feeding on bacterial diversity and community structure

**Figure 3:**
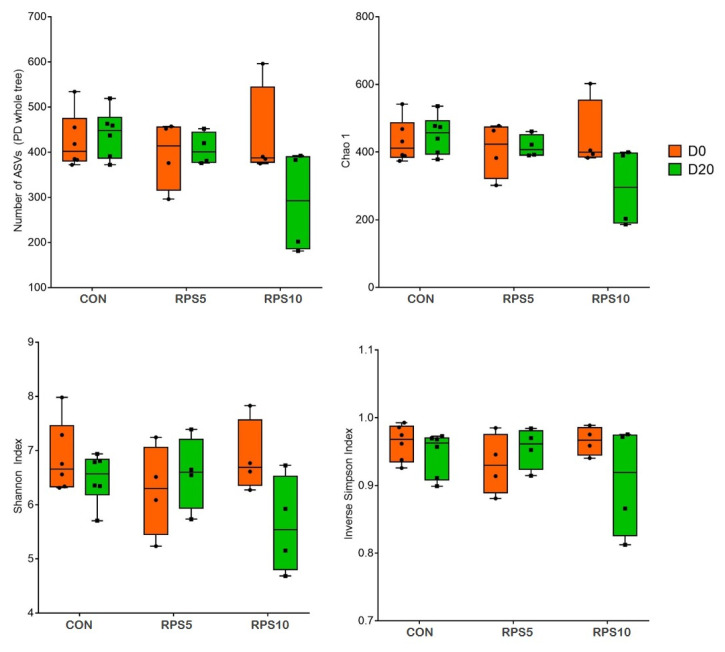
Comparison of alpha diversity between and within the fecal microbiota of CON, RPS5, and RPS10 at 0 and 20 dpf. The four indices included the number of ASVs, Chao 1 richness indices, and Shannon and inverse Simpson diversity indices. ASV, amplicon sequence variant; RPS, raw potato starch; CON, piglets fed corn/soybean control formula diet; RPS5, piglets fed control formula diet supplemented with 5% RPS; RPS10, piglets fed control formula diet supplemented with 10% RPS; dpf, days post feeding.

The alpha diversity indices measured in the dietary groups 20 dpf are shown in Figure 3. The Kruskal-Wallis test demonstrated that the four alpha diversity indices, the number of ASVs and Chao 1 for richness and Shannon and inverse Simpson indices for diversity were slightly lower in RPS5 and RPS10 than in CON.

**Figure 4:**
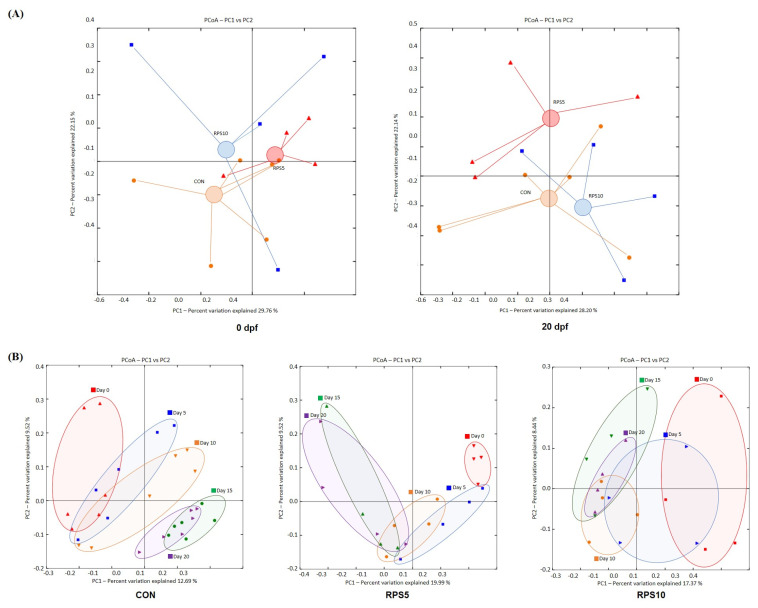
PCoA based on the weighted Unifrac distance showing the 16S rRNA gene-based dissimilarities calculated from the number of ASVs. (A) Beta diversity and bacterial community structure of the intestinal microbiota among CON, RPS5, and RPS10 at the end of the experiment (20 dpf). The center of the cluster of each group was determined based on the distribution of samples, representing the approximate average community composition. (B) Alteration in bacterial community structure during whole experimental period (0, 5, 10, 15, and 20 dpf) within each group. PCoA, principal coordinate analysis; ASV, amplicon sequence variant; RPS, raw potato starch; CON, piglets fed corn/soybean control formula diet; RPS5, piglets fed control formula diet supplemented with 5% RPS; RPS10, piglets fed control formula diet supplemented with 10% RPS; dpf, days post feeding.

Beta-diversity analysis was performed to investigate the structure of the bacterial community and the results are presented as the PCoA ordination plot based on unweighted UniFrac distance matrices. PCoA analysis was performed to assess beta diversity among CON, RPS5, and RPS10 and the PCoA ordination showed that the variation of the first principal component (PC1) and the second (PC2) were 29.76% and 22.15% at 0 dpf and 28.20% and 22.14% at 20 dpf, respectively (Figure 4A). Compared to that at 0 dpf, the bacterial community in RPS5 exhibited movement towards the direction of the CON group in the first PC1 at 20 dpf, albeit with a separation with a shift upward along the PC2 axis. In contrast, the bacterial communities in RPS10 showed more similarities with those in CON, with a negative shift along the PC2 axis. The PCoA analysis also demonstrated obvious changes in the intestinal bacterial communities within each group from 0 to 20 dpf (Figure 4B). Overall, feeding a 100% corn/soybean formula diet resulted in an obvious shift rightward along the PC1 axis and a downward shift along the PC2 axis from 0 to 20 dpf. Feeding RPS led to a shift in a direction opposite to that of CON, irrespective of the amount of RPS supplement. The PCoA structure of both RPS5 and RPS10 shifted leftward with negative values along the PC1 axis, and upward with positive values along PC2. In contrast, a complete separation of bacterial community structure was observed between 15 and 20 dpf in CON. Compared to that in CON, complete separation of the bacterial community was advanced by 5 days, which was observed between 10 and 15 dpf in both RPS5 and RPS10.

### Effects of RPS feeding on bacterial structure and composition

**Figure 5:**
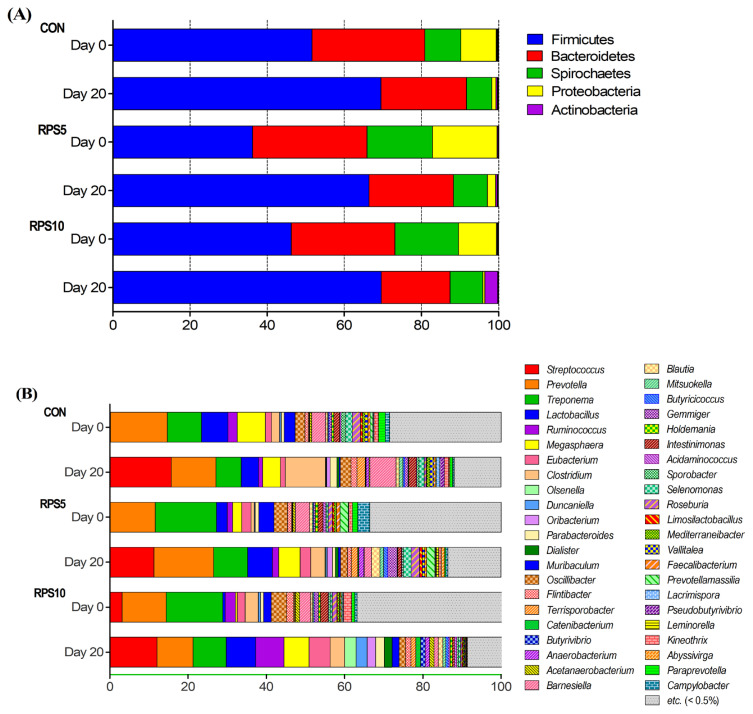
Alteration in the intestinal microbiota composition (A) at phylum and (B) genus levels in CON, RPS5, and RPS10 at the beginning (0 dpf) and the end of the experiment (20 dpf). RPS, raw potato starch; CON, piglets fed corn/soybean control formula; RPS5, piglets fed control formula diet supplemented with 5% RPS; RPS10, piglets fed control formula diet supplemented with 10% RPS; dpf, days post feeding.

Metagenomics analysis using high-throughput sequencing identified 16 phyla, 35 classes, 62 orders, 108 families, 336 genera, and 687 species across all samples. Among them, we used five phyla and 45 genera that comprised ≥0.5% of the microbial communities for further analysis (Supplementary Table S2). At the phylum level, Firmicutes (51.6% in CON, 36.3% in RPS5, and 46.2% in RPS10) was the most dominant, followed by Bacteroidetes (29.3% in CON, 29.6% in RPS5, and 26.9% in RPS10), Spirochaetes (9.3% in CON, 17.0% in RPS5, and 16.5% in RPS10), Proteobacteria (9.2% in CON, 16.7% in RPS5, and 9.8% in RPS10), and Actinobacteria (0.1% in CON, 0.2% in RPS5, and 0.1% in RPS10) in all the tested groups at the beginning of the experiment (Figure 5A). At 20 dpf, Firmicutes (69.5% in CON, 66.4% in RPS5, and 69.6% in RPS10) and Bacteroidetes (22.2% in CON, 22.0% in RPS5, and 17.8% in RPS10) were still predominant in all the groups. Furthermore, at 20 dpf the abundance of Firmicutes increased, while the abundance of Bacteriodetes, Proteobacteria (1.1% in CON, 2.1% in RPS5, and 3.4% in RPS10), and Spirochaetes (6.5% in CON, 8.8% in RPS5, and 8.5% in RPS10) decreased compared to those at 0 dpf. The abundance of Spirochaetes and Actinobacteria in both RPS5 and RPS10 groups was higher than that in CON. The Firmicutes to Bacteriodetes ratio (F/B ratio) in CON, RPS5, and RPS10, was 1.76, 1.22, and 1.72 at 0 dpf and reached 3.13, 3.02, and 3.91 at 20 dpf, respectively. However, the Wilcoxon rank-sum test results revealed that 5% and 10% RPS feeding did not significantly affect the relative abundance of fecal microbiota in any group at the phylum level. At the genus level, *Streptococcus* (15.8% in CON, 11.3% in RPS5, and 12.1% in RPS10), *Prevotella* (11.35% in CON, 15.2% in RPS5, and 9.2% in RPS10), and *Treponema* (6.5% in CON, 8.7% in RPS5, and 8.5% in RPS10) were the three most predominant genera in the experimental groups at 20 dpf (Figure 5B). Among the 45 main genera (>0.5% in any of the three groups), the abundance of *Roseburia* (p = 0.0381) in RPS5 was higher than that in CON, whereas that of *Barnesiella* (p = 0.0190), *Clostridium* (p = 0.0095), *Intestinimonas* (p = 0.0095), and *Mediterraneibacter* (p = 0.0172) decreased. In RPS10, *Olsenella* (p = 0.0381) was more abundant, whereas *Barnesiella* (p = 0.0095), *Intestinimonas* (p = 0.0095), *Campylobacter* (p = 0.0422), *Kineothrix* (p = 0.0139), *Paraprevotella* (p = 0.0139), and *Vallitalea* (p = 0.0139) were less abundant compared to CON.

**Figure 6:**
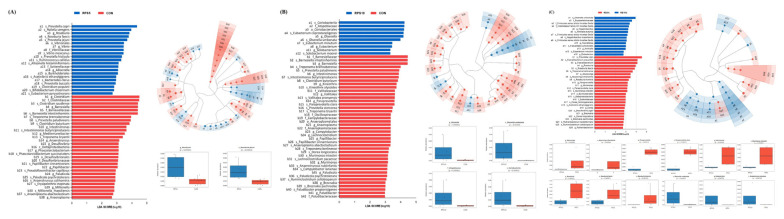
LEfSe revealed predicted biological effect sizes of differential taxa in fecal microbiota between (A) RPS5 and CON, (B) RPS10 and CON, and (C) RPS5 and RPS10 at 20 dpf. The LDA scores and cladogram show the statistically significant difference in the abundance and consistency of the detected bacterial taxa. LEfSe, linear discriminant analysis (LDA) effect size; CON, piglets fed corn/soybean control formula; RPS5, piglets fed control formula supplemented with 5% raw potato starch; RPS10, piglets fed control formula supplemented with 10% raw potato starch; dpf, days post feeding.

The LEfSe analysis showed significant differences in the abundance of bacterial taxa from all ASVs between the CON and the two RPS-fed groups (Figure 6). In total, 2 orders, 2 families, 3 genera, and 14 species were significantly more abundant in RPS5 than in CON. Whereas, 1 class, family, and order, 3 genera, and 4 species were significantly more abundant in RPS10 than those in CON. Among the 46 main genera in this study, only *Roseburia* with *R. faecis* were specifically enriched in RPS5 compared to that in CON. In RPS10, the abundance of class Coriobacteriia with the subtaxa order Coriobacteriales, family Atopobiaceae, genus *Olsenella*, and species *O. umbonata* increased significantly. The taxa related to the genera *Roseburia*, *Abyssivirga*, and *Paraprevotella* were significantly increased in RPS5, whereas those related to *Olsenella* and *Eubacterium* increased significantly in RPS10.

### Effect of RPS feeding on fecal SCFA concentration

**Figure 7:**
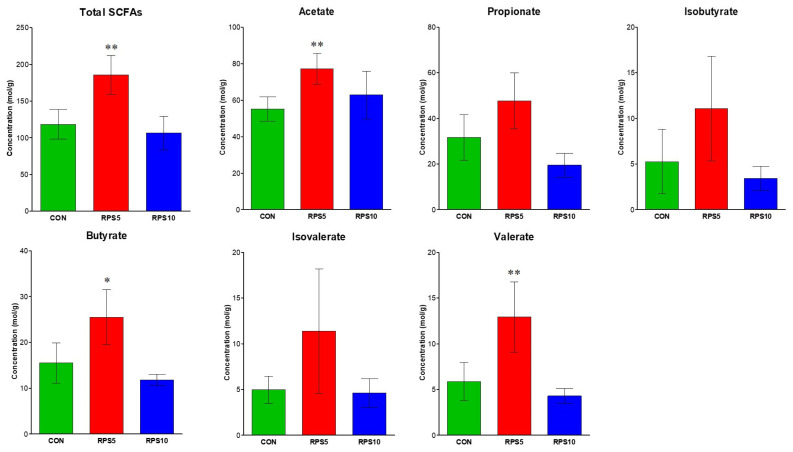
SCFA concentration (mol/g) in fecal samples of CON, RPS5, and RPS10 at 20 dpf. Total SCFAs are the sum of the following SCFAs: acetate, propionate, isobutyrate, butyrate, isovalerate, and valerate. SCFA, short chain fatty acid; RPS, raw potato starch; CON, piglets fed corn/soybean control formula diet; RPS5, piglets fed control formula diet supplemented with 5% RPS; RPS10, piglets fed control formula diet supplemented with 10% RPS; dpf, days post feeding. * p<0.05; ** p<0.01; *** p<0.001.

SCFA concentrations were evaluated in fecal samples collected at 20 dpf to investigate the effect of altered bacterial community structure and composition. As the changes in bacterial communities affected the concentration of SCFAs, the bacterial metabolic output differed between CON and RPS5 (Figure 7). In RPS5, the concentration of all the fecal SCFAs evaluated in this study tended to be higher than that in CON. Among them, the amounts of acetate (p = 0.0031), butyrate (p = 0.0232), valerate (p = 0.0095), and total SCFAs (p = 0.0067) were found to be higher than those in CON. However, the propionate, isobutyrate, and isovalerate levels were unaffected by 5% RPS consumption. In RPS10, all the fecal SCFA evaluated s in this study, except acetate, were lower than those in CON. Although the mean amount of acetate was higher in RPS10 compared to that in CON, the average total SCFAs were higher in CON than in RPS10.

## DISCUSSION

RPS is classified as RS type 2, which is not digested and absorbed in the small intestine because of its crystal structure within starch [10]. Owing to these characteristics, several studies have suggested that RPS consumption may lead to reduction in BW and ADG in pigs [6,12,13,22]. However, our study showed that both 5% and 10% RPS consumption did not significantly affect the BW and ADG of piglets during the 20 d experimental period, complying with the results of a few other studies [11,16].

RPS commonly passes through the small intestine and enters the large intestine. Subsequently, it is fermented in the large intestine, generating energy sources, such as SCFAs, for intestinal microbiota [16,23]. The concentration of total SCFAs, including those of acetate, butyrate, and valerate were increased in the fecal samples of piglets of RPS5 treatment group compared to those of CON. These results were supported by those of previous studies, which showed that RS feeding increased the weight of the total intestinal tract, indicating upregulation of fermentation activity and hence intestinal nutritional contents, including SCFAs [16,24]. Previous studies have also reported that RPS consumption can improve the intestinal mucosal barrier function, immunological tolerance, and generation of host defense peptides by altering gut bacterial composition and community structure, as well as the amounts of SCFAs in the host [11,23,25,26]; however, the total SFCA concentration did not increase in the RPS10 treatment group. Although other metabolites (e.g., succinate, lactate) might have been generated in RPS10, they were not measured in this study; hence, the overall results, including richness, diversity, and bacterial community alteration in RPS10 differed from those in RPS5, suggesting that feeding 5% RPS to weaned piglets for 20 d could improve their gut health.

Complying with the results of previous studies [11,23,25] RPS5 and RPS10 did not differ significantly in the abundance and diversity of bacterial communities. The results of this study revealed that Firmicutes and Bacteroidetes were the two most abundant phyla in all groups during the 20 d experimental period, consistent with the results of the previous studies [27,28]. We found that the F/B ratios of CON, RPS5, and RPS10 at 20 dpf were higher than those at 0 dpf by 1.37, 1.80, and 2.19 times, respectively. These results indicate that the abundance of some bacterial groups belonging to the phylum Firmicutes increased when the piglets were fed 5% and 10% RPS-supplemented diets. Previous studies have also reported that heavy piglets commonly show a high F/B ratio [27,29]. Thus, the 1.3- and 1.6-fold higher F/B ratio in RPS5 and RPS10 may indicate that RPS consumption could improve the growth performance of piglets.

Despite lack of significant changes at the phylum level, RPS feeding affected the abundance of several subtaxa and the bacterial composition at the genus and species levels. Compared to the control diet, consumption of 5% RPS significantly increased the abundance of *Roseburia*, similar to the results of previous studies on RS2 consumption in humans [18,30]. Bacteria are known to play an important role in intestinal epithelial permeability by fermenting polysaccharides and producing butyrate [18]. The concentration of fecal butyrate in RPS5 compared to that in CON indicated that *Roseburia* may contribute to an increase in butyrate production, providing piglet intestinal microbiota with a beneficial energy source [31]. In contrast, we observed a significant increase in the abundance of *Olsenella*, in particular, *O. umbonata* (p = 0.0209) in RPS10, which are obligate anaerobes known to inhabit the gastrointestinal tract of pigs [32]. Further, our results suggest that feeding 10% RPS induces an anaerobic intestinal environment. Additionally, as *Olsenella umbonata* is known to produce large amounts of lactic acid and small amounts of acetate by metabolizing glucose, there might be an increased production of lactic acid in the gastrointestinal tract of 10% RPS consuming pigs. Thus, *Olsenella* spp. might reduce the incidence of diarrhea, improve gut health, and promote the growth of piglets owing to these changes [32–34].

In contrast, RPS consumption also reduced the abundance of several taxa. In both RPS5 and RPS10 treatment groups, the abundance of the genera *Barnesiella* and *Intestinimonas* decreased as observed by the reduction in *B. intestinihominis* and *I. butyriciproducens* population in RPS5 (p = 0.0190 and p = 0.0095, respectively) and RPS10 (p = 0.0095 and p = 0.0095, respectively). Although several studies have reported the presence of *Barnesiella* and *Intestinimonas* in normal intestinal flora of healthy pigs, Hasan et al [35] suggested that they may act as opportunistic pathogens [36,37]. Furthermore, previous studies have reported that *Barnesiella* and *B. intestinihominis* produce succinate and acetate as end products of glucose metabolism [38], whereas, Intestinimonas and I. butyriciproducens can ferment and convert fructosyllysine or lysine to acetate and butyrate [36,39]. Nevertheless, the relatively lower abundance of Barnesiella- and Intestinimonas-related taxa affected the concentrations of fecal acetate and butyrate negligibly.

Furthermore, our results reveal that consumption of 5% RPS significantly reduced the abundance of *Clostridium* and *Mediterraneibacter* in this study. This diminished abundance of *Clostridium* was mainly due to a decrease in the abundance of *Clostridium saudiense* (p = 0.0381) and *Clostridium butyricum* (p = 0.0131). *C. saudiense* has been isolated from the porcine digestive tract and its surroundings, and has been reported to correlate negatively with ADG, carcass weight, and butyrate content in pigs [40,41]. In contrast, *C. butyricum* is a butyrate-producing bacterium that has been suggested to promote pig growth and improve nutrient utilization [42,43]; however, our results revealed that reduction in *C. butyricum* did not significantly affect butyrate production in RPS5, consistent with previous reports that this species did not affect the growth performance or butyric acid content [44,45]. A few studies have also suggested that the reduction in *M. faecis* and *M. glycyrrhizinilyticus* abundance decreased the abundance of the genus *Mediterraneibacter*. *M. faecis* is known to produce acetate, propionate, and butyrate by degrading cellulose [46,47]; however, our results indicated that a decrease in the abundance of this species negligibly affected the production of these SFCAs in RPS5. Information regarding the function or characteristics of *M. glycyrrhizinilyticus* is still scarce, necessitating further studies regarding the specific functions of *Clostridium* and *Mediterraneibacter* in pig intestines.

Additionally, our results reveal that consumption of 10% RPS showed significant reduction in the abundance of *Campylobacter*, including *C. lanienae* (p = 0.0234). *Campylobacter* is the causative agent of enteritis in humans [48] whereas, in pigs, the bacterium is a natural inhabitant of the healthy intestine and is an opportunistic pathogen that can cause diarrhea [49]. The abundance of *Kineothrix* and *Vallitalea* was lower as observed by the reduction in the abundance of *K. alysoide* (p = 0.0139) and *V. pronyensis* (p = 0.0139), respectively. *K. alysoide* is a saccharolytic butyrate-producing bacterium, which ferments glucose and produces acetate [50,51]; however, reduction in the abundance of these bacteria did not significantly affect acetate and butyrate production in this study. Furthermore, *Paraprevotella* and the subtaxon *P. clara* (p = 0.0139) were significantly less abundant in RPS fed groups than in CON. Complying with our results, the abundance of *Paraprevotella* and *P. clara* have been found to be lower in fecal samples of pigs and humans fed dietary fiber and RPS, respectively [52]. *P. clara* has been reported to produce succinate and acetate as the end products of glucose metabolism; however, the amount of acetate in the present study was negligibly affected by the decrease in the abundance of this bacterium.

Comparison between the two treatment groups revealed that *Roseburia*, *Paraprevotella*, and *Abyssivirga* were the most abundant genera in RPS5, whereas, *Olsenella* and *Eubacterium* were the most abundant genera in RPS10. *Paraprevotella* can degrade cellulose and hemicellulose and *Abyssivirga* can ferment carbohydrates [53,54] thus, an increase in the abundance of these genera post 5% RPS consumption may improve the nutritional availability and digestibility of high-fiber diets compared to that observed in 10% RPS consumption. However, their characteristics should be investigated further, as *Abyssivirga* has been isolated from human patients with atopic dermatitis [55]. In contrast, the abundance of *Olsenella* and *Eubacterium*-related taxa increased significantly in RPS10 and this increase in the abundance of butyrate-producing bacteria, may reinforce porcine gut health and barrier function [56]; however, the increase in abundance of these two genera did not upregulate butyrate production in RPS10.

## CONCLUSION

The present findings suggest that 5% RPS consumption can help maintain the balance of beneficial bacteria, promote SCFA production, and ultimately improve gut health of weaned piglets. Moreover, the results could improve the understanding of veterinary researchers regarding pig intestinal microbiota and determine the appropriate RPS-supplemented diet formulation with an optimal feeding period. Consumption of 5% RPS for 20 d can promote the gut health and growth performance of weaned piglets, possibly by suppressing the growth of various intestinal pathogens. Further, nutritional immunology studies are needed to determine the difference in the microbiota of pigs fed with 5% and 10% RPS and to clarify the mechanisms underlying the immune responses in these pigs.

## Figures and Tables

**Table 1:** Composition of the experimental diets

Items	Contents (%)		

	Treatment groups^1)^		Control group^1)^
	
	RPS10	RPS5	CON
Ingredients			
Corn	62.35	68.24	73.74
Soybean meal 44%	24.40	23.25	22.20
Soybean oil	0.10	0.33	0.86
L-Lysine-HCl	0.12	0.15	0.17
Dicalcium phosphate	1.20	1.15	1.15
Limestone	0.83	0.88	0.88
Vitamin-mineral premix^2)^	0.50	0.50	0.50
NaCl	0.50	0.50	0.50
Potato starch	10.00	5.00	-
Calculated composition			
Metabolizable energy (kcal/kg)	3,315	3,300	3,300
Crude protein	16.0	16.00	16.0
Lysine	0.95	0.95	0.95
Methionine	0.25	0.26	0.26
Calcium	0.66	0.66	0.66
Total protein	0.56	0.56	0.56

RPS, raw potato starch.

1) RPS, raw potato starch; CON, piglets fed corn/soybean control formula diet; RPS5, piglets fed control formula diet supplemented with 5% RPS; RPS10, piglets fed control formula diet supplemented with 10% RPS; dpf, days post feeding.

2) Values supplied per kilogram premix feed concentrations: Vitamin A, 5,000,000 IU; Vitamin E, 1,000 mg; Vitamin B_1_, 150 mg; Vitamin B_2_, 300 mg; Vitamin B_12_, 1,500 mg; Niacinamide, 1,500 mg; DL-calcium pantothenate, 1,000 mg; Folic acid, 200 mg; Vitamin H, 10 mg; Choline chloride, 2,000 mg; min 3,800 mg; Zinc, 1,500 mg; Iron, 4,000 mg; Cu, 500 mg; I, 250 mg; Co, 100 mg; Mg, 200 mg.

## SUPPLEMENTARY MATERIAL

We certify that there is no conflict of interest with any financial organization regarding the material discussed in the manuscript.

**Supplementary Table S1**. The arranged ASV tables generated by high throughput 16s rRNA metagenomics sequencing **Supplementary Table S2**. The 16 phyla and 336 genera identified by high-throughput 16s rRNA metagenomics sequencing in CON and RPS-fed groups

## References

[1] Bäumler AJ, Sperandio V (2016). Interactions between the microbiota and pathogenic bacteria in the gut. Nature.

[2] Mach N, Berri M, Estellé J (2015). Early-life establishment of the swine gut microbiome and impact on host phenotypes. Environ Microbiol Rep.

[3] Campbell JM, Crenshaw JD, Polo J (2013). The biological stress of early weaned piglets. J Anim Sci Biotechnol.

[4] Gresse R, Chaucheyras-Durand F, Fleury MA, Van de Wiele T, Forano E, Blanquet-Diot S (2017). Gut microbiota dysbiosis in postweaning piglets: Understanding the keys to health. Trends Microbiol.

[5] Moeser AJ, Pohl CS, Rajput M (2017). Weaning stress and gastrointestinal barrier development: Implications for lifelong gut health in pigs. Anim Nutr.

[6] Hermann-Bank ML, Skovgaard K, Stockmarr A (2015). Characterization of the bacterial gut microbiota of piglets suffering from new neonatal porcine diarrhoea. BMC Vet Res.

[7] Heo JM, Opapeju FO, Pluske JR, Kim JC, Hampson DJ, Nyachoti CM (2013). Gastrointestinal health and function in weaned pigs: a review of feeding strategies to control post-weaning diarrhoea without using in-feed antimicrobial compounds. J Anim Physiol Anim Nutr (Berl).

[8] Aarestrup FM, Jensen VF, Emborg HD, Jacobsen E, Wegener HC (2010). Changes in the use of antimicrobials and the effects on productivity of swine farms in Denmark. Am J Vet Res.

[9] Zhao J, Bai Y, Tao S (2019). Fiber-rich foods affected gut bacterial community and short-chain fatty acids production in pig model. J Funct Foods.

[10] Champ MM (2004). Physiological aspects of resistant starch and in vivo measurements. J AOAC Int.

[11] Trachsel J, Briggs C, Gabler NK, Allen HK, Loving CL (2019). Dietary resistant potato starch alters intestinal microbial communities and their metabolites, and markers of immune regulation and barrier function in swine. Front Immunol.

[12] Gerrits WJ, Bosch MW, van den Borne JJ (2012). Quantifying resistant starch using novel, in vivo methodology and the energetic utilization of fermented starch in pigs. J Nutr.

[13] Regmi PR, Metzler-Zebeli BU, Gänzle MG, van Kempen TATG, Zijlstra RT (2011). Starch with high amylose content and low in vitro digestibility increases intestinal nutrient flow and microbial fermentation and selectively promotes Bifidobacteria in pigs. J Nutr.

[14] Nielsen TS, Lærke HN, Theil PK (2014). Diets high in resistant starch and arabinoxylan modulate digestion processes and scfa pool size in the large intestine and faecal microbial composition in pigs. Br J Nutr.

[15] Guo L, Zhang D, Fu S (2021). Metagenomic sequencing analysis of the effects of colistin sulfate on the pig gut microbiome. Front Vet Sci.

[16] Fang L, Jiang X, Su Y, Zhu W (2014). Long-term intake of raw potato starch decreases back fat thickness and dressing percentage but has no effect on the longissimus muscle quality of growing–finishing pigs. Livest Sci.

[17] Bang SJ, Lee ES, Song EJ (2019). Effect of raw potato starch on the gut microbiome and metabolome in mice. Int J Biol Macromol.

[18] Hughes RL, Horn WH, Finnegan P (2021). Resistant starch type 2 from wheat reduces postprandial glycemic response with concurrent alterations in gut microbiota composition. Nutrients.

[19] Pauly C, Spring P, O’Doherty JV, Kragten SA, Bee G (2008). Performances, meat quality and boar taint of castrates and entire male pigs fed a standard and a raw potato starch-enriched diet. Animal.

[20] Vázquez-Baeza Y, Pirrung M, Gonzalez A, Knight R (2013). EMPeror: A tool for visualizing high-throughput microbial community data. GigaScience.

[21] Zhang D, Liu H, Wang S (2019). Fecal microbiota and its correlation with fatty acids and free amino acids metabolism in piglets after a Lactobacillus strain oral administration. Front Micobiol.

[22] Bindelle J, Leterme P, Buldgen A (2008). Nutritional and environmental consequences of dietary fibre in pig nutrition: a review. Biotechnol Agron Soc Environ.

[23] Sun Y, Su Y, Zhu W (2016). Microbiome-metabolome responses in the cecum and colon of pig to a high resistant starch diet. Front Microbiol.

[24] Hedemann MS, Bach Knudsen KEB (2007). Resistant starch for weaning pigs: effect on concentration of short chain fatty acids in digesta and intestinal morphology. Livest Sci.

[25] Sun Y, Zhou L, Fang L, Su Y, Zhu W (2015). Responses in colonic microbial community and gene expression of pigs to a long-term high resistant starch diet. Front Microbiol.

[26] Zhou L, Fang L, Sun Y, Su Y, Zhu W (2017). Effects of a diet high in resistant starch on fermentation end-products of protein and mucin secretion in the colons of pigs. Starch-Stärke.

[27] Ding X, Lan W, Liu G, Ni H, Gu JD (2019). Exploring possible associations of the intestine bacterial microbiome with the pre-weaned weight gaining performance of piglets in intensive pig production. Sci Rep.

[28] Kim HB, Isaacson RE (2015). The pig gut microbial diversity: Understanding the pig gut microbial ecology through the next generation high throughput sequencing. Vet Microbiol.

[29] Han GG, Lee JY, Jin GD (2017). Evaluating the association between body weight and the intestinal microbiota of weaned piglets via 16S rRNA sequencing. Appl Microbiol Biotechnol.

[30] Kemp JA, Regis de Paiva B, Fragoso Dos Santos H (2021). The impact of enriched resistant starch type-2 cookies on the gut microbiome in hemodialysis patients: a randomized controlled trial. Mol Nutr Food Res.

[31] Hamer HM, Jonkers D, Venema K, Vanhoutvin S, Troost FJ, Brummer RJ (2008). Review article: The role of butyrate on colonic function. Aliment Pharmacol Ther.

[32] Kraatz M, Wallace RJ, Svensson L (2011). Olsenella umbonata sp. nov., a microaerotolerant anaerobic lactic acid bacterium from the sheep rumen and pig jejunum, and emended descriptions of Olsenella, Olsenella uli and Olsenella profusa. Int J Syst Evol Microbiol.

[33] Li X, Jensen RL, Højberg O, Canibe N, Jensen BB (2015). Olsenella scatoligenes sp. Nov., a 3-methylindole-(skatole) and 4-methylphenol-(p-cresol) producing bacterium isolated from pig faeces. Int J Syst Evol Microbiol.

[34] Long SF, Xu YT, Pan L (2018). Mixed organic acids as antibiotic substitutes improve performance, serum immunity, intestinal morphology and microbiota for weaned piglets. Anim Feed Sci Technol.

[35] Hasan S, Saha S, Junnikkala S, Orro T, Peltoniemi O, Oliviero C (2018). Late gestation diet supplementation of resin acid-enriched composition increases sow colostrum immunoglobulin G content, piglet colostrum intake and improve sow gut microbiota. Animal.

[36] Ruzauskas M, Bartkiene E, Stankevicius A (2020). The influence of essential oils on gut microbial profiles in pigs. Animals (Basel).

[37] Wylensek D, Hitch TCA, Riedel T (2020). A collection of bacterial isolates from pig intestines revealed functional and taxonomic diversity. Nat Commun.

[38] Morotomi M, Nagai F, Sakon H, Tanaka R (2008). Dialister succinatiphilus sp. Nov. and Barnesiella intestinihominis sp. Nov., isolated from human faeces. Int J Syst Evol Microbiol.

[39] Bui TPN, Ritari J, Boeren S, Plugge CM, de Vos WM (2015). Production of butyrate from lysine and the amadori product fructoselysine by a human gut commensal. Nat Commun.

[40] Kumar H, Jang YN, Kim K, Park J, Jung MW, Park JE (2020). Compositional and functional characteristics of swine slurry microbes through 16S rRNA metagenomic sequencing approach. Animals.

[41] Torres-Pitarch A, Gardiner GE, Cormican P (2020). Effect of cereal soaking and carbohydrase supplementation on growth, nutrient digestibility and intestinal microbiota in liquid-fed grow-finishing pigs. Sci Rep.

[42] Casas GA, Blavi L, Cross TWL, Lee AH, Swanson KS, Stein HH (2020). Inclusion of the direct-fed microbial Clostridium butyricum in diets for weanling pigs increases growth performance and tends to increase villus height and crypt depth, but does not change intestinal microbial abundance. J Anim Sci.

[43] Hu X, Lin B, Luo M, Zheng X, Zhang H (2019). The isolation, identification, physiological property of pig-isolate Clostridium butyricum ly33 using lactic acid and its effects on intestinal function of weaned piglets. Ital J Anim Sci.

[44] Liu P, Zhao J, Wang W (2018). Dietary corn bran altered the diversity of microbial communities and cytokine production in weaned pigs. Front Microbiol.

[45] Zhang J, Chen X, Liu P (2018). Dietary Clostridium butyricum induces a phased shift in fecal microbiota structure and increases the acetic acid-producing bacteria in a weaned piglet model. J Agric Food Chem.

[46] Jin M, Kalainy S, Baskota N (2019). Faecal microbiota from patients with cirrhosis has a low capacity to ferment non-digestible carbohydrates into short-chain fatty acids. Liver Int.

[47] Togo AH, Diop A, Bittar F (2018). Description of Mediterraneibacter massiliensis, gen. nov., sp. nov., a new genus isolated from the gut microbiota of an obese patient and reclassification of Ruminococcus faecis, Ruminococcus lactaris, Ruminococcus torques, Ruminococcus gnavus and Clostridium glycyrrhizinilyticum as Mediterraneibacter faecis comb. nov., Mediterraneibacter lactaris comb. nov., Mediterraneibacter torques comb. nov., Mediterraneibacter gnavus comb. nov. and Mediterraneibacter glycyrrhizinilyticus comb. nov. Antonie van Leeuwenhoek.

[48] Lévesque S, Lemay F, Bekal S, Frost EH, Michaud S (2016). First reported case of Campylobacter lanienae enteritis in a human. JMM Case Rep.

[49] Burrough E, Terhorst S, Sahin O, Zhang Q (2013). Prevalence of campylobacter spp. Relative to other enteric pathogens in grow-finish pigs with diarrhea. Anaerobe.

[50] Ben Aissa FB, Postec A, Erauso G (2014). Vallitalea pronyensis sp. Nov., isolated from a marine alkaline hydrothermal chimney. Int J Syst Evol Microbiol.

[51] Haas KN, Blanchard JL (2017). Kineothrix alysoides, gen. Nov., sp. nov., a saccharolytic butyrate-producer within the family Lachnospiraceae. Int J Syst Evol Microbiol.

[52] Leser TD, Amenuvor JZ, Jensen TK, Lindecrona RH, Boye M, Møller K (2002). Culture-independent analysis of gut bacteria: the pig gastrointestinal tract microbiota revisited. Appl Environ Microbiol.

[53] Morotomi M, Nagai F, Sakon H, Tanaka R (2009). Paraprevotella clara gen. Nov., sp. Nov. and Paraprevotella xylaniphila sp. Nov., members of the family ‘Prevotellaceae’ isolated from human faeces. Int J Syst Evol Microbiol.

[54] Schouw A, Leiknes Eide TL, Stokke R, Pedersen RB, Steen IH, Bødtker G (2016). Abyssivirga alkaniphila gen. Nov., sp. Nov., an alkane-degrading, anaerobic bacterium from a deep-sea hydrothermal vent system, and emended descriptions of Natranaerovirga pectinivora and Natranaerovirga hydrolytica. Int J Syst Evol Microbiol.

[55] Climent E, Martinez-Blanch JF, Llobregat L (2021). Changes in gut microbiota correlates with response to treatment with probiotics in patients with atopic dermatitis. A post hoc analysis of a clinical trial. Microorganisms.

[56] Poco SE, Nakazawa F, Sato M, Hoshino E (1996). Eubacterium minutum sp. Nov., isolated from human periodontal pockets. Int J Syst Bacteriol.

