# Supplementary material for: Effect of feeding raw potato starch on the composition dynamics of the piglet intestinal microbiome
Source: Anim Biosci. 2022 Sep 2;35(11):1698–710. doi: 10.5713/ab.22.0045 (PMC9659463; doi:10.5713/ab.22.0045)
Supplement: Supplementary file 1 [file ab-22-0045-suppl.pdf]

Table S1. The arranged ASV tables generated by high throughput 16s rRNA metagenomic sequencing

| Query | | | | | | Subject | | | | | | Score | | Identities | | Gaps | | Strand | | Taxonomy | | | | | | | | | Filter |
|---|---|---|---|---|---|---|---|---|---|---|---|---|---|---|---|---|---|---|---|---|---|---|---|---|---|---|---|---|---|
| Name | Q_Length | Q_Best | Q_Start | Q_End | Q_Coverage | Description | Accession | S_Length | S_Start | S_End | S_Coverage | Bit | E-Value | I_Match/Total | I_Pct.(%) | G_Match/Total | G_Pct.(%) | Query | Subject | Organism | species | genus | family | order | class | phylum | kingdom | superkingdom | Filter |
| ASV1 | 427 | 3 | 1 | 427 | 100 | NR_041781.1 Streptococcus alactolyticus strain ATCC 43077 16S ribosomal RNA, partial sequence | NR_041781.1 | 1437 | 346 | 772 | 29.71 | 789.6 | 0 | 427/427 | 100 | 0/427 | 0 | Plus | Plus | Bacteria; Firmicutes; Bacilli; Lactobacillales; Streptococcaceae; Streptococcus; Streptococcus alactolyticus | Streptococcus alactolyticus | Streptococcus | Streptococcaceae | Lactobacillales | Bacilli | Firmicutes | | Bacteria | |
| ASV2 | 427 | 4 | 1 | 427 | 100 | NR_042802.1 Lactobacillus ultunensis strain Kx146C1 16S ribosomal RNA, partial sequence | NR_042802.1 | 1550 | 370 | 796 | 27.55 | 789.6 | 0 | 427/427 | 100 | 0/427 | 0 | Plus | Plus | Bacteria; Firmicutes; Bacilli; Lactobacillales; Lactobacillaceae; Lactobacillus; Lactobacillus ultunensis | Lactobacillus ultunensis | Lactobacillus | Lactobacillaceae | Lactobacillales | Bacilli | Firmicutes | | Bacteria | |
| ASV3 | 427 | 1 | 1 | 427 | 100 | NR_102980.1 Megasphaera elsdenii DSM 20460 16S ribosomal RNA, partial sequence | NR_102980.1 | 1552 | 369 | 795 | 27.51 | 789.6 | 0 | 427/427 | 100 | 0/427 | 0 | Plus | Plus | Bacteria; Firmicutes; Negativicutes; Veillonellales; Veillonellaceae; Megasphaera; Megasphaera elsdenii | Megasphaera elsdenii | Megasphaera | Veillonellaceae | Veillonellales | Negativicutes | Firmicutes | | Bacteria | |
| ASV4 | 402 | 2 | 1 | 402 | 100 | NR_144696.1 Clostridium saudiense strain JCC 16S ribosomal RNA, partial sequence | NR_144696.1 | 1478 | 350 | 751 | 27.2 | 704.7 | 0 | 395/402 | 98 | 0/402 | 0 | Plus | Plus | Bacteria; Firmicutes; Clostridia; Eubacteriales; Clostridiaceae; Clostridium; Clostridium saudiense | Clostridium saudiense | Clostridium | Clostridiaceae | Eubacteriales | Clostridia | Firmicutes | | Bacteria | |
| ASV5 | 427 | 1 | 1 | 427 | 100 | NR_025131.1 Treponema parvum strain OMZ 833 16S ribosomal RNA, partial sequence | NR_025131.1 | 1485 | 341 | 767 | 28.75 | 529.3 | 6E-150 | 381/428 | 89 | 2/428 | 0 | Plus | Plus | Bacteria; Spirochaetes; Spirochaetia; Spirochaetales; Spirochaetaceae; Treponema; Treponema parvum | Treponema parvum | Treponema | Spirochaetaceae | Spirochaetales | Spirochaetia | Spirochaetes | | Bacteria | |
| ASV6 | 404 | 1 | 1 | 404 | 100 | NR_026476.1 Succinivibrio dextrinosolvens strain 0554 16S ribosomal RNA, partial sequence | NR_026476.1 | 1463 | 331 | 732 | 27.48 | 656.7 | 0 | 388/404 | 96 | 2/404 | 0 | Plus | Plus | Bacteria; Proteobacteria; Gammaproteobacteria; Aeromonadales; Succinivibrionaceae; Succinivibrio; Succinivibrio dextrinosolvens | Succinivibrio dextrinosolvens | Succinivibrio | Succinivibrionaceae | Aeromonadales | Gammaproteobacteria | Proteobacteria | | Bacteria | |
| ASV7 | 427 | 1 | 1 | 427 | 100 | NR_074742.1 Treponema brennaborense strain DD5/3 16S ribosomal RNA, partial sequence | NR_074742.1 | 1530 | 351 | 776 | 27.84 | 593.9 | 2E-169 | 393/428 | 91 | 3/428 | 0 | Plus | Plus | Bacteria; Spirochaetes; Spirochaetia; Spirochaetales; Spirochaetaceae; Treponema; Treponema brennaborense | Treponema brennaborense | Treponema | Spirochaetaceae | Spirochaetales | Spirochaetia | Spirochaetes | | Bacteria | |
| ASV8 | 427 | 1 | 1 | 427 | 100 | NR_042942.1 Treponema porcinum strain 14V28 16S ribosomal RNA, partial sequence | NR_042942.1 | 1426 | 258 | 684 | 29.94 | 784.1 | 0 | 426/427 | 99 | 0/427 | 0 | Plus | Plus | Bacteria; Spirochaetes; Spirochaetia; Spirochaetales; Spirochaetaceae; Treponema; Treponema porcinum | Treponema porcinum | Treponema | Spirochaetaceae | Spirochaetales | Spirochaetia | Spirochaetes | | Bacteria | |
| ASV9 | 401 | 1 | 1 | 401 | 100 | NR_137408.1 Terrisporobacter petrolearius strain LAM0A37 16S ribosomal RNA, partial sequence | NR_137408.1 | 1405 | 307 | 707 | 28.54 | 725 | 0 | 398/401 | 99 | 0/401 | 0 | Plus | Plus | Bacteria; Firmicutes; Clostridia; Eubacteriales; Peptostreptococcaceae; Terrisporobacter; Terrisporobacter petrolearius | Terrisporobacter petrolearius | Terrisporobacter | Peptostreptococcaceae | Eubacteriales | Clostridia | Firmicutes | | Bacteria | |
| ASV10 | 421 | 2 | 1 | 421 | 100 | NR_113073.1 Barnesiella intestinihominis strain JCM 15079 16S ribosomal RNA, partial sequence | NR_113073.1 | 1481 | 346 | 767 | 28.49 | 534.8 | 1E-151 | 380/424 | 89 | 5/424 | 1 | Plus | Plus | Bacteria; Bacteroidetes; Bacteroidia; Bacteroidales; Barnesiellaceae; Barnesiella; Barnesiella intestinihominis | Barnesiella intestinihominis | Barnesiella | Barnesiellaceae | Bacteroidales | Bacteroidia | Bacteroidetes | | Bacteria | |
| ASV11 | 427 | 2 | 1 | 427 | 100 | NR_113306.1 Megasphaera elsdenii DSM 20460 16S ribosomal RNA, partial sequence | NR_113306.1 | 1174 | 232 | 658 | 36.37 | 785.9 | 0 | 426/427 | 99 | 0/427 | 0 | Plus | Plus | Bacteria; Firmicutes; Negativicutes; Veillonellales; Veillonellaceae; Megasphaera; Megasphaera elsdenii | Megasphaera elsdenii | Megasphaera | Veillonellaceae | Veillonellales | Negativicutes | Firmicutes | | Bacteria | |
| ASV12 | 404 | 1 | 1 | 404 | 100 | NR_104907.1 Eubacterium coprostanoligenes strain HL 16S ribosomal RNA, partial sequence | NR_104907.1 | 1446 | 332 | 734 | 27.87 | 573.6 | 3E-163 | 373/404 | 92 | 1/404 | 0 | Plus | Plus | Bacteria; Firmicutes; Clostridia; Eubacteriales; Eubacteriaceae; Eubacterium; Eubacterium coprostanoligenes | Eubacterium coprostanoligenes | Eubacterium | Eubacteriaceae | Eubacteriales | Clostridia | Firmicutes | | Bacteria | |
| ASV13 | 422 | 4 | 1 | 422 | 100 | NR_044864.1 Hallella seregens ATCC 51272 16S ribosomal RNA, partial sequence | NR_044864.1 | 1463 | 335 | 756 | 28.84 | 608.7 | 7E-174 | 391/422 | 92 | 0/422 | 0 | Plus | Plus | Bacteria; Bacteroidetes; Bacteroidia; Bacteroidales; Prevotellaceae; Prevotella; Hallella seregens | Hallella seregens | Prevotella | Prevotellaceae | Bacteroidales | Bacteroidia | Bacteroidetes | | Bacteria | |
| ASV14 | 427 | 1 | 1 | 427 | 100 | NR_042942.1 Treponema porcinum strain 14V28 16S ribosomal RNA, partial sequence | NR_042942.1 | 1426 | 258 | 684 | 29.94 | 584.7 | 1E-166 | 392/429 | 91 | 4/429 | 0 | Plus | Plus | Bacteria; Spirochaetes; Spirochaetia; Spirochaetales; Spirochaetaceae; Treponema; Treponema porcinum | Treponema porcinum | Treponema | Spirochaetaceae | Spirochaetales | Spirochaetia | Spirochaetes | | Bacteria | |
| ASV15 | 422 | 1 | 1 | 422 | 100 | NR_113411.1 Prevotella copri DSM 18205 strain JCM 13464 16S ribosomal RNA, partial sequence | NR_113411.1 | 1493 | 358 | 779 | 28.27 | 763.8 | 0 | 419/422 | 99 | 0/422 | 0 | Plus | Plus | Bacteria; Bacteroidetes; Bacteroidia; Bacteroidales; Prevotellaceae; Prevotella; Prevotella copri | Prevotella copri | Prevotella | Prevotellaceae | Bacteroidales | Bacteroidia | Bacteroidetes | | Bacteria | |
| ASV16 | 427 | 1 | 1 | 427 | 100 | NR_042942.1 Treponema porcinum strain 14V28 16S ribosomal RNA, partial sequence | NR_042942.1 | 1426 | 258 | 684 | 29.94 | 789.6 | 0 | 427/427 | 100 | 0/427 | 0 | Plus | Plus | Bacteria; Spirochaetes; Spirochaetia; Spirochaetales; Spirochaetaceae; Treponema; Treponema porcinum | Treponema porcinum | Treponema | Spirochaetaceae | Spirochaetales | Spirochaetia | Spirochaetes | | Bacteria | |
| ASV17 | 421 | 2 | 1 | 421 | 100 | NR_113073.1 Barnesiella intestinihominis strain JCM 15079 16S ribosomal RNA, partial sequence | NR_113073.1 | 1481 | 346 | 767 | 28.49 | 529.3 | 6E-150 | 379/424 | 89 | 5/424 | 1 | Plus | Plus | Bacteria; Bacteroidetes; Bacteroidia; Bacteroidales; Barnesiellaceae; Barnesiella; Barnesiella intestinihominis | Barnesiella intestinihominis | Barnesiella | Barnesiellaceae | Bacteroidales | Bacteroidia | Bacteroidetes | | Bacteria | |
| ASV18 | 402 | 2 | 1 | 402 | 100 | NR_042144.1 Clostridium butyricum strain VPI3266 16S ribosomal RNA, partial sequence | NR_042144.1 | 1425 | 338 | 739 | 28.21 | 743.5 | 0 | 402/402 | 100 | 0/402 | 0 | Plus | Plus | Bacteria; Firmicutes; Clostridia; Eubacteriales; Clostridiaceae; Clostridium; Clostridium butyricum | Clostridium butyricum | Clostridium | Clostridiaceae | Eubacteriales | Clostridia | Firmicutes | | Bacteria | |
| ASV19 | 427 | 2 | 1 | 427 | 100 | NR_029106.1 Lactobacillus delbrueckii subsp. indicus strain NCC725 16S ribosomal RNA, partial sequence | NR_029106.1 | 1515 | 348 | 774 | 28.18 | 789.6 | 0 | 427/427 | 100 | 0/427 | 0 | Plus | Plus | Bacteria; Firmicutes; Bacilli; Lactobacillales; Lactobacillaceae; Lactobacillus; Lactobacillus delbrueckii | Lactobacillus delbrueckii | Lactobacillus | Lactobacillaceae | Lactobacillales | Bacilli | Firmicutes | | Bacteria | |
| ASV20 | 427 | 1 | 1 | 427 | 100 | NR_041666.1 Dialister succinatiphilus YIT 11850 16S ribosomal RNA, partial sequence | NR_041666.1 | 1521 | 353 | 779 | 28.07 | 717.6 | 0 | 415/428 | 96 | 2/428 | 0 | Plus | Plus | Bacteria; Firmicutes; Negativicutes; Veillonellales; Veillonellaceae; Dialister; Dialister succinatiphilus | Dialister succinatiphilus | Dialister | Veillonellaceae | Veillonellales | Negativicutes | Firmicutes | | Bacteria | |
| ASV21 | 426 | 1 | 1 | 425 | 99.77 | NR_027526.1 Catenibacterium mitsuokai strain DSM 15897 16S ribosomal RNA, partial sequence | NR_027526.1 | 1485 | 345 | 767 | 28.48 | 717.6 | 0 | 413/425 | 97 | 2/425 | 0 | Plus | Plus | Bacteria; Firmicutes; Erysipelotrichia; Erysipelotrichales; Coprobacillaceae; Catenibacterium; Catenibacterium mitsuokai | Catenibacterium mitsuokai | Catenibacterium | Coprobacillaceae | Erysipelotrichales | Erysipelotrichia | Firmicutes | | Bacteria | |
| ASV23 | 427 | 5 | 1 | 427 | 100 | NR_119109.1 Pseudescherichia vulneris strain ATCC 33821 16S ribosomal RNA, partial sequence | NR_119109.1 | 1443 | 344 | 770 | 29.59 | 789.6 | 0 | 427/427 | 100 | 0/427 | 0 | Plus | Plus | Bacteria; Proteobacteria; Gammaproteobacteria; Enterobacterales; Enterobacteriaceae; Pseudescherichia; Pseudescherichia vulneris | Pseudescherichia vulneris | Pseudescherichia | Enterobacteriaceae | Enterobacterales | Gammaproteobacteria | Proteobacteria | | Bacteria | |
| ASV24 | 426 | 2 | 1 | 426 | 100 | NR_025333.1 Leminorella grimontii strain 81H-380. 16S ribosomal RNA, partial sequence | NR_025333.1 | 1482 | 321 | 747 | 28.81 | 472 | 1E-132 | 376/433 | 86 | 13/433 | 3 | Plus | Plus | Bacteria; Proteobacteria; Gammaproteobacteria; Enterobacterales; Budviciaceae; Leminorella; Leminorella grimontii | Leminorella grimontii | Leminorella | Budviciaceae | Enterobacterales | Gammaproteobacteria | Proteobacteria | | Bacteria | |
| ASV25 | 427 | 1 | 1 | 427 | 100 | NR_025131.1 Treponema parvum strain OMZ 833 16S ribosomal RNA, partial sequence | NR_025131.1 | 1485 | 341 | 767 | 28.75 | 523.7 | 3E-148 | 380/428 | 88 | 2/428 | 0 | Plus | Plus | Bacteria; Spirochaetes; Spirochaetia; Spirochaetales; Spirochaetaceae; Treponema; Treponema parvum | Treponema parvum | Treponema | Spirochaetaceae | Spirochaetales | Spirochaetia | Spirochaetes | | Bacteria | |
| ASV26 | 422 | 2 | 1 | 422 | 100 | NR_113119.1 Prevotella oris strain JCM 8540 16S ribosomal RNA, partial sequence | NR_113119.1 | 1481 | 345 | 766 | 28.49 | 586.5 | 3E-167 | 388/423 | 91 | 2/423 | 0 | Plus | Plus | Bacteria; Bacteroidetes; Bacteroidia; Bacteroidales; Prevotellaceae; Prevotella; Prevotella oris | Prevotella oris | Prevotella | Prevotellaceae | Bacteroidales | Bacteroidia | Bacteroidetes | | Bacteria | |
| ASV27 | 401 | 1 | 1 | 401 | 100 | NR_144740.1 Romboutsia timonensis strain DR1 16S ribosomal RNA, partial sequence | NR_144740.1 | 1466 | 338 | 738 | 27.35 | 741.6 | 0 | 401/401 | 100 | 0/401 | 0 | Plus | Plus | Bacteria; Firmicutes; Clostridia; Eubacteriales; Peptostreptococcaceae; Romboutsia; Romboutsia timonensis | Romboutsia timonensis | Romboutsia | Peptostreptococcaceae | Eubacteriales | Clostridia | Firmicutes | | Bacteria | |
| ASV28 | 422 | 4 | 1 | 422 | 100 | NR_044864.1 Hallella seregens ATCC 51272 16S ribosomal RNA, partial sequence | NR_044864.1 | 1463 | 335 | 756 | 28.84 | 614.2 | 2E-175 | 392/422 | 92 | 0/422 | 0 | Plus | Plus | Bacteria; Bacteroidetes; Bacteroidia; Bacteroidales; Prevotellaceae; Prevotella; Hallella seregens | Hallella seregens | Prevotella | Prevotellaceae | Bacteroidales | Bacteroidia | Bacteroidetes | | Bacteria | |
| ASV29 | 402 | 2 | 1 | 402 | 100 | NR_125571.1 Oribacterium asaccharolyticum ACB7 16S ribosomal RNA, partial sequence | NR_125571.1 | 1436 | 332 | 733 | 27.99 | 605 | 9E-173 | 379/404 | 93 | 4/404 | 0 | Plus | Plus | Bacteria; Firmicutes; Clostridia; Eubacteriales; Lachnospiraceae; Oribacterium; Oribacterium asaccharolyticum | Oribacterium asaccharolyticum | Oribacterium | Lachnospiraceae | Eubacteriales | Clostridia | Firmicutes | | Bacteria | |
| ASV30 | 427 | 1 | 1 | 427 | 100 | NR_029348.1 Treponema brennaborense strain DD5/3 16S ribosomal RNA, partial sequence | NR_029348.1 | 1497 | 305 | 732 | 28.59 | 630.8 | 2E-180 | 399/428 | 93 | 1/428 | 0 | Plus | Plus | Bacteria; Spirochaetes; Spirochaetia; Spirochaetales; Spirochaetaceae; Treponema; Treponema brennaborense | Treponema brennaborense | Treponema | Spirochaetaceae | Spirochaetales | Spirochaetia | Spirochaetes | | Bacteria | |
| ASV31 | 421 | 2 | 1 | 421 | 100 | NR_113073.1 Barnesiella intestinihominis strain JCM 15079 16S ribosomal RNA, partial sequence | NR_113073.1 | 1481 | 346 | 767 | 28.49 | 529.3 | 6E-150 | 379/424 | 89 | 5/424 | 1 | Plus | Plus | Bacteria; Bacteroidetes; Bacteroidia; Bacteroidales; Barnesiellaceae; Barnesiella; Barnesiella intestinihominis | Barnesiella intestinihominis | Barnesiella | Barnesiellaceae | Bacteroidales | Bacteroidia | Bacteroidetes | | Bacteria | |
| ASV33 | 405 | 1 | 1 | 405 | 100 | NR_026476.1 Succinivibrio dextrinosolvens strain 0554 16S ribosomal RNA, partial sequence | NR_026476.1 | 1463 | 331 | 732 | 27.48 | 684.4 | 0 | 394/405 | 97 | 3/405 | 0 | Plus | Plus | Bacteria; Proteobacteria; Gammaproteobacteria; Aeromonadales; Succinivibrionaceae; Succinivibrio; Succinivibrio dextrinosolvens | Succinivibrio dextrinosolvens | Succinivibrio | Succinivibrionaceae | Aeromonadales | Gammaproteobacteria | Proteobacteria | | Bacteria | |
| ASV34 | 421 | 2 | 1 | 421 | 100 | NR_113073.1 Barnesiella intestinihominis strain JCM 15079 16S ribosomal RNA, partial sequence | NR_113073.1 | 1481 | 346 | 767 | 28.49 | 534.8 | 1E-151 | 380/424 | 89 | 5/424 | 1 | Plus | Plus | Bacteria; Bacteroidetes; Bacteroidia; Bacteroidales; Barnesiellaceae; Barnesiella; Barnesiella intestinihominis | Barnesiella intestinihominis | Barnesiella | Barnesiellaceae | Bacteroidales | Bacteroidia | Bacteroidetes | | Bacteria | |
| ASV35 | 402 | 2 | 1 | 402 | 100 | NR_118674.1 Eubacterium ramulus ATCC 29099 16S ribosomal RNA, partial sequence | NR_118674.1 | 1474 | 373 | 774 | 27.27 | 599.4 | 4E-171 | 373/402 | 92 | 0/402 | 0 | Plus | Plus | Bacteria; Firmicutes; Clostridia; Eubacteriales; Eubacteriaceae; Eubacterium; Eubacterium ramulus | Eubacterium ramulus | Eubacterium | Eubacteriaceae | Eubacteriales | Clostridia | Firmicutes | | Bacteria | |
| ASV36 | 402 | 1 | 1 | 402 | 100 | NR_134005.1 Clostridium bornimense strain M2/40 16S ribosomal RNA, partial sequence | NR_134005.1 | 1363 | 284 | 685 | 29.49 | 743.5 | 0 | 402/402 | 100 | 0/402 | 0 | Plus | Plus | Bacteria; Firmicutes; Clostridia; Eubacteriales; Clostridiaceae; Clostridium; Clostridium bornimense | Clostridium bornimense | Clostridium | Clostridiaceae | Eubacteriales | Clostridia | Firmicutes | | Bacteria | |
| ASV37 | 404 | 1 | 1 | 404 | 100 | NR_118156.1 Oscillibacter ruminantium GH1 16S ribosomal RNA, partial sequence | NR_118156.1 | 1409 | 321 | 724 | 28.67 | 608.7 | 7E-174 | 380/405 | 93 | 2/405 | 0 | Plus | Plus | Bacteria; Firmicutes; Clostridia; Eubacteriales; Oscillospiraceae; Oscillibacter; Oscillibacter ruminantium | Oscillibacter ruminantium | Oscillibacter | Oscillospiraceae | Eubacteriales | Clostridia | Firmicutes | | Bacteria | |
| ASV38 | 404 | 1 | 1 | 404 | 100 | NR_025930.1 Ruminococcus bromii strain ATCC 27255 16S ribosomal RNA, partial sequence | NR_025930.1 | 1380 | 313 | 716 | 29.28 | 708.4 | 0 | 398/405 | 98 | 2/405 | 0 | Plus | Plus | Bacteria; Firmicutes; Clostridia; Eubacteriales; Oscillospiraceae; Ruminococcus; Ruminococcus bromii | Ruminococcus bromii | Ruminococcus | Oscillospiraceae | Eubacteriales | Clostridia | Firmicutes | | Bacteria | |
| ASV39 | 404 | 1 | 1 | 404 | 100 | NR_044490.1 Butyricicoccus pullicaecorum strain 25-3 16S ribosomal RNA, partial sequence | NR_044490.1 | 1482 | 366 | 770 | 27.33 | 625.3 | 7E-179 | 384/406 | 94 | 3/406 | 0 | Plus | Plus | Bacteria; Firmicutes; Clostridia; Eubacteriales; Clostridiaceae; Butyricicoccus; Butyricicoccus pullicaecorum | Butyricicoccus pullicaecorum | Butyricicoccus | Clostridiaceae | Eubacteriales | Clostridia | Firmicutes | | Bacteria | |
| ASV40 | 422 | 1 | 1 | 422 | 100 | NR_113122.1 Prevotella paludivivens strain JCM 13650 16S ribosomal RNA, partial sequence | NR_113122.1 | 1491 | 356 | 777 | 28.3 | 569.9 | 3E-162 | 384/422 | 90 | 0/422 | 0 | Plus | Plus | Bacteria; Bacteroidetes; Bacteroidia; Bacteroidales; Prevotellaceae; Prevotella; Prevotella paludivivens | Prevotella paludivivens | Prevotella | Prevotellaceae | Bacteroidales | Bacteroidia | Bacteroidetes | | Bacteria | |
| ASV41 | 423 | 1 | 1 | 423 | 100 | NR_041954.1 Prevotella brevis strain GA33 16S ribosomal RNA, partial sequence | NR_041954.1 | 1493 | 356 | 777 | 28.27 | 549.6 | 5E-156 | 381/424 | 89 | 3/424 | 0 | Plus | Plus | Bacteria; Bacteroidetes; Bacteroidia; Bacteroidales; Prevotellaceae; Prevotella; Prevotella brevis | Prevotella brevis | Prevotella | Prevotellaceae | Bacteroidales | Bacteroidia | Bacteroidetes | | Bacteria | |
| ASV43 | 422 | 2 | 1 | 422 | 100 | NR_113119.1 Prevotella oris strain JCM 8540 16S ribosomal RNA, partial sequence | NR_113119.1 | 1481 | 345 | 766 | 28.49 | 630.8 | 2E-180 | 396/423 | 93 | 2/423 | 0 | Plus | Plus | Bacteria; Bacteroidetes; Bacteroidia; Bacteroidales; Prevotellaceae; Prevotella; Prevotella oris | Prevotella oris | Prevotella | Prevotellaceae | Bacteroidales | Bacteroidia | Bacteroidetes | | Bacteria | |
| ASV44 | 427 | 4 | 1 | 427 | 100 | NR_042802.1 Lactobacillus ultunensis strain Kx146C1 16S ribosomal RNA, partial sequence | NR_042802.1 | 1550 | 370 | 796 | 27.55 | 784.1 | 0 | 426/427 | 99 | 0/427 | 0 | Plus | Plus | Bacteria; Firmicutes; Bacilli; Lactobacillales; Lactobacillaceae; Lactobacillus; Lactobacillus ultunensis | Lactobacillus ultunensis | Lactobacillus | Lactobacillaceae | Lactobacillales | Bacilli | Firmicutes | | Bacteria | |
| ASV45 | 402 | 1 | 1 | 402 | 100 | NR_044054.1 Blautia wexlerae DSM 19850 16S ribosomal RNA, partial sequence | NR_044054.1 | 1438 | 314 | 715 | 27.96 | 743.5 | 0 | 402/402 | 100 | 0/402 | 0 | Plus | Plus | Bacteria; Firmicutes; Clostridia; Eubacteriales; Lachnospiraceae; Blautia; Blautia wexlerae | Blautia wexlerae | Blautia | Lachnospiraceae | Eubacteriales | Clostridia | Firmicutes | | Bacteria | |
| ASV46 | 421 | 2 | 1 | 421 | 100 | NR_113073.1 Barnesiella intestinihominis strain JCM 15079 16S ribosomal RNA, partial sequence | NR_113073.1 | 1481 | 346 | 767 | 28.49 | 529.3 | 6E-150 | 379/424 | 89 | 5/424 | 1 | Plus | Plus | Bacteria; Bacteroidetes; Bacteroidia; Bacteroidales; Barnesiellaceae; Barnesiella; Barnesiella intestinihominis | Barnesiella intestinihominis | Barnesiella | Barnesiellaceae | Bacteroidales | Bacteroidia | Bacteroidetes | | Bacteria | |
| ASV47 | 422 | 1 | 1 | 422 | 100 | NR_037088.1 Alloprevotella tannerae strain VPI N14B 16S ribosomal RNA gene, partial sequence | NR_037088.1 | 1453 | 338 | 759 | 29.04 | 459.1 | 8E-129 | 370/428 | 86 | 12/428 | 2 | Plus | Plus | Bacteria; Bacteroidetes; Bacteroidia; Bacteroidales; Prevotellaceae; Alloprevotella; Alloprevotella tannerae | Alloprevotella tannerae | Alloprevotella | Prevotellaceae | Bacteroidales | Bacteroidia | Bacteroidetes | | Bacteria | |
| ASV48 | 427 | 1 | 1 | 427 | 100 | NR_041780.1 Streptococcus hyointestinalis strain ATCC 49169 16S ribosomal RNA, partial sequence | NR_041780.1 | 1439 | 348 | 774 | 29.67 | 789.6 | 0 | 427/427 | 100 | 0/427 | 0 | Plus | Plus | Bacteria; Firmicutes; Bacilli; Lactobacillales; Streptococcaceae; Streptococcus; Streptococcus hyointestinalis | Streptococcus hyointestinalis | Streptococcus | Streptococcaceae | Lactobacillales | Bacilli | Firmicutes | | Bacteria | |
| ASV49 | 402 | 1 | 1 | 402 | 100 | NR_028961.1 Faecalibacterium prausnitzii strain ATCC 27768 16S ribosomal RNA, partial sequence | NR_028961.1 | 1462 | 344 | 745 | 27.5 | 737.9 | 0 | 401/402 | 99 | 0/402 | 0 | Plus | Plus | Bacteria; Firmicutes; Clostridia; Eubacteriales; Oscillospiraceae; Faecalibacterium; Faecalibacterium prausnitzii | Faecalibacterium prausnitzii | Faecalibacterium | Oscillospiraceae | Eubacteriales | Clostridia | Firmicutes | | Bacteria | |
| ASV50 | 404 | 1 | 1 | 404 | 100 | NR_144611.1 Flintibacter butyricus strain BLS21 16S ribosomal RNA, partial sequence | NR_144611.1 | 808 | 299 | 703 | 50.12 | 586.5 | 3E-167 | 376/405 | 92 | 1/405 | 0 | Plus | Plus | Bacteria; Firmicutes; Clostridia; Eubacteriales; ; Flintibacter; Flintibacter butyricus | Flintibacter butyricus | Flintibacter | | Eubacteriales | Clostridia | Firmicutes | | Bacteria | |
| ASV51 | 422 | 1 | 1 | 422 | 100 | NR_113122.1 Prevotella paludivivens strain JCM 13650 16S ribosomal RNA, partial sequence | NR_113122.1 | 1491 | 356 | 777 | 28.3 | 575.4 | 7E-164 | 385/422 | 91 | 0/422 | 0 | Plus | Plus | Bacteria; Bacteroidetes; Bacteroidia; Bacteroidales; Prevotellaceae; Prevotella; Prevotella paludivivens | Prevotella paludivivens | Prevotella | Prevotellaceae | Bacteroidales | Bacteroidia | Bacteroidetes | | Bacteria | |
| ASV52 | 405 | 1 | 1 | 405 | 100 | NR_118554.1 Intestinimonas butyriciproducens strain SRB-521-5-I 16S ribosomal RNA, partial sequence | NR_118554.1 | 1475 | 346 | 750 | 27.46 | 593.9 | 2E-169 | 377/405 | 93 | 0/405 | 0 | Plus | Plus | Bacteria; Firmicutes; Clostridia; Eubacteriales; ; Intestinimonas; Intestinimonas butyriciproducens | Intestinimonas butyriciproducens | Intestinimonas | | Eubacteriales | Clostridia | Firmicutes | | Bacteria | |
| ASV53 | 422 | 1 | 1 | 422 | 100 | NR_041342.1 Parabacteroides distasonis strain JCM 5825 16S ribosomal RNA, partial sequence | NR_041342.1 | 1488 | 353 | 774 | 28.36 | 542.2 | 8E-154 | 380/423 | 89 | 2/423 | 0 | Plus | Plus | Bacteria; Bacteroidetes; Bacteroidia; Bacteroidales; Tannerellaceae; Parabacteroides; Parabacteroides distasonis | Parabacteroides distasonis | Parabacteroides | Tannerellaceae | Bacteroidales | Bacteroidia | Bacteroidetes | | Bacteria | |
| ASV54 | 402 | 1 | 1 | 402 | 100 | NR_042832.1 Roseburia faecis strain M72/1 16S ribosomal RNA, partial sequence | NR_042832.1 | 1456 | 347 | 749 | 27.68 | 721.3 | 0 | 399/403 | 99 | 1/403 | 0 | Plus | Plus | Bacteria; Firmicutes; Clostridia; Eubacteriales; Lachnospiraceae; Roseburia; Roseburia faecis | Roseburia faecis | Roseburia | Lachnospiraceae | Eubacteriales | Clostridia | Firmicutes | | Bacteria | |
| ASV55 | 427 | 1 | 1 | 427 | 100 | NR_028816.1 Turicibacter sanguinis strain MOL361 16S ribosomal RNA, partial sequence | NR_028816.1 | 1508 | 327 | 753 | 28.32 | 767.5 | 0 | 423/427 | 99 | 0/427 | 0 | Plus | Plus | Bacteria; Firmicutes; Erysipelotrichia; Erysipelotrichales; Turicibacteraceae; Turicibacter; Turicibacter sanguinis | Turicibacter sanguinis | Turicibacter | Turicibacteraceae | Erysipelotrichales | Erysipelotrichia | Firmicutes | | Bacteria | |
| ASV57 | 411 | 2 | 1 | 411 | 100 | NR_104968.1 Bifidobacterium thermophilum strain AS 1.2235 16S ribosomal RNA, partial sequence | NR_104968.1 | 1451 | 297 | 707 | 28.33 | 760.1 | 0 | 411/411 | 100 | 0/411 | 0 | Plus | Plus | Bacteria; Actinobacteria; Actinomycetia; Bifidobacteriales; Bifidobacteriaceae; Bifidobacterium; Bifidobacterium thermophilum | Bifidobacterium thermophilum | Bifidobacterium | Bifidobacteriaceae | Bifidobacteriales | Actinomycetia | Actinobacteria | | Bacteria | |
| ASV58 | 422 | 1 | 1 | 422 | 100 | NR_041342.1 Parabacteroides distasonis strain JCM 5825 16S ribosomal RNA, partial sequence | NR_041342.1 | 1488 | 353 | 774 | 28.36 | 553.3 | 4E-157 | 382/423 | 90 | 2/423 | 0 | Plus | Plus | Bacteria; Bacteroidetes; Bacteroidia; Bacteroidales; Tannerellaceae; Parabacteroides; Parabacteroides distasonis | Parabacteroides distasonis | Parabacteroides | Tannerellaceae | Bacteroidales | Bacteroidia | Bacteroidetes | | Bacteria | |
| ASV59 | 422 | 1 | 1 | 422 | 100 | NR_113411.1 Prevotella copri DSM 18205 strain JCM 13464 16S ribosomal RNA, partial sequence | NR_113411.1 | 1493 | 358 | 779 | 28.27 | 730.5 | 0 | 413/422 | 97 | 0/422 | 0 | Plus | Plus | Bacteria; Bacteroidetes; Bacteroidia; Bacteroidales; Prevotellaceae; Prevotella; Prevotella copri | Prevotella copri | Prevotella | Prevotellaceae | Bacteroidales | Bacteroidia | Bacteroidetes | | Bacteria | |
| ASV60 | 421 | 2 | 1 | 421 | 100 | NR_113073.1 Barnesiella intestinihominis strain JCM 15079 16S ribosomal RNA, partial sequence | NR_113073.1 | 1481 | 346 | 767 | 28.49 | 529.3 | 6E-150 | 379/424 | 89 | 5/424 | 1 | Plus | Plus | Bacteria; Bacteroidetes; Bacteroidia; Bacteroidales; Barnesiellaceae; Barnesiella; Barnesiella intestinihominis | Barnesiella intestinihominis | Barnesiella | Barnesiellaceae | Bacteroidales | Bacteroidia | Bacteroidetes | | Bacteria | |
| ASV61 | 402 | 1 | 1 | 402 | 100 | NR_104846.1 Gemmiger formicilis strain X2-56 16S ribosomal RNA, partial sequence | NR_104846.1 | 1426 | 305 | 706 | 28.19 | 743.5 | 0 | 402/402 | 100 | 0/402 | 0 | Plus | Plus | Bacteria; Firmicutes; Clostridia; Eubacteriales; ; Gemmiger; Gemmiger formicilis | Gemmiger formicilis | Gemmiger | | Eubacteriales | Clostridia | Firmicutes | | Bacteria | |
| ASV62 | 404 | 1 | 1 | 404 | 100 | NR_118156.1 Oscillibacter ruminantium GH1 16S ribosomal RNA, partial sequence | NR_118156.1 | 1409 | 321 | 724 | 28.67 | 608.7 | 7E-174 | 380/405 | 93 | 2/405 | 0 | Plus | Plus | Bacteria; Firmicutes; Clostridia; Eubacteriales; Oscillospiraceae; Oscillibacter; Oscillibacter ruminantium | Oscillibacter ruminantium | Oscillibacter | Oscillospiraceae | Eubacteriales | Clostridia | Firmicutes | | Bacteria | |
| ASV63 | 422 | 1 | 1 | 422 | 100 | NR_113411.1 Prevotella copri DSM 18205 strain JCM 13464 16S ribosomal RNA, partial sequence | NR_113411.1 | 1493 | 358 | 779 | 28.27 | 769.3 | 0 | 420/422 | 99 | 0/422 | 0 | Plus | Plus | Bacteria; Bacteroidetes; Bacteroidia; Bacteroidales; Prevotellaceae; Prevotella; Prevotella copri | Prevotella copri | Prevotella | Prevotellaceae | Bacteroidales | Bacteroidia | Bacteroidetes | | Bacteria | |
| ASV64 | 404 | 1 | 1 | 404 | 100 | NR_144611.1 Flintibacter butyricus strain BLS21 16S ribosomal RNA, partial sequence | NR_144611.1 | 808 | 299 | 703 | 50.12 | 586.5 | 3E-167 | 376/405 | 92 | 1/405 | 0 | Plus | Plus | Bacteria; Firmicutes; Clostridia; Eubacteriales; ; Flintibacter; Flintibacter butyricus | Flintibacter butyricus | Flintibacter | | Eubacteriales | Clostridia | Firmicutes | | Bacteria | |
| ASV65 | 403 | 1 | 1 | 403 | 100 | NR_042832.1 Roseburia faecis strain M72/1 16S ribosomal RNA, partial sequence | NR_042832.1 | 1456 | 347 | 749 | 27.68 | 745.3 | 0 | 403/403 | 100 | 0/403 | 0 | Plus | Plus | Bacteria; Firmicutes; Clostridia; Eubacteriales; Lachnospiraceae; Roseburia; Roseburia faecis | Roseburia faecis | Roseburia | Lachnospiraceae | Eubacteriales | Clostridia | Firmicutes | | Bacteria | |
| ASV66 | 408 | 1 | 1 | 408 | 100 | NR_116936.1 Olsenella umbonata strain lac31 16S ribosomal RNA, partial sequence | NR_116936.1 | 1428 | 328 | 735 | 28.57 | 754.6 | 0 | 408/408 | 100 | 0/408 | 0 | Plus | Plus | Bacteria; Actinobacteria; Coriobacteriia; Coriobacteriales; Atopobiaceae; Olsenella; Olsenella umbonata | Olsenella umbonata | Olsenella | Atopobiaceae | Coriobacteriales | Coriobacteriia | Actinobacteria | | Bacteria | |
| ASV67 | 422 | 1 | 1 | 422 | 100 | NR_041364.1 Prevotella stercorea DSM 18206 strain CB35 16S ribosomal RNA, partial sequence | NR_041364.1 | 1488 | 354 | 775 | 28.36 | 741.6 | 0 | 415/422 | 98 | 0/422 | 0 | Plus | Plus | Bacteria; Bacteroidetes; Bacteroidia; Bacteroidales; Prevotellaceae; Prevotella; Prevotella stercorea | Prevotella stercorea | Prevotella | Prevotellaceae | Bacteroidales | Bacteroidia | Bacteroidetes | | Bacteria | |
| ASV68 | 422 | 1 | 1 | 422 | 100 | NR_113411.1 Prevotella copri DSM 18205 strain JCM 13464 16S ribosomal RNA, partial sequence | NR_113411.1 | 1493 | 358 | 779 | 28.27 | 725 | 0 | 412/422 | 97 | 0/422 | 0 | Plus | Plus | Bacteria; Bacteroidetes; Bacteroidia; Bacteroidales; Prevotellaceae; Prevotella; Prevotella copri | Prevotella copri | Prevotella | Prevotellaceae | Bacteroidales | Bacteroidia | Bacteroidetes | | Bacteria | |
| ASV69 | 403 | 5 | 1 | 403 | 100 | NR_118521.1 Campylobacter lanienae NCTC 13004 strain CCUG 44467 16S ribosomal RNA, partial sequence | NR_118521.1 | 1434 | 328 | 730 | 28.1 | 701 | 0 | 395/403 | 98 | 0/403 | 0 | Plus | Plus | Bacteria; Proteobacteria; Epsilonproteobacteria; Campylobacterales; Campylobacteraceae; Campylobacter; Campylobacter lanienae | Campylobacter lanienae | Campylobacter | Campylobacteraceae | Campylobacterales | Epsilonproteobacteria | Proteobacteria | | Bacteria | |
| ASV70 | 404 | 1 | 1 | 404 | 100 | NR_118156.1 Oscillibacter ruminantium GH1 16S ribosomal RNA, partial sequence | NR_118156.1 | 1409 | 321 | 724 | 28.67 | 597.6 | 2E-170 | 378/405 | 93 | 2/405 | 0 | Plus | Plus | Bacteria; Firmicutes; Clostridia; Eubacteriales; Oscillospiraceae; Oscillibacter; Oscillibacter ruminantium | Oscillibacter ruminantium | Oscillibacter | Oscillospiraceae | Eubacteriales | Clostridia | Firmicutes | | Bacteria | |
| ASV71 | 422 | 1 | 1 | 422 | 100 | NR_113411.1 Prevotella copri DSM 18205 strain JCM 13464 16S ribosomal RNA, partial sequence | NR_113411.1 | 1493 | 358 | 779 | 28.27 | 725 | 0 | 412/422 | 97 | 0/422 | 0 | Plus | Plus | Bacteria; Bacteroidetes; Bacteroidia; Bacteroidales; Prevotellaceae; Prevotella; Prevotella copri | Prevotella copri | Prevotella | Prevotellaceae | Bacteroidales | Bacteroidia | Bacteroidetes | | Bacteria | |
| ASV72 | 422 | 2 | 1 | 422 | 100 | NR_113119.1 Prevotella oris strain JCM 8540 16S ribosomal RNA, partial sequence | NR_113119.1 | 1481 | 345 | 766 | 28.49 | 586.5 | 3E-167 | 388/423 | 91 | 2/423 | 0 | Plus | Plus | Bacteria; Bacteroidetes; Bacteroidia; Bacteroidales; Prevotellaceae; Prevotella; Prevotella oris | Prevotella oris | Prevotella | Prevotellaceae | Bacteroidales | Bacteroidia | Bacteroidetes | | Bacteria | |
| ASV73 | 402 | 1 | 1 | 402 | 100 | NR_104846.1 Gemmiger formicilis strain X2-56 16S ribosomal RNA, partial sequence | NR_104846.1 | 1426 | 305 | 706 | 28.19 | 715.8 | 0 | 397/402 | 98 | 0/402 | 0 | Plus | Plus | Bacteria; Firmicutes; Clostridia; Eubacteriales; ; Gemmiger; Gemmiger formicilis | Gemmiger formicilis | Gemmiger | | Eubacteriales | Clostridia | Firmicutes | | Bacteria | |
| ASV74 | 411 | 1 | 1 | 411 | 100 | NR_036854.2 Bifidobacterium boum strain RU917 16S ribosomal RNA, partial sequence | NR_036854.2 | 1514 | 343 | 753 | 27.15 | 760.1 | 0 | 411/411 | 100 | 0/411 | 0 | Plus | Plus | Bacteria; Actinobacteria; Actinomycetia; Bifidobacteriales; Bifidobacteriaceae; Bifidobacterium; Bifidobacterium boum | Bifidobacterium boum | Bifidobacterium | Bifidobacteriaceae | Bifidobacteriales | Actinomycetia | Actinobacteria | | Bacteria | |
| ASV75 | 405 | 1 | 1 | 405 | 100 | NR_144611.1 Flintibacter butyricus strain BLS21 16S ribosomal RNA, partial sequence | NR_144611.1 | 808 | 299 | 703 | 50.12 | 599.4 | 4E-171 | 378/405 | 93 | 0/405 | 0 | Plus | Plus | Bacteria; Firmicutes; Clostridia; Eubacteriales; ; Flintibacter; Flintibacter butyricus | Flintibacter butyricus | Flintibacter | | Eubacteriales | Clostridia | Firmicutes | | Bacteria | |
| ASV76 | 422 | 1 | 1 | 422 | 100 | NR_041364.1 Prevotella stercorea DSM 18206 strain CB35 16S ribosomal RNA, partial sequence | NR_041364.1 | 1488 | 354 | 775 | 28.36 | 747.2 | 0 | 416/422 | 98 | 0/422 | 0 | Plus | Plus | Bacteria; Bacteroidetes; Bacteroidia; Bacteroidales; Prevotellaceae; Prevotella; Prevotella stercorea | Prevotella stercorea | Prevotella | Prevotellaceae | Bacteroidales | Bacteroidia | Bacteroidetes | | Bacteria | |
| ASV77 | 427 | 1 | 1 | 427 | 100 | NR_117574.1 Lactobacillus johnsonii strain CIP 103620 16S ribosomal RNA, partial sequence | NR_117574.1 | 1543 | 366 | 792 | 27.67 | 789.6 | 0 | 427/427 | 100 | 0/427 | 0 | Plus | Plus | Bacteria; Firmicutes; Bacilli; Lactobacillales; Lactobacillaceae; Lactobacillus; Lactobacillus johnsonii | Lactobacillus johnsonii | Lactobacillus | Lactobacillaceae | Lactobacillales | Bacilli | Firmicutes | | Bacteria | |
| ASV78 | 402 | 1 | 1 | 402 | 100 | NR_029097.1 Oribacterium sinus strain AIP 354.02 16S ribosomal RNA, partial sequence | NR_029097.1 | 1374 | 308 | 709 | 29.26 | 660.4 | 0 | 387/402 | 96 | 0/402 | 0 | Plus | Plus | Bacteria; Firmicutes; Clostridia; Eubacteriales; Lachnospiraceae; Oribacterium; Oribacterium sinus | Oribacterium sinus | Oribacterium | Lachnospiraceae | Eubacteriales | Clostridia | Firmicutes | | Bacteria | |
| ASV79 | 428 | 1 | 1 | 428 | 100 | NR_104781.1 Treponema bryantii strain RUS-1 16S ribosomal RNA, partial sequence | NR_104781.1 | 1535 | 351 | 778 | 27.88 | 658.5 | 0 | 405/429 | 94 | 2/429 | 0 | Plus | Plus | Bacteria; Spirochaetes; Spirochaetia; Spirochaetales; Spirochaetaceae; Treponema; Treponema bryantii | Treponema bryantii | Treponema | Spirochaetaceae | Spirochaetales | Spirochaetia | Spirochaetes | | Bacteria | |
| ASV80 | 426 | 1 | 1 | 426 | 100 | NR_145570.1 Vibrio mexicanus strain CAIM 1540 16S ribosomal RNA, partial sequence | NR_145570.1 | 1436 | 324 | 750 | 29.74 | 484.9 | 1E-136 | 374/429 | 87 | 5/429 | 1 | Plus | Plus | Bacteria; Proteobacteria; Gammaproteobacteria; Vibrionales; Vibrionaceae; Vibrio; Vibrio mexicanus | Vibrio mexicanus | Vibrio | Vibrionaceae | Vibrionales | Gammaproteobacteria | Proteobacteria | | Bacteria | |
| ASV81 | 405 | 1 | 3 | 405 | 99.51 | NR_125464.1 Anaerobacterium chartisolvens strain T-1-35 16S ribosomal RNA, partial sequence | NR_125464.1 | 1451 | 344 | 745 | 27.71 | 472 | 1E-132 | 354/403 | 87 | 1/403 | 0 | Plus | Plus | Bacteria; Firmicutes; Clostridia; Eubacteriales; Oscillospiraceae; Anaerobacterium; Anaerobacterium chartisolvens | Anaerobacterium chartisolvens | Anaerobacterium | Oscillospiraceae | Eubacteriales | Clostridia | Firmicutes | | Bacteria | |
| ASV82 | 426 | 1 | 1 | 426 | 100 | NR_025333.1 Leminorella grimontii strain 81H-380. 16S ribosomal RNA, partial sequence | NR_025333.1 | 1482 | 321 | 747 | 28.81 | 477.6 | 2E-134 | 377/433 | 87 | 13/433 | 3 | Plus | Plus | Bacteria; Proteobacteria; Gammaproteobacteria; Enterobacterales; Budviciaceae; Leminorella; Leminorella grimontii | Leminorella grimontii | Leminorella | Budviciaceae | Enterobacterales | Gammaproteobacteria | Proteobacteria | | Bacteria | |
| ASV83 | 402 | 1 | 1 | 402 | 100 | NR_029239.3 Clostridium chartatabidum strain 163 16S ribosomal RNA, partial sequence | NR_029239.3 | 1460 | 323 | 724 | 27.53 | 710.2 | 0 | 396/402 | 98 | 0/402 | 0 | Plus | Plus | Bacteria; Firmicutes; Clostridia; Eubacteriales; Clostridiaceae; Clostridium; Clostridium chartatabidum | Clostridium chartatabidum | Clostridium | Clostridiaceae | Eubacteriales | Clostridia | Firmicutes | | Bacteria | |
| ASV84 | 422 | 1 | 1 | 422 | 100 | NR_028963.1 Anaerophaga thermohalophila DSM 12881 strain Fru22 16S ribosomal RNA, partial sequence | NR_028963.1 | 1512 | 344 | 766 | 27.98 | 453.5 | 4E-127 | 366/425 | 86 | 5/425 | 1 | Plus | Plus | Bacteria; Bacteroidetes; Bacteroidia; Marinilabiliales; Marinilabiliaceae; Anaerophaga; Anaerophaga thermohalophila | Anaerophaga thermohalophila | Anaerophaga | Marinilabiliaceae | Marinilabiliales | Bacteroidia | Bacteroidetes | | Bacteria | |
| ASV85 | 427 | 1 | 1 | 427 | 100 | NR_042797.1 Treponema berlinense strain 7CPL208 16S ribosomal RNA, partial sequence | NR_042797.1 | 1440 | 275 | 701 | 29.65 | 789.6 | 0 | 427/427 | 100 | 0/427 | 0 | Plus | Plus | Bacteria; Spirochaetes; Spirochaetia; Spirochaetales; Spirochaetaceae; Treponema; Treponema berlinense | Treponema berlinense | Treponema | Spirochaetaceae | Spirochaetales | Spirochaetia | Spirochaetes | | Bacteria | |
| ASV86 | 402 | 1 | 1 | 402 | 100 | NR_037068.1 [Clostridium] xylanolyticum strain DSM 6555 16S ribosomal RNA, partial sequence | NR_037068.1 | 1441 | 358 | 759 | 27.9 | 688.1 | 0 | 393/403 | 97 | 2/403 | 0 | Plus | Plus | Bacteria; Firmicutes; Clostridia; Eubacteriales; Lachnospiraceae; Lacrimispora; Lacrimispora xylanolytica | Lacrimispora xylanolytica | Lacrimispora | Lachnospiraceae | Eubacteriales | Clostridia | Firmicutes | | Bacteria | |
| ASV87 | 402 | 2 | 1 | 402 | 100 | NR_118690.1 [Ruminococcus] gnavus ATCC 29149 16S ribosomal RNA, partial sequence | NR_118690.1 | 1423 | 355 | 756 | 28.25 | 616.1 | 4E-176 | 379/402 | 94 | 0/402 | 0 | Plus | Plus | Bacteria; Firmicutes; Clostridia; Eubacteriales; Lachnospiraceae; Mediterraneibacter; [Ruminococcus] gnavus | [Ruminococcus] gnavus | Mediterraneibacter | Lachnospiraceae | Eubacteriales | Clostridia | Firmicutes | | Bacteria | |
| ASV88 | 402 | 1 | 1 | 402 | 100 | NR_029239.3 Clostridium chartatabidum strain 163 16S ribosomal RNA, partial sequence | NR_029239.3 | 1460 | 323 | 724 | 27.53 | 721.3 | 0 | 398/402 | 99 | 0/402 | 0 | Plus | Plus | Bacteria; Firmicutes; Clostridia; Eubacteriales; Clostridiaceae; Clostridium; Clostridium chartatabidum | Clostridium chartatabidum | Clostridium | Clostridiaceae | Eubacteriales | Clostridia | Firmicutes | | Bacteria | |
| ASV89 | 427 | 1 | 1 | 427 | 100 | NR_112902.1 Phascolarctobacterium succinatutens strain YIT 12067 16S ribosomal RNA, partial sequence | NR_112902.1 | 1610 | 444 | 870 | 26.52 | 761.9 | 0 | 422/427 | 98 | 0/427 | 0 | Plus | Plus | Bacteria; Firmicutes; Negativicutes; Acidaminococcales; Acidaminococcaceae; Phascolarctobacterium; Phascolarctobacterium succinatutens | Phascolarctobacterium succinatutens | Phascolarctobacterium | Acidaminococcaceae | Acidaminococcales | Negativicutes | Firmicutes | | Bacteria | |
| ASV90 | 422 | 1 | 1 | 422 | 100 | NR_113122.1 Prevotella paludivivens strain JCM 13650 16S ribosomal RNA, partial sequence | NR_113122.1 | 1491 | 356 | 777 | 28.3 | 569.9 | 3E-162 | 384/422 | 90 | 0/422 | 0 | Plus | Plus | Bacteria; Bacteroidetes; Bacteroidia; Bacteroidales; Prevotellaceae; Prevotella; Prevotella paludivivens | Prevotella paludivivens | Prevotella | Prevotellaceae | Bacteroidales | Bacteroidia | Bacteroidetes | | Bacteria | |
| ASV91 | 403 | 1 | 1 | 403 | 100 | NR_025930.1 Ruminococcus bromii strain ATCC 27255 16S ribosomal RNA, partial sequence | NR_025930.1 | 1380 | 313 | 716 | 29.28 | 706.5 | 0 | 397/404 | 98 | 1/404 | 0 | Plus | Plus | Bacteria; Firmicutes; Clostridia; Eubacteriales; Oscillospiraceae; Ruminococcus; Ruminococcus bromii | Ruminococcus bromii | Ruminococcus | Oscillospiraceae | Eubacteriales | Clostridia | Firmicutes | | Bacteria | |
| ASV92 | 422 | 1 | 1 | 422 | 100 | NR_041285.1 Prevotella multisaccharivorax strain PPPA20 16S ribosomal RNA, partial sequence | NR_041285.1 | 1491 | 356 | 777 | 28.3 | 619.7 | 3E-177 | 393/422 | 93 | 0/422 | 0 | Plus | Plus | Bacteria; Bacteroidetes; Bacteroidia; Bacteroidales; Prevotellaceae; Prevotella; Prevotella multisaccharivorax | Prevotella multisaccharivorax | Prevotella | Prevotellaceae | Bacteroidales | Bacteroidia | Bacteroidetes | | Bacteria | |
| ASV93 | 422 | 1 | 1 | 422 | 100 | NR_113411.1 Prevotella copri DSM 18205 strain JCM 13464 16S ribosomal RNA, partial sequence | NR_113411.1 | 1493 | 358 | 779 | 28.27 | 780.4 | 0 | 422/422 | 100 | 0/422 | 0 | Plus | Plus | Bacteria; Bacteroidetes; Bacteroidia; Bacteroidales; Prevotellaceae; Prevotella; Prevotella copri | Prevotella copri | Prevotella | Prevotellaceae | Bacteroidales | Bacteroidia | Bacteroidetes | | Bacteria | |
| ASV94 | 402 | 1 | 1 | 402 | 100 | NR_026315.1 Pseudobutyrivibrio ruminis strain DSM 9787 16S ribosomal RNA, partial sequence | NR_026315.1 | 1462 | 334 | 735 | 27.5 | 627.1 | 2E-179 | 382/403 | 94 | 2/403 | 0 | Plus | Plus | Bacteria; Firmicutes; Clostridia; Eubacteriales; Lachnospiraceae; Pseudobutyrivibrio; Pseudobutyrivibrio ruminis | Pseudobutyrivibrio ruminis | Pseudobutyrivibrio | Lachnospiraceae | Eubacteriales | Clostridia | Firmicutes | | Bacteria | |
| ASV95 | 401 | 1 | 1 | 401 | 100 | NR_027573.1 Intestinibacter bartlettii strain WAL 16138 16S ribosomal RNA, partial sequence | NR_027573.1 | 1429 | 320 | 720 | 28.06 | 702.8 | 0 | 394/401 | 98 | 0/401 | 0 | Plus | Plus | Bacteria; Firmicutes; Clostridia; Eubacteriales; Peptostreptococcaceae; Intestinibacter; Intestinibacter bartlettii | Intestinibacter bartlettii | Intestinibacter | Peptostreptococcaceae | Eubacteriales | Clostridia | Firmicutes | | Bacteria | |
| ASV96 | 422 | 1 | 1 | 422 | 100 | NR_041364.1 Prevotella stercorea DSM 18206 strain CB35 16S ribosomal RNA, partial sequence | NR_041364.1 | 1488 | 354 | 775 | 28.36 | 713.9 | 0 | 410/422 | 97 | 0/422 | 0 | Plus | Plus | Bacteria; Bacteroidetes; Bacteroidia; Bacteroidales; Prevotellaceae; Prevotella; Prevotella stercorea | Prevotella stercorea | Prevotella | Prevotellaceae | Bacteroidales | Bacteroidia | Bacteroidetes | | Bacteria | |
| ASV97 | 403 | 1 | 1 | 403 | 100 | NR_042930.1 Acetanaerobacterium elongatum strain Z7 16S ribosomal RNA, partial sequence | NR_042930.1 | 1533 | 383 | 783 | 26.16 | 555.1 | 9E-158 | 369/403 | 91 | 2/403 | 0 | Plus | Plus | Bacteria; Firmicutes; Clostridia; Eubacteriales; Oscillospiraceae; Acetanaerobacterium; Acetanaerobacterium elongatum | Acetanaerobacterium elongatum | Acetanaerobacterium | Oscillospiraceae | Eubacteriales | Clostridia | Firmicutes | | Bacteria | |
| ASV98 | 421 | 2 | 1 | 421 | 100 | NR_113077.1 Paraprevotella clara strain JCM 14859 16S ribosomal RNA, partial sequence | NR_113077.1 | 1480 | 346 | 766 | 28.45 | 512.6 | 6E-145 | 374/422 | 88 | 2/422 | 0 | Plus | Plus | Bacteria; Bacteroidetes; Bacteroidia; Bacteroidales; Prevotellaceae; Paraprevotella; Paraprevotella clara | Paraprevotella clara | Paraprevotella | Prevotellaceae | Bacteroidales | Bacteroidia | Bacteroidetes | | Bacteria | |
| ASV99 | 405 | 1 | 1 | 405 | 100 | NR_026476.1 Succinivibrio dextrinosolvens strain 0554 16S ribosomal RNA, partial sequence | NR_026476.1 | 1463 | 331 | 732 | 27.48 | 689.9 | 0 | 395/405 | 97 | 3/405 | 0 | Plus | Plus | Bacteria; Proteobacteria; Gammaproteobacteria; Aeromonadales; Succinivibrionaceae; Succinivibrio; Succinivibrio dextrinosolvens | Succinivibrio dextrinosolvens | Succinivibrio | Succinivibrionaceae | Aeromonadales | Gammaproteobacteria | Proteobacteria | | Bacteria | |
| ASV100 | 402 | 1 | 1 | 402 | 100 | NR_134005.1 Clostridium bornimense strain M2/40 16S ribosomal RNA, partial sequence | NR_134005.1 | 1363 | 284 | 685 | 29.49 | 732.4 | 0 | 400/402 | 99 | 0/402 | 0 | Plus | Plus | Bacteria; Firmicutes; Clostridia; Eubacteriales; Clostridiaceae; Clostridium; Clostridium bornimense | Clostridium bornimense | Clostridium | Clostridiaceae | Eubacteriales | Clostridia | Firmicutes | | Bacteria | |
| ASV101 | 427 | 2 | 1 | 427 | 100 | NR_029106.1 Lactobacillus delbrueckii subsp. indicus strain NCC725 16S ribosomal RNA, partial sequence | NR_029106.1 | 1515 | 348 | 774 | 28.18 | 767.5 | 0 | 423/427 | 99 | 0/427 | 0 | Plus | Plus | Bacteria; Firmicutes; Bacilli; Lactobacillales; Lactobacillaceae; Lactobacillus; Lactobacillus delbrueckii | Lactobacillus delbrueckii | Lactobacillus | Lactobacillaceae | Lactobacillales | Bacilli | Firmicutes | | Bacteria | |
| ASV102 | 403 | 1 | 1 | 403 | 100 | NR_029160.1 Ruminococcus callidus strain ATCC 27760 16S ribosomal RNA, partial sequence | NR_029160.1 | 1371 | 310 | 711 | 29.32 | 616.1 | 4E-176 | 382/405 | 94 | 5/405 | 1 | Plus | Plus | Bacteria; Firmicutes; Clostridia; Eubacteriales; Oscillospiraceae; Ruminococcus; Ruminococcus callidus | Ruminococcus callidus | Ruminococcus | Oscillospiraceae | Eubacteriales | Clostridia | Firmicutes | | Bacteria | |
| ASV103 | 402 | 3 | 1 | 402 | 100 | NR_156081.1 Kineothrix alysoides strain KNHs209 16S ribosomal RNA, partial sequence | NR_156081.1 | 1411 | 353 | 754 | 28.49 | 665.9 | 0 | 388/402 | 96 | 0/402 | 0 | Plus | Plus | Bacteria; Firmicutes; Clostridia; Eubacteriales; Lachnospiraceae; Kineothrix; Kineothrix alysoides | Kineothrix alysoides | Kineothrix | Lachnospiraceae | Eubacteriales | Clostridia | Firmicutes | | Bacteria | |
| ASV104 | 426 | 1 | 1 | 425 | 99.77 | NR_114696.1 Anaerovibrio lipolyticus DSM 3074 16S ribosomal RNA, partial sequence | NR_114696.1 | 1526 | 372 | 796 | 27.85 | 640.1 | 0 | 398/425 | 93 | 0/425 | 0 | Plus | Plus | Bacteria; Firmicutes; Negativicutes; Selenomonadales; Selenomonadaceae; Anaerovibrio; Anaerovibrio lipolyticus | Anaerovibrio lipolyticus | Anaerovibrio | Selenomonadaceae | Selenomonadales | Negativicutes | Firmicutes | | Bacteria | |
| ASV105 | 402 | 1 | 1 | 402 | 100 | NR_044048.1 Coprococcus comes ATCC 27758 16S ribosomal RNA, partial sequence | NR_044048.1 | 1453 | 345 | 746 | 27.67 | 743.5 | 0 | 402/402 | 100 | 0/402 | 0 | Plus | Plus | Bacteria; Firmicutes; Clostridia; Eubacteriales; Lachnospiraceae; Coprococcus; Coprococcus comes | Coprococcus comes | Coprococcus | Lachnospiraceae | Eubacteriales | Clostridia | Firmicutes | | Bacteria | |
| ASV106 | 406 | 3 | 1 | 406 | 100 | NR_116939.1 Olsenella profusa DSM 13989 strain CCUG 45371 16S ribosomal RNA, partial sequence | NR_116939.1 | 1428 | 328 | 735 | 28.57 | 669.6 | 0 | 393/408 | 96 | 2/408 | 0 | Plus | Plus | Bacteria; Actinobacteria; Coriobacteriia; Coriobacteriales; Atopobiaceae; Olsenella; Olsenella profusa | Olsenella profusa | Olsenella | Atopobiaceae | Coriobacteriales | Coriobacteriia | Actinobacteria | | Bacteria | |
| ASV107 | 422 | 1 | 1 | 422 | 100 | NR_113411.1 Prevotella copri DSM 18205 strain JCM 13464 16S ribosomal RNA, partial sequence | NR_113411.1 | 1493 | 358 | 779 | 28.27 | 713.9 | 0 | 410/422 | 97 | 0/422 | 0 | Plus | Plus | Bacteria; Bacteroidetes; Bacteroidia; Bacteroidales; Prevotellaceae; Prevotella; Prevotella copri | Prevotella copri | Prevotella | Prevotellaceae | Bacteroidales | Bacteroidia | Bacteroidetes | | Bacteria | |
| ASV109 | 422 | 1 | 1 | 422 | 100 | NR_113411.1 Prevotella copri DSM 18205 strain JCM 13464 16S ribosomal RNA, partial sequence | NR_113411.1 | 1493 | 358 | 779 | 28.27 | 603.1 | 3E-172 | 393/425 | 92 | 6/425 | 1 | Plus | Plus | Bacteria; Bacteroidetes; Bacteroidia; Bacteroidales; Prevotellaceae; Prevotella; Prevotella copri | Prevotella copri | Prevotella | Prevotellaceae | Bacteroidales | Bacteroidia | Bacteroidetes | | Bacteria | |
| ASV111 | 403 | 1 | 1 | 403 | 100 | NR_025930.1 Ruminococcus bromii strain ATCC 27255 16S ribosomal RNA, partial sequence | NR_025930.1 | 1380 | 313 | 716 | 29.28 | 619.7 | 3E-177 | 381/404 | 94 | 1/404 | 0 | Plus | Plus | Bacteria; Firmicutes; Clostridia; Eubacteriales; Oscillospiraceae; Ruminococcus; Ruminococcus bromii | Ruminococcus bromii | Ruminococcus | Oscillospiraceae | Eubacteriales | Clostridia | Firmicutes | | Bacteria | |
| ASV112 | 422 | 2 | 1 | 422 | 100 | NR_113411.1 Prevotella copri DSM 18205 strain JCM 13464 16S ribosomal RNA, partial sequence | NR_113411.1 | 1493 | 358 | 779 | 28.27 | 564.3 | 2E-160 | 383/422 | 90 | 0/422 | 0 | Plus | Plus | Bacteria; Bacteroidetes; Bacteroidia; Bacteroidales; Prevotellaceae; Prevotella; Prevotella copri | Prevotella copri | Prevotella | Prevotellaceae | Bacteroidales | Bacteroidia | Bacteroidetes | | Bacteria | |
| ASV113 | 420 | 1 | 1 | 420 | 100 | NR_144616.1 Muribaculum intestinale strain YL27 16S ribosomal RNA, partial sequence | NR_144616.1 | 1460 | 297 | 718 | 28.9 | 507.1 | 3E-143 | 378/427 | 88 | 12/427 | 2 | Plus | Plus | Bacteria; Bacteroidetes; Bacteroidia; Bacteroidales; Muribaculaceae; Muribaculum; Muribaculum intestinale | Muribaculum intestinale | Muribaculum | Muribaculaceae | Bacteroidales | Bacteroidia | Bacteroidetes | | Bacteria | |
| ASV114 | 427 | 1 | 1 | 427 | 100 | NR_024994.1 Lactobacillus mucosae strain S32 16S ribosomal RNA, partial sequence | NR_024994.1 | 1568 | 384 | 810 | 27.23 | 789.6 | 0 | 427/427 | 100 | 0/427 | 0 | Plus | Plus | Bacteria; Firmicutes; Bacilli; Lactobacillales; Lactobacillaceae; Limosilactobacillus; Limosilactobacillus mucosae | Limosilactobacillus mucosae | Limosilactobacillus | Lactobacillaceae | Lactobacillales | Bacilli | Firmicutes | | Bacteria | |
| ASV115 | 402 | 1 | 1 | 402 | 100 | NR_028961.1 Faecalibacterium prausnitzii strain ATCC 27768 16S ribosomal RNA, partial sequence | NR_028961.1 | 1462 | 344 | 745 | 27.5 | 715.8 | 0 | 397/402 | 98 | 0/402 | 0 | Plus | Plus | Bacteria; Firmicutes; Clostridia; Eubacteriales; Oscillospiraceae; Faecalibacterium; Faecalibacterium prausnitzii | Faecalibacterium prausnitzii | Faecalibacterium | Oscillospiraceae | Eubacteriales | Clostridia | Firmicutes | | Bacteria | |
| ASV116 | 426 | 1 | 1 | 426 | 100 | NR_041304.1 Sharpea azabuensis strain ST18 16S ribosomal RNA, partial sequence | NR_041304.1 | 1460 | 325 | 750 | 29.18 | 760.1 | 0 | 421/426 | 98 | 0/426 | 0 | Plus | Plus | Bacteria; Firmicutes; Erysipelotrichia; Erysipelotrichales; Coprobacillaceae; Sharpea; Sharpea azabuensis | Sharpea azabuensis | Sharpea | Coprobacillaceae | Erysipelotrichales | Erysipelotrichia | Firmicutes | | Bacteria | |
| ASV117 | 421 | 2 | 1 | 421 | 100 | NR_113039.1 Solobacterium moorei strain JCM 10645 16S ribosomal RNA, partial sequence | NR_113039.1 | 1486 | 352 | 772 | 28.33 | 573.6 | 3E-163 | 385/422 | 91 | 2/422 | 0 | Plus | Plus | Bacteria; Firmicutes; Erysipelotrichia; Erysipelotrichales; Erysipelotrichaceae; Solobacterium; Solobacterium moorei | Solobacterium moorei | Solobacterium | Erysipelotrichaceae | Erysipelotrichales | Erysipelotrichia | Firmicutes | | Bacteria | |
| ASV118 | 427 | 1 | 1 | 427 | 100 | NR_044111.1 Selenomonas bovis strain WG 16S ribosomal RNA, partial sequence | NR_044111.1 | 1525 | 370 | 796 | 28 | 662.2 | 0 | 405/428 | 94 | 2/428 | 0 | Plus | Plus | Bacteria; Firmicutes; Negativicutes; Selenomonadales; Selenomonadaceae; Selenomonas; Selenomonas bovis | Selenomonas bovis | Selenomonas | Selenomonadaceae | Selenomonadales | Negativicutes | Firmicutes | | Bacteria | |
| ASV119 | 403 | 2 | 1 | 403 | 100 | NR_024948.1 Campylobacter hyointestinalis subsp. lawsonii strain CHY5 16S ribosomal RNA, partial sequence | NR_024948.1 | 1419 | 318 | 720 | 28.4 | 712.1 | 0 | 397/403 | 98 | 0/403 | 0 | Plus | Plus | Bacteria; Proteobacteria; Epsilonproteobacteria; Campylobacterales; Campylobacteraceae; Campylobacter; Campylobacter hyointestinalis | Campylobacter hyointestinalis | Campylobacter | Campylobacteraceae | Campylobacterales | Epsilonproteobacteria | Proteobacteria | | Bacteria | |
| ASV120 | 405 | 1 | 1 | 405 | 100 | NR_144611.1 Flintibacter butyricus strain BLS21 16S ribosomal RNA, partial sequence | NR_144611.1 | 808 | 299 | 703 | 50.12 | 588.4 | 9E-168 | 376/405 | 92 | 0/405 | 0 | Plus | Plus | Bacteria; Firmicutes; Clostridia; Eubacteriales; ; Flintibacter; Flintibacter butyricus | Flintibacter butyricus | Flintibacter | | Eubacteriales | Clostridia | Firmicutes | | Bacteria | |
| ASV121 | 426 | 1 | 1 | 426 | 100 | NR_026205.1 Succiniclasticum ruminis strain SE10 16S ribosomal RNA, partial sequence | NR_026205.1 | 1505 | 354 | 780 | 28.37 | 610.5 | 2E-174 | 396/428 | 92 | 3/428 | 0 | Plus | Plus | Bacteria; Firmicutes; Negativicutes; Acidaminococcales; Acidaminococcaceae; Succiniclasticum; Succiniclasticum ruminis | Succiniclasticum ruminis | Succiniclasticum | Acidaminococcaceae | Acidaminococcales | Negativicutes | Firmicutes | | Bacteria | |
| ASV122 | 402 | 1 | 1 | 402 | 100 | NR_074634.1 [Eubacterium] rectale ATCC 33656 16S ribosomal RNA, partial sequence | NR_074634.1 | 1521 | 367 | 768 | 26.43 | 743.5 | 0 | 402/402 | 100 | 0/402 | 0 | Plus | Plus | Bacteria; Firmicutes; Clostridia; Eubacteriales; Lachnospiraceae; ; [Eubacterium] rectale | [Eubacterium] rectale | | Lachnospiraceae | Eubacteriales | Clostridia | Firmicutes | | Bacteria | |
| ASV123 | 403 | 1 | 1 | 403 | 100 | NR_042930.1 Acetanaerobacterium elongatum strain Z7 16S ribosomal RNA, partial sequence | NR_042930.1 | 1533 | 383 | 783 | 26.16 | 560.7 | 2E-159 | 370/403 | 91 | 2/403 | 0 | Plus | Plus | Bacteria; Firmicutes; Clostridia; Eubacteriales; Oscillospiraceae; Acetanaerobacterium; Acetanaerobacterium elongatum | Acetanaerobacterium elongatum | Acetanaerobacterium | Oscillospiraceae | Eubacteriales | Clostridia | Firmicutes | | Bacteria | |
| ASV124 | 405 | 2 | 3 | 405 | 99.51 | NR_144742.1 Christensenella massiliensis strain Marseille-P2438 16S ribosomal RNA, partial sequence | NR_144742.1 | 1499 | 371 | 773 | 26.88 | 545.9 | 6E-155 | 367/403 | 91 | 0/403 | 0 | Plus | Plus | Bacteria; Firmicutes; Clostridia; Eubacteriales; Christensenellaceae; Christensenella; Christensenella massiliensis | Christensenella massiliensis | Christensenella | Christensenellaceae | Eubacteriales | Clostridia | Firmicutes | | Bacteria | |
| ASV125 | 427 | 4 | 1 | 427 | 100 | NR_042802.1 Lactobacillus ultunensis strain Kx146C1 16S ribosomal RNA, partial sequence | NR_042802.1 | 1550 | 370 | 796 | 27.55 | 784.1 | 0 | 426/427 | 99 | 0/427 | 0 | Plus | Plus | Bacteria; Firmicutes; Bacilli; Lactobacillales; Lactobacillaceae; Lactobacillus; Lactobacillus ultunensis | Lactobacillus ultunensis | Lactobacillus | Lactobacillaceae | Lactobacillales | Bacilli | Firmicutes | | Bacteria | |
| ASV126 | 426 | 1 | 1 | 426 | 100 | NR_145570.1 Vibrio mexicanus strain CAIM 1540 16S ribosomal RNA, partial sequence | NR_145570.1 | 1436 | 324 | 750 | 29.74 | 473.9 | 3E-133 | 373/430 | 86 | 7/430 | 1 | Plus | Plus | Bacteria; Proteobacteria; Gammaproteobacteria; Vibrionales; Vibrionaceae; Vibrio; Vibrio mexicanus | Vibrio mexicanus | Vibrio | Vibrionaceae | Vibrionales | Gammaproteobacteria | Proteobacteria | | Bacteria | |
| ASV127 | 405 | 1 | 1 | 405 | 100 | NR_144611.1 Flintibacter butyricus strain BLS21 16S ribosomal RNA, partial sequence | NR_144611.1 | 808 | 299 | 703 | 50.12 | 588.4 | 9E-168 | 376/405 | 92 | 0/405 | 0 | Plus | Plus | Bacteria; Firmicutes; Clostridia; Eubacteriales; ; Flintibacter; Flintibacter butyricus | Flintibacter butyricus | Flintibacter | | Eubacteriales | Clostridia | Firmicutes | | Bacteria | |
| ASV128 | 402 | 1 | 1 | 402 | 100 | NR_156911.1 Fournierella massiliensis strain AT2 16S ribosomal RNA, partial sequence | NR_156911.1 | 1491 | 368 | 769 | 26.96 | 643.8 | 0 | 384/402 | 95 | 0/402 | 0 | Plus | Plus | Bacteria; Firmicutes; Clostridia; Eubacteriales; Oscillospiraceae; Fournierella; Fournierella massiliensis | Fournierella massiliensis | Fournierella | Oscillospiraceae | Eubacteriales | Clostridia | Firmicutes | | Bacteria | |
| ASV129 | 420 | 1 | 1 | 420 | 100 | NR_170509.1 Duncaniella freteri strain TLL-A3 16S ribosomal RNA, partial sequence | NR_170509.1 | 1538 | 364 | 784 | 27.37 | 455.4 | 1E-127 | 367/425 | 86 | 9/425 | 2 | Plus | Plus | Bacteria; Bacteroidetes; Bacteroidia; Bacteroidales; Muribaculaceae; Duncaniella; Duncaniella freteri | Duncaniella freteri | Duncaniella | Muribaculaceae | Bacteroidales | Bacteroidia | Bacteroidetes | | Bacteria | |
| ASV130 | 422 | 1 | 1 | 422 | 100 | NR_113411.1 Prevotella copri DSM 18205 strain JCM 13464 16S ribosomal RNA, partial sequence | NR_113411.1 | 1493 | 358 | 779 | 28.27 | 730.5 | 0 | 413/422 | 97 | 0/422 | 0 | Plus | Plus | Bacteria; Bacteroidetes; Bacteroidia; Bacteroidales; Prevotellaceae; Prevotella; Prevotella copri | Prevotella copri | Prevotella | Prevotellaceae | Bacteroidales | Bacteroidia | Bacteroidetes | | Bacteria | |
| ASV132 | 424 | 1 | 1 | 424 | 100 | NR_118334.1 Alloprevotella rava strain 81/4-12 16S ribosomal RNA, partial sequence | NR_118334.1 | 1455 | 338 | 759 | 29 | 605 | 1E-172 | 396/428 | 92 | 10/428 | 2 | Plus | Plus | Bacteria; Bacteroidetes; Bacteroidia; Bacteroidales; Prevotellaceae; Alloprevotella; Alloprevotella rava | Alloprevotella rava | Alloprevotella | Prevotellaceae | Bacteroidales | Bacteroidia | Bacteroidetes | | Bacteria | |
| ASV133 | 422 | 1 | 1 | 422 | 100 | NR_109628.1 Prevotella jejuni strain CD3:28 16S ribosomal RNA, partial sequence | NR_109628.1 | 1484 | 339 | 760 | 28.44 | 581 | 2E-165 | 386/422 | 91 | 0/422 | 0 | Plus | Plus | Bacteria; Bacteroidetes; Bacteroidia; Bacteroidales; Prevotellaceae; Prevotella; Prevotella jejuni | Prevotella jejuni | Prevotella | Prevotellaceae | Bacteroidales | Bacteroidia | Bacteroidetes | | Bacteria | |
| ASV134 | 401 | 2 | 1 | 401 | 100 | NR_144737.1 Emergencia timonensis strain SN18 16S ribosomal RNA, partial sequence | NR_144737.1 | 1480 | 364 | 767 | 27.3 | 605 | 9E-173 | 379/404 | 93 | 3/404 | 0 | Plus | Plus | Bacteria; Firmicutes; Clostridia; Eubacteriales; Clostridiales Family XIII. Incertae Sedis; Emergencia; Emergencia timonensis | Emergencia timonensis | Emergencia | Clostridiales Family XIII. Incertae Sedis | Eubacteriales | Clostridia | Firmicutes | | Bacteria | |
| ASV135 | 402 | 1 | 1 | 402 | 100 | NR_170495.1 Blautia faecicola strain KGMB01111 16S ribosomal RNA, complete sequence | NR_170495.1 | 1534 | 372 | 773 | 26.21 | 737.9 | 0 | 401/402 | 99 | 0/402 | 0 | Plus | Plus | Bacteria; Firmicutes; Clostridia; Eubacteriales; Lachnospiraceae; Blautia; Blautia faecicola | Blautia faecicola | Blautia | Lachnospiraceae | Eubacteriales | Clostridia | Firmicutes | | Bacteria | |
| ASV136 | 422 | 1 | 1 | 422 | 100 | NR_113411.1 Prevotella copri DSM 18205 strain JCM 13464 16S ribosomal RNA, partial sequence | NR_113411.1 | 1493 | 358 | 779 | 28.27 | 763.8 | 0 | 419/422 | 99 | 0/422 | 0 | Plus | Plus | Bacteria; Bacteroidetes; Bacteroidia; Bacteroidales; Prevotellaceae; Prevotella; Prevotella copri | Prevotella copri | Prevotella | Prevotellaceae | Bacteroidales | Bacteroidia | Bacteroidetes | | Bacteria | |
| ASV137 | 426 | 1 | 1 | 426 | 100 | NR_029335.1 Holdemania filiformis strain J1-31B-1 16S ribosomal RNA, partial sequence | NR_029335.1 | 1391 | 267 | 692 | 30.63 | 544 | 2E-154 | 383/427 | 89 | 2/427 | 0 | Plus | Plus | Bacteria; Firmicutes; Erysipelotrichia; Erysipelotrichales; Erysipelotrichaceae; Holdemania; Holdemania filiformis | Holdemania filiformis | Holdemania | Erysipelotrichaceae | Erysipelotrichales | Erysipelotrichia | Firmicutes | | Bacteria | |
| ASV138 | 401 | 1 | 1 | 401 | 100 | NR_027558.1 Anaerotruncus colihominis strain WAL 14565 16S ribosomal RNA, partial sequence | NR_027558.1 | 1365 | 309 | 709 | 29.38 | 603.1 | 3E-172 | 376/401 | 93 | 0/401 | 0 | Plus | Plus | Bacteria; Firmicutes; Clostridia; Eubacteriales; Oscillospiraceae; Anaerotruncus; Anaerotruncus colihominis | Anaerotruncus colihominis | Anaerotruncus | Oscillospiraceae | Eubacteriales | Clostridia | Firmicutes | | Bacteria | |
| ASV139 | 402 | 1 | 1 | 402 | 100 | NR_114326.1 Fusicatenibacter saccharivorans strain HT03-11 16S ribosomal RNA, partial sequence | NR_114326.1 | 1423 | 351 | 752 | 28.25 | 743.5 | 0 | 402/402 | 100 | 0/402 | 0 | Plus | Plus | Bacteria; Firmicutes; Clostridia; Eubacteriales; Lachnospiraceae; Fusicatenibacter; Fusicatenibacter saccharivorans | Fusicatenibacter saccharivorans | Fusicatenibacter | Lachnospiraceae | Eubacteriales | Clostridia | Firmicutes | | Bacteria | |
| ASV140 | 402 | 1 | 1 | 402 | 100 | NR_144727.1 Anaeromassilibacillus senegalensis strain mt9 16S ribosomal RNA, partial sequence | NR_144727.1 | 1478 | 352 | 753 | 27.2 | 466.5 | 4E-131 | 352/402 | 87 | 0/402 | 0 | Plus | Plus | Bacteria; Firmicutes; Clostridia; Eubacteriales; Oscillospiraceae; Anaeromassilibacillus; Anaeromassilibacillus senegalensis | Anaeromassilibacillus senegalensis | Anaeromassilibacillus | Oscillospiraceae | Eubacteriales | Clostridia | Firmicutes | | Bacteria | |
| ASV141 | 403 | 2 | 3 | 403 | 99.5 | NR_148837.1 Abyssivirga alkaniphila strain L81 16S ribosomal RNA, partial sequence | NR_148837.1 | 1520 | 366 | 765 | 26.32 | 451.7 | 1E-126 | 351/403 | 87 | 5/403 | 1 | Plus | Plus | Bacteria; Firmicutes; Clostridia; Eubacteriales; Lachnospiraceae; Abyssivirga; Abyssivirga alkaniphila | Abyssivirga alkaniphila | Abyssivirga | Lachnospiraceae | Eubacteriales | Clostridia | Firmicutes | | Bacteria | |
| ASV142 | 422 | 4 | 1 | 422 | 100 | NR_044864.1 Hallella seregens ATCC 51272 16S ribosomal RNA, partial sequence | NR_044864.1 | 1463 | 335 | 756 | 28.84 | 564.3 | 2E-160 | 383/422 | 90 | 0/422 | 0 | Plus | Plus | Bacteria; Bacteroidetes; Bacteroidia; Bacteroidales; Prevotellaceae; Prevotella; Hallella seregens | Hallella seregens | Prevotella | Prevotellaceae | Bacteroidales | Bacteroidia | Bacteroidetes | | Bacteria | |
| ASV143 | 403 | 3 | 1 | 403 | 100 | NR_118521.1 Campylobacter lanienae NCTC 13004 strain CCUG 44467 16S ribosomal RNA, partial sequence | NR_118521.1 | 1434 | 328 | 730 | 28.1 | 723.2 | 0 | 399/403 | 99 | 0/403 | 0 | Plus | Plus | Bacteria; Proteobacteria; Epsilonproteobacteria; Campylobacterales; Campylobacteraceae; Campylobacter; Campylobacter lanienae | Campylobacter lanienae | Campylobacter | Campylobacteraceae | Campylobacterales | Epsilonproteobacteria | Proteobacteria | | Bacteria | |
| ASV144 | 428 | 1 | 1 | 428 | 100 | NR_104781.1 Treponema bryantii strain RUS-1 16S ribosomal RNA, partial sequence | NR_104781.1 | 1535 | 351 | 778 | 27.88 | 641.9 | 0 | 401/428 | 93 | 0/428 | 0 | Plus | Plus | Bacteria; Spirochaetes; Spirochaetia; Spirochaetales; Spirochaetaceae; Treponema; Treponema bryantii | Treponema bryantii | Treponema | Spirochaetaceae | Spirochaetales | Spirochaetia | Spirochaetes | | Bacteria | |
| ASV146 | 427 | 2 | 1 | 426 | 99.77 | NR_027596.1 Mitsuokella multacida strain DSM 20544 16S ribosomal RNA, partial sequence | NR_027596.1 | 1511 | 346 | 771 | 28.19 | 656.7 | 0 | 403/427 | 94 | 2/427 | 0 | Plus | Plus | Bacteria; Firmicutes; Negativicutes; Selenomonadales; Selenomonadaceae; Mitsuokella; Mitsuokella multacida | Mitsuokella multacida | Mitsuokella | Selenomonadaceae | Selenomonadales | Negativicutes | Firmicutes | | Bacteria | |
| ASV147 | 404 | 1 | 1 | 404 | 100 | NR_118156.1 Oscillibacter ruminantium GH1 16S ribosomal RNA, partial sequence | NR_118156.1 | 1409 | 321 | 724 | 28.67 | 614.2 | 2E-175 | 381/405 | 94 | 2/405 | 0 | Plus | Plus | Bacteria; Firmicutes; Clostridia; Eubacteriales; Oscillospiraceae; Oscillibacter; Oscillibacter ruminantium | Oscillibacter ruminantium | Oscillibacter | Oscillospiraceae | Eubacteriales | Clostridia | Firmicutes | | Bacteria | |
| ASV148 | 405 | 2 | 1 | 405 | 100 | NR_118554.1 Intestinimonas butyriciproducens strain SRB-521-5-I 16S ribosomal RNA, partial sequence | NR_118554.1 | 1475 | 346 | 750 | 27.46 | 566.2 | 4E-161 | 372/405 | 91 | 0/405 | 0 | Plus | Plus | Bacteria; Firmicutes; Clostridia; Eubacteriales; ; Intestinimonas; Intestinimonas butyriciproducens | Intestinimonas butyriciproducens | Intestinimonas | | Eubacteriales | Clostridia | Firmicutes | | Bacteria | |
| ASV149 | 427 | 2 | 1 | 426 | 99.77 | NR_027596.1 Mitsuokella multacida strain DSM 20544 16S ribosomal RNA, partial sequence | NR_027596.1 | 1511 | 346 | 771 | 28.19 | 651.1 | 0 | 402/427 | 94 | 2/427 | 0 | Plus | Plus | Bacteria; Firmicutes; Negativicutes; Selenomonadales; Selenomonadaceae; Mitsuokella; Mitsuokella multacida | Mitsuokella multacida | Mitsuokella | Selenomonadaceae | Selenomonadales | Negativicutes | Firmicutes | | Bacteria | |
| ASV150 | 402 | 1 | 1 | 402 | 100 | NR_118692.1 Blautia obeum ATCC 29174 16S ribosomal RNA, partial sequence | NR_118692.1 | 1398 | 330 | 731 | 28.76 | 739.8 | 0 | 401/402 | 99 | 0/402 | 0 | Plus | Plus | Bacteria; Firmicutes; Clostridia; Eubacteriales; Lachnospiraceae; Blautia; Blautia obeum | Blautia obeum | Blautia | Lachnospiraceae | Eubacteriales | Clostridia | Firmicutes | | Bacteria | |
| ASV151 | 427 | 1 | 1 | 427 | 100 | NR_044731.2 Holdemanella biformis strain DSM 3989 16S ribosomal RNA, partial sequence | NR_044731.2 | 1487 | 364 | 789 | 28.65 | 739.8 | 0 | 418/427 | 97 | 1/427 | 0 | Plus | Plus | Bacteria; Firmicutes; Erysipelotrichia; Erysipelotrichales; Erysipelotrichaceae; Holdemanella; Holdemanella biformis | Holdemanella biformis | Holdemanella | Erysipelotrichaceae | Erysipelotrichales | Erysipelotrichia | Firmicutes | | Bacteria | |
| ASV152 | 422 | 1 | 1 | 422 | 100 | NR_041285.1 Prevotella multisaccharivorax strain PPPA20 16S ribosomal RNA, partial sequence | NR_041285.1 | 1491 | 356 | 777 | 28.3 | 619.7 | 3E-177 | 393/422 | 93 | 0/422 | 0 | Plus | Plus | Bacteria; Bacteroidetes; Bacteroidia; Bacteroidales; Prevotellaceae; Prevotella; Prevotella multisaccharivorax | Prevotella multisaccharivorax | Prevotella | Prevotellaceae | Bacteroidales | Bacteroidia | Bacteroidetes | | Bacteria | |
| ASV153 | 420 | 1 | 1 | 420 | 100 | NR_170509.1 Duncaniella freteri strain TLL-A3 16S ribosomal RNA, partial sequence | NR_170509.1 | 1538 | 364 | 784 | 27.37 | 460.9 | 2E-129 | 368/425 | 86 | 9/425 | 2 | Plus | Plus | Bacteria; Bacteroidetes; Bacteroidia; Bacteroidales; Muribaculaceae; Duncaniella; Duncaniella freteri | Duncaniella freteri | Duncaniella | Muribaculaceae | Bacteroidales | Bacteroidia | Bacteroidetes | | Bacteria | |
| ASV154 | 421 | 1 | 1 | 421 | 100 | NR_144616.1 Muribaculum intestinale strain YL27 16S ribosomal RNA, partial sequence | NR_144616.1 | 1460 | 297 | 718 | 28.9 | 512.6 | 6E-145 | 374/422 | 88 | 1/422 | 0 | Plus | Plus | Bacteria; Bacteroidetes; Bacteroidia; Bacteroidales; Muribaculaceae; Muribaculum; Muribaculum intestinale | Muribaculum intestinale | Muribaculum | Muribaculaceae | Bacteroidales | Bacteroidia | Bacteroidetes | | Bacteria | |
| ASV155 | 402 | 1 | 1 | 402 | 100 | NR_118673.1 [Eubacterium] hallii strain ATCC 27751 16S ribosomal RNA, partial sequence | NR_118673.1 | 1479 | 371 | 772 | 27.18 | 728.7 | 0 | 398/402 | 99 | 0/402 | 0 | Plus | Plus | Bacteria; Firmicutes; Clostridia; Eubacteriales; Lachnospiraceae; Anaerobutyricum; Anaerobutyricum hallii | Anaerobutyricum hallii | Anaerobutyricum | Lachnospiraceae | Eubacteriales | Clostridia | Firmicutes | | Bacteria | |
| ASV156 | 403 | 1 | 1 | 403 | 100 | NR_104907.1 Eubacterium coprostanoligenes strain HL 16S ribosomal RNA, partial sequence | NR_104907.1 | 1446 | 332 | 734 | 27.87 | 723.2 | 0 | 399/403 | 99 | 0/403 | 0 | Plus | Plus | Bacteria; Firmicutes; Clostridia; Eubacteriales; Eubacteriaceae; Eubacterium; Eubacterium coprostanoligenes | Eubacterium coprostanoligenes | Eubacterium | Eubacteriaceae | Eubacteriales | Clostridia | Firmicutes | | Bacteria | |
| ASV157 | 421 | 2 | 1 | 421 | 100 | NR_113077.1 Paraprevotella clara strain JCM 14859 16S ribosomal RNA, partial sequence | NR_113077.1 | 1480 | 346 | 766 | 28.45 | 512.6 | 6E-145 | 374/422 | 88 | 2/422 | 0 | Plus | Plus | Bacteria; Bacteroidetes; Bacteroidia; Bacteroidales; Prevotellaceae; Paraprevotella; Paraprevotella clara | Paraprevotella clara | Paraprevotella | Prevotellaceae | Bacteroidales | Bacteroidia | Bacteroidetes | | Bacteria | |
| ASV158 | 422 | 2 | 1 | 422 | 100 | NR_113117.1 Prevotella oralis ATCC 33269 strain JCM 12251 16S ribosomal RNA, partial sequence | NR_113117.1 | 1496 | 361 | 782 | 28.21 | 597.6 | 2E-170 | 390/423 | 92 | 2/423 | 0 | Plus | Plus | Bacteria; Bacteroidetes; Bacteroidia; Bacteroidales; Prevotellaceae; Prevotella; Prevotella oralis | Prevotella oralis | Prevotella | Prevotellaceae | Bacteroidales | Bacteroidia | Bacteroidetes | | Bacteria | |
| ASV159 | 405 | 1 | 1 | 405 | 100 | NR_144742.1 Christensenella massiliensis strain Marseille-P2438 16S ribosomal RNA, partial sequence | NR_144742.1 | 1499 | 369 | 773 | 27.02 | 494.2 | 2E-139 | 359/405 | 88 | 0/405 | 0 | Plus | Plus | Bacteria; Firmicutes; Clostridia; Eubacteriales; Christensenellaceae; Christensenella; Christensenella massiliensis | Christensenella massiliensis | Christensenella | Christensenellaceae | Eubacteriales | Clostridia | Firmicutes | | Bacteria | |
| ASV160 | 427 | 5 | 1 | 427 | 100 | NR_119109.1 Pseudescherichia vulneris strain ATCC 33821 16S ribosomal RNA, partial sequence | NR_119109.1 | 1443 | 344 | 770 | 29.59 | 784.1 | 0 | 426/427 | 99 | 0/427 | 0 | Plus | Plus | Bacteria; Proteobacteria; Gammaproteobacteria; Enterobacterales; Enterobacteriaceae; Pseudescherichia; Pseudescherichia vulneris | Pseudescherichia vulneris | Pseudescherichia | Enterobacteriaceae | Enterobacterales | Gammaproteobacteria | Proteobacteria | | Bacteria | |
| ASV162 | 422 | 2 | 1 | 422 | 100 | NR_074376.1 Parabacteroides distasonis ATCC 8503 16S ribosomal RNA, partial sequence | NR_074376.1 | 1497 | 368 | 789 | 28.19 | 564.3 | 2E-160 | 383/422 | 90 | 0/422 | 0 | Plus | Plus | Bacteria; Bacteroidetes; Bacteroidia; Bacteroidales; Tannerellaceae; Parabacteroides; Parabacteroides distasonis | Parabacteroides distasonis | Parabacteroides | Tannerellaceae | Bacteroidales | Bacteroidia | Bacteroidetes | | Bacteria | |
| ASV163 | 402 | 1 | 1 | 402 | 100 | NR_026106.1 [Eubacterium] cellulosolvens strain 6 16S ribosomal RNA, partial sequence | NR_026106.1 | 1489 | 333 | 734 | 27 | 632.7 | 0 | 382/402 | 95 | 0/402 | 0 | Plus | Plus | Bacteria; Firmicutes; Clostridia; Eubacteriales; Eubacteriaceae; Eubacterium; [Eubacterium] cellulosolvens | [Eubacterium] cellulosolvens | Eubacterium | Eubacteriaceae | Eubacteriales | Clostridia | Firmicutes | | Bacteria | |
| ASV164 | 421 | 2 | 1 | 421 | 100 | NR_113077.1 Paraprevotella clara strain JCM 14859 16S ribosomal RNA, partial sequence | NR_113077.1 | 1480 | 346 | 766 | 28.45 | 518.2 | 1E-146 | 375/422 | 88 | 2/422 | 0 | Plus | Plus | Bacteria; Bacteroidetes; Bacteroidia; Bacteroidales; Prevotellaceae; Paraprevotella; Paraprevotella clara | Paraprevotella clara | Paraprevotella | Prevotellaceae | Bacteroidales | Bacteroidia | Bacteroidetes | | Bacteria | |
| ASV165 | 404 | 1 | 1 | 404 | 100 | NR_118156.1 Oscillibacter ruminantium GH1 16S ribosomal RNA, partial sequence | NR_118156.1 | 1409 | 321 | 724 | 28.67 | 614.2 | 2E-175 | 381/405 | 94 | 2/405 | 0 | Plus | Plus | Bacteria; Firmicutes; Clostridia; Eubacteriales; Oscillospiraceae; Oscillibacter; Oscillibacter ruminantium | Oscillibacter ruminantium | Oscillibacter | Oscillospiraceae | Eubacteriales | Clostridia | Firmicutes | | Bacteria | |
| ASV166 | 421 | 1 | 1 | 421 | 100 | NR_144745.1 Culturomica massiliensis strain Marseille-P2698 16S ribosomal RNA, partial sequence | NR_144745.1 | 1492 | 356 | 776 | 28.22 | 778.6 | 0 | 421/421 | 100 | 0/421 | 0 | Plus | Plus | Bacteria; Bacteroidetes; Bacteroidia; Bacteroidales; Odoribacteraceae; Culturomica; Culturomica massiliensis | Culturomica massiliensis | Culturomica | Odoribacteraceae | Bacteroidales | Bacteroidia | Bacteroidetes | | Bacteria | |
| ASV167 | 403 | 1 | 3 | 403 | 99.5 | NR_125677.1 Vallitalea pronyensis strain FatNI3 16S ribosomal RNA, partial sequence | NR_125677.1 | 1480 | 365 | 764 | 27.03 | 440.6 | 3E-123 | 348/402 | 86 | 3/402 | 0 | Plus | Plus | Bacteria; Firmicutes; Clostridia; Eubacteriales; Vallitaleaceae; Vallitalea; Vallitalea pronyensis | Vallitalea pronyensis | Vallitalea | Vallitaleaceae | Eubacteriales | Clostridia | Firmicutes | | Bacteria | |
| ASV168 | 421 | 5 | 1 | 421 | 100 | NR_146813.2 Erysipelothrix larvae strain LV19 16S ribosomal RNA, complete sequence | NR_146813.2 | 1543 | 367 | 792 | 27.61 | 538.5 | 1E-152 | 382/426 | 89 | 5/426 | 1 | Plus | Plus | Bacteria; Firmicutes; Erysipelotrichia; Erysipelotrichales; Erysipelotrichaceae; Erysipelothrix; Erysipelothrix larvae | Erysipelothrix larvae | Erysipelothrix | Erysipelotrichaceae | Erysipelotrichales | Erysipelotrichia | Firmicutes | | Bacteria | |
| ASV169 | 422 | 3 | 1 | 422 | 100 | NR_113119.1 Prevotella oris strain JCM 8540 16S ribosomal RNA, partial sequence | NR_113119.1 | 1481 | 345 | 766 | 28.49 | 581 | 2E-165 | 387/423 | 91 | 2/423 | 0 | Plus | Plus | Bacteria; Bacteroidetes; Bacteroidia; Bacteroidales; Prevotellaceae; Prevotella; Prevotella oris | Prevotella oris | Prevotella | Prevotellaceae | Bacteroidales | Bacteroidia | Bacteroidetes | | Bacteria | |
| ASV170 | 427 | 1 | 1 | 427 | 100 | NR_044111.1 Selenomonas bovis strain WG 16S ribosomal RNA, partial sequence | NR_044111.1 | 1525 | 370 | 796 | 28 | 645.6 | 0 | 402/428 | 93 | 2/428 | 0 | Plus | Plus | Bacteria; Firmicutes; Negativicutes; Selenomonadales; Selenomonadaceae; Selenomonas; Selenomonas bovis | Selenomonas bovis | Selenomonas | Selenomonadaceae | Selenomonadales | Negativicutes | Firmicutes | | Bacteria | |
| ASV171 | 427 | 2 | 1 | 427 | 100 | NR_037018.1 Acidaminococcus fermentans strain VR4 16S ribosomal RNA gene, partial sequence | NR_037018.1 | 1516 | 349 | 775 | 28.17 | 789.6 | 0 | 427/427 | 100 | 0/427 | 0 | Plus | Plus | Bacteria; Firmicutes; Negativicutes; Acidaminococcales; Acidaminococcaceae; Acidaminococcus; Acidaminococcus fermentans | Acidaminococcus fermentans | Acidaminococcus | Acidaminococcaceae | Acidaminococcales | Negativicutes | Firmicutes | | Bacteria | |
| ASV172 | 426 | 1 | 1 | 426 | 100 | NR_114696.1 Anaerovibrio lipolyticus DSM 3074 16S ribosomal RNA, partial sequence | NR_114696.1 | 1526 | 372 | 797 | 27.92 | 695.5 | 0 | 408/426 | 95 | 0/426 | 0 | Plus | Plus | Bacteria; Firmicutes; Negativicutes; Selenomonadales; Selenomonadaceae; Anaerovibrio; Anaerovibrio lipolyticus | Anaerovibrio lipolyticus | Anaerovibrio | Selenomonadaceae | Selenomonadales | Negativicutes | Firmicutes | | Bacteria | |
| ASV174 | 421 | 1 | 1 | 421 | 100 | NR_144616.1 Muribaculum intestinale strain YL27 16S ribosomal RNA, partial sequence | NR_144616.1 | 1460 | 297 | 718 | 28.9 | 479.4 | 6E-135 | 373/427 | 87 | 11/427 | 2 | Plus | Plus | Bacteria; Bacteroidetes; Bacteroidia; Bacteroidales; Muribaculaceae; Muribaculum; Muribaculum intestinale | Muribaculum intestinale | Muribaculum | Muribaculaceae | Bacteroidales | Bacteroidia | Bacteroidetes | | Bacteria | |
| ASV175 | 422 | 2 | 1 | 422 | 100 | NR_074376.1 Parabacteroides distasonis ATCC 8503 16S ribosomal RNA, partial sequence | NR_074376.1 | 1497 | 368 | 789 | 28.19 | 558.8 | 8E-159 | 382/422 | 90 | 0/422 | 0 | Plus | Plus | Bacteria; Bacteroidetes; Bacteroidia; Bacteroidales; Tannerellaceae; Parabacteroides; Parabacteroides distasonis | Parabacteroides distasonis | Parabacteroides | Tannerellaceae | Bacteroidales | Bacteroidia | Bacteroidetes | | Bacteria | |
| ASV176 | 404 | 1 | 1 | 404 | 100 | NR_112097.1 Pseudoramibacter alactolyticus ATCC 23263 16S ribosomal RNA, partial sequence | NR_112097.1 | 1480 | 376 | 779 | 27.3 | 725 | 0 | 400/404 | 99 | 0/404 | 0 | Plus | Plus | Bacteria; Firmicutes; Clostridia; Eubacteriales; Eubacteriaceae; Pseudoramibacter; Pseudoramibacter alactolyticus | Pseudoramibacter alactolyticus | Pseudoramibacter | Eubacteriaceae | Eubacteriales | Clostridia | Firmicutes | | Bacteria | |
| ASV177 | 420 | 1 | 1 | 420 | 100 | NR_170509.1 Duncaniella freteri strain TLL-A3 16S ribosomal RNA, partial sequence | NR_170509.1 | 1538 | 364 | 784 | 27.37 | 466.5 | 5E-131 | 369/425 | 86 | 9/425 | 2 | Plus | Plus | Bacteria; Bacteroidetes; Bacteroidia; Bacteroidales; Muribaculaceae; Duncaniella; Duncaniella freteri | Duncaniella freteri | Duncaniella | Muribaculaceae | Bacteroidales | Bacteroidia | Bacteroidetes | | Bacteria | |
| ASV178 | 402 | 1 | 1 | 402 | 100 | NR_028883.1 Dorea longicatena strain 111-35 16S ribosomal RNA, partial sequence | NR_028883.1 | 1316 | 243 | 645 | 30.62 | 704.7 | 0 | 397/404 | 98 | 3/404 | 0 | Plus | Plus | Bacteria; Firmicutes; Clostridia; Eubacteriales; Lachnospiraceae; Dorea; Dorea longicatena | Dorea longicatena | Dorea | Lachnospiraceae | Eubacteriales | Clostridia | Firmicutes | | Bacteria | |
| ASV179 | 422 | 1 | 1 | 422 | 100 | NR_113411.1 Prevotella copri DSM 18205 strain JCM 13464 16S ribosomal RNA, partial sequence | NR_113411.1 | 1493 | 358 | 779 | 28.27 | 758.2 | 0 | 418/422 | 99 | 0/422 | 0 | Plus | Plus | Bacteria; Bacteroidetes; Bacteroidia; Bacteroidales; Prevotellaceae; Prevotella; Prevotella copri | Prevotella copri | Prevotella | Prevotellaceae | Bacteroidales | Bacteroidia | Bacteroidetes | | Bacteria | |
| ASV180 | 402 | 1 | 1 | 402 | 100 | NR_104846.1 Gemmiger formicilis strain X2-56 16S ribosomal RNA, partial sequence | NR_104846.1 | 1426 | 305 | 706 | 28.19 | 732.4 | 0 | 400/402 | 99 | 0/402 | 0 | Plus | Plus | Bacteria; Firmicutes; Clostridia; Eubacteriales; ; Gemmiger; Gemmiger formicilis | Gemmiger formicilis | Gemmiger | | Eubacteriales | Clostridia | Firmicutes | | Bacteria | |
| ASV181 | 404 | 1 | 1 | 404 | 100 | NR_118156.1 Oscillibacter ruminantium GH1 16S ribosomal RNA, partial sequence | NR_118156.1 | 1409 | 321 | 724 | 28.67 | 603.1 | 3E-172 | 379/405 | 93 | 2/405 | 0 | Plus | Plus | Bacteria; Firmicutes; Clostridia; Eubacteriales; Oscillospiraceae; Oscillibacter; Oscillibacter ruminantium | Oscillibacter ruminantium | Oscillibacter | Oscillospiraceae | Eubacteriales | Clostridia | Firmicutes | | Bacteria | |
| ASV182 | 420 | 1 | 1 | 420 | 100 | NR_170509.1 Duncaniella freteri strain TLL-A3 16S ribosomal RNA, partial sequence | NR_170509.1 | 1538 | 364 | 784 | 27.37 | 455.4 | 1E-127 | 367/425 | 86 | 9/425 | 2 | Plus | Plus | Bacteria; Bacteroidetes; Bacteroidia; Bacteroidales; Muribaculaceae; Duncaniella; Duncaniella freteri | Duncaniella freteri | Duncaniella | Muribaculaceae | Bacteroidales | Bacteroidia | Bacteroidetes | | Bacteria | |
| ASV183 | 427 | 2 | 1 | 427 | 100 | NR_029106.1 Lactobacillus delbrueckii subsp. indicus strain NCC725 16S ribosomal RNA, partial sequence | NR_029106.1 | 1515 | 348 | 774 | 28.18 | 778.6 | 0 | 425/427 | 99 | 0/427 | 0 | Plus | Plus | Bacteria; Firmicutes; Bacilli; Lactobacillales; Lactobacillaceae; Lactobacillus; Lactobacillus delbrueckii | Lactobacillus delbrueckii | Lactobacillus | Lactobacillaceae | Lactobacillales | Bacilli | Firmicutes | | Bacteria | |
| ASV184 | 422 | 1 | 1 | 422 | 100 | NR_041285.1 Prevotella multisaccharivorax strain PPPA20 16S ribosomal RNA, partial sequence | NR_041285.1 | 1491 | 356 | 777 | 28.3 | 597.6 | 2E-170 | 390/423 | 92 | 2/423 | 0 | Plus | Plus | Bacteria; Bacteroidetes; Bacteroidia; Bacteroidales; Prevotellaceae; Prevotella; Prevotella multisaccharivorax | Prevotella multisaccharivorax | Prevotella | Prevotellaceae | Bacteroidales | Bacteroidia | Bacteroidetes | | Bacteria | |
| ASV185 | 427 | 1 | 1 | 427 | 100 | NR_157635.1 Megasphaera hexanoica strain MH 16S ribosomal RNA, partial sequence | NR_157635.1 | 1561 | 372 | 798 | 27.35 | 789.6 | 0 | 427/427 | 100 | 0/427 | 0 | Plus | Plus | Bacteria; Firmicutes; Negativicutes; Veillonellales; Veillonellaceae; Megasphaera; Megasphaera hexanoica | Megasphaera hexanoica | Megasphaera | Veillonellaceae | Veillonellales | Negativicutes | Firmicutes | | Bacteria | |
| ASV186 | 404 | 3 | 1 | 404 | 100 | NR_156081.1 Kineothrix alysoides strain KNHs209 16S ribosomal RNA, partial sequence | NR_156081.1 | 1411 | 353 | 754 | 28.49 | 701 | 0 | 396/404 | 98 | 2/404 | 0 | Plus | Plus | Bacteria; Firmicutes; Clostridia; Eubacteriales; Lachnospiraceae; Kineothrix; Kineothrix alysoides | Kineothrix alysoides | Kineothrix | Lachnospiraceae | Eubacteriales | Clostridia | Firmicutes | | Bacteria | |
| ASV187 | 422 | 3 | 1 | 422 | 100 | NR_113119.1 Prevotella oris strain JCM 8540 16S ribosomal RNA, partial sequence | NR_113119.1 | 1481 | 345 | 766 | 28.49 | 586.5 | 3E-167 | 388/423 | 91 | 2/423 | 0 | Plus | Plus | Bacteria; Bacteroidetes; Bacteroidia; Bacteroidales; Prevotellaceae; Prevotella; Prevotella oris | Prevotella oris | Prevotella | Prevotellaceae | Bacteroidales | Bacteroidia | Bacteroidetes | | Bacteria | |
| ASV188 | 422 | 2 | 1 | 422 | 100 | NR_113105.1 Prevotella histicola strain JCM 15637 16S ribosomal RNA, partial sequence | NR_113105.1 | 1480 | 346 | 767 | 28.51 | 653 | 0 | 401/424 | 94 | 4/424 | 0 | Plus | Plus | Bacteria; Bacteroidetes; Bacteroidia; Bacteroidales; Prevotellaceae; Prevotella; Prevotella histicola | Prevotella histicola | Prevotella | Prevotellaceae | Bacteroidales | Bacteroidia | Bacteroidetes | | Bacteria | |
| ASV189 | 427 | 1 | 1 | 427 | 100 | NR_029196.1 Chlamydia suis strain S45 16S ribosomal RNA, partial sequence | NR_029196.1 | 1545 | 358 | 784 | 27.64 | 756.4 | 0 | 421/427 | 98 | 0/427 | 0 | Plus | Plus | Bacteria; Chlamydiae; Chlamydiia; Chlamydiales; Chlamydiaceae; Chlamydia; Chlamydia suis | Chlamydia suis | Chlamydia | Chlamydiaceae | Chlamydiales | Chlamydiia | Chlamydiae | | Bacteria | |
| ASV190 | 422 | 2 | 1 | 422 | 100 | NR_113119.1 Prevotella oris strain JCM 8540 16S ribosomal RNA, partial sequence | NR_113119.1 | 1481 | 345 | 766 | 28.49 | 525.6 | 8E-149 | 377/423 | 89 | 2/423 | 0 | Plus | Plus | Bacteria; Bacteroidetes; Bacteroidia; Bacteroidales; Prevotellaceae; Prevotella; Prevotella oris | Prevotella oris | Prevotella | Prevotellaceae | Bacteroidales | Bacteroidia | Bacteroidetes | | Bacteria | |
| ASV191 | 421 | 1 | 1 | 421 | 100 | NR_144616.1 Muribaculum intestinale strain YL27 16S ribosomal RNA, partial sequence | NR_144616.1 | 1460 | 297 | 718 | 28.9 | 512.6 | 6E-145 | 376/424 | 88 | 5/424 | 1 | Plus | Plus | Bacteria; Bacteroidetes; Bacteroidia; Bacteroidales; Muribaculaceae; Muribaculum; Muribaculum intestinale | Muribaculum intestinale | Muribaculum | Muribaculaceae | Bacteroidales | Bacteroidia | Bacteroidetes | | Bacteria | |
| ASV192 | 402 | 1 | 1 | 402 | 100 | NR_028961.1 Faecalibacterium prausnitzii strain ATCC 27768 16S ribosomal RNA, partial sequence | NR_028961.1 | 1462 | 344 | 745 | 27.5 | 743.5 | 0 | 402/402 | 100 | 0/402 | 0 | Plus | Plus | Bacteria; Firmicutes; Clostridia; Eubacteriales; Oscillospiraceae; Faecalibacterium; Faecalibacterium prausnitzii | Faecalibacterium prausnitzii | Faecalibacterium | Oscillospiraceae | Eubacteriales | Clostridia | Firmicutes | | Bacteria | |
| ASV193 | 427 | 1 | 1 | 426 | 99.77 | NR_027596.1 Mitsuokella multacida strain DSM 20544 16S ribosomal RNA, partial sequence | NR_027596.1 | 1511 | 346 | 771 | 28.19 | 662.2 | 0 | 403/426 | 94 | 0/426 | 0 | Plus | Plus | Bacteria; Firmicutes; Negativicutes; Selenomonadales; Selenomonadaceae; Mitsuokella; Mitsuokella multacida | Mitsuokella multacida | Mitsuokella | Selenomonadaceae | Selenomonadales | Negativicutes | Firmicutes | | Bacteria | |
| ASV194 | 405 | 1 | 1 | 405 | 100 | NR_118554.1 Intestinimonas butyriciproducens strain SRB-521-5-I 16S ribosomal RNA, partial sequence | NR_118554.1 | 1475 | 346 | 750 | 27.46 | 593.9 | 2E-169 | 377/405 | 93 | 0/405 | 0 | Plus | Plus | Bacteria; Firmicutes; Clostridia; Eubacteriales; ; Intestinimonas; Intestinimonas butyriciproducens | Intestinimonas butyriciproducens | Intestinimonas | | Eubacteriales | Clostridia | Firmicutes | | Bacteria | |
| ASV195 | 421 | 1 | 1 | 421 | 100 | NR_144616.1 Muribaculum intestinale strain YL27 16S ribosomal RNA, partial sequence | NR_144616.1 | 1460 | 297 | 718 | 28.9 | 529.3 | 6E-150 | 381/426 | 89 | 9/426 | 2 | Plus | Plus | Bacteria; Bacteroidetes; Bacteroidia; Bacteroidales; Muribaculaceae; Muribaculum; Muribaculum intestinale | Muribaculum intestinale | Muribaculum | Muribaculaceae | Bacteroidales | Bacteroidia | Bacteroidetes | | Bacteria | |
| ASV196 | 422 | 4 | 1 | 422 | 100 | NR_025300.1 Prevotella albensis strain M384 16S ribosomal RNA, partial sequence | NR_025300.1 | 1495 | 358 | 779 | 28.23 | 581 | 2E-165 | 387/423 | 91 | 2/423 | 0 | Plus | Plus | Bacteria; Bacteroidetes; Bacteroidia; Bacteroidales; Prevotellaceae; Prevotella; Prevotella albensis | Prevotella albensis | Prevotella | Prevotellaceae | Bacteroidales | Bacteroidia | Bacteroidetes | | Bacteria | |
| ASV197 | 421 | 1 | 1 | 421 | 100 | NR_170509.1 Duncaniella freteri strain TLL-A3 16S ribosomal RNA, partial sequence | NR_170509.1 | 1538 | 364 | 784 | 27.37 | 540.3 | 3E-153 | 383/426 | 89 | 10/426 | 2 | Plus | Plus | Bacteria; Bacteroidetes; Bacteroidia; Bacteroidales; Muribaculaceae; Duncaniella; Duncaniella freteri | Duncaniella freteri | Duncaniella | Muribaculaceae | Bacteroidales | Bacteroidia | Bacteroidetes | | Bacteria | |
| ASV198 | 402 | 1 | 1 | 402 | 100 | NR_104846.1 Gemmiger formicilis strain X2-56 16S ribosomal RNA, partial sequence | NR_104846.1 | 1426 | 305 | 706 | 28.19 | 737.9 | 0 | 401/402 | 99 | 0/402 | 0 | Plus | Plus | Bacteria; Firmicutes; Clostridia; Eubacteriales; ; Gemmiger; Gemmiger formicilis | Gemmiger formicilis | Gemmiger | | Eubacteriales | Clostridia | Firmicutes | | Bacteria | |
| ASV200 | 428 | 1 | 1 | 428 | 100 | NR_165711.1 Intestinibaculum porci strain SG0102 16S ribosomal RNA, partial sequence | NR_165711.1 | 1489 | 329 | 756 | 28.74 | 791.5 | 0 | 428/428 | 100 | 0/428 | 0 | Plus | Plus | Bacteria; Firmicutes; Erysipelotrichia; Erysipelotrichales; Erysipelotrichaceae; Intestinibaculum; Intestinibaculum porci | Intestinibaculum porci | Intestinibaculum | Erysipelotrichaceae | Erysipelotrichales | Erysipelotrichia | Firmicutes | | Bacteria | |
| ASV201 | 404 | 1 | 1 | 404 | 100 | NR_118156.1 Oscillibacter ruminantium GH1 16S ribosomal RNA, partial sequence | NR_118156.1 | 1409 | 321 | 724 | 28.67 | 575.4 | 7E-164 | 376/407 | 92 | 6/407 | 1 | Plus | Plus | Bacteria; Firmicutes; Clostridia; Eubacteriales; Oscillospiraceae; Oscillibacter; Oscillibacter ruminantium | Oscillibacter ruminantium | Oscillibacter | Oscillospiraceae | Eubacteriales | Clostridia | Firmicutes | | Bacteria | |
| ASV203 | 422 | 2 | 1 | 422 | 100 | NR_113117.1 Prevotella oralis ATCC 33269 strain JCM 12251 16S ribosomal RNA, partial sequence | NR_113117.1 | 1496 | 361 | 782 | 28.21 | 603.1 | 3E-172 | 391/423 | 92 | 2/423 | 0 | Plus | Plus | Bacteria; Bacteroidetes; Bacteroidia; Bacteroidales; Prevotellaceae; Prevotella; Prevotella oralis | Prevotella oralis | Prevotella | Prevotellaceae | Bacteroidales | Bacteroidia | Bacteroidetes | | Bacteria | |
| ASV204 | 402 | 2 | 1 | 402 | 100 | NR_156080.1 Kineothrix alysoides strain KNHs209 16S ribosomal RNA, partial sequence | NR_156080.1 | 1410 | 353 | 754 | 28.51 | 721.3 | 0 | 398/402 | 99 | 0/402 | 0 | Plus | Plus | Bacteria; Firmicutes; Clostridia; Eubacteriales; Lachnospiraceae; Kineothrix; Kineothrix alysoides | Kineothrix alysoides | Kineothrix | Lachnospiraceae | Eubacteriales | Clostridia | Firmicutes | | Bacteria | |
| ASV205 | 402 | 1 | 1 | 402 | 100 | NR_028961.1 Faecalibacterium prausnitzii strain ATCC 27768 16S ribosomal RNA, partial sequence | NR_028961.1 | 1462 | 344 | 745 | 27.5 | 726.9 | 0 | 399/402 | 99 | 0/402 | 0 | Plus | Plus | Bacteria; Firmicutes; Clostridia; Eubacteriales; Oscillospiraceae; Faecalibacterium; Faecalibacterium prausnitzii | Faecalibacterium prausnitzii | Faecalibacterium | Oscillospiraceae | Eubacteriales | Clostridia | Firmicutes | | Bacteria | |
| ASV206 | 427 | 1 | 1 | 427 | 100 | NR_102980.1 Megasphaera elsdenii DSM 20460 16S ribosomal RNA, partial sequence | NR_102980.1 | 1552 | 369 | 795 | 27.51 | 778.6 | 0 | 425/427 | 99 | 0/427 | 0 | Plus | Plus | Bacteria; Firmicutes; Negativicutes; Veillonellales; Veillonellaceae; Megasphaera; Megasphaera elsdenii | Megasphaera elsdenii | Megasphaera | Veillonellaceae | Veillonellales | Negativicutes | Firmicutes | | Bacteria | |
| ASV207 | 427 | 1 | 1 | 427 | 100 | NR_044111.1 Selenomonas bovis strain WG 16S ribosomal RNA, partial sequence | NR_044111.1 | 1525 | 370 | 796 | 28 | 778.6 | 0 | 425/427 | 99 | 0/427 | 0 | Plus | Plus | Bacteria; Firmicutes; Negativicutes; Selenomonadales; Selenomonadaceae; Selenomonas; Selenomonas bovis | Selenomonas bovis | Selenomonas | Selenomonadaceae | Selenomonadales | Negativicutes | Firmicutes | | Bacteria | |
| ASV208 | 404 | 2 | 1 | 404 | 100 | NR_074793.2 Oscillibacter valericigenes strain Sjm18-20 16S ribosomal RNA, complete sequence | NR_074793.2 | 1530 | 372 | 775 | 26.41 | 630.8 | 2E-180 | 383/404 | 94 | 0/404 | 0 | Plus | Plus | Bacteria; Firmicutes; Clostridia; Eubacteriales; Oscillospiraceae; Oscillibacter; Oscillibacter valericigenes | Oscillibacter valericigenes | Oscillibacter | Oscillospiraceae | Eubacteriales | Clostridia | Firmicutes | | Bacteria | |
| ASV209 | 426 | 1 | 1 | 426 | 100 | NR_134029.1 Faecalicoccus acidiformans strain 37-2a 16S ribosomal RNA, partial sequence | NR_134029.1 | 1380 | 357 | 782 | 30.87 | 610.5 | 2E-174 | 396/428 | 92 | 4/428 | 0 | Plus | Plus | Bacteria; Firmicutes; Erysipelotrichia; Erysipelotrichales; Erysipelotrichaceae; Faecalicoccus; Faecalicoccus acidiformans | Faecalicoccus acidiformans | Faecalicoccus | Erysipelotrichaceae | Erysipelotrichales | Erysipelotrichia | Firmicutes | | Bacteria | |
| ASV210 | 422 | 1 | 1 | 422 | 100 | NR_113411.1 Prevotella copri DSM 18205 strain JCM 13464 16S ribosomal RNA, partial sequence | NR_113411.1 | 1493 | 358 | 779 | 28.27 | 708.4 | 0 | 410/423 | 96 | 2/423 | 0 | Plus | Plus | Bacteria; Bacteroidetes; Bacteroidia; Bacteroidales; Prevotellaceae; Prevotella; Prevotella copri | Prevotella copri | Prevotella | Prevotellaceae | Bacteroidales | Bacteroidia | Bacteroidetes | | Bacteria | |
| ASV211 | 405 | 1 | 1 | 405 | 100 | NR_044490.1 Butyricicoccus pullicaecorum strain 25-3 16S ribosomal RNA, partial sequence | NR_044490.1 | 1482 | 366 | 770 | 27.33 | 643.8 | 0 | 386/405 | 95 | 0/405 | 0 | Plus | Plus | Bacteria; Firmicutes; Clostridia; Eubacteriales; Clostridiaceae; Butyricicoccus; Butyricicoccus pullicaecorum | Butyricicoccus pullicaecorum | Butyricicoccus | Clostridiaceae | Eubacteriales | Clostridia | Firmicutes | | Bacteria | |
| ASV212 | 422 | 1 | 1 | 422 | 100 | NR_113411.1 Prevotella copri DSM 18205 strain JCM 13464 16S ribosomal RNA, partial sequence | NR_113411.1 | 1493 | 358 | 779 | 28.27 | 719.5 | 0 | 411/422 | 97 | 0/422 | 0 | Plus | Plus | Bacteria; Bacteroidetes; Bacteroidia; Bacteroidales; Prevotellaceae; Prevotella; Prevotella copri | Prevotella copri | Prevotella | Prevotellaceae | Bacteroidales | Bacteroidia | Bacteroidetes | | Bacteria | |
| ASV213 | 405 | 1 | 1 | 405 | 100 | NR_104907.1 Eubacterium coprostanoligenes strain HL 16S ribosomal RNA, partial sequence | NR_104907.1 | 1446 | 332 | 734 | 27.87 | 475.7 | 7E-134 | 358/407 | 87 | 6/407 | 1 | Plus | Plus | Bacteria; Firmicutes; Clostridia; Eubacteriales; Eubacteriaceae; Eubacterium; Eubacterium coprostanoligenes | Eubacterium coprostanoligenes | Eubacterium | Eubacteriaceae | Eubacteriales | Clostridia | Firmicutes | | Bacteria | |
| ASV214 | 402 | 1 | 1 | 402 | 100 | NR_104846.1 Gemmiger formicilis strain X2-56 16S ribosomal RNA, partial sequence | NR_104846.1 | 1426 | 305 | 706 | 28.19 | 671.5 | 0 | 389/402 | 96 | 0/402 | 0 | Plus | Plus | Bacteria; Firmicutes; Clostridia; Eubacteriales; ; Gemmiger; Gemmiger formicilis | Gemmiger formicilis | Gemmiger | | Eubacteriales | Clostridia | Firmicutes | | Bacteria | |
| ASV215 | 403 | 1 | 1 | 403 | 100 | NR_102884.1 Ruminococcus champanellensis 18P13 = JCM 17042 16S ribosomal RNA, partial sequence | NR_102884.1 | 1494 | 348 | 749 | 26.91 | 577.3 | 2E-164 | 375/405 | 92 | 5/405 | 1 | Plus | Plus | Bacteria; Firmicutes; Clostridia; Eubacteriales; Oscillospiraceae; Ruminococcus; Ruminococcus champanellensis | Ruminococcus champanellensis | Ruminococcus | Oscillospiraceae | Eubacteriales | Clostridia | Firmicutes | | Bacteria | |
| ASV216 | 404 | 1 | 1 | 404 | 100 | NR_118156.1 Oscillibacter ruminantium GH1 16S ribosomal RNA, partial sequence | NR_118156.1 | 1409 | 321 | 724 | 28.67 | 664.1 | 0 | 389/404 | 96 | 0/404 | 0 | Plus | Plus | Bacteria; Firmicutes; Clostridia; Eubacteriales; Oscillospiraceae; Oscillibacter; Oscillibacter ruminantium | Oscillibacter ruminantium | Oscillibacter | Oscillospiraceae | Eubacteriales | Clostridia | Firmicutes | | Bacteria | |
| ASV217 | 405 | 1 | 1 | 405 | 100 | NR_118554.1 Intestinimonas butyriciproducens strain SRB-521-5-I 16S ribosomal RNA, partial sequence | NR_118554.1 | 1475 | 346 | 750 | 27.46 | 677 | 0 | 392/405 | 96 | 0/405 | 0 | Plus | Plus | Bacteria; Firmicutes; Clostridia; Eubacteriales; ; Intestinimonas; Intestinimonas butyriciproducens | Intestinimonas butyriciproducens | Intestinimonas | | Eubacteriales | Clostridia | Firmicutes | | Bacteria | |
| ASV218 | 422 | 5 | 1 | 422 | 100 | NR_044864.1 Hallella seregens ATCC 51272 16S ribosomal RNA, partial sequence | NR_044864.1 | 1463 | 335 | 756 | 28.84 | 619.7 | 3E-177 | 394/423 | 93 | 2/423 | 0 | Plus | Plus | Bacteria; Bacteroidetes; Bacteroidia; Bacteroidales; Prevotellaceae; Prevotella; Hallella seregens | Hallella seregens | Prevotella | Prevotellaceae | Bacteroidales | Bacteroidia | Bacteroidetes | | Bacteria | |
| ASV219 | 427 | 2 | 1 | 427 | 100 | NR_075036.1 Lactobacillus reuteri DSM 20016 16S ribosomal RNA, partial sequence | NR_075036.1 | 1569 | 384 | 810 | 27.21 | 789.6 | 0 | 427/427 | 100 | 0/427 | 0 | Plus | Plus | Bacteria; Firmicutes; Bacilli; Lactobacillales; Lactobacillaceae; Limosilactobacillus; Limosilactobacillus reuteri | Limosilactobacillus reuteri | Limosilactobacillus | Lactobacillaceae | Lactobacillales | Bacilli | Firmicutes | | Bacteria | |
| ASV220 | 402 | 1 | 1 | 402 | 100 | NR_134772.1 Murimonas intestini strain SRB-530-5-H 16S ribosomal RNA, partial sequence | NR_134772.1 | 1433 | 314 | 715 | 28.05 | 688.1 | 0 | 392/402 | 97 | 0/402 | 0 | Plus | Plus | Bacteria; Firmicutes; Clostridia; Eubacteriales; Lachnospiraceae; Murimonas; Murimonas intestini | Murimonas intestini | Murimonas | Lachnospiraceae | Eubacteriales | Clostridia | Firmicutes | | Bacteria | |
| ASV221 | 402 | 1 | 1 | 402 | 100 | NR_024661.1 Eubacterium ruminantium strain GA195 16S ribosomal RNA, partial sequence | NR_024661.1 | 1508 | 378 | 779 | 26.66 | 649.3 | 0 | 386/403 | 95 | 2/403 | 0 | Plus | Plus | Bacteria; Firmicutes; Clostridia; Eubacteriales; Eubacteriaceae; Eubacterium; Eubacterium ruminantium | Eubacterium ruminantium | Eubacterium | Eubacteriaceae | Eubacteriales | Clostridia | Firmicutes | | Bacteria | |
| ASV222 | 403 | 1 | 1 | 403 | 100 | NR_025930.1 Ruminococcus bromii strain ATCC 27255 16S ribosomal RNA, partial sequence | NR_025930.1 | 1380 | 313 | 716 | 29.28 | 662.2 | 0 | 389/404 | 96 | 1/404 | 0 | Plus | Plus | Bacteria; Firmicutes; Clostridia; Eubacteriales; Oscillospiraceae; Ruminococcus; Ruminococcus bromii | Ruminococcus bromii | Ruminococcus | Oscillospiraceae | Eubacteriales | Clostridia | Firmicutes | | Bacteria | |
| ASV223 | 403 | 1 | 3 | 403 | 99.5 | NR_125677.1 Vallitalea pronyensis strain FatNI3 16S ribosomal RNA, partial sequence | NR_125677.1 | 1480 | 365 | 764 | 27.03 | 457.2 | 3E-128 | 351/402 | 87 | 3/402 | 0 | Plus | Plus | Bacteria; Firmicutes; Clostridia; Eubacteriales; Vallitaleaceae; Vallitalea; Vallitalea pronyensis | Vallitalea pronyensis | Vallitalea | Vallitaleaceae | Eubacteriales | Clostridia | Firmicutes | | Bacteria | |
| ASV224 | 405 | 1 | 1 | 405 | 100 | NR_118554.1 Intestinimonas butyriciproducens strain SRB-521-5-I 16S ribosomal RNA, partial sequence | NR_118554.1 | 1475 | 346 | 750 | 27.46 | 582.8 | 4E-166 | 375/405 | 92 | 0/405 | 0 | Plus | Plus | Bacteria; Firmicutes; Clostridia; Eubacteriales; ; Intestinimonas; Intestinimonas butyriciproducens | Intestinimonas butyriciproducens | Intestinimonas | | Eubacteriales | Clostridia | Firmicutes | | Bacteria | |
| ASV225 | 427 | 1 | 1 | 427 | 100 | NR_112902.1 Phascolarctobacterium succinatutens strain YIT 12067 16S ribosomal RNA, partial sequence | NR_112902.1 | 1610 | 444 | 870 | 26.52 | 756.4 | 0 | 421/427 | 98 | 0/427 | 0 | Plus | Plus | Bacteria; Firmicutes; Negativicutes; Acidaminococcales; Acidaminococcaceae; Phascolarctobacterium; Phascolarctobacterium succinatutens | Phascolarctobacterium succinatutens | Phascolarctobacterium | Acidaminococcaceae | Acidaminococcales | Negativicutes | Firmicutes | | Bacteria | |
| ASV226 | 403 | 1 | 1 | 403 | 100 | NR_041960.1 Blautia luti strain BlnIX 16S ribosomal RNA, partial sequence | NR_041960.1 | 1308 | 239 | 641 | 30.81 | 723.2 | 0 | 400/404 | 99 | 2/404 | 0 | Plus | Plus | Bacteria; Firmicutes; Clostridia; Eubacteriales; Lachnospiraceae; Blautia; Blautia luti | Blautia luti | Blautia | Lachnospiraceae | Eubacteriales | Clostridia | Firmicutes | | Bacteria | |
| ASV227 | 422 | 1 | 1 | 422 | 100 | NR_041285.1 Prevotella multisaccharivorax strain PPPA20 16S ribosomal RNA, partial sequence | NR_041285.1 | 1491 | 356 | 777 | 28.3 | 603.1 | 3E-172 | 391/423 | 92 | 2/423 | 0 | Plus | Plus | Bacteria; Bacteroidetes; Bacteroidia; Bacteroidales; Prevotellaceae; Prevotella; Prevotella multisaccharivorax | Prevotella multisaccharivorax | Prevotella | Prevotellaceae | Bacteroidales | Bacteroidia | Bacteroidetes | | Bacteria | |
| ASV228 | 402 | 3 | 1 | 402 | 100 | NR_156081.1 Kineothrix alysoides strain KNHs209 16S ribosomal RNA, partial sequence | NR_156081.1 | 1411 | 353 | 754 | 28.49 | 671.5 | 0 | 389/402 | 96 | 0/402 | 0 | Plus | Plus | Bacteria; Firmicutes; Clostridia; Eubacteriales; Lachnospiraceae; Kineothrix; Kineothrix alysoides | Kineothrix alysoides | Kineothrix | Lachnospiraceae | Eubacteriales | Clostridia | Firmicutes | | Bacteria | |
| ASV229 | 402 | 1 | 1 | 402 | 100 | NR_074634.1 [Eubacterium] rectale ATCC 33656 16S ribosomal RNA, partial sequence | NR_074634.1 | 1521 | 367 | 768 | 26.43 | 726.9 | 0 | 399/402 | 99 | 0/402 | 0 | Plus | Plus | Bacteria; Firmicutes; Clostridia; Eubacteriales; Lachnospiraceae; ; [Eubacterium] rectale | [Eubacterium] rectale | | Lachnospiraceae | Eubacteriales | Clostridia | Firmicutes | | Bacteria | |
| ASV230 | 402 | 2 | 1 | 402 | 100 | NR_144696.1 Clostridium saudiense strain JCC 16S ribosomal RNA, partial sequence | NR_144696.1 | 1478 | 350 | 751 | 27.2 | 743.5 | 0 | 402/402 | 100 | 0/402 | 0 | Plus | Plus | Bacteria; Firmicutes; Clostridia; Eubacteriales; Clostridiaceae; Clostridium; Clostridium saudiense | Clostridium saudiense | Clostridium | Clostridiaceae | Eubacteriales | Clostridia | Firmicutes | | Bacteria | |
| ASV231 | 404 | 2 | 1 | 404 | 100 | NR_074793.2 Oscillibacter valericigenes strain Sjm18-20 16S ribosomal RNA, complete sequence | NR_074793.2 | 1530 | 372 | 775 | 26.41 | 641.9 | 0 | 385/404 | 95 | 0/404 | 0 | Plus | Plus | Bacteria; Firmicutes; Clostridia; Eubacteriales; Oscillospiraceae; Oscillibacter; Oscillibacter valericigenes | Oscillibacter valericigenes | Oscillibacter | Oscillospiraceae | Eubacteriales | Clostridia | Firmicutes | | Bacteria | |
| ASV232 | 404 | 2 | 1 | 404 | 100 | NR_074793.2 Oscillibacter valericigenes strain Sjm18-20 16S ribosomal RNA, complete sequence | NR_074793.2 | 1530 | 372 | 775 | 26.41 | 658.5 | 0 | 389/405 | 96 | 2/405 | 0 | Plus | Plus | Bacteria; Firmicutes; Clostridia; Eubacteriales; Oscillospiraceae; Oscillibacter; Oscillibacter valericigenes | Oscillibacter valericigenes | Oscillibacter | Oscillospiraceae | Eubacteriales | Clostridia | Firmicutes | | Bacteria | |
| ASV233 | 405 | 1 | 1 | 405 | 100 | NR_144742.1 Christensenella massiliensis strain Marseille-P2438 16S ribosomal RNA, partial sequence | NR_144742.1 | 1499 | 369 | 773 | 27.02 | 488.6 | 1E-137 | 358/405 | 88 | 0/405 | 0 | Plus | Plus | Bacteria; Firmicutes; Clostridia; Eubacteriales; Christensenellaceae; Christensenella; Christensenella massiliensis | Christensenella massiliensis | Christensenella | Christensenellaceae | Eubacteriales | Clostridia | Firmicutes | | Bacteria | |
| ASV234 | 427 | 1 | 15 | 427 | 96.72 | NR_025697.1 Spiroplasma alleghenense strain PLHS-1 16S ribosomal RNA, partial sequence | NR_025697.1 | 1516 | 359 | 770 | 27.18 | 420.3 | 4E-117 | 352/414 | 85 | 3/414 | 0 | Plus | Plus | Bacteria; Tenericutes; Mollicutes; Entomoplasmatales; Spiroplasmataceae; Spiroplasma; Spiroplasma alleghenense | Spiroplasma alleghenense | Spiroplasma | Spiroplasmataceae | Entomoplasmatales | Mollicutes | Tenericutes | | Bacteria | |
| ASV235 | 405 | 1 | 1 | 405 | 100 | NR_144742.1 Christensenella massiliensis strain Marseille-P2438 16S ribosomal RNA, partial sequence | NR_144742.1 | 1499 | 369 | 773 | 27.02 | 455.4 | 1E-127 | 352/405 | 86 | 0/405 | 0 | Plus | Plus | Bacteria; Firmicutes; Clostridia; Eubacteriales; Christensenellaceae; Christensenella; Christensenella massiliensis | Christensenella massiliensis | Christensenella | Christensenellaceae | Eubacteriales | Clostridia | Firmicutes | | Bacteria | |
| ASV236 | 404 | 3 | 1 | 404 | 100 | NR_074793.2 Oscillibacter valericigenes strain Sjm18-20 16S ribosomal RNA, complete sequence | NR_074793.2 | 1530 | 372 | 775 | 26.41 | 614.2 | 2E-175 | 381/405 | 94 | 2/405 | 0 | Plus | Plus | Bacteria; Firmicutes; Clostridia; Eubacteriales; Oscillospiraceae; Oscillibacter; Oscillibacter valericigenes | Oscillibacter valericigenes | Oscillibacter | Oscillospiraceae | Eubacteriales | Clostridia | Firmicutes | | Bacteria | |
| ASV237 | 402 | 1 | 1 | 402 | 100 | NR_026106.1 [Eubacterium] cellulosolvens strain 6 16S ribosomal RNA, partial sequence | NR_026106.1 | 1489 | 333 | 734 | 27 | 654.8 | 0 | 386/402 | 96 | 0/402 | 0 | Plus | Plus | Bacteria; Firmicutes; Clostridia; Eubacteriales; Eubacteriaceae; Eubacterium; [Eubacterium] cellulosolvens | [Eubacterium] cellulosolvens | Eubacterium | Eubacteriaceae | Eubacteriales | Clostridia | Firmicutes | | Bacteria | |
| ASV238 | 422 | 1 | 1 | 422 | 100 | NR_040877.1 Prevotella copri DSM 18205 strain CB7 16S ribosomal RNA, partial sequence | NR_040877.1 | 1494 | 358 | 779 | 28.25 | 704.7 | 0 | 408/422 | 96 | 0/422 | 0 | Plus | Plus | Bacteria; Bacteroidetes; Bacteroidia; Bacteroidales; Prevotellaceae; Prevotella; Prevotella copri | Prevotella copri | Prevotella | Prevotellaceae | Bacteroidales | Bacteroidia | Bacteroidetes | | Bacteria | |
| ASV239 | 421 | 2 | 1 | 421 | 100 | NR_113073.1 Barnesiella intestinihominis strain JCM 15079 16S ribosomal RNA, partial sequence | NR_113073.1 | 1481 | 346 | 767 | 28.49 | 496 | 6E-140 | 372/423 | 87 | 3/423 | 0 | Plus | Plus | Bacteria; Bacteroidetes; Bacteroidia; Bacteroidales; Barnesiellaceae; Barnesiella; Barnesiella intestinihominis | Barnesiella intestinihominis | Barnesiella | Barnesiellaceae | Bacteroidales | Bacteroidia | Bacteroidetes | | Bacteria | |
| ASV242 | 427 | 1 | 1 | 427 | 100 | NR_041666.1 Dialister succinatiphilus YIT 11850 16S ribosomal RNA, partial sequence | NR_041666.1 | 1521 | 353 | 779 | 28.07 | 789.6 | 0 | 427/427 | 100 | 0/427 | 0 | Plus | Plus | Bacteria; Firmicutes; Negativicutes; Veillonellales; Veillonellaceae; Dialister; Dialister succinatiphilus | Dialister succinatiphilus | Dialister | Veillonellaceae | Veillonellales | Negativicutes | Firmicutes | | Bacteria | |
| ASV243 | 404 | 5 | 1 | 404 | 100 | NR_115693.1 Gracilibacter thermotolerans JW/YJL-S1 16S ribosomal RNA, partial sequence | NR_115693.1 | 1526 | 459 | 861 | 26.41 | 479.4 | 6E-135 | 360/408 | 88 | 9/408 | 2 | Plus | Plus | Bacteria; Firmicutes; Clostridia; Eubacteriales; Gracilibacteraceae; Gracilibacter; Gracilibacter thermotolerans | Gracilibacter thermotolerans | Gracilibacter | Gracilibacteraceae | Eubacteriales | Clostridia | Firmicutes | | Bacteria | |
| ASV244 | 402 | 1 | 1 | 402 | 100 | NR_118673.1 [Eubacterium] hallii strain ATCC 27751 16S ribosomal RNA, partial sequence | NR_118673.1 | 1479 | 371 | 772 | 27.18 | 728.7 | 0 | 398/402 | 99 | 0/402 | 0 | Plus | Plus | Bacteria; Firmicutes; Clostridia; Eubacteriales; Lachnospiraceae; Anaerobutyricum; Anaerobutyricum hallii | Anaerobutyricum hallii | Anaerobutyricum | Lachnospiraceae | Eubacteriales | Clostridia | Firmicutes | | Bacteria | |
| ASV245 | 422 | 1 | 1 | 422 | 100 | NR_151886.1 Prevotella colorans strain A1336 16S ribosomal RNA, partial sequence | NR_151886.1 | 1491 | 358 | 779 | 28.3 | 614.2 | 2E-175 | 392/422 | 92 | 0/422 | 0 | Plus | Plus | Bacteria; Bacteroidetes; Bacteroidia; Bacteroidales; Prevotellaceae; Prevotella; Prevotella colorans | Prevotella colorans | Prevotella | Prevotellaceae | Bacteroidales | Bacteroidia | Bacteroidetes | | Bacteria | |
| ASV246 | 405 | 2 | 1 | 405 | 100 | NR_117718.1 Desulfotomaculum nigrificans strain NCIMB 8395 16S ribosomal RNA, partial sequence | NR_117718.1 | 1521 | 352 | 756 | 26.63 | 460.9 | 2E-129 | 357/409 | 87 | 8/409 | 1 | Plus | Plus | Bacteria; Firmicutes; Clostridia; E[illegible] | [illegible] | [illegible] | [illegible] | [illegible] | [illegible] | [illegible] | | [illegible] | |

| | | | | | | | | | | | | | | | | | | | | | | | | | | | |
|---|---|---|---|---|---|---|---|---|---|---|---|---|---|---|---|---|---|---|---|---|---|---|---|---|---|---|---|
| ASV259 | 422 | 1 | 1 | 422 | 100 | NR_113411.1 Prevotella copri DSM 18205 strain JCM 13464 16S ribosomal RNA, partial sequence | NR_113411.1 | 1493 | 358 | 779 | 28.27 | 708.4 | 0 | 409/422 | 96 | 0/422 | 0 | Plus | Plus | Bacteria; Bacteroidetes; Bacteroidia; Bacteroidales; Prevotellaceae; Prevotella; Prevotella copri | Prevotella copri | Prevotella | Prevotellaceae | Bacteroidales | Bacteroidia | Bacteroidetes | Bacteria |
| ASV260 | 405 | 1 | 1 | 405 | 100 | NR_148302.1 Geosporobacter ferrireducens strain IRF9 16S ribosomal RNA, partial sequence | NR_148302.1 | 1392 | 306 | 707 | 28.88 | 462.8 | 6E-130 | 358/409 | 87 | 11/409 | 2 | Plus | Plus | Bacteria; Firmicutes; Clostridia; Eubacteriales; Clostridiaceae; Geosporobacter; Geosporobacter ferrireducens | Geosporobacter ferrireducens | Geosporobacter | Clostridiaceae | Eubacteriales | Clostridia | Firmicutes | Bacteria |
| ASV261 | 427 | 2 | 1 | 427 | 100 | NR_104990.1 Desulfovibrio desulfuricans strain Essex 6 16S ribosomal RNA, partial sequence | NR_104990.1 | 1542 | 357 | 783 | 27.69 | 606.8 | 3E-173 | 394/427 | 92 | 0/427 | 0 | Plus | Plus | Bacteria; Proteobacteria; Deltaproteobacteria; Desulfovibrionales; Desulfovibrionaceae; Desulfovibrio; Desulfovibrio desulfuricans | Desulfovibrio desulfuricans | Desulfovibrio | Desulfovibrionaceae | Desulfovibrionales | Deltaproteobacteria | Proteobacteria | Bacteria |
| ASV262 | 422 | 1 | 1 | 422 | 100 | NR_041342.1 Parabacteroides distasonis strain JCM 5825 16S ribosomal RNA, partial sequence | NR_041342.1 | 1488 | 353 | 774 | 28.36 | 558.8 | 8E-159 | 383/423 | 90 | 2/423 | 0 | Plus | Plus | Bacteria; Bacteroidetes; Bacteroidia; Bacteroidales; Tannerellaceae; Parabacteroides; Parabacteroides distasonis | Parabacteroides distasonis | Parabacteroides | Tannerellaceae | Bacteroidales | Bacteroidia | Bacteroidetes | Bacteria |
| ASV263 | 421 | 1 | 1 | 421 | 100 | NR_144616.1 Muribaculum intestinale strain YL27 16S ribosomal RNA, partial sequence | NR_144616.1 | 1460 | 297 | 718 | 28.9 | 484.9 | 1E-136 | 374/427 | 87 | 11/427 | 2 | Plus | Plus | Bacteria; Bacteroidetes; Bacteroidia; Bacteroidales; Muribaculaceae; Muribaculum; Muribaculum intestinale | Muribaculum intestinale | Muribaculum | Muribaculaceae | Bacteroidales | Bacteroidia | Bacteroidetes | Bacteria |
| ASV264 | 422 | 1 | 1 | 422 | 100 | NR_109628.1 Prevotella jejuni strain CD3:28 16S ribosomal RNA, partial sequence | NR_109628.1 | 1484 | 339 | 760 | 28.44 | 597.6 | 2E-170 | 389/422 | 92 | 0/422 | 0 | Plus | Plus | Bacteria; Bacteroidetes; Bacteroidia; Bacteroidales; Prevotellaceae; Prevotella; Prevotella jejuni | Prevotella jejuni | Prevotella | Prevotellaceae | Bacteroidales | Bacteroidia | Bacteroidetes | Bacteria |
| ASV265 | 427 | 1 | 1 | 427 | 100 | NR_118847.1 Actinobacillus minor NM305 16S ribosomal RNA, partial sequence | NR_118847.1 | 1464 | 333 | 759 | 29.17 | 763.8 | 0 | 422/427 | 98 | 0/427 | 0 | Plus | Plus | Bacteria; Proteobacteria; Gammaproteobacteria; Pasteurellales; Pasteurellaceae; Actinobacillus; Actinobacillus minor | Actinobacillus minor | Actinobacillus | Pasteurellaceae | Pasteurellales | Gammaproteobacteria | Proteobacteria | Bacteria |
| ASV266 | 427 | 1 | 1 | 427 | 100 | NR_025131.1 Treponema parvum strain OMZ 833 16S ribosomal RNA, partial sequence | NR_025131.1 | 1485 | 341 | 767 | 28.75 | 595.7 | 6E-170 | 395/430 | 91 | 6/430 | 1 | Plus | Plus | Bacteria; Spirochaetes; Spirochaetia; Spirochaetales; Spirochaetaceae; Treponema; Treponema parvum | Treponema parvum | Treponema | Spirochaetaceae | Spirochaetales | Spirochaetia | Spirochaetes | Bacteria |
| ASV267 | 421 | 2 | 1 | 421 | 100 | NR_113077.1 Paraprevotella clara strain JCM 14859 16S ribosomal RNA, partial sequence | NR_113077.1 | 1480 | 346 | 766 | 28.45 | 512.6 | 6E-145 | 374/422 | 88 | 2/422 | 0 | Plus | Plus | Bacteria; Bacteroidetes; Bacteroidia; Bacteroidales; Prevotellaceae; Paraprevotella; Paraprevotella clara | Paraprevotella clara | Paraprevotella | Prevotellaceae | Bacteroidales | Bacteroidia | Bacteroidetes | Bacteria |
| ASV268 | 402 | 1 | 1 | 402 | 100 | NR_028961.1 Faecalibacterium prausnitzii strain ATCC 27768 16S ribosomal RNA, partial sequence | NR_028961.1 | 1462 | 344 | 745 | 27.5 | 726.9 | 0 | 399/402 | 99 | 0/402 | 0 | Plus | Plus | Bacteria; Firmicutes; Clostridia; Eubacteriales; Oscillospiraceae; Faecalibacterium; Faecalibacterium prausnitzii | Faecalibacterium prausnitzii | Faecalibacterium | Oscillospiraceae | Eubacteriales | Clostridia | Firmicutes | Bacteria |
| ASV269 | 427 | 1 | 1 | 427 | 100 | NR_041611.1 Lactobacillus ruminis strain NBRC 102161 16S ribosomal RNA, partial sequence | NR_041611.1 | 1488 | 352 | 778 | 28.7 | 789.6 | 0 | 427/427 | 100 | 0/427 | 0 | Plus | Plus | Bacteria; Firmicutes; Bacilli; Lactobacillales; Lactobacillaceae; Ligilactobacillus; Ligilactobacillus ruminis | Ligilactobacillus ruminis | Ligilactobacillus | Lactobacillaceae | Lactobacillales | Bacilli | Firmicutes | Bacteria |
| ASV270 | 422 | 2 | 1 | 422 | 100 | NR_113119.1 Prevotella oris strain JCM 8540 16S ribosomal RNA, partial sequence | NR_113119.1 | 1481 | 345 | 766 | 28.49 | 603.1 | 3E-172 | 392/424 | 92 | 4/424 | 0 | Plus | Plus | Bacteria; Bacteroidetes; Bacteroidia; Bacteroidales; Prevotellaceae; Prevotella; Prevotella oris | Prevotella oris | Prevotella | Prevotellaceae | Bacteroidales | Bacteroidia | Bacteroidetes | Bacteria |
| ASV271 | 427 | 1 | 1 | 427 | 100 | NR_113163.1 Faecalitalea cylindroides strain JCM 10261 16S ribosomal RNA, partial sequence | NR_113163.1 | 1504 | 359 | 784 | 28.32 | 643.8 | 0 | 402/428 | 93 | 3/428 | 0 | Plus | Plus | Bacteria; Firmicutes; Erysipelotrichia; Erysipelotrichales; Erysipelotrichaceae; Faecalitalea; Faecalitalea cylindroides | Faecalitalea cylindroides | Faecalitalea | Erysipelotrichaceae | Erysipelotrichales | Erysipelotrichia | Firmicutes | Bacteria |
| ASV272 | 426 | 1 | 1 | 426 | 100 | NR_025333.1 Leminorella grimontii strain 81H-380. 16S ribosomal RNA, partial sequence | NR_025333.1 | 1482 | 321 | 747 | 28.81 | 472 | 1E-132 | 376/433 | 86 | 13/433 | 3 | Plus | Plus | Bacteria; Proteobacteria; Gammaproteobacteria; Enterobacterales; Budviciaceae; Leminorella; Leminorella grimontii | Leminorella grimontii | Leminorella | Budviciaceae | Enterobacterales | Gammaproteobacteria | Proteobacteria | Bacteria |
| ASV273 | 426 | 2 | 1 | 426 | 100 | NR_025333.1 Leminorella grimontii strain 81H-380. 16S ribosomal RNA, partial sequence | NR_025333.1 | 1482 | 321 | 747 | 28.81 | 466.5 | 5E-131 | 375/433 | 86 | 13/433 | 3 | Plus | Plus | Bacteria; Proteobacteria; Gammaproteobacteria; Enterobacterales; Budviciaceae; Leminorella; Leminorella grimontii | Leminorella grimontii | Leminorella | Budviciaceae | Enterobacterales | Gammaproteobacteria | Proteobacteria | Bacteria |
| ASV274 | 402 | 1 | 1 | 402 | 100 | NR_118676.1 Eubacterium xylanophilum strain ATCC 35991 16S ribosomal RNA, partial sequence | NR_118676.1 | 1494 | 387 | 788 | 26.91 | 691.8 | 0 | 391/402 | 97 | 0/402 | 0 | Plus | Plus | Bacteria; Firmicutes; Clostridia; Eubacteriales; Eubacteriaceae; Eubacterium; Eubacterium xylanophilum | Eubacterium xylanophilum | Eubacterium | Eubacteriaceae | Eubacteriales | Clostridia | Firmicutes | Bacteria |
| ASV275 | 422 | 2 | 1 | 422 | 100 | NR_113117.1 Prevotella oralis ATCC 33269 strain JCM 12251 16S ribosomal RNA, partial sequence | NR_113117.1 | 1496 | 361 | 782 | 28.21 | 564.3 | 2E-160 | 385/424 | 90 | 4/424 | 0 | Plus | Plus | Bacteria; Bacteroidetes; Bacteroidia; Bacteroidales; Prevotellaceae; Prevotella; Prevotella oralis | Prevotella oralis | Prevotella | Prevotellaceae | Bacteroidales | Bacteroidia | Bacteroidetes | Bacteria |
| ASV276 | 422 | 3 | 1 | 422 | 100 | NR_113109.1 Prevotella loescheii strain JCM 8530 16S ribosomal RNA, partial sequence | NR_113109.1 | 1476 | 342 | 763 | 28.59 | 542.2 | 8E-154 | 381/424 | 89 | 4/424 | 0 | Plus | Plus | Bacteria; Bacteroidetes; Bacteroidia; Bacteroidales; Prevotellaceae; Prevotella; Prevotella loescheii | Prevotella loescheii | Prevotella | Prevotellaceae | Bacteroidales | Bacteroidia | Bacteroidetes | Bacteria |
| ASV277 | 403 | 1 | 1 | 403 | 100 | NR_074809.1 Roseburia hominis A2-183 16S ribosomal RNA, partial sequence | NR_074809.1 | 1535 | 367 | 769 | 26.25 | 745.3 | 0 | 403/403 | 100 | 0/403 | 0 | Plus | Plus | Bacteria; Firmicutes; Clostridia; Eubacteriales; Lachnospiraceae; Roseburia; Roseburia hominis | Roseburia hominis | Roseburia | Lachnospiraceae | Eubacteriales | Clostridia | Firmicutes | Bacteria |
| ASV278 | 404 | 1 | 1 | 404 | 100 | NR_118156.1 Oscillibacter ruminantium GH1 16S ribosomal RNA, partial sequence | NR_118156.1 | 1409 | 321 | 724 | 28.67 | 575.4 | 7E-164 | 376/407 | 92 | 6/407 | 1 | Plus | Plus | Bacteria; Firmicutes; Clostridia; Eubacteriales; Oscillospiraceae; Oscillibacter; Oscillibacter ruminantium | Oscillibacter ruminantium | Oscillibacter | Oscillospiraceae | Eubacteriales | Clostridia | Firmicutes | Bacteria |
| ASV279 | 422 | 1 | 1 | 422 | 100 | NR_041285.1 Prevotella multisaccharivorax strain PPPA20 16S ribosomal RNA, partial sequence | NR_041285.1 | 1491 | 356 | 777 | 28.3 | 597.6 | 2E-170 | 390/423 | 92 | 2/423 | 0 | Plus | Plus | Bacteria; Bacteroidetes; Bacteroidia; Bacteroidales; Prevotellaceae; Prevotella; Prevotella multisaccharivorax | Prevotella multisaccharivorax | Prevotella | Prevotellaceae | Bacteroidales | Bacteroidia | Bacteroidetes | Bacteria |
| ASV280 | 427 | 1 | 1 | 427 | 100 | NR_044111.1 Selenomonas bovis strain WG 16S ribosomal RNA, partial sequence | NR_044111.1 | 1525 | 370 | 796 | 28 | 662.2 | 0 | 407/430 | 94 | 6/430 | 1 | Plus | Plus | Bacteria; Firmicutes; Negativicutes; Selenomonadales; Selenomonadaceae; Selenomonas; Selenomonas bovis | Selenomonas bovis | Selenomonas | Selenomonadaceae | Selenomonadales | Negativicutes | Firmicutes | Bacteria |
| ASV281 | 402 | 1 | 1 | 402 | 100 | NR_118674.1 Eubacterium ramulus ATCC 29099 16S ribosomal RNA, partial sequence | NR_118674.1 | 1474 | 373 | 774 | 27.27 | 588.4 | 9E-168 | 371/402 | 92 | 0/402 | 0 | Plus | Plus | Bacteria; Firmicutes; Clostridia; Eubacteriales; Eubacteriaceae; Eubacterium; Eubacterium ramulus | Eubacterium ramulus | Eubacterium | Eubacteriaceae | Eubacteriales | Clostridia | Firmicutes | Bacteria |
| ASV282 | 403 | 1 | 3 | 403 | 99.5 | NR_125677.1 Vallitalea pronyensis strain FatNI3 16S ribosomal RNA, partial sequence | NR_125677.1 | 1480 | 365 | 764 | 27.03 | 451.7 | 1E-126 | 350/402 | 87 | 3/402 | 0 | Plus | Plus | Bacteria; Firmicutes; Clostridia; Eubacteriales; Vallitaleaceae; Vallitalea; Vallitalea pronyensis | Vallitalea pronyensis | Vallitalea | Vallitaleaceae | Eubacteriales | Clostridia | Firmicutes | Bacteria |
| ASV283 | 422 | 1 | 1 | 422 | 100 | NR_113411.1 Prevotella copri DSM 18205 strain JCM 13464 16S ribosomal RNA, partial sequence | NR_113411.1 | 1493 | 358 | 779 | 28.27 | 752.7 | 0 | 417/422 | 98 | 0/422 | 0 | Plus | Plus | Bacteria; Bacteroidetes; Bacteroidia; Bacteroidales; Prevotellaceae; Prevotella; Prevotella copri | Prevotella copri | Prevotella | Prevotellaceae | Bacteroidales | Bacteroidia | Bacteroidetes | Bacteria |
| ASV285 | 402 | 1 | 1 | 402 | 100 | NR_025931.1 Ruminococcus flavefaciens strain C94 16S ribosomal RNA, partial sequence | NR_025931.1 | 1388 | 325 | 726 | 28.96 | 721.3 | 0 | 398/402 | 99 | 0/402 | 0 | Plus | Plus | Bacteria; Firmicutes; Clostridia; Eubacteriales; Oscillospiraceae; Ruminococcus; Ruminococcus flavefaciens | Ruminococcus flavefaciens | Ruminococcus | Oscillospiraceae | Eubacteriales | Clostridia | Firmicutes | Bacteria |
| ASV286 | 403 | 1 | 1 | 403 | 100 | NR_042930.1 Acetanaerobacterium elongatum strain Z7 16S ribosomal RNA, partial sequence | NR_042930.1 | 1533 | 383 | 783 | 26.16 | 549.6 | 4E-156 | 368/403 | 91 | 2/403 | 0 | Plus | Plus | Bacteria; Firmicutes; Clostridia; Eubacteriales; Oscillospiraceae; Acetanaerobacterium; Acetanaerobacterium elongatum | Acetanaerobacterium elongatum | Acetanaerobacterium | Oscillospiraceae | Eubacteriales | Clostridia | Firmicutes | Bacteria |
| ASV287 | 402 | 1 | 1 | 402 | 100 | NR_118675.1 [Eubacterium] siraeum strain ATCC 29066 16S ribosomal RNA, partial sequence | NR_118675.1 | 1453 | 356 | 757 | 27.67 | 595.7 | 5E-170 | 374/402 | 93 | 0/402 | 0 | Plus | Plus | Bacteria; Firmicutes; Clostridia; Eubacteriales; Oscillospiraceae; ; [Eubacterium] siraeum | [Eubacterium] siraeum | | Oscillospiraceae | Eubacteriales | Clostridia | Firmicutes | Bacteria |
| ASV288 | 427 | 1 | 1 | 427 | 100 | NR_044111.1 Selenomonas bovis strain WG 16S ribosomal RNA, partial sequence | NR_044111.1 | 1525 | 370 | 796 | 28 | 629 | 6E-180 | 403/432 | 93 | 10/432 | 2 | Plus | Plus | Bacteria; Firmicutes; Negativicutes; Selenomonadales; Selenomonadaceae; Selenomonas; Selenomonas bovis | Selenomonas bovis | Selenomonas | Selenomonadaceae | Selenomonadales | Negativicutes | Firmicutes | Bacteria |
| ASV290 | 404 | 1 | 1 | 404 | 100 | NR_118156.1 Oscillibacter ruminantium GH1 16S ribosomal RNA, partial sequence | NR_118156.1 | 1409 | 321 | 724 | 28.67 | 608.7 | 7E-174 | 380/405 | 93 | 2/405 | 0 | Plus | Plus | Bacteria; Firmicutes; Clostridia; Eubacteriales; Oscillospiraceae; Oscillibacter; Oscillibacter ruminantium | Oscillibacter ruminantium | Oscillibacter | Oscillospiraceae | Eubacteriales | Clostridia | Firmicutes | Bacteria |
| ASV291 | 422 | 1 | 1 | 422 | 100 | NR_041285.1 Prevotella multisaccharivorax strain PPPA20 16S ribosomal RNA, partial sequence | NR_041285.1 | 1491 | 356 | 777 | 28.3 | 625.3 | 7E-179 | 394/422 | 93 | 0/422 | 0 | Plus | Plus | Bacteria; Bacteroidetes; Bacteroidia; Bacteroidales; Prevotellaceae; Prevotella; Prevotella multisaccharivorax | Prevotella multisaccharivorax | Prevotella | Prevotellaceae | Bacteroidales | Bacteroidia | Bacteroidetes | Bacteria |
| ASV292 | 402 | 1 | 1 | 402 | 100 | NR_134772.1 Murimonas intestini strain SRB-530-5-H 16S ribosomal RNA, partial sequence | NR_134772.1 | 1433 | 314 | 715 | 28.05 | 699.2 | 0 | 394/402 | 98 | 0/402 | 0 | Plus | Plus | Bacteria; Firmicutes; Clostridia; Eubacteriales; Lachnospiraceae; Murimonas; Murimonas intestini | Murimonas intestini | Murimonas | Lachnospiraceae | Eubacteriales | Clostridia | Firmicutes | Bacteria |
| ASV294 | 422 | 2 | 1 | 422 | 100 | NR_113119.1 Prevotella oris strain JCM 8540 16S ribosomal RNA, partial sequence | NR_113119.1 | 1481 | 345 | 766 | 28.49 | 564.3 | 2E-160 | 384/423 | 90 | 2/423 | 0 | Plus | Plus | Bacteria; Bacteroidetes; Bacteroidia; Bacteroidales; Prevotellaceae; Prevotella; Prevotella oris | Prevotella oris | Prevotella | Prevotellaceae | Bacteroidales | Bacteroidia | Bacteroidetes | Bacteria |
| ASV295 | 422 | 2 | 1 | 422 | 100 | NR_074376.1 Parabacteroides distasonis ATCC 8503 16S ribosomal RNA, partial sequence | NR_074376.1 | 1497 | 368 | 789 | 28.19 | 553.3 | 4E-157 | 383/424 | 90 | 4/424 | 0 | Plus | Plus | Bacteria; Bacteroidetes; Bacteroidia; Bacteroidales; Tannerellaceae; Parabacteroides; Parabacteroides distasonis | Parabacteroides distasonis | Parabacteroides | Tannerellaceae | Bacteroidales | Bacteroidia | Bacteroidetes | Bacteria |
| ASV296 | 422 | 1 | 1 | 422 | 100 | NR_113411.1 Prevotella copri DSM 18205 strain JCM 13464 16S ribosomal RNA, partial sequence | NR_113411.1 | 1493 | 358 | 779 | 28.27 | 763.8 | 0 | 419/422 | 99 | 0/422 | 0 | Plus | Plus | Bacteria; Bacteroidetes; Bacteroidia; Bacteroidales; Prevotellaceae; Prevotella; Prevotella copri | Prevotella copri | Prevotella | Prevotellaceae | Bacteroidales | Bacteroidia | Bacteroidetes | Bacteria |
| ASV297 | 422 | 2 | 1 | 422 | 100 | NR_113119.1 Prevotella oris strain JCM 8540 16S ribosomal RNA, partial sequence | NR_113119.1 | 1481 | 345 | 766 | 28.49 | 531.1 | 2E-150 | 378/423 | 89 | 2/423 | 0 | Plus | Plus | Bacteria; Bacteroidetes; Bacteroidia; Bacteroidales; Prevotellaceae; Prevotella; Prevotella oris | Prevotella oris | Prevotella | Prevotellaceae | Bacteroidales | Bacteroidia | Bacteroidetes | Bacteria |
| ASV298 | 422 | 1 | 1 | 422 | 100 | NR_024815.1 Prevotella shahii strain EHS11 16S ribosomal RNA, partial sequence | NR_024815.1 | 1490 | 356 | 777 | 28.32 | 547.7 | 2E-155 | 382/424 | 90 | 4/424 | 0 | Plus | Plus | Bacteria; Bacteroidetes; Bacteroidia; Bacteroidales; Prevotellaceae; Prevotella; Prevotella shahii | Prevotella shahii | Prevotella | Prevotellaceae | Bacteroidales | Bacteroidia | Bacteroidetes | Bacteria |
| ASV299 | 402 | 1 | 1 | 402 | 100 | NR_044054.1 Blautia wexlerae DSM 19850 16S ribosomal RNA, partial sequence | NR_044054.1 | 1438 | 314 | 715 | 27.96 | 732.4 | 0 | 400/402 | 99 | 0/402 | 0 | Plus | Plus | Bacteria; Firmicutes; Clostridia; Eubacteriales; Lachnospiraceae; Blautia; Blautia wexlerae | Blautia wexlerae | Blautia | Lachnospiraceae | Eubacteriales | Clostridia | Firmicutes | Bacteria |
| ASV300 | 422 | 1 | 1 | 422 | 100 | NR_113411.1 Prevotella copri DSM 18205 strain JCM 13464 16S ribosomal RNA, partial sequence | NR_113411.1 | 1493 | 358 | 779 | 28.27 | 774.9 | 0 | 421/422 | 99 | 0/422 | 0 | Plus | Plus | Bacteria; Bacteroidetes; Bacteroidia; Bacteroidales; Prevotellaceae; Prevotella; Prevotella copri | Prevotella copri | Prevotella | Prevotellaceae | Bacteroidales | Bacteroidia | Bacteroidetes | Bacteria |
| ASV301 | 402 | 4 | 1 | 402 | 100 | NR_134772.1 Murimonas intestini strain SRB-530-5-H 16S ribosomal RNA, partial sequence | NR_134772.1 | 1433 | 314 | 715 | 28.05 | 660.4 | 0 | 387/402 | 96 | 0/402 | 0 | Plus | Plus | Bacteria; Firmicutes; Clostridia; Eubacteriales; Lachnospiraceae; Murimonas; Murimonas intestini | Murimonas intestini | Murimonas | Lachnospiraceae | Eubacteriales | Clostridia | Firmicutes | Bacteria |
| ASV302 | 404 | 1 | 1 | 404 | 100 | NR_027203.1 Mogibacterium neglectum strain P9a-h 16S ribosomal RNA, partial sequence | NR_027203.1 | 1478 | 362 | 765 | 27.33 | 630.8 | 2E-180 | 383/404 | 94 | 0/404 | 0 | Plus | Plus | Bacteria; Firmicutes; Clostridia; Eubacteriales; Clostridiales Family XIII. Incertae Sedis; Mogibacterium; Mogibacterium neglectum | Mogibacterium neglectum | Mogibacterium | Clostridiales Family XIII. Incertae Sedis | Eubacteriales | Clostridia | Firmicutes | Bacteria |
| ASV305 | 422 | 3 | 1 | 422 | 100 | NR_113105.1 Prevotella histicola strain JCM 15637 16S ribosomal RNA, partial sequence | NR_113105.1 | 1480 | 346 | 767 | 28.51 | 630.8 | 2E-180 | 395/422 | 93 | 0/422 | 0 | Plus | Plus | Bacteria; Bacteroidetes; Bacteroidia; Bacteroidales; Prevotellaceae; Prevotella; Prevotella histicola | Prevotella histicola | Prevotella | Prevotellaceae | Bacteroidales | Bacteroidia | Bacteroidetes | Bacteria |
| ASV306 | 403 | 1 | 1 | 403 | 100 | NR_025930.1 Ruminococcus bromii strain ATCC 27255 16S ribosomal RNA, partial sequence | NR_025930.1 | 1380 | 313 | 716 | 29.28 | 734.2 | 0 | 402/404 | 99 | 1/404 | 0 | Plus | Plus | Bacteria; Firmicutes; Clostridia; Eubacteriales; Oscillospiraceae; Ruminococcus; Ruminococcus bromii | Ruminococcus bromii | Ruminococcus | Oscillospiraceae | Eubacteriales | Clostridia | Firmicutes | Bacteria |
| ASV307 | 422 | 2 | 1 | 422 | 100 | NR_113119.1 Prevotella oris strain JCM 8540 16S ribosomal RNA, partial sequence | NR_113119.1 | 1481 | 345 | 766 | 28.49 | 564.3 | 2E-160 | 385/424 | 90 | 4/424 | 0 | Plus | Plus | Bacteria; Bacteroidetes; Bacteroidia; Bacteroidales; Prevotellaceae; Prevotella; Prevotella oris | Prevotella oris | Prevotella | Prevotellaceae | Bacteroidales | Bacteroidia | Bacteroidetes | Bacteria |
| ASV308 | 427 | 1 | 1 | 427 | 100 | NR_074755.1 Treponema succinifaciens DSM 2489 16S ribosomal RNA, partial sequence | NR_074755.1 | 1531 | 353 | 779 | 27.89 | 789.6 | 0 | 427/427 | 100 | 0/427 | 0 | Plus | Plus | Bacteria; Spirochaetes; Spirochaetia; Spirochaetales; Spirochaetaceae; Treponema; Treponema succinifaciens | Treponema succinifaciens | Treponema | Spirochaetaceae | Spirochaetales | Spirochaetia | Spirochaetes | Bacteria |
| ASV309 | 402 | 1 | 1 | 402 | 100 | NR_026106.1 [Eubacterium] cellulosolvens strain 6 16S ribosomal RNA, partial sequence | NR_026106.1 | 1489 | 333 | 734 | 27 | 632.7 | 0 | 382/402 | 95 | 0/402 | 0 | Plus | Plus | Bacteria; Firmicutes; Clostridia; Eubacteriales; Eubacteriaceae; Eubacterium; [Eubacterium] cellulosolvens | [Eubacterium] cellulosolvens | Eubacterium | Eubacteriaceae | Eubacteriales | Clostridia | Firmicutes | Bacteria |
| ASV310 | 401 | 1 | 1 | 401 | 100 | NR_144749.1 Ihubacter massiliensis strain Marseille-P2843 16S ribosomal RNA, partial sequence | NR_144749.1 | 1479 | 364 | 766 | 27.25 | 544 | 2E-154 | 368/404 | 91 | 4/404 | 0 | Plus | Plus | Bacteria; Firmicutes; Clostridia; Eubacteriales; Clostridiales Family XIII. Incertae Sedis; Ihubacter; Ihubacter massiliensis | Ihubacter massiliensis | Ihubacter | Clostridiales Family XIII. Incertae Sedis | Eubacteriales | Clostridia | Firmicutes | Bacteria |
| ASV311 | 402 | 1 | 1 | 402 | 100 | NR_118674.1 Eubacterium ramulus ATCC 29099 16S ribosomal RNA, partial sequence | NR_118674.1 | 1474 | 373 | 774 | 27.27 | 593.9 | 2E-169 | 372/402 | 92 | 0/402 | 0 | Plus | Plus | Bacteria; Firmicutes; Clostridia; Eubacteriales; Eubacteriaceae; Eubacterium; Eubacterium ramulus | Eubacterium ramulus | Eubacterium | Eubacteriaceae | Eubacteriales | Clostridia | Firmicutes | Bacteria |
| ASV312 | 402 | 1 | 1 | 402 | 100 | NR_041960.1 Blautia luti strain BlnIX 16S ribosomal RNA, partial sequence | NR_041960.1 | 1308 | 239 | 641 | 30.81 | 737.9 | 0 | 402/403 | 99 | 1/403 | 0 | Plus | Plus | Bacteria; Firmicutes; Clostridia; Eubacteriales; Lachnospiraceae; Blautia; Blautia luti | Blautia luti | Blautia | Lachnospiraceae | Eubacteriales | Clostridia | Firmicutes | Bacteria |
| ASV313 | 427 | 1 | 1 | 427 | 100 | NR_112902.1 Phascolarctobacterium succinatutens strain YIT 12067 16S ribosomal RNA, partial sequence | NR_112902.1 | 1610 | 444 | 870 | 26.52 | 789.6 | 0 | 427/427 | 100 | 0/427 | 0 | Plus | Plus | Bacteria; Firmicutes; Negativicutes; Acidaminococcales; Acidaminococcaceae; Phascolarctobacterium; Phascolarctobacterium succinatutens | Phascolarctobacterium succinatutens | Phascolarctobacterium | Acidaminococcaceae | Acidaminococcales | Negativicutes | Firmicutes | Bacteria |
| ASV314 | 422 | 1 | 1 | 422 | 100 | NR_113411.1 Prevotella copri DSM 18205 strain JCM 13464 16S ribosomal RNA, partial sequence | NR_113411.1 | 1493 | 358 | 779 | 28.27 | 774.9 | 0 | 421/422 | 99 | 0/422 | 0 | Plus | Plus | Bacteria; Bacteroidetes; Bacteroidia; Bacteroidales; Prevotellaceae; Prevotella; Prevotella copri | Prevotella copri | Prevotella | Prevotellaceae | Bacteroidales | Bacteroidia | Bacteroidetes | Bacteria |
| ASV315 | 422 | 4 | 1 | 422 | 100 | NR_048864.1 Hallella seregens ATCC 51272 16S ribosomal RNA, partial sequence | NR_048864.1 | 1463 | 335 | 756 | 28.84 | 564.3 | 2E-160 | 383/422 | 90 | 0/422 | 0 | Plus | Plus | Bacteria; Bacteroidetes; Bacteroidia; Bacteroidales; Prevotellaceae; Prevotella; Hallella seregens | Hallella seregens | Prevotella | Prevotellaceae | Bacteroidales | Bacteroidia | Bacteroidetes | Bacteria |
| ASV317 | 404 | 1 | 1 | 404 | 100 | NR_118156.1 Oscillibacter ruminantium GH1 16S ribosomal RNA, partial sequence | NR_118156.1 | 1409 | 321 | 724 | 28.67 | 614.2 | 2E-175 | 382/406 | 94 | 4/406 | 0 | Plus | Plus | Bacteria; Firmicutes; Clostridia; Eubacteriales; Oscillospiraceae; Oscillibacter; Oscillibacter ruminantium | Oscillibacter ruminantium | Oscillibacter | Oscillospiraceae | Eubacteriales | Clostridia | Firmicutes | Bacteria |
| ASV318 | 422 | 3 | 1 | 422 | 100 | NR_041818.1 Bacteroides coprosuis DSM 18011 strain PC139 16S ribosomal RNA, partial sequence | NR_041818.1 | 1523 | 357 | 778 | 27.71 | 492.3 | 8E-139 | 373/425 | 87 | 6/425 | 1 | Plus | Plus | Bacteria; Bacteroidetes; Bacteroidia; Bacteroidales; Bacteroidaceae; Bacteroides; Bacteroides coprosuis | Bacteroides coprosuis | Bacteroides | Bacteroidaceae | Bacteroidales | Bacteroidia | Bacteroidetes | Bacteria |
| ASV319 | 404 | 1 | 1 | 404 | 100 | NR_025930.1 Ruminococcus bromii strain ATCC 27255 16S ribosomal RNA, partial sequence | NR_025930.1 | 1380 | 313 | 716 | 29.28 | 702.8 | 0 | 397/405 | 98 | 2/405 | 0 | Plus | Plus | Bacteria; Firmicutes; Clostridia; Eubacteriales; Oscillospiraceae; Ruminococcus; Ruminococcus bromii | Ruminococcus bromii | Ruminococcus | Oscillospiraceae | Eubacteriales | Clostridia | Firmicutes | Bacteria |
| ASV320 | 427 | 1 | 1 | 427 | 100 | NR_025762.1 Veillonella caviae strain PVT 16S ribosomal RNA, partial sequence | NR_025762.1 | 1402 | 321 | 747 | 30.46 | 684.4 | 0 | 410/429 | 95 | 4/429 | 0 | Plus | Plus | Bacteria; Firmicutes; Negativicutes; Veillonellales; Veillonellaceae; Veillonella; Veillonella caviae | Veillonella caviae | Veillonella | Veillonellaceae | Veillonellales | Negativicutes | Firmicutes | Bacteria |
| ASV321 | 423 | 1 | 1 | 423 | 100 | NR_144750.1 Prevotellamassilia timonensis strain Marseille-P2831 16S ribosomal RNA, partial sequence | NR_144750.1 | 1493 | 358 | 779 | 28.27 | 558.8 | 8E-159 | 385/425 | 90 | 5/425 | 1 | Plus | Plus | Bacteria; Bacteroidetes; Bacteroidia; Bacteroidales; Prevotellaceae; Prevotellamassilia; Prevotellamassilia timonensis | Prevotellamassilia timonensis | Prevotellamassilia | Prevotellaceae | Bacteroidales | Bacteroidia | Bacteroidetes | Bacteria |
| ASV322 | 405 | 1 | 1 | 405 | 100 | NR_118554.1 Intestinimonas butyriciproducens strain SRB-521-5-I 16S ribosomal RNA, partial sequence | NR_118554.1 | 1475 | 346 | 750 | 27.46 | 521.9 | 9E-148 | 367/408 | 89 | 6/408 | 1 | Plus | Plus | Bacteria; Firmicutes; Clostridia; Eubacteriales; ; Intestinimonas; Intestinimonas butyriciproducens | Intestinimonas butyriciproducens | Intestinimonas | | Eubacteriales | Clostridia | Firmicutes | Bacteria |
| ASV323 | 427 | 1 | 1 | 427 | 100 | NR_041778.1 Desulfovibrio piger strain ATCC 29098 16S ribosomal RNA, partial sequence | NR_041778.1 | 1542 | 357 | 783 | 27.69 | 778.6 | 0 | 425/427 | 99 | 0/427 | 0 | Plus | Plus | Bacteria; Proteobacteria; Deltaproteobacteria; Desulfovibrionales; Desulfovibrionaceae; Desulfovibrio; Desulfovibrio piger | Desulfovibrio piger | Desulfovibrio | Desulfovibrionaceae | Desulfovibrionales | Deltaproteobacteria | Proteobacteria | Bacteria |
| ASV324 | 423 | 1 | 1 | 423 | 100 | NR_147368.1 Millionella massiliensis strain Marseille-P3215 16S ribosomal RNA, partial sequence | NR_147368.1 | 1492 | 355 | 776 | 28.28 | 448 | 2E-125 | 368/428 | 85 | 11/428 | 2 | Plus | Plus | Bacteria; Bacteroidetes; Bacteroidia; Bacteroidales; Rikenellaceae; Millionella; Millionella massiliensis | Millionella massiliensis | Millionella | Rikenellaceae | Bacteroidales | Bacteroidia | Bacteroidetes | Bacteria |
| ASV325 | 422 | 1 | 1 | 422 | 100 | NR_041364.1 Prevotella stercorea DSM 18206 strain CB35 16S ribosomal RNA, partial sequence | NR_041364.1 | 1488 | 354 | 775 | 28.36 | 736.1 | 0 | 414/422 | 98 | 0/422 | 0 | Plus | Plus | Bacteria; Bacteroidetes; Bacteroidia; Bacteroidales; Prevotellaceae; Prevotella; Prevotella stercorea | Prevotella stercorea | Prevotella | Prevotellaceae | Bacteroidales | Bacteroidia | Bacteroidetes | Bacteria |
| ASV326 | 402 | 3 | 1 | 402 | 100 | NR_026409.1 [Desulfotomaculum] guttoideum strain DSM 4024 16S ribosomal RNA, partial sequence | NR_026409.1 | 1495 | 358 | 759 | 26.89 | 682.5 | 0 | 391/402 | 97 | 0/402 | 0 | Plus | Plus | Bacteria; Firmicutes; Clostridia; Eubacteriales; Lachnospiraceae; Lacrimispora; Lacrimispora sphenoides | Lacrimispora sphenoides | Lacrimispora | Lachnospiraceae | Eubacteriales | Clostridia | Firmicutes | Bacteria |
| ASV328 | 402 | 1 | 1 | 402 | 100 | NR_118675.1 [Eubacterium] siraeum strain ATCC 29066 16S ribosomal RNA, partial sequence | NR_118675.1 | 1453 | 356 | 757 | 27.67 | 706.5 | 0 | 394/402 | 98 | 0/402 | 0 | Plus | Plus | Bacteria; Firmicutes; Clostridia; Eubacteriales; Oscillospiraceae; ; [Eubacterium] siraeum | [Eubacterium] siraeum | | Oscillospiraceae | Eubacteriales | Clostridia | Firmicutes | Bacteria |
| ASV329 | 402 | 1 | 1 | 402 | 100 | NR_029239.3 Clostridium chartatabidum strain 163 16S ribosomal RNA, partial sequence | NR_029239.3 | 1460 | 323 | 724 | 27.53 | 737.9 | 0 | 401/402 | 99 | 0/402 | 0 | Plus | Plus | Bacteria; Firmicutes; Clostridia; Eubacteriales; Clostridiaceae; Clostridium; Clostridium chartatabidum | Clostridium chartatabidum | Clostridium | Clostridiaceae | Eubacteriales | Clostridia | Firmicutes | Bacteria |
| ASV330 | 401 | 1 | 1 | 401 | 100 | NR_137408.1 Terrisporobacter petrolearius strain LAM0A37 16S ribosomal RNA, partial sequence | NR_137408.1 | 1405 | 307 | 707 | 28.54 | 719.5 | 0 | 397/401 | 99 | 0/401 | 0 | Plus | Plus | Bacteria; Firmicutes; Clostridia; Eubacteriales; Peptostreptococcaceae; Terrisporobacter; Terrisporobacter petrolearius | Terrisporobacter petrolearius | Terrisporobacter | Peptostreptococcaceae | Eubacteriales | Clostridia | Firmicutes | Bacteria |
| ASV331 | 402 | 2 | 1 | 402 | 100 | NR_144696.1 Clostridium saudiense strain JCC 16S ribosomal RNA, partial sequence | NR_144696.1 | 1478 | 350 | 751 | 27.2 | 699.2 | 0 | 394/402 | 98 | 0/402 | 0 | Plus | Plus | Bacteria; Firmicutes; Clostridia; Eubacteriales; Clostridiaceae; Clostridium; Clostridium saudiense | Clostridium saudiense | Clostridium | Clostridiaceae | Eubacteriales | Clostridia | Firmicutes | Bacteria |
| ASV332 | 421 | 2 | 1 | 421 | 100 | NR_113077.1 Paraprevotella clara strain JCM 14859 16S ribosomal RNA, partial sequence | NR_113077.1 | 1480 | 346 | 766 | 28.45 | 473.9 | 3E-133 | 368/423 | 86 | 4/423 | 0 | Plus | Plus | Bacteria; Bacteroidetes; Bacteroidia; Bacteroidales; Prevotellaceae; Paraprevotella; Paraprevotella clara | Paraprevotella clara | Paraprevotella | Prevotellaceae | Bacteroidales | Bacteroidia | Bacteroidetes | Bacteria |
| ASV333 | 402 | 1 | 1 | 402 | 100 | NR_118554.1 Intestinimonas butyriciproducens strain SRB-521-5-I 16S ribosomal RNA, partial sequence | NR_118554.1 | 1475 | 346 | 750 | 27.46 | 496 | 6E-140 | 360/405 | 88 | 3/405 | 0 | Plus | Plus | Bacteria; Firmicutes; Clostridia; Eubacteriales; ; Intestinimonas; Intestinimonas butyriciproducens | Intestinimonas butyriciproducens | Intestinimonas | | Eubacteriales | Clostridia | Firmicutes | Bacteria |
| ASV334 | 422 | 1 | 1 | 422 | 100 | NR_113411.1 Prevotella copri DSM 18205 strain JCM 13464 16S ribosomal RNA, partial sequence | NR_113411.1 | 1493 | 358 | 779 | 28.27 | 608.7 | 7E-174 | 394/425 | 92 | 6/425 | 1 | Plus | Plus | Bacteria; Bacteroidetes; Bacteroidia; Bacteroidales; Prevotellaceae; Prevotella; Prevotella copri | Prevotella copri | Prevotella | Prevotellaceae | Bacteroidales | Bacteroidia | Bacteroidetes | Bacteria |
| ASV335 | 403 | 1 | 1 | 403 | 100 | NR_029160.1 Ruminococcus callidus strain ATCC 27760 16S ribosomal RNA, partial sequence | NR_029160.1 | 1371 | 310 | 711 | 29.32 | 616.1 | 4E-176 | 382/405 | 94 | 5/405 | 1 | Plus | Plus | Bacteria; Firmicutes; Clostridia; Eubacteriales; Oscillospiraceae; Ruminococcus; Ruminococcus callidus | Ruminococcus callidus | Ruminococcus | Oscillospiraceae | Eubacteriales | Clostridia | Firmicutes | Bacteria |
| ASV336 | 422 | 2 | 1 | 422 | 100 | NR_113117.1 Prevotella oralis ATCC 33269 strain JCM 12251 16S ribosomal RNA, partial sequence | NR_113117.1 | 1496 | 361 | 782 | 28.21 | 597.6 | 2E-170 | 390/423 | 92 | 2/423 | 0 | Plus | Plus | Bacteria; Bacteroidetes; Bacteroidia; Bacteroidales; Prevotellaceae; Prevotella; Prevotella oralis | Prevotella oralis | Prevotella | Prevotellaceae | Bacteroidales | Bacteroidia | Bacteroidetes | Bacteria |
| ASV337 | 402 | 1 | 1 | 402 | 100 | NR_029097.1 Oribacterium sinus strain AIP 354.02 16S ribosomal RNA, partial sequence | NR_029097.1 | 1374 | 308 | 709 | 29.26 | 654.8 | 0 | 386/402 | 96 | 0/402 | 0 | Plus | Plus | Bacteria; Firmicutes; Clostridia; Eubacteriales; Lachnospiraceae; Oribacterium; Oribacterium sinus | Oribacterium sinus | Oribacterium | Lachnospiraceae | Eubacteriales | Clostridia | Firmicutes | Bacteria |
| ASV338 | 403 | 1 | 3 | 403 | 99.5 | NR_125464.1 Anaerobacterium chartisolvens strain T-1-35 16S ribosomal RNA, partial sequence | NR_125464.1 | 1451 | 344 | 745 | 27.71 | 464.6 | 2E-130 | 352/402 | 87 | 1/402 | 0 | Plus | Plus | Bacteria; Firmicutes; Clostridia; Eubacteriales; Oscillospiraceae; Anaerobacterium; Anaerobacterium chartisolvens | Anaerobacterium chartisolvens | Anaerobacterium | Oscillospiraceae | Eubacteriales | Clostridia | Firmicutes | Bacteria |
| ASV339 | 402 | 1 | 1 | 402 | 100 | NR_074613.1 [Eubacterium] eligens ATCC 27750 16S ribosomal RNA, partial sequence | NR_074613.1 | 1519 | 367 | 768 | 26.46 | 726.9 | 0 | 399/402 | 99 | 0/402 | 0 | Plus | Plus | Bacteria; Firmicutes; Clostridia; Eubacteriales; Lachnospiraceae; Lachnospira; Lachnospira eligens | Lachnospira eligens | Lachnospira | Lachnospiraceae | Eubacteriales | Clostridia | Firmicutes | Bacteria |
| ASV340 | 421 | 1 | 1 | 421 | 100 | NR_144616.1 Muribaculum intestinale strain YL27 16S ribosomal RNA, partial sequence | NR_144616.1 | 1460 | 297 | 718 | 28.9 | 529.3 | 6E-150 | 379/424 | 89 | 5/424 | 1 | Plus | Plus | Bacteria; Bacteroidetes; Bacteroidia; Bacteroidales; Muribaculaceae; Muribaculum; Muribaculum intestinale | Muribaculum intestinale | Muribaculum | Muribaculaceae | Bacteroidales | Bacteroidia | Bacteroidetes | Bacteria |
| ASV342 | 427 | 1 | 1 | 427 | 100 | NR_112902.1 Phascolarctobacterium succinatutens strain YIT 12067 16S ribosomal RNA, partial sequence | NR_112902.1 | 1610 | 444 | 870 | 26.52 | 756.4 | 0 | 421/427 | 98 | 0/427 | 0 | Plus | Plus | Bacteria; Firmicutes; Negativicutes; Acidaminococcales; Acidaminococcaceae; Phascolarctobacterium; Phascolarctobacterium succinatutens | Phascolarctobacterium succinatutens | Phascolarctobacterium | Acidaminococcaceae | Acidaminococcales | Negativicutes | Firmicutes | Bacteria |
| ASV343 | 427 | 1 | 1 | 427 | 100 | NR_042942.1 Treponema porcinum strain 14V28 16S ribosomal RNA, partial sequence | NR_042942.1 | 1426 | 258 | 684 | 29.94 | 540.3 | 3E-153 | 384/429 | 89 | 4/429 | 0 | Plus | Plus | Bacteria; Spirochaetes; Spirochaetia; Spirochaetales; Spirochaetaceae; Treponema; Treponema porcinum | Treponema porcinum | Treponema | Spirochaetaceae | Spirochaetales | Spirochaetia | Spirochaetes | Bacteria |
| ASV344 | 423 | 1 | 1 | 423 | 100 | NR_144750.1 Prevotellamassilia timonensis strain Marseille-P2831 16S ribosomal RNA, partial sequence | NR_144750.1 | 1493 | 358 | 779 | 28.27 | 547.7 | 2E-155 | 384/426 | 90 | 7/426 | 1 | Plus | Plus | Bacteria; Bacteroidetes; Bacteroidia; Bacteroidales; Prevotellaceae; Prevotellamassilia; Prevotellamassilia timonensis | Prevotellamassilia timonensis | Prevotellamassilia | Prevotellaceae | Bacteroidales | Bacteroidia | Bacteroidetes | Bacteria |
| ASV345 | 404 | 1 | 1 | 404 | 100 | NR_026315.1 Pseudobutyrivibrio ruminis strain DSM 9787 16S ribosomal RNA, partial sequence | NR_026315.1 | 1462 | 334 | 735 | 27.5 | 673.3 | 0 | 391/404 | 96 | 2/404 | 0 | Plus | Plus | Bacteria; Firmicutes; Clostridia; Eubacteriales; Lachnospiraceae; Pseudobutyrivibrio; Pseudobutyrivibrio ruminis | Pseudobutyrivibrio ruminis | Pseudobutyrivibrio | Lachnospiraceae | Eubacteriales | Clostridia | Firmicutes | Bacteria |
| ASV346 | 402 | 1 | 1 | 402 | 100 | NR_074634.1 [Eubacterium] rectale ATCC 33656 16S ribosomal RNA, partial sequence | NR_074634.1 | 1521 | 367 | 768 | 26.43 | 737.9 | 0 | 401/402 | 99 | 0/402 | 0 | Plus | Plus | Bacteria; Firmicutes; Clostridia; Eubacteriales; Lachnospiraceae; ; [Eubacterium] rectale | [Eubacterium] rectale | | Lachnospiraceae | Eubacteriales | Clostridia | Firmicutes | Bacteria |
| ASV347 | 403 | 1 | 1 | 403 | 100 | NR_029160.1 Ruminococcus callidus strain ATCC 27760 16S ribosomal RNA, partial sequence | NR_029160.1 | 1371 | 310 | 711 | 29.32 | 632.7 | 0 | 385/405 | 95 | 5/405 | 1 | Plus | Plus | Bacteria; Firmicutes; Clostridia; Eubacteriales; Oscillospiraceae; Ruminococcus; Ruminococcus callidus | Ruminococcus callidus | Ruminococcus | Oscillospiraceae | Eubacteriales | Clostridia | Firmicutes | Bacteria |
| ASV348 | 427 | 1 | 1 | 427 | 100 | NR_044111.1 Selenomonas bovis strain WG 16S ribosomal RNA, partial sequence | NR_044111.1 | 1525 | 370 | 796 | 28 | 773 | 0 | 424/427 | 99 | 0/427 | 0 | Plus | Plus | Bacteria; Firmicutes; Negativicutes; Selenomonadales; Selenomonadaceae; Selenomonas; Selenomonas bovis | Selenomonas bovis | Selenomonas | Selenomonadaceae | Selenomonadales | Negativicutes | Firmicutes | Bacteria |
| ASV349 | 427 | 1 | 1 | 427 | 100 | NR_028796.1 Selenomonas noxia strain ATCC 43541 16S ribosomal RNA, partial sequence | NR_028796.1 | 1491 | 375 | 801 | 28.64 | 579.1 | 6E-165 | 391/429 | 91 | 4/429 | 0 | Plus | Plus | Bacteria; Firmicutes; Negativicutes; Selenomonadales; Selenomonadaceae; Selenomonas; Selenomonas noxia | Selenomonas noxia | Selenomonas | Selenomonadaceae | Selenomonadales | Negativicutes | Firmicutes | Bacteria |
| ASV350 | 427 | 4 | 1 | 427 | 100 | NR_042802.1 Lactobacillus ultunensis strain Kx146C1 16S ribosomal RNA, partial sequence | NR_042802.1 | 1550 | 370 | 796 | 27.55 | 784.1 | 0 | 426/427 | 99 | 0/427 | 0 | Plus | Plus | Bacteria; Firmicutes; Bacilli; Lactobacillales; Lactobacillaceae; Lactobacillus; Lactobacillus ultunensis | Lactobacillus ultunensis | Lactobacillus | Lactobacillaceae | Lactobacillales | Bacilli | Firmicutes | Bacteria |
| ASV351 | 402 | 1 | 1 | 402 | 100 | NR_044841.2 Clostridium aurantibutyricum strain NCIMB 10659 16S ribosomal RNA, partial sequence | NR_044841.2 | 1423 | 348 | 748 | 28.18 | 719.5 | 0 | 398/402 | 99 | 1/402 | 0 | Plus | Plus | Bacteria; Firmicutes; Clostridia; Eubacteriales; Clostridiaceae; Clostridium; Clostridium aurantibutyricum | Clostridium aurantibutyricum | Clostridium | Clostridiaceae | Eubacteriales | Clostridia | Firmicutes | Bacteria |
| ASV352 | 426 | 1 | 1 | 426 | 100 | NR_025594.1 Erysipelothrix inopinata strain 143-02 16S ribosomal RNA, partial sequence | NR_025594.1 | 1504 | 346 | 771 | 28.32 | 499.7 | 5E-141 | 378/430 | 87 | 8/430 | 1 | Plus | Plus | Bacteria; Firmicutes; Erysipelotrichia; Erysipelotrichales; Erysipelotrichaceae; Erysipelothrix; Erysipelothrix inopinata | Erysipelothrix inopinata | Erysipelothrix | Erysipelotrichaceae | Erysipelotrichales | Erysipelotrichia | Firmicutes | Bacteria |
| ASV353 | 408 | 1 | 1 | 408 | 100 | NR_029167.1 Anaeroplasma abactoclasticum strain 6-1 16S ribosomal RNA, partial sequence | NR_029167.1 | 1446 | 344 | 751 | 28.22 | 573.6 | 3E-163 | 378/411 | 91 | 6/411 | 1 | Plus | Plus | Bacteria; Tenericutes; Mollicutes; Anaeroplasmatales; Anaeroplasmataceae; Anaeroplasma; Anaeroplasma abactoclasticum | Anaeroplasma abactoclasticum | Anaeroplasma | Anaeroplasmataceae | Anaeroplasmatales | Mollicutes | Tenericutes | Bacteria |
| ASV354 | 427 | 1 | 1 | 427 | 100 | NR_041666.1 Dialister succinatiphilus YIT 11850 16S ribosomal RNA, partial sequence | NR_041666.1 | 1521 | 353 | 779 | 28.07 | 712.1 | 0 | 414/428 | 96 | 2/428 | 0 | Plus | Plus | Bacteria; Firmicutes; Negativicutes; Veillonellales; Veillonellaceae; Dialister; Dialister succinatiphilus | Dialister succinatiphilus | Dialister | Veillonellaceae | Veillonellales | Negativicutes | Firmicutes | Bacteria |
| ASV355 | 402 | 1 | 1 | 402 | 100 | NR_044645.2 Dorea formicigenerans strain ATCC 27755 16S ribosomal RNA, partial sequence | NR_044645.2 | 1479 | 372 | 773 | 27.18 | 728.7 | 0 | 398/402 | 99 | 0/402 | 0 | Plus | Plus | Bacteria; Firmicutes; Clostridia; Eubacteriales; Lachnospiraceae; Dorea; Dorea formicigenerans | Dorea formicigenerans | Dorea | Lachnospiraceae | Eubacteriales | Clostridia | Firmicutes | Bacteria |
| ASV356 | 402 | 1 | 1 | 402 | 100 | NR_118667.1 Lachnospira pectinoschiza strain 150-1 16S ribosomal RNA, partial sequence | NR_118667.1 | 1465 | 372 | 773 | 27.44 | 712.1 | 0 | 394/402 | 98 | 0/402 | 0 | Plus | Plus | Bacteria; Firmicutes; Clostridia; Eubacteriales; Lachnospiraceae; Lachnospira; Lachnospira pectinoschiza | Lachnospira pectinoschiza | Lachnospira | Lachnospiraceae | Eubacteriales | Clostridia | Firmicutes | Bacteria |
| ASV357 | 422 | 2 | 1 | 422 | 100 | NR_113119.1 Prevotella oris strain JCM 8540 16S ribosomal RNA, partial sequence | NR_113119.1 | 1481 | 345 | 766 | 28.49 | 581 | 2E-165 | 387/423 | 91 | 2/423 | 0 | Plus | Plus | Bacteria; Bacteroidetes; Bacteroidia; Bacteroidales; Prevotellaceae; Prevotella; Prevotella oris | Prevotella oris | Prevotella | Prevotellaceae | Bacteroidales | Bacteroidia | Bacteroidetes | Bacteria |
| ASV358 | 422 | 2 | 1 | 422 | 100 | NR_113117.1 Prevotella oralis ATCC 33269 strain JCM 12251 16S ribosomal RNA, partial sequence | NR_113117.1 | 1496 | 361 | 782 | 28.21 | 625.3 | 7E-179 | 394/422 | 93 | 0/422 | 0 | Plus | Plus | Bacteria; Bacteroidetes; Bacteroidia; Bacteroidales; Prevotellaceae; Prevotella; Prevotella oralis | Prevotella oralis | Prevotella | Prevotellaceae | Bacteroidales | Bacteroidia | Bacteroidetes | Bacteria |
| ASV359 | 422 | 3 | 1 | 422 | 100 | NR_113105.1 Prevotella histicola strain JCM 15637 16S ribosomal RNA, partial sequence | NR_113105.1 | 1480 | 346 | 767 | 28.51 | 592 | 7E-169 | 391/425 | 92 | 6/425 | 1 | Plus | Plus | Bacteria; Bacteroidetes; Bacteroidia; Bacteroidales; Prevotellaceae; Prevotella; Prevotella histicola | Prevotella histicola | Prevotella | Prevotellaceae | Bacteroidales | Bacteroidia | Bacteroidetes | Bacteria |
| ASV360 | 405 | 1 | 1 | 405 | 100 | NR_144611.1 Flintibacter butyricus strain BLS21 16S ribosomal RNA, partial sequence | NR_144611.1 | 808 | 299 | 703 | 50.12 | 577.3 | 2E-164 | 374/405 | 92 | 0/405 | 0 | Plus | Plus | Bacteria; Firmicutes; Clostridia; Eubacteriales; ; Flintibacter; Flintibacter butyricus | Flintibacter butyricus | Flintibacter | | Eubacteriales | Clostridia | Firmicutes | Bacteria |
| ASV361 | 421 | 2 | 1 | 421 | 100 | NR_074577.1 Paludibacter propionicigenes WB4 16S ribosomal RNA, partial sequence | NR_074577.1 | 1516 | 353 | 774 | 27.84 | 496 | 6E-140 | 372/423 | 87 | 3/423 | 0 | Plus | Plus | Bacteria; Bacteroidetes; Bacteroidia; Bacteroidales; Paludibacteraceae; Paludibacter; Paludibacter propionicigenes | Paludibacter propionicigenes | Paludibacter | Paludibacteraceae | Bacteroidales | Bacteroidia | Bacteroidetes | Bacteria |
| ASV363 | 421 | 1 | 1 | 421 | 100 | NR_144616.1 Muribaculum intestinale strain YL27 16S ribosomal RNA, partial sequence | NR_144616.1 | 1460 | 297 | 718 | 28.9 | 507.1 | 3E-143 | 373/422 | 88 | 1/422 | 0 | Plus | Plus | Bacteria; Bacteroidetes; Bacteroidia; Bacteroidales; Muribaculaceae; Muribaculum; Muribaculum intestinale | Muribaculum intestinale | Muribaculum | Muribaculaceae | Bacteroidales | Bacteroidia | Bacteroidetes | Bacteria |
| ASV364 | 427 | 2 | 1 | 427 | 100 | NR_037018.1 Acidaminococcus fermentans strain VR4 16S ribosomal RNA gene, partial sequence | NR_037018.1 | 1516 | 349 | 775 | 28.17 | 734.2 | 0 | 417/427 | 97 | 0/427 | 0 | Plus | Plus | Bacteria; Firmicutes; Negativicutes; Acidaminococcales; Acidaminococcaceae; Acidaminococcus; Acidaminococcus fermentans | Acidaminococcus fermentans | Acidaminococcus | Acidaminococcaceae | Acidaminococcales | Negativicutes | Firmicutes | Bacteria |
| ASV365 | 422 | 1 | 1 | 422 | 100 | NR_113411.1 Prevotella copri DSM 18205 strain JCM 13464 16S ribosomal RNA, partial sequence | NR_113411.1 | 1493 | 358 | 779 | 28.27 | 730.5 | 0 | 415/424 | 97 | 4/424 | 0 | Plus | Plus | Bacteria; Bacteroidetes; Bacteroidia; Bacteroidales; Prevotellaceae; Prevotella; Prevotella copri | Prevotella copri | Prevotella | Prevotellaceae | Bacteroidales | Bacteroidia | Bacteroidetes | Bacteria |
| ASV366 | 422 | 1 | 1 | 422 | 100 | NR_041342.1 Parabacteroides distasonis strain JCM 5825 16S ribosomal RNA, partial sequence | NR_041342.1 | 1488 | 353 | 774 | 28.36 | 542.2 | 8E-154 | 380/423 | 89 | 2/423 | 0 | Plus | Plus | Bacteria; Bacteroidetes; Bacteroidia; Bacteroidales; Tannerellaceae; Parabacteroides; Parabacteroides distasonis | Parabacteroides distasonis | Parabacteroides | Tannerellaceae | Bacteroidales | Bacteroidia | Bacteroidetes | Bacteria |
| ASV367 | 403 | 1 | 1 | 403 | 100 | NR_104907.1 Eubacterium coprostanoligenes strain HL 16S ribosomal RNA, partial sequence | NR_104907.1 | 1446 | 332 | 734 | 27.87 | 745.3 | 0 | 403/403 | 100 | 0/403 | 0 | Plus | Plus | Bacteria; Firmicutes; Clostridia; Eubacteriales; Eubacteriaceae; Eubacterium; Eubacterium coprostanoligenes | Eubacterium coprostanoligenes | Eubacterium | Eubacteriaceae | Eubacteriales | Clostridia | Firmicutes | Bacteria |
| ASV368 | 422 | 2 | 1 | 422 | 100 | NR_113119.1 Prevotella oris strain JCM 8540 16S ribosomal RNA, partial sequence | NR_113119.1 | 1481 | 345 | 766 | 28.49 | 625.3 | 7E-179 | 395/423 | 93 | 2/423 | 0 | Plus | Plus | Bacteria; Bacteroidetes; Bacteroidia; Bacteroidales; Prevotellaceae; Prevotella; Prevotella oris | Prevotella oris | Prevotella | Prevotellaceae | Bacteroidales | Bacteroidia | Bacteroidetes | Bacteria |
| ASV369 | 422 | 1 | 1 | 422 | 100 | NR_113411.1 Prevotella copri DSM 18205 strain JCM 13464 16S ribosomal RNA, partial sequence | NR_113411.1 | 1493 | 358 | 779 | 28.27 | 758.2 | 0 | 418/422 | 99 | 0/422 | 0 | Plus | Plus | Bacteria; Bacteroidetes; Bacteroidia; Bacteroidales; Prevotellaceae; Prevotella; Prevotella copri | Prevotella copri | Prevotella | Prevotellaceae | Bacteroidales | Bacteroidia | Bacteroidetes | Bacteria |
| ASV370 | 427 | 3 | 1 | 427 | 100 | NR_029106.1 Lactobacillus delbrueckii subsp. indicus strain NCC725 16S ribosomal RNA, partial sequence | NR_029106.1 | 1515 | 348 | 774 | 28.18 | 728.7 | 0 | 416/427 | 97 | 0/427 | 0 | Plus | Plus | Bacteria; Firmicutes; Bacilli; Lactobacillales; Lactobacillaceae; Lactobacillus; Lactobacillus delbrueckii | Lactobacillus delbrueckii | Lactobacillus | Lactobacillaceae | Lactobacillales | Bacilli | Firmicutes | Bacteria |
| ASV371 | 405 | 1 | 1 | 405 | 100 | NR_144611.1 Flintibacter butyricus strain BLS21 16S ribosomal RNA, partial sequence | NR_144611.1 | 808 | 299 | 703 | 50.12 | 710.2 | 0 | 398/405 | 98 | 0/405 | 0 | Plus | Plus | Bacteria; Firmicutes; Clostridia; Eubacteriales; ; Flintibacter; Flintibacter butyricus | Flintibacter butyricus | Flintibacter | | Eubacteriales | Clostridia | Firmicutes | Bacteria |
| ASV372 | 427 | 1 | 1 | 427 | 100 | NR_041666.1 Dialister succinatiphilus YIT 11850 16S ribosomal RNA, partial sequence | NR_041666.1 | 1521 | 353 | 779 | 28.07 | 712.1 | 0 | 414/428 | 96 | 2/428 | 0 | Plus | Plus | Bacteria; Firmicutes; Negativicutes; Veillonellales; Veillonellaceae; Dialister; Dialister succinatiphilus | Dialister succinatiphilus | Dialister | Veillonellaceae | Veillonellales | Negativicutes | Firmicutes | Bacteria |
| ASV373 | 405 | 1 | 1 | 405 | 100 | NR_118554.1 Intestinimonas butyriciproducens strain SRB-521-5-I 16S ribosomal RNA, partial sequence | NR_118554.1 | 1475 | 346 | 750 | 27.46 | 605 | 9E-173 | 379/405 | 93 | 0/405 | 0 | Plus | Plus | Bacteria; Firmicutes; Clostridia; Eubacteriales; ; Intestinimonas; Intestinimonas butyriciproducens | Intestinimonas butyriciproducens | Intestinimonas | | Eubacteriales | Clostridia | Firmicutes | Bacteria |
| ASV374 | 402 | 1 | 1 | 402 | 100 | NR_026146.1 Clostridium ventriculi strain DSM 286 16S ribosomal RNA, partial sequence | NR_026146.1 | 1461 | 328 | 728 | 27.45 | 736.1 | 0 | 401/402 | 99 | 1/402 | 0 | Plus | Plus | Bacteria; Firmicutes; Clostridia; Eubacteriales; Clostridiaceae; Clostridium; Clostridium ventriculi | Clostridium ventriculi | Clostridium | Clostridiaceae | Eubacteriales | Clostridia | Firmicutes | Bacteria |
| ASV375 | 403 | 1 | 1 | 403 | 100 | NR_025930.1 Ruminococcus bromii strain ATCC 27255 16S ribosomal RNA, partial sequence | NR_025930.1 | 1380 | 313 | 716 | 29.28 | 614.2 | 2E-175 | 380/404 | 94 | 1/404 | 0 | Plus | Plus | Bacteria; Firmicutes; Clostridia; Eubacteriales; Oscillospiraceae; Ruminococcus; Ruminococcus bromii | Ruminococcus bromii | Ruminococcus | Oscillospiraceae | Eubacteriales | Clostridia | Firmicutes | Bacteria |
| ASV376 | 422 | 1 | 3 | 422 | 99.53 | NR_133789.1 Mucilaginibacter defluvii strain A5 16S ribosomal RNA, partial sequence | NR_133789.1 | 1446 | 335 | 754 | 29.05 | 438.8 | 1E-122 | 361/422 | 85 | 4/422 | 0 | Plus | Plus | Bacteria; Bacteroidetes; Sphingobacteriia; Sphingobacteriales; Sphingobacteriaceae; Mucilaginibacter; Mucilaginibacter defluvii | Mucilaginibacter defluvii | Mucilaginibacter | Sphingobacteriaceae | Sphingobacteriales | Sphingobacteriia | Bacteroidetes | Bacteria |
| ASV377 | 420 | 1 | 1 | 420 | 100 | NR_170509.1 Duncaniella freteri strain TLL-A3 16S ribosomal RNA, partial sequence | NR_170509.1 | 1538 | 364 | 784 | 27.37 | 449.9 | 5E-126 | 366/425 | 86 | 9/425 | 2 | Plus | Plus | Bacteria; Bacteroidetes; Bacteroidia; Bacteroidales; Muribaculaceae; Duncaniella; Duncaniella freteri | Duncaniella freteri | Duncaniella | Muribaculaceae | Bacteroidales | Bacteroidia | Bacteroidetes | Bacteria |
| ASV378 | 422 | 1 | 1 | 422 | 100 | NR_041364.1 Prevotella stercorea DSM 18206 strain CB35 16S ribosomal RNA, partial sequence | NR_041364.1 | 1488 | 354 | 775 | 28.36 | 741.6 | 0 | 415/422 | 98 | 0/422 | 0 | Plus | Plus | Bacteria; Bacteroidetes; Bacteroidia; Bacteroidales; Prevotellaceae; Prevotella; Prevotella stercorea | Prevotella stercorea | Prevotella | Prevotellaceae | Bacteroidales | Bacteroidia | Bacteroidetes | Bacteria |
| ASV379 | 427 | 1 | 1 | 427 | 100 | NR_112652.1 Lactobacillus equicursoris DSM 19284 = JCM 14600 = CIP 110162 strain DI70 16S ribosomal RNA, partial sequence | NR_112652.1 | 1486 | 351 | 777 | 28.73 | 784.1 | 0 | 426/427 | 99 | 0/427 | 0 | Plus | Plus | Bacteria; Firmicutes; Bacilli; Lactobacillales; Lactobacillaceae; Lactobacillus; Lactobacillus equicursoris | Lactobacillus equicursoris | Lactobacillus | Lactobacillaceae | Lactobacillales | Bacilli | Firmicutes | Bacteria |
| ASV380 | 427 | 1 | 1 | 427 | 100 | NR_074742.1 Treponema brennaborense strain DD5/3 16S ribosomal RNA, partial sequence | NR_074742.1 | 1530 | 351 | 776 | 27.84 | 582.8 | 5E-166 | 390/427 | 91 | 1/427 | 0 | Plus | Plus | Bacteria; Spirochaetes; Spirochaetia; Spirochaetales; Spirochaetaceae; Treponema; Treponema brennaborense | Treponema brennaborense | Treponema | Spirochaetaceae | Spirochaetales | Spirochaetia | Spirochaetes | Bacteria |
| ASV382 | 402 | 1 | 1 | 402 | 100 | NR_118674.1 Eubacterium ramulus ATCC 29099 16S ribosomal RNA, partial sequence | NR_118674.1 | 1474 | 373 | 774 | 27.27 | 588.4 | 9E-168 | 371/402 | 92 | 0/402 | 0 | Plus | Plus | Bacteria; Firmicutes; Clostridia; Eubacteriales; Eubacteriaceae; Eubacterium; Eubacterium ramulus | Eubacterium ramulus | Eubacterium | Eubacteriaceae | Eubacteriales | Clostridia | Firmicutes | Bacteria |
| ASV383 | 405 | 1 | 1 | 405 | 100 | NR_044972.1 Sporobacter termitidis strain SYR 16S ribosomal RNA, partial sequence | NR_044972.1 | 1506 | 348 | 752 | 26.89 | 581 | 2E-165 | 375/406 | 92 | 2/406 | 0 | Plus | Plus | Bacteria; Firmicutes; Clostridia; Eubacteriales; Oscillospiraceae; Sporobacter; Sporobacter termitidis | Sporobacter termitidis | Sporobacter | Oscillospiraceae | Eubacteriales | Clostridia | Firmicutes | Bacteria |
| ASV384 | 402 | 1 | 1 | 402 | 100 | NR_144736.1 Neglecta timonensis strain SN17 16S ribosomal RNA, partial sequence | NR_144736.1 | 1503 | 377 | 778 | 26.75 | 682.5 | 0 | 392/403 | 97 | 2/403 | 0 | Plus | Plus | Bacteria; Firmicutes; Clostridia; Eubacteriales; ; Neglecta; Neglecta timonensis | Neglecta timonensis | Neglecta | | Eubacteriales | Clostridia | Firmicutes | Bacteria |
| ASV385 | 402 | 1 | 1 | 402 | 100 | NR_144727.1 Anaeromassilibacillus senegalensis strain mt9 16S ribosomal RNA, partial sequence | NR_144727.1 | 1478 | 352 | 753 | 27.2 | 632.7 | 0 | 384/404 | 95 | 4/404 | 0 | Plus | Plus | Bacteria; Firmicutes; Clostridia; Eubacteriales; Oscillospiraceae; Anaeromassilibacillus; Anaeromassilibacillus senegalensis | Anaeromassilibacillus senegalensis | Anaeromassilibacillus | Oscillospiraceae | Eubacteriales | Clostridia | Firmicutes | Bacteria |
| ASV386 | 402 | 1 | 1 | 402 | 100 | NR_170495.1 Blautia faecicola strain KGMB01111 16S ribosomal RNA, complete sequence | NR_170495.1 | 1534 | 372 | 773 | 26.21 | 577.3 | 2E-164 | 372/402 | 92 | 0/402 | 0 | Plus | Plus | Bacteria; Firmicutes; Clostridia; Eubacteriales; Lachnospiraceae; Blautia; Blautia faecicola | Blautia faecicola | Blautia | Lachnospiraceae | Eubacteriales | Clostridia | Firmicutes | Bacteria |
| ASV387 | 426 | 1 | 1 | 426 | 100 | NR_026205.1 Succiniclasticum ruminis strain SE10 16S ribosomal RNA, partial sequence | NR_026205.1 | 1505 | 354 | 780 | 28.37 | 610.5 | 2E-174 | 396/428 | 92 | 3/428 | 0 | Plus | Plus | Bacteria; Firmicutes; Negativicutes; Acidaminococcales; Acidaminococcaceae; Succiniclasticum; Succiniclasticum ruminis | Succiniclasticum ruminis | Succiniclasticum | Acidaminococcaceae | Acidaminococcales | Negativicutes | Firmicutes | Bacteria |
| ASV388 | 401 | 1 | 1 | 401 | 100 | NR_144737.1 Emergencia timonensis strain SN18 16S ribosomal RNA, partial sequence | NR_144737.1 | 1480 | 364 | 767 | 27.3 | 627.1 | 2E-179 | 383/404 | 94 | 3/404 | 0 | Plus | Plus | Bacteria; Firmicutes; Clostridia; Eubacteriales; Clostridiales Family XIII. Incertae Sedis; Emergencia; Emergencia timonensis | Emergencia timonensis | Emergencia | Clostridiales Family XIII. Incertae Sedis | Eubacteriales | Clostridia | Firmicutes | Bacteria |
| ASV390 | 403 | 1 | 3 | 403 | 99.5 | NR_125677.1 Vallitalea pronyensis strain FatNI3 16S ribosomal RNA, partial sequence | NR_125677.1 | 1480 | 365 | 764 | 27.03 | 451.7 | 1E-126 | 350/402 | 87 | 3/402 | 0 | Plus | Plus | Bacteria; Firmicutes; Clostridia; Eubacteriales; Vallitaleaceae; Vallitalea; Vallitalea pronyensis | Vallitalea pronyensis | Vallitalea | Vallitaleaceae | Eubacteriales | Clostridia | Firmicutes | Bacteria |
| ASV391 | 427 | 1 | 1 | 427 | 100 | NR_036788.2 Lactobacillus pontis strain LTH 2587 16S ribosomal RNA, partial sequence | NR_036788.2 | 1568 | 384 | 810 | 27.23 | 767.5 | 0 | 423/427 | 99 | 0/427 | 0 | Plus | Plus | Bacteria; Firmicutes; Bacilli; Lactobacillales; Lactobacillaceae; Limosilactobacillus; Limosilactobacillus pontis | Limosilactobacillus pontis | Limosilactobacillus | Lactobacillaceae | Lactobacillales | Bacilli | Firmicutes | Bacteria |
| ASV392 | 402 | 1 | 1 | 402 | 100 | NR_109014.1 Blautia faecis strain M25 16S ribosomal RNA, partial sequence | NR_109014.1 | 1262 | 212 | 613 | 31.85 | 699.2 | 0 | 394/402 | 98 | 0/402 | 0 | Plus | Plus | Bacteria; Firmicutes; Clostridia; Eubacteriales; Lachnospiraceae; Blautia; Blautia faecis | Blautia faecis | Blautia | Lachnospiraceae | Eubacteriales | Clostridia | Firmicutes | Bacteria |
| ASV393 | 403 | 1 | 3 | 403 | 99.5 | NR_125464.1 Anaerobacterium chartisolvens strain T-1-35 16S ribosomal RNA, partial sequence | NR_125464.1 | 1451 | 344 | 745 | 27.71 | 492.3 | 7E-139 | 357/402 | 88 | 1/402 | 0 | Plus | Plus | Bacteria; Firmicutes; Clostridia; Eubacteriales; Oscillospiraceae; Anaerobacterium; Anaerobacterium chartisolvens | Anaerobacterium chartisolvens | Anaerobacterium | Oscillospiraceae | Eubacteriales | Clostridia | Firmicutes | Bacteria |
| ASV394 | 422 | 1 | 1 | 422 | 100 | NR_043216.1 Prevotella loescheii strain NCTC 11321 16S ribosomal RNA, partial sequence | NR_043216.1 | 1451 | 336 | 757 | 29.08 | 605 | 1E-172 | 393/425 | 92 | 6/425 | 1 | Plus | Plus | Bacteria; Bacteroidetes; Bacteroidia; Bacteroidales; Prevotellaceae; Prevotella; Prevotella loescheii | Prevotella loescheii | Prevotella | Prevotellaceae | Bacteroidales | Bacteroidia | Bacteroidetes | Bacteria |
| ASV395 | 402 | 1 | 1 | 402 | 100 | NR_028883.1 Dorea longicatena strain 111-35 16S ribosomal RNA, partial sequence | NR_028883.1 | 1316 | 243 | 645 | 30.62 | 737.9 | 0 | 402/403 | 99 | 1/403 | 0 | Plus | Plus | Bacteria; Firmicutes; Clostridia; Eubacteriales; Lachnospiraceae; Dorea; Dorea longicatena | Dorea longicatena | Dorea | Lachnospiraceae | Eubacteriales | Clostridia | Firmicutes | Bacteria |
| ASV396 | 427 | 1 | 1 | 427 | 100 | NR_028796.1 Selenomonas noxia strain ATCC 43541 16S ribosomal RNA, partial sequence | NR_028796.1 | 1491 | 375 | 801 | 28.64 | 579.1 | 6E-165 | 391/429 | 91 | 4/429 | 0 | Plus | Plus | Bacteria; Firmicutes; Negativicutes; Selenomonadales; Selenomonadaceae; Selenomonas; Selenomonas noxia | Selenomonas noxia | Selenomonas | Selenomonadaceae | Selenomonadales | Negativicutes | Firmicutes | Bacteria |
| ASV397 | 404 | 1 | 1 | 404 | 100 | NR_025930.1 Ruminococcus bromii strain ATCC 27255 16S ribosomal RNA, partial sequence | NR_025930.1 | 1380 | 313 | 716 | 29.28 | 653 | 0 | 388/405 | 95 | 2/405 | 0 | Plus | Plus | Bacteria; Firmicutes; Clostridia; Eubacteriales; Oscillospiraceae; Ruminococcus; Ruminococcus bromii | Ruminococcus bromii | Ruminococcus | Oscillospiraceae | Eubacteriales | Clostridia | Firmicutes | Bacteria |
| ASV398 | 422 | 2 | 1 | 422 | 100 | NR_113117.1 Prevotella oralis ATCC 33269 strain JCM 12251 16S ribosomal RNA, partial sequence | NR_113117.1 | 1496 | 361 | 782 | 28.21 | 597.6 | 2E-170 | 390/423 | 92 | 2/423 | 0 | Plus | Plus | Bacteria; Bacteroidetes; Bacteroidia; Bacteroidales; Prevotellaceae; Prevotella; Prevotella oralis | Prevotella oralis | Prevotella | Prevotellaceae | Bacteroidales | Bacteroidia | Bacteroidetes | Bacteria |
| ASV399 | 402 | 1 | 1 | 402 | 100 | NR_147375.1 Colidextribacter massiliensis strain Marseille-P3083 16S ribosomal RNA, partial sequence | NR_147375.1 | 1487 | 364 | 765 | 27.03 | 632.7 | 0 | 382/402 | 95 | 0/402 | 0 | Plus | Plus | Bacteria; Firmicutes; Clostridia; Eubacteriales; ; Colidextribacter; Colidextribacter massiliensis | Colidextribacter massiliensis | Colidextribacter | | Eubacteriales | Clostridia | Firmicutes | Bacteria |
| ASV400 | 422 | 1 | 1 | 422 | 100 | NR_041364.1 Prevotella stercorea DSM 18206 strain CB35 16S ribosomal RNA, partial sequence | NR_041364.1 | 1488 | 354 | 775 | 28.36 | 719.5 | 0 | 411/422 | 97 | 0/422 | 0 | Plus | Plus | Bacteria; Bacteroidetes; Bacteroidia; Bacteroidales; Prevotellaceae; Prevotella; Prevotella stercorea | Prevotella stercorea | Prevotella | Prevotellaceae | Bacteroidales | Bacteroidia | Bacteroidetes | Bacteria |
| ASV401 | 402 | 1 | 1 | 402 | 100 | NR_102884.1 Ruminococcus champanellensis 18P13 = JCM 17042 16S ribosomal RNA, partial sequence | NR_102884.1 | 1494 | 348 | 749 | 26.91 | 610.5 | 2E-174 | 380/404 | 94 | 4/404 | 0 | Plus | Plus | Bacteria; Firmicutes; Clostridia; Eubacteriales; Oscillospiraceae; Ruminococcus; Ruminococcus champanellensis | Ruminococcus champanellensis | Ruminococcus | Oscillospiraceae | Eubacteriales | Clostridia | Firmicutes | Bacteria |
| ASV402 | 405 | 1 | 1 | 405 | 100 | NR_118554.1 Intestinimonas butyriciproducens strain SRB-521-5-I 16S ribosomal RNA, partial sequence | NR_118554.1 | 1475 | 346 | 750 | 27.46 | 593.9 | 2E-169 | 377/405 | 93 | 0/405 | 0 | Plus | Plus | Bacteria; Firmicutes; Clostridia; Eubacteriales; ; Intestinimonas; Intestinimonas butyriciproducens | Intestinimonas butyriciproducens | Intestinimonas | | Eubacteriales | Clostridia | Firmicutes | Bacteria |
| ASV403 | 423 | 1 | 1 | 423 | 100 | NR_144750.1 Prevotellamassilia timonensis strain Marseille-P2831 16S ribosomal RNA, partial sequence | NR_144750.1 | 1493 | 358 | 779 | 28.27 | 569.9 | 3E-162 | 387/425 | 91 | 5/425 | 1 | Plus | Plus | Bacteria; Bacteroidetes; Bacteroidia; Bacteroidales; Prevotellaceae; Prevotellamassilia; Prevotellamassilia timonensis | Prevotellamassilia timonensis | Prevotellamassilia | Prevotellaceae | Bacteroidales | Bacteroidia | Bacteroidetes | Bacteria |
| ASV404 | 402 | 1 | 1 | 402 | 100 | NR_109014.1 Blautia faecis strain M25 16S ribosomal RNA, partial sequence | NR_109014.1 | 1262 | 212 | 613 | 31.85 | 704.7 | 0 | 395/402 | 98 | 0/402 | 0 | Plus | Plus | Bacteria; Firmicutes; Clostridia; Eubacteriales; Lachnospiraceae; Blautia; Blautia faecis | Blautia faecis | Blautia | Lachnospiraceae | Eubacteriales | Clostridia | Firmicutes | Bacteria |
| ASV405 | 405 | 1 | 1 | 405 | 100 | NR_028827.1 Shuttleworthia satelles DSM 14600 strain VPI D143K-13 16S ribosomal RNA, partial sequence | NR_028827.1 | 1456 | 345 | 749 | 27.82 | 621.6 | 9E-178 | 383/406 | 94 | 2/406 | 0 | Plus | Plus | Bacteria; Firmicutes; Clostridia; Eubacteriales; Lachnospiraceae; Shuttleworthia; Shuttleworthia satelles | Shuttleworthia satelles | Shuttleworthia | Lachnospiraceae | Eubacteriales | Clostridia | Firmicutes | Bacteria |
| ASV406 | 422 | 1 | 1 | 422 | 100 | NR_041364.1 Prevotella stercorea DSM 18206 strain CB35 16S ribosomal RNA, partial sequence | NR_041364.1 | 1488 | 354 | 775 | 28.36 | 597.6 | 2E-170 | 389/422 | 92 | 0/422 | 0 | Plus | Plus | Bacteria; Bacteroidetes; Bacteroidia; Bacteroidales; Prevotellaceae; Prevotella; Prevotella stercorea | Prevotella stercorea | Prevotella | Prevotellaceae | Bacteroidales | Bacteroidia | Bacteroidetes | Bacteria |
| ASV407 | 420 | 1 | 1 | 420 | 100 | NR_170509.1 Duncaniella freteri strain TLL-A3 16S ribosomal RNA, partial sequence | NR_170509.1 | 1538 | 364 | 784 | 27.37 | 460.9 | 2E-129 | 368/425 | 86 | 9/425 | 2 | Plus | Plus | Bacteria; Bacteroidetes; Bacteroidia; Bacteroidales; Muribaculaceae; Duncaniella; Duncaniella freteri | Duncaniella freteri | Duncaniella | Muribaculaceae | Bacteroidales | Bacteroidia | Bacteroidetes | Bacteria |
| ASV408 | 422 | 1 | 1 | 422 | 100 | NR_041364.1 Prevotella stercorea DSM 18206 strain CB35 16S ribosomal RNA, partial sequence | NR_041364.1 | 1488 | 354 | 775 | 28.36 | 725 | 0 | 412/422 | 97 | 0/422 | 0 | Plus | Plus | Bacteria; Bacteroidetes; Bacteroidia; Bacteroidales; Prevotellaceae; Prevotella; Prevotella stercorea | Prevotella stercorea | Prevotella | Prevotellaceae | Bacteroidales | Bacteroidia | Bacteroidetes | Bacteria |
| ASV409 | 402 | 1 | 1 | 402 | 100 | NR_118692.1 Blautia obeum ATCC 29174 16S ribosomal RNA, partial sequence | NR_118692.1 | 1398 | 330 | 731 | 28.76 | 701 | 0 | 394/402 | 98 | 0/402 | 0 | Plus | Plus | Bacteria; Firmicutes; Clostridia; Eubacteriales; Lachnospiraceae; Blautia; Blautia obeum | Blautia obeum | Blautia | Lachnospiraceae | Eubacteriales | Clostridia | Firmicutes | Bacteria |
| ASV410 | 422 | 1 | 1 | 422 | 100 | NR_041285.1 Prevotella multisaccharivorax strain PPPA20 16S ribosomal RNA, partial sequence | NR_041285.1 | 1491 | 356 | 777 | 28.3 | 625.3 | 7E-179 | 394/422 | 93 | 0/422 | 0 | Plus | Plus | Bacteria; Bacteroidetes; Bacteroidia; Bacteroidales; Prevotellaceae; Prevotella; Prevotella multisaccharivorax | Prevotella multisaccharivorax | Prevotella | Prevotellaceae | Bacteroidales | Bacteroidia | Bacteroidetes | Bacteria |
| ASV411 | 402 | 1 | 1 | 402 | 100 | NR_109014.1 Blautia faecis strain M25 16S ribosomal RNA, partial sequence | NR_109014.1 | 1262 | 212 | 613 | 31.85 | 715.8 | 0 | 397/402 | 98 | 0/402 | 0 | Plus | Plus | Bacteria; Firmicutes; Clostridia; Eubacteriales; Lachnospiraceae; Blautia; Blautia faecis | Blautia faecis | Blautia | Lachnospiraceae | Eubacteriales | Clostridia | Firmicutes | Bacteria |
| ASV412 | 422 | 2 | 1 | 422 | 100 | NR_113117.1 Prevotella oralis ATCC 33269 strain JCM 12251 16S ribosomal RNA, partial sequence | NR_113117.1 | 1496 | 361 | 782 | 28.21 | 597.6 | 2E-170 | 390/423 | 92 | 2/423 | 0 | Plus | Plus | Bacteria; Bacteroidetes; Bacteroidia; Bacteroidales; Prevotellaceae; Prevotella; Prevotella oralis | Prevotella oralis | Prevotella | Prevotellaceae | Bacteroidales | Bacteroidia | Bacteroidetes | Bacteria |
| ASV413 | 427 | 1 | 1 | 427 | 100 | NR_041778.1 Desulfovibrio piger strain ATCC 29098 16S ribosomal RNA, partial sequence | NR_041778.1 | 1542 | 357 | 783 | 27.69 | 773 | 0 | 424/427 | 99 | 0/427 | 0 | Plus | Plus | Bacteria; Proteobacteria; Deltaproteobacteria; Desulfovibrionales; Desulfovibrionaceae; Desulfovibrio; Desulfovibrio piger | Desulfovibrio piger | Desulfovibrio | Desulfovibrionaceae | Desulfovibrionales | Deltaproteobacteria | Proteobacteria | Bacteria |
| ASV414 | 404 | 1 | 1 | 404 | 100 | NR_044972.1 Sporobacter termitidis strain SYR 16S ribosomal RNA, partial sequence | NR_044972.1 | 1506 | 348 | 752 | 26.89 | 617.9 | 1E-176 | 381/405 | 94 | 1/405 | 0 | Plus | Plus | Bacteria; Firmicutes; Clostridia; Eubacteriales; Oscillospiraceae; Sporobacter; Sporobacter termitidis | Sporobacter termitidis | Sporobacter | Oscillospiraceae | Eubacteriales | Clostridia | Firmicutes | Bacteria |
| ASV415 | 405 | 1 | 1 | 405 | 100 | NR_025025.1 Papillibacter cinnamivorans strain CIN1 16S ribosomal RNA, partial sequence | NR_025025.1 | 1491 | 349 | 751 | 27.03 | 536.6 | 3E-152 | 367/406 | 90 | 4/406 | 0 | Plus | Plus | Bacteria; Firmicutes; Clostridia; Eubacteriales; Oscillospiraceae; Papillibacter; Papillibacter cinnamivorans | Papillibacter cinnamivorans | Papillibacter | Oscillospiraceae | Eubacteriales | Clostridia | Firmicutes | Bacteria |
| ASV416 | 401 | 1 | 1 | 401 | 100 | NR_144737.1 Emergencia timonensis strain SN18 16S ribosomal RNA, partial sequence | NR_144737.1 | 1480 | 364 | 767 | 27.3 | 610.5 | 2E-174 | 380/404 | 94 | 3/404 | 0 | Plus | Plus | Bacteria; Firmicutes; Clostridia; Eubacteriales; Clostridiales Family XIII. Incertae Sedis; Emergencia; Emergencia timonensis | Emergencia timonensis | Emergencia | Clostridiales Family XIII. Incertae Sedis | Eubacteriales | Clostridia | Firmicutes | Bacteria |
| ASV417 | 422 | 1 | 1 | 422 | 100 | NR_144750.1 Prevotellamassilia timonensis strain Marseille-P2831 16S ribosomal RNA, partial sequence | NR_144750.1 | 1493 | 358 | 779 | 28.27 | 553.3 | 4E-157 | 381/422 | 90 | 0/422 | 0 | Plus | Plus | Bacteria; Bacteroidetes; Bacteroidia; Bacteroidales; Prevotellaceae; Prevotellamassilia; Prevotellamassilia timonensis | Prevotellamassilia timonensis | Prevotellamassilia | Prevotellaceae | Bacteroidales | Bacteroidia | Bacteroidetes | Bacteria |
| ASV418 | 405 | 1 | 1 | 405 | 100 | NR_044972.1 Sporobacter termitidis strain SYR 16S ribosomal RNA, partial sequence | NR_044972.1 | 1506 | 348 | 752 | 26.89 | 531.1 | 2E-150 | 366/406 | 90 | 2/406 | 0 | Plus | Plus | Bacteria; Firmicutes; Clostridia; Eubacteriales; Oscillospiraceae; Sporobacter; Sporobacter termitidis | Sporobacter termitidis | Sporobacter | Oscillospiraceae | Eubacteriales | Clostridia | Firmicutes | Bacteria |
| ASV419 | 403 | 1 | 1 | 403 | 100 | NR_104907.1 Eubacterium coprostanoligenes strain HL 16S ribosomal RNA, partial sequence | NR_104907.1 | 1446 | 332 | 734 | 27.87 | 568 | 1E-161 | 371/403 | 92 | 0/403 | 0 | Plus | Plus | Bacteria; Firmicutes; Clostridia; Eubacteriales; Eubacteriaceae; Eubacterium; Eubacterium coprostanoligenes | Eubacterium coprostanoligenes | Eubacterium | Eubacteriaceae | Eubacteriales | Clostridia | Firmicutes | Bacteria |
| ASV420 | 404 | 1 | 1 | 404 | 100 | NR_148619.1 Agathobacter ruminis strain JK623 16S ribosomal RNA, partial sequence | NR_148619.1 | 1434 | 318 | 721 | 28.17 | 730.5 | 0 | 402/405 | 99 | 2/405 | 0 | Plus | Plus | Bacteria; Firmicutes; Clostridia; Eubacteriales; Lachnospiraceae; Agathobacter; Agathobacter ruminis | Agathobacter ruminis | Agathobacter | Lachnospiraceae | Eubacteriales | Clostridia | Firmicutes | Bacteria |
| ASV421 | 403 | 1 | 1 | 403 | 100 | NR_113316.1 Collinsella aerofaciens strain JCM 10188 16S ribosomal RNA, partial sequence | NR_113316.1 | 1467 | 344 | 746 | 27.47 | 728.7 | 0 | 400/403 | 99 | 0/403 | 0 | Plus | Plus | Bacteria; Actinobacteria; Coriobacteriia; Coriobacteriales; Coriobacteriaceae; Collinsella; Collinsella aerofaciens | Collinsella aerofaciens | Collinsella | Coriobacteriaceae | Coriobacteriales | Coriobacteriia | Actinobacteria | Bacteria |
| ASV422 | 403 | 5 | 1 | 403 | 100 | NR_102884.1 Ruminococcus champanellensis 18P13 = JCM 17042 16S ribosomal RNA, partial sequence | NR_102884.1 | 1494 | 348 | 749 | 26.91 | 632.7 | 0 | 383/403 | 95 | 1/403 | 0 | Plus | Plus | Bacteria; Firmicutes; Clostridia; Eubacteriales; Oscillospiraceae; Ruminococcus; Ruminococcus champanellensis | Ruminococcus champanellensis | Ruminococcus | Oscillospiraceae | Eubacteriales | Clostridia | Firmicutes | Bacteria |
| ASV423 | 402 | 1 | 1 | 402 | 100 | NR_151895.1 Anaerocolumna cellulosilytica strain SN021 16S ribosomal RNA, partial sequence | NR_151895.1 | 1478 | 353 | 754 | 27.2 | 677 | 0 | 391/403 | 97 | 2/403 | 0 | Plus | Plus | Bacteria; Firmicutes; Clostridia; Eubacteriales; Lachnospiraceae; Anaerocolumna; Anaerocolumna cellulosilytica | Anaerocolumna cellulosilytica | Anaerocolumna | Lachnospiraceae | Eubacteriales | Clostridia | Firmicutes | Bacteria |
| ASV425 | 422 | 1 | 1 | 422 | 100 | NR_041364.1 Prevotella stercorea DSM 18206 strain CB35 16S ribosomal RNA, partial sequence | NR_041364.1 | 1488 | 354 | 775 | 28.36 | 708.4 | 0 | 409/422 | 96 | 0/422 | 0 | Plus | Plus | Bacteria; Bacteroidetes; Bacteroidia; Bacteroidales; Prevotellaceae; Prevotella; Prevotella stercorea | Prevotella stercorea | Prevotella | Prevotellaceae | Bacteroidales | Bacteroidia | Bacteroidetes | Bacteria |
| ASV426 | 422 | 1 | 1 | 422 | 100 | NR_113411.1 Prevotella copri DSM 18205 strain JCM 13464 16S ribosomal RNA, partial sequence | NR_113411.1 | 1493 | 358 | 779 | 28.27 | 747.2 | 0 | 416/422 | 98 | 0/422 | 0 | Plus | Plus | Bacteria; Bacteroidetes; Bacteroidia; Bacteroidales; Prevotellaceae; Prevotella; Prevotella copri | Prevotella copri | Prevotella | Prevotellaceae | Bacteroidales | Bacteroidia | Bacteroidetes | Bacteria |
| ASV427 | 422 | 2 | 1 | 422 | 100 | NR_113117.1 Prevotella oralis ATCC 33269 strain JCM 12251 16S ribosomal RNA, partial sequence | NR_113117.1 | 1496 | 361 | 782 | 28.21 | 619.7 | 3E-177 | 393/422 | 93 | 0/422 | 0 | Plus | Plus | Bacteria; Bacteroidetes; Bacteroidia; Bacteroidales; Prevotellaceae; Prevotella; Prevotella oralis | Prevotella oralis | Prevotella | Prevotellaceae | Bacteroidales | Bacteroidia | Bacteroidetes | Bacteria |
| ASV428 | 423 | 1 | 1 | 423 | 100 | NR_144750.1 Prevotellamassilia timonensis strain Marseille-P2831 16S ribosomal RNA, partial sequence | NR_144750.1 | 1493 | 358 | 779 | 28.27 | 564.3 | 2E-160 | 386/425 | 90 | 5/425 | 1 | Plus | Plus | Bacteria; Bacteroidetes; Bacteroidia; Bacteroidales; Prevotellaceae; Prevotellamassilia; Prevotellamassilia timonensis | Prevotellamassilia timonensis | Prevotellamassilia | Prevotellaceae | Bacteroidales | Bacteroidia | Bacteroidetes | Bacteria |
| ASV429 | 402 | 1 | 1 | 402 | 100 | NR_118675.1 [Eubacterium] siraeum strain ATCC 29066 16S ribosomal RNA, partial sequence | NR_118675.1 | 1453 | 356 | 757 | 27.67 | 595.7 | 5E-170 | 374/402 | 93 | 0/402 | 0 | Plus | Plus | Bacteria; Firmicutes; Clostridia; Eubacteriales; Oscillospiraceae; ; [Eubacterium] siraeum | [Eubacterium] siraeum | | Oscillospiraceae | Eubacteriales | Clostridia | Firmicutes | Bacteria |
| ASV430 | 405 | 1 | 1 | 405 | 100 | NR_104907.1 Eubacterium coprostanoligenes strain HL 16S ribosomal RNA, partial sequence | NR_104907.1 | 1446 | 332 | 734 | 27.87 | 608.7 | 7E-174 | 380/405 | 93 | 2/405 | 0 | Plus | Plus | Bacteria; Firmicutes; Clostridia; Eubacteriales; Eubacteriaceae; Eubacterium; Eubacterium coprostanoligenes | Eubacterium coprostanoligenes | Eubacterium | Eubacteriaceae | Eubacteriales | Clostridia | Firmicutes | Bacteria |
| ASV431 | 405 | 1 | 1 | 405 | 100 | NR_151982.1 Agathobaculum butyriciproducens strain SR79 16S ribosomal RNA, partial sequence | NR_151982.1 | 1383 | 265 | 669 | 29.28 | 749 | 0 | 405/405 | 100 | 0/405 | 0 | Plus | Plus | Bacteria; Firmicutes; Clostridia; Eubacteriales; Oscillospiraceae; Agathobaculum; Agathobaculum butyriciproducens | Agathobaculum butyriciproducens | Agathobaculum | Oscillospiraceae | Eubacteriales | Clostridia | Firmicutes | Bacteria |
| ASV433 | 403 | 1 | 1 | 403 | 100 | NR_104907.1 Eubacterium coprostanoligenes strain HL 16S ribosomal RNA, partial sequence | NR_104907.1 | 1446 | 332 | 734 | 27.87 | 562.5 | 6E-160 | 370/403 | 91 | 0/403 | 0 | Plus | Plus | Bacteria; Firmicutes; Clostridia; Eubacteriales; Eubacteriaceae; Eubacterium; Eubacterium coprostanoligenes | Eubacterium coprostanoligenes | Eubacterium | Eubacteriaceae | Eubacteriales | Clostridia | Firmicutes | Bacteria |
| ASV434 | 404 | 1 | 1 | 404 | 100 | NR_042074.1 Eubacterium pyruvativorans strain I-6 16S ribosomal RNA, partial sequence | NR_042074.1 | 1296 | 264 | 667 | 31.17 | 747.2 | 0 | 404/404 | 100 | 0/404 | 0 | Plus | Plus | Bacteria; Firmicutes; Clostridia; Eubacteriales; Eubacteriaceae; Eubacterium; Eubacterium pyruvativorans | Eubacterium pyruvativorans | Eubacterium | Eubacteriaceae | Eubacteriales | Clostridia | Firmicutes | Bacteria |
| ASV435 | 405 | 1 | 1 | 405 | 100 | NR_144611.1 Flintibacter butyricus strain BLS21 16S ribosomal RNA, partial sequence | NR_144611.1 | 808 | 299 | 703 | 50.12 | 538.5 | 9E-153 | 368/406 | 90 | 2/406 | 0 | Plus | Plus | Bacteria; Firmicutes; Clostridia; Eubacteriales; ; Flintibacter; Flintibacter butyricus | Flintibacter butyricus | Flintibacter | | Eubacteriales | Clostridia | Firmicutes | Bacteria |
| ASV436 | 427 | 1 | 1 | 427 | 100 | NR_041666.1 Dialister succinatiphilus YIT 11850 16S ribosomal RNA, partial sequence | NR_041666.1 | 1521 | 353 | 779 | 28.07 | 784.1 | 0 | 426/427 | 99 | 0/427 | 0 | Plus | Plus | Bacteria; Firmicutes; Negativicutes; Veillonellales; Veillonellaceae; Dialister; Dialister succinatiphilus | Dialister succinatiphilus | Dialister | Veillonellaceae | Veillonellales | Negativicutes | Firmicutes | Bacteria |
| ASV437 | 402 | 1 | 1 | 402 | 100 | NR_025931.1 Ruminococcus flavefaciens strain C94 16S ribosomal RNA, partial sequence | NR_025931.1 | 1388 | 325 | 726 | 28.96 | 726.9 | 0 | 399/402 | 99 | 0/402 | 0 | Plus | Plus | Bacteria; Firmicutes; Clostridia; Eubacteriales; Oscillospiraceae; Ruminococcus; Ruminococcus flavefaciens | Ruminococcus flavefaciens | Ruminococcus | Oscillospiraceae | Eubacteriales | Clostridia | Firmicutes | Bacteria |
| ASV438 | 402 | 2 | 1 | 402 | 100 | NR_104892.1 Clostridium moniliforme strain 2055 16S ribosomal RNA, partial sequence | NR_104892.1 | 1488 | 330 | 731 | 27.02 | 732.4 | 0 | 400/402 | 99 | 0/402 | 0 | Plus | Plus | Bacteria; Firmicutes; Clostridia; Eubacteriales; Clostridiaceae; Clostridium; Clostridium moniliforme | Clostridium moniliforme | Clostridium | Clostridiaceae | Eubacteriales | Clostridia | Firmicutes | Bacteria |
| ASV439 | 405 | 2 | 3 | 405 | 99.51 | NR_144742.1 Christensenella massiliensis strain Marseille-P2438 16S ribosomal RNA, partial sequence | NR_144742.1 | 1499 | 371 | 773 | 26.88 | 540.3 | 3E-153 | 366/403 | 90 | 0/403 | 0 | Plus | Plus | Bacteria; Firmicutes; Clostridia; Eubacteriales; Christensenellaceae; Christensenella; Christensenella massiliensis | Christensenella massiliensis | Christensenella | Christensenellaceae | Eubacteriales | Clostridia | Firmicutes | Bacteria |
| ASV440 | 422 | 2 | 1 | 422 | 100 | NR_113117.1 Prevotella oralis ATCC 33269 strain JCM 12251 16S ribosomal RNA, partial sequence | NR_113117.1 | 1496 | 361 | 782 | 28.21 | 608.7 | 7E-174 | 391/422 | 92 | 0/422 | 0 | Plus | Plus | Bacteria; Bacteroidetes; Bacteroidia; Bacteroidales; Prevotellaceae; Prevotella; Prevotella oralis | Prevotella oralis | Prevotella | Prevotellaceae | Bacteroidales | Bacteroidia | Bacteroidetes | Bacteria |
| ASV441 | 402 | 1 | 1 | 402 | 100 | NR_044289.1 [Clostridium] lavalense strain CCRI-9842 16S ribosomal RNA, partial sequence | NR_044289.1 | 1454 | 345 | 746 | 27.65 | 654.8 | 0 | 386/402 | 96 | 0/402 | 0 | Plus | Plus | Bacteria; Firmicutes; Clostridia; Eubacteriales; Lachnospiraceae; Enterocloster; Enterocloster lavalensis | Enterocloster lavalensis | Enterocloster | Lachnospiraceae | Eubacteriales | Clostridia | Firmicutes | Bacteria |
| ASV442 | 422 | 1 | 1 | 422 | 100 | NR_113098.1 Prevotella buccalis strain JCM 12246 16S ribosomal RNA, partial sequence | NR_113098.1 | 1493 | 355 | 777 | 28.27 | 630.8 | 2E-180 | 396/423 | 93 | 2/423 | 0 | Plus | Plus | Bacteria; Bacteroidetes; Bacteroidia; Bacteroidales; Prevotellaceae; Prevotella; Prevotella buccalis | Prevotella buccalis | Prevotella | Prevotellaceae | Bacteroidales | Bacteroidia | Bacteroidetes | Bacteria |
| ASV443 | 404 | 1 | 1 | 404 | 100 | NR_042074.1 Eubacterium pyruvativorans strain I-6 16S ribosomal RNA, partial sequence | NR_042074.1 | 1296 | 264 | 667 | 31.17 | 569.9 | 3E-162 | 372/404 | 92 | 0/404 | 0 | Plus | Plus | Bacteria; Firmicutes; Clostridia; Eubacteriales; Eubacteriaceae; Eubacterium; Eubacterium pyruvativorans | Eubacterium pyruvativorans | Eubacterium | Eubacteriaceae | Eubacteriales | Clostridia | Firmicutes | Bacteria |
| ASV444 | 405 | 1 | 1 | 405 | 100 | NR_118554.1 Intestinimonas butyriciproducens strain SRB-521-5-I 16S ribosomal RNA, partial sequence | NR_118554.1 | 1475 | 346 | 750 | 27.46 | 582.8 | 4E-166 | 375/405 | 92 | 0/405 | 0 | Plus | Plus | Bacteria; Firmicutes; Clostridia; Eubacteriales; ; Intestinimonas; Intestinimonas butyriciproducens | Intestinimonas butyriciproducens | Intestinimonas | | Eubacteriales | Clostridia | Firmicutes | Bacteria |
| ASV445 | 418 | 1 | 1 | 418 | 100 | NR_102893.1 Butyrivibrio proteoclasticus strain B316 16S ribosomal RNA, partial sequence | NR_102893.1 | 1537 | 367 | 782 | 27.07 | 593.9 | 2E-169 | 386/418 | 92 | 2/418 | 0 | Plus | Plus | Bacteria; Firmicutes; Clostridia; Eubacteriales; Lachnospiraceae; Butyrivibrio; Butyrivibrio proteoclasticus | Butyrivibrio proteoclasticus | Butyrivibrio | Lachnospiraceae | Eubacteriales | Clostridia | Firmicutes | Bacteria |
| ASV446 | 402 | 1 | 2 | 402 | 99.75 | NR_027579.1 Ruminococcus lactaris ATCC 29176 16S ribosomal RNA, partial sequence | NR_027579.1 | 1402 | 334 | 734 | 28.6 | 688.1 | 0 | 391/401 | 97 | 0/401 | 0 | Plus | Plus | Bacteria; Firmicutes; Clostridia; Eubacteriales; Lachnospiraceae; Mediterraneibacter; [Ruminococcus] lactaris | [Ruminococcus] lactaris | Mediterraneibacter | Lachnospiraceae | Eubacteriales | Clostridia | Firmicutes | Bacteria |
| ASV447 | 427 | 3 | 1 | 426 | 99.77 | NR_041781.1 Streptococcus alactolyticus strain ATCC 43077 16S ribosomal RNA, partial sequence | NR_041781.1 | 1437 | 346 | 771 | 29.65 | 771.2 | 0 | 423/426 | 99 | 0/426 | 0 | Plus | Plus | Bacteria; Firmicutes; Bacilli; Lactobacillales; Streptococcaceae; Streptococcus; Streptococcus alactolyticus | Streptococcus alactolyticus | Streptococcus | Streptococcaceae | Lactobacillales | Bacilli | Firmicutes | Bacteria |
| ASV448 | 404 | 1 | 1 | 404 | 100 | NR_118554.1 Intestinimonas butyriciproducens strain SRB-521-5-I 16S ribosomal RNA, partial sequence | NR_118554.1 | 1475 | 346 | 750 | 27.46 | 497.9 | 2E-140 | 360/405 | 88 | 1/405 | 0 | Plus | Plus | Bacteria; Firmicutes; Clostridia; Eubacteriales; ; Intestinimonas; Intestinimonas butyriciproducens | Intestinimonas butyriciproducens | Intestinimonas | | Eubacteriales | Clostridia | Firmicutes | Bacteria |
| ASV449 | 404 | 1 | 1 | 404 | 100 | NR_118156.1 Oscillibacter ruminantium GH1 16S ribosomal RNA, partial sequence | NR_118156.1 | 1409 | 321 | 724 | 28.67 | 614.2 | 2E-175 | 382/406 | 94 | 4/406 | 0 | Plus | Plus | Bacteria; Firmicutes; Clostridia; Eubacteriales; Oscillospiraceae; Oscillibacter; Oscillibacter ruminantium | Oscillibacter ruminantium | Oscillibacter | Oscillospiraceae | Eubacteriales | Clostridia | Firmicutes | Bacteria |
| ASV450 | 405 | 2 | 1 | 405 | 100 | NR_074629.1 Hungateiclostridium thermocellum strain ATCC 27405 16S ribosomal RNA, partial sequence | NR_074629.1 | 1521 | 358 | 762 | 26.63 | 533 | 4E-151 | 367/406 | 90 | 2/406 | 0 | Plus | Plus | Bacteria; Firmicutes; Clostridia; Eubacteriales; Oscillospiraceae; Acetivibrio; Acetivibrio thermocellus | Acetivibrio thermocellus | Acetivibrio | Oscillospiraceae | Eubacteriales | Clostridia | Firmicutes | Bacteria |
| ASV451 | 402 | 1 | 1 | 402 | 100 | NR_029239.3 Clostridium chartatabidum strain 163 16S ribosomal RNA, partial sequence | NR_029239.3 | 1460 | 323 | 724 | 27.53 | 715.8 | 0 | 397/402 | 98 | 0/402 | 0 | Plus | Plus | Bacteria; Firmicutes; Clostridia; Eubacteriales; Clostridiaceae; Clostridium; Clostridium chartatabidum | Clostridium chartatabidum | Clostridium | Clostridiaceae | Eubacteriales | Clostridia | Firmicutes | Bacteria |
| ASV453 | 402 | 2 | 1 | 402 | 100 | NR_042832.1 Roseburia faecis strain M72/1 16S ribosomal RNA, partial sequence | NR_042832.1 | 1456 | 347 | 749 | 27.68 | 654.8 | 0 | 387/403 | 96 | 1/403 | 0 | Plus | Plus | Bacteria; Firmicutes; Clostridia; Eubacteriales; Lachnospiraceae; Roseburia; Roseburia faecis | Roseburia faecis | Roseburia | Lachnospiraceae | Eubacteriales | Clostridia | Firmicutes | Bacteria |
| ASV454 | 423 | 1 | 1 | 423 | 100 | NR_147368.1 Millionella massiliensis strain Marseille-P3215 16S ribosomal RNA, partial sequence | NR_147368.1 | 1492 | 355 | 776 | 28.28 | 442.5 | 8E-124 | 367/428 | 85 | 11/428 | 2 | Plus | Plus | Bacteria; Bacteroidetes; Bacteroidia; Bacteroidales; Rikenellaceae; Millionella; Millionella massiliensis | Millionella massiliensis | Millionella | Rikenellaceae | Bacteroidales | Bacteroidia | Bacteroidetes | Bacteria |
| ASV455 | 427 | 1 | 1 | 427 | 100 | NR_027596.1 Mitsuokella multacida strain DSM 20544 16S ribosomal RNA, partial sequence | NR_027596.1 | 1511 | 346 | 772 | 28.26 | 785.9 | 0 | 426/427 | 99 | 0/427 | 0 | Plus | Plus | Bacteria; Firmicutes; Negativicutes; Selenomonadales; Selenomonadaceae; Mitsuokella; Mitsuokella multacida | Mitsuokella multacida | Mitsuokella | Selenomonadaceae | Selenomonadales | Negativicutes | Firmicutes | Bacteria |
| ASV456 | 404 | 2 | 1 | 404 | 100 | NR_074793.2 Oscillibacter valericigenes strain Sjm18-20 16S ribosomal RNA, complete sequence | NR_074793.2 | 1530 | 372 | 775 | 26.41 | 619.7 | 3E-177 | 381/404 | 94 | 0/404 | 0 | Plus | Plus | Bacteria; Firmicutes; Clostridia; Eubacteriales; Oscillospiraceae; Oscillibacter; Oscillibacter valericigenes | Oscillibacter valericigenes | Oscillibacter | Oscillospiraceae | Eubacteriales | Clostridia | Firmicutes | Bacteria |
| ASV457 | 427 | 1 | 1 | 427 | 100 | NR_044111.1 Selenomonas bovis strain WG 16S ribosomal RNA, partial sequence | NR_044111.1 | 1525 | 370 | 796 | 28 | 756.4 | 0 | 421/427 | 98 | 0/427 | 0 | Plus | Plus | Bacteria; Firmicutes; Negativicutes; Selenomonadales; Selenomonadaceae; Selenomonas; Selenomonas bovis | Selenomonas bovis | Selenomonas | Selenomonadaceae | Selenomonadales | Negativicutes | Firmicutes | Bacteria |
| ASV458 | 404 | 1 | 1 | 404 | 100 | NR_042832.1 Roseburia faecis strain M72/1 16S ribosomal RNA, partial sequence | NR_042832.1 | 1456 | 347 | 749 | 27.68 | 739.8 | 0 | 403/404 | 99 | 1/404 | 0 | Plus | Plus | Bacteria; Firmicutes; Clostridia; Eubacteriales; Lachnospiraceae; Roseburia; Roseburia faecis | Roseburia faecis | Roseburia | Lachnospiraceae | Eubacteriales | Clostridia | Firmicutes | Bacteria |
| ASV459 | 422 | 2 | 1 | 422 | 100 | NR_113117.1 Prevotella oralis ATCC 33269 strain JCM 12251 16S ribosomal RNA, partial sequence | NR_113117.1 | 1496 | 361 | 782 | 28.21 | 597.6 | 2E-170 | 390/423 | 92 | 2/423 | 0 | Plus | Plus | Bacteria; Bacteroidetes; Bacteroidia; Bacteroidales; Prevotellaceae; Prevotella; Prevotella oralis | Prevotella oralis | Prevotella | Prevotellaceae | Bacteroidales | Bacteroidia | Bacteroidetes | Bacteria |
| ASV460 | 404 | 1 | 1 | 404 | 100 | NR_118156.1 Oscillibacter ruminantium GH1 16S ribosomal RNA, partial sequence | NR_118156.1 | 1409 | 321 | 724 | 28.67 | 614.2 | 2E-175 | 381/405 | 94 | 2/405 | 0 | Plus | Plus | Bacteria; Firmicutes; Clostridia; Eubacteriales; Oscillospiraceae; Oscillibacter; Oscillibacter ruminantium | Oscillibacter ruminantium | Oscillibacter | Oscillospiraceae | Eubacteriales | Clostridia | Firmicutes | Bacteria |
| ASV461 | 422 | 5 | 1 | 422 | 100 | NR_113119.1 Prevotella oris strain JCM 8540 16S ribosomal RNA, partial sequence | NR_113119.1 | 1481 | 345 | 766 | 28.49 | 564.3 | 2E-160 | 385/424 | 90 | 4/424 | 0 | Plus | Plus | Bacteria; Bacteroidetes; Bacteroidia; Bacteroidales; Prevotellaceae; Prevotella; Prevotella oris | Prevotella oris | Prevotella | Prevotellaceae | Bacteroidales | Bacteroidia | Bacteroidetes | Bacteria |
| ASV462 | 402 | 1 | 1 | 402 | 100 | NR_118667.1 Lachnospira pectinoschiza strain 150-1 16S ribosomal RNA, partial sequence | NR_118667.1 | 1465 | 372 | 773 | 27.44 | 678.8 | 0 | 388/402 | 96 | 0/402 | 0 | Plus | Plus | Bacteria; Firmicutes; Clostridia; Eubacteriales; Lachnospiraceae; Lachnospira; Lachnospira pectinoschiza | Lachnospira pectinoschiza | Lachnospira | Lachnospiraceae | Eubacteriales | Clostridia | Firmicutes | Bacteria |
| ASV463 | 405 | 2 | 1 | 405 | 100 | NR_044972.1 Sporobacter termitidis strain SYR 16S ribosomal RNA, partial sequence | NR_044972.1 | 1506 | 348 | 752 | 26.89 | 575.4 | 7E-164 | 374/406 | 92 | 2/406 | 0 | Plus | Plus | Bacteria; Firmicutes; Clostridia; Eubacteriales; Oscillospiraceae; Sporobacter; Sporobacter termitidis | Sporobacter termitidis | Sporobacter | Oscillospiraceae | Eubacteriales | Clostridia | Firmicutes | Bacteria |
| ASV464 | 405 | 1 | 1 | 405 | 100 | NR_118554.1 Intestinimonas butyriciproducens strain SRB-521-5-I 16S ribosomal RNA, partial sequence | NR_118554.1 | 1475 | 346 | 750 | 27.46 | 533 | 4E-151 | 368/407 | 90 | 4/407 | 0 | Plus | Plus | Bacteria; Firmicutes; Clostridia; Eubacteriales; ; Intestinimonas; Intestinimonas butyriciproducens | Intestinimonas butyriciproducens | Intestinimonas | | Eubacteriales | Clostridia | Firmicutes | Bacteria |
| ASV465 | 422 | 2 | 1 | 422 | 100 | NR_113119.1 Prevotella oris strain JCM 8540 16S ribosomal RNA, partial sequence | NR_113119.1 | 1481 | 345 | 766 | 28.49 | 581 | 2E-165 | 387/423 | 91 | 2/423 | 0 | Plus | Plus | Bacteria; Bacteroidetes; Bacteroidia; Bacteroidales; Prevotellaceae; Prevotella; Prevotella oris | Prevotella oris | Prevotella | Prevotellaceae | Bacteroidales | Bacteroidia | Bacteroidetes | Bacteria |
| ASV466 | 422 | 1 | 1 | 422 | 100 | NR_109628.1 Prevotella jejuni strain CD3:28 16S ribosomal RNA, partial sequence | NR_109628.1 | 1484 | 339 | 760 | 28.44 | 586.5 | 3E-167 | 387/422 | 91 | 0/422 | 0 | Plus | Plus | Bacteria; Bacteroidetes; Bacteroidia; Bacteroidales; Prevotellaceae; Prevotella; Prevotella jejuni | Prevotella jejuni | Prevotella | Prevotellaceae | Bacteroidales | Bacteroidia | Bacteroidetes | Bacteria |
| ASV467 | 420 | 2 | 1 | 420 | 100 | NR_029335.1 Holdemania filiformis strain J1-31B-1 16S ribosomal RNA, partial sequence | NR_029335.1 | 1391 | 267 | 692 | 30.63 | 460.9 | 2E-129 | 368/426 | 86 | 6/426 | 1 | Plus | Plus | Bacteria; Firmicutes; Erysipelotrichia; Erysipelotrichales; Erysipelotrichaceae; Holdemania; Holdemania filiformis | Holdemania filiformis | Holdemania | Erysipelotrichaceae | Erysipelotrichales | Erysipelotrichia | Firmicutes | Bacteria |
| ASV468 | 422 | 1 | 1 | 422 | 100 | NR_041285.1 Prevotella multisaccharivorax strain PPPA20 16S ribosomal RNA, partial sequence | NR_041285.1 | 1491 | 356 | 777 | 28.3 | 619.7 | 3E-177 | 394/423 | 93 | 2/423 | 0 | Plus | Plus | Bacteria; Bacteroidetes; Bacteroidia; Bacteroidales; Prevotellaceae; Prevotella; Prevotella multisaccharivorax | Prevotella multisaccharivorax | Prevotella | Prevotellaceae | Bacteroidales | Bacteroidia | Bacteroidetes | Bacteria |
| ASV469 | 427 | 1 | 1 | 427 | 100 | NR_028840.1 Mitsuokella jalaludinii strain M9 16S ribosomal RNA, partial sequence | NR_028840.1 | 1543 | 362 | 788 | 27.67 | 761.9 | 0 | 422/427 | 98 | 0/427 | 0 | Plus | Plus | Bacteria; Firmicutes; Negativicutes; Selenomonadales; Selenomonadaceae; Mitsuokella; Mitsuokella jalaludinii | Mitsuokella jalaludinii | Mitsuokella | Selenomonadaceae | Selenomonadales | Negativicutes | Firmicutes | Bacteria |
| ASV470 | 427 | 4 | 1 | 427 | 100 | NR_042802.1 Lactobacillus ultunensis strain Kx146C1 16S ribosomal RNA, partial sequence | NR_042802.1 | 1550 | 370 | 796 | 27.55 | 784.1 | 0 | 426/427 | 99 | 0/427 | 0 | Plus | Plus | Bacteria; Firmicutes; Bacilli; Lactobacillales; Lactobacillaceae; Lactobacillus; Lactobacillus ultunensis | Lactobacillus ultunensis | Lactobacillus | Lactobacillaceae | Lactobacillales | Bacilli | Firmicutes | Bacteria |
| ASV471 | 422 | 1 | 1 | 422 | 100 | NR_113411.1 Prevotella copri DSM 18205 strain JCM 13464 16S ribosomal RNA, partial sequence | NR_113411.1 | 1493 | 358 | 779 | 28.27 | 730.5 | 0 | 413/422 | 97 | 0/422 | 0 | Plus | Plus | Bacteria; Bacteroidetes; Bacteroidia; Bacteroidales; Prevotellaceae; Prevotella; Prevotella copri | Prevotella copri | Prevotella | Prevotellaceae | Bacteroidales | Bacteroidia | Bacteroidetes | Bacteria |
| ASV472 | 408 | 1 | 1 | 408 | 100 | NR_134781.1 Olsenella scatoligenes strain SK9K4 16S ribosomal RNA, partial sequence | NR_134781.1 | 1469 | 348 | 755 | 27.77 | 754.6 | 0 | 408/408 | 100 | 0/408 | 0 | Plus | Plus | Bacteria; Actinobacteria; Coriobacteriia; Coriobacteriales; Atopobiaceae; Olsenella; Olsenella scatoligenes | Olsenella scatoligenes | Olsenella | Atopobiaceae | Coriobacteriales | Coriobacteriia | Actinobacteria | Bacteria |
| ASV473 | 427 | 3 | 1 | 427 | 100 | NR_041781.1 Streptococcus alactolyticus strain ATCC 43077 16S ribosomal RNA, partial sequence | NR_041781.1 | 1437 | 346 | 772 | 29.71 | 750.9 | 0 | 420/427 | 98 | 0/427 | 0 | Plus | Plus | Bacteria; Firmicutes; Bacilli; Lactobacillales; Streptococcaceae; Streptococcus; Streptococcus alactolyticus | Streptococcus alactolyticus | Streptococcus | Streptococcaceae | Lactobacillales | Bacilli | Firmicutes | Bacteria |
| ASV474 | 427 | 5 | 1 | 427 | 100 | NR_114743.1 Enterococcus hirae strain DSM 20160 16S ribosomal RNA, partial sequence | NR_114743.1 | 1535 | 359 | 785 | 27.82 | 789.6 | 0 | 427/427 | 100 | 0/427 | 0 | Plus | Plus | Bacteria; Firmicutes; Bacilli; Lactobacillales; Enterococcaceae; Enterococcus; Enterococcus hirae | Enterococcus hirae | Enterococcus | Enterococcaceae | Lactobacillales | Bacilli | Firmicutes | Bacteria |
| ASV475 | 406 | 1 | 1 | 406 | 100 | NR_026476.1 Succinivibrio dextrinosolvens strain 0554 16S ribosomal RNA, partial sequence | NR_026476.1 | 1463 | 331 | 732 | 27.48 | 680.7 | 0 | 394/406 | 97 | 4/406 | 0 | Plus | Plus | Bacteria; Proteobacteria; Gammaproteobacteria; Aeromonadales; Succinivibrionaceae; Succinivibrio; Succinivibrio dextrinosolvens | Succinivibrio dextrinosolvens | Succinivibrio | Succinivibrionaceae | Aeromonadales | Gammaproteobacteria | Proteobacteria | Bacteria |
| ASV476 | 404 | 1 | 1 | 404 | 100 | NR_042007.1 Roseburia inulinivorans DSM 16841 strain A2-194 16S ribosomal RNA, partial sequence | NR_042007.1 | 1454 | 360 | 763 | 27.79 | 747.2 | 0 | 404/404 | 100 | 0/404 | 0 | Plus | Plus | Bacteria; Firmicutes; Clostridia; Eubacteriales; Lachnospiraceae; Roseburia; Roseburia inulinivorans | Roseburia inulinivorans | Roseburia | Lachnospiraceae | Eubacteriales | Clostridia | Firmicutes | Bacteria |
| ASV477 | 402 | 1 | 1 | 402 | 100 | NR_144612.1 Frisingicoccus caecimuris strain PG-426-CC-1 16S ribosomal RNA, partial sequence | NR_144612.1 | 1448 | 367 | 768 | 27.76 | 710.2 | 0 | 396/402 | 98 | 0/402 | 0 | Plus | Plus | Bacteria; Firmicutes; Clostridia; Eubacteriales; Lachnospiraceae; Frisingicoccus; Frisingicoccus caecimuris | Frisingicoccus caecimuris | Frisingicoccus | Lachnospiraceae | Eubacteriales | Clostridia | Firmicutes | Bacteria |
| ASV478 | 404 | 2 | 1 | 404 | 100 | NR_074793.2 Oscillibacter valericigenes strain Sjm18-20 16S ribosomal RNA, complete sequence | NR_074793.2 | 1530 | 372 | 775 | 26.41 | 647.4 | 0 | 386/404 | 95 | 0/404 | 0 | Plus | Plus | Bacteria; Firmicutes; Clostridia; Eubacteriales; Oscillospiraceae; Oscillibacter; Oscillibacter valericigenes | Oscillibacter valericigenes | Oscillibacter | Oscillospiraceae | Eubacteriales | Clostridia | Firmicutes | Bacteria |
| ASV479 | 422 | 2 | 1 | 422 | 100 | NR_113119.1 Prevotella oris strain JCM 8540 16S ribosomal RNA, partial sequence | NR_113119.1 | 1481 | 345 | 766 | 28.49 | 619.7 | 3E-177 | 395/424 | 93 | 4/424 | 0 | Plus | Plus | Bacteria; Bacteroidetes; Bacteroidia; Bacteroidales; Prevotellaceae; Prevotella; Prevotella oris | Prevotella oris | Prevotella | Prevotellaceae | Bacteroidales | Bacteroidia | Bacteroidetes | Bacteria |
| ASV480 | 422 | 1 | 1 | 422 | 100 | NR_027196.1 Bacteroides stercoris ATCC 43183 16S ribosomal RNA, partial sequence | NR_027196.1 | 1342 | 334 | 755 | 31.45 | 665.9 | 0 | 401/422 | 95 | 0/422 | 0 | Plus | Plus | Bacteria; Bacteroidetes; Bacteroidia; Bacteroidales; Bacteroidaceae; Bacteroides; Bacteroides stercoris | Bacteroides stercoris | Bacteroides | Bacteroidaceae | Bacteroidales | Bacteroidia | Bacteroidetes | Bacteria |
| ASV481 | 403 | 1 | 3 | 403 | 99.5 | NR_125464.1 Anaerobacterium chartisolvens strain T-1-35 16S ribosomal RNA, partial sequence | NR_125464.1 | 1451 | 344 | 745 | 27.71 | 481.2 | 2E-135 | 355/402 | 88 | 1/402 | 0 | Plus | Plus | Bacteria; Firmicutes; Clostridia; Eubacteriales; Oscillospiraceae; Anaerobacterium; Anaerobacterium chartisolvens | Anaerobacterium chartisolvens | Anaerobacterium | Oscillospiraceae | Eubacteriales | Clostridia | Firmicutes | Bacteria |
| ASV482 | 422 | 1 | 1 | 422 | 100 | NR_144616.1 Muribaculum intestinale strain YL27 16S ribosomal RNA, partial sequence | NR_144616.1 | 1460 | 297 | 718 | 28.9 | 536.6 | 4E-152 | 382/426 | 89 | 8/426 | 1 | Plus | Plus | Bacteria; Bacteroidetes; Bacteroidia; Bacteroidales; Muribaculaceae; Muribaculum; Muribaculum intestinale | Muribaculum intestinale | Muribaculum | Muribaculaceae | Bacteroidales | Bacteroidia | Bacteroidetes | Bacteria |
| ASV483 | 427 | 2 | 1 | 427 | 100 | NR_037018.1 Acidaminococcus fermentans strain VR4 16S ribosomal RNA gene, partial sequence | NR_037018.1 | 1516 | 349 | 775 | 28.17 | 728.7 | 0 | 416/427 | 97 | 0/427 | 0 | Plus | Plus | Bacteria; Firmicutes; Negativicutes; Acidaminococcales; Acidaminococcaceae; Acidaminococcus; Acidaminococcus fermentans | Acidaminococcus fermentans | Acidaminococcus | Acidaminococcaceae | Acidaminococcales | Negativicutes | Firmicutes | Bacteria |
| ASV485 | 403 | 1 | 3 | 403 | 99.5 | NR_125464.1 Anaerobacterium chartisolvens strain T-1-35 16S ribosomal RNA, partial sequence | NR_125464.1 | 1451 | 344 | 745 | 27.71 | 486.8 | 3E-137 | 356/402 | 88 | 1/402 | 0 | Plus | Plus | Bacteria; Firmicutes; Clostridia; Eubacteriales; Oscillospiraceae; Anaerobacterium; Anaerobacterium chartisolvens | Anaerobacterium chartisolvens | Anaerobacterium | Oscillospiraceae | Eubacteriales | Clostridia | Firmicutes | Bacteria |
| ASV486 | 402 | 2 | 1 | 402 | 100 | NR_156080.1 Kineothrix alysoides strain KNHs209 16S ribosomal RNA, partial sequence | NR_156080.1 | 1410 | 353 | 754 | 28.51 | 693.6 | 0 | 393/402 | 97 | 0/402 | 0 | Plus | Plus | Bacteria; Firmicutes; Clostridia; Eubacteriales; Lachnospiraceae; Kineothrix; Kineothrix alysoides | Kineothrix alysoides | Kineothrix | Lachnospiraceae | Eubacteriales | Clostridia | Firmicutes | Bacteria |
| ASV487 | 402 | 1 | 1 | 402 | 100 | NR_116747.1 Ruminococcus faecis JCM 15917 strain Eg2 16S ribosomal RNA, partial sequence | NR_116747.1 | 1404 | 318 | 719 | 28.63 | 743.5 | 0 | 402/402 | 100 | 0/402 | 0 | Plus | Plus | Bacteria; Firmicutes; Clostridia; Eubacteriales; Lachnospiraceae; Mediterraneibacter; Mediterraneibacter faecis | Mediterraneibacter faecis | Mediterraneibacter | Lachnospiraceae | Eubacteriales | Clostridia | Firmicutes | Bacteria |
| ASV488 | 402 | 2 | 1 | 402 | 100 | NR_036869.2 Syntrophococcus sucromutans strain S195 16S ribosomal RNA, partial sequence | NR_036869.2 | 1507 | 359 | 760 | 26.68 | 649.3 | 0 | 385/402 | 95 | 0/402 | 0 | Plus | Plus | Bacteria; Firmicutes; Clostridia; Eubacteriales; Lachnospiraceae; Syntrophococcus; Syntrophococcus sucromutans | Syntrophococcus sucromutans | Syntrophococcus | Lachnospiraceae | Eubacteriales | Clostridia | Firmicutes | Bacteria |
| ASV489 | 422 | 1 | 1 | 422 | 100 | NR_113411.1 Prevotella copri DSM 18205 strain JCM 13464 16S ribosomal RNA, partial sequence | NR_113411.1 | 1493 | 358 | 779 | 28.27 | 774.9 | 0 | 421/422 | 99 | 0/422 | 0 | Plus | Plus | Bacteria; Bacteroidetes; Bacteroidia; Bacteroidales; Prevotellaceae; Prevotella; Prevotella copri | Prevotella copri | Prevotella | Prevotellaceae | Bacteroidales | Bacteroidia | Bacteroidetes | Bacteria |
| ASV491 | 405 | 1 | 1 | 405 | 100 | NR_044972.1 Sporobacter termitidis strain SYR 16S ribosomal RNA, partial sequence | NR_044972.1 | 1506 | 348 | 752 | 26.89 | 603.1 | 3E-172 | 378/405 | 93 | 0/405 | 0 | Plus | Plus | Bacteria; Firmicutes; Clostridia; Eubacteriales; Oscillospiraceae; Sporobacter; Sporobacter termitidis | Sporobacter termitidis | Sporobacter | Oscillospiraceae | Eubacteriales | Clostridia | Firmicutes | Bacteria |
| ASV492 | 427 | 1 | 1 | 427 | 100 | NR_042797.1 Treponema berlinense strain 7CPL208 16S ribosomal RNA, partial sequence | NR_042797.1 | 1440 | 275 | 701 | 29.65 | 701 | 0 | 411/427 | 96 | 0/427 | 0 | Plus | Plus | Bacteria; Spirochaetes; Spirochaetia; Spirochaetales; Spirochaetaceae; Treponema; Treponema berlinense | Treponema berlinense | Treponema | Spirochaetaceae | Spirochaetales | Spirochaetia | Spirochaetes | Bacteria |
| ASV493 | 406 | 1 | 1 | 406 | 100 | NR_144742.1 Christensenella massiliensis strain Marseille-P2438 16S ribosomal RNA, partial sequence | NR_144742.1 | 1499 | 369 | 773 | 27.02 | 533 | 4E-151 | 367/406 | 90 | 1/406 | 0 | Plus | Plus | Bacteria; Firmicutes; Clostridia; Eubacteriales; Christensenellaceae; Christensenella; Christensenella massiliensis | Christensenella massiliensis | Christensenella | Christensenellaceae | Eubacteriales | Clostridia | Firmicutes | Bacteria |
| ASV494 | 401 | 1 | 1 | 401 | 100 | NR_144749.1 Ihubacter massiliensis strain Marseille-P2843 16S ribosomal RNA, partial sequence | NR_144749.1 | 1479 | 364 | 766 | 27.25 | 599.4 | 4E-171 | 377/403 | 93 | 2/403 | 0 | Plus | Plus | Bacteria; Firmicutes; Clostridia; Eubacteriales; Clostridiales Family XIII. Incertae Sedis; Ihubacter; Ihubacter massiliensis | Ihubacter massiliensis | Ihubacter | Clostridiales Family XIII. Incertae Sedis | Eubacteriales | Clostridia | Firmicutes | Bacteria |
| ASV495 | 422 | 2 | 1 | 422 | 100 | NR_026242.1 Bacteroides caccae strain ATCC 43185 16S ribosomal RNA, partial sequence | NR_026242.1 | 1422 | 335 | 756 | 29.68 | 531.1 | 2E-150 | 382/427 | 89 | 10/427 | 2 | Plus | Plus | Bacteria; Bacteroidetes; Bacteroidia; Bacteroidales; Bacteroidaceae; Bacteroides; Bacteroides caccae | Bacteroides caccae | Bacteroides | Bacteroidaceae | Bacteroidales | Bacteroidia | Bacteroidetes | Bacteria |
| ASV496 | 408 | 5 | 1 | 408 | 100 | NR_134781.1 Olsenella scatoligenes strain SK9K4 16S ribosomal RNA, partial sequence | NR_134781.1 | 1469 | 348 | 755 | 27.77 | 632.7 | 0 | 386/408 | 94 | 0/408 | 0 | Plus | Plus | Bacteria; Actinobacteria; Coriobacteriia; Coriobacteriales; Atopobiaceae; Olsenella; Olsenella scatoligenes | Olsenella scatoligenes | Olsenella | Atopobiaceae | Coriobacteriales | Coriobacteriia | Actinobacteria | Bacteria |
| ASV497 | 404 | 1 | 1 | 404 | 100 | NR_025930.1 Ruminococcus bromii strain ATCC 27255 16S ribosomal RNA, partial sequence | NR_025930.1 | 1380 | 313 | 716 | 29.28 | 636.4 | 0 | 385/405 | 95 | 2/405 | 0 | Plus | Plus | Bacteria; Firmicutes; Clostridia; Eubacteriales; Oscillospiraceae; Ruminococcus; Ruminococcus bromii | Ruminococcus bromii | Ruminococcus | Oscillospiraceae | Eubacteriales | Clostridia | Firmicutes | Bacteria |
| ASV498 | 422 | 1 | 1 | 422 | 100 | NR_113411.1 Prevotella copri DSM 18205 strain JCM 13464 16S ribosomal RNA, partial sequence | NR_113411.1 | 1493 | 358 | 779 | 28.27 | 597.6 | 2E-170 | 389/422 | 92 | 0/422 | 0 | Plus | Plus | Bacteria; Bacteroidetes; Bacteroidia; Bacteroidales; Prevotellaceae; Prevotella; Prevotella copri | Prevotella copri | Prevotella | Prevotellaceae | Bacteroidales | Bacteroidia | Bacteroidetes | Bacteria |
| ASV499 | 403 | 2 | 3 | 403 | 99.5 | NR_148837.1 Abyssivirga alkaniphila strain L81 16S ribosomal RNA, partial sequence | NR_148837.1 | 1520 | 365 | 765 | 26.38 | 448 | 2E-125 | 351/404 | 86 | 6/404 | 1 | Plus | Plus | Bacteria; Firmicutes; Clostridia; Eubacteriales; Lachnospiraceae; Abyssivirga; Abyssivirga alkaniphila | Abyssivirga alkaniphila | Abyssivirga | Lachnospiraceae | Eubacteriales | Clostridia | Firmicutes | Bacteria |
| ASV500 | 404 | 1 | 1 | 404 | 100 | NR_118156.1 Oscillibacter ruminantium GH1 16S ribosomal RNA, partial sequence | NR_118156.1 | 1409 | 321 | 724 | 28.67 | 608.7 | 7E-174 | 380/405 | 93 | 2/405 | 0 | Plus | Plus | Bacteria; Firmicutes; Clostridia; Eubacteriales; Oscillospiraceae; Oscillibacter; Oscillibacter ruminantium | Oscillibacter ruminantium | Oscillibacter | Oscillospiraceae | Eubacteriales | Clostridia | Firmicutes | Bacteria |
| ASV501 | 427 | 1 | 1 | 426 | 99.77 | NR_027596.1 Mitsuokella multacida strain DSM 20544 16S ribosomal RNA, partial sequence | NR_027596.1 | 1511 | 346 | 771 | 28.19 | 673.3 | 0 | 405/426 | 95 | 0/426 | 0 | Plus | Plus | Bacteria; Firmicutes; Negativicutes; Selenomonadales; Selenomonadaceae; Mitsuokella; Mitsuokella multacida | Mitsuokella multacida | Mitsuokella | Selenomonadaceae | Selenomonadales | Negativicutes | Firmicutes | Bacteria |
| ASV502 | 402 | 3 | 1 | 402 | 100 | NR_156081.1 Kineothrix alysoides strain KNHs209 16S ribosomal RNA, partial sequence | NR_156081.1 | 1411 | 353 | 754 | 28.49 | 677 | 0 | 390/402 | 97 | 0/402 | 0 | Plus | Plus | Bacteria; Firmicutes; Clostridia; Eubacteriales; Lachnospiraceae; Kineothrix; Kineothrix alysoides | Kineothrix alysoides | Kineothrix | Lachnospiraceae | Eubacteriales | Clostridia | Firmicutes | Bacteria |
| ASV503 | 427 | 1 | 1 | 427 | 100 | NR_024994.1 Lactobacillus mucosae strain S32 16S ribosomal RNA, partial sequence | NR_024994.1 | 1568 | 384 | 810 | 27.23 | 784.1 | 0 | 426/427 | 99 | 0/427 | 0 | Plus | Plus | Bacteria; Firmicutes; Bacilli; Lactobacillales; Lactobacillaceae; Limosilactobacillus; Limosilactobacillus mucosae | Limosilactobacillus mucosae | Limosilactobacillus | Lactobacillaceae | Lactobacillales | Bacilli | Firmicutes | Bacteria |
| ASV504 | 421 | 2 | 1 | 421 | 100 | NR_074577.1 Paludibacter propionicigenes WB4 16S ribosomal RNA, partial sequence | NR_074577.1 | 1516 | 353 | 774 | 27.84 | 484.9 | 1E-136 | 370/423 | 87 | 3/423 | 0 | Plus | Plus | Bacteria; Bacteroidetes; Bacteroidia; Bacteroidales; Paludibacteraceae; Paludibacter; Paludibacter propionicigenes | Paludibacter propionicigenes | Paludibacter | Paludibacteraceae | Bacteroidales | Bacteroidia | Bacteroidetes | Bacteria |
| ASV505 | 421 | 1 | 1 | 421 | 100 | NR_144616.1 Muribaculum intestinale strain YL27 16S ribosomal RNA, partial sequence | NR_144616.1 | 1460 | 297 | 718 | 28.9 | 484.9 | 1E-136 | 372/425 | 87 | 7/425 | 1 | Plus | Plus | Bacteria; Bacteroidetes; Bacteroidia; Bacteroidales; Muribaculaceae; Muribaculum; Muribaculum intestinale | Muribaculum intestinale | Muribaculum | Muribaculaceae | Bacteroidales | Bacteroidia | Bacteroidetes | Bacteria |
| ASV506 | 421 | 1 | 1 | 421 | 100 | NR_144745.1 Culturomica massiliensis strain Marseille-P2698 16S ribosomal RNA, partial sequence | NR_144745.1 | 1492 | 356 | 776 | 28.22 | 761.9 | 0 | 418/421 | 99 | 0/421 | 0 | Plus | Plus | Bacteria; Bacteroidetes; Bacteroidia; Bacteroidales; Odoribacteraceae; Culturomica; Culturomica massiliensis | Culturomica massiliensis | Culturomica | Odoribacteraceae | Bacteroidales | Bacteroidia | Bacteroidetes | Bacteria |
| ASV507 | 427 | 2 | 1 | 427 | 100 | NR_075036.1 Lactobacillus reuteri DSM 20016 16S ribosomal RNA, partial sequence | NR_075036.1 | 1569 | 384 | 810 | 27.21 | 784.1 | 0 | 426/427 | 99 | 0/427 | 0 | Plus | Plus | Bacteria; Firmicutes; Bacilli; Lactobacillales; Lactobacillaceae; Limosilactobacillus; Limosilactobacillus reuteri | Limosilactobacillus reuteri | Limosilactobacillus | Lactobacillaceae | Lactobacillales | Bacilli | Firmicutes | Bacteria |
| ASV509 | 427 | 1 | 1 | 427 | 100 | NR_042942.1 Treponema porcinum strain 14V28 16S ribosomal RNA, partial sequence | NR_042942.1 | 1426 | 258 | 684 | 29.94 | 784.1 | 0 | 426/427 | 99 | 0/427 | 0 | Plus | Plus | Bacteria; Spirochaetes; Spirochaetia; Spirochaetales; Spirochaetaceae; Treponema; Treponema porcinum | Treponema porcinum | Treponema | Spirochaetaceae | Spirochaetales | Spirochaetia | Spirochaetes | Bacteria |
| ASV510 | 402 | 2 | 1 | 402 | 100 | NR_042144.1 Clostridium butyricum strain VPI3266 16S ribosomal RNA, partial sequence | NR_042144.1 | 1425 | 338 | 739 | 28.21 | 732.4 | 0 | 400/402 | 99 | 0/402 | 0 | Plus | Plus | Bacteria; Firmicutes; Clostridia; Eubacteriales; Clostridiaceae; Clostridium; Clostridium butyricum | Clostridium butyricum | Clostridium | Clostridiaceae | Eubacteriales | Clostridia | Firmicutes | Bacteria |
| ASV511 | 401 | 2 | 1 | 401 | 100 | NR_119074.1 Terrisporobacter glycolicus ATCC 14880 = DSM 1288 16S ribosomal RNA, partial sequence | NR_119074.1 | 1461 | 314 | 714 | 27.45 | 713.9 | 0 | 396/401 | 98 | 0/401 | 0 | Plus | Plus | Bacteria; Firmicutes; Clostridia; Eubacteriales; Peptostreptococcaceae; Terrisporobacter; Terrisporobacter glycolicus | Terrisporobacter glycolicus | Terrisporobacter | Peptostreptococcaceae | Eubacteriales | Clostridia | Firmicutes | Bacteria |
| ASV512 | 427 | 1 | 1 | 427 | 100 | NR_041778.1 Desulfovibrio piger strain ATCC 29098 16S ribosomal RNA, partial sequence | NR_041778.1 | 1542 | 357 | 783 | 27.69 | 667.8 | 0 | 405/427 | 94 | 0/427 | 0 | Plus | Plus | Bacteria; Proteobacteria; Deltaproteobacteria; Desulfovibrionales; Desulfovibrionaceae; Desulfovibrio; Desulfovibrio piger | Desulfovibrio piger | Desulfovibrio | Desulfovibrionaceae | Desulfovibrionales | Deltaproteobacteria | Proteobacteria | Bacteria |
| ASV513 | 422 | 1 | 1 | 422 | 100 | NR_041342.1 Parabacteroides distasonis strain JCM 5825 16S ribosomal RNA, partial sequence | NR_041342.1 | 1488 | 353 | 774 | 28.36 | 558.8 | 8E-159 | 382/422 | 90 | 0/422 | 0 | Plus | Plus | Bacteria; Bacteroidetes; Bacteroidia; Bacteroidales; Tannerellaceae; Parabacteroides; Parabacteroides distasonis | Parabacteroides distasonis | Parabacteroides | Tannerellaceae | Bacteroidales | Bacteroidia | Bacteroidetes | Bacteria |
| ASV514 | 402 | 1 | 1 | 402 | 100 | NR_024661.1 Eubacterium ruminantium strain GA195 16S ribosomal RNA, partial sequence | NR_024661.1 | 1508 | 378 | 779 | 26.66 | 688.1 | 0 | 392/402 | 97 | 0/402 | 0 | Plus | Plus | Bacteria; Firmicutes; Clostridia; Eubacteriales; Eubacteriaceae; Eubacterium; Eubacterium ruminantium | Eubacterium ruminantium | Eubacterium | Eubacteriaceae | Eubacteriales | Clostridia | Firmicutes | Bacteria |
| ASV515 | 428 | 1 | 1 | 428 | 100 | NR_104781.1 Treponema bryantii strain RUS-1 16S ribosomal RNA, partial sequence | NR_104781.1 | 1535 | 351 | 778 | 27.88 | 653 | 0 | 404/429 | 94 | 2/429 | 0 | Plus | Plus | Bacteria; Spirochaetes; Spirochaetia; Spirochaetales; Spirochaetaceae; Treponema; Treponema bryantii | Treponema bryantii | Treponema | Spirochaetaceae | Spirochaetales | Spirochaetia | Spirochaetes | Bacteria |
| ASV516 | 402 | 2 | 1 | 402 | 100 | NR_125571.1 Oribacterium asaccharolyticum ACB7 16S ribosomal RNA, partial sequence | NR_125571.1 | 1436 | 332 | 733 | 27.99 | 599.4 | 4E-171 | 378/404 | 93 | 4/404 | 0 | Plus | Plus | Bacteria; Firmicutes; Clostridia; Eubacteriales; Lachnospiraceae; Oribacterium; Oribacterium asaccharolyticum | Oribacterium asaccharolyticum | Oribacterium | Lachnospiraceae | Eubacteriales | Clostridia | Firmicutes | Bacteria |
| ASV517 | 421 | 2 | 1 | 421 | 100 | NR_113077.1 Paraprevotella clara strain JCM 14859 16S ribosomal RNA, partial sequence | NR_113077.1 | 1480 | 346 | 766 | 28.45 | 473.9 | 3E-133 | 368/423 | 86 | 4/423 | 0 | Plus | Plus | Bacteria; Bacteroidetes; Bacteroidia; Bacteroidales; Prevotellaceae; Paraprevotella; Paraprevotella clara | Paraprevotella clara | Paraprevotella | Prevotellaceae | Bacteroidales | Bacteroidia | Bacteroidetes | Bacteria |
| ASV518 | 422 | 2 | 1 | 422 | 100 | NR_113117.1 Prevotella oralis ATCC 33269 strain JCM 12251 16S ribosomal RNA, partial sequence | NR_113117.1 | 1496 | 361 | 782 | 28.21 | 603.1 | 3E-172 | 391/423 | 92 | 2/423 | 0 | Plus | Plus | Bacteria; Bacteroidetes; Bacteroidia; Bacteroidales; Prevotellaceae; Prevotella; Prevotella oralis | Prevotella oralis | Prevotella | Prevotellaceae | Bacteroidales | Bacteroidia | Bacteroidetes | Bacteria |
| ASV519 | 422 | 1 | 1 | 422 | 100 | NR_041364.1 Prevotella stercorea DSM 18206 strain CB35 16S ribosomal RNA, partial sequence | NR_041364.1 | 1488 | 354 | 775 | 28.36 | 708.4 | 0 | 409/422 | 96 | 0/422 | 0 | Plus | Plus | Bacteria; Bacteroidetes; Bacteroidia; Bacteroidales; Prevotellaceae; Prevotella; Prevotella stercorea | Prevotella stercorea | Prevotella | Prevotellaceae | Bacteroidales | Bacteroidia | Bacteroidetes | Bacteria |
| ASV520 | 405 | 5 | 1 | 405 | 100 | NR_115693.1 Gracilibacter thermotolerans JW/YJL-S1 16S ribosomal RNA, partial sequence | NR_115693.1 | 1526 | 459 | 861 | 26.41 | 464.6 | 2E-130 | 356/407 | 87 | 6/407 | 1 | Plus | Plus | Bacteria; Firmicutes; Clostridia; Eubacteriales; Gracilibacteraceae; Gracilibacter; Gracilibacter thermotolerans | Gracilibacter thermotolerans | Gracilibacter | Gracilibacteraceae | Eubacteriales | Clostridia | Firmicutes | Bacteria |
| ASV521 | 422 | 1 | 1 | 422 | 100 | NR_144616.1 Muribaculum intestinale strain YL27 16S ribosomal RNA, partial sequence | NR_144616.1 | 1460 | 297 | 718 | 28.9 | 536.6 | 4E-152 | 382/426 | 89 | 8/426 | 1 | Plus | Plus | Bacteria; Bacteroidetes; Bacteroidia; Bacteroidales; Muribaculaceae; Muribaculum; Muribaculum intestinale | Muribaculum intestinale | Muribaculum | Muribaculaceae | Bacteroidales | Bacteroidia | Bacteroidetes | Bacteria |

| | | | | | | | | | | | | | | | | | | | | | | | | | | | |
|---|---|---|---|---|---|---|---|---|---|---|---|---|---|---|---|---|---|---|---|---|---|---|---|---|---|---|---|
| ASV522 | 403 | 2 | 1 | 403 | 100 | NR_027557.1 Roseburia intestinalis L1-82 16S ribosomal RNA, partial sequence | NR_027557.1 | 1482 | 360 | 763 | 27.26 | 739.8 | 0 | 403/404 | 99 | 1/404 | 0 | Plus | Plus | Bacteria; Firmicutes; Clostridia; Eubacteriales; Lachnospiraceae; Roseburia; Roseburia intestinalis | Roseburia intestinalis | Roseburia | Lachnospiraceae | Eubacteriales | Clostridia | Firmicutes | Bacteria |
| ASV523 | 426 | 1 | 1 | 425 | 99.77 | NR_028840.1 Mitsuokella jalaludinii strain M9 16S ribosomal RNA, partial sequence | NR_028840.1 | 1543 | 362 | 787 | 27.61 | 653 | 0 | 403/427 | 94 | 3/427 | 0 | Plus | Plus | Bacteria; Firmicutes; Negativicutes; Selenomonadales; Selenomonadaceae; Mitsuokella; Mitsuokella jalaludinii | Mitsuokella jalaludinii | Mitsuokella | Selenomonadaceae | Selenomonadales | Negativicutes | Firmicutes | Bacteria |
| ASV524 | 422 | 3 | 1 | 422 | 100 | NR_113119.1 Prevotella oris strain JCM 8540 16S ribosomal RNA, partial sequence | NR_113119.1 | 1481 | 345 | 766 | 28.49 | 575.4 | 7E-164 | 386/423 | 91 | 2/423 | 0 | Plus | Plus | Bacteria; Bacteroidetes; Bacteroidia; Bacteroidales; Prevotellaceae; Prevotella; Prevotella oris | Prevotella oris | Prevotella | Prevotellaceae | Bacteroidales | Bacteroidia | Bacteroidetes | Bacteria |
| ASV525 | 402 | 2 | 1 | 402 | 100 | NR_170495.1 Blautia faecicola strain KGMB01111 16S ribosomal RNA, complete sequence | NR_170495.1 | 1534 | 372 | 773 | 26.21 | 654.8 | 0 | 386/402 | 96 | 0/402 | 0 | Plus | Plus | Bacteria; Firmicutes; Clostridia; Eubacteriales; Lachnospiraceae; Blautia; Blautia faecicola | Blautia faecicola | Blautia | Lachnospiraceae | Eubacteriales | Clostridia | Firmicutes | Bacteria |
| ASV526 | 405 | 1 | 1 | 405 | 100 | NR_042832.1 Roseburia faecis strain M72/1 16S ribosomal RNA, partial sequence | NR_042832.1 | 1456 | 347 | 749 | 27.68 | 686.2 | 0 | 394/405 | 97 | 2/405 | 0 | Plus | Plus | Bacteria; Firmicutes; Clostridia; Eubacteriales; Lachnospiraceae; Roseburia; Roseburia faecis | Roseburia faecis | Roseburia | Lachnospiraceae | Eubacteriales | Clostridia | Firmicutes | Bacteria |
| ASV527 | 427 | 1 | 1 | 426 | 99.77 | NR_027596.1 Mitsuokella multacida strain DSM 20544 16S ribosomal RNA, partial sequence | NR_027596.1 | 1511 | 346 | 771 | 28.19 | 656.7 | 0 | 402/426 | 94 | 0/426 | 0 | Plus | Plus | Bacteria; Firmicutes; Negativicutes; Selenomonadales; Selenomonadaceae; Mitsuokella; Mitsuokella multacida | Mitsuokella multacida | Mitsuokella | Selenomonadaceae | Selenomonadales | Negativicutes | Firmicutes | Bacteria |
| ASV528 | 402 | 1 | 1 | 402 | 100 | NR_169458.1 Tepidibaculum saccharolyticum strain STR9 16S ribosomal RNA, partial sequence | NR_169458.1 | 1477 | 362 | 763 | 27.22 | 610.5 | 2E-174 | 380/404 | 94 | 4/404 | 0 | Plus | Plus | Bacteria; Firmicutes; Clostridia; Eubacteriales; Oscillospiraceae; Tepidibaculum; Tepidibaculum saccharolyticum | Tepidibaculum saccharolyticum | Tepidibaculum | Oscillospiraceae | Eubacteriales | Clostridia | Firmicutes | Bacteria |
| ASV529 | 427 | 4 | 1 | 427 | 100 | NR_042802.1 Lactobacillus ultunensis strain Kx146C1 16S ribosomal RNA, partial sequence | NR_042802.1 | 1550 | 370 | 796 | 27.55 | 784.1 | 0 | 426/427 | 99 | 0/427 | 0 | Plus | Plus | Bacteria; Firmicutes; Bacilli; Lactobacillales; Lactobacillaceae; Lactobacillus; Lactobacillus ultunensis | Lactobacillus ultunensis | Lactobacillus | Lactobacillaceae | Lactobacillales | Bacilli | Firmicutes | Bacteria |
| ASV530 | 401 | 1 | 1 | 401 | 100 | NR_144749.1 Ihubacter massiliensis strain Marseille-P2843 16S ribosomal RNA, partial sequence | NR_144749.1 | 1479 | 364 | 766 | 27.25 | 582.8 | 4E-166 | 374/403 | 92 | 2/403 | 0 | Plus | Plus | Bacteria; Firmicutes; Clostridia; Eubacteriales; Clostridiales Family XIII. Incertae Sedis; Ihubacter; Ihubacter massiliensis | Ihubacter massiliensis | Ihubacter | Clostridiales Family XIII. Incertae Sedis | Eubacteriales | Clostridia | Firmicutes | Bacteria |
| ASV531 | 402 | 1 | 1 | 402 | 100 | NR_028883.1 Dorea longicatena strain 111-35 16S ribosomal RNA, partial sequence | NR_028883.1 | 1316 | 243 | 645 | 30.62 | 732.4 | 0 | 401/403 | 99 | 1/403 | 0 | Plus | Plus | Bacteria; Firmicutes; Clostridia; Eubacteriales; Lachnospiraceae; Dorea; Dorea longicatena | Dorea longicatena | Dorea | Lachnospiraceae | Eubacteriales | Clostridia | Firmicutes | Bacteria |
| ASV532 | 402 | 2 | 1 | 402 | 100 | NR_144696.1 Clostridium saudiense strain JCC 16S ribosomal RNA, partial sequence | NR_144696.1 | 1478 | 350 | 751 | 27.2 | 710.2 | 0 | 396/402 | 98 | 0/402 | 0 | Plus | Plus | Bacteria; Firmicutes; Clostridia; Eubacteriales; Clostridiaceae; Clostridium; Clostridium saudiense | Clostridium saudiense | Clostridium | Clostridiaceae | Eubacteriales | Clostridia | Firmicutes | Bacteria |
| ASV533 | 427 | 1 | 1 | 427 | 100 | NR_044111.1 Selenomonas bovis strain WG 16S ribosomal RNA, partial sequence | NR_044111.1 | 1525 | 370 | 796 | 28 | 678.8 | 0 | 407/427 | 95 | 0/427 | 0 | Plus | Plus | Bacteria; Firmicutes; Negativicutes; Selenomonadales; Selenomonadaceae; Selenomonas; Selenomonas bovis | Selenomonas bovis | Selenomonas | Selenomonadaceae | Selenomonadales | Negativicutes | Firmicutes | Bacteria |
| ASV534 | 402 | 1 | 1 | 402 | 100 | NR_119068.1 [Clostridium] aerotolerans strain DSM 5434 16S ribosomal RNA, partial sequence | NR_119068.1 | 1470 | 332 | 732 | 27.28 | 680.7 | 0 | 391/402 | 97 | 1/402 | 0 | Plus | Plus | Bacteria; Firmicutes; Clostridia; Eubacteriales; Lachnospiraceae; Lacrimispora; Lacrimispora aerotolerans | Lacrimispora aerotolerans | Lacrimispora | Lachnospiraceae | Eubacteriales | Clostridia | Firmicutes | Bacteria |
| ASV535 | 402 | 1 | 1 | 402 | 100 | NR_074613.1 [Eubacterium] eligens ATCC 27750 16S ribosomal RNA, partial sequence | NR_074613.1 | 1519 | 367 | 768 | 26.46 | 627.1 | 2E-179 | 381/402 | 94 | 0/402 | 0 | Plus | Plus | Bacteria; Firmicutes; Clostridia; Eubacteriales; Lachnospiraceae; Lachnospira; Lachnospira eligens | Lachnospira eligens | Lachnospira | Lachnospiraceae | Eubacteriales | Clostridia | Firmicutes | Bacteria |
| ASV536 | 402 | 1 | 1 | 402 | 100 | NR_028961.1 Faecalibacterium prausnitzii strain ATCC 27768 16S ribosomal RNA, partial sequence | NR_028961.1 | 1462 | 344 | 745 | 27.5 | 726.9 | 0 | 399/402 | 99 | 0/402 | 0 | Plus | Plus | Bacteria; Firmicutes; Clostridia; Eubacteriales; Oscillospiraceae; Faecalibacterium; Faecalibacterium prausnitzii | Faecalibacterium prausnitzii | Faecalibacterium | Oscillospiraceae | Eubacteriales | Clostridia | Firmicutes | Bacteria |
| ASV537 | 402 | 1 | 1 | 402 | 100 | NR_102884.1 Ruminococcus champanellensis 18P13 = JCM 17042 16S ribosomal RNA, partial sequence | NR_102884.1 | 1494 | 348 | 749 | 26.91 | 726.9 | 0 | 399/402 | 99 | 0/402 | 0 | Plus | Plus | Bacteria; Firmicutes; Clostridia; Eubacteriales; Oscillospiraceae; Ruminococcus; Ruminococcus champanellensis | Ruminococcus champanellensis | Ruminococcus | Oscillospiraceae | Eubacteriales | Clostridia | Firmicutes | Bacteria |
| ASV538 | 405 | 1 | 1 | 405 | 100 | NR_044972.1 Sporobacter termitidis strain SYR 16S ribosomal RNA, partial sequence | NR_044972.1 | 1506 | 348 | 752 | 26.89 | 542.2 | 7E-154 | 368/406 | 90 | 2/406 | 0 | Plus | Plus | Bacteria; Firmicutes; Clostridia; Eubacteriales; Oscillospiraceae; Sporobacter; Sporobacter termitidis | Sporobacter termitidis | Sporobacter | Oscillospiraceae | Eubacteriales | Clostridia | Firmicutes | Bacteria |
| ASV539 | 427 | 1 | 1 | 427 | 100 | NR_042797.1 Treponema berlinense strain 7CPL208 16S ribosomal RNA, partial sequence | NR_042797.1 | 1440 | 275 | 701 | 29.65 | 573.6 | 3E-163 | 388/427 | 90 | 0/427 | 0 | Plus | Plus | Bacteria; Spirochaetes; Spirochaetia; Spirochaetales; Spirochaetaceae; Treponema; Treponema berlinense | Treponema berlinense | Treponema | Spirochaetaceae | Spirochaetales | Spirochaetia | Spirochaetes | Bacteria |
| ASV540 | 401 | 1 | 1 | 401 | 100 | NR_144737.1 Emergencia timonensis strain SN18 16S ribosomal RNA, partial sequence | NR_144737.1 | 1480 | 364 | 767 | 27.3 | 621.6 | 9E-178 | 382/404 | 94 | 3/404 | 0 | Plus | Plus | Bacteria; Firmicutes; Clostridia; Eubacteriales; Clostridiales Family XIII. Incertae Sedis; Emergencia; Emergencia timonensis | Emergencia timonensis | Emergencia | Clostridiales Family XIII. Incertae Sedis | Eubacteriales | Clostridia | Firmicutes | Bacteria |
| ASV541 | 427 | 1 | 1 | 427 | 100 | NR_102980.1 Megasphaera elsdenii DSM 20460 16S ribosomal RNA, partial sequence | NR_102980.1 | 1552 | 369 | 795 | 27.51 | 784.1 | 0 | 426/427 | 99 | 0/427 | 0 | Plus | Plus | Bacteria; Firmicutes; Negativicutes; Veillonellales; Veillonellaceae; Megasphaera; Megasphaera elsdenii | Megasphaera elsdenii | Megasphaera | Veillonellaceae | Veillonellales | Negativicutes | Firmicutes | Bacteria |
| ASV542 | 421 | 1 | 1 | 421 | 100 | NR_144746.1 Marseilla massiliensis strain Marseille-P2475 16S ribosomal RNA, partial sequence | NR_144746.1 | 1494 | 356 | 777 | 28.25 | 470.2 | 4E-132 | 372/428 | 86 | 13/428 | 3 | Plus | Plus | Bacteria; Bacteroidetes; Bacteroidia; Bacteroidales; Prevotellaceae; Marseilla; Marseilla massiliensis | Marseilla massiliensis | Marseilla | Prevotellaceae | Bacteroidales | Bacteroidia | Bacteroidetes | Bacteria |
| ASV543 | 405 | 1 | 1 | 405 | 100 | NR_144742.1 Christensenella massiliensis strain Marseille-P2438 16S ribosomal RNA, partial sequence | NR_144742.1 | 1499 | 369 | 773 | 27.02 | 472 | 1E-132 | 355/405 | 87 | 0/405 | 0 | Plus | Plus | Bacteria; Firmicutes; Clostridia; Eubacteriales; Christensenellaceae; Christensenella; Christensenella massiliensis | Christensenella massiliensis | Christensenella | Christensenellaceae | Eubacteriales | Clostridia | Firmicutes | Bacteria |
| ASV544 | 426 | 1 | 1 | 426 | 100 | NR_044111.1 Selenomonas bovis strain WG 16S ribosomal RNA, partial sequence | NR_044111.1 | 1525 | 370 | 796 | 28 | 665.9 | 0 | 406/428 | 94 | 3/428 | 0 | Plus | Plus | Bacteria; Firmicutes; Negativicutes; Selenomonadales; Selenomonadaceae; Selenomonas; Selenomonas bovis | Selenomonas bovis | Selenomonas | Selenomonadaceae | Selenomonadales | Negativicutes | Firmicutes | Bacteria |
| ASV545 | 403 | 1 | 1 | 403 | 100 | NR_025930.1 Ruminococcus bromii strain ATCC 27255 16S ribosomal RNA, partial sequence | NR_025930.1 | 1380 | 313 | 716 | 29.28 | 619.7 | 3E-177 | 381/404 | 94 | 1/404 | 0 | Plus | Plus | Bacteria; Firmicutes; Clostridia; Eubacteriales; Oscillospiraceae; Ruminococcus; Ruminococcus bromii | Ruminococcus bromii | Ruminococcus | Oscillospiraceae | Eubacteriales | Clostridia | Firmicutes | Bacteria |
| ASV546 | 420 | 1 | 1 | 420 | 100 | NR_170509.1 Duncaniella freteri strain TLL-A3 16S ribosomal RNA, partial sequence | NR_170509.1 | 1538 | 364 | 784 | 27.37 | 449.9 | 5E-126 | 366/425 | 86 | 9/425 | 2 | Plus | Plus | Bacteria; Bacteroidetes; Bacteroidia; Bacteroidales; Muribaculaceae; Duncaniella; Duncaniella freteri | Duncaniella freteri | Duncaniella | Muribaculaceae | Bacteroidales | Bacteroidia | Bacteroidetes | Bacteria |
| ASV547 | 427 | 1 | 1 | 427 | 100 | NR_102980.1 Megasphaera elsdenii DSM 20460 16S ribosomal RNA, partial sequence | NR_102980.1 | 1552 | 369 | 795 | 27.51 | 756.4 | 0 | 421/427 | 98 | 0/427 | 0 | Plus | Plus | Bacteria; Firmicutes; Negativicutes; Veillonellales; Veillonellaceae; Megasphaera; Megasphaera elsdenii | Megasphaera elsdenii | Megasphaera | Veillonellaceae | Veillonellales | Negativicutes | Firmicutes | Bacteria |
| ASV548 | 404 | 1 | 1 | 404 | 100 | NR_025930.1 Ruminococcus bromii strain ATCC 27255 16S ribosomal RNA, partial sequence | NR_025930.1 | 1380 | 313 | 716 | 29.28 | 702.8 | 0 | 397/405 | 98 | 2/405 | 0 | Plus | Plus | Bacteria; Firmicutes; Clostridia; Eubacteriales; Oscillospiraceae; Ruminococcus; Ruminococcus bromii | Ruminococcus bromii | Ruminococcus | Oscillospiraceae | Eubacteriales | Clostridia | Firmicutes | Bacteria |
| ASV549 | 416 | 1 | 1 | 416 | 100 | NR_102893.1 Butyrivibrio proteoclasticus strain B316 16S ribosomal RNA, partial sequence | NR_102893.1 | 1537 | 367 | 782 | 27.07 | 614.2 | 2E-175 | 390/418 | 93 | 4/418 | 0 | Plus | Plus | Bacteria; Firmicutes; Clostridia; Eubacteriales; Lachnospiraceae; Butyrivibrio; Butyrivibrio proteoclasticus | Butyrivibrio proteoclasticus | Butyrivibrio | Lachnospiraceae | Eubacteriales | Clostridia | Firmicutes | Bacteria |
| ASV550 | 427 | 2 | 1 | 427 | 100 | NR_028797.1 Selenomonas infelix strain VPI D 75 B-30 16S ribosomal RNA, partial sequence | NR_028797.1 | 1524 | 373 | 799 | 28.02 | 601.3 | 1E-171 | 393/427 | 92 | 0/427 | 0 | Plus | Plus | Bacteria; Firmicutes; Negativicutes; Selenomonadales; Selenomonadaceae; Selenomonas; Selenomonas infelix | Selenomonas infelix | Selenomonas | Selenomonadaceae | Selenomonadales | Negativicutes | Firmicutes | Bacteria |
| ASV551 | 423 | 1 | 1 | 423 | 100 | NR_144750.1 Prevotellamassilia timonensis strain Marseille-P2831 16S ribosomal RNA, partial sequence | NR_144750.1 | 1493 | 358 | 779 | 28.27 | 564.3 | 2E-160 | 386/425 | 90 | 5/425 | 1 | Plus | Plus | Bacteria; Bacteroidetes; Bacteroidia; Bacteroidales; Prevotellaceae; Prevotellamassilia; Prevotellamassilia timonensis | Prevotellamassilia timonensis | Prevotellamassilia | Prevotellaceae | Bacteroidales | Bacteroidia | Bacteroidetes | Bacteria |
| ASV552 | 402 | 1 | 1 | 402 | 100 | NR_029160.1 Ruminococcus callidus strain ATCC 27760 16S ribosomal RNA, partial sequence | NR_029160.1 | 1371 | 310 | 711 | 29.32 | 737.9 | 0 | 401/402 | 99 | 0/402 | 0 | Plus | Plus | Bacteria; Firmicutes; Clostridia; Eubacteriales; Oscillospiraceae; Ruminococcus; Ruminococcus callidus | Ruminococcus callidus | Ruminococcus | Oscillospiraceae | Eubacteriales | Clostridia | Firmicutes | Bacteria |
| ASV553 | 429 | 1 | 1 | 429 | 100 | NR_025079.1 Desulfomonile limimaris strain DCB-M 16S ribosomal RNA, partial sequence | NR_025079.1 | 1523 | 363 | 790 | 28.1 | 436.9 | 4E-122 | 366/430 | 85 | 3/430 | 0 | Plus | Plus | Bacteria; Proteobacteria; Deltaproteobacteria; Syntrophobacterales; Syntrophaceae; Desulfomonile; Desulfomonile limimaris | Desulfomonile limimaris | Desulfomonile | Syntrophaceae | Syntrophobacterales | Deltaproteobacteria | Proteobacteria | Bacteria |
| ASV554 | 423 | 1 | 1 | 423 | 100 | NR_041954.1 Prevotella brevis strain GA33 16S ribosomal RNA, partial sequence | NR_041954.1 | 1493 | 356 | 777 | 28.27 | 555.1 | 1E-157 | 382/424 | 90 | 3/424 | 0 | Plus | Plus | Bacteria; Bacteroidetes; Bacteroidia; Bacteroidales; Prevotellaceae; Prevotella; Prevotella brevis | Prevotella brevis | Prevotella | Prevotellaceae | Bacteroidales | Bacteroidia | Bacteroidetes | Bacteria |
| ASV555 | 402 | 1 | 2 | 402 | 99.75 | NR_027579.1 Ruminococcus lactaris ATCC 29176 16S ribosomal RNA, partial sequence | NR_027579.1 | 1402 | 334 | 734 | 28.6 | 682.5 | 0 | 390/401 | 97 | 0/401 | 0 | Plus | Plus | Bacteria; Firmicutes; Clostridia; Eubacteriales; Lachnospiraceae; Mediterraneibacter; [Ruminococcus] lactaris | [Ruminococcus] lactaris | Mediterraneibacter | Lachnospiraceae | Eubacteriales | Clostridia | Firmicutes | Bacteria |
| ASV556 | 422 | 1 | 1 | 422 | 100 | NR_151886.1 Prevotella colorans strain A1336 16S ribosomal RNA, partial sequence | NR_151886.1 | 1491 | 358 | 779 | 28.3 | 619.7 | 3E-177 | 393/422 | 93 | 0/422 | 0 | Plus | Plus | Bacteria; Bacteroidetes; Bacteroidia; Bacteroidales; Prevotellaceae; Prevotella; Prevotella colorans | Prevotella colorans | Prevotella | Prevotellaceae | Bacteroidales | Bacteroidia | Bacteroidetes | Bacteria |
| ASV557 | 401 | 1 | 1 | 401 | 100 | NR_144749.1 Ihubacter massiliensis strain Marseille-P2843 16S ribosomal RNA, partial sequence | NR_144749.1 | 1479 | 364 | 766 | 27.25 | 582.8 | 4E-166 | 375/404 | 92 | 4/404 | 0 | Plus | Plus | Bacteria; Firmicutes; Clostridia; Eubacteriales; Clostridiales Family XIII. Incertae Sedis; Ihubacter; Ihubacter massiliensis | Ihubacter massiliensis | Ihubacter | Clostridiales Family XIII. Incertae Sedis | Eubacteriales | Clostridia | Firmicutes | Bacteria |
| ASV558 | 403 | 2 | 3 | 403 | 99.5 | NR_125677.1 Vallitalea pronyensis strain FatNI3 16S ribosomal RNA, partial sequence | NR_125677.1 | 1480 | 365 | 764 | 27.03 | 457.2 | 3E-128 | 353/404 | 87 | 7/404 | 1 | Plus | Plus | Bacteria; Firmicutes; Clostridia; Eubacteriales; Vallitaleaceae; Vallitalea; Vallitalea pronyensis | Vallitalea pronyensis | Vallitalea | Vallitaleaceae | Eubacteriales | Clostridia | Firmicutes | Bacteria |
| ASV559 | 405 | 1 | 1 | 405 | 100 | NR_044972.1 Sporobacter termitidis strain SYR 16S ribosomal RNA, partial sequence | NR_044972.1 | 1506 | 348 | 752 | 26.89 | 581 | 2E-165 | 374/405 | 92 | 0/405 | 0 | Plus | Plus | Bacteria; Firmicutes; Clostridia; Eubacteriales; Oscillospiraceae; Sporobacter; Sporobacter termitidis | Sporobacter termitidis | Sporobacter | Oscillospiraceae | Eubacteriales | Clostridia | Firmicutes | Bacteria |
| ASV560 | 422 | 1 | 1 | 422 | 100 | NR_024816.1 Prevotella salivae strain EPSA11 16S ribosomal RNA, partial sequence | NR_024816.1 | 1493 | 356 | 777 | 28.27 | 625.3 | 7E-179 | 394/422 | 93 | 0/422 | 0 | Plus | Plus | Bacteria; Bacteroidetes; Bacteroidia; Bacteroidales; Prevotellaceae; Prevotella; Prevotella salivae | Prevotella salivae | Prevotella | Prevotellaceae | Bacteroidales | Bacteroidia | Bacteroidetes | Bacteria |
| ASV561 | 403 | 1 | 3 | 403 | 99.5 | NR_125464.1 Anaerobacterium chartisolvens strain T-1-35 16S ribosomal RNA, partial sequence | NR_125464.1 | 1451 | 344 | 745 | 27.71 | 475.7 | 7E-134 | 356/404 | 88 | 5/404 | 1 | Plus | Plus | Bacteria; Firmicutes; Clostridia; Eubacteriales; Oscillospiraceae; Anaerobacterium; Anaerobacterium chartisolvens | Anaerobacterium chartisolvens | Anaerobacterium | Oscillospiraceae | Eubacteriales | Clostridia | Firmicutes | Bacteria |
| ASV562 | 406 | 1 | 1 | 406 | 100 | NR_115340.1 Saccharofermentans acetigenes strain P6 16S ribosomal RNA, partial sequence | NR_115340.1 | 1510 | 353 | 757 | 26.82 | 499.7 | 4E-141 | 362/407 | 88 | 3/407 | 0 | Plus | Plus | Bacteria; Firmicutes; Clostridia; Eubacteriales; Oscillospiraceae; Saccharofermentans; Saccharofermentans acetigenes | Saccharofermentans acetigenes | Saccharofermentans | Oscillospiraceae | Eubacteriales | Clostridia | Firmicutes | Bacteria |
| ASV563 | 403 | 1 | 1 | 403 | 100 | NR_042832.1 Roseburia faecis strain M72/1 16S ribosomal RNA, partial sequence | NR_042832.1 | 1456 | 347 | 749 | 27.68 | 717.6 | 0 | 398/403 | 98 | 0/403 | 0 | Plus | Plus | Bacteria; Firmicutes; Clostridia; Eubacteriales; Lachnospiraceae; Roseburia; Roseburia faecis | Roseburia faecis | Roseburia | Lachnospiraceae | Eubacteriales | Clostridia | Firmicutes | Bacteria |
| ASV564 | 402 | 2 | 1 | 402 | 100 | NR_104899.1 Bacteroides xylanolyticus strain X5-1 16S ribosomal RNA, partial sequence | NR_104899.1 | 1480 | 345 | 746 | 27.16 | 710.2 | 0 | 396/402 | 98 | 0/402 | 0 | Plus | Plus | Bacteria; Firmicutes; Clostridia; Eubacteriales; Clostridiaceae; Hungatella; Hungatella xylanolytica | Hungatella xylanolytica | Hungatella | Clostridiaceae | Eubacteriales | Clostridia | Firmicutes | Bacteria |
| ASV565 | 402 | 1 | 2 | 402 | 99.75 | NR_027579.1 Ruminococcus lactaris ATCC 29176 16S ribosomal RNA, partial sequence | NR_027579.1 | 1402 | 334 | 734 | 28.6 | 671.5 | 0 | 388/401 | 96 | 0/401 | 0 | Plus | Plus | Bacteria; Firmicutes; Clostridia; Eubacteriales; Lachnospiraceae; Mediterraneibacter; [Ruminococcus] lactaris | [Ruminococcus] lactaris | Mediterraneibacter | Lachnospiraceae | Eubacteriales | Clostridia | Firmicutes | Bacteria |
| ASV566 | 422 | 1 | 1 | 422 | 100 | NR_024815.1 Prevotella shahii strain EHS11 16S ribosomal RNA, partial sequence | NR_024815.1 | 1490 | 356 | 777 | 28.32 | 542.2 | 8E-154 | 381/424 | 89 | 4/424 | 0 | Plus | Plus | Bacteria; Bacteroidetes; Bacteroidia; Bacteroidales; Prevotellaceae; Prevotella; Prevotella shahii | Prevotella shahii | Prevotella | Prevotellaceae | Bacteroidales | Bacteroidia | Bacteroidetes | Bacteria |
| ASV567 | 402 | 1 | 1 | 402 | 100 | NR_042832.1 Roseburia faecis strain M72/1 16S ribosomal RNA, partial sequence | NR_042832.1 | 1456 | 347 | 749 | 27.68 | 665.9 | 0 | 389/403 | 96 | 1/403 | 0 | Plus | Plus | Bacteria; Firmicutes; Clostridia; Eubacteriales; Lachnospiraceae; Roseburia; Roseburia faecis | Roseburia faecis | Roseburia | Lachnospiraceae | Eubacteriales | Clostridia | Firmicutes | Bacteria |
| ASV568 | 427 | 1 | 1 | 427 | 100 | NR_044111.1 Selenomonas bovis strain WG 16S ribosomal RNA, partial sequence | NR_044111.1 | 1525 | 370 | 796 | 28 | 678.8 | 0 | 409/429 | 95 | 4/429 | 0 | Plus | Plus | Bacteria; Firmicutes; Negativicutes; Selenomonadales; Selenomonadaceae; Selenomonas; Selenomonas bovis | Selenomonas bovis | Selenomonas | Selenomonadaceae | Selenomonadales | Negativicutes | Firmicutes | Bacteria |
| ASV569 | 421 | 1 | 1 | 421 | 100 | NR_144616.1 Muribaculum intestinale strain YL27 16S ribosomal RNA, partial sequence | NR_144616.1 | 1460 | 297 | 718 | 28.9 | 484.9 | 1E-136 | 372/425 | 87 | 7/425 | 1 | Plus | Plus | Bacteria; Bacteroidetes; Bacteroidia; Bacteroidales; Muribaculaceae; Muribaculum; Muribaculum intestinale | Muribaculum intestinale | Muribaculum | Muribaculaceae | Bacteroidales | Bacteroidia | Bacteroidetes | Bacteria |
| ASV570 | 403 | 1 | 3 | 403 | 99.5 | NR_125677.1 Vallitalea pronyensis strain FatNI3 16S ribosomal RNA, partial sequence | NR_125677.1 | 1480 | 365 | 764 | 27.03 | 462.8 | 6E-130 | 352/402 | 87 | 3/402 | 0 | Plus | Plus | Bacteria; Firmicutes; Clostridia; Eubacteriales; Vallitaleaceae; Vallitalea; Vallitalea pronyensis | Vallitalea pronyensis | Vallitalea | Vallitaleaceae | Eubacteriales | Clostridia | Firmicutes | Bacteria |
| ASV571 | 427 | 1 | 1 | 427 | 100 | NR_044111.1 Selenomonas bovis strain WG 16S ribosomal RNA, partial sequence | NR_044111.1 | 1525 | 370 | 796 | 28 | 662.2 | 0 | 406/429 | 94 | 4/429 | 0 | Plus | Plus | Bacteria; Firmicutes; Negativicutes; Selenomonadales; Selenomonadaceae; Selenomonas; Selenomonas bovis | Selenomonas bovis | Selenomonas | Selenomonadaceae | Selenomonadales | Negativicutes | Firmicutes | Bacteria |
| ASV572 | 401 | 1 | 1 | 401 | 100 | NR_144749.1 Ihubacter massiliensis strain Marseille-P2843 16S ribosomal RNA, partial sequence | NR_144749.1 | 1479 | 364 | 766 | 27.25 | 521.9 | 9E-148 | 364/404 | 90 | 4/404 | 0 | Plus | Plus | Bacteria; Firmicutes; Clostridia; Eubacteriales; Clostridiales Family XIII. Incertae Sedis; Ihubacter; Ihubacter massiliensis | Ihubacter massiliensis | Ihubacter | Clostridiales Family XIII. Incertae Sedis | Eubacteriales | Clostridia | Firmicutes | Bacteria |
| ASV573 | 427 | 1 | 1 | 427 | 100 | NR_102980.1 Megasphaera elsdenii DSM 20460 16S ribosomal RNA, partial sequence | NR_102980.1 | 1552 | 369 | 795 | 27.51 | 784.1 | 0 | 426/427 | 99 | 0/427 | 0 | Plus | Plus | Bacteria; Firmicutes; Negativicutes; Veillonellales; Veillonellaceae; Megasphaera; Megasphaera elsdenii | Megasphaera elsdenii | Megasphaera | Veillonellaceae | Veillonellales | Negativicutes | Firmicutes | Bacteria |
| ASV574 | 427 | 1 | 1 | 427 | 100 | NR_044111.1 Selenomonas bovis strain WG 16S ribosomal RNA, partial sequence | NR_044111.1 | 1525 | 370 | 796 | 28 | 695.5 | 0 | 410/427 | 96 | 0/427 | 0 | Plus | Plus | Bacteria; Firmicutes; Negativicutes; Selenomonadales; Selenomonadaceae; Selenomonas; Selenomonas bovis | Selenomonas bovis | Selenomonas | Selenomonadaceae | Selenomonadales | Negativicutes | Firmicutes | Bacteria |
| ASV575 | 405 | 2 | 1 | 405 | 100 | NR_029356.1 Flavonifractor plautii strain 265 16S ribosomal RNA, partial sequence | NR_029356.1 | 1550 | 353 | 757 | 26.13 | 494.2 | 2E-139 | 359/405 | 88 | 0/405 | 0 | Plus | Plus | Bacteria; Firmicutes; Clostridia; Eubacteriales; Oscillospiraceae; Flavonifractor; Flavonifractor plautii | Flavonifractor plautii | Flavonifractor | Oscillospiraceae | Eubacteriales | Clostridia | Firmicutes | Bacteria |
| ASV576 | 426 | 1 | 1 | 426 | 100 | NR_029335.1 Holdemania filiformis strain J1-31B-1 16S ribosomal RNA, partial sequence | NR_029335.1 | 1391 | 267 | 692 | 30.63 | 544 | 2E-154 | 382/426 | 89 | 0/426 | 0 | Plus | Plus | Bacteria; Firmicutes; Erysipelotrichia; Erysipelotrichales; Erysipelotrichaceae; Holdemania; Holdemania filiformis | Holdemania filiformis | Holdemania | Erysipelotrichaceae | Erysipelotrichales | Erysipelotrichia | Firmicutes | Bacteria |
| ASV577 | 404 | 1 | 1 | 404 | 100 | NR_118156.1 Oscillibacter ruminantium GH1 16S ribosomal RNA, partial sequence | NR_118156.1 | 1409 | 321 | 724 | 28.67 | 448 | 2E-125 | 354/408 | 86 | 8/408 | 1 | Plus | Plus | Bacteria; Firmicutes; Clostridia; Eubacteriales; Oscillospiraceae; Oscillibacter; Oscillibacter ruminantium | Oscillibacter ruminantium | Oscillibacter | Oscillospiraceae | Eubacteriales | Clostridia | Firmicutes | Bacteria |
| ASV578 | 404 | 2 | 1 | 404 | 100 | NR_074793.2 Oscillibacter valericigenes strain Sjm18-20 16S ribosomal RNA, complete sequence | NR_074793.2 | 1530 | 372 | 775 | 26.41 | 614.2 | 2E-175 | 380/404 | 94 | 0/404 | 0 | Plus | Plus | Bacteria; Firmicutes; Clostridia; Eubacteriales; Oscillospiraceae; Oscillibacter; Oscillibacter valericigenes | Oscillibacter valericigenes | Oscillibacter | Oscillospiraceae | Eubacteriales | Clostridia | Firmicutes | Bacteria |
| ASV579 | 422 | 1 | 1 | 422 | 100 | NR_040877.1 Prevotella copri DSM 18205 strain CB7 16S ribosomal RNA, partial sequence | NR_040877.1 | 1494 | 358 | 779 | 28.25 | 754.6 | 0 | 417/422 | 98 | 0/422 | 0 | Plus | Plus | Bacteria; Bacteroidetes; Bacteroidia; Bacteroidales; Prevotellaceae; Prevotella; Prevotella copri | Prevotella copri | Prevotella | Prevotellaceae | Bacteroidales | Bacteroidia | Bacteroidetes | Bacteria |
| ASV580 | 404 | 1 | 1 | 404 | 100 | NR_118156.1 Oscillibacter ruminantium GH1 16S ribosomal RNA, partial sequence | NR_118156.1 | 1409 | 321 | 724 | 28.67 | 630.8 | 2E-180 | 383/404 | 94 | 0/404 | 0 | Plus | Plus | Bacteria; Firmicutes; Clostridia; Eubacteriales; Oscillospiraceae; Oscillibacter; Oscillibacter ruminantium | Oscillibacter ruminantium | Oscillibacter | Oscillospiraceae | Eubacteriales | Clostridia | Firmicutes | Bacteria |
| ASV581 | 421 | 1 | 1 | 421 | 100 | NR_041691.1 Butyricimonas virosa strain MT12 16S ribosomal RNA, partial sequence | NR_041691.1 | 1486 | 352 | 772 | 28.33 | 778.6 | 0 | 421/421 | 100 | 0/421 | 0 | Plus | Plus | Bacteria; Bacteroidetes; Bacteroidia; Bacteroidales; Odoribacteraceae; Butyricimonas; Butyricimonas virosa | Butyricimonas virosa | Butyricimonas | Odoribacteraceae | Bacteroidales | Bacteroidia | Bacteroidetes | Bacteria |
| ASV582 | 426 | 1 | 1 | 425 | 99.77 | NR_027526.1 Catenibacterium mitsuokai strain DSM 15897 16S ribosomal RNA, partial sequence | NR_027526.1 | 1485 | 345 | 767 | 28.48 | 712.1 | 0 | 412/425 | 96 | 2/425 | 0 | Plus | Plus | Bacteria; Firmicutes; Erysipelotrichia; Erysipelotrichales; Coprobacillaceae; Catenibacterium; Catenibacterium mitsuokai | Catenibacterium mitsuokai | Catenibacterium | Coprobacillaceae | Erysipelotrichales | Erysipelotrichia | Firmicutes | Bacteria |
| ASV584 | 402 | 1 | 1 | 402 | 100 | NR_029239.3 Clostridium chartatabidum strain 163 16S ribosomal RNA, partial sequence | NR_029239.3 | 1460 | 323 | 724 | 27.53 | 721.3 | 0 | 398/402 | 99 | 0/402 | 0 | Plus | Plus | Bacteria; Firmicutes; Clostridia; Eubacteriales; Clostridiaceae; Clostridium; Clostridium chartatabidum | Clostridium chartatabidum | Clostridium | Clostridiaceae | Eubacteriales | Clostridia | Firmicutes | Bacteria |
| ASV585 | 427 | 1 | 1 | 427 | 100 | NR_025131.1 Treponema parvum strain OMZ 833 16S ribosomal RNA, partial sequence | NR_025131.1 | 1485 | 341 | 767 | 28.75 | 523.7 | 3E-148 | 380/428 | 88 | 2/428 | 0 | Plus | Plus | Bacteria; Spirochaetes; Spirochaetia; Spirochaetales; Spirochaetaceae; Treponema; Treponema parvum | Treponema parvum | Treponema | Spirochaetaceae | Spirochaetales | Spirochaetia | Spirochaetes | Bacteria |
| ASV586 | 420 | 1 | 1 | 420 | 100 | NR_170509.1 Duncaniella freteri strain TLL-A3 16S ribosomal RNA, partial sequence | NR_170509.1 | 1538 | 364 | 784 | 27.37 | 449.9 | 5E-126 | 364/423 | 86 | 5/423 | 1 | Plus | Plus | Bacteria; Bacteroidetes; Bacteroidia; Bacteroidales; Muribaculaceae; Duncaniella; Duncaniella freteri | Duncaniella freteri | Duncaniella | Muribaculaceae | Bacteroidales | Bacteroidia | Bacteroidetes | Bacteria |
| ASV587 | 405 | 1 | 1 | 405 | 100 | NR_118554.1 Intestinimonas butyriciproducens strain SRB-521-5-I 16S ribosomal RNA, partial sequence | NR_118554.1 | 1475 | 346 | 750 | 27.46 | 488.6 | 1E-137 | 362/409 | 88 | 8/409 | 1 | Plus | Plus | Bacteria; Firmicutes; Clostridia; Eubacteriales; ; Intestinimonas; Intestinimonas butyriciproducens | Intestinimonas butyriciproducens | Intestinimonas | | Eubacteriales | Clostridia | Firmicutes | Bacteria |
| ASV588 | 422 | 2 | 1 | 422 | 100 | NR_113117.1 Prevotella oralis ATCC 33269 strain JCM 12251 16S ribosomal RNA, partial sequence | NR_113117.1 | 1496 | 361 | 782 | 28.21 | 630.8 | 2E-180 | 395/422 | 93 | 0/422 | 0 | Plus | Plus | Bacteria; Bacteroidetes; Bacteroidia; Bacteroidales; Prevotellaceae; Prevotella; Prevotella oralis | Prevotella oralis | Prevotella | Prevotellaceae | Bacteroidales | Bacteroidia | Bacteroidetes | Bacteria |
| ASV589 | 404 | 2 | 1 | 404 | 100 | NR_118156.1 Oscillibacter ruminantium GH1 16S ribosomal RNA, partial sequence | NR_118156.1 | 1409 | 321 | 724 | 28.67 | 470.2 | 3E-132 | 357/407 | 87 | 6/407 | 1 | Plus | Plus | Bacteria; Firmicutes; Clostridia; Eubacteriales; Oscillospiraceae; Oscillibacter; Oscillibacter ruminantium | Oscillibacter ruminantium | Oscillibacter | Oscillospiraceae | Eubacteriales | Clostridia | Firmicutes | Bacteria |
| ASV590 | 402 | 5 | 1 | 402 | 100 | NR_102884.1 Ruminococcus champanellensis 18P13 = JCM 17042 16S ribosomal RNA, partial sequence | NR_102884.1 | 1494 | 348 | 749 | 26.91 | 599.4 | 4E-171 | 376/402 | 93 | 0/402 | 0 | Plus | Plus | Bacteria; Firmicutes; Clostridia; Eubacteriales; Oscillospiraceae; Ruminococcus; Ruminococcus champanellensis | Ruminococcus champanellensis | Ruminococcus | Oscillospiraceae | Eubacteriales | Clostridia | Firmicutes | Bacteria |
| ASV591 | 422 | 1 | 1 | 422 | 100 | NR_041342.1 Parabacteroides distasonis strain JCM 5825 16S ribosomal RNA, partial sequence | NR_041342.1 | 1488 | 353 | 774 | 28.36 | 558.8 | 8E-159 | 383/423 | 90 | 2/423 | 0 | Plus | Plus | Bacteria; Bacteroidetes; Bacteroidia; Bacteroidales; Tannerellaceae; Parabacteroides; Parabacteroides distasonis | Parabacteroides distasonis | Parabacteroides | Tannerellaceae | Bacteroidales | Bacteroidia | Bacteroidetes | Bacteria |
| ASV592 | 405 | 1 | 1 | 405 | 100 | NR_044972.1 Sporobacter termitidis strain SYR 16S ribosomal RNA, partial sequence | NR_044972.1 | 1506 | 348 | 752 | 26.89 | 592 | 7E-169 | 377/406 | 92 | 2/406 | 0 | Plus | Plus | Bacteria; Firmicutes; Clostridia; Eubacteriales; Oscillospiraceae; Sporobacter; Sporobacter termitidis | Sporobacter termitidis | Sporobacter | Oscillospiraceae | Eubacteriales | Clostridia | Firmicutes | Bacteria |
| ASV593 | 429 | 1 | 1 | 429 | 100 | NR_025079.1 Desulfomonile limimaris strain DCB-M 16S ribosomal RNA, partial sequence | NR_025079.1 | 1523 | 363 | 790 | 28.1 | 436.9 | 4E-122 | 366/430 | 85 | 3/430 | 0 | Plus | Plus | Bacteria; Proteobacteria; Deltaproteobacteria; Syntrophobacterales; Syntrophaceae; Desulfomonile; Desulfomonile limimaris | Desulfomonile limimaris | Desulfomonile | Syntrophaceae | Syntrophobacterales | Deltaproteobacteria | Proteobacteria | Bacteria |
| ASV594 | 427 | 1 | 1 | 427 | 100 | NR_028797.1 Selenomonas infelix strain VPI D 75 B-30 16S ribosomal RNA, partial sequence | NR_028797.1 | 1524 | 373 | 799 | 28.02 | 606.8 | 3E-173 | 394/427 | 92 | 0/427 | 0 | Plus | Plus | Bacteria; Firmicutes; Negativicutes; Selenomonadales; Selenomonadaceae; Selenomonas; Selenomonas infelix | Selenomonas infelix | Selenomonas | Selenomonadaceae | Selenomonadales | Negativicutes | Firmicutes | Bacteria |
| ASV595 | 404 | 5 | 1 | 404 | 100 | NR_115693.1 Gracilibacter thermotolerans JW/YJL-S1 16S ribosomal RNA, partial sequence | NR_115693.1 | 1526 | 459 | 861 | 26.41 | 484.9 | 1E-136 | 360/407 | 88 | 7/407 | 1 | Plus | Plus | Bacteria; Firmicutes; Clostridia; Eubacteriales; Gracilibacteraceae; Gracilibacter; Gracilibacter thermotolerans | Gracilibacter thermotolerans | Gracilibacter | Gracilibacteraceae | Eubacteriales | Clostridia | Firmicutes | Bacteria |
| ASV596 | 422 | 1 | 1 | 422 | 100 | NR_151886.1 Prevotella colorans strain A1336 16S ribosomal RNA, partial sequence | NR_151886.1 | 1491 | 358 | 779 | 28.3 | 625.3 | 7E-179 | 394/422 | 93 | 0/422 | 0 | Plus | Plus | Bacteria; Bacteroidetes; Bacteroidia; Bacteroidales; Prevotellaceae; Prevotella; Prevotella colorans | Prevotella colorans | Prevotella | Prevotellaceae | Bacteroidales | Bacteroidia | Bacteroidetes | Bacteria |
| ASV597 | 402 | 1 | 1 | 402 | 100 | NR_044054.1 Blautia wexlerae DSM 19850 16S ribosomal RNA, partial sequence | NR_044054.1 | 1438 | 314 | 715 | 27.96 | 710.2 | 0 | 396/402 | 98 | 0/402 | 0 | Plus | Plus | Bacteria; Firmicutes; Clostridia; Eubacteriales; Lachnospiraceae; Blautia; Blautia wexlerae | Blautia wexlerae | Blautia | Lachnospiraceae | Eubacteriales | Clostridia | Firmicutes | Bacteria |
| ASV598 | 422 | 4 | 1 | 422 | 100 | NR_044864.1 Hallella seregens ATCC 51272 16S ribosomal RNA, partial sequence | NR_044864.1 | 1463 | 335 | 756 | 28.84 | 603.1 | 3E-172 | 390/422 | 92 | 0/422 | 0 | Plus | Plus | Bacteria; Bacteroidetes; Bacteroidia; Bacteroidales; Prevotellaceae; Prevotella; Hallella seregens | Hallella seregens | Prevotella | Prevotellaceae | Bacteroidales | Bacteroidia | Bacteroidetes | Bacteria |
| ASV599 | 427 | 1 | 1 | 426 | 99.77 | NR_027596.1 Mitsuokella multacida strain DSM 20544 16S ribosomal RNA, partial sequence | NR_027596.1 | 1511 | 346 | 771 | 28.19 | 656.7 | 0 | 402/426 | 94 | 0/426 | 0 | Plus | Plus | Bacteria; Firmicutes; Negativicutes; Selenomonadales; Selenomonadaceae; Mitsuokella; Mitsuokella multacida | Mitsuokella multacida | Mitsuokella | Selenomonadaceae | Selenomonadales | Negativicutes | Firmicutes | Bacteria |
| ASV600 | 422 | 1 | 1 | 422 | 100 | NR_113411.1 Prevotella copri DSM 18205 strain JCM 13464 16S ribosomal RNA, partial sequence | NR_113411.1 | 1493 | 358 | 779 | 28.27 | 752.7 | 0 | 417/422 | 98 | 0/422 | 0 | Plus | Plus | Bacteria; Bacteroidetes; Bacteroidia; Bacteroidales; Prevotellaceae; Prevotella; Prevotella copri | Prevotella copri | Prevotella | Prevotellaceae | Bacteroidales | Bacteroidia | Bacteroidetes | Bacteria |
| ASV601 | 422 | 1 | 1 | 422 | 100 | NR_113411.1 Prevotella copri DSM 18205 strain JCM 13464 16S ribosomal RNA, partial sequence | NR_113411.1 | 1493 | 358 | 779 | 28.27 | 758.2 | 0 | 418/422 | 99 | 0/422 | 0 | Plus | Plus | Bacteria; Bacteroidetes; Bacteroidia; Bacteroidales; Prevotellaceae; Prevotella; Prevotella copri | Prevotella copri | Prevotella | Prevotellaceae | Bacteroidales | Bacteroidia | Bacteroidetes | Bacteria |
| ASV603 | 404 | 1 | 1 | 404 | 100 | NR_028911.1 Anaerovorax odorimutans strain NorPut 16S ribosomal RNA, partial sequence | NR_028911.1 | 1488 | 352 | 755 | 27.15 | 569.9 | 3E-162 | 373/405 | 92 | 2/405 | 0 | Plus | Plus | Bacteria; Firmicutes; Clostridia; Eubacteriales; Clostridiales Family XIII. Incertae Sedis; Anaerovorax; Anaerovorax odorimutans | Anaerovorax odorimutans | Anaerovorax | Clostridiales Family XIII. Incertae Sedis | Eubacteriales | Clostridia | Firmicutes | Bacteria |
| ASV604 | 405 | 1 | 1 | 405 | 100 | NR_044972.1 Sporobacter termitidis strain SYR 16S ribosomal RNA, partial sequence | NR_044972.1 | 1506 | 348 | 752 | 26.89 | 569.9 | 3E-162 | 373/406 | 91 | 2/406 | 0 | Plus | Plus | Bacteria; Firmicutes; Clostridia; Eubacteriales; Oscillospiraceae; Sporobacter; Sporobacter termitidis | Sporobacter termitidis | Sporobacter | Oscillospiraceae | Eubacteriales | Clostridia | Firmicutes | Bacteria |
| ASV605 | 402 | 4 | 1 | 402 | 100 | NR_026493.1 [Clostridium] indolis strain 7 16S ribosomal RNA, partial sequence | NR_026493.1 | 1488 | 355 | 756 | 27.02 | 660.4 | 0 | 387/402 | 96 | 0/402 | 0 | Plus | Plus | Bacteria; Firmicutes; Clostridia; Eubacteriales; Lachnospiraceae; Lacrimispora; Lacrimispora indolis | Lacrimispora indolis | Lacrimispora | Lachnospiraceae | Eubacteriales | Clostridia | Firmicutes | Bacteria |
| ASV606 | 402 | 1 | 1 | 402 | 100 | NR_134772.1 Murimonas intestini strain SRB-530-5-H 16S ribosomal RNA, partial sequence | NR_134772.1 | 1433 | 314 | 715 | 28.05 | 682.5 | 0 | 391/402 | 97 | 0/402 | 0 | Plus | Plus | Bacteria; Firmicutes; Clostridia; Eubacteriales; Lachnospiraceae; Murimonas; Murimonas intestini | Murimonas intestini | Murimonas | Lachnospiraceae | Eubacteriales | Clostridia | Firmicutes | Bacteria |
| ASV607 | 402 | 2 | 1 | 402 | 100 | NR_024661.1 Eubacterium ruminantium strain GA195 16S ribosomal RNA, partial sequence | NR_024661.1 | 1508 | 378 | 779 | 26.66 | 643.8 | 0 | 384/402 | 95 | 0/402 | 0 | Plus | Plus | Bacteria; Firmicutes; Clostridia; Eubacteriales; Eubacteriaceae; Eubacterium; Eubacterium ruminantium | Eubacterium ruminantium | Eubacterium | Eubacteriaceae | Eubacteriales | Clostridia | Firmicutes | Bacteria |
| ASV608 | 427 | 1 | 1 | 427 | 100 | NR_042797.1 Treponema berlinense strain 7CPL208 16S ribosomal RNA, partial sequence | NR_042797.1 | 1440 | 275 | 701 | 29.65 | 701 | 0 | 411/427 | 96 | 0/427 | 0 | Plus | Plus | Bacteria; Spirochaetes; Spirochaetia; Spirochaetales; Spirochaetaceae; Treponema; Treponema berlinense | Treponema berlinense | Treponema | Spirochaetaceae | Spirochaetales | Spirochaetia | Spirochaetes | Bacteria |
| ASV609 | 422 | 3 | 1 | 422 | 100 | NR_113411.1 Prevotella copri DSM 18205 strain JCM 13464 16S ribosomal RNA, partial sequence | NR_113411.1 | 1493 | 358 | 779 | 28.27 | 597.6 | 2E-170 | 391/424 | 92 | 4/424 | 0 | Plus | Plus | Bacteria; Bacteroidetes; Bacteroidia; Bacteroidales; Prevotellaceae; Prevotella; Prevotella copri | Prevotella copri | Prevotella | Prevotellaceae | Bacteroidales | Bacteroidia | Bacteroidetes | Bacteria |
| ASV610 | 405 | 1 | 1 | 405 | 100 | NR_044972.1 Sporobacter termitidis strain SYR 16S ribosomal RNA, partial sequence | NR_044972.1 | 1506 | 348 | 752 | 26.89 | 575.4 | 7E-164 | 374/406 | 92 | 2/406 | 0 | Plus | Plus | Bacteria; Firmicutes; Clostridia; Eubacteriales; Oscillospiraceae; Sporobacter; Sporobacter termitidis | Sporobacter termitidis | Sporobacter | Oscillospiraceae | Eubacteriales | Clostridia | Firmicutes | Bacteria |
| ASV611 | 422 | 1 | 1 | 422 | 100 | NR_041285.1 Prevotella multisaccharivorax strain PPPA20 16S ribosomal RNA, partial sequence | NR_041285.1 | 1491 | 356 | 777 | 28.3 | 647.4 | 0 | 399/423 | 94 | 2/423 | 0 | Plus | Plus | Bacteria; Bacteroidetes; Bacteroidia; Bacteroidales; Prevotellaceae; Prevotella; Prevotella multisaccharivorax | Prevotella multisaccharivorax | Prevotella | Prevotellaceae | Bacteroidales | Bacteroidia | Bacteroidetes | Bacteria |
| ASV612 | 405 | 1 | 1 | 405 | 100 | NR_112900.1 Christensenella minuta strain YIT 12065 16S ribosomal RNA, partial sequence | NR_112900.1 | 1497 | 358 | 762 | 27.05 | 533 | 4E-151 | 369/408 | 90 | 6/408 | 1 | Plus | Plus | Bacteria; Firmicutes; Clostridia; Eubacteriales; Christensenellaceae; Christensenella; Christensenella minuta | Christensenella minuta | Christensenella | Christensenellaceae | Eubacteriales | Clostridia | Firmicutes | Bacteria |
| ASV615 | 402 | 1 | 1 | 402 | 100 | NR_118673.1 [Eubacterium] hallii strain ATCC 27751 16S ribosomal RNA, partial sequence | NR_118673.1 | 1479 | 371 | 772 | 27.18 | 699.2 | 0 | 393/402 | 97 | 0/402 | 0 | Plus | Plus | Bacteria; Firmicutes; Clostridia; Eubacteriales; Lachnospiraceae; Anaerobutyricum; Anaerobutyricum hallii | Anaerobutyricum hallii | Anaerobutyricum | Lachnospiraceae | Eubacteriales | Clostridia | Firmicutes | Bacteria |
| ASV616 | 405 | 2 | 1 | 405 | 100 | NR_156080.1 Kineothrix alysoides strain KNHs209 16S ribosomal RNA, partial sequence | NR_156080.1 | 1410 | 353 | 754 | 28.51 | 701 | 0 | 397/405 | 98 | 3/405 | 0 | Plus | Plus | Bacteria; Firmicutes; Clostridia; Eubacteriales; Lachnospiraceae; Kineothrix; Kineothrix alysoides | Kineothrix alysoides | Kineothrix | Lachnospiraceae | Eubacteriales | Clostridia | Firmicutes | Bacteria |
| ASV617 | 405 | 1 | 1 | 405 | 100 | NR_118554.1 Intestinimonas butyriciproducens strain SRB-521-5-I 16S ribosomal RNA, partial sequence | NR_118554.1 | 1475 | 346 | 750 | 27.46 | 533 | 4E-151 | 366/405 | 90 | 0/405 | 0 | Plus | Plus | Bacteria; Firmicutes; Clostridia; Eubacteriales; ; Intestinimonas; Intestinimonas butyriciproducens | Intestinimonas butyriciproducens | Intestinimonas | | Eubacteriales | Clostridia | Firmicutes | Bacteria |
| ASV618 | 427 | 1 | 1 | 427 | 100 | NR_044111.1 Selenomonas bovis strain WG 16S ribosomal RNA, partial sequence | NR_044111.1 | 1525 | 370 | 796 | 28 | 656.7 | 0 | 406/430 | 94 | 6/430 | 1 | Plus | Plus | Bacteria; Firmicutes; Negativicutes; Selenomonadales; Selenomonadaceae; Selenomonas; Selenomonas bovis | Selenomonas bovis | Selenomonas | Selenomonadaceae | Selenomonadales | Negativicutes | Firmicutes | Bacteria |
| ASV619 | 402 | 1 | 1 | 402 | 100 | NR_024750.1 Coprococcus catus strain VPI-C6-61 16S ribosomal RNA, partial sequence | NR_024750.1 | 1315 | 317 | 719 | 30.65 | 734.2 | 0 | 401/403 | 99 | 1/403 | 0 | Plus | Plus | Bacteria; Firmicutes; Clostridia; Eubacteriales; Lachnospiraceae; Coprococcus; Coprococcus catus | Coprococcus catus | Coprococcus | Lachnospiraceae | Eubacteriales | Clostridia | Firmicutes | Bacteria |
| ASV620 | 422 | 2 | 1 | 422 | 100 | NR_113119.1 Prevotella oris strain JCM 8540 16S ribosomal RNA, partial sequence | NR_113119.1 | 1481 | 345 | 766 | 28.49 | 575.4 | 7E-164 | 386/423 | 91 | 2/423 | 0 | Plus | Plus | Bacteria; Bacteroidetes; Bacteroidia; Bacteroidales; Prevotellaceae; Prevotella; Prevotella oris | Prevotella oris | Prevotella | Prevotellaceae | Bacteroidales | Bacteroidia | Bacteroidetes | Bacteria |
| ASV621 | 405 | 1 | 1 | 405 | 100 | NR_144611.1 Flintibacter butyricus strain BLS21 16S ribosomal RNA, partial sequence | NR_144611.1 | 808 | 299 | 703 | 50.12 | 616.1 | 4E-176 | 381/405 | 94 | 0/405 | 0 | Plus | Plus | Bacteria; Firmicutes; Clostridia; Eubacteriales; ; Flintibacter; Flintibacter butyricus | Flintibacter butyricus | Flintibacter | | Eubacteriales | Clostridia | Firmicutes | Bacteria |
| ASV622 | 405 | 1 | 1 | 405 | 100 | NR_159227.1 Monoglobus pectinilyticus strain 14 16S ribosomal RNA, partial sequence | NR_159227.1 | 1493 | 368 | 769 | 26.93 | 501.6 | 1E-141 | 362/406 | 89 | 5/406 | 1 | Plus | Plus | Bacteria; Firmicutes; Clostridia; Eubacteriales; Oscillospiraceae; Monoglobus; Monoglobus pectinilyticus | Monoglobus pectinilyticus | Monoglobus | Oscillospiraceae | Eubacteriales | Clostridia | Firmicutes | Bacteria |
| ASV623 | 403 | 1 | 1 | 403 | 100 | NR_148619.1 Agathobacter ruminis strain JK623 16S ribosomal RNA, partial sequence | NR_148619.1 | 1434 | 318 | 721 | 28.17 | 701 | 0 | 396/404 | 98 | 1/404 | 0 | Plus | Plus | Bacteria; Firmicutes; Clostridia; Eubacteriales; Lachnospiraceae; Agathobacter; Agathobacter ruminis | Agathobacter ruminis | Agathobacter | Lachnospiraceae | Eubacteriales | Clostridia | Firmicutes | Bacteria |
| ASV624 | 404 | 1 | 1 | 404 | 100 | NR_025930.1 Ruminococcus bromii strain ATCC 27255 16S ribosomal RNA, partial sequence | NR_025930.1 | 1380 | 313 | 716 | 29.28 | 647.4 | 0 | 387/405 | 95 | 2/405 | 0 | Plus | Plus | Bacteria; Firmicutes; Clostridia; Eubacteriales; Oscillospiraceae; Ruminococcus; Ruminococcus bromii | Ruminococcus bromii | Ruminococcus | Oscillospiraceae | Eubacteriales | Clostridia | Firmicutes | Bacteria |
| ASV625 | 427 | 5 | 1 | 427 | 100 | NR_042111.1 Lactobacillus gallinarum strain ATCC 33199 16S ribosomal RNA, partial sequence | NR_042111.1 | 1546 | 362 | 788 | 27.62 | 789.6 | 0 | 427/427 | 100 | 0/427 | 0 | Plus | Plus | Bacteria; Firmicutes; Bacilli; Lactobacillales; Lactobacillaceae; Lactobacillus; Lactobacillus gallinarum | Lactobacillus gallinarum | Lactobacillus | Lactobacillaceae | Lactobacillales | Bacilli | Firmicutes | Bacteria |
| ASV626 | 427 | 1 | 1 | 427 | 100 | NR_027596.1 Mitsuokella multacida strain DSM 20544 16S ribosomal RNA, partial sequence | NR_027596.1 | 1511 | 346 | 772 | 28.26 | 758.2 | 0 | 421/427 | 98 | 0/427 | 0 | Plus | Plus | Bacteria; Firmicutes; Negativicutes; Selenomonadales; Selenomonadaceae; Mitsuokella; Mitsuokella multacida | Mitsuokella multacida | Mitsuokella | Selenomonadaceae | Selenomonadales | Negativicutes | Firmicutes | Bacteria |
| ASV627 | 420 | 1 | 1 | 420 | 100 | NR_144616.1 Muribaculum intestinale strain YL27 16S ribosomal RNA, partial sequence | NR_144616.1 | 1460 | 297 | 718 | 28.9 | 518.2 | 1E-146 | 376/423 | 88 | 4/423 | 0 | Plus | Plus | Bacteria; Bacteroidetes; Bacteroidia; Bacteroidales; Muribaculaceae; Muribaculum; Muribaculum intestinale | Muribaculum intestinale | Muribaculum | Muribaculaceae | Bacteroidales | Bacteroidia | Bacteroidetes | Bacteria |
| ASV628 | 427 | 1 | 1 | 427 | 100 | NR_112902.1 Phascolarctobacterium succinatutens strain YIT 12067 16S ribosomal RNA, partial sequence | NR_112902.1 | 1610 | 444 | 870 | 26.52 | 767.5 | 0 | 423/427 | 99 | [illegible] | [illegible] | [illegible] | [illegible] | [illegible] | [illegible] | [illegible] | [illegible] | [illegible] | [illegible] | [illegible] | [illegible] |
| ASV629 | 422 | 1 | 1 | 422 | 100 | NR_040877.1 Prevotella copri DSM 18205 strain CB7 16S ribosomal RNA, partial sequence | NR_040877.1 | 1494 | 358 | 779 | 28.25 | 749 | 0 | 416/422 | 98 | [illegible] | [illegible] | [illegible] | [illegible] | [illegible] | [illegible] | [illegible] | [illegible] | [illegible] | [illegible] | [illegible] | [illegible] |
| ASV630 | 403 | 1 | 1 | 403 | 100 | NR_144611.1 Flintibacter butyricus strain BLS21 16S ribosomal RNA, partial sequence | NR_144611.1 | 808 | 299 | 703 | 50.12 | 508.9 | 7E-144 | 365/408 | 89 | [illegible] | [illegible] | [illegible] | [illegible] | [illegible] | [illegible] | [illegible] | [illegible] | [illegible] | [illegible] | [illegible] | [illegible] |
| ASV631 | 422 | 2 | 1 | 422 | 100 | NR_113206.1 Bacteroides faecichinchillae JCM 17102 16S ribosomal RNA, partial sequence | NR_113206.1 | 1486 | 353 | 774 | 28.4 | 536.6 | 4E-152 | 382/426 | 89 | [illegible] | [illegible] | [illegible] | [illegible] | [illegible] | [illegible] | [illegible] | [illegible] | [illegible] | [illegible] | [illegible] | [illegible] |
| ASV632 | 404 | 2 | 1 | 404 | 100 | NR_074793.2 Oscillibacter valericigenes strain Sjm18-20 16S ribosomal RNA, complete sequence | NR_074793.2 | 1530 | 372 | 775 | 26.41 | 619.7 | 3E-177 | 381/404 | 94 | [illegible] | [illegible] | [illegible] | [illegible] | [illegible] | [illegible] | [illegible] | [illegible] | [illegible] | [illegible] | [illegible] | [illegible] |
| ASV633 | 407 | 1 | 1 | 407 | 100 | NR_118047.1 Pseudoscardovia suis strain DPTE4 16S ribosomal RNA, partial sequence | NR_118047.1 | 1360 | 281 | 687 | 29.93 | 752.7 | 0 | 407/407 | 100 | [illegible] | [illegible] | [illegible] | [illegible] | [illegible] | [illegible] | [illegible] | [illegible] | [illegible] | [illegible] | [illegible] | [illegible] |
| ASV634 | 428 | 1 | 1 | 428 | 100 | NR_104781.1 Treponema bryantii strain RUS-1 16S ribosomal RNA, partial sequence | NR_104781.1 | 1535 | 351 | 778 | 27.88 | 636.4 | 0 | 400/428 | 93 | [illegible] | [illegible] | [illegible] | [illegible] | [illegible] | [illegible] | [illegible] | [illegible] | [illegible] | [illegible] | [illegible] | [illegible] |
| ASV635 | 427 | 1 | 1 | 427 | 100 | NR_044111.1 Selenomonas bovis strain WG 16S ribosomal RNA, partial sequence | NR_044111.1 | 1525 | 370 | 796 | 28 | 761.9 | 0 | 422/427 | 98 | [illegible] | [illegible] | [illegible] | [illegible] | [illegible] | [illegible] | [illegible] | [illegible] | [illegible] | [illegible] | [illegible] | [illegible] |
| ASV636 | 427 | 1 | 1 | 427 | 100 | NR_044111.1 Selenomonas bovis strain WG 16S ribosomal RNA, partial sequence | NR_044111.1 | 1525 | 370 | 796 | 28 | 767.5 | 0 | 423/427 | 99 | [illegible] | [illegible] | [illegible] | [illegible] | [illegible] | [illegible] | [illegible] | [illegible] | [illegible] | [illegible] | [illegible] | [illegible] |
| ASV637 | 402 | 1 | 1 | 402 | 100 | NR_122051.1 Clostridium saccharobutylicum strain NCP 262 16S ribosomal RNA, partial sequence | NR_122051.1 | 1501 | 348 | 749 | 26.78 | 682.5 | 0 | 391/402 | 97 | [illegible] | [illegible] | [illegible] | [illegible] | [illegible] | [illegible] | [illegible] | [illegible] | [illegible] | [illegible] | [illegible] | [illegible] |
| ASV638 | 410 | 1 | 1 | 410 | 100 | NR_152695.1 Akkermansia glycaniphila strain Pyt 16S ribosomal RNA, partial sequence | NR_152695.1 | 1438 | 317 | 726 | 28.51 | 603.1 | 3E-172 | 382/410 | 93 | [illegible] | [illegible] | [illegible] | [illegible] | [illegible] | [illegible] | [illegible] | [illegible] | [illegible] | [illegible] | [illegible] | [illegible] |
| ASV639 | 404 | 5 | 1 | 404 | 100 | NR_115693.1 Gracilibacter thermotolerans JW/YJL-S1 16S ribosomal RNA, partial sequence | NR_115693.1 | 1526 | 459 | 861 | 26.41 | 490.5 | 3E-138 | 362/408 | 88 | [illegible] | [illegible] | [illegible] | [illegible] | [illegible] | [illegible] | [illegible] | [illegible] | [illegible] | [illegible] | [illegible] | [illegible] |
| ASV640 | 427 | 1 | 1 | 427 | 100 | NR_112652.1 Lactobacillus equicursoris DSM 19284 = JCM 14600 = CIP 110162 strain DI70 16S ribosomal RNA, partial sequence | NR_112652.1 | 1486 | 351 | 777 | 28.73 | 712.1 | 0 | 413/427 | 96 | [illegible] | [illegible] | [illegible] | [illegible] | [illegible] | [illegible] | [illegible] | [illegible] | [illegible] | [illegible] | [illegible] | [illegible] |
| ASV641 | 402 | 2 | 1 | 402 | 100 | NR_144696.1 Clostridium saudiense strain JCC 16S ribosomal RNA, partial sequence | NR_144696.1 | 1478 | 350 | 751 | 27.2 | 699.2 | 0 | 394/402 | 98 | [illegible] | [illegible] | [illegible] | [illegible] | [illegible] | [illegible] | [illegible] | [illegible] | [illegible] | [illegible] | [illegible] | [illegible] |
| ASV642 | 404 | 1 | 1 | 404 | 100 | NR_025670.1 Pseudoflavonifractor capillosus ATCC 29799 16S ribosomal RNA, partial sequence | NR_025670.1 | 1483 | 346 | 750 | 27.31 | 608.7 | 7E-174 | 380/405 | 93 | [illegible] | [illegible] | [illegible] | [illegible] | [illegible] | [illegible] | [illegible] | [illegible] | [illegible] | [illegible] | [illegible] | [illegible] |
| ASV643 | 427 | 3 | 1 | 427 | 100 | NR_029106.1 Lactobacillus delbrueckii subsp. indicus strain NCC725 16S ribosomal RNA, partial sequence | NR_029106.1 | 1515 | 348 | 774 | 28.18 | 701 | 0 | 412/428 | 96 | [illegible] | [illegible] | [illegible] | [illegible] | [illegible] | [illegible] | [illegible] | [illegible] | [illegible] | [illegible] | [illegible] | [illegible] |
| ASV644 | 404 | 1 | 1 | 404 | 100 | NR_118156.1 Oscillibacter ruminantium GH1 16S ribosomal RNA, partial sequence | NR_118156.1 | 1409 | 321 | 724 | 28.67 | 603.1 | 3E-172 | 379/405 | 93 | [illegible] | [illegible] | [illegible] | [illegible] | [illegible] | [illegible] | [illegible] | [illegible] | [illegible] | [illegible] | [illegible] | [illegible] |
| ASV645 | 422 | 2 | 1 | 422 | 100 | NR_113117.1 Prevotella oralis ATCC 33269 strain JCM 12251 16S ribosomal RNA, partial sequence | NR_113117.1 | 1496 | 361 | 782 | 28.21 | 575.4 | 7E-164 | 386/423 | 91 | [illegible] | [illegible] | [illegible] | [illegible] | [illegible] | [illegible] | [illegible] | [illegible] | [illegible] | [illegible] | [illegible] | [illegible] |
| ASV647 | 402 | 1 | 1 | 402 | 100 | NR_104846.1 Gemmiger formicilis strain X2-56 16S ribosomal RNA, partial sequence | NR_104846.1 | 1426 | 305 | 706 | 28.19 | 732.4 | 0 | 400/402 | 99 | [illegible] | [illegible] | [illegible] | [illegible] | [illegible] | [illegible] | [illegible] | [illegible] | [illegible] | [illegible] | [illegible] | [illegible] |
| ASV648 | 422 | 1 | 1 | 422 | 100 | NR_040877.1 Prevotella copri DSM 18205 strain CB7 16S ribosomal RNA, partial sequence | NR_040877.1 | 1494 | 358 | 779 | 28.25 | 765.6 | 0 | 419/422 | 99 | [illegible] | [illegible] | [illegible] | [illegible] | [illegible] | [illegible] | [illegible] | [illegible] | [illegible] | [illegible] | [illegible] | [illegible] |
| ASV649 | 427 | 2 | 1 | 426 | 99.77 | NR_027596.1 Mitsuokella multacida strain DSM 20544 16S ribosomal RNA, partial sequence | NR_027596.1 | 1511 | 346 | 771 | 28.19 | 651.1 | 0 | 402/427 | 94 | [illegible] | [illegible] | [illegible] | [illegible] | [illegible] | [illegible] | [illegible] | [illegible] | [illegible] | [illegible] | [illegible] | [illegible] |
| ASV650 | 422 | 2 | 1 | 422 | 100 | NR_113119.1 Prevotella oris strain JCM 8540 16S ribosomal RNA, partial sequence | NR_113119.1 | 1481 | 345 | 766 | 28.49 | 581 | 2E-165 | 387/423 | 91 | [illegible] | [illegible] | [illegible] | [illegible] | [illegible] | [illegible] | [illegible] | [illegible] | [illegible] | [illegible] | [illegible] | [illegible] |
| ASV651 | 427 | 2 | 1 | 427 | 100 | NR_029106.1 Lactobacillus delbrueckii subsp. indicus strain NCC725 16S ribosomal RNA, partial sequence | NR_029106.1 | 1515 | 348 | 774 | 28.18 | 784.1 | 0 | 426/427 | 99 | [illegible] | [illegible] | [illegible] | [illegible] | [illegible] | [illegible] | [illegible] | [illegible] | [illegible] | [illegible] | [illegible] | [illegible] |
| ASV652 | 425 | 1 | 1 | 425 | 100 | NR_029325.1 Schwartzia succinivorans strain S1-1 16S ribosomal RNA, partial sequence | NR_029325.1 | 1489 | 344 | 769 | 28.61 | 470.2 | 4E-132 | 373/430 | 86 | [illegible] | [illegible] | [illegible] | [illegible] | [illegible] | [illegible] | [illegible] | [illegible] | [illegible] | [illegible] | [illegible] | [illegible] |
| ASV653 | 421 | 1 | 1 | 421 | 100 | NR_144746.1 Marseilla massiliensis strain Marseille-P2475 16S ribosomal RNA, partial sequence | NR_144746.1 | 1494 | 356 | 777 | 28.25 | 448 | 2E-125 | 368/428 | 85 | [illegible] | [illegible] | [illegible] | [illegible] | [illegible] | [illegible] | [illegible] | [illegible] | [illegible] | [illegible] | [illegible] | [illegible] |
| ASV654 | 421 | 1 | 1 | 421 | 100 | NR_144616.1 Muribaculum intestinale strain YL27 16S ribosomal RNA, partial sequence | NR_144616.1 | 1460 | 297 | 718 | 28.9 | 479.4 | 6E-135 | 373/427 | 87 | [illegible] | [illegible] | [illegible] | [illegible] | [illegible] | [illegible] | [illegible] | [illegible] | [illegible] | [illegible] | [illegible] | [illegible] |
| ASV655 | 421 | 1 | 1 | 421 | 100 | NR_144616.1 Muribaculum intestinale strain YL27 16S ribosomal RNA, partial sequence | NR_144616.1 | 1460 | 297 | 718 | 28.9 | 490.5 | 3E-138 | 371/423 | 87 | [illegible] | [illegible] | [illegible] | [illegible] | [illegible] | [illegible] | [illegible] | [illegible] | [illegible] | [illegible] | [illegible] | [illegible] |
| ASV656 | 405 | 1 | 1 | 405 | 100 | NR_118554.1 Intestinimonas butyriciproducens strain SRB-521-5-I 16S ribosomal RNA, partial sequence | NR_118554.1 | 1475 | 346 | 750 | 27.46 | 577.3 | 2E-164 | 374/405 | 92 | [illegible] | [illegible] | [illegible] | [illegible] | [illegible] | [illegible] | [illegible] | [illegible] | [illegible] | [illegible] | [illegible] | [illegible] |
| ASV657 | 427 | 1 | 1 | 427 | 100 | NR_074755.1 Treponema succinifaciens DSM 2489 16S ribosomal RNA, partial sequence | NR_074755.1 | 1531 | 353 | 779 | 27.89 | 778.6 | 0 | 425/427 | 99 | [illegible] | [illegible] | [illegible] | [illegible] | [illegible] | [illegible] | [illegible] | [illegible] | [illegible] | [illegible] | [illegible] | [illegible] |
| ASV658 | 404 | 1 | 1 | 404 | 100 | NR_025127.1 Lachnobacterium bovis strain LRC 5382 16S ribosomal RNA, partial sequence | NR_025127.1 | 1453 | 362 | 765 | 27.8 | 658.5 | 0 | 388/404 | 96 | [illegible] | [illegible] | [illegible] | [illegible] | [illegible] | [illegible] | [illegible] | [illegible] | [illegible] | [illegible] | [illegible] | [illegible] |
| ASV659 | 405 | 2 | 1 | 405 | 100 | NR_102884.1 Ruminococcus champanellensis 18P13 = JCM 17042 16S ribosomal RNA, partial sequence | NR_102884.1 | 1494 | 348 | 749 | 26.91 | 606.8 | 3E-173 | 380/405 | 93 | [illegible] | [illegible] | [illegible] | [illegible] | [illegible] | [illegible] | [illegible] | [illegible] | [illegible] | [illegible] | [illegible] | [illegible] |
| ASV660 | 405 | 1 | 1 | 405 | 100 | NR_026102.1 Ruminiclostridium papyrosolvens DSM 2782 16S ribosomal RNA, partial sequence | NR_026102.1 | 1491 | 330 | 733 | 27.1 | 486.8 | 3E-137 | 358/405 | 88 | [illegible] | [illegible] | [illegible] | [illegible] | [illegible] | [illegible] | [illegible] | [illegible] | [illegible] | [illegible] | [illegible] | [illegible] |
| ASV662 | 402 | 1 | 1 | 402 | 100 | NR_170495.1 Blautia faecicola strain KGMB01111 16S ribosomal RNA, complete sequence | NR_170495.1 | 1534 | 372 | 773 | 26.21 | 577.3 | 2E-164 | 372/402 | 92 | [illegible] | [illegible] | [illegible] | [illegible] | [illegible] | [illegible] | [illegible] | [illegible] | [illegible] | [illegible] | [illegible] | [illegible] |
| ASV663 | 404 | 1 | 1 | 404 | 100 | NR_144611.1 Flintibacter butyricus strain BLS21 16S ribosomal RNA, partial sequence | NR_144611.1 | 808 | 299 | 703 | 50.12 | 586.5 | 3E-167 | 376/405 | 92 | [illegible] | [illegible] | [illegible] | [illegible] | [illegible] | [illegible] | [illegible] | [illegible] | [illegible] | [illegible] | [illegible] | [illegible] |
| ASV664 | 403 | 5 | 1 | 403 | 100 | NR_118521.1 Campylobacter lanienae NCTC 13004 strain CCUG 44467 16S ribosomal RNA, partial sequence | NR_118521.1 | 1434 | 328 | 730 | 28.1 | 695.5 | 0 | 394/403 | 97 | [illegible] | [illegible] | [illegible] | [illegible] | [illegible] | [illegible] | [illegible] | [illegible] | [illegible] | [illegible] | [illegible] | [illegible] |
| ASV665 | 403 | 1 | 1 | 403 | 100 | NR_104907.1 Eubacterium coprostanoligenes strain HL 16S ribosomal RNA, partial sequence | NR_104907.1 | 1446 | 332 | 734 | 27.87 | 717.6 | 0 | 398/403 | 98 | [illegible] | [illegible] | [illegible] | [illegible] | [illegible] | [illegible] | [illegible] | [illegible] | [illegible] | [illegible] | [illegible] | [illegible] |
| ASV666 | 402 | 1 | 1 | 402 | 100 | NR_026103.1 [Clostridium] populeti strain 743A 16S ribosomal RNA, partial sequence | NR_026103.1 | 1489 | 333 | 734 | 27 | 627.1 | 2E-179 | 381/402 | 94 | [illegible] | [illegible] | [illegible] | [illegible] | [illegible] | [illegible] | [illegible] | [illegible] | [illegible] | [illegible] | [illegible] | [illegible] |
| ASV667 | 402 | 1 | 1 | 402 | 100 | NR_074613.1 [Eubacterium] eligens ATCC 27750 16S ribosomal RNA, partial sequence | NR_074613.1 | 1519 | 367 | 768 | 26.46 | 715.8 | 0 | 397/402 | 98 | [illegible] | [illegible] | [illegible] | [illegible] | [illegible] | [illegible] | [illegible] | [illegible] | [illegible] | [illegible] | [illegible] | [illegible] |
| ASV668 | 402 | 1 | 1 | 402 | 100 | NR_042200.1 [Clostridium] asparagiforme strain N6 16S ribosomal RNA, partial sequence | NR_042200.1 | 1468 | 335 | 736 | 27.38 | 688.1 | 0 | 392/402 | 97 | [illegible] | [illegible] | [illegible] | [illegible] | [illegible] | [illegible] | [illegible] | [illegible] | [illegible] | [illegible] | [illegible] | [illegible] |
| ASV669 | 403 | 1 | 3 | 403 | 99.5 | NR_125677.1 Vallitalea pronyensis strain FatNI3 16S ribosomal RNA, partial sequence | NR_125677.1 | 1480 | 365 | 764 | 27.03 | 473.9 | 3E-133 | 354/402 | 88 | [illegible] | [illegible] | [illegible] | [illegible] | [illegible] | [illegible] | [illegible] | [illegible] | [illegible] | [illegible] | [illegible] | [illegible] |
| ASV670 | 404 | 1 | 1 | 404 | 100 | NR_042074.1 Eubacterium pyruvativorans strain I-6 16S ribosomal RNA, partial sequence | NR_042074.1 | 1296 | 264 | 667 | 31.17 | 741.6 | 0 | 403/404 | 99 | [illegible] | [illegible] | [illegible] | [illegible] | [illegible] | [illegible] | [illegible] | [illegible] | [illegible] | [illegible] | [illegible] | [illegible] |
| ASV671 | 402 | 1 | 1 | 402 | 100 | NR_147400.1 Clostridium merdae strain Marseille-P2953 16S ribosomal RNA, partial sequence | NR_147400.1 | 1482 | 356 | 757 | 27.13 | 455.4 | 1E-127 | 350/402 | 87 | [illegible] | [illegible] | [illegible] | [illegible] | [illegible] | [illegible] | [illegible] | [illegible] | [illegible] | [illegible] | [illegible] | [illegible] |
| ASV672 | 402 | 2 | 1 | 402 | 100 | NR_042144.1 Clostridium butyricum strain VPI3266 16S ribosomal RNA, partial sequence | NR_042144.1 | 1425 | 338 | 739 | 28.21 | 699.2 | 0 | 394/402 | 98 | [illegible] | [illegible] | [illegible] | [illegible] | [illegible] | [illegible] | [illegible] | [illegible] | [illegible] | [illegible] | [illegible] | [illegible] |
| ASV673 | 426 | 1 | 1 | 426 | 100 | NR_029335.1 Holdemania filiformis strain J1-31B-1 16S ribosomal RNA, partial sequence | NR_029335.1 | 1391 | 267 | 692 | 30.63 | 544 | 2E-154 | 382/426 | 89 | [illegible] | [illegible] | [illegible] | [illegible] | [illegible] | [illegible] | [illegible] | [illegible] | [illegible] | [illegible] | [illegible] | [illegible] |
| ASV674 | 404 | 1 | 1 | 404 | 100 | NR_042832.1 Roseburia faecis strain M72/1 16S ribosomal RNA, partial sequence | NR_042832.1 | 1456 | 347 | 749 | 27.68 | 667.8 | 0 | 390/404 | 96 | [illegible] | [illegible] | [illegible] | [illegible] | [illegible] | [illegible] | [illegible] | [illegible] | [illegible] | [illegible] | [illegible] | [illegible] |
| ASV675 | 422 | 1 | 1 | 422 | 100 | NR_113411.1 Prevotella copri DSM 18205 strain JCM 13464 16S ribosomal RNA, partial sequence | NR_113411.1 | 1493 | 358 | 779 | 28.27 | 763.8 | 0 | 419/422 | 99 | [illegible] | [illegible] | [illegible] | [illegible] | [illegible] | [illegible] | [illegible] | [illegible] | [illegible] | [illegible] | [illegible] | [illegible] |
| ASV676 | 403 | 1 | 3 | 403 | 99.5 | NR_125464.1 Anaerobacterium chartisolvens strain T-1-35 16S ribosomal RNA, partial sequence | NR_125464.1 | 1451 | 344 | 745 | 27.71 | 492.3 | 7E-139 | 357/402 | 88 | [illegible] | [illegible] | [illegible] | [illegible] | [illegible] | [illegible] | [illegible] | [illegible] | [illegible] | [illegible] | [illegible] | [illegible] |
| ASV677 | 420 | 1 | 1 | 420 | 100 | NR_170509.1 Duncaniella freteri strain TLL-A3 16S ribosomal RNA, partial sequence | NR_170509.1 | 1538 | 364 | 784 | 27.37 | 455.4 | 1E-127 | 367/425 | 86 | [illegible] | [illegible] | [illegible] | [illegible] | [illegible] | [illegible] | [illegible] | [illegible] | [illegible] | [illegible] | [illegible] | [illegible] |
| ASV678 | 402 | 1 | 1 | 402 | 100 | NR_115340.1 Saccharofermentans acetigenes strain P6 16S ribosomal RNA, partial sequence | NR_115340.1 | 1510 | 353 | 757 | 26.82 | 490.5 | 3E-138 | 359/405 | 88 | [illegible] | [illegible] | [illegible] | [illegible] | [illegible] | [illegible] | [illegible] | [illegible] | [illegible] | [illegible] | [illegible] | [illegible] |
| ASV679 | 427 | 1 | 1 | 427 | 100 | NR_041666.1 Dialister succinatiphilus YIT 11850 16S ribosomal RNA, partial sequence | NR_041666.1 | 1521 | 353 | 779 | 28.07 | 706.5 | 0 | 413/428 | 96 | [illegible] | [illegible] | [illegible] | [illegible] | [illegible] | [illegible] | [illegible] | [illegible] | [illegible] | [illegible] | [illegible] | [illegible] |
| ASV680 | 422 | 1 | 1 | 422 | 100 | NR_113411.1 Prevotella copri DSM 18205 strain JCM 13464 16S ribosomal RNA, partial sequence | NR_113411.1 | 1493 | 358 | 779 | 28.27 | 752.7 | 0 | 417/422 | 98 | [illegible] | [illegible] | [illegible] | [illegible] | [illegible] | [illegible] | [illegible] | [illegible] | [illegible] | [illegible] | [illegible] | [illegible] |
| ASV681 | 402 | 1 | 1 | 402 | 100 | NR_074613.1 [Eubacterium] eligens ATCC 27750 16S ribosomal RNA, partial sequence | NR_074613.1 | 1519 | 367 | 768 | 26.46 | 627.1 | 2E-179 | 382/403 | 94 | [illegible] | [illegible] | [illegible] | [illegible] | [illegible] | [illegible] | [illegible] | [illegible] | [illegible] | [illegible] | [illegible] | [illegible] |
| ASV682 | 422 | 2 | 1 | 422 | 100 | NR_113119.1 Prevotella oris strain JCM 8540 16S ribosomal RNA, partial sequence | NR_113119.1 | 1481 | 345 | 766 | 28.49 | 608.7 | 7E-174 | 393/424 | 92 | [illegible] | [illegible] | [illegible] | [illegible] | [illegible] | [illegible] | [illegible] | [illegible] | [illegible] | [illegible] | [illegible] | [illegible] |
| ASV683 | 422 | 1 | 1 | 422 | 100 | NR_041285.1 Prevotella multisaccharivorax strain PPPA20 16S ribosomal RNA, partial sequence | NR_041285.1 | 1491 | 356 | 777 | 28.3 | 603.1 | 3E-172 | 391/423 | 92 | [illegible] | [illegible] | [illegible] | [illegible] | [illegible] | [illegible] | [illegible] | [illegible] | [illegible] | [illegible] | [illegible] | [illegible] |
| ASV685 | 427 | 1 | 1 | 427 | 100 | NR_042942.1 Treponema porcinum strain 14V28 16S ribosomal RNA, partial sequence | NR_042942.1 | 1426 | 258 | 684 | 29.94 | 784.1 | 0 | 426/427 | 99 | [illegible] | [illegible] | [illegible] | [illegible] | [illegible] | [illegible] | [illegible] | [illegible] | [illegible] | [illegible] | [illegible] | [illegible] |
| ASV686 | 421 | 1 | 1 | 421 | 100 | NR_144750.1 Prevotellamassilia timonensis strain Marseille-P2831 16S ribosomal RNA, partial sequence | NR_144750.1 | 1493 | 358 | 779 | 28.27 | 651.1 | 0 | 399/422 | 94 | [illegible] | [illegible] | [illegible] | [illegible] | [illegible] | [illegible] | [illegible] | [illegible] | [illegible] | [illegible] | [illegible] | [illegible] |
| ASV687 | 405 | 1 | 1 | 405 | 100 | NR_144611.1 Flintibacter butyricus strain BLS21 16S ribosomal RNA, partial sequence | NR_144611.1 | 808 | 299 | 703 | 50.12 | 627.1 | 2E-179 | 383/405 | 94 | [illegible] | [illegible] | [illegible] | [illegible] | [illegible] | [illegible] | [illegible] | [illegible] | [illegible] | [illegible] | [illegible] | [illegible] |
| ASV688 | 422 | 5 | 1 | 422 | 100 | NR_044864.1 Hallella seregens ATCC 51272 16S ribosomal RNA, partial sequence | NR_044864.1 | 1463 | 335 | 756 | 28.84 | 608.7 | 7E-174 | 392/423 | 92 | [illegible] | [illegible] | [illegible] | [illegible] | [illegible] | [illegible] | [illegible] | [illegible] | [illegible] | [illegible] | [illegible] | [illegible] |
| ASV689 | 427 | 5 | 1 | 427 | 100 | NR_119109.1 Pseudescherichia vulneris strain ATCC 33821 16S ribosomal RNA, partial sequence | NR_119109.1 | 1443 | 344 | 770 | 29.59 | 784.1 | 0 | 426/427 | 99 | [illegible] | [illegible] | [illegible] | [illegible] | [illegible] | [illegible] | [illegible] | [illegible] | [illegible] | [illegible] | [illegible] | [illegible] |
| ASV690 | 406 | 1 | 1 | 406 | 100 | NR_165727.1 Anaerotignum faecicola strain KGMB03357 16S ribosomal RNA, complete sequence | NR_165727.1 | 1535 | 375 | 780 | 26.45 | 706.5 | 0 | 398/406 | 98 | [illegible] | [illegible] | [illegible] | [illegible] | [illegible] | [illegible] | [illegible] | [illegible] | [illegible] | [illegible] | [illegible] | [illegible] |
| ASV691 | 422 | 2 | 1 | 422 | 100 | NR_113117.1 Prevotella oralis ATCC 33269 strain JCM 12251 16S ribosomal RNA, partial sequence | NR_113117.1 | 1496 | 361 | 782 | 28.21 | 614.2 | 2E-175 | 392/422 | 92 | [illegible] | [illegible] | [illegible] | [illegible] | [illegible] | [illegible] | [illegible] | [illegible] | [illegible] | [illegible] | [illegible] | [illegible] |
| ASV692 | 404 | 1 | 1 | 404 | 100 | NR_042007.1 Roseburia inulinivorans DSM 16841 strain A2-194 16S ribosomal RNA, partial sequence | NR_042007.1 | 1454 | 360 | 763 | 27.79 | 691.8 | 0 | 394/404 | 97 | [illegible] | [illegible] | [illegible] | [illegible] | [illegible] | [illegible] | [illegible] | [illegible] | [illegible] | [illegible] | [illegible] | [illegible] |
| ASV693 | 421 | 1 | 1 | 421 | 100 | NR_144616.1 Muribaculum intestinale strain YL27 16S ribosomal RNA, partial sequence | NR_144616.1 | 1460 | 297 | 718 | 28.9 | 523.7 | 3E-148 | 380/426 | 89 | [illegible] | [illegible] | [illegible] | [illegible] | [illegible] | [illegible] | [illegible] | [illegible] | [illegible] | [illegible] | [illegible] | [illegible] |
| ASV694 | 405 | 3 | 1 | 405 | 100 | NR_115307.1 Ethanoligenens harbinense YUAN-3 16S ribosomal RNA, partial sequence | NR_115307.1 | 1497 | 340 | 743 | 26.99 | 531.1 | 2E-150 | 368/407 | 90 | [illegible] | [illegible] | [illegible] | [illegible] | [illegible] | [illegible] | [illegible] | [illegible] | [illegible] | [illegible] | [illegible] | [illegible] |
| ASV695 | 422 | 1 | 1 | 422 | 100 | NR_041364.1 Prevotella stercorea DSM 18206 strain CB35 16S ribosomal RNA, partial sequence | NR_041364.1 | 1488 | 354 | 775 | 28.36 | 603.1 | 3E-172 | 390/422 | 92 | [illegible] | [illegible] | [illegible] | [illegible] | [illegible] | [illegible] | [illegible] | [illegible] | [illegible] | [illegible] | [illegible] | [illegible] |
| ASV696 | 404 | 2 | 1 | 404 | 100 | NR_114326.1 Fusicatenibacter saccharivorans strain HT03-11 16S ribosomal RNA, partial sequence | NR_114326.1 | 1423 | 351 | 752 | 28.25 | 667.8 | 0 | 390/404 | 96 | [illegible] | [illegible] | [illegible] | [illegible] | [illegible] | [illegible] | [illegible] | [illegible] | [illegible] | [illegible] | [illegible] | [illegible] |
| ASV697 | 422 | 2 | 1 | 422 | 100 | NR_113119.1 Prevotella oris strain JCM 8540 16S ribosomal RNA, partial sequence | NR_113119.1 | 1481 | 345 | 766 | 28.49 | 581 | 2E-165 | 387/423 | 91 | [illegible] | [illegible] | [illegible] | [illegible] | [illegible] | [illegible] | [illegible] | [illegible] | [illegible] | [illegible] | [illegible] | [illegible] |
| ASV698 | 421 | 1 | 1 | 421 | 100 | NR_144616.1 Muribaculum intestinale strain YL27 16S ribosomal RNA, partial sequence | NR_144616.1 | 1460 | 297 | 718 | 28.9 | 490.5 | 3E-138 | 371/423 | 87 | [illegible] | [illegible] | [illegible] | [illegible] | [illegible] | [illegible] | [illegible] | [illegible] | [illegible] | [illegible] | [illegible] | [illegible] |
| ASV699 | 426 | 1 | 1 | 426 | 100 | NR_134177.1 Pleomorphochaeta multiformis strain MO-SPC2 16S ribosomal RNA, partial sequence | NR_134177.1 | 1466 | 334 | 760 | 29.13 | 510.8 | 2E-144 | 381/431 | 88 | [illegible] | [illegible] | [illegible] | [illegible] | [illegible] | [illegible] | [illegible] | [illegible] | [illegible] | [illegible] | [illegible] | [illegible] |
| ASV700 | 418 | 1 | 1 | 418 | 100 | NR_025525.1 Butyrivibrio hungatei strain JK 615 16S ribosomal RNA, partial sequence | NR_025525.1 | 1475 | 335 | 749 | 28.14 | 586.5 | 3E-167 | 386/419 | 92 | [illegible] | [illegible] | [illegible] | [illegible] | [illegible] | [illegible] | [illegible] | [illegible] | [illegible] | [illegible] | [illegible] | [illegible] |
| ASV701 | 427 | 1 | 1 | 427 | 100 | NR_102980.1 Megasphaera elsdenii DSM 20460 16S ribosomal RNA, partial sequence | NR_102980.1 | 1552 | 369 | 795 | 27.51 | 773 | 0 | 424/427 | 99 | [illegible] | [illegible] | [illegible] | [illegible] | [illegible] | [illegible] | [illegible] | [illegible] | [illegible] | [illegible] | [illegible] | [illegible] |
| ASV702 | 427 | 1 | 1 | 427 | 100 | NR_044111.1 Selenomonas bovis strain WG 16S ribosomal RNA, partial sequence | NR_044111.1 | 1525 | 370 | 796 | 28 | 634.5 | 0 | 404/432 | 93 | [illegible] | [illegible] | [illegible] | [illegible] | [illegible] | [illegible] | [illegible] | [illegible] | [illegible] | [illegible] | [illegible] | [illegible] |
| ASV704 | 426 | 1 | 1 | 426 | 100 | NR_114696.1 Anaerovibrio lipolyticus DSM 3074 16S ribosomal RNA, partial sequence | NR_114696.1 | 1526 | 372 | 797 | 27.92 | 688.1 | 0 | 410/428 | 95 | [illegible] | [illegible] | [illegible] | [illegible] | [illegible] | [illegible] | [illegible] | [illegible] | [illegible] | [illegible] | [illegible] | [illegible] |
| ASV705 | 426 | 1 | 1 | 426 | 100 | NR_156083.1 Pleomorphochaeta caudata strain SEBR 4223 16S ribosomal RNA, partial sequence | NR_156083.1 | 1400 | 349 | 775 | 30.5 | 516.3 | 5E-146 | 380/429 | 88 | [illegible] | [illegible] | [illegible] | [illegible] | [illegible] | [illegible] | [illegible] | [illegible] | [illegible] | [illegible] | [illegible] | [illegible] |
| ASV706 | 402 | 1 | 1 | 402 | 100 | NR_025931.1 Ruminococcus flavefaciens strain C94 16S ribosomal RNA, partial sequence | NR_025931.1 | 1388 | 325 | 726 | 28.96 | 632.7 | 0 | 382/402 | 95 | [illegible] | [illegible] | [illegible] | [illegible] | [illegible] | [illegible] | [illegible] | [illegible] | [illegible] | [illegible] | [illegible] | [illegible] |
| ASV707 | 427 | 1 | 1 | 427 | 100 | NR_044111.1 Selenomonas bovis strain WG 16S ribosomal RNA, partial sequence | NR_044111.1 | 1525 | 370 | 796 | 28 | 667.8 | 0 | 407/429 | 94 | [illegible] | [illegible] | [illegible] | [illegible] | [illegible] | [illegible] | [illegible] | [illegible] | [illegible] | [illegible] | [illegible] | [illegible] |
| ASV708 | 418 | 1 | 1 | 418 | 100 | NR_102893.1 Butyrivibrio proteoclasticus strain B316 16S ribosomal RNA, partial sequence | NR_102893.1 | 1537 | 367 | 782 | 27.07 | 582.8 | 4E-166 | 385/419 | 91 | [illegible] | [illegible] | [illegible] | [illegible] | [illegible] | [illegible] | [illegible] | [illegible] | [illegible] | [illegible] | [illegible] | [illegible] |
| ASV709 | 422 | 1 | 1 | 422 | 100 | NR_112943.1 Bacteroides stercoris ATCC 43183 strain JCM 9496 16S ribosomal RNA, partial sequence | NR_112943.1 | 1476 | 343 | 764 | 28.59 | 647.4 | 0 | 398/422 | 94 | [illegible] | [illegible] | [illegible] | [illegible] | [illegible] | [illegible] | [illegible] | [illegible] | [illegible] | [illegible] | [illegible] | [illegible] |
| ASV710 | 404 | 1 | 1 | 404 | 100 | NR_074579.1 Desulfotomaculum nigrificans strain CO-1-SRB 16S ribosomal RNA, partial sequence | NR_074579.1 | 1516 | 353 | 757 | 26.72 | 425.9 | 8E-119 | 349/407 | 85 | [illegible] | [illegible] | [illegible] | [illegible] | [illegible] | [illegible] | [illegible] | [illegible] | [illegible] | [illegible] | [illegible] | [illegible] |
| ASV711 | 402 | 3 | 3 | 402 | 99.5 | NR_117867.1 Blautia stercoris strain GAM6-1 16S ribosomal RNA, partial sequence | NR_117867.1 | 1372 | 319 | 718 | 29.15 | 706.5 | 0 | 394/400 | 98 | [illegible] | [illegible] | [illegible] | [illegible] | [illegible] | [illegible] | [illegible] | [illegible] | [illegible] | [illegible] | [illegible] | [illegible] |
| ASV712 | 402 | 1 | 1 | 402 | 100 | NR_026315.1 Pseudobutyrivibrio ruminis strain DSM 9787 16S ribosomal RNA, partial sequence | NR_026315.1 | 1462 | 334 | 735 | 27.5 | 599.4 | 4E-171 | 376/402 | 93 | [illegible] | [illegible] | [illegible] | [illegible] | [illegible] | [illegible] | [illegible] | [illegible] | [illegible] | [illegible] | [illegible] | [illegible] |
| ASV713 | 405 | 1 | 1 | 405 | 100 | NR_044972.1 Sporobacter termitidis strain SYR 16S ribosomal RNA, partial sequence | NR_044972.1 | 1506 | 348 | 752 | 26.89 | 603.1 | 3E-172 | 378/405 | 93 | [illegible] | [illegible] | [illegible] | [illegible] | [illegible] | [illegible] | [illegible] | [illegible] | [illegible] | [illegible] | [illegible] | [illegible] |
| ASV714 | 422 | 1 | 1 | 422 | 100 | NR_024816.1 Prevotella salivae strain EPSA11 16S ribosomal RNA, partial sequence | NR_024816.1 | 1493 | 356 | 777 | 28.27 | 586.5 | 3E-167 | 387/422 | 91 | [illegible] | [illegible] | [illegible] | [illegible] | [illegible] | [illegible] | [illegible] | [illegible] | [illegible] | [illegible] | [illegible] | [illegible] |
| ASV715 | 421 | 2 | 1 | 421 | 100 | NR_113077.1 Paraprevotella clara strain JCM 14859 16S ribosomal RNA, partial sequence | NR_113077.1 | 1480 | 346 | 766 | 28.45 | 490.5 | 3E-138 | 370/422 | 87 | [illegible] | [illegible] | [illegible] | [illegible] | [illegible] | [illegible] | [illegible] | [illegible] | [illegible] | [illegible] | [illegible] | [illegible] |
| ASV717 | 427 | 1 | 1 | 427 | 100 | NR_102980.1 Megasphaera elsdenii DSM 20460 16S ribosomal RNA, partial sequence | NR_102980.1 | 1552 | 369 | 795 | 27.51 | 784.1 | 0 | 426/427 | 99 | [illegible] | [illegible] | [illegible] | [illegible] | [illegible] | [illegible] | [illegible] | [illegible] | [illegible] | [illegible] | [illegible] | [illegible] |
| ASV719 | 427 | 1 | 1 | 427 | 100 | NR_028796.1 Selenomonas noxia strain ATCC 43541 16S ribosomal RNA, partial sequence | NR_028796.1 | 1491 | 375 | 801 | 28.64 | 573.6 | 3E-163 | 391/430 | 90 | [illegible] | [illegible] | [illegible] | [illegible] | [illegible] | [illegible] | [illegible] | [illegible] | [illegible] | [illegible] | [illegible] | [illegible] |
| ASV720 | 422 | 1 | 1 | 422 | 100 | NR_113411.1 Prevotella copri DSM 18205 strain JCM 13464 16S ribosomal RNA, partial sequence | NR_113411.1 | 1493 | 358 | 779 | 28.27 | 736.1 | 0 | 414/422 | 98 | [illegible] | [illegible] | [illegible] | [illegible] | [illegible] | [illegible] | [illegible] | [illegible] | [illegible] | [illegible] | [illegible] | [illegible] |
| ASV721 | 404 | 1 | 1 | 404 | 100 | NR_026315.1 Pseudobutyrivibrio ruminis strain DSM 9787 16S ribosomal RNA, partial sequence | NR_026315.1 | 1462 | 334 | 735 | 27.5 | 678.8 | 0 | 392/404 | 97 | [illegible] | [illegible] | [illegible] | [illegible] | [illegible] | [illegible] | [illegible] | [illegible] | [illegible] | [illegible] | [illegible] | [illegible] |
| ASV722 | 423 | 1 | 1 | 423 | 100 | NR_144750.1 Prevotellamassilia timonensis strain Marseille-P2831 16S ribosomal RNA, partial sequence | NR_144750.1 | 1493 | 358 | 779 | 28.27 | 575.4 | 7E-164 | 388/425 | 91 | [illegible] | [illegible] | [illegible] | [illegible] | [illegible] | [illegible] | [illegible] | [illegible] | [illegible] | [illegible] | [illegible] | [illegible] |
| ASV723 | 422 | 2 | 1 | 422 | 100 | NR_113206.1 Bacteroides faecichinchillae JCM 17102 16S ribosomal RNA, partial sequence | NR_113206.1 | 1486 | 353 | 774 | 28.4 | 542.2 | 8E-154 | 383/426 | 89 | [illegible] | [illegible] | [illegible] | [illegible] | [illegible] | [illegible] | [illegible] | [illegible] | [illegible] | [illegible] | [illegible] | [illegible] |
| ASV724 | 422 | 1 | 1 | 422 | 100 | NR_040877.1 Prevotella copri DSM 18205 strain CB7 16S ribosomal RNA, partial sequence | NR_040877.1 | 1494 | 358 | 779 | 28.25 | 743.5 | 0 | 415/422 | 98 | [illegible] | [illegible] | [illegible] | [illegible] | [illegible] | [illegible] | [illegible] | [illegible] | [illegible] | [illegible] | [illegible] | [illegible] |
| ASV725 | 402 | 1 | 1 | 402 | 100 | NR_024661.1 Eubacterium ruminantium strain GA195 16S ribosomal RNA, partial sequence | NR_024661.1 | 1508 | 378 | 779 | 26.66 | 677 | 0 | 390/402 | 97 | [illegible] | [illegible] | [illegible] | [illegible] | [illegible] | [illegible] | [illegible] | [illegible] | [illegible] | [illegible] | [illegible] | [illegible] |
| ASV726 | 422 | 2 | 1 | 422 | 100 | NR_114009.1 Galbibacter mesophilus strain NBRC 101624 16S ribosomal RNA, partial sequence | NR_114009.1 | 1445 | 328 | 749 | 29.2 | 431.4 | 2E-120 | 360/423 | 85 | [illegible] | [illegible] | [illegible] | [illegible] | [illegible] | [illegible] | [illegible] | [illegible] | [illegible] | [illegible] | [illegible] | [illegible] |
| ASV727 | 427 | 1 | 1 | 427 | 100 | NR_044111.1 Selenomonas bovis strain WG 16S ribosomal RNA, partial sequence | NR_044111.1 | 1525 | 370 | 796 | 28 | 651.1 | 0 | 404/429 | 94 | [illegible] | [illegible] | [illegible] | [illegible] | [illegible] | [illegible] | [illegible] | [illegible] | [illegible] | [illegible] | [illegible] | [illegible] |
| ASV728 | 402 | 2 | 1 | 402 | 100 | NR_029239.3 Clostridium chartatabidum strain 163 16S ribosomal RNA, partial sequence | NR_029239.3 | 1460 | 323 | 724 | 27.53 | 699.2 | 0 | 394/402 | 98 | [illegible] | [illegible] | [illegible] | [illegible] | [illegible] | [illegible] | [illegible] | [illegible] | [illegible] | [illegible] | [illegible] | [illegible] |
| ASV729 | 406 | 1 | 1 | 406 | 100 | NR_115340.1 Saccharofermentans acetigenes strain P6 16S ribosomal RNA, partial sequence | NR_115340.1 | 1510 | 353 | 757 | 26.82 | 494.2 | 2E-139 | 361/407 | 88 | [illegible] | [illegible] | [illegible] | [illegible] | [illegible] | [illegible] | [illegible] | [illegible] | [illegible] | [illegible] | [illegible] | [illegible] |
| ASV730 | 427 | 1 | 1 | 427 | 100 | NR_044111.1 Selenomonas bovis strain WG 16S ribosomal RNA, partial sequence | NR_044111.1 | 1525 | 370 | 796 | 28 | 684.4 | 0 | 408/427 | 95 | [illegible] | [illegible] | [illegible] | [illegible] | [illegible] | [illegible] | [illegible] | [illegible] | [illegible] | [illegible] | [illegible] | [illegible] |
| ASV731 | 405 | 1 | 1 | 405 | 100 | NR_026102.1 Ruminiclostridium papyrosolvens DSM 2782 16S ribosomal RNA, partial sequence | NR_026102.1 | 1491 | 330 | 733 | 27.1 | 486.8 | 3E-137 | 358/405 | 88 | [illegible] | [illegible] | [illegible] | [illegible] | [illegible] | [illegible] | [illegible] | [illegible] | [illegible] | [illegible] | [illegible] | [illegible] |
| ASV733 | 405 | 1 | 1 | 405 | 100 | NR_114789.1 [Clostridium] leptum strain DSM 753 16S ribosomal RNA, partial sequence | NR_114789.1 | 1505 | 364 | 765 | 26.71 | 579.1 | 6E-165 | 376/406 | 92 | [illegible] | [illegible] | [illegible] | [illegible] | [illegible] | [illegible] | [illegible] | [illegible] | [illegible] | [illegible] | [illegible] | [illegible] |
| ASV734 | 421 | 2 | 1 | 421 | 100 | NR_113073.1 Barnesiella intestinihominis strain JCM 15079 16S ribosomal RNA, partial sequence | NR_113073.1 | 1481 | 346 | 767 | 28.49 | 496 | 6E-140 | 374/425 | 88 | [illegible] | [illegible] | [illegible] | [illegible] | [illegible] | [illegible] | [illegible] | [illegible] | [illegible] | [illegible] | [illegible] | [illegible] |
| ASV735 | 405 | 1 | 1 | 405 | 100 | NR_152059.1 Harryflintia acetispora strain V20-281a 16S ribosomal RNA, partial sequence | NR_152059.1 | 1363 | 289 | 692 | 29.64 | 486.8 | 3E-137 | 362/409 | 88 | [illegible] | [illegible] | [illegible] | [illegible] | [illegible] | [illegible] | [illegible] | [illegible] | [illegible] | [illegible] | [illegible] | [illegible] |
| ASV736 | 421 | 1 | 1 | 421 | 100 | NR_144616.1 Muribaculum intestinale strain YL27 16S ribosomal RNA, partial sequence | NR_144616.1 | 1460 | 297 | 718 | 28.9 | 507.1 | 3E-143 | 373/422 | 88 | [illegible] | [illegible] | [illegible] | [illegible] | [illegible] | [illegible] | [illegible] | [illegible] | [illegible] | [illegible] | [illegible] | [illegible] |
| ASV737 | 402 | 1 | 1 | 402 | 100 | NR_026103.1 [Clostridium] populeti strain 743A 16S ribosomal RNA, partial sequence | NR_026103.1 | 1489 | 333 | 734 | 27 | 621.6 | 9E-178 | 380/402 | 94 | [illegible] | [illegible] | [illegible] | [illegible] | [illegible] | [illegible] | [illegible] | [illegible] | [illegible] | [illegible] | [illegible] | [illegible] |
| ASV739 | 422 | 1 | 3 | 422 | 99.53 | NR_044119.1 Parapedobacter soli strain DCY14 16S ribosomal RNA, partial sequence | NR_044119.1 | 1468 | 345 | 764 | 28.61 | 427.7 | 2E-119 | 358/421 | 85 | [illegible] | [illegible] | [illegible] | [illegible] | [illegible] | [illegible] | [illegible] | [illegible] | [illegible] | [illegible] | [illegible] | [illegible] |
| ASV740 | 402 | 1 | 1 | 402 | 100 | NR_118675.1 [Eubacterium] siraeum strain ATCC 29066 16S ribosomal RNA, partial sequence | NR_118675.1 | 1453 | 356 | 757 | 27.67 | 560.7 | 2E-159 | 371/405 | 91 | [illegible] | [illegible] | [illegible] | [illegible] | [illegible] | [illegible] | [illegible] | [illegible] | [illegible] | [illegible] | [illegible] | [illegible] |
| ASV741 | 422 | 1 | 1 | 422 | 100 | NR_041343.1 Parabacteroides merdae strain JCM 9497 16S ribosomal RNA, partial sequence | NR_041343.1 | 1492 | 357 | 778 | 28.28 | 664.1 | 0 | 402/423 | 95 | [illegible] | [illegible] | [illegible] | [illegible] | [illegible] | [illegible] | [illegible] | [illegible] | [illegible] | [illegible] | [illegible] | [illegible] |
| ASV742 | 421 | 2 | 1 | 421 | 100 | NR_113073.1 Barnesiella intestinihominis strain JCM 15079 16S ribosomal RNA, partial sequence | NR_113073.1 | 1481 | 346 | 767 | 28.49 | 468.3 | 1E-131 | 367/423 | 86 | [illegible] | [illegible] | [illegible] | [illegible] | [illegible] | [illegible] | [illegible] | [illegible] | [illegible] | [illegible] | [illegible] | [illegible] |
| ASV743 | 426 | 2 | 1 | 426 | 100 | NR_042260.1 Sphaerochaeta coccoides strain SPN1 16S ribosomal RNA, partial sequence | NR_042260.1 | 1500 | 320 | 746 | 28.47 | 521.9 | 1E-147 | 382/430 | 88 | [illegible] | [illegible] | [illegible] | [illegible] | [illegible] | [illegible] | [illegible] | [illegible] | [illegible] | [illegible] | [illegible] | [illegible] |
| ASV744 | 422 | 1 | 1 | 422 | 100 | NR_113411.1 Prevotella copri DSM 18205 strain JCM 13464 16S ribosomal RNA, partial sequence | NR_113411.1 | 1493 | 358 | 779 | 28.27 | 774.9 | 0 | 421/422 | 99 | [illegible] | [illegible] | [illegible] | [illegible] | [illegible] | [illegible] | [illegible] | [illegible] | [illegible] | [illegible] | [illegible] | [illegible] |
| ASV745 | 422 | 1 | 1 | 422 | 100 | NR_151886.1 Prevotella colorans strain A1336 16S ribosomal RNA, partial sequence | NR_151886.1 | 1491 | 358 | 779 | 28.3 | 625.3 | 7E-179 | 394/422 | 93 | [illegible] | [illegible] | [illegible] | [illegible] | [illegible] | [illegible] | [illegible] | [illegible] | [illegible] | [illegible] | [illegible] | [illegible] |
| ASV746 | 427 | 1 | 1 | 427 | 100 | NR_112902.1 Phascolarctobacterium succinatutens strain YIT 12067 16S ribosomal RNA, partial sequence | NR_112902.1 | 1610 | 444 | 870 | 26.52 | 784.1 | 0 | 426/427 | 99 | [illegible] | [illegible] | [illegible] | [illegible] | [illegible] | [illegible] | [illegible] | [illegible] | [illegible] | [illegible] | [illegible] | [illegible] |
| ASV747 | 405 | 1 | 1 | 405 | 100 | NR_118554.1 Intestinimonas butyriciproducens strain SRB-521-5-I 16S ribosomal RNA, partial sequence | NR_118554.1 | 1475 | 346 | 750 | 27.46 | 510.8 | 2E-144 | 363/406 | 89 | [illegible] | [illegible] | [illegible] | [illegible] | [illegible] | [illegible] | [illegible] | [illegible] | [illegible] | [illegible] | [illegible] | [illegible] |
| ASV748 | 402 | 3 | 1 | 402 | 100 | NR_113231.1 Blautia glucerasea strain JCM 17039 16S ribosomal RNA, partial sequence | NR_113231.1 | 1491 | 364 | 765 | 26.96 | 588.4 | 9E-168 | 374/402 | 93 | [illegible] | [illegible] | [illegible] | [illegible] | [illegible] | [illegible] | [illegible] | [illegible] | [illegible] | [illegible] | [illegible] | [illegible] |
| ASV749 | 427 | 2 | 1 | 427 | 100 | NR_037018.1 Acidaminococcus fermentans strain VR4 16S ribosomal RNA gene, partial sequence | NR_037018.1 | 1516 | 349 | 775 | 28.17 | 717.6 | 0 | 414/427 | 96 | [illegible] | [illegible] | [illegible] | [illegible] | [illegible] | [illegible] | [illegible] | [illegible] | [illegible] | [illegible] | [illegible] | [illegible] |
| ASV750 | 401 | 1 | 1 | 401 | 100 | NR_158111.1 Paludicola psychrotolerans strain NC1253 16S ribosomal RNA, partial sequence | NR_158111.1 | 1490 | 366 | 766 | 26.91 | 625.3 | 7E-179 | 381/402 | 94 | [illegible] | [illegible] | [illegible] | [illegible] | [illegible] | [illegible] | [illegible] | [illegible] | [illegible] | [illegible] | [illegible] | [illegible] |
| ASV751 | 402 | 1 | 1 | 402 | 100 | NR_170495.1 Blautia faecicola strain KGMB01111 16S ribosomal RNA, complete sequence | NR_170495.1 | 1534 | 372 | 773 | 26.21 | 688.1 | 0 | 392/402 | 97 | [illegible] | [illegible] | [illegible] | [illegible] | [illegible] | [illegible] | [illegible] | [illegible] | [illegible] | [illegible] | [illegible] | [illegible] |
| ASV752 | 404 | 1 | 1 | 404 | 100 | NR_044490.1 Butyricicoccus pullicaecorum strain 25-3 16S ribosomal RNA, partial sequence | NR_044490.1 | 1482 | 366 | 770 | 27.33 | 641.9 | 0 | 387/406 | 95 | [illegible] | [illegible] | [illegible] | [illegible] | [illegible] | [illegible] | [illegible] | [illegible] | [illegible] | [illegible] | [illegible] | [illegible] |
| ASV753 | 426 | 1 | 1 | 425 | 99.77 | NR_028840.1 Mitsuokella jalaludinii strain M9 16S ribosomal RNA, partial sequence | NR_028840.1 | 1543 | 362 | 787 | 27.61 | 647.4 | 0 | 404/429 | 94 | [illegible] | [illegible] | [illegible] | [illegible] | [illegible] | [illegible] | [illegible] | [illegible] | [illegible] | [illegible] | [illegible] | [illegible] |
| ASV754 | 405 | 1 | 1 | 405 | 100 | NR_118554.1 Intestinimonas butyriciproducens strain SRB-521-5-I 16S ribosomal RNA, partial sequence | NR_118554.1 | 1475 | 346 | 750 | 27.46 | 538.5 | 9E-153 | 369/407 | 90 | [illegible] | [illegible] | [illegible] | [illegible] | [illegible] | [illegible] | [illegible] | [illegible] | [illegible] | [illegible] | [illegible] | [illegible] |
| ASV755 | 419 | 1 | 1 | 419 | 100 | NR_037116.1 Bifidobacterium choerinum strain Su 806 16S ribosomal RNA, partial sequence | NR_037116.1 | 1530 | 346 | 764 | 27.39 | 774.9 | 0 | 419/419 | 100 | [illegible] | [illegible] | [illegible] | [illegible] | [illegible] | [illegible] | [illegible] | [illegible] | [illegible] | [illegible] | [illegible] | [illegible] |
| ASV756 | 422 | 1 | 1 | 422 | 100 | NR_113411.1 Prevotella copri DSM 18205 strain JCM 13464 16S ribosomal RNA, partial sequence | NR_113411.1 | 1493 | 358 | 779 | 28.27 | 603.1 | 3E-172 | 391/423 | 92 | [illegible] | [illegible] | [illegible] | [illegible] | [illegible] | [illegible] | [illegible] | [illegible] | [illegible] | [illegible] | [illegible] | [illegible] |
| ASV758 | 404 | 1 | 1 | 404 | 100 | NR_026315.1 Pseudobutyrivibrio ruminis strain DSM 9787 16S ribosomal RNA, partial sequence | NR_026315.1 | 1462 | 334 | 735 | 27.5 | 689.9 | 0 | 394/404 | 97 | [illegible] | [illegible] | [illegible] | [illegible] | [illegible] | [illegible] | [illegible] | [illegible] | [illegible] | [illegible] | [illegible] | [illegible] |
| ASV759 | 427 | 2 | 1 | 427 | 100 | NR_113306.1 Megasphaera elsdenii DSM 20460 16S ribosomal RNA, partial sequence | NR_113306.1 | 1174 | 232 | 658 | 36.37 | 780.4 | 0 | 425/427 | 99 | [illegible] | [illegible] | [illegible] | [illegible] | [illegible] | [illegible] | [illegible] | [illegible] | [illegible] | [illegible] | [illegible] | [illegible] |
| ASV760 | 403 | 1 | 3 | 403 | 99.5 | NR_125464.1 Anaerobacterium chartisolvens strain T-1-35 16S ribosomal RNA, partial sequence | NR_125464.1 | 1451 | 344 | 745 | 27.71 | 486.8 | 3E-137 | 356/402 | 88 | [illegible] | [illegible] | [illegible] | [illegible] | [illegible] | [illegible] | [illegible] | [illegible] | [illegible] | [illegible] | [illegible] | [illegible] |
| ASV761 | 402 | 1 | 1 | 402 | 100 | NR_042832.1 Roseburia faecis strain M72/1 16S ribosomal RNA, partial sequence | NR_042832.1 | 1456 | 347 | 749 | 27.68 | 665.9 | 0 | 389/403 | 96 | [illegible] | [illegible] | [illegible] | [illegible] | [illegible] | [illegible] | [illegible] | [illegible] | [illegible] | [illegible] | [illegible] | [illegible] |
| ASV762 | 421 | 2 | 1 | 421 | 100 | NR_113073.1 Barnesiella intestinihominis strain JCM 15079 16S ribosomal RNA, partial sequence | NR_113073.1 | 1481 | 346 | 767 | 28.49 | 496 | 6E-140 | 372/423 | 87 | [illegible] | [illegible] | [illegible] | [illegible] | [illegible] | [illegible] | [illegible] | [illegible] | [illegible] | [illegible] | [illegible] | [illegible] |
| ASV763 | 405 | 1 | 1 | 405 | 100 | NR_144613.1 Irregularibacter muris strain 2PG-426-CC-4.2 16S ribosomal RNA, partial sequence | NR_144613.1 | 1431 | 347 | 751 | 28.3 | 472 | 1E-132 | 359/409 | 87 | [illegible] | [illegible] | [illegible] | [illegible] | [illegible] | [illegible] | [illegible] | [illegible] | [illegible] | [illegible] | [illegible] | [illegible] |
| ASV764 | 401 | 2 | 1 | 401 | 100 | NR_119074.1 Terrisporobacter glycolicus ATCC 14880 = DSM 1288 16S ribosomal RNA, partial sequence | NR_119074.1 | 1461 | 314 | 714 | 27.45 | 725 | 0 | 398/401 | 99 | [illegible] | [illegible] | [illegible] | [illegible] | [illegible] | [illegible] | [illegible] | [illegible] | [illegible] | [illegible] | [illegible] | [illegible] |
| ASV766 | 422 | 2 | 1 | 422 | 100 | NR_113105.1 Prevotella histicola strain JCM 15637 16S ribosomal RNA, partial sequence | NR_113105.1 | 1480 | 346 | 767 | 28.51 | 641.9 | 0 | 398/423 | 94 | [illegible] | [illegible] | [illegible] | [illegible] | [illegible] | [illegible] | [illegible] | [illegible] | [illegible] | [illegible] | [illegible] | [illegible] |
| ASV767 | 422 | 2 | 1 | 422 | 100 | NR_113105.1 Prevotella histicola strain JCM 15637 16S ribosomal RNA, partial sequence | NR_113105.1 | 1480 | 346 | 767 | 28.51 | 647.4 | 0 | 399/423 | 94 | [illegible] | [illegible] | [illegible] | [illegible] | [illegible] | [illegible] | [illegible] | [illegible] | [illegible] | [illegible] | [illegible] | [illegible] |
| ASV768 | 402 | 1 | 1 | 402 | 100 | NR_026312.1 Blautia schinkii strain B 16S ribosomal RNA, partial sequence | NR_026312.1 | 1482 | 344 | 745 | 27.13 | 701 | 0 | 394/402 | 98 | [illegible] | [illegible] | [illegible] | [illegible] | [illegible] | [illegible] | [illegible] | [illegible] | [illegible] | [illegible] | [illegible] | [illegible] |
| ASV769 | 402 | 1 | 1 | 402 | 100 | NR_118554.1 Intestinimonas butyriciproducens strain SRB-521-5-I 16S ribosomal RNA, partial sequence | NR_118554.1 | 1475 | 346 | 750 | 27.46 | 551.4 | 1E-156 | 371/406 | 91 | [illegible] | [illegible] | [illegible] | [illegible] | [illegible] | [illegible] | [illegible] | [illegible] | [illegible] | [illegible] | [illegible] | [illegible] |
| ASV770 | 422 | 1 | 1 | 422 | 100 | NR_041285.1 Prevotella multisaccharivorax strain PPPA20 16S ribosomal RNA, partial sequence | NR_041285.1 | 1491 | 356 | 777 | 28.3 | 630.8 | 2E-180 | 396/423 | 93 | [illegible] | [illegible] | [illegible] | [illegible] | [illegible] | [illegible] | [illegible] | [illegible] | [illegible] | [illegible] | [illegible] | [illegible] |
| ASV771 | 404 | 1 | 1 | 404 | 100 | NR_118156.1 Oscillibacter ruminantium GH1 16S ribosomal RNA, partial sequence | NR_118156.1 | 1409 | 321 | 724 | 28.67 | 614.2 | 2E-175 | 382/406 | 94 | [illegible] | [illegible] | [illegible] | [illegible] | [illegible] | [illegible] | [illegible] | [illegible] | [illegible] | [illegible] | [illegible] | [illegible] |
| ASV772 | 405 | 1 | 1 | 405 | 100 | NR_044972.1 Sporobacter termitidis strain SYR 16S ribosomal RNA, partial sequence | NR_044972.1 | 1506 | 348 | 752 | 26.89 | 536.6 | 3E-152 | 367/406 | 90 | [illegible] | [illegible] | [illegible] | [illegible] | [illegible] | [illegible] | [illegible] | [illegible] | [illegible] | [illegible] | [illegible] | [illegible] |
| ASV773 | 426 | 2 | 1 | 426 | 100 | NR_042260.1 Sphaerochaeta coccoides strain SPN1 16S ribosomal RNA, partial sequence | NR_042260.1 | 1500 | 320 | 746 | 28.47 | 510.8 | 2E-144 | 378/428 | 88 | [illegible] | [illegible] | [illegible] | [illegible] | [illegible] | [illegible] | [illegible] | [illegible] | [illegible] | [illegible] | [illegible] | [illegible] |
| ASV774 | 426 | 1 | 1 | 426 | 100 | NR_114696.1 Anaerovibrio lipolyticus DSM 3074 16S ribosomal RNA, partial sequence | NR_114696.1 | 1526 | 372 | 797 | 27.92 | 706.5 | 0 | 410/426 | 96 | [illegible] | [illegible] | [illegible] | [illegible] | [illegible] | [illegible] | [illegible] | [illegible] | [illegible] | [illegible] | [illegible] | [illegible] |
| ASV775 | 426 | 1 | 1 | 426 | 100 | NR_114696.1 Anaerovibrio lipolyticus DSM 3074 16S ribosomal RNA, partial sequence | NR_114696.1 | 1526 | 372 | 797 | 27.92 | 664.1 | 0 | 403/426 | 94 | [illegible] | [illegible] | [illegible] | [illegible] | [illegible] | [illegible] | [illegible] | [illegible] | [illegible] | [illegible] | [illegible] | [illegible] |
| ASV776 | 404 | 1 | 1 | 404 | 100 | NR_102884.1 Ruminococcus champanellensis 18P13 = JCM 17042 16S ribosomal RNA, partial sequence | NR_102884.1 | 1494 | 348 | 749 | 26.91 | 695.5 | 0 | 395/404 | 97 | [illegible] | [illegible] | [illegible] | [illegible] | [illegible] | [illegible] | [illegible] | [illegible] | [illegible] | [illegible] | [illegible] | [illegible] |
| ASV777 | 402 | 2 | 1 | 402 | 100 | NR_118778.1 [Eubacterium] minutum strain SC 87K 16S ribosomal RNA, partial sequence | NR_118778.1 | 1474 | 344 | 746 | 27.34 | 621.6 | 9E-178 | 381/403 | 94 | [illegible] | [illegible] | [illegible] | [illegible] | [illegible] | [illegible] | [illegible] | [illegible] | [illegible] | [illegible] | [illegible] | [illegible] |
| ASV778 | 402 | 1 | 1 | 402 | 100 | NR_151895.1 Anaerocolumna cellulosilytica strain SN021 16S ribosomal RNA, partial sequence | NR_151895.1 | 1478 | 353 | 754 | 27.2 | 665.9 | 0 | 388/402 | 96 | [illegible] | [illegible] | [illegible] | [illegible] | [illegible] | [illegible] | [illegible] | [illegible] | [illegible] | [illegible] | [illegible] | [illegible] |
| ASV779 | 422 | 2 | 1 | 422 | 100 | NR_113065.1 Bacteroides clarus YIT 12056 strain JCM 16067 16S ribosomal RNA, partial sequence | NR_113065.1 | 1488 | 355 | 776 | 28.36 | 525.6 | 8E-149 | 377/423 | 89 | [illegible] | [illegible] | [illegible] | [illegible] | [illegible] | [illegible] | [illegible] | [illegible] | [illegible] | [illegible] | [illegible] | [illegible] |
| ASV780 | 403 | 1 | 1 | 403 | 100 | NR_104907.1 Eubacterium coprostanoligenes strain HL 16S ribosomal RNA, partial sequence | NR_104907.1 | 1446 | 332 | 734 | 27.87 | 728.7 | 0 | 400/403 | 99 | [illegible] | [illegible] | [illegible] | [illegible] | [illegible] | [illegible] | [illegible] | [illegible] | [illegible] | [illegible] | [illegible] | [illegible] |
| ASV781 | 422 | 5 | 1 | 422 | 100 | NR_044864.1 Hallella seregens ATCC 51272 16S ribosomal RNA, partial sequence | NR_044864.1 | 1463 | 335 | 756 | 28.84 | 603.1 | 3E-172 | 391/423 | 92 | [illegible] | [illegible] | [illegible] | [illegible] | [illegible] | [illegible] | [illegible] | [illegible] | [illegible] | [illegible] | [illegible] | [illegible] |

| | | | | | | | | | | | | | | | | | | | | | | | | | | | | |
|---|---|---|---|---|---|---|---|---|---|---|---|---|---|---|---|---|---|---|---|---|---|---|---|---|---|---|---|---|
| ASV782 | 427 | 2 | 1 | 427 | 100 | NR_037018.1 | Acidaminococcus fermentans strain VR4 16S ribosomal RNA gene, partial sequence | NR_037018.1 | 1516 | 349 | 775 | 28.17 | 739.8 | 0 | 418/427 | 97 | 0/427 | 0 | Plus | Plus | Bacteria; Firmicutes; Negativicutes; Acidaminococcales; Acidaminococcaceae; Acidaminococcus; Acidaminococcus fermentans | Acidaminococcus fermentans | Acidaminococcus | Acidaminococcaceae | Acidaminococcales | Negativicutes | Firmicutes | Bacteria |
| ASV783 | 402 | 2 | 1 | 402 | 100 | NR_042144.1 | Clostridium butyricum strain VPI3266 16S ribosomal RNA, partial sequence | NR_042144.1 | 1425 | 338 | 739 | 28.21 | 737.9 | 0 | 401/402 | 99 | 0/402 | 0 | Plus | Plus | Bacteria; Firmicutes; Clostridia; Eubacteriales; Clostridiaceae; Clostridium; Clostridium butyricum | Clostridium butyricum | Clostridium | Clostridiaceae | Eubacteriales | Clostridia | Firmicutes | Bacteria |
| ASV784 | 402 | 3 | 1 | 402 | 100 | NR_117139.2 | Anaerostipes hadrus strain DSM 3319 16S ribosomal RNA, partial sequence | NR_117139.2 | 1521 | 363 | 764 | 26.43 | 677 | 0 | 390/402 | 97 | 0/402 | 0 | Plus | Plus | Bacteria; Firmicutes; Clostridia; Eubacteriales; Lachnospiraceae; Anaerostipes; Anaerostipes hadrus | Anaerostipes hadrus | Anaerostipes | Lachnospiraceae | Eubacteriales | Clostridia | Firmicutes | Bacteria |
| ASV785 | 402 | 1 | 1 | 402 | 100 | NR_104846.1 | Gemmiger formicilis strain X2-56 16S ribosomal RNA, partial sequence | NR_104846.1 | 1426 | 305 | 706 | 28.19 | 654.8 | 0 | 386/402 | 96 | 0/402 | 0 | Plus | Plus | Bacteria; Firmicutes; Clostridia; Eubacteriales; ; Gemmiger; Gemmiger formicilis | Gemmiger formicilis | Gemmiger | | Eubacteriales | Clostridia | Firmicutes | Bacteria |
| ASV786 | 403 | 1 | 1 | 403 | 100 | NR_074809.1 | Roseburia hominis A2-183 16S ribosomal RNA, partial sequence | NR_074809.1 | 1535 | 367 | 769 | 26.25 | 739.8 | 0 | 402/403 | 99 | 0/403 | 0 | Plus | Plus | Bacteria; Firmicutes; Clostridia; Eubacteriales; Lachnospiraceae; Roseburia; Roseburia hominis | Roseburia hominis | Roseburia | Lachnospiraceae | Eubacteriales | Clostridia | Firmicutes | Bacteria |
| ASV787 | 402 | 1 | 1 | 402 | 100 | NR_026204.1 | [Clostridium] viride strain T2-7 16S ribosomal RNA, partial sequence | NR_026204.1 | 1482 | 345 | 749 | 27.33 | 542.2 | 7E-154 | 368/405 | 90 | 3/405 | 0 | Plus | Plus | Bacteria; Firmicutes; Clostridia; Eubacteriales; Oscillospiraceae; ; [Clostridium] viride | [Clostridium] viride | | Oscillospiraceae | Eubacteriales | Clostridia | Firmicutes | Bacteria |
| ASV788 | 401 | 1 | 1 | 401 | 100 | NR_027573.1 | Intestinibacter bartlettii strain WAL 16138 16S ribosomal RNA, partial sequence | NR_027573.1 | 1429 | 320 | 720 | 28.06 | 725 | 0 | 398/401 | 99 | 0/401 | 0 | Plus | Plus | Bacteria; Firmicutes; Clostridia; Eubacteriales; Peptostreptococcaceae; Intestinibacter; Intestinibacter bartlettii | Intestinibacter bartlettii | Intestinibacter | Peptostreptococcaceae | Eubacteriales | Clostridia | Firmicutes | Bacteria |
| ASV790 | 402 | 2 | 1 | 402 | 100 | NR_042144.1 | Clostridium butyricum strain VPI3266 16S ribosomal RNA, partial sequence | NR_042144.1 | 1425 | 338 | 739 | 28.21 | 737.9 | 0 | 401/402 | 99 | 0/402 | 0 | Plus | Plus | Bacteria; Firmicutes; Clostridia; Eubacteriales; Clostridiaceae; Clostridium; Clostridium butyricum | Clostridium butyricum | Clostridium | Clostridiaceae | Eubacteriales | Clostridia | Firmicutes | Bacteria |
| ASV791 | 402 | 3 | 1 | 402 | 100 | NR_171444.1 | Blautia argi strain N6H1-15 16S ribosomal RNA, partial sequence | NR_171444.1 | 1409 | 318 | 719 | 28.53 | 582.8 | 4E-166 | 374/403 | 92 | 2/403 | 0 | Plus | Plus | Bacteria; Firmicutes; Clostridia; Eubacteriales; Lachnospiraceae; Blautia; Blautia argi | Blautia argi | Blautia | Lachnospiraceae | Eubacteriales | Clostridia | Firmicutes | Bacteria |
| ASV792 | 401 | 1 | 1 | 401 | 100 | NR_144737.1 | Emergencia timonensis strain SN18 16S ribosomal RNA, partial sequence | NR_144737.1 | 1480 | 364 | 767 | 27.3 | 616.1 | 4E-176 | 381/404 | 94 | 3/404 | 0 | Plus | Plus | Bacteria; Firmicutes; Clostridia; Eubacteriales; Clostridiales Family XIII. Incertae Sedis; Emergencia; Emergencia timonensis | Emergencia timonensis | Emergencia | Clostridiales Family XIII. Incertae Sedis | Eubacteriales | Clostridia | Firmicutes | Bacteria |
| ASV793 | 422 | 1 | 1 | 422 | 100 | NR_144616.1 | Muribaculum intestinale strain YL27 16S ribosomal RNA, partial sequence | NR_144616.1 | 1460 | 297 | 718 | 28.9 | 569.9 | 3E-162 | 388/426 | 91 | 8/426 | 1 | Plus | Plus | Bacteria; Bacteroidetes; Bacteroidia; Bacteroidales; Muribaculaceae; Muribaculum; Muribaculum intestinale | Muribaculum intestinale | Muribaculum | Muribaculaceae | Bacteroidales | Bacteroidia | Bacteroidetes | Bacteria |
| ASV794 | 422 | 1 | 1 | 422 | 100 | NR_113411.1 | Prevotella copri DSM 18205 strain JCM 13464 16S ribosomal RNA, partial sequence | NR_113411.1 | 1493 | 358 | 779 | 28.27 | 741.6 | 0 | 415/422 | 98 | 0/422 | 0 | Plus | Plus | Bacteria; Bacteroidetes; Bacteroidia; Bacteroidales; Prevotellaceae; Prevotella; Prevotella copri | Prevotella copri | Prevotella | Prevotellaceae | Bacteroidales | Bacteroidia | Bacteroidetes | Bacteria |
| ASV795 | 421 | 1 | 1 | 421 | 100 | NR_029335.1 | Holdemania filiformis strain J1-31B-1 16S ribosomal RNA, partial sequence | NR_029335.1 | 1391 | 267 | 692 | 30.63 | 533 | 5E-151 | 381/426 | 89 | 5/426 | 1 | Plus | Plus | Bacteria; Firmicutes; Erysipelotrichia; Erysipelotrichales; Erysipelotrichaceae; Holdemania; Holdemania filiformis | Holdemania filiformis | Holdemania | Erysipelotrichaceae | Erysipelotrichales | Erysipelotrichia | Firmicutes | Bacteria |
| ASV796 | 422 | 1 | 1 | 422 | 100 | NR_041342.1 | Parabacteroides distasonis strain JCM 5825 16S ribosomal RNA, partial sequence | NR_041342.1 | 1488 | 353 | 774 | 28.36 | 547.7 | 2E-155 | 380/422 | 90 | 0/422 | 0 | Plus | Plus | Bacteria; Bacteroidetes; Bacteroidia; Bacteroidales; Tannerellaceae; Parabacteroides; Parabacteroides distasonis | Parabacteroides distasonis | Parabacteroides | Tannerellaceae | Bacteroidales | Bacteroidia | Bacteroidetes | Bacteria |
| ASV797 | 426 | 1 | 1 | 426 | 100 | NR_029325.1 | Schwartzia succinivorans strain S1-1 16S ribosomal RNA, partial sequence | NR_029325.1 | 1489 | 344 | 769 | 28.61 | 787.8 | 0 | 426/426 | 100 | 0/426 | 0 | Plus | Plus | Bacteria; Firmicutes; Negativicutes; Selenomonadales; Selenomonadaceae; Schwartzia; Schwartzia succinivorans | Schwartzia succinivorans | Schwartzia | Selenomonadaceae | Selenomonadales | Negativicutes | Firmicutes | Bacteria |
| ASV798 | 404 | 1 | 1 | 404 | 100 | NR_118156.1 | Oscillibacter ruminantium GH1 16S ribosomal RNA, partial sequence | NR_118156.1 | 1409 | 321 | 724 | 28.67 | 641.9 | 0 | 385/404 | 95 | 0/404 | 0 | Plus | Plus | Bacteria; Firmicutes; Clostridia; Eubacteriales; Oscillospiraceae; Oscillibacter; Oscillibacter ruminantium | Oscillibacter ruminantium | Oscillibacter | Oscillospiraceae | Eubacteriales | Clostridia | Firmicutes | Bacteria |
| ASV799 | 422 | 4 | 1 | 422 | 100 | NR_044864.1 | Hallella seregens ATCC 51272 16S ribosomal RNA, partial sequence | NR_044864.1 | 1463 | 335 | 756 | 28.84 | 558.8 | 8E-159 | 382/422 | 90 | 0/422 | 0 | Plus | Plus | Bacteria; Bacteroidetes; Bacteroidia; Bacteroidales; Prevotellaceae; Prevotella; Hallella seregens | Hallella seregens | Prevotella | Prevotellaceae | Bacteroidales | Bacteroidia | Bacteroidetes | Bacteria |
| ASV800 | 427 | 2 | 1 | 427 | 100 | NR_029106.1 | Lactobacillus delbrueckii subsp. indicus strain NCC725 16S ribosomal RNA, partial sequence | NR_029106.1 | 1515 | 348 | 774 | 28.18 | 784.1 | 0 | 426/427 | 99 | 0/427 | 0 | Plus | Plus | Bacteria; Firmicutes; Bacilli; Lactobacillales; Lactobacillaceae; Lactobacillus; Lactobacillus delbrueckii | Lactobacillus delbrueckii | Lactobacillus | Lactobacillaceae | Lactobacillales | Bacilli | Firmicutes | Bacteria |
| ASV801 | 404 | 1 | 1 | 404 | 100 | NR_118156.1 | Oscillibacter ruminantium GH1 16S ribosomal RNA, partial sequence | NR_118156.1 | 1409 | 321 | 724 | 28.67 | 619.7 | 3E-177 | 381/404 | 94 | 0/404 | 0 | Plus | Plus | Bacteria; Firmicutes; Clostridia; Eubacteriales; Oscillospiraceae; Oscillibacter; Oscillibacter ruminantium | Oscillibacter ruminantium | Oscillibacter | Oscillospiraceae | Eubacteriales | Clostridia | Firmicutes | Bacteria |
| ASV802 | 402 | 1 | 1 | 402 | 100 | NR_025670.1 | Pseudoflavonifractor capillosus ATCC 29799 16S ribosomal RNA, partial sequence | NR_025670.1 | 1483 | 346 | 750 | 27.31 | 496 | 6E-140 | 362/407 | 88 | 7/407 | 1 | Plus | Plus | Bacteria; Firmicutes; Clostridia; Eubacteriales; Oscillospiraceae; Pseudoflavonifractor; Pseudoflavonifractor capillosus | Pseudoflavonifractor capillosus | Pseudoflavonifractor | Oscillospiraceae | Eubacteriales | Clostridia | Firmicutes | Bacteria |
| ASV803 | 427 | 1 | 1 | 427 | 100 | NR_028862.1 | Allisonella histaminiformans strain MR2 16S ribosomal RNA, partial sequence | NR_028862.1 | 1537 | 376 | 802 | 27.78 | 784.1 | 0 | 426/427 | 99 | 0/427 | 0 | Plus | Plus | Bacteria; Firmicutes; Negativicutes; Veillonellales; Veillonellaceae; Allisonella; Allisonella histaminiformans | Allisonella histaminiformans | Allisonella | Veillonellaceae | Veillonellales | Negativicutes | Firmicutes | Bacteria |
| ASV804 | 404 | 1 | 1 | 404 | 100 | NR_118156.1 | Oscillibacter ruminantium GH1 16S ribosomal RNA, partial sequence | NR_118156.1 | 1409 | 321 | 724 | 28.67 | 614.2 | 2E-175 | 381/405 | 94 | 2/405 | 0 | Plus | Plus | Bacteria; Firmicutes; Clostridia; Eubacteriales; Oscillospiraceae; Oscillibacter; Oscillibacter ruminantium | Oscillibacter ruminantium | Oscillibacter | Oscillospiraceae | Eubacteriales | Clostridia | Firmicutes | Bacteria |
| ASV805 | 422 | 1 | 1 | 422 | 100 | NR_113411.1 | Prevotella copri DSM 18205 strain JCM 13464 16S ribosomal RNA, partial sequence | NR_113411.1 | 1493 | 358 | 779 | 28.27 | 747.2 | 0 | 416/422 | 98 | 0/422 | 0 | Plus | Plus | Bacteria; Bacteroidetes; Bacteroidia; Bacteroidales; Prevotellaceae; Prevotella; Prevotella copri | Prevotella copri | Prevotella | Prevotellaceae | Bacteroidales | Bacteroidia | Bacteroidetes | Bacteria |
| ASV806 | 403 | 1 | 3 | 403 | 99.5 | NR_125464.1 | Anaerobacterium chartisolvens strain T-1-35 16S ribosomal RNA, partial sequence | NR_125464.1 | 1451 | 344 | 745 | 27.71 | 481.2 | 2E-135 | 357/404 | 88 | 5/404 | 1 | Plus | Plus | Bacteria; Firmicutes; Clostridia; Eubacteriales; Oscillospiraceae; Anaerobacterium; Anaerobacterium chartisolvens | Anaerobacterium chartisolvens | Anaerobacterium | Oscillospiraceae | Eubacteriales | Clostridia | Firmicutes | Bacteria |
| ASV807 | 422 | 1 | 1 | 422 | 100 | NR_024816.1 | Prevotella salivae strain EPSA11 16S ribosomal RNA, partial sequence | NR_024816.1 | 1493 | 356 | 777 | 28.27 | 603.1 | 3E-172 | 390/422 | 92 | 0/422 | 0 | Plus | Plus | Bacteria; Bacteroidetes; Bacteroidia; Bacteroidales; Prevotellaceae; Prevotella; Prevotella salivae | Prevotella salivae | Prevotella | Prevotellaceae | Bacteroidales | Bacteroidia | Bacteroidetes | Bacteria |
| ASV808 | 402 | 1 | 1 | 402 | 100 | NR_074634.1 | [Eubacterium] rectale ATCC 33656 16S ribosomal RNA, partial sequence | NR_074634.1 | 1521 | 367 | 768 | 26.43 | 737.9 | 0 | 401/402 | 99 | 0/402 | 0 | Plus | Plus | Bacteria; Firmicutes; Clostridia; Eubacteriales; Lachnospiraceae; ; [Eubacterium] rectale | [Eubacterium] rectale | | Lachnospiraceae | Eubacteriales | Clostridia | Firmicutes | Bacteria |
| ASV809 | 402 | 5 | 1 | 402 | 100 | NR_147396.1 | Lachnoclostridium pacaense strain Marseille-P3100 16S ribosomal RNA, partial sequence | NR_147396.1 | 1453 | 345 | 746 | 27.67 | 643.8 | 0 | 384/402 | 95 | 0/402 | 0 | Plus | Plus | Bacteria; Firmicutes; Clostridia; Eubacteriales; Lachnospiraceae; Lachnoclostridium; Lachnoclostridium pacaense | Lachnoclostridium pacaense | Lachnoclostridium | Lachnospiraceae | Eubacteriales | Clostridia | Firmicutes | Bacteria |
| ASV810 | 402 | 1 | 1 | 402 | 100 | NR_042832.1 | Roseburia faecis strain M72/1 16S ribosomal RNA, partial sequence | NR_042832.1 | 1456 | 347 | 749 | 27.68 | 688.1 | 0 | 393/403 | 97 | 1/403 | 0 | Plus | Plus | Bacteria; Firmicutes; Clostridia; Eubacteriales; Lachnospiraceae; Roseburia; Roseburia faecis | Roseburia faecis | Roseburia | Lachnospiraceae | Eubacteriales | Clostridia | Firmicutes | Bacteria |
| ASV812 | 402 | 1 | 1 | 402 | 100 | NR_025917.1 | Hungateiclostridium cellulolyticum strain CD2 16S ribosomal RNA, partial sequence | NR_025917.1 | 1464 | 326 | 726 | 27.39 | 531.1 | 2E-150 | 364/402 | 90 | 1/402 | 0 | Plus | Plus | Bacteria; Firmicutes; Clostridia; Eubacteriales; Oscillospiraceae; Acetivibrio; Acetivibrio cellulolyticus | Acetivibrio cellulolyticus | Acetivibrio | Oscillospiraceae | Eubacteriales | Clostridia | Firmicutes | Bacteria |
| ASV813 | 422 | 2 | 1 | 422 | 100 | NR_113119.1 | Prevotella oris strain JCM 8540 16S ribosomal RNA, partial sequence | NR_113119.1 | 1481 | 345 | 766 | 28.49 | 597.6 | 2E-170 | 391/424 | 92 | 4/424 | 0 | Plus | Plus | Bacteria; Bacteroidetes; Bacteroidia; Bacteroidales; Prevotellaceae; Prevotella; Prevotella oris | Prevotella oris | Prevotella | Prevotellaceae | Bacteroidales | Bacteroidia | Bacteroidetes | Bacteria |
| ASV814 | 422 | 1 | 1 | 422 | 100 | NR_041446.1 | Bacteroides barnesiae strain BL2 16S ribosomal RNA, partial sequence | NR_041446.1 | 1456 | 340 | 761 | 28.98 | 592 | 7E-169 | 391/425 | 92 | 6/425 | 1 | Plus | Plus | Bacteria; Bacteroidetes; Bacteroidia; Bacteroidales; ; Phocaeicola; Phocaeicola barnesiae | Phocaeicola barnesiae | Phocaeicola | | Bacteroidales | Bacteroidia | Bacteroidetes | Bacteria |
| ASV815 | 422 | 1 | 1 | 422 | 100 | NR_170509.1 | Duncaniella freteri strain TLL-A3 16S ribosomal RNA, partial sequence | NR_170509.1 | 1538 | 364 | 784 | 27.37 | 551.4 | 1E-156 | 383/424 | 90 | 5/424 | 1 | Plus | Plus | Bacteria; Bacteroidetes; Bacteroidia; Bacteroidales; Muribaculaceae; Duncaniella; Duncaniella freteri | Duncaniella freteri | Duncaniella | Muribaculaceae | Bacteroidales | Bacteroidia | Bacteroidetes | Bacteria |
| ASV816 | 422 | 5 | 1 | 422 | 100 | NR_044864.1 | Hallella seregens ATCC 51272 16S ribosomal RNA, partial sequence | NR_044864.1 | 1463 | 335 | 756 | 28.84 | 614.2 | 2E-175 | 393/423 | 92 | 2/423 | 0 | Plus | Plus | Bacteria; Bacteroidetes; Bacteroidia; Bacteroidales; Prevotellaceae; Prevotella; Hallella seregens | Hallella seregens | Prevotella | Prevotellaceae | Bacteroidales | Bacteroidia | Bacteroidetes | Bacteria |
| ASV817 | 422 | 1 | 1 | 422 | 100 | NR_040877.1 | Prevotella copri DSM 18205 strain CB7 16S ribosomal RNA, partial sequence | NR_040877.1 | 1494 | 358 | 779 | 28.25 | 699.2 | 0 | 407/422 | 96 | 0/422 | 0 | Plus | Plus | Bacteria; Bacteroidetes; Bacteroidia; Bacteroidales; Prevotellaceae; Prevotella; Prevotella copri | Prevotella copri | Prevotella | Prevotellaceae | Bacteroidales | Bacteroidia | Bacteroidetes | Bacteria |
| ASV818 | 404 | 1 | 1 | 404 | 100 | NR_118156.1 | Oscillibacter ruminantium GH1 16S ribosomal RNA, partial sequence | NR_118156.1 | 1409 | 321 | 724 | 28.67 | 636.4 | 0 | 384/404 | 95 | 0/404 | 0 | Plus | Plus | Bacteria; Firmicutes; Clostridia; Eubacteriales; Oscillospiraceae; Oscillibacter; Oscillibacter ruminantium | Oscillibacter ruminantium | Oscillibacter | Oscillospiraceae | Eubacteriales | Clostridia | Firmicutes | Bacteria |
| ASV819 | 404 | 1 | 1 | 404 | 100 | NR_147370.1 | Pseudoflavonifractor phocaeensis strain Marseille-P3064 16S ribosomal RNA, partial sequence | NR_147370.1 | 1495 | 366 | 770 | 27.09 | 564.3 | 2E-160 | 372/405 | 91 | 1/405 | 0 | Plus | Plus | Bacteria; Firmicutes; Clostridia; Eubacteriales; Oscillospiraceae; Pseudoflavonifractor; Pseudoflavonifractor phocaeensis | Pseudoflavonifractor phocaeensis | Pseudoflavonifractor | Oscillospiraceae | Eubacteriales | Clostridia | Firmicutes | Bacteria |
| ASV820 | 402 | 1 | 1 | 402 | 100 | NR_102884.1 | Ruminococcus champanellensis 18P13 = JCM 17042 16S ribosomal RNA, partial sequence | NR_102884.1 | 1494 | 348 | 749 | 26.91 | 704.7 | 0 | 395/402 | 98 | 0/402 | 0 | Plus | Plus | Bacteria; Firmicutes; Clostridia; Eubacteriales; Oscillospiraceae; Ruminococcus; Ruminococcus champanellensis | Ruminococcus champanellensis | Ruminococcus | Oscillospiraceae | Eubacteriales | Clostridia | Firmicutes | Bacteria |
| ASV821 | 427 | 1 | 1 | 426 | 99.77 | NR_027596.1 | Mitsuokella multacida strain DSM 20544 16S ribosomal RNA, partial sequence | NR_027596.1 | 1511 | 346 | 771 | 28.19 | 651.1 | 0 | 401/426 | 94 | 0/426 | 0 | Plus | Plus | Bacteria; Firmicutes; Negativicutes; Selenomonadales; Selenomonadaceae; Mitsuokella; Mitsuokella multacida | Mitsuokella multacida | Mitsuokella | Selenomonadaceae | Selenomonadales | Negativicutes | Firmicutes | Bacteria |
| ASV822 | 422 | 1 | 1 | 422 | 100 | NR_146018.1 | Paludibacter jiangxiensis strain NM7 16S ribosomal RNA, partial sequence | NR_146018.1 | 1469 | 340 | 761 | 28.73 | 497.9 | 2E-140 | 375/426 | 88 | 8/426 | 1 | Plus | Plus | Bacteria; Bacteroidetes; Bacteroidia; Bacteroidales; Paludibacteraceae; Paludibacter; Paludibacter jiangxiensis | Paludibacter jiangxiensis | Paludibacter | Paludibacteraceae | Bacteroidales | Bacteroidia | Bacteroidetes | Bacteria |
| ASV823 | 422 | 2 | 1 | 422 | 100 | NR_074515.1 | Bacteroides vulgatus ATCC 8482 16S ribosomal RNA, partial sequence | NR_074515.1 | 1510 | 367 | 788 | 27.95 | 780.4 | 0 | 422/422 | 100 | 0/422 | 0 | Plus | Plus | Bacteria; Bacteroidetes; Bacteroidia; Bacteroidales; ; Phocaeicola; Phocaeicola vulgatus | Phocaeicola vulgatus | Phocaeicola | | Bacteroidales | Bacteroidia | Bacteroidetes | Bacteria |
| ASV824 | 405 | 1 | 1 | 405 | 100 | NR_044972.1 | Sporobacter termitidis strain SYR 16S ribosomal RNA, partial sequence | NR_044972.1 | 1506 | 348 | 752 | 26.89 | 542.2 | 7E-154 | 368/406 | 90 | 2/406 | 0 | Plus | Plus | Bacteria; Firmicutes; Clostridia; Eubacteriales; Oscillospiraceae; Sporobacter; Sporobacter termitidis | Sporobacter termitidis | Sporobacter | Oscillospiraceae | Eubacteriales | Clostridia | Firmicutes | Bacteria |
| ASV825 | 422 | 2 | 1 | 422 | 100 | NR_074376.1 | Parabacteroides distasonis ATCC 8503 16S ribosomal RNA, partial sequence | NR_074376.1 | 1497 | 368 | 789 | 28.19 | 569.9 | 3E-162 | 384/422 | 90 | 0/422 | 0 | Plus | Plus | Bacteria; Bacteroidetes; Bacteroidia; Bacteroidales; Tannerellaceae; Parabacteroides; Parabacteroides distasonis | Parabacteroides distasonis | Parabacteroides | Tannerellaceae | Bacteroidales | Bacteroidia | Bacteroidetes | Bacteria |
| ASV826 | 422 | 1 | 1 | 422 | 100 | NR_027196.1 | Bacteroides stercoris ATCC 43183 16S ribosomal RNA, partial sequence | NR_027196.1 | 1342 | 334 | 755 | 31.45 | 671.5 | 0 | 402/422 | 95 | 0/422 | 0 | Plus | Plus | Bacteria; Bacteroidetes; Bacteroidia; Bacteroidales; Bacteroidaceae; Bacteroides; Bacteroides stercoris | Bacteroides stercoris | Bacteroides | Bacteroidaceae | Bacteroidales | Bacteroidia | Bacteroidetes | Bacteria |
| ASV827 | 403 | 1 | 3 | 403 | 99.5 | NR_044490.1 | Butyricicoccus pullicaecorum strain 25-3 16S ribosomal RNA, partial sequence | NR_044490.1 | 1482 | 368 | 770 | 27.19 | 693.6 | 0 | 394/403 | 97 | 2/403 | 0 | Plus | Plus | Bacteria; Firmicutes; Clostridia; Eubacteriales; Clostridiaceae; Butyricicoccus; Butyricicoccus pullicaecorum | Butyricicoccus pullicaecorum | Butyricicoccus | Clostridiaceae | Eubacteriales | Clostridia | Firmicutes | Bacteria |
| ASV828 | 422 | 2 | 1 | 422 | 100 | NR_113076.1 | Parabacteroides goldsteinii DSM 19448 = WAL 12034 strain JCM 13446 16S ribosomal RNA, partial sequence | NR_113076.1 | 1476 | 341 | 762 | 28.59 | 497.9 | 2E-140 | 372/423 | 87 | 2/423 | 0 | Plus | Plus | Bacteria; Bacteroidetes; Bacteroidia; Bacteroidales; Tannerellaceae; Parabacteroides; Parabacteroides goldsteinii | Parabacteroides goldsteinii | Parabacteroides | Tannerellaceae | Bacteroidales | Bacteroidia | Bacteroidetes | Bacteria |
| ASV829 | 423 | 1 | 1 | 423 | 100 | NR_144750.1 | Prevotellamassilia timonensis strain Marseille-P2831 16S ribosomal RNA, partial sequence | NR_144750.1 | 1493 | 358 | 779 | 28.27 | 569.9 | 3E-162 | 387/425 | 91 | 5/425 | 1 | Plus | Plus | Bacteria; Bacteroidetes; Bacteroidia; Bacteroidales; Prevotellaceae; Prevotellamassilia; Prevotellamassilia timonensis | Prevotellamassilia timonensis | Prevotellamassilia | Prevotellaceae | Bacteroidales | Bacteroidia | Bacteroidetes | Bacteria |
| ASV830 | 427 | 1 | 1 | 426 | 99.77 | NR_044111.1 | Selenomonas bovis strain WG 16S ribosomal RNA, partial sequence | NR_044111.1 | 1525 | 370 | 795 | 27.93 | 754.6 | 0 | 420/426 | 98 | 0/426 | 0 | Plus | Plus | Bacteria; Firmicutes; Negativicutes; Selenomonadales; Selenomonadaceae; Selenomonas; Selenomonas bovis | Selenomonas bovis | Selenomonas | Selenomonadaceae | Selenomonadales | Negativicutes | Firmicutes | Bacteria |
| ASV831 | 402 | 5 | 1 | 402 | 100 | NR_042200.1 | [Clostridium] asparagiforme strain N6 16S ribosomal RNA, partial sequence | NR_042200.1 | 1468 | 335 | 736 | 27.38 | 638.2 | 0 | 386/405 | 95 | 6/405 | 1 | Plus | Plus | Bacteria; Firmicutes; Clostridia; Eubacteriales; Lachnospiraceae; Enterocloster; Enterocloster asparagiformis | Enterocloster asparagiformis | Enterocloster | Lachnospiraceae | Eubacteriales | Clostridia | Firmicutes | Bacteria |
| ASV832 | 422 | 1 | 1 | 422 | 100 | NR_113411.1 | Prevotella copri DSM 18205 strain JCM 13464 16S ribosomal RNA, partial sequence | NR_113411.1 | 1493 | 358 | 779 | 28.27 | 708.4 | 0 | 409/422 | 96 | 0/422 | 0 | Plus | Plus | Bacteria; Bacteroidetes; Bacteroidia; Bacteroidales; Prevotellaceae; Prevotella; Prevotella copri | Prevotella copri | Prevotella | Prevotellaceae | Bacteroidales | Bacteroidia | Bacteroidetes | Bacteria |
| ASV833 | 422 | 2 | 1 | 422 | 100 | NR_074376.1 | Parabacteroides distasonis ATCC 8503 16S ribosomal RNA, partial sequence | NR_074376.1 | 1497 | 368 | 789 | 28.19 | 575.4 | 7E-164 | 385/422 | 91 | 0/422 | 0 | Plus | Plus | Bacteria; Bacteroidetes; Bacteroidia; Bacteroidales; Tannerellaceae; Parabacteroides; Parabacteroides distasonis | Parabacteroides distasonis | Parabacteroides | Tannerellaceae | Bacteroidales | Bacteroidia | Bacteroidetes | Bacteria |
| ASV834 | 403 | 1 | 3 | 403 | 99.5 | NR_125677.1 | Vallitalea pronyensis strain FatNI3 16S ribosomal RNA, partial sequence | NR_125677.1 | 1480 | 365 | 764 | 27.03 | 462.8 | 6E-130 | 352/402 | 87 | 3/402 | 0 | Plus | Plus | Bacteria; Firmicutes; Clostridia; Eubacteriales; Vallitaleaceae; Vallitalea; Vallitalea pronyensis | Vallitalea pronyensis | Vallitalea | Vallitaleaceae | Eubacteriales | Clostridia | Firmicutes | Bacteria |
| ASV835 | 422 | 1 | 1 | 422 | 100 | NR_144750.1 | Prevotellamassilia timonensis strain Marseille-P2831 16S ribosomal RNA, partial sequence | NR_144750.1 | 1493 | 358 | 779 | 28.27 | 547.7 | 2E-155 | 380/422 | 90 | 0/422 | 0 | Plus | Plus | Bacteria; Bacteroidetes; Bacteroidia; Bacteroidales; Prevotellaceae; Prevotellamassilia; Prevotellamassilia timonensis | Prevotellamassilia timonensis | Prevotellamassilia | Prevotellaceae | Bacteroidales | Bacteroidia | Bacteroidetes | Bacteria |
| ASV836 | 402 | 5 | 1 | 402 | 100 | NR_113388.1 | Clostridium beijerinckii strain JCM 1390 16S ribosomal RNA, partial sequence | NR_113388.1 | 1473 | 348 | 749 | 27.29 | 688.1 | 0 | 392/402 | 97 | 0/402 | 0 | Plus | Plus | Bacteria; Firmicutes; Clostridia; Eubacteriales; Clostridiaceae; Clostridium; Clostridium beijerinckii | Clostridium beijerinckii | Clostridium | Clostridiaceae | Eubacteriales | Clostridia | Firmicutes | Bacteria |
| ASV837 | 404 | 1 | 1 | 404 | 100 | NR_028911.1 | Anaerovorax odorimutans strain NorPut 16S ribosomal RNA, partial sequence | NR_028911.1 | 1488 | 352 | 755 | 27.15 | 520 | 3E-147 | 366/407 | 89 | 6/407 | 1 | Plus | Plus | Bacteria; Firmicutes; Clostridia; Eubacteriales; Clostridiales Family XIII. Incertae Sedis; Anaerovorax; Anaerovorax odorimutans | Anaerovorax odorimutans | Anaerovorax | Clostridiales Family XIII. Incertae Sedis | Eubacteriales | Clostridia | Firmicutes | Bacteria |
| ASV838 | 403 | 1 | 1 | 403 | 100 | NR_104907.1 | Eubacterium coprostanoligenes strain HL 16S ribosomal RNA, partial sequence | NR_104907.1 | 1446 | 332 | 734 | 27.87 | 656.7 | 0 | 387/403 | 96 | 0/403 | 0 | Plus | Plus | Bacteria; Firmicutes; Clostridia; Eubacteriales; Eubacteriaceae; Eubacterium; Eubacterium coprostanoligenes | Eubacterium coprostanoligenes | Eubacterium | Eubacteriaceae | Eubacteriales | Clostridia | Firmicutes | Bacteria |
| ASV839 | 422 | 1 | 1 | 422 | 100 | NR_041342.1 | Parabacteroides distasonis strain JCM 5825 16S ribosomal RNA, partial sequence | NR_041342.1 | 1488 | 353 | 774 | 28.36 | 547.7 | 2E-155 | 380/422 | 90 | 0/422 | 0 | Plus | Plus | Bacteria; Bacteroidetes; Bacteroidia; Bacteroidales; Tannerellaceae; Parabacteroides; Parabacteroides distasonis | Parabacteroides distasonis | Parabacteroides | Tannerellaceae | Bacteroidales | Bacteroidia | Bacteroidetes | Bacteria |
| ASV840 | 422 | 1 | 1 | 422 | 100 | NR_024816.1 | Prevotella salivae strain EPSA11 16S ribosomal RNA, partial sequence | NR_024816.1 | 1493 | 356 | 777 | 28.27 | 608.7 | 7E-174 | 391/422 | 92 | 0/422 | 0 | Plus | Plus | Bacteria; Bacteroidetes; Bacteroidia; Bacteroidales; Prevotellaceae; Prevotella; Prevotella salivae | Prevotella salivae | Prevotella | Prevotellaceae | Bacteroidales | Bacteroidia | Bacteroidetes | Bacteria |
| ASV841 | 405 | 1 | 1 | 405 | 100 | NR_044972.1 | Sporobacter termitidis strain SYR 16S ribosomal RNA, partial sequence | NR_044972.1 | 1506 | 348 | 752 | 26.89 | 536.6 | 3E-152 | 367/406 | 90 | 2/406 | 0 | Plus | Plus | Bacteria; Firmicutes; Clostridia; Eubacteriales; Oscillospiraceae; Sporobacter; Sporobacter termitidis | Sporobacter termitidis | Sporobacter | Oscillospiraceae | Eubacteriales | Clostridia | Firmicutes | Bacteria |
| ASV842 | 404 | 1 | 1 | 404 | 100 | NR_025670.1 | Pseudoflavonifractor capillosus ATCC 29799 16S ribosomal RNA, partial sequence | NR_025670.1 | 1483 | 346 | 750 | 27.31 | 453.5 | 3E-127 | 352/405 | 86 | 1/405 | 0 | Plus | Plus | Bacteria; Firmicutes; Clostridia; Eubacteriales; Oscillospiraceae; Pseudoflavonifractor; Pseudoflavonifractor capillosus | Pseudoflavonifractor capillosus | Pseudoflavonifractor | Oscillospiraceae | Eubacteriales | Clostridia | Firmicutes | Bacteria |
| ASV843 | 426 | 1 | 1 | 426 | 100 | NR_114696.1 | Anaerovibrio lipolyticus DSM 3074 16S ribosomal RNA, partial sequence | NR_114696.1 | 1526 | 372 | 797 | 27.92 | 653 | 0 | 401/426 | 94 | 0/426 | 0 | Plus | Plus | Bacteria; Firmicutes; Negativicutes; Selenomonadales; Selenomonadaceae; Anaerovibrio; Anaerovibrio lipolyticus | Anaerovibrio lipolyticus | Anaerovibrio | Selenomonadaceae | Selenomonadales | Negativicutes | Firmicutes | Bacteria |
| ASV844 | 402 | 1 | 1 | 402 | 100 | NR_118692.1 | Blautia obeum ATCC 29174 16S ribosomal RNA, partial sequence | NR_118692.1 | 1398 | 330 | 731 | 28.76 | 706.5 | 0 | 395/402 | 98 | 0/402 | 0 | Plus | Plus | Bacteria; Firmicutes; Clostridia; Eubacteriales; Lachnospiraceae; Blautia; Blautia obeum | Blautia obeum | Blautia | Lachnospiraceae | Eubacteriales | Clostridia | Firmicutes | Bacteria |
| ASV845 | 404 | 3 | 1 | 404 | 100 | NR_074793.2 | Oscillibacter valericigenes strain Sjm18-20 16S ribosomal RNA, complete sequence | NR_074793.2 | 1530 | 372 | 775 | 26.41 | 647.4 | 0 | 386/404 | 95 | 0/404 | 0 | Plus | Plus | Bacteria; Firmicutes; Clostridia; Eubacteriales; Oscillospiraceae; Oscillibacter; Oscillibacter valericigenes | Oscillibacter valericigenes | Oscillibacter | Oscillospiraceae | Eubacteriales | Clostridia | Firmicutes | Bacteria |
| ASV846 | 427 | 1 | 1 | 427 | 100 | NR_147376.1 | Caecibacter massiliensis strain Marseille-P2974 16S ribosomal RNA, partial sequence | NR_147376.1 | 1530 | 371 | 797 | 27.91 | 785.9 | 0 | 426/427 | 99 | 0/427 | 0 | Plus | Plus | Bacteria; Firmicutes; Negativicutes; Veillonellales; Veillonellaceae; Caecibacter; Caecibacter massiliensis | Caecibacter massiliensis | Caecibacter | Veillonellaceae | Veillonellales | Negativicutes | Firmicutes | Bacteria |
| ASV847 | 405 | 1 | 3 | 405 | 99.51 | NR_125464.1 | Anaerobacterium chartisolvens strain T-1-35 16S ribosomal RNA, partial sequence | NR_125464.1 | 1451 | 344 | 745 | 27.71 | 466.5 | 4E-131 | 353/403 | 87 | 1/403 | 0 | Plus | Plus | Bacteria; Firmicutes; Clostridia; Eubacteriales; Oscillospiraceae; Anaerobacterium; Anaerobacterium chartisolvens | Anaerobacterium chartisolvens | Anaerobacterium | Oscillospiraceae | Eubacteriales | Clostridia | Firmicutes | Bacteria |
| ASV848 | 421 | 2 | 1 | 421 | 100 | NR_113077.1 | Paraprevotella clara strain JCM 14859 16S ribosomal RNA, partial sequence | NR_113077.1 | 1480 | 346 | 766 | 28.45 | 484.9 | 1E-136 | 369/422 | 87 | 2/422 | 0 | Plus | Plus | Bacteria; Bacteroidetes; Bacteroidia; Bacteroidales; Prevotellaceae; Paraprevotella; Paraprevotella clara | Paraprevotella clara | Paraprevotella | Prevotellaceae | Bacteroidales | Bacteroidia | Bacteroidetes | Bacteria |
| ASV849 | 402 | 1 | 1 | 402 | 100 | NR_151894.1 | Anaerotaenia torta strain FH052 16S ribosomal RNA, partial sequence | NR_151894.1 | 1479 | 352 | 753 | 27.18 | 621.6 | 9E-178 | 380/402 | 94 | 0/402 | 0 | Plus | Plus | Bacteria; Firmicutes; Clostridia; Eubacteriales; Lachnospiraceae; Anaerotaenia; Anaerotaenia torta | Anaerotaenia torta | Anaerotaenia | Lachnospiraceae | Eubacteriales | Clostridia | Firmicutes | Bacteria |
| ASV850 | 402 | 2 | 1 | 402 | 100 | NR_118643.1 | Eisenbergiella tayi strain B086562 16S ribosomal RNA, partial sequence | NR_118643.1 | 1390 | 271 | 672 | 28.92 | 621.6 | 9E-178 | 380/402 | 94 | 0/402 | 0 | Plus | Plus | Bacteria; Firmicutes; Clostridia; Eubacteriales; Lachnospiraceae; Eisenbergiella; Eisenbergiella tayi | Eisenbergiella tayi | Eisenbergiella | Lachnospiraceae | Eubacteriales | Clostridia | Firmicutes | Bacteria |
| ASV851 | 422 | 1 | 1 | 422 | 100 | NR_041285.1 | Prevotella multisaccharivorax strain PPPA20 16S ribosomal RNA, partial sequence | NR_041285.1 | 1491 | 356 | 777 | 28.3 | 641.9 | 0 | 398/423 | 94 | 2/423 | 0 | Plus | Plus | Bacteria; Bacteroidetes; Bacteroidia; Bacteroidales; Prevotellaceae; Prevotella; Prevotella multisaccharivorax | Prevotella multisaccharivorax | Prevotella | Prevotellaceae | Bacteroidales | Bacteroidia | Bacteroidetes | Bacteria |
| ASV852 | 426 | 1 | 1 | 425 | 99.77 | NR_029325.1 | Schwartzia succinivorans strain S1-1 16S ribosomal RNA, partial sequence | NR_029325.1 | 1489 | 344 | 768 | 28.54 | 719.5 | 0 | 413/425 | 97 | 0/425 | 0 | Plus | Plus | Bacteria; Firmicutes; Negativicutes; Selenomonadales; Selenomonadaceae; Schwartzia; Schwartzia succinivorans | Schwartzia succinivorans | Schwartzia | Selenomonadaceae | Selenomonadales | Negativicutes | Firmicutes | Bacteria |
| ASV853 | 404 | 1 | 1 | 404 | 100 | NR_118156.1 | Oscillibacter ruminantium GH1 16S ribosomal RNA, partial sequence | NR_118156.1 | 1409 | 321 | 724 | 28.67 | 664.1 | 0 | 389/404 | 96 | 0/404 | 0 | Plus | Plus | Bacteria; Firmicutes; Clostridia; Eubacteriales; Oscillospiraceae; Oscillibacter; Oscillibacter ruminantium | Oscillibacter ruminantium | Oscillibacter | Oscillospiraceae | Eubacteriales | Clostridia | Firmicutes | Bacteria |
| ASV854 | 402 | 3 | 1 | 402 | 100 | NR_114315.1 | Blautia luti DSM 14534 16S ribosomal RNA, partial sequence | NR_114315.1 | 1481 | 353 | 754 | 27.14 | 688.1 | 0 | 392/402 | 97 | 0/402 | 0 | Plus | Plus | Bacteria; Firmicutes; Clostridia; Eubacteriales; Lachnospiraceae; Blautia; Blautia luti | Blautia luti | Blautia | Lachnospiraceae | Eubacteriales | Clostridia | Firmicutes | Bacteria |
| ASV855 | 402 | 1 | 1 | 402 | 100 | NR_026312.1 | Blautia schinkii strain B 16S ribosomal RNA, partial sequence | NR_026312.1 | 1482 | 344 | 745 | 27.13 | 701 | 0 | 394/402 | 98 | 0/402 | 0 | Plus | Plus | Bacteria; Firmicutes; Clostridia; Eubacteriales; Lachnospiraceae; Blautia; Blautia schinkii | Blautia schinkii | Blautia | Lachnospiraceae | Eubacteriales | Clostridia | Firmicutes | Bacteria |
| ASV856 | 427 | 2 | 1 | 427 | 100 | NR_112652.1 | Lactobacillus equicursoris DSM 19284 = JCM 14600 = CIP 110162 strain DI70 16S ribosomal RNA, partial sequence | NR_112652.1 | 1486 | 351 | 777 | 28.73 | 706.5 | 0 | 412/427 | 96 | 0/427 | 0 | Plus | Plus | Bacteria; Firmicutes; Bacilli; Lactobacillales; Lactobacillaceae; Lactobacillus; Lactobacillus equicursoris | Lactobacillus equicursoris | Lactobacillus | Lactobacillaceae | Lactobacillales | Bacilli | Firmicutes | Bacteria |
| ASV857 | 402 | 1 | 1 | 402 | 100 | NR_026106.1 | [Eubacterium] cellulosolvens strain 6 16S ribosomal RNA, partial sequence | NR_026106.1 | 1489 | 333 | 734 | 27 | 671.5 | 0 | 389/402 | 96 | 0/402 | 0 | Plus | Plus | Bacteria; Firmicutes; Clostridia; Eubacteriales; Eubacteriaceae; Eubacterium; [Eubacterium] cellulosolvens | [Eubacterium] cellulosolvens | Eubacterium | Eubacteriaceae | Eubacteriales | Clostridia | Firmicutes | Bacteria |
| ASV858 | 427 | 1 | 1 | 427 | 100 | NR_041611.1 | Lactobacillus ruminis strain NBRC 102161 16S ribosomal RNA, partial sequence | NR_041611.1 | 1488 | 352 | 778 | 28.7 | 784.1 | 0 | 426/427 | 99 | 0/427 | 0 | Plus | Plus | Bacteria; Firmicutes; Bacilli; Lactobacillales; Lactobacillaceae; Ligilactobacillus; Ligilactobacillus ruminis | Ligilactobacillus ruminis | Ligilactobacillus | Lactobacillaceae | Lactobacillales | Bacilli | Firmicutes | Bacteria |
| ASV859 | 422 | 1 | 1 | 422 | 100 | NR_024816.1 | Prevotella salivae strain EPSA11 16S ribosomal RNA, partial sequence | NR_024816.1 | 1493 | 356 | 777 | 28.27 | 630.8 | 2E-180 | 395/422 | 93 | 0/422 | 0 | Plus | Plus | Bacteria; Bacteroidetes; Bacteroidia; Bacteroidales; Prevotellaceae; Prevotella; Prevotella salivae | Prevotella salivae | Prevotella | Prevotellaceae | Bacteroidales | Bacteroidia | Bacteroidetes | Bacteria |
| ASV860 | 404 | 1 | 1 | 404 | 100 | NR_118673.1 | [Eubacterium] hallii strain ATCC 27751 16S ribosomal RNA, partial sequence | NR_118673.1 | 1479 | 371 | 772 | 27.18 | 675.1 | 0 | 390/404 | 96 | 2/404 | 0 | Plus | Plus | Bacteria; Firmicutes; Clostridia; Eubacteriales; Lachnospiraceae; Anaerobutyricum; Anaerobutyricum hallii | Anaerobutyricum hallii | Anaerobutyricum | Lachnospiraceae | Eubacteriales | Clostridia | Firmicutes | Bacteria |
| ASV861 | 403 | 1 | 1 | 403 | 100 | NR_104907.1 | Eubacterium coprostanoligenes strain HL 16S ribosomal RNA, partial sequence | NR_104907.1 | 1446 | 332 | 734 | 27.87 | 562.5 | 6E-160 | 370/403 | 91 | 0/403 | 0 | Plus | Plus | Bacteria; Firmicutes; Clostridia; Eubacteriales; Eubacteriaceae; Eubacterium; Eubacterium coprostanoligenes | Eubacterium coprostanoligenes | Eubacterium | Eubacteriaceae | Eubacteriales | Clostridia | Firmicutes | Bacteria |
| ASV862 | 422 | 1 | 1 | 422 | 100 | NR_113411.1 | Prevotella copri DSM 18205 strain JCM 13464 16S ribosomal RNA, partial sequence | NR_113411.1 | 1493 | 358 | 779 | 28.27 | 763.8 | 0 | 419/422 | 99 | 0/422 | 0 | Plus | Plus | Bacteria; Bacteroidetes; Bacteroidia; Bacteroidales; Prevotellaceae; Prevotella; Prevotella copri | Prevotella copri | Prevotella | Prevotellaceae | Bacteroidales | Bacteroidia | Bacteroidetes | Bacteria |
| ASV863 | 405 | 1 | 1 | 405 | 100 | NR_118554.1 | Intestinimonas butyriciproducens strain SRB-521-5-I 16S ribosomal RNA, partial sequence | NR_118554.1 | 1475 | 346 | 750 | 27.46 | 516.3 | 4E-146 | 363/405 | 89 | 0/405 | 0 | Plus | Plus | Bacteria; Firmicutes; Clostridia; Eubacteriales; ; Intestinimonas; Intestinimonas butyriciproducens | Intestinimonas butyriciproducens | Intestinimonas | | Eubacteriales | Clostridia | Firmicutes | Bacteria |
| ASV864 | 405 | 2 | 1 | 405 | 100 | NG_041947.1 | Ruminiclostridium cellulolyticum H10 16S ribosomal RNA gene, complete sequence | NG_041947.1 | 1641 | 486 | 890 | 24.68 | 538.5 | 9E-153 | 372/410 | 90 | 10/410 | 2 | Plus | Plus | Bacteria; Firmicutes; Clostridia; Eubacteriales; Oscillospiraceae; Ruminiclostridium; Ruminiclostridium cellulolyticum | Ruminiclostridium cellulolyticum | Ruminiclostridium | Oscillospiraceae | Eubacteriales | Clostridia | Firmicutes | Bacteria |
| ASV865 | 402 | 1 | 2 | 402 | 99.75 | NR_027579.1 | Ruminococcus lactaris ATCC 29176 16S ribosomal RNA, partial sequence | NR_027579.1 | 1402 | 334 | 734 | 28.6 | 693.6 | 0 | 392/401 | 97 | 0/401 | 0 | Plus | Plus | Bacteria; Firmicutes; Clostridia; Eubacteriales; Lachnospiraceae; Mediterraneibacter; [Ruminococcus] lactaris | [Ruminococcus] lactaris | Mediterraneibacter | Lachnospiraceae | Eubacteriales | Clostridia | Firmicutes | Bacteria |
| ASV866 | 422 | 4 | 1 | 422 | 100 | NR_116726.1 | Prevotella scopos JCM 17725 strain W2052 16S ribosomal RNA, partial sequence | NR_116726.1 | 1453 | 338 | 759 | 29.04 | 581 | 2E-165 | 387/423 | 91 | 2/423 | 0 | Plus | Plus | Bacteria; Bacteroidetes; Bacteroidia; Bacteroidales; Prevotellaceae; Prevotella; Prevotella scopos | Prevotella scopos | Prevotella | Prevotellaceae | Bacteroidales | Bacteroidia | Bacteroidetes | Bacteria |
| ASV867 | 422 | 2 | 1 | 422 | 100 | NR_113117.1 | Prevotella oralis ATCC 33269 strain JCM 12251 16S ribosomal RNA, partial sequence | NR_113117.1 | 1496 | 361 | 782 | 28.21 | 597.6 | 2E-170 | 390/423 | 92 | 2/423 | 0 | Plus | Plus | Bacteria; Bacteroidetes; Bacteroidia; Bacteroidales; Prevotellaceae; Prevotella; Prevotella oralis | Prevotella oralis | Prevotella | Prevotellaceae | Bacteroidales | Bacteroidia | Bacteroidetes | Bacteria |
| ASV868 | 402 | 1 | 1 | 402 | 100 | NR_026204.1 | [Clostridium] viride strain T2-7 16S ribosomal RNA, partial sequence | NR_026204.1 | 1482 | 345 | 749 | 27.33 | 542.2 | 7E-154 | 368/405 | 90 | 3/405 | 0 | Plus | Plus | Bacteria; Firmicutes; Clostridia; Eubacteriales; Oscillospiraceae; ; [Clostridium] viride | [Clostridium] viride | | Oscillospiraceae | Eubacteriales | Clostridia | Firmicutes | Bacteria |
| ASV869 | 422 | 2 | 1 | 422 | 100 | NR_113119.1 | Prevotella oris strain JCM 8540 16S ribosomal RNA, partial sequence | NR_113119.1 | 1481 | 345 | 766 | 28.49 | 575.4 | 7E-164 | 386/423 | 91 | 2/423 | 0 | Plus | Plus | Bacteria; Bacteroidetes; Bacteroidia; Bacteroidales; Prevotellaceae; Prevotella; Prevotella oris | Prevotella oris | Prevotella | Prevotellaceae | Bacteroidales | Bacteroidia | Bacteroidetes | Bacteria |
| ASV870 | 404 | 1 | 1 | 404 | 100 | NR_151900.1 | Ruthenibacterium lactatiformans strain 585-1 16S ribosomal RNA, partial sequence | NR_151900.1 | 1519 | 368 | 769 | 26.46 | 512.6 | 6E-145 | 365/407 | 89 | 8/407 | 1 | Plus | Plus | Bacteria; Firmicutes; Clostridia; Eubacteriales; Oscillospiraceae; Ruthenibacterium; Ruthenibacterium lactatiformans | Ruthenibacterium lactatiformans | Ruthenibacterium | Oscillospiraceae | Eubacteriales | Clostridia | Firmicutes | Bacteria |
| ASV871 | 404 | 1 | 1 | 404 | 100 | NR_144611.1 | Flintibacter butyricus strain BLS21 16S ribosomal RNA, partial sequence | NR_144611.1 | 808 | 299 | 703 | 50.12 | 514.5 | 2E-145 | 365/407 | 89 | 5/407 | 1 | Plus | Plus | Bacteria; Firmicutes; Clostridia; Eubacteriales; ; Flintibacter; Flintibacter butyricus | Flintibacter butyricus | Flintibacter | | Eubacteriales | Clostridia | Firmicutes | Bacteria |
| ASV872 | 427 | 1 | 4 | 427 | 99.3 | NR_146687.1 | Breznakia pachnodae strain Pei061 16S ribosomal RNA, partial sequence | NR_146687.1 | 1499 | 370 | 792 | 28.22 | 433.2 | 5E-121 | 364/427 | 85 | 7/427 | 1 | Plus | Plus | Bacteria; Firmicutes; Erysipelotrichia; Erysipelotrichales; Erysipelotrichaceae; Breznakia; Breznakia pachnodae | Breznakia pachnodae | Breznakia | Erysipelotrichaceae | Erysipelotrichales | Erysipelotrichia | Firmicutes | Bacteria |
| ASV873 | 423 | 1 | 1 | 423 | 100 | NR_144750.1 | Prevotellamassilia timonensis strain Marseille-P2831 16S ribosomal RNA, partial sequence | NR_144750.1 | 1493 | 358 | 779 | 28.27 | 564.3 | 2E-160 | 386/425 | 90 | 5/425 | 1 | Plus | Plus | Bacteria; Bacteroidetes; Bacteroidia; Bacteroidales; Prevotellaceae; Prevotellamassilia; Prevotellamassilia timonensis | Prevotellamassilia timonensis | Prevotellamassilia | Prevotellaceae | Bacteroidales | Bacteroidia | Bacteroidetes | Bacteria |
| ASV874 | 422 | 1 | 1 | 422 | 100 | NR_024815.1 | Prevotella shahii strain EHS11 16S ribosomal RNA, partial sequence | NR_024815.1 | 1490 | 356 | 777 | 28.32 | 603.1 | 3E-172 | 392/424 | 92 | 4/424 | 0 | Plus | Plus | Bacteria; Bacteroidetes; Bacteroidia; Bacteroidales; Prevotellaceae; Prevotella; Prevotella shahii | Prevotella shahii | Prevotella | Prevotellaceae | Bacteroidales | Bacteroidia | Bacteroidetes | Bacteria |
| ASV875 | 404 | 1 | 1 | 404 | 100 | NR_118156.1 | Oscillibacter ruminantium GH1 16S ribosomal RNA, partial sequence | NR_118156.1 | 1409 | 321 | 724 | 28.67 | 647.4 | 0 | 386/404 | 95 | 0/404 | 0 | Plus | Plus | Bacteria; Firmicutes; Clostridia; Eubacteriales; Oscillospiraceae; Oscillibacter; Oscillibacter ruminantium | Oscillibacter ruminantium | Oscillibacter | Oscillospiraceae | Eubacteriales | Clostridia | Firmicutes | Bacteria |
| ASV876 | 405 | 1 | 1 | 405 | 100 | NR_044972.1 | Sporobacter termitidis strain SYR 16S ribosomal RNA, partial sequence | NR_044972.1 | 1506 | 348 | 752 | 26.89 | 553.3 | 3E-157 | 370/406 | 91 | 2/406 | 0 | Plus | Plus | Bacteria; Firmicutes; Clostridia; Eubacteriales; Oscillospiraceae; Sporobacter; Sporobacter termitidis | Sporobacter termitidis | Sporobacter | Oscillospiraceae | Eubacteriales | Clostridia | Firmicutes | Bacteria |
| ASV877 | 422 | 2 | 1 | 422 | 100 | NR_113119.1 | Prevotella oris strain JCM 8540 16S ribosomal RNA, partial sequence | NR_113119.1 | 1481 | 345 | 766 | 28.49 | 641.9 | 0 | 398/423 | 94 | 2/423 | 0 | Plus | Plus | Bacteria; Bacteroidetes; Bacteroidia; Bacteroidales; Prevotellaceae; Prevotella; Prevotella oris | Prevotella oris | Prevotella | Prevotellaceae | Bacteroidales | Bacteroidia | Bacteroidetes | Bacteria |
| ASV878 | 422 | 1 | 1 | 422 | 100 | NR_041364.1 | Prevotella stercorea DSM 18206 strain CB35 16S ribosomal RNA, partial sequence | NR_041364.1 | 1488 | 354 | 775 | 28.36 | 608.7 | 7E-174 | 391/422 | 92 | 0/422 | 0 | Plus | Plus | Bacteria; Bacteroidetes; Bacteroidia; Bacteroidales; Prevotellaceae; Prevotella; Prevotella stercorea | Prevotella stercorea | Prevotella | Prevotellaceae | Bacteroidales | Bacteroidia | Bacteroidetes | Bacteria |
| ASV879 | 422 | 1 | 1 | 422 | 100 | NR_041364.1 | Prevotella stercorea DSM 18206 strain CB35 16S ribosomal RNA, partial sequence | NR_041364.1 | 1488 | 354 | 775 | 28.36 | 719.5 | 0 | 411/422 | 97 | 0/422 | 0 | Plus | Plus | Bacteria; Bacteroidetes; Bacteroidia; Bacteroidales; Prevotellaceae; Prevotella; Prevotella stercorea | Prevotella stercorea | Prevotella | Prevotellaceae | Bacteroidales | Bacteroidia | Bacteroidetes | Bacteria |
| ASV880 | 403 | 1 | 1 | 403 | 100 | NR_151900.1 | Ruthenibacterium lactatiformans strain 585-1 16S ribosomal RNA, partial sequence | NR_151900.1 | 1519 | 368 | 769 | 26.46 | 516.3 | 4E-146 | 365/406 | 89 | 7/406 | 1 | Plus | Plus | Bacteria; Firmicutes; Clostridia; Eubacteriales; Oscillospiraceae; Ruthenibacterium; Ruthenibacterium lactatiformans | Ruthenibacterium lactatiformans | Ruthenibacterium | Oscillospiraceae | Eubacteriales | Clostridia | Firmicutes | Bacteria |
| ASV881 | 401 | 1 | 1 | 401 | 100 | NR_159296.1 | Aminipila butyrica strain FH042 16S ribosomal RNA, partial sequence | NR_159296.1 | 1460 | 352 | 755 | 27.67 | 610.5 | 2E-174 | 380/404 | 94 | 3/404 | 0 | Plus | Plus | Bacteria; Firmicutes; Clostridia; Eubacteriales; Clostridiales Family XIII. Incertae Sedis; Aminipila; Aminipila butyrica | Aminipila butyrica | Aminipila | Clostridiales Family XIII. Incertae Sedis | Eubacteriales | Clostridia | Firmicutes | Bacteria |
| ASV882 | 422 | 1 | 1 | 422 | 100 | NR_040877.1 | Prevotella copri DSM 18205 strain CB7 16S ribosomal RNA, partial sequence | NR_040877.1 | 1494 | 358 | 779 | 28.25 | 754.6 | 0 | 417/422 | 98 | 0/422 | 0 | Plus | Plus | Bacteria; Bacteroidetes; Bacteroidia; Bacteroidales; Prevotellaceae; Prevotella; Prevotella copri | Prevotella copri | Prevotella | Prevotellaceae | Bacteroidales | Bacteroidia | Bacteroidetes | Bacteria |
| ASV883 | 423 | 1 | 1 | 423 | 100 | NR_144750.1 | Prevotellamassilia timonensis strain Marseille-P2831 16S ribosomal RNA, partial sequence | NR_144750.1 | 1493 | 358 | 779 | 28.27 | 575.4 | 7E-164 | 388/425 | 91 | 5/425 | 1 | Plus | Plus | Bacteria; Bacteroidetes; Bacteroidia; Bacteroidales; Prevotellaceae; Prevotellamassilia; Prevotellamassilia timonensis | Prevotellamassilia timonensis | Prevotellamassilia | Prevotellaceae | Bacteroidales | Bacteroidia | Bacteroidetes | Bacteria |
| ASV884 | 402 | 2 | 3 | 402 | 99.5 | NR_148837.1 | Abyssivirga alkaniphila strain L81 16S ribosomal RNA, partial sequence | NR_148837.1 | 1520 | 366 | 765 | 26.32 | 484.9 | 1E-136 | 356/402 | 88 | 4/402 | 0 | Plus | Plus | Bacteria; Firmicutes; Clostridia; Eubacteriales; Lachnospiraceae; Abyssivirga; Abyssivirga alkaniphila | Abyssivirga alkaniphila | Abyssivirga | Lachnospiraceae | Eubacteriales | Clostridia | Firmicutes | Bacteria |
| ASV885 | 423 | 1 | 1 | 423 | 100 | NR_147368.1 | Millionella massiliensis strain Marseille-P3215 16S ribosomal RNA, partial sequence | NR_147368.1 | 1492 | 355 | 776 | 28.28 | 448 | 2E-125 | 368/428 | 85 | 11/428 | 2 | Plus | Plus | Bacteria; Bacteroidetes; Bacteroidia; Bacteroidales; Rikenellaceae; Millionella; Millionella massiliensis | Millionella massiliensis | Millionella | Rikenellaceae | Bacteroidales | Bacteroidia | Bacteroidetes | Bacteria |
| ASV886 | 408 | 5 | 1 | 408 | 100 | NR_134781.1 | Olsenella scatoligenes strain SK9K4 16S ribosomal RNA, partial sequence | NR_134781.1 | 1469 | 348 | 755 | 27.77 | 627.1 | 2E-179 | 385/408 | 94 | 0/408 | 0 | Plus | Plus | Bacteria; Actinobacteria; Coriobacteriia; Coriobacteriales; Atopobiaceae; Olsenella; Olsenella scatoligenes | Olsenella scatoligenes | Olsenella | Atopobiaceae | Coriobacteriales | Coriobacteriia | Actinobacteria | Bacteria |
| ASV887 | 403 | 3 | 1 | 403 | 100 | NR_074809.1 | Roseburia hominis A2-183 16S ribosomal RNA, partial sequence | NR_074809.1 | 1535 | 367 | 769 | 26.25 | 734.2 | 0 | 401/403 | 99 | 0/403 | 0 | Plus | Plus | Bacteria; Firmicutes; Clostridia; Eubacteriales; Lachnospiraceae; Roseburia; Roseburia hominis | Roseburia hominis | Roseburia | Lachnospiraceae | Eubacteriales | Clostridia | Firmicutes | Bacteria |
| ASV888 | 427 | 1 | 1 | 427 | 100 | NR_028840.1 | Mitsuokella jalaludinii strain M9 16S ribosomal RNA, partial sequence | NR_028840.1 | 1543 | 362 | 788 | 27.67 | 784.1 | 0 | 426/427 | 99 | 0/427 | 0 | Plus | Plus | Bacteria; Firmicutes; Negativicutes; Selenomonadales; Selenomonadaceae; Mitsuokella; Mitsuokella jalaludinii | Mitsuokella jalaludinii | Mitsuokella | Selenomonadaceae | Selenomonadales | Negativicutes | Firmicutes | Bacteria |
| ASV889 | 402 | 3 | 1 | 402 | 100 | NR_156081.1 | Kineothrix alysoides strain KNHs209 16S ribosomal RNA, partial sequence | NR_156081.1 | 1411 | 353 | 754 | 28.49 | 699.2 | 0 | 394/402 | 98 | 0/402 | 0 | Plus | Plus | Bacteria; Firmicutes; Clostridia; Eubacteriales; Lachnospiraceae; Kineothrix; Kineothrix alysoides | Kineothrix alysoides | Kineothrix | Lachnospiraceae | Eubacteriales | Clostridia | Firmicutes | Bacteria |
| ASV891 | 423 | 1 | 1 | 423 | 100 | NR_147368.1 | Millionella massiliensis strain Marseille-P3215 16S ribosomal RNA, partial sequence | NR_147368.1 | 1492 | 355 | 776 | 28.28 | 453.5 | 4E-127 | 369/428 | 86 | 11/428 | 2 | Plus | Plus | Bacteria; Bacteroidetes; Bacteroidia; Bacteroidales; Rikenellaceae; Millionella; Millionella massiliensis | Millionella massiliensis | Millionella | Rikenellaceae | Bacteroidales | Bacteroidia | Bacteroidetes | Bacteria |
| ASV893 | 402 | 1 | 1 | 402 | 100 | NR_074634.1 | [Eubacterium] rectale ATCC 33656 16S ribosomal RNA, partial sequence | NR_074634.1 | 1521 | 367 | 768 | 26.43 | 721.3 | 0 | 398/402 | 99 | 0/402 | 0 | Plus | Plus | Bacteria; Firmicutes; Clostridia; Eubacteriales; Lachnospiraceae; ; [Eubacterium] rectale | [Eubacterium] rectale | | Lachnospiraceae | Eubacteriales | Clostridia | Firmicutes | Bacteria |
| ASV894 | 427 | 2 | 1 | 427 | 100 | NR_037018.1 | Acidaminococcus fermentans strain VR4 16S ribosomal RNA gene, partial sequence | NR_037018.1 | 1516 | 349 | 775 | 28.17 | 784.1 | 0 | 426/427 | 99 | 0/427 | 0 | Plus | Plus | Bacteria; Firmicutes; Negativicutes; Acidaminococcales; Acidaminococcaceae; Acidaminococcus; Acidaminococcus fermentans | Acidaminococcus fermentans | Acidaminococcus | Acidaminococcaceae | Acidaminococcales | Negativicutes | Firmicutes | Bacteria |
| ASV895 | 423 | 1 | 1 | 423 | 100 | NR_144750.1 | Prevotellamassilia timonensis strain Marseille-P2831 16S ribosomal RNA, partial sequence | NR_144750.1 | 1493 | 358 | 779 | 28.27 | 547.7 | 2E-155 | 384/426 | 90 | 7/426 | 1 | Plus | Plus | Bacteria; Bacteroidetes; Bacteroidia; Bacteroidales; Prevotellaceae; Prevotellamassilia; Prevotellamassilia timonensis | Prevotellamassilia timonensis | Prevotellamassilia | Prevotellaceae | Bacteroidales | Bacteroidia | Bacteroidetes | Bacteria |
| ASV896 | 422 | 1 | 1 | 422 | 100 | NR_024816.1 | Prevotella salivae strain EPSA11 16S ribosomal RNA, partial sequence | NR_024816.1 | 1493 | 356 | 777 | 28.27 | 608.7 | 7E-174 | 391/422 | 92 | 0/422 | 0 | Plus | Plus | Bacteria; Bacteroidetes; Bacteroidia; Bacteroidales; Prevotellaceae; Prevotella; Prevotella salivae | Prevotella salivae | Prevotella | Prevotellaceae | Bacteroidales | Bacteroidia | Bacteroidetes | Bacteria |
| ASV897 | 406 | 1 | 1 | 406 | 100 | NR_115340.1 | Saccharofermentans acetigenes strain P6 16S ribosomal RNA, partial sequence | NR_115340.1 | 1510 | 353 | 757 | 26.82 | 505.3 | 1E-142 | 363/407 | 89 | 3/407 | 0 | Plus | Plus | Bacteria; Firmicutes; Clostridia; Eubacteriales; Oscillospiraceae; Saccharofermentans; Saccharofermentans acetigenes | Saccharofermentans acetigenes | Saccharofermentans | Oscillospiraceae | Eubacteriales | Clostridia | Firmicutes | Bacteria |
| ASV899 | 422 | 2 | 1 | 422 | 100 | NR_074376.1 | Parabacteroides distasonis ATCC 8503 16S ribosomal RNA, partial sequence | NR_074376.1 | 1497 | 368 | 789 | 28.19 | 575.4 | 7E-164 | 385/422 | 91 | 0/422 | 0 | Plus | Plus | Bacteria; Bacteroidetes; Bacteroidia; Bacteroidales; Tannerellaceae; Parabacteroides; Parabacteroides distasonis | Parabacteroides distasonis | Parabacteroides | Tannerellaceae | Bacteroidales | Bacteroidia | Bacteroidetes | Bacteria |
| ASV900 | 405 | 1 | 1 | 405 | 100 | NR_144611.1 | Flintibacter butyricus strain BLS21 16S ribosomal RNA, partial sequence | NR_144611.1 | 808 | 299 | 703 | 50.12 | 599.4 | 4E-171 | 378/405 | 93 | 0/405 | 0 | Plus | Plus | Bacteria; Firmicutes; Clostridia; Eubacteriales; ; Flintibacter; Flintibacter butyricus | Flintibacter butyricus | Flintibacter | | Eubacteriales | Clostridia | Firmicutes | Bacteria |
| ASV901 | 402 | 2 | 1 | 402 | 100 | NR_156080.1 | Kineothrix alysoides strain KNHs209 16S ribosomal RNA, partial sequence | NR_156080.1 | 1410 | 353 | 754 | 28.51 | 654.8 | 0 | 386/402 | 96 | 0/402 | 0 | Plus | Plus | Bacteria; Firmicutes; Clostridia; Eubacteriales; Lachnospiraceae; Kineothrix; Kineothrix alysoides | Kineothrix alysoides | Kineothrix | Lachnospiraceae | Eubacteriales | Clostridia | Firmicutes | Bacteria |
| ASV902 | 402 | 4 | 1 | 402 | 100 | NR_042200.1 | [Clostridium] asparagiforme strain N6 16S ribosomal RNA, partial sequence | NR_042200.1 | 1468 | 335 | 736 | 27.38 | 699.2 | 0 | 394/402 | 98 | 0/402 | 0 | Plus | Plus | Bacteria; Firmicutes; Clostridia; Eubacteriales; Lachnospiraceae; Enterocloster; Enterocloster asparagiformis | Enterocloster asparagiformis | Enterocloster | Lachnospiraceae | Eubacteriales | Clostridia | Firmicutes | Bacteria |
| ASV903 | 422 | 1 | 1 | 422 | 100 | NR_041364.1 | Prevotella stercorea DSM 18206 strain CB35 16S ribosomal RNA, partial sequence | NR_041364.1 | 1488 | 354 | 775 | 28.36 | 741.6 | 0 | 415/422 | 98 | 0/422 | 0 | Plus | Plus | Bacteria; Bacteroidetes; Bacteroidia; Bacteroidales; Prevotellaceae; Prevotella; Prevotella stercorea | Prevotella stercorea | Prevotella | Prevotellaceae | Bacteroidales | Bacteroidia | Bacteroidetes | Bacteria |
| ASV904 | 422 | 1 | 1 | 422 | 100 | NR_041364.1 | Prevotella stercorea DSM 18206 strain CB35 16S ribosomal RNA, partial sequence | NR_041364.1 | 1488 | 354 | 775 | 28.36 | 708.4 | 0 | 409/422 | 96 | 0/422 | 0 | Plus | Plus | Bacteria; Bacteroidetes; Bacteroidia; Bacteroidales; Prevotellaceae; Prevotella; Prevotella stercorea | Prevotella stercorea | Prevotella | Prevotellaceae | Bacteroidales | Bacteroidia | Bacteroidetes | Bacteria |
| ASV905 | 403 | 4 | 1 | 403 | 100 | NR_118520.1 | Campylobacter jejuni subsp. jejuni ATCC 33560 16S ribosomal RNA, partial sequence | NR_118520.1 | 1435 | 329 | 731 | 28.08 | 745.3 | 0 | 403/403 | 100 | 0/403 | 0 | Plus | Plus | Bacteria; Proteobacteria; Epsilonproteobacteria; Campylobacterales; Campylobacteraceae; Campylobacter; Campylobacter jejuni | Campylobacter jejuni | Campylobacter | Campylobacteraceae | Campylobacterales | Epsilonproteobacteria | Proteobacteria | Bacteria |
| ASV906 | 422 | 1 | 1 | 422 | 100 | NR_113411.1 | Prevotella copri DSM 18205 strain JCM 13464 16S ribosomal RNA, partial sequence | NR_113411.1 | 1493 | 358 | 779 | 28.27 | 614.2 | 2E-175 | 393/423 | 92 | 2/423 | 0 | Plus | Plus | Bacteria; Bacteroidetes; Bacteroidia; Bacteroidales; Prevotellaceae; Prevotella; Prevotella copri | Prevotella copri | Prevotella | Prevotellaceae | Bacteroidales | Bacteroidia | Bacteroidetes | Bacteria |
| ASV907 | 402 | 1 | 1 | 402 | 100 | NR_119085.1 | [Clostridium] polysaccharolyticum strain DSM 1801 16S ribosomal RNA, partial sequence | NR_119085.1 | 1503 | 364 | 763 | 26.61 | 669.6 | 0 | 389/402 | 96 | 2/402 | 0 | Plus | Plus | Bacteria; Firmicutes; Clostridia; Eubacteriales; Lachnospiraceae; Lachnoclostridium; [Clostridium] polysaccharolyticum | [Clostridium] polysaccharolyticum | Lachnoclostridium | Lachnospiraceae | Eubacteriales | Clostridia | Firmicutes | Bacteria |
| ASV908 | 403 | 4 | 1 | 403 | 100 | NR_074809.1 | Roseburia hominis A2-183 16S ribosomal RNA, partial sequence | NR_074809.1 | 1535 | 367 | 769 | 26.25 | 734.2 | 0 | 401/403 | 99 | 0/403 | 0 | Plus | Plus | Bacteria; Firmicutes; Clostridia; Eubacteriales; Lachnospiraceae; Roseburia; Roseburia hominis | Roseburia hominis | Roseburia | Lachnospiraceae | Eubacteriales | Clostridia | Firmicutes | Bacteria |
| ASV909 | 422 | 2 | 1 | 422 | 100 | NR_113119.1 | Prevotella oris strain JCM 8540 16S ribosomal RNA, partial sequence | NR_113119.1 | 1481 | 345 | 766 | 28.49 | 581 | 2E-165 | 387/423 | 91 | 2/423 | 0 | Plus | Plus | Bacteria; Bacteroidetes; Bacteroidia; Bacteroidales; Prevotellaceae; Prevotella; Prevotella oris | Prevotella oris | Prevotella | Prevotellaceae | Bacteroidales | Bacteroidia | Bacteroidetes | Bacteria |
| ASV910 | 426 | 1 | 4 | 426 | 99.3 | NR_146687.1 | Breznakia pachnodae strain Pei061 16S ribosomal RNA, partial sequence | NR_146687.1 | 1499 | 370 | 792 | 28.22 | 449.9 | 5E-126 | 366/426 | 85 | 6/426 | 1 | Plus | Plus | Bacteria; Firmicutes; Erysipelotrichia; Erysipelotrichales; Erysipelotrichaceae; Breznakia; Breznakia pachnodae | Breznakia pachnodae | Breznakia | Erysipelotrichaceae | Erysipelotrichales | Erysipelotrichia | Firmicutes | Bacteria |
| ASV911 | 408 | 5 | 1 | 408 | 100 | NR_074414.1 | Olsenella uli DSM 7084 16S ribosomal RNA, partial sequence | NR_074414.1 | 1503 | 348 | 755 | 27.15 | 688.1 | 0 | 396/408 | 97 | 0/408 | 0 | Plus | Plus | Bacteria; Actinobacteria; Coriobacteriia; Coriobacteriales; Atopobiaceae; Olsenella; Olsenella uli | Olsenella uli | Olsenella | Atopobiaceae | Coriobacteriales | Coriobacteriia | Actinobacteria | Bacteria |
| ASV913 | 426 | 2 | 1 | 426 | 100 | NR_042260.1 | Sphaerochaeta coccoides strain SPN1 16S ribosomal RNA, partial sequence | NR_042260.1 | 1500 | 320 | 746 | 28.47 | 505.3 | 1E-142 | 378/429 | 88 | 5/429 | 1 | Plus | Plus | Bacteria; Spirochaetes; Spirochaetia; Spirochaetales; Spirochaetaceae; Sphaerochaeta; Sphaerochaeta coccoides | Sphaerochaeta coccoides | Sphaerochaeta | Spirochaetaceae | Spirochaetales | Spirochaetia | Spirochaetes | Bacteria |
| ASV914 | 427 | 1 | 1 | 427 | 100 | NR_044111.1 | Selenomonas bovis strain WG 16S ribosomal RNA, partial sequence | NR_044111.1 | 1525 | 370 | 796 | 28 | 761.9 | 0 | 422/427 | 98 | 0/427 | 0 | Plus | Plus | Bacteria; Firmicutes; Negativicutes; Selenomonadales; Selenomonadaceae; Selenomonas; Selenomonas bovis | Selenomonas bovis | Selenomonas | Selenomonadaceae | Selenomonadales | Negativicutes | Firmicutes | Bacteria |
| ASV915 | 403 | 1 | 3 | 403 | 99.5 | NR_125464.1 | Anaerobacterium chartisolvens strain T-1-35 16S ribosomal RNA, partial sequence | NR_125464.1 | 1451 | 344 | 745 | 27.71 | 481.2 | 2E-135 | 355/402 | 88 | 1/402 | 0 | Plus | Plus | Bacteria; Firmicutes; Clostridia; Eubacteriales; Oscillospiraceae; Anaerobacterium; Anaerobacterium chartisolvens | Anaerobacterium chartisolvens | Anaerobacterium | Oscillospiraceae | Eubacteriales | Clostridia | Firmicutes | Bacteria |
| ASV916 | 402 | 3 | 1 | 402 | 100 | NR_156081.1 | Kineothrix alysoides strain KNHs209 16S ribosomal RNA, partial sequence | NR_156081.1 | 1411 | 353 | 754 | 28.49 | 704.7 | 0 | 395/402 | 98 | 0/402 | 0 | Plus | Plus | Bacteria; Firmicutes; Clostridia; Eubacteriales; Lachnospiraceae; Kineothrix; Kineothrix alysoides | Kineothrix alysoides | Kineothrix | Lachnospiraceae | Eubacteriales | Clostridia | Firmicutes | Bacteria |
| ASV917 | 422 | 1 | 1 | 422 | 100 | NR_024815.1 | Prevotella shahii strain EHS11 16S ribosomal RNA, partial sequence | NR_024815.1 | 1490 | 356 | 777 | 28.32 | 608.7 | 7E-174 | 393/424 | 92 | 4/424 | 0 | Plus | Plus | Bacteria; Bacteroidetes; Bacteroidia; Bacteroidales; Prevotellaceae; Prevotella; Prevotella shahii | Prevotella shahii | Prevotella | Prevotellaceae | Bacteroidales | Bacteroidia | Bacteroidetes | Bacteria |
| ASV918 | 402 | 1 | 1 | 402 | 100 | NR_026204.1 | [Clostridium] viride strain T2-7 16S ribosomal RNA, partial sequence | NR_026204.1 | 1482 | 345 | 749 | 27.33 | 536.6 | 3E-152 | 367/405 | 90 | 3/405 | 0 | Plus | Plus | Bacteria; Firmicutes; Clostridia; Eubacteriales; Oscillospiraceae; ; [Clostridium] viride | [Clostridium] viride | | Oscillospiraceae | Eubacteriales | Clostridia | Firmicutes | Bacteria |
| ASV919 | 402 | 1 | 1 | 402 | 100 | NR_026476.1 | Succinivibrio dextrinosolvens strain 0554 16S ribosomal RNA, partial sequence | NR_026476.1 | 1463 | 331 | 732 | 27.48 | 665.9 | 0 | 388/402 | 96 | 0/402 | 0 | Plus | Plus | Bacteria; Proteobacteria; Gammaproteobacteria; Aeromonadales; Succinivibrionaceae; Succinivibrio; Succinivibrio dextrinosolvens | Succinivibrio dextrinosolvens | Succinivibrio | Succinivibrionaceae | Aeromonadales | Gammaproteobacteria | Proteobacteria | Bacteria |
| ASV920 | 422 | 1 | 1 | 422 | 100 | NR_144750.1 | Prevotellamassilia timonensis strain Marseille-P2831 16S ribosomal RNA, partial sequence | NR_144750.1 | 1493 | 358 | 779 | 28.27 | 553.3 | 4E-157 | 381/422 | 90 | 0/422 | 0 | Plus | Plus | Bacteria; Bacteroidetes; Bacteroidia; Bacteroidales; Prevotellaceae; Prevotellamassilia; Prevotellamassilia timonensis | Prevotellamassilia timonensis | Prevotellamassilia | Prevotellaceae | Bacteroidales | Bacteroidia | Bacteroidetes | Bacteria |
| ASV921 | 405 | 5 | 4 | 405 | 99.26 | NR_115693.1 | Gracilibacter thermotolerans JW/YJL-S1 16S ribosomal RNA, partial sequence | NR_115693.1 | 1526 | 462 | 861 | 26.21 | 470.2 | 3E-132 | 354/403 | 87 | 4/403 | 0 | Plus | Plus | Bacteria; Firmicutes; Clostridia; Eubacteriales; Gracilibacteraceae; Gracilibacter; Gracilibacter thermotolerans | Gracilibacter thermotolerans | Gracilibacter | Gracilibacteraceae | Eubacteriales | Clostridia | Firmicutes | Bacteria |
| ASV922 | 422 | 2 | 1 | 422 | 100 | NR_113119.1 | Prevotella oris strain JCM 8540 16S ribosomal RNA, partial sequence | NR_113119.1 | 1481 | 345 | 766 | 28.49 | 603.1 | 3E-172 | 392/424 | 92 | 4/424 | 0 | Plus | Plus | Bacteria; Bacteroidetes; Bacteroidia; Bacteroidales; Prevotellaceae; Prevotella; Prevotella oris | Prevotella oris | Prevotella | Prevotellaceae | Bacteroidales | Bacteroidia | Bacteroidetes | Bacteria |
| ASV923 | 427 | 2 | 1 | 427 | 100 | NR_037018.1 | Acidaminococcus fermentans strain VR4 16S ribosomal RNA gene, partial sequence | NR_037018.1 | 1516 | 349 | 775 | 28.17 | 728.7 | 0 | 416/427 | 97 | 0/427 | 0 | Plus | Plus | Bacteria; Firmicutes; Negativicutes; Acidaminococcales; Acidaminococcaceae; Acidaminococcus; Acidaminococcus fermentans | Acidaminococcus fermentans | Acidaminococcus | Acidaminococcaceae | Acidaminococcales | Negativicutes | Firmicutes | Bacteria |
| ASV925 | 427 | 1 | 1 | 427 | 100 | NR_027596.1 | Mitsuokella multacida strain DSM 20544 16S ribosomal RNA, partial sequence | NR_027596.1 | 1511 | 346 | 772 | 28.26 | 702.8 | 0 | 413/429 | 96 | 4/429 | 0 | Plus | Plus | Bacteria; Firmicutes; Negativicutes; Selenomonadales; Selenomonadaceae; Mitsuokella; Mitsuokella multacida | Mitsuokella multacida | Mitsuokella | Selenomonadaceae | Selenomonadales | Negativicutes | Firmicutes | Bacteria |
| ASV926 | 405 | 3 | 1 | 405 | 100 | NR_115307.1 | Ethanoligenens harbinense YUAN-3 16S ribosomal RNA, partial sequence | NR_115307.1 | 1497 | 340 | 743 | 26.99 | 558.8 | 7E-159 | 373/407 | 91 | 5/407 | 1 | Plus | Plus | Bacteria; Firmicutes; Clostridia; Eubacteriales; Oscillospiraceae; Ethanoligenens; Ethanoligenens harbinense | Ethanoligenens harbinense | Ethanoligenens | Oscillospiraceae | Eubacteriales | Clostridia | Firmicutes | Bacteria |
| ASV927 | 402 | 1 | 1 | 402 | 100 | NR_042832.1 | Roseburia faecis strain M72/1 16S ribosomal RNA, partial sequence | NR_042832.1 | 1456 | 347 | 749 | 27.68 | 665.9 | 0 | 390/404 | 96 | 3/404 | 0 | Plus | Plus | Bacteria; Firmicutes; Clostridia; Eubacteriales; Lachnospiraceae; Roseburia; Roseburia faecis | Roseburia faecis | Roseburia | Lachnospiraceae | Eubacteriales | Clostridia | Firmicutes | Bacteria |
| ASV928 | 425 | 1 | 1 | 425 | 100 | NR_029325.1 | Schwartzia succinivorans strain S1-1 16S ribosomal RNA, partial sequence | NR_029325.1 | 1489 | 344 | 769 | 28.61 | 470.2 | 4E-132 | 371/428 | 86 | 5/428 | 1 | Plus | Plus | Bacteria; Firmicutes; Negativicutes; Selenomonadales; Selenomonadaceae; Schwartzia; Schwartzia succinivorans | Schwartzia succinivorans | Schwartzia | Selenomonadaceae | Selenomonadales | Negativicutes | Firmicutes | Bacteria |
| ASV929 | 405 | 1 | 1 | 405 | 100 | NR_125464.1 | Anaerobacterium chartisolvens strain T-1-35 16S ribosomal RNA, partial sequence | NR_125464.1 | 1451 | 342 | 745 | 27.84 | 575.4 | 7E-164 | 374/405 | 92 | 1/405 | 0 | Plus | Plus | Bacteria; Firmicutes; Clostridia; Eubacteriales; Oscillospiraceae; Anaerobacterium; Anaerobacterium chartisolvens | Anaerobacterium chartisolvens | Anaerobacterium | Oscillospiraceae | Eubacteriales | Clostridia | Firmicutes | Bacteria |
| ASV930 | 402 | 1 | 1 | 402 | 100 | NR_024661.1 | Eubacterium ruminantium strain GA195 16S ribosomal RNA, partial sequence | NR_024661.1 | 1508 | 378 | 779 | 26.66 | 654.8 | 0 | 386/402 | 96 | 0/402 | 0 | Plus | Plus | Bacteria; Firmicutes; Clostridia; Eubacteriales; Eubacteriaceae; Eubacterium; Eubacterium ruminantium | Eubacterium ruminantium | Eubacterium | Eubacteriaceae | Eubacteriales | Clostridia | Firmicutes | Bacteria |
| ASV931 | 423 | 1 | 1 | 423 | 100 | NR_144750.1 | Prevotellamassilia timonensis strain Marseille-P2831 16S ribosomal RNA, partial sequence | NR_144750.1 | 1493 | 358 | 779 | 28.27 | 542.2 | 8E-154 | 383/426 | 89 | 7/426 | 1 | Plus | Plus | Bacteria; Bacteroidetes; Bacteroidia; Bacteroidales; Prevotellaceae; Prevotellamassilia; Prevotellamassilia timonensis | Prevotellamassilia timonensis | Prevotellamassilia | Prevotellaceae | Bacteroidales | Bacteroidia | Bacteroidetes | Bacteria |
| ASV932 | 405 | 1 | 1 | 405 | 100 | NR_044972.1 | Sporobacter termitidis strain SYR 16S ribosomal RNA, partial sequence | NR_044972.1 | 1506 | 348 | 752 | 26.89 | 569.9 | 3E-162 | 373/406 | 91 | 2/406 | 0 | Plus | Plus | Bacteria; Firmicutes; Clostridia; Eubacteriales; Oscillospiraceae; Sporobacter; Sporobacter termitidis | Sporobacter termitidis | Sporobacter | Oscillospiraceae | Eubacteriales | Clostridia | Firmicutes | Bacteria |
| ASV933 | 403 | 3 | 1 | 403 | 100 | NR_118521.1 | Campylobacter lanienae NCTC 13004 strain CCUG 44467 16S ribosomal RNA, partial sequence | NR_118521.1 | 1434 | 328 | 730 | 28.1 | 717.6 | 0 | 398/403 | 98 | 0/403 | 0 | Plus | Plus | Bacteria; Proteobacteria; Epsilonproteobacteria; Campylobacterales; Campylobacteraceae; Campylobacter; Campylobacter lanienae | Campylobacter lanienae | Campylobacter | Campylobacteraceae | Campylobacterales | Epsilonproteobacteria | Proteobacteria | Bacteria |
| ASV934 | 403 | 1 | 3 | 403 | 99.5 | NR_125464.1 | Anaerobacterium chartisolvens strain T-1-35 16S ribosomal RNA, partial sequence | NR_125464.1 | 1451 | 344 | 745 | 27.71 | 486.8 | 3E-137 | 356/402 | 88 | 1/402 | 0 | Plus | Plus | Bacteria; Firmicutes; Clostridia; Eubacteriales; Oscillospiraceae; Anaerobacterium; Anaerobacterium chartisolvens | Anaerobacterium chartisolvens | Anaerobacterium | Oscillospiraceae | Eubacteriales | Clostridia | Firmicutes | Bacteria |
| ASV935 | 422 | 1 | 1 | 422 | 100 | NR_043805.1 | Olivibacter sitiensis strain AW-6 16S ribosomal RNA, partial sequence | NR_043805.1 | 1477 | 328 | 749 | 28.57 | 459.1 | 8E-129 | 365/423 | 86 | 2/423 | 0 | Plus | Plus | Bacteria; Bacteroidetes; Sphingobacteriia; Sphingobacteriales; Sphingobacteriaceae; Olivibacter; Olivibacter sitiensis | Olivibacter sitiensis | Olivibacter | Sphingobacteriaceae | Sphingobacteriales | Sphingobacteriia | Bacteroidetes | Bacteria |
| ASV936 | 422 | 2 | 1 | 422 | 100 | NR_113076.1 | Parabacteroides goldsteinii DSM 19448 = WAL 12034 strain JCM 13446 16S ribosomal RNA, partial sequence | NR_113076.1 | 1476 | 341 | 762 | 28.59 | 525.6 | 8E-149 | 376/422 | 89 | 0/422 | 0 | Plus | Plus | Bacteria; Bacteroidetes; Bacteroidia; Bacteroidales; Tannerellaceae; Parabacteroides; Parabacteroides goldsteinii | Parabacteroides goldsteinii | Parabacteroides | Tannerellaceae | Bacteroidales | Bacteroidia | Bacteroidetes | Bacteria |
| ASV939 | 423 | 1 | 1 | 423 | 100 | NR_144750.1 | Prevotellamassilia timonensis strain Marseille-P2831 16S ribosomal RNA, partial sequence | NR_144750.1 | 1493 | 358 | 779 | 28.27 | 564.3 | 2E-160 | 386/425 | 90 | 5/425 | 1 | Plus | Plus | Bacteria; Bacteroidetes; Bacteroidia; Bacteroidales; Prevotellaceae; Prevotellamassilia; Prevotellamassilia timonensis | Prevotellamassilia timonensis | Prevotellamassilia | Prevotellaceae | Bacteroidales | Bacteroidia | Bacteroidetes | Bacteria |
| ASV940 | 405 | 1 | 1 | 405 | 100 | NR_118554.1 | Intestinimonas butyriciproducens strain SRB-521-5-I 16S ribosomal RNA, partial sequence | NR_118554.1 | 1475 | 346 | 750 | 27.46 | 516.3 | 4E-146 | 366/408 | 89 | 6/408 | 1 | Plus | Plus | Bacteria; Firmicutes; Clostridia; Eubacteriales; ; Intestinimonas; Intestinimonas butyriciproducens | Intestinimonas butyriciproducens | Intestinimonas | | Eubacteriales | Clostridia | Firmicutes | Bacteria |
| ASV941 | 402 | 1 | 1 | 402 | 100 | NR_118675.1 | [Eubacterium] siraeum strain ATCC 29066 16S ribosomal RNA, partial sequence | NR_118675.1 | 1453 | 356 | 757 | 27.67 | 640.1 | 0 | 383/403 | 95 | 2/403 | 0 | Plus | Plus | Bacteria; Firmicutes; Clostridia; Eubacteriales; Oscillospiraceae; ; [Eubacterium] siraeum | [Eubacterium] siraeum | | Oscillospiraceae | Eubacteriales | Clostridia | Firmicutes | Bacteria |
| ASV942 | 403 | 2 | 1 | 403 | 100 | NR_024948.1 | Campylobacter hyointestinalis subsp. lawsonii strain CHY5 16S ribosomal RNA, partial sequence | NR_024948.1 | 1419 | 318 | 720 | 28.4 | 717.6 | 0 | 398/403 | 98 | 0/403 | 0 | Plus | Plus | Bacteria; Proteobacteria; Epsilonproteobacteria; Campylobacterales; Campylobacteraceae; Campylobacter; Campylobacter hyointestinalis | Campylobacter hyointestinalis | Campylobacter | Campylobacteraceae | Campylobacterales | Epsilonproteobacteria | Proteobacteria | Bacteria |
| ASV943 | 422 | 4 | 1 | 422 | 100 | NR_113122.1 | Prevotella paludivivens strain JCM 13650 16S ribosomal RNA, partial sequence | NR_113122.1 | 1491 | 356 | 777 | 28.3 | 575.4 | 7E-164 | 386/423 | 91 | 2/423 | 0 | Plus | Plus | Bacteria; Bacteroidetes; Bacteroidia; Bacteroidales; Prevotellaceae; Prevotella; Prevotella paludivivens | Prevotella paludivivens | Prevotella | Prevotellaceae | Bacteroidales | Bacteroidia | Bacteroidetes | Bacteria |
| ASV944 | 403 | 1 | 1 | 403 | 100 | NR_118554.1 | Intestinimonas butyriciproducens strain SRB-521-5-I 16S ribosomal RNA, partial sequence | NR_118554.1 | 1475 | 346 | 750 | 27.46 | 508.9 | 7E-144 | 362/405 | 89 | 2/405 | 0 | Plus | Plus | Bacteria; Firmicutes; Clostridia; Eubacteriales; ; Intestinimonas; Intestinimonas butyriciproducens | Intestinimonas butyriciproducens | Intestinimonas | | Eubacteriales | Clostridia | Firmicutes | Bacteria |
| ASV945 | 402 | 5 | 1 | 402 | 100 | NR_156081.1 | Kineothrix alysoides strain KNHs209 16S ribosomal RNA, partial sequence | NR_156081.1 | 1411 | 353 | 754 | 28.49 | 660.4 | 0 | 387/402 | 96 | 0/402 | 0 | Plus | Plus | Bacteria; Firmicutes; Clostridia; Eubacteriales; Lachnospiraceae; Kineothrix; Kineothrix alysoides | Kineothrix alysoides | Kineothrix | Lachnospiraceae | Eubacteriales | Clostridia | Firmicutes | Bacteria |
| ASV946 | 422 | 2 | 1 | 422 | 100 | NR_113105.1 | Prevotella histicola strain JCM 15637 16S ribosomal RNA, partial sequence | NR_113105.1 | 1480 | 346 | 767 | 28.51 | 647.4 | 0 | 400/424 | 94 | 4/424 | 0 | Plus | Plus | Bacteria; Bacteroidetes; Bacteroidia; Bacteroidales; Prevotellaceae; Prevotella; Prevotella histicola | Prevotella histicola | Prevotella | Prevotellaceae | Bacteroidales | Bacteroidia | Bacteroidetes | Bacteria |
| ASV947 | 402 | 1 | 1 | 402 | 100 | NR_118675.1 | [Eubacterium] siraeum strain ATCC 29066 16S ribosomal RNA, partial sequence | NR_118675.1 | 1453 | 356 | 757 | 27.67 | 617.9 | 1E-176 | 378/402 | 94 | 0/402 | 0 | Plus | Plus | Bacteria; Firmicutes; Clostridia; Eubacteriales; Oscillospiraceae; ; [Eubacterium] siraeum | [Eubacterium] siraeum | | Oscillospiraceae | Eubacteriales | Clostridia | Firmicutes | Bacteria |
| ASV948 | 423 | 1 | 1 | 423 | 100 | NR_144750.1 | Prevotellamassilia timonensis strain Marseille-P2831 16S ribosomal RNA, partial sequence | NR_144750.1 | 1493 | 358 | 779 | 28.27 | 581 | 2E-165 | 389/425 | 91 | 5/425 | 1 | Plus | Plus | Bacteria; Bacteroidetes; Bacteroidia; Bacteroidales; Prevotellaceae; Prevotellamassilia; Prevotellamassilia timonensis | Prevotellamassilia timonensis | Prevotellamassilia | Prevotellaceae | Bacteroidales | Bacteroidia | Bacteroidetes | Bacteria |
| ASV949 | 427 | 1 | 1 | 427 | 100 | NR_041304.1 | Sharpea azabuensis strain ST18 16S ribosomal RNA, partial sequence | NR_041304.1 | 1460 | 325 | 750 | 29.18 | 760.1 | 0 | 422/427 | 98 | 1/427 | 0 | Plus | Plus | Bacteria; Firmicutes; Erysipelotrichia; Erysipelotrichales; Coprobacillaceae; Sharpea; Sharpea azabuensis | Sharpea azabuensis | Sharpea | Coprobacillaceae | Erysipelotrichales | Erysipelotrichia | Firmicutes | Bacteria |
| ASV950 | 421 | 2 | 1 | 421 | 100 | NR_029335.1 | Holdemania filiformis strain J1-31B-1 16S ribosomal RNA, partial sequence | NR_029335.1 | 1391 | 267 | 692 | 30.63 | 593.9 | 2E-169 | 392/426 | 92 | 5/426 | 1 | Plus | Plus | Bacteria; Firmicutes; Erysipelotrichia; Erysipelotrichales; Erysipelotrichaceae; Holdemania; Holdemania filiformis | Holdemania filiformis | Holdemania | Erysipelotrichaceae | Erysipelotrichales | Erysipelotrichia | Firmicutes | Bacteria |
| ASV951 | 422 | 1 | 1 | 422 | 100 | NR_113411.1 | Prevotella copri DSM 18205 strain JCM 13464 16S ribosomal RNA, partial sequence | NR_113411.1 | 1493 | 358 | 779 | 28.27 | 603.1 | 3E-172 | 391/423 | 92 | 2/423 | 0 | Plus | Plus | Bacteria; Bacteroidetes; Bacteroidia; Bacteroidales; Prevotellaceae; Prevotella; Prevotella copri | Prevotella copri | Prevotella | Prevotellaceae | Bacteroidales | Bacteroidia | Bacteroidetes | Bacteria |
| ASV953 | 422 | 1 | 1 | 422 | 100 | NR_113411.1 | Prevotella copri DSM 18205 strain JCM 13464 16S ribosomal RNA, partial sequence | NR_113411.1 | 1493 | 358 | 779 | 28.27 | 725 | 0 | 412/422 | 97 | 0/422 | 0 | Plus | Plus | Bacteria; Bacteroidetes; Bacteroidia; Bacteroidales; Prevotellaceae; Prevotella; Prevotella copri | Prevotella copri | Prevotella | Prevotellaceae | Bacteroidales | Bacteroidia | Bacteroidetes | Bacteria |
| ASV954 | 405 | 1 | 1 | 405 | 100 | NR_147370.1 | Pseudoflavonifractor phocaeensis strain Marseille-P3064 16S ribosomal RNA, partial sequence | NR_147370.1 | 1495 | 366 | 770 | 27.09 | 688.1 | 0 | 394/405 | 97 | 0/405 | 0 | Plus | Plus | Bacteria; Firmicutes; Clostridia; Eubacteriales; Oscillospiraceae; Pseudoflavonifractor; Pseudoflavonifractor phocaeensis | Pseudoflavonifractor phocaeensis | Pseudoflavonifractor | Oscillospiraceae | Eubacteriales | Clostridia | Firmicutes | Bacteria |
| ASV955 | 422 | 2 | 1 | 422 | 100 | NR_113119.1 | Prevotella oris strain JCM 8540 16S ribosomal RNA, partial sequence | NR_113119.1 | 1481 | 345 | 766 | 28.49 | 558.8 | 8E-159 | 384/424 | 90 | 4/424 | 0 | Plus | Plus | Bacteria; Bacteroidetes; Bacteroidia; Bacteroidales; Prevotellaceae; Prevotella; Prevotella oris | Prevotella oris | Prevotella | Prevotellaceae | Bacteroidales | Bacteroidia | Bacteroidetes | Bacteria |
| ASV956 | 405 | 1 | 1 | 405 | 100 | NR_118554.1 | Intestinimonas butyriciproducens strain SRB-521-5-I 16S ribosomal RNA, partial sequence | NR_118554.1 | 1475 | 346 | 750 | 27.46 | 582.8 | 4E-166 | 375/405 | 92 | 0/405 | 0 | Plus | Plus | Bacteria; Firmicutes; Clostridia; Eubacteriales; ; Intestinimonas; Intestinimonas butyriciproducens | Intestinimonas butyriciproducens | Intestinimonas | | Eubacteriales | Clostridia | Firmicutes | Bacteria |
| ASV957 | 422 | 2 | 1 | 422 | 100 | NR_074577.1 | Paludibacter propionicigenes WB4 16S ribosomal RNA, partial sequence | NR_074577.1 | 1516 | 353 | 774 | 27.84 | 497.9 | 2E-140 | 373/424 | 87 | 4/424 | 0 | Plus | Plus | Bacteria; Bacteroidetes; Bacteroidia; Bacteroidales; Paludibacteraceae; Paludibacter; Paludibacter propionicigenes | Paludibacter propionicigenes | Paludibacter | Paludibacteraceae | Bacteroidales | Bacteroidia | Bacteroidetes | Bacteria |
| ASV958 | 403 | 1 | 1 | 403 | 100 | NR_144748.1 | Phocea massiliensis strain Marseille-P2769 16S ribosomal RNA, partial sequence | NR_144748.1 | 1476 | 352 | 752 | 27.17 | 577.3 | 2E-164 | 373/403 | 92 | 2/403 | 0 | Plus | Plus | Bacteria; Firmicutes; Clostridia; Eubacteriales; Oscillospiraceae; Phocea; Phocea massiliensis | Phocea massiliensis | Phocea | Oscillospiraceae | Eubacteriales | Clostridia | Firmicutes | Bacteria |
| ASV959 | 402 | 1 | 1 | 402 | 100 | NR_074613.1 | [Eubacterium] eligens ATCC 27750 16S ribosomal RNA, partial sequence | NR_074613.1 | 1519 | 367 | 768 | 26.46 | 743.5 | 0 | 402/402 | 100 | 0/402 | 0 | Plus | Plus | Bacteria; Firmicutes; Clostridia; Eubacteriales; Lachnospiraceae; Lachnospira; Lachnospira eligens | Lachnospira eligens | Lachnospira | Lachnospiraceae | Eubacteriales | Clostridia | Firmicutes | Bacteria |
| ASV960 | 405 | 1 | 1 | 405 | 100 | NR_042834.1 | Alkalibacter saccharofermentans strain Z-79820 16S ribosomal RNA, partial sequence | NR_042834.1 | 1465 | 338 | 741 | 27.58 | 475.7 | 7E-134 | 357/406 | 87 | 3/406 | 0 | Plus | Plus | Bacteria; Firmicutes; Clostridia; Eubacteriales; Eubacteriaceae; Alkalibacter; Alkalibacter saccharofermentans | Alkalibacter saccharofermentans | Alkalibacter | Eubacteriaceae | Eubacteriales | Clostridia | Firmicutes | Bacteria |
| ASV961 | 427 | 1 | 1 | 427 | 100 | NR_044111.1 | Selenomonas bovis strain WG 16S ribosomal RNA, partial sequence | NR_044111.1 | 1525 | 370 | 796 | 28 | 667.8 | 0 | 406/428 | 94 | 2/428 | 0 | Plus | Plus | Bacteria; Firmicutes; Negativicutes; Selenomonadales; Selenomonadaceae; Selenomonas; Selenomonas bovis | Selenomonas bovis | Selenomonas | Selenomonadaceae | Selenomonadales | Negativicutes | Firmicutes | Bacteria |
| ASV962 | 403 | 1 | 1 | 403 | 100 | NR_104907.1 | Eubacterium coprostanoligenes strain HL 16S ribosomal RNA, partial sequence | NR_104907.1 | 1446 | 332 | 734 | 27.87 | 706.5 | 0 | 396/403 | 98 | 0/403 | 0 | Plus | Plus | Bacteria; Firmicutes; Clostridia; Eubacteriales; Eubacteriaceae; Eubacterium; Eubacterium coprostanoligenes | Eubacterium coprostanoligenes | Eubacterium | Eubacteriaceae | Eubacteriales | Clostridia | Firmicutes | Bacteria |
| ASV963 | 402 | 3 | 1 | 402 | 100 | NR_156081.1 | Kineothrix alysoides strain KNHs209 16S ribosomal RNA, partial sequence | NR_156081.1 | 1411 | 353 | 754 | 28.49 | 682.5 | 0 | 391/402 | 97 | 0/402 | 0 | Plus | Plus | Bacteria; Firmicutes; Clostridia; Eubacteriales; Lachnospiraceae; Kineothrix; Kineothrix alysoides | Kineothrix alysoides | Kineothrix | Lachnospiraceae | Eubacteriales | Clostridia | Firmicutes | Bacteria |
| ASV964 | 422 | 2 | 1 | 422 | 100 | NR_113119.1 | Prevotella oris strain JCM 8540 16S ribosomal RNA, partial sequence | NR_113119.1 | 1481 | 345 | 766 | 28.49 | 619.7 | 3E-177 | 395/424 | 93 | 4/424 | 0 | Plus | Plus | Bacteria; Bacteroidetes; Bacteroidia; Bacteroidales; Prevotellaceae; Prevotella; Prevotella oris | Prevotella oris | Prevotella | Prevotellaceae | Bacteroidales | Bacteroidia | Bacteroidetes | Bacteria |
| ASV965 | 427 | 1 | 1 | 427 | 100 | NR_027596.1 | Mitsuokella multacida strain DSM 20544 16S ribosomal RNA, partial sequence | NR_027596.1 | 1511 | 346 | 772 | 28.26 | 747.2 | 0 | 419/427 | 98 | 0/427 | 0 | Plus | Plus | Bacteria; Firmicutes; Negativicutes; Selenomonadales; Selenomonadaceae; Mitsuokella; Mitsuokella multacida | Mitsuokella multacida | Mitsuokella | Selenomonadaceae | Selenomonadales | Negativicutes | Firmicutes | Bacteria |
| ASV966 | 422 | 1 | 1 | 422 | 100 | NR_024815.1 | Prevotella shahii strain EHS11 16S ribosomal RNA, partial sequence | NR_024815.1 | 1490 | 356 | 777 | 28.32 | 608.7 | 7E-174 | 393/424 | 92 | 4/424 | 0 | Plus | Plus | Bacteria; Bacteroidetes; Bacteroidia; Bacteroidales; Prevotellaceae; Prevotella; Prevotella shahii | Prevotella shahii | Prevotella | Prevotellaceae | Bacteroidales | Bacteroidia | Bacteroidetes | Bacteria |
| ASV967 | 402 | 4 | 1 | 402 | 100 | NR_118730.1 | [Clostridium] symbiosum strain ATCC 14940 16S ribosomal RNA, partial sequence | NR_118730.1 | 1478 | 372 | 773 | 27.2 | 671.5 | 0 | 387/402 | 96 | 0/402 | 0 | Plus | Plus | Bacteria; Firmicutes; Clostridia; Eubacteriales; Lachnospiraceae; Lachnoclostridium; [Clostridium] symbiosum | [Clostridium] symbiosum | Lachnoclostridium | Lachnospiraceae | Eubacteriales | Clostridia | Firmicutes | Bacteria |
| ASV968 | 422 | 2 | 1 | 422 | 100 | NR_074376.1 | Parabacteroides distasonis ATCC 8503 16S ribosomal RNA, partial sequence | NR_074376.1 | 1497 | 368 | 789 | 28.19 | 553.3 | 4E-157 | 383/424 | 90 | 4/424 | 0 | Plus | Plus | Bacteria; Bacteroidetes; Bacteroidia; Bacteroidales; Tannerellaceae; Parabacteroides; Parabacteroides distasonis | Parabacteroides distasonis | Parabacteroides | Tannerellaceae | Bacteroidales | Bacteroidia | Bacteroidetes | Bacteria |
| ASV969 | 404 | 1 | 1 | 404 | 100 | NR_044503.1 | Thermotalea metallivorans strain B2-1 16S ribosomal RNA, partial sequence | NR_044503.1 | 1627 | 467 | 868 | 24.71 | 479.4 | 6E-135 | 362/410 | 88 | 14/410 | 3 | Plus | Plus | Bacteria; Firmicutes; Clostridia; Eubacteriales; Clostridiaceae; Thermotalea; Thermotalea metallivorans | Thermotalea metallivorans | Thermotalea | Clostridiaceae | Eubacteriales | Clostridia | Firmicutes | Bacteria |
| ASV970 | 404 | 1 | 1 | 404 | 100 | NR_118156.1 | Oscillibacter ruminantium GH1 16S ribosomal RNA, partial sequence | NR_118156.1 | 1409 | 321 | 724 | 28.67 | 636.4 | 0 | 384/404 | 95 | 0/404 | 0 | Plus | Plus | Bacteria; Firmicutes; Clostridia; Eubacteriales; Oscillospiraceae; Oscillibacter; Oscillibacter ruminantium | Oscillibacter ruminantium | Oscillibacter | Oscillospiraceae | Eubacteriales | Clostridia | Firmicutes | Bacteria |
| ASV971 | 428 | 2 | 1 | 427 | 99.77 | NR_116868.1 | Desulfosporosinus acidiphilus SJ4 16S ribosomal RNA, partial sequence | NR_116868.1 | 1538 | 369 | 795 | 27.76 | 496 | 6E-140 | 376/429 | 87 | 4/429 | 0 | Plus | Plus | Bacteria; Firmicutes; Clostridia; Eubacteriales; Peptococcaceae; Desulfosporosinus; Desulfosporosinus acidiphilus | Desulfosporosinus acidiphilus | Desulfosporosinus | Peptococcaceae | Eubacteriales | Clostridia | Firmicutes | Bacteria |
| ASV972 | 421 | 1 | 1 | 421 | 100 | NR_144616.1 | Muribaculum intestinale strain YL27 16S ribosomal RNA, partial sequence | NR_144616.1 | 1460 | 297 | 718 | 28.9 | 579.1 | 6E-165 | 388/424 | 91 | 5/424 | 1 | Plus | Plus | Bacteria; Bacteroidetes; Bacteroidia; Bacteroidales; Muribaculaceae; Muribaculum; Muribaculum intestinale | Muribaculum intestinale | Muribaculum | Muribaculaceae | Bacteroidales | Bacteroidia | Bacteroidetes | Bacteria |
| ASV973 | 405 | 3 | 1 | 405 | 100 | NR_115307.1 | Ethanoligenens harbinense YUAN-3 16S ribosomal RNA, partial sequence | NR_115307.1 | 1497 | 340 | 743 | 26.99 | 558.8 | 7E-159 | 373/407 | 91 | 5/407 | 1 | Plus | Plus | Bacteria; Firmicutes; Clostridia; Eubacteriales; Oscillospiraceae; Ethanoligenens; Ethanoligenens harbinense | Ethanoligenens harbinense | Ethanoligenens | Oscillospiraceae | Eubacteriales | Clostridia | Firmicutes | Bacteria |
| ASV974 | 405 | 1 | 1 | 405 | 100 | NR_118554.1 | Intestinimonas butyriciproducens strain SRB-521-5-I 16S ribosomal RNA, partial sequence | NR_118554.1 | 1475 | 346 | 750 | 27.46 | 527.4 | 2E-149 | 369/409 | 90 | 8/409 | 1 | Plus | Plus | Bacteria; Firmicutes; Clostridia; Eubacteriales; ; Intestinimonas; Intestinimonas butyriciproducens | Intestinimonas butyriciproducens | Intestinimonas | | Eubacteriales | Clostridia | Firmicutes | Bacteria |
| ASV978 | 427 | 3 | 1 | 427 | 100 | NR_113393.1 | Peptococcus niger strain JCM 6506 16S ribosomal RNA, partial sequence | NR_113393.1 | 1528 | 374 | 800 | 27.95 | 629 | 6E-180 | 398/427 | 93 | 0/427 | 0 | Plus | Plus | Bacteria; Firmicutes; Clostridia; Eubacteriales; Peptococcaceae; Peptococcus; Peptococcus niger | Peptococcus niger | Peptococcus | Peptococcaceae | Eubacteriales | Clostridia | Firmicutes | Bacteria |
| ASV979 | 405 | 4 | 1 | 405 | 100 | NR_115307.1 | Ethanoligenens harbinense YUAN-3 16S ribosomal RNA, partial sequence | NR_115307.1 | 1497 | 340 | 743 | 26.99 | 531.1 | 2E-150 | 369/408 | 90 | 7/408 | 1 | Plus | Plus | Bacteria; Firmicutes; Clostridia; Eubacteriales; Oscillospiraceae; Ethanoligenens; Ethanoligenens harbinense | Ethanoligenens harbinense | Ethanoligenens | Oscillospiraceae | Eubacteriales | Clostridia | Firmicutes | Bacteria |
| ASV980 | 404 | 1 | 1 | 404 | 100 | NR_025127.1 | Lachnobacterium bovis strain LRC 5382 16S ribosomal RNA, partial sequence | NR_025127.1 | 1453 | 362 | 765 | 27.8 | 658.5 | 0 | 388/404 | 96 | 0/404 | 0 | Plus | Plus | Bacteria; Firmicutes; Clostridia; Eubacteriales; Lachnospiraceae; Lachnobacterium; Lachnobacterium bovis | Lachnobacterium bovis | Lachnobacterium | Lachnospiraceae | Eubacteriales | Clostridia | Firmicutes | Bacteria |
| ASV981 | 422 | 2 | 1 | 422 | 100 | NR_113117.1 | Prevotella oralis ATCC 33269 strain JCM 12251 16S ribosomal RNA, partial sequence | NR_113117.1 | 1496 | 361 | 782 | 28.21 | 603.1 | 3E-172 | 391/423 | 92 | 2/423 | 0 | Plus | Plus | Bacteria; Bacteroidetes; Bacteroidia; Bacteroidales; Prevotellaceae; Prevotella; Prevotella oralis | Prevotella oralis | Prevotella | Prevotellaceae | Bacteroidales | Bacteroidia | Bacteroidetes | Bacteria |
| ASV982 | 405 | 1 | 1 | 405 | 100 | NR_147398.1 | Anaerotruncus rubiinfantis strain MT15 16S ribosomal RNA, partial sequence | NR_147398.1 | 1490 | 368 | 768 | 26.91 | 490.5 | 3E-138 | 362/408 | 88 | 10/408 | 2 | Plus | Plus | Bacteria; Firmicutes; Clostridia; Eubacteriales; Oscillospiraceae; Anaerotruncus; Anaerotruncus rubiinfantis | Anaerotruncus rubiinfantis | Anaerotruncus | Oscillospiraceae | Eubacteriales | Clostridia | Firmicutes | Bacteria |
| ASV983 | 422 | 2 | 1 | 422 | 100 | NR_113119.1 | Prevotella oris strain JCM 8540 16S ribosomal RNA, partial sequence | NR_113119.1 | 1481 | 345 | 766 | 28.49 | 586.5 | 3E-167 | 387/422 | 91 | 0/422 | 0 | Plus | Plus | Bacteria; Bacteroidetes; Bacteroidia; Bacteroidales; Prevotellaceae; Prevotella; Prevotella oris | Prevotella oris | Prevotella | Prevotellaceae | Bacteroidales | Bacteroidia | Bacteroidetes | Bacteria |
| ASV984 | 418 | 1 | 1 | 418 | 100 | NR_102893.1 | Butyrivibrio proteoclasticus strain B316 16S ribosomal RNA, partial sequence | NR_102893.1 | 1537 | 367 | 782 | 27.07 | 588.4 | 1E-167 | 385/418 | 92 | 2/418 | 0 | Plus | Plus | Bacteria; Firmicutes; Clostridia; Eubacteriales; Lachnospiraceae; Butyrivibrio; Butyrivibrio proteoclasticus | Butyrivibrio proteoclasticus | Butyrivibrio | Lachnospiraceae | Eubacteriales | Clostridia | Firmicutes | Bacteria |
| ASV985 | 427 | 1 | 1 | 427 | 100 | NR_104781.1 | Treponema bryantii strain RUS-1 16S ribosomal RNA, partial sequence | NR_104781.1 | 1535 | 351 | 778 | 27.88 | 557 | 3E-158 | 386/428 | 90 | 1/428 | 0 | Plus | Plus | Bacteria; Spirochaetes; Spirochaetia; Spirochaetales; Spirochaetaceae; Treponema; Treponema bryantii | Treponema bryantii | Treponema | Spirochaetaceae | Spirochaetales | Spirochaetia | Spirochaetes | Bacteria |
| ASV986 | 422 | 1 | 1 | 422 | 100 | NR_146018.1 | Paludibacter jiangxiensis strain NM7 16S ribosomal RNA, partial sequence | NR_146018.1 | 1469 | 340 | 761 | 28.73 | 503.4 | 4E-142 | 375/425 | 88 | 6/425 | 1 | Plus | Plus | Bacteria; Bacteroidetes; Bacteroidia; Bacteroidales; Paludibacteraceae; Paludibacter; Paludibacter jiangxiensis | Paludibacter jiangxiensis | Paludibacter | Paludibacteraceae | Bacteroidales | Bacteroidia | Bacteroidetes | Bacteria |
| ASV987 | 402 | 1 | 1 | 402 | 100 | NR_029239.3 | Clostridium chartatabidum strain 163 16S ribosomal RNA, partial sequence | NR_029239.3 | 1460 | 323 | 724 | 27.53 | 710.2 | 0 | 396/402 | 98 | 0/402 | 0 | Plus | Plus | Bacteria; Firmicutes; Clostridia; Eubacteriales; Clostridiaceae; Clostridium; Clostridium chartatabidum | Clostridium chartatabidum | Clostridium | Clostridiaceae | Eubacteriales | Clostridia | Firmicutes | Bacteria |
| ASV989 | 403 | 1 | 1 | 403 | 100 | NR_147378.1 | Negativibacillus massiliensis strain Marseille-P3213 16S ribosomal RNA, partial sequence | NR_147378.1 | 1479 | 356 | 758 | 27.25 | 706.5 | 0 | 396/403 | 98 | 0/403 | 0 | Plus | Plus | Bacteria; Firmicutes; ; ; ; Negativibacillus; Negativibacillus massiliensis | Negativibacillus massiliensis | Negativibacillus | | | | Firmicutes | Bacteria |
| ASV991 | 404 | 1 | 1 | 404 | 100 | NR_118156.1 | Oscillibacter ruminantium GH1 16S ribosomal RNA, partial sequence | NR_118156.1 | 1409 | 321 | 724 | 28.67 | 475.7 | 7E-134 | 358/407 | 87 | 6/407 | 1 | Plus | Plus | Bacteria; Firmicutes; Clostridia; Eubacteriales; Oscillospiraceae; Oscillibacter; Oscillibacter ruminantium | Oscillibacter ruminantium | Oscillibacter | Oscillospiraceae | Eubacteriales | Clostridia | Firmicutes | Bacteria |
| ASV992 | 421 | 2 | 1 | 421 | 100 | NR_113073.1 | Barnesiella intestinihominis strain JCM 15079 16S ribosomal RNA, partial sequence | NR_113073.1 | 1481 | 346 | 767 | 28.49 | 468.3 | 1E-131 | 367/423 | 86 | 3/423 | 0 | Plus | Plus | Bacteria; Bacteroidetes; Bacteroidia; Bacteroidales; Barnesiellaceae; Barnesiella; Barnesiella intestinihominis | Barnesiella intestinihominis | Barnesiella | Barnesiellaceae | Bacteroidales | Bacteroidia | Bacteroidetes | Bacteria |
| ASV993 | 422 | 1 | 1 | 422 | 100 | NR_041364.1 | Prevotella stercorea DSM 18206 strain CB35 16S ribosomal RNA, partial sequence | NR_041364.1 | 1488 | 354 | 775 | 28.36 | 603.1 | 3E-172 | 390/422 | 92 | 0/422 | 0 | Plus | Plus | Bacteria; Bacteroidetes; Bacteroidia; Bacteroidales; Prevotellaceae; Prevotella; Prevotella stercorea | Prevotella stercorea | Prevotella | Prevotellaceae | Bacteroidales | Bacteroidia | Bacteroidetes | Bacteria |
| ASV994 | 421 | 2 | 1 | 421 | 100 | NR_113076.1 | Parabacteroides goldsteinii DSM 19448 = WAL 12034 strain JCM 13446 16S ribosomal RNA, partial sequence | NR_113076.1 | 1476 | 341 | 762 | 28.59 | 529.3 | 6E-150 | 378/423 | 89 | 3/423 | 0 | Plus | Plus | Bacteria; Bacteroidetes; Bacteroidia; Bacteroidales; Tannerellaceae; Parabacteroides; Parabacteroides goldsteinii | Parabacteroides goldsteinii | Parabacteroides | Tannerellaceae | Bacteroidales | Bacteroidia | Bacteroidetes | Bacteria |
| ASV995 | 405 | 1 | 1 | 405 | 100 | NR_118554.1 | Intestinimonas butyriciproducens strain SRB-521-5-I 16S ribosomal RNA, partial sequence | NR_118554.1 | 1475 | 346 | 750 | 27.46 | 533 | 4E-151 | 367/406 | 90 | 2/406 | 0 | Plus | Plus | Bacteria; Firmicutes; Clostridia; Eubacteriales; ; Intestinimonas; Intestinimonas butyriciproducens | Intestinimonas butyriciproducens | Intestinimonas | | Eubacteriales | Clostridia | Firmicutes | Bacteria |
| ASV996 | 421 | 2 | 1 | 421 | 100 | NR_113077.1 | Paraprevotella clara strain JCM 14859 16S ribosomal RNA, partial sequence | NR_113077.1 | 1480 | 346 | 766 | 28.45 | 507.1 | 3E-143 | 373/422 | 88 | 2/422 | 0 | Plus | Plus | Bacteria; Bacteroidetes; Bacteroidia; Bacteroidales; Prevotellaceae; Paraprevotella; Paraprevotella clara | Paraprevotella clara | Paraprevotella | Prevotellaceae | Bacteroidales | Bacteroidia | Bacteroidetes | Bacteria |
| ASV997 | 405 | 1 | 1 | 405 | 100 | NR_044972.1 | Sporobacter termitidis strain SYR 16S ribosomal RNA, partial sequence | NR_044972.1 | 1506 | 348 | 752 | 26.89 | 536.6 | 3E-152 | 367/406 | 90 | 2/406 | 0 | Plus | Plus | Bacteria; Firmicutes; Clostridia; Eubacteriales; Oscillospiraceae; Sporobacter; Sporobacter termitidis | Sporobacter termitidis | Sporobacter | Oscillospiraceae | Eubacteriales | Clostridia | Firmicutes | Bacteria |
| ASV998 | 427 | 1 | 1 | 427 | 100 | NR_041666.1 | Dialister succinatiphilus YIT 11850 16S ribosomal RNA, partial sequence | NR_041666.1 | 1521 | 353 | 779 | 28.07 | 784.1 | 0 | 426/427 | 99 | 0/427 | 0 | Plus | Plus | Bacteria; Firmicutes; Negativicutes; Veillonellales; Veillonellaceae; Dialister; Dialister succinatiphilus | Dialister succinatiphilus | Dialister | Veillonellaceae | Veillonellales | Negativicutes | Firmicutes | Bacteria |
| ASV999 | 402 | 1 | 1 | 402 | 100 | NR_029097.1 | Oribacterium sinus strain AIP 354.02 16S ribosomal RNA, partial sequence | NR_029097.1 | 1374 | 308 | 709 | 29.26 | 616.1 | 4E-176 | 380/403 | 94 | 2/403 | 0 | Plus | Plus | Bacteria; Firmicutes; Clostridia; Eubacteriales; Lachnospiraceae; Oribacterium; Oribacterium sinus | Oribacterium sinus | Oribacterium | Lachnospiraceae | Eubacteriales | Clostridia | Firmicutes | Bacteria |
| ASV1000 | 404 | 2 | 1 | 404 | 100 | NR_074793.2 | Oscillibacter valericigenes strain Sjm18-20 16S ribosomal RNA, complete sequence | NR_074793.2 | 1530 | 372 | 775 | 26.41 | 647.4 | 0 | 386/404 | 95 | 0/404 | 0 | Plus | Plus | Bacteria; Firmicutes; Clostridia; Eubacteriales; Oscillospiraceae; Oscillibacter; Oscillibacter valericigenes | Oscillibacter valericigenes | Oscillibacter | Oscillospiraceae | Eubacteriales | Clostridia | Firmicutes | Bacteria |
| ASV1001 | 422 | 1 | 1 | 422 | 100 | NR_041364.1 | Prevotella stercorea DSM 18206 strain CB35 16S ribosomal RNA, partial sequence | NR_041364.1 | 1488 | 354 | 775 | 28.36 | 725 | 0 | 412/422 | 97 | 0/422 | 0 | Plus | Plus | Bacteria; Bacteroidetes; Bacteroidia; Bacteroidales; Prevotellaceae; Prevotella; Prevotella stercorea | Prevotella stercorea | Prevotella | Prevotellaceae | Bacteroidales | Bacteroidia | Bacteroidetes | Bacteria |
| ASV1002 | 404 | 1 | 1 | 404 | 100 | NR_118156.1 | Oscillibacter ruminantium GH1 16S ribosomal RNA, partial sequence | NR_118156.1 | 1409 | 321 | 724 | 28.67 | 608.7 | 7E-174 | 381/406 | 93 | 4/406 | 0 | Plus | Plus | Bacteria; Firmicutes; Clostridia; Eubacteriales; Oscillospiraceae; Oscillibacter; Oscillibacter ruminantium | Oscillibacter ruminantium | Oscillibacter | Oscillospiraceae | Eubacteriales | Clostridia | Firmicutes | Bacteria |
| ASV1003 | 422 | 2 | 1 | 422 | 100 | NR_114009.1 | Galbibacter mesophilus strain NBRC 101624 16S ribosomal RNA, partial sequence | NR_114009.1 | 1445 | 328 | 749 | 29.2 | 431.4 | 2E-120 | 360/423 | 85 | 2/423 | 0 | Plus | Plus | Bacteria; Bacteroidetes; Flavobacteriia; Flavobacteriales; Flavobacteriaceae; Galbibacter; Galbibacter mesophilus | Galbibacter mesophilus | Galbibacter | Flavobacteriaceae | Flavobacteriales | Flavobacteriia | Bacteroidetes | Bacteria |
| ASV1004 | 405 | 1 | 1 | 405 | 100 | NR_114789.1 | [Clostridium] leptum strain DSM 753 16S ribosomal RNA, partial sequence | NR_114789.1 | 1505 | 364 | 765 | 26.71 | 529.3 | 6E-150 | 366/405 | 90 | 3/405 | 0 | Plus | Plus | Bacteria; Firmicutes; Clostridia; Eubacteriales; Oscillospiraceae; ; [Clostridium] leptum | [Clostridium] leptum | | Oscillospiraceae | Eubacteriales | Clostridia | Firmicutes | Bacteria |
| ASV1005 | 422 | 1 | 1 | 422 | 100 | NR_113411.1 | Prevotella copri DSM 18205 strain JCM 13464 16S ribosomal RNA, partial sequence | NR_113411.1 | 1493 | 358 | 779 | 28.27 | 702.8 | 0 | 409/423 | 96 | 2/423 | 0 | Plus | Plus | Bacteria; Bacteroidetes; Bacteroidia; Bacteroidales; Prevotellaceae; Prevotella; Prevotella copri | Prevotella copri | Prevotella | Prevotellaceae | Bacteroidales | Bacteroidia | Bacteroidetes | Bacteria |
| ASV1006 | 427 | 1 | 1 | 427 | 100 | NR_112652.1 | Lactobacillus equicursoris DSM 19284 = JCM 14600 = CIP 110162 strain DI70 16S ribosomal RNA, partial sequence | NR_112652.1 | 1486 | 351 | 777 | 28.73 | 789.6 | 0 | 427/427 | 100 | 0/427 | 0 | Plus | Plus | Bacteria; Firmicutes; Bacilli; Lactobacillales; Lactobacillaceae; Lactobacillus; Lactobacillus equicursoris | Lactobacillus equicursoris | Lactobacillus | Lactobacillaceae | Lactobacillales | Bacilli | Firmicutes | Bacteria |
| ASV1007 | 427 | 1 | 1 | 427 | 100 | NR_118847.1 | Actinobacillus minor NM305 16S ribosomal RNA, partial sequence | NR_118847.1 | 1464 | 333 | 759 | 29.17 | 774.9 | 0 | 424/427 | 99 | 0/427 | 0 | Plus | Plus | Bacteria; Proteobacteria; Gammaproteobacteria; Pasteurellales; Pasteurellaceae; Actinobacillus; Actinobacillus minor | Actinobacillus minor | Actinobacillus | Pasteurellaceae | Pasteurellales | Gammaproteobacteria | Proteobacteria | Bacteria |
| ASV1008 | 406 | 1 | 1 | 406 | 100 | NR_165727.1 | Anaerotignum faecicola strain KGMB03357 16S ribosomal RNA, complete sequence | NR_165727.1 | 1535 | 375 | 780 | 26.45 | 640.1 | 0 | 387/407 | 95 | 2/407 | 0 | Plus | Plus | Bacteria; Firmicutes; Clostridia; Eubacteriales; Lachnospiraceae; Anaerotignum; Anaerotignum faecicola | Anaerotignum faecicola | Anaerotignum | Lachnospiraceae | Eubacteriales | Clostridia | Firmicutes | Bacteria |
| ASV1009 | 402 | 1 | 1 | 402 | 100 | NR_118673.1 | [Eubacterium] hallii strain ATCC 27751 16S ribosomal RNA, partial sequence | NR_118673.1 | 1479 | 371 | 772 | 27.18 | 638.2 | 0 | 382/402 | 95 | 0/402 | 0 | Plus | Plus | Bacteria; Firmicutes; Clostridia; Eubacteriales; Lachnospiraceae; Anaerobutyricum; Anaerobutyricum hallii | Anaerobutyricum hallii | Anaerobutyricum | Lachnospiraceae | Eubacteriales | Clostridia | Firmicutes | Bacteria |
| ASV1010 | 405 | 1 | 1 | 405 | 100 | NR_147370.1 | Pseudoflavonifractor phocaeensis strain Marseille-P3064 16S ribosomal RNA, partial sequence | NR_147370.1 | 1495 | 366 | 770 | 27.09 | 688.1 | 0 | 394/405 | 97 | 0/405 | 0 | Plus | Plus | Bacteria; Firmicutes; Clostridia; Eubacteriales; Oscillospiraceae; Pseudoflavonifractor; Pseudoflavonifractor phocaeensis | Pseudoflavonifractor phocaeensis | Pseudoflavonifractor | Oscillospiraceae | Eubacteriales | Clostridia | Firmicutes | Bacteria |
| ASV1011 | 422 | 2 | 1 | 422 | 100 | NR_113119.1 | Prevotella oris strain JCM 8540 16S ribosomal RNA, partial sequence | NR_113119.1 | 1481 | 345 | 766 | 28.49 | 603.1 | 3E-172 | 392/424 | 92 | 4/424 | 0 | Plus | Plus | Bacteria; Bacteroidetes; Bacteroidia; Bacteroidales; Prevotellaceae; Prevotella; Prevotella oris | Prevotella oris | Prevotella | Prevotellaceae | Bacteroidales | Bacteroidia | Bacteroidetes | Bacteria |
| ASV1013 | 402 | 1 | 1 | 402 | 100 | NR_113021.1 | Clostridium paraputrificum strain JCM 1293 16S ribosomal RNA, partial sequence | NR_113021.1 | 1476 | 348 | 749 | 27.24 | 704.7 | 0 | 395/402 | 98 | 0/402 | 0 | Plus | Plus | Bacteria; Firmicutes; Clostridia; Eubacteriales; Clostridiaceae; Clostridium; Clostridium paraputrificum | Clostridium paraputrificum | Clostridium | Clostridiaceae | Eubacteriales | Clostridia | Firmicutes | Bacteria |
| ASV1014 | 405 | 3 | 1 | 405 | 100 | NR_115307.1 | Ethanoligenens harbinense YUAN-3 16S ribosomal RNA, partial sequence | NR_115307.1 | 1497 | 340 | 743 | 26.99 | 531.1 | 2E-150 | 368/407 | 90 | 5/407 | 1 | Plus | Plus | Bacteria; Firmicutes; Clostridia; Eubacteriales; Oscillospiraceae; Ethanoligenens; Ethanoligenens harbinense | Ethanoligenens harbinense | Ethanoligenens | Oscillospiraceae | Eubacteriales | Clostridia | Firmicutes | Bacteria |
| ASV1015 | 402 | 1 | 1 | 402 | 100 | NR_104846.1 | Gemmiger formicilis strain X2-56 16S ribosomal RNA, partial sequence | NR_104846.1 | 1426 | 305 | 706 | 28.19 | 737.9 | 0 | 401/402 | 99 | 0/402 | 0 | Plus | Plus | Bacteria; Firmicutes; Clostridia; Eubacteriales; ; Gemmiger; Gemmiger formicilis | Gemmiger formicilis | Gemmiger | | Eubacteriales | Clostridia | Firmicutes | Bacteria |
| ASV1016 | 404 | 1 | 1 | 404 | 100 | NR_144611.1 | Flintibacter butyricus strain BLS21 16S ribosomal RNA, partial sequence | NR_144611.1 | 808 | 299 | 703 | 50.12 | 630.8 | 2E-180 | 384/405 | 94 | 1/405 | 0 | Plus | Plus | Bacteria; Firmicutes; Clostridia; Eubacteriales; ; Flintibacter; Flintibacter butyricus | Flintibacter butyricus | Flintibacter | | Eubacteriales | Clostridia | Firmicutes | Bacteria |
| ASV1017 | 405 | 2 | 1 | 405 | 100 | NR_074629.1 | Hungateiclostridium thermocellum strain ATCC 27405 16S ribosomal RNA, partial sequence | NR_074629.1 | 1521 | 358 | 762 | 26.63 | 571.7 | 9E-163 | 373/405 | 92 | 0/405 | 0 | Plus | Plus | Bacteria; Firmicutes; Clostridia; Eubacteriales; Oscillospiraceae; Acetivibrio; Acetivibrio thermocellus | Acetivibrio thermocellus | Acetivibrio | Oscillospiraceae | Eubacteriales | Clostridia | Firmicutes | Bacteria |
| ASV1018 | 426 | 1 | 1 | 426 | 100 | NR_041304.1 | Sharpea azabuensis strain ST18 16S ribosomal RNA, partial sequence | NR_041304.1 | 1460 | 325 | 750 | 29.18 | 754.6 | 0 | 420/426 | 98 | 0/426 | 0 | Plus | Plus | Bacteria; Firmicutes; Erysipelotrichia; Erysipelotrichales; Coprobacillaceae; Sharpea; Sharpea azabuensis | Sharpea azabuensis | Sharpea | Coprobacillaceae | Erysipelotrichales | Erysipelotrichia | Firmicutes | Bacteria |
| ASV1019 | 402 | 1 | 1 | 402 | 100 | NR_118673.1 | [Eubacterium] hallii strain ATCC 27751 16S ribosomal RNA, partial sequence | NR_118673.1 | 1479 | 371 | 772 | 27.18 | 699.2 | 0 | 393/402 | 97 | 0/402 | 0 | Plus | Plus | Bacteria; Firmicutes; Clostridia; Eubacteriales; Lachnospiraceae; Anaerobutyricum; Anaerobutyricum hallii | Anaerobutyricum hallii | Anaerobutyricum | Lachnospiraceae | Eubacteriales | Clostridia | Firmicutes | Bacteria |
| ASV1020 | 422 | 1 | 1 | 422 | 100 | NR_144750.1 | Prevotellamassilia timonensis strain Marseille-P2831 16S ribosomal RNA, partial sequence | NR_144750.1 | 1493 | 358 | 779 | 28.27 | 664.1 | 0 | 401/422 | 95 | 0/422 | 0 | Plus | Plus | Bacteria; Bacteroidetes; Bacteroidia; Bacteroidales; Prevotellaceae; Prevotellamassilia; Prevotellamassilia timonensis | Prevotellamassilia timonensis | Prevotellamassilia | Prevotellaceae | Bacteroidales | Bacteroidia | Bacteroidetes | Bacteria |
| ASV1021 | 403 | 2 | 3 | 403 | 99.5 | NR_148837.1 | Abyssivirga alkaniphila strain L81 16S ribosomal RNA, partial sequence | NR_148837.1 | 1520 | 366 | 765 | 26.32 | 446.2 | 6E-125 | 350/403 | 86 | 5/403 | 1 | Plus | Plus | Bacteria; Firmicutes; Clostridia; Eubacteriales; Lachnospiraceae; Abyssivirga; Abyssivirga alkaniphila | Abyssivirga alkaniphila | Abyssivirga | Lachnospiraceae | Eubacteriales | Clostridia | Firmicutes | Bacteria |
| ASV1022 | 405 | 1 | 1 | 405 | 100 | NR_118554.1 | Intestinimonas butyriciproducens strain SRB-521-5-I 16S ribosomal RNA, partial sequence | NR_118554.1 | 1475 | 346 | 750 | 27.46 | 588.4 | 9E-168 | 376/405 | 92 | 0/405 | 0 | Plus | Plus | Bacteria; Firmicutes; Clostridia; Eubacteriales; ; Intestinimonas; Intestinimonas butyriciproducens | Intestinimonas butyriciproducens | Intestinimonas | | Eubacteriales | Clostridia | Firmicutes | Bacteria |
| ASV1023 | 405 | 1 | 1 | 405 | 100 | NR_144611.1 | Flintibacter butyricus strain BLS21 16S ribosomal RNA, partial sequence | NR_144611.1 | 808 | 299 | 703 | 50.12 | 693.6 | 0 | 395/405 | 97 | 0/405 | 0 | Plus | Plus | Bacteria; Firmicutes; Clostridia; Eubacteriales; ; Flintibacter; Flintibacter butyricus | Flintibacter butyricus | Flintibacter | | Eubacteriales | Clostridia | Firmicutes | Bacteria |
| ASV1024 | 427 | 1 | 1 | 427 | 100 | NR_112902.1 | Phascolarctobacterium succinatutens strain YIT 12067 16S ribosomal RNA, partial sequence | NR_112902.1 | 1610 | 444 | 870 | 26.52 | 761.9 | 0 | 422/427 | 98 | 0/427 | 0 | Plus | Plus | Bacteria; Firmicutes; Negativicutes; Acidaminococcales; Acidaminococcaceae; Phascolarctobacterium; Phascolarctobacterium succinatutens | Phascolarctobacterium succinatutens | Phascolarctobacterium | Acidaminococcaceae | Acidaminococcales | Negativicutes | Firmicutes | Bacteria |
| ASV1025 | 428 | 1 | 1 | 428 | 100 | NR_104781.1 | Treponema bryantii strain RUS-1 16S ribosomal RNA, partial sequence | NR_104781.1 | 1535 | 351 | 778 | 27.88 | 641.9 | 0 | 402/429 | 93 | 2/429 | 0 | Plus | Plus | Bacteria; Spirochaetes; Spirochaetia; Spirochaetales; Spirochaetaceae; Treponema; Treponema bryantii | Treponema bryantii | Treponema | Spirochaetaceae | Spirochaetales | Spirochaetia | Spirochaetes | Bacteria |
| ASV1026 | 422 | 2 | 1 | 422 | 100 | NR_113117.1 | Prevotella oralis ATCC 33269 strain JCM 12251 16S ribosomal RNA, partial sequence | NR_113117.1 | 1496 | 361 | 782 | 28.21 | 625.3 | 7E-179 | 394/422 | 93 | 0/422 | 0 | Plus | Plus | Bacteria; Bacteroidetes; Bacteroidia; Bacteroidales; Prevotellaceae; Prevotella; Prevotella oralis | Prevotella oralis | Prevotella | Prevotellaceae | Bacteroidales | Bacteroidia | Bacteroidetes | Bacteria |
| ASV1028 | 404 | 1 | 1 | 404 | 100 | NR_118554.1 | Intestinimonas butyriciproducens strain SRB-521-5-I 16S ribosomal RNA, partial sequence | NR_118554.1 | 1475 | 346 | 750 | 27.46 | 514.5 | 2E-145 | 363/405 | 89 | 1/405 | 0 | Plus | Plus | Bacteria; Firmicutes; Clostridia; Eubacteriales; ; Intestinimonas; Intestinimonas butyriciproducens | Intestinimonas butyriciproducens | Intestinimonas | | Eubacteriales | Clostridia | Firmicutes | Bacteria |
| ASV1029 | 427 | 2 | 1 | 427 | 100 | NR_037018.1 | Acidaminococcus fermentans strain VR4 16S ribosomal RNA gene, partial sequence | NR_037018.1 | 1516 | 349 | 775 | 28.17 | 756.4 | 0 | 421/427 | 98 | 0/427 | 0 | Plus | Plus | Bacteria; Firmicutes; Negativicutes; Acidaminococcales; Acidaminococcaceae; Acidaminococcus; Acidaminococcus fermentans | Acidaminococcus fermentans | Acidaminococcus | Acidaminococcaceae | Acidaminococcales | Negativicutes | Firmicutes | Bacteria |
| ASV1030 | 426 | 1 | 1 | 426 | 100 | NR_114696.1 | Anaerovibrio lipolyticus DSM 3074 16S ribosomal RNA, partial sequence | NR_114696.1 | 1526 | 372 | 797 | 27.92 | 688.1 | 0 | 411/429 | 95 | 6/429 | 1 | Plus | Plus | Bacteria; Firmicutes; Negativicutes; Selenomonadales; Selenomonadaceae; Anaerovibrio; Anaerovibrio lipolyticus | Anaerovibrio lipolyticus | Anaerovibrio | Selenomonadaceae | Selenomonadales | Negativicutes | Firmicutes | Bacteria |
| ASV1031 | 428 | 1 | 1 | 428 | 100 | NR_104781.1 | Treponema bryantii strain RUS-1 16S ribosomal RNA, partial sequence | NR_104781.1 | 1535 | 351 | 778 | 27.88 | 636.4 | 0 | 401/429 | 93 | 2/429 | 0 | Plus | Plus | Bacteria; Spirochaetes; Spirochaetia; Spirochaetales; Spirochaetaceae; Treponema; Treponema bryantii | Treponema bryantii | Treponema | Spirochaetaceae | Spirochaetales | Spirochaetia | Spirochaetes | Bacteria |
| ASV1032 | 402 | 1 | 1 | 402 | 100 | NR_118675.1 | [Eubacterium] siraeum strain ATCC 29066 16S ribosomal RNA, partial sequence | NR_118675.1 | 1453 | 356 | 757 | 27.67 | 712.1 | 0 | 395/402 | 98 | 0/402 | 0 | Plus | Plus | Bacteria; Firmicutes; Clostridia; Eubacteriales; Oscillospiraceae; ; [Eubacterium] siraeum | [Eubacterium] siraeum | | Oscillospiraceae | Eubacteriales | Clostridia | Firmicutes | Bacteria |
| ASV1033 | 402 | 1 | 1 | 402 | 100 | NR_102884.1 | Ruminococcus champanellensis 18P13 = JCM 17042 16S ribosomal RNA, partial sequence | NR_102884.1 | 1494 | 348 | 749 | 26.91 | 582.8 | 4E-166 | 373/402 | 92 | 0/402 | 0 | Plus | Plus | Bacteria; Firmicutes; Clostridia; Eubacteriales; Oscillospiraceae; Ruminococcus; Ruminococcus champanellensis | Ruminococcus champanellensis | Ruminococcus | Oscillospiraceae | Eubacteriales | Clostridia | Firmicutes | Bacteria |
| ASV1034 | 422 | 5 | 1 | 422 | 100 | NR_044864.1 | Hallella seregens ATCC 51272 16S ribosomal RNA, partial sequence | NR_044864.1 | 1463 | 335 | 756 | 28.84 | 614.2 | 2E-175 | 393/423 | 92 | 2/423 | 0 | Plus | Plus | Bacteria; Bacteroidetes; Bacteroidia; Bacteroidales; Prevotellaceae; Prevotella; Hallella seregens | Hallella seregens | Prevotella | Prevotellaceae | Bacteroidales | Bacteroidia | Bacteroidetes | Bacteria |
| ASV1035 | 405 | 1 | 1 | 405 | 100 | NR_144742.1 | Christensenella massiliensis strain Marseille-P2438 16S ribosomal RNA, partial sequence | NR_144742.1 | 1499 | 369 | 773 | 27.02 | 472 | 1E-132 | 358/408 | 87 | 6/408 | 1 | Plus | Plus | Bacteria; Firmicutes; Clostridia; Eubacteriales; Christensenellaceae; Christensenella; Christensenella massiliensis | Christensenella massiliensis | Christensenella | Christensenellaceae | Eubacteriales | Clostridia | Firmicutes | Bacteria |
| ASV1036 | 402 | 1 | 1 | 402 | 100 | NR_025931.1 | Ruminococcus flavefaciens strain C94 16S ribosomal RNA, partial sequence | NR_025931.1 | 1388 | 325 | 726 | 28.96 | 638.2 | 0 | 383/402 | 95 | 0/402 | 0 | Plus | Plus | Bacteria; Firmicutes; Clostridia; Eubacteriales; Oscillospiraceae; Ruminococcus; Ruminococcus flavefaciens | Ruminococcus flavefaciens | Ruminococcus | Oscillospiraceae | Eubacteriales | Clostridia | Firmicutes | Bacteria |
| ASV1037 | 421 | 1 | 1 | 421 | 100 | NR_144616.1 | Muribaculum intestinale strain YL27 16S ribosomal RNA, partial sequence | NR_144616.1 | 1460 | 297 | 718 | 28.9 | 557 | 3E-158 | 385/425 | 90 | 7/425 | 1 | Plus | Plus | Bacteria; Bacteroidetes; Bacteroidia; Bacteroidales; Muribaculaceae; Muribaculum; Muribaculum intestinale | Muribaculum intestinale | Muribaculum | Muribaculaceae | Bacteroidales | Bacteroidia | Bacteroidetes | Bacteria |
| ASV1038 | 427 | 2 | 1 | 427 | 100 | NR_037018.1 | Acidaminococcus fermentans strain VR4 16S ribosomal RNA gene, partial sequence | NR_037018.1 | 1516 | 349 | 775 | 28.17 | 761.9 | 0 | 422/427 | 98 | 0/427 | 0 | Plus | Plus | Bacteria; Firmicutes; Negativicutes; Acidaminococcales; Acidaminococcaceae; Acidaminococcus; Acidaminococcus fermentans | Acidaminococcus fermentans | Acidaminococcus | Acidaminococcaceae | Acidaminococcales | Negativicutes | Firmicutes | Bacteria |
| ASV1040 | 421 | 2 | 1 | 421 | 100 | NR_113073.1 | Barnesiella intestinihominis strain JCM 15079 16S ribosomal RNA, partial sequence | NR_113073.1 | 1481 | 346 | 767 | 28.49 | 462.8 | 6E-130 | 366/423 | 86 | 3/423 | 0 | Plus | Plus | Bacteria; Bacteroidetes; Bacteroidia; Bacteroidales; Barnesiellaceae; Barnesiella; Barnesiella intestinihominis | Barnesiella intestinihominis | Barnesiella | Barnesiellaceae | Bacteroidales | Bacteroidia | Bacteroidetes | Bacteria |
| ASV1041 | 405 | 2 | 1 | 405 | 100 | NR_144742.1 | Christensenella massiliensis strain Marseille-P2438 16S ribosomal RNA, partial sequence | NR_144742.1 | 1499 | 369 | 773 | 27.02 | 455.4 | 1E-127 | 352/405 | 86 | 0/405 | 0 | Plus | Plus | Bacteria; Firmicutes; Clostridia; Eubacteriales; Christensenellaceae; Christensenella; Christensenella massiliensis | Christensenella massiliensis | Christensenella | Christensenellaceae | Eubacteriales | Clostridia | Firmicutes | Bacteria |
| ASV1042 | 402 | 3 | 1 | 402 | 100 | NR_026409.1 | [Desulfotomaculum] guttoideum strain DSM 4024 16S ribosomal RNA, partial sequence | NR_026409.1 | 1495 | 358 | 759 | 26.89 | 677 | 0 | 390/402 | 97 | 0/402 | 0 | Plus | Plus | Bacteria; Firmicutes; Clostridia; Eubacteriales; Lachnospiraceae; Lacrimispora; Lacrimispora sphenoides | Lacrimispora sphenoides | Lacrimispora | Lachnospiraceae | Eubacteriales | Clostridia | Firmicutes | Bacteria |
| ASV1043 | 422 | 2 | 1 | 422 | 100 | NR_113117.1 | Prevotella oralis ATCC 33269 strain JCM 12251 16S ribosomal RNA, partial sequence | NR_113117.1 | 1496 | 361 | 782 | 28.21 | 630.8 | 2E-180 | 395/422 | 93 | 0/422 | 0 | Plus | Plus | Bacteria; Bacteroidetes; Bacteroidia; Bacteroidales; Prevotellaceae; Prevotella; Prevotella oralis | Prevotella oralis | Prevotella | Prevotellaceae | Bacteroidales | Bacteroidia | Bacteroidetes | Bacteria |
| ASV1044 | 422 | 1 | 1 | 422 | 100 | NR_144747.1 | Mediterranea massiliensis strain Marseille-P2645 16S ribosomal RNA, partial sequence | NR_144747.1 | 1491 | 354 | 775 | 28.3 | 741.6 | 0 | 415/422 | 98 | 0/422 | 0 | Plus | Plus | Bacteria; Bacteroidetes; Bacteroidia; Bacteroidales; Bacteroidaceae; Mediterranea; Mediterranea massiliensis | Mediterranea massiliensis | Mediterranea | Bacteroidaceae | Bacteroidales | Bacteroidia | Bacteroidetes | Bacteria |
| ASV1045 | 404 | 1 | 1 | 404 | 100 | NR_118156.1 | Oscillibacter ruminantium GH1 16S ribosomal RNA, partial sequence | NR_118156.1 | 1409 | 321 | 724 | 28.67 | 625.3 | 7E-179 | 382/404 | 94 | 0/404 | 0 | Plus | Plus | Bacteria; Firmicutes; Clostridia; Eubacteriales; Oscillospiraceae; Oscillibacter; Oscillibacter ruminantium | Oscillibacter ruminantium | Oscillibacter | Oscillospiraceae | Eubacteriales | Clostridia | Firmicutes | Bacteria |

| | | | | | | | |
|---|---|---|---|---|---|---|---|
| ASV1046 | 428 | 1 | 1 | 428 | 100 | NR_104781.1 Treponema bryantii strain RUS-1 16S ribosomal RNA, partial sequence | NR_104781.1 |
| ASV1047 | 405 | 2 | 1 | 405 | 100 | NR_147370.1 Pseudoflavonifractor phocaeensis strain Marseille-P3064 16S ribosomal RNA, partial sequence | NR_147370.1 |
| ASV1048 | 427 | 1 | 1 | 427 | 100 | NR_044111.1 Selenomonas bovis strain WG 16S ribosomal RNA, partial sequence | NR_044111.1 |
| ASV1049 | 404 | 1 | 1 | 404 | 100 | NR_125464.1 Anaerobacterium chartisolvens strain T-1-35 16S ribosomal RNA, partial sequence | NR_125464.1 |
| ASV1050 | 402 | 2 | 1 | 402 | 100 | NR_115105.1 Helicobacter equorum strain CCUG 52199 16S ribosomal RNA, partial sequence | NR_115105.1 |
| ASV1051 | 402 | 1 | 3 | 402 | 99.5 | NR_125677.1 Vallitalea pronyensis strain FatNI3 16S ribosomal RNA, partial sequence | NR_125677.1 |
| ASV1052 | 401 | 1 | 1 | 401 | 100 | NR_144737.1 Emergencia timonensis strain SN18 16S ribosomal RNA, partial sequence | NR_144737.1 |
| ASV1053 | 429 | 1 | 1 | 429 | 100 | NR_042769.1 Geobacter bemidjiensis Bem 16S ribosomal RNA, partial sequence | NR_042769.1 |
| ASV1054 | 422 | 2 | 1 | 422 | 100 | NR_113119.1 Prevotella oris strain JCM 8540 16S ribosomal RNA, partial sequence | NR_113119.1 |
| ASV1055 | 427 | 1 | 1 | 427 | 100 | NR_157643.1 Treponema rectale strain CHPA 16S ribosomal RNA, partial sequence | NR_157643.1 |
| ASV1056 | 422 | 1 | 1 | 422 | 100 | NR_113411.1 Prevotella copri DSM 18205 strain JCM 13464 16S ribosomal RNA, partial sequence | NR_113411.1 |
| ASV1057 | 422 | 1 | 1 | 422 | 100 | NR_041364.1 Prevotella stercorea DSM 18206 strain CB35 16S ribosomal RNA, partial sequence | NR_041364.1 |
| ASV1058 | 422 | 1 | 1 | 422 | 100 | NR_151886.1 Prevotella colorans strain A1336 16S ribosomal RNA, partial sequence | NR_151886.1 |
| ASV1059 | 427 | 1 | 1 | 427 | 100 | NR_027596.1 Mitsuokella multacida strain DSM 20544 16S ribosomal RNA, partial sequence | NR_027596.1 |
| ASV1060 | 422 | 1 | 1 | 422 | 100 | NR_037088.1 Alloprevotella tannerae strain VPI N14B 16S ribosomal RNA gene, partial sequence | NR_037088.1 |
| ASV1061 | 427 | 1 | 1 | 427 | 100 | NR_028840.1 Mitsuokella jalaludinii strain M9 16S ribosomal RNA, partial sequence | NR_028840.1 |
| ASV1062 | 427 | 1 | 1 | 427 | 100 | NR_026032.1 [Pasteurella] aerogenes strain P-172-71 16S ribosomal RNA, partial sequence | NR_026032.1 |
| ASV1063 | 405 | 1 | 1 | 405 | 100 | NR_159227.1 Monoglobus pectinilyticus strain 14 16S ribosomal RNA, partial sequence | NR_159227.1 |
| ASV1064 | 427 | 1 | 1 | 427 | 100 | NR_042896.1 Mucispirillum schaedleri strain HRI I17 16S ribosomal RNA, partial sequence | NR_042896.1 |
| ASV1065 | 427 | 1 | 1 | 427 | 100 | NR_041666.1 Dialister succinatiphilus YIT 11850 16S ribosomal RNA, partial sequence | NR_041666.1 |
| ASV1066 | 403 | 2 | 1 | 403 | 100 | NR_024948.1 Campylobacter hyointestinalis subsp. lawsonii strain CHY5 16S ribosomal RNA, partial sequence | NR_024948.1 |
| ASV1067 | 423 | 1 | 1 | 423 | 100 | NR_144750.1 Prevotellamassilia timonensis strain Marseille-P2831 16S ribosomal RNA, partial sequence | NR_144750.1 |
| ASV1069 | 403 | 1 | 1 | 403 | 100 | NR_104907.1 Eubacterium coprostanoligenes strain HL 16S ribosomal RNA, partial sequence | NR_104907.1 |
| ASV1070 | 422 | 2 | 1 | 422 | 100 | NR_040864.1 Bacteroides eggerthii strain DSM 20697 16S ribosomal RNA, partial sequence | NR_040864.1 |
| ASV1071 | 422 | 2 | 1 | 422 | 100 | NR_113117.1 Prevotella oralis ATCC 33269 strain JCM 12251 16S ribosomal RNA, partial sequence | NR_113117.1 |
| ASV1072 | 405 | 3 | 1 | 405 | 100 | NR_118554.1 Intestinimonas butyriciproducens strain SRB-521-5-I 16S ribosomal RNA, partial sequence | NR_118554.1 |
| ASV1073 | 403 | 1 | 1 | 403 | 100 | NR_151900.1 Ruthenibacterium lactatiformans strain 585-1 16S ribosomal RNA, partial sequence | NR_151900.1 |
| ASV1074 | 405 | 2 | 1 | 405 | 100 | NR_144742.1 Christensenella massiliensis strain Marseille-P2438 16S ribosomal RNA, partial sequence | NR_144742.1 |
| ASV1075 | 404 | 1 | 1 | 404 | 100 | NR_025670.1 Pseudoflavonifractor capillosus ATCC 29799 16S ribosomal RNA, partial sequence | NR_025670.1 |
| ASV1076 | 404 | 1 | 1 | 404 | 100 | NR_026476.1 Succinivibrio dextrinosolvens strain 0554 16S ribosomal RNA, partial sequence | NR_026476.1 |
| ASV1077 | 403 | 1 | 1 | 403 | 100 | NR_104907.1 Eubacterium coprostanoligenes strain HL 16S ribosomal RNA, partial sequence | NR_104907.1 |
| ASV1078 | 427 | 1 | 1 | 427 | 100 | NR_044111.1 Selenomonas bovis strain WG 16S ribosomal RNA, partial sequence | NR_044111.1 |
| ASV1079 | 402 | 1 | 1 | 402 | 100 | NR_119085.1 [Clostridium] polysaccharolyticum strain DSM 1801 16S ribosomal RNA, partial sequence | NR_119085.1 |
| ASV1080 | 402 | 1 | 1 | 402 | 100 | NR_074613.1 [Eubacterium] eligens ATCC 27750 16S ribosomal RNA, partial sequence | NR_074613.1 |
| ASV1081 | 402 | 1 | 1 | 402 | 100 | NR_028883.1 Dorea longicatena strain 111-35 16S ribosomal RNA, partial sequence | NR_028883.1 |
| ASV1082 | 422 | 1 | 1 | 422 | 100 | NR_113411.1 Prevotella copri DSM 18205 strain JCM 13464 16S ribosomal RNA, partial sequence | NR_113411.1 |
| ASV1083 | 405 | 1 | 1 | 405 | 100 | NR_044972.1 Sporobacter termitidis strain SYR 16S ribosomal RNA, partial sequence | NR_044972.1 |
| ASV1086 | 427 | 2 | 1 | 427 | 100 | NR_116167.1 Aggregatibacter aphrophilus ATCC 33389 strain CIP 70.73 16S ribosomal RNA, partial sequence | NR_116167.1 |
| ASV1087 | 405 | 3 | 1 | 405 | 100 | NR_119068.1 [Clostridium] aerotolerans strain DSM 5434 16S ribosomal RNA, partial sequence | NR_119068.1 |
| ASV1088 | 404 | 1 | 1 | 404 | 100 | NR_144611.1 Flintibacter butyricus strain BLS21 16S ribosomal RNA, partial sequence | NR_144611.1 |
| ASV1089 | 427 | 1 | 1 | 427 | 100 | NR_027596.1 Mitsuokella multacida strain DSM 20544 16S ribosomal RNA, partial sequence | NR_027596.1 |
| ASV1090 | 405 | 1 | 1 | 405 | 100 | NR_044972.1 Sporobacter termitidis strain SYR 16S ribosomal RNA, partial sequence | NR_044972.1 |
| ASV1091 | 405 | 1 | 1 | 405 | 100 | NR_042834.1 Alkalibacter saccharofermentans strain Z-79820 16S ribosomal RNA, partial sequence | NR_042834.1 |
| ASV1092 | 408 | 1 | 1 | 408 | 100 | NR_029167.1 Anaeroplasma abactoclasticum strain 6-1 16S ribosomal RNA, partial sequence | NR_029167.1 |
| ASV1093 | 404 | 1 | 1 | 404 | 100 | NR_118156.1 Oscillibacter ruminantium GH1 16S ribosomal RNA, partial sequence | NR_118156.1 |
| ASV1094 | 403 | 1 | 1 | 403 | 100 | NR_104907.1 Eubacterium coprostanoligenes strain HL 16S ribosomal RNA, partial sequence | NR_104907.1 |
| ASV1095 | 427 | 1 | 1 | 427 | 100 | NR_028840.1 Mitsuokella jalaludinii strain M9 16S ribosomal RNA, partial sequence | NR_028840.1 |
| ASV1096 | 405 | 1 | 1 | 405 | 100 | NR_025670.1 Pseudoflavonifractor capillosus ATCC 29799 16S ribosomal RNA, partial sequence | NR_025670.1 |
| ASV1097 | 402 | 4 | 1 | 402 | 100 | NR_118730.1 [Clostridium] symbiosum strain ATCC 14940 16S ribosomal RNA, partial sequence | NR_118730.1 |
| ASV1099 | 402 | 1 | 1 | 402 | 100 | NR_151895.1 Anaerocolumna cellulosilytica strain SN021 16S ribosomal RNA, partial sequence | NR_151895.1 |
| ASV1100 | 420 | 1 | 1 | 420 | 100 | NR_170509.1 Duncaniella freteri strain TLL-A3 16S ribosomal RNA, partial sequence | NR_170509.1 |
| ASV1101 | 419 | 1 | 1 | 419 | 100 | NR_042150.1 Fibrobacter intestinalis strain NR9 16S ribosomal RNA, partial sequence | NR_042150.1 |
| ASV1103 | 428 | 1 | 1 | 428 | 100 | NR_104781.1 Treponema bryantii strain RUS-1 16S ribosomal RNA, partial sequence | NR_104781.1 |
| ASV1104 | 402 | 1 | 1 | 402 | 100 | NR_159227.1 Monoglobus pectinilyticus strain 14 16S ribosomal RNA, partial sequence | NR_159227.1 |
| ASV1105 | 405 | 1 | 1 | 405 | 100 | NR_044972.1 Sporobacter termitidis strain SYR 16S ribosomal RNA, partial sequence | NR_044972.1 |
| ASV1106 | 422 | 1 | 1 | 422 | 100 | NR_041342.1 Parabacteroides distasonis strain JCM 5825 16S ribosomal RNA, partial sequence | NR_041342.1 |
| ASV1107 | 403 | 1 | 1 | 403 | 100 | NR_112553.1 [Clostridium] glycyrrhizinilyticum strain ZM35 16S ribosomal RNA, partial sequence | NR_112553.1 |
| ASV1108 | 422 | 2 | 1 | 422 | 100 | NR_074376.1 Parabacteroides distasonis ATCC 8503 16S ribosomal RNA, partial sequence | NR_074376.1 |
| ASV1109 | 402 | 1 | 1 | 402 | 100 | NR_029239.3 Clostridium chartatabidum strain 163 16S ribosomal RNA, partial sequence | NR_029239.3 |
| ASV1110 | 427 | 1 | 1 | 427 | 100 | NR_042942.1 Treponema porcinum strain 14V28 16S ribosomal RNA, partial sequence | NR_042942.1 |
| ASV1111 | 405 | 1 | 1 | 405 | 100 | NR_114789.1 [Clostridium] leptum strain DSM 753 16S ribosomal RNA, partial sequence | NR_114789.1 |
| ASV1112 | 420 | 1 | 1 | 420 | 100 | NR_144616.1 Muribaculum intestinale strain YL27 16S ribosomal RNA, partial sequence | NR_144616.1 |
| ASV1113 | 422 | 1 | 1 | 422 | 100 | NR_144750.1 Prevotellamassilia timonensis strain Marseille-P2831 16S ribosomal RNA, partial sequence | NR_144750.1 |
| ASV1114 | 405 | 1 | 1 | 405 | 100 | NR_044972.1 Sporobacter termitidis strain SYR 16S ribosomal RNA, partial sequence | NR_044972.1 |
| ASV1115 | 402 | 4 | 1 | 402 | 100 | NR_042200.1 [Clostridium] asparagiforme strain N6 16S ribosomal RNA, partial sequence | NR_042200.1 |
| ASV1116 | 422 | 2 | 1 | 422 | 100 | NR_113117.1 Prevotella oralis ATCC 33269 strain JCM 12251 16S ribosomal RNA, partial sequence | NR_113117.1 |
| ASV1117 | 422 | 2 | 1 | 422 | 100 | NR_113105.1 Prevotella histicola strain JCM 15637 16S ribosomal RNA, partial sequence | NR_113105.1 |
| ASV1118 | 402 | 1 | 1 | 402 | 100 | NR_044049.1 Coprococcus eutactus strain ATCC 27759 16S ribosomal RNA, partial sequence | NR_044049.1 |
| ASV1119 | 427 | 2 | 1 | 427 | 100 | NR_025453.1 Streptococcus gallinaceus strain CCUG 42692 16S ribosomal RNA, partial sequence | NR_025453.1 |
| ASV1120 | 427 | 1 | 1 | 427 | 100 | NR_108477.1 Streptococcus porcorum strain 682/03 16S ribosomal RNA, partial sequence | NR_108477.1 |
| ASV1121 | 427 | 1 | 1 | 427 | 100 | NR_044731.2 Holdemanella biformis strain DSM 3989 16S ribosomal RNA, partial sequence | NR_044731.2 |
| ASV1122 | 404 | 2 | 1 | 404 | 100 | NR_074793.2 Oscillibacter valericigenes strain Sjm18-20 16S ribosomal RNA, complete sequence | NR_074793.2 |
| ASV1123 | 401 | 1 | 1 | 401 | 100 | NR_144749.1 Ihubacter massiliensis strain Marseille-P2843 16S ribosomal RNA, partial sequence | NR_144749.1 |
| ASV1124 | 402 | 1 | 1 | 402 | 100 | NR_147375.1 Colidextribacter massiliensis strain Marseille-P3083 16S ribosomal RNA, partial sequence | NR_147375.1 |
| ASV1125 | 418 | 1 | 1 | 418 | 100 | NR_102893.1 Butyrivibrio proteoclasticus strain B316 16S ribosomal RNA, partial sequence | NR_102893.1 |
| ASV1126 | 422 | 1 | 1 | 422 | 100 | NR_041342.1 Parabacteroides distasonis strain JCM 5825 16S ribosomal RNA, partial sequence | NR_041342.1 |
| ASV1127 | 422 | 2 | 1 | 422 | 100 | NR_113119.1 Prevotella oris strain JCM 8540 16S ribosomal RNA, partial sequence | NR_113119.1 |
| ASV1128 | 405 | 1 | 1 | 405 | 100 | NR_147370.1 Pseudoflavonifractor phocaeensis strain Marseille-P3064 16S ribosomal RNA, partial sequence | NR_147370.1 |
| ASV1129 | 421 | 2 | 1 | 421 | 100 | NR_113073.1 Barnesiella intestinihominis strain JCM 15079 16S ribosomal RNA, partial sequence | NR_113073.1 |
| ASV1130 | 421 | 2 | 1 | 421 | 100 | NR_113077.1 Paraprevotella clara strain JCM 14859 16S ribosomal RNA, partial sequence | NR_113077.1 |
| ASV1131 | 403 | 2 | 1 | 403 | 100 | NR_024948.1 Campylobacter hyointestinalis subsp. lawsonii strain CHY5 16S ribosomal RNA, partial sequence | NR_024948.1 |
| ASV1132 | 402 | 1 | 1 | 402 | 100 | NR_118675.1 [Eubacterium] siraeum strain ATCC 29066 16S ribosomal RNA, partial sequence | NR_118675.1 |
| ASV1133 | 405 | 1 | 1 | 405 | 100 | NR_044972.1 Sporobacter termitidis strain SYR 16S ribosomal RNA, partial sequence | NR_044972.1 |
| ASV1134 | 405 | 1 | 1 | 405 | 100 | NR_144611.1 Flintibacter butyricus strain BLS21 16S ribosomal RNA, partial sequence | NR_144611.1 |
| ASV1135 | 427 | 1 | 1 | 427 | 100 | NR_028840.1 Mitsuokella jalaludinii strain M9 16S ribosomal RNA, partial sequence | NR_028840.1 |
| ASV1136 | 427 | 1 | 1 | 427 | 100 | NR_028840.1 Mitsuokella jalaludinii strain M9 16S ribosomal RNA, partial sequence | NR_028840.1 |
| ASV1137 | 402 | 2 | 1 | 402 | 100 | NR_113231.1 Blautia glucerasea strain JCM 17039 16S ribosomal RNA, partial sequence | NR_113231.1 |
| ASV1138 | 422 | 1 | 1 | 422 | 100 | NR_113411.1 Prevotella copri DSM 18205 strain JCM 13464 16S ribosomal RNA, partial sequence | NR_113411.1 |
| ASV1139 | 422 | 1 | 1 | 422 | 100 | NR_151886.1 Prevotella colorans strain A1336 16S ribosomal RNA, partial sequence | NR_151886.1 |
| ASV1140 | 427 | 1 | 1 | 427 | 100 | NR_041611.1 Lactobacillus ruminis strain NBRC 102161 16S ribosomal RNA, partial sequence | NR_041611.1 |
| ASV1141 | 427 | 1 | 1 | 427 | 100 | NR_044111.1 Selenomonas bovis strain WG 16S ribosomal RNA, partial sequence | NR_044111.1 |
| ASV1142 | 404 | 1 | 1 | 404 | 100 | NR_118156.1 Oscillibacter ruminantium GH1 16S ribosomal RNA, partial sequence | NR_118156.1 |
| ASV1143 | 403 | 1 | 1 | 403 | 100 | NR_029160.1 Ruminococcus callidus strain ATCC 27760 16S ribosomal RNA, partial sequence | NR_029160.1 |
| ASV1145 | 422 | 1 | 1 | 422 | 100 | NR_112717.1 Geofilum rubicundum JCM 15548 strain JAM-BA0501 16S ribosomal RNA, partial sequence | NR_112717.1 |
| ASV1146 | 403 | 1 | 1 | 403 | 100 | NR_152059.1 Harryflintia acetispora strain V20-281a 16S ribosomal RNA, partial sequence | NR_152059.1 |
| ASV1147 | 418 | 1 | 1 | 418 | 100 | NR_102893.1 Butyrivibrio proteoclasticus strain B316 16S ribosomal RNA, partial sequence | NR_102893.1 |
| ASV1148 | 427 | 3 | 1 | 427 | 100 | NR_041781.1 Streptococcus alactolyticus strain ATCC 43077 16S ribosomal RNA, partial sequence | NR_041781.1 |
| ASV1149 | 402 | 2 | 1 | 402 | 100 | NR_042144.1 Clostridium butyricum strain VPI3266 16S ribosomal RNA, partial sequence | NR_042144.1 |
| ASV1150 | 405 | 1 | 1 | 405 | 100 | NR_044972.1 Sporobacter termitidis strain SYR 16S ribosomal RNA, partial sequence | NR_044972.1 |
| ASV1151 | 403 | 1 | 1 | 403 | 100 | NR_147375.1 Colidextribacter massiliensis strain Marseille-P3083 16S ribosomal RNA, partial sequence | NR_147375.1 |
| ASV1152 | 421 | 2 | 1 | 421 | 100 | NR_113077.1 Paraprevotella clara strain JCM 14859 16S ribosomal RNA, partial sequence | NR_113077.1 |
| ASV1153 | 401 | 1 | 1 | 401 | 100 | NR_144740.1 Romboutsia timonensis strain DR1 16S ribosomal RNA, partial sequence | NR_144740.1 |
| ASV1154 | 404 | 2 | 1 | 404 | 100 | NR_027557.1 Roseburia intestinalis L1-82 16S ribosomal RNA, partial sequence | NR_027557.1 |
| ASV1155 | 427 | 1 | 1 | 427 | 100 | NR_042942.1 Treponema porcinum strain 14V28 16S ribosomal RNA, partial sequence | NR_042942.1 |
| ASV1156 | 422 | 2 | 1 | 422 | 100 | NR_074577.1 Paludibacter propionicigenes WB4 16S ribosomal RNA, partial sequence | NR_074577.1 |
| ASV1157 | 422 | 2 | 1 | 422 | 100 | NR_113119.1 Prevotella oris strain JCM 8540 16S ribosomal RNA, partial sequence | NR_113119.1 |
| ASV1158 | 402 | 1 | 1 | 402 | 100 | NR_044049.1 Coprococcus eutactus strain ATCC 27759 16S ribosomal RNA, partial sequence | NR_044049.1 |
| ASV1159 | 402 | 1 | 1 | 402 | 100 | NR_118675.1 [Eubacterium] siraeum strain ATCC 29066 16S ribosomal RNA, partial sequence | NR_118675.1 |
| ASV1160 | 422 | 2 | 1 | 422 | 100 | NR_113119.1 Prevotella oris strain JCM 8540 16S ribosomal RNA, partial sequence | NR_113119.1 |
| ASV1163 | 401 | 1 | 1 | 401 | 100 | NR_144749.1 Ihubacter massiliensis strain Marseille-P2843 16S ribosomal RNA, partial sequence | NR_144749.1 |
| ASV1164 | 401 | 1 | 1 | 401 | 100 | NR_144740.1 Romboutsia timonensis strain DR1 16S ribosomal RNA, partial sequence | NR_144740.1 |
| ASV1165 | 421 | 1 | 1 | 421 | 100 | NR_144616.1 Muribaculum intestinale strain YL27 16S ribosomal RNA, partial sequence | NR_144616.1 |
| ASV1166 | 401 | 1 | 1 | 401 | 100 | NR_159296.1 Aminipila butyrica strain FH042 16S ribosomal RNA, partial sequence | NR_159296.1 |
| ASV1167 | 427 | 1 | 1 | 427 | 100 | NR_044111.1 Selenomonas bovis strain WG 16S ribosomal RNA, partial sequence | NR_044111.1 |
| ASV1168 | 404 | 5 | 1 | 404 | 100 | NR_115693.1 Gracilibacter thermotolerans JW/YJL-S1 16S ribosomal RNA, partial sequence | NR_115693.1 |
| ASV1169 | 402 | 1 | 4 | 402 | 99.25 | NR_104907.1 Eubacterium coprostanoligenes strain HL 16S ribosomal RNA, partial sequence | NR_104907.1 |
| ASV1171 | 405 | 1 | 1 | 405 | 100 | NR_118554.1 Intestinimonas butyriciproducens strain SRB-521-5-I 16S ribosomal RNA, partial sequence | NR_118554.1 |
| ASV1172 | 405 | 1 | 1 | 405 | 100 | NR_044972.1 Sporobacter termitidis strain SYR 16S ribosomal RNA, partial sequence | NR_044972.1 |
| ASV1173 | 422 | 2 | 1 | 422 | 100 | NR_113117.1 Prevotella oralis ATCC 33269 strain JCM 12251 16S ribosomal RNA, partial sequence | NR_113117.1 |
| ASV1174 | 402 | 1 | 1 | 402 | 100 | NR_042953.1 Anaerosporobacter mobilis strain IMSNU 40011 16S ribosomal RNA, partial sequence | NR_042953.1 |
| ASV1175 | 421 | 1 | 1 | 421 | 100 | NR_118334.1 Alloprevotella rava strain 81/4-12 16S ribosomal RNA, partial sequence | NR_118334.1 |
| ASV1176 | 421 | 1 | 1 | 421 | 100 | NR_144616.1 Muribaculum intestinale strain YL27 16S ribosomal RNA, partial sequence | NR_144616.1 |
| ASV1177 | 405 | 1 | 1 | 405 | 100 | NR_042834.1 Alkalibacter saccharofermentans strain Z-79820 16S ribosomal RNA, partial sequence | NR_042834.1 |
| ASV1178 | 422 | 2 | 1 | 422 | 100 | NR_074376.1 Parabacteroides distasonis ATCC 8503 16S ribosomal RNA, partial sequence | NR_074376.1 |
| ASV1179 | 403 | 1 | 3 | 403 | 99.5 | NR_125464.1 Anaerobacterium chartisolvens strain T-1-35 16S ribosomal RNA, partial sequence | NR_125464.1 |
| ASV1180 | 405 | 1 | 1 | 405 | 100 | NR_118554.1 Intestinimonas butyriciproducens strain SRB-521-5-I 16S ribosomal RNA, partial sequence | NR_118554.1 |
| ASV1181 | 427 | 1 | 1 | 427 | 100 | NR_028840.1 Mitsuokella jalaludinii strain M9 16S ribosomal RNA, partial sequence | NR_028840.1 |
| ASV1182 | 405 | 1 | 1 | 405 | 100 | NR_144743.1 Christensenella timonensis strain Marseille-P2437 16S ribosomal RNA, partial sequence | NR_144743.1 |
| ASV1183 | 402 | 1 | 1 | 402 | 100 | NR_025931.1 Ruminococcus flavefaciens strain C94 16S ribosomal RNA, partial sequence | NR_025931.1 |
| ASV1184 | 404 | 2 | 1 | 404 | 100 | NR_114306.1 Aerosakkonema funiforme strain Lao26 16S ribosomal RNA, partial sequence | NR_114306.1 |
| ASV1185 | 402 | 2 | 1 | 402 | 100 | NR_125571.1 Oribacterium asaccharolyticum ACB7 16S ribosomal RNA, partial sequence | NR_125571.1 |
| ASV1186 | 402 | 4 | 1 | 402 | 100 | NR_074399.1 Ruminococcus albus 7 = DSM 20455 16S ribosomal RNA, partial sequence | NR_074399.1 |
| ASV1187 | 404 | 1 | 4 | 404 | 99.26 | NR_104907.1 Eubacterium coprostanoligenes strain HL 16S ribosomal RNA, partial sequence | NR_104907.1 |
| ASV1188 | 404 | 1 | 1 | 404 | 100 | NR_112900.1 Christensenella minuta strain YIT 12065 16S ribosomal RNA, partial sequence | NR_112900.1 |
| ASV1189 | 404 | 5 | 1 | 404 | 100 | NR_115693.1 Gracilibacter thermotolerans JW/YJL-S1 16S ribosomal RNA, partial sequence | NR_115693.1 |
| ASV1190 | 422 | 3 | 1 | 422 | 100 | NR_113119.1 Prevotella oris strain JCM 8540 16S ribosomal RNA, partial sequence | NR_113119.1 |
| ASV1191 | 404 | 1 | 1 | 404 | 100 | NR_042074.1 Eubacterium pyruvativorans strain I-6 16S ribosomal RNA, partial sequence | NR_042074.1 |
| ASV1192 | 427 | 2 | 1 | 427 | 100 | NR_028725.2 Lactobacillus salivarius strain HO 66 16S ribosomal RNA, partial sequence | NR_028725.2 |
| ASV1193 | 427 | 4 | 1 | 427 | 100 | NR_041781.1 Streptococcus alactolyticus strain ATCC 43077 16S ribosomal RNA, partial sequence | NR_041781.1 |
| ASV1194 | 402 | 1 | 1 | 400 | 99.5 | NR_104911.1 Vampirovibrio chlorellavorus strain ICPB 3707 16S ribosomal RNA, partial sequence | NR_104911.1 |
| ASV1195 | 422 | 1 | 1 | 422 | 100 | NR_041364.1 Prevotella stercorea DSM 18206 strain CB35 16S ribosomal RNA, partial sequence | NR_041364.1 |
| ASV1196 | 422 | 1 | 1 | 422 | 100 | NR_041364.1 Prevotella stercorea DSM 18206 strain CB35 16S ribosomal RNA, partial sequence | NR_041364.1 |
| ASV1197 | 402 | 1 | 1 | 402 | 100 | NR_028961.1 Faecalibacterium prausnitzii strain ATCC 27768 16S ribosomal RNA, partial sequence | NR_028961.1 |
| ASV1198 | 422 | 2 | 1 | 422 | 100 | NR_113117.1 Prevotella oralis ATCC 33269 strain JCM 12251 16S ribosomal RNA, partial sequence | NR_113117.1 |
| ASV1199 | 421 | 4 | 1 | 421 | 100 | NR_117779.1 Anaerorhabdus furcosa 16S ribosomal RNA, partial sequence | NR_117779.1 |
| ASV1200 | 427 | 1 | 1 | 427 | 100 | NR_042871.1 [Actinobacillus] rossii strain ATCC 27072 16S ribosomal RNA, partial sequence | NR_042871.1 |
| ASV1201 | 404 | 1 | 1 | 404 | 100 | NR_118156.1 Oscillibacter ruminantium GH1 16S ribosomal RNA, partial sequence | NR_118156.1 |
| ASV1202 | 422 | 1 | 1 | 422 | 100 | NR_043216.1 Prevotella loescheii strain NCTC 11321 16S ribosomal RNA, partial sequence | NR_043216.1 |
| ASV1203 | 405 | 1 | 1 | 405 | 100 | NR_144743.1 Christensenella timonensis strain Marseille-P2437 16S ribosomal RNA, partial sequence | NR_144743.1 |
| ASV1204 | 405 | 1 | 1 | 405 | 100 | NR_044972.1 Sporobacter termitidis strain SYR 16S ribosomal RNA, partial sequence | NR_044972.1 |
| ASV1205 | 405 | 1 | 3 | 405 | 99.51 | NR_112900.1 Christensenella minuta strain YIT 12065 16S ribosomal RNA, partial sequence | NR_112900.1 |
| ASV1206 | 427 | 1 | 1 | 426 | 99.77 | NR_044111.1 Selenomonas bovis strain WG 16S ribosomal RNA, partial sequence | NR_044111.1 |
| ASV1207 | 422 | 2 | 1 | 422 | 100 | NR_113117.1 Prevotella oralis ATCC 33269 strain JCM 12251 16S ribosomal RNA, partial sequence | NR_113117.1 |
| ASV1208 | 422 | 1 | 1 | 422 | 100 | NR_024816.1 Prevotella salivae strain EPSA11 16S ribosomal RNA, partial sequence | NR_024816.1 |
| ASV1209 | 402 | 1 | 1 | 402 | 100 | NR_134772.1 Murimonas intestini strain SRB-530-5-H 16S ribosomal RNA, partial sequence | NR_134772.1 |
| ASV1210 | 405 | 1 | 4 | 405 | 99.26 | NR_144742.1 Christensenella massiliensis strain Marseille-P2438 16S ribosomal RNA, partial sequence | NR_144742.1 |
| ASV1212 | 422 | 2 | 1 | 422 | 100 | NR_074577.1 Paludibacter propionicigenes WB4 16S ribosomal RNA, partial sequence | NR_074577.1 |
| ASV1213 | 422 | 2 | 1 | 422 | 100 | NR_113105.1 Prevotella histicola strain JCM 15637 16S ribosomal RNA, partial sequence | NR_113105.1 |
| ASV1214 | 422 | 1 | 1 | 422 | 100 | NR_151886.1 Prevotella colorans strain A1336 16S ribosomal RNA, partial sequence | NR_151886.1 |
| ASV1215 | 422 | 1 | 1 | 422 | 100 | NR_113411.1 Prevotella copri DSM 18205 strain JCM 13464 16S ribosomal RNA, partial sequence | NR_113411.1 |
| ASV1216 | 402 | 1 | 1 | 402 | 100 | NR_102884.1 Ruminococcus champanellensis 18P13 = JCM 17042 16S ribosomal RNA, partial sequence | NR_102884.1 |
| ASV1217 | 422 | 1 | 1 | 422 | 100 | NR_040877.1 Prevotella copri DSM 18205 strain CB7 16S ribosomal RNA, partial sequence | NR_040877.1 |
| ASV1218 | 404 | 1 | 1 | 404 | 100 | NR_028911.1 Anaerovorax odorimutans strain NorPut 16S ribosomal RNA, partial sequence | NR_028911.1 |
| ASV1220 | 427 | 1 | 1 | 427 | 100 | NR_044111.1 Selenomonas bovis strain WG 16S ribosomal RNA, partial sequence | NR_044111.1 |
| ASV1221 | 418 | 1 | 1 | 418 | 100 | NR_102893.1 Butyrivibrio proteoclasticus strain B316 16S ribosomal RNA, partial sequence | NR_102893.1 |
| ASV1222 | 427 | 1 | 1 | 427 | 100 | NR_044111.1 Selenomonas bovis strain WG 16S ribosomal RNA, partial sequence | NR_044111.1 |
| ASV1223 | 402 | 2 | 1 | 402 | 100 | NR_113231.1 Blautia glucerasea strain JCM 17039 16S ribosomal RNA, partial sequence | NR_113231.1 |
| ASV1224 | 405 | 1 | 1 | 405 | 100 | NR_044972.1 Sporobacter termitidis strain SYR 16S ribosomal RNA, partial sequence | NR_044972.1 |
| ASV1225 | 422 | 1 | 1 | 422 | 100 | NR_113411.1 Prevotella copri DSM 18205 strain JCM 13464 16S ribosomal RNA, partial sequence | NR_113411.1 |
| ASV1226 | 427 | 2 | 1 | 427 | 100 | NR_037018.1 Acidaminococcus fermentans strain VR4 16S ribosomal RNA gene, partial sequence | NR_037018.1 |
| ASV1227 | 422 | 5 | 1 | 422 | 100 | NR_113119.1 Prevotella oris strain JCM 8540 16S ribosomal RNA, partial sequence | NR_113119.1 |
| ASV1228 | 402 | 1 | 1 | 402 | 100 | NR_029160.1 Ruminococcus callidus strain ATCC 27760 16S ribosomal RNA, partial sequence | NR_029160.1 |
| ASV1229 | 427 | 1 | 1 | 427 | 100 | NR_042942.1 Treponema porcinum strain 14V28 16S ribosomal RNA, partial sequence | NR_042942.1 |
| ASV1230 | 402 | 1 | 1 | 402 | 100 | NR_028961.1 Faecalibacterium prausnitzii strain ATCC 27768 16S ribosomal RNA, partial sequence | NR_028961.1 |
| ASV1231 | 404 | 1 | 1 | 404 | 100 | NR_118156.1 Oscillibacter ruminantium GH1 16S ribosomal RNA, partial sequence | NR_118156.1 |
| ASV1233 | 405 | 2 | 1 | 405 | 100 | NR_144742.1 Christensenella massiliensis strain Marseille-P2438 16S ribosomal RNA, partial sequence | NR_144742.1 |
| ASV1234 | 405 | 1 | 1 | 405 | 100 | NR_044972.1 Sporobacter termitidis strain SYR 16S ribosomal RNA, partial sequence | NR_044972.1 |
| ASV1235 | 404 | 1 | 1 | 404 | 100 | NR_026476.1 Succinivibrio dextrinosolvens strain 0554 16S ribosomal RNA, partial sequence | NR_026476.1 |
| ASV1236 | 402 | 1 | 1 | 402 | 100 | NR_029239.3 Clostridium chartatabidum strain 163 16S ribosomal RNA, partial sequence | NR_029239.3 |
| ASV1237 | 427 | 1 | 1 | 427 | 100 | NR_044111.1 Selenomonas bovis strain WG 16S ribosomal RNA, partial sequence | NR_044111.1 |
| ASV1238 | 426 | 1 | 1 | 426 | 100 | NR_026205.1 Succiniclasticum ruminis strain SE10 16S ribosomal RNA, partial sequence | NR_026205.1 |
| ASV1239 | 402 | 1 | 1 | 402 | 100 | NR_102884.1 Ruminococcus champanellensis 18P13 = JCM 17042 16S ribosomal RNA, partial sequence | NR_102884.1 |
| ASV1240 | 401 | 1 | 1 | 401 | 100 | NR_158111.1 Paludicola psychrotolerans strain NC1253 16S ribosomal RNA, partial sequence | NR_158111.1 |
| ASV1241 | 420 | 1 | 1 | 420 | 100 | NR_119166.1 Parabacteroides merdae ATCC 43184 16S ribosomal RNA, partial sequence | NR_119166.1 |
| ASV1242 | 403 | 1 | 1 | 403 | 100 | NR_104907.1 Eubacterium coprostanoligenes strain HL 16S ribosomal RNA, partial sequence | NR_104907.1 |
| ASV1243 | 404 | 1 | 1 | 404 | 100 | NR_026476.1 Succinivibrio dextrinosolvens strain 0554 16S ribosomal RNA, partial sequence | NR_026476.1 |
| ASV1244 | 405 | 2 | 1 | 405 | 100 | NR_027557.1 Roseburia intestinalis L1-82 16S ribosomal RNA, partial sequence | NR_027557.1 |
| ASV1245 | 402 | 1 | 1 | 402 | 100 | NR_170495.1 Blautia faecicola strain KGMB01111 16S ribosomal RNA, complete sequence | NR_170495.1 |
| ASV1246 | 403 | 1 | 3 | 403 | 99.5 | NR_125677.1 Vallitalea pronyensis strain FatNI3 16S ribosomal RNA, partial sequence | NR_125677.1 |
| ASV1247 | 401 | 1 | 1 | 401 | 100 | NR_137408.1 Terrisporobacter petrolearius strain LAM0A37 16S ribosomal RNA, partial sequence | NR_137408.1 |
| ASV1248 | 404 | 1 | 1 | 404 | 100 | NR_152059.1 Harryflintia acetispora strain V20-281a 16S ribosomal RNA, partial sequence | NR_152059.1 |
| ASV1249 | 422 | 2 | 1 | 422 | 100 | NR_074376.1 Parabacteroides distasonis ATCC 8503 16S ribosomal RNA, partial sequence | NR_074376.1 |
| ASV1250 | 405 | 1 | 1 | 405 | 100 | NR_044972.1 Sporobacter termitidis strain SYR 16S ribosomal RNA, partial sequence | NR_044972.1 |
| ASV1251 | 405 | 2 | 1 | 405 | 100 | NR_115340.1 Saccharofermentans acetigenes strain P6 16S ribosomal RNA, partial sequence | NR_115340.1 |
| ASV1252 | 402 | 1 | 1 | 402 | 100 | NR_029097.1 Oribacterium sinus strain AIP 354.02 16S ribosomal RNA, partial sequence | NR_029097.1 |
| ASV1253 | 402 | 1 | 1 | 402 | 100 | NR_026074.1 Helicobacter rodentium strain MIT 95-1707 16S ribosomal RNA, partial sequence | NR_026074.1 |
| ASV1254 | 404 | 3 | 1 | 404 | 100 | NR_074793.2 Oscillibacter valericigenes strain Sjm18-20 16S ribosomal RNA, complete sequence | NR_074793.2 |
| ASV1255 | 402 | 2 | 1 | 402 | 100 | NR_144696.1 Clostridium saudiense strain JCC 16S ribosomal RNA, partial sequence | NR_144696.1 |
| ASV1256 | 403 | 1 | 1 | 403 | 100 | NR_118554.1 Intestinimonas butyriciproducens strain SRB-521-5-I 16S ribosomal RNA, partial sequence | NR_118554.1 |
| ASV1257 | 426 | 1 | 1 | 426 | 100 | NR_044111.1 Selenomonas bovis strain WG 16S ribosomal RNA, partial sequence | NR_044111.1 |
| ASV1258 | 404 | 1 | 1 | 404 | 100 | NR_102884.1 Ruminococcus champanellensis 18P13 = JCM 17042 16S ribosomal RNA, partial sequence | NR_102884.1 |
| ASV1259 | 403 | 1 | 1 | 403 | 100 | NR_042832.1 Roseburia faecis strain M72/1 16S ribosomal RNA, partial sequence | NR_042832.1 |
| ASV1260 | 422 | 1 | 1 | 422 | 100 | NR_027196.1 Bacteroides stercoris ATCC 43183 16S ribosomal RNA, partial sequence | NR_027196.1 |
| ASV1261 | 420 | 1 | 1 | 420 | 100 | NR_170509.1 Duncaniella freteri strain TLL-A3 16S ribosomal RNA, partial sequence | NR_170509.1 |
| ASV1262 | 427 | 1 | 1 | 427 | 100 | NR_102980.1 Megasphaera elsdenii DSM 20460 16S ribosomal RNA, partial sequence | NR_102980.1 |
| ASV1263 | 403 | 1 | 1 | 403 | 100 | NR_118554.1 Intestinimonas butyriciproducens strain SRB-521-5-I 16S ribosomal RNA, partial sequence | NR_118554.1 |
| ASV1264 | 405 | 1 | 1 | 405 | 100 | NR_144611.1 Flintibacter butyricus strain BLS21 16S ribosomal RNA, partial sequence | NR_144611.1 |
| ASV1265 | 427 | 1 | 1 | 427 | 100 | NR_028840.1 Mitsuokella jalaludinii strain M9 16S ribosomal RNA, partial sequence | NR_028840.1 |
| ASV1266 | 402 | 4 | 1 | 402 | 100 | NR_074399.1 Ruminococcus albus 7 = DSM 20455 16S ribosomal RNA, partial sequence | NR_074399.1 |
| ASV1267 | 422 | 2 | 1 | 422 | 100 | NR_113119.1 Prevotella oris strain JCM 8540 16S ribosomal RNA, partial sequence | NR_113119.1 |
| ASV1268 | 427 | 1 | 1 | 427 | 100 | NR_041778.1 Desulfovibrio piger strain ATCC 29098 16S ribosomal RNA, partial sequence | NR_041778.1 |
| ASV1269 | 402 | 1 | 2 | 402 | 99.75 | NR_027579.1 Ruminococcus lactaris ATCC 29176 16S ribosomal RNA, partial sequence | NR_027579.1 |
| ASV1270 | 403 | 1 | 3 | 403 | 99.5 | NR_125677.1 Vallitalea pronyensis strain FatNI3 16S ribosomal RNA, partial sequence | NR_125677.1 |
| ASV1271 | 403 | 1 | 3 | 403 | 99.5 | NR_125677.1 Vallitalea pronyensis strain FatNI3 16S ribosomal RNA, partial sequence | NR_125677.1 |
| ASV1272 | 406 | 1 | 1 | 406 | 100 | NR_115340.1 Saccharofermentans acetigenes strain P6 16S ribosomal RNA, partial sequence | NR_115340.1 |
| ASV1273 | 403 | 3 | 1 | 403 | 100 | NR_118521.1 Campylobacter lanienae NCTC 13004 strain CCUG 44467 16S ribosomal RNA, partial sequence | NR_118521.1 |
| ASV1274 | 403 | 1 | 1 | 403 | 100 | NR_104907.1 Eubacterium coprostanoligenes strain HL 16S ribosomal RNA, partial sequence | NR_104907.1 |
| ASV1275 | 427 | 1 | 1 | 427 | 100 | NR_026030.1 Actinobacillus porcinus strain NM319 16S ribosomal RNA, partial sequence | NR_026030.1 |
| ASV1276 | 402 | 1 | 1 | 402 | 100 | NR_028961.1 Faecalibacterium prausnitzii strain ATCC 27768 16S ribosomal RNA, partial sequence | NR_028961.1 |
| ASV1277 | 427 | 1 | 1 | 427 | 100 | NR_044111.1 Selenomonas bovis strain WG 16S ribosomal RNA, partial sequence | NR_044111.1 |
| ASV1278 | 402 | 1 | 3 | 402 | 99.5 | NR_125677.1 Vallitalea pronyensis strain FatNI3 16S ribosomal RNA, partial sequence | NR_125677.1 |
| ASV1280 | 405 | 1 | 1 | 405 | 100 | NR_104907.1 Eubacterium coprostanoligenes strain HL 16S ribosomal RNA, partial sequence | NR_104907.1 |
| ASV1281 | 402 | 1 | 1 | 402 | 100 | NR_026106.1 [Eubacterium] cellulosolvens strain 6 16S ribosomal RNA, partial sequence | NR_026106.1 |
| ASV1282 | 402 | 1 | 1 | 402 | 100 | NR_042832.1 Roseburia faecis strain M72/1 16S ribosomal RNA, partial sequence | NR_042832.1 |
| ASV1283 | 405 | 1 | 4 | 405 | 99.26 | NR_115269.1 Catabacter hongkongensis strain HKU16 16S ribosomal RNA, partial sequence | NR_115269.1 |
| ASV1284 | 428 | 1 | 1 | 428 | 100 | NR_153710.1 Peptococcus simiae strain M108 16S ribosomal RNA, partial sequence | NR_153710.1 |
| ASV1285 | 422 | 2 | 1 | 422 | 100 | NR_113076.1 Parabacteroides goldsteinii DSM 19448 = WAL 12034 strain JCM 13446 16S ribosomal RNA, partial sequence | NR_113076.1 |
| ASV1286 | 427 | 1 | 1 | 427 | 100 | NR_044111.1 Selenomonas bovis strain WG 16S ribosomal RNA, partial sequence | NR_044111.1 |
| ASV1287 | 405 | 3 | 1 | 405 | 100 | NR_115307.1 Ethanoligenens harbinense YUAN-3 16S ribosomal RNA, partial sequence | NR_115307.1 |
| ASV1288 | 427 | 1 | 1 | 427 | 100 | NR_028840.1 Mitsuokella jalaludinii strain M9 16S ribosomal RNA, partial sequence | NR_028840.1 |
| ASV1289 | 421 | 2 | 1 | 421 | 100 | NR_117779.1 Anaerorhabdus furcosa 16S ribosomal RNA, partial sequence | NR_117779.1 |
| ASV1290 | 403 | 5 | 1 | 403 | 100 | NR_118521.1 Campylobacter lanienae NCTC 13004 strain CCUG 44467 16S ribosomal RNA, partial sequence | NR_118521.1 |
| ASV1291 | 420 | 1 | 1 | 420 | 100 | NR_144616.1 Muribaculum intestinale strain YL27 16S ribosomal RNA, partial sequence | NR_144616.1 |
| ASV1292 | 426 | 1 | 1 | 426 | 100 | NR_170394.1 Erysipelothrix piscisicarius strain 15TAL0474 16S ribosomal RNA, complete sequence | NR_170394.1 |
| ASV1293 | 405 | 1 | 1 | 405 | 100 | NR_144742.1 Christensenella massiliensis strain Marseille-P2438 16S ribosomal RNA, partial sequence | NR_144742.1 |
| ASV1294 | 402 | 1 | 1 | 402 | 100 | NR_026204.1 [Clostridium] viride strain T2-7 16S ribosomal RNA, partial sequence | NR_026204.1 |
| ASV1295 | 405 | 1 | 1 | 405 | 100 | NR_144611.1 Flintibacter butyricus strain BLS21 16S ribosomal RNA, partial sequence | NR_144611.1 |
| ASV1296 | 421 | 4 | 1 | 421 | 100 | NR_117779.1 Anaerorhabdus furcosa 16S ribosomal RNA, partial sequence | NR_117779.1 |
| ASV1297 | 403 | 2 | 1 | 403 | 100 | NR_027557.1 Roseburia intestinalis L1-82 16S ribosomal RNA, partial sequence | NR_027557.1 |
| ASV1298 | 422 | 1 | 1 | 422 | 100 | NR_144750.1 Prevotellamassilia timonensis strain Marseille-P2831 16S ribosomal RNA, partial sequence | NR_144750.1 |
| ASV1299 | 402 | 1 | 1 | 402 | 100 | NR_147375.1 Colidextribacter massiliensis strain Marseille-P3083 16S ribosomal RNA, partial sequence | NR_147375.1 |
| ASV1300 | 402 | 1 | 1 | 402 | 100 | NR_042200.1 [Clostridium] asparagiforme strain N6 16S ribosomal RNA, partial sequence | NR_042200.1 |
| ASV1301 | 402 | 1 | 1 | 402 | 100 | NR_118676.1 Eubacterium xylanophilum strain ATCC 35991 16S ribosomal RNA, partial sequence | NR_118676.1 |
| ASV1302 | 405 | 1 | 1 | 405 | 100 | NR_144742.1 Christensenella massiliensis strain Marseille-P2438 16S ribosomal RNA, partial sequence | NR_144742.1 |
| ASV1304 | 402 | 2 | 1 | 402 | 100 | NR_125571.1 Oribacterium asaccharolyticum ACB7 16S ribosomal RNA, partial sequence | NR_125571.1 |
| ASV1305 | 422 | 1 | 1 | 422 | 100 | NR_144616.1 Muribaculum intestinale strain YL27 16S ribosomal RNA, partial sequence | NR_144616.1 |

| | | | | | | | | | | | | | | | | |
|---|---|---|---|---|---|---|---|---|---|---|---|---|---|---|---|---|
| ASV1306 | 427 | 1 | 1 | 427 | 100 | NR_026413.1 Desulfovibrio intestinalis strain KMS2 16S ribosomal RNA, partial sequence | NR_026413.1 | 1509 | 337 | 763 | 28.3 | 612.4 | 6E-175 | 397/429 | 92 | 4/429 |
| ASV1307 | 422 | 1 | 1 | 422 | 100 | NR_144616.1 Muribaculum intestinale strain YL27 16S ribosomal RNA, partial sequence | NR_144616.1 | 1460 | 297 | 718 | 28.9 | 603.1 | 3E-172 | 390/422 | 92 | 0/422 |
| ASV1308 | 422 | 2 | 1 | 422 | 100 | NR_113065.1 Bacteroides clarus YIT 12056 strain JCM 16067 16S ribosomal RNA, partial sequence | NR_113065.1 | 1488 | 355 | 776 | 28.36 | 597.6 | 2E-170 | 391/424 | 92 | 4/424 |
| ASV1309 | 404 | 2 | 1 | 404 | 100 | NR_159296.1 Aminipila butyrica strain FH042 16S ribosomal RNA, partial sequence | NR_159296.1 | 1460 | 352 | 755 | 27.67 | 508.9 | 7E-144 | 361/404 | 89 | 0/404 |
| ASV1310 | 422 | 2 | 1 | 422 | 100 | NR_113076.1 Parabacteroides goldsteinii DSM 19448 = WAL 12034 strain JCM 13446 16S ribosomal RNA, partial sequence | NR_113076.1 | 1476 | 341 | 762 | 28.59 | 531.1 | 2E-150 | 377/422 | 89 | 0/422 |
| ASV1311 | 402 | 1 | 1 | 402 | 100 | NR_074809.1 Roseburia hominis A2-183 16S ribosomal RNA, partial sequence | NR_074809.1 | 1535 | 367 | 769 | 26.25 | 610.5 | 2E-174 | 379/403 | 94 | 1/403 |
| ASV1312 | 423 | 1 | 1 | 423 | 100 | NR_102887.1 Prevotella ruminicola strain Bryant 23 16S ribosomal RNA, partial sequence | NR_102887.1 | 1519 | 354 | 775 | 27.78 | 558.8 | 8E-159 | 385/425 | 90 | 5/425 |
| ASV1313 | 405 | 1 | 1 | 405 | 100 | NR_159227.1 Monoglobus pectinilyticus strain 14 16S ribosomal RNA, partial sequence | NR_159227.1 | 1493 | 368 | 769 | 26.93 | 512.6 | 6E-145 | 365/407 | 89 | 7/407 |
| ASV1314 | 402 | 4 | 1 | 402 | 100 | NR_074399.1 Ruminococcus albus 7 = DSM 20455 16S ribosomal RNA, partial sequence | NR_074399.1 | 1500 | 350 | 751 | 26.8 | 605 | 9E-173 | 377/402 | 93 | 0/402 |
| ASV1315 | 403 | 1 | 1 | 403 | 100 | NR_104907.1 Eubacterium coprostanoligenes strain HL 16S ribosomal RNA, partial sequence | NR_104907.1 | 1446 | 332 | 734 | 27.87 | 651.1 | 0 | 386/403 | 95 | 0/403 |
| ASV1316 | 408 | 2 | 1 | 408 | 100 | NR_044858.1 Trueperella pyogenes strain NCTC 5224 16S ribosomal RNA, partial sequence | NR_044858.1 | 1323 | 320 | 728 | 30.91 | 710.2 | 0 | 401/409 | 98 | 1/409 |
| ASV1317 | 422 | 1 | 1 | 422 | 100 | NR_146018.1 Paludibacter jiangxiensis strain NM7 16S ribosomal RNA, partial sequence | NR_146018.1 | 1469 | 340 | 761 | 28.73 | 497.9 | 2E-140 | 374/425 | 88 | 6/425 |
| ASV1318 | 421 | 1 | 1 | 421 | 100 | NR_113097.1 Prevotella buccae ATCC 33574 strain JCM 12245 16S ribosomal RNA, partial sequence | NR_113097.1 | 1480 | 343 | 764 | 28.51 | 451.7 | 1E-126 | 366/425 | 86 | 7/425 |
| ASV1319 | 422 | 1 | 1 | 422 | 100 | NR_113153.1 Alistipes shahii strain JCM 16773 16S ribosomal RNA, partial sequence | NR_113153.1 | 1486 | 351 | 772 | 28.4 | 774.9 | 0 | 421/422 | 99 | 0/422 |
| ASV1320 | 422 | 1 | 1 | 422 | 100 | NR_113153.1 Alistipes shahii strain JCM 16773 16S ribosomal RNA, partial sequence | NR_113153.1 | 1486 | 351 | 772 | 28.4 | 780.4 | 0 | 422/422 | 100 | 0/422 |
| ASV1321 | 423 | 1 | 1 | 423 | 100 | NR_144750.1 Prevotellamassilia timonensis strain Marseille-P2831 16S ribosomal RNA, partial sequence | NR_144750.1 | 1493 | 358 | 779 | 28.27 | 553.3 | 4E-157 | 384/425 | 90 | 5/425 |
| ASV1322 | 402 | 1 | 1 | 402 | 100 | NR_044048.1 Coprococcus comes ATCC 27758 16S ribosomal RNA, partial sequence | NR_044048.1 | 1453 | 345 | 746 | 27.67 | 721.3 | 0 | 398/402 | 99 | 0/402 |
| ASV1323 | 405 | 2 | 1 | 405 | 100 | NR_044972.1 Sporobacter termitidis strain SYR 16S ribosomal RNA, partial sequence | NR_044972.1 | 1506 | 348 | 752 | 26.89 | 575.4 | 7E-164 | 374/406 | 92 | 2/406 |
| ASV1324 | 422 | 1 | 1 | 422 | 100 | NR_043216.1 Prevotella loescheii strain NCTC 11321 16S ribosomal RNA, partial sequence | NR_043216.1 | 1451 | 336 | 757 | 29.08 | 599.4 | 4E-171 | 392/425 | 92 | 6/425 |
| ASV1325 | 404 | 1 | 1 | 404 | 100 | NR_044503.1 Thermotalea metallivorans strain B2-1 16S ribosomal RNA, partial sequence | NR_044503.1 | 1627 | 467 | 868 | 24.71 | 479.4 | 6E-135 | 362/410 | 88 | 14/410 |
| ASV1326 | 422 | 2 | 1 | 422 | 100 | NR_113117.1 Prevotella oralis ATCC 33269 strain JCM 12251 16S ribosomal RNA, partial sequence | NR_113117.1 | 1496 | 361 | 782 | 28.21 | 625.3 | 7E-179 | 395/423 | 93 | 2/423 |
| ASV1327 | 427 | 1 | 1 | 427 | 100 | NR_026413.1 Desulfovibrio intestinalis strain KMS2 16S ribosomal RNA, partial sequence | NR_026413.1 | 1509 | 337 | 763 | 28.3 | 612.4 | 6E-175 | 397/429 | 92 | 4/429 |
| ASV1328 | 427 | 1 | 1 | 427 | 100 | NR_108477.1 Streptococcus porcorum strain 682/03 16S ribosomal RNA, partial sequence | NR_108477.1 | 1380 | 268 | 694 | 30.94 | 773 | 0 | 424/427 | 99 | 0/427 |
| ASV1329 | 427 | 1 | 1 | 427 | 100 | NR_044111.1 Selenomonas bovis strain WG 16S ribosomal RNA, partial sequence | NR_044111.1 | 1525 | 370 | 796 | 28 | 634.5 | 0 | 401/429 | 93 | 4/429 |
| ASV1330 | 403 | 5 | 1 | 403 | 100 | NR_118521.1 Campylobacter lanienae NCTC 13004 strain CCUG 44467 16S ribosomal RNA, partial sequence | NR_118521.1 | 1434 | 328 | 730 | 28.1 | 695.5 | 0 | 394/403 | 97 | 0/403 |
| ASV1331 | 405 | 1 | 1 | 405 | 100 | NR_118554.1 Intestinimonas butyriciproducens strain SRB-521-5-I 16S ribosomal RNA, partial sequence | NR_118554.1 | 1475 | 346 | 750 | 27.46 | 483.1 | 4E-136 | 359/407 | 88 | 4/407 |
| ASV1332 | 422 | 2 | 1 | 422 | 100 | NR_113119.1 Prevotella oris strain JCM 8540 16S ribosomal RNA, partial sequence | NR_113119.1 | 1481 | 345 | 766 | 28.49 | 603.1 | 3E-172 | 392/424 | 92 | 4/424 |
| ASV1333 | 405 | 1 | 1 | 405 | 100 | NR_144742.1 Christensenella massiliensis strain Marseille-P2438 16S ribosomal RNA, partial sequence | NR_144742.1 | 1499 | 369 | 773 | 27.02 | 433.2 | 5E-121 | 354/411 | 86 | 12/411 |
| ASV1334 | 404 | 1 | 1 | 404 | 100 | NR_118156.1 Oscillibacter ruminantium GH1 16S ribosomal RNA, partial sequence | NR_118156.1 | 1409 | 321 | 724 | 28.67 | 619.7 | 3E-177 | 381/404 | 94 | 0/404 |
| ASV1335 | 422 | 2 | 1 | 422 | 100 | NR_114009.1 Galbibacter mesophilus strain NBRC 101624 16S ribosomal RNA, partial sequence | NR_114009.1 | 1445 | 328 | 749 | 29.2 | 431.4 | 2E-120 | 360/423 | 85 | 2/423 |
| ASV1336 | 402 | 1 | 1 | 402 | 100 | NR_026476.1 Succinivibrio dextrinosolvens strain 0554 16S ribosomal RNA, partial sequence | NR_026476.1 | 1463 | 331 | 732 | 27.48 | 593.9 | 2E-169 | 375/402 | 93 | 0/402 |
| ASV1337 | 422 | 2 | 1 | 422 | 100 | NR_113117.1 Prevotella oralis ATCC 33269 strain JCM 12251 16S ribosomal RNA, partial sequence | NR_113117.1 | 1496 | 361 | 782 | 28.21 | 531.1 | 2E-150 | 379/424 | 89 | 4/424 |
| ASV1338 | 419 | 1 | 1 | 419 | 100 | NR_042150.1 Fibrobacter intestinalis strain NR9 16S ribosomal RNA, partial sequence | NR_042150.1 | 1436 | 325 | 743 | 29.18 | 608.7 | 7E-174 | 390/420 | 92 | 2/420 |
| ASV1339 | 403 | 1 | 3 | 403 | 99.5 | NR_125677.1 Vallitalea pronyensis strain FatNI3 16S ribosomal RNA, partial sequence | NR_125677.1 | 1480 | 365 | 764 | 27.03 | 451.7 | 1E-126 | 351/403 | 87 | 5/403 |
| ASV1340 | 420 | 1 | 1 | 420 | 100 | NR_170509.1 Duncaniella freteri strain TLL-A3 16S ribosomal RNA, partial sequence | NR_170509.1 | 1538 | 364 | 784 | 27.37 | 449.9 | 5E-126 | 366/425 | 86 | 9/425 |
| ASV1341 | 427 | 1 | 1 | 426 | 99.77 | NR_027596.1 Mitsuokella multacida strain DSM 20544 16S ribosomal RNA, partial sequence | NR_027596.1 | 1511 | 346 | 771 | 28.19 | 656.7 | 0 | 402/426 | 94 | 0/426 |
| ASV1342 | 404 | 2 | 1 | 404 | 100 | NR_074793.2 Oscillibacter valericigenes strain Sjm18-20 16S ribosomal RNA, complete sequence | NR_074793.2 | 1530 | 372 | 775 | 26.41 | 647.4 | 0 | 386/404 | 95 | 0/404 |
| ASV1344 | 422 | 2 | 1 | 422 | 100 | NR_113119.1 Prevotella oris strain JCM 8540 16S ribosomal RNA, partial sequence | NR_113119.1 | 1481 | 345 | 766 | 28.49 | 575.4 | 7E-164 | 386/423 | 91 | 2/423 |
| ASV1345 | 427 | 1 | 1 | 427 | 100 | NR_042871.1 [Actinobacillus] rossii strain ATCC 27072 16S ribosomal RNA, partial sequence | NR_042871.1 | 1364 | 333 | 759 | 31.3 | 732.4 | 0 | 416/427 | 97 | 0/427 |
| ASV1346 | 402 | 2 | 1 | 402 | 100 | NR_113231.1 Blautia glucerasea strain JCM 17039 16S ribosomal RNA, partial sequence | NR_113231.1 | 1491 | 364 | 765 | 26.96 | 616.1 | 4E-176 | 379/402 | 94 | 0/402 |
| ASV1347 | 427 | 5 | 1 | 427 | 100 | NR_148863.1 Alkalispirochaeta cellulosivorans strain JC227 16S ribosomal RNA, partial sequence | NR_148863.1 | 1446 | 301 | 727 | 29.53 | 479.4 | 6E-135 | 372/428 | 86 | 2/428 |
| ASV1348 | 427 | 1 | 1 | 427 | 100 | NR_041666.1 Dialister succinatiphilus YIT 11850 16S ribosomal RNA, partial sequence | NR_041666.1 | 1521 | 353 | 779 | 28.07 | 784.1 | 0 | 426/427 | 99 | 0/427 |
| ASV1349 | 427 | 2 | 1 | 427 | 100 | NR_026030.1 Actinobacillus porcinus strain NM319 16S ribosomal RNA, partial sequence | NR_026030.1 | 1466 | 335 | 761 | 29.13 | 778.6 | 0 | 425/427 | 99 | 0/427 |
| ASV1350 | 422 | 2 | 1 | 422 | 100 | NR_113076.1 Parabacteroides goldsteinii DSM 19448 = WAL 12034 strain JCM 13446 16S ribosomal RNA, partial sequence | NR_113076.1 | 1476 | 341 | 762 | 28.59 | 503.4 | 4E-142 | 372/422 | 88 | 0/422 |
| ASV1351 | 402 | 2 | 1 | 402 | 100 | NR_152095.1 Herbinix luporum strain SD1D 16S ribosomal RNA, partial sequence | NR_152095.1 | 1521 | 365 | 766 | 26.43 | 693.6 | 0 | 393/402 | 97 | 0/402 |
| ASV1352 | 421 | 2 | 1 | 421 | 100 | NR_113076.1 Parabacteroides goldsteinii DSM 19448 = WAL 12034 strain JCM 13446 16S ribosomal RNA, partial sequence | NR_113076.1 | 1476 | 341 | 762 | 28.59 | 529.3 | 6E-150 | 378/423 | 89 | 3/423 |
| ASV1353 | 422 | 1 | 1 | 422 | 100 | NR_146018.1 Paludibacter jiangxiensis strain NM7 16S ribosomal RNA, partial sequence | NR_146018.1 | 1469 | 340 | 761 | 28.73 | 503.4 | 4E-142 | 375/425 | 88 | 6/425 |
| ASV1354 | 405 | 1 | 3 | 405 | 99.51 | NR_026312.1 Blautia schinkii strain B 16S ribosomal RNA, partial sequence | NR_026312.1 | 1482 | 346 | 745 | 26.99 | 466.5 | 4E-131 | 354/404 | 87 | 5/404 |
| ASV1355 | 427 | 2 | 1 | 427 | 100 | NR_028797.1 Selenomonas infelix strain VPI D 75 B-30 16S ribosomal RNA, partial sequence | NR_028797.1 | 1524 | 373 | 799 | 28.02 | 606.8 | 3E-173 | 394/427 | 92 | 0/427 |
| ASV1356 | 422 | 1 | 1 | 422 | 100 | NR_113411.1 Prevotella copri DSM 18205 strain JCM 13464 16S ribosomal RNA, partial sequence | NR_113411.1 | 1493 | 358 | 779 | 28.27 | 769.3 | 0 | 420/422 | 99 | 0/422 |
| ASV1357 | 426 | 1 | 1 | 426 | 100 | NR_125628.1 Holdemania massiliensis AP2 16S ribosomal RNA, partial sequence | NR_125628.1 | 1499 | 353 | 778 | 28.42 | 449.9 | 5E-126 | 368/429 | 85 | 6/429 |
| ASV1359 | 422 | 1 | 1 | 422 | 100 | NR_113208.1 Parabacteroides chinchillae strain JCM 17104 16S ribosomal RNA, partial sequence | NR_113208.1 | 1489 | 353 | 774 | 28.34 | 647.4 | 0 | 399/423 | 94 | 2/423 |
| ASV1360 | 405 | 1 | 1 | 405 | 100 | NR_118554.1 Intestinimonas butyriciproducens strain SRB-521-5-I 16S ribosomal RNA, partial sequence | NR_118554.1 | 1475 | 346 | 750 | 27.46 | 577.3 | 2E-164 | 374/405 | 92 | 0/405 |
| ASV1361 | 419 | 1 | 1 | 419 | 100 | NR_104844.1 Fibrobacter succinogenes subsp. elongatus strain HM2 16S ribosomal RNA, partial sequence | NR_104844.1 | 1431 | 319 | 737 | 29.28 | 636.4 | 0 | 395/420 | 94 | 2/420 |
| ASV1362 | 419 | 1 | 1 | 419 | 100 | NR_104844.1 Fibrobacter succinogenes subsp. elongatus strain HM2 16S ribosomal RNA, partial sequence | NR_104844.1 | 1431 | 319 | 737 | 29.28 | 636.4 | 0 | 395/420 | 94 | 2/420 |
| ASV1363 | 403 | 1 | 3 | 403 | 99.5 | NR_102987.1 Hungateiclostridium clariflavum DSM 19732 16S ribosomal RNA, partial sequence | NR_102987.1 | 1634 | 483 | 882 | 24.48 | 473.9 | 3E-133 | 353/401 | 88 | 1/401 |
| ASV1364 | 422 | 1 | 1 | 422 | 100 | NR_041364.1 Prevotella stercorea DSM 18206 strain CB35 16S ribosomal RNA, partial sequence | NR_041364.1 | 1488 | 354 | 775 | 28.36 | 725 | 0 | 412/422 | 97 | 0/422 |
| ASV1365 | 402 | 1 | 1 | 402 | 100 | NR_152095.1 Herbinix luporum strain SD1D 16S ribosomal RNA, partial sequence | NR_152095.1 | 1521 | 365 | 766 | 26.43 | 682.5 | 0 | 391/402 | 97 | 0/402 |
| ASV1366 | 401 | 1 | 1 | 401 | 100 | NR_158111.1 Paludicola psychrotolerans strain NC1253 16S ribosomal RNA, partial sequence | NR_158111.1 | 1490 | 366 | 766 | 26.91 | 625.3 | 7E-179 | 380/401 | 94 | 0/401 |
| ASV1367 | 402 | 1 | 1 | 402 | 100 | NR_102987.1 Hungateiclostridium clariflavum DSM 19732 16S ribosomal RNA, partial sequence | NR_102987.1 | 1634 | 481 | 882 | 24.6 | 516.3 | 4E-146 | 361/402 | 89 | 0/402 |
| ASV1368 | 402 | 1 | 1 | 402 | 100 | NR_151895.1 Anaerocolumna cellulosilytica strain SN021 16S ribosomal RNA, partial sequence | NR_151895.1 | 1478 | 353 | 754 | 27.2 | 671.5 | 0 | 389/402 | 96 | 0/402 |
| ASV1369 | 404 | 1 | 1 | 404 | 100 | NR_118156.1 Oscillibacter ruminantium GH1 16S ribosomal RNA, partial sequence | NR_118156.1 | 1409 | 321 | 724 | 28.67 | 597.6 | 2E-170 | 378/405 | 93 | 2/405 |
| ASV1370 | 404 | 1 | 1 | 404 | 100 | NR_118156.1 Oscillibacter ruminantium GH1 16S ribosomal RNA, partial sequence | NR_118156.1 | 1409 | 321 | 724 | 28.67 | 658.5 | 0 | 390/406 | 96 | 4/406 |
| ASV1371 | 422 | 1 | 1 | 422 | 100 | NR_113411.1 Prevotella copri DSM 18205 strain JCM 13464 16S ribosomal RNA, partial sequence | NR_113411.1 | 1493 | 358 | 779 | 28.27 | 586.5 | 3E-167 | 387/422 | 91 | 0/422 |
| ASV1372 | 427 | 1 | 1 | 427 | 100 | NR_041666.1 Dialister succinatiphilus YIT 11850 16S ribosomal RNA, partial sequence | NR_041666.1 | 1521 | 353 | 779 | 28.07 | 706.5 | 0 | 413/428 | 96 | 2/428 |
| ASV1373 | 427 | 1 | 1 | 427 | 100 | NR_026030.1 Actinobacillus porcinus strain NM319 16S ribosomal RNA, partial sequence | NR_026030.1 | 1466 | 335 | 761 | 29.13 | 784.1 | 0 | 426/427 | 99 | 0/427 |
| ASV1374 | 422 | 3 | 1 | 422 | 100 | NR_074577.1 Paludibacter propionicigenes WB4 16S ribosomal RNA, partial sequence | NR_074577.1 | 1516 | 353 | 774 | 27.84 | 486.8 | 4E-137 | 371/424 | 87 | 4/424 |
| ASV1375 | 402 | 5 | 1 | 402 | 100 | NR_115693.1 Gracilibacter thermotolerans JW/YJL-S1 16S ribosomal RNA, partial sequence | NR_115693.1 | 1526 | 459 | 861 | 26.41 | 527.4 | 2E-149 | 366/405 | 90 | 5/405 |
| ASV1376 | 403 | 1 | 1 | 403 | 100 | NR_104907.1 Eubacterium coprostanoligenes strain HL 16S ribosomal RNA, partial sequence | NR_104907.1 | 1446 | 332 | 734 | 27.87 | 573.6 | 3E-163 | 372/403 | 92 | 0/403 |
| ASV1377 | 405 | 1 | 1 | 405 | 100 | NR_118554.1 Intestinimonas butyriciproducens strain SRB-521-5-I 16S ribosomal RNA, partial sequence | NR_118554.1 | 1475 | 346 | 750 | 27.46 | 499.7 | 4E-141 | 360/405 | 88 | 0/405 |
| ASV1378 | 427 | 1 | 1 | 427 | 100 | NR_041666.1 Dialister succinatiphilus YIT 11850 16S ribosomal RNA, partial sequence | NR_041666.1 | 1521 | 353 | 779 | 28.07 | 778.6 | 0 | 425/427 | 99 | 0/427 |
| ASV1379 | 421 | 2 | 1 | 421 | 100 | NR_113073.1 Barnesiella intestinihominis strain JCM 15079 16S ribosomal RNA, partial sequence | NR_113073.1 | 1481 | 346 | 767 | 28.49 | 479.4 | 6E-135 | 369/423 | 87 | 3/423 |
| ASV1380 | 402 | 1 | 1 | 402 | 100 | NR_104846.1 Gemmiger formicilis strain X2-56 16S ribosomal RNA, partial sequence | NR_104846.1 | 1426 | 305 | 706 | 28.19 | 682.5 | 0 | 391/402 | 97 | 0/402 |
| ASV1381 | 427 | 2 | 1 | 427 | 100 | NR_037018.1 Acidaminococcus fermentans strain VR4 16S ribosomal RNA gene, partial sequence | NR_037018.1 | 1516 | 349 | 775 | 28.17 | 784.1 | 0 | 426/427 | 99 | 0/427 |
| ASV1382 | 427 | 3 | 3 | 427 | 99.53 | NR_041781.1 Streptococcus alactolyticus strain ATCC 43077 16S ribosomal RNA, partial sequence | NR_041781.1 | 1437 | 348 | 772 | 29.58 | 619.7 | 3E-177 | 401/431 | 93 | 12/431 |
| ASV1383 | 427 | 1 | 1 | 426 | 99.77 | NR_027596.1 Mitsuokella multacida strain DSM 20544 16S ribosomal RNA, partial sequence | NR_027596.1 | 1511 | 346 | 771 | 28.19 | 656.7 | 0 | 402/426 | 94 | 0/426 |
| ASV1384 | 427 | 3 | 1 | 427 | 100 | NR_119168.1 Veillonella parvula DSM 2008 16S ribosomal RNA, partial sequence | NR_119168.1 | 1513 | 348 | 774 | 28.22 | 789.6 | 0 | 427/427 | 100 | 0/427 |
| ASV1385 | 405 | 1 | 1 | 405 | 100 | NR_144742.1 Christensenella massiliensis strain Marseille-P2438 16S ribosomal RNA, partial sequence | NR_144742.1 | 1499 | 369 | 773 | 27.02 | 466.5 | 4E-131 | 354/405 | 87 | 0/405 |
| ASV1386 | 422 | 2 | 1 | 422 | 100 | NR_113123.1 Prevotella timonensis 4401737 = DSM 22865 = JCM 15640 16S ribosomal RNA, partial sequence | NR_113123.1 | 1493 | 356 | 777 | 28.27 | 608.7 | 7E-174 | 392/423 | 92 | 2/423 |
| ASV1387 | 404 | 2 | 1 | 404 | 100 | NR_074793.2 Oscillibacter valericigenes strain Sjm18-20 16S ribosomal RNA, complete sequence | NR_074793.2 | 1530 | 372 | 775 | 26.41 | 625.3 | 7E-179 | 382/404 | 94 | 0/404 |
| ASV1388 | 420 | 2 | 1 | 420 | 100 | NR_029335.1 Holdemania filiformis strain J1-31B-1 16S ribosomal RNA, partial sequence | NR_029335.1 | 1391 | 267 | 692 | 30.63 | 460.9 | 2E-129 | 368/426 | 86 | 6/426 |
| ASV1389 | 403 | 1 | 3 | 403 | 99.5 | NR_125677.1 Vallitalea pronyensis strain FatNI3 16S ribosomal RNA, partial sequence | NR_125677.1 | 1480 | 365 | 764 | 27.03 | 440.6 | 3E-123 | 348/402 | 86 | 3/402 |
| ASV1390 | 422 | 1 | 1 | 422 | 100 | NR_112943.1 Bacteroides stercoris ATCC 43183 strain JCM 9496 16S ribosomal RNA, partial sequence | NR_112943.1 | 1476 | 343 | 764 | 28.59 | 680.7 | 0 | 404/422 | 95 | 0/422 |
| ASV1391 | 402 | 1 | 1 | 402 | 100 | NR_144736.1 Neglecta timonensis strain SN17 16S ribosomal RNA, partial sequence | NR_144736.1 | 1503 | 377 | 778 | 26.75 | 671.5 | 0 | 390/403 | 96 | 2/403 |
| ASV1392 | 403 | 1 | 1 | 403 | 100 | NR_144609.1 Enterorhabdus muris strain WCA-131-CoC-2 16S ribosomal RNA, partial sequence | NR_144609.1 | 1399 | 336 | 738 | 28.81 | 623.4 | 3E-178 | 381/403 | 94 | 0/403 |
| ASV1393 | 427 | 2 | 1 | 427 | 100 | NR_029106.1 Lactobacillus delbrueckii subsp. indicus strain NCC725 16S ribosomal RNA, partial sequence | NR_029106.1 | 1515 | 348 | 774 | 28.18 | 773 | 0 | 424/427 | 99 | 0/427 |
| ASV1394 | 402 | 1 | 2 | 402 | 99.75 | NR_027579.1 Ruminococcus lactaris ATCC 29176 16S ribosomal RNA, partial sequence | NR_027579.1 | 1402 | 334 | 734 | 28.6 | 671.5 | 0 | 388/401 | 96 | 0/401 |
| ASV1395 | 402 | 2 | 1 | 402 | 100 | NR_042144.1 Clostridium butyricum strain VPI3266 16S ribosomal RNA, partial sequence | NR_042144.1 | 1425 | 338 | 739 | 28.21 | 677 | 0 | 390/402 | 97 | 0/402 |
| ASV1396 | 402 | 1 | 1 | 402 | 100 | NR_151900.1 Ruthenibacterium lactatiformans strain 585-1 16S ribosomal RNA, partial sequence | NR_151900.1 | 1519 | 368 | 769 | 26.46 | 743.5 | 0 | 402/402 | 100 | 0/402 |
| ASV1397 | 405 | 1 | 1 | 405 | 100 | NR_118554.1 Intestinimonas butyriciproducens strain SRB-521-5-I 16S ribosomal RNA, partial sequence | NR_118554.1 | 1475 | 346 | 750 | 27.46 | 494.2 | 2E-139 | 359/405 | 88 | 0/405 |
| ASV1398 | 404 | 1 | 1 | 404 | 100 | NR_118156.1 Oscillibacter ruminantium GH1 16S ribosomal RNA, partial sequence | NR_118156.1 | 1409 | 321 | 724 | 28.67 | 658.5 | 0 | 388/404 | 96 | 0/404 |
| ASV1400 | 402 | 1 | 1 | 402 | 100 | NR_074613.1 [Eubacterium] eligens ATCC 27750 16S ribosomal RNA, partial sequence | NR_074613.1 | 1519 | 367 | 768 | 26.46 | 737.9 | 0 | 401/402 | 99 | 0/402 |
| ASV1401 | 427 | 1 | 1 | 426 | 99.77 | NR_027596.1 Mitsuokella multacida strain DSM 20544 16S ribosomal RNA, partial sequence | NR_027596.1 | 1511 | 346 | 771 | 28.19 | 656.7 | 0 | 402/426 | 94 | 0/426 |
| ASV1402 | 404 | 1 | 1 | 404 | 100 | NR_025025.1 Papillibacter cinnamivorans strain CIN1 16S ribosomal RNA, partial sequence | NR_025025.1 | 1491 | 349 | 751 | 27.03 | 595.7 | 5E-170 | 377/405 | 93 | 3/405 |
| ASV1403 | 403 | 1 | 3 | 403 | 99.5 | NR_125464.1 Anaerobacterium chartisolvens strain T-1-35 16S ribosomal RNA, partial sequence | NR_125464.1 | 1451 | 344 | 745 | 27.71 | 492.3 | 7E-139 | 358/403 | 88 | 3/403 |
| ASV1405 | 421 | 2 | 1 | 421 | 100 | NR_113077.1 Paraprevotella clara strain JCM 14859 16S ribosomal RNA, partial sequence | NR_113077.1 | 1480 | 346 | 766 | 28.45 | 473.9 | 3E-133 | 368/423 | 86 | 4/423 |
| ASV1406 | 422 | 1 | 1 | 422 | 100 | NR_113208.1 Parabacteroides chinchillae strain JCM 17104 16S ribosomal RNA, partial sequence | NR_113208.1 | 1489 | 353 | 774 | 28.34 | 647.4 | 0 | 399/423 | 94 | 2/423 |
| ASV1407 | 427 | 1 | 1 | 427 | 100 | NR_024994.1 Lactobacillus mucosae strain S32 16S ribosomal RNA, partial sequence | NR_024994.1 | 1568 | 384 | 810 | 27.23 | 778.6 | 0 | 425/427 | 99 | 0/427 |
| ASV1408 | 421 | 1 | 1 | 421 | 100 | NR_144616.1 Muribaculum intestinale strain YL27 16S ribosomal RNA, partial sequence | NR_144616.1 | 1460 | 297 | 718 | 28.9 | 534.8 | 1E-151 | 382/426 | 89 | 9/426 |
| ASV1409 | 422 | 2 | 1 | 422 | 100 | NR_113117.1 Prevotella oralis ATCC 33269 strain JCM 12251 16S ribosomal RNA, partial sequence | NR_113117.1 | 1496 | 361 | 782 | 28.21 | 536.6 | 4E-152 | 380/424 | 89 | 4/424 |
| ASV1410 | 405 | 1 | 1 | 405 | 100 | NR_118554.1 Intestinimonas butyriciproducens strain SRB-521-5-I 16S ribosomal RNA, partial sequence | NR_118554.1 | 1475 | 346 | 750 | 27.46 | 582.8 | 4E-166 | 375/405 | 92 | 0/405 |
| ASV1411 | 416 | 2 | 1 | 416 | 100 | NR_024851.1 Azoarcus indigens strain VB32 16S ribosomal RNA, partial sequence | NR_024851.1 | 1484 | 353 | 779 | 28.77 | 424 | 3E-118 | 365/429 | 85 | 15/429 |
| ASV1412 | 422 | 1 | 1 | 422 | 100 | NR_112835.1 Parabacteroides gordonii strain MS-1 16S ribosomal RNA, partial sequence | NR_112835.1 | 1485 | 353 | 773 | 28.35 | 773 | 0 | 421/422 | 99 | 1/422 |
| ASV1413 | 427 | 2 | 1 | 427 | 100 | NR_037018.1 Acidaminococcus fermentans strain VR4 16S ribosomal RNA gene, partial sequence | NR_037018.1 | 1516 | 349 | 775 | 28.17 | 773 | 0 | 424/427 | 99 | 0/427 |
| ASV1414 | 427 | 5 | 3 | 427 | 99.53 | NR_133868.1 Streptococcus phocae subsp. salmonis strain C-4 16S ribosomal RNA, partial sequence | NR_133868.1 | 1410 | 293 | 717 | 30.14 | 581 | 2E-165 | 393/430 | 91 | 10/430 |
| ASV1415 | 422 | 2 | 1 | 422 | 100 | NR_113119.1 Prevotella oris strain JCM 8540 16S ribosomal RNA, partial sequence | NR_113119.1 | 1481 | 345 | 766 | 28.49 | 592 | 7E-169 | 389/423 | 91 | 2/423 |
| ASV1416 | 426 | 1 | 1 | 426 | 100 | NR_029335.1 Holdemania filiformis strain J1-31B-1 16S ribosomal RNA, partial sequence | NR_029335.1 | 1391 | 267 | 692 | 30.63 | 582.8 | 5E-166 | 390/427 | 91 | 2/427 |
| ASV1418 | 405 | 1 | 1 | 405 | 100 | NR_044616.1 Synergistes jonesii strain 78-1 16S ribosomal RNA, partial sequence | NR_044616.1 | 1355 | 345 | 749 | 29.89 | 732.4 | 0 | 401/405 | 99 | 0/405 |
| ASV1419 | 405 | 1 | 1 | 405 | 100 | NR_118554.1 Intestinimonas butyriciproducens strain SRB-521-5-I 16S ribosomal RNA, partial sequence | NR_118554.1 | 1475 | 346 | 750 | 27.46 | 521.9 | 9E-148 | 364/405 | 89 | 0/405 |
| ASV1420 | 422 | 1 | 1 | 422 | 100 | NR_043805.1 Olivibacter sitiensis strain AW-6 16S ribosomal RNA, partial sequence | NR_043805.1 | 1477 | 328 | 749 | 28.57 | 453.5 | 4E-127 | 364/423 | 86 | 2/423 |
| ASV1421 | 402 | 3 | 1 | 402 | 100 | NR_156081.1 Kineothrix alysoides strain KNHs209 16S ribosomal RNA, partial sequence | NR_156081.1 | 1411 | 353 | 754 | 28.49 | 665.9 | 0 | 388/402 | 96 | 0/402 |
| ASV1422 | 427 | 1 | 1 | 427 | 100 | NR_028796.1 Selenomonas noxia strain ATCC 43541 16S ribosomal RNA, partial sequence | NR_028796.1 | 1491 | 375 | 801 | 28.64 | 579.1 | 6E-165 | 391/429 | 91 | 4/429 |
| ASV1423 | 427 | 1 | 1 | 427 | 100 | NR_102980.1 Megasphaera elsdenii DSM 20460 16S ribosomal RNA, partial sequence | NR_102980.1 | 1552 | 369 | 795 | 27.51 | 784.1 | 0 | 426/427 | 99 | 0/427 |
| ASV1424 | 426 | 1 | 1 | 426 | 100 | NR_026205.1 Succiniclasticum ruminis strain SE10 16S ribosomal RNA, partial sequence | NR_026205.1 | 1505 | 354 | 780 | 28.37 | 605 | 1E-172 | 395/428 | 92 | 3/428 |
| ASV1425 | 405 | 3 | 1 | 405 | 100 | NR_115307.1 Ethanoligenens harbinense YUAN-3 16S ribosomal RNA, partial sequence | NR_115307.1 | 1497 | 340 | 743 | 26.99 | 564.3 | 2E-160 | 374/407 | 91 | 5/407 |
| ASV1426 | 421 | 1 | 1 | 421 | 100 | NR_144616.1 Muribaculum intestinale strain YL27 16S ribosomal RNA, partial sequence | NR_144616.1 | 1460 | 297 | 718 | 28.9 | 540.3 | 3E-153 | 381/424 | 89 | 5/424 |
| ASV1427 | 421 | 1 | 1 | 421 | 100 | NR_144616.1 Muribaculum intestinale strain YL27 16S ribosomal RNA, partial sequence | NR_144616.1 | 1460 | 297 | 718 | 28.9 | 501.6 | 1E-141 | 375/425 | 88 | 7/425 |
| ASV1428 | 405 | 2 | 1 | 405 | 100 | NR_074629.1 Hungateiclostridium thermocellum strain ATCC 27405 16S ribosomal RNA, partial sequence | NR_074629.1 | 1521 | 358 | 762 | 26.63 | 588.4 | 9E-168 | 379/408 | 92 | 6/408 |
| ASV1429 | 422 | 1 | 1 | 422 | 100 | NR_113411.1 Prevotella copri DSM 18205 strain JCM 13464 16S ribosomal RNA, partial sequence | NR_113411.1 | 1493 | 358 | 779 | 28.27 | 702.8 | 0 | 409/423 | 96 | 2/423 |
| ASV1430 | 427 | 1 | 1 | 427 | 100 | NR_036788.2 Lactobacillus pontis strain LTH 2587 16S ribosomal RNA, partial sequence | NR_036788.2 | 1568 | 384 | 810 | 27.23 | 761.9 | 0 | 422/427 | 98 | 0/427 |
| ASV1431 | 421 | 1 | 1 | 421 | 100 | NR_144616.1 Muribaculum intestinale strain YL27 16S ribosomal RNA, partial sequence | NR_144616.1 | 1460 | 297 | 718 | 28.9 | 507.1 | 3E-143 | 373/422 | 88 | 1/422 |
| ASV1433 | 402 | 1 | 1 | 402 | 100 | NR_118676.1 Eubacterium xylanophilum strain ATCC 35991 16S ribosomal RNA, partial sequence | NR_118676.1 | 1494 | 387 | 788 | 26.91 | 697.3 | 0 | 392/402 | 97 | 0/402 |
| ASV1434 | 425 | 1 | 1 | 425 | 100 | NR_029325.1 Schwartzia succinivorans strain S1-1 16S ribosomal RNA, partial sequence | NR_029325.1 | 1489 | 344 | 769 | 28.61 | 470.2 | 4E-132 | 372/429 | 86 | 7/429 |
| ASV1435 | 402 | 1 | 1 | 402 | 100 | NR_042832.1 Roseburia faecis strain M72/1 16S ribosomal RNA, partial sequence | NR_042832.1 | 1456 | 347 | 749 | 27.68 | 665.9 | 0 | 390/404 | 96 | 3/404 |
| ASV1436 | 403 | 1 | 3 | 403 | 99.5 | NR_125677.1 Vallitalea pronyensis strain FatNI3 16S ribosomal RNA, partial sequence | NR_125677.1 | 1480 | 365 | 764 | 27.03 | 490.5 | 3E-138 | 360/405 | 88 | 9/405 |
| ASV1437 | 422 | 1 | 1 | 422 | 100 | NR_041364.1 Prevotella stercorea DSM 18206 strain CB35 16S ribosomal RNA, partial sequence | NR_041364.1 | 1488 | 354 | 775 | 28.36 | 713.9 | 0 | 410/422 | 97 | 0/422 |
| ASV1438 | 404 | 2 | 1 | 404 | 100 | NR_114326.1 Fusicatenibacter saccharivorans strain HT03-11 16S ribosomal RNA, partial sequence | NR_114326.1 | 1423 | 351 | 752 | 28.25 | 662.2 | 0 | 389/404 | 96 | 2/404 |
| ASV1439 | 403 | 1 | 1 | 403 | 100 | NR_025930.1 Ruminococcus bromii strain ATCC 27255 16S ribosomal RNA, partial sequence | NR_025930.1 | 1380 | 313 | 716 | 29.28 | 579.1 | 6E-165 | 374/404 | 92 | 1/404 |
| ASV1440 | 421 | 2 | 1 | 421 | 100 | NR_113077.1 Paraprevotella clara strain JCM 14859 16S ribosomal RNA, partial sequence | NR_113077.1 | 1480 | 346 | 766 | 28.45 | 468.3 | 1E-131 | 367/423 | 86 | 4/423 |
| ASV1441 | 402 | 3 | 1 | 402 | 100 | NR_156081.1 Kineothrix alysoides strain KNHs209 16S ribosomal RNA, partial sequence | NR_156081.1 | 1411 | 353 | 754 | 28.49 | 693.6 | 0 | 393/402 | 97 | 0/402 |
| ASV1442 | 404 | 1 | 1 | 404 | 100 | NR_042007.1 Roseburia inulinivorans DSM 16841 strain A2-194 16S ribosomal RNA, partial sequence | NR_042007.1 | 1454 | 360 | 763 | 27.79 | 741.6 | 0 | 403/404 | 99 | 0/404 |
| ASV1443 | 402 | 1 | 1 | 402 | 100 | NR_104846.1 Gemmiger formicilis strain X2-56 16S ribosomal RNA, partial sequence | NR_104846.1 | 1426 | 305 | 706 | 28.19 | 737.9 | 0 | 401/402 | 99 | 0/402 |
| ASV1444 | 403 | 1 | 3 | 403 | 99.5 | NR_125677.1 Vallitalea pronyensis strain FatNI3 16S ribosomal RNA, partial sequence | NR_125677.1 | 1480 | 365 | 764 | 27.03 | 484.9 | 1E-136 | 357/403 | 88 | 5/403 |
| ASV1445 | 422 | 1 | 1 | 422 | 100 | NR_041364.1 Prevotella stercorea DSM 18206 strain CB35 16S ribosomal RNA, partial sequence | NR_041364.1 | 1488 | 354 | 775 | 28.36 | 719.5 | 0 | 411/422 | 97 | 0/422 |
| ASV1446 | 421 | 1 | 1 | 421 | 100 | NR_144616.1 Muribaculum intestinale strain YL27 16S ribosomal RNA, partial sequence | NR_144616.1 | 1460 | 297 | 718 | 28.9 | 484.9 | 1E-136 | 372/425 | 87 | 7/425 |
| ASV1447 | 405 | 1 | 1 | 405 | 100 | NR_125464.1 Anaerobacterium chartisolvens strain T-1-35 16S ribosomal RNA, partial sequence | NR_125464.1 | 1451 | 342 | 745 | 27.84 | 575.4 | 7E-164 | 374/405 | 92 | 1/405 |
| ASV1448 | 427 | 1 | 1 | 427 | 100 | NR_028797.1 Selenomonas infelix strain VPI D 75 B-30 16S ribosomal RNA, partial sequence | NR_028797.1 | 1524 | 373 | 799 | 28.02 | 612.4 | 6E-175 | 395/427 | 92 | 0/427 |
| ASV1449 | 401 | 1 | 1 | 401 | 100 | NR_144737.1 Emergencia timonensis strain SN18 16S ribosomal RNA, partial sequence | NR_144737.1 | 1480 | 364 | 767 | 27.3 | 621.6 | 9E-178 | 382/404 | 94 | 3/404 |
| ASV1450 | 427 | 1 | 1 | 427 | 100 | NR_117574.1 Lactobacillus johnsonii strain CIP 103620 16S ribosomal RNA, partial sequence | NR_117574.1 | 1543 | 366 | 792 | 27.67 | 784.1 | 0 | 426/427 | 99 | 0/427 |
| ASV1451 | 427 | 2 | 1 | 426 | 99.77 | NR_027596.1 Mitsuokella multacida strain DSM 20544 16S ribosomal RNA, partial sequence | NR_027596.1 | 1511 | 346 | 771 | 28.19 | 632.7 | 0 | 400/428 | 93 | 4/428 |
| ASV1452 | 422 | 1 | 1 | 422 | 100 | NR_040877.1 Prevotella copri DSM 18205 strain CB7 16S ribosomal RNA, partial sequence | NR_040877.1 | 1494 | 358 | 779 | 28.25 | 737.9 | 0 | 414/422 | 98 | 0/422 |
| ASV1453 | 402 | 1 | 1 | 402 | 100 | NR_074613.1 [Eubacterium] eligens ATCC 27750 16S ribosomal RNA, partial sequence | NR_074613.1 | 1519 | 367 | 768 | 26.46 | 599.4 | 4E-171 | 376/402 | 93 | 0/402 |
| ASV1454 | 403 | 1 | 3 | 403 | 99.5 | NR_125464.1 Anaerobacterium chartisolvens strain T-1-35 16S ribosomal RNA, partial sequence | NR_125464.1 | 1451 | 344 | 745 | 27.71 | 492.3 | 7E-139 | 357/402 | 88 | 1/402 |
| ASV1455 | 422 | 1 | 1 | 422 | 100 | NR_041364.1 Prevotella stercorea DSM 18206 strain CB35 16S ribosomal RNA, partial sequence | NR_041364.1 | 1488 | 354 | 775 | 28.36 | 713.9 | 0 | 410/422 | 97 | 0/422 |
| ASV1456 | 402 | 1 | 1 | 402 | 100 | NR_113021.1 Clostridium paraputrificum strain JCM 1293 16S ribosomal RNA, partial sequence | NR_113021.1 | 1476 | 348 | 749 | 27.24 | 638.2 | 0 | 383/402 | 95 | 0/402 |
| ASV1457 | 427 | 3 | 1 | 427 | 100 | NR_041781.1 Streptococcus alactolyticus strain ATCC 43077 16S ribosomal RNA, partial sequence | NR_041781.1 | 1437 | 346 | 772 | 29.71 | 784.1 | 0 | 426/427 | 99 | 0/427 |
| ASV1458 | 422 | 1 | 1 | 422 | 100 | NR_144616.1 Muribaculum intestinale strain YL27 16S ribosomal RNA, partial sequence | NR_144616.1 | 1460 | 297 | 718 | 28.9 | 603.1 | 3E-172 | 390/422 | 92 | 0/422 |
| ASV1459 | 402 | 1 | 1 | 402 | 100 | NR_159227.1 Monoglobus pectinilyticus strain 14 16S ribosomal RNA, partial sequence | NR_159227.1 | 1493 | 368 | 769 | 26.93 | 660.4 | 0 | 387/402 | 96 | 0/402 |
| ASV1460 | 422 | 2 | 1 | 422 | 100 | NR_113105.1 Prevotella histicola strain JCM 15637 16S ribosomal RNA, partial sequence | NR_113105.1 | 1480 | 346 | 767 | 28.51 | 636.4 | 0 | 397/423 | 93 | 2/423 |
| ASV1461 | 421 | 1 | 1 | 421 | 100 | NR_144616.1 Muribaculum intestinale strain YL27 16S ribosomal RNA, partial sequence | NR_144616.1 | 1460 | 297 | 718 | 28.9 | 484.9 | 1E-136 | 374/427 | 87 | 11/427 |
| ASV1462 | 405 | 3 | 1 | 405 | 100 | NR_115307.1 Ethanoligenens harbinense YUAN-3 16S ribosomal RNA, partial sequence | NR_115307.1 | 1497 | 340 | 743 | 26.99 | 520 | 3E-147 | 366/407 | 89 | 5/407 |
| ASV1463 | 402 | 2 | 1 | 402 | 100 | NR_041960.1 Blautia luti strain BInIX 16S ribosomal RNA, partial sequence | NR_041960.1 | 1308 | 239 | 641 | 30.81 | 693.6 | 0 | 394/403 | 97 | 1/403 |
| ASV1465 | 401 | 1 | 1 | 401 | 100 | NR_144740.1 Romboutsia timonensis strain DR1 16S ribosomal RNA, partial sequence | NR_144740.1 | 1466 | 338 | 738 | 27.35 | 736.1 | 0 | 400/401 | 99 | 0/401 |
| ASV1466 | 404 | 1 | 1 | 404 | 100 | NR_025670.1 Pseudoflavonifractor capillosus ATCC 29799 16S ribosomal RNA, partial sequence | NR_025670.1 | 1483 | 346 | 750 | 27.31 | 514.5 | 2E-145 | 363/405 | 89 | 1/405 |
| ASV1467 | 405 | 1 | 1 | 405 | 100 | NR_144611.1 Flintibacter butyricus strain BLS21 16S ribosomal RNA, partial sequence | NR_144611.1 | 808 | 299 | 703 | 50.12 | 527.4 | 2E-149 | 366/406 | 90 | 2/406 |
| ASV1468 | 402 | 2 | 1 | 402 | 100 | NR_114315.1 Blautia luti DSM 14534 16S ribosomal RNA, partial sequence | NR_114315.1 | 1481 | 353 | 754 | 27.14 | 688.1 | 0 | 392/402 | 97 | 0/402 |
| ASV1469 | 402 | 1 | 1 | 402 | 100 | NR_024750.1 Coprococcus catus strain VPI-C6-61 16S ribosomal RNA, partial sequence | NR_024750.1 | 1315 | 317 | 719 | 30.65 | 723.2 | 0 | 399/403 | 99 | 1/403 |
| ASV1470 | 427 | 1 | 1 | 427 | 100 | NR_042797.1 Treponema berlinense strain 7CPL208 16S ribosomal RNA, partial sequence | NR_042797.1 | 1440 | 275 | 701 | 29.65 | 695.5 | 0 | 410/427 | 96 | 0/427 |
| ASV1471 | 422 | 3 | 1 | 422 | 100 | NR_074577.1 Paludibacter propionicigenes WB4 16S ribosomal RNA, partial sequence | NR_074577.1 | 1516 | 353 | 774 | 27.84 | 514.5 | 2E-145 | 377/425 | 88 | 6/425 |
| ASV1472 | 403 | 1 | 3 | 403 | 99.5 | NR_125464.1 Anaerobacterium chartisolvens strain T-1-35 16S ribosomal RNA, partial sequence | NR_125464.1 | 1451 | 344 | 745 | 27.71 | 481.2 | 2E-135 | 355/402 | 88 | 1/402 |
| ASV1473 | 405 | 1 | 1 | 405 | 100 | NR_148302.1 Geosporobacter ferrireducens strain IRF9 16S ribosomal RNA, partial sequence | NR_148302.1 | 1392 | 306 | 707 | 28.88 | 479.4 | 6E-135 | 359/407 | 88 | 7/407 |
| ASV1474 | 422 | 3 | 1 | 422 | 100 | NR_113411.1 Prevotella copri DSM 18205 strain JCM 13464 16S ribosomal RNA, partial sequence | NR_113411.1 | 1493 | 358 | 779 | 28.27 | 592 | 7E-169 | 390/424 | 91 | 4/424 |
| ASV1476 | 426 | 2 | 1 | 426 | 100 | NR_029325.1 Schwartzia succinivorans strain S1-1 16S ribosomal RNA, partial sequence | NR_029325.1 | 1489 | 344 | 769 | 28.61 | 455.4 | 1E-127 | 366/426 | 85 | 0/426 |
| ASV1477 | 427 | 1 | 1 | 427 | 100 | NR_044111.1 Selenomonas bovis strain WG 16S ribosomal RNA, partial sequence | NR_044111.1 | 1525 | 370 | 796 | 28 | 629 | 6E-180 | 403/432 | 93 | 10/432 |
| ASV1478 | 401 | 1 | 1 | 401 | 100 | NR_027558.1 Anaerotruncus colihominis strain WAL 14565 16S ribosomal RNA, partial sequence | NR_027558.1 | 1365 | 309 | 709 | 29.38 | 603.1 | 3E-172 | 376/401 | 93 | 0/401 |
| ASV1479 | 422 | 1 | 1 | 422 | 100 | NR_043805.1 Olivibacter sitiensis strain AW-6 16S ribosomal RNA, partial sequence | NR_043805.1 | 1477 | 328 | 749 | 28.57 | 459.1 | 8E-129 | 366/424 | 86 | 4/424 |
| ASV1480 | 422 | 1 | 1 | 422 | 100 | NR_041342.1 Parabacteroides distasonis strain JCM 5825 16S ribosomal RNA, partial sequence | NR_041342.1 | 1488 | 353 | 774 | 28.36 | 569.9 | 3E-162 | 384/422 | 90 | 0/422 |
| ASV1481 | 402 | 3 | 1 | 402 | 100 | NR_114728.1 Clostridium tertium strain ATCC 14573 16S ribosomal RNA, partial sequence | NR_114728.1 | 1415 | 304 | 705 | 28.41 | 616.1 | 4E-176 | 380/403 | 94 | 2/403 |
| ASV1482 | 401 | 1 | 1 | 401 | 100 | NR_144740.1 Romboutsia timonensis strain DR1 16S ribosomal RNA, partial sequence | NR_144740.1 | 1466 | 338 | 738 | 27.35 | 736.1 | 0 | 400/401 | 99 | 0/401 |
| ASV1483 | 422 | 2 | 1 | 422 | 100 | NR_113070.1 Bacteroides oleiciplenus YIT 12058 strain JCM 16102 16S ribosomal RNA, partial sequence | NR_113070.1 | 1485 | 351 | 772 | 28.42 | 653 | 0 | 399/422 | 94 | 0/422 |
| ASV1484 | 422 | 1 | 1 | 422 | 100 | NR_041364.1 Prevotella stercorea DSM 18206 strain CB35 16S ribosomal RNA, partial sequence | NR_041364.1 | 1488 | 354 | 775 | 28.36 | 603.1 | 3E-172 | 392/424 | 92 | 4/424 |
| ASV1486 | 403 | 1 | 3 | 403 | 99.5 | NR_125464.1 Anaerobacterium chartisolvens strain T-1-35 16S ribosomal RNA, partial sequence | NR_125464.1 | 1451 | 344 | 745 | 27.71 | 497.9 | 2E-140 | 358/402 | 89 | 1/402 |
| ASV1487 | 421 | 1 | 1 | 421 | 100 | NR_144616.1 Muribaculum intestinale strain YL27 16S ribosomal RNA, partial sequence | NR_144616.1 | 1460 | 297 | 718 | 28.9 | 507.1 | 3E-143 | 373/422 | 88 | 1/422 |
| ASV1489 | 403 | 1 | 1 | 403 | 100 | NR_147378.1 Negativibacillus massiliensis strain Marseille-P3213 16S ribosomal RNA, partial sequence | NR_147378.1 | 1479 | 356 | 758 | 27.25 | 745.3 | 0 | 403/403 | 100 | 0/403 |
| ASV1490 | 423 | 1 | 1 | 423 | 100 | NR_144750.1 Prevotellamassilia timonensis strain Marseille-P2831 16S ribosomal RNA, partial sequence | NR_144750.1 | 1493 | 358 | 779 | 28.27 | 553.3 | 4E-157 | 385/426 | 90 | 7/426 |
| ASV1491 | 402 | 2 | 1 | 402 | 100 | NR_115104.1 Helicobacter canadensis strain CCUG 47163 16S ribosomal RNA, partial sequence | NR_115104.1 | 1471 | 314 | 715 | 27.33 | 743.5 | 0 | 402/402 | 100 | 0/402 |
| ASV1492 | 402 | 1 | 1 | 402 | 100 | NR_151895.1 Anaerocolumna cellulosilytica strain SN021 16S ribosomal RNA, partial sequence | NR_151895.1 | 1478 | 353 | 754 | 27.2 | 660.4 | 0 | 387/402 | 96 | 0/402 |
| ASV1493 | 421 | 1 | 1 | 421 | 100 | NR_113097.1 Prevotella buccae ATCC 33574 strain JCM 12245 16S ribosomal RNA, partial sequence | NR_113097.1 | 1480 | 343 | 764 | 28.51 | 457.2 | 3E-128 | 367/425 | 86 | 7/425 |
| ASV1494 | 402 | 1 | 4 | 402 | 99.25 | NR_104907.1 Eubacterium coprostanoligenes strain HL 16S ribosomal RNA, partial sequence | NR_104907.1 | 1446 | 334 | 734 | 27.73 | 551.4 | 1E-156 | 367/401 | 91 | 2/401 |
| ASV1495 | 402 | 1 | 1 | 402 | 100 | NR_151900.1 Ruthenibacterium lactatiformans strain 585-1 16S ribosomal RNA, partial sequence | NR_151900.1 | 1519 | 368 | 769 | 26.46 | 621.6 | 9E-178 | 380/402 | 94 | 0/402 |
| ASV1496 | 402 | 1 | 1 | 402 | 100 | NR_042832.1 Roseburia faecis strain M72/1 16S ribosomal RNA, partial sequence | NR_042832.1 | 1456 | 347 | 749 | 27.68 | 682.5 | 0 | 392/403 | 97 | 1/403 |
| ASV1497 | 405 | 1 | 1 | 405 | 100 | NR_026061.1 Desulfohalotomaculum halophilum strain SEBR 3139 16S ribosomal RNA, partial sequence | NR_026061.1 | 1526 | 365 | 768 | 26.47 | 481.2 | 2E-135 | 357/405 | 88 | 1/405 |
| ASV1498 | 402 | 5 | 1 | 402 | 100 | NR_113231.1 Blautia glucerasea strain JCM 17039 16S ribosomal RNA, partial sequence | NR_113231.1 | 1491 | 364 | 765 | 26.96 | 577.3 | 2E-164 | 374/404 | 92 | 4/404 |
| ASV1499 | 402 | 1 | 1 | 402 | 100 | NR_024661.1 Eubacterium ruminantium strain GA195 16S ribosomal RNA, partial sequence | NR_024661.1 | 1508 | 378 | 779 | 26.66 | 682.5 | 0 | 391/402 | 97 | 0/402 |
| ASV1500 | 402 | 1 | 1 | 402 | 100 | NR_159227.1 Monoglobus pectinilyticus strain 14 16S ribosomal RNA, partial sequence | NR_159227.1 | 1493 | 368 | 769 | 26.93 | 654.8 | 0 | 386/402 | 96 | 0/402 |
| ASV1501 | 405 | 1 | 1 | 405 | 100 | NR_044972.1 Sporobacter termitidis strain SYR 16S ribosomal RNA, partial sequence | NR_044972.1 | 1506 | 348 | 752 | 26.89 | 531.1 | 2E-150 | 366/406 | 90 | 2/406 |
| ASV1502 | 422 | 1 | 1 | 422 | 100 | NR_041342.1 Parabacteroides distasonis strain JCM 5825 16S ribosomal RNA, partial sequence | NR_041342.1 | 1488 | 353 | 774 | 28.36 | 558.8 | 8E-159 | 382/422 | 90 | 0/422 |
| ASV1503 | 401 | 1 | 1 | 401 | 100 | NR_029355.1 [Clostridium] methylpentosum strain R2 16S ribosomal RNA, partial sequence | NR_029355.1 | 1435 | 349 | 750 | 28.01 | 581 | 2E-165 | 373/402 | 92 | 1/402 |
| ASV1504 | 402 | 1 | 1 | 402 | 100 | NR_102884.1 Ruminococcus champanellensis 18P13 = JCM 17042 16S ribosomal RNA, partial sequence | NR_102884.1 | 1494 | 348 | 749 | 26.91 | 643.8 | 0 | 386/404 | 95 | 4/404 |
| ASV1505 | 404 | 1 | 1 | 404 | 100 | NR_118156.1 Oscillibacter ruminantium GH1 16S ribosomal RNA, partial sequence | NR_118156.1 | 1409 | 321 | 724 | 28.67 | 619.7 | 3E-177 | 381/404 | 94 | 0/404 |
| ASV1506 | 402 | 1 | 1 | 402 | 100 | NR_156911.1 Fournierella massiliensis strain AT2 16S ribosomal RNA, partial sequence | NR_156911.1 | 1491 | 368 | 769 | 26.96 | 643.8 | 0 | 384/402 | 95 | 0/402 |
| ASV1507 | 427 | 1 | 1 | 427 | 100 | NR_028862.1 Allisonella histaminiformans strain MR2 16S ribosomal RNA, partial sequence | NR_028862.1 | 1537 | 376 | 802 | 27.78 | 773 | 0 | 424/427 | 99 | 0/427 |
| ASV1508 | 427 | 1 | 4 | 427 | 99.3 | NR_146687.1 Breznakia pachnodae strain Pei061 16S ribosomal RNA, partial sequence | NR_146687.1 | 1499 | 370 | 792 | 28.22 | 427.7 | 2E-119 | 363/427 | 85 | 7/427 |
| ASV1509 | 422 | 1 | 1 | 422 | 100 | NR_113411.1 Prevotella copri DSM 18205 strain JCM 13464 16S ribosomal RNA, partial sequence | NR_113411.1 | 1493 | 358 | 779 | 28.27 | 603.1 | 3E-172 | 390/422 | 92 | 0/422 |
| ASV1510 | 404 | 1 | 1 | 404 | 100 | NR_118156.1 Oscillibacter ruminantium GH1 16S ribosomal RNA, partial sequence | NR_118156.1 | 1409 | 321 | 724 | 28.67 | 664.1 | 0 | 391/406 | 96 | 4/406 |
| ASV1511 | 402 | 1 | 3 | 402 | 99.5 | NR_125677.1 Vallitalea pronyensis strain FatNI3 16S ribosomal RNA, partial sequence | NR_125677.1 | 1480 | 365 | 764 | 27.03 | 451.7 | 1E-126 | 349/401 | 87 | 2/401 |
| ASV1513 | 422 | 1 | 1 | 422 | 100 | NR_146018.1 Paludibacter jiangxiensis strain NM7 16S ribosomal RNA, partial sequence | NR_146018.1 | 1469 | 340 | 761 | 28.73 | 492.3 | 8E-139 | 374/426 | 87 | 8/426 |
| ASV1514 | 427 | 1 | 1 | 427 | 100 | NR_025131.1 Treponema parvum strain OMZ 833 16S ribosomal RNA, partial sequence | NR_025131.1 | 1485 | 341 | 767 | 28.75 | 590.2 | 3E-168 | 394/430 | 91 | 6/430 |
| ASV1515 | 427 | 1 | 1 | 427 | 100 | NR_153710.1 Peptococcus simiae strain M108 16S ribosomal RNA, partial sequence | NR_153710.1 | 1488 | 354 | 780 | 28.7 | 612.4 | 6E-175 | 399/431 | 92 | 8/431 |
| ASV1516 | 402 | 1 | 1 | 402 | 100 | NR_044049.1 Coprococcus eutactus strain ATCC 27759 16S ribosomal RNA, partial sequence | NR_044049.1 | 1440 | 333 | 734 | 27.92 | 693.6 | 0 | 393/402 | 97 | 0/402 |
| ASV1517 | 402 | 2 | 1 | 402 | 100 | NR_042144.1 Clostridium butyricum strain VPI3266 16S ribosomal RNA, partial sequence | NR_042144.1 | 1425 | 338 | 739 | 28.21 | 737.9 | 0 | 401/402 | 99 | 0/402 |
| ASV1518 | 427 | 1 | 1 | 427 | 100 | NR_157635.1 Megasphaera hexanoica strain MH 16S ribosomal RNA, partial sequence | NR_157635.1 | 1561 | 372 | 798 | 27.35 | 784.1 | 0 | 426/427 | 99 | 0/427 |
| ASV1519 | 402 | 1 | 1 | 402 | 100 | NR_026476.1 Succinivibrio dextrinosolvens strain 0554 16S ribosomal RNA, partial sequence | NR_026476.1 | 1463 | 331 | 732 | 27.48 | 677 | 0 | 390/402 | 97 | 0/402 |
| ASV1520 | 427 | 1 | 1 | 427 | 100 | NR_147405.1 Mailhella massiliensis strain Marseille-P3199 16S ribosomal RNA, partial sequence | NR_147405.1 | 1511 | 355 | 781 | 28.26 | 723.2 | 0 | 415/427 | 97 | 0/427 |
| ASV1521 | 404 | 1 | 1 | 404 | 100 | NR_044972.1 Sporobacter termitidis strain SYR 16S ribosomal RNA, partial sequence | NR_044972.1 | 1506 | 348 | 752 | 26.89 | 606.8 | 3E-173 | 379/405 | 93 | 1/405 |
| ASV1522 | 421 | 1 | 1 | 421 | 100 | NR_118334.1 Alloprevotella rava strain 81/4-12 16S ribosomal RNA, partial sequence | NR_118334.1 | 1455 | 338 | 759 | 29 | 579.1 | 6E-165 | 387/423 | 91 | 3/423 |
| ASV1523 | 402 | 1 | 1 | 402 | 100 | NR_118675.1 [Eubacterium] siraeum strain ATCC 29066 16S ribosomal RNA, partial sequence | NR_118675.1 | 1453 | 356 | 757 | 27.67 | 623.4 | 3E-178 | 379/402 | 94 | 0/402 |
| ASV1524 | 422 | 1 | 1 | 422 | 100 | NR_024816.1 Prevotella salivae strain EPSA11 16S ribosomal RNA, partial sequence | NR_024816.1 | 1493 | 356 | 777 | 28.27 | 603.1 | 3E-172 | 391/423 | 92 | 2/423 |
| ASV1525 | 403 | 1 | 1 | 403 | 100 | NR_104907.1 Eubacterium coprostanoligenes strain HL 16S ribosomal RNA, partial sequence | NR_104907.1 | 1446 | 332 | 734 | 27.87 | 656.7 | 0 | 387/403 | 96 | 0/403 |
| ASV1526 | 427 | 1 | 1 | 427 | 100 | NR_028840.1 Mitsuokella jalaludinii strain M9 16S ribosomal RNA, partial sequence | NR_028840.1 | 1543 | 362 | 788 | 27.67 | 706.5 | 0 | 414/429 | 96 | 4/429 |
| ASV1527 | 405 | 1 | 1 | 405 | 100 | NR_144742.1 Christensenella massiliensis strain Marseille-P2438 16S ribosomal RNA, partial sequence | NR_144742.1 | 1499 | 369 | 773 | 27.02 | 516.3 | 4E-146 | 364/406 | 89 | 2/406 |
| ASV1528 | 402 | 5 | 1 | 402 | 100 | NR_113027.1 Clostridium subterminale strain JCM 1417 16S ribosomal RNA, partial sequence | NR_113027.1 | 1470 | 346 | 747 | 27.35 | 677 | 0 | 390/402 | 97 | 0/402 |
| ASV1529 | 402 | 1 | 1 | 402 | 100 | NR_041882.1 Robinsoniella peoriensis strain PPC31 16S ribosomal RNA, partial sequence | NR_041882.1 | 1525 | 366 | 767 | 26.36 | 699.2 | 0 | 394/402 | 98 | 0/402 |
| ASV1530 | 404 | 2 | 1 | 404 | 100 | NR_074793.2 Oscillibacter valericigenes strain Sjm18-20 16S ribosomal RNA, complete sequence | NR_074793.2 | 1530 | 372 | 775 | 26.41 | 619.7 | 3E-177 | 381/404 | 94 | 0/404 |
| ASV1531 | 427 | 2 | 1 | 427 | 100 | NR_037018.1 Acidaminococcus fermentans strain VR4 16S ribosomal RNA gene, partial sequence | NR_037018.1 | 1516 | 349 | 775 | 28.17 | 784.1 | 0 | 426/427 | 99 | 0/427 |
| ASV1532 | 405 | 1 | 1 | 405 | 100 | NR_159227.1 Monoglobus pectinilyticus strain 14 16S ribosomal RNA, partial sequence | NR_159227.1 | 1493 | 368 | 769 | 26.93 | 562.5 | 6E-160 | 373/406 | 91 | 5/406 |
| ASV1533 | 422 | 2 | 1 | 422 | 100 | NR_113119.1 Prevotella oris strain JCM 8540 16S ribosomal RNA, partial sequence | NR_113119.1 | 1481 | 345 | 766 | 28.49 | 575.4 | 7E-164 | 387/424 | 91 | 4/424 |
| ASV1534 | 405 | 1 | 1 | 405 | 100 | NR_159227.1 Monoglobus pectinilyticus strain 14 16S ribosomal RNA, partial sequence | NR_159227.1 | 1493 | 368 | 769 | 26.93 | 512.6 | 6E-145 | 365/407 | 89 | 7/407 |
| ASV1535 | 405 | 1 | 1 | 405 | 100 | NR_042834.1 Alkalibacter saccharofermentans strain Z-79820 16S ribosomal RNA, partial sequence | NR_042834.1 | 1465 | 338 | 741 | 27.58 | 464.6 | 2E-130 | 355/406 | 87 | 3/406 |
| ASV1536 | 404 | 2 | 1 | 404 | 100 | NR_074793.2 Oscillibacter valericigenes strain Sjm18-20 16S ribosomal RNA, complete sequence | NR_074793.2 | 1530 | 372 | 775 | 26.41 | 636.4 | 0 | 384/404 | 95 | 0/404 |
| ASV1537 | 402 | 1 | 1 | 402 | 100 | NR_118692.1 Blautia obeum ATCC 29174 16S ribosomal RNA, partial sequence | NR_118692.1 | 1398 | 330 | 731 | 28.76 | 706.5 | 0 | 395/402 | 98 | 0/402 |
| ASV1539 | 405 | 2 | 3 | 405 | 99.51 | NG_041947.1 Ruminiclostridium cellulolyticum H10 16S ribosomal RNA gene, complete sequence | NG_041947.1 | 1641 | 488 | 890 | 24.56 | 501.6 | 1E-141 | 360/404 | 89 | 2/404 |
| ASV1540 | 421 | 1 | 1 | 421 | 100 | NR_144616.1 Muribaculum intestinale strain YL27 16S ribosomal RNA, partial sequence | NR_144616.1 | 1460 | 297 | 718 | 28.9 | 473.9 | 3E-133 | 372/427 | 87 | 11/427 |
| ASV1541 | 427 | 1 | 1 | 427 | 100 | NR_028862.1 Allisonella histaminiformans strain MR2 16S ribosomal RNA, partial sequence | NR_028862.1 | 1537 | 376 | 802 | 27.78 | 778.6 | 0 | 425/427 | 99 | 0/427 |
| ASV1542 | 427 | 1 | 1 | 427 | 100 | NR_104990.1 Desulfovibrio desulfuricans strain Essex 6 16S ribosomal RNA, partial sequence | NR_104990.1 | 1542 | 357 | 783 | 27.69 | 728.7 | 0 | 416/427 | 97 | 0/427 |
| ASV1543 | 404 | 1 | 3 | 404 | 99.5 | NR_144605.1 Acutalibacter muris strain KB18 16S ribosomal RNA, partial sequence | NR_144605.1 | 1510 | 387 | 785 | 26.42 | 424 | 3E-118 | 347/404 | 85 | 7/404 |
| ASV1545 | 421 | 1 | 1 | 421 | 100 | NR_144616.1 Muribaculum intestinale strain YL27 16S ribosomal RNA, partial sequence | NR_144616.1 | 1460 | 297 | 718 | 28.9 | 501.6 | 1E-141 | 372/422 | 88 | 1/422 |
| ASV1546 | 419 | 1 | 1 | 419 | 100 | NR_104844.1 Fibrobacter succinogenes subsp. elongatus strain HM2 16S ribosomal RNA, partial sequence | NR_104844.1 | 1431 | 319 | 737 | 29.28 | 630.8 | 2E-180 | 394/420 | 93 | 2/420 |
| ASV1547 | 422 | 1 | 1 | 422 | 100 | NR_041342.1 Parabacteroides distasonis strain JCM 5825 16S ribosomal RNA, partial sequence | NR_041342.1 | 1488 | 353 | 774 | 28.36 | 553.3 | 4E-157 | 382/423 | 90 | 2/423 |
| ASV1548 | 427 | 2 | 1 | 427 | 100 | NR_075036.1 Lactobacillus reuteri DSM 20016 16S ribosomal RNA, partial sequence | NR_075036.1 | 1569 | 384 | 810 | 27.21 | 778.6 | 0 | 425/427 | 99 | 0/427 |
| ASV1549 | 400 | 3 | 1 | 400 | 100 | NR_074575.1 Anaerococcus prevotii DSM 20548 16S ribosomal RNA, partial sequence | NR_074575.1 | 1515 | 364 | 763 | 26.4 | 739.8 | 0 | 400/400 | 100 | 0/400 |
| ASV1550 | 402 | 1 | 1 | 402 | 100 | NR_102987.1 Hungateiclostridium clariflavum DSM 19732 16S ribosomal RNA, partial sequence | NR_102987.1 | 1634 | 481 | 882 | 24.6 | 560.7 | 2E-159 | 371/404 | 91 | 4/404 |
| ASV1551 | 403 | 1 | 1 | 403 | 100 | NR_024829.1 Hungateiclostridium straminisolvens strain CSK1 16S ribosomal RNA, partial sequence | NR_024829.1 | 1447 | 338 | 742 | 27.99 | 558.8 | 7E-159 | 372/406 | 91 | 4/406 |
| ASV1553 | 403 | 1 | 3 | 403 | 99.5 | NR_125677.1 Vallitalea pronyensis strain FatNI3 16S ribosomal RNA, partial sequence | NR_125677.1 | 1480 | 365 | 764 | 27.03 | 479.4 | 6E-135 | 356/403 | 88 | 5/403 |
| ASV1554 | 405 | 1 | 1 | 405 | 100 | NR_025651.1 Anaerotignum lactatifermentans strain G17 16S ribosomal RNA, partial sequence | NR_025651.1 | 1497 | 367 | 771 | 27.05 | 743.5 | 0 | 404/405 | 99 | 0/405 |
| ASV1556 | 405 | 1 | 1 | 405 | 100 | NR_144742.1 Christensenella massiliensis strain Marseille-P2438 16S ribosomal RNA, partial sequence | NR_144742.1 | 1499 | 369 | 773 | 27.02 | 472 | 1E-132 | 356/406 | 87 | 2/406 |
| ASV1557 | 422 | 1 | 1 | 422 | 100 | NR_041446.1 Bacteroides barnesiae strain BL2 16S ribosomal RNA, partial sequence | NR_041446.1 | 1456 | 340 | 761 | 28.98 | 569.9 | 3E-162 | 386/424 | 91 | 4/424 |
| ASV1558 | 405 | 1 | 1 | 405 | 100 | NR_044972.1 Sporobacter termitidis strain SYR 16S ribosomal RNA, partial sequence | NR_044972.1 | 1506 | 348 | 752 | 26.89 | 547.7 | 2E-155 | 370/407 | 90 | 4/407 |
| ASV1559 | 427 | 1 | 1 | 426 | 99.77 | NR_027596.1 Mitsuokella multacida strain DSM 20544 16S ribosomal RNA, partial sequence | NR_027596.1 | 1511 | 346 | 771 | 28.19 | 651.1 | 0 | 401/426 | 94 | 0/426 |
| ASV1560 | 427 | 1 | 1 | 427 | 100 | NR_028840.1 Mitsuokella jalaludinii strain M9 16S ribosomal RNA, partial sequence | NR_028840.1 | 1543 | 362 | 788 | 27.67 | 784.1 | 0 | 426/427 | 99 | 0/427 |
| ASV1562 | 404 | 1 | 1 | 404 | 100 | NR_104907.1 Eubacterium coprostanoligenes strain HL 16S ribosomal RNA, partial sequence | NR_104907.1 | 1446 | 332 | 734 | 27.87 | 573.6 | 3E-163 | 373/404 | 92 | 1/404 |
| ASV1563 | 421 | 1 | 1 | 421 | 100 | NR_113097.1 Prevotella buccae ATCC 33574 strain JCM 12245 16S ribosomal RNA, partial sequence | NR_113097.1 | 1480 | 343 | 764 | 28.51 | 468.3 | 1E-131 | 373/429 | 86 | 15/429 |
| ASV1564 | 422 | 1 | 1 | 422 | 100 | NR_041364.1 Prevotella stercorea DSM 18206 strain CB35 16S ribosomal RNA, partial sequence | NR_041364.1 | 1488 | 354 | 775 | 28.36 | 575.4 | 7E-164 | 387/424 | 91 | 4/424 |
| ASV1565 | 405 | 1 | 1 | 405 | 100 | NR_144742.1 Christensenella massiliensis strain Marseille-P2438 16S ribosomal RNA, partial sequence | NR_144742.1 | 1499 | 369 | 773 | 27.02 | 499.7 | 4E-141 | 360/405 | 88 | 0/405 |
| ASV1566 | 405 | 2 | 3 | 405 | 99.51 | NR_113360.1 Ruminiclostridium cellobioparum DSM 1351 = ATCC 15832 strain JCM 1422 16S ribosomal RNA, partial sequence | NR_113360.1 | 1484 | 358 | 760 | 27.16 | 490.5 | 3E-138 | 357/403 | 88 | 0/403 |
| ASV1567 | 402 | 3 | 1 | 402 | 100 | NR_118128.1 [Clostridium] clostridioforme strain ATCC 25537 16S ribosomal RNA, partial sequence | NR_118128.1 | 1310 | 191 | 592 | 30.69 | 743.5 | 0 | 402/402 | 100 | 0/402 |

| | | | | | | | | | | | |
|---|---|---|---|---|---|---|---|---|---|---|---|
| ASV1306 | 0 | Plus | Plus | Bacteria; Proteobacteria; Deltaproteobacteria; Desulfovibrionales; Desulfovibrionaceae; Desulfovibrio; Desulfovibrio intestinalis | Desulfovibrio intestinalis | Desulfovibrio | Desulfovibrionaceae | Desulfovibrionales | Deltaproteobacteria | Proteobacteria | Bacteria |
| ASV1307 | 0 | Plus | Plus | Bacteria; Bacteroidetes; Bacteroidia; Bacteroidales; Muribaculaceae; Muribaculum; Muribaculum intestinale | Muribaculum intestinale | Muribaculum | Muribaculaceae | Bacteroidales | Bacteroidia | Bacteroidetes | Bacteria |
| ASV1308 | 0 | Plus | Plus | Bacteria; Bacteroidetes; Bacteroidia; Bacteroidales; Bacteroidaceae; Bacteroides; Bacteroides clarus | Bacteroides clarus | Bacteroides | Bacteroidaceae | Bacteroidales | Bacteroidia | Bacteroidetes | Bacteria |
| ASV1309 | 0 | Plus | Plus | Bacteria; Firmicutes; Clostridia; Eubacteriales; Clostridiales Family XIII. Incertae Sedis; Aminipila; Aminipila butyrica | Aminipila butyrica | Aminipila | Clostridiales Family XIII. Incertae Sedis | Eubacteriales | Clostridia | Firmicutes | Bacteria |
| ASV1310 | 0 | Plus | Plus | Bacteria; Bacteroidetes; Bacteroidia; Bacteroidales; Tannerellaceae; Parabacteroides; Parabacteroides goldsteinii | Parabacteroides goldsteinii | Parabacteroides | Tannerellaceae | Bacteroidales | Bacteroidia | Bacteroidetes | Bacteria |
| ASV1311 | 0 | Plus | Plus | Bacteria; Firmicutes; Clostridia; Eubacteriales; Lachnospiraceae; Roseburia; Roseburia hominis | Roseburia hominis | Roseburia | Lachnospiraceae | Eubacteriales | Clostridia | Firmicutes | Bacteria |
| ASV1312 | 1 | Plus | Plus | Bacteria; Bacteroidetes; Bacteroidia; Bacteroidales; Prevotellaceae; Prevotella; Prevotella ruminicola | Prevotella ruminicola | Prevotella | Prevotellaceae | Bacteroidales | Bacteroidia | Bacteroidetes | Bacteria |
| ASV1313 | 1 | Plus | Plus | Bacteria; Firmicutes; Clostridia; Eubacteriales; Oscillospiraceae; Monoglobus; Monoglobus pectinilyticus | Monoglobus pectinilyticus | Monoglobus | Oscillospiraceae | Eubacteriales | Clostridia | Firmicutes | Bacteria |
| ASV1314 | 0 | Plus | Plus | Bacteria; Firmicutes; Clostridia; Eubacteriales; Oscillospiraceae; Ruminococcus; Ruminococcus albus | Ruminococcus albus | Ruminococcus | Oscillospiraceae | Eubacteriales | Clostridia | Firmicutes | Bacteria |
| ASV1315 | 0 | Plus | Plus | Bacteria; Firmicutes; Clostridia; Eubacteriales; Eubacteriaceae; Eubacterium; Eubacterium coprostanoligenes | Eubacterium coprostanoligenes | Eubacterium | Eubacteriaceae | Eubacteriales | Clostridia | Firmicutes | Bacteria |
| ASV1316 | 0 | Plus | Plus | Bacteria; Actinobacteria; Actinomycetia; Actinomycetales; Actinomycetaceae; Trueperella; Trueperella pyogenes | Trueperella pyogenes | Trueperella | Actinomycetaceae | Actinomycetales | Actinomycetia | Actinobacteria | Bacteria |
| ASV1317 | 1 | Plus | Plus | Bacteria; Bacteroidetes; Bacteroidia; Bacteroidales; Paludibacteraceae; Paludibacter; Paludibacter jiangxiensis | Paludibacter jiangxiensis | Paludibacter | Paludibacteraceae | Bacteroidales | Bacteroidia | Bacteroidetes | Bacteria |
| ASV1318 | 1 | Plus | Plus | Bacteria; Bacteroidetes; Bacteroidia; Bacteroidales; Prevotellaceae; Prevotella; Prevotella buccae | Prevotella buccae | Prevotella | Prevotellaceae | Bacteroidales | Bacteroidia | Bacteroidetes | Bacteria |
| ASV1319 | 0 | Plus | Plus | Bacteria; Bacteroidetes; Bacteroidia; Bacteroidales; Rikenellaceae; Alistipes; Alistipes shahii | Alistipes shahii | Alistipes | Rikenellaceae | Bacteroidales | Bacteroidia | Bacteroidetes | Bacteria |
| ASV1320 | 0 | Plus | Plus | Bacteria; Bacteroidetes; Bacteroidia; Bacteroidales; Rikenellaceae; Alistipes; Alistipes shahii | Alistipes shahii | Alistipes | Rikenellaceae | Bacteroidales | Bacteroidia | Bacteroidetes | Bacteria |
| ASV1321 | 1 | Plus | Plus | Bacteria; Bacteroidetes; Bacteroidia; Bacteroidales; Prevotellaceae; Prevotellamassilia; Prevotellamassilia timonensis | Prevotellamassilia timonensis | Prevotellamassilia | Prevotellaceae | Bacteroidales | Bacteroidia | Bacteroidetes | Bacteria |
| ASV1322 | 0 | Plus | Plus | Bacteria; Firmicutes; Clostridia; Eubacteriales; Lachnospiraceae; Coprococcus; Coprococcus comes | Coprococcus comes | Coprococcus | Lachnospiraceae | Eubacteriales | Clostridia | Firmicutes | Bacteria |
| ASV1323 | 0 | Plus | Plus | Bacteria; Firmicutes; Clostridia; Eubacteriales; Oscillospiraceae; Sporobacter; Sporobacter termitidis | Sporobacter termitidis | Sporobacter | Oscillospiraceae | Eubacteriales | Clostridia | Firmicutes | Bacteria |
| ASV1324 | 1 | Plus | Plus | Bacteria; Bacteroidetes; Bacteroidia; Bacteroidales; Prevotellaceae; Prevotella; Prevotella loescheii | Prevotella loescheii | Prevotella | Prevotellaceae | Bacteroidales | Bacteroidia | Bacteroidetes | Bacteria |
| ASV1325 | 3 | Plus | Plus | Bacteria; Firmicutes; Clostridia; Eubacteriales; Clostridiaceae; Thermotalea; Thermotalea metallivorans | Thermotalea metallivorans | Thermotalea | Clostridiaceae | Eubacteriales | Clostridia | Firmicutes | Bacteria |
| ASV1326 | 0 | Plus | Plus | Bacteria; Bacteroidetes; Bacteroidia; Bacteroidales; Prevotellaceae; Prevotella; Prevotella oralis | Prevotella oralis | Prevotella | Prevotellaceae | Bacteroidales | Bacteroidia | Bacteroidetes | Bacteria |
| ASV1327 | 0 | Plus | Plus | Bacteria; Proteobacteria; Deltaproteobacteria; Desulfovibrionales; Desulfovibrionaceae; Desulfovibrio; Desulfovibrio intestinalis | Desulfovibrio intestinalis | Desulfovibrio | Desulfovibrionaceae | Desulfovibrionales | Deltaproteobacteria | Proteobacteria | Bacteria |
| ASV1328 | 0 | Plus | Plus | Bacteria; Firmicutes; Bacilli; Lactobacillales; Streptococcaceae; Streptococcus; Streptococcus porcorum | Streptococcus porcorum | Streptococcus | Streptococcaceae | Lactobacillales | Bacilli | Firmicutes | Bacteria |

| | | | | | | | | | | | | | | | |
|---|---|---|---|---|---|---|---|---|---|---|---|---|---|---|---|
| ASV1568 | 422 | 2 | 1 | 422 | 100 | NR_113119.1 Prevotella oris strain JCM 8540 16S ribosomal RNA, partial sequence | NR_113119.1 | 1481 | 345 | 766 | 28.49 | 614.2 | 2E-175 | 393/423 | 92 |
| ASV1569 | 427 | 1 | 1 | 426 | 99.77 | NR_027596.1 Mitsuokella multacida strain DSM 20544 16S ribosomal RNA, partial sequence | NR_027596.1 | 1511 | 346 | 771 | 28.19 | 684.4 | 0 | 407/426 | 95 |
| ASV1570 | 404 | 1 | 1 | 404 | 100 | NR_119085.1 [Clostridium] polysaccharolyticum strain DSM 1801 16S ribosomal RNA, partial sequence | NR_119085.1 | 1503 | 364 | 763 | 26.61 | 566.2 | 4E-161 | 374/406 | 92 |
| ASV1571 | 427 | 1 | 1 | 427 | 100 | NR_042896.1 Mucispirillum schaedleri strain HRI I17 16S ribosomal RNA, partial sequence | NR_042896.1 | 1471 | 333 | 759 | 29.03 | 723.2 | 0 | 415/427 | 97 |
| ASV1572 | 422 | 3 | 1 | 422 | 100 | NR_113094.1 Prevotella baroniae strain JCM 13447 16S ribosomal RNA, partial sequence | NR_113094.1 | 1494 | 358 | 779 | 28.25 | 592 | 7E-169 | 389/423 | 91 |
| ASV1573 | 403 | 1 | 3 | 403 | 99.5 | NR_125464.1 Anaerobacterium chartisolvens strain T-1-35 16S ribosomal RNA, partial sequence | NR_125464.1 | 1451 | 344 | 745 | 27.71 | 475.7 | 7E-134 | 354/402 | 88 |
| ASV1574 | 404 | 2 | 1 | 404 | 100 | NR_118156.1 Oscillibacter ruminantium GH1 16S ribosomal RNA, partial sequence | NR_118156.1 | 1409 | 321 | 724 | 28.67 | 470.2 | 3E-132 | 357/407 | 87 |
| ASV1575 | 412 | 1 | 1 | 412 | 100 | NR_026450.1 Ruminobacter amylophilus strain H 18 16S ribosomal RNA, complete sequence | NR_026450.1 | 1539 | 351 | 777 | 27.75 | 575.4 | 7E-164 | 393/429 | 91 |
| ASV1576 | 422 | 5 | 1 | 422 | 100 | NR_044864.1 Hallella seregens ATCC 51272 16S ribosomal RNA, partial sequence | NR_044864.1 | 1463 | 335 | 756 | 28.84 | 614.2 | 2E-175 | 393/423 | 92 |
| ASV1577 | 422 | 1 | 1 | 422 | 100 | NR_040877.1 Prevotella copri DSM 18205 strain CB7 16S ribosomal RNA, partial sequence | NR_040877.1 | 1494 | 358 | 779 | 28.25 | 721.3 | 0 | 411/422 | 97 |
| ASV1578 | 402 | 1 | 1 | 402 | 100 | NR_118675.1 [Eubacterium] siraeum strain ATCC 29066 16S ribosomal RNA, partial sequence | NR_118675.1 | 1453 | 356 | 757 | 27.67 | 617.9 | 1E-176 | 378/402 | 94 |
| ASV1579 | 422 | 1 | 1 | 422 | 100 | NR_113411.1 Prevotella copri DSM 18205 strain JCM 13464 16S ribosomal RNA, partial sequence | NR_113411.1 | 1493 | 358 | 779 | 28.27 | 725 | 0 | 412/422 | 97 |
| ASV1580 | 402 | 1 | 1 | 402 | 100 | NR_109014.1 Blautia faecis strain M25 16S ribosomal RNA, partial sequence | NR_109014.1 | 1262 | 212 | 613 | 31.85 | 699.2 | 0 | 394/402 | 98 |
| ASV1582 | 402 | 1 | 1 | 402 | 100 | NR_147375.1 Colidextribacter massiliensis strain Marseille-P3083 16S ribosomal RNA, partial sequence | NR_147375.1 | 1487 | 364 | 765 | 27.03 | 483.1 | 4E-136 | 356/403 | 88 |
| ASV1583 | 402 | 1 | 1 | 402 | 100 | NR_074613.1 [Eubacterium] eligens ATCC 27750 16S ribosomal RNA, partial sequence | NR_074613.1 | 1519 | 367 | 768 | 26.46 | 605 | 9E-173 | 377/402 | 93 |
| ASV1584 | 427 | 1 | 1 | 427 | 100 | NR_042797.1 Treponema berlinense strain 7CPL208 16S ribosomal RNA, partial sequence | NR_042797.1 | 1440 | 275 | 701 | 29.65 | 706.5 | 0 | 412/427 | 96 |
| ASV1585 | 403 | 2 | 1 | 403 | 100 | NR_042200.1 [Clostridium] asparagiforme strain N6 16S ribosomal RNA, partial sequence | NR_042200.1 | 1468 | 335 | 736 | 27.38 | 677 | 0 | 391/403 | 97 |
| ASV1586 | 420 | 1 | 1 | 420 | 100 | NR_144616.1 Muribaculum intestinale strain YL27 16S ribosomal RNA, partial sequence | NR_144616.1 | 1460 | 297 | 718 | 28.9 | 579.1 | 6E-165 | 386/422 | 91 |
| ASV1587 | 402 | 1 | 1 | 402 | 100 | NR_025931.1 Ruminococcus flavefaciens strain C94 16S ribosomal RNA, partial sequence | NR_025931.1 | 1388 | 325 | 726 | 28.96 | 632.7 | 0 | 382/402 | 95 |
| ASV1588 | 403 | 1 | 1 | 403 | 100 | NR_104907.1 Eubacterium coprostanoligenes strain HL 16S ribosomal RNA, partial sequence | NR_104907.1 | 1446 | 332 | 734 | 27.87 | 651.1 | 0 | 386/403 | 95 |
| ASV1589 | 427 | 1 | 1 | 427 | 100 | NR_042797.1 Treponema berlinense strain 7CPL208 16S ribosomal RNA, partial sequence | NR_042797.1 | 1440 | 275 | 701 | 29.65 | 695.5 | 0 | 410/427 | 96 |
| ASV1590 | 405 | 2 | 1 | 405 | 100 | NR_074114.1 Elusimicrobium minutum strain Pei191 16S ribosomal RNA, partial sequence | NR_074114.1 | 1433 | 356 | 760 | 28.26 | 654.8 | 0 | 388/405 | 95 |
| ASV1591 | 428 | 4 | 1 | 427 | 99.77 | NR_156976.1 Desulfosporosinus fructosivorans strain 63.6F 16S ribosomal RNA, partial sequence | NR_156976.1 | 1529 | 350 | 776 | 27.93 | 496 | 6E-140 | 377/430 | 87 |
| ASV1592 | 401 | 1 | 1 | 401 | 100 | NR_158111.1 Paludicola psychrotolerans strain NC1253 16S ribosomal RNA, partial sequence | NR_158111.1 | 1490 | 366 | 766 | 26.91 | 647.4 | 0 | 384/401 | 95 |
| ASV1593 | 402 | 2 | 1 | 402 | 100 | NR_042144.1 Clostridium butyricum strain VPI3266 16S ribosomal RNA, partial sequence | NR_042144.1 | 1425 | 338 | 739 | 28.21 | 737.9 | 0 | 401/402 | 99 |
| ASV1594 | 405 | 1 | 1 | 405 | 100 | NR_042834.1 Alkalibacter saccharofermentans strain Z-79820 16S ribosomal RNA, partial sequence | NR_042834.1 | 1465 | 338 | 741 | 27.58 | 470.2 | 3E-132 | 356/406 | 87 |
| ASV1595 | 422 | 2 | 1 | 422 | 100 | NR_113119.1 Prevotella oris strain JCM 8540 16S ribosomal RNA, partial sequence | NR_113119.1 | 1481 | 345 | 766 | 28.49 | 581 | 2E-165 | 387/423 | 91 |
| ASV1596 | 422 | 1 | 1 | 422 | 100 | NR_113411.1 Prevotella copri DSM 18205 strain JCM 13464 16S ribosomal RNA, partial sequence | NR_113411.1 | 1493 | 358 | 779 | 28.27 | 603.1 | 3E-172 | 390/422 | 92 |
| ASV1597 | 404 | 1 | 1 | 404 | 100 | NR_115340.1 Saccharofermentans acetigenes strain P6 16S ribosomal RNA, partial sequence | NR_115340.1 | 1510 | 353 | 757 | 26.82 | 431.4 | 2E-120 | 350/407 | 85 |
| ASV1598 | 422 | 3 | 1 | 422 | 100 | NR_113109.1 Prevotella loescheii strain JCM 8530 16S ribosomal RNA, partial sequence | NR_113109.1 | 1476 | 342 | 763 | 28.59 | 547.7 | 2E-155 | 382/424 | 90 |
| ASV1599 | 427 | 1 | 1 | 427 | 100 | NR_027596.1 Mitsuokella multacida strain DSM 20544 16S ribosomal RNA, partial sequence | NR_027596.1 | 1511 | 346 | 772 | 28.26 | 785.9 | 0 | 426/427 | 99 |
| ASV1600 | 405 | 1 | 1 | 405 | 100 | NR_144611.1 Flintibacter butyricus strain BLS21 16S ribosomal RNA, partial sequence | NR_144611.1 | 808 | 299 | 703 | 50.12 | 599.4 | 4E-171 | 378/405 | 93 |
| ASV1601 | 418 | 1 | 1 | 418 | 100 | NR_102893.1 Butyrivibrio proteoclasticus strain B316 16S ribosomal RNA, partial sequence | NR_102893.1 | 1537 | 367 | 782 | 27.07 | 588.4 | 1E-167 | 385/418 | 92 |
| ASV1602 | 402 | 1 | 1 | 402 | 100 | NR_028961.1 Faecalibacterium prausnitzii strain ATCC 27768 16S ribosomal RNA, partial sequence | NR_028961.1 | 1462 | 344 | 745 | 27.5 | 732.4 | 0 | 400/402 | 99 |
| ASV1603 | 422 | 1 | 3 | 422 | 99.53 | NR_044119.1 Parapedobacter soli strain DCY14 16S ribosomal RNA, partial sequence | NR_044119.1 | 1468 | 345 | 764 | 28.61 | 427.7 | 2E-119 | 358/421 | 85 |
| ASV1604 | 422 | 1 | 1 | 422 | 100 | NR_113411.1 Prevotella copri DSM 18205 strain JCM 13464 16S ribosomal RNA, partial sequence | NR_113411.1 | 1493 | 358 | 779 | 28.27 | 647.4 | 0 | 398/422 | 94 |
| ASV1605 | 405 | 1 | 1 | 405 | 100 | NR_044972.1 Sporobacter termitidis strain SYR 16S ribosomal RNA, partial sequence | NR_044972.1 | 1506 | 348 | 752 | 26.89 | 547.7 | 2E-155 | 369/406 | 90 |
| ASV1606 | 404 | 1 | 1 | 404 | 100 | NR_145666.1 Rarimicrobium hominis strain ADV70 16S ribosomal RNA, partial sequence | NR_145666.1 | 1392 | 298 | 701 | 29.02 | 525.6 | 7E-149 | 366/406 | 90 |
| ASV1607 | 402 | 1 | 1 | 402 | 100 | NR_147396.1 Lachnoclostridium pacaense strain Marseille-P3100 16S ribosomal RNA, partial sequence | NR_147396.1 | 1453 | 345 | 746 | 27.67 | 677 | 0 | 390/402 | 97 |
| ASV1608 | 405 | 1 | 1 | 405 | 100 | NR_159227.1 Monoglobus pectinilyticus strain 14 16S ribosomal RNA, partial sequence | NR_159227.1 | 1493 | 368 | 769 | 26.93 | 518.2 | 1E-146 | 366/407 | 89 |
| ASV1609 | 422 | 1 | 1 | 422 | 100 | NR_041364.1 Prevotella stercorea DSM 18206 strain CB35 16S ribosomal RNA, partial sequence | NR_041364.1 | 1488 | 354 | 775 | 28.36 | 713.9 | 0 | 410/422 | 97 |
| ASV1610 | 427 | 1 | 1 | 427 | 100 | NR_104781.1 Treponema bryantii strain RUS-1 16S ribosomal RNA, partial sequence | NR_104781.1 | 1535 | 351 | 778 | 27.88 | 562.5 | 6E-160 | 387/428 | 90 |
| ASV1611 | 428 | 1 | 1 | 428 | 100 | NR_104781.1 Treponema bryantii strain RUS-1 16S ribosomal RNA, partial sequence | NR_104781.1 | 1535 | 351 | 778 | 27.88 | 664.1 | 0 | 405/428 | 94 |
| ASV1612 | 422 | 2 | 1 | 422 | 100 | NR_074577.1 Paludibacter propionicigenes WB4 16S ribosomal RNA, partial sequence | NR_074577.1 | 1516 | 353 | 774 | 27.84 | 486.8 | 4E-137 | 372/425 | 87 |
| ASV1613 | 403 | 1 | 1 | 403 | 100 | NR_025930.1 Ruminococcus bromii strain ATCC 27255 16S ribosomal RNA, partial sequence | NR_025930.1 | 1380 | 313 | 716 | 29.28 | 723.2 | 0 | 400/404 | 99 |
| ASV1614 | 402 | 1 | 1 | 402 | 100 | NR_029097.1 Oribacterium sinus strain AIP 354.02 16S ribosomal RNA, partial sequence | NR_029097.1 | 1374 | 308 | 709 | 29.26 | 649.3 | 0 | 385/402 | 95 |
| ASV1615 | 427 | 2 | 1 | 427 | 100 | NR_029106.1 Lactobacillus delbrueckii subsp. indicus strain NCC725 16S ribosomal RNA, partial sequence | NR_029106.1 | 1515 | 348 | 774 | 28.18 | 773 | 0 | 424/427 | 99 |
| ASV1616 | 421 | 1 | 1 | 421 | 100 | NR_144616.1 Muribaculum intestinale strain YL27 16S ribosomal RNA, partial sequence | NR_144616.1 | 1460 | 297 | 718 | 28.9 | 468.3 | 1E-131 | 367/423 | 86 |
| ASV1617 | 427 | 2 | 1 | 427 | 100 | NR_025516.1 Streptococcus ratti strain ATCC 19645 16S ribosomal RNA, partial sequence | NR_025516.1 | 1444 | 347 | 773 | 29.57 | 739.8 | 0 | 418/427 | 97 |
| ASV1618 | 402 | 1 | 1 | 402 | 100 | NR_104735.1 Butyrivibrio crossotus strain DSM 2876 16S ribosomal RNA, partial sequence | NR_104735.1 | 1511 | 353 | 754 | 26.6 | 682.5 | 0 | 391/402 | 97 |
| ASV1619 | 402 | 1 | 1 | 402 | 100 | NR_151894.1 Anaerotaenia torta strain FH052 16S ribosomal RNA, partial sequence | NR_151894.1 | 1479 | 352 | 753 | 27.18 | 643.8 | 0 | 384/402 | 95 |
| ASV1620 | 427 | 2 | 1 | 426 | 99.77 | NR_027596.1 Mitsuokella multacida strain DSM 20544 16S ribosomal RNA, partial sequence | NR_027596.1 | 1511 | 346 | 771 | 28.19 | 651.1 | 0 | 402/427 | 94 |
| ASV1621 | 422 | 2 | 1 | 422 | 100 | NR_113117.1 Prevotella oralis ATCC 33269 strain JCM 12251 16S ribosomal RNA, partial sequence | NR_113117.1 | 1496 | 361 | 782 | 28.21 | 542.2 | 8E-154 | 381/424 | 89 |
| ASV1622 | 405 | 1 | 1 | 405 | 100 | NR_144611.1 Flintibacter butyricus strain BLS21 16S ribosomal RNA, partial sequence | NR_144611.1 | 808 | 299 | 703 | 50.12 | 593.9 | 2E-169 | 377/405 | 93 |
| ASV1623 | 405 | 1 | 1 | 405 | 100 | NR_115340.1 Saccharofermentans acetigenes strain P6 16S ribosomal RNA, partial sequence | NR_115340.1 | 1510 | 353 | 757 | 26.82 | 499.7 | 4E-141 | 361/406 | 88 |
| ASV1624 | 402 | 2 | 1 | 402 | 100 | NR_026074.1 Helicobacter rodentium strain MIT 95-1707 16S ribosomal RNA, partial sequence | NR_026074.1 | 1423 | 317 | 718 | 28.25 | 699.2 | 0 | 394/402 | 98 |
| ASV1625 | 421 | 2 | 1 | 421 | 100 | NR_113073.1 Barnesiella intestinihominis strain JCM 15079 16S ribosomal RNA, partial sequence | NR_113073.1 | 1481 | 346 | 767 | 28.49 | 490.5 | 3E-138 | 371/423 | 87 |
| ASV1626 | 424 | 1 | 1 | 424 | 100 | NR_118334.1 Alloprevotella rava strain 81/4-12 16S ribosomal RNA, partial sequence | NR_118334.1 | 1455 | 338 | 759 | 29 | 477.6 | 2E-134 | 372/427 | 87 |
| ASV1627 | 405 | 1 | 1 | 405 | 100 | NR_159227.1 Monoglobus pectinilyticus strain 14 16S ribosomal RNA, partial sequence | NR_159227.1 | 1493 | 368 | 769 | 26.93 | 540.3 | 3E-153 | 368/405 | 90 |
| ASV1628 | 427 | 1 | 1 | 427 | 100 | NR_157643.1 Treponema rectale strain CHPA 16S ribosomal RNA, partial sequence | NR_157643.1 | 1309 | 259 | 685 | 32.62 | 579.1 | 6E-165 | 392/430 | 91 |
| ASV1629 | 427 | 1 | 4 | 427 | 99.3 | NR_146687.1 Breznakia pachnodae strain Pei061 16S ribosomal RNA, partial sequence | NR_146687.1 | 1499 | 370 | 792 | 28.22 | 455.4 | 1E-127 | 367/426 | 86 |
| ASV1630 | 404 | 3 | 1 | 404 | 100 | NG_041947.1 Ruminiclostridium cellulolyticum H10 16S ribosomal RNA gene, complete sequence | NG_041947.1 | 1641 | 486 | 890 | 24.68 | 581 | 2E-165 | 375/405 | 92 |
| ASV1631 | 428 | 1 | 1 | 428 | 100 | NR_104781.1 Treponema bryantii strain RUS-1 16S ribosomal RNA, partial sequence | NR_104781.1 | 1535 | 351 | 778 | 27.88 | 558.8 | 8E-159 | 390/432 | 90 |
| ASV1632 | 422 | 1 | 1 | 422 | 100 | NR_144616.1 Muribaculum intestinale strain YL27 16S ribosomal RNA, partial sequence | NR_144616.1 | 1460 | 297 | 718 | 28.9 | 569.9 | 3E-162 | 388/426 | 91 |
| ASV1633 | 422 | 3 | 1 | 422 | 100 | NR_113109.1 Prevotella loescheii strain JCM 8530 16S ribosomal RNA, partial sequence | NR_113109.1 | 1476 | 342 | 763 | 28.59 | 603.1 | 3E-172 | 392/424 | 92 |
| ASV1635 | 427 | 2 | 1 | 426 | 99.77 | NR_027596.1 Mitsuokella multacida strain DSM 20544 16S ribosomal RNA, partial sequence | NR_027596.1 | 1511 | 346 | 771 | 28.19 | 662.2 | 0 | 404/427 | 94 |
| ASV1636 | 403 | 1 | 3 | 403 | 99.5 | NR_125677.1 Vallitalea pronyensis strain FatNI3 16S ribosomal RNA, partial sequence | NR_125677.1 | 1480 | 365 | 764 | 27.03 | 435.1 | 1E-121 | 347/402 | 86 |
| ASV1638 | 422 | 2 | 1 | 422 | 100 | NR_113119.1 Prevotella oris strain JCM 8540 16S ribosomal RNA, partial sequence | NR_113119.1 | 1481 | 345 | 766 | 28.49 | 597.6 | 2E-170 | 391/424 | 92 |
| ASV1639 | 422 | 4 | 1 | 422 | 100 | NR_044864.1 Hallella seregens ATCC 51272 16S ribosomal RNA, partial sequence | NR_044864.1 | 1463 | 335 | 756 | 28.84 | 608.7 | 7E-174 | 391/422 | 92 |
| ASV1640 | 421 | 2 | 1 | 421 | 100 | NR_113073.1 Barnesiella intestinihominis strain JCM 15079 16S ribosomal RNA, partial sequence | NR_113073.1 | 1481 | 346 | 767 | 28.49 | 523.7 | 3E-148 | 378/424 | 89 |
| ASV1641 | 424 | 2 | 1 | 424 | 100 | NR_144750.1 Prevotellamassilia timonensis strain Marseille-P2831 16S ribosomal RNA, partial sequence | NR_144750.1 | 1493 | 358 | 779 | 28.27 | 477.6 | 2E-134 | 371/426 | 87 |
| ASV1642 | 401 | 1 | 1 | 401 | 100 | NR_158111.1 Paludicola psychrotolerans strain NC1253 16S ribosomal RNA, partial sequence | NR_158111.1 | 1490 | 366 | 766 | 26.91 | 653 | 0 | 385/401 | 96 |
| ASV1643 | 423 | 1 | 1 | 423 | 100 | NR_144750.1 Prevotellamassilia timonensis strain Marseille-P2831 16S ribosomal RNA, partial sequence | NR_144750.1 | 1493 | 358 | 779 | 28.27 | 569.9 | 3E-162 | 387/425 | 91 |
| ASV1644 | 427 | 1 | 1 | 427 | 100 | NR_025131.1 Treponema parvum strain OMZ 833 16S ribosomal RNA, partial sequence | NR_025131.1 | 1485 | 341 | 767 | 28.75 | 601.3 | 1E-171 | 396/430 | 92 |
| ASV1645 | 405 | 1 | 1 | 405 | 100 | NR_118554.1 Intestinimonas butyriciproducens strain SRB-521-5-I 16S ribosomal RNA, partial sequence | NR_118554.1 | 1475 | 346 | 750 | 27.46 | 483.1 | 4E-136 | 358/406 | 88 |
| ASV1646 | 403 | 1 | 1 | 403 | 100 | NR_102884.1 Ruminococcus champanellensis 18P13 = JCM 17042 16S ribosomal RNA, partial sequence | NR_102884.1 | 1494 | 348 | 749 | 26.91 | 632.7 | 0 | 383/403 | 95 |
| ASV1647 | 422 | 2 | 1 | 422 | 100 | NR_109309.1 Mangroviflexus xiamenensis strain P2 16S ribosomal RNA, partial sequence | NR_109309.1 | 1440 | 326 | 747 | 29.31 | 448 | 2E-125 | 364/424 | 85 |
| ASV1648 | 403 | 1 | 3 | 403 | 99.5 | NR_026104.1 Ruminiclostridium cellobioparum DSM 1351 = ATCC 15832 16S ribosomal RNA, partial sequence | NR_026104.1 | 1487 | 331 | 733 | 27.1 | 466.5 | 4E-131 | 353/403 | 87 |
| ASV1649 | 427 | 2 | 1 | 427 | 100 | NR_118848.1 Actinobacillus indolicus strain 46KC2 16S ribosomal RNA, partial sequence | NR_118848.1 | 1464 | 333 | 759 | 29.17 | 745.3 | 0 | 419/427 | 98 |
| ASV1651 | 405 | 1 | 1 | 405 | 100 | NR_112900.1 Christensenella minuta strain YIT 12065 16S ribosomal RNA, partial sequence | NR_112900.1 | 1497 | 358 | 762 | 27.05 | 588.4 | 9E-168 | 376/405 | 92 |
| ASV1652 | 404 | 2 | 3 | 404 | 99.5 | NG_041947.1 Ruminiclostridium cellulolyticum H10 16S ribosomal RNA gene, complete sequence | NG_041947.1 | 1641 | 488 | 890 | 24.56 | 460.9 | 2E-129 | 352/403 | 87 |
| ASV1653 | 402 | 3 | 1 | 402 | 100 | NR_115549.1 Rhodospirillum rubrum ATCC 11170 16S ribosomal RNA, partial sequence | NR_115549.1 | 1406 | 299 | 700 | 28.59 | 438.8 | 1E-122 | 349/404 | 86 |
| ASV1654 | 402 | 1 | 1 | 402 | 100 | NR_104846.1 Gemmiger formicilis strain X2-56 16S ribosomal RNA, partial sequence | NR_104846.1 | 1426 | 305 | 706 | 28.19 | 704.7 | 0 | 395/402 | 98 |
| ASV1655 | 422 | 1 | 1 | 422 | 100 | NR_040877.1 Prevotella copri DSM 18205 strain CB7 16S ribosomal RNA, partial sequence | NR_040877.1 | 1494 | 358 | 779 | 28.25 | 760.1 | 0 | 418/422 | 99 |
| ASV1656 | 405 | 1 | 1 | 405 | 100 | NR_025025.1 Papillibacter cinnamivorans strain CIN1 16S ribosomal RNA, partial sequence | NR_025025.1 | 1491 | 349 | 751 | 27.03 | 581 | 2E-165 | 375/406 | 92 |
| ASV1657 | 403 | 1 | 3 | 403 | 99.5 | NR_102987.1 Hungateiclostridium clariflavum DSM 19732 16S ribosomal RNA, partial sequence | NR_102987.1 | 1634 | 483 | 882 | 24.48 | 468.3 | 1E-131 | 352/401 | 87 |
| ASV1658 | 427 | 1 | 1 | 427 | 100 | NR_147381.1 Duodenibacillus massiliensis strain Marseille-P2968 16S ribosomal RNA, partial sequence | NR_147381.1 | 1497 | 349 | 775 | 28.52 | 678.8 | 0 | 410/430 | 95 |
| ASV1659 | 402 | 1 | 1 | 402 | 100 | NR_152688.1 Falcatimonas natans strain WN011 16S ribosomal RNA, partial sequence | NR_152688.1 | 1484 | 365 | 766 | 27.09 | 638.2 | 0 | 383/402 | 95 |
| ASV1661 | 402 | 1 | 1 | 402 | 100 | NR_104846.1 Gemmiger formicilis strain X2-56 16S ribosomal RNA, partial sequence | NR_104846.1 | 1426 | 305 | 706 | 28.19 | 737.9 | 0 | 401/402 | 99 |
| ASV1662 | 427 | 2 | 1 | 427 | 100 | NR_037018.1 Acidaminococcus fermentans strain VR4 16S ribosomal RNA gene, partial sequence | NR_037018.1 | 1516 | 349 | 775 | 28.17 | 745.3 | 0 | 419/427 | 98 |
| ASV1663 | 422 | 2 | 1 | 422 | 100 | NR_113117.1 Prevotella oralis ATCC 33269 strain JCM 12251 16S ribosomal RNA, partial sequence | NR_113117.1 | 1496 | 361 | 782 | 28.21 | 603.1 | 3E-172 | 391/423 | 92 |
| ASV1664 | 422 | 2 | 1 | 422 | 100 | NR_113119.1 Prevotella oris strain JCM 8540 16S ribosomal RNA, partial sequence | NR_113119.1 | 1481 | 345 | 766 | 28.49 | 625.3 | 7E-179 | 395/423 | 93 |
| ASV1665 | 422 | 1 | 1 | 422 | 100 | NR_113411.1 Prevotella copri DSM 18205 strain JCM 13464 16S ribosomal RNA, partial sequence | NR_113411.1 | 1493 | 358 | 779 | 28.27 | 597.6 | 2E-170 | 390/423 | 92 |
| ASV1666 | 403 | 1 | 3 | 403 | 99.5 | NR_125464.1 Anaerobacterium chartisolvens strain T-1-35 16S ribosomal RNA, partial sequence | NR_125464.1 | 1451 | 344 | 745 | 27.71 | 481.2 | 2E-135 | 355/402 | 88 |
| ASV1667 | 422 | 2 | 1 | 422 | 100 | NR_113119.1 Prevotella oris strain JCM 8540 16S ribosomal RNA, partial sequence | NR_113119.1 | 1481 | 345 | 766 | 28.49 | 581 | 2E-165 | 387/423 | 91 |
| ASV1668 | 403 | 1 | 3 | 403 | 99.5 | NR_125677.1 Vallitalea pronyensis strain FatNI3 16S ribosomal RNA, partial sequence | NR_125677.1 | 1480 | 365 | 764 | 27.03 | 484.9 | 1E-136 | 357/403 | 88 |
| ASV1669 | 401 | 1 | 1 | 401 | 100 | NR_027228.1 Helcococcus ovis strain s840-96-2 16S ribosomal RNA, partial sequence | NR_027228.1 | 1423 | 353 | 753 | 28.18 | 730.5 | 0 | 399/401 | 99 |
| ASV1670 | 405 | 1 | 1 | 405 | 100 | NR_144742.1 Christensenella massiliensis strain Marseille-P2438 16S ribosomal RNA, partial sequence | NR_144742.1 | 1499 | 369 | 773 | 27.02 | 449.9 | 5E-126 | 352/406 | 86 |
| ASV1671 | 420 | 1 | 1 | 420 | 100 | NR_119166.1 Parabacteroides merdae ATCC 43184 16S ribosomal RNA, partial sequence | NR_119166.1 | 1405 | 336 | 757 | 30.04 | 459.1 | 8E-129 | 365/423 | 86 |
| ASV1672 | 421 | 1 | 1 | 421 | 100 | NR_144616.1 Muribaculum intestinale strain YL27 16S ribosomal RNA, partial sequence | NR_144616.1 | 1460 | 297 | 718 | 28.9 | 468.3 | 1E-131 | 371/427 | 86 |
| ASV1673 | 405 | 1 | 1 | 405 | 100 | NR_118554.1 Intestinimonas butyriciproducens strain SRB-521-5-I 16S ribosomal RNA, partial sequence | NR_118554.1 | 1475 | 346 | 750 | 27.46 | 477.6 | 2E-134 | 357/406 | 87 |
| ASV1674 | 427 | 1 | 1 | 427 | 100 | NR_157643.1 Treponema rectale strain CHPA 16S ribosomal RNA, partial sequence | NR_157643.1 | 1309 | 259 | 685 | 32.62 | 584.7 | 1E-166 | 393/430 | 91 |
| ASV1675 | 403 | 1 | 1 | 403 | 100 | NR_025930.1 Ruminococcus bromii strain ATCC 27255 16S ribosomal RNA, partial sequence | NR_025930.1 | 1380 | 313 | 716 | 29.28 | 728.7 | 0 | 401/404 | 99 |
| ASV1676 | 403 | 1 | 1 | 403 | 100 | NR_104907.1 Eubacterium coprostanoligenes strain HL 16S ribosomal RNA, partial sequence | NR_104907.1 | 1446 | 332 | 734 | 27.87 | 701 | 0 | 395/403 | 98 |
| ASV1677 | 402 | 2 | 1 | 402 | 100 | NR_042144.1 Clostridium butyricum strain VPI3266 16S ribosomal RNA, partial sequence | NR_042144.1 | 1425 | 338 | 739 | 28.21 | 726.9 | 0 | 399/402 | 99 |
| ASV1678 | 403 | 1 | 1 | 403 | 100 | NR_042930.1 Acetanaerobacterium elongatum strain Z7 16S ribosomal RNA, partial sequence | NR_042930.1 | 1533 | 383 | 783 | 26.16 | 560.7 | 2E-159 | 370/403 | 91 |
| ASV1679 | 402 | 1 | 1 | 402 | 100 | NR_026493.1 [Clostridium] indolis strain 7 16S ribosomal RNA, partial sequence | NR_026493.1 | 1488 | 355 | 756 | 27.02 | 682.5 | 0 | 391/402 | 97 |
| ASV1680 | 427 | 1 | 1 | 427 | 100 | NR_041778.1 Desulfovibrio piger strain ATCC 29098 16S ribosomal RNA, partial sequence | NR_041778.1 | 1542 | 357 | 783 | 27.69 | 773 | 0 | 424/427 | 99 |
| ASV1681 | 402 | 1 | 1 | 402 | 100 | NR_029239.3 Clostridium chartatabidum strain 163 16S ribosomal RNA, partial sequence | NR_029239.3 | 1460 | 323 | 724 | 27.53 | 704.7 | 0 | 395/402 | 98 |
| ASV1682 | 404 | 1 | 1 | 404 | 100 | NR_042832.1 Roseburia faecis strain M72/1 16S ribosomal RNA, partial sequence | NR_042832.1 | 1456 | 347 | 749 | 27.68 | 734.2 | 0 | 402/404 | 99 |
| ASV1683 | 401 | 1 | 1 | 401 | 100 | NR_159296.1 Aminipila butyrica strain FH042 16S ribosomal RNA, partial sequence | NR_159296.1 | 1460 | 352 | 755 | 27.67 | 610.5 | 2E-174 | 380/404 | 94 |
| ASV1684 | 427 | 1 | 1 | 427 | 100 | NR_027596.1 Mitsuokella multacida strain DSM 20544 16S ribosomal RNA, partial sequence | NR_027596.1 | 1511 | 346 | 772 | 28.26 | 780.4 | 0 | 425/427 | 99 |
| ASV1685 | 403 | 2 | 1 | 403 | 100 | NR_147398.1 Anaerotruncus rubiinfantis strain MT15 16S ribosomal RNA, partial sequence | NR_147398.1 | 1490 | 368 | 768 | 26.91 | 549.6 | 4E-156 | 369/404 | 91 |
| ASV1686 | 405 | 1 | 1 | 405 | 100 | NR_133738.1 Desulfohalotomaculum tongense strain TGB60-1 16S ribosomal RNA, partial sequence | NR_133738.1 | 1447 | 352 | 756 | 27.99 | 466.5 | 4E-131 | 355/406 | 87 |
| ASV1687 | 427 | 1 | 1 | 426 | 99.77 | NR_028840.1 Mitsuokella jalaludinii strain M9 16S ribosomal RNA, partial sequence | NR_028840.1 | 1543 | 362 | 787 | 27.61 | 677 | 0 | 406/426 | 95 |
| ASV1688 | 403 | 1 | 3 | 403 | 99.5 | NR_125464.1 Anaerobacterium chartisolvens strain T-1-35 16S ribosomal RNA, partial sequence | NR_125464.1 | 1451 | 344 | 745 | 27.71 | 481.2 | 2E-135 | 355/402 | 88 |
| ASV1689 | 405 | 1 | 1 | 405 | 100 | NR_118554.1 Intestinimonas butyriciproducens strain SRB-521-5-I 16S ribosomal RNA, partial sequence | NR_118554.1 | 1475 | 346 | 750 | 27.46 | 538.5 | 9E-153 | 368/406 | 90 |
| ASV1690 | 422 | 2 | 1 | 422 | 100 | NR_040864.1 Bacteroides eggerthii strain DSM 20697 16S ribosomal RNA, partial sequence | NR_040864.1 | 1425 | 353 | 774 | 29.61 | 769.3 | 0 | 420/422 | 99 |
| ASV1691 | 422 | 2 | 1 | 422 | 100 | NR_113070.1 Bacteroides oleiciplenus YIT 12058 strain JCM 16102 16S ribosomal RNA, partial sequence | NR_113070.1 | 1485 | 351 | 772 | 28.42 | 658.5 | 0 | 400/422 | 94 |
| ASV1692 | 402 | 1 | 1 | 402 | 100 | NR_118676.1 Eubacterium xylanophilum strain ATCC 35991 16S ribosomal RNA, partial sequence | NR_118676.1 | 1494 | 387 | 788 | 26.91 | 669.6 | 0 | 387/402 | 96 |
| ASV1694 | 405 | 1 | 1 | 405 | 100 | NR_145929.1 Caproiciproducens galactitolivorans strain BS-1 16S ribosomal RNA, partial sequence | NR_145929.1 | 1445 | 336 | 741 | 28.1 | 521.9 | 9E-148 | 366/407 | 89 |
| ASV1695 | 403 | 1 | 3 | 403 | 99.5 | NR_125677.1 Vallitalea pronyensis strain FatNI3 16S ribosomal RNA, partial sequence | NR_125677.1 | 1480 | 365 | 764 | 27.03 | 479.4 | 6E-135 | 356/403 | 88 |
| ASV1696 | 427 | 1 | 1 | 426 | 99.77 | NR_027596.1 Mitsuokella multacida strain DSM 20544 16S ribosomal RNA, partial sequence | NR_027596.1 | 1511 | 346 | 771 | 28.19 | 640.1 | 0 | 399/426 | 93 |
| ASV1697 | 402 | 1 | 1 | 402 | 100 | NR_118667.1 Lachnospira pectinoschiza strain 150-1 16S ribosomal RNA, partial sequence | NR_118667.1 | 1465 | 372 | 773 | 27.44 | 701 | 0 | 392/402 | 97 |
| ASV1698 | 427 | 1 | 1 | 427 | 100 | NR_028862.1 Allisonella histaminiformans strain MR2 16S ribosomal RNA, partial sequence | NR_028862.1 | 1537 | 376 | 802 | 27.78 | 789.6 | 0 | 427/427 | 100 |
| ASV1699 | 422 | 1 | 1 | 422 | 100 | NR_113411.1 Prevotella copri DSM 18205 strain JCM 13464 16S ribosomal RNA, partial sequence | NR_113411.1 | 1493 | 358 | 779 | 28.27 | 725 | 0 | 412/422 | 97 |
| ASV1700 | 422 | 1 | 1 | 422 | 100 | NR_113411.1 Prevotella copri DSM 18205 strain JCM 13464 16S ribosomal RNA, partial sequence | NR_113411.1 | 1493 | 358 | 779 | 28.27 | 592 | 7E-169 | 390/424 | 91 |
| ASV1701 | 422 | 1 | 1 | 422 | 100 | NR_146018.1 Paludibacter jiangxiensis strain NM7 16S ribosomal RNA, partial sequence | NR_146018.1 | 1469 | 340 | 761 | 28.73 | 492.3 | 8E-139 | 375/427 | 87 |
| ASV1702 | 402 | 1 | 1 | 402 | 100 | NR_159227.1 Monoglobus pectinilyticus strain 14 16S ribosomal RNA, partial sequence | NR_159227.1 | 1493 | 368 | 769 | 26.93 | 555.1 | 9E-158 | 372/406 | 91 |
| ASV1703 | 405 | 1 | 1 | 405 | 100 | NR_144743.1 Christensenella timonensis strain Marseille-P2437 16S ribosomal RNA, partial sequence | NR_144743.1 | 1498 | 369 | 773 | 27.04 | 460.9 | 2E-129 | 354/406 | 87 |
| ASV1704 | 405 | 1 | 1 | 405 | 100 | NR_044490.1 Butyricicoccus pullicaecorum strain 25-3 16S ribosomal RNA, partial sequence | NR_044490.1 | 1482 | 366 | 770 | 27.33 | 649.3 | 0 | 387/405 | 95 |
| ASV1705 | 403 | 1 | 1 | 403 | 100 | NR_159296.1 Aminipila butyrica strain FH042 16S ribosomal RNA, partial sequence | NR_159296.1 | 1460 | 352 | 755 | 27.67 | 601.3 | 1E-171 | 378/404 | 93 |
| ASV1706 | 422 | 1 | 1 | 422 | 100 | NR_041364.1 Prevotella stercorea DSM 18206 strain CB35 16S ribosomal RNA, partial sequence | NR_041364.1 | 1488 | 354 | 775 | 28.36 | 708.4 | 0 | 409/422 | 96 |
| ASV1707 | 402 | 1 | 1 | 402 | 100 | NR_042832.1 Roseburia faecis strain M72/1 16S ribosomal RNA, partial sequence | NR_042832.1 | 1456 | 347 | 749 | 27.68 | 660.4 | 0 | 388/403 | 96 |
| ASV1708 | 423 | 1 | 1 | 423 | 100 | NR_144750.1 Prevotellamassilia timonensis strain Marseille-P2831 16S ribosomal RNA, partial sequence | NR_144750.1 | 1493 | 358 | 779 | 28.27 | 553.3 | 4E-157 | 385/426 | 90 |
| ASV1709 | 402 | 1 | 1 | 402 | 100 | NR_147396.1 Lachnoclostridium pacaense strain Marseille-P3100 16S ribosomal RNA, partial sequence | NR_147396.1 | 1453 | 345 | 746 | 27.67 | 699.2 | 0 | 394/402 | 98 |
| ASV1710 | 402 | 1 | 1 | 402 | 100 | NR_134772.1 Murimonas intestini strain SRB-530-5-H 16S ribosomal RNA, partial sequence | NR_134772.1 | 1433 | 314 | 715 | 28.05 | 688.1 | 0 | 392/402 | 97 |
| ASV1711 | 403 | 1 | 3 | 403 | 99.5 | NR_125464.1 Anaerobacterium chartisolvens strain T-1-35 16S ribosomal RNA, partial sequence | NR_125464.1 | 1451 | 344 | 745 | 27.71 | 497.9 | 2E-140 | 358/402 | 89 |
| ASV1712 | 403 | 1 | 1 | 403 | 100 | NR_134706.1 Campylobacter iguaniorum strain 1485E 16S ribosomal RNA, partial sequence | NR_134706.1 | 1503 | 351 | 753 | 26.81 | 695.5 | 0 | 394/403 | 97 |
| ASV1713 | 422 | 1 | 1 | 422 | 100 | NR_041364.1 Prevotella stercorea DSM 18206 strain CB35 16S ribosomal RNA, partial sequence | NR_041364.1 | 1488 | 354 | 775 | 28.36 | 719.5 | 0 | 411/422 | 97 |
| ASV1714 | 403 | 1 | 3 | 403 | 99.5 | NR_125464.1 Anaerobacterium chartisolvens strain T-1-35 16S ribosomal RNA, partial sequence | NR_125464.1 | 1451 | 344 | 745 | 27.71 | 492.3 | 7E-139 | 358/403 | 88 |
| ASV1715 | 418 | 1 | 1 | 418 | 100 | NR_025525.1 Butyrivibrio hungatei strain JK 615 16S ribosomal RNA, partial sequence | NR_025525.1 | 1475 | 335 | 749 | 28.14 | 597.6 | 2E-170 | 388/419 | 92 |
| ASV1716 | 426 | 1 | 1 | 425 | 99.77 | NR_028840.1 Mitsuokella jalaludinii strain M9 16S ribosomal RNA, partial sequence | NR_028840.1 | 1543 | 362 | 787 | 27.61 | 658.5 | 0 | 406/429 | 94 |
| ASV1717 | 402 | 1 | 1 | 402 | 100 | NR_102884.1 Ruminococcus champanellensis 18P13 = JCM 17042 16S ribosomal RNA, partial sequence | NR_102884.1 | 1494 | 348 | 749 | 26.91 | 505.3 | 9E-143 | 363/406 | 89 |
| ASV1718 | 422 | 1 | 1 | 422 | 100 | NR_113411.1 Prevotella copri DSM 18205 strain JCM 13464 16S ribosomal RNA, partial sequence | NR_113411.1 | 1493 | 358 | 779 | 28.27 | 592 | 7E-169 | 389/423 | 91 |
| ASV1719 | 402 | 3 | 1 | 402 | 100 | NR_156081.1 Kineothrix alysoides strain KNHs209 16S ribosomal RNA, partial sequence | NR_156081.1 | 1411 | 353 | 754 | 28.49 | 671.5 | 0 | 389/402 | 96 |
| ASV1720 | 402 | 1 | 1 | 402 | 100 | NR_026146.1 Clostridium ventriculi strain DSM 286 16S ribosomal RNA, partial sequence | NR_026146.1 | 1461 | 328 | 728 | 27.45 | 730.5 | 0 | 400/402 | 99 |
| ASV1721 | 402 | 1 | 1 | 402 | 100 | NR_028961.1 Faecalibacterium prausnitzii strain ATCC 27768 16S ribosomal RNA, partial sequence | NR_028961.1 | 1462 | 344 | 745 | 27.5 | 671.5 | 0 | 389/402 | 96 |
| ASV1722 | 402 | 1 | 1 | 402 | 100 | NR_029160.1 Ruminococcus callidus strain ATCC 27760 16S ribosomal RNA, partial sequence | NR_029160.1 | 1371 | 310 | 711 | 29.32 | 654.8 | 0 | 388/404 | 96 |
| ASV1723 | 404 | 1 | 1 | 404 | 100 | NR_026330.1 Eubacterium callanderi strain DSM 3662 16S ribosomal RNA, partial sequence | NR_026330.1 | 1474 | 342 | 745 | 27.41 | 747.2 | 0 | 404/404 | 100 |
| ASV1724 | 405 | 1 | 1 | 405 | 100 | NR_044972.1 Sporobacter termitidis strain SYR 16S ribosomal RNA, partial sequence | NR_044972.1 | 1506 | 348 | 752 | 26.89 | 608.7 | 7E-174 | 380/406 | 93 |
| ASV1725 | 402 | 1 | 1 | 402 | 100 | NR_104735.1 Butyrivibrio crossotus strain DSM 2876 16S ribosomal RNA, partial sequence | NR_104735.1 | 1511 | 353 | 754 | 26.6 | 671.5 | 0 | 389/402 | 96 |
| ASV1726 | 421 | 1 | 1 | 421 | 100 | NR_144616.1 Muribaculum intestinale strain YL27 16S ribosomal RNA, partial sequence | NR_144616.1 | 1460 | 297 | 718 | 28.9 | 584.7 | 1E-166 | 390/425 | 91 |
| ASV1727 | 402 | 1 | 1 | 402 | 100 | NR_074634.1 [Eubacterium] rectale ATCC 33656 16S ribosomal RNA, partial sequence | NR_074634.1 | 1521 | 367 | 768 | 26.43 | 737.9 | 0 | 401/402 | 99 |
| ASV1728 | 402 | 2 | 1 | 402 | 100 | NR_118674.1 Eubacterium ramulus ATCC 29099 16S ribosomal RNA, partial sequence | NR_118674.1 | 1474 | 373 | 774 | 27.27 | 593.9 | 2E-169 | 372/402 | 92 |
| ASV1729 | 422 | 1 | 1 | 422 | 100 | NR_041343.1 Parabacteroides merdae strain JCM 9497 16S ribosomal RNA, partial sequence | NR_041343.1 | 1492 | 357 | 778 | 28.28 | 680.7 | 0 | 404/422 | 95 |
| ASV1731 | 402 | 1 | 2 | 402 | 99.75 | NR_027579.1 Ruminococcus lactaris ATCC 29176 16S ribosomal RNA, partial sequence | NR_027579.1 | 1402 | 334 | 734 | 28.6 | 671.5 | 0 | 388/401 | 96 |
| ASV1732 | 427 | 1 | 1 | 427 | 100 | NR_028797.1 Selenomonas infelix strain VPI D 75 B-30 16S ribosomal RNA, partial sequence | NR_028797.1 | 1524 | 373 | 799 | 28.02 | 617.9 | 1E-176 | 396/427 | 92 |
| ASV1734 | 422 | 4 | 1 | 422 | 100 | NR_044864.1 Hallella seregens ATCC 51272 16S ribosomal RNA, partial sequence | NR_044864.1 | 1463 | 335 | 756 | 28.84 | 564.3 | 2E-160 | 385/424 | 90 |
| ASV1735 | 427 | 1 | 1 | 427 | 100 | NR_042797.1 Treponema berlinense strain 7CPL208 16S ribosomal RNA, partial sequence | NR_042797.1 | 1440 | 275 | 701 | 29.65 | 784.1 | 0 | 426/427 | 99 |
| ASV1736 | 422 | 1 | 1 | 422 | 100 | NR_041364.1 Prevotella stercorea DSM 18206 strain CB35 16S ribosomal RNA, partial sequence | NR_041364.1 | 1488 | 354 | 775 | 28.36 | 553.3 | 4E-157 | 382/423 | 90 |
| ASV1737 | 402 | 3 | 1 | 402 | 100 | NR_156081.1 Kineothrix alysoides strain KNHs209 16S ribosomal RNA, partial sequence | NR_156081.1 | 1411 | 353 | 754 | 28.49 | 688.1 | 0 | 392/402 | 97 |
| ASV1738 | 403 | 1 | 1 | 403 | 100 | NR_109636.1 Cloacibacillus porcorum strain CL-84 16S ribosomal RNA, partial sequence | NR_109636.1 | 1447 | 320 | 721 | 27.78 | 654.8 | 0 | 387/403 | 96 |
| ASV1739 | 402 | 1 | 1 | 402 | 100 | NR_028961.1 Faecalibacterium prausnitzii strain ATCC 27768 16S ribosomal RNA, partial sequence | NR_028961.1 | 1462 | 344 | 745 | 27.5 | 654.8 | 0 | 386/402 | 96 |
| ASV1740 | 421 | 1 | 1 | 421 | 100 | NR_144616.1 Muribaculum intestinale strain YL27 16S ribosomal RNA, partial sequence | NR_144616.1 | 1460 | 297 | 718 | 28.9 | 501.6 | 1E-141 | 377/427 | 88 |
| ASV1742 | 403 | 1 | 1 | 403 | 100 | NR_025930.1 Ruminococcus bromii strain ATCC 27255 16S ribosomal RNA, partial sequence | NR_025930.1 | 1380 | 313 | 716 | 29.28 | 579.1 | 6E-165 | 374/404 | 92 |
| ASV1743 | 408 | 1 | 1 | 408 | 100 | NR_044675.2 Anaeroplasma bactoclasticum strain JR 16S ribosomal RNA, partial sequence | NR_044675.2 | 1435 | 336 | 746 | 28.64 | 486.8 | 3E-137 | 368/417 | 88 |
| ASV1744 | 427 | 1 | 1 | 427 | 100 | NR_036788.2 Lactobacillus pontis strain LTH 2587 16S ribosomal RNA, partial sequence | NR_036788.2 | 1568 | 384 | 810 | 27.23 | 761.9 | 0 | 422/427 | 98 |
| ASV1745 | 405 | 1 | 1 | 405 | 100 | NR_044972.1 Sporobacter termitidis strain SYR 16S ribosomal RNA, partial sequence | NR_044972.1 | 1506 | 348 | 752 | 26.89 | 553.3 | 3E-157 | 371/407 | 91 |
| ASV1746 | 402 | 1 | 1 | 402 | 100 | NR_159227.1 Monoglobus pectinilyticus strain 14 16S ribosomal RNA, partial sequence | NR_159227.1 | 1493 | 368 | 769 | 26.93 | 649.3 | 0 | 386/403 | 95 |
| ASV1747 | 402 | 2 | 1 | 402 | 100 | NR_117147.1 Faecalicatena contorta strain DSM 3982 16S ribosomal RNA, partial sequence | NR_117147.1 | 1524 | 363 | 764 | 26.38 | 726.9 | 0 | 399/402 | 99 |
| ASV1748 | 423 | 1 | 1 | 423 | 100 | NR_144750.1 Prevotellamassilia timonensis strain Marseille-P2831 16S ribosomal RNA, partial sequence | NR_144750.1 | 1493 | 358 | 779 | 28.27 | 575.4 | 7E-164 | 388/425 | 91 |
| ASV1749 | 423 | 1 | 1 | 423 | 100 | NR_144750.1 Prevotellamassilia timonensis strain Marseille-P2831 16S ribosomal RNA, partial sequence | NR_144750.1 | 1493 | 358 | 779 | 28.27 | 564.3 | 2E-160 | 386/425 | 90 |
| ASV1750 | 402 | 5 | 1 | 402 | 100 | NR_115693.1 Gracilibacter thermotolerans JW/YJL-S1 16S ribosomal RNA, partial sequence | NR_115693.1 | 1526 | 459 | 861 | 26.41 | 444.3 | 2E-124 | 351/405 | 86 |
| ASV1751 | 427 | 1 | 1 | 426 | 99.77 | NR_044111.1 Selenomonas bovis strain WG 16S ribosomal RNA, partial sequence | NR_044111.1 | 1525 | 370 | 795 | 27.93 | 732.4 | 0 | 416/426 | 97 |
| ASV1754 | 423 | 1 | 1 | 423 | 100 | NR_144750.1 Prevotellamassilia timonensis strain Marseille-P2831 16S ribosomal RNA, partial sequence | NR_144750.1 | 1493 | 358 | 779 | 28.27 | 564.3 | 2E-160 | 386/425 | 90 |
| ASV1755 | 405 | 1 | 3 | 405 | 99.51 | NR_144743.1 Christensenella timonensis strain Marseille-P2437 16S ribosomal RNA, partial sequence | NR_144743.1 | 1498 | 371 | 773 | 26.9 | 473.9 | 3E-133 | 354/403 | 87 |
| ASV1756 | 427 | 5 | 1 | 427 | 100 | NR_146813.2 Erysipelothrix larvae strain LV19 16S ribosomal RNA, complete sequence | NR_146813.2 | 1543 | 367 | 792 | 27.61 | 444.3 | 2E-124 | 370/432 | 85 |
| ASV1758 | 426 | 1 | 1 | 425 | 99.77 | NR_114696.1 Anaerovibrio lipolyticus DSM 3074 16S ribosomal RNA, partial sequence | NR_114696.1 | 1526 | 372 | 796 | 27.85 | 675.1 | 0 | 407/427 | 95 |
| ASV1759 | 427 | 5 | 4 | 427 | 99.3 | NR_146813.2 Erysipelothrix larvae strain LV19 16S ribosomal RNA, complete sequence | NR_146813.2 | 1543 | 370 | 792 | 27.41 | 444.3 | 2E-124 | 366/427 | 85 |
| ASV1760 | 427 | 1 | 1 | 427 | 100 | NR_102980.1 Megasphaera elsdenii DSM 20460 16S ribosomal RNA, partial sequence | NR_102980.1 | 1552 | 369 | 795 | 27.51 | 606.8 | 3E-173 | 394/427 | 92 |
| ASV1761 | 402 | 1 | 1 | 402 | 100 | NR_029097.1 Oribacterium sinus strain AIP 354.02 16S ribosomal RNA, partial sequence | NR_029097.1 | 1374 | 308 | 709 | 29.26 | 649.3 | 0 | 385/402 | 95 |
| ASV1762 | 404 | 1 | 1 | 404 | 100 | NR_026476.1 Succinivibrio dextrinosolvens strain 0554 16S ribosomal RNA, partial sequence | NR_026476.1 | 1463 | 331 | 732 | 27.48 | 651.1 | 0 | 387/404 | 95 |
| ASV1763 | 422 | 1 | 1 | 422 | 100 | NR_041342.1 Parabacteroides distasonis strain JCM 5825 16S ribosomal RNA, partial sequence | NR_041342.1 | 1488 | 353 | 774 | 28.36 | 553.3 | 4E-157 | 381/422 | 90 |
| ASV1764 | 402 | 1 | 1 | 402 | 100 | NR_029160.1 Ruminococcus callidus strain ATCC 27760 16S ribosomal RNA, partial sequence | NR_029160.1 | 1371 | 310 | 711 | 29.32 | 649.3 | 0 | 387/404 | 95 |
| ASV1765 | 422 | 1 | 1 | 422 | 100 | NR_118219.1 Alistipes senegalensis JC50 16S ribosomal RNA, partial sequence | NR_118219.1 | 1480 | 349 | 770 | 28.51 | 780.4 | 0 | 422/422 | 100 |
| ASV1766 | 403 | 2 | 1 | 403 | 100 | NR_024948.1 Campylobacter hyointestinalis subsp. lawsonii strain CHY5 16S ribosomal RNA, partial sequence | NR_024948.1 | 1419 | 318 | 720 | 28.4 | 706.5 | 0 | 396/403 | 98 |
| ASV1767 | 404 | 1 | 1 | 404 | 100 | NR_118156.1 Oscillibacter ruminantium GH1 16S ribosomal RNA, partial sequence | NR_118156.1 | 1409 | 321 | 724 | 28.67 | 608.7 | 7E-174 | 379/404 | 93 |
| ASV1768 | 427 | 1 | 15 | 427 | 96.72 | NR_104750.1 Spiroplasma clarkii strain CN-5 16S ribosomal RNA, partial sequence | NR_104750.1 | 1507 | 351 | 762 | 27.34 | 435.1 | 1E-121 | 355/414 | 85 |
| ASV1769 | 402 | 1 | 1 | 402 | 100 | NR_028961.1 Faecalibacterium prausnitzii strain ATCC 27768 16S ribosomal RNA, partial sequence | NR_028961.1 | 1462 | 344 | 745 | 27.5 | 732.4 | 0 | 400/402 | 99 |
| ASV1770 | 422 | 1 | 1 | 422 | 100 | NR_041364.1 Prevotella stercorea DSM 18206 strain CB35 16S ribosomal RNA, partial sequence | NR_041364.1 | 1488 | 354 | 775 | 28.36 | 702.8 | 0 | 408/422 | 96 |
| ASV1771 | 403 | 1 | 1 | 403 | 100 | NR_144611.1 Flintibacter butyricus strain BLS21 16S ribosomal RNA, partial sequence | NR_144611.1 | 808 | 299 | 703 | 50.12 | 514.5 | 2E-145 | 366/408 | 89 |
| ASV1772 | 421 | 2 | 1 | 421 | 100 | NR_113077.1 Paraprevotella clara strain JCM 14859 16S ribosomal RNA, partial sequence | NR_113077.1 | 1480 | 346 | 766 | 28.45 | 479.4 | 6E-135 | 370/424 | 87 |
| ASV1773 | 427 | 1 | 1 | 427 | 100 | NR_044190.1 Streptococcus caballi strain 151 16S ribosomal RNA, partial sequence | NR_044190.1 | 1430 | 313 | 739 | 29.86 | 789.6 | 0 | 427/427 | 100 |
| ASV1774 | 422 | 1 | 1 | 422 | 100 | NR_144616.1 Muribaculum intestinale strain YL27 16S ribosomal RNA, partial sequence | NR_144616.1 | 1460 | 297 | 718 | 28.9 | 542.2 | 8E-154 | 381/424 | 89 |
| ASV1775 | 402 | 3 | 1 | 402 | 100 | NR_151917.1 Helicobacter apri strain A19 16S ribosomal RNA, partial sequence | NR_151917.1 | 1099 | 36 | 437 | 36.58 | 693.6 | 0 | 393/402 | 97 |
| ASV1776 | 402 | 1 | 1 | 402 | 100 | NR_118675.1 [Eubacterium] siraeum strain ATCC 29066 16S ribosomal RNA, partial sequence | NR_118675.1 | 1453 | 356 | 757 | 27.67 | 595.7 | 5E-170 | 374/402 | 93 |
| ASV1777 | 427 | 1 | 1 | 427 | 100 | NR_113259.1 Lactobacillus agilis strain JCM 1187 16S ribosomal RNA, partial sequence | NR_113259.1 | 1485 | 351 | 777 | 28.75 | 789.6 | 0 | 427/427 | 100 |
| ASV1779 | 422 | 1 | 1 | 422 | 100 | NR_041464.1 Parabacteroides johnsonii strain M-165 16S ribosomal RNA, partial sequence | NR_041464.1 | 1492 | 357 | 778 | 28.28 | 680.7 | 0 | 404/422 | 95 |
| ASV1780 | 427 | 3 | 1 | 427 | 100 | NR_036918.1 Streptococcus suis strain S735 16S ribosomal RNA, partial sequence | NR_036918.1 | 1535 | 354 | 780 | 27.82 | 789.6 | 0 | 427/427 | 100 |
| ASV1781 | 421 | 1 | 1 | 421 | 100 | NR_144616.1 Muribaculum intestinale strain YL27 16S ribosomal RNA, partial sequence | NR_144616.1 | 1460 | 297 | 718 | 28.9 | 479.4 | 6E-135 | 373/427 | 87 |
| ASV1782 | 402 | 1 | 2 | 402 | 99.75 | NR_027579.1 Ruminococcus lactaris ATCC 29176 16S ribosomal RNA, partial sequence | NR_027579.1 | 1402 | 334 | 734 | 28.6 | 682.5 | 0 | 390/401 | 97 |
| ASV1783 | 405 | 1 | 1 | 405 | 100 | NR_147370.1 Pseudoflavonifractor phocaeensis strain Marseille-P3064 16S ribosomal RNA, partial sequence | NR_147370.1 | 1495 | 366 | 770 | 27.09 | 571.7 | 9E-163 | 376/408 | 92 |
| ASV1784 | 402 | 1 | 1 | 402 | 100 | NR_134772.1 Murimonas intestini strain SRB-530-5-H 16S ribosomal RNA, partial sequence | NR_134772.1 | 1433 | 314 | 715 | 28.05 | 677 | 0 | 390/402 | 97 |
| ASV1785 | 421 | 1 | 1 | 421 | 100 | NR_144616.1 Muribaculum intestinale strain YL27 16S ribosomal RNA, partial sequence | NR_144616.1 | 1460 | 297 | 718 | 28.9 | 523.7 | 3E-148 | 380/426 | 89 |
| ASV1787 | 427 | 1 | 1 | 427 | 100 | NR_042942.1 Treponema porcinum strain 14V28 16S ribosomal RNA, partial sequence | NR_042942.1 | 1426 | 258 | 684 | 29.94 | 767.5 | 0 | 423/427 | 99 |
| ASV1788 | 403 | 2 | 1 | 403 | 100 | NR_134091.1 Mobilitalea sibirica strain P3M-3 16S ribosomal RNA, partial sequence | NR_134091.1 | 1444 | 352 | 753 | 27.84 | 654.8 | 0 | 387/403 | 96 |
| ASV1789 | 407 | 1 | 1 | 407 | 100 | NR_109752.1 Rothia endophytica strain YIM 67072 16S ribosomal RNA, partial sequence | NR_109752.1 | 1520 | 363 | 769 | 26.78 | 747.2 | 0 | 406/407 | 99 |
| ASV1790 | 422 | 1 | 1 | 422 | 100 | NR_041446.1 Bacteroides barnesiae strain BL2 16S ribosomal RNA, partial sequence | NR_041446.1 | 1456 | 340 | 761 | 28.98 | 603.1 | 3E-172 | 393/425 | 92 |
| ASV1792 | 427 | 1 | 1 | 427 | 100 | NR_153710.1 Peptococcus simiae strain M108 16S ribosomal RNA, partial sequence | NR_153710.1 | 1488 | 354 | 780 | 28.7 | 612.4 | 6E-175 | 399/431 | 92 |
| ASV1793 | 422 | 1 | 1 | 422 | 100 | NR_041342.1 Parabacteroides distasonis strain JCM 5825 16S ribosomal RNA, partial sequence | NR_041342.1 | 1488 | 353 | 774 | 28.36 | 547.7 | 2E-155 | 381/423 | 90 |
| ASV1794 | 426 | 1 | 1 | 425 | 99.77 | NR_027526.1 Catenibacterium mitsuokai strain DSM 15897 16S ribosomal RNA, partial sequence | NR_027526.1 | 1485 | 345 | 767 | 28.48 | 712.1 | 0 | 412/425 | 96 |
| ASV1795 | 402 | 1 | 1 | 402 | 100 | NR_170495.1 Blautia faecicola strain KGMB01111 16S ribosomal RNA, complete sequence | NR_170495.1 | 1534 | 372 | 773 | 26.21 | 582.8 | 4E-166 | 373/402 | 92 |
| ASV1796 | 402 | 1 | 1 | 402 | 100 | NR_028961.1 Faecalibacterium prausnitzii strain ATCC 27768 16S ribosomal RNA, partial sequence | NR_028961.1 | 1462 | 344 | 745 | 27.5 | 721.3 | 0 | 398/402 | 99 |
| ASV1797 | 422 | 2 | 1 | 422 | 100 | NR_113105.1 Prevotella histicola strain JCM 15637 16S ribosomal RNA, partial sequence | NR_113105.1 | 1480 | 346 | 767 | 28.51 | 636.4 | 0 | 398/424 | 93 |
| ASV1798 | 422 | 1 | 1 | 422 | 100 | NR_113206.1 Bacteroides faecichinchillae JCM 17102 16S ribosomal RNA, partial sequence | NR_113206.1 | 1486 | 353 | 774 | 28.4 | 569.9 | 3E-162 | 386/424 | 91 |
| ASV1799 | 402 | 5 | 1 | 402 | 100 | NR_156081.1 Kineothrix alysoides strain KNHs209 16S ribosomal RNA, partial sequence | NR_156081.1 | 1411 | 353 | 754 | 28.49 | 693.6 | 0 | 394/403 | 97 |
| ASV1800 | 402 | 2 | 1 | 402 | 100 | NR_042200.1 [Clostridium] asparagiforme strain N6 16S ribosomal RNA, partial sequence | NR_042200.1 | 1468 | 335 | 736 | 27.38 | 682.5 | 0 | 391/402 | 97 |
| ASV1801 | 422 | 1 | 1 | 422 | 100 | NR_041342.1 Parabacteroides distasonis strain JCM 5825 16S ribosomal RNA, partial sequence | NR_041342.1 | 1488 | 353 | 774 | 28.36 | 564.3 | 2E-160 | 383/422 | 90 |
| ASV1802 | 421 | 1 | 1 | 421 | 100 | NR_126194.1 Butyricimonas faecihominis strain 180-3 16S ribosomal RNA, partial sequence | NR_126194.1 | 1486 | 352 | 772 | 28.33 | 562.5 | 6E-160 | 383/423 | 90 |
| ASV1803 | 402 | 1 | 1 | 402 | 100 | NR_113021.1 Clostridium paraputrificum strain JCM 1293 16S ribosomal RNA, partial sequence | NR_113021.1 | 1476 | 348 | 749 | 27.24 | 710.2 | 0 | 396/402 | 98 |
| ASV1804 | 405 | 1 | 3 | 405 | 99.51 | NR_125464.1 Anaerobacterium chartisolvens strain T-1-35 16S ribosomal RNA, partial sequence | NR_125464.1 | 1451 | 344 | 745 | 27.71 | 466.5 | 4E-131 | 353/403 | 87 |
| ASV1805 | 428 | 1 | 1 | 428 | 100 | NR_104781.1 Treponema bryantii strain RUS-1 16S ribosomal RNA, partial sequence | NR_104781.1 | 1535 | 351 | 778 | 27.88 | 653 | 0 | 404/429 | 94 |
| ASV1806 | 402 | 1 | 1 | 402 | 100 | NR_029239.3 Clostridium chartatabidum strain 163 16S ribosomal RNA, partial sequence | NR_029239.3 | 1460 | 323 | 724 | 27.53 | 715.8 | 0 | 397/402 | 98 |
| ASV1807 | 405 | 1 | 1 | 405 | 100 | NR_147370.1 Pseudoflavonifractor phocaeensis strain Marseille-P3064 16S ribosomal RNA, partial sequence | NR_147370.1 | 1495 | 366 | 770 | 27.09 | 494.2 | 2E-139 | 359/405 | 88 |
| ASV1808 | 421 | 5 | 1 | 421 | 100 | NR_146813.2 Erysipelothrix larvae strain LV19 16S ribosomal RNA, complete sequence | NR_146813.2 | 1543 | 367 | 792 | 27.61 | 527.4 | 2E-149 | 380/426 | 89 |
| ASV1809 | 405 | 1 | 1 | 405 | 100 | NR_044972.1 Sporobacter termitidis strain SYR 16S ribosomal RNA, partial sequence | NR_044972.1 | 1506 | 348 | 752 | 26.89 | 603.1 | 3E-172 | 379/406 | 93 |
| ASV1810 | 427 | 1 | 1 | 427 | 100 | NR_074755.1 Treponema succinifaciens DSM 2489 16S ribosomal RNA, partial sequence | NR_074755.1 | 1531 | 353 | 779 | 27.89 | 473.9 | 3E-133 | 373/430 | 86 |
| ASV1811 | 422 | 2 | 1 | 422 | 100 | NR_113117.1 Prevotella oralis ATCC 33269 strain JCM 12251 16S ribosomal RNA, partial sequence | NR_113117.1 | 1496 | 361 | 782 | 28.21 | 625.3 | 7E-179 | 395/423 | 93 |
| ASV1812 | 401 | 1 | 1 | 401 | 100 | NR_027558.1 Anaerotruncus colihominis strain WAL 14565 16S ribosomal RNA, partial sequence | NR_027558.1 | 1365 | 309 | 709 | 29.38 | 608.7 | 7E-174 | 377/401 | 94 |
| ASV1813 | 405 | 1 | 1 | 405 | 100 | NR_044972.1 Sporobacter termitidis strain SYR 16S ribosomal RNA, partial sequence | NR_044972.1 | 1506 | 348 | 752 | 26.89 | 614.2 | 2E-175 | 381/406 | 93 |
| ASV1814 | 421 | 2 | 1 | 421 | 100 | NR_113073.1 Barnesiella intestinihominis strain JCM 15079 16S ribosomal RNA, partial sequence | NR_113073.1 | 1481 | 346 | 767 | 28.49 | 496 | 6E-140 | 372/423 | 87 |
| ASV1815 | 427 | 1 | 1 | 427 | 100 | NR_026030.1 Actinobacillus porcinus strain NM319 16S ribosomal RNA, partial sequence | NR_026030.1 | 1466 | 335 | 761 | 29.13 | 773 | 0 | 424/427 | 99 |
| ASV1816 | 422 | 1 | 1 | 422 | 100 | NR_113411.1 Prevotella copri DSM 18205 strain JCM 13464 16S ribosomal RNA, partial sequence | NR_113411.1 | 1493 | 358 | 779 | 28.27 | 758.2 | 0 | 418/422 | 99 |
| ASV1817 | 422 | 1 | 1 | 422 | 100 | NR_043216.1 Prevotella loescheii strain NCTC 11321 16S ribosomal RNA, partial sequence | NR_043216.1 | 1451 | 336 | 757 | 29.08 | 568 | 1E-161 | 384/423 | 90 |
| ASV1818 | 426 | 1 | 1 | 426 | 100 | NR_114696.1 Anaerovibrio lipolyticus DSM 3074 16S ribosomal RNA, partial sequence | NR_114696.1 | 1526 | 372 | 797 | 27.92 | 699.2 | 0 | 412/428 | 96 |
| ASV1819 | 402 | 1 | 1 | 402 | 100 | NR_028961.1 Faecalibacterium prausnitzii strain ATCC 27768 16S ribosomal RNA, partial sequence | NR_028961.1 | 1462 | 344 | 745 | 27.5 | 649.3 | 0 | 385/402 | 95 |
| ASV1820 | 422 | 1 | 1 | 422 | 100 | NR_028963.1 Anaerophaga thermohalophila DSM 12881 strain Fru22 16S ribosomal RNA, partial sequence | NR_028963.1 | 1512 | 344 | 766 | 27.98 | 448 | 2E-125 | 365/425 | 85 |
| ASV1821 | 405 | 1 | 1 | 405 | 100 | NR_025025.1 Papillibacter cinnamivorans strain CIN1 16S ribosomal RNA, partial sequence | NR_025025.1 | 1491 | 349 | 751 | 27.03 | 569.9 | 3E-162 | 373/406 | 91 |
| ASV1822 | 402 | 2 | 1 | 402 | 100 | NR_156080.1 Kineothrix alysoides strain KNHs209 16S ribosomal RNA, partial sequence | NR_156080.1 | 1410 | 353 | 754 | 28.51 | 671.5 | 0 | 390/403 | 96 |
| ASV1823 | 401 | 1 | 1 | 401 | 100 | NR_144749.1 Ihubacter massiliensis strain Marseille-P2843 16S ribosomal RNA, partial sequence | NR_144749.1 | 1479 | 364 | 766 | 27.25 | 538.5 | 9E-153 | 366/403 | 90 |
| ASV1824 | 405 | 1 | 1 | 405 | 100 | NR_151982.1 Agathobaculum butyriciproducens strain SR79 16S ribosomal RNA, partial sequence | NR_151982.1 | 1383 | 265 | 669 | 29.28 | 737.9 | 0 | 403/405 | 99 |
| ASV1825 | 422 | 2 | 1 | 422 | 100 | NR_113117.1 Prevotella oralis ATCC 33269 strain JCM 12251 16S ribosomal RNA, partial sequence | NR_113117.1 | 1496 | 361 | 782 | 28.21 | 603.1 | 3E-172 | 391/423 | 92 |
| ASV1826 | 402 | 1 | 1 | 402 | 100 | NR_074634.1 [Eubacterium] rectale ATCC 33656 16S ribosomal RNA, partial sequence | NR_074634.1 | 1521 | 367 | 768 | 26.43 | 721.3 | 0 | 398/402 | 99 |
| ASV1827 | 427 | 1 | 1 | 427 | 100 | NR_042896.1 Mucispirillum schaedleri strain HRI I17 16S ribosomal RNA, partial sequence | NR_042896.1 | 1471 | 333 | 759 | 29.03 | 701 | 0 | 411/427 | 96 |
| ASV1828 | 422 | 1 | 1 | 422 | 100 | NR_041364.1 Prevotella stercorea DSM 18206 strain CB35 16S ribosomal RNA, partial sequence | NR_041364.1 | 1488 | 354 | 775 | 28.36 | 553.3 | 4E-157 | 382/423 | 90 |

| | | | | | | | | | | | | | | | | | | | | | | | | | | | |
|---|---|---|---|---|---|---|---|---|---|---|---|---|---|---|---|---|---|---|---|---|---|---|---|---|---|---|---|
| ASV1829 | 422 | 2 | 1 | 422 | 100 | NR_074376.1 Parabacteroides distasonis ATCC 8503 16S ribosomal RNA, partial sequence | NR_074376.1 | 1497 | 368 | 789 | 28.19 | 569.9 | 3E-162 | 384/422 | 90 | 0/422 | 0 | Plus | Plus | Bacteria; Bacteroidetes; Bacteroidia; Bacteroidales; Tannerellaceae; Parabacteroides; Parabacteroides distasonis | Parabacteroides distasonis | Parabacteroides | Tannerellaceae | Bacteroidales | Bacteroidia | Bacteroidetes | Bacteria |
| ASV1830 | 421 | 5 | 1 | 421 | 100 | NR_025594.1 Erysipelothrix inopinata strain 143-02 16S ribosomal RNA, partial sequence | NR_025594.1 | 1504 | 346 | 771 | 28.32 | 538.5 | 1E-152 | 383/427 | 89 | 7/427 | 1 | Plus | Plus | Bacteria; Firmicutes; Erysipelotrichia; Erysipelotrichales; Erysipelotrichaceae; Erysipelothrix; Erysipelothrix inopinata | Erysipelothrix inopinata | Erysipelothrix | Erysipelotrichaceae | Erysipelotrichales | Erysipelotrichia | Firmicutes | Bacteria |
| ASV1831 | 421 | 1 | 1 | 421 | 100 | NR_144616.1 Muribaculum intestinale strain YL27 16S ribosomal RNA, partial sequence | NR_144616.1 | 1460 | 297 | 718 | 28.9 | 595.7 | 6E-170 | 389/422 | 92 | 1/422 | 0 | Plus | Plus | Bacteria; Bacteroidetes; Bacteroidia; Bacteroidales; Muribaculaceae; Muribaculum; Muribaculum intestinale | Muribaculum intestinale | Muribaculum | Muribaculaceae | Bacteroidales | Bacteroidia | Bacteroidetes | Bacteria |
| ASV1832 | 403 | 1 | 1 | 403 | 100 | NR_104907.1 Eubacterium coprostanoligenes strain HL 16S ribosomal RNA, partial sequence | NR_104907.1 | 1446 | 332 | 734 | 27.87 | 728.7 | 0 | 400/403 | 99 | 0/403 | 0 | Plus | Plus | Bacteria; Firmicutes; Clostridia; Eubacteriales; Eubacteriaceae; Eubacterium; Eubacterium coprostanoligenes | Eubacterium coprostanoligenes | Eubacterium | Eubacteriaceae | Eubacteriales | Clostridia | Firmicutes | Bacteria |
| ASV1833 | 428 | 1 | 1 | 428 | 100 | NR_104781.1 Treponema bryantii strain RUS-1 16S ribosomal RNA, partial sequence | NR_104781.1 | 1535 | 351 | 778 | 27.88 | 542.2 | 8E-154 | 387/432 | 89 | 8/432 | 1 | Plus | Plus | Bacteria; Spirochaetes; Spirochaetia; Spirochaetales; Spirochaetaceae; Treponema; Treponema bryantii | Treponema bryantii | Treponema | Spirochaetaceae | Spirochaetales | Spirochaetia | Spirochaetes | Bacteria |
| ASV1834 | 403 | 1 | 3 | 403 | 99.5 | NR_125464.1 Anaerobacterium chartisolvens strain T-1-35 16S ribosomal RNA, partial sequence | NR_125464.1 | 1451 | 344 | 745 | 27.71 | 481.2 | 2E-135 | 355/402 | 88 | 1/402 | 0 | Plus | Plus | Bacteria; Firmicutes; Clostridia; Eubacteriales; Oscillospiraceae; Anaerobacterium; Anaerobacterium chartisolvens | Anaerobacterium chartisolvens | Anaerobacterium | Oscillospiraceae | Eubacteriales | Clostridia | Firmicutes | Bacteria |
| ASV1835 | 403 | 1 | 3 | 403 | 99.5 | NR_125677.1 Vallitalea pronyensis strain FatNI3 16S ribosomal RNA, partial sequence | NR_125677.1 | 1480 | 365 | 764 | 27.03 | 479.4 | 6E-135 | 356/403 | 88 | 5/403 | 1 | Plus | Plus | Bacteria; Firmicutes; Clostridia; Eubacteriales; Vallitaleaceae; Vallitalea; Vallitalea pronyensis | Vallitalea pronyensis | Vallitalea | Vallitaleaceae | Eubacteriales | Clostridia | Firmicutes | Bacteria |
| ASV1836 | 427 | 2 | 1 | 427 | 100 | NR_041778.1 Desulfovibrio piger strain ATCC 29098 16S ribosomal RNA, partial sequence | NR_041778.1 | 1542 | 357 | 783 | 27.69 | 689.9 | 0 | 410/428 | 95 | 2/428 | 0 | Plus | Plus | Bacteria; Proteobacteria; Deltaproteobacteria; Desulfovibrionales; Desulfovibrionaceae; Desulfovibrio; Desulfovibrio piger | Desulfovibrio piger | Desulfovibrio | Desulfovibrionaceae | Desulfovibrionales | Deltaproteobacteria | Proteobacteria | Bacteria |
| ASV1837 | 423 | 1 | 1 | 423 | 100 | NR_144750.1 Prevotellamassilia timonensis strain Marseille-P2831 16S ribosomal RNA, partial sequence | NR_144750.1 | 1493 | 358 | 779 | 28.27 | 553.3 | 4E-157 | 385/426 | 90 | 7/426 | 1 | Plus | Plus | Bacteria; Bacteroidetes; Bacteroidia; Bacteroidales; Prevotellaceae; Prevotellamassilia; Prevotellamassilia timonensis | Prevotellamassilia timonensis | Prevotellamassilia | Prevotellaceae | Bacteroidales | Bacteroidia | Bacteroidetes | Bacteria |
| ASV1838 | 402 | 1 | 1 | 402 | 100 | NR_025206.1 Hespellia porcina strain PC80 16S ribosomal RNA, partial sequence | NR_025206.1 | 1449 | 331 | 732 | 27.74 | 638.2 | 0 | 383/402 | 95 | 0/402 | 0 | Plus | Plus | Bacteria; Firmicutes; Clostridia; Eubacteriales; Lachnospiraceae; Hespellia; Hespellia porcina | Hespellia porcina | Hespellia | Lachnospiraceae | Eubacteriales | Clostridia | Firmicutes | Bacteria |
| ASV1839 | 403 | 1 | 3 | 403 | 99.5 | NR_026104.1 Ruminiclostridium cellobioparum DSM 1351 = ATCC 15832 16S ribosomal RNA, partial sequence | NR_026104.1 | 1487 | 331 | 733 | 27.1 | 477.6 | 2E-134 | 355/403 | 88 | 2/403 | 0 | Plus | Plus | Bacteria; Firmicutes; Clostridia; Eubacteriales; Oscillospiraceae; Ruminiclostridium; Ruminiclostridium cellobioparum | Ruminiclostridium cellobioparum | Ruminiclostridium | Oscillospiraceae | Eubacteriales | Clostridia | Firmicutes | Bacteria |
| ASV1840 | 427 | 1 | 1 | 427 | 100 | NR_041778.1 Desulfovibrio piger strain ATCC 29098 16S ribosomal RNA, partial sequence | NR_041778.1 | 1542 | 357 | 783 | 27.69 | 662.2 | 0 | 404/427 | 94 | 0/427 | 0 | Plus | Plus | Bacteria; Proteobacteria; Deltaproteobacteria; Desulfovibrionales; Desulfovibrionaceae; Desulfovibrio; Desulfovibrio piger | Desulfovibrio piger | Desulfovibrio | Desulfovibrionaceae | Desulfovibrionales | Deltaproteobacteria | Proteobacteria | Bacteria |
| ASV1841 | 423 | 1 | 1 | 423 | 100 | NR_144750.1 Prevotellamassilia timonensis strain Marseille-P2831 16S ribosomal RNA, partial sequence | NR_144750.1 | 1493 | 358 | 779 | 28.27 | 564.3 | 2E-160 | 387/426 | 90 | 7/426 | 1 | Plus | Plus | Bacteria; Bacteroidetes; Bacteroidia; Bacteroidales; Prevotellaceae; Prevotellamassilia; Prevotellamassilia timonensis | Prevotellamassilia timonensis | Prevotellamassilia | Prevotellaceae | Bacteroidales | Bacteroidia | Bacteroidetes | Bacteria |
| ASV1842 | 422 | 1 | 1 | 422 | 100 | NR_144750.1 Prevotellamassilia timonensis strain Marseille-P2831 16S ribosomal RNA, partial sequence | NR_144750.1 | 1493 | 358 | 779 | 28.27 | 658.5 | 0 | 401/423 | 94 | 2/423 | 0 | Plus | Plus | Bacteria; Bacteroidetes; Bacteroidia; Bacteroidales; Prevotellaceae; Prevotellamassilia; Prevotellamassilia timonensis | Prevotellamassilia timonensis | Prevotellamassilia | Prevotellaceae | Bacteroidales | Bacteroidia | Bacteroidetes | Bacteria |
| ASV1843 | 427 | 1 | 1 | 427 | 100 | NR_115212.1 Actinobacillus porcinus strain CCUG 38924 16S ribosomal RNA, partial sequence | NR_115212.1 | 1364 | 333 | 759 | 31.3 | 767.5 | 0 | 422/427 | 98 | 0/427 | 0 | Plus | Plus | Bacteria; Proteobacteria; Gammaproteobacteria; Pasteurellales; Pasteurellaceae; Actinobacillus; Actinobacillus porcinus | Actinobacillus porcinus | Actinobacillus | Pasteurellaceae | Pasteurellales | Gammaproteobacteria | Proteobacteria | Bacteria |
| ASV1844 | 422 | 1 | 1 | 422 | 100 | NR_041285.1 Prevotella multisaccharivorax strain PPPA20 16S ribosomal RNA, partial sequence | NR_041285.1 | 1491 | 356 | 777 | 28.3 | 619.7 | 3E-177 | 394/423 | 93 | 2/423 | 0 | Plus | Plus | Bacteria; Bacteroidetes; Bacteroidia; Bacteroidales; Prevotellaceae; Prevotella; Prevotella multisaccharivorax | Prevotella multisaccharivorax | Prevotella | Prevotellaceae | Bacteroidales | Bacteroidia | Bacteroidetes | Bacteria |
| ASV1845 | 402 | 2 | 1 | 402 | 100 | NR_104899.1 Bacteroides xylanolyticus strain X5-1 16S ribosomal RNA, partial sequence | NR_104899.1 | 1480 | 345 | 746 | 27.16 | 688.1 | 0 | 392/402 | 97 | 0/402 | 0 | Plus | Plus | Bacteria; Firmicutes; Clostridia; Eubacteriales; Clostridiaceae; Hungatella; Hungatella xylanolytica | Hungatella xylanolytica | Hungatella | Clostridiaceae | Eubacteriales | Clostridia | Firmicutes | Bacteria |
| ASV1846 | 422 | 3 | 1 | 422 | 100 | NR_040866.1 Bacteroides uniformis strain JCM 5828 16S ribosomal RNA, partial sequence | NR_040866.1 | 1425 | 351 | 772 | 29.61 | 680.7 | 0 | 405/423 | 95 | 2/423 | 0 | Plus | Plus | Bacteria; Bacteroidetes; Bacteroidia; Bacteroidales; Bacteroidaceae; Bacteroides; Bacteroides uniformis | Bacteroides uniformis | Bacteroides | Bacteroidaceae | Bacteroidales | Bacteroidia | Bacteroidetes | Bacteria |
| ASV1847 | 422 | 1 | 1 | 422 | 100 | NR_041364.1 Prevotella stercorea DSM 18206 strain CB35 16S ribosomal RNA, partial sequence | NR_041364.1 | 1488 | 354 | 775 | 28.36 | 713.9 | 0 | 410/422 | 97 | 0/422 | 0 | Plus | Plus | Bacteria; Bacteroidetes; Bacteroidia; Bacteroidales; Prevotellaceae; Prevotella; Prevotella stercorea | Prevotella stercorea | Prevotella | Prevotellaceae | Bacteroidales | Bacteroidia | Bacteroidetes | Bacteria |
| ASV1848 | 419 | 2 | 1 | 419 | 100 | NR_043442.2 Bifidobacterium pseudolongum strain PNC-2-9G 16S ribosomal RNA, partial sequence | NR_043442.2 | 1525 | 347 | 765 | 27.48 | 774.9 | 0 | 419/419 | 100 | 0/419 | 0 | Plus | Plus | Bacteria; Actinobacteria; Actinomycetia; Bifidobacteriales; Bifidobacteriaceae; Bifidobacterium; Bifidobacterium pseudolongum | Bifidobacterium pseudolongum | Bifidobacterium | Bifidobacteriaceae | Bifidobacteriales | Actinomycetia | Actinobacteria | Bacteria |
| ASV1849 | 402 | 4 | 1 | 402 | 100 | NR_114392.1 Faecalicatena orotica strain JCM 1429 16S ribosomal RNA, partial sequence | NR_114392.1 | 1494 | 367 | 768 | 26.91 | 704.7 | 0 | 395/402 | 98 | 0/402 | 0 | Plus | Plus | Bacteria; Firmicutes; Clostridia; Eubacteriales; Lachnospiraceae; Faecalicatena; Faecalicatena orotica | Faecalicatena orotica | Faecalicatena | Lachnospiraceae | Eubacteriales | Clostridia | Firmicutes | Bacteria |
| ASV1850 | 422 | 1 | 1 | 422 | 100 | NR_024815.1 Prevotella shahii strain EHS11 16S ribosomal RNA, partial sequence | NR_024815.1 | 1490 | 356 | 777 | 28.32 | 603.1 | 3E-172 | 392/424 | 92 | 4/424 | 0 | Plus | Plus | Bacteria; Bacteroidetes; Bacteroidia; Bacteroidales; Prevotellaceae; Prevotella; Prevotella shahii | Prevotella shahii | Prevotella | Prevotellaceae | Bacteroidales | Bacteroidia | Bacteroidetes | Bacteria |
| ASV1851 | 421 | 1 | 1 | 421 | 100 | NR_144616.1 Muribaculum intestinale strain YL27 16S ribosomal RNA, partial sequence | NR_144616.1 | 1460 | 297 | 718 | 28.9 | 507.1 | 3E-143 | 373/422 | 88 | 1/422 | 0 | Plus | Plus | Bacteria; Bacteroidetes; Bacteroidia; Bacteroidales; Muribaculaceae; Muribaculum; Muribaculum intestinale | Muribaculum intestinale | Muribaculum | Muribaculaceae | Bacteroidales | Bacteroidia | Bacteroidetes | Bacteria |
| ASV1852 | 405 | 1 | 1 | 405 | 100 | NR_144742.1 Christensenella massiliensis strain Marseille-P2438 16S ribosomal RNA, partial sequence | NR_144742.1 | 1499 | 369 | 773 | 27.02 | 472 | 1E-132 | 356/406 | 87 | 2/406 | 0 | Plus | Plus | Bacteria; Firmicutes; Clostridia; Eubacteriales; Christensenellaceae; Christensenella; Christensenella massiliensis | Christensenella massiliensis | Christensenella | Christensenellaceae | Eubacteriales | Clostridia | Firmicutes | Bacteria |
| ASV1853 | 405 | 1 | 1 | 405 | 100 | NR_145929.1 Caproiciproducens galactitolivorans strain BS-1 16S ribosomal RNA, partial sequence | NR_145929.1 | 1445 | 336 | 741 | 28.1 | 538.5 | 9E-153 | 372/410 | 90 | 9/410 | 2 | Plus | Plus | Bacteria; Firmicutes; Clostridia; Eubacteriales; Oscillospiraceae; Caproiciproducens; Caproiciproducens galactitolivorans | Caproiciproducens galactitolivorans | Caproiciproducens | Oscillospiraceae | Eubacteriales | Clostridia | Firmicutes | Bacteria |
| ASV1854 | 403 | 1 | 1 | 403 | 100 | NR_104907.1 Eubacterium coprostanoligenes strain HL 16S ribosomal RNA, partial sequence | NR_104907.1 | 1446 | 332 | 734 | 27.87 | 640.1 | 0 | 385/404 | 95 | 2/404 | 0 | Plus | Plus | Bacteria; Firmicutes; Clostridia; Eubacteriales; Eubacteriaceae; Eubacterium; Eubacterium coprostanoligenes | Eubacterium coprostanoligenes | Eubacterium | Eubacteriaceae | Eubacteriales | Clostridia | Firmicutes | Bacteria |
| ASV1855 | 403 | 1 | 3 | 403 | 99.5 | NR_102987.1 Hungateiclostridium clariflavum DSM 19732 16S ribosomal RNA, partial sequence | NR_102987.1 | 1634 | 483 | 882 | 24.48 | 468.3 | 1E-131 | 352/401 | 87 | 1/401 | 0 | Plus | Plus | Bacteria; Firmicutes; Clostridia; Eubacteriales; Oscillospiraceae; Acetivibrio; Acetivibrio clariflavus | Acetivibrio clariflavus | Acetivibrio | Oscillospiraceae | Eubacteriales | Clostridia | Firmicutes | Bacteria |
| ASV1856 | 402 | 2 | 1 | 402 | 100 | NR_156911.1 Fournierella massiliensis strain AT2 16S ribosomal RNA, partial sequence | NR_156911.1 | 1491 | 368 | 769 | 26.96 | 632.7 | 0 | 383/403 | 95 | 2/403 | 0 | Plus | Plus | Bacteria; Firmicutes; Clostridia; Eubacteriales; Oscillospiraceae; Fournierella; Fournierella massiliensis | Fournierella massiliensis | Fournierella | Oscillospiraceae | Eubacteriales | Clostridia | Firmicutes | Bacteria |
| ASV1857 | 403 | 1 | 3 | 403 | 99.5 | NR_026476.1 Succinivibrio dextrinosolvens strain 0554 16S ribosomal RNA, partial sequence | NR_026476.1 | 1463 | 333 | 732 | 27.34 | 579.1 | 6E-165 | 373/402 | 92 | 3/402 | 0 | Plus | Plus | Bacteria; Proteobacteria; Gammaproteobacteria; Aeromonadales; Succinivibrionaceae; Succinivibrio; Succinivibrio dextrinosolvens | Succinivibrio dextrinosolvens | Succinivibrio | Succinivibrionaceae | Aeromonadales | Gammaproteobacteria | Proteobacteria | Bacteria |
| ASV1858 | 427 | 2 | 1 | 426 | 99.77 | NR_027596.1 Mitsuokella multacida strain DSM 20544 16S ribosomal RNA, partial sequence | NR_027596.1 | 1511 | 346 | 771 | 28.19 | 651.1 | 0 | 402/427 | 94 | 2/427 | 0 | Plus | Plus | Bacteria; Firmicutes; Negativicutes; Selenomonadales; Selenomonadaceae; Mitsuokella; Mitsuokella multacida | Mitsuokella multacida | Mitsuokella | Selenomonadaceae | Selenomonadales | Negativicutes | Firmicutes | Bacteria |
| ASV1859 | 403 | 1 | 1 | 403 | 100 | NR_102987.1 Hungateiclostridium clariflavum DSM 19732 16S ribosomal RNA, partial sequence | NR_102987.1 | 1634 | 481 | 882 | 24.6 | 605 | 9E-173 | 378/403 | 93 | 1/403 | 0 | Plus | Plus | Bacteria; Firmicutes; Clostridia; Eubacteriales; Oscillospiraceae; Acetivibrio; Acetivibrio clariflavus | Acetivibrio clariflavus | Acetivibrio | Oscillospiraceae | Eubacteriales | Clostridia | Firmicutes | Bacteria |
| ASV1860 | 404 | 1 | 1 | 404 | 100 | NR_118156.1 Oscillibacter ruminantium GH1 16S ribosomal RNA, partial sequence | NR_118156.1 | 1409 | 321 | 724 | 28.67 | 608.7 | 7E-174 | 380/405 | 93 | 2/405 | 0 | Plus | Plus | Bacteria; Firmicutes; Clostridia; Eubacteriales; Oscillospiraceae; Oscillibacter; Oscillibacter ruminantium | Oscillibacter ruminantium | Oscillibacter | Oscillospiraceae | Eubacteriales | Clostridia | Firmicutes | Bacteria |
| ASV1861 | 422 | 2 | 1 | 422 | 100 | NR_113119.1 Prevotella oris strain JCM 8540 16S ribosomal RNA, partial sequence | NR_113119.1 | 1481 | 345 | 766 | 28.49 | 569.9 | 3E-162 | 387/425 | 91 | 6/425 | 1 | Plus | Plus | Bacteria; Bacteroidetes; Bacteroidia; Bacteroidales; Prevotellaceae; Prevotella; Prevotella oris | Prevotella oris | Prevotella | Prevotellaceae | Bacteroidales | Bacteroidia | Bacteroidetes | Bacteria |
| ASV1862 | 404 | 1 | 1 | 404 | 100 | NR_118156.1 Oscillibacter ruminantium GH1 16S ribosomal RNA, partial sequence | NR_118156.1 | 1409 | 321 | 724 | 28.67 | 658.5 | 0 | 390/406 | 96 | 4/406 | 0 | Plus | Plus | Bacteria; Firmicutes; Clostridia; Eubacteriales; Oscillospiraceae; Oscillibacter; Oscillibacter ruminantium | Oscillibacter ruminantium | Oscillibacter | Oscillospiraceae | Eubacteriales | Clostridia | Firmicutes | Bacteria |
| ASV1863 | 405 | 5 | 1 | 405 | 100 | NR_115693.1 Gracilibacter thermotolerans JW/YJL-S1 16S ribosomal RNA, partial sequence | NR_115693.1 | 1526 | 459 | 861 | 26.41 | 525.6 | 7E-149 | 365/405 | 90 | 2/405 | 0 | Plus | Plus | Bacteria; Firmicutes; Clostridia; Eubacteriales; Gracilibacteraceae; Gracilibacter; Gracilibacter thermotolerans | Gracilibacter thermotolerans | Gracilibacter | Gracilibacteraceae | Eubacteriales | Clostridia | Firmicutes | Bacteria |
| ASV1864 | 421 | 1 | 1 | 421 | 100 | NR_144746.1 Marseilla massiliensis strain Marseille-P2475 16S ribosomal RNA, partial sequence | NR_144746.1 | 1494 | 356 | 777 | 28.25 | 436.9 | 4E-122 | 366/428 | 85 | 13/428 | 3 | Plus | Plus | Bacteria; Bacteroidetes; Bacteroidia; Bacteroidales; Prevotellaceae; Marseilla; Marseilla massiliensis | Marseilla massiliensis | Marseilla | Prevotellaceae | Bacteroidales | Bacteroidia | Bacteroidetes | Bacteria |
| ASV1865 | 405 | 1 | 1 | 405 | 100 | NR_044972.1 Sporobacter termitidis strain SYR 16S ribosomal RNA, partial sequence | NR_044972.1 | 1506 | 348 | 752 | 26.89 | 536.6 | 3E-152 | 367/406 | 90 | 2/406 | 0 | Plus | Plus | Bacteria; Firmicutes; Clostridia; Eubacteriales; Oscillospiraceae; Sporobacter; Sporobacter termitidis | Sporobacter termitidis | Sporobacter | Oscillospiraceae | Eubacteriales | Clostridia | Firmicutes | Bacteria |
| ASV1866 | 402 | 1 | 1 | 402 | 100 | NR_042200.1 [Clostridium] asparagiforme strain N6 16S ribosomal RNA, partial sequence | NR_042200.1 | 1468 | 335 | 736 | 27.38 | 693.6 | 0 | 393/402 | 97 | 0/402 | 0 | Plus | Plus | Bacteria; Firmicutes; Clostridia; Eubacteriales; Lachnospiraceae; Enterocloster; Enterocloster asparagiformis | Enterocloster asparagiformis | Enterocloster | Lachnospiraceae | Eubacteriales | Clostridia | Firmicutes | Bacteria |
| ASV1867 | 422 | 1 | 1 | 422 | 100 | NR_027196.1 Bacteroides stercoris ATCC 43183 16S ribosomal RNA, partial sequence | NR_027196.1 | 1342 | 334 | 755 | 31.45 | 671.5 | 0 | 402/422 | 95 | 0/422 | 0 | Plus | Plus | Bacteria; Bacteroidetes; Bacteroidia; Bacteroidales; Bacteroidaceae; Bacteroides; Bacteroides stercoris | Bacteroides stercoris | Bacteroides | Bacteroidaceae | Bacteroidales | Bacteroidia | Bacteroidetes | Bacteria |
| ASV1868 | 422 | 1 | 1 | 422 | 100 | NR_041364.1 Prevotella stercorea DSM 18206 strain CB35 16S ribosomal RNA, partial sequence | NR_041364.1 | 1488 | 354 | 775 | 28.36 | 597.6 | 2E-170 | 389/422 | 92 | 0/422 | 0 | Plus | Plus | Bacteria; Bacteroidetes; Bacteroidia; Bacteroidales; Prevotellaceae; Prevotella; Prevotella stercorea | Prevotella stercorea | Prevotella | Prevotellaceae | Bacteroidales | Bacteroidia | Bacteroidetes | Bacteria |
| ASV1869 | 401 | 1 | 1 | 401 | 100 | NR_144749.1 Ihubacter massiliensis strain Marseille-P2843 16S ribosomal RNA, partial sequence | NR_144749.1 | 1479 | 364 | 766 | 27.25 | 577.3 | 2E-164 | 374/404 | 92 | 4/404 | 0 | Plus | Plus | Bacteria; Firmicutes; Clostridia; Eubacteriales; Clostridiales Family XIII. Incertae Sedis; Ihubacter; Ihubacter massiliensis | Ihubacter massiliensis | Ihubacter | Clostridiales Family XIII. Incertae Sedis | Eubacteriales | Clostridia | Firmicutes | Bacteria |
| ASV1870 | 427 | 1 | 1 | 427 | 100 | NR_044111.1 Selenomonas bovis strain WG 16S ribosomal RNA, partial sequence | NR_044111.1 | 1525 | 370 | 796 | 28 | 773 | 0 | 424/427 | 99 | 0/427 | 0 | Plus | Plus | Bacteria; Firmicutes; Negativicutes; Selenomonadales; Selenomonadaceae; Selenomonas; Selenomonas bovis | Selenomonas bovis | Selenomonas | Selenomonadaceae | Selenomonadales | Negativicutes | Firmicutes | Bacteria |
| ASV1871 | 427 | 1 | 1 | 427 | 100 | NR_044111.1 Selenomonas bovis strain WG 16S ribosomal RNA, partial sequence | NR_044111.1 | 1525 | 370 | 796 | 28 | 662.2 | 0 | 406/429 | 94 | 4/429 | 0 | Plus | Plus | Bacteria; Firmicutes; Negativicutes; Selenomonadales; Selenomonadaceae; Selenomonas; Selenomonas bovis | Selenomonas bovis | Selenomonas | Selenomonadaceae | Selenomonadales | Negativicutes | Firmicutes | Bacteria |
| ASV1872 | 405 | 1 | 1 | 405 | 100 | NR_044972.1 Sporobacter termitidis strain SYR 16S ribosomal RNA, partial sequence | NR_044972.1 | 1506 | 348 | 752 | 26.89 | 525.6 | 7E-149 | 365/406 | 89 | 2/406 | 0 | Plus | Plus | Bacteria; Firmicutes; Clostridia; Eubacteriales; Oscillospiraceae; Sporobacter; Sporobacter termitidis | Sporobacter termitidis | Sporobacter | Oscillospiraceae | Eubacteriales | Clostridia | Firmicutes | Bacteria |
| ASV1873 | 422 | 1 | 1 | 422 | 100 | NR_113411.1 Prevotella copri DSM 18205 strain JCM 13464 16S ribosomal RNA, partial sequence | NR_113411.1 | 1493 | 358 | 779 | 28.27 | 752.7 | 0 | 417/422 | 98 | 0/422 | 0 | Plus | Plus | Bacteria; Bacteroidetes; Bacteroidia; Bacteroidales; Prevotellaceae; Prevotella; Prevotella copri | Prevotella copri | Prevotella | Prevotellaceae | Bacteroidales | Bacteroidia | Bacteroidetes | Bacteria |
| ASV1874 | 401 | 1 | 1 | 401 | 100 | NR_144740.1 Romboutsia timonensis strain DR1 16S ribosomal RNA, partial sequence | NR_144740.1 | 1466 | 338 | 738 | 27.35 | 736.1 | 0 | 400/401 | 99 | 0/401 | 0 | Plus | Plus | Bacteria; Firmicutes; Clostridia; Eubacteriales; Peptostreptococcaceae; Romboutsia; Romboutsia timonensis | Romboutsia timonensis | Romboutsia | Peptostreptococcaceae | Eubacteriales | Clostridia | Firmicutes | Bacteria |
| ASV1875 | 402 | 1 | 1 | 402 | 100 | NR_028961.1 Faecalibacterium prausnitzii strain ATCC 27768 16S ribosomal RNA, partial sequence | NR_028961.1 | 1462 | 344 | 745 | 27.5 | 610.5 | 2E-174 | 379/403 | 94 | 2/403 | 0 | Plus | Plus | Bacteria; Firmicutes; Clostridia; Eubacteriales; Oscillospiraceae; Faecalibacterium; Faecalibacterium prausnitzii | Faecalibacterium prausnitzii | Faecalibacterium | Oscillospiraceae | Eubacteriales | Clostridia | Firmicutes | Bacteria |
| ASV1876 | 422 | 1 | 1 | 422 | 100 | NR_041364.1 Prevotella stercorea DSM 18206 strain CB35 16S ribosomal RNA, partial sequence | NR_041364.1 | 1488 | 354 | 775 | 28.36 | 558.8 | 8E-159 | 383/423 | 90 | 2/423 | 0 | Plus | Plus | Bacteria; Bacteroidetes; Bacteroidia; Bacteroidales; Prevotellaceae; Prevotella; Prevotella stercorea | Prevotella stercorea | Prevotella | Prevotellaceae | Bacteroidales | Bacteroidia | Bacteroidetes | Bacteria |
| ASV1877 | 426 | 3 | 1 | 426 | 100 | NR_102964.1 Sphaerochaeta pleomorpha strain Grapes 16S ribosomal RNA, partial sequence | NR_102964.1 | 1529 | 350 | 776 | 27.93 | 510.8 | 2E-144 | 377/427 | 88 | 1/427 | 0 | Plus | Plus | Bacteria; Spirochaetes; Spirochaetia; Spirochaetales; Spirochaetaceae; Sphaerochaeta; Sphaerochaeta pleomorpha | Sphaerochaeta pleomorpha | Sphaerochaeta | Spirochaetaceae | Spirochaetales | Spirochaetia | Spirochaetes | Bacteria |
| ASV1878 | 404 | 1 | 1 | 404 | 100 | NR_118156.1 Oscillibacter ruminantium GH1 16S ribosomal RNA, partial sequence | NR_118156.1 | 1409 | 321 | 724 | 28.67 | 686.2 | 0 | 393/404 | 97 | 0/404 | 0 | Plus | Plus | Bacteria; Firmicutes; Clostridia; Eubacteriales; Oscillospiraceae; Oscillibacter; Oscillibacter ruminantium | Oscillibacter ruminantium | Oscillibacter | Oscillospiraceae | Eubacteriales | Clostridia | Firmicutes | Bacteria |
| ASV1879 | 405 | 1 | 1 | 405 | 100 | NR_144611.1 Flintibacter butyricus strain BLS21 16S ribosomal RNA, partial sequence | NR_144611.1 | 808 | 299 | 703 | 50.12 | 721.3 | 0 | 400/405 | 98 | 0/405 | 0 | Plus | Plus | Bacteria; Firmicutes; Clostridia; Eubacteriales; ; Flintibacter; Flintibacter butyricus | Flintibacter butyricus | Flintibacter | | Eubacteriales | Clostridia | Firmicutes | Bacteria |
| ASV1880 | 422 | 1 | 1 | 422 | 100 | NR_041342.1 Parabacteroides distasonis strain JCM 5825 16S ribosomal RNA, partial sequence | NR_041342.1 | 1488 | 353 | 774 | 28.36 | 763.8 | 0 | 419/422 | 99 | 0/422 | 0 | Plus | Plus | Bacteria; Bacteroidetes; Bacteroidia; Bacteroidales; Tannerellaceae; Parabacteroides; Parabacteroides distasonis | Parabacteroides distasonis | Parabacteroides | Tannerellaceae | Bacteroidales | Bacteroidia | Bacteroidetes | Bacteria |
| ASV1881 | 421 | 2 | 1 | 421 | 100 | NR_113073.1 Barnesiella intestinihominis strain JCM 15079 16S ribosomal RNA, partial sequence | NR_113073.1 | 1481 | 346 | 767 | 28.49 | 540.3 | 3E-153 | 381/424 | 89 | 5/424 | 1 | Plus | Plus | Bacteria; Bacteroidetes; Bacteroidia; Bacteroidales; Barnesiellaceae; Barnesiella; Barnesiella intestinihominis | Barnesiella intestinihominis | Barnesiella | Barnesiellaceae | Bacteroidales | Bacteroidia | Bacteroidetes | Bacteria |
| ASV1882 | 402 | 1 | 1 | 402 | 100 | NR_044022.2 Howardella ureilytica strain GPC 589 16S ribosomal RNA, partial sequence | NR_044022.2 | 1514 | 366 | 767 | 26.55 | 743.5 | 0 | 402/402 | 100 | 0/402 | 0 | Plus | Plus | Bacteria; Firmicutes; Clostridia; Eubacteriales; ; Howardella; Howardella ureilytica | Howardella ureilytica | Howardella | | Eubacteriales | Clostridia | Firmicutes | Bacteria |
| ASV1883 | 421 | 1 | 1 | 421 | 100 | NR_144616.1 Muribaculum intestinale strain YL27 16S ribosomal RNA, partial sequence | NR_144616.1 | 1460 | 297 | 718 | 28.9 | 529.3 | 6E-150 | 381/426 | 89 | 9/426 | 2 | Plus | Plus | Bacteria; Bacteroidetes; Bacteroidia; Bacteroidales; Muribaculaceae; Muribaculum; Muribaculum intestinale | Muribaculum intestinale | Muribaculum | Muribaculaceae | Bacteroidales | Bacteroidia | Bacteroidetes | Bacteria |
| ASV1884 | 403 | 1 | 1 | 401 | 99.5 | NR_104911.1 Vampirovibrio chlorellavorus strain ICPB 3707 16S ribosomal RNA, partial sequence | NR_104911.1 | 1446 | 333 | 732 | 27.66 | 424 | 3E-118 | 346/403 | 85 | 5/403 | 1 | Plus | Plus | Bacteria; Candidatus Melainabacteria; ; Vampirovibrionales; ; Vampirovibrio; Vampirovibrio chlorellavorus | Vampirovibrio chlorellavorus | Vampirovibrio | | Vampirovibrionales | | Candidatus Melainabacteria | Bacteria |
| ASV1885 | 422 | 1 | 1 | 422 | 100 | NR_113411.1 Prevotella copri DSM 18205 strain JCM 13464 16S ribosomal RNA, partial sequence | NR_113411.1 | 1493 | 358 | 779 | 28.27 | 747.2 | 0 | 416/422 | 98 | 0/422 | 0 | Plus | Plus | Bacteria; Bacteroidetes; Bacteroidia; Bacteroidales; Prevotellaceae; Prevotella; Prevotella copri | Prevotella copri | Prevotella | Prevotellaceae | Bacteroidales | Bacteroidia | Bacteroidetes | Bacteria |
| ASV1886 | 403 | 5 | 3 | 403 | 99.5 | NR_115693.1 Gracilibacter thermotolerans JW/YJL-S1 16S ribosomal RNA, partial sequence | NR_115693.1 | 1526 | 461 | 861 | 26.28 | 453.5 | 3E-127 | 349/401 | 87 | 0/401 | 0 | Plus | Plus | Bacteria; Firmicutes; Clostridia; Eubacteriales; Gracilibacteraceae; Gracilibacter; Gracilibacter thermotolerans | Gracilibacter thermotolerans | Gracilibacter | Gracilibacteraceae | Eubacteriales | Clostridia | Firmicutes | Bacteria |
| ASV1887 | 422 | 2 | 1 | 422 | 100 | NR_074376.1 Parabacteroides distasonis ATCC 8503 16S ribosomal RNA, partial sequence | NR_074376.1 | 1497 | 368 | 789 | 28.19 | 553.3 | 4E-157 | 381/422 | 90 | 0/422 | 0 | Plus | Plus | Bacteria; Bacteroidetes; Bacteroidia; Bacteroidales; Tannerellaceae; Parabacteroides; Parabacteroides distasonis | Parabacteroides distasonis | Parabacteroides | Tannerellaceae | Bacteroidales | Bacteroidia | Bacteroidetes | Bacteria |
| ASV1889 | 403 | 1 | 3 | 403 | 99.5 | NR_125677.1 Vallitalea pronyensis strain FatNI3 16S ribosomal RNA, partial sequence | NR_125677.1 | 1480 | 365 | 764 | 27.03 | 479.4 | 6E-135 | 356/403 | 88 | 5/403 | 1 | Plus | Plus | Bacteria; Firmicutes; Clostridia; Eubacteriales; Vallitaleaceae; Vallitalea; Vallitalea pronyensis | Vallitalea pronyensis | Vallitalea | Vallitaleaceae | Eubacteriales | Clostridia | Firmicutes | Bacteria |
| ASV1890 | 427 | 2 | 1 | 427 | 100 | NR_152055.1 Pontibacillus salipaludis strain 9DM 16S ribosomal RNA, partial sequence | NR_152055.1 | 1476 | 329 | 755 | 28.93 | 490.5 | 3E-138 | 375/429 | 87 | 4/429 | 0 | Plus | Plus | Bacteria; Firmicutes; Bacilli; Bacillales; Bacillaceae; Pontibacillus; Pontibacillus salipaludis | Pontibacillus salipaludis | Pontibacillus | Bacillaceae | Bacillales | Bacilli | Firmicutes | Bacteria |
| ASV1891 | 422 | 2 | 1 | 422 | 100 | NR_113117.1 Prevotella oralis ATCC 33269 strain JCM 12251 16S ribosomal RNA, partial sequence | NR_113117.1 | 1496 | 361 | 782 | 28.21 | 608.7 | 7E-174 | 392/423 | 92 | 2/423 | 0 | Plus | Plus | Bacteria; Bacteroidetes; Bacteroidia; Bacteroidales; Prevotellaceae; Prevotella; Prevotella oralis | Prevotella oralis | Prevotella | Prevotellaceae | Bacteroidales | Bacteroidia | Bacteroidetes | Bacteria |
| ASV1893 | 422 | 1 | 1 | 422 | 100 | NR_113411.1 Prevotella copri DSM 18205 strain JCM 13464 16S ribosomal RNA, partial sequence | NR_113411.1 | 1493 | 358 | 779 | 28.27 | 702.8 | 0 | 409/423 | 96 | 2/423 | 0 | Plus | Plus | Bacteria; Bacteroidetes; Bacteroidia; Bacteroidales; Prevotellaceae; Prevotella; Prevotella copri | Prevotella copri | Prevotella | Prevotellaceae | Bacteroidales | Bacteroidia | Bacteroidetes | Bacteria |
| ASV1894 | 426 | 1 | 1 | 426 | 100 | NR_144615.1 Longibaculum muris strain MT10-315-CC-1.2-2 16S ribosomal RNA, partial sequence | NR_144615.1 | 1465 | 333 | 758 | 29.08 | 693.6 | 0 | 409/426 | 96 | 0/426 | 0 | Plus | Plus | Bacteria; Firmicutes; Erysipelotrichia; Erysipelotrichales; Erysipelotrichaceae; Longibaculum; Longibaculum muris | Longibaculum muris | Longibaculum | Erysipelotrichaceae | Erysipelotrichales | Erysipelotrichia | Firmicutes | Bacteria |
| ASV1895 | 402 | 4 | 1 | 402 | 100 | NR_074399.1 Ruminococcus albus 7 = DSM 20455 16S ribosomal RNA, partial sequence | NR_074399.1 | 1500 | 350 | 751 | 26.8 | 599.4 | 4E-171 | 376/402 | 93 | 0/402 | 0 | Plus | Plus | Bacteria; Firmicutes; Clostridia; Eubacteriales; Oscillospiraceae; Ruminococcus; Ruminococcus albus | Ruminococcus albus | Ruminococcus | Oscillospiraceae | Eubacteriales | Clostridia | Firmicutes | Bacteria |
| ASV1896 | 402 | 1 | 1 | 402 | 100 | NR_102884.1 Ruminococcus champanellensis 18P13 = JCM 17042 16S ribosomal RNA, partial sequence | NR_102884.1 | 1494 | 348 | 749 | 26.91 | 649.3 | 0 | 386/403 | 95 | 2/403 | 0 | Plus | Plus | Bacteria; Firmicutes; Clostridia; Eubacteriales; Oscillospiraceae; Ruminococcus; Ruminococcus champanellensis | Ruminococcus champanellensis | Ruminococcus | Oscillospiraceae | Eubacteriales | Clostridia | Firmicutes | Bacteria |
| ASV1897 | 427 | 1 | 1 | 427 | 100 | NR_044111.1 Selenomonas bovis strain WG 16S ribosomal RNA, partial sequence | NR_044111.1 | 1525 | 370 | 796 | 28 | 673.3 | 0 | 406/427 | 95 | 0/427 | 0 | Plus | Plus | Bacteria; Firmicutes; Negativicutes; Selenomonadales; Selenomonadaceae; Selenomonas; Selenomonas bovis | Selenomonas bovis | Selenomonas | Selenomonadaceae | Selenomonadales | Negativicutes | Firmicutes | Bacteria |
| ASV1899 | 404 | 3 | 1 | 404 | 100 | NR_115307.1 Ethanoligenens harbinense YUAN-3 16S ribosomal RNA, partial sequence | NR_115307.1 | 1497 | 340 | 743 | 26.99 | 564.3 | 2E-160 | 373/406 | 91 | 4/406 | 0 | Plus | Plus | Bacteria; Firmicutes; Clostridia; Eubacteriales; Oscillospiraceae; Ethanoligenens; Ethanoligenens harbinense | Ethanoligenens harbinense | Ethanoligenens | Oscillospiraceae | Eubacteriales | Clostridia | Firmicutes | Bacteria |
| ASV1900 | 423 | 1 | 1 | 423 | 100 | NR_144750.1 Prevotellamassilia timonensis strain Marseille-P2831 16S ribosomal RNA, partial sequence | NR_144750.1 | 1493 | 358 | 779 | 28.27 | 564.3 | 2E-160 | 387/426 | 90 | 7/426 | 1 | Plus | Plus | Bacteria; Bacteroidetes; Bacteroidia; Bacteroidales; Prevotellaceae; Prevotellamassilia; Prevotellamassilia timonensis | Prevotellamassilia timonensis | Prevotellamassilia | Prevotellaceae | Bacteroidales | Bacteroidia | Bacteroidetes | Bacteria |
| ASV1901 | 421 | 2 | 1 | 421 | 100 | NR_113073.1 Barnesiella intestinihominis strain JCM 15079 16S ribosomal RNA, partial sequence | NR_113073.1 | 1481 | 346 | 767 | 28.49 | 529.3 | 6E-150 | 379/424 | 89 | 5/424 | 1 | Plus | Plus | Bacteria; Bacteroidetes; Bacteroidia; Bacteroidales; Barnesiellaceae; Barnesiella; Barnesiella intestinihominis | Barnesiella intestinihominis | Barnesiella | Barnesiellaceae | Bacteroidales | Bacteroidia | Bacteroidetes | Bacteria |
| ASV1902 | 422 | 2 | 1 | 422 | 100 | NR_113119.1 Prevotella oris strain JCM 8540 16S ribosomal RNA, partial sequence | NR_113119.1 | 1481 | 345 | 766 | 28.49 | 581 | 2E-165 | 387/423 | 91 | 2/423 | 0 | Plus | Plus | Bacteria; Bacteroidetes; Bacteroidia; Bacteroidales; Prevotellaceae; Prevotella; Prevotella oris | Prevotella oris | Prevotella | Prevotellaceae | Bacteroidales | Bacteroidia | Bacteroidetes | Bacteria |
| ASV1903 | 402 | 3 | 1 | 402 | 100 | NR_156081.1 Kineothrix alysoides strain KNHs209 16S ribosomal RNA, partial sequence | NR_156081.1 | 1411 | 353 | 754 | 28.49 | 693.6 | 0 | 393/402 | 97 | 0/402 | 0 | Plus | Plus | Bacteria; Firmicutes; Clostridia; Eubacteriales; Lachnospiraceae; Kineothrix; Kineothrix alysoides | Kineothrix alysoides | Kineothrix | Lachnospiraceae | Eubacteriales | Clostridia | Firmicutes | Bacteria |
| ASV1904 | 402 | 5 | 1 | 402 | 100 | NR_156081.1 Kineothrix alysoides strain KNHs209 16S ribosomal RNA, partial sequence | NR_156081.1 | 1411 | 353 | 754 | 28.49 | 688.1 | 0 | 392/402 | 97 | 0/402 | 0 | Plus | Plus | Bacteria; Firmicutes; Clostridia; Eubacteriales; Lachnospiraceae; Kineothrix; Kineothrix alysoides | Kineothrix alysoides | Kineothrix | Lachnospiraceae | Eubacteriales | Clostridia | Firmicutes | Bacteria |
| ASV1905 | 402 | 1 | 1 | 402 | 100 | NR_102884.1 Ruminococcus champanellensis 18P13 = JCM 17042 16S ribosomal RNA, partial sequence | NR_102884.1 | 1494 | 348 | 749 | 26.91 | 627.1 | 2E-179 | 381/402 | 94 | 0/402 | 0 | Plus | Plus | Bacteria; Firmicutes; Clostridia; Eubacteriales; Oscillospiraceae; Ruminococcus; Ruminococcus champanellensis | Ruminococcus champanellensis | Ruminococcus | Oscillospiraceae | Eubacteriales | Clostridia | Firmicutes | Bacteria |
| ASV1906 | 404 | 1 | 1 | 404 | 100 | NR_118156.1 Oscillibacter ruminantium GH1 16S ribosomal RNA, partial sequence | NR_118156.1 | 1409 | 321 | 724 | 28.67 | 625.3 | 7E-179 | 383/405 | 94 | 2/405 | 0 | Plus | Plus | Bacteria; Firmicutes; Clostridia; Eubacteriales; Oscillospiraceae; Oscillibacter; Oscillibacter ruminantium | Oscillibacter ruminantium | Oscillibacter | Oscillospiraceae | Eubacteriales | Clostridia | Firmicutes | Bacteria |
| ASV1907 | 402 | 1 | 1 | 402 | 100 | NR_147375.1 Colidextribacter massiliensis strain Marseille-P3083 16S ribosomal RNA, partial sequence | NR_147375.1 | 1487 | 364 | 765 | 27.03 | 593.9 | 2E-169 | 375/402 | 93 | 0/402 | 0 | Plus | Plus | Bacteria; Firmicutes; Clostridia; Eubacteriales; ; Colidextribacter; Colidextribacter massiliensis | Colidextribacter massiliensis | Colidextribacter | | Eubacteriales | Clostridia | Firmicutes | Bacteria |
| ASV1908 | 405 | 1 | 1 | 405 | 100 | NR_146837.2 Fusobacterium gastrosuis strain CDW1 16S ribosomal RNA, complete sequence | NR_146837.2 | 1506 | 343 | 747 | 26.89 | 749 | 0 | 405/405 | 100 | 0/405 | 0 | Plus | Plus | Bacteria; Fusobacteria; Fusobacteriia; Fusobacteriales; Fusobacteriaceae; Fusobacterium; Fusobacterium gastrosuis | Fusobacterium gastrosuis | Fusobacterium | Fusobacteriaceae | Fusobacteriales | Fusobacteriia | Fusobacteria | Bacteria |
| ASV1909 | 422 | 1 | 1 | 422 | 100 | NR_027196.1 Bacteroides stercoris ATCC 43183 16S ribosomal RNA, partial sequence | NR_027196.1 | 1342 | 334 | 755 | 31.45 | 671.5 | 0 | 402/422 | 95 | 0/422 | 0 | Plus | Plus | Bacteria; Bacteroidetes; Bacteroidia; Bacteroidales; Bacteroidaceae; Bacteroides; Bacteroides stercoris | Bacteroides stercoris | Bacteroides | Bacteroidaceae | Bacteroidales | Bacteroidia | Bacteroidetes | Bacteria |
| ASV1910 | 422 | 1 | 1 | 422 | 100 | NR_113411.1 Prevotella copri DSM 18205 strain JCM 13464 16S ribosomal RNA, partial sequence | NR_113411.1 | 1493 | 358 | 779 | 28.27 | 608.7 | 7E-174 | 391/422 | 92 | 0/422 | 0 | Plus | Plus | Bacteria; Bacteroidetes; Bacteroidia; Bacteroidales; Prevotellaceae; Prevotella; Prevotella copri | Prevotella copri | Prevotella | Prevotellaceae | Bacteroidales | Bacteroidia | Bacteroidetes | Bacteria |
| ASV1911 | 426 | 1 | 1 | 421 | 98.83 | NR_027526.1 Catenibacterium mitsuokai strain DSM 15897 16S ribosomal RNA, partial sequence | NR_027526.1 | 1485 | 345 | 763 | 28.22 | 649.3 | 0 | 398/421 | 94 | 2/421 | 0 | Plus | Plus | Bacteria; Firmicutes; Erysipelotrichia; Erysipelotrichales; Coprobacillaceae; Catenibacterium; Catenibacterium mitsuokai | Catenibacterium mitsuokai | Catenibacterium | Coprobacillaceae | Erysipelotrichales | Erysipelotrichia | Firmicutes | Bacteria |
| ASV1912 | 402 | 1 | 1 | 402 | 100 | NR_104783.1 Acetivibrio ethanolgignens strain 77-6 16S ribosomal RNA, partial sequence | NR_104783.1 | 1529 | 370 | 771 | 26.29 | 665.9 | 0 | 388/402 | 96 | 0/402 | 0 | Plus | Plus | Bacteria; Firmicutes; Clostridia; Eubacteriales; Oscillospiraceae; Acetivibrio; Acetivibrio ethanolgignens | Acetivibrio ethanolgignens | Acetivibrio | Oscillospiraceae | Eubacteriales | Clostridia | Firmicutes | Bacteria |
| ASV1913 | 421 | 1 | 1 | 421 | 100 | NR_144616.1 Muribaculum intestinale strain YL27 16S ribosomal RNA, partial sequence | NR_144616.1 | 1460 | 297 | 718 | 28.9 | 496 | 6E-140 | 374/425 | 88 | 7/425 | 1 | Plus | Plus | Bacteria; Bacteroidetes; Bacteroidia; Bacteroidales; Muribaculaceae; Muribaculum; Muribaculum intestinale | Muribaculum intestinale | Muribaculum | Muribaculaceae | Bacteroidales | Bacteroidia | Bacteroidetes | Bacteria |
| ASV1914 | 402 | 1 | 1 | 402 | 100 | NR_134772.1 Murimonas intestini strain SRB-530-5-H 16S ribosomal RNA, partial sequence | NR_134772.1 | 1433 | 314 | 715 | 28.05 | 699.2 | 0 | 394/402 | 98 | 0/402 | 0 | Plus | Plus | Bacteria; Firmicutes; Clostridia; Eubacteriales; Lachnospiraceae; Murimonas; Murimonas intestini | Murimonas intestini | Murimonas | Lachnospiraceae | Eubacteriales | Clostridia | Firmicutes | Bacteria |
| ASV1915 | 402 | 1 | 1 | 402 | 100 | NR_147380.1 Eggerthella timonensis strain Marseille-P3135 16S ribosomal RNA, partial sequence | NR_147380.1 | 1472 | 348 | 749 | 27.31 | 588.4 | 9E-168 | 374/402 | 93 | 0/402 | 0 | Plus | Plus | Bacteria; Actinobacteria; Coriobacteriia; Eggerthellales; Eggerthellaceae; Eggerthella; Eggerthella timonensis | Eggerthella timonensis | Eggerthella | Eggerthellaceae | Eggerthellales | Coriobacteriia | Actinobacteria | Bacteria |
| ASV1918 | 427 | 1 | 1 | 427 | 100 | NR_042649.1 Streptococcus plurextorum strain 1956-02 16S ribosomal RNA, partial sequence | NR_042649.1 | 1451 | 345 | 771 | 29.43 | 789.6 | 0 | 427/427 | 100 | 0/427 | 0 | Plus | Plus | Bacteria; Firmicutes; Bacilli; Lactobacillales; Streptococcaceae; Streptococcus; Streptococcus plurextorum | Streptococcus plurextorum | Streptococcus | Streptococcaceae | Lactobacillales | Bacilli | Firmicutes | Bacteria |
| ASV1919 | 405 | 1 | 1 | 405 | 100 | NR_144742.1 Christensenella massiliensis strain Marseille-P2438 16S ribosomal RNA, partial sequence | NR_144742.1 | 1499 | 369 | 773 | 27.02 | 472 | 1E-132 | 358/408 | 87 | 6/408 | 1 | Plus | Plus | Bacteria; Firmicutes; Clostridia; Eubacteriales; Christensenellaceae; Christensenella; Christensenella massiliensis | Christensenella massiliensis | Christensenella | Christensenellaceae | Eubacteriales | Clostridia | Firmicutes | Bacteria |
| ASV1920 | 405 | 1 | 1 | 405 | 100 | NR_118554.1 Intestinimonas butyriciproducens strain SRB-521-5-I 16S ribosomal RNA, partial sequence | NR_118554.1 | 1475 | 346 | 750 | 27.46 | 505.3 | 1E-142 | 361/405 | 89 | 0/405 | 0 | Plus | Plus | Bacteria; Firmicutes; Clostridia; Eubacteriales; ; Intestinimonas; Intestinimonas butyriciproducens | Intestinimonas butyriciproducens | Intestinimonas | | Eubacteriales | Clostridia | Firmicutes | Bacteria |
| ASV1921 | 401 | 3 | 1 | 401 | 100 | NR_116939.1 Olsenella profusa DSM 13989 strain CCUG 45371 16S ribosomal RNA, partial sequence | NR_116939.1 | 1428 | 328 | 735 | 28.57 | 569.9 | 3E-162 | 376/408 | 92 | 7/408 | 1 | Plus | Plus | Bacteria; Actinobacteria; Coriobacteriia; Coriobacteriales; Atopobiaceae; Olsenella; Olsenella profusa | Olsenella profusa | Olsenella | Atopobiaceae | Coriobacteriales | Coriobacteriia | Actinobacteria | Bacteria |
| ASV1922 | 427 | 1 | 1 | 427 | 100 | NR_041666.1 Dialister succinatiphilus strain YIT 11850 16S ribosomal RNA, partial sequence | NR_041666.1 | 1521 | 353 | 779 | 28.07 | 778.6 | 0 | 425/427 | 99 | 0/427 | 0 | Plus | Plus | Bacteria; Firmicutes; Negativicutes; Veillonellales; Veillonellaceae; Dialister; Dialister succinatiphilus | Dialister succinatiphilus | Dialister | Veillonellaceae | Veillonellales | Negativicutes | Firmicutes | Bacteria |
| ASV1923 | 402 | 1 | 1 | 402 | 100 | NR_028961.1 Faecalibacterium prausnitzii strain ATCC 27768 16S ribosomal RNA, partial sequence | NR_028961.1 | 1462 | 344 | 745 | 27.5 | 721.3 | 0 | 398/402 | 99 | 0/402 | 0 | Plus | Plus | Bacteria; Firmicutes; Clostridia; Eubacteriales; Oscillospiraceae; Faecalibacterium; Faecalibacterium prausnitzii | Faecalibacterium prausnitzii | Faecalibacterium | Oscillospiraceae | Eubacteriales | Clostridia | Firmicutes | Bacteria |
| ASV1924 | 405 | 1 | 1 | 405 | 100 | NR_118554.1 Intestinimonas butyriciproducens strain SRB-521-5-I 16S ribosomal RNA, partial sequence | NR_118554.1 | 1475 | 346 | 750 | 27.46 | 584.4 | 9E-168 | 376/405 | 92 | 0/405 | 0 | Plus | Plus | Bacteria; Firmicutes; Clostridia; Eubacteriales; ; Intestinimonas; Intestinimonas butyriciproducens | Intestinimonas butyriciproducens | Intestinimonas | | Eubacteriales | Clostridia | Firmicutes | Bacteria |
| ASV1925 | 422 | 1 | 1 | 422 | 100 | NR_024815.1 Prevotella shahii strain EHS11 16S ribosomal RNA, partial sequence | NR_024815.1 | 1490 | 356 | 777 | 28.32 | 603.1 | 3E-172 | 392/424 | 92 | 4/424 | 0 | Plus | Plus | Bacteria; Bacteroidetes; Bacteroidia; Bacteroidales; Prevotellaceae; Prevotella; Prevotella shahii | Prevotella shahii | Prevotella | Prevotellaceae | Bacteroidales | Bacteroidia | Bacteroidetes | Bacteria |
| ASV1926 | 422 | 1 | 1 | 422 | 100 | NR_040877.1 Prevotella copri DSM 18205 strain C87 16S ribosomal RNA, partial sequence | NR_040877.1 | 1494 | 358 | 779 | 28.25 | 693.6 | 0 | 406/422 | 96 | 0/422 | 0 | Plus | Plus | Bacteria; Bacteroidetes; Bacteroidia; Bacteroidales; Prevotellaceae; Prevotella; Prevotella copri | Prevotella copri | Prevotella | Prevotellaceae | Bacteroidales | Bacteroidia | Bacteroidetes | Bacteria |
| ASV1927 | 402 | 1 | 1 | 402 | 100 | NR_118675.1 [Eubacterium] siraeum strain ATCC 29066 16S ribosomal RNA, partial sequence | NR_118675.1 | 1453 | 356 | 757 | 27.67 | 601.3 | 1E-171 | 375/402 | 93 | 0/402 | 0 | Plus | Plus | Bacteria; Firmicutes; Clostridia; Eubacteriales; Oscillospiraceae; ; [Eubacterium] siraeum | [Eubacterium] siraeum | | Oscillospiraceae | Eubacteriales | Clostridia | Firmicutes | Bacteria |
| ASV1928 | 402 | 1 | 1 | 402 | 100 | NR_152688.1 Falcatimonas natans strain WN011 16S ribosomal RNA, partial sequence | NR_152688.1 | 1484 | 365 | 766 | 27.09 | 649.3 | 0 | 385/402 | 95 | 0/402 | 0 | Plus | Plus | Bacteria; Firmicutes; Clostridia; Eubacteriales; Clostridiaceae; Falcatimonas; Falcatimonas natans | Falcatimonas natans | Falcatimonas | Clostridiaceae | Eubacteriales | Clostridia | Firmicutes | Bacteria |
| ASV1929 | 402 | 1 | 1 | 402 | 100 | NR_026020.1 Clostridium septicum strain Pasteur III 16S ribosomal RNA, partial sequence | NR_026020.1 | 1507 | 350 | 751 | 26.68 | 732.4 | 0 | 400/402 | 99 | 0/402 | 0 | Plus | Plus | Bacteria; Firmicutes; Clostridia; Eubacteriales; Clostridiaceae; Clostridium; Clostridium septicum | Clostridium septicum | Clostridium | Clostridiaceae | Eubacteriales | Clostridia | Firmicutes | Bacteria |
| ASV1930 | 422 | 2 | 1 | 422 | 100 | NR_113117.1 Prevotella oralis ATCC 33269 strain JCM 12251 16S ribosomal RNA, partial sequence | NR_113117.1 | 1496 | 361 | 782 | 28.21 | 586.5 | 3E-167 | 387/422 | 91 | 0/422 | 0 | Plus | Plus | Bacteria; Bacteroidetes; Bacteroidia; Bacteroidales; Prevotellaceae; Prevotella; Prevotella oralis | Prevotella oralis | Prevotella | Prevotellaceae | Bacteroidales | Bacteroidia | Bacteroidetes | Bacteria |
| ASV1931 | 422 | 1 | 1 | 422 | 100 | NR_024816.1 Prevotella salivae strain EPSA11 16S ribosomal RNA, partial sequence | NR_024816.1 | 1493 | 356 | 777 | 28.27 | 603.1 | 3E-172 | 391/423 | 92 | 2/423 | 0 | Plus | Plus | Bacteria; Bacteroidetes; Bacteroidia; Bacteroidales; Prevotellaceae; Prevotella; Prevotella salivae | Prevotella salivae | Prevotella | Prevotellaceae | Bacteroidales | Bacteroidia | Bacteroidetes | Bacteria |
| ASV1932 | 422 | 1 | 1 | 422 | 100 | NR_024815.1 Prevotella shahii strain EHS11 16S ribosomal RNA, partial sequence | NR_024815.1 | 1490 | 356 | 777 | 28.32 | 553.3 | 4E-157 | 383/424 | 90 | 4/424 | 0 | Plus | Plus | Bacteria; Bacteroidetes; Bacteroidia; Bacteroidales; Prevotellaceae; Prevotella; Prevotella shahii | Prevotella shahii | Prevotella | Prevotellaceae | Bacteroidales | Bacteroidia | Bacteroidetes | Bacteria |
| ASV1933 | 402 | 1 | 1 | 402 | 100 | NR_148619.1 Agathobacter ruminis strain JK623 16S ribosomal RNA, partial sequence | NR_148619.1 | 1434 | 318 | [illegible] | 28.17 | 723.2 | 0 | 400/404 | 99 | 2/404 | 0 | Plus | Plus | Bacteria; Firmicutes; Clostridia; Eubacteriales; Lachnospiraceae; Agathobacter; Agathobacter ruminis | Agathobacter ruminis | Agathobacter | Lachnospiraceae | Eubacteriales | Clostridia | Firmicutes | Bacteria |
| ASV1934 | 426 | 2 | 1 | 426 | 100 | NR_025333.1 Leminorella grimontii strain 81H-380 16S ribosomal RNA, partial sequence | NR_025333.1 | 1482 | 321 | 747 | 28.81 | 466.5 | 5E-131 | 375/433 | 86 | 13/433 | 3 | Plus | Plus | Bacteria; Proteobacteria; Gammaproteobacteria; Enterobacterales; Budviciaceae; Leminorella; Leminorella grimontii | Leminorella grimontii | Leminorella | Budviciaceae | Enterobacterales | Gammaproteobacteria | Proteobacteria | Bacteria |
| ASV1935 | 403 | 2 | 3 | 403 | 99.5 | NR_125677.1 Vallitalea pronyensis strain FatNI3 16S ribosomal RNA, partial sequence | NR_125677.1 | 1480 | 365 | 764 | 27.03 | 451.7 | 1E-126 | 352/404 | 87 | 7/404 | 1 | Plus | Plus | Bacteria; Firmicutes; Clostridia; Eubacteriales; Vallitaleaceae; Vallitalea; Vallitalea pronyensis | Vallitalea pronyensis | Vallitalea | Vallitaleaceae | Eubacteriales | Clostridia | Firmicutes | Bacteria |
| ASV1936 | 403 | 4 | 1 | 403 | 100 | NR_074399.1 Ruminococcus albus 7 = DSM 20455 16S ribosomal RNA, partial sequence | NR_074399.1 | 1500 | 350 | 751 | 26.8 | 660.4 | 0 | 388/403 | 96 | 1/403 | 0 | Plus | Plus | Bacteria; Firmicutes; Clostridia; Eubacteriales; Oscillospiraceae; Ruminococcus; Ruminococcus albus | Ruminococcus albus | Ruminococcus | Oscillospiraceae | Eubacteriales | Clostridia | Firmicutes | Bacteria |
| ASV1937 | 422 | 1 | 1 | 422 | 100 | NR_041342.1 Parabacteroides distasonis strain JCM 5825 16S ribosomal RNA, partial sequence | NR_041342.1 | 1488 | 353 | 774 | 28.36 | 564.3 | 2E-160 | 384/423 | 90 | 2/423 | 0 | Plus | Plus | Bacteria; Bacteroidetes; Bacteroidia; Bacteroidales; Tannerellaceae; Parabacteroides; Parabacteroides distasonis | Parabacteroides distasonis | Parabacteroides | Tannerellaceae | Bacteroidales | Bacteroidia | Bacteroidetes | Bacteria |
| ASV1938 | 427 | 1 | 1 | 427 | 100 | NR_118847.1 Actinobacillus minor NM305 16S ribosomal RNA, partial sequence | NR_118847.1 | 1464 | 333 | 759 | 29.17 | 774.9 | 0 | 424/427 | 99 | 0/427 | 0 | Plus | Plus | Bacteria; Proteobacteria; Gammaproteobacteria; Pasteurellales; Pasteurellaceae; Actinobacillus; Actinobacillus minor | Actinobacillus minor | Actinobacillus | Pasteurellaceae | Pasteurellales | Gammaproteobacteria | Proteobacteria | Bacteria |
| ASV1939 | 428 | 1 | 1 | 427 | 99.77 | NR_026038.1 Desulfitobacterium chlororespirans strain Co23 16S ribosomal RNA, partial sequence | NR_026038.1 | 1507 | 348 | 774 | 28.33 | 501.6 | 1E-141 | 376/428 | 87 | 2/428 | 0 | Plus | Plus | Bacteria; Firmicutes; Clostridia; Eubacteriales; Peptococcaceae; Desulfitobacterium; Desulfitobacterium chlororespirans | Desulfitobacterium chlororespirans | Desulfitobacterium | Peptococcaceae | Eubacteriales | Clostridia | Firmicutes | Bacteria |
| ASV1940 | 427 | 1 | 1 | 427 | 100 | NR_025762.1 Veillonella caviae strain PV1 16S ribosomal RNA, partial sequence | NR_025762.1 | 1402 | 321 | 747 | 30.46 | 778.6 | 0 | 425/427 | 99 | 0/427 | 0 | Plus | Plus | Bacteria; Firmicutes; Negativicutes; Veillonellales; Veillonellaceae; Veillonella; Veillonella caviae | Veillonella caviae | Veillonella | Veillonellaceae | Veillonellales | Negativicutes | Firmicutes | Bacteria |
| ASV1941 | 428 | 1 | 1 | 428 | 100 | NR_104781.1 Treponema bryantii strain RUS-1 16S ribosomal RNA, partial sequence | NR_104781.1 | 1535 | 351 | 778 | 27.88 | 636.4 | 0 | 401/429 | 93 | 2/429 | 0 | Plus | Plus | Bacteria; Spirochaetes; Spirochaetia; Spirochaetales; Spirochaetaceae; Treponema; Treponema bryantii | Treponema bryantii | Treponema | Spirochaetaceae | Spirochaetales | Spirochaetia | Spirochaetes | Bacteria |
| ASV1942 | 403 | 5 | 1 | 403 | 100 | NR_115693.1 Gracilibacter thermotolerans JW/YJL-S1 16S ribosomal RNA, partial sequence | NR_115693.1 | 1526 | 459 | 861 | 26.41 | 512.6 | 6E-145 | 361/403 | 89 | 0/403 | 0 | Plus | Plus | Bacteria; Firmicutes; Clostridia; Eubacteriales; Gracilibacteraceae; Gracilibacter; Gracilibacter thermotolerans | Gracilibacter thermotolerans | Gracilibacter | Gracilibacteraceae | Eubacteriales | Clostridia | Firmicutes | Bacteria |
| ASV1943 | 401 | 5 | 1 | 401 | 100 | NR_112038.1 Eggerthella lenta strain JCM 9979 16S ribosomal RNA, partial sequence | NR_112038.1 | 1472 | 351 | 752 | 27.31 | 558.8 | 7E-159 | 370/403 | 91 | 3/403 | 0 | Plus | Plus | Bacteria; Actinobacteria; Coriobacteriia; Eggerthellales; Eggerthellaceae; Eggerthella; Eggerthella lenta | Eggerthella lenta | Eggerthella | Eggerthellaceae | Eggerthellales | Coriobacteriia | Actinobacteria | Bacteria |
| ASV1944 | 422 | 1 | 1 | 422 | 100 | NR_145844.1 Bacteroides caecigallinarum strain C13EG111 16S ribosomal RNA, partial sequence | NR_145844.1 | 1489 | 356 | 777 | 28.34 | 614.2 | 2E-175 | 393/423 | 92 | 2/423 | 0 | Plus | Plus | Bacteria; Bacteroidetes; Bacteroidia; Bacteroidales; Bacteroidaceae; Bacteroides; Bacteroides caecigallinarum | Bacteroides caecigallinarum | Bacteroides | Bacteroidaceae | Bacteroidales | Bacteroidia | Bacteroidetes | Bacteria |
| ASV1945 | 402 | 2 | 1 | 402 | 100 | NR_042144.1 Clostridium butyricum strain VPI3266 16S ribosomal RNA, partial sequence | NR_042144.1 | 1425 | 338 | 739 | 28.21 | 732.4 | 0 | 400/402 | 99 | 0/402 | 0 | Plus | Plus | Bacteria; Firmicutes; Clostridia; Eubacteriales; Clostridiaceae; Clostridium; Clostridium butyricum | Clostridium butyricum | Clostridium | Clostridiaceae | Eubacteriales | Clostridia | Firmicutes | Bacteria |
| ASV1946 | 421 | 1 | 1 | 421 | 100 | NR_113097.1 Prevotella buccae ATCC 33574 strain JCM 12245 16S ribosomal RNA, partial sequence | NR_113097.1 | 1480 | 343 | 764 | 28.51 | 451.7 | 1E-126 | 366/425 | 86 | 7/425 | 1 | Plus | Plus | Bacteria; Bacteroidetes; Bacteroidia; Bacteroidales; Prevotellaceae; Prevotella; Prevotella buccae | Prevotella buccae | Prevotella | Prevotellaceae | Bacteroidales | Bacteroidia | Bacteroidetes | Bacteria |
| ASV1948 | 404 | 1 | 1 | 404 | 100 | NR_042832.1 Roseburia faecis strain M72/1 16S ribosomal RNA, partial sequence | NR_042832.1 | 1456 | 347 | 749 | 27.68 | 734.2 | 0 | 402/404 | 99 | 1/404 | 0 | Plus | Plus | Bacteria; Firmicutes; Clostridia; Eubacteriales; Lachnospiraceae; Roseburia; Roseburia faecis | Roseburia faecis | Roseburia | Lachnospiraceae | Eubacteriales | Clostridia | Firmicutes | Bacteria |
| ASV1949 | 426 | 1 | 1 | 426 | 100 | NR_041304.1 Sharpea azabuensis strain ST18 16S ribosomal RNA, partial sequence | NR_041304.1 | 1460 | 325 | 750 | 29.18 | 754.6 | 0 | 420/426 | 98 | 0/426 | 0 | Plus | Plus | Bacteria; Firmicutes; Erysipelotrichia; Erysipelotrichales; Coprobacillaceae; Sharpea; Sharpea azabuensis | Sharpea azabuensis | Sharpea | Coprobacillaceae | Erysipelotrichales | Erysipelotrichia | Firmicutes | Bacteria |
| ASV1950 | 421 | 2 | 1 | 421 | 100 | NR_113073.1 Barnesiella intestinihominis strain JCM 15079 16S ribosomal RNA, partial sequence | NR_113073.1 | 1481 | 346 | 767 | 28.49 | 496 | 6E-140 | 372/423 | 87 | 3/423 | 1 | Plus | Plus | Bacteria; Bacteroidetes; Bacteroidia; Bacteroidales; Barnesiellaceae; Barnesiella; Barnesiella intestinihominis | Barnesiella intestinihominis | Barnesiella | Barnesiellaceae | Bacteroidales | Bacteroidia | Bacteroidetes | Bacteria |
| ASV1952 | 403 | 2 | 3 | 403 | 99.5 | NR_125677.1 Vallitalea pronyensis strain FatNI3 16S ribosomal RNA, partial sequence | NR_125677.1 | 1480 | 365 | 764 | 27.03 | 457.2 | 3E-128 | 351/402 | 87 | 3/402 | 0 | Plus | Plus | Bacteria; Firmicutes; Clostridia; Eubacteriales; Vallitaleaceae; Vallitalea; Vallitalea pronyensis | Vallitalea pronyensis | Vallitalea | Vallitaleaceae | Eubacteriales | Clostridia | Firmicutes | Bacteria |
| ASV1954 | 405 | 2 | 1 | 405 | 100 | NR_074629.1 Hungateiclostridium thermocellum strain ATCC 27405 16S ribosomal RNA, partial sequence | NR_074629.1 | 1521 | 358 | 762 | 26.63 | 571.7 | 9E-163 | 375/407 | 92 | 4/407 | 0 | Plus | Plus | Bacteria; Firmicutes; Clostridia; Eubacteriales; Oscillospiraceae; Acetivibrio; Acetivibrio thermocellus | Acetivibrio thermocellus | Acetivibrio | Oscillospiraceae | Eubacteriales | Clostridia | Firmicutes | Bacteria |
| ASV1955 | 427 | 1 | 1 | 427 | 100 | NR_112902.1 Phascolarctobacterium succinatutens strain YIT 12067 16S ribosomal RNA, partial sequence | NR_112902.1 | 1610 | 444 | 870 | 26.52 | 761.9 | 0 | 422/427 | 98 | 0/427 | 0 | Plus | Plus | Bacteria; Firmicutes; Negativicutes; Acidaminococcales; Acidaminococcaceae; Phascolarctobacterium; Phascolarctobacterium succinatutens | Phascolarctobacterium succinatutens | Phascolarctobacterium | Acidaminococcaceae | Acidaminococcales | Negativicutes | Firmicutes | Bacteria |
| ASV1956 | 405 | 1 | 1 | 405 | 100 | NR_044972.1 Sporobacter termitidis strain SYR 16S ribosomal RNA, partial sequence | NR_044972.1 | 1506 | 348 | 752 | 26.89 | 531.1 | 2E-150 | 366/406 | 90 | 2/406 | 0 | Plus | Plus | Bacteria; Firmicutes; Clostridia; Eubacteriales; Oscillospiraceae; Sporobacter; Sporobacter termitidis | Sporobacter termitidis | Sporobacter | Oscillospiraceae | Eubacteriales | Clostridia | Firmicutes | Bacteria |
| ASV1957 | 421 | 1 | 1 | 421 | 100 | NR_117779.1 Anaerorhabdus furcosa 16S ribosomal RNA, partial sequence | NR_117779.1 | 1488 | 350 | 770 | 28.29 | 562.5 | 6E-160 | 383/422 | 90 | 2/422 | 0 | Plus | Plus | Bacteria; Firmicutes; Erysipelotrichia; Erysipelotrichales; Erysipelotrichaceae; Anaerorhabdus; Anaerorhabdus furcosa | Anaerorhabdus furcosa | Anaerorhabdus | Erysipelotrichaceae | Erysipelotrichales | Erysipelotrichia | Firmicutes | Bacteria |
| ASV1958 | 427 | 2 | 1 | 427 | 100 | NR_075036.1 Lactobacillus reuteri DSM 20016 16S ribosomal RNA, partial sequence | NR_075036.1 | 1569 | 384 | 810 | 27.21 | 778.6 | 0 | 425/427 | 99 | 0/427 | 0 | Plus | Plus | Bacteria; Firmicutes; Bacilli; Lactobacillales; Lactobacillaceae; Limosilactobacillus; Limosilactobacillus reuteri | Limosilactobacillus reuteri | Limosilactobacillus | Lactobacillaceae | Lactobacillales | Bacilli | Firmicutes | Bacteria |
| ASV1959 | 402 | 2 | 1 | 402 | 100 | NR_156080.1 Kineothrix alysoides strain KNHs209 16S ribosomal RNA, partial sequence | NR_156080.1 | 1410 | 353 | 754 | 28.51 | 671.5 | 0 | 389/402 | 96 | 0/402 | 0 | Plus | Plus | Bacteria; Firmicutes; Clostridia; Eubacteriales; Lachnospiraceae; Kineothrix; Kineothrix alysoides | Kineothrix alysoides | Kineothrix | Lachnospiraceae | Eubacteriales | Clostridia | Firmicutes | Bacteria |
| ASV1960 | 422 | 1 | 1 | 422 | 100 | NR_113411.1 Prevotella copri DSM 18205 strain JCM 13464 16S ribosomal RNA, partial sequence | NR_113411.1 | 1493 | 358 | 779 | 28.27 | 758.2 | 0 | 418/422 | 99 | 0/422 | 0 | Plus | Plus | Bacteria; Bacteroidetes; Bacteroidia; Bacteroidales; Prevotellaceae; Prevotella; Prevotella copri | Prevotella copri | Prevotella | Prevotellaceae | Bacteroidales | Bacteroidia | Bacteroidetes | Bacteria |
| ASV1961 | 405 | 1 | 1 | 405 | 100 | NR_148302.1 Geosporobacter ferrireducens strain IRF9 16S ribosomal RNA, partial sequence | NR_148302.1 | 1392 | 306 | 707 | 28.88 | 468.3 | 1E-131 | 357/407 | 87 | 7/407 | 1 | Plus | Plus | Bacteria; Firmicutes; Clostridia; Eubacteriales; Clostridiaceae; Geosporobacter; Geosporobacter ferrireducens | Geosporobacter ferrireducens | Geosporobacter | Clostridiaceae | Eubacteriales | Clostridia | Firmicutes | Bacteria |
| ASV1962 | 405 | 1 | 1 | 405 | 100 | NR_144611.1 Flintibacter butyricus strain BLS21 16S ribosomal RNA, partial sequence | NR_144611.1 | 808 | 299 | 703 | 50.12 | 693.6 | 0 | 397/407 | 97 | 4/407 | 0 | Plus | Plus | Bacteria; Firmicutes; Clostridia; Eubacteriales; ; Flintibacter; Flintibacter butyricus | Flintibacter butyricus | Flintibacter | | Eubacteriales | Clostridia | Firmicutes | Bacteria |
| ASV1963 | 422 | 1 | 1 | 422 | 100 | NR_024816.1 Prevotella salivae strain EPSA11 16S ribosomal RNA, partial sequence | NR_024816.1 | 1493 | 356 | 777 | 28.27 | 581 | 2E-165 | 386/422 | 91 | 0/422 | 0 | Plus | Plus | Bacteria; Bacteroidetes; Bacteroidia; Bacteroidales; Prevotellaceae; Prevotella; Prevotella salivae | Prevotella salivae | Prevotella | Prevotellaceae | Bacteroidales | Bacteroidia | Bacteroidetes | Bacteria |
| ASV1964 | 426 | 1 | 1 | 426 | 100 | NR_114696.1 Anaerovibrio lipolyticus DSM 3074 16S ribosomal RNA, partial sequence | NR_114696.1 | 1526 | 372 | 797 | 27.92 | 699.2 | 0 | 413/429 | 96 | 6/429 | 1 | Plus | Plus | Bacteria; Firmicutes; Negativicutes; Selenomonadales; Selenomonadaceae; Anaerovibrio; Anaerovibrio lipolyticus | Anaerovibrio lipolyticus | Anaerovibrio | Selenomonadaceae | Selenomonadales | Negativicutes | Firmicutes | Bacteria |
| ASV1965 | 422 | 1 | 1 | 422 | 100 | NR_144616.1 Muribaculum intestinale strain YL27 16S ribosomal RNA, partial sequence | NR_144616.1 | 1460 | 297 | 718 | 28.9 | 597.6 | 2E-170 | 389/422 | 92 | 0/422 | 0 | Plus | Plus | Bacteria; Bacteroidetes; Bacteroidia; Bacteroidales; Muribaculaceae; Muribaculum; Muribaculum intestinale | Muribaculum intestinale | Muribaculum | Muribaculaceae | Bacteroidales | Bacteroidia | Bacteroidetes | Bacteria |
| ASV1966 | 404 | 2 | 1 | 404 | 100 | NR_114326.1 Fusicatenibacter saccharivorans strain HT03-11 16S ribosomal RNA, partial sequence | NR_114326.1 | 1423 | 351 | 752 | 28.25 | 662.2 | 0 | 389/404 | 96 | 2/404 | 0 | Plus | Plus | Bacteria; Firmicutes; Clostridia; Eubacteriales; Lachnospiraceae; Fusicatenibacter; Fusicatenibacter saccharivorans | Fusicatenibacter saccharivorans | Fusicatenibacter | Lachnospiraceae | Eubacteriales | Clostridia | Firmicutes | Bacteria |
| ASV1967 | 427 | 1 | 1 | 427 | 100 | NR_027596.1 Mitsuokella multacida strain DSM 20544 16S ribosomal RNA, partial sequence | NR_027596.1 | 1511 | 346 | 772 | 28.26 | 752.7 | 0 | 420/427 | 98 | 0/427 | 0 | Plus | Plus | Bacteria; Firmicutes; Negativicutes; Selenomonadales; Selenomonadaceae; Mitsuokella; Mitsuokella multacida | Mitsuokella multacida | Mitsuokella | Selenomonadaceae | Selenomonadales | Negativicutes | Firmicutes | Bacteria |
| ASV1968 | 404 | 1 | 1 | 404 | 100 | NR_042832.1 Roseburia faecis strain M72/1 16S ribosomal RNA, partial sequence | NR_042832.1 | 1456 | 347 | 749 | 27.68 | 734.2 | 0 | 402/404 | 99 | 1/404 | 0 | Plus | Plus | Bacteria; Firmicutes; Clostridia; Eubacteriales; Lachnospiraceae; Roseburia; Roseburia faecis | Roseburia faecis | Roseburia | Lachnospiraceae | Eubacteriales | Clostridia | Firmicutes | Bacteria |
| ASV1969 | 402 | 1 | 1 | 402 | 100 | NR_044049.1 Coprococcus eutactus strain ATCC 27759 16S ribosomal RNA, partial sequence | NR_044049.1 | 1440 | 333 | 734 | 27.92 | 737.9 | 0 | 401/402 | 99 | 0/402 | 0 | Plus | Plus | Bacteria; Firmicutes; Clostridia; Eubacteriales; Lachnospiraceae; Coprococcus; Coprococcus eutactus | Coprococcus eutactus | Coprococcus | Lachnospiraceae | Eubacteriales | Clostridia | Firmicutes | Bacteria |
| ASV1970 | 421 | 2 | 1 | 421 | 100 | NR_113077.1 Paraprevotella clara strain JCM 14859 16S ribosomal RNA, partial sequence | NR_113077.1 | 1480 | 346 | 766 | 28.45 | 479.4 | 6E-135 | 370/424 | 87 | 6/424 | 1 | Plus | Plus | Bacteria; Bacteroidetes; Bacteroidia; Bacteroidales; Prevotellaceae; Paraprevotella; Paraprevotella clara | Paraprevotella clara | Paraprevotella | Prevotellaceae | Bacteroidales | Bacteroidia | Bacteroidetes | Bacteria |
| ASV1972 | 402 | 1 | 1 | 402 | 100 | NR_028785.1 [Clostridium] scindens strain ATCC 35704 16S ribosomal RNA, partial sequence | NR_028785.1 | 1529 | 368 | 769 | 26.29 | 743.5 | 0 | 402/402 | 100 | 0/402 | 0 | Plus | Plus | Bacteria; Firmicutes; Clostridia; Eubacteriales; Lachnospiraceae; Lachnoclostridium; [Clostridium] scindens | [Clostridium] scindens | Lachnoclostridium | Lachnospiraceae | Eubacteriales | Clostridia | Firmicutes | Bacteria |
| ASV1973 | 422 | 1 | 1 | 422 | 100 | NR_113411.1 Prevotella copri DSM 18205 strain JCM 13464 16S ribosomal RNA, partial sequence | NR_113411.1 | 1493 | 358 | 779 | 28.27 | 592 | 7E-169 | 390/424 | 91 | 4/424 | 0 | Plus | Plus | Bacteria; Bacteroidetes; Bacteroidia; Bacteroidales; Prevotellaceae; Prevotella; Prevotella copri | Prevotella copri | Prevotella | Prevotellaceae | Bacteroidales | Bacteroidia | Bacteroidetes | Bacteria |
| ASV1974 | 421 | 1 | 1 | 421 | 100 | NR_113097.1 Prevotella buccae ATCC 33574 strain JCM 12245 16S ribosomal RNA, partial sequence | NR_113097.1 | 1480 | 343 | 764 | 28.51 | 457.2 | 3E-128 | 367/425 | 86 | 7/425 | 1 | Plus | Plus | Bacteria; Bacteroidetes; Bacteroidia; Bacteroidales; Prevotellaceae; Prevotella; Prevotella buccae | Prevotella buccae | Prevotella | Prevotellaceae | Bacteroidales | Bacteroidia | Bacteroidetes | Bacteria |
| ASV1975 | 405 | 1 | 1 | 405 | 100 | NR_144742.1 Christensenella massiliensis strain Marseille-P2438 16S ribosomal RNA, partial sequence | NR_144742.1 | 1499 | 369 | 773 | 27.02 | 444.3 | 2E-124 | 352/407 | 86 | 4/407 | 0 | Plus | Plus | Bacteria; Firmicutes; Clostridia; Eubacteriales; Christensenellaceae; Christensenella; Christensenella massiliensis | Christensenella massiliensis | Christensenella | Christensenellaceae | Eubacteriales | Clostridia | Firmicutes | Bacteria |
| ASV1976 | 422 | 2 | 1 | 422 | 100 | NR_113119.1 Prevotella oris strain JCM 8540 16S ribosomal RNA, partial sequence | NR_113119.1 | 1481 | 345 | 766 | 28.49 | 619.7 | 3E-177 | 395/424 | 93 | 4/424 | 0 | Plus | Plus | Bacteria; Bacteroidetes; Bacteroidia; Bacteroidales; Prevotellaceae; Prevotella; Prevotella oris | Prevotella oris | Prevotella | Prevotellaceae | Bacteroidales | Bacteroidia | Bacteroidetes | Bacteria |
| ASV1977 | 402 | 1 | 1 | 402 | 100 | NR_026312.1 Blautia schinkii strain B 16S ribosomal RNA, partial sequence | NR_026312.1 | 1482 | 344 | 745 | 27.13 | 695.5 | 0 | 393/402 | 97 | 0/402 | 0 | Plus | Plus | Bacteria; Firmicutes; Clostridia; Eubacteriales; Lachnospiraceae; Blautia; Blautia schinkii | Blautia schinkii | Blautia | Lachnospiraceae | Eubacteriales | Clostridia | Firmicutes | Bacteria |
| ASV1978 | 405 | 1 | 4 | 405 | 99.26 | NR_042834.1 Alkalibacter saccharofermentans strain Z-79820 16S ribosomal RNA, partial sequence | NR_042834.1 | 1465 | 341 | 741 | 27.37 | 464.6 | 2E-130 | 354/404 | 87 | 5/404 | 1 | Plus | Plus | Bacteria; Firmicutes; Clostridia; Eubacteriales; Eubacteriaceae; Alkalibacter; Alkalibacter saccharofermentans | Alkalibacter saccharofermentans | Alkalibacter | Eubacteriaceae | Eubacteriales | Clostridia | Firmicutes | Bacteria |
| ASV1979 | 404 | 1 | 1 | 404 | 100 | NR_044490.1 Butyricicoccus pullicaecorum strain 25-3 16S ribosomal RNA, partial sequence | NR_044490.1 | 1482 | 366 | 770 | 27.33 | 603.1 | 3E-172 | 380/406 | 93 | 3/406 | 0 | Plus | Plus | Bacteria; Firmicutes; Clostridia; Eubacteriales; Clostridiaceae; Butyricicoccus; Butyricicoccus pullicaecorum | Butyricicoccus pullicaecorum | Butyricicoccus | Clostridiaceae | Eubacteriales | Clostridia | Firmicutes | Bacteria |
| ASV1981 | 405 | 1 | 1 | 405 | 100 | NR_044972.1 Sporobacter termitidis strain SYR 16S ribosomal RNA, partial sequence | NR_044972.1 | 1506 | 348 | 752 | 26.89 | 608.7 | 7E-174 | 380/406 | 93 | 2/406 | 0 | Plus | Plus | Bacteria; Firmicutes; Clostridia; Eubacteriales; Oscillospiraceae; Sporobacter; Sporobacter termitidis | Sporobacter termitidis | Sporobacter | Oscillospiraceae | Eubacteriales | Clostridia | Firmicutes | Bacteria |
| ASV1982 | 422 | 1 | 1 | 422 | 100 | NR_040877.1 Prevotella copri DSM 18205 strain C87 16S ribosomal RNA, partial sequence | NR_040877.1 | 1494 | 358 | 779 | 28.25 | 732.4 | 0 | 413/422 | 97 | 0/422 | 0 | Plus | Plus | Bacteria; Bacteroidetes; Bacteroidia; Bacteroidales; Prevotellaceae; Prevotella; Prevotella copri | Prevotella copri | Prevotella | Prevotellaceae | Bacteroidales | Bacteroidia | Bacteroidetes | Bacteria |
| ASV1983 | 402 | 1 | 1 | 402 | 100 | NR_118674.1 Eubacterium ramulus ATCC 29099 16S ribosomal RNA, partial sequence | NR_118674.1 | 1474 | 373 | 774 | 27.27 | 688.1 | 0 | 389/402 | 96 | 0/402 | 0 | Plus | Plus | Bacteria; Firmicutes; Clostridia; Eubacteriales; Eubacteriaceae; Eubacterium; Eubacterium ramulus | Eubacterium ramulus | Eubacterium | Eubacteriaceae | Eubacteriales | Clostridia | Firmicutes | Bacteria |
| ASV1984 | 403 | 1 | 3 | 403 | 99.5 | NR_125464.1 Anaerobacterium chartisolvens strain T-1-35 16S ribosomal RNA, partial sequence | NR_125464.1 | 1451 | 344 | 745 | 27.71 | 481.2 | 2E-135 | 357/404 | 88 | 5/404 | 1 | Plus | Plus | Bacteria; Firmicutes; Clostridia; Eubacteriales; Oscillospiraceae; Anaerobacterium; Anaerobacterium chartisolvens | Anaerobacterium chartisolvens | Anaerobacterium | Oscillospiraceae | Eubacteriales | Clostridia | Firmicutes | Bacteria |
| ASV1985 | 422 | 2 | 1 | 422 | 100 | NR_113119.1 Prevotella oris strain JCM 8540 16S ribosomal RNA, partial sequence | NR_113119.1 | 1481 | 345 | 766 | 28.49 | 575.4 | 7E-164 | 386/423 | 91 | 2/423 | 0 | Plus | Plus | Bacteria; Bacteroidetes; Bacteroidia; Bacteroidales; Prevotellaceae; Prevotella; Prevotella oris | Prevotella oris | Prevotella | Prevotellaceae | Bacteroidales | Bacteroidia | Bacteroidetes | Bacteria |
| ASV1986 | 422 | 1 | 1 | 422 | 100 | NR_074577.1 Paludibacter propionicigenes WB4 16S ribosomal RNA, partial sequence | NR_074577.1 | 1516 | 353 | 774 | 27.84 | 520 | 4E-147 | 378/425 | 88 | 6/425 | 1 | Plus | Plus | Bacteria; Bacteroidetes; Bacteroidia; Bacteroidales; Paludibacteraceae; Paludibacter; Paludibacter propionicigenes | Paludibacter propionicigenes | Paludibacter | Paludibacteraceae | Bacteroidales | Bacteroidia | Bacteroidetes | Bacteria |
| ASV1987 | 422 | 2 | 1 | 422 | 100 | NR_113119.1 Prevotella oris strain JCM 8540 16S ribosomal RNA, partial sequence | NR_113119.1 | 1481 | 345 | 766 | 28.49 | 536.6 | 4E-152 | 379/423 | 89 | 2/423 | 0 | Plus | Plus | Bacteria; Bacteroidetes; Bacteroidia; Bacteroidales; Prevotellaceae; Prevotella; Prevotella oris | Prevotella oris | Prevotella | Prevotellaceae | Bacteroidales | Bacteroidia | Bacteroidetes | Bacteria |
| ASV1988 | 427 | 1 | 1 | 427 | 100 | NR_026450.1 Ruminobacter amylophilus strain H 18 16S ribosomal RNA, complete sequence | NR_026450.1 | 1539 | 351 | 777 | 27.75 | 728.7 | 0 | 417/428 | 97 | 2/428 | 0 | Plus | Plus | Bacteria; Proteobacteria; Gammaproteobacteria; Aeromonadales; Succinivibrionaceae; Ruminobacter; Ruminobacter amylophilus | Ruminobacter amylophilus | Ruminobacter | Succinivibrionaceae | Aeromonadales | Gammaproteobacteria | Proteobacteria | Bacteria |
| ASV1989 | 405 | 5 | 1 | 405 | 100 | NR_115693.1 Gracilibacter thermotolerans JW/YJL-S1 16S ribosomal RNA, partial sequence | NR_115693.1 | 1526 | 459 | 861 | 26.41 | 464.6 | 2E-130 | 356/407 | 87 | 6/407 | 1 | Plus | Plus | Bacteria; Firmicutes; Clostridia; Eubacteriales; Gracilibacteraceae; Gracilibacter; Gracilibacter thermotolerans | Gracilibacter thermotolerans | Gracilibacter | Gracilibacteraceae | Eubacteriales | Clostridia | Firmicutes | Bacteria |
| ASV1990 | 422 | 1 | 1 | 422 | 100 | NR_144616.1 Muribaculum intestinale strain YL27 16S ribosomal RNA, partial sequence | NR_144616.1 | 1460 | 297 | 718 | 28.9 | 569.9 | 3E-162 | 388/426 | 91 | 8/426 | 1 | Plus | Plus | Bacteria; Bacteroidetes; Bacteroidia; Bacteroidales; Muribaculaceae; Muribaculum; Muribaculum intestinale | Muribaculum intestinale | Muribaculum | Muribaculaceae | Bacteroidales | Bacteroidia | Bacteroidetes | Bacteria |
| ASV1991 | 404 | 1 | 1 | 404 | 100 | NR_118156.1 Oscillibacter ruminantium GH1 16S ribosomal RNA, partial sequence | NR_118156.1 | 1409 | 321 | 724 | 28.67 | 636.4 | 0 | 384/404 | 95 | 0/404 | 0 | Plus | Plus | Bacteria; Firmicutes; Clostridia; Eubacteriales; Oscillospiraceae; Oscillibacter; Oscillibacter ruminantium | Oscillibacter ruminantium | Oscillibacter | Oscillospiraceae | Eubacteriales | Clostridia | Firmicutes | Bacteria |
| ASV1992 | 402 | 1 | 1 | 402 | 100 | NR_112553.1 [Clostridium] glycyrrhizinilyticum strain ZM35 16S ribosomal RNA, partial sequence | NR_112553.1 | 1493 | 365 | 767 | 26.99 | 693.6 | 0 | 394/403 | 97 | 1/403 | 0 | Plus | Plus | Bacteria; Firmicutes; Clostridia; Eubacteriales; Lachnospiraceae; Mediterraneibacter; Mediterraneibacter glycyrrhizinilyticus | Mediterraneibacter glycyrrhizinilyticus | Mediterraneibacter | Lachnospiraceae | Eubacteriales | Clostridia | Firmicutes | Bacteria |
| ASV1993 | 428 | 1 | 1 | 428 | 100 | NR_165711.1 Intestinibaculum porci strain SG0102 16S ribosomal RNA, partial sequence | NR_165711.1 | 1489 | 329 | 756 | 28.74 | 785.9 | 0 | 427/428 | 99 | 0/428 | 0 | Plus | Plus | Bacteria; Firmicutes; Erysipelotrichia; Erysipelotrichales; Erysipelotrichaceae; Intestinibaculum; Intestinibaculum porci | Intestinibaculum porci | Intestinibaculum | Erysipelotrichaceae | Erysipelotrichales | Erysipelotrichia | Firmicutes | Bacteria |
| ASV1994 | 426 | 1 | 1 | 426 | 100 | NR_028840.1 Mitsuokella jalaludinii strain M9 16S ribosomal RNA, partial sequence | NR_028840.1 | 1543 | 362 | 788 | 27.67 | 665.9 | 0 | 406/428 | 94 | 3/428 | 0 | Plus | Plus | Bacteria; Firmicutes; Negativicutes; Selenomonadales; Selenomonadaceae; Mitsuokella; Mitsuokella jalaludinii | Mitsuokella jalaludinii | Mitsuokella | Selenomonadaceae | Selenomonadales | Negativicutes | Firmicutes | Bacteria |
| ASV1995 | 405 | 1 | 3 | 405 | 99.51 | NR_125464.1 Anaerobacterium chartisolvens strain T-1-35 16S ribosomal RNA, partial sequence | NR_125464.1 | 1451 | 344 | 745 | 27.71 | 466.5 | 4E-131 | 353/403 | 87 | 1/403 | 0 | Plus | Plus | Bacteria; Firmicutes; Clostridia; Eubacteriales; Oscillospiraceae; Anaerobacterium; Anaerobacterium chartisolvens | Anaerobacterium chartisolvens | Anaerobacterium | Oscillospiraceae | Eubacteriales | Clostridia | Firmicutes | Bacteria |
| ASV1996 | 403 | 1 | 3 | 403 | 99.5 | NR_125464.1 Anaerobacterium chartisolvens strain T-1-35 16S ribosomal RNA, partial sequence | NR_125464.1 | 1451 | 344 | 745 | 27.71 | 481.2 | 2E-135 | 355/402 | 88 | 1/402 | 0 | Plus | Plus | Bacteria; Firmicutes; Clostridia; Eubacteriales; Oscillospiraceae; Anaerobacterium; Anaerobacterium chartisolvens | Anaerobacterium chartisolvens | Anaerobacterium | Oscillospiraceae | Eubacteriales | Clostridia | Firmicutes | Bacteria |
| ASV1997 | 422 | 1 | 1 | 422 | 100 | NR_027196.1 Bacteroides stercoris ATCC 43183 16S ribosomal RNA, partial sequence | NR_027196.1 | 1342 | 334 | 755 | 31.45 | 665.9 | 0 | 401/422 | 95 | 0/422 | 0 | Plus | Plus | Bacteria; Bacteroidetes; Bacteroidia; Bacteroidales; Bacteroidaceae; Bacteroides; Bacteroides stercoris | Bacteroides stercoris | Bacteroides | Bacteroidaceae | Bacteroidales | Bacteroidia | Bacteroidetes | Bacteria |
| ASV1998 | 429 | 1 | 1 | 429 | 100 | NR_042769.1 Geobacter bemidjiensis Bem 16S ribosomal RNA, partial sequence | NR_042769.1 | 1354 | 342 | 768 | 31.54 | 436.9 | 4E-122 | 368/432 | 85 | 8/432 | 1 | Plus | Plus | Bacteria; Proteobacteria; Deltaproteobacteria; Desulfuromonadales; Geobacteraceae; Geobacter; Geobacter bemidjiensis | Geobacter bemidjiensis | Geobacter | Geobacteraceae | Desulfuromonadales | Deltaproteobacteria | Proteobacteria | Bacteria |
| ASV1999 | 405 | 1 | 1 | 405 | 100 | NR_044972.1 Sporobacter termitidis strain SYR 16S ribosomal RNA, partial sequence | NR_044972.1 | 1506 | 348 | 752 | 26.89 | 597.6 | 2E-170 | 378/406 | 93 | 2/406 | 0 | Plus | Plus | Bacteria; Firmicutes; Clostridia; Eubacteriales; Oscillospiraceae; Sporobacter; Sporobacter termitidis | Sporobacter termitidis | Sporobacter | Oscillospiraceae | Eubacteriales | Clostridia | Firmicutes | Bacteria |
| ASV2000 | 422 | 1 | 1 | 422 | 100 | NR_113208.1 Parabacteroides chinchillae strain JCM 17104 16S ribosomal RNA, partial sequence | NR_113208.1 | 1489 | 353 | 774 | 28.34 | 653 | 0 | 400/423 | 94 | 2/423 | 0 | Plus | Plus | Bacteria; Bacteroidetes; Bacteroidia; Bacteroidales; Tannerellaceae; Parabacteroides; Parabacteroides chinchillae | Parabacteroides chinchillae | Parabacteroides | Tannerellaceae | Bacteroidales | Bacteroidia | Bacteroidetes | Bacteria |
| ASV2001 | 402 | 1 | 1 | 402 | 100 | NR_169458.1 Tepidibaculum saccharolyticum strain STR9 16S ribosomal RNA, partial sequence | NR_169458.1 | 1477 | 362 | 763 | 27.22 | 599.4 | 4E-171 | 378/404 | 93 | 4/404 | 0 | Plus | Plus | Bacteria; Firmicutes; Clostridia; Eubacteriales; Oscillospiraceae; Tepidibaculum; Tepidibaculum saccharolyticum | Tepidibaculum saccharolyticum | Tepidibaculum | Oscillospiraceae | Eubacteriales | Clostridia | Firmicutes | Bacteria |
| ASV2002 | 405 | 1 | 1 | 405 | 100 | NR_117734.1 Fusobacterium mortiferum strain DSM 19809 16S ribosomal RNA, partial sequence | NR_117734.1 | 1482 | 326 | 730 | 27.33 | 749 | 0 | 405/405 | 100 | 0/405 | 0 | Plus | Plus | Bacteria; Fusobacteria; Fusobacteriia; Fusobacteriales; Fusobacteriaceae; Fusobacterium; Fusobacterium mortiferum | Fusobacterium mortiferum | Fusobacterium | Fusobacteriaceae | Fusobacteriales | Fusobacteriia | Fusobacteria | Bacteria |
| ASV2003 | 421 | 2 | 1 | 421 | 100 | NR_126195.1 Butyricimonas paravirosa strain 214-4 16S ribosomal RNA, partial sequence | NR_126195.1 | 1486 | 352 | 772 | 28.33 | 568 | 1E-161 | 385/423 | 91 | 4/423 | 0 | Plus | Plus | Bacteria; Bacteroidetes; Bacteroidia; Bacteroidales; Odoribacteraceae; Butyricimonas; Butyricimonas paravirosa | Butyricimonas paravirosa | Butyricimonas | Odoribacteraceae | Bacteroidales | Bacteroidia | Bacteroidetes | Bacteria |
| ASV2004 | 402 | 2 | 1 | 402 | 100 | NR_151894.1 Anaerotaenia torta strain FH052 16S ribosomal RNA, partial sequence | NR_151894.1 | 1479 | 352 | 753 | 27.18 | 688.1 | 0 | 392/402 | 97 | 0/402 | 0 | Plus | Plus | Bacteria; Firmicutes; Clostridia; Eubacteriales; Lachnospiraceae; Anaerotaenia; Anaerotaenia torta | Anaerotaenia torta | Anaerotaenia | Lachnospiraceae | Eubacteriales | Clostridia | Firmicutes | Bacteria |
| ASV2005 | 401 | 2 | 1 | 401 | 100 | NR_029355.1 [Clostridium] methylpentosum strain R2 16S ribosomal RNA, partial sequence | NR_029355.1 | 1435 | 349 | 750 | 28.01 | 520 | 3E-147 | 364/404 | 90 | 5/404 | 1 | Plus | Plus | Bacteria; Firmicutes; Clostridia; Eubacteriales; Oscillospiraceae; ; [Clostridium] methylpentosum | [Clostridium] methylpentosum | | Oscillospiraceae | Eubacteriales | Clostridia | Firmicutes | Bacteria |
| ASV2006 | 403 | 1 | 3 | 403 | 99.5 | NR_026104.1 Ruminiclostridium cellobioparum DSM 1351 = ATCC 15832 16S ribosomal RNA, partial sequence | NR_026104.1 | 1487 | 331 | 733 | 27.1 | 460.9 | 2E-129 | 352/403 | 87 | 2/403 | 0 | Plus | Plus | Bacteria; Firmicutes; Clostridia; Eubacteriales; Oscillospiraceae; Ruminiclostridium; Ruminiclostridium cellobioparum | Ruminiclostridium cellobioparum | Ruminiclostridium | Oscillospiraceae | Eubacteriales | Clostridia | Firmicutes | Bacteria |
| ASV2007 | 427 | 1 | 1 | 427 | 100 | NR_042871.1 [Actinobacillus] rossii strain ATCC 27072 16S ribosomal RNA, partial sequence | NR_042871.1 | 1364 | 333 | 759 | 31.3 | 771.2 | 0 | 423/427 | 99 | 0/427 | 0 | Plus | Plus | Bacteria; Proteobacteria; Gammaproteobacteria; Pasteurellales; Pasteurellaceae; ; [Actinobacillus] rossii | [Actinobacillus] rossii | | Pasteurellaceae | Pasteurellales | Gammaproteobacteria | Proteobacteria | Bacteria |
| ASV2009 | 426 | 1 | 1 | 426 | 100 | NR_029335.1 Holdemania filiformis strain J1-31B-1 16S ribosomal RNA, partial sequence | NR_029335.1 | 1391 | 267 | 692 | 30.63 | 455.4 | 1E-127 | 367/427 | 85 | 2/427 | 0 | Plus | Plus | Bacteria; Firmicutes; Erysipelotrichia; Erysipelotrichales; Erysipelotrichaceae; Holdemania; Holdemania filiformis | Holdemania filiformis | Holdemania | Erysipelotrichaceae | Erysipelotrichales | Erysipelotrichia | Firmicutes | Bacteria |
| ASV2010 | 422 | 1 | 1 | 422 | 100 | NR_041364.1 Prevotella stercorea DSM 18206 strain CB35 16S ribosomal RNA, partial sequence | NR_041364.1 | 1488 | 354 | 775 | 28.36 | 713.9 | 0 | 410/422 | 97 | 0/422 | 0 | Plus | Plus | Bacteria; Bacteroidetes; Bacteroidia; Bacteroidales; Prevotellaceae; Prevotella; Prevotella stercorea | Prevotella stercorea | Prevotella | Prevotellaceae | Bacteroidales | Bacteroidia | Bacteroidetes | Bacteria |
| ASV2011 | 402 | 1 | 1 | 402 | 100 | NR_026074.1 Helicobacter rodentium strain MIT 95-1707 16S ribosomal RNA, partial sequence | NR_026074.1 | 1423 | 317 | 718 | 28.25 | 737.9 | 0 | 401/402 | 99 | 0/402 | 0 | Plus | Plus | Bacteria; Proteobacteria; Epsilonproteobacteria; Campylobacterales; Helicobacteraceae; Helicobacter; Helicobacter rodentium | Helicobacter rodentium | Helicobacter | Helicobacteraceae | Campylobacterales | Epsilonproteobacteria | Proteobacteria | Bacteria |
| ASV2012 | 402 | 1 | 1 | 402 | 100 | NR_028883.1 Dorea longicatena strain 111-35 16S ribosomal RNA, partial sequence | NR_028883.1 | 1316 | 243 | 645 | 30.62 | 699.2 | 0 | 396/404 | 98 | 3/404 | 0 | Plus | Plus | Bacteria; Firmicutes; Clostridia; Eubacteriales; Lachnospiraceae; Dorea; Dorea longicatena | Dorea longicatena | Dorea | Lachnospiraceae | Eubacteriales | Clostridia | Firmicutes | Bacteria |
| ASV2013 | 427 | 3 | 1 | 427 | 100 | NR_041781.1 Streptococcus alactolyticus strain ATCC 43077 16S ribosomal RNA, partial sequence | NR_041781.1 | 1437 | 346 | 772 | 29.71 | 606.8 | 3E-173 | 398/431 | 92 | 8/431 | 1 | Plus | Plus | Bacteria; Firmicutes; Bacilli; Lactobacillales; Streptococcaceae; Streptococcus; Streptococcus alactolyticus | Streptococcus alactolyticus | Streptococcus | Streptococcaceae | Lactobacillales | Bacilli | Firmicutes | Bacteria |
| ASV2014 | 408 | 1 | 1 | 408 | 100 | NR_163613.1 Parolsenella catena strain 2CBEGH3 16S ribosomal RNA, partial sequence | NR_163613.1 | 1478 | 350 | 757 | 27.6 | 754.6 | 0 | 408/408 | 100 | 0/408 | 0 | Plus | Plus | Bacteria; Actinobacteria; Coriobacteriia; Coriobacteriales; Atopobiaceae; Parolsenella; Parolsenella catena | Parolsenella catena | Parolsenella | Atopobiaceae | Coriobacteriales | Coriobacteriia | Actinobacteria | Bacteria |
| ASV2015 | 420 | 1 | 1 | 420 | 100 | NR_144616.1 Muribaculum intestinale strain YL27 16S ribosomal RNA, partial sequence | NR_144616.1 | 1460 | 297 | 718 | 28.9 | 579.1 | 6E-165 | 386/422 | 91 | 2/422 | 0 | Plus | Plus | Bacteria; Bacteroidetes; Bacteroidia; Bacteroidales; Muribaculaceae; Muribaculum; Muribaculum intestinale | Muribaculum intestinale | Muribaculum | Muribaculaceae | Bacteroidales | Bacteroidia | Bacteroidetes | Bacteria |
| ASV2016 | 403 | 2 | 3 | 403 | 99.5 | NG_041947.1 Ruminiclostridium cellulolyticum H10 16S ribosomal RNA gene, complete sequence | NG_041947.1 | 1641 | 488 | 890 | 24.56 | 477.6 | 2E-134 | 355/403 | 88 | 2/403 | 0 | Plus | Plus | Bacteria; Firmicutes; Clostridia; Eubacteriales; Oscillospiraceae; Ruminiclostridium; Ruminiclostridium cellulolyticum | Ruminiclostridium cellulolyticum | Ruminiclostridium | Oscillospiraceae | Eubacteriales | Clostridia | Firmicutes | Bacteria |
| ASV2017 | 402 | 1 | 1 | 402 | 100 | NR_104846.1 Gemmiger formicilis strain X2-56 16S ribosomal RNA, partial sequence | NR_104846.1 | 1426 | 305 | 706 | 28.19 | 726.9 | 0 | 399/402 | 99 | 0/402 | 0 | Plus | Plus | Bacteria; Firmicutes; Clostridia; Eubacteriales; ; Gemmiger; Gemmiger formicilis | Gemmiger formicilis | Gemmiger | | Eubacteriales | Clostridia | Firmicutes | Bacteria |
| ASV2018 | 402 | 1 | 1 | 402 | 100 | NR_042832.1 Roseburia faecis strain M72/1 16S ribosomal RNA, partial sequence | NR_042832.1 | 1456 | 347 | 749 | 27.68 | 677 | 0 | 391/403 | 97 | 1/403 | 0 | Plus | Plus | Bacteria; Firmicutes; Clostridia; Eubacteriales; Lachnospiraceae; Roseburia; Roseburia faecis | Roseburia faecis | Roseburia | Lachnospiraceae | Eubacteriales | Clostridia | Firmicutes | Bacteria |
| ASV2019 | 405 | 1 | 1 | 405 | 100 | NR_044972.1 Sporobacter termitidis strain SYR 16S ribosomal RNA, partial sequence | NR_044972.1 | 1506 | 348 | 752 | 26.89 | 547.7 | 2E-155 | 369/406 | 90 | 2/406 | 0 | Plus | Plus | Bacteria; Firmicutes; Clostridia; Eubacteriales; Oscillospiraceae; Sporobacter; Sporobacter termitidis | Sporobacter termitidis | Sporobacter | Oscillospiraceae | Eubacteriales | Clostridia | Firmicutes | Bacteria |
| ASV2020 | 422 | 2 | 1 | 422 | 100 | NR_113119.1 Prevotella oris strain JCM 8540 16S ribosomal RNA, partial sequence | NR_113119.1 | 1481 | 345 | 766 | 28.49 | 597.6 | 2E-170 | 391/424 | 92 | 4/424 | 0 | Plus | Plus | Bacteria; Bacteroidetes; Bacteroidia; Bacteroidales; Prevotellaceae; Prevotella; Prevotella oris | Prevotella oris | Prevotella | Prevotellaceae | Bacteroidales | Bacteroidia | Bacteroidetes | Bacteria |
| ASV2021 | 402 | 3 | 3 | 402 | 99.5 | NR_117867.1 Blautia stercoris strain GAM6-1 16S ribosomal RNA, partial sequence | NR_117867.1 | 1372 | 319 | 718 | 29.15 | 701 | 0 | 393/400 | 98 | 0/400 | 0 | Plus | Plus | Bacteria; Firmicutes; Clostridia; Eubacteriales; Lachnospiraceae; Blautia; Blautia stercoris | Blautia stercoris | Blautia | Lachnospiraceae | Eubacteriales | Clostridia | Firmicutes | Bacteria |
| ASV2022 | 427 | 1 | 1 | 427 | 100 | NR_042896.1 Mucispirillum schaedleri strain HRI I17 16S ribosomal RNA, partial sequence | NR_042896.1 | 1471 | 333 | 759 | 29.03 | 723.2 | 0 | 415/427 | 97 | 0/427 | 0 | Plus | Plus | Bacteria; Deferribacteres; Deferribacteres; Deferribacterales; Deferribacteraceae; Mucispirillum; Mucispirillum schaedleri | Mucispirillum schaedleri | Mucispirillum | Deferribacteraceae | Deferribacterales | Deferribacteres | Deferribacteres | Bacteria |
| ASV2023 | 422 | 1 | 1 | 422 | 100 | NR_144750.1 Prevotellamassilia timonensis strain Marseille-P2831 16S ribosomal RNA, partial sequence | NR_144750.1 | 1493 | 358 | 779 | 28.27 | 547.7 | 2E-155 | 380/422 | 90 | 0/422 | 0 | Plus | Plus | Bacteria; Bacteroidetes; Bacteroidia; Bacteroidales; Prevotellaceae; Prevotellamassilia; Prevotellamassilia timonensis | Prevotellamassilia timonensis | Prevotellamassilia | Prevotellaceae | Bacteroidales | Bacteroidia | Bacteroidetes | Bacteria |
| ASV2024 | 423 | 1 | 1 | 423 | 100 | NR_144750.1 Prevotellamassilia timonensis strain Marseille-P2831 16S ribosomal RNA, partial sequence | NR_144750.1 | 1493 | 358 | 779 | 28.27 | 558.8 | 8E-159 | 386/426 | 90 | 7/426 | 1 | Plus | Plus | Bacteria; Bacteroidetes; Bacteroidia; Bacteroidales; Prevotellaceae; Prevotellamassilia; Prevotellamassilia timonensis | Prevotellamassilia timonensis | Prevotellamassilia | Prevotellaceae | Bacteroidales | Bacteroidia | Bacteroidetes | Bacteria |
| ASV2025 | 427 | 1 | 1 | 427 | 100 | NR_024994.1 Lactobacillus mucosae strain S32 16S ribosomal RNA, partial sequence | NR_024994.1 | 1568 | 384 | 810 | 27.23 | 784.1 | 0 | 426/427 | 99 | 0/427 | 0 | Plus | Plus | Bacteria; Firmicutes; Bacilli; Lactobacillales; Lactobacillaceae; Limosilactobacillus; Limosilactobacillus mucosae | Limosilactobacillus mucosae | Limosilactobacillus | Lactobacillaceae | Lactobacillales | Bacilli | Firmicutes | Bacteria |
| ASV2026 | 402 | 1 | 1 | 402 | 100 | NR_029239.3 Clostridium chartatabidum strain 163 16S ribosomal RNA, partial sequence | NR_029239.3 | 1460 | 323 | 724 | 27.53 | 704.7 | 0 | 395/402 | 98 | 0/402 | 0 | Plus | Plus | Bacteria; Firmicutes; Clostridia; Eubacteriales; Clostridiaceae; Clostridium; Clostridium chartatabidum | Clostridium chartatabidum | Clostridium | Clostridiaceae | Eubacteriales | Clostridia | Firmicutes | Bacteria |
| ASV2027 | 407 | 1 | 1 | 407 | 100 | NR_025310.1 Rothia nasimurium strain CCUG 35957 16S ribosomal RNA, partial sequence | NR_025310.1 | 1481 | 332 | 738 | 27.48 | 736.1 | 0 | 404/407 | 99 | 0/407 | 0 | Plus | Plus | Bacteria; Actinobacteria; Actinomycetia; Micrococcales; Micrococcaceae; Rothia; Rothia nasimurium | Rothia nasimurium | Rothia | Micrococcaceae | Micrococcales | Actinomycetia | Actinobacteria | Bacteria |
| ASV2028 | 402 | 1 | 1 | 402 | 100 | NR_144612.1 Frisingicoccus caecimuris strain PG-426-CC-1 16S ribosomal RNA, partial sequence | NR_144612.1 | 1448 | 367 | 768 | 27.76 | 704.7 | 0 | 395/402 | 98 | 0/402 | 0 | Plus | Plus | Bacteria; Firmicutes; Clostridia; Eubacteriales; Lachnospiraceae; Frisingicoccus; Frisingicoccus caecimuris | Frisingicoccus caecimuris | Frisingicoccus | Lachnospiraceae | Eubacteriales | Clostridia | Firmicutes | Bacteria |
| ASV2029 | 402 | 1 | 1 | 402 | 100 | NR_144612.1 Frisingicoccus caecimuris strain PG-426-CC-1 16S ribosomal RNA, partial sequence | NR_144612.1 | 1448 | 367 | 768 | 27.76 | 710.2 | 0 | 396/402 | 98 | 0/402 | 0 | Plus | Plus | Bacteria; Firmicutes; Clostridia; Eubacteriales; Lachnospiraceae; Frisingicoccus; Frisingicoccus caecimuris | Frisingicoccus caecimuris | Frisingicoccus | Lachnospiraceae | Eubacteriales | Clostridia | Firmicutes | Bacteria |
| ASV2030 | 405 | 1 | 1 | 405 | 100 | NR_144611.1 Flintibacter butyricus strain BLS21 16S ribosomal RNA, partial sequence | NR_144611.1 | 808 | 299 | 703 | 50.12 | 499.7 | 4E-141 | 361/406 | 88 | 2/406 | 0 | Plus | Plus | Bacteria; Firmicutes; Clostridia; Eubacteriales; ; Flintibacter; Flintibacter butyricus | Flintibacter butyricus | Flintibacter | | Eubacteriales | Clostridia | Firmicutes | Bacteria |
| ASV2031 | 427 | 2 | 15 | 427 | 96.72 | NR_025713.1 Spiroplasma velocicrescens strain MQ-4 16S ribosomal RNA, partial sequence | NR_025713.1 | 1515 | 359 | 770 | 27.19 | 440.6 | 3E-123 | 356/414 | 85 | 3/414 | 0 | Plus | Plus | Bacteria; Tenericutes; Mollicutes; Entomoplasmatales; Spiroplasmataceae; Spiroplasma; Spiroplasma velocicrescens | Spiroplasma velocicrescens | Spiroplasma | Spiroplasmataceae | Entomoplasmatales | Mollicutes | Tenericutes | Bacteria |
| ASV2032 | 404 | 1 | 1 | 404 | 100 | NR_115340.1 Saccharofermentans acetigenes strain P6 16S ribosomal RNA, partial sequence | NR_115340.1 | 1510 | 353 | 757 | 26.82 | 414.8 | 2E-115 | 350/410 | 85 | 11/410 | 2 | Plus | Plus | Bacteria; Firmicutes; Clostridia; Eubacteriales; Oscillospiraceae; Saccharofermentans; Saccharofermentans acetigenes | Saccharofermentans acetigenes | Saccharofermentans | Oscillospiraceae | Eubacteriales | Clostridia | Firmicutes | Bacteria |
| ASV2033 | 422 | 1 | 1 | 422 | 100 | NR_024815.1 Prevotella shahii strain EHS11 16S ribosomal RNA, partial sequence | NR_024815.1 | 1490 | 356 | 777 | 28.32 | 597.6 | 2E-170 | 391/424 | 92 | 4/424 | 0 | Plus | Plus | Bacteria; Bacteroidetes; Bacteroidia; Bacteroidales; Prevotellaceae; Prevotella; Prevotella shahii | Prevotella shahii | Prevotella | Prevotellaceae | Bacteroidales | Bacteroidia | Bacteroidetes | Bacteria |
| ASV2034 | 405 | 1 | 1 | 405 | 100 | NR_146837.2 Fusobacterium gastrosuis strain CDW1 16S ribosomal RNA, complete sequence | NR_146837.2 | 1506 | 343 | 747 | 26.89 | 737.9 | 0 | 403/405 | 99 | 0/405 | 0 | Plus | Plus | Bacteria; Fusobacteria; Fusobacteriia; Fusobacteriales; Fusobacteriaceae; Fusobacterium; Fusobacterium gastrosuis | Fusobacterium gastrosuis | Fusobacterium | Fusobacteriaceae | Fusobacteriales | Fusobacteriia | Fusobacteria | Bacteria |
| ASV2035 | 422 | 1 | 1 | 422 | 100 | NR_113411.1 Prevotella copri DSM 18205 strain JCM 13464 16S ribosomal RNA, partial sequence | NR_113411.1 | 1493 | 358 | 779 | 28.27 | 741.6 | 0 | 415/422 | 98 | 0/422 | 0 | Plus | Plus | Bacteria; Bacteroidetes; Bacteroidia; Bacteroidales; Prevotellaceae; Prevotella; Prevotella copri | Prevotella copri | Prevotella | Prevotellaceae | Bacteroidales | Bacteroidia | Bacteroidetes | Bacteria |
| ASV2036 | 427 | 1 | 1 | 427 | 100 | NR_025600.1 Sutterella stercoricanis strain 5BAC4 16S ribosomal RNA, partial sequence | NR_025600.1 | 1455 | 319 | 745 | 29.35 | 761.9 | 0 | 422/427 | 98 | 0/427 | 0 | Plus | Plus | Bacteria; Proteobacteria; Betaproteobacteria; Burkholderiales; Sutterellaceae; Sutterella; Sutterella stercoricanis | Sutterella stercoricanis | Sutterella | Sutterellaceae | Burkholderiales | Betaproteobacteria | Proteobacteria | Bacteria |
| ASV2037 | 422 | 2 | 1 | 422 | 100 | NR_113119.1 Prevotella oris strain JCM 8540 16S ribosomal RNA, partial sequence | NR_113119.1 | 1481 | 345 | 766 | 28.49 | 619.7 | 3E-177 | 394/423 | 93 | 2/423 | 0 | Plus | Plus | Bacteria; Bacteroidetes; Bacteroidia; Bacteroidales; Prevotellaceae; Prevotella; Prevotella oris | Prevotella oris | Prevotella | Prevotellaceae | Bacteroidales | Bacteroidia | Bacteroidetes | Bacteria |
| ASV2038 | 422 | 1 | 1 | 422 | 100 | NR_113411.1 Prevotella copri DSM 18205 strain JCM 13464 16S ribosomal RNA, partial sequence | NR_113411.1 | 1493 | 358 | 779 | 28.27 | 619.7 | 3E-177 | 394/423 | 93 | 2/423 | 0 | Plus | Plus | Bacteria; Bacteroidetes; Bacteroidia; Bacteroidales; Prevotellaceae; Prevotella; Prevotella copri | Prevotella copri | Prevotella | Prevotellaceae | Bacteroidales | Bacteroidia | Bacteroidetes | Bacteria |
| ASV2039 | 422 | 1 | 1 | 422 | 100 | NR_024815.1 Prevotella shahii strain EHS11 16S ribosomal RNA, partial sequence | NR_024815.1 | 1490 | 356 | 777 | 28.32 | 547.7 | 2E-155 | 382/424 | 90 | 4/424 | 0 | Plus | Plus | Bacteria; Bacteroidetes; Bacteroidia; Bacteroidales; Prevotellaceae; Prevotella; Prevotella shahii | Prevotella shahii | Prevotella | Prevotellaceae | Bacteroidales | Bacteroidia | Bacteroidetes | Bacteria |
| ASV2040 | 421 | 1 | 1 | 421 | 100 | NR_144616.1 Muribaculum intestinale strain YL27 16S ribosomal RNA, partial sequence | NR_144616.1 | 1460 | 297 | 718 | 28.9 | 496 | 6E-140 | 376/427 | 88 | 11/427 | 2 | Plus | Plus | Bacteria; Bacteroidetes; Bacteroidia; Bacteroidales; Muribaculaceae; Muribaculum; Muribaculum intestinale | Muribaculum intestinale | Muribaculum | Muribaculaceae | Bacteroidales | Bacteroidia | Bacteroidetes | Bacteria |
| ASV2041 | 421 | 1 | 1 | 421 | 100 | NR_144616.1 Muribaculum intestinale strain YL27 16S ribosomal RNA, partial sequence | NR_144616.1 | 1460 | 297 | 718 | 28.9 | 484.9 | 1E-136 | 375/428 | 87 | 13/428 | 3 | Plus | Plus | Bacteria; Bacteroidetes; Bacteroidia; Bacteroidales; Muribaculaceae; Muribaculum; Muribaculum intestinale | Muribaculum intestinale | Muribaculum | Muribaculaceae | Bacteroidales | Bacteroidia | Bacteroidetes | Bacteria |
| ASV2042 | 402 | 1 | 1 | 402 | 100 | NR_029239.3 Clostridium chartatabidum strain 163 16S ribosomal RNA, partial sequence | NR_029239.3 | 1460 | 323 | 724 | 27.53 | 715.8 | 0 | 397/402 | 98 | 0/402 | 0 | Plus | Plus | Bacteria; Firmicutes; Clostridia; Eubacteriales; Clostridiaceae; Clostridium; Clostridium chartatabidum | Clostridium chartatabidum | Clostridium | Clostridiaceae | Eubacteriales | Clostridia | Firmicutes | Bacteria |
| ASV2043 | 404 | 1 | 1 | 404 | 100 | NR_114789.1 [Clostridium] leptum strain DSM 753 16S ribosomal RNA, partial sequence | NR_114789.1 | 1505 | 364 | 765 | 26.71 | 518.2 | 1E-146 | 364/405 | 89 | 4/405 | 0 | Plus | Plus | Bacteria; Firmicutes; Clostridia; Eubacteriales; Oscillospiraceae; ; [Clostridium] leptum | [Clostridium] leptum | | Oscillospiraceae | Eubacteriales | Clostridia | Firmicutes | Bacteria |
| ASV2044 | 422 | 2 | 1 | 422 | 100 | NR_074376.1 Parabacteroides distasonis ATCC 8503 16S ribosomal RNA, partial sequence | NR_074376.1 | 1497 | 368 | 789 | 28.19 | 575.4 | 7E-164 | 385/422 | 91 | 0/422 | 0 | Plus | Plus | Bacteria; Bacteroidetes; Bacteroidia; Bacteroidales; Tannerellaceae; Parabacteroides; Parabacteroides distasonis | Parabacteroides distasonis | Parabacteroides | Tannerellaceae | Bacteroidales | Bacteroidia | Bacteroidetes | Bacteria |
| ASV2045 | 405 | 2 | 1 | 405 | 100 | NR_074629.1 Hungateiclostridium thermocellum strain ATCC 27405 16S ribosomal RNA, partial sequence | NR_074629.1 | 1521 | 358 | 762 | 26.63 | 510.8 | 2E-144 | 362/405 | 89 | 0/405 | 0 | Plus | Plus | Bacteria; Firmicutes; Clostridia; Eubacteriales; Oscillospiraceae; Acetivibrio; Acetivibrio thermocellus | Acetivibrio thermocellus | Acetivibrio | Oscillospiraceae | Eubacteriales | Clostridia | Firmicutes | Bacteria |
| ASV2046 | 403 | 1 | 1 | 403 | 100 | NR_147398.1 Anaerotruncus rubiinfantis strain MT15 16S ribosomal RNA, partial sequence | NR_147398.1 | 1490 | 368 | 768 | 26.91 | 549.6 | 4E-156 | 369/404 | 91 | 4/404 | 0 | Plus | Plus | Bacteria; Firmicutes; Clostridia; Eubacteriales; Oscillospiraceae; Anaerotruncus; Anaerotruncus rubiinfantis | Anaerotruncus rubiinfantis | Anaerotruncus | Oscillospiraceae | Eubacteriales | Clostridia | Firmicutes | Bacteria |
| ASV2047 | 405 | 1 | 1 | 405 | 100 | NR_044972.1 Sporobacter termitidis strain SYR 16S ribosomal RNA, partial sequence | NR_044972.1 | 1506 | 348 | 752 | 26.89 | 547.7 | 2E-155 | 369/406 | 90 | 2/406 | 0 | Plus | Plus | Bacteria; Firmicutes; Clostridia; Eubacteriales; Oscillospiraceae; Sporobacter; Sporobacter termitidis | Sporobacter termitidis | Sporobacter | Oscillospiraceae | Eubacteriales | Clostridia | Firmicutes | Bacteria |
| ASV2048 | 426 | 2 | 1 | 426 | 100 | NR_042260.1 Sphaerochaeta coccoides strain SPN1 16S ribosomal RNA, partial sequence | NR_042260.1 | 1500 | 320 | 746 | 28.47 | 510.8 | 2E-144 | 379/429 | 88 | 5/429 | 1 | Plus | Plus | Bacteria; Spirochaetes; Spirochaetia; Spirochaetales; Spirochaetaceae; Sphaerochaeta; Sphaerochaeta coccoides | Sphaerochaeta coccoides | Sphaerochaeta | Spirochaetaceae | Spirochaetales | Spirochaetia | Spirochaetes | Bacteria |
| ASV2049 | 405 | 1 | 1 | 405 | 100 | NR_144742.1 Christensenella massiliensis strain Marseille-P2438 16S ribosomal RNA, partial sequence | NR_144742.1 | 1499 | 369 | 773 | 27.02 | 472 | 1E-132 | 355/405 | 87 | 0/405 | 0 | Plus | Plus | Bacteria; Firmicutes; Clostridia; Eubacteriales; Christensenellaceae; Christensenella; Christensenella massiliensis | Christensenella massiliensis | Christensenella | Christensenellaceae | Eubacteriales | Clostridia | Firmicutes | Bacteria |
| ASV2050 | 422 | 1 | 1 | 422 | 100 | NR_113411.1 Prevotella copri DSM 18205 strain JCM 13464 16S ribosomal RNA, partial sequence | NR_113411.1 | 1493 | 358 | 779 | 28.27 | 597.6 | 2E-170 | 389/422 | 92 | 0/422 | 0 | Plus | Plus | Bacteria; Bacteroidetes; Bacteroidia; Bacteroidales; Prevotellaceae; Prevotella; Prevotella copri | Prevotella copri | Prevotella | Prevotellaceae | Bacteroidales | Bacteroidia | Bacteroidetes | Bacteria |
| ASV2051 | 422 | 1 | 1 | 422 | 100 | NR_113411.1 Prevotella copri DSM 18205 strain JCM 13464 16S ribosomal RNA, partial sequence | NR_113411.1 | 1493 | 358 | 779 | 28.27 | 608.7 | 7E-174 | 394/425 | 92 | 6/425 | 1 | Plus | Plus | Bacteria; Bacteroidetes; Bacteroidia; Bacteroidales; Prevotellaceae; Prevotella; Prevotella copri | Prevotella copri | Prevotella | Prevotellaceae | Bacteroidales | Bacteroidia | Bacteroidetes | Bacteria |
| ASV2052 | 427 | 1 | 1 | 426 | 99.77 | NR_027596.1 Mitsuokella multacida strain DSM 20544 16S ribosomal RNA, partial sequence | NR_027596.1 | 1511 | 346 | 771 | 28.19 | 651.1 | 0 | 401/426 | 94 | 0/426 | 0 | Plus | Plus | Bacteria; Firmicutes; Negativicutes; Selenomonadales; Selenomonadaceae; Mitsuokella; Mitsuokella multacida | Mitsuokella multacida | Mitsuokella | Selenomonadaceae | Selenomonadales | Negativicutes | Firmicutes | Bacteria |
| ASV2053 | 422 | 3 | 1 | 422 | 100 | NR_113110.1 Prevotella maculosa strain JCM 15638 16S ribosomal RNA, partial sequence | NR_113110.1 | 1405 | 269 | 690 | 30.04 | 653 | 0 | 399/422 | 94 | 0/422 | 0 | Plus | Plus | Bacteria; Bacteroidetes; Bacteroidia; Bacteroidales; Prevotellaceae; Prevotella; Prevotella maculosa | Prevotella maculosa | Prevotella | Prevotellaceae | Bacteroidales | Bacteroidia | Bacteroidetes | Bacteria |
| ASV2054 | 402 | 1 | 1 | 402 | 100 | NR_028827.1 Shuttleworthia satelles DSM 14600 strain VPI D143K-13 16S ribosomal RNA, partial sequence | NR_028827.1 | 1456 | 345 | 749 | 27.82 | 601.3 | 1E-171 | 379/405 | 93 | 3/405 | 0 | Plus | Plus | Bacteria; Firmicutes; Clostridia; Eubacteriales; Lachnospiraceae; Shuttleworthia; Shuttleworthia satelles | Shuttleworthia satelles | Shuttleworthia | Lachnospiraceae | Eubacteriales | Clostridia | Firmicutes | Bacteria |
| ASV2055 | 403 | 1 | 1 | 403 | 100 | NR_025930.1 Ruminococcus bromii strain ATCC 27255 16S ribosomal RNA, partial sequence | NR_025930.1 | 1380 | 313 | 716 | 29.28 | 584.7 | 1E-166 | 375/404 | 92 | 1/404 | 0 | Plus | Plus | Bacteria; Firmicutes; Clostridia; Eubacteriales; Oscillospiraceae; Ruminococcus; Ruminococcus bromii | Ruminococcus bromii | Ruminococcus | Oscillospiraceae | Eubacteriales | Clostridia | Firmicutes | Bacteria |
| ASV2056 | 402 | 1 | 1 | 402 | 100 | NR_029239.3 Clostridium chartatabidum strain 163 16S ribosomal RNA, partial sequence | NR_029239.3 | 1460 | 323 | 724 | 27.53 | 699.2 | 0 | 394/402 | 98 | 0/402 | 0 | Plus | Plus | Bacteria; Firmicutes; Clostridia; Eubacteriales; Clostridiaceae; Clostridium; Clostridium chartatabidum | Clostridium chartatabidum | Clostridium | Clostridiaceae | Eubacteriales | Clostridia | Firmicutes | Bacteria |
| ASV2057 | 404 | 1 | 1 | 404 | 100 | NR_044490.1 Butyricicoccus pullicaecorum strain 25-3 16S ribosomal RNA, partial sequence | NR_044490.1 | 1482 | 366 | 770 | 27.33 | 597.6 | 2E-170 | 379/406 | 93 | 3/406 | 0 | Plus | Plus | Bacteria; Firmicutes; Clostridia; Eubacteriales; Clostridiaceae; Butyricicoccus; Butyricicoccus pullicaecorum | Butyricicoccus pullicaecorum | Butyricicoccus | Clostridiaceae | Eubacteriales | Clostridia | Firmicutes | Bacteria |
| ASV2058 | 403 | 1 | 3 | 403 | 99.5 | NR_125677.1 Vallitalea pronyensis strain FatNI3 16S ribosomal RNA, partial sequence | NR_125677.1 | 1480 | 365 | 764 | 27.03 | 462.8 | 6E-130 | 353/403 | 87 | 5/403 | 1 | Plus | Plus | Bacteria; Firmicutes; Clostridia; Eubacteriales; Vallitaleaceae; Vallitalea; Vallitalea pronyensis | Vallitalea pronyensis | Vallitalea | Vallitaleaceae | Eubacteriales | Clostridia | Firmicutes | Bacteria |
| ASV2061 | 411 | 1 | 1 | 411 | 100 | NR_036854.2 Bifidobacterium boum strain RU917 16S ribosomal RNA, partial sequence | NR_036854.2 | 1514 | 343 | 753 | 27.15 | 749 | 0 | 409/411 | 99 | 0/411 | 0 | Plus | Plus | Bacteria; Actinobacteria; Actinomycetia; Bifidobacteriales; Bifidobacteriaceae; Bifidobacterium; Bifidobacterium boum | Bifidobacterium boum | Bifidobacterium | Bifidobacteriaceae | Bifidobacteriales | Actinomycetia | Actinobacteria | Bacteria |
| ASV2062 | 401 | 1 | 1 | 401 | 100 | NR_144749.1 Ihubacter massiliensis strain Marseille-P2843 16S ribosomal RNA, partial sequence | NR_144749.1 | 1479 | 364 | 766 | 27.25 | 632.7 | 0 | 383/403 | 95 | 2/403 | 0 | Plus | Plus | Bacteria; Firmicutes; Clostridia; Eubacteriales; Clostridiales Family XIII. Incertae Sedis; Ihubacter; Ihubacter massiliensis | Ihubacter massiliensis | Ihubacter | Clostridiales Family XIII. Incertae Sedis | Eubacteriales | Clostridia | Firmicutes | Bacteria |
| ASV2063 | 422 | 1 | 1 | 422 | 100 | NR_112835.1 Parabacteroides gordonii strain MS-1 16S ribosomal RNA, partial sequence | NR_112835.1 | 1485 | 353 | 773 | 28.35 | 767.5 | 0 | 420/422 | 99 | 1/422 | 0 | Plus | Plus | Bacteria; Bacteroidetes; Bacteroidia; Bacteroidales; Tannerellaceae; Parabacteroides; Parabacteroides gordonii | Parabacteroides gordonii | Parabacteroides | Tannerellaceae | Bacteroidales | Bacteroidia | Bacteroidetes | Bacteria |
| ASV2064 | 405 | 1 | 1 | 405 | 100 | NR_118554.1 Intestinimonas butyriciproducens strain SRB-521-5-I 16S ribosomal RNA, partial sequence | NR_118554.1 | 1475 | 346 | 750 | 27.46 | 505.3 | 1E-142 | 362/406 | 89 | 2/406 | 0 | Plus | Plus | Bacteria; Firmicutes; Clostridia; Eubacteriales; ; Intestinimonas; Intestinimonas butyriciproducens | Intestinimonas butyriciproducens | Intestinimonas | | Eubacteriales | Clostridia | Firmicutes | Bacteria |
| ASV2065 | 402 | 2 | 1 | 402 | 100 | NR_137405.1 Anaerobium acetethylicum strain GluBS11 16S ribosomal RNA, partial sequence | NR_137405.1 | 1402 | 324 | 725 | 28.67 | 649.3 | 0 | 385/402 | 95 | 0/402 | 0 | Plus | Plus | Bacteria; Firmicutes; Clostridia; Eubacteriales; Lachnospiraceae; Anaerobium; Anaerobium acetethylicum | Anaerobium acetethylicum | Anaerobium | Lachnospiraceae | Eubacteriales | Clostridia | Firmicutes | Bacteria |
| ASV2066 | 402 | 1 | 1 | 402 | 100 | NR_026476.1 Succinivibrio dextrinosolvens strain 0554 16S ribosomal RNA, partial sequence | NR_026476.1 | 1463 | 331 | 732 | 27.48 | 544 | 2E-154 | 367/403 | 91 | 2/403 | 0 | Plus | Plus | Bacteria; Proteobacteria; Gammaproteobacteria; Aeromonadales; Succinivibrionaceae; Succinivibrio; Succinivibrio dextrinosolvens | Succinivibrio dextrinosolvens | Succinivibrio | Succinivibrionaceae | Aeromonadales | Gammaproteobacteria | Proteobacteria | Bacteria |
| ASV2067 | 427 | 1 | 1 | 427 | 100 | NR_042942.1 Treponema porcinum strain 14V28 16S ribosomal RNA, partial sequence | NR_042942.1 | 1426 | 258 | 684 | 29.94 | 778.6 | 0 | 425/427 | 99 | 0/427 | 0 | Plus | Plus | Bacteria; Spirochaetes; Spirochaetia; Spirochaetales; Spirochaetaceae; Treponema; Treponema porcinum | Treponema porcinum | Treponema | Spirochaetaceae | Spirochaetales | Spirochaetia | Spirochaetes | Bacteria |
| ASV2068 | 403 | 1 | 1 | 398 | 98.76 | NR_104911.1 Vampirovibrio chlorellavorus strain ICPB 3707 16S ribosomal RNA, partial sequence | NR_104911.1 | 1446 | 333 | 729 | 27.46 | 418.5 | 1E-116 | 343/400 | 85 | 5/400 | 1 | Plus | Plus | Bacteria; Candidatus Melainabacteria; ; Vampirovibrionales; ; Vampirovibrio; Vampirovibrio chlorellavorus | Vampirovibrio chlorellavorus | Vampirovibrio | | Vampirovibrionales | | Candidatus Melainabacteria | Bacteria |
| ASV2069 | 405 | 2 | 1 | 405 | 100 | NR_042007.1 Roseburia inulinivorans DSM 16841 strain A2-194 16S ribosomal RNA, partial sequence | NR_042007.1 | 1454 | 360 | 763 | 27.79 | 664.1 | 0 | 392/407 | 96 | 5/407 | 1 | Plus | Plus | Bacteria; Firmicutes; Clostridia; Eubacteriales; Lachnospiraceae; Roseburia; Roseburia inulinivorans | Roseburia inulinivorans | Roseburia | Lachnospiraceae | Eubacteriales | Clostridia | Firmicutes | Bacteria |
| ASV2070 | 427 | 1 | 1 | 427 | 100 | NR_042797.1 Treponema berlinense strain 7CPL208 16S ribosomal RNA, partial sequence | NR_042797.1 | 1440 | 275 | 701 | 29.65 | 579.1 | 6E-165 | 389/427 | 91 | 0/427 | 0 | Plus | Plus | Bacteria; Spirochaetes; Spirochaetia; Spirochaetales; Spirochaetaceae; Treponema; Treponema berlinense | Treponema berlinense | Treponema | Spirochaetaceae | Spirochaetales | Spirochaetia | Spirochaetes | Bacteria |
| ASV2071 | 405 | 1 | 1 | 405 | 100 | NR_104907.1 Eubacterium coprostanoligenes strain HL 16S ribosomal RNA, partial sequence | NR_104907.1 | 1446 | 332 | 734 | 27.87 | 586.5 | 3E-167 | 376/405 | 92 | 2/405 | 0 | Plus | Plus | Bacteria; Firmicutes; Clostridia; Eubacteriales; Eubacteriaceae; Eubacterium; Eubacterium coprostanoligenes | Eubacterium coprostanoligenes | Eubacterium | Eubacteriaceae | Eubacteriales | Clostridia | Firmicutes | Bacteria |
| ASV2072 | 421 | 1 | 1 | 421 | 100 | NR_144616.1 Muribaculum intestinale strain YL27 16S ribosomal RNA, partial sequence | NR_144616.1 | 1460 | 297 | 718 | 28.9 | 484.9 | 1E-136 | 369/422 | 87 | 1/422 | 0 | Plus | Plus | Bacteria; Bacteroidetes; Bacteroidia; Bacteroidales; Muribaculaceae; Muribaculum; Muribaculum intestinale | Muribaculum intestinale | Muribaculum | Muribaculaceae | Bacteroidales | Bacteroidia | Bacteroidetes | Bacteria |
| ASV2073 | 403 | 1 | 1 | 403 | 100 | NR_029160.1 Ruminococcus callidus strain ATCC 27760 16S ribosomal RNA, partial sequence | NR_029160.1 | 1371 | 310 | 711 | 29.32 | 610.5 | 2E-174 | 381/405 | 94 | 5/405 | 1 | Plus | Plus | Bacteria; Firmicutes; Clostridia; Eubacteriales; Oscillospiraceae; Ruminococcus; Ruminococcus callidus | Ruminococcus callidus | Ruminococcus | Oscillospiraceae | Eubacteriales | Clostridia | Firmicutes | Bacteria |
| ASV2074 | 422 | 2 | 1 | 422 | 100 | NR_118334.1 Alloprevotella rava strain 81/4-12 16S ribosomal RNA, partial sequence | NR_118334.1 | 1455 | 338 | 759 | 29 | 542.2 | 8E-154 | 384/427 | 89 | 10/427 | 2 | Plus | Plus | Bacteria; Bacteroidetes; Bacteroidia; Bacteroidales; Prevotellaceae; Alloprevotella; Alloprevotella rava | Alloprevotella rava | Alloprevotella | Prevotellaceae | Bacteroidales | Bacteroidia | Bacteroidetes | Bacteria |
| ASV2075 | 422 | 1 | 1 | 422 | 100 | NR_041342.1 Parabacteroides distasonis strain JCM 5825 16S ribosomal RNA, partial sequence | NR_041342.1 | 1488 | 353 | 774 | 28.36 | 641.9 | 0 | 397/422 | 94 | 0/422 | 0 | Plus | Plus | Bacteria; Bacteroidetes; Bacteroidia; Bacteroidales; Tannerellaceae; Parabacteroides; Parabacteroides distasonis | Parabacteroides distasonis | Parabacteroides | Tannerellaceae | Bacteroidales | Bacteroidia | Bacteroidetes | Bacteria |
| ASV2076 | 402 | 1 | 1 | 402 | 100 | NR_134005.1 Clostridium bornimense strain M2/40 16S ribosomal RNA, partial sequence | NR_134005.1 | 1363 | 284 | 685 | 29.49 | 737.9 | 0 | 401/402 | 99 | 0/402 | 0 | Plus | Plus | Bacteria; Firmicutes; Clostridia; Eubacteriales; Clostridiaceae; Clostridium; Clostridium bornimense | Clostridium bornimense | Clostridium | Clostridiaceae | Eubacteriales | Clostridia | Firmicutes | Bacteria |
| ASV2077 | 422 | 1 | 1 | 422 | 100 | NR_041364.1 Prevotella stercorea DSM 18206 strain CB35 16S ribosomal RNA, partial sequence | NR_041364.1 | 1488 | 354 | 775 | 28.36 | 547.7 | 2E-155 | 381/423 | 90 | 2/423 | 0 | Plus | Plus | Bacteria; Bacteroidetes; Bacteroidia; Bacteroidales; Prevotellaceae; Prevotella; Prevotella stercorea | Prevotella stercorea | Prevotella | Prevotellaceae | Bacteroidales | Bacteroidia | Bacteroidetes | Bacteria |
| ASV2078 | 405 | 1 | 1 | 405 | 100 | NR_044508.1 Thermotalea metallivorans strain B2-1 16S ribosomal RNA, partial sequence | NR_044508.1 | 1627 | 467 | 868 | 24.77 | 451.7 | 1E-126 | 352/405 | 86 | 3/405 | 0 | Plus | Plus | Bacteria; Firmicutes; Clostridia; Eubacteriales; Clostridiaceae; Thermotalea; Thermotalea metallivorans | Thermotalea metallivorans | Thermotalea | Clostridiaceae | Eubacteriales | Clostridia | Firmicutes | Bacteria |
| ASV2079 | 405 | 1 | 1 | 405 | 100 | NR_118554.1 Intestinimonas butyriciproducens strain SRB-521-5-I 16S ribosomal RNA, partial sequence | NR_118554.1 | 1475 | 346 | 750 | 27.46 | 494.2 | 2E-139 | 361/407 | 88 | 4/407 | 0 | Plus | Plus | Bacteria; Firmicutes; Clostridia; Eubacteriales; ; Intestinimonas; Intestinimonas butyriciproducens | Intestinimonas butyriciproducens | Intestinimonas | | Eubacteriales | Clostridia | Firmicutes | Bacteria |
| ASV2080 | 422 | 1 | 1 | 422 | 100 | NR_041342.1 Parabacteroides distasonis strain JCM 5825 16S ribosomal RNA, partial sequence | NR_041342.1 | 1488 | 353 | 774 | 28.36 | 575.4 | 7E-164 | 385/422 | 91 | 0/422 | 0 | Plus | Plus | Bacteria; Bacteroidetes; Bacteroidia; Bacteroidales; Tannerellaceae; Parabacteroides; Parabacteroides distasonis | Parabacteroides distasonis | Parabacteroides | Tannerellaceae | Bacteroidales | Bacteroidia | Bacteroidetes | Bacteria |
| ASV2081 | 427 | 2 | 1 | 426 | 99.77 | NR_117778.1 Sutterella wadsworthensis strain WAL 9799 16S ribosomal RNA, partial sequence | NR_117778.1 | 1462 | 310 | 735 | 29.14 | 605 | 1E-172 | 396/429 | 92 | 6/429 | 1 | Plus | Plus | Bacteria; Proteobacteria; Betaproteobacteria; Burkholderiales; Sutterellaceae; Sutterella; Sutterella wadsworthensis | Sutterella wadsworthensis | Sutterella | Sutterellaceae | Burkholderiales | Betaproteobacteria | Proteobacteria | Bacteria |

| | | | | | | | | | | | | | | | | | | | | | | | | | | | | |
|---|---|---|---|---|---|---|---|---|---|---|---|---|---|---|---|---|---|---|---|---|---|---|---|---|---|---|---|---|
| ASV2088 | 423 | 1 | 1 | 423 | 100 | NR_144750.1 Prevotellamassilia timonensis strain Marseille-P2831 16S ribosomal RNA, partial sequence | NR_144750.1 | 1493 | 358 | 779 | 28.27 | 542.2 | 8E-154 | 383/426 | 89 | 7/426 | 1 | Plus | Plus | Bacteria; Bacteroidetes; Bacteroidia; Bacteroidales; Prevotellaceae; Prevotellamassilia; Prevotellamassilia timonensis | Prevotellamassilia timonensis | Prevotellamassilia timonensis | Prevotellamassilia | Prevotellaceae | Bacteroidales | Bacteroidia | Bacteroidetes | Bacteria |
| ASV2089 | 402 | 3 | 1 | 402 | 100 | NR_114315.1 Blautia luti DSM 14534 16S ribosomal RNA, partial sequence | NR_114315.1 | 1481 | 353 | 754 | 27.14 | 677 | 0 | 390/402 | 97 | 0/402 | 0 | Plus | Plus | Bacteria; Firmicutes; Clostridia; Eubacteriales; Lachnospiraceae; Blautia; Blautia luti | Blautia luti | Blautia luti | Blautia | Lachnospiraceae | Eubacteriales | Clostridia | Firmicutes | Bacteria |
| ASV2090 | 402 | 1 | 1 | 402 | 100 | NR_104846.1 Gemmiger formicilis strain X2-56 16S ribosomal RNA, partial sequence | NR_104846.1 | 1426 | 305 | 706 | 28.19 | 737.9 | 0 | 401/402 | 99 | 0/402 | 0 | Plus | Plus | Bacteria; Firmicutes; Clostridia; Eubacteriales; ; Gemmiger; Gemmiger formicilis | Gemmiger formicilis | Gemmiger formicilis | Gemmiger | | Eubacteriales | Clostridia | Firmicutes | Bacteria |
| ASV2091 | 427 | 2 | 1 | 427 | 100 | NR_118848.1 Actinobacillus indolicus strain 46KC2 16S ribosomal RNA, partial sequence | NR_118848.1 | 1464 | 333 | 759 | 29.17 | 756.4 | 0 | 421/427 | 98 | 0/427 | 0 | Plus | Plus | Bacteria; Proteobacteria; Gammaproteobacteria; Pasteurellales; Pasteurellaceae; Actinobacillus; Actinobacillus indolicus | Actinobacillus indolicus | Actinobacillus indolicus | Actinobacillus | Pasteurellaceae | Pasteurellales | Gammaproteobacteria | Proteobacteria | Bacteria |
| ASV2092 | 422 | 2 | 1 | 422 | 100 | NR_113117.1 Prevotella oralis ATCC 33269 strain JCM 12251 16S ribosomal RNA, partial sequence | NR_113117.1 | 1496 | 361 | 782 | 28.21 | 630.8 | 2E-180 | 395/422 | 93 | 0/422 | 0 | Plus | Plus | Bacteria; Bacteroidetes; Bacteroidia; Bacteroidales; Prevotellaceae; Prevotella; Prevotella oralis | Prevotella oralis | Prevotella oralis | Prevotella | Prevotellaceae | Bacteroidales | Bacteroidia | Bacteroidetes | Bacteria |
| ASV2094 | 422 | 1 | 1 | 422 | 100 | NR_145844.1 Bacteroides caecigallinarum strain C13EG111 16S ribosomal RNA, partial sequence | NR_145844.1 | 1489 | 356 | 777 | 28.34 | 608.7 | 7E-174 | 392/423 | 92 | 2/423 | 0 | Plus | Plus | Bacteria; Bacteroidetes; Bacteroidia; Bacteroidales; Bacteroidaceae; Bacteroides; Bacteroides caecigallinarum | Bacteroides caecigallinarum | Bacteroides caecigallinarum | Bacteroides | Bacteroidaceae | Bacteroidales | Bacteroidia | Bacteroidetes | Bacteria |
| ASV2095 | 422 | 2 | 1 | 422 | 100 | NR_113119.1 Prevotella oris strain JCM 8540 16S ribosomal RNA, partial sequence | NR_113119.1 | 1481 | 345 | 766 | 28.49 | 619.7 | 3E-177 | 394/423 | 93 | 2/423 | 0 | Plus | Plus | Bacteria; Bacteroidetes; Bacteroidia; Bacteroidales; Prevotellaceae; Prevotella; Prevotella oris | Prevotella oris | Prevotella oris | Prevotella | Prevotellaceae | Bacteroidales | Bacteroidia | Bacteroidetes | Bacteria |
| ASV2096 | 402 | 1 | 1 | 402 | 100 | NR_024661.1 Eubacterium ruminantium strain GA195 16S ribosomal RNA, partial sequence | NR_024661.1 | 1508 | 378 | 779 | 26.66 | 671.5 | 0 | 389/402 | 96 | 0/402 | 0 | Plus | Plus | Bacteria; Firmicutes; Clostridia; Eubacteriales; Eubacteriaceae; Eubacterium; Eubacterium ruminantium | Eubacterium ruminantium | Eubacterium ruminantium | Eubacterium | Eubacteriaceae | Eubacteriales | Clostridia | Firmicutes | Bacteria |
| ASV2097 | 428 | 1 | 1 | 411 | 96.03 | NR_112902.1 Phascolarctobacterium succinatutens strain YIT 12067 16S ribosomal RNA, partial sequence | NR_112902.1 | 1610 | 444 | 853 | 25.47 | 475.7 | 8E-134 | 365/416 | 87 | 11/416 | 2 | Plus | Plus | Bacteria; Firmicutes; Negativicutes; Acidaminococcales; Acidaminococcaceae; Phascolarctobacterium; Phascolarctobacterium succinatutens | Phascolarctobacterium succinatutens | Phascolarctobacterium succinatutens | Phascolarctobacterium | Acidaminococcaceae | Acidaminococcales | Negativicutes | Firmicutes | Bacteria |
| ASV2098 | 422 | 2 | 1 | 422 | 100 | NR_113105.1 Prevotella histicola strain JCM 15637 16S ribosomal RNA, partial sequence | NR_113105.1 | 1480 | 346 | 767 | 28.51 | 641.9 | 0 | 399/424 | 94 | 4/424 | 0 | Plus | Plus | Bacteria; Bacteroidetes; Bacteroidia; Bacteroidales; Prevotellaceae; Prevotella; Prevotella histicola | Prevotella histicola | Prevotella histicola | Prevotella | Prevotellaceae | Bacteroidales | Bacteroidia | Bacteroidetes | Bacteria |
| ASV2099 | 405 | 2 | 1 | 405 | 100 | NR_165727.1 Anaerotignum faecicola strain KGMB03357 16S ribosomal RNA, complete sequence | NR_165727.1 | 1535 | 375 | 780 | 26.45 | 605 | 9E-173 | 380/406 | 93 | 1/406 | 0 | Plus | Plus | Bacteria; Firmicutes; Clostridia; Eubacteriales; Lachnospiraceae; Anaerotignum; Anaerotignum faecicola | Anaerotignum faecicola | Anaerotignum faecicola | Anaerotignum | Lachnospiraceae | Eubacteriales | Clostridia | Firmicutes | Bacteria |
| ASV2100 | 403 | 1 | 3 | 403 | 99.5 | NR_125677.1 Vallitalea pronyensis strain FatNI3 16S ribosomal RNA, partial sequence | NR_125677.1 | 1480 | 365 | 764 | 27.03 | 457.2 | 3E-128 | 351/402 | 87 | 3/402 | 0 | Plus | Plus | Bacteria; Firmicutes; Clostridia; Eubacteriales; Vallitaleaceae; Vallitalea; Vallitalea pronyensis | Vallitalea pronyensis | Vallitalea pronyensis | Vallitalea | Vallitaleaceae | Eubacteriales | Clostridia | Firmicutes | Bacteria |
| ASV2101 | 422 | 1 | 1 | 422 | 100 | NR_113411.1 Prevotella copri DSM 18205 strain JCM 13464 16S ribosomal RNA, partial sequence | NR_113411.1 | 1493 | 358 | 779 | 28.27 | 758.2 | 0 | 418/422 | 99 | 0/422 | 0 | Plus | Plus | Bacteria; Bacteroidetes; Bacteroidia; Bacteroidales; Prevotellaceae; Prevotella; Prevotella copri | Prevotella copri | Prevotella copri | Prevotella | Prevotellaceae | Bacteroidales | Bacteroidia | Bacteroidetes | Bacteria |
| ASV2102 | 418 | 1 | 1 | 418 | 100 | NR_102893.1 Butyrivibrio proteoclasticus strain B316 16S ribosomal RNA, partial sequence | NR_102893.1 | 1537 | 367 | 782 | 27.07 | 571.7 | 1E-162 | 383/419 | 91 | 4/419 | 0 | Plus | Plus | Bacteria; Firmicutes; Clostridia; Eubacteriales; Lachnospiraceae; Butyrivibrio; Butyrivibrio proteoclasticus | Butyrivibrio proteoclasticus | Butyrivibrio proteoclasticus | Butyrivibrio | Lachnospiraceae | Eubacteriales | Clostridia | Firmicutes | Bacteria |
| ASV2103 | 405 | 1 | 1 | 405 | 100 | NR_118554.1 Intestinimonas butyriciproducens strain SRB-521-5-I 16S ribosomal RNA, partial sequence | NR_118554.1 | 1475 | 346 | 750 | 27.46 | 593.9 | 2E-169 | 377/405 | 93 | 0/405 | 0 | Plus | Plus | Bacteria; Firmicutes; Clostridia; Eubacteriales; ; Intestinimonas; Intestinimonas butyriciproducens | Intestinimonas butyriciproducens | Intestinimonas butyriciproducens | Intestinimonas | | Eubacteriales | Clostridia | Firmicutes | Bacteria |
| ASV2104 | 402 | 1 | 1 | 402 | 100 | NR_170495.1 Blautia faecicola strain KGMB01111 16S ribosomal RNA, complete sequence | NR_170495.1 | 1534 | 372 | 773 | 26.21 | 671.5 | 0 | 389/402 | 96 | 0/402 | 0 | Plus | Plus | Bacteria; Firmicutes; Clostridia; Eubacteriales; Lachnospiraceae; Blautia; Blautia faecicola | Blautia faecicola | Blautia faecicola | Blautia | Lachnospiraceae | Eubacteriales | Clostridia | Firmicutes | Bacteria |
| ASV2105 | 421 | 1 | 1 | 421 | 100 | NR_144616.1 Muribaculum intestinale strain YL27 16S ribosomal RNA, partial sequence | NR_144616.1 | 1460 | 297 | 718 | 28.9 | 490.5 | 3E-138 | 372/424 | 87 | 5/424 | 1 | Plus | Plus | Bacteria; Bacteroidetes; Bacteroidia; Bacteroidales; Muribaculaceae; Muribaculum; Muribaculum intestinale | Muribaculum intestinale | Muribaculum intestinale | Muribaculum | Muribaculaceae | Bacteroidales | Bacteroidia | Bacteroidetes | Bacteria |
| ASV2106 | 427 | 1 | 1 | 427 | 100 | NR_044111.1 Selenomonas bovis strain WG 16S ribosomal RNA, partial sequence | NR_044111.1 | 1525 | 370 | 796 | 28 | 695.5 | 0 | 410/427 | 96 | 0/427 | 0 | Plus | Plus | Bacteria; Firmicutes; Negativicutes; Selenomonadales; Selenomonadaceae; Selenomonas; Selenomonas bovis | Selenomonas bovis | Selenomonas bovis | Selenomonas | Selenomonadaceae | Selenomonadales | Negativicutes | Firmicutes | Bacteria |
| ASV2108 | 403 | 1 | 1 | 403 | 100 | NR_159296.1 Aminipila butyrica strain FH042 16S ribosomal RNA, partial sequence | NR_159296.1 | 1460 | 352 | 755 | 27.67 | 623.4 | 3E-178 | 382/404 | 94 | 1/404 | 0 | Plus | Plus | Bacteria; Firmicutes; Clostridia; Eubacteriales; Clostridiales Family XIII. Incertae Sedis; Aminipila; Aminipila butyrica | Aminipila butyrica | Aminipila butyrica | Aminipila | Clostridiales Family XIII. Incertae Sedis | Eubacteriales | Clostridia | Firmicutes | Bacteria |
| ASV2109 | 402 | 1 | 1 | 402 | 100 | NR_104783.1 Acetivibrio ethanolgignens strain 77-6 16S ribosomal RNA, partial sequence | NR_104783.1 | 1529 | 370 | 771 | 26.29 | 743.5 | 0 | 402/402 | 100 | 0/402 | 0 | Plus | Plus | Bacteria; Firmicutes; Clostridia; Eubacteriales; Oscillospiraceae; Acetivibrio; Acetivibrio ethanolgignens | Acetivibrio ethanolgignens | Acetivibrio ethanolgignens | Acetivibrio | Oscillospiraceae | Eubacteriales | Clostridia | Firmicutes | Bacteria |
| ASV2110 | 405 | 1 | 1 | 405 | 100 | NR_112900.1 Christensenella minuta strain YIT 12065 16S ribosomal RNA, partial sequence | NR_112900.1 | 1497 | 358 | 762 | 27.05 | 533 | 4E-151 | 369/408 | 90 | 6/408 | 1 | Plus | Plus | Bacteria; Firmicutes; Clostridia; Eubacteriales; Christensenellaceae; Christensenella; Christensenella minuta | Christensenella minuta | Christensenella minuta | Christensenella | Christensenellaceae | Eubacteriales | Clostridia | Firmicutes | Bacteria |
| ASV2111 | 403 | 1 | 1 | 403 | 100 | NR_102987.1 Hungateiclostridium clariflavum DSM 19732 16S ribosomal RNA, partial sequence | NR_102987.1 | 1634 | 481 | 882 | 24.6 | 616.1 | 4E-176 | 380/403 | 94 | 1/403 | 0 | Plus | Plus | Bacteria; Firmicutes; Clostridia; Eubacteriales; Oscillospiraceae; Acetivibrio; Acetivibrio clariflavus | Acetivibrio clariflavus | Acetivibrio clariflavus | Acetivibrio | Oscillospiraceae | Eubacteriales | Clostridia | Firmicutes | Bacteria |
| ASV2112 | 422 | 1 | 1 | 422 | 100 | NR_146018.1 Paludibacter jiangxiensis strain NM7 16S ribosomal RNA, partial sequence | NR_146018.1 | 1469 | 340 | 761 | 28.73 | 492.3 | 8E-139 | 375/427 | 87 | 10/427 | 2 | Plus | Plus | Bacteria; Bacteroidetes; Bacteroidia; Bacteroidales; Paludibacteraceae; Paludibacter; Paludibacter jiangxiensis | Paludibacter jiangxiensis | Paludibacter jiangxiensis | Paludibacter | Paludibacteraceae | Bacteroidales | Bacteroidia | Bacteroidetes | Bacteria |
| ASV2113 | 422 | 3 | 1 | 422 | 100 | NR_113094.1 Prevotella baroniae strain JCM 13447 16S ribosomal RNA, partial sequence | NR_113094.1 | 1494 | 358 | 779 | 28.25 | 564.3 | 2E-160 | 384/423 | 90 | 2/423 | 0 | Plus | Plus | Bacteria; Bacteroidetes; Bacteroidia; Bacteroidales; Prevotellaceae; Prevotella; Prevotella baroniae | Prevotella baroniae | Prevotella baroniae | Prevotella | Prevotellaceae | Bacteroidales | Bacteroidia | Bacteroidetes | Bacteria |
| ASV2114 | 402 | 1 | 3 | 402 | 99.5 | NR_026476.1 Succinivibrio dextrinosolvens strain 0554 16S ribosomal RNA, partial sequence | NR_026476.1 | 1463 | 333 | 732 | 27.34 | 545.9 | 6E-155 | 366/401 | 91 | 2/401 | 0 | Plus | Plus | Bacteria; Proteobacteria; Gammaproteobacteria; Aeromonadales; Succinivibrionaceae; Succinivibrio; Succinivibrio dextrinosolvens | Succinivibrio dextrinosolvens | Succinivibrio dextrinosolvens | Succinivibrio | Succinivibrionaceae | Aeromonadales | Gammaproteobacteria | Proteobacteria | Bacteria |
| ASV2115 | 428 | 1 | 1 | 428 | 100 | NR_104781.1 Treponema bryantii strain RUS-1 16S ribosomal RNA, partial sequence | NR_104781.1 | 1535 | 351 | 778 | 27.88 | 531.1 | 2E-150 | 385/432 | 89 | 8/432 | 1 | Plus | Plus | Bacteria; Spirochaetes; Spirochaetia; Spirochaetales; Spirochaetaceae; Treponema; Treponema bryantii | Treponema bryantii | Treponema bryantii | Treponema | Spirochaetaceae | Spirochaetales | Spirochaetia | Spirochaetes | Bacteria |
| ASV2116 | 405 | 1 | 1 | 405 | 100 | NR_118554.1 Intestinimonas butyriciproducens strain SRB-521-5-I 16S ribosomal RNA, partial sequence | NR_118554.1 | 1475 | 346 | 750 | 27.46 | 588.4 | 9E-168 | 376/405 | 92 | 0/405 | 0 | Plus | Plus | Bacteria; Firmicutes; Clostridia; Eubacteriales; ; Intestinimonas; Intestinimonas butyriciproducens | Intestinimonas butyriciproducens | Intestinimonas butyriciproducens | Intestinimonas | | Eubacteriales | Clostridia | Firmicutes | Bacteria |
| ASV2117 | 403 | 1 | 1 | 403 | 100 | NR_029160.1 Ruminococcus callidus strain ATCC 27760 16S ribosomal RNA, partial sequence | NR_029160.1 | 1371 | 310 | 711 | 29.32 | 627.1 | 2E-179 | 384/405 | 94 | 5/405 | 1 | Plus | Plus | Bacteria; Firmicutes; Clostridia; Eubacteriales; Oscillospiraceae; Ruminococcus; Ruminococcus callidus | Ruminococcus callidus | Ruminococcus callidus | Ruminococcus | Oscillospiraceae | Eubacteriales | Clostridia | Firmicutes | Bacteria |
| ASV2118 | 419 | 1 | 1 | 419 | 100 | NR_037116.1 Bifidobacterium choerinum strain Su 806 16S ribosomal RNA, partial sequence | NR_037116.1 | 1530 | 346 | 764 | 27.39 | 763.8 | 0 | 417/419 | 99 | 0/419 | 0 | Plus | Plus | Bacteria; Actinobacteria; Actinomycetia; Bifidobacteriales; Bifidobacteriaceae; Bifidobacterium; Bifidobacterium choerinum | Bifidobacterium choerinum | Bifidobacterium choerinum | Bifidobacterium | Bifidobacteriaceae | Bifidobacteriales | Actinomycetia | Actinobacteria | Bacteria |
| ASV2119 | 427 | 5 | 4 | 427 | 99.3 | NR_146813.2 Erysipelothrix larvae strain LV19 16S ribosomal RNA, complete sequence | NR_146813.2 | 1543 | 370 | 792 | 27.41 | 427.7 | 2E-119 | 364/428 | 85 | 9/428 | 2 | Plus | Plus | Bacteria; Firmicutes; Erysipelotrichia; Erysipelotrichales; Erysipelotrichaceae; Erysipelothrix; Erysipelothrix larvae | Erysipelothrix larvae | Erysipelothrix larvae | Erysipelothrix | Erysipelotrichaceae | Erysipelotrichales | Erysipelotrichia | Firmicutes | Bacteria |
| ASV2120 | 421 | 2 | 1 | 421 | 100 | NR_113073.1 Barnesiella intestinihominis strain JCM 15079 16S ribosomal RNA, partial sequence | NR_113073.1 | 1481 | 346 | 767 | 28.49 | 523.7 | 3E-148 | 378/424 | 89 | 5/424 | 1 | Plus | Plus | Bacteria; Bacteroidetes; Bacteroidia; Bacteroidales; Barnesiellaceae; Barnesiella; Barnesiella intestinihominis | Barnesiella intestinihominis | Barnesiella intestinihominis | Barnesiella | Barnesiellaceae | Bacteroidales | Bacteroidia | Bacteroidetes | Bacteria |
| ASV2121 | 421 | 1 | 1 | 421 | 100 | NR_144616.1 Muribaculum intestinale strain YL27 16S ribosomal RNA, partial sequence | NR_144616.1 | 1460 | 297 | 718 | 28.9 | 468.3 | 1E-131 | 367/423 | 86 | 3/423 | 0 | Plus | Plus | Bacteria; Bacteroidetes; Bacteroidia; Bacteroidales; Muribaculaceae; Muribaculum; Muribaculum intestinale | Muribaculum intestinale | Muribaculum intestinale | Muribaculum | Muribaculaceae | Bacteroidales | Bacteroidia | Bacteroidetes | Bacteria |
| [illegible] | [illegible] | [illegible] | [illegible] | [illegible] | [illegible] | [illegible] | [illegible] | [illegible] | [illegible] | [illegible] | [illegible] | [illegible] | [illegible] | [illegible] | [illegible] | [illegible] | [illegible] | [illegible] | [illegible] | [illegible] | [illegible] | [illegible] | [illegible] | [illegible] | [illegible] | [illegible] | [illegible] | [illegible] |

| | | | | | | | | | | | | | | | | | | | | | | | | | | | | |
|---|---|---|---|---|---|---|---|---|---|---|---|---|---|---|---|---|---|---|---|---|---|---|---|---|---|---|---|---|
| ASV2353 | 404 | 3 | 1 | 404 | 100 | NR_074793.2 Oscillibacter valericigenes strain Sjm18-20 16S ribosomal RNA, complete sequence | NR_074793.2 | 1530 | 372 | 775 | 26.41 | 636.4 | 0 | 384/404 | 95 | 0/404 | 0 | Plus | Plus | Bacteria; Firmicutes; Clostridia; Eubacteriales; Oscillospiraceae; Oscillibacter; Oscillibacter valericigenes | Oscillibacter valericigenes | Oscillibacter | Oscillospiraceae | Eubacteriales | Clostridia | Firmicutes | Bacteria | Bacteria |
| ASV2354 | 422 | 3 | 1 | 422 | 100 | NR_119164.1 Bacteroides fragilis strain ATCC 25285 16S ribosomal RNA, partial sequence | NR_119164.1 | 1436 | 334 | 755 | 29.39 | 780.4 | 0 | 422/422 | 100 | 0/422 | 0 | Plus | Plus | Bacteria; Bacteroidetes; Bacteroidia; Bacteroidales; Bacteroidaceae; Bacteroides; Bacteroides fragilis | Bacteroides fragilis | Bacteroides | Bacteroidaceae | Bacteroidales | Bacteroidia | Bacteroidetes | Bacteria | Bacteria |
| ASV2355 | 427 | 1 | 1 | 427 | 100 | NR_026332.1 Shigella dysenteriae strain ATCC 13313 16S ribosomal RNA, partial sequence | NR_026332.1 | 1487 | 338 | 764 | 28.72 | 761.9 | 0 | 422/427 | 98 | 0/427 | 0 | Plus | Plus | Bacteria; Proteobacteria; Gammaproteobacteria; Enterobacterales; Enterobacteriaceae; Shigella; Shigella dysenteriae | Shigella dysenteriae | Shigella | Enterobacteriaceae | Enterobacterales | Gammaproteobacteria | Proteobacteria | Bacteria | Bacteria |
| ASV2356 | 427 | 2 | 1 | 427 | 100 | NR_037018.1 Acidaminococcus fermentans strain VR4 16S ribosomal RNA gene, partial sequence | NR_037018.1 | 1516 | 349 | 775 | 28.17 | 734.2 | 0 | 417/427 | 97 | 0/427 | 0 | Plus | Plus | Bacteria; Firmicutes; Negativicutes; Acidaminococcales; Acidaminococcaceae; Acidaminococcus; Acidaminococcus fermentans | Acidaminococcus fermentans | Acidaminococcus | Acidaminococcaceae | Acidaminococcales | Negativicutes | Firmicutes | Bacteria | Bacteria |
| ASV2357 | 405 | 1 | 1 | 405 | 100 | NR_118554.1 Intestinimonas butyriciproducens strain SRB-521-5-I 16S ribosomal RNA, partial sequence | NR_118554.1 | 1475 | 346 | 750 | 27.46 | 671.5 | 0 | 391/405 | 96 | 0/405 | 0 | Plus | Plus | Bacteria; Firmicutes; Clostridia; Eubacteriales; ; Intestinimonas; Intestinimonas butyriciproducens | Intestinimonas butyriciproducens | Intestinimonas | | Eubacteriales | Clostridia | Firmicutes | Bacteria | Bacteria |
| ASV2358 | 403 | 1 | 3 | 403 | 99.5 | NR_125464.1 Anaerobacterium chartisolvens strain T-1-35 16S ribosomal RNA, partial sequence | NR_125464.1 | 1451 | 344 | 745 | 27.71 | 475.7 | 7E-134 | 354/402 | 88 | 1/402 | 0 | Plus | Plus | Bacteria; Firmicutes; Clostridia; Eubacteriales; Oscillospiraceae; Anaerobacterium; Anaerobacterium chartisolvens | Anaerobacterium chartisolvens | Anaerobacterium | Oscillospiraceae | Eubacteriales | Clostridia | Firmicutes | Bacteria | Bacteria |
| ASV2359 | 420 | 1 | 1 | 420 | 100 | NR_144616.1 Muribaculum intestinale strain YL27 16S ribosomal RNA, partial sequence | NR_144616.1 | 1460 | 297 | 718 | 28.9 | 545.9 | 6E-155 | 384/426 | 90 | 10/426 | 2 | Plus | Plus | Bacteria; Bacteroidetes; Bacteroidia; Bacteroidales; Muribaculaceae; Muribaculum; Muribaculum intestinale | Muribaculum intestinale | Muribaculum | Muribaculaceae | Bacteroidales | Bacteroidia | Bacteroidetes | Bacteria | Bacteria |
| ASV2360 | 422 | 1 | 1 | 422 | 100 | NR_113411.1 Prevotella copri DSM 18205 strain JCM 13464 16S ribosomal RNA, partial sequence | NR_113411.1 | 1493 | 358 | 779 | 28.27 | 736.1 | 0 | 414/422 | 98 | 0/422 | 0 | Plus | Plus | Bacteria; Bacteroidetes; Bacteroidia; Bacteroidales; Prevotellaceae; Prevotella; Prevotella copri | Prevotella copri | Prevotella | Prevotellaceae | Bacteroidales | Bacteroidia | Bacteroidetes | Bacteria | Bacteria |
| ASV2361 | 422 | 1 | 1 | 422 | 100 | NR_156844.1 Ruficoccus amylovorans strain CC-MHH0563 16S ribosomal RNA, partial sequence | NR_156844.1 | 1471 | 344 | 763 | 28.55 | 507.1 | 3E-143 | 374/423 | 88 | 4/423 | 0 | Plus | Plus | Bacteria; Verrucomicrobia; Opitutae; Puniceicoccales; Puniceicoccaceae; Ruficoccus; Ruficoccus amylovorans | Ruficoccus amylovorans | Ruficoccus | Puniceicoccaceae | Puniceicoccales | Opitutae | Verrucomicrobia | Bacteria | Bacteria |
| ASV2362 | 426 | 1 | 1 | 425 | 99.77 | NR_114696.1 Anaerovibrio lipolyticus DSM 3074 16S ribosomal RNA, partial sequence | NR_114696.1 | 1526 | 372 | 796 | 27.85 | 634.5 | 0 | 397/425 | 93 | 0/425 | 0 | Plus | Plus | Bacteria; Firmicutes; Negativicutes; Selenomonadales; Selenomonadaceae; Anaerovibrio; Anaerovibrio lipolyticus | Anaerovibrio lipolyticus | Anaerovibrio | Selenomonadaceae | Selenomonadales | Negativicutes | Firmicutes | Bacteria | Bacteria |
| ASV2363 | 402 | 1 | 1 | 402 | 100 | NR_102884.1 Ruminococcus champanellensis 18P13 = JCM 17042 16S ribosomal RNA, partial sequence | NR_102884.1 | 1494 | 348 | 749 | 26.91 | 593.9 | 2E-169 | 375/402 | 93 | 0/402 | 0 | Plus | Plus | Bacteria; Firmicutes; Clostridia; Eubacteriales; Oscillospiraceae; Ruminococcus; Ruminococcus champanellensis | Ruminococcus champanellensis | Ruminococcus | Oscillospiraceae | Eubacteriales | Clostridia | Firmicutes | Bacteria | Bacteria |
| ASV2364 | 422 | 3 | 1 | 422 | 100 | NR_113109.1 Prevotella loescheii strain JCM 8530 16S ribosomal RNA, partial sequence | NR_113109.1 | 1476 | 342 | 763 | 28.59 | 597.6 | 2E-170 | 391/424 | 92 | 4/424 | 0 | Plus | Plus | Bacteria; Bacteroidetes; Bacteroidia; Bacteroidales; Prevotellaceae; Prevotella; Prevotella loescheii | Prevotella loescheii | Prevotella | Prevotellaceae | Bacteroidales | Bacteroidia | Bacteroidetes | Bacteria | Bacteria |
| ASV2365 | 403 | 1 | 3 | 403 | 99.5 | NR_125464.1 Anaerobacterium chartisolvens strain T-1-35 16S ribosomal RNA, partial sequence | NR_125464.1 | 1451 | 344 | 745 | 27.71 | 475.7 | 7E-134 | 354/402 | 88 | 1/402 | 0 | Plus | Plus | Bacteria; Firmicutes; Clostridia; Eubacteriales; Oscillospiraceae; Anaerobacterium; Anaerobacterium chartisolvens | Anaerobacterium chartisolvens | Anaerobacterium | Oscillospiraceae | Eubacteriales | Clostridia | Firmicutes | Bacteria | Bacteria |
| ASV2366 | 427 | 1 | 1 | 427 | 100 | NR_147405.1 Mailhella massiliensis strain Marseille-P3199 16S ribosomal RNA, partial sequence | NR_147405.1 | 1511 | 355 | 781 | 28.26 | 557 | 3E-158 | 387/429 | 90 | 4/429 | 0 | Plus | Plus | Bacteria; Proteobacteria; Deltaproteobacteria; Desulfovibrionales; Desulfovibrionaceae; Mailhella; Mailhella massiliensis | Mailhella massiliensis | Mailhella | Desulfovibrionaceae | Desulfovibrionales | Deltaproteobacteria | Proteobacteria | Bacteria | Bacteria |
| ASV2367 | 422 | 1 | 1 | 422 | 100 | NR_113096.1 Prevotella bivia strain JCM 6331 16S ribosomal RNA, partial sequence | NR_113096.1 | 1490 | 356 | 777 | 28.32 | 597.6 | 2E-170 | 390/423 | 92 | 2/423 | 0 | Plus | Plus | Bacteria; Bacteroidetes; Bacteroidia; Bacteroidales; Prevotellaceae; Prevotella; Prevotella bivia | Prevotella bivia | Prevotella | Prevotellaceae | Bacteroidales | Bacteroidia | Bacteroidetes | Bacteria | Bacteria |
| ASV2368 | 402 | 4 | 1 | 402 | 100 | NR_118730.1 [Clostridium] symbiosum strain ATCC 14940 16S ribosomal RNA, partial sequence | NR_118730.1 | 1478 | 372 | 773 | 27.2 | 677 | 0 | 388/402 | 96 | 0/402 | 0 | Plus | Plus | Bacteria; Firmicutes; Clostridia; Eubacteriales; Lachnospiraceae; Lachnoclostridium; [Clostridium] symbiosum | [Clostridium] symbiosum | Lachnoclostridium | Lachnospiraceae | Eubacteriales | Clostridia | Firmicutes | Bacteria | Bacteria |
| ASV2369 | 402 | 3 | 1 | 402 | 100 | NR_156081.1 Kineothrix alysoides strain KNHs209 16S ribosomal RNA, partial sequence | NR_156081.1 | 1411 | 353 | 754 | 28.49 | 693.6 | 0 | 394/403 | 97 | 2/403 | 0 | Plus | Plus | Bacteria; Firmicutes; Clostridia; Eubacteriales; Lachnospiraceae; Kineothrix; Kineothrix alysoides | Kineothrix alysoides | Kineothrix | Lachnospiraceae | Eubacteriales | Clostridia | Firmicutes | Bacteria | Bacteria |
| ASV2370 | 402 | 5 | 1 | 402 | 100 | NR_113231.1 Blautia glucerasea strain JCM 17039 16S ribosomal RNA, partial sequence | NR_113231.1 | 1491 | 364 | 765 | 26.96 | 571.7 | 9E-163 | 373/404 | 92 | 4/404 | 0 | Plus | Plus | Bacteria; Firmicutes; Clostridia; Eubacteriales; Lachnospiraceae; Blautia; Blautia glucerasea | Blautia glucerasea | Blautia | Lachnospiraceae | Eubacteriales | Clostridia | Firmicutes | Bacteria | Bacteria |
| ASV2371 | 422 | 1 | 1 | 422 | 100 | NR_144616.1 Muribaculum intestinale strain YL27 16S ribosomal RNA, partial sequence | NR_144616.1 | 1460 | 297 | 718 | 28.9 | 597.6 | 2E-170 | 390/423 | 92 | 2/423 | 0 | Plus | Plus | Bacteria; Bacteroidetes; Bacteroidia; Bacteroidales; Muribaculaceae; Muribaculum; Muribaculum intestinale | Muribaculum intestinale | Muribaculum | Muribaculaceae | Bacteroidales | Bacteroidia | Bacteroidetes | Bacteria | Bacteria |
| ASV2372 | 401 | 1 | 1 | 401 | 100 | NR_159227.1 Monoglobus pectinilyticus strain 14 16S ribosomal RNA, partial sequence | NR_159227.1 | 1493 | 368 | 769 | 26.93 | 525.6 | 7E-149 | 364/403 | 90 | 3/403 | 0 | Plus | Plus | Bacteria; Firmicutes; Clostridia; Eubacteriales; Oscillospiraceae; Monoglobus; Monoglobus pectinilyticus | Monoglobus pectinilyticus | Monoglobus | Oscillospiraceae | Eubacteriales | Clostridia | Firmicutes | Bacteria | Bacteria |
| ASV2373 | 403 | 1 | 3 | 403 | 99.5 | NR_125677.1 Vallitalea pronyensis strain FatNI3 16S ribosomal RNA, partial sequence | NR_125677.1 | 1480 | 365 | 764 | 27.03 | 479.4 | 6E-135 | 357/404 | 88 | 7/404 | 1 | Plus | Plus | Bacteria; Firmicutes; Clostridia; Eubacteriales; Vallitaleaceae; Vallitalea; Vallitalea pronyensis | Vallitalea pronyensis | Vallitalea | Vallitaleaceae | Eubacteriales | Clostridia | Firmicutes | Bacteria | Bacteria |
| ASV2375 | 402 | 1 | 1 | 402 | 100 | NR_029160.1 Ruminococcus callidus strain ATCC 27760 16S ribosomal RNA, partial sequence | NR_029160.1 | 1371 | 310 | 711 | 29.32 | 671.5 | 0 | 389/402 | 96 | 0/402 | 0 | Plus | Plus | Bacteria; Firmicutes; Clostridia; Eubacteriales; Oscillospiraceae; Ruminococcus; Ruminococcus callidus | Ruminococcus callidus | Ruminococcus | Oscillospiraceae | Eubacteriales | Clostridia | Firmicutes | Bacteria | Bacteria |
| ASV2376 | 422 | 1 | 1 | 422 | 100 | NR_041364.1 Prevotella stercorea DSM 18206 strain CB35 16S ribosomal RNA, partial sequence | NR_041364.1 | 1488 | 354 | 775 | 28.36 | 713.9 | 0 | 410/422 | 97 | 0/422 | 0 | Plus | Plus | Bacteria; Bacteroidetes; Bacteroidia; Bacteroidales; Prevotellaceae; Prevotella; Prevotella stercorea | Prevotella stercorea | Prevotella | Prevotellaceae | Bacteroidales | Bacteroidia | Bacteroidetes | Bacteria | Bacteria |
| [illegible] | [illegible] | [illegible] | [illegible] | [illegible] | [illegible] | [illegible] | [illegible] | [illegible] | [illegible] | [illegible] | [illegible] | [illegible] | [illegible] | [illegible] | [illegible] | [illegible] | [illegible] | [illegible] | [illegible] | [illegible] | [illegible] | [illegible] | [illegible] | [illegible] | [illegible] | [illegible] | [illegible] | [illegible] |

| | | | | | | | | | | | | | | | | | | | | | | | | | | | |
|---|---|---|---|---|---|---|---|---|---|---|---|---|---|---|---|---|---|---|---|---|---|---|---|---|---|---|---|
| ASV2627 | 427 | 5 | 4 | 427 | 99.3 | NR_025594.1 Erysipelothrix inopinata strain 143-02 16S ribosomal RNA, partial sequence | NR_025594.1 | 1504 | 349 | 771 | 28.13 | 433.2 | 5E-121 | 362/425 | 85 | 3/425 | 0 | Plus | Plus | Bacteria; Firmicutes; Erysipelotrichia; Erysipelotrichales; Erysipelotrichaceae; Erysipelothrix; Erysipelothrix inopinata | Erysipelothrix inopinata | Erysipelothrix | Erysipelotrichaceae | Erysipelotrichales | Erysipelotrichia | Firmicutes | Bacteria |
| ASV2629 | 427 | 1 | 1 | 426 | 99.77 | NR_028840.1 Mitsuokella jalaludinii strain M9 16S ribosomal RNA, partial sequence | NR_028840.1 | 1543 | 362 | 787 | 27.61 | 682.5 | 0 | 407/426 | 95 | 0/426 | 0 | Plus | Plus | Bacteria; Firmicutes; Negativicutes; Selenomonadales; Selenomonadaceae; Mitsuokella; Mitsuokella jalaludinii | Mitsuokella jalaludinii | Mitsuokella | Selenomonadaceae | Selenomonadales | Negativicutes | Firmicutes | Bacteria |
| ASV2630 | 422 | 1 | 1 | 422 | 100 | NR_144750.1 Prevotellamassilia timonensis strain Marseille-P2831 16S ribosomal RNA, partial sequence | NR_144750.1 | 1493 | 358 | 779 | 28.27 | 658.5 | 0 | 401/423 | 94 | 2/423 | 0 | Plus | Plus | Bacteria; Bacteroidetes; Bacteroidia; Bacteroidales; Prevotellaceae; Prevotellamassilia; Prevotellamassilia timonensis | Prevotellamassilia timonensis | Prevotellamassilia | Prevotellaceae | Bacteroidales | Bacteroidia | Bacteroidetes | Bacteria |
| ASV2631 | 405 | 2 | 1 | 405 | 100 | NR_144696.1 Clostridium saudiense strain JCC 16S ribosomal RNA, partial sequence | NR_144696.1 | 1478 | 350 | 751 | 27.2 | 584.7 | 1E-166 | 376/405 | 92 | 3/405 | 0 | Plus | Plus | Bacteria; Firmicutes; Clostridia; Eubacteriales; Clostridiaceae; Clostridium; Clostridium saudiense | Clostridium saudiense | Clostridium | Clostridiaceae | Eubacteriales | Clostridia | Firmicutes | Bacteria |
| ASV2632 | 427 | 2 | 1 | 426 | 99.77 | NR_117778.1 Sutterella wadsworthensis strain WAL 9799 16S ribosomal RNA, partial sequence | NR_117778.1 | 1462 | 310 | 735 | 29.14 | 605 | 1E-172 | 396/429 | 92 | 6/429 | 1 | Plus | Plus | Bacteria; Proteobacteria; Betaproteobacteria; Burkholderiales; Sutterellaceae; Sutterella; Sutterella wadsworthensis | Sutterella wadsworthensis | Sutterella | Sutterellaceae | Burkholderiales | Betaproteobacteria | Proteobacteria | Bacteria |
| ASV2633 | 427 | 3 | 1 | 427 | 100 | NR_036918.1 Streptococcus suis strain S735 16S ribosomal RNA, partial sequence | NR_036918.1 | 1535 | 354 | 780 | 27.82 | 784.1 | 0 | 426/427 | 99 | 0/427 | 0 | Plus | Plus | Bacteria; Firmicutes; Bacilli; Lactobacillales; Streptococcaceae; Streptococcus; Streptococcus suis | Streptococcus suis | Streptococcus | Streptococcaceae | Lactobacillales | Bacilli | Firmicutes | Bacteria |
| ASV2634 | 422 | 1 | 1 | 422 | 100 | NR_113411.1 Prevotella copri DSM 18205 strain JCM 13464 16S ribosomal RNA, partial sequence | NR_113411.1 | 1493 | 358 | 779 | 28.27 | 763.8 | 0 | 419/422 | 99 | 0/422 | 0 | Plus | Plus | Bacteria; Bacteroidetes; Bacteroidia; Bacteroidales; Prevotellaceae; Prevotella; Prevotella copri | Prevotella copri | Prevotella | Prevotellaceae | Bacteroidales | Bacteroidia | Bacteroidetes | Bacteria |
| ASV2635 | 422 | 1 | 1 | 422 | 100 | NR_041285.1 Prevotella multisaccharivorax strain PPPA20 16S ribosomal RNA, partial sequence | NR_041285.1 | 1491 | 356 | 777 | 28.3 | 641.9 | 0 | 397/422 | 94 | 0/422 | 0 | Plus | Plus | Bacteria; Bacteroidetes; Bacteroidia; Bacteroidales; Prevotellaceae; Prevotella; Prevotella multisaccharivorax | Prevotella multisaccharivorax | Prevotella | Prevotellaceae | Bacteroidales | Bacteroidia | Bacteroidetes | Bacteria |
| ASV2636 | 405 | 2 | 1 | 405 | 100 | NR_156080.1 Kineothrix alysoides strain KNHs209 16S ribosomal RNA, partial sequence | NR_156080.1 | 1410 | 353 | 754 | 28.51 | 701 | 0 | 397/405 | 98 | 3/405 | 0 | Plus | Plus | Bacteria; Firmicutes; Clostridia; Eubacteriales; Lachnospiraceae; Kineothrix; Kineothrix alysoides | Kineothrix alysoides | Kineothrix | Lachnospiraceae | Eubacteriales | Clostridia | Firmicutes | Bacteria |
| ASV2637 | 427 | 1 | 1 | 427 | 100 | NR_112902.1 Phascolarctobacterium succinatutens strain YIT 12067 16S ribosomal RNA, partial sequence | NR_112902.1 | 1610 | 444 | 870 | 26.52 | 756.4 | 0 | 421/427 | 98 | 0/427 | 0 | Plus | Plus | Bacteria; Firmicutes; Negativicutes; Acidaminococcales; Acidaminococcaceae; Phascolarctobacterium; Phascolarctobacterium succinatutens | Phascolarctobacterium succinatutens | Phascolarctobacterium | Acidaminococcaceae | Acidaminococcales | Negativicutes | Firmicutes | Bacteria |
| ASV2638 | 422 | 1 | 1 | 422 | 100 | NR_113411.1 Prevotella copri DSM 18205 strain JCM 13464 16S ribosomal RNA, partial sequence | NR_113411.1 | 1493 | 358 | 779 | 28.27 | 608.7 | 7E-174 | 391/422 | 92 | 0/422 | 0 | Plus | Plus | Bacteria; Bacteroidetes; Bacteroidia; Bacteroidales; Prevotellaceae; Prevotella; Prevotella copri | Prevotella copri | Prevotella | Prevotellaceae | Bacteroidales | Bacteroidia | Bacteroidetes | Bacteria |
| ASV2639 | 405 | 2 | 1 | 405 | 100 | NR_156080.1 Kineothrix alysoides strain KNHs209 16S ribosomal RNA, partial sequence | NR_156080.1 | 1410 | 353 | 754 | 28.51 | 712.1 | 0 | 399/405 | 98 | 3/405 | 0 | Plus | Plus | Bacteria; Firmicutes; Clostridia; Eubacteriales; Lachnospiraceae; Kineothrix; Kineothrix alysoides | Kineothrix alysoides | Kineothrix | Lachnospiraceae | Eubacteriales | Clostridia | Firmicutes | Bacteria |
| ASV2640 | 405 | 1 | 1 | 405 | 100 | NR_159227.1 Monoglobus pectinilyticus strain 14 16S ribosomal RNA, partial sequence | NR_159227.1 | 1493 | 368 | 769 | 26.93 | 518.2 | 1E-146 | 364/405 | 89 | 3/405 | 0 | Plus | Plus | Bacteria; Firmicutes; Clostridia; Eubacteriales; Oscillospiraceae; Monoglobus; Monoglobus pectinilyticus | Monoglobus pectinilyticus | Monoglobus | Oscillospiraceae | Eubacteriales | Clostridia | Firmicutes | Bacteria |
| ASV2641 | 403 | 3 | 1 | 403 | 100 | NR_114315.1 Blautia luti DSM 14534 16S ribosomal RNA, partial sequence | NR_114315.1 | 1481 | 353 | 754 | 27.14 | 682.5 | 0 | 393/404 | 97 | 3/404 | 0 | Plus | Plus | Bacteria; Firmicutes; Clostridia; Eubacteriales; Lachnospiraceae; Blautia; Blautia luti | Blautia luti | Blautia | Lachnospiraceae | Eubacteriales | Clostridia | Firmicutes | Bacteria |
| ASV2642 | 422 | 1 | 1 | 422 | 100 | NR_144750.1 Prevotellamassilia timonensis strain Marseille-P2831 16S ribosomal RNA, partial sequence | NR_144750.1 | 1493 | 358 | 779 | 28.27 | 542.2 | 8E-154 | 379/422 | 89 | 0/422 | 0 | Plus | Plus | Bacteria; Bacteroidetes; Bacteroidia; Bacteroidales; Prevotellaceae; Prevotellamassilia; Prevotellamassilia timonensis | Prevotellamassilia timonensis | Prevotellamassilia | Prevotellaceae | Bacteroidales | Bacteroidia | Bacteroidetes | Bacteria |
| ASV2643 | 421 | 1 | 1 | 421 | 100 | NR_144616.1 Muribaculum intestinale strain YL27 16S ribosomal RNA, partial sequence | NR_144616.1 | 1460 | 297 | 718 | 28.9 | 507.1 | 3E-143 | 374/423 | 88 | 3/423 | 0 | Plus | Plus | Bacteria; Bacteroidetes; Bacteroidia; Bacteroidales; Muribaculaceae; Muribaculum; Muribaculum intestinale | Muribaculum intestinale | Muribaculum | Muribaculaceae | Bacteroidales | Bacteroidia | Bacteroidetes | Bacteria |
| ASV2645 | 404 | 2 | 1 | 404 | 100 | NR_159296.1 Aminipila butyrica strain FH042 16S ribosomal RNA, partial sequence | NR_159296.1 | 1460 | 352 | 755 | 27.67 | 614.2 | 2E-175 | 380/404 | 94 | 0/404 | 0 | Plus | Plus | Bacteria; Firmicutes; Clostridia; Eubacteriales; Clostridiales Family XIII. Incertae Sedis; Aminipila; Aminipila butyrica | Aminipila butyrica | Aminipila | Clostridiales Family XIII. Incertae Sedis | Eubacteriales | Clostridia | Firmicutes | Bacteria |
| ASV2646 | 422 | 1 | 1 | 422 | 100 | NR_041364.1 Prevotella stercorea DSM 18206 strain CB35 16S ribosomal RNA, partial sequence | NR_041364.1 | 1488 | 354 | 775 | 28.36 | 558.8 | 8E-159 | 383/423 | 90 | 2/423 | 0 | Plus | Plus | Bacteria; Bacteroidetes; Bacteroidia; Bacteroidales; Prevotellaceae; Prevotella; Prevotella stercorea | Prevotella stercorea | Prevotella | Prevotellaceae | Bacteroidales | Bacteroidia | Bacteroidetes | Bacteria |
| ASV2647 | 405 | 1 | 1 | 405 | 100 | NR_044616.1 Synergistes jonesii strain 78-1 16S ribosomal RNA, partial sequence | NR_044616.1 | 1355 | 345 | 749 | 29.89 | 721.3 | 0 | 399/405 | 98 | 0/405 | 0 | Plus | Plus | Bacteria; Synergistetes; Synergistia; Synergistales; Synergistaceae; Synergistes; Synergistes jonesii | Synergistes jonesii | Synergistes | Synergistaceae | Synergistales | Synergistia | Synergistetes | Bacteria |
| ASV2648 | 402 | 5 | 1 | 402 | 100 | NR_147395.1 Blautia marasmi strain Marseille-P2377 16S ribosomal RNA, partial sequence | NR_147395.1 | 1496 | 365 | 766 | 26.87 | 693.6 | 0 | 394/403 | 97 | 2/403 | 0 | Plus | Plus | Bacteria; Firmicutes; Clostridia; Eubacteriales; Lachnospiraceae; Blautia; Blautia marasmi | Blautia marasmi | Blautia | Lachnospiraceae | Eubacteriales | Clostridia | Firmicutes | Bacteria |
| ASV2649 | 404 | 1 | 1 | 404 | 100 | NR_145666.1 Rarimicrobium hominis strain ADV70 16S ribosomal RNA, partial sequence | NR_145666.1 | 1392 | 298 | 701 | 29.02 | 547.7 | 2E-155 | 374/410 | 91 | 12/410 | 2 | Plus | Plus | Bacteria; Synergistetes; Synergistia; Synergistales; Synergistaceae; Rarimicrobium; Rarimicrobium hominis | Rarimicrobium hominis | Rarimicrobium | Synergistaceae | Synergistales | Synergistia | Synergistetes | Bacteria |
| ASV2650 | 421 | 2 | 1 | 421 | 100 | NR_113073.1 Barnesiella intestinihominis strain JCM 15079 16S ribosomal RNA, partial sequence | NR_113073.1 | 1481 | 346 | 767 | 28.49 | 523.7 | 3E-148 | 378/424 | 89 | 5/424 | 1 | Plus | Plus | Bacteria; Bacteroidetes; Bacteroidia; Bacteroidales; Barnesiellaceae; Barnesiella; Barnesiella intestinihominis | Barnesiella intestinihominis | Barnesiella | Barnesiellaceae | Bacteroidales | Bacteroidia | Bacteroidetes | Bacteria |
| ASV2651 | 428 | 3 | 1 | 428 | 100 | NR_026413.1 Desulfovibrio intestinalis strain KMS2 16S ribosomal RNA, partial sequence | NR_026413.1 | 1509 | 337 | 763 | 28.3 | 612.4 | 6E-175 | 398/430 | 92 | 5/430 | 1 | Plus | Plus | Bacteria; Proteobacteria; Deltaproteobacteria; Desulfovibrionales; Desulfovibrionaceae; Desulfovibrio; Desulfovibrio intestinalis | Desulfovibrio intestinalis | Desulfovibrio | Desulfovibrionaceae | Desulfovibrionales | Deltaproteobacteria | Proteobacteria | Bacteria |
| ASV2653 | 402 | 1 | 1 | 402 | 100 | NR_026314.1 Blautia hydrogenotrophica strain S5a36 16S ribosomal RNA, partial sequence | NR_026314.1 | 1458 | 347 | 748 | 27.57 | 616.1 | 4E-176 | 380/403 | 94 | 2/403 | 0 | Plus | Plus | Bacteria; Firmicutes; Clostridia; Eubacteriales; Lachnospiraceae; Blautia; Blautia hydrogenotrophica | Blautia hydrogenotrophica | Blautia | Lachnospiraceae | Eubacteriales | Clostridia | Firmicutes | Bacteria |
| ASV2654 | 427 | 2 | 3 | 427 | 99.53 | NR_029106.1 Lactobacillus delbrueckii subsp. indicus strain NCC725 16S ribosomal RNA, partial sequence | NR_029106.1 | 1515 | 350 | 774 | 28.05 | 730.5 | 0 | 415/425 | 97 | 0/425 | 0 | Plus | Plus | Bacteria; Firmicutes; Bacilli; Lactobacillales; Lactobacillaceae; Lactobacillus; Lactobacillus delbrueckii | Lactobacillus delbrueckii | Lactobacillus | Lactobacillaceae | Lactobacillales | Bacilli | Firmicutes | Bacteria |
| ASV2655 | 427 | 1 | 1 | 427 | 100 | NR_028840.1 Mitsuokella jalaludinii strain M9 16S ribosomal RNA, partial sequence | NR_028840.1 | 1543 | 362 | 788 | 27.67 | 773 | 0 | 424/427 | 99 | 0/427 | 0 | Plus | Plus | Bacteria; Firmicutes; Negativicutes; Selenomonadales; Selenomonadaceae; Mitsuokella; Mitsuokella jalaludinii | Mitsuokella jalaludinii | Mitsuokella | Selenomonadaceae | Selenomonadales | Negativicutes | Firmicutes | Bacteria |
| ASV2656 | 402 | 1 | 1 | 393 | 97.76 | NR_118673.1 [Eubacterium] hallii strain ATCC 27751 16S ribosomal RNA, partial sequence | NR_118673.1 | 1479 | 371 | 763 | 26.57 | 424 | 3E-118 | 339/395 | 85 | 4/395 | 1 | Plus | Plus | Bacteria; Firmicutes; Clostridia; Eubacteriales; Lachnospiraceae; Anaerobutyricum; Anaerobutyricum hallii | Anaerobutyricum hallii | Anaerobutyricum | Lachnospiraceae | Eubacteriales | Clostridia | Firmicutes | Bacteria |
| ASV2657 | 427 | 1 | 1 | 427 | 100 | NR_146687.1 Breznakia pachnodae strain Pei061 16S ribosomal RNA, partial sequence | NR_146687.1 | 1499 | 367 | 792 | 28.42 | 460.9 | 2E-129 | 371/430 | 86 | 7/430 | 1 | Plus | Plus | Bacteria; Firmicutes; Erysipelotrichia; Erysipelotrichales; Erysipelotrichaceae; Breznakia; Breznakia pachnodae | Breznakia pachnodae | Breznakia | Erysipelotrichaceae | Erysipelotrichales | Erysipelotrichia | Firmicutes | Bacteria |
| ASV2658 | 402 | 1 | 1 | 402 | 100 | NR_028961.1 Faecalibacterium prausnitzii strain ATCC 27768 16S ribosomal RNA, partial sequence | NR_028961.1 | 1462 | 344 | 745 | 27.5 | 649.3 | 0 | 385/402 | 95 | 0/402 | 0 | Plus | Plus | Bacteria; Firmicutes; Clostridia; Eubacteriales; Oscillospiraceae; Faecalibacterium; Faecalibacterium prausnitzii | Faecalibacterium prausnitzii | Faecalibacterium | Oscillospiraceae | Eubacteriales | Clostridia | Firmicutes | Bacteria |
| ASV2659 | 401 | 1 | 1 | 401 | 100 | NR_152059.1 Harryflintia acetispora strain V20-281a 16S ribosomal RNA, partial sequence | NR_152059.1 | 1363 | 289 | 692 | 29.64 | 599.4 | 4E-171 | 378/404 | 93 | 3/404 | 0 | Plus | Plus | Bacteria; Firmicutes; Clostridia; Eubacteriales; Oscillospiraceae; Harryflintia; Harryflintia acetispora | Harryflintia acetispora | Harryflintia | Oscillospiraceae | Eubacteriales | Clostridia | Firmicutes | Bacteria |
| ASV2660 | 427 | 2 | 1 | 427 | 100 | NR_037018.1 Acidaminococcus fermentans strain VR4 16S ribosomal RNA gene, partial sequence | NR_037018.1 | 1516 | 349 | 775 | 28.17 | 734.2 | 0 | 417/427 | 97 | 0/427 | 0 | Plus | Plus | Bacteria; Firmicutes; Negativicutes; Acidaminococcales; Acidaminococcaceae; Acidaminococcus; Acidaminococcus fermentans | Acidaminococcus fermentans | Acidaminococcus | Acidaminococcaceae | Acidaminococcales | Negativicutes | Firmicutes | Bacteria |
| ASV2661 | 427 | 2 | 15 | 427 | 96.72 | NR_025713.1 Spiroplasma velocicrescens strain MQ-4 16S ribosomal RNA, partial sequence | NR_025713.1 | 1515 | 359 | 770 | 27.19 | 435.1 | 1E-121 | 355/414 | 85 | 3/414 | 0 | Plus | Plus | Bacteria; Tenericutes; Mollicutes; Entomoplasmatales; Spiroplasmataceae; Spiroplasma; Spiroplasma velocicrescens | Spiroplasma velocicrescens | Spiroplasma | Spiroplasmataceae | Entomoplasmatales | Mollicutes | Tenericutes | Bacteria |
| ASV2662 | 402 | 1 | 1 | 402 | 100 | NR_028961.1 Faecalibacterium prausnitzii strain ATCC 27768 16S ribosomal RNA, partial sequence | NR_028961.1 | 1462 | 344 | 745 | 27.5 | 732.4 | 0 | 400/402 | 99 | 0/402 | 0 | Plus | Plus | Bacteria; Firmicutes; Clostridia; Eubacteriales; Oscillospiraceae; Faecalibacterium; Faecalibacterium prausnitzii | Faecalibacterium prausnitzii | Faecalibacterium | Oscillospiraceae | Eubacteriales | Clostridia | Firmicutes | Bacteria |
| ASV2663 | 404 | 1 | 1 | 404 | 100 | NR_042074.1 Eubacterium pyruvativorans strain I-6 16S ribosomal RNA, partial sequence | NR_042074.1 | 1296 | 264 | 667 | 31.17 | 575.4 | 7E-164 | 373/404 | 92 | 0/404 | 0 | Plus | Plus | Bacteria; Firmicutes; Clostridia; Eubacteriales; Eubacteriaceae; Eubacterium; Eubacterium pyruvativorans | Eubacterium pyruvativorans | Eubacterium | Eubacteriaceae | Eubacteriales | Clostridia | Firmicutes | Bacteria |
| ASV2664 | 419 | 1 | 1 | 419 | 100 | NR_042150.1 Fibrobacter intestinalis strain NR9 16S ribosomal RNA, partial sequence | NR_042150.1 | 1436 | 325 | 743 | 29.18 | 603.1 | 3E-172 | 389/420 | 92 | 2/420 | 0 | Plus | Plus | Bacteria; Fibrobacteres; Fibrobacteria; Fibrobacterales; Fibrobacteraceae; Fibrobacter; Fibrobacter intestinalis | Fibrobacter intestinalis | Fibrobacter | Fibrobacteraceae | Fibrobacterales | Fibrobacteria | Fibrobacteres | Bacteria |
| ASV2666 | 402 | 1 | 1 | 402 | 100 | NR_117867.1 Blautia stercoris strain GAM6-1 16S ribosomal RNA, partial sequence | NR_117867.1 | 1372 | 317 | 718 | 29.3 | 527.4 | 2E-149 | 364/403 | 90 | 2/403 | 0 | Plus | Plus | Bacteria; Firmicutes; Clostridia; Eubacteriales; Lachnospiraceae; Blautia; Blautia stercoris | Blautia stercoris | Blautia | Lachnospiraceae | Eubacteriales | Clostridia | Firmicutes | Bacteria |
| ASV2667 | 426 | 1 | 1 | 424 | 99.53 | NR_044731.2 Holdemanella biformis strain DSM 3989 16S ribosomal RNA, partial sequence | NR_044731.2 | 1487 | 364 | 787 | 28.51 | 708.4 | 0 | 410/424 | 96 | 0/424 | 0 | Plus | Plus | Bacteria; Firmicutes; Erysipelotrichia; Erysipelotrichales; Erysipelotrichaceae; Holdemanella; Holdemanella biformis | Holdemanella biformis | Holdemanella | Erysipelotrichaceae | Erysipelotrichales | Erysipelotrichia | Firmicutes | Bacteria |
| ASV2669 | 402 | 1 | 1 | 402 | 100 | NR_152095.1 Herbinix luporum strain SD1D 16S ribosomal RNA, partial sequence | NR_152095.1 | 1521 | 365 | 766 | 26.43 | 688.1 | 0 | 392/402 | 97 | 0/402 | 0 | Plus | Plus | Bacteria; Firmicutes; Clostridia; Eubacteriales; Lachnospiraceae; Herbinix; Herbinix luporum | Herbinix luporum | Herbinix | Lachnospiraceae | Eubacteriales | Clostridia | Firmicutes | Bacteria |
| ASV2670 | 402 | 1 | 1 | 402 | 100 | NR_134772.1 Murimonas intestini strain SRB-530-5-H 16S ribosomal RNA, partial sequence | NR_134772.1 | 1433 | 314 | 715 | 28.05 | 688.1 | 0 | 392/402 | 97 | 0/402 | 0 | Plus | Plus | Bacteria; Firmicutes; Clostridia; Eubacteriales; Lachnospiraceae; Murimonas; Murimonas intestini | Murimonas intestini | Murimonas | Lachnospiraceae | Eubacteriales | Clostridia | Firmicutes | Bacteria |
| ASV2672 | 421 | 1 | 1 | 421 | 100 | NR_113411.1 Prevotella copri DSM 18205 strain JCM 13464 16S ribosomal RNA, partial sequence | NR_113411.1 | 1493 | 358 | 779 | 28.27 | 468.3 | 1E-131 | 374/430 | 86 | 17/430 | 3 | Plus | Plus | Bacteria; Bacteroidetes; Bacteroidia; Bacteroidales; Prevotellaceae; Prevotella; Prevotella copri | Prevotella copri | Prevotella | Prevotellaceae | Bacteroidales | Bacteroidia | Bacteroidetes | Bacteria |
| ASV2673 | 423 | 1 | 1 | 423 | 100 | NR_144750.1 Prevotellamassilia timonensis strain Marseille-P2831 16S ribosomal RNA, partial sequence | NR_144750.1 | 1493 | 358 | 779 | 28.27 | 553.3 | 4E-157 | 384/425 | 90 | 5/425 | 1 | Plus | Plus | Bacteria; Bacteroidetes; Bacteroidia; Bacteroidales; Prevotellaceae; Prevotellamassilia; Prevotellamassilia timonensis | Prevotellamassilia timonensis | Prevotellamassilia | Prevotellaceae | Bacteroidales | Bacteroidia | Bacteroidetes | Bacteria |
| ASV2674 | 427 | 4 | 3 | 427 | 99.53 | NR_041781.1 Streptococcus alactolyticus strain ATCC 43077 16S ribosomal RNA, partial sequence | NR_041781.1 | 1437 | 348 | 772 | 29.58 | 603.1 | 3E-172 | 398/431 | 92 | 12/431 | 2 | Plus | Plus | Bacteria; Firmicutes; Bacilli; Lactobacillales; Streptococcaceae; Streptococcus; Streptococcus alactolyticus | Streptococcus alactolyticus | Streptococcus | Streptococcaceae | Lactobacillales | Bacilli | Firmicutes | Bacteria |
| ASV2675 | 427 | 2 | 1 | 427 | 100 | NR_118848.1 Actinobacillus indolicus strain 46KC2 16S ribosomal RNA, partial sequence | NR_118848.1 | 1464 | 333 | 759 | 29.17 | 723.2 | 0 | 415/427 | 97 | 0/427 | 0 | Plus | Plus | Bacteria; Proteobacteria; Gammaproteobacteria; Pasteurellales; Pasteurellaceae; Actinobacillus; Actinobacillus indolicus | Actinobacillus indolicus | Actinobacillus | Pasteurellaceae | Pasteurellales | Gammaproteobacteria | Proteobacteria | Bacteria |
| ASV2676 | 427 | 1 | 1 | 427 | 100 | NR_113259.1 Lactobacillus agilis strain JCM 1187 16S ribosomal RNA, partial sequence | NR_113259.1 | 1485 | 351 | 777 | 28.75 | 778.6 | 0 | 425/427 | 99 | 0/427 | 0 | Plus | Plus | Bacteria; Firmicutes; Bacilli; Lactobacillales; Lactobacillaceae; Ligilactobacillus; Ligilactobacillus agilis | Ligilactobacillus agilis | Ligilactobacillus | Lactobacillaceae | Lactobacillales | Bacilli | Firmicutes | Bacteria |
| ASV2679 | 422 | 1 | 1 | 422 | 100 | NR_043216.1 Prevotella loescheii strain NCTC 11321 16S ribosomal RNA, partial sequence | NR_043216.1 | 1451 | 336 | 757 | 29.08 | 593.9 | 2E-169 | 391/425 | 92 | 6/425 | 1 | Plus | Plus | Bacteria; Bacteroidetes; Bacteroidia; Bacteroidales; Prevotellaceae; Prevotella; Prevotella loescheii | Prevotella loescheii | Prevotella | Prevotellaceae | Bacteroidales | Bacteroidia | Bacteroidetes | Bacteria |
| ASV2680 | 422 | 2 | 1 | 422 | 100 | NR_113076.1 Parabacteroides goldsteinii DSM 19448 = WAL 12034 strain JCM 13446 16S ribosomal RNA, partial sequence | NR_113076.1 | 1476 | 341 | 762 | 28.59 | 503.4 | 4E-142 | 372/422 | 88 | 0/422 | 0 | Plus | Plus | Bacteria; Bacteroidetes; Bacteroidia; Bacteroidales; Tannerellaceae; Parabacteroides; Parabacteroides goldsteinii | Parabacteroides goldsteinii | Parabacteroides | Tannerellaceae | Bacteroidales | Bacteroidia | Bacteroidetes | Bacteria |
| ASV2681 | 402 | 2 | 1 | 402 | 100 | NR_156080.1 Kineothrix alysoides strain KNHs209 16S ribosomal RNA, partial sequence | NR_156080.1 | 1410 | 353 | 754 | 28.51 | 688.1 | 0 | 392/402 | 97 | 0/402 | 0 | Plus | Plus | Bacteria; Firmicutes; Clostridia; Eubacteriales; Lachnospiraceae; Kineothrix; Kineothrix alysoides | Kineothrix alysoides | Kineothrix | Lachnospiraceae | Eubacteriales | Clostridia | Firmicutes | Bacteria |
| ASV2682 | 404 | 1 | 1 | 404 | 100 | NR_026476.1 Succinivibrio dextrinosolvens strain 0554 16S ribosomal RNA, partial sequence | NR_026476.1 | 1463 | 331 | 732 | 27.48 | 651.1 | 0 | 387/404 | 95 | 2/404 | 0 | Plus | Plus | Bacteria; Proteobacteria; Gammaproteobacteria; Aeromonadales; Succinivibrionaceae; Succinivibrio; Succinivibrio dextrinosolvens | Succinivibrio dextrinosolvens | Succinivibrio | Succinivibrionaceae | Aeromonadales | Gammaproteobacteria | Proteobacteria | Bacteria |
| ASV2683 | 405 | 1 | 1 | 405 | 100 | NR_026476.1 Succinivibrio dextrinosolvens strain 0554 16S ribosomal RNA, partial sequence | NR_026476.1 | 1463 | 331 | 732 | 27.48 | 678.8 | 0 | 393/405 | 97 | 3/405 | 0 | Plus | Plus | Bacteria; Proteobacteria; Gammaproteobacteria; Aeromonadales; Succinivibrionaceae; Succinivibrio; Succinivibrio dextrinosolvens | Succinivibrio dextrinosolvens | Succinivibrio | Succinivibrionaceae | Aeromonadales | Gammaproteobacteria | Proteobacteria | Bacteria |
| ASV2684 | 426 | 1 | 1 | 426 | 100 | NR_029335.1 Holdemania filiformis strain J1-31B-1 16S ribosomal RNA, partial sequence | NR_029335.1 | 1391 | 267 | 692 | 30.63 | 555.1 | 1E-157 | 385/427 | 90 | 2/427 | 0 | Plus | Plus | Bacteria; Firmicutes; Erysipelotrichia; Erysipelotrichales; Erysipelotrichaceae; Holdemania; Holdemania filiformis | Holdemania filiformis | Holdemania | Erysipelotrichaceae | Erysipelotrichales | Erysipelotrichia | Firmicutes | Bacteria |
| ASV2685 | 422 | 1 | 1 | 422 | 100 | NR_041364.1 Prevotella stercorea DSM 18206 strain CB35 16S ribosomal RNA, partial sequence | NR_041364.1 | 1488 | 354 | 775 | 28.36 | 553.3 | 4E-157 | 382/423 | 90 | 2/423 | 0 | Plus | Plus | Bacteria; Bacteroidetes; Bacteroidia; Bacteroidales; Prevotellaceae; Prevotella; Prevotella stercorea | Prevotella stercorea | Prevotella | Prevotellaceae | Bacteroidales | Bacteroidia | Bacteroidetes | Bacteria |
| ASV2686 | 404 | 1 | 1 | 404 | 100 | NR_151900.1 Ruthenibacterium lactatiformans strain 585-1 16S ribosomal RNA, partial sequence | NR_151900.1 | 1519 | 368 | 769 | 26.46 | 512.6 | 6E-145 | 365/407 | 89 | 8/407 | 1 | Plus | Plus | Bacteria; Firmicutes; Clostridia; Eubacteriales; Oscillospiraceae; Ruthenibacterium; Ruthenibacterium lactatiformans | Ruthenibacterium lactatiformans | Ruthenibacterium | Oscillospiraceae | Eubacteriales | Clostridia | Firmicutes | Bacteria |
| ASV2687 | 421 | 1 | 1 | 421 | 100 | NR_144750.1 Prevotellamassilia timonensis strain Marseille-P2831 16S ribosomal RNA, partial sequence | NR_144750.1 | 1493 | 358 | 779 | 28.27 | 656.7 | 0 | 400/422 | 94 | 1/422 | 0 | Plus | Plus | Bacteria; Bacteroidetes; Bacteroidia; Bacteroidales; Prevotellaceae; Prevotellamassilia; Prevotellamassilia timonensis | Prevotellamassilia timonensis | Prevotellamassilia | Prevotellaceae | Bacteroidales | Bacteroidia | Bacteroidetes | Bacteria |
| ASV2688 | 422 | 2 | 1 | 422 | 100 | NR_074376.1 Parabacteroides distasonis ATCC 8503 16S ribosomal RNA, partial sequence | NR_074376.1 | 1497 | 368 | 789 | 28.19 | 575.4 | 7E-164 | 385/422 | 91 | 0/422 | 0 | Plus | Plus | Bacteria; Bacteroidetes; Bacteroidia; Bacteroidales; Tannerellaceae; Parabacteroides; Parabacteroides distasonis | Parabacteroides distasonis | Parabacteroides | Tannerellaceae | Bacteroidales | Bacteroidia | Bacteroidetes | Bacteria |
| ASV2689 | 402 | 1 | 1 | 402 | 100 | NR_118730.1 [Clostridium] symbiosum strain ATCC 14940 16S ribosomal RNA, partial sequence | NR_118730.1 | 1478 | 372 | 773 | 27.2 | 721.3 | 0 | 396/402 | 98 | 0/402 | 0 | Plus | Plus | Bacteria; Firmicutes; Clostridia; Eubacteriales; Lachnospiraceae; Lachnoclostridium; [Clostridium] symbiosum | [Clostridium] symbiosum | Lachnoclostridium | Lachnospiraceae | Eubacteriales | Clostridia | Firmicutes | Bacteria |
| ASV2690 | 427 | 1 | 1 | 427 | 100 | NR_042961.1 Acholeplasma parvum strain H23M 16S ribosomal RNA, partial sequence | NR_042961.1 | 1426 | 315 | 740 | 29.87 | 472 | 1E-132 | 377/434 | 86 | 15/434 | 3 | Plus | Plus | Bacteria; Tenericutes; Mollicutes; Acholeplasmatales; Acholeplasmataceae; Acholeplasma; Acholeplasma parvum | Acholeplasma parvum | Acholeplasma | Acholeplasmataceae | Acholeplasmatales | Mollicutes | Tenericutes | Bacteria |
| ASV2691 | 405 | 1 | 1 | 405 | 100 | NR_115269.1 Catabacter hongkongensis strain HKU16 16S ribosomal RNA, partial sequence | NR_115269.1 | 1453 | 349 | 753 | 27.87 | 422.2 | 1E-117 | 347/406 | 85 | 2/406 | 0 | Plus | Plus | Bacteria; Firmicutes; Clostridia; Eubacteriales; Christensenellaceae; Christensenella; Christensenella hongkongensis | Christensenella hongkongensis | Christensenella | Christensenellaceae | Eubacteriales | Clostridia | Firmicutes | Bacteria |
| ASV2692 | 405 | 2 | 1 | 405 | 100 | NR_029356.1 Flavonifractor plautii strain 265 16S ribosomal RNA, partial sequence | NR_029356.1 | 1550 | 353 | 757 | 26.13 | 671.5 | 0 | 392/406 | 96 | 2/406 | 0 | Plus | Plus | Bacteria; Firmicutes; Clostridia; Eubacteriales; Oscillospiraceae; Flavonifractor; Flavonifractor plautii | Flavonifractor plautii | Flavonifractor | Oscillospiraceae | Eubacteriales | Clostridia | Firmicutes | Bacteria |
| ASV2693 | 426 | 2 | 1 | 426 | 100 | NR_042260.1 Sphaerochaeta coccoides strain SPN1 16S ribosomal RNA, partial sequence | NR_042260.1 | 1500 | 320 | 746 | 28.47 | 499.7 | 5E-141 | 377/429 | 87 | 5/429 | 1 | Plus | Plus | Bacteria; Spirochaetes; Spirochaetia; Spirochaetales; Spirochaetaceae; Sphaerochaeta; Sphaerochaeta coccoides | Sphaerochaeta coccoides | Sphaerochaeta | Spirochaetaceae | Spirochaetales | Spirochaetia | Spirochaetes | Bacteria |
| ASV2694 | 404 | 1 | 1 | 404 | 100 | NR_118156.1 Oscillibacter ruminantium GH1 16S ribosomal RNA, partial sequence | NR_118156.1 | 1409 | 321 | 724 | 28.67 | 658.5 | 0 | 390/406 | 96 | 4/406 | 0 | Plus | Plus | Bacteria; Firmicutes; Clostridia; Eubacteriales; Oscillospiraceae; Oscillibacter; Oscillibacter ruminantium | Oscillibacter ruminantium | Oscillibacter | Oscillospiraceae | Eubacteriales | Clostridia | Firmicutes | Bacteria |
| ASV2695 | 422 | 3 | 1 | 422 | 100 | NR_041818.1 Bacteroides coprosuis DSM 18011 strain PC139 16S ribosomal RNA, partial sequence | NR_041818.1 | 1523 | 357 | 778 | 27.71 | 486.8 | 4E-137 | 372/425 | 87 | 6/425 | 1 | Plus | Plus | Bacteria; Bacteroidetes; Bacteroidia; Bacteroidales; Bacteroidaceae; Bacteroides; Bacteroides coprosuis | Bacteroides coprosuis | Bacteroides | Bacteroidaceae | Bacteroidales | Bacteroidia | Bacteroidetes | Bacteria |
| ASV2696 | 405 | 2 | 1 | 405 | 100 | NR_156080.1 Kineothrix alysoides strain KNHs209 16S ribosomal RNA, partial sequence | NR_156080.1 | 1410 | 353 | 754 | 28.51 | 695.5 | 0 | 396/405 | 97 | 3/405 | 0 | Plus | Plus | Bacteria; Firmicutes; Clostridia; Eubacteriales; Lachnospiraceae; Kineothrix; Kineothrix alysoides | Kineothrix alysoides | Kineothrix | Lachnospiraceae | Eubacteriales | Clostridia | Firmicutes | Bacteria |
| ASV2697 | 403 | 1 | 3 | 403 | 99.5 | NR_125464.1 Anaerobacterium chartisolvens strain T-1-35 16S ribosomal RNA, partial sequence | NR_125464.1 | 1451 | 344 | 745 | 27.71 | 464.6 | 2E-130 | 352/402 | 87 | 1/402 | 0 | Plus | Plus | Bacteria; Firmicutes; Clostridia; Eubacteriales; Oscillospiraceae; Anaerobacterium; Anaerobacterium chartisolvens | Anaerobacterium chartisolvens | Anaerobacterium | Oscillospiraceae | Eubacteriales | Clostridia | Firmicutes | Bacteria |
| ASV2698 | 405 | 1 | 1 | 405 | 100 | NR_144611.1 Flintibacter butyricus strain BLS21 16S ribosomal RNA, partial sequence | NR_144611.1 | 808 | 299 | 703 | 50.12 | 477.6 | 2E-134 | 357/406 | 87 | 2/406 | 0 | Plus | Plus | Bacteria; Firmicutes; Clostridia; Eubacteriales; ; Flintibacter; Flintibacter butyricus | Flintibacter butyricus | Flintibacter | | Eubacteriales | Clostridia | Firmicutes | Bacteria |
| ASV2699 | 402 | 2 | 1 | 402 | 100 | NR_115549.1 Rhodospirillum rubrum ATCC 11170 16S ribosomal RNA, partial sequence | NR_115549.1 | 1406 | 299 | 700 | 28.59 | 449.9 | 4E-126 | 351/404 | 86 | 4/404 | 0 | Plus | Plus | Bacteria; Proteobacteria; Alphaproteobacteria; Rhodospirillales; Rhodospirillaceae; Rhodospirillum; Rhodospirillum rubrum | Rhodospirillum rubrum | Rhodospirillum | Rhodospirillaceae | Rhodospirillales | Alphaproteobacteria | Proteobacteria | Bacteria |
| ASV2700 | 422 | 1 | 1 | 422 | 100 | NR_041343.1 Parabacteroides merdae strain JCM 9497 16S ribosomal RNA, partial sequence | NR_041343.1 | 1492 | 357 | 778 | 28.28 | 780.4 | 0 | 422/422 | 100 | 0/422 | 0 | Plus | Plus | Bacteria; Bacteroidetes; Bacteroidia; Bacteroidales; Tannerellaceae; Parabacteroides; Parabacteroides merdae | Parabacteroides merdae | Parabacteroides | Tannerellaceae | Bacteroidales | Bacteroidia | Bacteroidetes | Bacteria |
| ASV2701 | 422 | 3 | 1 | 422 | 100 | NR_113411.1 Prevotella copri DSM 18205 strain JCM 13464 16S ribosomal RNA, partial sequence | NR_113411.1 | 1493 | 358 | 779 | 28.27 | 592 | 7E-169 | 389/423 | 91 | 2/423 | 0 | Plus | Plus | Bacteria; Bacteroidetes; Bacteroidia; Bacteroidales; Prevotellaceae; Prevotella; Prevotella copri | Prevotella copri | Prevotella | Prevotellaceae | Bacteroidales | Bacteroidia | Bacteroidetes | Bacteria |
| ASV2702 | 427 | 1 | 1 | 427 | 100 | NR_042942.1 Treponema porcinum strain 14V28 16S ribosomal RNA, partial sequence | NR_042942.1 | 1426 | 258 | 684 | 29.94 | 695.5 | 0 | 410/427 | 96 | 0/427 | 0 | Plus | Plus | Bacteria; Spirochaetes; Spirochaetia; Spirochaetales; Spirochaetaceae; Treponema; Treponema porcinum | Treponema porcinum | Treponema | Spirochaetaceae | Spirochaetales | Spirochaetia | Spirochaetes | Bacteria |
| ASV2703 | 427 | 1 | 4 | 427 | 99.3 | NR_146687.1 Breznakia pachnodae strain Pei061 16S ribosomal RNA, partial sequence | NR_146687.1 | 1499 | 370 | 792 | 28.22 | 494.2 | 2E-139 | 374/426 | 87 | 5/426 | 1 | Plus | Plus | Bacteria; Firmicutes; Erysipelotrichia; Erysipelotrichales; Erysipelotrichaceae; Breznakia; Breznakia pachnodae | Breznakia pachnodae | Breznakia | Erysipelotrichaceae | Erysipelotrichales | Erysipelotrichia | Firmicutes | Bacteria |
| ASV2705 | 403 | 1 | 1 | 403 | 100 | NR_025930.1 Ruminococcus bromii strain ATCC 27255 16S ribosomal RNA, partial sequence | NR_025930.1 | 1380 | 313 | 716 | 29.28 | 551.4 | 1E-156 | 369/404 | 91 | 1/404 | 0 | Plus | Plus | Bacteria; Firmicutes; Clostridia; Eubacteriales; Oscillospiraceae; Ruminococcus; Ruminococcus bromii | Ruminococcus bromii | Ruminococcus | Oscillospiraceae | Eubacteriales | Clostridia | Firmicutes | Bacteria |
| ASV2706 | 422 | 2 | 1 | 422 | 100 | NR_113117.1 Prevotella oralis ATCC 33269 strain JCM 12251 16S ribosomal RNA, partial sequence | NR_113117.1 | 1496 | 361 | 782 | 28.21 | 603.1 | 3E-172 | 391/423 | 92 | 2/423 | 0 | Plus | Plus | Bacteria; Bacteroidetes; Bacteroidia; Bacteroidales; Prevotellaceae; Prevotella; Prevotella oralis | Prevotella oralis | Prevotella | Prevotellaceae | Bacteroidales | Bacteroidia | Bacteroidetes | Bacteria |
| ASV2707 | 423 | 1 | 1 | 423 | 100 | NR_144750.1 Prevotellamassilia timonensis strain Marseille-P2831 16S ribosomal RNA, partial sequence | NR_144750.1 | 1493 | 358 | 779 | 28.27 | 564.3 | 2E-160 | 386/425 | 90 | 5/425 | 1 | Plus | Plus | Bacteria; Bacteroidetes; Bacteroidia; Bacteroidales; Prevotellaceae; Prevotellamassilia; Prevotellamassilia timonensis | Prevotellamassilia timonensis | Prevotellamassilia | Prevotellaceae | Bacteroidales | Bacteroidia | Bacteroidetes | Bacteria |
| ASV2708 | 422 | 2 | 1 | 422 | 100 | NR_112931.1 Bacteroides acidifaciens strain JCM 10556 16S ribosomal RNA, partial sequence | NR_112931.1 | 1490 | 357 | 778 | 28.32 | 774.9 | 0 | 421/422 | 99 | 0/422 | 0 | Plus | Plus | Bacteria; Bacteroidetes; Bacteroidia; Bacteroidales; Bacteroidaceae; Bacteroides; Bacteroides acidifaciens | Bacteroides acidifaciens | Bacteroides | Bacteroidaceae | Bacteroidales | Bacteroidia | Bacteroidetes | Bacteria |
| ASV2709 | 402 | 1 | 1 | 402 | 100 | NR_151900.1 Ruthenibacterium lactatiformans strain 585-1 16S ribosomal RNA, partial sequence | NR_151900.1 | 1519 | 368 | 769 | 26.46 | 616.1 | 4E-176 | 379/402 | 94 | 0/402 | 0 | Plus | Plus | Bacteria; Firmicutes; Clostridia; Eubacteriales; Oscillospiraceae; Ruthenibacterium; Ruthenibacterium lactatiformans | Ruthenibacterium lactatiformans | Ruthenibacterium | Oscillospiraceae | Eubacteriales | Clostridia | Firmicutes | Bacteria |
| ASV2710 | 401 | 1 | 1 | 401 | 100 | NR_158111.1 Paludicola psychrotolerans strain NC1253 16S ribosomal RNA, partial sequence | NR_158111.1 | 1490 | 366 | 766 | 26.91 | 625.3 | 7E-179 | 380/401 | 94 | 0/401 | 0 | Plus | Plus | Bacteria; Firmicutes; Clostridia; Eubacteriales; Oscillospiraceae; Paludicola; Paludicola psychrotolerans | Paludicola psychrotolerans | Paludicola | Oscillospiraceae | Eubacteriales | Clostridia | Firmicutes | Bacteria |
| ASV2711 | 427 | 1 | 1 | 427 | 100 | NR_041055.1 Streptococcus orisuis strain NUM 1001 16S ribosomal RNA, partial sequence | NR_041055.1 | 1554 | 368 | 794 | 27.48 | 789.6 | 0 | 427/427 | 100 | 0/427 | 0 | Plus | Plus | Bacteria; Firmicutes; Bacilli; Lactobacillales; Streptococcaceae; Streptococcus; Streptococcus orisuis | Streptococcus orisuis | Streptococcus | Streptococcaceae | Lactobacillales | Bacilli | Firmicutes | Bacteria |
| ASV2712 | 422 | 1 | 1 | 422 | 100 | NR_041364.1 Prevotella stercorea DSM 18206 strain CB35 16S ribosomal RNA, partial sequence | NR_041364.1 | 1488 | 354 | 775 | 28.36 | 730.5 | 0 | 413/422 | 97 | 0/422 | 0 | Plus | Plus | Bacteria; Bacteroidetes; Bacteroidia; Bacteroidales; Prevotellaceae; Prevotella; Prevotella stercorea | Prevotella stercorea | Prevotella | Prevotellaceae | Bacteroidales | Bacteroidia | Bacteroidetes | Bacteria |
| ASV2713 | 403 | 1 | 1 | 403 | 100 | NR_025930.1 Ruminococcus bromii strain ATCC 27255 16S ribosomal RNA, partial sequence | NR_025930.1 | 1380 | 313 | 716 | 29.28 | 645.6 | 0 | 386/404 | 95 | 1/404 | 0 | Plus | Plus | Bacteria; Firmicutes; Clostridia; Eubacteriales; Oscillospiraceae; Ruminococcus; Ruminococcus bromii | Ruminococcus bromii | Ruminococcus | Oscillospiraceae | Eubacteriales | Clostridia | Firmicutes | Bacteria |
| ASV2715 | 422 | 1 | 1 | 422 | 100 | NR_144750.1 Prevotellamassilia timonensis strain Marseille-P2831 16S ribosomal RNA, partial sequence | NR_144750.1 | 1493 | 358 | 779 | 28.27 | 542.2 | 8E-154 | 379/422 | 89 | 0/422 | 0 | Plus | Plus | Bacteria; Bacteroidetes; Bacteroidia; Bacteroidales; Prevotellaceae; Prevotellamassilia; Prevotellamassilia timonensis | Prevotellamassilia timonensis | Prevotellamassilia | Prevotellaceae | Bacteroidales | Bacteroidia | Bacteroidetes | Bacteria |
| ASV2716 | 422 | 2 | 1 | 422 | 100 | NR_113119.1 Prevotella oris strain JCM 8540 16S ribosomal RNA, partial sequence | NR_113119.1 | 1481 | 345 | 766 | 28.49 | 531.1 | 2E-150 | 378/423 | 89 | 2/423 | 0 | Plus | Plus | Bacteria; Bacteroidetes; Bacteroidia; Bacteroidales; Prevotellaceae; Prevotella; Prevotella oris | Prevotella oris | Prevotella | Prevotellaceae | Bacteroidales | Bacteroidia | Bacteroidetes | Bacteria |
| ASV2717 | 402 | 2 | 3 | 402 | 99.5 | NR_024948.1 Campylobacter hyointestinalis subsp. lawsonii strain CHY5 16S ribosomal RNA, partial sequence | NR_024948.1 | 1419 | 320 | 720 | 28.26 | 606.8 | 3E-173 | 378/402 | 94 | 3/402 | 0 | Plus | Plus | Bacteria; Proteobacteria; Epsilonproteobacteria; Campylobacterales; Campylobacteraceae; Campylobacter; Campylobacter hyointestinalis | Campylobacter hyointestinalis | Campylobacter | Campylobacteraceae | Campylobacterales | Epsilonproteobacteria | Proteobacteria | Bacteria |
| ASV2718 | 405 | 1 | 1 | 405 | 100 | NR_159227.1 Monoglobus pectinilyticus strain 14 16S ribosomal RNA, partial sequence | NR_159227.1 | 1493 | 368 | 769 | 26.93 | 562.5 | 6E-160 | 373/406 | 91 | 5/406 | 1 | Plus | Plus | Bacteria; Firmicutes; Clostridia; Eubacteriales; Oscillospiraceae; Monoglobus; Monoglobus pectinilyticus | Monoglobus pectinilyticus | Monoglobus | Oscillospiraceae | Eubacteriales | Clostridia | Firmicutes | Bacteria |
| ASV2719 | 422 | 2 | 1 | 422 | 100 | NR_113122.1 Prevotella paludivivens strain JCM 13650 16S ribosomal RNA, partial sequence | NR_113122.1 | 1491 | 356 | 777 | 28.3 | 630.8 | 2E-180 | 395/422 | 93 | 0/422 | 0 | Plus | Plus | Bacteria; Bacteroidetes; Bacteroidia; Bacteroidales; Prevotellaceae; Prevotella; Prevotella paludivivens | Prevotella paludivivens | Prevotella | Prevotellaceae | Bacteroidales | Bacteroidia | Bacteroidetes | Bacteria |
| ASV2720 | 404 | 1 | 1 | 404 | 100 | NR_118156.1 Oscillibacter ruminantium GH1 16S ribosomal RNA, partial sequence | NR_118156.1 | 1409 | 321 | 724 | 28.67 | 653 | 0 | 389/406 | 95 | 4/406 | 0 | Plus | Plus | Bacteria; Firmicutes; Clostridia; Eubacteriales; Oscillospiraceae; Oscillibacter; Oscillibacter ruminantium | Oscillibacter ruminantium | Oscillibacter | Oscillospiraceae | Eubacteriales | Clostridia | Firmicutes | Bacteria |
| ASV2721 | 405 | 1 | 1 | 405 | 100 | NR_104907.1 Eubacterium coprostanoligenes strain HL 16S ribosomal RNA, partial sequence | NR_104907.1 | 1446 | 332 | 734 | 27.87 | 558.8 | 7E-159 | 371/405 | 91 | 2/405 | 0 | Plus | Plus | Bacteria; Firmicutes; Clostridia; Eubacteriales; Eubacteriaceae; Eubacterium; Eubacterium coprostanoligenes | Eubacterium coprostanoligenes | Eubacterium | Eubacteriaceae | Eubacteriales | Clostridia | Firmicutes | Bacteria |
| ASV2722 | 428 | 1 | 1 | 411 | 96.03 | NR_112902.1 Phascolarctobacterium succinatutens strain YIT 12067 16S ribosomal RNA, partial sequence | NR_112902.1 | 1610 | 444 | 853 | 25.47 | 453.5 | 4E-127 | 361/416 | 86 | 11/416 | 2 | Plus | Plus | Bacteria; Firmicutes; Negativicutes; Acidaminococcales; Acidaminococcaceae; Phascolarctobacterium; Phascolarctobacterium succinatutens | Phascolarctobacterium succinatutens | Phascolarctobacterium | Acidaminococcaceae | Acidaminococcales | Negativicutes | Firmicutes | Bacteria |
| ASV2724 | 405 | 1 | 1 | 405 | 100 | NR_144742.1 Christensenella massiliensis strain Marseille-P2438 16S ribosomal RNA, partial sequence | NR_144742.1 | 1499 | 369 | 773 | 27.02 | 472 | 1E-132 | 355/405 | 87 | 0/405 | 0 | Plus | Plus | Bacteria; Firmicutes; Clostridia; Eubacteriales; Christensenellaceae; Christensenella; Christensenella massiliensis | Christensenella massiliensis | Christensenella | Christensenellaceae | Eubacteriales | Clostridia | Firmicutes | Bacteria |
| ASV2725 | 403 | 1 | 1 | 403 | 100 | NR_029160.1 Ruminococcus callidus strain ATCC 27760 16S ribosomal RNA, partial sequence | NR_029160.1 | 1371 | 310 | 711 | 29.32 | 610.5 | 2E-174 | 381/405 | 94 | 5/405 | 1 | Plus | Plus | Bacteria; Firmicutes; Clostridia; Eubacteriales; Oscillospiraceae; Ruminococcus; Ruminococcus callidus | Ruminococcus callidus | Ruminococcus | Oscillospiraceae | Eubacteriales | Clostridia | Firmicutes | Bacteria |
| ASV2727 | 403 | 1 | 1 | 401 | 99.5 | NR_104911.1 Vampirovibrio chlorellavorus strain ICPB 3707 16S ribosomal RNA, partial sequence | NR_104911.1 | 1446 | 333 | 732 | 27.66 | 424 | 3E-118 | 344/401 | 85 | 1/401 | 0 | Plus | Plus | Bacteria; Candidatus Melainabacteria; ; Vampirovibrionales; ; Vampirovibrio; Vampirovibrio chlorellavorus | Vampirovibrio chlorellavorus | Vampirovibrio | | Vampirovibrionales | | Candidatus Melainabacteria | Bacteria |
| ASV2728 | 403 | 1 | 1 | 403 | 100 | NR_025930.1 Ruminococcus bromii strain ATCC 27255 16S ribosomal RNA, partial sequence | NR_025930.1 | 1380 | 313 | 716 | 29.28 | 575.4 | 7E-164 | 373/404 | 92 | 1/404 | 0 | Plus | Plus | Bacteria; Firmicutes; Clostridia; Eubacteriales; Oscillospiraceae; Ruminococcus; Ruminococcus bromii | Ruminococcus bromii | Ruminococcus | Oscillospiraceae | Eubacteriales | Clostridia | Firmicutes | Bacteria |
| ASV2729 | 405 | 1 | 1 | 405 | 100 | NR_044972.1 Sporobacter termitidis strain SYR 16S ribosomal RNA, partial sequence | NR_044972.1 | 1506 | 348 | 752 | 26.89 | 608.7 | 7E-174 | 379/405 | 93 | 0/405 | 0 | Plus | Plus | Bacteria; Firmicutes; Clostridia; Eubacteriales; Oscillospiraceae; Sporobacter; Sporobacter termitidis | Sporobacter termitidis | Sporobacter | Oscillospiraceae | Eubacteriales | Clostridia | Firmicutes | Bacteria |
| ASV2730 | 427 | 1 | 1 | 427 | 100 | NR_157643.1 Treponema rectale strain CHPA 16S ribosomal RNA, partial sequence | NR_157643.1 | 1309 | 259 | 685 | 32.62 | 584.7 | 1E-166 | 393/430 | 91 | 6/430 | 1 | Plus | Plus | Bacteria; Spirochaetes; Spirochaetia; Spirochaetales; Spirochaetaceae; Treponema; Treponema rectale | Treponema rectale | Treponema | Spirochaetaceae | Spirochaetales | Spirochaetia | Spirochaetes | Bacteria |
| ASV2731 | 402 | 1 | 1 | 402 | 100 | NR_151894.1 Anaerotaenia torta strain FH052 16S ribosomal RNA, partial sequence | NR_151894.1 | 1479 | 352 | 753 | 27.18 | 649.3 | 0 | 385/402 | 95 | 0/402 | 0 | Plus | Plus | Bacteria; Firmicutes; Clostridia; Eubacteriales; Lachnospiraceae; Anaerotaenia; Anaerotaenia torta | Anaerotaenia torta | Anaerotaenia | Lachnospiraceae | Eubacteriales | Clostridia | Firmicutes | Bacteria |
| ASV2732 | 423 | 1 | 1 | 423 | 100 | NR_144750.1 Prevotellamassilia timonensis strain Marseille-P2831 16S ribosomal RNA, partial sequence | NR_144750.1 | 1493 | 358 | 779 | 28.27 | 547.7 | 2E-155 | 383/425 | 90 | 5/425 | 1 | Plus | Plus | Bacteria; Bacteroidetes; Bacteroidia; Bacteroidales; Prevotellaceae; Prevotellamassilia; Prevotellamassilia timonensis | Prevotellamassilia timonensis | Prevotellamassilia | Prevotellaceae | Bacteroidales | Bacteroidia | Bacteroidetes | Bacteria |
| ASV2733 | 405 | 1 | 1 | 405 | 100 | NR_104907.1 Eubacterium coprostanoligenes strain HL 16S ribosomal RNA, partial sequence | NR_104907.1 | 1446 | 332 | 734 | 27.87 | 542.2 | 7E-154 | 368/405 | 90 | 2/405 | 0 | Plus | Plus | Bacteria; Firmicutes; Clostridia; Eubacteriales; Eubacteriaceae; Eubacterium; Eubacterium coprostanoligenes | Eubacterium coprostanoligenes | Eubacterium | Eubacteriaceae | Eubacteriales | Clostridia | Firmicutes | Bacteria |
| ASV2735 | 422 | 1 | 1 | 422 | 100 | NR_113195.1 Bacteroides sartorii JCM 17136 = DSM 21941 16S ribosomal RNA, partial sequence | NR_113195.1 | 1490 | 355 | 776 | 28.32 | 780.4 | 0 | 422/422 | 100 | 0/422 | 0 | Plus | Plus | Bacteria; Bacteroidetes; Bacteroidia; Bacteroidales; ; Phocaeicola; Phocaeicola sartorii | Phocaeicola sartorii | Phocaeicola | | Bacteroidales | Bacteroidia | Bacteroidetes | Bacteria |
| ASV2736 | 405 | 1 | 1 | 405 | 100 | NR_044490.1 Butyricicoccus pullicaecorum strain 25-3 16S ribosomal RNA, partial sequence | NR_044490.1 | 1482 | 366 | 770 | 27.33 | 438.8 | 1E-122 | 350/406 | 86 | 2/406 | 0 | Plus | Plus | Bacteria; Firmicutes; Clostridia; Eubacteriales; Clostridiaceae; Butyricicoccus; Butyricicoccus pullicaecorum | Butyricicoccus pullicaecorum | Butyricicoccus | Clostridiaceae | Eubacteriales | Clostridia | Firmicutes | Bacteria |
| ASV2737 | 427 | 1 | 1 | 427 | 100 | NR_044912.1 Streptococcus hyovaginalis strain SHV515 16S ribosomal RNA, partial sequence | NR_044912.1 | 1464 | 292 | 718 | 29.17 | 785.9 | 0 | 426/427 | 99 | 0/427 | 0 | Plus | Plus | Bacteria; Firmicutes; Bacilli; Lactobacillales; Streptococcaceae; Streptococcus; Streptococcus hyovaginalis | Streptococcus hyovaginalis | Streptococcus | Streptococcaceae | Lactobacillales | Bacilli | Firmicutes | Bacteria |
| ASV2738 | 405 | 3 | 1 | 405 | 100 | NR_115307.1 Ethanoligenens harbinense YUAN-3 16S ribosomal RNA, partial sequence | NR_115307.1 | 1497 | 340 | 743 | 26.99 | 569.9 | 3E-162 | 374/406 | 92 | 3/406 | 0 | Plus | Plus | Bacteria; Firmicutes; Clostridia; Eubacteriales; Oscillospiraceae; Ethanoligenens; Ethanoligenens harbinense | Ethanoligenens harbinense | Ethanoligenens | Oscillospiraceae | Eubacteriales | Clostridia | Firmicutes | Bacteria |
| ASV2739 | 427 | 2 | 1 | 426 | 99.77 | NR_037018.1 Acidaminococcus fermentans strain VR4 16S ribosomal RNA gene, partial sequence | NR_037018.1 | 1516 | 349 | 774 | 28.1 | 677 | 0 | 406/426 | 95 | 0/426 | 0 | Plus | Plus | Bacteria; Firmicutes; Negativicutes; Acidaminococcales; Acidaminococcaceae; Acidaminococcus; Acidaminococcus fermentans | Acidaminococcus fermentans | Acidaminococcus | Acidaminococcaceae | Acidaminococcales | Negativicutes | Firmicutes | Bacteria |
| ASV2740 | 404 | 1 | 1 | 404 | 100 | NR_144737.1 Emergencia timonensis strain SN18 16S ribosomal RNA, partial sequence | NR_144737.1 | 1480 | 364 | 767 | 27.3 | 619.7 | 3E-177 | 381/404 | 94 | 0/404 | 0 | Plus | Plus | Bacteria; Firmicutes; Clostridia; Eubacteriales; Clostridiales Family XIII. Incertae Sedis; Emergencia; Emergencia timonensis | Emergencia timonensis | Emergencia | Clostridiales Family XIII. Incertae Sedis | Eubacteriales | Clostridia | Firmicutes | Bacteria |
| ASV2741 | 422 | 1 | 1 | 422 | 100 | NR_041446.1 Bacteroides barnesiae strain BL2 16S ribosomal RNA, partial sequence | NR_041446.1 | 1456 | 340 | 761 | 28.98 | 575.4 | 7E-164 | 391/428 | 91 | 12/428 | 2 | Plus | Plus | Bacteria; Bacteroidetes; Bacteroidia; Bacteroidales; ; Phocaeicola; Phocaeicola barnesiae | Phocaeicola barnesiae | Phocaeicola | | Bacteroidales | Bacteroidia | Bacteroidetes | Bacteria |
| ASV2742 | 405 | 2 | 1 | 405 | 100 | NR_144742.1 Christensenella massiliensis strain Marseille-P2438 16S ribosomal RNA, partial sequence | NR_144742.1 | 1499 | 369 | 773 | 27.02 | 460.9 | 2E-129 | 353/405 | 87 | 0/405 | 0 | Plus | Plus | Bacteria; Firmicutes; Clostridia; Eubacteriales; Christensenellaceae; Christensenella; Christensenella massiliensis | Christensenella massiliensis | Christensenella | Christensenellaceae | Eubacteriales | Clostridia | Firmicutes | Bacteria |
| ASV2743 | 402 | 5 | 1 | 402 | 100 | NR_147395.1 Blautia marasmi strain Marseille-P2377 16S ribosomal RNA, partial sequence | NR_147395.1 | 1496 | 365 | 766 | 26.87 | 743.5 | 0 | 402/402 | 100 | 0/402 | 0 | Plus | Plus | Bacteria; Firmicutes; Clostridia; Eubacteriales; Lachnospiraceae; Blautia; Blautia marasmi | Blautia marasmi | Blautia | Lachnospiraceae | Eubacteriales | Clostridia | Firmicutes | Bacteria |
| ASV2744 | 402 | 1 | 1 | 402 | 100 | NR_044972.1 Sporobacter termitidis strain SYR 16S ribosomal RNA, partial sequence | NR_044972.1 | 1506 | 348 | 752 | 26.89 | 616.1 | 4E-176 | 381/405 | 94 | 3/405 | 0 | Plus | Plus | Bacteria; Firmicutes; Clostridia; Eubacteriales; Oscillospiraceae; Sporobacter; Sporobacter termitidis | Sporobacter termitidis | Sporobacter | Oscillospiraceae | Eubacteriales | Clostridia | Firmicutes | Bacteria |
| ASV2745 | 402 | 1 | 1 | 402 | 100 | NR_148620.1 Lachnotalea glycerini strain : DLD10 16S ribosomal RNA, partial sequence | NR_148620.1 | 1207 | 212 | 613 | 33.31 | 660.4 | 0 | 387/402 | 96 | 0/402 | 0 | Plus | Plus | Bacteria; Firmicutes; Clostridia; Eubacteriales; Lachnospiraceae; Lachnotalea; Lachnotalea glycerini | Lachnotalea glycerini | Lachnotalea | Lachnospiraceae | Eubacteriales | Clostridia | Firmicutes | Bacteria |
| ASV2746 | 428 | 1 | 1 | 428 | 100 | NR_117375.1 Streptococcus danieliae strain ERD01G 16S ribosomal RNA, partial sequence | NR_117375.1 | 1419 | 314 | 740 | 30.09 | 523.7 | 3E-148 | 386/434 | 88 | 13/434 | 2 | Plus | Plus | Bacteria; Firmicutes; Bacilli; Lactobacillales; Streptococcaceae; Streptococcus; Streptococcus danieliae | Streptococcus danieliae | Streptococcus | Streptococcaceae | Lactobacillales | Bacilli | Firmicutes | Bacteria |
| ASV2747 | 405 | 1 | 1 | 405 | 100 | NR_044490.1 Butyricicoccus pullicaecorum strain 25-3 16S ribosomal RNA, partial sequence | NR_044490.1 | 1482 | 366 | 770 | 27.33 | 693.6 | 0 | 395/405 | 97 | 0/405 | 0 | Plus | Plus | Bacteria; Firmicutes; Clostridia; Eubacteriales; Clostridiaceae; Butyricicoccus; Butyricicoccus pullicaecorum | Butyricicoccus pullicaecorum | Butyricicoccus | Clostridiaceae | Eubacteriales | Clostridia | Firmicutes | Bacteria |
| ASV2748 | 421 | 1 | 1 | 421 | 100 | NR_144616.1 Muribaculum intestinale strain YL27 16S ribosomal RNA, partial sequence | NR_144616.1 | 1460 | 297 | 718 | 28.9 | 512.6 | 6E-145 | 376/424 | 88 | 5/424 | 1 | Plus | Plus | Bacteria; Bacteroidetes; Bacteroidia; Bacteroidales; Muribaculaceae; Muribaculum; Muribaculum intestinale | Muribaculum intestinale | Muribaculum | Muribaculaceae | Bacteroidales | Bacteroidia | Bacteroidetes | Bacteria |
| ASV2749 | 422 | 1 | 1 | 422 | 100 | NR_027196.1 Bacteroides stercoris ATCC 43183 16S ribosomal RNA, partial sequence | NR_027196.1 | 1342 | 334 | 755 | 31.45 | 649.3 | 0 | 398/422 | 94 | 0/422 | 0 | Plus | Plus | Bacteria; Bacteroidetes; Bacteroidia; Bacteroidales; Bacteroidaceae; Bacteroides; Bacteroides stercoris | Bacteroides stercoris | Bacteroides | Bacteroidaceae | Bacteroidales | Bacteroidia | Bacteroidetes | Bacteria |
| ASV2750 | 402 | 2 | 3 | 402 | 99.5 | NR_024948.1 Campylobacter hyointestinalis subsp. lawsonii strain CHY5 16S ribosomal RNA, partial sequence | NR_024948.1 | 1419 | 320 | 720 | 28.26 | 601.3 | 1E-171 | 379/404 | 93 | 7/404 | 1 | Plus | Plus | Bacteria; Proteobacteria; Epsilonproteobacteria; Campylobacterales; Campylobacteraceae; Campylobacter; Campylobacter hyointestinalis | Campylobacter hyointestinalis | Campylobacter | Campylobacteraceae | Campylobacterales | Epsilonproteobacteria | Proteobacteria | Bacteria |
| ASV2751 | 421 | 1 | 1 | 421 | 100 | NR_144616.1 Muribaculum intestinale strain YL27 16S ribosomal RNA, partial sequence | NR_144616.1 | 1460 | 297 | 718 | 28.9 | 501.6 | 1E-141 | 379/429 | 88 | 15/429 | 3 | Plus | Plus | Bacteria; Bacteroidetes; Bacteroidia; Bacteroidales; Muribaculaceae; Muribaculum; Muribaculum intestinale | Muribaculum intestinale | Muribaculum | Muribaculaceae | Bacteroidales | Bacteroidia | Bacteroidetes | Bacteria |
| ASV2752 | 404 | 1 | 1 | 404 | 100 | NR_144611.1 Flintibacter butyricus strain BLS21 16S ribosomal RNA, partial sequence | NR_144611.1 | 808 | 299 | 703 | 50.12 | 619.7 | 3E-177 | 382/405 | 94 | 1/405 | 0 | Plus | Plus | Bacteria; Firmicutes; Clostridia; Eubacteriales; ; Flintibacter; Flintibacter butyricus | Flintibacter butyricus | Flintibacter | | Eubacteriales | Clostridia | Firmicutes | Bacteria |
| ASV2753 | 427 | 1 | 1 | 427 | 100 | NR_112902.1 Phascolarctobacterium succinatutens strain YIT 12067 16S ribosomal RNA, partial sequence | NR_112902.1 | 1610 | 444 | 870 | 26.52 | 778.6 | 0 | 425/427 | 99 | 0/427 | 0 | Plus | Plus | Bacteria; Firmicutes; Negativicutes; Acidaminococcales; Acidaminococcaceae; Phascolarctobacterium; Phascolarctobacterium succinatutens | Phascolarctobacterium succinatutens | Phascolarctobacterium | Acidaminococcaceae | Acidaminococcales | Negativicutes | Firmicutes | Bacteria |
| ASV2754 | 402 | 3 | 1 | 402 | 100 | NR_156081.1 Kineothrix alysoides strain KNHs209 16S ribosomal RNA, partial sequence | NR_156081.1 | 1411 | 353 | 754 | 28.49 | 699.2 | 0 | 394/402 | 98 | 0/402 | 0 | Plus | Plus | Bacteria; Firmicutes; Clostridia; Eubacteriales; Lachnospiraceae; Kineothrix; Kineothrix alysoides | Kineothrix alysoides | Kineothrix | Lachnospiraceae | Eubacteriales | Clostridia | Firmicutes | Bacteria |
| ASV2756 | 422 | 4 | 1 | 422 | 100 | NR_044864.1 Hallella seregens ATCC 51272 16S ribosomal RNA, partial sequence | NR_044864.1 | 1463 | 335 | 756 | 28.84 | 603.1 | 3E-172 | 391/423 | 92 | 2/423 | 0 | Plus | Plus | Bacteria; Bacteroidetes; Bacteroidia; Bacteroidales; Prevotellaceae; Prevotella; Hallella seregens | Hallella seregens | Prevotella | Prevotellaceae | Bacteroidales | Bacteroidia | Bacteroidetes | Bacteria |
| ASV2757 | 402 | 2 | 1 | 402 | 100 | NR_024948.1 Campylobacter hyointestinalis subsp. lawsonii strain CHY5 16S ribosomal RNA, partial sequence | NR_024948.1 | 1419 | 318 | 720 | 28.4 | 555.1 | 9E-158 | 372/406 | 91 | 7/406 | 1 | Plus | Plus | Bacteria; Proteobacteria; Epsilonproteobacteria; Campylobacterales; Campylobacteraceae; Campylobacter; Campylobacter hyointestinalis | Campylobacter hyointestinalis | Campylobacter | Campylobacteraceae | Campylobacterales | Epsilonproteobacteria | Proteobacteria | Bacteria |
| ASV2758 | 422 | 1 | 1 | 422 | 100 | NR_144747.1 Mediterranea massiliensis strain Marseille-P2645 16S ribosomal RNA, partial sequence | NR_144747.1 | 1491 | 354 | 775 | 28.3 | 581 | 2E-165 | 386/422 | 91 | 0/422 | 0 | Plus | Plus | Bacteria; Bacteroidetes; Bacteroidia; Bacteroidales; Bacteroidaceae; Mediterranea; Mediterranea massiliensis | Mediterranea massiliensis | Mediterranea | Bacteroidaceae | Bacteroidales | Bacteroidia | Bacteroidetes | Bacteria |
| ASV2760 | 405 | 1 | 1 | 405 | 100 | NR_144611.1 Flintibacter butyricus strain BLS21 16S ribosomal RNA, partial sequence | NR_144611.1 | 808 | 299 | 703 | 50.12 | 533 | 4E-151 | 366/405 | 90 | 0/405 | 0 | Plus | Plus | Bacteria; Firmicutes; Clostridia; Eubacteriales; ; Flintibacter; Flintibacter butyricus | Flintibacter butyricus | Flintibacter | | Eubacteriales | Clostridia | Firmicutes | Bacteria |
| ASV2761 | 420 | 1 | 1 | 420 | 100 | NR_144616.1 Muribaculum intestinale strain YL27 16S ribosomal RNA, partial sequence | NR_144616.1 | 1460 | 297 | 718 | 28.9 | 584.7 | 1E-166 | 387/422 | 91 | 2/422 | 0 | Plus | Plus | Bacteria; Bacteroidetes; Bacteroidia; Bacteroidales; Muribaculaceae; Muribaculum; Muribaculum intestinale | Muribaculum intestinale | Muribaculum | Muribaculaceae | Bacteroidales | Bacteroidia | Bacteroidetes | Bacteria |
| ASV2762 | 422 | 1 | 1 | 422 | 100 | NR_041364.1 Prevotella stercorea DSM 18206 strain CB35 16S ribosomal RNA, partial sequence | NR_041364.1 | 1488 | 354 | 775 | 28.36 | 708.4 | 0 | 409/422 | 96 | 0/422 | 0 | Plus | Plus | Bacteria; Bacteroidetes; Bacteroidia; Bacteroidales; Prevotellaceae; Prevotella; Prevotella stercorea | Prevotella stercorea | Prevotella | Prevotellaceae | Bacteroidales | Bacteroidia | Bacteroidetes | Bacteria |
| ASV2763 | 405 | 1 | 1 | 405 | 100 | NR_159227.1 Monoglobus pectinilyticus strain 14 16S ribosomal RNA, partial sequence | NR_159227.1 | 1493 | 368 | 769 | 26.93 | 501.6 | 1E-141 | 363/407 | 89 | 7/407 | 1 | Plus | Plus | Bacteria; Firmicutes; Clostridia; Eubacteriales; Oscillospiraceae; Monoglobus; Monoglobus pectinilyticus | Monoglobus pectinilyticus | Monoglobus | Oscillospiraceae | Eubacteriales | Clostridia | Firmicutes | Bacteria |
| ASV2764 | 422 | 1 | 1 | 422 | 100 | NR_146018.1 Paludibacter jiangxiensis strain NM7 16S ribosomal RNA, partial sequence | NR_146018.1 | 1469 | 340 | 761 | 28.73 | 492.3 | 8E-139 | 374/426 | 87 | 8/426 | 1 | Plus | Plus | Bacteria; Bacteroidetes; Bacteroidia; Bacteroidales; Paludibacteraceae; Paludibacter; Paludibacter jiangxiensis | Paludibacter jiangxiensis | Paludibacter | Paludibacteraceae | Bacteroidales | Bacteroidia | Bacteroidetes | Bacteria |
| ASV2765 | 427 | 4 | 1 | 422 | 98.83 | NR_042802.1 Lactobacillus ultunensis strain Kx146C1 16S ribosomal RNA, partial sequence | NR_042802.1 | 1550 | 370 | 791 | 27.23 | 725 | 0 | 412/422 | 97 | 0/422 | 0 | Plus | Plus | Bacteria; Firmicutes; Bacilli; Lactobacillales; Lactobacillaceae; Lactobacillus; Lactobacillus ultunensis | Lactobacillus ultunensis | Lactobacillus | Lactobacillaceae | Lactobacillales | Bacilli | Firmicutes | Bacteria |
| ASV2766 | 422 | 2 | 1 | 422 | 100 | NR_074376.1 Parabacteroides distasonis ATCC 8503 16S ribosomal RNA, partial sequence | NR_074376.1 | 1497 | 368 | 789 | 28.19 | 542.2 | 8E-154 | 381/424 | 89 | 4/424 | 0 | Plus | Plus | Bacteria; Bacteroidetes; Bacteroidia; Bacteroidales; Tannerellaceae; Parabacteroides; Parabacteroides distasonis | Parabacteroides distasonis | Parabacteroides | Tannerellaceae | Bacteroidales | Bacteroidia | Bacteroidetes | Bacteria |
| ASV2767 | 402 | 1 | 1 | 402 | 100 | NR_026204.1 [Clostridium] viride strain T2-7 16S ribosomal RNA, partial sequence | NR_026204.1 | 1482 | 345 | 749 | 27.33 | 547.7 | 2E-155 | 369/405 | 91 | 3/405 | 0 | Plus | Plus | Bacteria; Firmicutes; Clostridia; Eubacteriales; Oscillospiraceae; ; [Clostridium] viride | [Clostridium] viride | | Oscillospiraceae | Eubacteriales | Clostridia | Firmicutes | Bacteria |
| ASV2768 | 422 | 1 | 1 | 422 | 100 | NR_024815.1 Prevotella shahii strain EHS11 16S ribosomal RNA, partial sequence | NR_024815.1 | 1490 | 356 | 777 | 28.32 | 603.1 | 3E-172 | 392/424 | 92 | 4/424 | 0 | Plus | Plus | Bacteria; Bacteroidetes; Bacteroidia; Bacteroidales; Prevotellaceae; Prevotella; Prevotella shahii | Prevotella shahii | | Prevotellaceae | Bacteroidales | Bacteroidia | Bacteroidetes | Bacteria |
| ASV2769 | 428 | 5 | 1 | 428 | 100 | NR_042052.1 Streptococcus equinus ATCC 9812 16S ribosomal RNA, partial sequence | NR_042052.1 | 1433 | 260 | 686 | 29.8 | 484.9 | 1E-136 | 374/429 | 87 | 3/429 | 0 | Plus | Plus | Bacteria; Firmicutes; Bacilli; Lactobacillales; Streptococcaceae; Streptococcus; Streptococcus equinus | Streptococcus equinus | Streptococcus | Streptococcaceae | Lactobacillales | Bacilli | Firmicutes | Bacteria |
| ASV2770 | 403 | 1 | 1 | 403 | 100 | NR_041497.1 Ruminiclostridium sufflavum strain CDT-1 16S ribosomal RNA, partial sequence | NR_041497.1 | 1436 | 310 | 714 | 28.2 | 597.6 | 2E-170 | 378/405 | 93 | 2/405 | 0 | Plus | Plus | Bacteria; Firmicutes; Clostridia; Eubacteriales; Oscillospiraceae; Ruminiclostridium; Ruminiclostridium sufflavum | Ruminiclostridium sufflavum | Ruminiclostridium | Oscillospiraceae | Eubacteriales | Clostridia | Firmicutes | Bacteria |
| ASV2771 | 403 | 1 | 1 | 403 | 100 | NR_148619.1 Agathobacter ruminis strain JK623 16S ribosomal RNA, partial sequence | NR_148619.1 | 1434 | 318 | 721 | 28.17 | 695.5 | 0 | 395/404 | 97 | 1/404 | 0 | Plus | Plus | Bacteria; Firmicutes; Clostridia; Eubacteriales; Lachnospiraceae; Agathobacter; Agathobacter ruminis | Agathobacter ruminis | Agathobacter | Lachnospiraceae | Eubacteriales | Clostridia | Firmicutes | Bacteria |
| ASV2773 | 422 | 1 | 1 | 422 | 100 | NR_109628.1 Prevotella jejuni strain CD3:28 16S ribosomal RNA, partial sequence | NR_109628.1 | 1484 | 339 | 760 | 28.44 | 575.4 | 7E-164 | 385/422 | 91 | 0/422 | 0 | Plus | Plus | Bacteria; Bacteroidetes; Bacteroidia; Bacteroidales; Prevotellaceae; Prevotella; Prevotella jejuni | Prevotella jejuni | Prevotella | Prevotellaceae | Bacteroidales | Bacteroidia | Bacteroidetes | Bacteria |
| ASV2774 | 405 | 1 | 1 | 405 | 100 | NR_044503.1 Thermotalea metallivorans strain B2-1 16S ribosomal RNA, partial sequence | NR_044503.1 | 1627 | 467 | 868 | 24.71 | 457.2 | 3E-128 | 356/408 | 87 | 9/408 | 2 | Plus | Plus | Bacteria; Firmicutes; Clostridia; Eubacteriales; Clostridiaceae; Thermotalea; Thermotalea metallivorans | Thermotalea metallivorans | Thermotalea | Clostridiaceae | Eubacteriales | Clostridia | Firmicutes | Bacteria |
| ASV2775 | 427 | 1 | 1 | 427 | 100 | NR_042649.1 Streptococcus plurextorum strain 1956-02 16S ribosomal RNA, partial sequence | NR_042649.1 | 1451 | 345 | 771 | 29.43 | 784.1 | 0 | 426/427 | 99 | 0/427 | 0 | Plus | Plus | Bacteria; Firmicutes; Bacilli; Lactobacillales; Streptococcaceae; Streptococcus; Streptococcus plurextorum | Streptococcus plurextorum | Streptococcus | Streptococcaceae | Lactobacillales | Bacilli | Firmicutes | Bacteria |
| ASV2776 | 402 | 1 | 1 | 402 | 100 | NR_118673.1 [Eubacterium] hallii strain ATCC 27751 16S ribosomal RNA, partial sequence | NR_118673.1 | 1479 | 371 | 772 | 27.18 | 544 | 2E-154 | 365/402 | 90 | 0/402 | 0 | Plus | Plus | Bacteria; Firmicutes; Clostridia; Eubacteriales; Lachnospiraceae; Anaerobutyricum; Anaerobutyricum hallii | Anaerobutyricum hallii | Anaerobutyricum | Lachnospiraceae | Eubacteriales | Clostridia | Firmicutes | Bacteria |
| ASV2777 | 402 | 5 | 1 | 402 | 100 | NR_042200.1 [Clostridium] asparagiforme strain N6 16S ribosomal RNA, partial sequence | NR_042200.1 | 1468 | 335 | 736 | 27.38 | 677 | 0 | 390/402 | 97 | 0/402 | 0 | Plus | Plus | Bacteria; Firmicutes; Clostridia; Eubacteriales; Lachnospiraceae; Enterocloster; Enterocloster asparagiformis | Enterocloster asparagiformis | Enterocloster | Lachnospiraceae | Eubacteriales | Clostridia | Firmicutes | Bacteria |
| ASV2778 | 402 | 1 | 1 | 402 | 100 | NR_159261.1 Bifidobacterium callitrichidarum strain TRI 5 16S ribosomal RNA, partial sequence | NR_159261.1 | 1374 | 279 | 686 | 29.69 | 538.5 | 9E-153 | 370/408 | 90 | 6/408 | 1 | Plus | Plus | Bacteria; Actinobacteria; Actinomycetia; Bifidobacteriales; Bifidobacteriaceae; Bifidobacterium; Bifidobacterium callitrichidarum | Bifidobacterium callitrichidarum | Bifidobacterium | Bifidobacteriaceae | Bifidobacteriales | Actinomycetia | Actinobacteria | Bacteria |
| ASV2779 | 425 | 1 | 1 | 425 | 100 | NR_029325.1 Schwartzia succinivorans strain S1-1 16S ribosomal RNA, partial sequence | NR_029325.1 | 1489 | 344 | 769 | 28.61 | 464.6 | 2E-130 | 371/429 | 86 | 7/429 | 1 | Plus | Plus | Bacteria; Firmicutes; Negativicutes; Selenomonadales; Selenomonadaceae; Schwartzia; Schwartzia succinivorans | Schwartzia succinivorans | Schwartzia | Selenomonadaceae | Selenomonadales | Negativicutes | Firmicutes | Bacteria |
| ASV2780 | 427 | 4 | 1 | 427 | 100 | NR_042802.1 Lactobacillus ultunensis strain Kx146C1 16S ribosomal RNA, partial sequence | NR_042802.1 | 1550 | 370 | 796 | 27.55 | 756.4 | 0 | 421/427 | 98 | 0/427 | 0 | Plus | Plus | Bacteria; Firmicutes; Bacilli; Lactobacillales; Lactobacillaceae; Lactobacillus; Lactobacillus ultunensis | Lactobacillus ultunensis | Lactobacillus | Lactobacillaceae | Lactobacillales | Bacilli | Firmicutes | Bacteria |
| ASV2781 | 402 | 1 | 1 | 402 | 100 | NR_026204.1 [Clostridium] viride strain T2-7 16S ribosomal RNA, partial sequence | NR_026204.1 | 1482 | 345 | 749 | 27.33 | 653 | 0 | 388/405 | 95 | 3/405 | 0 | Plus | Plus | Bacteria; Firmicutes; Clostridia; Eubacteriales; Oscillospiraceae; ; [Clostridium] viride | [Clostridium] viride | | Oscillospiraceae | Eubacteriales | Clostridia | Firmicutes | Bacteria |
| ASV2782 | 405 | 1 | 1 | 405 | 100 | NR_113170.1 Oligosphaera ethanolica strain 8KG-4 16S ribosomal RNA, partial sequence | NR_113170.1 | 1434 | 320 | 724 | 28.24 | 521.9 | 9E-148 | 365/406 | 89 | 2/406 | 0 | Plus | Plus | Bacteria; Lentisphaerae; Oligosphaeria; Oligosphaerales; Oligosphaeraceae; Oligosphaera; Oligosphaera ethanolica | Oligosphaera ethanolica | Oligosphaera | Oligosphaeraceae | Oligosphaerales | Oligosphaeria | Lentisphaerae | Bacteria |
| ASV2783 | 404 | 2 | 1 | 404 | 100 | NR_117718.1 Desulfotomaculum nigrificans strain NCIMB 8395 16S ribosomal RNA, partial sequence | NR_117718.1 | 1521 | 352 | 756 | 26.63 | 431.4 | 2E-120 | 349/406 | 85 | 3/406 | 0 | Plus | Plus | Bacteria; Firmicutes; Clostridia; Eubacteriales; Peptococcaceae; Desulfotomaculum; Desulfotomaculum nigrificans | Desulfotomaculum nigrificans | Desulfotomaculum | Peptococcaceae | Eubacteriales | Clostridia | Firmicutes | Bacteria |
| ASV2784 | 402 | 1 | 1 | 402 | 100 | NR_029239.3 Clostridium chartatabidum strain 163 16S ribosomal RNA, partial sequence | NR_029239.3 | 1460 | 323 | 724 | 27.53 | 704.7 | 0 | 395/402 | 98 | 0/402 | 0 | Plus | Plus | Bacteria; Firmicutes; Clostridia; Eubacteriales; Clostridiaceae; Clostridium; Clostridium chartatabidum | Clostridium chartatabidum | Clostridium | Clostridiaceae | Eubacteriales | Clostridia | Firmicutes | Bacteria |
| ASV2785 | 403 | 1 | 3 | 403 | 99.5 | NR_125464.1 Anaerobacterium chartisolvens strain T-1-35 16S ribosomal RNA, partial sequence | NR_125464.1 | 1451 | 344 | 745 | 27.71 | 475.7 | 7E-134 | 354/402 | 88 | 1/402 | 0 | Plus | Plus | Bacteria; Firmicutes; Clostridia; Eubacteriales; Oscillospiraceae; Anaerobacterium; Anaerobacterium chartisolvens | Anaerobacterium chartisolvens | Anaerobacterium | Oscillospiraceae | Eubacteriales | Clostridia | Firmicutes | Bacteria |
| ASV2786 | 405 | 1 | 1 | 405 | 100 | NR_118554.1 Intestinimonas butyriciproducens strain SRB-521-5-I 16S ribosomal RNA, partial sequence | NR_118554.1 | 1475 | 346 | 750 | 27.46 | 605 | 9E-173 | 379/405 | 93 | 0/405 | 0 | Plus | Plus | Bacteria; Firmicutes; Clostridia; Eubacteriales; ; Intestinimonas; Intestinimonas butyriciproducens | Intestinimonas butyriciproducens | Intestinimonas | | Eubacteriales | Clostridia | Firmicutes | Bacteria |
| ASV2787 | 404 | 1 | 1 | 404 | 100 | NR_118156.1 Oscillibacter ruminantium GH1 16S ribosomal RNA, partial sequence | NR_118156.1 | 1409 | 321 | 724 | 28.67 | 669.6 | 0 | 392/406 | 96 | 4/406 | 0 | Plus | Plus | Bacteria; Firmicutes; Clostridia; Eubacteriales; Oscillospiraceae; Oscillibacter; Oscillibacter ruminantium | Oscillibacter ruminantium | Oscillibacter | Oscillospiraceae | Eubacteriales | Clostridia | Firmicutes | Bacteria |
| ASV2789 | 402 | 1 | 1 | 402 | 100 | NR_102987.1 Hungateiclostridium clariflavum DSM 19732 16S ribosomal RNA, partial sequence | NR_102987.1 | 1634 | 481 | 882 | 24.6 | 527.4 | 2E-149 | 364/403 | 90 | 2/403 | 0 | Plus | Plus | Bacteria; Firmicutes; Clostridia; Eubacteriales; Oscillospiraceae; Acetivibrio; Acetivibrio clariflavus | Acetivibrio clariflavus | Acetivibrio | Oscillospiraceae | Eubacteriales | Clostridia | Firmicutes | Bacteria |
| ASV2790 | 422 | 2 | 1 | 422 | 100 | NR_040866.1 Bacteroides uniformis strain JCM 5828 16S ribosomal RNA, partial sequence | NR_040866.1 | 1425 | 351 | 772 | 29.61 | 774.9 | 0 | 421/422 | 99 | 0/422 | 0 | Plus | Plus | Bacteria; Bacteroidetes; Bacteroidia; Bacteroidales; Bacteroidaceae; Bacteroides; Bacteroides uniformis | Bacteroides uniformis | Bacteroides | Bacteroidaceae | Bacteroidales | Bacteroidia | Bacteroidetes | Bacteria |
| ASV2791 | 427 | 1 | 1 | 427 | 100 | NR_026450.1 Ruminobacter amylophilus strain H 18 16S ribosomal RNA, complete sequence | NR_026450.1 | 1539 | 351 | 777 | 27.75 | 723.2 | 0 | 416/428 | 97 | 2/428 | 0 | Plus | Plus | Bacteria; Proteobacteria; Gammaproteobacteria; Aeromonadales; Succinivibrionaceae; Ruminobacter; Ruminobacter amylophilus | Ruminobacter amylophilus | Ruminobacter | Succinivibrionaceae | Aeromonadales | Gammaproteobacteria | Proteobacteria | Bacteria |
| ASV2792 | 405 | 1 | 1 | 405 | 100 | NR_118554.1 Intestinimonas butyriciproducens strain SRB-521-5-I 16S ribosomal RNA, partial sequence | NR_118554.1 | 1475 | 346 | 750 | 27.46 | 749 | 0 | 405/405 | 100 | 0/405 | 0 | Plus | Plus | Bacteria; Firmicutes; Clostridia; Eubacteriales; ; Intestinimonas; Intestinimonas butyriciproducens | Intestinimonas butyriciproducens | Intestinimonas | | Eubacteriales | Clostridia | Firmicutes | Bacteria |
| ASV2793 | 402 | 1 | 1 | 402 | 100 | NR_026074.1 Helicobacter rodentium strain MIT 95-1707 16S ribosomal RNA, partial sequence | NR_026074.1 | 1423 | 317 | 718 | 28.25 | 737.9 | 0 | 401/402 | 99 | 0/402 | 0 | Plus | Plus | Bacteria; Proteobacteria; Epsilonproteobacteria; Campylobacterales; Helicobacteraceae; Helicobacter; Helicobacter rodentium | Helicobacter rodentium | Helicobacter | Helicobacteraceae | Campylobacterales | Epsilonproteobacteria | Proteobacteria | Bacteria |
| ASV2794 | 422 | 2 | 1 | 422 | 100 | NR_074376.1 Parabacteroides distasonis ATCC 8503 16S ribosomal RNA, partial sequence | NR_074376.1 | 1497 | 368 | 789 | 28.19 | 564.3 | 2E-160 | 383/422 | 90 | 0/422 | 0 | Plus | Plus | Bacteria; Bacteroidetes; Bacteroidia; Bacteroidales; Tannerellaceae; Parabacteroides; Parabacteroides distasonis | Parabacteroides distasonis | Parabacteroides | Tannerellaceae | Bacteroidales | Bacteroidia | Bacteroidetes | Bacteria |
| ASV2795 | 421 | 1 | 1 | 421 | 100 | NR_144616.1 Muribaculum intestinale strain YL27 16S ribosomal RNA, partial sequence | NR_144616.1 | 1460 | 297 | 718 | 28.9 | 501.6 | 1E-141 | 373/423 | 88 | 3/423 | 0 | Plus | Plus | Bacteria; Bacteroidetes; Bacteroidia; Bacteroidales; Muribaculaceae; Muribaculum; Muribaculum intestinale | Muribaculum intestinale | Muribaculum | Muribaculaceae | Bacteroidales | Bacteroidia | Bacteroidetes | Bacteria |
| ASV2796 | 427 | 1 | 1 | 427 | 100 | NR_147405.1 Mailhella massiliensis strain Marseille-P3199 16S ribosomal RNA, partial sequence | NR_147405.1 | 1511 | 355 | 781 | 28.26 | 750.9 | 0 | 420/427 | 98 | 0/427 | 0 | Plus | Plus | Bacteria; Proteobacteria; Deltaproteobacteria; Desulfovibrionales; Desulfovibrionaceae; Mailhella; Mailhella massiliensis | Mailhella massiliensis | Mailhella | Desulfovibrionaceae | Desulfovibrionales | Deltaproteobacteria | Proteobacteria | Bacteria |
| ASV2797 | 422 | 1 | 1 | 422 | 100 | NR_112943.1 Bacteroides stercoris ATCC 43183 strain JCM 9496 16S ribosomal RNA, partial sequence | NR_112943.1 | 1476 | 343 | 764 | 28.59 | 686.2 | 0 | 405/422 | 95 | 0/422 | 0 | Plus | Plus | Bacteria; Bacteroidetes; Bacteroidia; Bacteroidales; Bacteroidaceae; Bacteroides; Bacteroides stercoris | Bacteroides stercoris | Bacteroides | Bacteroidaceae | Bacteroidales | Bacteroidia | Bacteroidetes | Bacteria |
| ASV2798 | 421 | 1 | 1 | 421 | 100 | NR_041342.1 Parabacteroides distasonis strain JCM 5825 16S ribosomal RNA, partial sequence | NR_041342.1 | 1488 | 353 | 774 | 28.36 | 457.2 | 3E-128 | 368/426 | 86 | 9/426 | 2 | Plus | Plus | Bacteria; Bacteroidetes; Bacteroidia; Bacteroidales; Tannerellaceae; Parabacteroides; Parabacteroides distasonis | Parabacteroides distasonis | Parabacteroides | Tannerellaceae | Bacteroidales | Bacteroidia | Bacteroidetes | Bacteria |
| ASV2800 | 422 | 2 | 1 | 422 | 100 | NR_113119.1 Prevotella oris strain JCM 8540 16S ribosomal RNA, partial sequence | NR_113119.1 | 1481 | 345 | 766 | 28.49 | 536.6 | 4E-152 | 379/423 | 89 | 2/423 | 0 | Plus | Plus | Bacteria; Bacteroidetes; Bacteroidia; Bacteroidales; Prevotellaceae; Prevotella; Prevotella oris | Prevotella oris | Prevotella | Prevotellaceae | Bacteroidales | Bacteroidia | Bacteroidetes | Bacteria |
| ASV2801 | 427 | 2 | 1 | 427 | 100 | NR_113306.1 Megasphaera elsdenii DSM 20460 16S ribosomal RNA, partial sequence | NR_113306.1 | 1174 | 232 | 658 | 36.37 | 629 | 6E-180 | 398/427 | 93 | 0/427 | 0 | Plus | Plus | Bacteria; Firmicutes; Negativicutes; Veillonellales; Veillonellaceae; Megasphaera; Megasphaera elsdenii | Megasphaera elsdenii | Megasphaera | Veillonellaceae | Veillonellales | Negativicutes | Firmicutes | Bacteria |
| ASV2802 | 427 | 1 | 1 | 426 | 99.77 | NR_027596.1 Mitsuokella multacida strain DSM 20544 16S ribosomal RNA, partial sequence | NR_027596.1 | 1511 | 346 | 771 | 28.19 | 662.2 | 0 | 404/427 | 94 | 2/427 | 0 | Plus | Plus | Bacteria; Firmicutes; Negativicutes; Selenomonadales; Selenomonadaceae; Mitsuokella; Mitsuokella multacida | Mitsuokella multacida | Mitsuokella | Selenomonadaceae | Selenomonadales | Negativicutes | Firmicutes | Bacteria |
| ASV2803 | 402 | 1 | 1 | 402 | 100 | NR_118670.1 Eubacterium ventriosum strain ATCC 27560 16S ribosomal RNA, partial sequence | NR_118670.1 | 1481 | 374 | 775 | 27.14 | 695.5 | 0 | 392/402 | 97 | 0/402 | 0 | Plus | Plus | Bacteria; Firmicutes; Clostridia; Eubacteriales; Eubacteriaceae; Eubacterium; Eubacterium ventriosum | Eubacterium ventriosum | Eubacterium | Eubacteriaceae | Eubacteriales | Clostridia | Firmicutes | Bacteria |
| ASV2804 | 401 | 1 | 1 | 401 | 100 | NR_042930.1 Acetanaerobacterium elongatum strain Z7 16S ribosomal RNA, partial sequence | NR_042930.1 | 1533 | 383 | 783 | 26.16 | 581 | 2E-165 | 372/401 | 92 | 0/401 | 0 | Plus | Plus | Bacteria; Firmicutes; Clostridia; Eubacteriales; Oscillospiraceae; Acetanaerobacterium; Acetanaerobacterium elongatum | Acetanaerobacterium elongatum | Acetanaerobacterium | Oscillospiraceae | Eubacteriales | Clostridia | Firmicutes | Bacteria |
| ASV2805 | 405 | 2 | 3 | 405 | 99.51 | NG_041947.1 Ruminiclostridium cellulolyticum H10 16S ribosomal RNA gene, complete sequence | NG_041947.1 | 1641 | 488 | 890 | 24.56 | 479.4 | 6E-135 | 355/403 | 88 | 0/403 | 0 | Plus | Plus | Bacteria; Firmicutes; Clostridia; Eubacteriales; Oscillospiraceae; Ruminiclostridium; Ruminiclostridium cellulolyticum | Ruminiclostridium cellulolyticum | Ruminiclostridium | Oscillospiraceae | Eubacteriales | Clostridia | Firmicutes | Bacteria |
| ASV2808 | 418 | 2 | 1 | 418 | 100 | NR_114069.1 Zoogloea oryzae strain NBRC 102407 16S ribosomal RNA, partial sequence | NR_114069.1 | 1463 | 325 | 751 | 29.19 | 431.4 | 2E-120 | 366/429 | 85 | 13/429 | 3 | Plus | Plus | Bacteria; Proteobacteria; Betaproteobacteria; Rhodocyclales; Zoogloeaceae; Zoogloea; Zoogloea oryzae | Zoogloea oryzae | Zoogloea | Zoogloeaceae | Rhodocyclales | Betaproteobacteria | Proteobacteria | Bacteria |
| ASV2810 | 405 | 1 | 1 | 405 | 100 | NR_144611.1 Flintibacter butyricus strain BLS21 16S ribosomal RNA, partial sequence | NR_144611.1 | 808 | 299 | 703 | 50.12 | 582.8 | 4E-166 | 375/405 | 92 | 0/405 | 0 | Plus | Plus | Bacteria; Firmicutes; Clostridia; Eubacteriales; ; Flintibacter; Flintibacter butyricus | Flintibacter butyricus | Flintibacter | | Eubacteriales | Clostridia | Firmicutes | Bacteria |
| ASV2811 | 421 | 4 | 1 | 421 | 100 | NR_117779.1 Anaerorhabdus furcosa 16S ribosomal RNA, partial sequence | NR_117779.1 | 1488 | 350 | 770 | 28.29 | 562.5 | 6E-160 | 383/422 | 90 | 2/422 | 0 | Plus | Plus | Bacteria; Firmicutes; Erysipelotrichia; Erysipelotrichales; Erysipelotrichaceae; Anaerorhabdus; Anaerorhabdus furcosa | Anaerorhabdus furcosa | Anaerorhabdus | Erysipelotrichaceae | Erysipelotrichales | Erysipelotrichia | Firmicutes | Bacteria |
| ASV2813 | 421 | 2 | 1 | 421 | 100 | NR_113073.1 Barnesiella intestinihominis strain JCM 15079 16S ribosomal RNA, partial sequence | NR_113073.1 | 1481 | 346 | 767 | 28.49 | 512.6 | 6E-145 | 377/425 | 88 | 7/425 | 1 | Plus | Plus | Bacteria; Bacteroidetes; Bacteroidia; Bacteroidales; Barnesiellaceae; Barnesiella; Barnesiella intestinihominis | Barnesiella intestinihominis | Barnesiella | Barnesiellaceae | Bacteroidales | Bacteroidia | Bacteroidetes | Bacteria |
| ASV2814 | 401 | 1 | 1 | 401 | 100 | NR_137408.1 Terrisporobacter petrolearius strain LAM0A37 16S ribosomal RNA, partial sequence | NR_137408.1 | 1405 | 307 | 707 | 28.54 | 708.4 | 0 | 395/401 | 98 | 0/401 | 0 | Plus | Plus | Bacteria; Firmicutes; Clostridia; Eubacteriales; Peptostreptococcaceae; Terrisporobacter; Terrisporobacter petrolearius | Terrisporobacter petrolearius | Terrisporobacter | Peptostreptococcaceae | Eubacteriales | Clostridia | Firmicutes | Bacteria |
| ASV2815 | 417 | 1 | 1 | 417 | 100 | NR_144740.1 Romboutsia timonensis strain DR1 16S ribosomal RNA, partial sequence | NR_144740.1 | 1466 | 338 | 738 | 27.35 | 562.5 | 6E-160 | 382/417 | 91 | 16/417 | 3 | Plus | Plus | Bacteria; Firmicutes; Clostridia; Eubacteriales; Peptostreptococcaceae; Romboutsia; Romboutsia timonensis | Romboutsia timonensis | Romboutsia | Peptostreptococcaceae | Eubacteriales | Clostridia | Firmicutes | Bacteria |
| ASV2816 | 422 | 1 | 1 | 422 | 100 | NR_041464.1 Parabacteroides johnsonii strain M-165 16S ribosomal RNA, partial sequence | NR_041464.1 | 1492 | 357 | 778 | 28.28 | 675.1 | 0 | 403/422 | 95 | 0/422 | 0 | Plus | Plus | Bacteria; Bacteroidetes; Bacteroidia; Bacteroidales; Tannerellaceae; Parabacteroides; Parabacteroides johnsonii | Parabacteroides johnsonii | Parabacteroides | Tannerellaceae | Bacteroidales | Bacteroidia | Bacteroidetes | Bacteria |
| ASV2817 | 404 | 1 | 1 | 404 | 100 | NR_118156.1 Oscillibacter ruminantium GH1 16S ribosomal RNA, partial sequence | NR_118156.1 | 1409 | 321 | 724 | 28.67 | 647.4 | 0 | 386/404 | 95 | 0/404 | 0 | Plus | Plus | Bacteria; Firmicutes; Clostridia; Eubacteriales; Oscillospiraceae; Oscillibacter; Oscillibacter ruminantium | Oscillibacter ruminantium | Oscillibacter | Oscillospiraceae | Eubacteriales | Clostridia | Firmicutes | Bacteria |
| ASV2818 | 421 | 2 | 1 | 421 | 100 | NR_113077.1 Paraprevotella clara strain JCM 14859 16S ribosomal RNA, partial sequence | NR_113077.1 | 1480 | 346 | 766 | 28.45 | 507.1 | 3E-143 | 373/422 | 88 | 2/422 | 0 | Plus | Plus | Bacteria; Bacteroidetes; Bacteroidia; Bacteroidales; Prevotellaceae; Paraprevotella; Paraprevotella clara | Paraprevotella clara | Paraprevotella | Prevotellaceae | Bacteroidales | Bacteroidia | Bacteroidetes | Bacteria |
| ASV2820 | 422 | 1 | 1 | 422 | 100 | NR_041342.1 Parabacteroides distasonis strain JCM 5825 16S ribosomal RNA, partial sequence | NR_041342.1 | 1488 | 353 | 774 | 28.36 | 564.3 | 2E-160 | 383/422 | 90 | 0/422 | 0 | Plus | Plus | Bacteria; Bacteroidetes; Bacteroidia; Bacteroidales; Tannerellaceae; Parabacteroides; Parabacteroides distasonis | Parabacteroides distasonis | Parabacteroides | Tannerellaceae | Bacteroidales | Bacteroidia | Bacteroidetes | Bacteria |
| ASV2821 | 427 | 1 | 1 | 427 | 100 | NR_041667.1 Parasutterella excrementihominis strain YIT 11859 16S ribosomal RNA, partial sequence | NR_041667.1 | 1494 | 337 | 763 | 28.58 | 778.6 | 0 | 425/427 | 99 | 0/427 | 0 | Plus | Plus | Bacteria; Proteobacteria; Betaproteobacteria; Burkholderiales; Sutterellaceae; Parasutterella; Parasutterella excrementihominis | Parasutterella excrementihominis | Parasutterella | Sutterellaceae | Burkholderiales | Betaproteobacteria | Proteobacteria | Bacteria |
| ASV2822 | 402 | 1 | 1 | 402 | 100 | NR_144612.1 Frisingicoccus caecimuris strain PG-426-CC-1 16S ribosomal RNA, partial sequence | NR_144612.1 | 1448 | 367 | 768 | 27.76 | 665.9 | 0 | 388/402 | 96 | 0/402 | 0 | Plus | Plus | Bacteria; Firmicutes; Clostridia; Eubacteriales; Lachnospiraceae; Frisingicoccus; Frisingicoccus caecimuris | Frisingicoccus caecimuris | Frisingicoccus | Lachnospiraceae | Eubacteriales | Clostridia | Firmicutes | Bacteria |
| ASV2824 | 405 | 1 | 1 | 405 | 100 | NR_044972.1 Sporobacter termitidis strain SYR 16S ribosomal RNA, partial sequence | NR_044972.1 | 1506 | 348 | 752 | 26.89 | 564.3 | 2E-160 | 371/405 | 91 | 0/405 | 0 | Plus | Plus | Bacteria; Firmicutes; Clostridia; Eubacteriales; Oscillospiraceae; Sporobacter; Sporobacter termitidis | Sporobacter termitidis | Sporobacter | Oscillospiraceae | Eubacteriales | Clostridia | Firmicutes | Bacteria |
| ASV2825 | 405 | 1 | 1 | 405 | 100 | NR_044972.1 Sporobacter termitidis strain SYR 16S ribosomal RNA, partial sequence | NR_044972.1 | 1506 | 348 | 752 | 26.89 | 592 | 7E-169 | 377/406 | 92 | 2/406 | 0 | Plus | Plus | Bacteria; Firmicutes; Clostridia; Eubacteriales; Oscillospiraceae; Sporobacter; Sporobacter termitidis | Sporobacter termitidis | Sporobacter | Oscillospiraceae | Eubacteriales | Clostridia | Firmicutes | Bacteria |
| ASV2826 | 405 | 1 | 1 | 405 | 100 | NR_144611.1 Flintibacter butyricus strain BLS21 16S ribosomal RNA, partial sequence | NR_144611.1 | 808 | 299 | 703 | 50.12 | 610.5 | 2E-174 | 380/405 | 93 | 0/405 | 0 | Plus | Plus | Bacteria; Firmicutes; Clostridia; Eubacteriales; ; Flintibacter; Flintibacter butyricus | Flintibacter butyricus | Flintibacter | | Eubacteriales | Clostridia | Firmicutes | Bacteria |
| ASV2827 | 402 | 2 | 1 | 402 | 100 | NR_024661.1 Eubacterium ruminantium strain GA195 16S ribosomal RNA, partial sequence | NR_024661.1 | 1508 | 378 | 779 | 26.66 | 477.6 | 2E-134 | 354/402 | 88 | 0/402 | 0 | Plus | Plus | Bacteria; Firmicutes; Clostridia; Eubacteriales; Eubacteriaceae; Eubacterium; Eubacterium ruminantium | Eubacterium ruminantium | Eubacterium | Eubacteriaceae | Eubacteriales | Clostridia | Firmicutes | Bacteria |
| ASV2828 | 401 | 1 | 1 | 401 | 100 | NR_043551.1 Lactonifactor longoviformis strain ED-Mt61/PYG-s6 16S ribosomal RNA, partial sequence | NR_043551.1 | 1438 | 366 | 767 | 27.96 | 448 | 2E-125 | 352/405 | 86 | 7/405 | 1 | Plus | Plus | Bacteria; Firmicutes; Clostridia; Eubacteriales; Clostridiaceae; Lactonifactor; Lactonifactor longoviformis | Lactonifactor longoviformis | Lactonifactor | Clostridiaceae | Eubacteriales | Clostridia | Firmicutes | Bacteria |
| ASV2830 | 427 | 1 | 1 | 426 | 99.77 | NR_041666.1 Dialister succinatiphilus YIT 11850 16S ribosomal RNA, partial sequence | NR_041666.1 | 1521 | 353 | 778 | 28.01 | 671.5 | 0 | 405/426 | 95 | 0/426 | 0 | Plus | Plus | Bacteria; Firmicutes; Negativicutes; Veillonellales; Veillonellaceae; Dialister; Dialister succinatiphilus | Dialister succinatiphilus | Dialister | Veillonellaceae | Veillonellales | Negativicutes | Firmicutes | Bacteria |
| ASV2831 | 403 | 1 | 1 | 403 | 100 | NR_147375.1 Colidextribacter massiliensis strain Marseille-P3083 16S ribosomal RNA, partial sequence | NR_147375.1 | 1487 | 364 | 765 | 27.03 | 505.3 | 9E-143 | 361/404 | 89 | 3/404 | 0 | Plus | Plus | Bacteria; Firmicutes; Clostridia; Eubacteriales; ; Colidextribacter; Colidextribacter massiliensis | Colidextribacter massiliensis | Colidextribacter | | Eubacteriales | Clostridia | Firmicutes | Bacteria |
| ASV2832 | 404 | 1 | 1 | 404 | 100 | NR_144611.1 Flintibacter butyricus strain BLS21 16S ribosomal RNA, partial sequence | NR_144611.1 | 808 | 299 | 703 | 50.12 | 630.8 | 2E-180 | 384/405 | 94 | 1/405 | 0 | Plus | Plus | Bacteria; Firmicutes; Clostridia; Eubacteriales; ; Flintibacter; Flintibacter butyricus | Flintibacter butyricus | Flintibacter | | Eubacteriales | Clostridia | Firmicutes | Bacteria |
| ASV2833 | 427 | 1 | 1 | 427 | 100 | NR_113163.1 Faecalitalea cylindroides strain JCM 10261 16S ribosomal RNA, partial sequence | NR_113163.1 | 1504 | 359 | 784 | 28.32 | 638.2 | 0 | 402/429 | 93 | 5/429 | 1 | Plus | Plus | Bacteria; Firmicutes; Erysipelotrichia; Erysipelotrichales; Erysipelotrichaceae; Faecalitalea; Faecalitalea cylindroides | Faecalitalea cylindroides | Faecalitalea | Erysipelotrichaceae | Erysipelotrichales | Erysipelotrichia | Firmicutes | Bacteria |
| ASV2834 | 402 | 1 | 1 | 402 | 100 | NR_116747.1 Ruminococcus faecis JCM 15917 strain Eg2 16S ribosomal RNA, partial sequence | NR_116747.1 | 1404 | 318 | 719 | 28.63 | 671.5 | 0 | 389/402 | 96 | 0/402 | 0 | Plus | Plus | Bacteria; Firmicutes; Clostridia; Eubacteriales; Lachnospiraceae; Mediterraneibacter; Mediterraneibacter faecis | Mediterraneibacter faecis | Mediterraneibacter | Lachnospiraceae | Eubacteriales | Clostridia | Firmicutes | Bacteria |
| ASV2835 | 405 | 2 | 1 | 405 | 100 | NR_025352.1 Clostridium akagii strain CK58 16S ribosomal RNA, partial sequence | NR_025352.1 | 1492 | 339 | 740 | 26.94 | 545.9 | 6E-155 | 371/407 | 91 | 7/407 | 1 | Plus | Plus | Bacteria; Firmicutes; Clostridia; Eubacteriales; Clostridiaceae; Clostridium; Clostridium akagii | Clostridium akagii | Clostridium | Clostridiaceae | Eubacteriales | Clostridia | Firmicutes | Bacteria |
| ASV2836 | 421 | 2 | 1 | 421 | 100 | NR_113073.1 Barnesiella intestinihominis strain JCM 15079 16S ribosomal RNA, partial sequence | NR_113073.1 | 1481 | 346 | 767 | 28.49 | 496 | 6E-140 | 372/423 | 87 | 3/423 | 0 | Plus | Plus | Bacteria; Bacteroidetes; Bacteroidia; Bacteroidales; Barnesiellaceae; Barnesiella; Barnesiella intestinihominis | Barnesiella intestinihominis | Barnesiella | Barnesiellaceae | Bacteroidales | Bacteroidia | Bacteroidetes | Bacteria |
| ASV2837 | 405 | 5 | 1 | 405 | 100 | NR_115693.1 Gracilibacter thermotolerans JW/YJL-S1 16S ribosomal RNA, partial sequence | NR_115693.1 | 1526 | 459 | 861 | 26.41 | 508.9 | 7E-144 | 365/408 | 89 | 8/408 | 1 | Plus | Plus | Bacteria; Firmicutes; Clostridia; Eubacteriales; Gracilibacteraceae; Gracilibacter; Gracilibacter thermotolerans | Gracilibacter thermotolerans | Gracilibacter | Gracilibacteraceae | Eubacteriales | Clostridia | Firmicutes | Bacteria |
| ASV2838 | 422 | 1 | 1 | 422 | 100 | NR_113208.1 Parabacteroides chinchillae strain JCM 17104 16S ribosomal RNA, partial sequence | NR_113208.1 | 1489 | 353 | 774 | 28.34 | 647.4 | 0 | 399/423 | 94 | 2/423 | 0 | Plus | Plus | Bacteria; Bacteroidetes; Bacteroidia; Bacteroidales; Tannerellaceae; Parabacteroides; Parabacteroides chinchillae | Parabacteroides chinchillae | Parabacteroides | Tannerellaceae | Bacteroidales | Bacteroidia | Bacteroidetes | Bacteria |
| ASV2839 | 402 | 5 | 3 | 402 | 99.5 | NR_118511.1 Campylobacter coli strain ATCC 33559 16S ribosomal RNA, partial sequence | NR_118511.1 | 1435 | 331 | 731 | 27.94 | 590.2 | 3E-168 | 377/404 | 93 | 7/404 | 1 | Plus | Plus | Bacteria; Proteobacteria; Epsilonproteobacteria; Campylobacterales; Campylobacteraceae; Campylobacter; Campylobacter coli | Campylobacter coli | Campylobacter | Campylobacteraceae | Campylobacterales | Epsilonproteobacteria | Proteobacteria | Bacteria |
| ASV2840 | 402 | 1 | 1 | 402 | 100 | NR_144727.1 Anaeromassilibacillus senegalensis strain mt9 16S ribosomal RNA, partial sequence | NR_144727.1 | 1478 | 352 | 753 | 27.2 | 627.1 | 2E-179 | 382/403 | 94 | 2/403 | 0 | Plus | Plus | Bacteria; Firmicutes; Clostridia; Eubacteriales; Oscillospiraceae; Anaeromassilibacillus; Anaeromassilibacillus senegalensis | Anaeromassilibacillus senegalensis | Anaeromassilibacillus | Oscillospiraceae | Eubacteriales | Clostridia | Firmicutes | Bacteria |
| ASV2841 | 404 | 1 | 1 | 404 | 100 | NR_027203.1 Mogibacterium neglectum strain P9a-h 16S ribosomal RNA, partial sequence | NR_027203.1 | 1478 | 362 | 765 | 27.33 | 581 | 2E-165 | 375/405 | 92 | 2/405 | 0 | Plus | Plus | Bacteria; Firmicutes; Clostridia; Eubacteriales; Clostridiales Family XIII. Incertae Sedis; Mogibacterium; Mogibacterium neglectum | Mogibacterium neglectum | Mogibacterium | Clostridiales Family XIII. Incertae Sedis | Eubacteriales | Clostridia | Firmicutes | Bacteria |
| ASV2842 | 428 | 1 | 1 | 428 | 100 | NR_028833.1 Streptococcus sinensis strain HKU4 16S ribosomal RNA, partial sequence | NR_028833.1 | 1512 | 364 | 790 | 28.24 | 473.9 | 3E-133 | 375/432 | 86 | 9/432 | 2 | Plus | Plus | Bacteria; Firmicutes; Bacilli; Lactobacillales; Streptococcaceae; Streptococcus; Streptococcus sinensis | Streptococcus sinensis | Streptococcus | Streptococcaceae | Lactobacillales | Bacilli | Firmicutes | Bacteria |
| ASV2843 | 422 | 3 | 1 | 422 | 100 | NR_113119.1 Prevotella oris strain JCM 8540 16S ribosomal RNA, partial sequence | NR_113119.1 | 1481 | 345 | 766 | 28.49 | 597.6 | 2E-170 | 391/424 | 92 | 4/424 | 0 | Plus | Plus | Bacteria; Bacteroidetes; Bacteroidia; Bacteroidales; Prevotellaceae; Prevotella; Prevotella oris | Prevotella oris | Prevotella | Prevotellaceae | Bacteroidales | Bacteroidia | Bacteroidetes | Bacteria |
| ASV2844 | 404 | 4 | 1 | 404 | 100 | NR_115307.1 Ethanoligenens harbinense YUAN-3 16S ribosomal RNA, partial sequence | NR_115307.1 | 1497 | 340 | 743 | 26.99 | 514.5 | 2E-145 | 364/406 | 89 | 4/406 | 0 | Plus | Plus | Bacteria; Firmicutes; Clostridia; Eubacteriales; Oscillospiraceae; Ethanoligenens; Ethanoligenens harbinense | Ethanoligenens harbinense | Ethanoligenens | Oscillospiraceae | Eubacteriales | Clostridia | Firmicutes | Bacteria |
| ASV2845 | 418 | 1 | 1 | 418 | 100 | NR_116008.1 Pandoraea thiooxydans strain ATSB16 16S ribosomal RNA, partial sequence | NR_116008.1 | 1410 | 307 | 733 | 30.28 | 425.9 | 8E-119 | 364/428 | 85 | 11/428 | 2 | Plus | Plus | Bacteria; Proteobacteria; Betaproteobacteria; Burkholderiales; Burkholderiaceae; Pandoraea; Pandoraea thiooxydans | Pandoraea thiooxydans | Pandoraea | Burkholderiaceae | Burkholderiales | Betaproteobacteria | Proteobacteria | Bacteria |
| ASV2846 | 403 | 1 | 1 | 403 | 100 | NR_029160.1 Ruminococcus callidus strain ATCC 27760 16S ribosomal RNA, partial sequence | NR_029160.1 | 1371 | 310 | 711 | 29.32 | 627.1 | 2E-179 | 384/405 | 94 | 5/405 | 1 | Plus | Plus | Bacteria; Firmicutes; Clostridia; Eubacteriales; Oscillospiraceae; Ruminococcus; Ruminococcus callidus | Ruminococcus callidus | Ruminococcus | Oscillospiraceae | Eubacteriales | Clostridia | Firmicutes | Bacteria |
| ASV2847 | 422 | 1 | 1 | 422 | 100 | NR_144750.1 Prevotellamassilia timonensis strain Marseille-P2831 16S ribosomal RNA, partial sequence | NR_144750.1 | 1493 | 358 | 779 | 28.27 | 536.6 | 4E-152 | 378/422 | 89 | 0/422 | 0 | Plus | Plus | Bacteria; Bacteroidetes; Bacteroidia; Bacteroidales; Prevotellaceae; Prevotellamassilia; Prevotellamassilia timonensis | Prevotellamassilia timonensis | Prevotellamassilia | Prevotellaceae | Bacteroidales | Bacteroidia | Bacteroidetes | Bacteria |
| ASV2848 | 402 | 2 | 1 | 402 | 100 | NR_117147.1 Faecalicatena contorta strain DSM 3982 16S ribosomal RNA, partial sequence | NR_117147.1 | 1524 | 363 | 764 | 26.38 | 704.7 | 0 | 395/402 | 98 | 0/402 | 0 | Plus | Plus | Bacteria; Firmicutes; Clostridia; Eubacteriales; Lachnospiraceae; Faecalicatena; Faecalicatena contorta | Faecalicatena contorta | Faecalicatena | Lachnospiraceae | Eubacteriales | Clostridia | Firmicutes | Bacteria |
| ASV2850 | 403 | 1 | 1 | 401 | 99.5 | NR_104911.1 Vampirovibrio chlorellavorus strain ICPB 3707 16S ribosomal RNA, partial sequence | NR_104911.1 | 1446 | 333 | 732 | 27.66 | 418.5 | 1E-116 | 343/401 | 85 | 1/401 | 0 | Plus | Plus | Bacteria; Candidatus Melainabacteria; ; Vampirovibrionales; ; Vampirovibrio; Vampirovibrio chlorellavorus | Vampirovibrio chlorellavorus | Vampirovibrio | | Vampirovibrionales | | Candidatus Melainabacteria | Bacteria |
| ASV2851 | 427 | 1 | 1 | 427 | 100 | NR_024994.1 Lactobacillus mucosae strain S32 16S ribosomal RNA, partial sequence | NR_024994.1 | 1568 | 384 | 810 | 27.23 | 778.6 | 0 | 425/427 | 99 | 0/427 | 0 | Plus | Plus | Bacteria; Firmicutes; Bacilli; Lactobacillales; Lactobacillaceae; Limosilactobacillus; Limosilactobacillus mucosae | Limosilactobacillus mucosae | Limosilactobacillus | Lactobacillaceae | Lactobacillales | Bacilli | Firmicutes | Bacteria |
| ASV2852 | 422 | 1 | 1 | 422 | 100 | NR_113411.1 Prevotella copri DSM 18205 strain JCM 13464 16S ribosomal RNA, partial sequence | NR_113411.1 | 1493 | 358 | 779 | 28.27 | 752.7 | 0 | 417/422 | 98 | 0/422 | 0 | Plus | Plus | Bacteria; Bacteroidetes; Bacteroidia; Bacteroidales; Prevotellaceae; Prevotella; Prevotella copri | Prevotella copri | Prevotella | Prevotellaceae | Bacteroidales | Bacteroidia | Bacteroidetes | Bacteria |
| ASV2853 | 402 | 4 | 1 | 402 | 100 | NR_074399.1 Ruminococcus albus 7 = DSM 20455 16S ribosomal RNA, partial sequence | NR_074399.1 | 1500 | 350 | 751 | 26.8 | 593.9 | 2E-169 | 375/402 | 93 | 0/402 | 0 | Plus | Plus | Bacteria; Firmicutes; Clostridia; Eubacteriales; Oscillospiraceae; Ruminococcus; Ruminococcus albus | Ruminococcus albus | Ruminococcus | Oscillospiraceae | Eubacteriales | Clostridia | Firmicutes | Bacteria |
| ASV2854 | 402 | 2 | 1 | 402 | 100 | NR_156080.1 Kineothrix alysoides strain KNHs209 16S ribosomal RNA, partial sequence | NR_156080.1 | 1410 | 353 | 754 | 28.51 | 665.9 | 0 | 388/402 | 96 | 0/402 | 0 | Plus | Plus | Bacteria; Firmicutes; Clostridia; Eubacteriales; Lachnospiraceae; Kineothrix; Kineothrix alysoides | Kineothrix alysoides | Kineothrix | Lachnospiraceae | Eubacteriales | Clostridia | Firmicutes | Bacteria |
| ASV2855 | 404 | 1 | 1 | 404 | 100 | NR_074536.1 Cellulosilyticum lentocellum DSM 5427 16S ribosomal RNA, partial sequence | NR_074536.1 | 1512 | 351 | 754 | 26.72 | 581 | 2E-165 | 374/404 | 92 | 0/404 | 0 | Plus | Plus | Bacteria; Firmicutes; Clostridia; Eubacteriales; Lachnospiraceae; Cellulosilyticum; Cellulosilyticum lentocellum | Cellulosilyticum lentocellum | Cellulosilyticum | Lachnospiraceae | Eubacteriales | Clostridia | Firmicutes | Bacteria |
| ASV2856 | 402 | 1 | 1 | 402 | 100 | NR_104783.1 Acetivibrio ethanolgignens strain 77-6 16S ribosomal RNA, partial sequence | NR_104783.1 | 1529 | 370 | 771 | 26.29 | 643.8 | 0 | 385/403 | 95 | 2/403 | 0 | Plus | Plus | Bacteria; Firmicutes; Clostridia; Eubacteriales; Oscillospiraceae; Acetivibrio; Acetivibrio ethanolgignens | Acetivibrio ethanolgignens | Acetivibrio | Oscillospiraceae | Eubacteriales | Clostridia | Firmicutes | Bacteria |
| ASV2857 | 404 | 2 | 1 | 404 | 100 | NR_117718.1 Desulfotomaculum nigrificans strain NCIMB 8395 16S ribosomal RNA, partial sequence | NR_117718.1 | 1521 | 352 | 756 | 26.63 | 431.4 | 2E-120 | 352/409 | 86 | 9/409 | 2 | Plus | Plus | Bacteria; Firmicutes; Clostridia; Eubacteriales; Peptococcaceae; Desulfotomaculum; Desulfotomaculum nigrificans | Desulfotomaculum nigrificans | Desulfotomaculum | Peptococcaceae | Eubacteriales | Clostridia | Firmicutes | Bacteria |
| ASV2858 | 403 | 1 | 1 | 403 | 100 | NR_144740.1 Romboutsia timonensis strain DR1 16S ribosomal RNA, partial sequence | NR_144740.1 | 1466 | 338 | 738 | 27.35 | 599.4 | 4E-171 | 377/403 | 93 | 2/403 | 0 | Plus | Plus | Bacteria; Firmicutes; Clostridia; Eubacteriales; Peptostreptococcaceae; Romboutsia; Romboutsia timonensis | Romboutsia timonensis | Romboutsia | Peptostreptococcaceae | Eubacteriales | Clostridia | Firmicutes | Bacteria |
| ASV2859 | 403 | 1 | 1 | 403 | 100 | NR_042832.1 Roseburia faecis strain M72/1 16S ribosomal RNA, partial sequence | NR_042832.1 | 1456 | 347 | 749 | 27.68 | 712.1 | 0 | 397/403 | 98 | 0/403 | 0 | Plus | Plus | Bacteria; Firmicutes; Clostridia; Eubacteriales; Lachnospiraceae; Roseburia; Roseburia faecis | Roseburia faecis | Roseburia | Lachnospiraceae | Eubacteriales | Clostridia | Firmicutes | Bacteria |
| ASV2860 | 404 | 1 | 1 | 404 | 100 | NR_074536.1 Cellulosilyticum lentocellum DSM 5427 16S ribosomal RNA, partial sequence | NR_074536.1 | 1512 | 351 | 754 | 26.72 | 608.7 | 7E-174 | 380/405 | 93 | 2/405 | 0 | Plus | Plus | Bacteria; Firmicutes; Clostridia; Eubacteriales; Lachnospiraceae; Cellulosilyticum; Cellulosilyticum lentocellum | Cellulosilyticum lentocellum | Cellulosilyticum | Lachnospiraceae | Eubacteriales | Clostridia | Firmicutes | Bacteria |
| ASV2861 | 427 | 2 | 1 | 427 | 100 | NR_026030.1 Actinobacillus porcinus strain NM319 16S ribosomal RNA, partial sequence | NR_026030.1 | 1466 | 335 | 761 | 29.13 | 773 | 0 | 424/427 | 99 | 0/427 | 0 | Plus | Plus | Bacteria; Proteobacteria; Gammaproteobacteria; Pasteurellales; Pasteurellaceae; Actinobacillus; Actinobacillus porcinus | Actinobacillus porcinus | Actinobacillus | Pasteurellaceae | Pasteurellales | Gammaproteobacteria | Proteobacteria | Bacteria |
| ASV2862 | 428 | 3 | 1 | 428 | 100 | NR_026413.1 Desulfovibrio intestinalis strain KMS2 16S ribosomal RNA, partial sequence | NR_026413.1 | 1509 | 337 | 763 | 28.3 | 617.9 | 1E-176 | 399/430 | 92 | 5/430 | 1 | Plus | Plus | Bacteria; Proteobacteria; Deltaproteobacteria; Desulfovibrionales; Desulfovibrionaceae; Desulfovibrio; Desulfovibrio intestinalis | Desulfovibrio intestinalis | Desulfovibrio | Desulfovibrionaceae | Desulfovibrionales | Deltaproteobacteria | Proteobacteria | Bacteria |
| ASV2863 | 421 | 1 | 1 | 421 | 100 | NR_144616.1 Muribaculum intestinale strain YL27 16S ribosomal RNA, partial sequence | NR_144616.1 | 1460 | 297 | 718 | 28.9 | 512.6 | 6E-145 | 374/422 | 88 | 1/422 | 0 | Plus | Plus | Bacteria; Bacteroidetes; Bacteroidia; Bacteroidales; Muribaculaceae; Muribaculum; Muribaculum intestinale | Muribaculum intestinale | Muribaculum | Muribaculaceae | Bacteroidales | Bacteroidia | Bacteroidetes | Bacteria |
| ASV2864 | 402 | 1 | 1 | 402 | 100 | NR_114789.1 [Clostridium] leptum strain DSM 753 16S ribosomal RNA, partial sequence | NR_114789.1 | 1505 | 364 | 765 | 26.71 | 571.7 | 9E-163 | 371/402 | 92 | 0/402 | 0 | Plus | Plus | Bacteria; Firmicutes; Clostridia; Eubacteriales; Oscillospiraceae; ; [Clostridium] leptum | [Clostridium] leptum | | Oscillospiraceae | Eubacteriales | Clostridia | Firmicutes | Bacteria |
| ASV2865 | 427 | 2 | 1 | 427 | 100 | NR_028725.2 Lactobacillus salivarius strain HO 66 16S ribosomal RNA, partial sequence | NR_028725.2 | 1542 | 371 | 797 | 27.69 | 750.9 | 0 | 420/427 | 98 | 0/427 | 0 | Plus | Plus | Bacteria; Firmicutes; Bacilli; Lactobacillales; Lactobacillaceae; Ligilactobacillus; Ligilactobacillus salivarius | Ligilactobacillus salivarius | Ligilactobacillus | Lactobacillaceae | Lactobacillales | Bacilli | Firmicutes | Bacteria |
| ASV2866 | 427 | 2 | 1 | 427 | 100 | NR_037018.1 Acidaminococcus fermentans strain VR4 16S ribosomal RNA gene, partial sequence | NR_037018.1 | 1516 | 349 | 775 | 28.17 | 734.2 | 0 | 417/427 | 97 | 0/427 | 0 | Plus | Plus | Bacteria; Firmicutes; Negativicutes; Acidaminococcales; Acidaminococcaceae; Acidaminococcus; Acidaminococcus fermentans | Acidaminococcus fermentans | Acidaminococcus | Acidaminococcaceae | Acidaminococcales | Negativicutes | Firmicutes | Bacteria |
| ASV2867 | 405 | 1 | 1 | 405 | 100 | NR_144742.1 Christensenella massiliensis strain Marseille-P2438 16S ribosomal RNA, partial sequence | NR_144742.1 | 1499 | 369 | 773 | 27.02 | 466.5 | 4E-131 | 354/405 | 87 | 0/405 | 0 | Plus | Plus | Bacteria; Firmicutes; Clostridia; Eubacteriales; Christensenellaceae; Christensenella; Christensenella massiliensis | Christensenella massiliensis | Christensenella | Christensenellaceae | Eubacteriales | Clostridia | Firmicutes | Bacteria |
| ASV2868 | 404 | 1 | 1 | 404 | 100 | NR_118156.1 Oscillibacter ruminantium GH1 16S ribosomal RNA, partial sequence | NR_118156.1 | 1409 | 321 | 724 | 28.67 | 630.8 | 2E-180 | 383/404 | 94 | 0/404 | 0 | Plus | Plus | Bacteria; Firmicutes; Clostridia; Eubacteriales; Oscillospiraceae; Oscillibacter; Oscillibacter ruminantium | Oscillibacter ruminantium | Oscillibacter | Oscillospiraceae | Eubacteriales | Clostridia | Firmicutes | Bacteria |
| ASV2869 | 422 | 2 | 1 | 422 | 100 | NR_113117.1 Prevotella oralis ATCC 33269 strain JCM 12251 16S ribosomal RNA, partial sequence | NR_113117.1 | 1496 | 361 | 782 | 28.21 | 536.6 | 4E-152 | 380/424 | 89 | 4/424 | 0 | Plus | Plus | Bacteria; Bacteroidetes; Bacteroidia; Bacteroidales; Prevotellaceae; Prevotella; Prevotella oralis | Prevotella oralis | Prevotella | Prevotellaceae | Bacteroidales | Bacteroidia | Bacteroidetes | Bacteria |
| ASV2871 | 402 | 1 | 1 | 402 | 100 | NR_151900.1 Ruthenibacterium lactatiformans strain 585-1 16S ribosomal RNA, partial sequence | NR_151900.1 | 1519 | 368 | 769 | 26.46 | 593.9 | 2E-169 | 375/402 | 93 | 0/402 | 0 | Plus | Plus | Bacteria; Firmicutes; Clostridia; Eubacteriales; Oscillospiraceae; Ruthenibacterium; Ruthenibacterium lactatiformans | Ruthenibacterium lactatiformans | Ruthenibacterium | Oscillospiraceae | Eubacteriales | Clostridia | Firmicutes | Bacteria |
| ASV2874 | 402 | 1 | 1 | 402 | 100 | NR_151900.1 Ruthenibacterium lactatiformans strain 585-1 16S ribosomal RNA, partial sequence | NR_151900.1 | 1519 | 368 | 769 | 26.46 | 593.9 | 2E-169 | 375/402 | 93 | 0/402 | 0 | Plus | Plus | Bacteria; Firmicutes; Clostridia; Eubacteriales; Oscillospiraceae; Ruthenibacterium; Ruthenibacterium lactatiformans | Ruthenibacterium lactatiformans | Ruthenibacterium | Oscillospiraceae | Eubacteriales | Clostridia | Firmicutes | Bacteria |
| ASV2875 | 422 | 1 | 1 | 422 | 100 | NR_041364.1 Prevotella stercorea DSM 18206 strain CB35 16S ribosomal RNA, partial sequence | NR_041364.1 | 1488 | 354 | 775 | 28.36 | 553.3 | 4E-157 | 382/423 | 90 | 2/423 | 0 | Plus | Plus | Bacteria; Bacteroidetes; Bacteroidia; Bacteroidales; Prevotellaceae; Prevotella; Prevotella stercorea | Prevotella stercorea | Prevotella | Prevotellaceae | Bacteroidales | Bacteroidia | Bacteroidetes | Bacteria |
| ASV2876 | 422 | 2 | 1 | 422 | 100 | NR_113117.1 Prevotella oralis ATCC 33269 strain JCM 12251 16S ribosomal RNA, partial sequence | NR_113117.1 | 1496 | 361 | 782 | 28.21 | 619.7 | 3E-177 | 393/422 | 93 | 0/422 | 0 | Plus | Plus | Bacteria; Bacteroidetes; Bacteroidia; Bacteroidales; Prevotellaceae; Prevotella; Prevotella oralis | Prevotella oralis | Prevotella | Prevotellaceae | Bacteroidales | Bacteroidia | Bacteroidetes | Bacteria |
| ASV2879 | 405 | 2 | 1 | 405 | 100 | NR_118554.1 Intestinimonas butyriciproducens strain SRB-521-5-I 16S ribosomal RNA, partial sequence | NR_118554.1 | 1475 | 346 | 750 | 27.46 | 499.7 | 4E-141 | 360/405 | 88 | 0/405 | 0 | Plus | Plus | Bacteria; Firmicutes; Clostridia; Eubacteriales; ; Intestinimonas; Intestinimonas butyriciproducens | Intestinimonas butyriciproducens | Intestinimonas | | Eubacteriales | Clostridia | Firmicutes | Bacteria |
| ASV2880 | 404 | 1 | 1 | 404 | 100 | NR_025127.1 Lachnobacterium bovis strain LRC 5382 16S ribosomal RNA, partial sequence | NR_025127.1 | 1453 | 362 | 765 | 27.8 | 647.4 | 0 | 386/404 | 95 | 0/404 | 0 | Plus | Plus | Bacteria; Firmicutes; Clostridia; Eubacteriales; Lachnospiraceae; Lachnobacterium; Lachnobacterium bovis | Lachnobacterium bovis | Lachnobacterium | Lachnospiraceae | Eubacteriales | Clostridia | Firmicutes | Bacteria |
| ASV2882 | 427 | 1 | 1 | 427 | 100 | NR_044111.1 Selenomonas bovis strain WG 16S ribosomal RNA, partial sequence | NR_044111.1 | 1525 | 370 | 796 | 28 | 761.9 | 0 | 422/427 | 98 | 0/427 | 0 | Plus | Plus | Bacteria; Firmicutes; Negativicutes; Selenomonadales; Selenomonadaceae; Selenomonas; Selenomonas bovis | Selenomonas bovis | Selenomonas | Selenomonadaceae | Selenomonadales | Negativicutes | Firmicutes | Bacteria |
| ASV2883 | 402 | 1 | 1 | 402 | 100 | NR_029097.1 Oribacterium sinus strain AIP 354.02 16S ribosomal RNA, partial sequence | NR_029097.1 | 1374 | 308 | 709 | 29.26 | 654.8 | 0 | 386/402 | 96 | 0/402 | 0 | Plus | Plus | Bacteria; Firmicutes; Clostridia; Eubacteriales; Lachnospiraceae; Oribacterium; Oribacterium sinus | Oribacterium sinus | Oribacterium | Lachnospiraceae | Eubacteriales | Clostridia | Firmicutes | Bacteria |
| ASV2884 | 403 | 1 | 3 | 403 | 99.5 | NR_125677.1 Vallitalea pronyensis strain FatNI3 16S ribosomal RNA, partial sequence | NR_125677.1 | 1480 | 365 | 764 | 27.03 | 457.2 | 3E-128 | 352/403 | 87 | 5/403 | 1 | Plus | Plus | Bacteria; Firmicutes; Clostridia; Eubacteriales; Vallitaleaceae; Vallitalea; Vallitalea pronyensis | Vallitalea pronyensis | Vallitalea | Vallitaleaceae | Eubacteriales | Clostridia | Firmicutes | Bacteria |
| ASV2885 | 427 | 5 | 15 | 427 | 96.72 | NR_103946.1 Spiroplasma syrphidicola EA-1 16S ribosomal RNA, partial sequence | NR_103946.1 | 1508 | 355 | 766 | 27.32 | 429.5 | 6E-120 | 354/414 | 85 | 3/414 | 0 | Plus | Plus | Bacteria; Tenericutes; Mollicutes; Entomoplasmatales; Spiroplasmataceae; Spiroplasma; Spiroplasma syrphidicola | Spiroplasma syrphidicola | Spiroplasma | Spiroplasmataceae | Entomoplasmatales | Mollicutes | Tenericutes | Bacteria |
| ASV2886 | 422 | 1 | 1 | 422 | 100 | NR_113411.1 Prevotella copri DSM 18205 strain JCM 13464 16S ribosomal RNA, partial sequence | NR_113411.1 | 1493 | 358 | 779 | 28.27 | 581 | 2E-165 | 387/423 | 91 | 2/423 | 0 | Plus | Plus | Bacteria; Bacteroidetes; Bacteroidia; Bacteroidales; Prevotellaceae; Prevotella; Prevotella copri | Prevotella copri | Prevotella | Prevotellaceae | Bacteroidales | Bacteroidia | Bacteroidetes | Bacteria |
| ASV2888 | 402 | 1 | 1 | 402 | 100 | NR_104846.1 Gemmiger formicilis strain X2-56 16S ribosomal RNA, partial sequence | NR_104846.1 | 1426 | 305 | 706 | 28.19 | 699.2 | 0 | 394/402 | 98 | 0/402 | 0 | Plus | Plus | Bacteria; Firmicutes; Clostridia; Eubacteriales; ; Gemmiger; Gemmiger formicilis | Gemmiger formicilis | Gemmiger | | Eubacteriales | Clostridia | Firmicutes | Bacteria |
| ASV2889 | 402 | 2 | 1 | 402 | 100 | NR_104892.1 Clostridium moniliforme strain 2055 16S ribosomal RNA, partial sequence | NR_104892.1 | 1488 | 330 | 731 | 27.02 | 643.8 | 0 | 384/402 | 95 | 0/402 | 0 | Plus | Plus | Bacteria; Firmicutes; Clostridia; Eubacteriales; Clostridiaceae; Clostridium; Clostridium moniliforme | Clostridium moniliforme | Clostridium | Clostridiaceae | Eubacteriales | Clostridia | Firmicutes | Bacteria |
| ASV2890 | 421 | 2 | 1 | 421 | 100 | NR_113039.1 Solobacterium moorei strain JCM 10645 16S ribosomal RNA, partial sequence | NR_113039.1 | 1486 | 352 | 772 | 28.33 | 534.8 | 1E-151 | 379/423 | 89 | 4/423 | 0 | Plus | Plus | Bacteria; Firmicutes; Erysipelotrichia; Erysipelotrichales; Erysipelotrichaceae; Solobacterium; Solobacterium moorei | Solobacterium moorei | Solobacterium | Erysipelotrichaceae | Erysipelotrichales | Erysipelotrichia | Firmicutes | Bacteria |
| ASV2891 | 427 | 3 | 1 | 427 | 100 | NR_036918.1 Streptococcus suis strain S735 16S ribosomal RNA, partial sequence | NR_036918.1 | 1535 | 354 | 780 | 27.82 | 784.1 | 0 | 426/427 | 99 | 0/427 | 0 | Plus | Plus | Bacteria; Firmicutes; Bacilli; Lactobacillales; Streptococcaceae; Streptococcus; Streptococcus suis | Streptococcus suis | Streptococcus | Streptococcaceae | Lactobacillales | Bacilli | Firmicutes | Bacteria |
| ASV2892 | 402 | 3 | 1 | 402 | 100 | NR_114728.1 Clostridium tertium strain ATCC 14573 16S ribosomal RNA, partial sequence | NR_114728.1 | 1415 | 304 | 705 | 28.41 | 588.4 | 9E-168 | 375/403 | 93 | 2/403 | 0 | Plus | Plus | Bacteria; Firmicutes; Clostridia; Eubacteriales; Clostridiaceae; Clostridium; Clostridium tertium | Clostridium tertium | Clostridium | Clostridiaceae | Eubacteriales | Clostridia | Firmicutes | Bacteria |
| ASV2893 | 402 | 1 | 1 | 402 | 100 | NR_044425.1 Hydrogenoanaerobacterium saccharovorans strain SW512 16S ribosomal RNA, partial sequence | NR_044425.1 | 1510 | 363 | 764 | 26.62 | 616.1 | 4E-176 | 379/402 | 94 | 0/402 | 0 | Plus | Plus | Bacteria; Firmicutes; Clostridia; Eubacteriales; Oscillospiraceae; Hydrogenoanaerobacterium; Hydrogenoanaerobacterium saccharovorans | Hydrogenoanaerobacterium saccharovorans | Hydrogenoanaerobacterium | Oscillospiraceae | Eubacteriales | Clostridia | Firmicutes | Bacteria |
| ASV2894 | 427 | 2 | 1 | 427 | 100 | NR_037018.1 Acidaminococcus fermentans strain VR4 16S ribosomal RNA gene, partial sequence | NR_037018.1 | 1516 | 349 | 775 | 28.17 | 706.5 | 0 | 412/427 | 96 | 0/427 | 0 | Plus | Plus | Bacteria; Firmicutes; Negativicutes; Acidaminococcales; Acidaminococcaceae; Acidaminococcus; Acidaminococcus fermentans | Acidaminococcus fermentans | Acidaminococcus | Acidaminococcaceae | Acidaminococcales | Negativicutes | Firmicutes | Bacteria |

| | | | | | | | | | | | | | | | | | | | | | | | | | | | |
|---|---|---|---|---|---|---|---|---|---|---|---|---|---|---|---|---|---|---|---|---|---|---|---|---|---|---|---|
| ASV2908 | 403 | 2 | 1 | 403 | 100 | NR_102987.1 Hungateiclostridium clariflavum DSM 19732 16S ribosomal RNA, partial sequence | NR_102987.1 | 1634 | 481 | 882 | 24.6 | 588.4 | 9E-168 | 375/403 | 93 | 1/403 | 0 | Plus | Plus | Bacteria; Firmicutes; Clostridia; Eubacteriales; Oscillospiraceae; Acetivibrio; Acetivibrio clariflavus | Acetivibrio clariflavus | Acetivibrio | Oscillospiraceae | Eubacteriales | Clostridia | Firmicutes | Bacteria |
| ASV2909 | 406 | 1 | 1 | 406 | 100 | NR_115340.1 Saccharofermentans acetigenes strain P6 16S ribosomal RNA, partial sequence | NR_115340.1 | 1510 | 353 | 757 | 26.82 | 422.2 | 1E-117 | 349/408 | 85 | 5/408 | 1 | Plus | Plus | Bacteria; Firmicutes; Clostridia; Eubacteriales; Oscillospiraceae; Saccharofermentans; Saccharofermentans acetigenes | Saccharofermentans acetigenes | Saccharofermentans | Oscillospiraceae | Eubacteriales | Clostridia | Firmicutes | Bacteria |
| ASV2910 | 421 | 1 | 1 | 421 | 100 | NR_113097.1 Prevotella buccae ATCC 33574 strain JCM 12245 16S ribosomal RNA, partial sequence | NR_113097.1 | 1480 | 343 | 764 | 28.51 | 457.2 | 3E-128 | 367/425 | 86 | 7/425 | 1 | Plus | Plus | Bacteria; Bacteroidetes; Bacteroidia; Bacteroidales; Prevotellaceae; Prevotella; Prevotella buccae | Prevotella buccae | Prevotella | Prevotellaceae | Bacteroidales | Bacteroidia | Bacteroidetes | Bacteria |
| ASV2911 | 405 | 1 | 1 | 405 | 100 | NR_144736.1 Neglecta timonensis strain SN17 16S ribosomal RNA, partial sequence | NR_144736.1 | 1503 | 377 | 778 | 26.75 | 523.7 | 3E-148 | 365/405 | 90 | 3/405 | 0 | Plus | Plus | Bacteria; Firmicutes; Clostridia; Eubacteriales; ; Neglecta; Neglecta timonensis | Neglecta timonensis | Neglecta | | Eubacteriales | Clostridia | Firmicutes | Bacteria |
| ASV2912 | 405 | 1 | 1 | 405 | 100 | NR_042834.1 Alkalibacter saccharofermentans strain Z-79820 16S ribosomal RNA, partial sequence | NR_042834.1 | 1465 | 338 | 741 | 27.58 | 475.7 | 7E-134 | 357/406 | 87 | 3/406 | 0 | Plus | Plus | Bacteria; Firmicutes; Clostridia; Eubacteriales; Eubacteriaceae; Alkalibacter; Alkalibacter saccharofermentans | Alkalibacter saccharofermentans | Alkalibacter | Eubacteriaceae | Eubacteriales | Clostridia | Firmicutes | Bacteria |
| ASV2913 | 401 | 1 | 1 | 399 | 99.5 | NR_044716.2 Clostridium carnis strain ATCC 25777 16S ribosomal RNA, partial sequence | NR_044716.2 | 1455 | 357 | 756 | 27.49 | 446.2 | 6E-125 | 348/401 | 86 | 3/401 | 0 | Plus | Plus | Bacteria; Firmicutes; Clostridia; Eubacteriales; Clostridiaceae; Clostridium; Clostridium carnis | Clostridium carnis | Clostridium | Clostridiaceae | Eubacteriales | Clostridia | Firmicutes | Bacteria |
| ASV2914 | 422 | 1 | 1 | 422 | 100 | NR_112943.1 Bacteroides stercoris ATCC 43183 strain JCM 9496 16S ribosomal RNA, partial sequence | NR_112943.1 | 1476 | 343 | 764 | 28.59 | 680.7 | 0 | 404/422 | 95 | 0/422 | 0 | Plus | Plus | Bacteria; Bacteroidetes; Bacteroidia; Bacteroidales; Bacteroidaceae; Bacteroides; Bacteroides stercoris | Bacteroides stercoris | Bacteroides | Bacteroidaceae | Bacteroidales | Bacteroidia | Bacteroidetes | Bacteria |
| ASV2915 | 404 | 1 | 1 | 404 | 100 | NR_159296.1 Aminipila butyrica strain FH042 16S ribosomal RNA, partial sequence | NR_159296.1 | 1460 | 352 | 755 | 27.67 | 697.3 | 0 | 396/405 | 97 | 2/405 | 0 | Plus | Plus | Bacteria; Firmicutes; Clostridia; Eubacteriales; Clostridiales Family XIII. Incertae Sedis; Aminipila; Aminipila butyrica | Aminipila butyrica | Aminipila | Clostridiales Family XIII. Incertae Sedis | Eubacteriales | Clostridia | Firmicutes | Bacteria |
| ASV2916 | 422 | 1 | 1 | 422 | 100 | NR_145844.1 Bacteroides caecigallinarum strain C13EG111 16S ribosomal RNA, partial sequence | NR_145844.1 | 1489 | 356 | 777 | 28.34 | 614.2 | 2E-175 | 393/423 | 92 | 2/423 | 0 | Plus | Plus | Bacteria; Bacteroidetes; Bacteroidia; Bacteroidales; Bacteroidaceae; Bacteroides; Bacteroides caecigallinarum | Bacteroides caecigallinarum | Bacteroides | Bacteroidaceae | Bacteroidales | Bacteroidia | Bacteroidetes | Bacteria |
| ASV2917 | 422 | 1 | 1 | 422 | 100 | NR_113208.1 Parabacteroides chinchillae strain JCM 17104 16S ribosomal RNA, partial sequence | NR_113208.1 | 1489 | 353 | 774 | 28.34 | 625.3 | 7E-179 | 395/423 | 93 | 2/423 | 0 | Plus | Plus | Bacteria; Bacteroidetes; Bacteroidia; Bacteroidales; Tannerellaceae; Parabacteroides; Parabacteroides chinchillae | Parabacteroides chinchillae | Parabacteroides | Tannerellaceae | Bacteroidales | Bacteroidia | Bacteroidetes | Bacteria |
| ASV2918 | 427 | 2 | 1 | 426 | 99.77 | NR_117778.1 Sutterella wadsworthensis strain WAL 9799 16S ribosomal RNA, partial sequence | NR_117778.1 | 1462 | 310 | 735 | 29.14 | 616.1 | 4E-176 | 395/426 | 92 | 0/426 | 0 | Plus | Plus | Bacteria; Proteobacteria; Betaproteobacteria; Burkholderiales; Sutterellaceae; Sutterella; Sutterella wadsworthensis | Sutterella wadsworthensis | Sutterella | Sutterellaceae | Burkholderiales | Betaproteobacteria | Proteobacteria | Bacteria |
| ASV2919 | 405 | 1 | 1 | 405 | 100 | NR_147370.1 Pseudoflavonifractor phocaeensis strain Marseille-P3064 16S ribosomal RNA, partial sequence | NR_147370.1 | 1495 | 366 | 770 | 27.09 | 499.7 | 4E-141 | 362/407 | 88 | 4/407 | 0 | Plus | Plus | Bacteria; Firmicutes; Clostridia; Eubacteriales; Oscillospiraceae; Pseudoflavonifractor; Pseudoflavonifractor phocaeensis | Pseudoflavonifractor phocaeensis | Pseudoflavonifractor | Oscillospiraceae | Eubacteriales | Clostridia | Firmicutes | Bacteria |
| ASV2920 | 402 | 1 | 1 | 402 | 100 | NR_159227.1 Monoglobus pectinilyticus strain 14 16S ribosomal RNA, partial sequence | NR_159227.1 | 1493 | 368 | 769 | 26.93 | 649.3 | 0 | 385/402 | 95 | 0/402 | 0 | Plus | Plus | Bacteria; Firmicutes; Clostridia; Eubacteriales; Oscillospiraceae; Monoglobus; Monoglobus pectinilyticus | Monoglobus pectinilyticus | Monoglobus | Oscillospiraceae | Eubacteriales | Clostridia | Firmicutes | Bacteria |
| ASV2921 | 402 | 1 | 1 | 402 | 100 | NR_114789.1 [Clostridium] leptum strain DSM 753 16S ribosomal RNA, partial sequence | NR_114789.1 | 1505 | 364 | 765 | 26.71 | 605 | 9E-173 | 377/402 | 93 | 0/402 | 0 | Plus | Plus | Bacteria; Firmicutes; Clostridia; Eubacteriales; Oscillospiraceae; ; [Clostridium] leptum | [Clostridium] leptum | | Oscillospiraceae | Eubacteriales | Clostridia | Firmicutes | Bacteria |
| ASV2923 | 402 | 1 | 1 | 402 | 100 | NR_158111.1 Paludicola psychrotolerans strain NC1253 16S ribosomal RNA, partial sequence | NR_158111.1 | 1490 | 366 | 766 | 26.91 | 653 | 0 | 386/402 | 96 | 1/402 | 0 | Plus | Plus | Bacteria; Firmicutes; Clostridia; Eubacteriales; Oscillospiraceae; Paludicola; Paludicola psychrotolerans | Paludicola psychrotolerans | Paludicola | Oscillospiraceae | Eubacteriales | Clostridia | Firmicutes | Bacteria |
| ASV2924 | 402 | 1 | 1 | 402 | 100 | NR_159227.1 Monoglobus pectinilyticus strain 14 16S ribosomal RNA, partial sequence | NR_159227.1 | 1493 | 368 | 769 | 26.93 | 638.2 | 0 | 384/403 | 95 | 2/403 | 0 | Plus | Plus | Bacteria; Firmicutes; Clostridia; Eubacteriales; Oscillospiraceae; Monoglobus; Monoglobus pectinilyticus | Monoglobus pectinilyticus | Monoglobus | Oscillospiraceae | Eubacteriales | Clostridia | Firmicutes | Bacteria |
| ASV2925 | 402 | 1 | 1 | 402 | 100 | NR_118675.1 [Eubacterium] siraeum strain ATCC 29066 16S ribosomal RNA, partial sequence | NR_118675.1 | 1453 | 356 | 757 | 27.67 | 595.7 | 5E-170 | 374/402 | 93 | 0/402 | 0 | Plus | Plus | Bacteria; Firmicutes; Clostridia; Eubacteriales; Oscillospiraceae; ; [Eubacterium] siraeum | [Eubacterium] siraeum | | Oscillospiraceae | Eubacteriales | Clostridia | Firmicutes | Bacteria |
| ASV2926 | 418 | 1 | 1 | 418 | 100 | NR_102893.1 Butyrivibrio proteoclasticus strain B316 16S ribosomal RNA, partial sequence | NR_102893.1 | 1537 | 367 | 782 | 27.07 | 605 | 9E-173 | 388/418 | 92 | 2/418 | 0 | Plus | Plus | Bacteria; Firmicutes; Clostridia; Eubacteriales; Lachnospiraceae; Butyrivibrio; Butyrivibrio proteoclasticus | Butyrivibrio proteoclasticus | Butyrivibrio | Lachnospiraceae | Eubacteriales | Clostridia | Firmicutes | Bacteria |
| ASV2927 | 427 | 1 | 4 | 427 | 99.3 | NR_146687.1 Breznakia pachnodae strain Pei061 16S ribosomal RNA, partial sequence | NR_146687.1 | 1499 | 370 | 792 | 28.22 | 444.3 | 2E-124 | 366/427 | 85 | 7/427 | 1 | Plus | Plus | Bacteria; Firmicutes; Erysipelotrichia; Erysipelotrichales; Erysipelotrichaceae; Breznakia; Breznakia pachnodae | Breznakia pachnodae | Breznakia | Erysipelotrichaceae | Erysipelotrichales | Erysipelotrichia | Firmicutes | Bacteria |
| ASV2928 | 402 | 1 | 1 | 402 | 100 | NR_134005.1 Clostridium bornimense strain M2/40 16S ribosomal RNA, partial sequence | NR_134005.1 | 1363 | 284 | 685 | 29.49 | 571.7 | 9E-163 | 372/403 | 92 | 2/403 | 0 | Plus | Plus | Bacteria; Firmicutes; Clostridia; Eubacteriales; Clostridiaceae; Clostridium; Clostridium bornimense | Clostridium bornimense | Clostridium | Clostridiaceae | Eubacteriales | Clostridia | Firmicutes | Bacteria |
| ASV2929 | 422 | 2 | 1 | 422 | 100 | NR_113117.1 Prevotella oralis ATCC 33269 strain JCM 12251 16S ribosomal RNA, partial sequence | NR_113117.1 | 1496 | 361 | 782 | 28.21 | 614.2 | 2E-175 | 393/423 | 92 | 2/423 | 0 | Plus | Plus | Bacteria; Bacteroidetes; Bacteroidia; Bacteroidales; Prevotellaceae; Prevotella; Prevotella oralis | Prevotella oralis | Prevotella | Prevotellaceae | Bacteroidales | Bacteroidia | Bacteroidetes | Bacteria |
| ASV2930 | 427 | 1 | 1 | 427 | 100 | NR_041666.1 Dialister succinatiphilus YIT 11850 16S ribosomal RNA, partial sequence | NR_041666.1 | 1521 | 353 | 779 | 28.07 | 435.1 | 1E-121 | 366/430 | 85 | 6/430 | 1 | Plus | Plus | Bacteria; Firmicutes; Negativicutes; Veillonellales; Veillonellaceae; Dialister; Dialister succinatiphilus | Dialister succinatiphilus | Dialister | Veillonellaceae | Veillonellales | Negativicutes | Firmicutes | Bacteria |
| ASV2931 | 426 | 1 | 1 | 425 | 99.77 | NR_027526.1 Catenibacterium mitsuokai strain DSM 15897 16S ribosomal RNA, partial sequence | NR_027526.1 | 1485 | 345 | 767 | 28.48 | 712.1 | 0 | 412/425 | 96 | 2/425 | 0 | Plus | Plus | Bacteria; Firmicutes; Erysipelotrichia; Erysipelotrichales; Coprobacillaceae; Catenibacterium; Catenibacterium mitsuokai | Catenibacterium mitsuokai | Catenibacterium | Coprobacillaceae | Erysipelotrichales | Erysipelotrichia | Firmicutes | Bacteria |
| ASV2932 | 427 | 4 | 1 | 422 | 98.83 | NR_042802.1 Lactobacillus ultunensis strain Kx146C1 16S ribosomal RNA, partial sequence | NR_042802.1 | 1550 | 370 | 791 | 27.23 | 736.1 | 0 | 414/422 | 98 | 0/422 | 0 | Plus | Plus | Bacteria; Firmicutes; Bacilli; Lactobacillales; Lactobacillaceae; Lactobacillus; Lactobacillus ultunensis | Lactobacillus ultunensis | Lactobacillus | Lactobacillaceae | Lactobacillales | Bacilli | Firmicutes | Bacteria |
| ASV2933 | 403 | 1 | 3 | 403 | 99.5 | NR_125677.1 Vallitalea pronyensis strain FatNI3 16S ribosomal RNA, partial sequence | NR_125677.1 | 1480 | 365 | 764 | 27.03 | 484.9 | 1E-136 | 357/403 | 88 | 5/403 | 1 | Plus | Plus | Bacteria; Firmicutes; Clostridia; Eubacteriales; Vallitaleaceae; Vallitalea; Vallitalea pronyensis | Vallitalea pronyensis | Vallitalea | Vallitaleaceae | Eubacteriales | Clostridia | Firmicutes | Bacteria |
| ASV2934 | 423 | 1 | 1 | 423 | 100 | NR_144750.1 Prevotellamassilia timonensis strain Marseille-P2831 16S ribosomal RNA, partial sequence | NR_144750.1 | 1493 | 358 | 779 | 28.27 | 553.3 | 4E-157 | 384/425 | 90 | 5/425 | 1 | Plus | Plus | Bacteria; Bacteroidetes; Bacteroidia; Bacteroidales; Prevotellaceae; Prevotellamassilia; Prevotellamassilia timonensis | Prevotellamassilia timonensis | Prevotellamassilia | Prevotellaceae | Bacteroidales | Bacteroidia | Bacteroidetes | Bacteria |
| ASV2935 | 427 | 1 | 1 | 427 | 100 | NR_108477.1 Streptococcus porcorum strain 682/03 16S ribosomal RNA, partial sequence | NR_108477.1 | 1380 | 268 | 694 | 30.94 | 761.9 | 0 | 422/427 | 98 | 0/427 | 0 | Plus | Plus | Bacteria; Firmicutes; Bacilli; Lactobacillales; Streptococcaceae; Streptococcus; Streptococcus porcorum | Streptococcus porcorum | Streptococcus | Streptococcaceae | Lactobacillales | Bacilli | Firmicutes | Bacteria |
| ASV2936 | 422 | 3 | 1 | 422 | 100 | NR_113105.1 Prevotella histicola strain JCM 15637 16S ribosomal RNA, partial sequence | NR_113105.1 | 1480 | 346 | 767 | 28.51 | 630.8 | 2E-180 | 397/424 | 93 | 4/424 | 0 | Plus | Plus | Bacteria; Bacteroidetes; Bacteroidia; Bacteroidales; Prevotellaceae; Prevotella; Prevotella histicola | Prevotella histicola | Prevotella | Prevotellaceae | Bacteroidales | Bacteroidia | Bacteroidetes | Bacteria |
| ASV2937 | 402 | 1 | 1 | 402 | 100 | NR_114789.1 [Clostridium] leptum strain DSM 753 16S ribosomal RNA, partial sequence | NR_114789.1 | 1505 | 364 | 765 | 26.71 | 643.8 | 0 | 384/402 | 95 | 0/402 | 0 | Plus | Plus | Bacteria; Firmicutes; Clostridia; Eubacteriales; Oscillospiraceae; ; [Clostridium] leptum | [Clostridium] leptum | | Oscillospiraceae | Eubacteriales | Clostridia | Firmicutes | Bacteria |
| ASV2938 | 404 | 2 | 1 | 404 | 100 | NR_074793.2 Oscillibacter valericigenes strain Sjm18-20 16S ribosomal RNA, complete sequence | NR_074793.2 | 1530 | 372 | 775 | 26.41 | 636.4 | 0 | 384/404 | 95 | 0/404 | 0 | Plus | Plus | Bacteria; Firmicutes; Clostridia; Eubacteriales; Oscillospiraceae; Oscillibacter; Oscillibacter valericigenes | Oscillibacter valericigenes | Oscillibacter | Oscillospiraceae | Eubacteriales | Clostridia | Firmicutes | Bacteria |
| ASV2939 | 421 | 1 | 1 | 421 | 100 | NR_144616.1 Muribaculum intestinale strain YL27 16S ribosomal RNA, partial sequence | NR_144616.1 | 1460 | 297 | 718 | 28.9 | 557 | 3E-158 | 385/425 | 90 | 7/425 | 1 | Plus | Plus | Bacteria; Bacteroidetes; Bacteroidia; Bacteroidales; Muribaculaceae; Muribaculum; Muribaculum intestinale | Muribaculum intestinale | Muribaculum | Muribaculaceae | Bacteroidales | Bacteroidia | Bacteroidetes | Bacteria |
| ASV2941 | 422 | 1 | 1 | 422 | 100 | NR_113208.1 Parabacteroides chinchillae strain JCM 17104 16S ribosomal RNA, partial sequence | NR_113208.1 | 1489 | 353 | 774 | 28.34 | 636.4 | 0 | 397/423 | 93 | 2/423 | 0 | Plus | Plus | Bacteria; Bacteroidetes; Bacteroidia; Bacteroidales; Tannerellaceae; Parabacteroides; Parabacteroides chinchillae | Parabacteroides chinchillae | Parabacteroides | Tannerellaceae | Bacteroidales | Bacteroidia | Bacteroidetes | Bacteria |
| ASV2942 | 402 | 2 | 3 | 402 | 99.5 | NR_148837.1 Abyssivirga alkaniphila strain L81 16S ribosomal RNA, partial sequence | NR_148837.1 | 1520 | 366 | 765 | 26.32 | 490.5 | 3E-138 | 356/401 | 88 | 2/401 | 0 | Plus | Plus | Bacteria; Firmicutes; Clostridia; Eubacteriales; Lachnospiraceae; Abyssivirga; Abyssivirga alkaniphila | Abyssivirga alkaniphila | Abyssivirga | Lachnospiraceae | Eubacteriales | Clostridia | Firmicutes | Bacteria |
| ASV2943 | 422 | 1 | 1 | 422 | 100 | NR_144750.1 Prevotellamassilia timonensis strain Marseille-P2831 16S ribosomal RNA, partial sequence | NR_144750.1 | 1493 | 358 | 779 | 28.27 | 653 | 0 | 399/422 | 94 | 0/422 | 0 | Plus | Plus | Bacteria; Bacteroidetes; Bacteroidia; Bacteroidales; Prevotellaceae; Prevotellamassilia; Prevotellamassilia timonensis | Prevotellamassilia timonensis | Prevotellamassilia | Prevotellaceae | Bacteroidales | Bacteroidia | Bacteroidetes | Bacteria |
| ASV2944 | 405 | 2 | 3 | 405 | 99.51 | NR_074629.1 Hungateiclostridium thermocellum strain ATCC 27405 16S ribosomal RNA, partial sequence | NR_074629.1 | 1521 | 360 | 762 | 26.5 | 468.3 | 1E-131 | 353/403 | 87 | 0/403 | 0 | Plus | Plus | Bacteria; Firmicutes; Clostridia; Eubacteriales; Oscillospiraceae; Acetivibrio; Acetivibrio thermocellus | Acetivibrio thermocellus | Acetivibrio | Oscillospiraceae | Eubacteriales | Clostridia | Firmicutes | Bacteria |
| ASV2945 | 404 | 1 | 1 | 404 | 100 | NR_116001.1 Cellulosilyticum ruminicola JCM 14822 strain H1 16S ribosomal RNA, partial sequence | NR_116001.1 | 1547 | 389 | 792 | 26.12 | 630.8 | 2E-180 | 384/405 | 94 | 2/405 | 0 | Plus | Plus | Bacteria; Firmicutes; Clostridia; Eubacteriales; Lachnospiraceae; Cellulosilyticum; Cellulosilyticum ruminicola | Cellulosilyticum ruminicola | Cellulosilyticum | Lachnospiraceae | Eubacteriales | Clostridia | Firmicutes | Bacteria |
| ASV2946 | 422 | 2 | 1 | 422 | 100 | NR_115301.1 Bacteroides ovatus strain CIP 103756 16S ribosomal RNA, partial sequence | NR_115301.1 | 1451 | 337 | 758 | 29.08 | 774.9 | 0 | 421/422 | 99 | 0/422 | 0 | Plus | Plus | Bacteria; Bacteroidetes; Bacteroidia; Bacteroidales; Bacteroidaceae; Bacteroides; Bacteroides ovatus | Bacteroides ovatus | Bacteroides | Bacteroidaceae | Bacteroidales | Bacteroidia | Bacteroidetes | Bacteria |
| ASV2947 | 402 | 1 | 1 | 402 | 100 | NR_118675.1 [Eubacterium] siraeum strain ATCC 29066 16S ribosomal RNA, partial sequence | NR_118675.1 | 1453 | 356 | 757 | 27.67 | 617.9 | 1E-176 | 378/402 | 94 | 0/402 | 0 | Plus | Plus | Bacteria; Firmicutes; Clostridia; Eubacteriales; Oscillospiraceae; ; [Eubacterium] siraeum | [Eubacterium] siraeum | | Oscillospiraceae | Eubacteriales | Clostridia | Firmicutes | Bacteria |
| ASV2948 | 401 | 1 | 1 | 401 | 100 | NR_144609.1 Enterorhabdus muris strain WCA-131-CoC-2 16S ribosomal RNA, partial sequence | NR_144609.1 | 1399 | 336 | 738 | 28.81 | 544 | 2E-154 | 369/405 | 91 | 6/405 | 1 | Plus | Plus | Bacteria; Actinobacteria; Coriobacteriia; Eggerthellales; Eggerthellaceae; Adlercreutzia; Adlercreutzia muris | Adlercreutzia muris | Adlercreutzia | Eggerthellaceae | Eggerthellales | Coriobacteriia | Actinobacteria | Bacteria |
| ASV2949 | 402 | 2 | 1 | 402 | 100 | NR_102884.1 Ruminococcus champanellensis 18P13 = JCM 17042 16S ribosomal RNA, partial sequence | NR_102884.1 | 1494 | 348 | 749 | 26.91 | 605 | 9E-173 | 379/404 | 93 | 4/404 | 0 | Plus | Plus | Bacteria; Firmicutes; Clostridia; Eubacteriales; Oscillospiraceae; Ruminococcus; Ruminococcus champanellensis | Ruminococcus champanellensis | Ruminococcus | Oscillospiraceae | Eubacteriales | Clostridia | Firmicutes | Bacteria |
| ASV2950 | 402 | 1 | 1 | 402 | 100 | NR_102884.1 Ruminococcus champanellensis 18P13 = JCM 17042 16S ribosomal RNA, partial sequence | NR_102884.1 | 1494 | 348 | 749 | 26.91 | 715.8 | 0 | 397/402 | 98 | 0/402 | 0 | Plus | Plus | Bacteria; Firmicutes; Clostridia; Eubacteriales; Oscillospiraceae; Ruminococcus; Ruminococcus champanellensis | Ruminococcus champanellensis | Ruminococcus | Oscillospiraceae | Eubacteriales | Clostridia | Firmicutes | Bacteria |
| ASV2951 | 405 | 3 | 1 | 405 | 100 | NR_115307.1 Ethanoligenens harbinense YUAN-3 16S ribosomal RNA, partial sequence | NR_115307.1 | 1497 | 340 | 743 | 26.99 | 547.7 | 2E-155 | 371/407 | 91 | 5/407 | 1 | Plus | Plus | Bacteria; Firmicutes; Clostridia; Eubacteriales; Oscillospiraceae; Ethanoligenens; Ethanoligenens harbinense | Ethanoligenens harbinense | Ethanoligenens | Oscillospiraceae | Eubacteriales | Clostridia | Firmicutes | Bacteria |
| ASV2952 | 421 | 1 | 1 | 421 | 100 | NR_144616.1 Muribaculum intestinale strain YL27 16S ribosomal RNA, partial sequence | NR_144616.1 | 1460 | 297 | 718 | 28.9 | 479.4 | 6E-135 | 369/423 | 87 | 3/423 | 0 | Plus | Plus | Bacteria; Bacteroidetes; Bacteroidia; Bacteroidales; Muribaculaceae; Muribaculum; Muribaculum intestinale | Muribaculum intestinale | Muribaculum | Muribaculaceae | Bacteroidales | Bacteroidia | Bacteroidetes | Bacteria |
| ASV2953 | 422 | 1 | 1 | 422 | 100 | NR_041342.1 Parabacteroides distasonis strain JCM 5825 16S ribosomal RNA, partial sequence | NR_041342.1 | 1488 | 353 | 774 | 28.36 | 763.8 | 0 | 419/422 | 99 | 0/422 | 0 | Plus | Plus | Bacteria; Bacteroidetes; Bacteroidia; Bacteroidales; Tannerellaceae; Parabacteroides; Parabacteroides distasonis | Parabacteroides distasonis | Parabacteroides | Tannerellaceae | Bacteroidales | Bacteroidia | Bacteroidetes | Bacteria |
| ASV2954 | 422 | 1 | 1 | 422 | 100 | NR_144745.1 Culturomica massiliensis strain Marseille-P2698 16S ribosomal RNA, partial sequence | NR_144745.1 | 1492 | 356 | 776 | 28.22 | 595.7 | 6E-170 | 391/424 | 92 | 5/424 | 1 | Plus | Plus | Bacteria; Bacteroidetes; Bacteroidia; Bacteroidales; Odoribacteraceae; Culturomica; Culturomica massiliensis | Culturomica massiliensis | Culturomica | Odoribacteraceae | Bacteroidales | Bacteroidia | Bacteroidetes | Bacteria |
| ASV2955 | 422 | 1 | 1 | 422 | 100 | NR_040877.1 Prevotella copri DSM 18205 strain CB7 16S ribosomal RNA, partial sequence | NR_040877.1 | 1494 | 358 | 779 | 28.25 | 571.7 | 1E-162 | 384/422 | 90 | 0/422 | 0 | Plus | Plus | Bacteria; Bacteroidetes; Bacteroidia; Bacteroidales; Prevotellaceae; Prevotella; Prevotella copri | Prevotella copri | Prevotella | Prevotellaceae | Bacteroidales | Bacteroidia | Bacteroidetes | Bacteria |
| ASV2956 | 405 | 1 | 1 | 405 | 100 | NR_114789.1 [Clostridium] leptum strain DSM 753 16S ribosomal RNA, partial sequence | NR_114789.1 | 1505 | 364 | 765 | 26.71 | 529.3 | 6E-150 | 366/405 | 90 | 3/405 | 0 | Plus | Plus | Bacteria; Firmicutes; Clostridia; Eubacteriales; Oscillospiraceae; ; [Clostridium] leptum | [Clostridium] leptum | | Oscillospiraceae | Eubacteriales | Clostridia | Firmicutes | Bacteria |
| ASV2958 | 405 | 2 | 1 | 405 | 100 | NR_074114.1 Elusimicrobium minutum strain Pei191 16S ribosomal RNA, partial sequence | NR_074114.1 | 1433 | 356 | 760 | 28.26 | 654.8 | 0 | 390/407 | 95 | 4/407 | 0 | Plus | Plus | Bacteria; Elusimicrobia; Elusimicrobia; Elusimicrobiales; Elusimicrobiaceae; Elusimicrobium; Elusimicrobium minutum | Elusimicrobium minutum | Elusimicrobium | Elusimicrobiaceae | Elusimicrobiales | Elusimicrobia | Elusimicrobia | Bacteria |
| ASV2959 | 404 | 1 | 1 | 404 | 100 | NR_118156.1 Oscillibacter ruminantium GH1 16S ribosomal RNA, partial sequence | NR_118156.1 | 1409 | 321 | 724 | 28.67 | 653 | 0 | 389/406 | 95 | 4/406 | 0 | Plus | Plus | Bacteria; Firmicutes; Clostridia; Eubacteriales; Oscillospiraceae; Oscillibacter; Oscillibacter ruminantium | Oscillibacter ruminantium | Oscillibacter | Oscillospiraceae | Eubacteriales | Clostridia | Firmicutes | Bacteria |
| ASV2960 | 427 | 1 | 1 | 427 | 100 | NR_042871.1 [Actinobacillus] rossii strain ATCC 27072 16S ribosomal RNA, partial sequence | NR_042871.1 | 1364 | 333 | 759 | 31.3 | 726.9 | 0 | 415/427 | 97 | 0/427 | 0 | Plus | Plus | Bacteria; Proteobacteria; Gammaproteobacteria; Pasteurellales; Pasteurellaceae; ; [Actinobacillus] rossii | [Actinobacillus] rossii | | Pasteurellaceae | Pasteurellales | Gammaproteobacteria | Proteobacteria | Bacteria |
| ASV2961 | 402 | 1 | 1 | 402 | 100 | NR_029239.3 Clostridium chartatabidum strain 163 16S ribosomal RNA, partial sequence | NR_029239.3 | 1460 | 323 | 724 | 27.53 | 621.6 | 9E-178 | 380/402 | 94 | 0/402 | 0 | Plus | Plus | Bacteria; Firmicutes; Clostridia; Eubacteriales; Clostridiaceae; Clostridium; Clostridium chartatabidum | Clostridium chartatabidum | Clostridium | Clostridiaceae | Eubacteriales | Clostridia | Firmicutes | Bacteria |
| ASV2963 | 422 | 2 | 1 | 422 | 100 | NR_151886.1 Prevotella colorans strain A1336 16S ribosomal RNA, partial sequence | NR_151886.1 | 1491 | 358 | 779 | 28.3 | 603.1 | 3E-172 | 391/423 | 92 | 2/423 | 0 | Plus | Plus | Bacteria; Bacteroidetes; Bacteroidia; Bacteroidales; Prevotellaceae; Prevotella; Prevotella colorans | Prevotella colorans | Prevotella | Prevotellaceae | Bacteroidales | Bacteroidia | Bacteroidetes | Bacteria |
| ASV2964 | 422 | 4 | 1 | 422 | 100 | NR_044864.1 Hallella seregens ATCC 51272 16S ribosomal RNA, partial sequence | NR_044864.1 | 1463 | 335 | 756 | 28.84 | 603.1 | 3E-172 | 390/422 | 92 | 0/422 | 0 | Plus | Plus | Bacteria; Bacteroidetes; Bacteroidia; Bacteroidales; Prevotellaceae; Prevotella; Hallella seregens | Hallella seregens | Prevotella | Prevotellaceae | Bacteroidales | Bacteroidia | Bacteroidetes | Bacteria |
| ASV2965 | 402 | 1 | 1 | 402 | 100 | NR_159227.1 Monoglobus pectinilyticus strain 14 16S ribosomal RNA, partial sequence | NR_159227.1 | 1493 | 368 | 769 | 26.93 | 538.5 | 9E-153 | 367/404 | 90 | 4/404 | 0 | Plus | Plus | Bacteria; Firmicutes; Clostridia; Eubacteriales; Oscillospiraceae; Monoglobus; Monoglobus pectinilyticus | Monoglobus pectinilyticus | Monoglobus | Oscillospiraceae | Eubacteriales | Clostridia | Firmicutes | Bacteria |
| ASV2966 | 405 | 1 | 1 | 405 | 100 | NR_118554.1 Intestinimonas butyriciproducens strain SRB-521-5-I 16S ribosomal RNA, partial sequence | NR_118554.1 | 1475 | 346 | 750 | 27.46 | 593.9 | 2E-169 | 377/405 | 93 | 0/405 | 0 | Plus | Plus | Bacteria; Firmicutes; Clostridia; Eubacteriales; ; Intestinimonas; Intestinimonas butyriciproducens | Intestinimonas butyriciproducens | Intestinimonas | | Eubacteriales | Clostridia | Firmicutes | Bacteria |
| ASV2967 | 427 | 3 | 15 | 427 | 96.72 | NR_042854.1 Entomoplasma melaleucae strain M1 16S ribosomal RNA, partial sequence | NR_042854.1 | 1494 | 343 | 754 | 27.58 | 424 | 3E-118 | 352/413 | 85 | 1/413 | 0 | Plus | Plus | Bacteria; Tenericutes; Mollicutes; Entomoplasmatales; Entomoplasmataceae; Entomoplasma; Entomoplasma melaleucae | Entomoplasma melaleucae | Entomoplasma | Entomoplasmataceae | Entomoplasmatales | Mollicutes | Tenericutes | Bacteria |
| ASV2968 | 404 | 3 | 1 | 404 | 100 | NR_074793.2 Oscillibacter valericigenes strain Sjm18-20 16S ribosomal RNA, complete sequence | NR_074793.2 | 1530 | 372 | 775 | 26.41 | 564.3 | 2E-160 | 372/405 | 91 | 2/405 | 0 | Plus | Plus | Bacteria; Firmicutes; Clostridia; Eubacteriales; Oscillospiraceae; Oscillibacter; Oscillibacter valericigenes | Oscillibacter valericigenes | Oscillibacter | Oscillospiraceae | Eubacteriales | Clostridia | Firmicutes | Bacteria |
| ASV2969 | 426 | 2 | 1 | 426 | 100 | NR_133868.1 Streptococcus phocae subsp. salmonis strain C-4 16S ribosomal RNA, partial sequence | NR_133868.1 | 1410 | 291 | 717 | 30.28 | 599.4 | 4E-171 | 393/427 | 92 | 1/427 | 0 | Plus | Plus | Bacteria; Firmicutes; Bacilli; Lactobacillales; Streptococcaceae; Streptococcus; Streptococcus phocae | Streptococcus phocae | Streptococcus | Streptococcaceae | Lactobacillales | Bacilli | Firmicutes | Bacteria |
| ASV2970 | 428 | 4 | 3 | 428 | 99.53 | NR_042802.1 Lactobacillus ultunensis strain Kx146C1 16S ribosomal RNA, partial sequence | NR_042802.1 | 1550 | 372 | 796 | 27.42 | 581 | 2E-165 | 392/429 | 91 | 7/429 | 1 | Plus | Plus | Bacteria; Firmicutes; Bacilli; Lactobacillales; Lactobacillaceae; Lactobacillus; Lactobacillus ultunensis | Lactobacillus ultunensis | Lactobacillus | Lactobacillaceae | Lactobacillales | Bacilli | Firmicutes | Bacteria |
| ASV2971 | 357 | 1 | 44 | 357 | 87.96 | NR_042942.1 Treponema porcinum strain 14V28 16S ribosomal RNA, partial sequence | NR_042942.1 | 1426 | 371 | 684 | 22.02 | 575.4 | 6E-164 | 313/314 | 99 | 0/314 | 0 | Plus | Plus | Bacteria; Spirochaetes; Spirochaetia; Spirochaetales; Spirochaetaceae; Treponema; Treponema porcinum | Treponema porcinum | Treponema | Spirochaetaceae | Spirochaetales | Spirochaetia | Spirochaetes | Bacteria |
| ASV2973 | 404 | 1 | 1 | 404 | 100 | NR_042007.1 Roseburia inulinivorans DSM 16841 strain A2-194 16S ribosomal RNA, partial sequence | NR_042007.1 | 1454 | 360 | 763 | 27.79 | 686.2 | 0 | 393/404 | 97 | 0/404 | 0 | Plus | Plus | Bacteria; Firmicutes; Clostridia; Eubacteriales; Lachnospiraceae; Roseburia; Roseburia inulinivorans | Roseburia inulinivorans | Roseburia | Lachnospiraceae | Eubacteriales | Clostridia | Firmicutes | Bacteria |
| ASV2974 | 401 | 3 | 1 | 401 | 100 | NR_041781.1 Streptococcus alactolyticus strain ATCC 43077 16S ribosomal RNA, partial sequence | NR_041781.1 | 1437 | 346 | 772 | 29.71 | 527.4 | 2E-149 | 384/427 | 89 | 26/427 | 6 | Plus | Plus | Bacteria; Firmicutes; Bacilli; Lactobacillales; Streptococcaceae; Streptococcus; Streptococcus alactolyticus | Streptococcus alactolyticus | Streptococcus | Streptococcaceae | Lactobacillales | Bacilli | Firmicutes | Bacteria |
| ASV2976 | 427 | 2 | 1 | 427 | 100 | NR_029106.1 Lactobacillus delbrueckii subsp. indicus strain NCC725 16S ribosomal RNA, partial sequence | NR_029106.1 | 1515 | 348 | 774 | 28.18 | 767.5 | 0 | 423/427 | 99 | 0/427 | 0 | Plus | Plus | Bacteria; Firmicutes; Bacilli; Lactobacillales; Lactobacillaceae; Lactobacillus; Lactobacillus delbrueckii | Lactobacillus delbrueckii | Lactobacillus | Lactobacillaceae | Lactobacillales | Bacilli | Firmicutes | Bacteria |
| ASV2977 | 403 | 1 | 3 | 403 | 99.5 | NR_125464.1 Anaerobacterium chartisolvens strain T-1-35 16S ribosomal RNA, partial sequence | NR_125464.1 | 1451 | 344 | 745 | 27.71 | 492.3 | 7E-139 | 357/402 | 88 | 1/402 | 0 | Plus | Plus | Bacteria; Firmicutes; Clostridia; Eubacteriales; Oscillospiraceae; Anaerobacterium; Anaerobacterium chartisolvens | Anaerobacterium chartisolvens | Anaerobacterium | Oscillospiraceae | Eubacteriales | Clostridia | Firmicutes | Bacteria |
| ASV2978 | 423 | 1 | 1 | 423 | 100 | NR_144750.1 Prevotellamassilia timonensis strain Marseille-P2831 16S ribosomal RNA, partial sequence | NR_144750.1 | 1493 | 358 | 779 | 28.27 | 575.4 | 7E-164 | 388/425 | 91 | 5/425 | 1 | Plus | Plus | Bacteria; Bacteroidetes; Bacteroidia; Bacteroidales; Prevotellaceae; Prevotellamassilia; Prevotellamassilia timonensis | Prevotellamassilia timonensis | Prevotellamassilia | Prevotellaceae | Bacteroidales | Bacteroidia | Bacteroidetes | Bacteria |
| ASV2979 | 427 | 2 | 1 | 427 | 100 | NR_104990.1 Desulfovibrio desulfuricans strain Essex 6 16S ribosomal RNA, partial sequence | NR_104990.1 | 1542 | 357 | 783 | 27.69 | 673.3 | 0 | 407/428 | 95 | 2/428 | 0 | Plus | Plus | Bacteria; Proteobacteria; Deltaproteobacteria; Desulfovibrionales; Desulfovibrionaceae; Desulfovibrio; Desulfovibrio desulfuricans | Desulfovibrio desulfuricans | Desulfovibrio | Desulfovibrionaceae | Desulfovibrionales | Deltaproteobacteria | Proteobacteria | Bacteria |
| ASV2980 | 403 | 1 | 1 | 403 | 100 | NR_144608.1 Cuneatibacter caecimuris strain BARN-424-CC-10 16S ribosomal RNA, partial sequence | NR_144608.1 | 1485 | 365 | 768 | 27.21 | 684.4 | 0 | 394/405 | 97 | 3/405 | 0 | Plus | Plus | Bacteria; Firmicutes; Clostridia; Eubacteriales; Lachnospiraceae; Cuneatibacter; Cuneatibacter caecimuris | Cuneatibacter caecimuris | Cuneatibacter | Lachnospiraceae | Eubacteriales | Clostridia | Firmicutes | Bacteria |
| ASV2981 | 422 | 2 | 1 | 422 | 100 | NR_113119.1 Prevotella oris strain JCM 8540 16S ribosomal RNA, partial sequence | NR_113119.1 | 1481 | 345 | 766 | 28.49 | 581 | 2E-165 | 387/423 | 91 | 2/423 | 0 | Plus | Plus | Bacteria; Bacteroidetes; Bacteroidia; Bacteroidales; Prevotellaceae; Prevotella; Prevotella oris | Prevotella oris | Prevotella | Prevotellaceae | Bacteroidales | Bacteroidia | Bacteroidetes | Bacteria |
| ASV2982 | 405 | 3 | 1 | 405 | 100 | NR_113360.1 Ruminiclostridium cellobioparum DSM 1351 = ATCC 15832 strain JCM 1422 16S ribosomal RNA, partial sequence | NR_113360.1 | 1484 | 356 | 760 | 27.29 | 449.9 | 5E-126 | 354/408 | 86 | 6/408 | 1 | Plus | Plus | Bacteria; Firmicutes; Clostridia; Eubacteriales; Oscillospiraceae; Ruminiclostridium; Ruminiclostridium cellobioparum | Ruminiclostridium cellobioparum | Ruminiclostridium | Oscillospiraceae | Eubacteriales | Clostridia | Firmicutes | Bacteria |
| ASV2983 | 421 | 1 | 1 | 421 | 100 | NR_144606.1 Bacteroides caecimuris strain I48 16S ribosomal RNA, partial sequence | NR_144606.1 | 1517 | 357 | 778 | 27.82 | 446.2 | 6E-125 | 363/423 | 85 | 3/423 | 0 | Plus | Plus | Bacteria; Bacteroidetes; Bacteroidia; Bacteroidales; Bacteroidaceae; Bacteroides; Bacteroides caecimuris | Bacteroides caecimuris | Bacteroides | Bacteroidaceae | Bacteroidales | Bacteroidia | Bacteroidetes | Bacteria |
| ASV2985 | 422 | 1 | 1 | 422 | 100 | NR_041364.1 Prevotella stercorea DSM 18206 strain CB35 16S ribosomal RNA, partial sequence | NR_041364.1 | 1488 | 354 | 775 | 28.36 | 564.3 | 2E-160 | 384/423 | 90 | 2/423 | 0 | Plus | Plus | Bacteria; Bacteroidetes; Bacteroidia; Bacteroidales; Prevotellaceae; Prevotella; Prevotella stercorea | Prevotella stercorea | Prevotella | Prevotellaceae | Bacteroidales | Bacteroidia | Bacteroidetes | Bacteria |
| ASV2986 | 403 | 1 | 1 | 401 | 99.5 | NR_104911.1 Vampirovibrio chlorellavorus strain ICPB 3707 16S ribosomal RNA, partial sequence | NR_104911.1 | 1446 | 333 | 732 | 27.66 | 412.9 | 6E-115 | 343/402 | 85 | 3/402 | 0 | Plus | Plus | Bacteria; Candidatus Melainabacteria; ; Vampirovibrionales; ; Vampirovibrio; Vampirovibrio chlorellavorus | Vampirovibrio chlorellavorus | Vampirovibrio | | Vampirovibrionales | | Candidatus Melainabacteria | Bacteria |
| ASV2987 | 402 | 1 | 1 | 402 | 100 | NR_151895.1 Anaerocolumna cellulosilytica strain SN021 16S ribosomal RNA, partial sequence | NR_151895.1 | 1478 | 353 | 754 | 27.2 | 660.4 | 0 | 389/404 | 96 | 4/404 | 0 | Plus | Plus | Bacteria; Firmicutes; Clostridia; Eubacteriales; Lachnospiraceae; Anaerocolumna; Anaerocolumna cellulosilytica | Anaerocolumna cellulosilytica | Anaerocolumna | Lachnospiraceae | Eubacteriales | Clostridia | Firmicutes | Bacteria |
| ASV2988 | 421 | 2 | 1 | 421 | 100 | NR_113039.1 Solobacterium moorei strain JCM 10645 16S ribosomal RNA, partial sequence | NR_113039.1 | 1486 | 352 | 772 | 28.33 | 562.5 | 6E-160 | 385/424 | 90 | 6/424 | 1 | Plus | Plus | Bacteria; Firmicutes; Erysipelotrichia; Erysipelotrichales; Erysipelotrichaceae; Solobacterium; Solobacterium moorei | Solobacterium moorei | Solobacterium | Erysipelotrichaceae | Erysipelotrichales | Erysipelotrichia | Firmicutes | Bacteria |
| ASV2989 | 405 | 2 | 1 | 405 | 100 | NR_156080.1 Kineothrix alysoides strain KNHs209 16S ribosomal RNA, partial sequence | NR_156080.1 | 1410 | 353 | 754 | 28.51 | 701 | 0 | 397/405 | 98 | 3/405 | 0 | Plus | Plus | Bacteria; Firmicutes; Clostridia; Eubacteriales; Lachnospiraceae; Kineothrix; Kineothrix alysoides | Kineothrix alysoides | Kineothrix | Lachnospiraceae | Eubacteriales | Clostridia | Firmicutes | Bacteria |
| ASV2990 | 428 | 3 | 1 | 428 | 100 | NR_026413.1 Desulfovibrio intestinalis strain KMS2 16S ribosomal RNA, partial sequence | NR_026413.1 | 1509 | 337 | 763 | 28.3 | 606.8 | 3E-173 | 397/430 | 92 | 5/430 | 1 | Plus | Plus | Bacteria; Proteobacteria; Deltaproteobacteria; Desulfovibrionales; Desulfovibrionaceae; Desulfovibrio; Desulfovibrio intestinalis | Desulfovibrio intestinalis | Desulfovibrio | Desulfovibrionaceae | Desulfovibrionales | Deltaproteobacteria | Proteobacteria | Bacteria |
| ASV2991 | 404 | 1 | 1 | 404 | 100 | NR_026179.1 Clostridium acetireducens strain 30A 16S ribosomal RNA, partial sequence | NR_026179.1 | 1502 | 349 | 750 | 26.76 | 457.2 | 3E-128 | 353/405 | 87 | 4/405 | 0 | Plus | Plus | Bacteria; Firmicutes; Clostridia; Eubacteriales; Clostridiaceae; Clostridium; Clostridium acetireducens | Clostridium acetireducens | Clostridium | Clostridiaceae | Eubacteriales | Clostridia | Firmicutes | Bacteria |
| ASV2992 | 403 | 5 | 3 | 403 | 99.5 | NR_115693.1 Gracilibacter thermotolerans JW/YJL-S1 16S ribosomal RNA, partial sequence | NR_115693.1 | 1526 | 461 | 861 | 26.28 | 448 | 2E-125 | 348/401 | 86 | 0/401 | 0 | Plus | Plus | Bacteria; Firmicutes; Clostridia; Eubacteriales; Gracilibacteraceae; Gracilibacter; Gracilibacter thermotolerans | Gracilibacter thermotolerans | Gracilibacter | Gracilibacteraceae | Eubacteriales | Clostridia | Firmicutes | Bacteria |
| ASV2993 | 421 | 1 | 1 | 421 | 100 | NR_144616.1 Muribaculum intestinale strain YL27 16S ribosomal RNA, partial sequence | NR_144616.1 | 1460 | 297 | 718 | 28.9 | 490.5 | 3E-138 | 371/423 | 87 | 3/423 | 0 | Plus | Plus | Bacteria; Bacteroidetes; Bacteroidia; Bacteroidales; Muribaculaceae; Muribaculum; Muribaculum intestinale | Muribaculum intestinale | Muribaculum | Muribaculaceae | Bacteroidales | Bacteroidia | Bacteroidetes | Bacteria |
| ASV2994 | 424 | 1 | 1 | 424 | 100 | NR_144750.1 Prevotellamassilia timonensis strain Marseille-P2831 16S ribosomal RNA, partial sequence | NR_144750.1 | 1493 | 358 | 779 | 28.27 | 477.6 | 2E-134 | 371/426 | 87 | 6/426 | 1 | Plus | Plus | Bacteria; Bacteroidetes; Bacteroidia; Bacteroidales; Prevotellaceae; Prevotellamassilia; Prevotellamassilia timonensis | Prevotellamassilia timonensis | Prevotellamassilia | Prevotellaceae | Bacteroidales | Bacteroidia | Bacteroidetes | Bacteria |
| ASV2995 | 401 | 1 | 1 | 401 | 100 | NR_158111.1 Paludicola psychrotolerans strain NC1253 16S ribosomal RNA, partial sequence | NR_158111.1 | 1490 | 366 | 766 | 26.91 | 636.4 | 0 | 382/401 | 95 | 0/401 | 0 | Plus | Plus | Bacteria; Firmicutes; Clostridia; Eubacteriales; Oscillospiraceae; Paludicola; Paludicola psychrotolerans | Paludicola psychrotolerans | Paludicola | Oscillospiraceae | Eubacteriales | Clostridia | Firmicutes | Bacteria |
| ASV2996 | 404 | 2 | 3 | 404 | 99.5 | NR_024948.1 Campylobacter hyointestinalis subsp. lawsonii strain CHY5 16S ribosomal RNA, partial sequence | NR_024948.1 | 1419 | 320 | 720 | 28.26 | 581 | 2E-165 | 373/402 | 92 | 1/402 | 0 | Plus | Plus | Bacteria; Proteobacteria; Epsilonproteobacteria; Campylobacterales; Campylobacteraceae; Campylobacter; Campylobacter hyointestinalis | Campylobacter hyointestinalis | Campylobacter | Campylobacteraceae | Campylobacterales | Epsilonproteobacteria | Proteobacteria | Bacteria |
| ASV2997 | 422 | 1 | 1 | 422 | 100 | NR_074577.1 Paludibacter propionicigenes WB4 16S ribosomal RNA, partial sequence | NR_074577.1 | 1516 | 353 | 774 | 27.84 | 525.6 | 8E-149 | 379/425 | 89 | 6/425 | 1 | Plus | Plus | Bacteria; Bacteroidetes; Bacteroidia; Bacteroidales; Paludibacteraceae; Paludibacter; Paludibacter propionicigenes | Paludibacter propionicigenes | Paludibacter | Paludibacteraceae | Bacteroidales | Bacteroidia | Bacteroidetes | Bacteria |
| ASV2998 | 402 | 1 | 1 | 402 | 100 | NR_028883.1 Dorea longicatena strain 111-35 16S ribosomal RNA, partial sequence | NR_028883.1 | 1316 | 243 | 645 | 30.62 | 704.7 | 0 | 396/403 | 98 | 1/403 | 0 | Plus | Plus | Bacteria; Firmicutes; Clostridia; Eubacteriales; Lachnospiraceae; Dorea; Dorea longicatena | Dorea longicatena | Dorea | Lachnospiraceae | Eubacteriales | Clostridia | Firmicutes | Bacteria |
| ASV3001 | 422 | 1 | 1 | 422 | 100 | NR_041342.1 Parabacteroides distasonis strain JCM 5825 16S ribosomal RNA, partial sequence | NR_041342.1 | 1488 | 353 | 774 | 28.36 | 558.8 | 8E-159 | 384/424 | 90 | 4/424 | 0 | Plus | Plus | Bacteria; Bacteroidetes; Bacteroidia; Bacteroidales; Tannerellaceae; Parabacteroides; Parabacteroides distasonis | Parabacteroides distasonis | Parabacteroides | Tannerellaceae | Bacteroidales | Bacteroidia | Bacteroidetes | Bacteria |
| ASV3002 | 422 | 1 | 1 | 422 | 100 | NR_113411.1 Prevotella copri DSM 18205 strain JCM 13464 16S ribosomal RNA, partial sequence | NR_113411.1 | 1493 | 358 | 779 | 28.27 | 592 | 7E-169 | 389/423 | 91 | 2/423 | 0 | Plus | Plus | Bacteria; Bacteroidetes; Bacteroidia; Bacteroidales; Prevotellaceae; Prevotella; Prevotella copri | Prevotella copri | Prevotella | Prevotellaceae | Bacteroidales | Bacteroidia | Bacteroidetes | Bacteria |
| ASV3003 | 405 | 1 | 1 | 405 | 100 | NR_044972.1 Sporobacter termitidis strain SYR 16S ribosomal RNA, partial sequence | NR_044972.1 | 1506 | 348 | 752 | 26.89 | 569.9 | 3E-162 | 373/406 | 91 | 2/406 | 0 | Plus | Plus | Bacteria; Firmicutes; Clostridia; Eubacteriales; Oscillospiraceae; Sporobacter; Sporobacter termitidis | Sporobacter termitidis | Sporobacter | Oscillospiraceae | Eubacteriales | Clostridia | Firmicutes | Bacteria |
| ASV3004 | 401 | 1 | 1 | 401 | 100 | NR_024750.1 Coprococcus catus strain VPI-C6-61 16S ribosomal RNA, partial sequence | NR_024750.1 | 1315 | 317 | 719 | 30.65 | 425.9 | 8E-119 | 346/404 | 85 | 4/404 | 0 | Plus | Plus | Bacteria; Firmicutes; Clostridia; Eubacteriales; Lachnospiraceae; Coprococcus; Coprococcus catus | Coprococcus catus | Coprococcus | Lachnospiraceae | Eubacteriales | Clostridia | Firmicutes | Bacteria |
| ASV3005 | 422 | 2 | 1 | 422 | 100 | NR_113117.1 Prevotella oralis ATCC 33269 strain JCM 12251 16S ribosomal RNA, partial sequence | NR_113117.1 | 1496 | 361 | 782 | 28.21 | 603.1 | 3E-172 | 391/423 | 92 | 2/423 | 0 | Plus | Plus | Bacteria; Bacteroidetes; Bacteroidia; Bacteroidales; Prevotellaceae; Prevotella; Prevotella oralis | Prevotella oralis | Prevotella | Prevotellaceae | Bacteroidales | Bacteroidia | Bacteroidetes | Bacteria |
| ASV3006 | 422 | 1 | 1 | 422 | 100 | NR_024815.1 Prevotella shahii strain EHS11 16S ribosomal RNA, partial sequence | NR_024815.1 | 1490 | 356 | 777 | 28.32 | 603.1 | 3E-172 | 392/424 | 92 | 4/424 | 0 | Plus | Plus | Bacteria; Bacteroidetes; Bacteroidia; Bacteroidales; Prevotellaceae; Prevotella; Prevotella shahii | Prevotella shahii | Prevotella | Prevotellaceae | Bacteroidales | Bacteroidia | Bacteroidetes | Bacteria |
| ASV3007 | 405 | 1 | 1 | 405 | 100 | NR_148302.1 Geosporobacter ferrireducens strain IRF9 16S ribosomal RNA, partial sequence | NR_148302.1 | 1392 | 306 | 707 | 28.88 | 473.9 | 3E-133 | 358/407 | 87 | 7/407 | 1 | Plus | Plus | Bacteria; Firmicutes; Clostridia; Eubacteriales; Clostridiaceae; Geosporobacter; Geosporobacter ferrireducens | Geosporobacter ferrireducens | Geosporobacter | Clostridiaceae | Eubacteriales | Clostridia | Firmicutes | Bacteria |
| ASV3008 | 402 | 1 | 1 | 401 | 99.75 | NR_159227.1 Monoglobus pectinilyticus strain 14 16S ribosomal RNA, partial sequence | NR_159227.1 | 1493 | 368 | 768 | 26.86 | 531.1 | 2E-150 | 367/405 | 90 | 8/405 | 1 | Plus | Plus | Bacteria; Firmicutes; Clostridia; Eubacteriales; Oscillospiraceae; Monoglobus; Monoglobus pectinilyticus | Monoglobus pectinilyticus | Monoglobus | Oscillospiraceae | Eubacteriales | Clostridia | Firmicutes | Bacteria |
| ASV3009 | 421 | 2 | 1 | 421 | 100 | NR_117779.1 Anaerorhabdus furcosa 16S ribosomal RNA, partial sequence | NR_117779.1 | 1488 | 350 | 770 | 28.29 | 562.5 | 6E-160 | 384/423 | 90 | 4/423 | 0 | Plus | Plus | Bacteria; Firmicutes; Erysipelotrichia; Erysipelotrichales; Erysipelotrichaceae; Anaerorhabdus; Anaerorhabdus furcosa | Anaerorhabdus furcosa | Anaerorhabdus | Erysipelotrichaceae | Erysipelotrichales | Erysipelotrichia | Firmicutes | Bacteria |
| ASV3010 | 427 | 3 | 1 | 427 | 100 | NR_114852.1 Streptococcus ferus strain 8S1 16S ribosomal RNA, partial sequence | NR_114852.1 | 1444 | 347 | 773 | 29.57 | 789.6 | 0 | 427/427 | 100 | 0/427 | 0 | Plus | Plus | Bacteria; Firmicutes; Bacilli; Lactobacillales; Streptococcaceae; Streptococcus; Streptococcus ferus | Streptococcus ferus | Streptococcus | Streptococcaceae | Lactobacillales | Bacilli | Firmicutes | Bacteria |
| ASV3011 | 421 | 1 | 1 | 421 | 100 | NR_144616.1 Muribaculum intestinale strain YL27 16S ribosomal RNA, partial sequence | NR_144616.1 | 1460 | 297 | 718 | 28.9 | 595.7 | 6E-170 | 392/425 | 92 | 7/425 | 1 | Plus | Plus | Bacteria; Bacteroidetes; Bacteroidia; Bacteroidales; Muribaculaceae; Muribaculum; Muribaculum intestinale | Muribaculum intestinale | Muribaculum | Muribaculaceae | Bacteroidales | Bacteroidia | Bacteroidetes | Bacteria |
| ASV3012 | 423 | 1 | 1 | 423 | 100 | NR_041364.1 Prevotella stercorea DSM 18206 strain CB35 16S ribosomal RNA, partial sequence | NR_041364.1 | 1488 | 354 | 775 | 28.36 | 542.2 | 8E-154 | 381/424 | 89 | 3/424 | 0 | Plus | Plus | Bacteria; Bacteroidetes; Bacteroidia; Bacteroidales; Prevotellaceae; Prevotella; Prevotella stercorea | Prevotella stercorea | Prevotella | Prevotellaceae | Bacteroidales | Bacteroidia | Bacteroidetes | Bacteria |
| ASV3013 | 405 | 1 | 1 | 405 | 100 | NR_118554.1 Intestinimonas butyriciproducens strain SRB-521-5-I 16S ribosomal RNA, partial sequence | NR_118554.1 | 1475 | 346 | 750 | 27.46 | 488.6 | 1E-137 | 359/406 | 88 | 2/406 | 0 | Plus | Plus | Bacteria; Firmicutes; Clostridia; Eubacteriales; ; Intestinimonas; Intestinimonas butyriciproducens | Intestinimonas butyriciproducens | Intestinimonas | | Eubacteriales | Clostridia | Firmicutes | Bacteria |
| ASV3014 | 422 | 2 | 1 | 422 | 100 | NR_113065.1 Bacteroides clarus YIT 12056 strain JCM 16067 16S ribosomal RNA, partial sequence | NR_113065.1 | 1488 | 355 | 776 | 28.36 | 536.6 | 4E-152 | 382/426 | 89 | 8/426 | 1 | Plus | Plus | Bacteria; Bacteroidetes; Bacteroidia; Bacteroidales; Bacteroidaceae; Bacteroides; Bacteroides clarus | Bacteroides clarus | Bacteroides | Bacteroidaceae | Bacteroidales | Bacteroidia | Bacteroidetes | Bacteria |
| ASV3015 | 402 | 4 | 1 | 402 | 100 | NR_118128.1 [Clostridium] clostridioforme strain ATCC 25537 16S ribosomal RNA, partial sequence | NR_118128.1 | 1310 | 191 | 592 | 30.69 | 643.8 | 0 | 386/404 | 95 | 4/404 | 0 | Plus | Plus | Bacteria; Firmicutes; Clostridia; Eubacteriales; Lachnospiraceae; Enterocloster; Enterocloster clostridioformis | Enterocloster clostridioformis | Enterocloster | Lachnospiraceae | Eubacteriales | Clostridia | Firmicutes | Bacteria |
| ASV3017 | 422 | 4 | 1 | 422 | 100 | NR_043016.1 Bacteroides salyersiae strain WAL 10018 16S ribosomal RNA, partial sequence | NR_043016.1 | 1419 | 333 | 754 | 29.74 | 564.3 | 2E-160 | 383/422 | 90 | 0/422 | 0 | Plus | Plus | Bacteria; Bacteroidetes; Bacteroidia; Bacteroidales; Bacteroidaceae; Bacteroides; Bacteroides salyersiae | Bacteroides salyersiae | Bacteroides | Bacteroidaceae | Bacteroidales | Bacteroidia | Bacteroidetes | Bacteria |
| ASV3018 | 427 | 1 | 1 | 427 | 100 | NR_041611.1 Lactobacillus ruminis strain NBRC 102161 16S ribosomal RNA, partial sequence | NR_041611.1 | 1488 | 352 | 778 | 28.7 | 784.1 | 0 | 426/427 | 99 | 0/427 | 0 | Plus | Plus | Bacteria; Firmicutes; Bacilli; Lactobacillales; Lactobacillaceae; Ligilactobacillus; Ligilactobacillus ruminis | Ligilactobacillus ruminis | Ligilactobacillus | Lactobacillaceae | Lactobacillales | Bacilli | Firmicutes | Bacteria |
| ASV3019 | 422 | 1 | 1 | 422 | 100 | NR_113411.1 Prevotella copri DSM 18205 strain JCM 13464 16S ribosomal RNA, partial sequence | NR_113411.1 | 1493 | 358 | 779 | 28.27 | 592 | 7E-169 | 388/422 | 91 | 0/422 | 0 | Plus | Plus | Bacteria; Bacteroidetes; Bacteroidia; Bacteroidales; Prevotellaceae; Prevotella; Prevotella copri | Prevotella copri | Prevotella | Prevotellaceae | Bacteroidales | Bacteroidia | Bacteroidetes | Bacteria |
| ASV3020 | 402 | 1 | 1 | 402 | 100 | NR_159227.1 Monoglobus pectinilyticus strain 14 16S ribosomal RNA, partial sequence | NR_159227.1 | 1493 | 368 | 769 | 26.93 | 654.8 | 0 | 387/403 | 96 | 2/403 | 0 | Plus | Plus | Bacteria; Firmicutes; Clostridia; Eubacteriales; Oscillospiraceae; Monoglobus; Monoglobus pectinilyticus | Monoglobus pectinilyticus | Monoglobus | Oscillospiraceae | Eubacteriales | Clostridia | Firmicutes | Bacteria |
| ASV3022 | 405 | 1 | 1 | 405 | 100 | NR_044972.1 Sporobacter termitidis strain SYR 16S ribosomal RNA, partial sequence | NR_044972.1 | 1506 | 348 | 752 | 26.89 | 575.4 | 7E-164 | 374/406 | 92 | 2/406 | 0 | Plus | Plus | Bacteria; Firmicutes; Clostridia; Eubacteriales; Oscillospiraceae; Sporobacter; Sporobacter termitidis | Sporobacter termitidis | Sporobacter | Oscillospiraceae | Eubacteriales | Clostridia | Firmicutes | Bacteria |
| ASV3023 | 405 | 1 | 1 | 405 | 100 | NR_044972.1 Sporobacter termitidis strain SYR 16S ribosomal RNA, partial sequence | NR_044972.1 | 1506 | 348 | 752 | 26.89 | 592 | 7E-169 | 377/406 | 92 | 2/406 | 0 | Plus | Plus | Bacteria; Firmicutes; Clostridia; Eubacteriales; Oscillospiraceae; Sporobacter; Sporobacter termitidis | Sporobacter termitidis | Sporobacter | Oscillospiraceae | Eubacteriales | Clostridia | Firmicutes | Bacteria |
| ASV3024 | 402 | 1 | 1 | 402 | 100 | NR_144727.1 Anaeromassilibacillus senegalensis strain mt9 16S ribosomal RNA, partial sequence | NR_144727.1 | 1478 | 352 | 753 | 27.2 | 549.6 | 4E-156 | 371/406 | 91 | 8/406 | 1 | Plus | Plus | Bacteria; Firmicutes; Clostridia; Eubacteriales; Oscillospiraceae; Anaeromassilibacillus; Anaeromassilibacillus senegalensis | Anaeromassilibacillus senegalensis | Anaeromassilibacillus | Oscillospiraceae | Eubacteriales | Clostridia | Firmicutes | Bacteria |
| ASV3026 | 422 | 2 | 1 | 422 | 100 | NR_113119.1 Prevotella oris strain JCM 8540 16S ribosomal RNA, partial sequence | NR_113119.1 | 1481 | 345 | 766 | 28.49 | 608.7 | 7E-174 | 393/424 | 92 | 4/424 | 0 | Plus | Plus | Bacteria; Bacteroidetes; Bacteroidia; Bacteroidales; Prevotellaceae; Prevotella; Prevotella oris | Prevotella oris | Prevotella | Prevotellaceae | Bacteroidales | Bacteroidia | Bacteroidetes | Bacteria |
| ASV3027 | 402 | 1 | 1 | 402 | 100 | NR_109636.1 Cloacibacillus porcorum strain CL-84 16S ribosomal RNA, partial sequence | NR_109636.1 | 1447 | 320 | 721 | 27.78 | 732.4 | 0 | 400/402 | 99 | 0/402 | 0 | Plus | Plus | Bacteria; Synergistetes; Synergistia; Synergistales; Synergistaceae; Cloacibacillus; Cloacibacillus porcorum | Cloacibacillus porcorum | Cloacibacillus | Synergistaceae | Synergistales | Synergistia | Synergistetes | Bacteria |
| ASV3030 | 402 | 3 | 1 | 402 | 100 | NR_112169.1 Clostridium perfringens ATCC 13124 16S ribosomal RNA, partial sequence | NR_112169.1 | 1460 | 324 | 725 | 27.53 | 743.5 | 0 | 402/402 | 100 | 0/402 | 0 | Plus | Plus | Bacteria; Firmicutes; Clostridia; Eubacteriales; Clostridiaceae; Clostridium; Clostridium perfringens | Clostridium perfringens | Clostridium | Clostridiaceae | Eubacteriales | Clostridia | Firmicutes | Bacteria |
| ASV3031 | 405 | 1 | 1 | 405 | 100 | NR_118554.1 Intestinimonas butyriciproducens strain SRB-521-5-I 16S ribosomal RNA, partial sequence | NR_118554.1 | 1475 | 346 | 750 | 27.46 | 494.2 | 2E-139 | 359/405 | 88 | 0/405 | 0 | Plus | Plus | Bacteria; Firmicutes; Clostridia; Eubacteriales; ; Intestinimonas; Intestinimonas butyriciproducens | Intestinimonas butyriciproducens | Intestinimonas | | Eubacteriales | Clostridia | Firmicutes | Bacteria |
| ASV3032 | 405 | 1 | 1 | 405 | 100 | NR_114789.1 [Clostridium] leptum strain DSM 753 16S ribosomal RNA, partial sequence | NR_114789.1 | 1505 | 364 | 765 | 26.71 | 518.2 | 1E-146 | 364/405 | 89 | 3/405 | 0 | Plus | Plus | Bacteria; Firmicutes; Clostridia; Eubacteriales; Oscillospiraceae; ; [Clostridium] leptum | [Clostridium] leptum | | Oscillospiraceae | Eubacteriales | Clostridia | Firmicutes | Bacteria |
| ASV3033 | 402 | 1 | 1 | 402 | 100 | NR_028883.1 Dorea longicatena strain 111-35 16S ribosomal RNA, partial sequence | NR_028883.1 | 1316 | 243 | 645 | 30.62 | 732.4 | 0 | 401/403 | 99 | 1/403 | 0 | Plus | Plus | Bacteria; Firmicutes; Clostridia; Eubacteriales; Lachnospiraceae; Dorea; Dorea longicatena | Dorea longicatena | Dorea | Lachnospiraceae | Eubacteriales | Clostridia | Firmicutes | Bacteria |
| ASV3037 | 403 | 2 | 1 | 403 | 100 | NR_113188.1 Slackia isoflavoniconvertens strain JCM 16137 16S ribosomal RNA, partial sequence | NR_113188.1 | 1473 | 348 | 750 | 27.36 | 745.3 | 0 | 403/403 | 100 | 0/403 | 0 | Plus | Plus | Bacteria; Actinobacteria; Coriobacteriia; Eggerthellales; Eggerthellaceae; Slackia; Slackia isoflavoniconvertens | Slackia isoflavoniconvertens | Slackia | Eggerthellaceae | Eggerthellales | Coriobacteriia | Actinobacteria | Bacteria |
| ASV3039 | 405 | 1 | 3 | 405 | 99.51 | NR_108636.1 Natranaerovirga pectinivora strain AP3 16S ribosomal RNA, partial sequence | NR_108636.1 | 1497 | 367 | 766 | 26.72 | 481.2 | 2E-135 | 356/403 | 88 | 3/403 | 0 | Plus | Plus | Bacteria; Firmicutes; Clostridia; Eubacteriales; ; Natranaerovirga; Natranaerovirga pectinivora | Natranaerovirga pectinivora | Natranaerovirga | | Eubacteriales | Clostridia | Firmicutes | Bacteria |
| ASV3040 | 402 | 2 | 1 | 402 | 100 | NR_137405.1 Anaerobium acetethylicum strain GluBS11 16S ribosomal RNA, partial sequence | NR_137405.1 | 1402 | 324 | 725 | 28.67 | 654.8 | 0 | 386/402 | 96 | 0/402 | 0 | Plus | Plus | Bacteria; Firmicutes; Clostridia; Eubacteriales; Lachnospiraceae; Anaerobium; Anaerobium acetethylicum | Anaerobium acetethylicum | Anaerobium | Lachnospiraceae | Eubacteriales | Clostridia | Firmicutes | Bacteria |
| ASV3041 | 403 | 1 | 3 | 403 | 99.5 | NR_125464.1 Anaerobacterium chartisolvens strain T-1-35 16S ribosomal RNA, partial sequence | NR_125464.1 | 1451 | 344 | 745 | 27.71 | 475.7 | 7E-134 | 354/402 | 88 | 1/402 | 0 | Plus | Plus | Bacteria; Firmicutes; Clostridia; Eubacteriales; Oscillospiraceae; Anaerobacterium; Anaerobacterium chartisolvens | Anaerobacterium chartisolvens | Anaerobacterium | Oscillospiraceae | Eubacteriales | Clostridia | Firmicutes | Bacteria |
| ASV3042 | 421 | 3 | 1 | 421 | 100 | NR_121773.2 Barnesiella viscericola strain C46 16S ribosomal RNA, complete sequence | NR_121773.2 | 1532 | 365 | 786 | 27.55 | 473.9 | 3E-133 | 369/424 | 87 | 5/424 | 1 | Plus | Plus | Bacteria; Bacteroidetes; Bacteroidia; Bacteroidales; Barnesiellaceae; Barnesiella; Barnesiella viscericola | Barnesiella viscericola | Barnesiella | Barnesiellaceae | Bacteroidales | Bacteroidia | Bacteroidetes | Bacteria |
| ASV3043 | 421 | 1 | 1 | 421 | 100 | NR_041343.1 Parabacteroides merdae strain JCM 9497 16S ribosomal RNA, partial sequence | NR_041343.1 | 1492 | 357 | 778 | 28.28 | 512.6 | 6E-145 | 375/423 | 88 | 3/423 | 0 | Plus | Plus | Bacteria; Bacteroidetes; Bacteroidia; Bacteroidales; Tannerellaceae; Parabacteroides; Parabacteroides merdae | Parabacteroides merdae | Parabacteroides | Tannerellaceae | Bacteroidales | Bacteroidia | Bacteroidetes | Bacteria |
| ASV3044 | 405 | 1 | 1 | 405 | 100 | NR_118554.1 Intestinimonas butyriciproducens strain SRB-521-5-I 16S ribosomal RNA, partial sequence | NR_118554.1 | 1475 | 346 | 750 | 27.46 | 588.4 | 9E-168 | 376/405 | 92 | 0/405 | 0 | Plus | Plus | Bacteria; Firmicutes; Clostridia; Eubacteriales; ; Intestinimonas; Intestinimonas butyriciproducens | Intestinimonas butyriciproducens | Intestinimonas | | Eubacteriales | Clostridia | Firmicutes | Bacteria |
| ASV3045 | 425 | 5 | 1 | 425 | 100 | NR_146813.2 Erysipelothrix larvae strain LV19 16S ribosomal RNA, complete sequence | NR_146813.2 | 1543 | 367 | 792 | 27.61 | 436.9 | 4E-122 | 366/429 | 85 | 7/429 | 1 | Plus | Plus | Bacteria; Firmicutes; Erysipelotrichia; Erysipelotrichales; Erysipelotrichaceae; Erysipelothrix; Erysipelothrix larvae | Erysipelothrix larvae | Erysipelothrix | Erysipelotrichaceae | Erysipelotrichales | Erysipelotrichia | Firmicutes | Bacteria |
| ASV3046 | 405 | 1 | 1 | 405 | 100 | NR_025025.1 Papillibacter cinnamivorans strain CIN1 16S ribosomal RNA, partial sequence | NR_025025.1 | 1491 | 349 | 751 | 27.03 | 614.2 | 2E-175 | 381/406 | 93 | 4/406 | 0 | Plus | Plus | Bacteria; Firmicutes; Clostridia; Eubacteriales; Oscillospiraceae; Papillibacter; Papillibacter cinnamivorans | Papillibacter cinnamivorans | Papillibacter | Oscillospiraceae | Eubacteriales | Clostridia | Firmicutes | Bacteria |
| ASV3047 | 422 | 4 | 1 | 422 | 100 | NR_025300.1 Prevotella albensis strain M384 16S ribosomal RNA, partial sequence | NR_025300.1 | 1495 | 358 | 779 | 28.23 | 603.1 | 3E-172 | 391/423 | 92 | 2/423 | 0 | Plus | Plus | Bacteria; Bacteroidetes; Bacteroidia; Bacteroidales; Prevotellaceae; Prevotella; Prevotella albensis | Prevotella albensis | Prevotella | Prevotellaceae | Bacteroidales | Bacteroidia | Bacteroidetes | Bacteria |
| ASV3048 | 427 | 4 | 3 | 427 | 99.53 | NR_041781.1 Streptococcus alactolyticus strain ATCC 43077 16S ribosomal RNA, partial sequence | NR_041781.1 | 1437 | 348 | 772 | 29.58 | 536.6 | 4E-152 | 384/429 | 89 | 8/429 | 1 | Plus | Plus | Bacteria; Firmicutes; Bacilli; Lactobacillales; Streptococcaceae; Streptococcus; Streptococcus alactolyticus | Streptococcus alactolyticus | Streptococcus | Streptococcaceae | Lactobacillales | Bacilli | Firmicutes | Bacteria |
| ASV3049 | 405 | 1 | 1 | 405 | 100 | NR_159227.1 Monoglobus pectinilyticus strain 14 16S ribosomal RNA, partial sequence | NR_159227.1 | 1493 | 368 | 769 | 26.93 | 545.9 | 6E-155 | 372/408 | 91 | 9/408 | 2 | Plus | Plus | Bacteria; Firmicutes; Clostridia; Eubacteriales; Oscillospiraceae; Monoglobus; Monoglobus pectinilyticus | Monoglobus pectinilyticus | Monoglobus | Oscillospiraceae | Eubacteriales | Clostridia | Firmicutes | Bacteria |
| ASV3050 | 402 | 1 | 1 | 402 | 100 | NR_118674.1 Eubacterium ramulus ATCC 29099 16S ribosomal RNA, partial sequence | NR_118674.1 | 1474 | 373 | 774 | 27.27 | 693.6 | 0 | 390/402 | 97 | 0/402 | 0 | Plus | Plus | Bacteria; Firmicutes; Clostridia; Eubacteriales; Eubacteriaceae; Eubacterium; Eubacterium ramulus | Eubacterium ramulus | Eubacterium | Eubacteriaceae | Eubacteriales | Clostridia | Firmicutes | Bacteria |
| ASV3051 | 402 | 1 | 1 | 402 | 100 | NR_151894.1 Anaerotaenia torta strain FH052 16S ribosomal RNA, partial sequence | NR_151894.1 | 1479 | 352 | 753 | 27.18 | 638.2 | 0 | 383/402 | 95 | 0/402 | 0 | Plus | Plus | Bacteria; Firmicutes; Clostridia; Eubacteriales; Lachnospiraceae; Anaerotaenia; Anaerotaenia torta | Anaerotaenia torta | Anaerotaenia | Lachnospiraceae | Eubacteriales | Clostridia | Firmicutes | Bacteria |
| ASV3052 | 402 | 1 | 1 | 402 | 100 | NR_026074.1 Helicobacter rodentium strain MIT 95-1707 16S ribosomal RNA, partial sequence | NR_026074.1 | 1423 | 317 | 718 | 28.25 | 737.9 | 0 | 401/402 | 99 | 0/402 | 0 | Plus | Plus | Bacteria; Proteobacteria; Epsilonproteobacteria; Campylobacterales; Helicobacteraceae; Helicobacter; Helicobacter rodentium | Helicobacter rodentium | Helicobacter | Helicobacteraceae | Campylobacterales | Epsilonproteobacteria | Proteobacteria | Bacteria |
| ASV3053 | 401 | 2 | 1 | 401 | 100 | NR_113383.1 Finegoldia magna strain JCM 1766 16S ribosomal RNA, partial sequence | NR_113383.1 | 1483 | 364 | 764 | 27.04 | 736.1 | 0 | 400/401 | 99 | 0/401 | 0 | Plus | Plus | Bacteria; Firmicutes; Tissierellia; Tissierellales; Peptoniphilaceae; Finegoldia; Finegoldia magna | Finegoldia magna | Finegoldia | Peptoniphilaceae | Tissierellales | Tissierellia | Firmicutes | Bacteria |
| ASV3054 | 427 | 3 | 1 | 427 | 100 | NR_036918.1 Streptococcus suis strain S735 16S ribosomal RNA, partial sequence | NR_036918.1 | 1535 | 354 | 780 | 27.82 | 784.1 | 0 | 426/427 | 99 | 0/427 | 0 | Plus | Plus | Bacteria; Firmicutes; Bacilli; Lactobacillales; Streptococcaceae; Streptococcus; Streptococcus suis | Streptococcus suis | Streptococcus | Streptococcaceae | Lactobacillales | Bacilli | Firmicutes | Bacteria |
| ASV3057 | 402 | 1 | 1 | 402 | 100 | NR_118675.1 [Eubacterium] siraeum strain ATCC 29066 16S ribosomal RNA, partial sequence | NR_118675.1 | 1453 | 356 | 757 | 27.67 | 584.7 | 1E-166 | 372/402 | 92 | 0/402 | 0 | Plus | Plus | Bacteria; Firmicutes; Clostridia; Eubacteriales; Oscillospiraceae; ; [Eubacterium] siraeum | [Eubacterium] siraeum | | Oscillospiraceae | Eubacteriales | Clostridia | Firmicutes | Bacteria |
| ASV3058 | 405 | 1 | 1 | 405 | 100 | NR_115269.1 Catabacter hongkongensis strain HKU16 16S ribosomal RNA, partial sequence | NR_115269.1 | 1453 | 349 | 753 | 27.87 | 422.2 | 1E-117 | 346/405 | 85 | 0/405 | 0 | Plus | Plus | Bacteria; Firmicutes; Clostridia; Eubacteriales; Christensenellaceae; Christensenella; Christensenella hongkongensis | Christensenella hongkongensis | Christensenella | Christensenellaceae | Eubacteriales | Clostridia | Firmicutes | Bacteria |
| ASV3059 | 422 | 1 | 1 | 422 | 100 | NR_112943.1 Bacteroides stercoris ATCC 43183 strain JCM 9496 16S ribosomal RNA, partial sequence | NR_112943.1 | 1476 | 343 | 764 | 28.59 | 653 | 0 | 399/422 | 94 | 0/422 | 0 | Plus | Plus | Bacteria; Bacteroidetes; Bacteroidia; Bacteroidales; Bacteroidaceae; Bacteroides; Bacteroides stercoris | Bacteroides stercoris | Bacteroides | Bacteroidaceae | Bacteroidales | Bacteroidia | Bacteroidetes | Bacteria |
| ASV3060 | 427 | 2 | 1 | 427 | 100 | NR_040837.1 Erysipelothrix rhusiopathiae strain ATCC 19414 16S ribosomal RNA, partial sequence | NR_040837.1 | 1497 | 356 | 781 | 28.46 | 455.4 | 1E-127 | 371/431 | 86 | 9/431 | 2 | Plus | Plus | Bacteria; Firmicutes; Erysipelotrichia; Erysipelotrichales; Erysipelotrichaceae; Erysipelothrix; Erysipelothrix rhusiopathiae | Erysipelothrix rhusiopathiae | Erysipelothrix | Erysipelotrichaceae | Erysipelotrichales | Erysipelotrichia | Firmicutes | Bacteria |
| ASV3062 | 422 | 1 | 1 | 422 | 100 | NR_041285.1 Prevotella multisaccharivorax strain PPPA20 16S ribosomal RNA, partial sequence | NR_041285.1 | 1491 | 356 | 777 | 28.3 | 603.1 | 3E-172 | 390/422 | 92 | 0/422 | 0 | Plus | Plus | Bacteria; Bacteroidetes; Bacteroidia; Bacteroidales; Prevotellaceae; Prevotella; Prevotella multisaccharivorax | Prevotella multisaccharivorax | Prevotella | Prevotellaceae | Bacteroidales | Bacteroidia | Bacteroidetes | Bacteria |
| ASV3063 | 402 | 1 | 1 | 393 | 97.76 | NR_118673.1 [Eubacterium] hallii strain ATCC 27751 16S ribosomal RNA, partial sequence | NR_118673.1 | 1479 | 371 | 763 | 26.57 | 424 | 3E-118 | 338/394 | 85 | 2/394 | 0 | Plus | Plus | Bacteria; Firmicutes; Clostridia; Eubacteriales; Lachnospiraceae; Anaerobutyricum; Anaerobutyricum hallii | Anaerobutyricum hallii | Anaerobutyricum | Lachnospiraceae | Eubacteriales | Clostridia | Firmicutes | Bacteria |
| ASV3064 | 402 | 1 | 1 | 402 | 100 | NR_152059.1 Harryflintia acetispora strain V20-281a 16S ribosomal RNA, partial sequence | NR_152059.1 | 1363 | 289 | 692 | 29.64 | 579.1 | 5E-165 | 376/406 | 92 | 6/406 | 1 | Plus | Plus | Bacteria; Firmicutes; Clostridia; Eubacteriales; Oscillospiraceae; Harryflintia; Harryflintia acetispora | Harryflintia acetispora | Harryflintia | Oscillospiraceae | Eubacteriales | Clostridia | Firmicutes | Bacteria |
| ASV3065 | 402 | 2 | 1 | 402 | 100 | NR_026204.1 [Clostridium] viride strain T2-7 16S ribosomal RNA, partial sequence | NR_026204.1 | 1482 | 345 | 749 | 27.33 | 520 | 3E-147 | 365/406 | 89 | 5/406 | 1 | Plus | Plus | Bacteria; Firmicutes; Clostridia; Eubacteriales; Oscillospiraceae; ; [Clostridium] viride | [Clostridium] viride | | Oscillospiraceae | Eubacteriales | Clostridia | Firmicutes | Bacteria |
| ASV3066 | 402 | 1 | 1 | 402 | 100 | NR_102884.1 Ruminococcus champanellensis 18P13 = JCM 17042 16S ribosomal RNA, partial sequence | NR_102884.1 | 1494 | 348 | 749 | 26.91 | 605 | 9E-173 | 379/404 | 93 | 4/404 | 0 | Plus | Plus | Bacteria; Firmicutes; Clostridia; Eubacteriales; Oscillospiraceae; Ruminococcus; Ruminococcus champanellensis | Ruminococcus champanellensis | Ruminococcus | Oscillospiraceae | Eubacteriales | Clostridia | Firmicutes | Bacteria |
| ASV3068 | 405 | 2 | 1 | 405 | 100 | NR_144742.1 Christensenella massiliensis strain Marseille-P2438 16S ribosomal RNA, partial sequence | NR_144742.1 | 1499 | 369 | 773 | 27.02 | 460.9 | 2E-129 | 354/406 | 87 | 2/406 | 0 | Plus | Plus | Bacteria; Firmicutes; Clostridia; Eubacteriales; Christensenellaceae; Christensenella; Christensenella massiliensis | Christensenella massiliensis | Christensenella | Christensenellaceae | Eubacteriales | Clostridia | Firmicutes | Bacteria |
| ASV3069 | 427 | 1 | 1 | 427 | 100 | NR_026413.1 Desulfovibrio intestinalis strain KMS2 16S ribosomal RNA, partial sequence | NR_026413.1 | 1509 | 337 | 763 | 28.3 | 651.1 | 0 | 402/427 | 94 | 0/427 | 0 | Plus | Plus | Bacteria; Proteobacteria; Deltaproteobacteria; Desulfovibrionales; Desulfovibrionaceae; Desulfovibrio; Desulfovibrio intestinalis | Desulfovibrio intestinalis | Desulfovibrio | Desulfovibrionaceae | Desulfovibrionales | Deltaproteobacteria | Proteobacteria | Bacteria |
| ASV3070 | 403 | 4 | 1 | 403 | 100 | NR_074809.1 Roseburia hominis A2-183 16S ribosomal RNA, partial sequence | NR_074809.1 | 1535 | 367 | 769 | 26.25 | 723.2 | 0 | 399/403 | 99 | 0/403 | 0 | Plus | Plus | Bacteria; Firmicutes; Clostridia; Eubacteriales; Lachnospiraceae; Roseburia; Roseburia hominis | Roseburia hominis | Roseburia | Lachnospiraceae | Eubacteriales | Clostridia | Firmicutes | Bacteria |
| ASV3072 | 422 | 1 | 1 | 422 | 100 | NR_113411.1 Prevotella copri DSM 18205 strain JCM 13464 16S ribosomal RNA, partial sequence | NR_113411.1 | 1493 | 358 | 779 | 28.27 | 581 | 2E-165 | 386/422 | 91 | 0/422 | 0 | Plus | Plus | Bacteria; Bacteroidetes; Bacteroidia; Bacteroidales; Prevotellaceae; Prevotella; Prevotella copri | Prevotella copri | Prevotella | Prevotellaceae | Bacteroidales | Bacteroidia | Bacteroidetes | Bacteria |
| ASV3073 | 402 | 2 | 1 | 402 | 100 | NR_113231.1 Blautia glucerasea strain JCM 17039 16S ribosomal RNA, partial sequence | NR_113231.1 | 1491 | 364 | 765 | 26.96 | 715.8 | 0 | 397/402 | 98 | 0/402 | 0 | Plus | Plus | Bacteria; Firmicutes; Clostridia; Eubacteriales; Lachnospiraceae; Blautia; Blautia glucerasea | Blautia glucerasea | Blautia | Lachnospiraceae | Eubacteriales | Clostridia | Firmicutes | Bacteria |
| ASV3074 | 422 | 1 | 1 | 422 | 100 | NR_041446.1 Bacteroides barnesiae strain BL2 16S ribosomal RNA, partial sequence | NR_041446.1 | 1456 | 340 | 761 | 28.98 | 569.9 | 3E-162 | 386/424 | 91 | 4/424 | 0 | Plus | Plus | Bacteria; Bacteroidetes; Bacteroidia; Bacteroidales; ; Phocaeicola; Phocaeicola barnesiae | Phocaeicola barnesiae | Phocaeicola | | Bacteroidales | Bacteroidia | Bacteroidetes | Bacteria |
| ASV3075 | 428 | 1 | 1 | 428 | 100 | NR_104781.1 Treponema bryantii strain RUS-1 16S ribosomal RNA, partial sequence | NR_104781.1 | 1535 | 351 | 778 | 27.88 | 641.9 | 0 | 401/428 | 93 | 0/428 | 0 | Plus | Plus | Bacteria; Spirochaetes; Spirochaetia; Spirochaetales; Spirochaetaceae; Treponema; Treponema bryantii | Treponema bryantii | Treponema | Spirochaetaceae | Spirochaetales | Spirochaetia | Spirochaetes | Bacteria |
| ASV3076 | 405 | 1 | 1 | 405 | 100 | NR_042834.1 Alkalibacter saccharofermentans strain Z-79820 16S ribosomal RNA, partial sequence | NR_042834.1 | 1465 | 338 | 741 | 27.58 | 453.5 | 3E-127 | 353/406 | 86 | 3/406 | 0 | Plus | Plus | Bacteria; Firmicutes; Clostridia; Eubacteriales; Eubacteriaceae; Alkalibacter; Alkalibacter saccharofermentans | Alkalibacter saccharofermentans | Alkalibacter | Eubacteriaceae | Eubacteriales | Clostridia | Firmicutes | Bacteria |
| ASV3078 | 403 | 1 | 1 | 403 | 100 | NR_118554.1 Intestinimonas butyriciproducens strain SRB-521-5-I 16S ribosomal RNA, partial sequence | NR_118554.1 | 1475 | 346 | 750 | 27.46 | 520 | 3E-147 | 364/405 | 89 | 2/405 | 0 | Plus | Plus | Bacteria; Firmicutes; Clostridia; Eubacteriales; ; Intestinimonas; Intestinimonas butyriciproducens | Intestinimonas butyriciproducens | Intestinimonas | | Eubacteriales | Clostridia | Firmicutes | Bacteria |
| ASV3079 | 427 | 1 | 1 | 427 | 100 | NR_042961.1 Acholeplasma parvum strain H23M 16S ribosomal RNA, partial sequence | NR_042961.1 | 1426 | 315 | 740 | 29.87 | 466.5 | 5E-131 | 376/434 | 86 | 15/434 | 3 | Plus | Plus | Bacteria; Tenericutes; Mollicutes; Acholeplasmatales; Acholeplasmataceae; Acholeplasma; Acholeplasma parvum | Acholeplasma parvum | Acholeplasma | Acholeplasmataceae | Acholeplasmatales | Mollicutes | Tenericutes | Bacteria |
| ASV3080 | 402 | 2 | 1 | 402 | 100 | NR_156080.1 Kineothrix alysoides strain KNHs209 16S ribosomal RNA, partial sequence | NR_156080.1 | 1410 | 353 | 754 | 28.51 | 721.3 | 0 | 398/402 | 99 | 0/402 | 0 | Plus | Plus | Bacteria; Firmicutes; Clostridia; Eubacteriales; Lachnospiraceae; Kineothrix; Kineothrix alysoides | Kineothrix alysoides | Kineothrix | Lachnospiraceae | Eubacteriales | Clostridia | Firmicutes | Bacteria |
| ASV3081 | 402 | 4 | 1 | 402 | 100 | NR_114315.1 Blautia luti DSM 14534 16S ribosomal RNA, partial sequence | NR_114315.1 | 1481 | 353 | 754 | 27.14 | 521.9 | 9E-148 | 363/403 | 90 | 2/403 | 0 | Plus | Plus | Bacteria; Firmicutes; Clostridia; Eubacteriales; Lachnospiraceae; Blautia; Blautia luti | Blautia luti | Blautia | Lachnospiraceae | Eubacteriales | Clostridia | Firmicutes | Bacteria |
| ASV3082 | 405 | 3 | 1 | 405 | 100 | NR_147396.1 Lachnoclostridium pacaense strain Marseille-P3100 16S ribosomal RNA, partial sequence | NR_147396.1 | 1453 | 345 | 746 | 27.67 | 656.7 | 0 | 389/405 | 96 | 3/405 | 0 | Plus | Plus | Bacteria; Firmicutes; Clostridia; Eubacteriales; Lachnospiraceae; Lachnoclostridium; Lachnoclostridium pacaense | Lachnoclostridium pacaense | Lachnoclostridium | Lachnospiraceae | Eubacteriales | Clostridia | Firmicutes | Bacteria |
| ASV3083 | 405 | 1 | 1 | 405 | 100 | NR_118554.1 Intestinimonas butyriciproducens strain SRB-521-5-I 16S ribosomal RNA, partial sequence | NR_118554.1 | 1475 | 346 | 750 | 27.46 | 516.3 | 4E-146 | 364/406 | 89 | 2/406 | 0 | Plus | Plus | Bacteria; Firmicutes; Clostridia; Eubacteriales; ; Intestinimonas; Intestinimonas butyriciproducens | Intestinimonas butyriciproducens | Intestinimonas | | Eubacteriales | Clostridia | Firmicutes | Bacteria |
| ASV3084 | 427 | 2 | 1 | 427 | 100 | NR_037018.1 Acidaminococcus fermentans strain VR4 16S ribosomal RNA gene, partial sequence | NR_037018.1 | 1516 | 349 | 775 | 28.17 | 717.6 | 0 | 414/427 | 96 | 0/427 | 0 | Plus | Plus | Bacteria; Firmicutes; Negativicutes; Acidaminococcales; Acidaminococcaceae; Acidaminococcus; Acidaminococcus fermentans | Acidaminococcus fermentans | Acidaminococcus | Acidaminococcaceae | Acidaminococcales | Negativicutes | Firmicutes | Bacteria |
| ASV3086 | 422 | 2 | 1 | 422 | 100 | NR_113119.1 Prevotella oris strain JCM 8540 16S ribosomal RNA, partial sequence | NR_113119.1 | 1481 | 345 | 766 | 28.49 | 625.3 | 7E-179 | 396/424 | 93 | 4/424 | 0 | Plus | Plus | Bacteria; Bacteroidetes; Bacteroidia; Bacteroidales; Prevotellaceae; Prevotella; Prevotella oris | Prevotella oris | Prevotella | Prevotellaceae | Bacteroidales | Bacteroidia | Bacteroidetes | Bacteria |
| ASV3087 | 402 | 1 | 1 | 402 | 100 | NR_152688.1 Falcatimonas natans strain WN011 16S ribosomal RNA, partial sequence | NR_152688.1 | 1484 | 365 | 766 | 27.09 | 654.8 | 0 | 386/402 | 96 | 0/402 | 0 | Plus | Plus | Bacteria; Firmicutes; Clostridia; Eubacteriales; Clostridiaceae; Falcatimonas; Falcatimonas natans | Falcatimonas natans | Falcatimonas | Clostridiaceae | Eubacteriales | Clostridia | Firmicutes | Bacteria |
| ASV3088 | 405 | 1 | 1 | 405 | 100 | NR_144742.1 Christensenella massiliensis strain Marseille-P2438 16S ribosomal RNA, partial sequence | NR_144742.1 | 1499 | 369 | 773 | 27.02 | 560.7 | 2E-159 | 373/407 | 91 | 4/407 | 0 | Plus | Plus | Bacteria; Firmicutes; Clostridia; Eubacteriales; Christensenellaceae; Christensenella; Christensenella massiliensis | Christensenella massiliensis | Christensenella | Christensenellaceae | Eubacteriales | Clostridia | Firmicutes | Bacteria |
| ASV3089 | 427 | 1 | 1 | 427 | 100 | NR_042896.1 Mucispirillum schaedleri strain HRI I17 16S ribosomal RNA, partial sequence | NR_042896.1 | 1471 | 333 | 759 | 29.03 | 706.5 | 0 | 412/427 | 96 | 0/427 | 0 | Plus | Plus | Bacteria; Deferribacteres; Deferribacteres; Deferribacterales; Deferribacteraceae; Mucispirillum; Mucispirillum schaedleri | Mucispirillum schaedleri | Mucispirillum | Deferribacteraceae | Deferribacterales | Deferribacteres | Deferribacteres | Bacteria |
| ASV3090 | 426 | 1 | 1 | 426 | 100 | NR_028840.1 Mitsuokella jalaludinii strain M9 16S ribosomal RNA, partial sequence | NR_028840.1 | 1543 | 362 | 788 | 27.67 | 627.1 | 2E-179 | 399/428 | 93 | 3/428 | 0 | Plus | Plus | Bacteria; Firmicutes; Negativicutes; Selenomonadales; Selenomonadaceae; Mitsuokella; Mitsuokella jalaludinii | Mitsuokella jalaludinii | Mitsuokella | Selenomonadaceae | Selenomonadales | Negativicutes | Firmicutes | Bacteria |
| ASV3091 | 404 | 1 | 1 | 404 | 100 | NR_026476.1 Succinivibrio dextrinosolvens strain 0554 16S ribosomal RNA, partial sequence | NR_026476.1 | 1463 | 331 | 732 | 27.48 | 507.1 | 3E-143 | 361/404 | 89 | 2/404 | 0 | Plus | Plus | Bacteria; Proteobacteria; Gammaproteobacteria; Aeromonadales; Succinivibrionaceae; Succinivibrio; Succinivibrio dextrinosolvens | Succinivibrio dextrinosolvens | Succinivibrio | Succinivibrionaceae | Aeromonadales | Gammaproteobacteria | Proteobacteria | Bacteria |
| ASV3092 | 427 | 1 | 1 | 427 | 100 | NR_153710.1 Peptococcus simiae strain M108 16S ribosomal RNA, partial sequence | NR_153710.1 | 1488 | 354 | 780 | 28.7 | 606.8 | 3E-173 | 398/431 | 92 | 8/431 | 1 | Plus | Plus | Bacteria; Firmicutes; Clostridia; Eubacteriales; Peptococcaceae; Peptococcus; Peptococcus simiae | Peptococcus simiae | Peptococcus | Peptococcaceae | Eubacteriales | Clostridia | Firmicutes | Bacteria |
| ASV3093 | 402 | 3 | 1 | 402 | 100 | NR_151917.1 Helicobacter apri strain A19 16S ribosomal RNA, partial sequence | NR_151917.1 | 1099 | 36 | 437 | 36.58 | 699.2 | 0 | 394/402 | 98 | 0/402 | 0 | Plus | Plus | Bacteria; Proteobacteria; Epsilonproteobacteria; Campylobacterales; Helicobacteraceae; Helicobacter; Helicobacter apri | Helicobacter apri | Helicobacter | Helicobacteraceae | Campylobacterales | Epsilonproteobacteria | Proteobacteria | Bacteria |
| ASV3094 | 405 | 1 | 1 | 405 | 100 | NR_159227.1 Monoglobus pectinilyticus strain 14 16S ribosomal RNA, partial sequence | NR_159227.1 | 1493 | 368 | 769 | 26.93 | 534.8 | 1E-151 | 367/405 | 90 | 3/405 | 0 | Plus | Plus | Bacteria; Firmicutes; Clostridia; Eubacteriales; Oscillospiraceae; Monoglobus; Monoglobus pectinilyticus | Monoglobus pectinilyticus | Monoglobus | Oscillospiraceae | Eubacteriales | Clostridia | Firmicutes | Bacteria |
| ASV3095 | 405 | 1 | 1 | 405 | 100 | NR_118554.1 Intestinimonas butyriciproducens strain SRB-521-5-I 16S ribosomal RNA, partial sequence | NR_118554.1 | 1475 | 346 | 750 | 27.46 | 499.7 | 4E-141 | 361/406 | 88 | 2/406 | 0 | Plus | Plus | Bacteria; Firmicutes; Clostridia; Eubacteriales; ; Intestinimonas; Intestinimonas butyriciproducens | Intestinimonas butyriciproducens | Intestinimonas | | Eubacteriales | Clostridia | Firmicutes | Bacteria |
| ASV3096 | 427 | 2 | 3 | 427 | 99.53 | NR_133868.1 Streptococcus phocae subsp. salmonis strain C-4 16S ribosomal RNA, partial sequence | NR_133868.1 | 1410 | 293 | 717 | 30.14 | 547.7 | 2E-155 | 386/429 | 89 | 8/429 | 1 | Plus | Plus | Bacteria; Firmicutes; Bacilli; Lactobacillales; Streptococcaceae; Streptococcus; Streptococcus phocae | Streptococcus phocae | Streptococcus | Streptococcaceae | Lactobacillales | Bacilli | Firmicutes | Bacteria |
| ASV3098 | 422 | 1 | 1 | 422 | 100 | NR_041342.1 Parabacteroides distasonis strain JCM 5825 16S ribosomal RNA, partial sequence | NR_041342.1 | 1488 | 353 | 774 | 28.36 | 581 | 2E-165 | 386/422 | 91 | 0/422 | 0 | Plus | Plus | Bacteria; Bacteroidetes; Bacteroidia; Bacteroidales; Tannerellaceae; Parabacteroides; Parabacteroides distasonis | Parabacteroides distasonis | Parabacteroides | Tannerellaceae | Bacteroidales | Bacteroidia | Bacteroidetes | Bacteria |
| ASV3099 | 427 | 1 | 1 | 427 | 100 | NR_028840.1 Mitsuokella jalaludinii strain M9 16S ribosomal RNA, partial sequence | NR_028840.1 | 1543 | 362 | 788 | 27.67 | 706.5 | 0 | 413/428 | 96 | 2/428 | 0 | Plus | Plus | Bacteria; Firmicutes; Negativicutes; Selenomonadales; Selenomonadaceae; Mitsuokella; Mitsuokella jalaludinii | Mitsuokella jalaludinii | Mitsuokella | Selenomonadaceae | Selenomonadales | Negativicutes | Firmicutes | Bacteria |
| ASV3100 | 427 | 1 | 1 | 427 | 100 | NR_044111.1 Selenomonas bovis strain WG 16S ribosomal RNA, partial sequence | NR_044111.1 | 1525 | 370 | 796 | 28 | 767.5 | 0 | 423/427 | 99 | 0/427 | 0 | Plus | Plus | Bacteria; Firmicutes; Negativicutes; Selenomonadales; Selenomonadaceae; Selenomonas; Selenomonas bovis | Selenomonas bovis | Selenomonas | Selenomonadaceae | Selenomonadales | Negativicutes | Firmicutes | Bacteria |
| ASV3101 | 422 | 2 | 1 | 422 | 100 | NR_113119.1 Prevotella oris strain JCM 8540 16S ribosomal RNA, partial sequence | NR_113119.1 | 1481 | 345 | 766 | 28.49 | 608.7 | 7E-174 | 392/423 | 92 | 2/423 | 0 | Plus | Plus | Bacteria; Bacteroidetes; Bacteroidia; Bacteroidales; Prevotellaceae; Prevotella; Prevotella oris | Prevotella oris | Prevotella | Prevotellaceae | Bacteroidales | Bacteroidia | Bacteroidetes | Bacteria |
| ASV3102 | 409 | 2 | 1 | 409 | 100 | NR_144696.1 Clostridium saudiense strain JCC 16S ribosomal RNA, partial sequence | NR_144696.1 | 1478 | 350 | 751 | 27.2 | 560.7 | 2E-159 | 375/409 | 91 | 7/409 | 1 | Plus | Plus | Bacteria; Firmicutes; Clostridia; Eubacteriales; Clostridiaceae; Clostridium; Clostridium saudiense | Clostridium saudiense | Clostridium | Clostridiaceae | Eubacteriales | Clostridia | Firmicutes | Bacteria |
| ASV3103 | 427 | 2 | 1 | 426 | 99.77 | NR_037018.1 Acidaminococcus fermentans strain VR4 16S ribosomal RNA gene, partial sequence | NR_037018.1 | 1516 | 349 | 774 | 28.1 | 699.2 | 0 | 410/426 | 96 | 0/426 | 0 | Plus | Plus | Bacteria; Firmicutes; Negativicutes; Acidaminococcales; Acidaminococcaceae; Acidaminococcus; Acidaminococcus fermentans | Acidaminococcus fermentans | Acidaminococcus | Acidaminococcaceae | Acidaminococcales | Negativicutes | Firmicutes | Bacteria |
| ASV3104 | 427 | 2 | 1 | 426 | 99.77 | NR_037018.1 Acidaminococcus fermentans strain VR4 16S ribosomal RNA gene, partial sequence | NR_037018.1 | 1516 | 349 | 774 | 28.1 | 704.7 | 0 | 411/426 | 96 | 0/426 | 0 | Plus | Plus | Bacteria; Firmicutes; Negativicutes; Acidaminococcales; Acidaminococcaceae; Acidaminococcus; Acidaminococcus fermentans | Acidaminococcus fermentans | Acidaminococcus | Acidaminococcaceae | Acidaminococcales | Negativicutes | Firmicutes | Bacteria |
| ASV3105 | 421 | 1 | 1 | 421 | 100 | NR_041342.1 Parabacteroides distasonis strain JCM 5825 16S ribosomal RNA, partial sequence | NR_041342.1 | 1488 | 353 | 774 | 28.36 | 451.7 | 1E-126 | 365/424 | 86 | 5/424 | 1 | Plus | Plus | Bacteria; Bacteroidetes; Bacteroidia; Bacteroidales; Tannerellaceae; Parabacteroides; Parabacteroides distasonis | Parabacteroides distasonis | Parabacteroides | Tannerellaceae | Bacteroidales | Bacteroidia | Bacteroidetes | Bacteria |
| ASV3106 | 402 | 1 | 1 | 402 | 100 | NR_118670.1 Eubacterium ventriosum strain ATCC 27560 16S ribosomal RNA, partial sequence | NR_118670.1 | 1481 | 374 | 775 | 27.14 | 712.1 | 0 | 395/402 | 98 | 0/402 | 0 | Plus | Plus | Bacteria; Firmicutes; Clostridia; Eubacteriales; Eubacteriaceae; Eubacterium; Eubacterium ventriosum | Eubacterium ventriosum | Eubacterium | Eubacteriaceae | Eubacteriales | Clostridia | Firmicutes | Bacteria |
| ASV3107 | 428 | 1 | 3 | 428 | 99.53 | NR_126243.1 Lactobacillus delbrueckii subsp. jakobsenii strain ZN7a-9 16S ribosomal RNA, partial sequence | NR_126243.1 | 1382 | 271 | 696 | 30.82 | 577.3 | 2E-164 | 391/429 | 91 | 6/429 | 1 | Plus | Plus | Bacteria; Firmicutes; Bacilli; Lactobacillales; Lactobacillaceae; Lactobacillus; Lactobacillus delbrueckii | Lactobacillus delbrueckii | Lactobacillus | Lactobacillaceae | Lactobacillales | Bacilli | Firmicutes | Bacteria |
| ASV3109 | 405 | 2 | 1 | 405 | 100 | NR_147375.1 Colidextribacter massiliensis strain Marseille-P3083 16S ribosomal RNA, partial sequence | NR_147375.1 | 1487 | 364 | 765 | 27.03 | 529.3 | 6E-150 | 366/405 | 90 | 3/405 | 0 | Plus | Plus | Bacteria; Firmicutes; Clostridia; Eubacteriales; ; Colidextribacter; Colidextribacter massiliensis | Colidextribacter massiliensis | Colidextribacter | | Eubacteriales | Clostridia | Firmicutes | Bacteria |
| ASV3110 | 418 | 1 | 1 | 418 | 100 | NR_116008.1 Pandoraea thiooxydans strain ATSB16 16S ribosomal RNA, partial sequence | NR_116008.1 | 1410 | 307 | 733 | 30.28 | 431.4 | 2E-120 | 365/428 | 85 | 11/428 | 2 | Plus | Plus | Bacteria; Proteobacteria; Betaproteobacteria; Burkholderiales; Burkholderiaceae; Pandoraea; Pandoraea thiooxydans | Pandoraea thiooxydans | Pandoraea | Burkholderiaceae | Burkholderiales | Betaproteobacteria | Proteobacteria | Bacteria |
| ASV3111 | 405 | 1 | 4 | 405 | 99.26 | NR_026476.1 Succinivibrio dextrinosolvens strain 0554 16S ribosomal RNA, partial sequence | NR_026476.1 | 1463 | 334 | 732 | 27.27 | 551.4 | 1E-156 | 369/403 | 91 | 5/403 | 1 | Plus | Plus | Bacteria; Proteobacteria; Gammaproteobacteria; Aeromonadales; Succinivibrionaceae; Succinivibrio; Succinivibrio dextrinosolvens | Succinivibrio dextrinosolvens | Succinivibrio | Succinivibrionaceae | Aeromonadales | Gammaproteobacteria | Proteobacteria | Bacteria |
| ASV3112 | 405 | 4 | 3 | 405 | 99.51 | NR_118511.1 Campylobacter coli strain ATCC 33559 16S ribosomal RNA, partial sequence | NR_118511.1 | 1435 | 331 | 731 | 27.94 | 571.7 | 9E-163 | 372/403 | 92 | 2/403 | 0 | Plus | Plus | Bacteria; Proteobacteria; Epsilonproteobacteria; Campylobacterales; Campylobacteraceae; Campylobacter; Campylobacter coli | Campylobacter coli | Campylobacter | Campylobacteraceae | Campylobacterales | Epsilonproteobacteria | Proteobacteria | Bacteria |
| ASV3114 | 402 | 1 | 1 | 402 | 100 | NR_102884.1 Ruminococcus champanellensis 18P13 = JCM 17042 16S ribosomal RNA, partial sequence | NR_102884.1 | 1494 | 348 | 749 | 26.91 | 643.8 | 0 | 385/403 | 95 | 2/403 | 0 | Plus | Plus | Bacteria; Firmicutes; Clostridia; Eubacteriales; Oscillospiraceae; Ruminococcus; Ruminococcus champanellensis | Ruminococcus champanellensis | Ruminococcus | Oscillospiraceae | Eubacteriales | Clostridia | Firmicutes | Bacteria |
| ASV3115 | 427 | 1 | 1 | 427 | 100 | NR_041780.1 Streptococcus hyointestinalis strain ATCC 49169 16S ribosomal RNA, partial sequence | NR_041780.1 | 1439 | 348 | 774 | 29.67 | 778.6 | 0 | 425/427 | 99 | 0/427 | 0 | Plus | Plus | Bacteria; Firmicutes; Bacilli; Lactobacillales; Streptococcaceae; Streptococcus; Streptococcus hyointestinalis | Streptococcus hyointestinalis | Streptococcus | Streptococcaceae | Lactobacillales | Bacilli | Firmicutes | Bacteria |
| ASV3116 | 405 | 2 | 3 | 405 | 99.51 | NR_148837.1 Abyssivirga alkaniphila strain L81 16S ribosomal RNA, partial sequence | NR_148837.1 | 1520 | 366 | 765 | 26.32 | 470.2 | 3E-132 | 354/403 | 87 | 3/403 | 0 | Plus | Plus | Bacteria; Firmicutes; Clostridia; Eubacteriales; Lachnospiraceae; Abyssivirga; Abyssivirga alkaniphila | Abyssivirga alkaniphila | Abyssivirga | Lachnospiraceae | Eubacteriales | Clostridia | Firmicutes | Bacteria |
| ASV3119 | 427 | 2 | 1 | 427 | 100 | NR_112903.1 Parasutterella secunda strain YIT 12071 16S ribosomal RNA, partial sequence | NR_112903.1 | 1485 | 329 | 755 | 28.75 | 789.6 | 0 | 427/427 | 100 | 0/427 | 0 | Plus | Plus | Bacteria; Proteobacteria; Betaproteobacteria; Burkholderiales; Sutterellaceae; Parasutterella; Parasutterella secunda | Parasutterella secunda | Parasutterella | Sutterellaceae | Burkholderiales | Betaproteobacteria | Proteobacteria | Bacteria |
| ASV3120 | 427 | 1 | 1 | 427 | 100 | NR_042896.1 Mucispirillum schaedleri strain HRI I17 16S ribosomal RNA, partial sequence | NR_042896.1 | 1471 | 333 | 759 | 29.03 | 717.6 | 0 | 414/427 | 96 | 0/427 | 0 | Plus | Plus | Bacteria; Deferribacteres; Deferribacteres; Deferribacterales; Deferribacteraceae; Mucispirillum; Mucispirillum schaedleri | Mucispirillum schaedleri | Mucispirillum | Deferribacteraceae | Deferribacterales | Deferribacteres | Deferribacteres | Bacteria |
| ASV3121 | 402 | 1 | 1 | 402 | 100 | NR_024661.1 Eubacterium ruminantium strain GA195 16S ribosomal RNA, partial sequence | NR_024661.1 | 1508 | 378 | 779 | 26.66 | 654.8 | 0 | 387/403 | 96 | 2/403 | 0 | Plus | Plus | Bacteria; Firmicutes; Clostridia; Eubacteriales; Eubacteriaceae; Eubacterium; Eubacterium ruminantium | Eubacterium ruminantium | Eubacterium | Eubacteriaceae | Eubacteriales | Clostridia | Firmicutes | Bacteria |
| ASV3122 | 405 | 5 | 1 | 405 | 100 | NR_156081.1 Kineothrix alysoides strain KNHs209 16S ribosomal RNA, partial sequence | NR_156081.1 | 1411 | 353 | 754 | 28.49 | 689.9 | 0 | 396/406 | 97 | 5/406 | 1 | Plus | Plus | Bacteria; Firmicutes; Clostridia; Eubacteriales; Lachnospiraceae; Kineothrix; Kineothrix alysoides | Kineothrix alysoides | Kineothrix | Lachnospiraceae | Eubacteriales | Clostridia | Firmicutes | Bacteria |
| ASV3123 | 427 | 2 | 1 | 427 | 100 | NR_157643.1 Treponema rectale strain CHPA 16S ribosomal RNA, partial sequence | NR_157643.1 | 1309 | 259 | 685 | 32.62 | 579.1 | 6E-165 | 392/430 | 91 | 6/430 | 1 | Plus | Plus | Bacteria; Spirochaetes; Spirochaetia; Spirochaetales; Spirochaetaceae; Treponema; Treponema rectale | Treponema rectale | Treponema | Spirochaetaceae | Spirochaetales | Spirochaetia | Spirochaetes | Bacteria |
| ASV3124 | 422 | 1 | 1 | 422 | 100 | NR_113411.1 Prevotella copri DSM 18205 strain JCM 13464 16S ribosomal RNA, partial sequence | NR_113411.1 | 1493 | 358 | 779 | 28.27 | 619.7 | 3E-177 | 394/423 | 93 | 2/423 | 0 | Plus | Plus | Bacteria; Bacteroidetes; Bacteroidia; Bacteroidales; Prevotellaceae; Prevotella; Prevotella copri | Prevotella copri | Prevotella | Prevotellaceae | Bacteroidales | Bacteroidia | Bacteroidetes | Bacteria |
| ASV3125 | 402 | 1 | 1 | 402 | 100 | NR_044054.1 Blautia wexlerae DSM 19850 16S ribosomal RNA, partial sequence | NR_044054.1 | 1438 | 314 | 715 | 27.96 | 721.3 | 0 | 398/402 | 99 | 0/402 | 0 | Plus | Plus | Bacteria; Firmicutes; Clostridia; Eubacteriales; Lachnospiraceae; Blautia; Blautia wexlerae | Blautia wexlerae | Blautia | Lachnospiraceae | Eubacteriales | Clostridia | Firmicutes | Bacteria |
| ASV3126 | 403 | 1 | 1 | 403 | 100 | NR_117912.1 Defluviitalea saccharophila strain LIND6LT2 16S ribosomal RNA, partial sequence | NR_117912.1 | 1495 | 342 | 746 | 27.09 | 564.3 | 2E-160 | 373/406 | 91 | 4/406 | 0 | Plus | Plus | Bacteria; Firmicutes; Clostridia; Eubacteriales; Defluviitaleaceae; Defluviitalea; Defluviitalea saccharophila | Defluviitalea saccharophila | Defluviitalea | Defluviitaleaceae | Eubacteriales | Clostridia | Firmicutes | Bacteria |
| ASV3128 | 427 | 1 | 1 | 427 | 100 | NR_112902.1 Phascolarctobacterium succinatutens strain YIT 12067 16S ribosomal RNA, partial sequence | NR_112902.1 | 1610 | 444 | 870 | 26.52 | 773 | 0 | 424/427 | 99 | 0/427 | 0 | Plus | Plus | Bacteria; Firmicutes; Negativicutes; Acidaminococcales; Acidaminococcaceae; Phascolarctobacterium; Phascolarctobacterium succinatutens | Phascolarctobacterium succinatutens | Phascolarctobacterium | Acidaminococcaceae | Acidaminococcales | Negativicutes | Firmicutes | Bacteria |
| ASV3129 | 422 | 1 | 1 | 422 | 100 | NR_118334.1 Alloprevotella rava strain 81/4-12 16S ribosomal RNA, partial sequence | NR_118334.1 | 1455 | 338 | 759 | 29 | 531.1 | 2E-150 | 383/428 | 89 | 12/428 | 2 | Plus | Plus | Bacteria; Bacteroidetes; Bacteroidia; Bacteroidales; Prevotellaceae; Alloprevotella; Alloprevotella rava | Alloprevotella rava | Alloprevotella | Prevotellaceae | Bacteroidales | Bacteroidia | Bacteroidetes | Bacteria |
| ASV3130 | 403 | 4 | 4 | 403 | 99.26 | NR_118511.1 Campylobacter coli strain ATCC 33559 16S ribosomal RNA, partial sequence | NR_118511.1 | 1435 | 332 | 731 | 27.87 | 623.4 | 3E-178 | 379/400 | 94 | 0/400 | 0 | Plus | Plus | Bacteria; Proteobacteria; Epsilonproteobacteria; Campylobacterales; Campylobacteraceae; Campylobacter; Campylobacter coli | Campylobacter coli | Campylobacter | Campylobacteraceae | Campylobacterales | Epsilonproteobacteria | Proteobacteria | Bacteria |
| ASV3134 | 422 | 2 | 1 | 422 | 100 | NR_113119.1 Prevotella oris strain JCM 8540 16S ribosomal RNA, partial sequence | NR_113119.1 | 1481 | 345 | 766 | 28.49 | 575.4 | 7E-164 | 385/422 | 91 | 0/422 | 0 | Plus | Plus | Bacteria; Bacteroidetes; Bacteroidia; Bacteroidales; Prevotellaceae; Prevotella; Prevotella oris | Prevotella oris | Prevotella | Prevotellaceae | Bacteroidales | Bacteroidia | Bacteroidetes | Bacteria |
| ASV3135 | 405 | 1 | 1 | 405 | 100 | NR_144611.1 Flintibacter butyricus strain BLS21 16S ribosomal RNA, partial sequence | NR_144611.1 | 808 | 299 | 703 | 50.12 | 494.2 | 2E-139 | 360/406 | 88 | 2/406 | 0 | Plus | Plus | Bacteria; Firmicutes; Clostridia; Eubacteriales; ; Flintibacter; Flintibacter butyricus | Flintibacter butyricus | Flintibacter | | Eubacteriales | Clostridia | Firmicutes | Bacteria |
| ASV3136 | 402 | 1 | 1 | 402 | 100 | NR_044289.1 [Clostridium] lavalense strain CCRI-9842 16S ribosomal RNA, partial sequence | NR_044289.1 | 1454 | 345 | 746 | 27.65 | 688.1 | 0 | 392/402 | 97 | 0/402 | 0 | Plus | Plus | Bacteria; Firmicutes; Clostridia; Eubacteriales; Lachnospiraceae; Enterocloster; Enterocloster lavalensis | Enterocloster lavalensis | Enterocloster | Lachnospiraceae | Eubacteriales | Clostridia | Firmicutes | Bacteria |
| ASV3137 | 422 | 1 | 1 | 422 | 100 | NR_041364.1 Prevotella stercorea DSM 18206 strain CB35 16S ribosomal RNA, partial sequence | NR_041364.1 | 1488 | 354 | 775 | 28.36 | 497.9 | 2E-140 | 372/423 | 87 | 2/423 | 0 | Plus | Plus | Bacteria; Bacteroidetes; Bacteroidia; Bacteroidales; Prevotellaceae; Prevotella; Prevotella stercorea | Prevotella stercorea | Prevotella | Prevotellaceae | Bacteroidales | Bacteroidia | Bacteroidetes | Bacteria |
| ASV3138 | 427 | 1 | 1 | 425 | 99.53 | NR_102980.1 Megasphaera elsdenii DSM 20460 16S ribosomal RNA, partial sequence | NR_102980.1 | 1552 | 369 | 793 | 27.38 | 697.3 | 0 | 409/425 | 96 | 0/425 | 0 | Plus | Plus | Bacteria; Firmicutes; Negativicutes; Veillonellales; Veillonellaceae; Megasphaera; Megasphaera elsdenii | Megasphaera elsdenii | Megasphaera | Veillonellaceae | Veillonellales | Negativicutes | Firmicutes | Bacteria |
| ASV3139 | 427 | 1 | 1 | 427 | 100 | NR_044111.1 Selenomonas bovis strain WG 16S ribosomal RNA, partial sequence | NR_044111.1 | 1525 | 370 | 796 | 28 | 689.9 | 0 | 409/427 | 95 | 0/427 | 0 | Plus | Plus | Bacteria; Firmicutes; Negativicutes; Selenomonadales; Selenomonadaceae; Selenomonas; Selenomonas bovis | Selenomonas bovis | Selenomonas | Selenomonadaceae | Selenomonadales | Negativicutes | Firmicutes | Bacteria |
| ASV3140 | 403 | 2 | 1 | 403 | 100 | NR_156912.1 Defluviitalea raffinosedens strain A6 16S ribosomal RNA, partial sequence | NR_156912.1 | 1413 | 324 | 728 | 28.66 | 564.3 | 2E-160 | 374/407 | 91 | 6/407 | 1 | Plus | Plus | Bacteria; Firmicutes; Clostridia; Eubacteriales; Defluviitaleaceae; Defluviitalea; Defluviitalea raffinosedens | Defluviitalea raffinosedens | Defluviitalea | Defluviitaleaceae | Eubacteriales | Clostridia | Firmicutes | Bacteria |
| ASV3141 | 404 | 2 | 1 | 404 | 100 | NR_074793.2 Oscillibacter valericigenes strain Sjm18-20 16S ribosomal RNA, complete sequence | NR_074793.2 | 1530 | 372 | 775 | 26.41 | 641.9 | 0 | 385/404 | 95 | 0/404 | 0 | Plus | Plus | Bacteria; Firmicutes; Clostridia; Eubacteriales; Oscillospiraceae; Oscillibacter; Oscillibacter valericigenes | Oscillibacter valericigenes | Oscillibacter | Oscillospiraceae | Eubacteriales | Clostridia | Firmicutes | Bacteria |
| ASV3144 | 422 | 1 | 1 | 422 | 100 | NR_144750.1 Prevotellamassilia timonensis strain Marseille-P2831 16S ribosomal RNA, partial sequence | NR_144750.1 | 1493 | 358 | 779 | 28.27 | 536.6 | 4E-152 | 378/422 | 89 | 0/422 | 0 | Plus | Plus | Bacteria; Bacteroidetes; Bacteroidia; Bacteroidales; Prevotellaceae; Prevotellamassilia; Prevotellamassilia timonensis | Prevotellamassilia timonensis | Prevotellamassilia | Prevotellaceae | Bacteroidales | Bacteroidia | Bacteroidetes | Bacteria |
| ASV3145 | 402 | 1 | 1 | 402 | 100 | NR_137405.1 Anaerobium acetethylicum strain GluBS11 16S ribosomal RNA, partial sequence | NR_137405.1 | 1402 | 324 | 725 | 28.67 | 654.8 | 0 | 386/402 | 96 | 0/402 | 0 | Plus | Plus | Bacteria; Firmicutes; Clostridia; Eubacteriales; Lachnospiraceae; Anaerobium; Anaerobium acetethylicum | Anaerobium acetethylicum | Anaerobium | Lachnospiraceae | Eubacteriales | Clostridia | Firmicutes | Bacteria |
| ASV3146 | 422 | 1 | 1 | 422 | 100 | NR_113411.1 Prevotella copri DSM 18205 strain JCM 13464 16S ribosomal RNA, partial sequence | NR_113411.1 | 1493 | 358 | 779 | 28.27 | 697.3 | 0 | 407/422 | 96 | 0/422 | 0 | Plus | Plus | Bacteria; Bacteroidetes; Bacteroidia; Bacteroidales; Prevotellaceae; Prevotella; Prevotella copri | Prevotella copri | Prevotella | Prevotellaceae | Bacteroidales | Bacteroidia | Bacteroidetes | Bacteria |
| ASV3147 | 401 | 1 | 1 | 401 | 100 | NR_117374.1 Parvibacter caecicola strain NR06 16S ribosomal RNA, partial sequence | NR_117374.1 | 1442 | 336 | 736 | 27.81 | 592 | 7E-169 | 374/401 | 93 | 0/401 | 0 | Plus | Plus | Bacteria; Actinobacteria; Coriobacteriia; Coriobacteriales; Coriobacteriaceae; Parvibacter; Parvibacter caecicola | Parvibacter caecicola | Parvibacter | Coriobacteriaceae | Coriobacteriales | Coriobacteriia | Actinobacteria | Bacteria |
| ASV3149 | 403 | 1 | 1 | 403 | 100 | NR_137408.1 Terrisporobacter petrolearius strain LAM0A37 16S ribosomal RNA, partial sequence | NR_137408.1 | 1405 | 307 | 707 | 28.54 | 638.2 | 0 | 386/405 | 95 | 6/405 | 1 | Plus | Plus | Bacteria; Firmicutes; Clostridia; Eubacteriales; Peptostreptococcaceae; Terrisporobacter; Terrisporobacter petrolearius | Terrisporobacter petrolearius | Terrisporobacter | Peptostreptococcaceae | Eubacteriales | Clostridia | Firmicutes | Bacteria |
| ASV3150 | 404 | 1 | 1 | 404 | 100 | NR_025670.1 Pseudoflavonifractor capillosus ATCC 29799 16S ribosomal RNA, partial sequence | NR_025670.1 | 1483 | 346 | 750 | 27.31 | 503.4 | 3E-142 | 361/405 | 89 | 1/405 | 0 | Plus | Plus | Bacteria; Firmicutes; Clostridia; Eubacteriales; Oscillospiraceae; Pseudoflavonifractor; Pseudoflavonifractor capillosus | Pseudoflavonifractor capillosus | Pseudoflavonifractor | Oscillospiraceae | Eubacteriales | Clostridia | Firmicutes | Bacteria |
| ASV3151 | 403 | 2 | 1 | 394 | 97.77 | NR_027557.1 Roseburia intestinalis L1-82 16S ribosomal RNA, partial sequence | NR_027557.1 | 1482 | 360 | 754 | 26.65 | 446.2 | 6E-125 | 344/395 | 87 | 1/395 | 0 | Plus | Plus | Bacteria; Firmicutes; Clostridia; Eubacteriales; Lachnospiraceae; Roseburia; Roseburia intestinalis | Roseburia intestinalis | Roseburia | Lachnospiraceae | Eubacteriales | Clostridia | Firmicutes | Bacteria |
| ASV3152 | 421 | 3 | 1 | 421 | 100 | NR_148821.1 Bacteroides caecicola strain C13EG70 16S ribosomal RNA, partial sequence | NR_148821.1 | 1492 | 356 | 777 | 28.28 | 435.1 | 1E-121 | 364/426 | 85 | 9/426 | 2 | Plus | Plus | Bacteria; Bacteroidetes; Bacteroidia; Bacteroidales; Bacteroidaceae; Bacteroides; Bacteroides caecicola | Bacteroides caecicola | Bacteroides | Bacteroidaceae | Bacteroidales | Bacteroidia | Bacteroidetes | Bacteria |
| ASV3153 | 405 | 1 | 1 | 405 | 100 | NR_147370.1 Pseudoflavonifractor phocaeensis strain Marseille-P3064 16S ribosomal RNA, partial sequence | NR_147370.1 | 1495 | 366 | 770 | 27.09 | 737.9 | 0 | 403/405 | 99 | 0/405 | 0 | Plus | Plus | Bacteria; Firmicutes; Clostridia; Eubacteriales; Oscillospiraceae; Pseudoflavonifractor; Pseudoflavonifractor phocaeensis | Pseudoflavonifractor phocaeensis | Pseudoflavonifractor | Oscillospiraceae | Eubacteriales | Clostridia | Firmicutes | Bacteria |
| ASV3154 | 403 | 1 | 3 | 403 | 99.5 | NR_125677.1 Vallitalea pronyensis strain FatNI3 16S ribosomal RNA, partial sequence | NR_125677.1 | 1480 | 365 | 764 | 27.03 | 440.6 | 3E-123 | 348/402 | 86 | 3/402 | 0 | Plus | Plus | Bacteria; Firmicutes; Clostridia; Eubacteriales; Vallitaleaceae; Vallitalea; Vallitalea pronyensis | Vallitalea pronyensis | Vallitalea | Vallitaleaceae | Eubacteriales | Clostridia | Firmicutes | Bacteria |
| ASV3155 | 401 | 1 | 1 | 401 | 100 | NR_074634.1 [Eubacterium] rectale ATCC 33656 16S ribosomal RNA, partial sequence | NR_074634.1 | 1521 | 367 | 768 | 26.43 | 420.3 | 4E-117 | 346/404 | 85 | 5/404 | 1 | Plus | Plus | Bacteria; Firmicutes; Clostridia; Eubacteriales; Lachnospiraceae; ; [Eubacterium] rectale | [Eubacterium] rectale | | Lachnospiraceae | Eubacteriales | Clostridia | Firmicutes | Bacteria |
| ASV3156 | 405 | 2 | 1 | 405 | 100 | NR_074629.1 Hungateiclostridium thermocellum strain ATCC 27405 16S ribosomal RNA, partial sequence | NR_074629.1 | 1521 | 358 | 762 | 26.63 | 466.5 | 4E-131 | 356/407 | 87 | 4/407 | 0 | Plus | Plus | Bacteria; Firmicutes; Clostridia; Eubacteriales; Oscillospiraceae; Acetivibrio; Acetivibrio thermocellus | Acetivibrio thermocellus | Acetivibrio | Oscillospiraceae | Eubacteriales | Clostridia | Firmicutes | Bacteria |
| ASV3157 | 402 | 1 | 1 | 402 | 100 | NR_118667.1 Lachnospira pectinoschiza strain 150-1 16S ribosomal RNA, partial sequence | NR_118667.1 | 1465 | 372 | 773 | 27.44 | 717.6 | 0 | 395/402 | 98 | 0/402 | 0 | Plus | Plus | Bacteria; Firmicutes; Clostridia; Eubacteriales; Lachnospiraceae; Lachnospira; Lachnospira pectinoschiza | Lachnospira pectinoschiza | Lachnospira | Lachnospiraceae | Eubacteriales | Clostridia | Firmicutes | Bacteria |
| ASV3158 | 427 | 1 | 1 | 427 | 100 | NR_042896.1 Mucispirillum schaedleri strain HRI I17 16S ribosomal RNA, partial sequence | NR_042896.1 | 1471 | 333 | 759 | 29.03 | 717.6 | 0 | 414/427 | 96 | 0/427 | 0 | Plus | Plus | Bacteria; Deferribacteres; Deferribacteres; Deferribacterales; Deferribacteraceae; Mucispirillum; Mucispirillum schaedleri | Mucispirillum schaedleri | Mucispirillum | Deferribacteraceae | Deferribacterales | Deferribacteres | Deferribacteres | Bacteria |
| ASV3159 | 427 | 5 | 4 | 427 | 99.3 | NR_146813.2 Erysipelothrix larvae strain LV19 16S ribosomal RNA, complete sequence | NR_146813.2 | 1543 | 370 | 792 | 27.41 | 427.7 | 2E-119 | 363/427 | 85 | 7/427 | 1 | Plus | Plus | Bacteria; Firmicutes; Erysipelotrichia; Erysipelotrichales; Erysipelotrichaceae; Erysipelothrix; Erysipelothrix larvae | Erysipelothrix larvae | Erysipelothrix | Erysipelotrichaceae | Erysipelotrichales | Erysipelotrichia | Firmicutes | Bacteria |
| ASV3160 | 422 | 2 | 1 | 422 | 100 | NR_113119.1 Prevotella oris strain JCM 8540 16S ribosomal RNA, partial sequence | NR_113119.1 | 1481 | 345 | 766 | 28.49 | 569.9 | 3E-162 | 386/424 | 91 | 4/424 | 0 | Plus | Plus | Bacteria; Bacteroidetes; Bacteroidia; Bacteroidales; Prevotellaceae; Prevotella; Prevotella oris | Prevotella oris | Prevotella | Prevotellaceae | Bacteroidales | Bacteroidia | Bacteroidetes | Bacteria |
| ASV3161 | 403 | 1 | 3 | 403 | 99.5 | NR_125677.1 Vallitalea pronyensis strain FatNI3 16S ribosomal RNA, partial sequence | NR_125677.1 | 1480 | 365 | 764 | 27.03 | 462.8 | 6E-130 | 354/404 | 87 | 7/404 | 1 | Plus | Plus | Bacteria; Firmicutes; Clostridia; Eubacteriales; Vallitaleaceae; Vallitalea; Vallitalea pronyensis | Vallitalea pronyensis | Vallitalea | Vallitaleaceae | Eubacteriales | Clostridia | Firmicutes | Bacteria |
| ASV3162 | 404 | 1 | 1 | 404 | 100 | NR_112097.1 Pseudoramibacter alactolyticus ATCC 23263 16S ribosomal RNA, partial sequence | NR_112097.1 | 1480 | 376 | 779 | 27.3 | 675.1 | 0 | 392/405 | 96 | 2/405 | 0 | Plus | Plus | Bacteria; Firmicutes; Clostridia; Eubacteriales; Eubacteriaceae; Pseudoramibacter; Pseudoramibacter alactolyticus | Pseudoramibacter alactolyticus | Pseudoramibacter | Eubacteriaceae | Eubacteriales | Clostridia | Firmicutes | Bacteria |
| ASV3163 | 427 | 2 | 1 | 426 | 99.77 | NR_037018.1 Acidaminococcus fermentans strain VR4 16S ribosomal RNA gene, partial sequence | NR_037018.1 | 1516 | 349 | 774 | 28.1 | 721.3 | 0 | 414/426 | 97 | 0/426 | 0 | Plus | Plus | Bacteria; Firmicutes; Negativicutes; Acidaminococcales; Acidaminococcaceae; Acidaminococcus; Acidaminococcus fermentans | Acidaminococcus fermentans | Acidaminococcus | Acidaminococcaceae | Acidaminococcales | Negativicutes | Firmicutes | Bacteria |
| ASV3164 | 427 | 2 | 1 | 427 | 100 | NR_043231.1 Dialister propionicifaciens strain ADV 1053.03 16S ribosomal RNA, partial sequence | NR_043231.1 | 1396 | 300 | 726 | 30.59 | 617.9 | 1E-176 | 397/428 | 92 | 2/428 | 0 | Plus | Plus | Bacteria; Firmicutes; Negativicutes; Veillonellales; Veillonellaceae; Dialister; Dialister propionicifaciens | Dialister propionicifaciens | Dialister | Veillonellaceae | Veillonellales | Negativicutes | Firmicutes | Bacteria |
| ASV3167 | 403 | 1 | 3 | 403 | 99.5 | NR_125464.1 Anaerobacterium chartisolvens strain T-1-35 16S ribosomal RNA, partial sequence | NR_125464.1 | 1451 | 344 | 745 | 27.71 | 486.8 | 3E-137 | 356/402 | 88 | 1/402 | 0 | Plus | Plus | Bacteria; Firmicutes; Clostridia; Eubacteriales; Oscillospiraceae; Anaerobacterium; Anaerobacterium chartisolvens | Anaerobacterium chartisolvens | Anaerobacterium | Oscillospiraceae | Eubacteriales | Clostridia | Firmicutes | Bacteria |
| ASV3168 | 428 | 2 | 1 | 428 | 100 | NR_026030.1 Actinobacillus porcinus strain NM319 16S ribosomal RNA, partial sequence | NR_026030.1 | 1466 | 335 | 761 | 29.13 | 756.4 | 0 | 422/428 | 98 | 1/428 | 0 | Plus | Plus | Bacteria; Proteobacteria; Gammaproteobacteria; Pasteurellales; Pasteurellaceae; Actinobacillus; Actinobacillus porcinus | Actinobacillus porcinus | Actinobacillus | Pasteurellaceae | Pasteurellales | Gammaproteobacteria | Proteobacteria | Bacteria |
| ASV3170 | 422 | 1 | 1 | 422 | 100 | NR_113411.1 Prevotella copri DSM 18205 strain JCM 13464 16S ribosomal RNA, partial sequence | NR_113411.1 | 1493 | 358 | 779 | 28.27 | 697.3 | 0 | 407/422 | 96 | 0/422 | 0 | Plus | Plus | Bacteria; Bacteroidetes; Bacteroidia; Bacteroidales; Prevotellaceae; Prevotella; Prevotella copri | Prevotella copri | Prevotella | Prevotellaceae | Bacteroidales | Bacteroidia | Bacteroidetes | Bacteria |
| ASV3172 | 422 | 2 | 1 | 422 | 100 | NR_074376.1 Parabacteroides distasonis ATCC 8503 16S ribosomal RNA, partial sequence | NR_074376.1 | 1497 | 368 | 789 | 28.19 | 553.3 | 4E-157 | 381/422 | 90 | 0/422 | 0 | Plus | Plus | Bacteria; Bacteroidetes; Bacteroidia; Bacteroidales; Tannerellaceae; Parabacteroides; Parabacteroides distasonis | Parabacteroides distasonis | Parabacteroides | Tannerellaceae | Bacteroidales | Bacteroidia | Bacteroidetes | Bacteria |
| ASV3173 | 403 | 1 | 1 | 403 | 100 | NR_159261.1 Bifidobacterium callitrichidarum strain TRI 5 16S ribosomal RNA, partial sequence | NR_159261.1 | 1374 | 279 | 686 | 29.69 | 527.4 | 2E-149 | 369/409 | 90 | 7/409 | 1 | Plus | Plus | Bacteria; Actinobacteria; Actinomycetia; Bifidobacteriales; Bifidobacteriaceae; Bifidobacterium; Bifidobacterium callitrichidarum | Bifidobacterium callitrichidarum | Bifidobacterium | Bifidobacteriaceae | Bifidobacteriales | Actinomycetia | Actinobacteria | Bacteria |
| ASV3174 | 422 | 1 | 1 | 422 | 100 | NR_040877.1 Prevotella copri DSM 18205 strain CB7 16S ribosomal RNA, partial sequence | NR_040877.1 | 1494 | 358 | 779 | 28.25 | 749 | 0 | 416/422 | 98 | 0/422 | 0 | Plus | Plus | Bacteria; Bacteroidetes; Bacteroidia; Bacteroidales; Prevotellaceae; Prevotella; Prevotella copri | Prevotella copri | Prevotella | Prevotellaceae | Bacteroidales | Bacteroidia | Bacteroidetes | Bacteria |
| ASV3175 | 421 | 1 | 1 | 421 | 100 | NR_118334.1 Alloprevotella rava strain 81/4-12 16S ribosomal RNA, partial sequence | NR_118334.1 | 1455 | 338 | 759 | 29 | 584.7 | 1E-166 | 387/422 | 91 | 1/422 | 0 | Plus | Plus | Bacteria; Bacteroidetes; Bacteroidia; Bacteroidales; Prevotellaceae; Alloprevotella; Alloprevotella rava | Alloprevotella rava | Alloprevotella | Prevotellaceae | Bacteroidales | Bacteroidia | Bacteroidetes | Bacteria |
| ASV3176 | 404 | 1 | 1 | 404 | 100 | NR_119085.1 [Clostridium] polysaccharolyticum strain DSM 1801 16S ribosomal RNA, partial sequence | NR_119085.1 | 1503 | 364 | 763 | 26.61 | 444.3 | 2E-124 | 352/406 | 86 | 8/406 | 1 | Plus | Plus | Bacteria; Firmicutes; Clostridia; Eubacteriales; Lachnospiraceae; Lachnoclostridium; [Clostridium] polysaccharolyticum | [Clostridium] polysaccharolyticum | Lachnoclostridium | Lachnospiraceae | Eubacteriales | Clostridia | Firmicutes | Bacteria |
| ASV3177 | 403 | 1 | 1 | 403 | 100 | NR_104907.1 Eubacterium coprostanoligenes strain HL 16S ribosomal RNA, partial sequence | NR_104907.1 | 1446 | 332 | 734 | 27.87 | 701 | 0 | 395/403 | 98 | 0/403 | 0 | Plus | Plus | Bacteria; Firmicutes; Clostridia; Eubacteriales; Eubacteriaceae; Eubacterium; Eubacterium coprostanoligenes | Eubacterium coprostanoligenes | Eubacterium | Eubacteriaceae | Eubacteriales | Clostridia | Firmicutes | Bacteria |
| ASV3178 | 405 | 1 | 1 | 405 | 100 | NR_159227.1 Monoglobus pectinilyticus strain 14 16S ribosomal RNA, partial sequence | NR_159227.1 | 1493 | 368 | 769 | 26.93 | 507.1 | 3E-143 | 366/409 | 89 | 11/409 | 2 | Plus | Plus | Bacteria; Firmicutes; Clostridia; Eubacteriales; Oscillospiraceae; Monoglobus; Monoglobus pectinilyticus | Monoglobus pectinilyticus | Monoglobus | Oscillospiraceae | Eubacteriales | Clostridia | Firmicutes | Bacteria |
| ASV3179 | 401 | 3 | 1 | 401 | 100 | NR_116939.1 Olsenella profusa DSM 13989 strain CCUG 45371 16S ribosomal RNA, partial sequence | NR_116939.1 | 1428 | 328 | 735 | 28.57 | 531.1 | 2E-150 | 369/408 | 90 | 7/408 | 1 | Plus | Plus | Bacteria; Actinobacteria; Coriobacteriia; Coriobacteriales; Atopobiaceae; Olsenella; Olsenella profusa | Olsenella profusa | Olsenella | Atopobiaceae | Coriobacteriales | Coriobacteriia | Actinobacteria | Bacteria |
| ASV3180 | 405 | 1 | 1 | 405 | 100 | NR_144611.1 Flintibacter butyricus strain BLS21 16S ribosomal RNA, partial sequence | NR_144611.1 | 808 | 299 | 703 | 50.12 | 577.3 | 2E-164 | 374/405 | 92 | 0/405 | 0 | Plus | Plus | Bacteria; Firmicutes; Clostridia; Eubacteriales; ; Flintibacter; Flintibacter butyricus | Flintibacter butyricus | Flintibacter | | Eubacteriales | Clostridia | Firmicutes | Bacteria |
| [illegible] | [illegible] | [illegible] | [illegible] | [illegible] | [illegible] | [illegible] | [illegible] | [illegible] | [illegible] | [illegible] | [illegible] | [illegible] | [illegible] | [illegible] | [illegible] | [illegible] | [illegible] | [illegible] | [illegible] | [illegible] | [illegible] | [illegible] | [illegible] | [illegible] | [illegible] | [illegible] | [illegible] |

| | | | | | | | | | | | | | | | |
|---|---|---|---|---|---|---|---|---|---|---|---|---|---|---|---|
| ASV3198 | 422 | 5 | 1 | 422 | 100 | NR_119109.1 Pseudescherichia vulneris strain ATCC 33821 16S ribosomal RNA, partial sequence | NR_119109.1 | 1443 | 344 | 770 | 29.59 | 529.3 | 6E-150 | 385/431 | 89 |
| ASV3199 | 422 | 1 | 1 | 422 | 100 | NR_024815.1 Prevotella shahii strain EHS11 16S ribosomal RNA, partial sequence | NR_024815.1 | 1490 | 356 | 777 | 28.32 | 603.1 | 3E-172 | 392/424 | 92 |
| ASV3200 | 405 | 3 | 1 | 405 | 100 | NR_115307.1 Ethanoligenens harbinense YUAN-3 16S ribosomal RNA, partial sequence | NR_115307.1 | 1497 | 340 | 743 | 26.99 | 497.9 | 2E-140 | 362/407 | 88 |
| ASV3201 | 402 | 1 | 1 | 402 | 100 | NR_104783.1 Acetivibrio ethanolgignens strain 77-6 16S ribosomal RNA, partial sequence | NR_104783.1 | 1529 | 370 | 771 | 26.29 | 710.2 | 0 | 396/402 | 98 |
| ASV3202 | 427 | 1 | 1 | 427 | 100 | NR_029348.1 Treponema brennaborense strain DD5/3 16S ribosomal RNA, partial sequence | NR_029348.1 | 1497 | 305 | 732 | 28.59 | 459.1 | 8E-129 | 369/429 | 86 |
| ASV3203 | 422 | 2 | 1 | 422 | 100 | NR_113068.1 Bacteroides fluxus YIT 12057 strain JCM 16101 16S ribosomal RNA, partial sequence | NR_113068.1 | 1486 | 353 | 774 | 28.4 | 780.4 | 0 | 422/422 | 100 |
| ASV3204 | 401 | 1 | 1 | 401 | 100 | NR_119085.1 [Clostridium] polysaccharolyticum strain DSM 1801 16S ribosomal RNA, partial sequence | NR_119085.1 | 1503 | 364 | 763 | 26.61 | 429.5 | 6E-120 | 347/403 | 86 |
| ASV3205 | 427 | 1 | 1 | 427 | 100 | NR_028816.1 Turicibacter sanguinis strain MOL361 16S ribosomal RNA, partial sequence | NR_028816.1 | 1508 | 327 | 753 | 28.32 | 451.7 | 1E-126 | 366/427 | 85 |
| ASV3206 | 422 | 1 | 1 | 422 | 100 | NR_118219.1 Alistipes senegalensis JC50 16S ribosomal RNA, partial sequence | NR_118219.1 | 1480 | 349 | 770 | 28.51 | 774.9 | 0 | 421/422 | 99 |
| ASV3209 | 405 | 2 | 1 | 405 | 100 | NR_074114.1 Elusimicrobium minutum strain Pei191 16S ribosomal RNA, partial sequence | NR_074114.1 | 1433 | 356 | 760 | 28.26 | 660.4 | 0 | 391/407 | 96 |
| ASV3210 | 422 | 2 | 1 | 422 | 100 | NR_116726.1 Prevotella scopos JCM 17725 strain W2052 16S ribosomal RNA, partial sequence | NR_116726.1 | 1453 | 338 | 759 | 29.04 | 597.6 | 2E-170 | 390/423 | 92 |
| ASV3211 | 405 | 1 | 1 | 405 | 100 | NR_118554.1 Intestinimonas butyriciproducens strain SRB-521-5-I 16S ribosomal RNA, partial sequence | NR_118554.1 | 1475 | 346 | 750 | 27.46 | 510.8 | 2E-144 | 363/406 | 89 |
| ASV3212 | 402 | 1 | 5 | 402 | 99 | NR_117516.1 Devosia psychrophila strain Cr7-05 16S ribosomal RNA, partial sequence | NR_117516.1 | 1474 | 321 | 718 | 27 | 442.5 | 8E-124 | 347/400 | 86 |
| ASV3213 | 403 | 1 | 1 | 403 | 100 | NR_118554.1 Intestinimonas butyriciproducens strain SRB-521-5-I 16S ribosomal RNA, partial sequence | NR_118554.1 | 1475 | 346 | 750 | 27.46 | 525.6 | 7E-149 | 365/405 | 90 |
| ASV3214 | 422 | 2 | 1 | 422 | 100 | NR_113119.1 Prevotella oris strain JCM 8540 16S ribosomal RNA, partial sequence | NR_113119.1 | 1481 | 345 | 766 | 28.49 | 603.1 | 3E-172 | 392/424 | 92 |
| ASV3216 | 405 | 1 | 1 | 405 | 100 | NR_144742.1 Christensenella massiliensis strain Marseille-P2438 16S ribosomal RNA, partial sequence | NR_144742.1 | 1499 | 369 | 773 | 27.02 | 466.5 | 4E-131 | 354/405 | 87 |
| ASV3218 | 427 | 1 | 1 | 427 | 100 | NR_028816.1 Turicibacter sanguinis strain MOL361 16S ribosomal RNA, partial sequence | NR_028816.1 | 1508 | 327 | 753 | 28.32 | 446.2 | 6E-125 | 365/427 | 85 |
| ASV3219 | 402 | 2 | 3 | 402 | 99.5 | NR_024948.1 Campylobacter hyointestinalis subsp. lawsonii strain CHY5 16S ribosomal RNA, partial sequence | NR_024948.1 | 1419 | 320 | 720 | 28.26 | 590.2 | 3E-168 | 377/404 | 93 |
| ASV3220 | 427 | 4 | 3 | 427 | 99.53 | NR_041781.1 Streptococcus alactolyticus strain ATCC 43077 16S ribosomal RNA, partial sequence | NR_041781.1 | 1437 | 348 | 772 | 29.58 | 553.3 | 4E-157 | 387/429 | 90 |
| ASV3221 | 419 | 1 | 1 | 419 | 100 | NR_042150.1 Fibrobacter intestinalis strain NR9 16S ribosomal RNA, partial sequence | NR_042150.1 | 1436 | 325 | 743 | 29.18 | 769.3 | 0 | 418/419 | 99 |
| ASV3222 | 405 | 1 | 1 | 405 | 100 | NR_042834.1 Alkalibacter saccharofermentans strain Z-79820 16S ribosomal RNA, partial sequence | NR_042834.1 | 1465 | 338 | 741 | 27.58 | 420.3 | 4E-117 | 348/407 | 85 |
| ASV3223 | 422 | 1 | 1 | 422 | 100 | NR_041508.1 Barnesiella viscericola strain C46 16S ribosomal RNA, partial sequence | NR_041508.1 | 1461 | 342 | 762 | 28.82 | 457.2 | 3E-128 | 365/423 | 86 |
| ASV3224 | 426 | 1 | 1 | 425 | 99.77 | NR_028840.1 Mitsuokella jalaludinii strain M9 16S ribosomal RNA, partial sequence | NR_028840.1 | 1543 | 362 | 787 | 27.61 | 641.9 | 0 | 401/427 | 93 |
| ASV3225 | 404 | 1 | 1 | 404 | 100 | NR_102884.1 Ruminococcus champanellensis 18P13 = JCM 17042 16S ribosomal RNA, partial sequence | NR_102884.1 | 1494 | 348 | 749 | 26.91 | 689.9 | 0 | 394/404 | 97 |
| ASV3226 | 405 | 3 | 1 | 405 | 100 | NR_115307.1 Ethanoligenens harbinense YUAN-3 16S ribosomal RNA, partial sequence | NR_115307.1 | 1497 | 340 | 743 | 26.99 | 558.8 | 7E-159 | 373/407 | 91 |
| ASV3227 | 402 | 2 | 1 | 402 | 100 | NR_042144.1 Clostridium butyricum strain VPI3266 16S ribosomal RNA, partial sequence | NR_042144.1 | 1425 | 338 | 739 | 28.21 | 688.1 | 0 | 392/402 | 97 |
| ASV3229 | 403 | 1 | 1 | 403 | 100 | NR_119085.1 [Clostridium] polysaccharolyticum strain DSM 1801 16S ribosomal RNA, partial sequence | NR_119085.1 | 1503 | 364 | 763 | 26.61 | 630.8 | 1E-180 | 383/403 | 95 |
| ASV3230 | 404 | 3 | 1 | 404 | 100 | NR_074793.2 Oscillibacter valericigenes strain Sjm18-20 16S ribosomal RNA, complete sequence | NR_074793.2 | 1530 | 372 | 775 | 26.41 | 614.2 | 2E-175 | 381/405 | 94 |
| ASV3231 | 427 | 2 | 1 | 427 | 100 | NR_037018.1 Acidaminococcus fermentans strain VR4 16S ribosomal RNA gene, partial sequence | NR_037018.1 | 1516 | 349 | 775 | 28.17 | 728.7 | 0 | 416/427 | 97 |
| ASV3233 | 402 | 1 | 1 | 402 | 100 | NR_159227.1 Monoglobus pectinilyticus strain 14 16S ribosomal RNA, partial sequence | NR_159227.1 | 1493 | 368 | 769 | 26.93 | 660.4 | 0 | 387/402 | 96 |
| ASV3234 | 405 | 2 | 1 | 405 | 100 | NR_165727.1 Anaerotignum faecicola strain KGMB03357 16S ribosomal RNA, complete sequence | NR_165727.1 | 1535 | 375 | 780 | 26.45 | 610.5 | 2E-174 | 381/406 | 93 |
| ASV3235 | 402 | 2 | 1 | 402 | 100 | NR_042144.1 Clostridium butyricum strain VPI3266 16S ribosomal RNA, partial sequence | NR_042144.1 | 1425 | 338 | 739 | 28.21 | 726.9 | 0 | 399/402 | 99 |
| ASV3237 | 426 | 1 | 1 | 426 | 100 | NR_114696.1 Anaerovibrio lipolyticus DSM 3074 16S ribosomal RNA, partial sequence | NR_114696.1 | 1526 | 372 | 797 | 27.92 | 701 | 0 | 409/426 | 96 |
| ASV3238 | 404 | 1 | 1 | 404 | 100 | NR_104907.1 Eubacterium coprostanoligenes strain HL 16S ribosomal RNA, partial sequence | NR_104907.1 | 1446 | 332 | 734 | 27.87 | 573.6 | 3E-163 | 373/404 | 92 |

[illegible]

| | | | | | | | | | | | | | | | | | | | | | | | | | | | |
|---|---|---|---|---|---|---|---|---|---|---|---|---|---|---|---|---|---|---|---|---|---|---|---|---|---|---|---|
| ASV3505 | 403 | 2 | 1 | 403 | 100 | NR_156080.1 Kineothrix alysoides strain KNHs209 16S ribosomal RNA, partial sequence | NR_156080.1 | 1410 | 353 | 754 | 28.51 | 654.8 | 0 | 387/403 | 96 | 1/403 | 0 | Plus | Plus | Bacteria; Firmicutes; Clostridia; Eubacteriales; Lachnospiraceae; Kineothrix; Kineothrix alysoides | Kineothrix alysoides | Kineothrix | Lachnospiraceae | Eubacteriales | Clostridia | Firmicutes | Bacteria |
| ASV3506 | 402 | 3 | 1 | 402 | 100 | NR_114728.1 Clostridium tertium strain ATCC 14573 16S ribosomal RNA, partial sequence | NR_114728.1 | 1415 | 304 | 705 | 28.41 | 621.6 | 9E-178 | 380/402 | 94 | 0/402 | 0 | Plus | Plus | Bacteria; Firmicutes; Clostridia; Eubacteriales; Clostridiaceae; Clostridium; Clostridium tertium | Clostridium tertium | Clostridium | Clostridiaceae | Eubacteriales | Clostridia | Firmicutes | Bacteria |
| ASV3508 | 422 | 1 | 1 | 422 | 100 | NR_144750.1 Prevotellamassilia timonensis strain Marseille-P2831 16S ribosomal RNA, partial sequence | NR_144750.1 | 1493 | 358 | 779 | 28.27 | 542.2 | 8E-154 | 379/422 | 89 | 0/422 | 0 | Plus | Plus | Bacteria; Bacteroidetes; Bacteroidia; Bacteroidales; Prevotellaceae; Prevotellamassilia; Prevotellamassilia timonensis | Prevotellamassilia timonensis | Prevotellamassilia | Prevotellaceae | Bacteroidales | Bacteroidia | Bacteroidetes | Bacteria |
| ASV3510 | 422 | 1 | 1 | 422 | 100 | NR_041285.1 Prevotella multisaccharivorax strain PPPA20 16S ribosomal RNA, partial sequence | NR_041285.1 | 1491 | 356 | 777 | 28.3 | 569.9 | 3E-162 | 385/423 | 91 | 2/423 | 0 | Plus | Plus | Bacteria; Bacteroidetes; Bacteroidia; Bacteroidales; Prevotellaceae; Prevotella; Prevotella multisaccharivorax | Prevotella multisaccharivorax | Prevotella | Prevotellaceae | Bacteroidales | Bacteroidia | Bacteroidetes | Bacteria |
| ASV3512 | 421 | 2 | 1 | 421 | 100 | NR_113073.1 Barnesiella intestinihominis strain JCM 15079 16S ribosomal RNA, partial sequence | NR_113073.1 | 1481 | 346 | 767 | 28.49 | 512.6 | 6E-145 | 377/425 | 88 | 7/425 | 1 | Plus | Plus | Bacteria; Bacteroidetes; Bacteroidia; Bacteroidales; Barnesiellaceae; Barnesiella; Barnesiella intestinihominis | Barnesiella intestinihominis | Barnesiella | Barnesiellaceae | Bacteroidales | Bacteroidia | Bacteroidetes | Bacteria |
| ASV3513 | 405 | 5 | 1 | 405 | 100 | NR_114728.1 Clostridium tertium strain ATCC 14573 16S ribosomal RNA, partial sequence | NR_114728.1 | 1415 | 304 | 705 | 28.41 | 595.7 | 5E-170 | 378/405 | 93 | 3/405 | 0 | Plus | Plus | Bacteria; Firmicutes; Clostridia; Eubacteriales; Clostridiaceae; Clostridium; Clostridium tertium | Clostridium tertium | Clostridium | Clostridiaceae | Eubacteriales | Clostridia | Firmicutes | Bacteria |
| ASV3515 | 404 | 1 | 1 | 404 | 100 | NR_025930.1 Ruminococcus bromii strain ATCC 27255 16S ribosomal RNA, partial sequence | NR_025930.1 | 1380 | 313 | 716 | 29.28 | 653 | 0 | 388/405 | 95 | 2/405 | 0 | Plus | Plus | Bacteria; Firmicutes; Clostridia; Eubacteriales; Oscillospiraceae; Ruminococcus; Ruminococcus bromii | Ruminococcus bromii | Ruminococcus | Oscillospiraceae | Eubacteriales | Clostridia | Firmicutes | Bacteria |
| ASV3516 | 427 | 1 | 1 | 427 | 100 | NR_108477.1 Streptococcus porcorum strain 682/03 16S ribosomal RNA, partial sequence | NR_108477.1 | 1380 | 268 | 694 | 30.94 | 728.7 | 0 | 419/430 | 97 | 6/430 | 1 | Plus | Plus | Bacteria; Firmicutes; Bacilli; Lactobacillales; Streptococcaceae; Streptococcus; Streptococcus porcorum | Streptococcus porcorum | Streptococcus | Streptococcaceae | Lactobacillales | Bacilli | Firmicutes | Bacteria |
| ASV3517 | 402 | 1 | 1 | 402 | 100 | NR_118651.1 [Clostridium] aminophilum strain F 16S ribosomal RNA, partial sequence | NR_118651.1 | 1477 | 372 | 773 | 27.22 | 510.8 | 2E-144 | 361/404 | 89 | 4/404 | 0 | Plus | Plus | Bacteria; Firmicutes; Clostridia; Eubacteriales; Lachnospiraceae; Lachnoclostridium; [Clostridium] aminophilum | [Clostridium] aminophilum | Lachnoclostridium | Lachnospiraceae | Eubacteriales | Clostridia | Firmicutes | Bacteria |
| ASV3518 | 427 | 1 | 1 | 427 | 100 | NR_028862.1 Allisonella histaminiformans strain MR2 16S ribosomal RNA, partial sequence | NR_028862.1 | 1537 | 376 | 802 | 27.78 | 767.5 | 0 | 423/427 | 99 | 0/427 | 0 | Plus | Plus | Bacteria; Firmicutes; Negativicutes; Veillonellales; Veillonellaceae; Allisonella; Allisonella histaminiformans | Allisonella histaminiformans | Allisonella | Veillonellaceae | Veillonellales | Negativicutes | Firmicutes | Bacteria |
| ASV3521 | 404 | 1 | 1 | 404 | 100 | NR_118554.1 Intestinimonas butyriciproducens strain SRB-521-5-I 16S ribosomal RNA, partial sequence | NR_118554.1 | 1475 | 346 | 750 | 27.46 | 547.7 | 2E-155 | 369/405 | 91 | 1/405 | 0 | Plus | Plus | Bacteria; Firmicutes; Clostridia; Eubacteriales; ; Intestinimonas; Intestinimonas butyriciproducens | Intestinimonas butyriciproducens | Intestinimonas | | Eubacteriales | Clostridia | Firmicutes | Bacteria |
| ASV3522 | 404 | 1 | 1 | 404 | 100 | NR_114326.1 Fusicatenibacter saccharivorans strain HT03-11 16S ribosomal RNA, partial sequence | NR_114326.1 | 1423 | 351 | 752 | 28.25 | 507.1 | 3E-143 | 361/404 | 89 | 2/404 | 0 | Plus | Plus | Bacteria; Firmicutes; Clostridia; Eubacteriales; Lachnospiraceae; Fusicatenibacter; Fusicatenibacter saccharivorans | Fusicatenibacter saccharivorans | Fusicatenibacter | Lachnospiraceae | Eubacteriales | Clostridia | Firmicutes | Bacteria |
| ASV3525 | 404 | 1 | 1 | 404 | 100 | NR_118156.1 Oscillibacter ruminantium GH1 16S ribosomal RNA, partial sequence | NR_118156.1 | 1409 | 321 | 724 | 28.67 | 603.1 | 3E-172 | 379/405 | 93 | 2/405 | 0 | Plus | Plus | Bacteria; Firmicutes; Clostridia; Eubacteriales; Oscillospiraceae; Oscillibacter; Oscillibacter ruminantium | Oscillibacter ruminantium | Oscillibacter | Oscillospiraceae | Eubacteriales | Clostridia | Firmicutes | Bacteria |
| ASV3526 | 403 | 2 | 2 | 403 | 99.75 | NR_027579.1 Ruminococcus lactaris ATCC 29176 16S ribosomal RNA, partial sequence | NR_027579.1 | 1402 | 334 | 734 | 28.6 | 671.5 | 0 | 389/402 | 96 | 1/402 | 0 | Plus | Plus | Bacteria; Firmicutes; Clostridia; Eubacteriales; Lachnospiraceae; Mediterraneibacter; [Ruminococcus] lactaris | [Ruminococcus] lactaris | Mediterraneibacter | Lachnospiraceae | Eubacteriales | Clostridia | Firmicutes | Bacteria |
| ASV3528 | 422 | 1 | 1 | 422 | 100 | NR_041364.1 Prevotella stercorea DSM 18206 strain CB35 16S ribosomal RNA, partial sequence | NR_041364.1 | 1488 | 354 | 775 | 28.36 | 547.7 | 2E-155 | 381/423 | 90 | 2/423 | 0 | Plus | Plus | Bacteria; Bacteroidetes; Bacteroidia; Bacteroidales; Prevotellaceae; Prevotella; Prevotella stercorea | Prevotella stercorea | Prevotella | Prevotellaceae | Bacteroidales | Bacteroidia | Bacteroidetes | Bacteria |
| ASV3529 | 422 | 1 | 1 | 422 | 100 | NR_144750.1 Prevotellamassilia timonensis strain Marseille-P2831 16S ribosomal RNA, partial sequence | NR_144750.1 | 1493 | 358 | 779 | 28.27 | 664.1 | 0 | 401/422 | 95 | 0/422 | 0 | Plus | Plus | Bacteria; Bacteroidetes; Bacteroidia; Bacteroidales; Prevotellaceae; Prevotellamassilia; Prevotellamassilia timonensis | Prevotellamassilia timonensis | Prevotellamassilia | Prevotellaceae | Bacteroidales | Bacteroidia | Bacteroidetes | Bacteria |
| ASV3530 | 402 | 1 | 1 | 402 | 100 | NR_074613.1 [Eubacterium] eligens ATCC 27750 16S ribosomal RNA, partial sequence | NR_074613.1 | 1519 | 367 | 768 | 26.46 | 599.4 | 4E-171 | 376/402 | 93 | 0/402 | 0 | Plus | Plus | Bacteria; Firmicutes; Clostridia; Eubacteriales; Lachnospiraceae; Lachnospira; Lachnospira eligens | Lachnospira eligens | Lachnospira | Lachnospiraceae | Eubacteriales | Clostridia | Firmicutes | Bacteria |
| ASV3531 | 402 | 5 | 1 | 402 | 100 | NR_171498.1 Clostridium fermenticellae strain JN500901 16S ribosomal RNA, partial sequence | NR_171498.1 | 1382 | 302 | 703 | 29.09 | 444.3 | 2E-124 | 351/405 | 86 | 6/405 | 1 | Plus | Plus | Bacteria; Firmicutes; Clostridia; Eubacteriales; Clostridiaceae; Clostridium; Clostridium fermenticellae | Clostridium fermenticellae | Clostridium | Clostridiaceae | Eubacteriales | Clostridia | Firmicutes | Bacteria |
| ASV3532 | 422 | 4 | 1 | 422 | 100 | NR_044864.1 Hallella seregens ATCC 51272 16S ribosomal RNA, partial sequence | NR_044864.1 | 1463 | 335 | 756 | 28.84 | 575.4 | 7E-164 | 385/422 | 91 | 0/422 | 0 | Plus | Plus | Bacteria; Bacteroidetes; Bacteroidia; Bacteroidales; Prevotellaceae; Prevotella; Hallella seregens | Hallella seregens | Prevotella | Prevotellaceae | Bacteroidales | Bacteroidia | Bacteroidetes | Bacteria |
| ASV3534 | 422 | 2 | 1 | 422 | 100 | NR_074376.1 Parabacteroides distasonis ATCC 8503 16S ribosomal RNA, partial sequence | NR_074376.1 | 1497 | 368 | 789 | 28.19 | 564.3 | 2E-160 | 383/422 | 90 | 0/422 | 0 | Plus | Plus | Bacteria; Bacteroidetes; Bacteroidia; Bacteroidales; Tannerellaceae; Parabacteroides; Parabacteroides distasonis | Parabacteroides distasonis | Parabacteroides | Tannerellaceae | Bacteroidales | Bacteroidia | Bacteroidetes | Bacteria |
| ASV3535 | 401 | 1 | 1 | 401 | 100 | NR_027573.1 Intestinibacter bartlettii strain WAL 16138 16S ribosomal RNA, partial sequence | NR_027573.1 | 1429 | 320 | 720 | 28.06 | 608.7 | 7E-174 | 377/401 | 94 | 0/401 | 0 | Plus | Plus | Bacteria; Firmicutes; Clostridia; Eubacteriales; Peptostreptococcaceae; Intestinibacter; Intestinibacter bartlettii | Intestinibacter bartlettii | Intestinibacter | Peptostreptococcaceae | Eubacteriales | Clostridia | Firmicutes | Bacteria |
| ASV3536 | 403 | 2 | 3 | 403 | 99.5 | NR_074536.1 Cellulosilyticum lentocellum DSM 5427 16S ribosomal RNA, partial sequence | NR_074536.1 | 1512 | 353 | 754 | 26.59 | 414.8 | 2E-115 | 343/402 | 85 | 1/402 | 0 | Plus | Plus | Bacteria; Firmicutes; Clostridia; Eubacteriales; Lachnospiraceae; Cellulosilyticum; Cellulosilyticum lentocellum | Cellulosilyticum lentocellum | Cellulosilyticum | Lachnospiraceae | Eubacteriales | Clostridia | Firmicutes | Bacteria |
| ASV3537 | 402 | 1 | 5 | 402 | 99 | NR_025127.1 Lachnobacterium bovis strain LRC 5382 16S ribosomal RNA, partial sequence | NR_025127.1 | 1453 | 366 | 765 | 27.53 | 411.1 | 2E-114 | 343/402 | 85 | 6/402 | 1 | Plus | Plus | Bacteria; Firmicutes; Clostridia; Eubacteriales; Lachnospiraceae; Lachnobacterium; Lachnobacterium bovis | Lachnobacterium bovis | Lachnobacterium | Lachnospiraceae | Eubacteriales | Clostridia | Firmicutes | Bacteria |
| [illegible] | [illegible] | [illegible] | [illegible] | [illegible] | [illegible] | [illegible] | [illegible] | [illegible] | [illegible] | [illegible] | [illegible] | [illegible] | [illegible] | [illegible] | [illegible] | [illegible] | [illegible] | [illegible] | [illegible] | [illegible] | [illegible] | [illegible] | [illegible] | [illegible] | [illegible] | [illegible] | [illegible] |

| | | | | | | | | | | | | | | | | | | | | | | | | | | | |
|---|---|---|---|---|---|---|---|---|---|---|---|---|---|---|---|---|---|---|---|---|---|---|---|---|---|---|---|
| ASV3845 | 427 | 1 | 1 | 427 | 100 | NR_146687.1 Breznakia pachnodae strain Pei061 16S ribosomal RNA, partial sequence | NR_146687.1 | 1499 | 367 | 792 | 28.42 | 455.4 | 1E-127 | 372/432 | 86 | 11/432 | 2 | Plus | Plus | Bacteria; Firmicutes; Erysipelotrichia; Erysipelotrichales; Erysipelotrichaceae; Breznakia; Breznakia pachnodae | Breznakia pachnodae | Breznakia | Erysipelotrichaceae | Erysipelotrichales | Erysipelotrichia | Firmicutes | Bacteria |
| ASV3848 | 403 | 5 | 1 | 403 | 100 | NR_114728.1 Clostridium tertium strain ATCC 14573 16S ribosomal RNA, partial sequence | NR_114728.1 | 1415 | 304 | 705 | 28.41 | 593.9 | 2E-169 | 379/406 | 93 | 7/406 | 1 | Plus | Plus | Bacteria; Firmicutes; Clostridia; Eubacteriales; Clostridiaceae; Clostridium; Clostridium tertium | Clostridium tertium | Clostridium | Clostridiaceae | Eubacteriales | Clostridia | Firmicutes | Bacteria |
| ASV3849 | 422 | 1 | 1 | 422 | 100 | NR_113207.1 Bacteroides stercorirosoris JCM 17103 16S ribosomal RNA, partial sequence | NR_113207.1 | 1484 | 351 | 772 | 28.44 | 752.7 | 0 | 417/422 | 98 | 0/422 | 0 | Plus | Plus | Bacteria; Bacteroidetes; Bacteroidia; Bacteroidales; Bacteroidaceae; Bacteroides; Bacteroides stercorirosoris | Bacteroides stercorirosoris | Bacteroides | Bacteroidaceae | Bacteroidales | Bacteroidia | Bacteroidetes | Bacteria |
| ASV3850 | 402 | 1 | 1 | 402 | 100 | NR_024836.1 Helicobacter ganmani strain CMRI H02 16S ribosomal RNA, partial sequence | NR_024836.1 | 1418 | 313 | 714 | 28.35 | 743.5 | 0 | 402/402 | 100 | 0/402 | 0 | Plus | Plus | Bacteria; Proteobacteria; Epsilonproteobacteria; Campylobacterales; Helicobacteraceae; Helicobacter; Helicobacter ganmani | Helicobacter ganmani | Helicobacter | Helicobacteraceae | Campylobacterales | Epsilonproteobacteria | Proteobacteria | Bacteria |
| ASV3852 | 427 | 1 | 1 | 427 | 100 | NR_044111.1 Selenomonas bovis strain WG 16S ribosomal RNA, partial sequence | NR_044111.1 | 1525 | 370 | 796 | 28 | 634.5 | 0 | 404/432 | 93 | 10/432 | 2 | Plus | Plus | Bacteria; Firmicutes; Negativicutes; Selenomonadales; Selenomonadaceae; Selenomonas; Selenomonas bovis | Selenomonas bovis | Selenomonas | Selenomonadaceae | Selenomonadales | Negativicutes | Firmicutes | Bacteria |
| ASV3854 | 427 | 1 | 1 | 426 | 99.77 | NR_044111.1 Selenomonas bovis strain WG 16S ribosomal RNA, partial sequence | NR_044111.1 | 1525 | 370 | 795 | 27.93 | 610.5 | 2E-174 | 396/428 | 92 | 4/428 | 0 | Plus | Plus | Bacteria; Firmicutes; Negativicutes; Selenomonadales; Selenomonadaceae; Selenomonas; Selenomonas bovis | Selenomonas bovis | Selenomonas | Selenomonadaceae | Selenomonadales | Negativicutes | Firmicutes | Bacteria |
| ASV3855 | 402 | 1 | 1 | 402 | 100 | NR_026476.1 Succinivibrio dextrinosolvens strain 0554 16S ribosomal RNA, partial sequence | NR_026476.1 | 1463 | 331 | 732 | 27.48 | 499.7 | 4E-141 | 358/402 | 89 | 0/402 | 0 | Plus | Plus | Bacteria; Proteobacteria; Gammaproteobacteria; Aeromonadales; Succinivibrionaceae; Succinivibrio; Succinivibrio dextrinosolvens | Succinivibrio dextrinosolvens | Succinivibrio | Succinivibrionaceae | Aeromonadales | Gammaproteobacteria | Proteobacteria | Bacteria |
| ASV3856 | 420 | 3 | 1 | 420 | 100 | NR_041781.1 Streptococcus alactolyticus strain ATCC 43077 16S ribosomal RNA, partial sequence | NR_041781.1 | 1437 | 346 | 772 | 29.71 | 560.7 | 2E-159 | 388/428 | 90 | 9/428 | 2 | Plus | Plus | Bacteria; Firmicutes; Bacilli; Lactobacillales; Streptococcaceae; Streptococcus; Streptococcus alactolyticus | Streptococcus alactolyticus | Streptococcus | Streptococcaceae | Lactobacillales | Bacilli | Firmicutes | Bacteria |
| ASV3857 | 427 | 2 | 1 | 427 | 100 | NR_117375.1 Streptococcus danieliae strain ERD01G 16S ribosomal RNA, partial sequence | NR_117375.1 | 1419 | 314 | 740 | 30.09 | 557 | 3E-158 | 386/428 | 90 | 2/428 | 0 | Plus | Plus | Bacteria; Firmicutes; Bacilli; Lactobacillales; Streptococcaceae; Streptococcus; Streptococcus danieliae | Streptococcus danieliae | Streptococcus | Streptococcaceae | Lactobacillales | Bacilli | Firmicutes | Bacteria |
| ASV3858 | 427 | 4 | 1 | 427 | 100 | NR_041781.1 Streptococcus alactolyticus strain ATCC 43077 16S ribosomal RNA, partial sequence | NR_041781.1 | 1437 | 346 | 772 | 29.71 | 623.4 | 3E-178 | 401/431 | 93 | 8/431 | 1 | Plus | Plus | Bacteria; Firmicutes; Bacilli; Lactobacillales; Streptococcaceae; Streptococcus; Streptococcus alactolyticus | Streptococcus alactolyticus | Streptococcus | Streptococcaceae | Lactobacillales | Bacilli | Firmicutes | Bacteria |
| ASV3860 | 428 | 1 | 1 | 427 | 99.77 | NR_102980.1 Megasphaera elsdenii DSM 20460 16S ribosomal RNA, partial sequence | NR_102980.1 | 1552 | 369 | 794 | 27.45 | 560.7 | 2E-159 | 388/429 | 90 | 5/429 | 1 | Plus | Plus | Bacteria; Firmicutes; Negativicutes; Veillonellales; Veillonellaceae; Megasphaera; Megasphaera elsdenii | Megasphaera elsdenii | Megasphaera | Veillonellaceae | Veillonellales | Negativicutes | Firmicutes | Bacteria |
| ASV3862 | 427 | 1 | 1 | 427 | 100 | NR_025600.1 Sutterella stercoricanis strain 5BAC4 16S ribosomal RNA, partial sequence | NR_025600.1 | 1455 | 319 | 745 | 29.35 | 761.9 | 0 | 422/427 | 98 | 0/427 | 0 | Plus | Plus | Bacteria; Proteobacteria; Betaproteobacteria; Burkholderiales; Sutterellaceae; Sutterella; Sutterella stercoricanis | Sutterella stercoricanis | Sutterella | Sutterellaceae | Burkholderiales | Betaproteobacteria | Proteobacteria | Bacteria |
| ASV3864 | 421 | 2 | 1 | 421 | 100 | NR_113039.1 Solobacterium moorei strain JCM 10645 16S ribosomal RNA, partial sequence | NR_113039.1 | 1486 | 352 | 772 | 28.33 | 568 | 1E-161 | 384/422 | 90 | 2/422 | 0 | Plus | Plus | Bacteria; Firmicutes; Erysipelotrichia; Erysipelotrichales; Erysipelotrichaceae; Solobacterium; Solobacterium moorei | Solobacterium moorei | Solobacterium | Erysipelotrichaceae | Erysipelotrichales | Erysipelotrichia | Firmicutes | Bacteria |
| ASV3865 | 402 | 1 | 1 | 402 | 100 | NR_159261.1 Bifidobacterium callitrichidarum strain TRI 5 16S ribosomal RNA, partial sequence | NR_159261.1 | 1374 | 279 | 686 | 29.69 | 560.7 | 2E-159 | 374/408 | 91 | 6/408 | 1 | Plus | Plus | Bacteria; Actinobacteria; Actinomycetia; Bifidobacteriales; Bifidobacteriaceae; Bifidobacterium; Bifidobacterium callitrichidarum | Bifidobacterium callitrichidarum | Bifidobacterium | Bifidobacteriaceae | Bifidobacteriales | Actinomycetia | Actinobacteria | Bacteria |
| ASV3867 | 402 | 3 | 1 | 402 | 100 | NG_042882.1 Helicobacter mastomyrinus strain MIT 97-5574 16S ribosomal RNA gene, partial sequence | NG_042882.1 | 1760 | 623 | 1024 | 22.84 | 732.4 | 0 | 400/402 | 99 | 0/402 | 0 | Plus | Plus | Bacteria; Proteobacteria; Epsilonproteobacteria; Campylobacterales; Helicobacteraceae; Helicobacter; Helicobacter mastomyrinus | Helicobacter mastomyrinus | Helicobacter | Helicobacteraceae | Campylobacterales | Epsilonproteobacteria | Proteobacteria | Bacteria |
| ASV3868 | 422 | 1 | 1 | 422 | 100 | NR_171512.1 Marinilabilia rubra strain WTE16 16S ribosomal RNA, partial sequence | NR_171512.1 | 1444 | 330 | 751 | 29.22 | 448 | 2E-125 | 366/426 | 85 | 8/426 | 1 | Plus | Plus | Bacteria; Bacteroidetes; Bacteroidia; Marinilabiliales; Marinilabiliaceae; Marinilabilia; Marinilabilia rubra | Marinilabilia rubra | Marinilabilia | Marinilabiliaceae | Marinilabiliales | Bacteroidia | Bacteroidetes | Bacteria |
| ASV3870 | 402 | 1 | 1 | 402 | 100 | NR_074634.1 [Eubacterium] rectale ATCC 33656 16S ribosomal RNA, partial sequence | NR_074634.1 | 1521 | 367 | 768 | 26.43 | 527.4 | 2E-149 | 363/402 | 90 | 0/402 | 0 | Plus | Plus | Bacteria; Firmicutes; Clostridia; Eubacteriales; Lachnospiraceae; ; [Eubacterium] rectale | [Eubacterium] rectale | | Lachnospiraceae | Eubacteriales | Clostridia | Firmicutes | Bacteria |
| ASV3873 | 422 | 1 | 1 | 422 | 100 | NR_113208.1 Parabacteroides chinchillae strain JCM 17104 16S ribosomal RNA, partial sequence | NR_113208.1 | 1489 | 353 | 774 | 28.34 | 625.3 | 7E-179 | 395/423 | 93 | 2/423 | 0 | Plus | Plus | Bacteria; Bacteroidetes; Bacteroidia; Bacteroidales; Tannerellaceae; Parabacteroides; Parabacteroides chinchillae | Parabacteroides chinchillae | Parabacteroides | Tannerellaceae | Bacteroidales | Bacteroidia | Bacteroidetes | Bacteria |
| ASV3874 | 423 | 1 | 1 | 423 | 100 | NR_144750.1 Prevotellamassilia timonensis strain Marseille-P2831 16S ribosomal RNA, partial sequence | NR_144750.1 | 1493 | 358 | 779 | 28.27 | 569.9 | 3E-162 | 387/425 | 91 | 5/425 | 1 | Plus | Plus | Bacteria; Bacteroidetes; Bacteroidia; Bacteroidales; Prevotellaceae; Prevotellamassilia; Prevotellamassilia timonensis | Prevotellamassilia timonensis | Prevotellamassilia | Prevotellaceae | Bacteroidales | Bacteroidia | Bacteroidetes | Bacteria |
| ASV3875 | 405 | 1 | 1 | 405 | 100 | NR_133738.1 Desulfohalotomaculum tongense strain TGB60-1 16S ribosomal RNA, partial sequence | NR_133738.1 | 1447 | 352 | 756 | 27.99 | 477.6 | 2E-134 | 357/406 | 87 | 2/406 | 0 | Plus | Plus | Bacteria; Firmicutes; Clostridia; Eubacteriales; Peptococcaceae; Desulfohalotomaculum; Desulfohalotomaculum tongense | Desulfohalotomaculum tongense | Desulfohalotomaculum | Peptococcaceae | Eubacteriales | Clostridia | Firmicutes | Bacteria |
| ASV3876 | 422 | 4 | 1 | 422 | 100 | NR_113123.1 Prevotella timonensis 4401737 = DSM 22865 = JCM 15640 16S ribosomal RNA, partial sequence | NR_113123.1 | 1493 | 356 | 777 | 28.27 | 603.1 | 3E-172 | 391/423 | 92 | 2/423 | 0 | Plus | Plus | Bacteria; Bacteroidetes; Bacteroidia; Bacteroidales; Prevotellaceae; Prevotella; Prevotella timonensis | Prevotella timonensis | Prevotella | Prevotellaceae | Bacteroidales | Bacteroidia | Bacteroidetes | Bacteria |
| ASV3877 | 422 | 1 | 1 | 422 | 100 | NR_040783.1 Bifidobacterium breve DSM 20213 = JCM 1192 16S ribosomal RNA, partial sequence | NR_040783.1 | 1520 | 345 | 756 | 27.11 | 577.3 | 2E-164 | 387/422 | 91 | 10/422 | 2 | Plus | Plus | Bacteria; Actinobacteria; Actinomycetia; Bifidobacteriales; Bifidobacteriaceae; Bifidobacterium; Bifidobacterium breve | Bifidobacterium breve | Bifidobacterium | Bifidobacteriaceae | Bifidobacteriales | Actinomycetia | Actinobacteria | Bacteria |
| ASV3878 | 422 | 1 | 1 | 422 | 100 | NR_113208.1 Parabacteroides chinchillae strain JCM 17104 16S ribosomal RNA, partial sequence | NR_113208.1 | 1489 | 353 | 774 | 28.34 | 641.9 | 0 | 398/423 | 94 | 2/423 | 0 | Plus | Plus | Bacteria; Bacteroidetes; Bacteroidia; Bacteroidales; Tannerellaceae; Parabacteroides; Parabacteroides chinchillae | Parabacteroides chinchillae | Parabacteroides | Tannerellaceae | Bacteroidales | Bacteroidia | Bacteroidetes | Bacteria |
| ASV3879 | 405 | 1 | 1 | 405 | 100 | NR_159227.1 Monoglobus pectinilyticus strain 14 16S ribosomal RNA, partial sequence | NR_159227.1 | 1493 | 368 | 769 | 26.93 | 573.6 | 3E-163 | 376/407 | 92 | 7/407 | 1 | Plus | Plus | Bacteria; Firmicutes; Clostridia; Eubacteriales; Oscillospiraceae; Monoglobus; Monoglobus pectinilyticus | Monoglobus pectinilyticus | Monoglobus | Oscillospiraceae | Eubacteriales | Clostridia | Firmicutes | Bacteria |
| ASV3882 | 402 | 1 | 3 | 402 | 99.5 | NR_125677.1 Vallitalea pronyensis strain FatNI3 16S ribosomal RNA, partial sequence | NR_125677.1 | 1480 | 365 | 764 | 27.03 | 490.5 | 3E-138 | 357/402 | 88 | 4/402 | 0 | Plus | Plus | Bacteria; Firmicutes; Clostridia; Eubacteriales; Vallitaleaceae; Vallitalea; Vallitalea pronyensis | Vallitalea pronyensis | Vallitalea | Vallitaleaceae | Eubacteriales | Clostridia | Firmicutes | Bacteria |
| ASV3883 | 422 | 2 | 1 | 405 | 95.97 | NR_113102.1 Prevotella dentasini JCM 15908 16S ribosomal RNA, partial sequence | NR_113102.1 | 1494 | 360 | 764 | 27.11 | 516.3 | 5E-146 | 363/405 | 89 | 0/405 | 0 | Plus | Plus | Bacteria; Bacteroidetes; Bacteroidia; Bacteroidales; Prevotellaceae; Prevotella; Prevotella dentasini | Prevotella dentasini | Prevotella | Prevotellaceae | Bacteroidales | Bacteroidia | Bacteroidetes | Bacteria |
| ASV3884 | 402 | 1 | 1 | 402 | 100 | NR_028961.1 Faecalibacterium prausnitzii strain ATCC 27768 16S ribosomal RNA, partial sequence | NR_028961.1 | 1462 | 344 | 745 | 27.5 | 571.7 | 9E-163 | 373/404 | 92 | 4/404 | 0 | Plus | Plus | Bacteria; Firmicutes; Clostridia; Eubacteriales; Oscillospiraceae; Faecalibacterium; Faecalibacterium prausnitzii | Faecalibacterium prausnitzii | Faecalibacterium | Oscillospiraceae | Eubacteriales | Clostridia | Firmicutes | Bacteria |
| ASV3885 | 405 | 1 | 1 | 355 | 87.65 | NR_026476.1 Succinivibrio dextrinosolvens strain 0554 16S ribosomal RNA, partial sequence | NR_026476.1 | 1463 | 331 | 682 | 24.06 | 536.6 | 3E-152 | 334/355 | 94 | 3/355 | 0 | Plus | Plus | Bacteria; Proteobacteria; Gammaproteobacteria; Aeromonadales; Succinivibrionaceae; Succinivibrio; Succinivibrio dextrinosolvens | Succinivibrio dextrinosolvens | Succinivibrio | Succinivibrionaceae | Aeromonadales | Gammaproteobacteria | Proteobacteria | Bacteria |
| ASV3886 | 402 | 2 | 1 | 402 | 100 | NR_115104.1 Helicobacter canadensis strain CCUG 47163 16S ribosomal RNA, partial sequence | NR_115104.1 | 1471 | 314 | 715 | 27.33 | 732.4 | 0 | 400/402 | 99 | 0/402 | 0 | Plus | Plus | Bacteria; Proteobacteria; Epsilonproteobacteria; Campylobacterales; Helicobacteraceae; Helicobacter; Helicobacter canadensis | Helicobacter canadensis | Helicobacter | Helicobacteraceae | Campylobacterales | Epsilonproteobacteria | Proteobacteria | Bacteria |
| ASV3887 | 401 | 1 | 1 | 401 | 100 | NR_158111.1 Paludicola psychrotolerans strain NC1253 16S ribosomal RNA, partial sequence | NR_158111.1 | 1490 | 366 | 766 | 26.91 | 625.3 | 7E-179 | 380/401 | 94 | 0/401 | 0 | Plus | Plus | Bacteria; Firmicutes; Clostridia; Eubacteriales; Oscillospiraceae; Paludicola; Paludicola psychrotolerans | Paludicola psychrotolerans | Paludicola | Oscillospiraceae | Eubacteriales | Clostridia | Firmicutes | Bacteria |
| ASV3888 | 421 | 1 | 1 | 421 | 100 | NR_169358.1 Butyricimonas phoceensis strain AT9 16S ribosomal RNA, partial sequence | NR_169358.1 | 1488 | 352 | 772 | 28.29 | 773 | 0 | 420/421 | 99 | 0/421 | 0 | Plus | Plus | Bacteria; Bacteroidetes; Bacteroidia; Bacteroidales; Odoribacteraceae; Butyricimonas; Butyricimonas phoceensis | Butyricimonas phoceensis | Butyricimonas | Odoribacteraceae | Bacteroidales | Bacteroidia | Bacteroidetes | Bacteria |
| ASV3892 | 404 | 1 | 3 | 404 | 99.5 | NR_125455.1 Hydrogenispora ethanolica strain LX-B 16S ribosomal RNA, partial sequence | NR_125455.1 | 1443 | 337 | 736 | 27.72 | 420.3 | 4E-117 | 348/406 | 85 | 10/406 | 2 | Plus | Plus | Bacteria; Firmicutes; ; ; ; Hydrogenispora; Hydrogenispora ethanolica | Hydrogenispora ethanolica | Hydrogenispora | | | | Firmicutes | Bacteria |
| ASV3893 | 422 | 1 | 1 | 422 | 100 | NR_024570.1 Escherichia coli strain U 5/41 16S ribosomal RNA, partial sequence | NR_024570.1 | 1450 | 349 | 775 | 29.45 | 542.2 | 8E-154 | 386/430 | 89 | 11/430 | 2 | Plus | Plus | Bacteria; Proteobacteria; Gammaproteobacteria; Enterobacterales; Enterobacteriaceae; Escherichia; Escherichia coli | Escherichia coli | Escherichia | Enterobacteriaceae | Enterobacterales | Gammaproteobacteria | Proteobacteria | Bacteria |
| ASV3894 | 405 | 1 | 1 | 405 | 100 | NR_044972.1 Sporobacter termitidis strain SYR 16S ribosomal RNA, partial sequence | NR_044972.1 | 1506 | 348 | 752 | 26.89 | 569.9 | 3E-162 | 372/405 | 91 | 0/405 | 0 | Plus | Plus | Bacteria; Firmicutes; Clostridia; Eubacteriales; Oscillospiraceae; Sporobacter; Sporobacter termitidis | Sporobacter termitidis | Sporobacter | Oscillospiraceae | Eubacteriales | Clostridia | Firmicutes | Bacteria |
| ASV3895 | 405 | 1 | 1 | 405 | 100 | NR_147370.1 Pseudoflavonifractor phocaeensis strain Marseille-P3064 16S ribosomal RNA, partial sequence | NR_147370.1 | 1495 | 366 | 770 | 27.09 | 505.3 | 1E-142 | 363/407 | 89 | 4/407 | 0 | Plus | Plus | Bacteria; Firmicutes; Clostridia; Eubacteriales; Oscillospiraceae; Pseudoflavonifractor; Pseudoflavonifractor phocaeensis | Pseudoflavonifractor phocaeensis | Pseudoflavonifractor | Oscillospiraceae | Eubacteriales | Clostridia | Firmicutes | Bacteria |
| ASV3898 | 421 | 2 | 1 | 421 | 100 | NR_126195.1 Butyricimonas paravirosa strain 214-4 16S ribosomal RNA, partial sequence | NR_126195.1 | 1486 | 352 | 772 | 28.33 | 573.6 | 3E-163 | 386/423 | 91 | 4/423 | 0 | Plus | Plus | Bacteria; Bacteroidetes; Bacteroidia; Bacteroidales; Odoribacteraceae; Butyricimonas; Butyricimonas paravirosa | Butyricimonas paravirosa | Butyricimonas | Odoribacteraceae | Bacteroidales | Bacteroidia | Bacteroidetes | Bacteria |
| ASV3899 | 405 | 1 | 3 | 405 | 99.51 | NR_028683.1 Acidaminobacter hydrogenoformans strain glu 65 16S ribosomal RNA, partial sequence | NR_028683.1 | 1468 | 347 | 747 | 27.32 | 449.9 | 5E-126 | 353/406 | 86 | 8/406 | 1 | Plus | Plus | Bacteria; Firmicutes; Clostridia; Eubacteriales; Clostridiales Family XII. Incertae Sedis; Acidaminobacter; Acidaminobacter hydrogenoformans | Acidaminobacter hydrogenoformans | Acidaminobacter | Clostridiales Family XII. Incertae Sedis | Eubacteriales | Clostridia | Firmicutes | Bacteria |
| ASV3902 | 404 | 3 | 1 | 389 | 96.29 | NR_027557.1 Roseburia intestinalis L1-82 16S ribosomal RNA, partial sequence | NR_027557.1 | 1482 | 360 | 748 | 26.25 | 403.7 | 4E-112 | 333/390 | 85 | 2/390 | 0 | Plus | Plus | Bacteria; Firmicutes; Clostridia; Eubacteriales; Lachnospiraceae; Roseburia; Roseburia intestinalis | Roseburia intestinalis | Roseburia | Lachnospiraceae | Eubacteriales | Clostridia | Firmicutes | Bacteria |
| ASV3904 | 404 | 1 | 1 | 404 | 100 | NR_118675.1 [Eubacterium] siraeum strain ATCC 29066 16S ribosomal RNA, partial sequence | NR_118675.1 | 1453 | 356 | 757 | 27.67 | 586.5 | 3E-167 | 374/404 | 92 | 2/404 | 0 | Plus | Plus | Bacteria; Firmicutes; Clostridia; Eubacteriales; Oscillospiraceae; ; [Eubacterium] siraeum | [Eubacterium] siraeum | | Oscillospiraceae | Eubacteriales | Clostridia | Firmicutes | Bacteria |
| ASV3905 | 422 | 3 | 3 | 422 | 99.53 | NR_109750.1 Parapedobacter pyrenivorans strain P-4 16S ribosomal RNA, partial sequence | NR_109750.1 | 1490 | 356 | 775 | 28.19 | 433.2 | 5E-121 | 362/424 | 85 | 8/424 | 1 | Plus | Plus | Bacteria; Bacteroidetes; Sphingobacteriia; Sphingobacteriales; Sphingobacteriaceae; Parapedobacter; Parapedobacter pyrenivorans | Parapedobacter pyrenivorans | Parapedobacter | Sphingobacteriaceae | Sphingobacteriales | Sphingobacteriia | Bacteroidetes | Bacteria |
| ASV3907 | 404 | 1 | 1 | 404 | 100 | NR_036928.1 Hungatella hathewayi strain 1313 16S ribosomal RNA, partial sequence | NR_036928.1 | 1431 | 350 | 751 | 28.09 | 484.9 | 1E-136 | 358/405 | 88 | 4/405 | 0 | Plus | Plus | Bacteria; Firmicutes; Clostridia; Eubacteriales; Clostridiaceae; Hungatella; Hungatella hathewayi | Hungatella hathewayi | Hungatella | Clostridiaceae | Eubacteriales | Clostridia | Firmicutes | Bacteria |
| ASV3909 | 402 | 1 | 1 | 402 | 100 | NR_159227.1 Monoglobus pectinilyticus strain 14 16S ribosomal RNA, partial sequence | NR_159227.1 | 1493 | 368 | 769 | 26.93 | 649.3 | 0 | 385/402 | 95 | 0/402 | 0 | Plus | Plus | Bacteria; Firmicutes; Clostridia; Eubacteriales; Oscillospiraceae; Monoglobus; Monoglobus pectinilyticus | Monoglobus pectinilyticus | Monoglobus | Oscillospiraceae | Eubacteriales | Clostridia | Firmicutes | Bacteria |
| ASV3910 | 402 | 2 | 1 | 402 | 100 | NR_102884.1 Ruminococcus champanellensis 18P13 = JCM 17042 16S ribosomal RNA, partial sequence | NR_102884.1 | 1494 | 348 | 749 | 26.91 | 571.7 | 9E-163 | 373/404 | 92 | 4/404 | 0 | Plus | Plus | Bacteria; Firmicutes; Clostridia; Eubacteriales; Oscillospiraceae; Ruminococcus; Ruminococcus champanellensis | Ruminococcus champanellensis | Ruminococcus | Oscillospiraceae | Eubacteriales | Clostridia | Firmicutes | Bacteria |
| ASV3911 | 426 | 1 | 1 | 426 | 100 | NR_134177.1 Pleomorphochaeta multiformis strain MO-SPC2 16S ribosomal RNA, partial sequence | NR_134177.1 | 1466 | 334 | 760 | 29.13 | 505.3 | 1E-142 | 380/431 | 88 | 9/431 | 2 | Plus | Plus | Bacteria; Spirochaetes; Spirochaetia; Spirochaetales; Spirochaetaceae; Pleomorphochaeta; Pleomorphochaeta multiformis | Pleomorphochaeta multiformis | Pleomorphochaeta | Spirochaetaceae | Spirochaetales | Spirochaetia | Spirochaetes | Bacteria |
| ASV3912 | 402 | 1 | 1 | 402 | 100 | NR_042953.1 Anaerosporobacter mobilis strain IMSNU 40011 16S ribosomal RNA, partial sequence | NR_042953.1 | 1390 | 325 | 726 | 28.92 | 654.8 | 0 | 387/403 | 96 | 2/403 | 0 | Plus | Plus | Bacteria; Firmicutes; Clostridia; Eubacteriales; Lachnospiraceae; Anaerosporobacter; Anaerosporobacter mobilis | Anaerosporobacter mobilis | Anaerosporobacter | Lachnospiraceae | Eubacteriales | Clostridia | Firmicutes | Bacteria |
| ASV3914 | 427 | 1 | 2 | 427 | 99.77 | NR_118039.1 Noviherbaspirillum canariense strain SUEMI03 16S ribosomal RNA, partial sequence | NR_118039.1 | 1526 | 348 | 773 | 27.92 | 726.9 | 0 | 415/426 | 97 | 0/426 | 0 | Plus | Plus | Bacteria; Proteobacteria; Betaproteobacteria; Burkholderiales; Oxalobacteraceae; Noviherbaspirillum; Noviherbaspirillum canariense | Noviherbaspirillum canariense | Noviherbaspirillum | Oxalobacteraceae | Burkholderiales | Betaproteobacteria | Proteobacteria | Bacteria |
| ASV3915 | 404 | 1 | 1 | 404 | 100 | NR_118156.1 Oscillibacter ruminantium GH1 16S ribosomal RNA, partial sequence | NR_118156.1 | 1409 | 321 | 724 | 28.67 | 508.9 | 7E-144 | 365/408 | 89 | 8/408 | 1 | Plus | Plus | Bacteria; Firmicutes; Clostridia; Eubacteriales; Oscillospiraceae; Oscillibacter; Oscillibacter ruminantium | Oscillibacter ruminantium | Oscillibacter | Oscillospiraceae | Eubacteriales | Clostridia | Firmicutes | Bacteria |
| ASV3916 | 427 | 1 | 1 | 427 | 100 | NR_113163.1 Faecalitalea cylindroides strain JCM 10261 16S ribosomal RNA, partial sequence | NR_113163.1 | 1504 | 359 | 784 | 28.32 | 638.2 | 0 | 400/427 | 93 | 1/427 | 0 | Plus | Plus | Bacteria; Firmicutes; Erysipelotrichia; Erysipelotrichales; Erysipelotrichaceae; Faecalitalea; Faecalitalea cylindroides | Faecalitalea cylindroides | Faecalitalea | Erysipelotrichaceae | Erysipelotrichales | Erysipelotrichia | Firmicutes | Bacteria |
| ASV3917 | 402 | 2 | 3 | 402 | 99.5 | NR_024948.1 Campylobacter hyointestinalis subsp. lawsonii strain CHY5 16S ribosomal RNA, partial sequence | NR_024948.1 | 1419 | 320 | 720 | 28.26 | 601.3 | 1E-171 | 377/402 | 93 | 3/402 | 0 | Plus | Plus | Bacteria; Proteobacteria; Epsilonproteobacteria; Campylobacterales; Campylobacteraceae; Campylobacter; Campylobacter hyointestinalis | Campylobacter hyointestinalis | Campylobacter | Campylobacteraceae | Campylobacterales | Epsilonproteobacteria | Proteobacteria | Bacteria |
| ASV3919 | 422 | 1 | 1 | 422 | 100 | NR_113411.1 Prevotella copri DSM 18205 strain JCM 13464 16S ribosomal RNA, partial sequence | NR_113411.1 | 1493 | 358 | 779 | 28.27 | 686.2 | 0 | 405/422 | 95 | 0/422 | 0 | Plus | Plus | Bacteria; Bacteroidetes; Bacteroidia; Bacteroidales; Prevotellaceae; Prevotella; Prevotella copri | Prevotella copri | Prevotella | Prevotellaceae | Bacteroidales | Bacteroidia | Bacteroidetes | Bacteria |
| ASV3920 | 422 | 2 | 1 | 422 | 100 | NR_113208.1 Parabacteroides chinchillae strain JCM 17104 16S ribosomal RNA, partial sequence | NR_113208.1 | 1489 | 353 | 774 | 28.34 | 614.2 | 2E-175 | 393/423 | 92 | 2/423 | 0 | Plus | Plus | Bacteria; Bacteroidetes; Bacteroidia; Bacteroidales; Tannerellaceae; Parabacteroides; Parabacteroides chinchillae | Parabacteroides chinchillae | Parabacteroides | Tannerellaceae | Bacteroidales | Bacteroidia | Bacteroidetes | Bacteria |
| ASV3921 | 402 | 1 | 1 | 402 | 100 | NR_074634.1 [Eubacterium] rectale ATCC 33656 16S ribosomal RNA, partial sequence | NR_074634.1 | 1521 | 367 | 768 | 26.43 | 494.2 | 2E-139 | 357/402 | 88 | 0/402 | 0 | Plus | Plus | Bacteria; Firmicutes; Clostridia; Eubacteriales; Lachnospiraceae; ; [Eubacterium] rectale | [Eubacterium] rectale | | Lachnospiraceae | Eubacteriales | Clostridia | Firmicutes | Bacteria |
| ASV3922 | 402 | 2 | 1 | 402 | 100 | NR_156080.1 Kineothrix alysoides strain KNHs209 16S ribosomal RNA, partial sequence | NR_156080.1 | 1410 | 353 | 754 | 28.51 | 704.7 | 0 | 395/402 | 98 | 0/402 | 0 | Plus | Plus | Bacteria; Firmicutes; Clostridia; Eubacteriales; Lachnospiraceae; Kineothrix; Kineothrix alysoides | Kineothrix alysoides | Kineothrix | Lachnospiraceae | Eubacteriales | Clostridia | Firmicutes | Bacteria |
| ASV3923 | 422 | 1 | 1 | 422 | 100 | NR_041364.1 Prevotella stercorea DSM 18206 strain CB35 16S ribosomal RNA, partial sequence | NR_041364.1 | 1488 | 354 | 775 | 28.36 | 630.8 | 2E-180 | 395/422 | 93 | 0/422 | 0 | Plus | Plus | Bacteria; Bacteroidetes; Bacteroidia; Bacteroidales; Prevotellaceae; Prevotella; Prevotella stercorea | Prevotella stercorea | Prevotella | Prevotellaceae | Bacteroidales | Bacteroidia | Bacteroidetes | Bacteria |
| ASV3924 | 402 | 2 | 1 | 402 | 100 | NR_144696.1 Clostridium saudiense strain JCC 16S ribosomal RNA, partial sequence | NR_144696.1 | 1478 | 350 | 751 | 27.2 | 616.1 | 4E-176 | 379/402 | 94 | 0/402 | 0 | Plus | Plus | Bacteria; Firmicutes; Clostridia; Eubacteriales; Clostridiaceae; Clostridium; Clostridium saudiense | Clostridium saudiense | Clostridium | Clostridiaceae | Eubacteriales | Clostridia | Firmicutes | Bacteria |
| ASV3925 | 401 | 1 | 1 | 401 | 100 | NR_144749.1 Ihubacter massiliensis strain Marseille-P2843 16S ribosomal RNA, partial sequence | NR_144749.1 | 1479 | 364 | 766 | 27.25 | 649.3 | 0 | 386/403 | 95 | 2/403 | 0 | Plus | Plus | Bacteria; Firmicutes; Clostridia; Eubacteriales; Clostridiales Family XIII. Incertae Sedis; Ihubacter; Ihubacter massiliensis | Ihubacter massiliensis | Ihubacter | Clostridiales Family XIII. Incertae Sedis | Eubacteriales | Clostridia | Firmicutes | Bacteria |
| ASV3926 | 402 | 1 | 1 | 402 | 100 | NR_118730.1 [Clostridium] symbiosum strain ATCC 14940 16S ribosomal RNA, partial sequence | NR_118730.1 | 1478 | 372 | 773 | 27.2 | 510.8 | 2E-144 | 358/402 | 89 | 0/402 | 0 | Plus | Plus | Bacteria; Firmicutes; Clostridia; Eubacteriales; Lachnospiraceae; Lachnoclostridium; [Clostridium] symbiosum | [Clostridium] symbiosum | Lachnoclostridium | Lachnospiraceae | Eubacteriales | Clostridia | Firmicutes | Bacteria |
| ASV3927 | 405 | 1 | 1 | 405 | 100 | NR_026476.1 Succinivibrio dextrinosolvens strain 0554 16S ribosomal RNA, partial sequence | NR_026476.1 | 1463 | 331 | 732 | 27.48 | 501.6 | 1E-141 | 361/405 | 89 | 3/405 | 0 | Plus | Plus | Bacteria; Proteobacteria; Gammaproteobacteria; Aeromonadales; Succinivibrionaceae; Succinivibrio; Succinivibrio dextrinosolvens | Succinivibrio dextrinosolvens | Succinivibrio | Succinivibrionaceae | Aeromonadales | Gammaproteobacteria | Proteobacteria | Bacteria |
| ASV3928 | 403 | 1 | 1 | 403 | 100 | NR_117912.1 Defluviitalea saccharophila strain LIND6LT2 16S ribosomal RNA, partial sequence | NR_117912.1 | 1495 | 342 | 746 | 27.09 | 581 | 2E-165 | 376/406 | 92 | 4/406 | 0 | Plus | Plus | Bacteria; Firmicutes; Clostridia; Eubacteriales; Defluviitaleaceae; Defluviitalea; Defluviitalea saccharophila | Defluviitalea saccharophila | Defluviitalea | Defluviitaleaceae | Eubacteriales | Clostridia | Firmicutes | Bacteria |
| ASV3930 | 405 | 1 | 3 | 405 | 99.51 | NR_112900.1 Christensenella minuta strain YIT 12065 16S ribosomal RNA, partial sequence | NR_112900.1 | 1497 | 360 | 762 | 26.92 | 579.1 | 6E-165 | 374/404 | 92 | 2/404 | 0 | Plus | Plus | Bacteria; Firmicutes; Clostridia; Eubacteriales; Christensenellaceae; Christensenella; Christensenella minuta | Christensenella minuta | Christensenella | Christensenellaceae | Eubacteriales | Clostridia | Firmicutes | Bacteria |
| ASV3933 | 422 | 1 | 1 | 422 | 100 | NR_144750.1 Prevotellamassilia timonensis strain Marseille-P2831 16S ribosomal RNA, partial sequence | NR_144750.1 | 1493 | 358 | 779 | 28.27 | 664.1 | 0 | 402/423 | 95 | 2/423 | 0 | Plus | Plus | Bacteria; Bacteroidetes; Bacteroidia; Bacteroidales; Prevotellaceae; Prevotellamassilia; Prevotellamassilia timonensis | Prevotellamassilia timonensis | Prevotellamassilia | Prevotellaceae | Bacteroidales | Bacteroidia | Bacteroidetes | Bacteria |
| ASV3935 | 427 | 1 | 1 | 427 | 100 | NR_147401.1 Sutterella massiliensis strain Marseille-P2435 16S ribosomal RNA, partial sequence | NR_147401.1 | 1498 | 349 | 775 | 28.5 | 758.2 | 0 | 421/427 | 98 | 0/427 | 0 | Plus | Plus | Bacteria; Proteobacteria; Betaproteobacteria; Burkholderiales; Sutterellaceae; Sutterella; Sutterella massiliensis | Sutterella massiliensis | Sutterella | Sutterellaceae | Burkholderiales | Betaproteobacteria | Proteobacteria | Bacteria |
| ASV3936 | 405 | 1 | 1 | 405 | 100 | NR_134005.1 Clostridium bornimense strain M2/40 16S ribosomal RNA, partial sequence | NR_134005.1 | 1363 | 284 | 685 | 29.49 | 579.1 | 6E-165 | 379/409 | 92 | 11/409 | 2 | Plus | Plus | Bacteria; Firmicutes; Clostridia; Eubacteriales; Clostridiaceae; Clostridium; Clostridium bornimense | Clostridium bornimense | Clostridium | Clostridiaceae | Eubacteriales | Clostridia | Firmicutes | Bacteria |
| ASV3937 | 422 | 1 | 1 | 422 | 100 | NR_113411.1 Prevotella copri DSM 18205 strain JCM 13464 16S ribosomal RNA, partial sequence | NR_113411.1 | 1493 | 358 | 779 | 28.27 | 697.3 | 0 | 407/422 | 96 | 0/422 | 0 | Plus | Plus | Bacteria; Bacteroidetes; Bacteroidia; Bacteroidales; Prevotellaceae; Prevotella; Prevotella copri | Prevotella copri | Prevotella | Prevotellaceae | Bacteroidales | Bacteroidia | Bacteroidetes | Bacteria |
| ASV3938 | 427 | 1 | 1 | 427 | 100 | NR_102980.1 Megasphaera elsdenii DSM 20460 16S ribosomal RNA, partial sequence | NR_102980.1 | 1552 | 369 | 795 | 27.51 | 629 | 6E-180 | 398/427 | 93 | 0/427 | 0 | Plus | Plus | Bacteria; Firmicutes; Negativicutes; Veillonellales; Veillonellaceae; Megasphaera; Megasphaera elsdenii | Megasphaera elsdenii | Megasphaera | Veillonellaceae | Veillonellales | Negativicutes | Firmicutes | Bacteria |
| ASV3940 | 405 | 1 | 1 | 405 | 100 | NR_044972.1 Sporobacter termitidis strain SYR 16S ribosomal RNA, partial sequence | NR_044972.1 | 1506 | 348 | 752 | 26.89 | 608.7 | 7E-174 | 380/406 | 93 | 2/406 | 0 | Plus | Plus | Bacteria; Firmicutes; Clostridia; Eubacteriales; Oscillospiraceae; Sporobacter; Sporobacter termitidis | Sporobacter termitidis | Sporobacter | Oscillospiraceae | Eubacteriales | Clostridia | Firmicutes | Bacteria |
| ASV3942 | 405 | 1 | 3 | 405 | 99.51 | NR_112900.1 Christensenella minuta strain YIT 12065 16S ribosomal RNA, partial sequence | NR_112900.1 | 1497 | 360 | 762 | 26.92 | 479.4 | 6E-135 | 361/409 | 88 | 12/409 | 2 | Plus | Plus | Bacteria; Firmicutes; Clostridia; Eubacteriales; Christensenellaceae; Christensenella; Christensenella minuta | Christensenella minuta | Christensenella | Christensenellaceae | Eubacteriales | Clostridia | Firmicutes | Bacteria |
| ASV3944 | 401 | 1 | 1 | 400 | 99.75 | NR_159227.1 Monoglobus pectinilyticus strain 14 16S ribosomal RNA, partial sequence | NR_159227.1 | 1493 | 368 | 768 | 26.86 | 512.6 | 6E-145 | 361/402 | 89 | 3/402 | 0 | Plus | Plus | Bacteria; Firmicutes; Clostridia; Eubacteriales; Oscillospiraceae; Monoglobus; Monoglobus pectinilyticus | Monoglobus pectinilyticus | Monoglobus | Oscillospiraceae | Eubacteriales | Clostridia | Firmicutes | Bacteria |
| ASV3945 | 404 | 4 | 3 | 404 | 99.5 | NR_042802.1 Lactobacillus ultunensis strain Kx146C1 16S ribosomal RNA, partial sequence | NR_042802.1 | 1550 | 372 | 796 | 27.42 | 542.2 | 7E-154 | 385/425 | 90 | 23/425 | 5 | Plus | Plus | Bacteria; Firmicutes; Bacilli; Lactobacillales; Lactobacillaceae; Lactobacillus; Lactobacillus ultunensis | Lactobacillus ultunensis | Lactobacillus | Lactobacillaceae | Lactobacillales | Bacilli | Firmicutes | Bacteria |
| ASV3946 | 422 | 1 | 1 | 422 | 100 | NR_144616.1 Muribaculum intestinale strain YL27 16S ribosomal RNA, partial sequence | NR_144616.1 | 1460 | 297 | 718 | 28.9 | 547.7 | 2E-155 | 383/425 | 90 | 6/425 | 1 | Plus | Plus | Bacteria; Bacteroidetes; Bacteroidia; Bacteroidales; Muribaculaceae; Muribaculum; Muribaculum intestinale | Muribaculum intestinale | Muribaculum | Muribaculaceae | Bacteroidales | Bacteroidia | Bacteroidetes | Bacteria |
| ASV3950 | 422 | 1 | 1 | 413 | 97.87 | NR_113411.1 Prevotella copri DSM 18205 strain JCM 13464 16S ribosomal RNA, partial sequence | NR_113411.1 | 1493 | 358 | 770 | 27.66 | 625.3 | 7E-179 | 388/413 | 93 | 0/413 | 0 | Plus | Plus | Bacteria; Bacteroidetes; Bacteroidia; Bacteroidales; Prevotellaceae; Prevotella; Prevotella copri | Prevotella copri | Prevotella | Prevotellaceae | Bacteroidales | Bacteroidia | Bacteroidetes | Bacteria |
| ASV3952 | 427 | 1 | 1 | 427 | 100 | NR_114696.1 Anaerovibrio lipolyticus DSM 3074 16S ribosomal RNA, partial sequence | NR_114696.1 | 1526 | 372 | 797 | 27.92 | 529.3 | 6E-150 | 385/432 | 89 | 11/432 | 2 | Plus | Plus | Bacteria; Firmicutes; Negativicutes; Selenomonadales; Selenomonadaceae; Anaerovibrio; Anaerovibrio lipolyticus | Anaerovibrio lipolyticus | Anaerovibrio | Selenomonadaceae | Selenomonadales | Negativicutes | Firmicutes | Bacteria |
| ASV3953 | 426 | 2 | 1 | 426 | 100 | NR_116012.1 Streptococcus equi subsp. ruminatorum CECT 5772 strain CCUG 47520 16S ribosomal RNA, partial sequence | NR_116012.1 | 1470 | 337 | 763 | 29.05 | 616.1 | 4E-176 | 400/431 | 92 | 9/431 | 2 | Plus | Plus | Bacteria; Firmicutes; Bacilli; Lactobacillales; Streptococcaceae; Streptococcus; Streptococcus equi | Streptococcus equi | Streptococcus | Streptococcaceae | Lactobacillales | Bacilli | Firmicutes | Bacteria |
| ASV3954 | 422 | 2 | 1 | 405 | 95.97 | NR_113105.1 Prevotella histicola strain JCM 15637 16S ribosomal RNA, partial sequence | NR_113105.1 | 1480 | 346 | 750 | 27.36 | 460.9 | 2E-129 | 353/405 | 87 | 0/405 | 0 | Plus | Plus | Bacteria; Bacteroidetes; Bacteroidia; Bacteroidales; Prevotellaceae; Prevotella; Prevotella histicola | Prevotella histicola | Prevotella | Prevotellaceae | Bacteroidales | Bacteroidia | Bacteroidetes | Bacteria |
| ASV3955 | 404 | 1 | 1 | 404 | 100 | NR_144611.1 Flintibacter butyricus strain BLS21 16S ribosomal RNA, partial sequence | NR_144611.1 | 808 | 299 | 703 | 50.12 | 508.9 | 7E-144 | 364/407 | 89 | 5/407 | 1 | Plus | Plus | Bacteria; Firmicutes; Clostridia; Eubacteriales; ; Flintibacter; Flintibacter butyricus | Flintibacter butyricus | Flintibacter | | Eubacteriales | Clostridia | Firmicutes | Bacteria |
| ASV3956 | 422 | 1 | 1 | 407 | 96.45 | NR_113411.1 Prevotella copri DSM 18205 strain JCM 13464 16S ribosomal RNA, partial sequence | NR_113411.1 | 1493 | 358 | 764 | 27.26 | 653 | 0 | 389/407 | 95 | 0/407 | 0 | Plus | Plus | Bacteria; Bacteroidetes; Bacteroidia; Bacteroidales; Prevotellaceae; Prevotella; Prevotella copri | Prevotella copri | Prevotella | Prevotellaceae | Bacteroidales | Bacteroidia | Bacteroidetes | Bacteria |
| ASV3957 | 422 | 1 | 1 | 407 | 96.45 | NR_113411.1 Prevotella copri DSM 18205 strain JCM 13464 16S ribosomal RNA, partial sequence | NR_113411.1 | 1493 | 358 | 764 | 27.26 | 641.9 | 0 | 387/407 | 95 | 0/407 | 0 | Plus | Plus | Bacteria; Bacteroidetes; Bacteroidia; Bacteroidales; Prevotellaceae; Prevotella; Prevotella copri | Prevotella copri | Prevotella | Prevotellaceae | Bacteroidales | Bacteroidia | Bacteroidetes | Bacteria |
| ASV3958 | 427 | 1 | 1 | 426 | 99.77 | NR_044111.1 Selenomonas bovis strain WG 16S ribosomal RNA, partial sequence | NR_044111.1 | 1525 | 370 | 795 | 27.93 | 710.2 | 0 | 412/426 | 96 | 0/426 | 0 | Plus | Plus | Bacteria; Firmicutes; Negativicutes; Selenomonadales; Selenomonadaceae; Selenomonas; Selenomonas bovis | Selenomonas bovis | Selenomonas | Selenomonadaceae | Selenomonadales | Negativicutes | Firmicutes | Bacteria |
| ASV3961 | 404 | 1 | 1 | 404 | 100 | NR_028911.1 Anaerovorax odorimutans strain NorPut 16S ribosomal RNA, partial sequence | NR_028911.1 | 1488 | 352 | 755 | 27.15 | 514.5 | 2E-145 | 365/407 | 89 | 6/407 | 1 | Plus | Plus | Bacteria; Firmicutes; Clostridia; Eubacteriales; Clostridiales Family XIII. Incertae Sedis; Anaerovorax; Anaerovorax odorimutans | Anaerovorax odorimutans | Anaerovorax | Clostridiales Family XIII. Incertae Sedis | Eubacteriales | Clostridia | Firmicutes | Bacteria |
| ASV3962 | 427 | 1 | 1 | 427 | 100 | NR_074742.1 Treponema brennaborense strain DD5/3 16S ribosomal RNA, partial sequence | NR_074742.1 | 1530 | 351 | 776 | 27.84 | 521.9 | 1E-147 | 381/429 | 88 | 5/429 | 1 | Plus | Plus | Bacteria; Spirochaetes; Spirochaetia; Spirochaetales; Spirochaetaceae; Treponema; Treponema brennaborense | Treponema brennaborense | Treponema | Spirochaetaceae | Spirochaetales | Spirochaetia | Spirochaetes | Bacteria |
| ASV3963 | 428 | 1 | 1 | 427 | 99.77 | NR_121797.1 Desulfitobacterium hafniense DCB-2 16S ribosomal RNA, partial sequence | NR_121797.1 | 1671 | 485 | 911 | 25.55 | 501.6 | 1E-141 | 378/430 | 87 | 6/430 | 1 | Plus | Plus | Bacteria; Firmicutes; Clostridia; Eubacteriales; Peptococcaceae; Desulfitobacterium; Desulfitobacterium hafniense | Desulfitobacterium hafniense | Desulfitobacterium | Peptococcaceae | Eubacteriales | Clostridia | Firmicutes | Bacteria |
| ASV3964 | 427 | 1 | 15 | 427 | 96.72 | NR_104750.1 Spiroplasma clarkii strain CN-5 16S ribosomal RNA, partial sequence | NR_104750.1 | 1507 | 351 | 762 | 27.34 | 440.6 | 3E-123 | 356/414 | 85 | 3/414 | 0 | Plus | Plus | Bacteria; Tenericutes; Mollicutes; Entomoplasmatales; Spiroplasmataceae; Spiroplasma; Spiroplasma clarkii | Spiroplasma clarkii | Spiroplasma | Spiroplasmataceae | Entomoplasmatales | Mollicutes | Tenericutes | Bacteria |
| ASV3968 | 402 | 1 | 1 | 400 | 99.5 | NR_041882.1 Robinsoniella peoriensis strain PPC31 16S ribosomal RNA, partial sequence | NR_041882.1 | 1525 | 366 | 765 | 26.23 | 484.9 | 1E-136 | 354/400 | 88 | 0/400 | 0 | Plus | Plus | Bacteria; Firmicutes; Clostridia; Eubacteriales; Lachnospiraceae; Robinsoniella; Robinsoniella peoriensis | Robinsoniella peoriensis | Robinsoniella | Lachnospiraceae | Eubacteriales | Clostridia | Firmicutes | Bacteria |
| ASV3970 | 421 | 1 | 1 | 421 | 100 | NR_144616.1 Muribaculum intestinale strain YL27 16S ribosomal RNA, partial sequence | NR_144616.1 | 1460 | 297 | 718 | 28.9 | 473.9 | 3E-133 | 367/422 | 86 | 1/422 | 0 | Plus | Plus | Bacteria; Bacteroidetes; Bacteroidia; Bacteroidales; Muribaculaceae; Muribaculum; Muribaculum intestinale | Muribaculum intestinale | Muribaculum | Muribaculaceae | Bacteroidales | Bacteroidia | Bacteroidetes | Bacteria |
| ASV3972 | 405 | 4 | 3 | 405 | 99.51 | NR_042802.1 Lactobacillus ultunensis strain Kx146C1 16S ribosomal RNA, partial sequence | NR_042802.1 | 1550 | 372 | 796 | 27.42 | 533 | 4E-151 | 383/425 | 90 | 22/425 | 5 | Plus | Plus | Bacteria; Firmicutes; Bacilli; Lactobacillales; Lactobacillaceae; Lactobacillus; Lactobacillus ultunensis | Lactobacillus ultunensis | Lactobacillus | Lactobacillaceae | Lactobacillales | Bacilli | Firmicutes | Bacteria |
| ASV3974 | 405 | 1 | 1 | 405 | 100 | NR_144611.1 Flintibacter butyricus strain BLS21 16S ribosomal RNA, partial sequence | NR_144611.1 | 808 | 299 | 703 | 50.12 | 566.2 | 4E-161 | 372/405 | 91 | 0/405 | 0 | Plus | Plus | Bacteria; Firmicutes; Clostridia; Eubacteriales; ; Flintibacter; Flintibacter butyricus | Flintibacter butyricus | Flintibacter | | Eubacteriales | Clostridia | Firmicutes | Bacteria |
| ASV3975 | 405 | 1 | 1 | 405 | 100 | NR_104907.1 Eubacterium coprostanoligenes strain HL 16S ribosomal RNA, partial sequence | NR_104907.1 | 1446 | 332 | 734 | 27.87 | 464.6 | 2E-130 | 356/407 | 87 | 6/407 | 1 | Plus | Plus | Bacteria; Firmicutes; Clostridia; Eubacteriales; Eubacteriaceae; Eubacterium; Eubacterium coprostanoligenes | Eubacterium coprostanoligenes | Eubacterium | Eubacteriaceae | Eubacteriales | Clostridia | Firmicutes | Bacteria |
| ASV3977 | 405 | 1 | 1 | 405 | 100 | NR_145929.1 Caproiciproducens galactitolivorans strain BS-1 16S ribosomal RNA, partial sequence | NR_145929.1 | 1445 | 336 | 741 | 28.1 | 577.3 | 2E-164 | 375/406 | 92 | 1/406 | 0 | Plus | Plus | Bacteria; Firmicutes; Clostridia; Eubacteriales; Oscillospiraceae; Caproiciproducens; Caproiciproducens galactitolivorans | Caproiciproducens galactitolivorans | Caproiciproducens | Oscillospiraceae | Eubacteriales | Clostridia | Firmicutes | Bacteria |
| ASV3978 | 423 | 1 | 1 | 423 | 100 | NR_147368.1 Millionella massiliensis strain Marseille-P3215 16S ribosomal RNA, partial sequence | NR_147368.1 | 1492 | 355 | 776 | 28.28 | 431.4 | 2E-120 | 366/429 | 85 | 13/429 | 3 | Plus | Plus | Bacteria; Bacteroidetes; Bacteroidia; Bacteroidales; Rikenellaceae; Millionella; Millionella massiliensis | Millionella massiliensis | Millionella | Rikenellaceae | Bacteroidales | Bacteroidia | Bacteroidetes | Bacteria |
| ASV3979 | 402 | 1 | 1 | 402 | 100 | NR_042200.1 [Clostridium] asparagiforme strain N6 16S ribosomal RNA, partial sequence | NR_042200.1 | 1468 | 335 | 736 | 27.38 | 654.8 | 0 | 386/402 | 96 | 0/402 | 0 | Plus | Plus | Bacteria; Firmicutes; Clostridia; Eubacteriales; Lachnospiraceae; Enterocloster; Enterocloster asparagiformis | Enterocloster asparagiformis | Enterocloster | Lachnospiraceae | Eubacteriales | Clostridia | Firmicutes | Bacteria |
| ASV3981 | 404 | 5 | 1 | 404 | 100 | NR_113360.1 Ruminiclostridium cellobioparum DSM 1351 = ATCC 15832 strain JCM 1422 16S ribosomal RNA, partial sequence | NR_113360.1 | 1484 | 356 | 760 | 27.29 | 547.7 | 2E-155 | 369/405 | 91 | 1/405 | 0 | Plus | Plus | Bacteria; Firmicutes; Clostridia; Eubacteriales; Oscillospiraceae; Ruminiclostridium; Ruminiclostridium cellobioparum | Ruminiclostridium cellobioparum | Ruminiclostridium | Oscillospiraceae | Eubacteriales | Clostridia | Firmicutes | Bacteria |
| ASV3982 | 402 | 4 | 3 | 402 | 99.5 | NR_118511.1 Campylobacter coli strain ATCC 33559 16S ribosomal RNA, partial sequence | NR_118511.1 | 1435 | 331 | 731 | 27.94 | 595.7 | 5E-170 | 378/404 | 93 | 7/404 | 1 | Plus | Plus | Bacteria; Proteobacteria; Epsilonproteobacteria; Campylobacterales; Campylobacteraceae; Campylobacter; Campylobacter coli | Campylobacter coli | Campylobacter | Campylobacteraceae | Campylobacterales | Epsilonproteobacteria | Proteobacteria | Bacteria |
| ASV3984 | 428 | 1 | 1 | 427 | 99.77 | NR_156094.1 Desulfitispora elongata strain Acr1 16S ribosomal RNA, partial sequence | NR_156094.1 | 1491 | 392 | 818 | 28.64 | 475.7 | 8E-134 | 373/430 | 86 | 6/430 | 1 | Plus | Plus | Bacteria; Firmicutes; Clostridia; Eubacteriales; Peptococcaceae; Desulfitispora; Desulfitispora elongata | Desulfitispora elongata | Desulfitispora | Peptococcaceae | Eubacteriales | Clostridia | Firmicutes | Bacteria |
| ASV3985 | 405 | 1 | 1 | 405 | 100 | NR_115269.1 Catabacter hongkongensis strain HKU16 16S ribosomal RNA, partial sequence | NR_115269.1 | 1453 | 349 | 753 | 27.87 | 444.3 | 2E-124 | 350/405 | 86 | 0/405 | 0 | Plus | Plus | Bacteria; Firmicutes; Clostridia; Eubacteriales; Christensenellaceae; Christensenella; Christensenella hongkongensis | Christensenella hongkongensis | Christensenella | Christensenellaceae | Eubacteriales | Clostridia | Firmicutes | Bacteria |
| ASV3988 | 408 | 4 | 1 | 408 | 100 | NR_074414.1 Olsenella uli DSM 7084 16S ribosomal RNA, partial sequence | NR_074414.1 | 1503 | 348 | 755 | 27.15 | 582.8 | 4E-166 | 377/408 | 92 | 0/408 | 0 | Plus | Plus | Bacteria; Actinobacteria; Coriobacteriia; Coriobacteriales; Atopobiaceae; Olsenella; Olsenella uli | Olsenella uli | Olsenella | Atopobiaceae | Coriobacteriales | Coriobacteriia | Actinobacteria | Bacteria |
| ASV3989 | 403 | 1 | 1 | 403 | 100 | NR_117912.1 Defluviitalea saccharophila strain LIND6LT2 16S ribosomal RNA, partial sequence | NR_117912.1 | 1495 | 342 | 746 | 27.09 | 564.3 | 2E-160 | 373/406 | 91 | 4/406 | 0 | Plus | Plus | Bacteria; Firmicutes; Clostridia; Eubacteriales; Defluviitaleaceae; Defluviitalea; Defluviitalea saccharophila | Defluviitalea saccharophila | Defluviitalea | Defluviitaleaceae | Eubacteriales | Clostridia | Firmicutes | Bacteria |
| ASV3990 | 427 | 1 | 1 | 427 | 100 | NR_044111.1 Selenomonas bovis strain WG 16S ribosomal RNA, partial sequence | NR_044111.1 | 1525 | 370 | 796 | 28 | 761.9 | 0 | 422/427 | 98 | 0/427 | 0 | Plus | Plus | Bacteria; Firmicutes; Negativicutes; Selenomonadales; Selenomonadaceae; Selenomonas; Selenomonas bovis | Selenomonas bovis | Selenomonas | Selenomonadaceae | Selenomonadales | Negativicutes | Firmicutes | Bacteria |
| ASV3991 | 404 | 1 | 1 | 404 | 100 | NR_118156.1 Oscillibacter ruminantium GH1 16S ribosomal RNA, partial sequence | NR_118156.1 | 1409 | 321 | 724 | 28.67 | 614.2 | 2E-175 | 381/405 | 94 | 2/405 | 0 | Plus | Plus | Bacteria; Firmicutes; Clostridia; Eubacteriales; Oscillospiraceae; Oscillibacter; Oscillibacter ruminantium | Oscillibacter ruminantium | Oscillibacter | Oscillospiraceae | Eubacteriales | Clostridia | Firmicutes | Bacteria |
| ASV3994 | 422 | 1 | 1 | 422 | 100 | NR_113411.1 Prevotella copri DSM 18205 strain JCM 13464 16S ribosomal RNA, partial sequence | NR_113411.1 | 1493 | 358 | 779 | 28.27 | 542.2 | 8E-154 | 383/426 | 89 | 8/426 | 1 | Plus | Plus | Bacteria; Bacteroidetes; Bacteroidia; Bacteroidales; Prevotellaceae; Prevotella; Prevotella copri | Prevotella copri | Prevotella | Prevotellaceae | Bacteroidales | Bacteroidia | Bacteroidetes | Bacteria |
| ASV3995 | 422 | 2 | 1 | 422 | 100 | NR_113119.1 Prevotella oris strain JCM 8540 16S ribosomal RNA, partial sequence | NR_113119.1 | 1481 | 345 | 766 | 28.49 | 603.1 | 3E-172 | 392/424 | 92 | 4/424 | 0 | Plus | Plus | Bacteria; Bacteroidetes; Bacteroidia; Bacteroidales; Prevotellaceae; Prevotella; Prevotella oris | Prevotella oris | Prevotella | Prevotellaceae | Bacteroidales | Bacteroidia | Bacteroidetes | Bacteria |
| ASV3996 | 427 | 4 | 3 | 427 | 99.53 | NR_042802.1 Lactobacillus ultunensis strain Kx146C1 16S ribosomal RNA, partial sequence | NR_042802.1 | 1550 | 372 | 796 | 27.42 | 608.7 | 7E-174 | 394/426 | 92 | 2/426 | 0 | Plus | Plus | Bacteria; Firmicutes; Bacilli; Lactobacillales; Lactobacillaceae; Lactobacillus; Lactobacillus ultunensis | Lactobacillus ultunensis | Lactobacillus | Lactobacillaceae | Lactobacillales | Bacilli | Firmicutes | Bacteria |
| ASV3997 | 401 | 1 | 1 | 401 | 100 | NR_158111.1 Paludicola psychrotolerans strain NC1253 16S ribosomal RNA, partial sequence | NR_158111.1 | 1490 | 366 | 766 | 26.91 | 619.7 | 3E-177 | 380/402 | 94 | 2/402 | 0 | Plus | Plus | Bacteria; Firmicutes; Clostridia; Eubacteriales; Oscillospiraceae; Paludicola; Paludicola psychrotolerans | Paludicola psychrotolerans | Paludicola | Oscillospiraceae | Eubacteriales | Clostridia | Firmicutes | Bacteria |
| ASV4003 | 401 | 1 | 1 | 401 | 100 | NR_169458.1 Tepidibaculum saccharolyticum strain STR9 16S ribosomal RNA, partial sequence | NR_169458.1 | 1477 | 362 | 762 | 27.22 | 520 | 3E-147 | 364/404 | 90 | 5/404 | 1 | Plus | Plus | Bacteria; Firmicutes; Clostridia; Eubacteriales; Oscillospiraceae; Tepidibaculum; Tepidibaculum saccharolyticum | Tepidibaculum saccharolyticum | Tepidibaculum | Oscillospiraceae | Eubacteriales | Clostridia | Firmicutes | Bacteria |
| ASV4005 | 401 | 1 | 1 | 401 | 100 | NR_028911.1 Anaerovorax odorimutans strain NorPut 16S ribosomal RNA, partial sequence | NR_028911.1 | 1488 | 352 | 755 | 27.15 | 621.6 | 9E-178 | 383/405 | 94 | 5/405 | 1 | Plus | Plus | Bacteria; Firmicutes; Clostridia; Eubacteriales; Clostridiales Family XIII. Incertae Sedis; Anaerovorax; Anaerovorax odorimutans | Anaerovorax odorimutans | Anaerovorax | Clostridiales Family XIII. Incertae Sedis | Eubacteriales | Clostridia | Firmicutes | Bacteria |
| ASV4006 | 427 | 1 | 1 | 427 | 100 | NR_028862.1 Allisonella histaminiformans strain MR2 16S ribosomal RNA, partial sequence | NR_028862.1 | 1537 | 376 | 802 | 27.78 | 734.2 | 0 | 417/427 | 97 | 0/427 | 0 | Plus | Plus | Bacteria; Firmicutes; Negativicutes; Veillonellales; Veillonellaceae; Allisonella; Allisonella histaminiformans | Allisonella histaminiformans | Allisonella | Veillonellaceae | Veillonellales | Negativicutes | Firmicutes | Bacteria |
| ASV4007 | 405 | 3 | 3 | 405 | 99.51 | NR_148837.1 Abyssivirga alkaniphila strain L81 16S ribosomal RNA, partial sequence | NR_148837.1 | 1520 | 366 | 765 | 26.32 | 464.6 | 2E-130 | 353/403 | 87 | 3/403 | 0 | Plus | Plus | Bacteria; Firmicutes; Clostridia; Eubacteriales; Lachnospiraceae; Abyssivirga; Abyssivirga alkaniphila | Abyssivirga alkaniphila | Abyssivirga | Lachnospiraceae | Eubacteriales | Clostridia | Firmicutes | Bacteria |
| ASV4008 | 402 | 1 | 1 | 402 | 100 | NR_158111.1 Paludicola psychrotolerans strain NC1253 16S ribosomal RNA, partial sequence | NR_158111.1 | 1490 | 366 | 766 | 26.91 | 547.7 | 2E-155 | 371/406 | 91 | 9/406 | 2 | Plus | Plus | Bacteria; Firmicutes; Clostridia; Eubacteriales; Oscillospiraceae; Paludicola; Paludicola psychrotolerans | Paludicola psychrotolerans | Paludicola | Oscillospiraceae | Eubacteriales | Clostridia | Firmicutes | Bacteria |
| ASV4009 | 422 | 1 | 1 | 422 | 100 | NR_136443.1 Porphyromonas pogonae strain MI 10-1288 16S ribosomal RNA, partial sequence | NR_136443.1 | 1429 | 317 | 739 | 29.6 | 475.7 | 8E-134 | 368/423 | 86 | 1/423 | 0 | Plus | Plus | Bacteria; Bacteroidetes; Bacteroidia; Bacteroidales; Porphyromonadaceae; Porphyromonas; Porphyromonas pogonae | Porphyromonas pogonae | Porphyromonas | Porphyromonadaceae | Bacteroidales | Bacteroidia | Bacteroidetes | Bacteria |
| ASV4010 | 422 | 1 | 1 | 422 | 100 | NR_149795.1 Lentimicrobium saccharophilum strain TBC1 16S ribosomal RNA, partial sequence | NR_149795.1 | 1446 | 330 | 751 | 29.18 | 486.8 | 4E-137 | 369/422 | 87 | 0/422 | 0 | Plus | Plus | Bacteria; Bacteroidetes; Bacteroidia; Bacteroidales; Lentimicrobiaceae; Lentimicrobium; Lentimicrobium saccharophilum | Lentimicrobium saccharophilum | Lentimicrobium | Lentimicrobiaceae | Bacteroidales | Bacteroidia | Bacteroidetes | Bacteria |
| ASV4011 | 428 | 1 | 1 | 428 | 100 | NR_027596.1 Mitsuokella multacida strain DSM 20544 16S ribosomal RNA, partial sequence | NR_027596.1 | 1511 | 346 | 772 | 28.26 | 542.2 | 8E-154 | 388/433 | 89 | 11/433 | 2 | Plus | Plus | Bacteria; Firmicutes; Negativicutes; Selenomonadales; Selenomonadaceae; Mitsuokella; Mitsuokella multacida | Mitsuokella multacida | Mitsuokella | Selenomonadaceae | Selenomonadales | Negativicutes | Firmicutes | Bacteria |
| ASV4012 | 422 | 1 | 1 | 422 | 100 | NR_121773.2 Barnesiella viscericola strain C46 16S ribosomal RNA, complete sequence | NR_121773.2 | 1532 | 365 | 786 | 27.55 | 448.6 | 8E-124 | 366/427 | 85 | 10/427 | 2 | Plus | Plus | Bacteria; Bacteroidetes; Bacteroidia; Bacteroidales; Barnesiellaceae; Barnesiella; Barnesiella viscericola | Barnesiella viscericola | Barnesiella | Barnesiellaceae | Bacteroidales | Bacteroidia | Bacteroidetes | Bacteria |
| ASV4013 | 402 | 1 | 1 | 402 | 100 | NR_148574.1 Raoultibacter timonensis strain Marseille-P3277 16S ribosomal RNA, partial sequence | NR_148574.1 | 1472 | 348 | 748 | 27.24 | 625.3 | 7E-179 | 381/402 | 94 | 1/402 | 0 | Plus | Plus | Bacteria; Actinobacteria; Coriobacteriia; Eggerthellales; Eggerthellaceae; Raoultibacter; Raoultibacter timonensis | Raoultibacter timonensis | Raoultibacter | Eggerthellaceae | Eggerthellales | Coriobacteriia | Actinobacteria | Bacteria |
| ASV4014 | 402 | 1 | 1 | 402 | 100 | NR_147379.1 Collinsella bouchesdurhonensis strain Marseille-P3296 16S ribosomal RNA, partial sequence | NR_147379.1 | 1471 | 345 | 746 | 27.33 | 743.5 | 0 | 402/402 | 100 | 0/402 | 0 | Plus | Plus | Bacteria; Actinobacteria; Coriobacteriia; Coriobacteriales; Coriobacteriaceae; Collinsella; Collinsella bouchesdurhonensis | Collinsella bouchesdurhonensis | Collinsella | Coriobacteriaceae | Coriobacteriales | Coriobacteriia | Actinobacteria | Bacteria |
| ASV4015 | 404 | 4 | 1 | 404 | 100 | NR_074399.1 Ruminococcus albus 7 = DSM 20455 16S ribosomal RNA, partial sequence | NR_074399.1 | 1500 | 350 | 751 | 26.8 | 612.4 | 5E-175 | 380/404 | 94 | 2/404 | 0 | Plus | Plus | Bacteria; Firmicutes; Clostridia; Eubacteriales; Oscillospiraceae; Ruminococcus; Ruminococcus albus | Ruminococcus albus | Ruminococcus | Oscillospiraceae | Eubacteriales | Clostridia | Firmicutes | Bacteria |
| ASV4017 | 405 | 2 | 14 | 405 | 96.79 | NR_074629.1 Hungateiclostridium thermocellum strain ATCC 27405 16S ribosomal RNA, partial sequence | NR_074629.1 | 1521 | 371 | 762 | 25.77 | 503.4 | 3E-142 | 352/392 | 89 | 0/392 | 0 | Plus | Plus | Bacteria; Firmicutes; Clostridia; Eubacteriales; Oscillospiraceae; Acetivibrio; Acetivibrio thermocellus | Acetivibrio thermocellus | Acetivibrio | Oscillospiraceae | Eubacteriales | Clostridia | Firmicutes | Bacteria |
| ASV4018 | 408 | 3 | 1 | 408 | 100 | NR_116939.1 Olsenella profusa DSM 13989 strain CCUG 45371 16S ribosomal RNA, partial sequence | NR_116939.1 | 1428 | 328 | 735 | 28.57 | 654.8 | 0 | 390/408 | 95 | 0/408 | 0 | Plus | Plus | Bacteria; Actinobacteria; Coriobacteriia; Coriobacteriales; Atopobiaceae; Olsenella; Olsenella profusa | Olsenella profusa | Olsenella | Atopobiaceae | Coriobacteriales | Coriobacteriia | Actinobacteria | Bacteria |
| ASV4019 | 421 | 1 | 1 | 421 | 100 | NR_144616.1 Muribaculum intestinale strain YL27 16S ribosomal RNA, partial sequence | NR_144616.1 | 1460 | 297 | 718 | 28.9 | 496 | 6E-140 | 372/423 | 87 | 3/423 | 0 | Plus | Plus | Bacteria; Bacteroidetes; Bacteroidia; Bacteroidales; Muribaculaceae; Muribaculum; Muribaculum intestinale | Muribaculum intestinale | Muribaculum | Muribaculaceae | Bacteroidales | Bacteroidia | Bacteroidetes | Bacteria |
| ASV4020 | 402 | 1 | 1 | 402 | 100 | NR_102884.1 Ruminococcus champanellensis 18P13 = JCM 17042 16S ribosomal RNA, partial sequence | NR_102884.1 | 1494 | 348 | 749 | 26.91 | 605 | 9E-173 | 379/404 | 93 | 4/404 | 0 | Plus | Plus | Bacteria; Firmicutes; Clostridia; Eubacteriales; Oscillospiraceae; Ruminococcus; Ruminococcus champanellensis | Ruminococcus champanellensis | Ruminococcus | Oscillospiraceae | Eubacteriales | Clostridia | Firmicutes | Bacteria |
| ASV4022 | 402 | 5 | 1 | 387 | 96.27 | NR_114392.1 Faecalicatena orotica strain JCM 1429 16S ribosomal RNA, partial sequence | NR_114392.1 | 1494 | 367 | 753 | 25.9 | 394.5 | 2E-109 | 331/389 | 85 | 4/389 | 1 | Plus | Plus | Bacteria; Firmicutes; Clostridia; Eubacteriales; Lachnospiraceae; Faecalicatena; Faecalicatena orotica | Faecalicatena orotica | Faecalicatena | Lachnospiraceae | Eubacteriales | Clostridia | Firmicutes | Bacteria |
| ASV4025 | 428 | 2 | 1 | 427 | 99.77 | NR_037018.1 Acidaminococcus fermentans strain VR4 16S ribosomal RNA gene, partial sequence | NR_037018.1 | 1516 | 349 | 774 | 28.1 | 544 | 2E-154 | 384/428 | 89 | 3/428 | 0 | Plus | Plus | Bacteria; Firmicutes; Negativicutes; Acidaminococcales; Acidaminococcaceae; Acidaminococcus; Acidaminococcus fermentans | Acidaminococcus fermentans | Acidaminococcus | Acidaminococcaceae | Acidaminococcales | Negativicutes | Firmicutes | Bacteria |
| ASV4026 | 404 | 1 | 3 | 404 | 99.5 | NR_028883.1 Dorea longicatena strain 111-35 16S ribosomal RNA, partial sequence | NR_028883.1 | 1316 | 245 | 645 | 30.47 | 575.4 | 7E-164 | 375/405 | 92 | 7/405 | 1 | Plus | Plus | Bacteria; Firmicutes; Clostridia; Eubacteriales; Lachnospiraceae; Dorea; Dorea longicatena | Dorea longicatena | Dorea | Lachnospiraceae | Eubacteriales | Clostridia | Firmicutes | Bacteria |
| ASV4028 | 402 | 4 | 1 | 400 | 99.5 | NR_117139.2 Anaerostipes hadrus strain DSM 3319 16S ribosomal RNA, partial sequence | NR_117139.2 | 1521 | 363 | 762 | 26.3 | 617.9 | 1E-176 | 378/400 | 94 | 0/400 | 0 | Plus | Plus | Bacteria; Firmicutes; Clostridia; Eubacteriales; Lachnospiraceae; Anaerostipes; Anaerostipes hadrus | Anaerostipes hadrus | Anaerostipes | Lachnospiraceae | Eubacteriales | Clostridia | Firmicutes | Bacteria |
| ASV4029 | 402 | 1 | 1 | 402 | 100 | NR_112652.1 Lactobacillus equicursoris DSM 19284 = JCM 14600 = CIP 110162 strain DI70 16S ribosomal RNA, partial sequence | NR_112652.1 | 1486 | 351 | 777 | 28.73 | 538.5 | 9E-153 | 386/427 | 90 | 25/427 | 5 | Plus | Plus | Bacteria; Firmicutes; Bacilli; Lactobacillales; Lactobacillaceae; Lactobacillus; Lactobacillus equicursoris | Lactobacillus equicursoris | Lactobacillus | Lactobacillaceae | Lactobacillales | Bacilli | Firmicutes | Bacteria |
| ASV4031 | 404 | 1 | 1 | 404 | 100 | NR_044054.1 Blautia wexlerae DSM 19850 16S ribosomal RNA, partial sequence | NR_044054.1 | 1438 | 314 | 715 | 27.96 | 590.2 | 3E-168 | 378/406 | 93 | 6/406 | 1 | Plus | Plus | Bacteria; Firmicutes; Clostridia; Eubacteriales; Lachnospiraceae; Blautia; Blautia wexlerae | Blautia wexlerae | Blautia | Lachnospiraceae | Eubacteriales | Clostridia | Firmicutes | Bacteria |
| ASV4033 | 401 | 1 | 1 | 401 | 100 | NR_144749.1 Ihubacter massiliensis strain Marseille-P2843 16S ribosomal RNA, partial sequence | NR_144749.1 | 1479 | 364 | 766 | 27.25 | 538.5 | 9E-153 | 366/403 | 90 | 2/403 | 0 | Plus | Plus | Bacteria; Firmicutes; Clostridia; Eubacteriales; Clostridiales Family XIII. Incertae Sedis; Ihubacter; Ihubacter massiliensis | Ihubacter massiliensis | Ihubacter | Clostridiales Family XIII. Incertae Sedis | Eubacteriales | Clostridia | Firmicutes | Bacteria |
| ASV4034 | 405 | 1 | 3 | 405 | 99.51 | NR_028816.1 Turicibacter sanguinis strain MOL361 16S ribosomal RNA, partial sequence | NR_028816.1 | 1508 | 329 | 753 | 28.18 | 549.6 | 4E-156 | 386/425 | 90 | 22/425 | 5 | Plus | Plus | Bacteria; Firmicutes; Erysipelotrichia; Erysipelotrichales; Turicibacteraceae; Turicibacter; Turicibacter sanguinis | Turicibacter sanguinis | Turicibacter | Turicibacteraceae | Erysipelotrichales | Erysipelotrichia | Firmicutes | Bacteria |
| ASV4037 | 403 | 1 | 1 | 403 | 100 | NR_147375.1 Colidextribacter massiliensis strain Marseille-P3083 16S ribosomal RNA, partial sequence | NR_147375.1 | 1487 | 364 | 765 | 27.03 | 521.9 | 9E-148 | 364/404 | 90 | 3/404 | 0 | Plus | Plus | Bacteria; Firmicutes; Clostridia; Eubacteriales; ; Colidextribacter; Colidextribacter massiliensis | Colidextribacter massiliensis | Colidextribacter | | Eubacteriales | Clostridia | Firmicutes | Bacteria |
| ASV4039 | 401 | 2 | 1 | 401 | 100 | NR_144748.1 Phocea massiliensis strain Marseille-P2769 16S ribosomal RNA, partial sequence | NR_144748.1 | 1476 | 352 | 752 | 27.17 | 608.7 | 7E-174 | 377/401 | 94 | 0/401 | 0 | Plus | Plus | Bacteria; Firmicutes; Clostridia; Eubacteriales; Oscillospiraceae; Phocea; Phocea massiliensis | Phocea massiliensis | Phocea | Oscillospiraceae | Eubacteriales | Clostridia | Firmicutes | Bacteria |
| ASV4040 | 402 | 1 | 1 | 402 | 100 | NR_027562.1 Anaerofustis stercorihominis strain WAL 14563 16S ribosomal RNA, partial sequence | NR_027562.1 | 1462 | 335 | 736 | 27.5 | 693.6 | 0 | 394/403 | 97 | 2/403 | 0 | Plus | Plus | Bacteria; Firmicutes; Clostridia; Eubacteriales; Eubacteriaceae; Anaerofustis; Anaerofustis stercorihominis | Anaerofustis stercorihominis | Anaerofustis | Eubacteriaceae | Eubacteriales | Clostridia | Firmicutes | Bacteria |
| ASV4041 | 421 | 2 | 1 | 421 | 100 | NR_113208.1 Parabacteroides chinchillae strain JCM 17104 16S ribosomal RNA, partial sequence | NR_113208.1 | 1489 | 353 | 774 | 28.34 | 490.5 | 3E-138 | 372/424 | 87 | 5/424 | 1 | Plus | Plus | Bacteria; Bacteroidetes; Bacteroidia; Bacteroidales; Tannerellaceae; Parabacteroides; Parabacteroides chinchillae | Parabacteroides chinchillae | Parabacteroides | Tannerellaceae | Bacteroidales | Bacteroidia | Bacteroidetes | Bacteria |
| ASV4042 | 405 | 1 | 4 | 405 | 99.26 | NR_115269.1 Catabacter hongkongensis strain HKU16 16S ribosomal RNA, partial sequence | NR_115269.1 | 1453 | 352 | 753 | 27.67 | 510.8 | 2E-144 | 360/402 | 89 | 0/402 | 0 | Plus | Plus | Bacteria; Firmicutes; Clostridia; Eubacteriales; Christensenellaceae; Christensenella; Christensenella hongkongensis | Christensenella hongkongensis | Christensenella | Christensenellaceae | Eubacteriales | Clostridia | Firmicutes | Bacteria |
| ASV4043 | 405 | 3 | 1 | 405 | 100 | NR_115307.1 Ethanoligenens harbinense YUAN-3 16S ribosomal RNA, partial sequence | NR_115307.1 | 1497 | 340 | 743 | 26.99 | 542.2 | 7E-154 | 369/406 | 90 | 3/406 | 0 | Plus | Plus | Bacteria; Firmicutes; Clostridia; Eubacteriales; Oscillospiraceae; Ethanoligenens; Ethanoligenens harbinense | Ethanoligenens harbinense | Ethanoligenens | Oscillospiraceae | Eubacteriales | Clostridia | Firmicutes | Bacteria |
| ASV4044 | 427 | 4 | 4 | 427 | 99.3 | NR_042802.1 Lactobacillus ultunensis strain Kx146C1 16S ribosomal RNA, partial sequence | NR_042802.1 | 1550 | 373 | 796 | 27.35 | 545.9 | 6E-155 | 389/432 | 90 | 16/432 | 3 | Plus | Plus | Bacteria; Firmicutes; Bacilli; Lactobacillales; Lactobacillaceae; Lactobacillus; Lactobacillus ultunensis | Lactobacillus ultunensis | Lactobacillus | Lactobacillaceae | Lactobacillales | Bacilli | Firmicutes | Bacteria |
| ASV4046 | 404 | 1 | 1 | 404 | 100 | NR_041778.1 Desulfovibrio piger strain ATCC 29098 16S ribosomal RNA, partial sequence | NR_041778.1 | 1542 | 357 | 783 | 27.69 | 545.9 | 6E-155 | 387/427 | 90 | 23/427 | 5 | Plus | Plus | Bacteria; Proteobacteria; Deltaproteobacteria; Desulfovibrionales; Desulfovibrionaceae; Desulfovibrio; Desulfovibrio piger | Desulfovibrio piger | Desulfovibrio | Desulfovibrionaceae | Desulfovibrionales | Deltaproteobacteria | Proteobacteria | Bacteria |
| ASV4047 | 427 | 2 | 1 | 426 | 99.77 | NR_113306.1 Megasphaera elsdenii DSM 20460 16S ribosomal RNA, partial sequence | NR_113306.1 | 1174 | 232 | 657 | 36.29 | 651.1 | 0 | 401/426 | 94 | 0/426 | 0 | Plus | Plus | Bacteria; Firmicutes; Negativicutes; Veillonellales; Veillonellaceae; Megasphaera; Megasphaera elsdenii | Megasphaera elsdenii | Megasphaera | Veillonellaceae | Veillonellales | Negativicutes | Firmicutes | Bacteria |
| ASV4048 | 405 | 2 | 1 | 405 | 100 | NR_074114.1 Elusimicrobium minutum strain Pei191 16S ribosomal RNA, partial sequence | NR_074114.1 | 1433 | 356 | 760 | 28.26 | 632.7 | 0 | 384/405 | 94 | 0/405 | 0 | Plus | Plus | Bacteria; Elusimicrobia; Elusimicrobia; Elusimicrobiales; Elusimicrobiaceae; Elusimicrobium; Elusimicrobium minutum | Elusimicrobium minutum | Elusimicrobium | Elusimicrobiaceae | Elusimicrobiales | Elusimicrobia | Elusimicrobia | Bacteria |
| ASV4049 | 421 | 1 | 1 | 421 | 100 | NR_118334.1 Alloprevotella rava strain 81/4-12 16S ribosomal RNA, partial sequence | NR_118334.1 | 1455 | 338 | 759 | 29 | 590.2 | 3E-168 | 389/423 | 91 | 3/423 | 0 | Plus | Plus | Bacteria; Bacteroidetes; Bacteroidia; Bacteroidales; Prevotellaceae; Alloprevotella; Alloprevotella rava | Alloprevotella rava | Alloprevotella | Prevotellaceae | Bacteroidales | Bacteroidia | Bacteroidetes | Bacteria |
| ASV4050 | 422 | 2 | 1 | 422 | 100 | NR_113119.1 Prevotella oris strain JCM 8540 16S ribosomal RNA, partial sequence | NR_113119.1 | 1481 | 345 | 766 | 28.49 | 520 | 4E-147 | 376/423 | 88 | 2/423 | 0 | Plus | Plus | Bacteria; Bacteroidetes; Bacteroidia; Bacteroidales; Prevotellaceae; Prevotella; Prevotella oris | Prevotella oris | Prevotella | Prevotellaceae | Bacteroidales | Bacteroidia | Bacteroidetes | Bacteria |
| ASV4051 | 422 | 1 | 1 | 422 | 100 | NR_144750.1 Prevotellamassilia timonensis strain Marseille-P2831 16S ribosomal RNA, partial sequence | NR_144750.1 | 1493 | 358 | 779 | 28.27 | 630.8 | 2E-180 | 397/424 | 93 | 4/424 | 0 | Plus | Plus | Bacteria; Bacteroidetes; Bacteroidia; Bacteroidales; Prevotellaceae; Prevotellamassilia; Prevotellamassilia timonensis | Prevotellamassilia timonensis | Prevotellamassilia | Prevotellaceae | Bacteroidales | Bacteroidia | Bacteroidetes | Bacteria |
| ASV4052 | 422 | 2 | 1 | 422 | 100 | NR_113105.1 Prevotella histicola strain JCM 15637 16S ribosomal RNA, partial sequence | NR_113105.1 | 1480 | 346 | 767 | 28.51 | 641.9 | 0 | 401/426 | 94 | 8/426 | 1 | Plus | Plus | Bacteria; Bacteroidetes; Bacteroidia; Bacteroidales; Prevotellaceae; Prevotella; Prevotella histicola | Prevotella histicola | Prevotella | Prevotellaceae | Bacteroidales | Bacteroidia | Bacteroidetes | Bacteria |
| ASV4054 | 423 | 1 | 1 | 423 | 100 | NR_042842.1 Prevotella denticola strain ATCC 35308 16S ribosomal RNA, partial sequence | NR_042842.1 | 1506 | 338 | 759 | 28.02 | 516.3 | 5E-146 | 377/425 | 88 | 5/425 | 1 | Plus | Plus | Bacteria; Bacteroidetes; Bacteroidia; Bacteroidales; Prevotellaceae; Prevotella; Prevotella denticola | Prevotella denticola | Prevotella | Prevotellaceae | Bacteroidales | Bacteroidia | Bacteroidetes | Bacteria |
| ASV4056 | 427 | 3 | 1 | 427 | 100 | NR_169348.1 Sutterella faecalis strain KGMB03119 16S ribosomal RNA, partial sequence | NR_169348.1 | 1367 | 336 | 762 | 31.24 | 617.9 | 1E-176 | 397/428 | 92 | 2/428 | 0 | Plus | Plus | Bacteria; Proteobacteria; Betaproteobacteria; Burkholderiales; Sutterellaceae; Sutterella; Sutterella faecalis | Sutterella faecalis | Sutterella | Sutterellaceae | Burkholderiales | Betaproteobacteria | Proteobacteria | Bacteria |
| ASV4057 | 427 | 1 | 1 | 427 | 100 | NR_028816.1 Turicibacter sanguinis strain MOL361 16S ribosomal RNA, partial sequence | NR_028816.1 | 1508 | 327 | 753 | 28.32 | 728.7 | 0 | 416/427 | 97 | 0/427 | 0 | Plus | Plus | Bacteria; Firmicutes; Erysipelotrichia; Erysipelotrichales; Turicibacteraceae; Turicibacter; Turicibacter sanguinis | Turicibacter sanguinis | Turicibacter | Turicibacteraceae | Erysipelotrichales | Erysipelotrichia | Firmicutes | Bacteria |
| ASV4058 | 428 | 1 | 3 | 428 | 99.53 | NR_043577.1 Pelosinus fermentans DSM 17108 strain R7 16S ribosomal RNA, partial sequence | NR_043577.1 | 1404 | 248 | 672 | 30.27 | 459.1 | 8E-129 | 370/429 | 86 | 7/429 | 1 | Plus | Plus | Bacteria; Firmicutes; Negativicutes; Selenomonadales; Sporomusaceae; Pelosinus; Pelosinus fermentans | Pelosinus fermentans | Pelosinus | Sporomusaceae | Selenomonadales | Negativicutes | Firmicutes | Bacteria |
| ASV4060 | 424 | 1 | 1 | 424 | 100 | NR_041364.1 Prevotella stercorea DSM 18206 strain CB35 16S ribosomal RNA, partial sequence | NR_041364.1 | 1488 | 354 | 775 | 28.36 | 605 | 1E-172 | 392/424 | 92 | 2/424 | 0 | Plus | Plus | Bacteria; Bacteroidetes; Bacteroidia; Bacteroidales; Prevotellaceae; Prevotella; Prevotella stercorea | Prevotella stercorea | Prevotella | Prevotellaceae | Bacteroidales | Bacteroidia | Bacteroidetes | Bacteria |
| ASV4061 | 421 | 3 | 1 | 421 | 100 | NR_121773.2 Barnesiella viscericola strain C46 16S ribosomal RNA, complete sequence | NR_121773.2 | 1532 | 365 | 786 | 27.55 | 473.9 | 3E-133 | 367/422 | 86 | 1/422 | 0 | Plus | Plus | Bacteria; Bacteroidetes; Bacteroidia; Bacteroidales; Barnesiellaceae; Barnesiella; Barnesiella viscericola | Barnesiella viscericola | Barnesiella | Barnesiellaceae | Bacteroidales | Bacteroidia | Bacteroidetes | Bacteria |
| ASV4062 | 422 | 1 | 1 | 407 | 96.45 | NR_113411.1 Prevotella copri DSM 18205 strain JCM 13464 16S ribosomal RNA, partial sequence | NR_113411.1 | 1493 | 358 | 764 | 27.26 | 614.2 | 2E-175 | 383/408 | 93 | 2/408 | 0 | Plus | Plus | Bacteria; Bacteroidetes; Bacteroidia; Bacteroidales; Prevotellaceae; Prevotella; Prevotella copri | Prevotella copri | Prevotella | Prevotellaceae | Bacteroidales | Bacteroidia | Bacteroidetes | Bacteria |
| ASV4063 | 422 | 4 | 1 | 422 | 100 | NR_113123.1 Prevotella timonensis 4401737 = DSM 22865 = JCM 15640 16S ribosomal RNA, partial sequence | NR_113123.1 | 1493 | 356 | 777 | 28.27 | 492.3 | 8E-139 | 371/423 | 87 | 2/423 | 0 | Plus | Plus | Bacteria; Bacteroidetes; Bacteroidia; Bacteroidales; Prevotellaceae; Prevotella; Prevotella timonensis | Prevotella timonensis | Prevotella | Prevotellaceae | Bacteroidales | Bacteroidia | Bacteroidetes | Bacteria |
| ASV4064 | 405 | 2 | 3 | 405 | 99.51 | NR_074629.1 Hungateiclostridium thermocellum strain ATCC 27405 16S ribosomal RNA, partial sequence | NR_074629.1 | 1521 | 360 | 762 | 26.5 | 435.1 | 1E-121 | 349/405 | 86 | 4/405 | 0 | Plus | Plus | Bacteria; Firmicutes; Clostridia; Eubacteriales; Oscillospiraceae; Acetivibrio; Acetivibrio thermocellus | Acetivibrio thermocellus | Acetivibrio | Oscillospiraceae | Eubacteriales | Clostridia | Firmicutes | Bacteria |
| ASV4065 | 403 | 3 | 1 | 403 | 100 | NR_134714.1 Clostridium oryzae strain KC3 16S ribosomal RNA, partial sequence | NR_134714.1 | 1458 | 320 | 721 | 27.57 | 466.5 | 4E-131 | 355/405 | 87 | 5/405 | 1 | Plus | Plus | Bacteria; Firmicutes; Clostridia; Eubacteriales; Clostridiaceae; Clostridium; Clostridium oryzae | Clostridium oryzae | Clostridium | Clostridiaceae | Eubacteriales | Clostridia | Firmicutes | Bacteria |
| ASV4066 | 402 | 2 | 3 | 402 | 99.5 | NR_024948.1 Campylobacter hyointestinalis subsp. lawsonii strain CHY5 16S ribosomal RNA, partial sequence | NR_024948.1 | 1419 | 320 | 720 | 28.26 | 590.2 | 3E-168 | 377/404 | 93 | 7/404 | 1 | Plus | Plus | Bacteria; Proteobacteria; Epsilonproteobacteria; Campylobacterales; Campylobacteraceae; Campylobacter; Campylobacter hyointestinalis | Campylobacter hyointestinalis | Campylobacter | Campylobacteraceae | Campylobacterales | Epsilonproteobacteria | Proteobacteria | Bacteria |
| ASV4068 | 405 | 1 | 1 | 405 | 100 | NR_029239.3 Clostridium chartatabidum strain 163 16S ribosomal RNA, partial sequence | NR_029239.3 | 1460 | 323 | 724 | 27.53 | 507.1 | 3E-143 | 365/408 | 89 | 9/408 | 2 | Plus | Plus | Bacteria; Firmicutes; Clostridia; Eubacteriales; Clostridiaceae; Clostridium; Clostridium chartatabidum | Clostridium chartatabidum | Clostridium | Clostridiaceae | Eubacteriales | Clostridia | Firmicutes | Bacteria |
| ASV4070 | 405 | 1 | 1 | 405 | 100 | NR_144743.1 Christensenella timonensis strain Marseille-P2437 16S ribosomal RNA, partial sequence | NR_144743.1 | 1498 | 369 | 773 | 27.04 | 516.3 | 4E-146 | 366/408 | 89 | 6/408 | 1 | Plus | Plus | Bacteria; Firmicutes; Clostridia; Eubacteriales; Christensenellaceae; Christensenella; Christensenella timonensis | Christensenella timonensis | Christensenella | Christensenellaceae | Eubacteriales | Clostridia | Firmicutes | Bacteria |
| ASV4071 | 402 | 1 | 1 | 402 | 100 | NR_102884.1 Ruminococcus champanellensis 18P13 = JCM 17042 16S ribosomal RNA, partial sequence | NR_102884.1 | 1494 | 348 | 749 | 26.91 | 588.4 | 9E-168 | 376/404 | 93 | 4/404 | 0 | Plus | Plus | Bacteria; Firmicutes; Clostridia; Eubacteriales; Oscillospiraceae; Ruminococcus; Ruminococcus champanellensis | Ruminococcus champanellensis | Ruminococcus | Oscillospiraceae | Eubacteriales | Clostridia | Firmicutes | Bacteria |
| ASV4073 | 420 | 1 | 1 | 420 | 100 | NR_144616.1 Muribaculum intestinale strain YL27 16S ribosomal RNA, partial sequence | NR_144616.1 | 1460 | 297 | 718 | 28.9 | 573.6 | 3E-163 | 388/425 | 91 | 8/425 | 1 | Plus | Plus | Bacteria; Bacteroidetes; Bacteroidia; Bacteroidales; Muribaculaceae; Muribaculum; Muribaculum intestinale | Muribaculum intestinale | Muribaculum | Muribaculaceae | Bacteroidales | Bacteroidia | Bacteroidetes | Bacteria |
| ASV4074 | 406 | 1 | 1 | 391 | 96.31 | NR_115340.1 Saccharofermentans acetigenes strain P6 16S ribosomal RNA, partial sequence | NR_115340.1 | 1510 | 353 | 742 | 25.83 | 416.6 | 5E-116 | 337/392 | 85 | 3/392 | 0 | Plus | Plus | Bacteria; Firmicutes; Clostridia; Eubacteriales; Oscillospiraceae; Saccharofermentans; Saccharofermentans acetigenes | Saccharofermentans acetigenes | Saccharofermentans | Oscillospiraceae | Eubacteriales | Clostridia | Firmicutes | Bacteria |
| ASV4075 | 405 | 1 | 1 | 404 | 99.75 | NR_144742.1 Christensenella massiliensis strain Marseille-P2438 16S ribosomal RNA, partial sequence | NR_144742.1 | 1499 | 369 | 772 | 26.95 | 486.8 | 3E-137 | 357/404 | 88 | 0/404 | 0 | Plus | Plus | Bacteria; Firmicutes; Clostridia; Eubacteriales; Christensenellaceae; Christensenella; Christensenella massiliensis | Christensenella massiliensis | Christensenella | Christensenellaceae | Eubacteriales | Clostridia | Firmicutes | Bacteria |
| ASV4076 | 427 | 2 | 1 | 427 | 100 | NR_037018.1 Acidaminococcus fermentans strain VR4 16S ribosomal RNA gene, partial sequence | NR_037018.1 | 1516 | 349 | 775 | 28.17 | 645.6 | 0 | 401/427 | 93 | 0/427 | 0 | Plus | Plus | Bacteria; Firmicutes; Negativicutes; Acidaminococcales; Acidaminococcaceae; Acidaminococcus; Acidaminococcus fermentans | Acidaminococcus fermentans | Acidaminococcus | Acidaminococcaceae | Acidaminococcales | Negativicutes | Firmicutes | Bacteria |
| ASV4077 | 421 | 2 | 1 | 421 | 100 | NR_113077.1 Paraprevotella clara strain JCM 14859 16S ribosomal RNA, partial sequence | NR_113077.1 | 1480 | 346 | 766 | 28.45 | 473.9 | 3E-133 | 366/421 | 86 | 0/421 | 0 | Plus | Plus | Bacteria; Bacteroidetes; Bacteroidia; Bacteroidales; Prevotellaceae; Paraprevotella; Paraprevotella clara | Paraprevotella clara | Paraprevotella | Prevotellaceae | Bacteroidales | Bacteroidia | Bacteroidetes | Bacteria |
| ASV4080 | 406 | 1 | 1 | 406 | 100 | NR_158113.1 Anaerotignum aminivorans strain SH021 16S ribosomal RNA, partial sequence | NR_158113.1 | 1471 | 345 | 749 | 27.53 | 665.9 | 0 | 391/406 | 96 | 1/406 | 0 | Plus | Plus | Bacteria; Firmicutes; Clostridia; Eubacteriales; Lachnospiraceae; Anaerotignum; Anaerotignum aminivorans | Anaerotignum aminivorans | Anaerotignum | Lachnospiraceae | Eubacteriales | Clostridia | Firmicutes | Bacteria |
| ASV4083 | 401 | 1 | 1 | 401 | 100 | NR_144740.1 Romboutsia timonensis strain DR1 16S ribosomal RNA, partial sequence | NR_144740.1 | 1466 | 338 | 738 | 27.35 | 630.8 | 1E-180 | 381/401 | 95 | 0/401 | 0 | Plus | Plus | Bacteria; Firmicutes; Clostridia; Eubacteriales; Peptostreptococcaceae; Romboutsia; Romboutsia timonensis | Romboutsia timonensis | Romboutsia | Peptostreptococcaceae | Eubacteriales | Clostridia | Firmicutes | Bacteria |
| ASV4084 | 403 | 2 | 1 | 403 | 100 | NR_144737.1 Emergencia timonensis strain SN18 16S ribosomal RNA, partial sequence | NR_144737.1 | 1480 | 364 | 767 | 27.3 | 529.3 | 6E-150 | 367/406 | 90 | 5/406 | 1 | Plus | Plus | Bacteria; Firmicutes; Clostridia; Eubacteriales; Clostridiales Family XIII. Incertae Sedis; Emergencia; Emergencia timonensis | Emergencia timonensis | Emergencia | Clostridiales Family XIII. Incertae Sedis | Eubacteriales | Clostridia | Firmicutes | Bacteria |
| ASV4085 | 402 | 2 | 1 | 402 | 100 | NR_102880.1 Acetoanaerobium sticklandii strain DSM 519 16S ribosomal RNA, partial sequence | NR_102880.1 | 1499 | 345 | 745 | 26.75 | 492.3 | 7E-139 | 359/404 | 88 | 5/404 | 1 | Plus | Plus | Bacteria; Firmicutes; Clostridia; Eubacteriales; Peptostreptococcaceae; Acetoanaerobium; Acetoanaerobium sticklandii | Acetoanaerobium sticklandii | Acetoanaerobium | Peptostreptococcaceae | Eubacteriales | Clostridia | Firmicutes | Bacteria |
| ASV4086 | 422 | 1 | 1 | 422 | 100 | NR_113411.1 Prevotella copri DSM 18205 strain JCM 13464 16S ribosomal RNA, partial sequence | NR_113411.1 | 1493 | 358 | 779 | 28.27 | 586.5 | 3E-167 | 388/423 | 91 | 2/423 | 0 | Plus | Plus | Bacteria; Bacteroidetes; Bacteroidia; Bacteroidales; Prevotellaceae; Prevotella; Prevotella copri | Prevotella copri | Prevotella | Prevotellaceae | Bacteroidales | Bacteroidia | Bacteroidetes | Bacteria |
| ASV4087 | 402 | 1 | 1 | 402 | 100 | NR_118675.1 [Eubacterium] siraeum strain ATCC 29066 16S ribosomal RNA, partial sequence | NR_118675.1 | 1453 | 356 | 757 | 27.67 | 595.7 | 5E-170 | 374/402 | 93 | 0/402 | 0 | Plus | Plus | Bacteria; Firmicutes; Clostridia; Eubacteriales; Oscillospiraceae; ; [Eubacterium] siraeum | [Eubacterium] siraeum | | Oscillospiraceae | Eubacteriales | Clostridia | Firmicutes | Bacteria |
| ASV4089 | 404 | 2 | 1 | 404 | 100 | NR_074793.2 Oscillibacter valericigenes strain Sjm18-20 16S ribosomal RNA, complete sequence | NR_074793.2 | 1530 | 372 | 775 | 26.41 | 592 | 7E-169 | 376/404 | 93 | 0/404 | 0 | Plus | Plus | Bacteria; Firmicutes; Clostridia; Eubacteriales; Oscillospiraceae; Oscillibacter; Oscillibacter valericigenes | Oscillibacter valericigenes | Oscillibacter | Oscillospiraceae | Eubacteriales | Clostridia | Firmicutes | Bacteria |
| ASV4092 | 422 | 1 | 1 | 422 | 100 | NR_040877.1 Prevotella copri DSM 18205 strain C87 16S ribosomal RNA, partial sequence | NR_040877.1 | 1494 | 358 | 779 | 28.25 | 721.3 | 0 | 411/422 | 97 | 0/422 | 0 | Plus | Plus | Bacteria; Bacteroidetes; Bacteroidia; Bacteroidales; Prevotellaceae; Prevotella; Prevotella copri | Prevotella copri | Prevotella | Prevotellaceae | Bacteroidales | Bacteroidia | Bacteroidetes | Bacteria |
| ASV4093 | 422 | 1 | 1 | 422 | 100 | NR_145844.1 Bacteroides caecigallinarum strain C13EG111 16S ribosomal RNA, partial sequence | NR_145844.1 | 1489 | 356 | 777 | 28.34 | 442.5 | 8E-124 | 363/424 | 85 | 4/424 | 0 | Plus | Plus | Bacteria; Bacteroidetes; Bacteroidia; Bacteroidales; Bacteroidaceae; Bacteroides; Bacteroides caecigallinarum | Bacteroides caecigallinarum | Bacteroides | Bacteroidaceae | Bacteroidales | Bacteroidia | Bacteroidetes | Bacteria |
| ASV4094 | 405 | 1 | 1 | 405 | 100 | NR_024829.1 Hungateiclostridium straminisolvens strain CSK1 16S ribosomal RNA, partial sequence | NR_024829.1 | 1447 | 338 | 742 | 27.99 | 593.9 | 2E-169 | 377/405 | 93 | 0/405 | 0 | Plus | Plus | Bacteria; Firmicutes; Clostridia; Eubacteriales; Oscillospiraceae; Acetivibrio; Acetivibrio straminisolvens | Acetivibrio straminisolvens | Acetivibrio | Oscillospiraceae | Eubacteriales | Clostridia | Firmicutes | Bacteria |
| ASV4095 | 402 | 2 | 1 | 402 | 100 | NR_104892.1 Clostridium moniliforme strain 2055 16S ribosomal RNA, partial sequence | NR_104892.1 | 1488 | 330 | 731 | 27.02 | 671.5 | 0 | 389/402 | 96 | 0/402 | 0 | Plus | Plus | Bacteria; Firmicutes; Clostridia; Eubacteriales; Clostridiaceae; Clostridium; Clostridium moniliforme | Clostridium moniliforme | Clostridium | Clostridiaceae | Eubacteriales | Clostridia | Firmicutes | Bacteria |
| ASV4096 | 405 | 1 | 1 | 405 | 100 | NR_028816.1 Turicibacter sanguinis strain MOL361 16S ribosomal RNA, partial sequence | NR_028816.1 | 1508 | 327 | 753 | 28.32 | 569.9 | 3E-162 | 391/427 | 91 | 22/427 | 5 | Plus | Plus | Bacteria; Firmicutes; Erysipelotrichia; Erysipelotrichales; Turicibacteraceae; Turicibacter; Turicibacter sanguinis | Turicibacter sanguinis | Turicibacter | Turicibacteraceae | Erysipelotrichales | Erysipelotrichia | Firmicutes | Bacteria |
| ASV4097 | 405 | 1 | 3 | 405 | 99.51 | NR_026476.1 Succinivibrio dextrinosolvens strain 0554 16S ribosomal RNA, partial sequence | NR_026476.1 | 1463 | 333 | 732 | 27.34 | 481.2 | 2E-135 | 356/403 | 88 | 3/403 | 0 | Plus | Plus | Bacteria; Proteobacteria; Gammaproteobacteria; Aeromonadales; Succinivibrionaceae; Succinivibrio; Succinivibrio dextrinosolvens | Succinivibrio dextrinosolvens | Succinivibrio | Succinivibrionaceae | Aeromonadales | Gammaproteobacteria | Proteobacteria | Bacteria |
| ASV4100 | 422 | 5 | 1 | 422 | 100 | NR_113411.1 Prevotella copri DSM 18205 strain JCM 13464 16S ribosomal RNA, partial sequence | NR_113411.1 | 1493 | 358 | 779 | 28.27 | 464.6 | 2E-130 | 368/425 | 86 | 6/425 | 1 | Plus | Plus | Bacteria; Bacteroidetes; Bacteroidia; Bacteroidales; Prevotellaceae; Prevotella; Prevotella copri | Prevotella copri | Prevotella | Prevotellaceae | Bacteroidales | Bacteroidia | Bacteroidetes | Bacteria |
| ASV4101 | 422 | 2 | 1 | 422 | 100 | NR_113119.1 Prevotella oris strain JCM 8540 16S ribosomal RNA, partial sequence | NR_113119.1 | 1481 | 345 | 766 | 28.49 | 459.1 | 8E-129 | 370/428 | 86 | 12/428 | 2 | Plus | Plus | Bacteria; Bacteroidetes; Bacteroidia; Bacteroidales; Prevotellaceae; Prevotella; Prevotella oris | Prevotella oris | Prevotella | Prevotellaceae | Bacteroidales | Bacteroidia | Bacteroidetes | Bacteria |
| ASV4102 | 402 | 5 | 1 | 402 | 100 | NR_117563.1 Faecalicatena fissicatena 16S ribosomal RNA, partial sequence | NR_117563.1 | 1526 | 368 | 769 | 26.34 | 527.4 | 2E-149 | 366/405 | 90 | 6/405 | 1 | Plus | Plus | Bacteria; Firmicutes; Clostridia; Eubacteriales; Lachnospiraceae; Faecalicatena; Faecalicatena fissicatena | Faecalicatena fissicatena | Faecalicatena | Lachnospiraceae | Eubacteriales | Clostridia | Firmicutes | Bacteria |
| ASV4103 | 427 | 1 | 1 | 427 | 100 | NR_044111.1 Selenomonas bovis strain WG 16S ribosomal RNA, partial sequence | NR_044111.1 | 1525 | 370 | 796 | 28 | 750.9 | 0 | 420/427 | 98 | 0/427 | 0 | Plus | Plus | Bacteria; Firmicutes; Negativicutes; Selenomonadales; Selenomonadaceae; Selenomonas; Selenomonas bovis | Selenomonas bovis | Selenomonas | Selenomonadaceae | Selenomonadales | Negativicutes | Firmicutes | Bacteria |
| ASV4107 | 402 | 4 | 1 | 402 | 100 | NR_074399.1 Ruminococcus albus 7 = DSM 20455 16S ribosomal RNA, partial sequence | NR_074399.1 | 1500 | 350 | 751 | 26.8 | 483.1 | 4E-136 | 356/403 | 88 | 2/403 | 0 | Plus | Plus | Bacteria; Firmicutes; Clostridia; Eubacteriales; Oscillospiraceae; Ruminococcus; Ruminococcus albus | Ruminococcus albus | Ruminococcus | Oscillospiraceae | Eubacteriales | Clostridia | Firmicutes | Bacteria |
| ASV4108 | 402 | 1 | 2 | 402 | 99.75 | NR_027579.1 Ruminococcus lactaris ATCC 29176 16S ribosomal RNA, partial sequence | NR_027579.1 | 1402 | 334 | 734 | 28.6 | 665.9 | 0 | 387/401 | 96 | 0/401 | 0 | Plus | Plus | Bacteria; Firmicutes; Clostridia; Eubacteriales; Lachnospiraceae; Mediterraneibacter; [Ruminococcus] lactaris | [Ruminococcus] lactaris | Mediterraneibacter | Lachnospiraceae | Eubacteriales | Clostridia | Firmicutes | Bacteria |
| ASV4109 | 405 | 3 | 1 | 405 | 100 | NR_114728.1 Clostridium tertium strain ATCC 14573 16S ribosomal RNA, partial sequence | NR_114728.1 | 1415 | 304 | 705 | 28.41 | 590.2 | 3E-168 | 379/407 | 93 | 7/407 | 1 | Plus | Plus | Bacteria; Firmicutes; Clostridia; Eubacteriales; Clostridiaceae; Clostridium; Clostridium tertium | Clostridium tertium | Clostridium | Clostridiaceae | Eubacteriales | Clostridia | Firmicutes | Bacteria |
| ASV4110 | 422 | 2 | 1 | 422 | 100 | NR_113151.1 Alistipes onderdonkii strain JCM 16771 16S ribosomal RNA, partial sequence | NR_113151.1 | 1489 | 355 | 776 | 28.33 | 686.2 | 0 | 405/422 | 95 | 0/422 | 0 | Plus | Plus | Bacteria; Bacteroidetes; Bacteroidia; Bacteroidales; Rikenellaceae; Alistipes; Alistipes onderdonkii | Alistipes onderdonkii | Alistipes | Rikenellaceae | Bacteroidales | Bacteroidia | Bacteroidetes | Bacteria |
| ASV4111 | 402 | 3 | 1 | 402 | 100 | NR_114728.1 Clostridium tertium strain ATCC 14573 16S ribosomal RNA, partial sequence | NR_114728.1 | 1415 | 304 | 705 | 28.41 | 544 | 2E-154 | 367/403 | 91 | 2/403 | 0 | Plus | Plus | Bacteria; Firmicutes; Clostridia; Eubacteriales; Clostridiaceae; Clostridium; Clostridium tertium | Clostridium tertium | Clostridium | Clostridiaceae | Eubacteriales | Clostridia | Firmicutes | Bacteria |
| ASV4115 | 422 | 1 | 1 | 422 | 100 | NR_113411.1 Prevotella copri DSM 18205 strain JCM 13464 16S ribosomal RNA, partial sequence | NR_113411.1 | 1493 | 358 | 779 | 28.27 | 641.9 | 0 | 398/423 | 94 | 2/423 | 0 | Plus | Plus | Bacteria; Bacteroidetes; Bacteroidia; Bacteroidales; Prevotellaceae; Prevotella; Prevotella copri | Prevotella copri | Prevotella | Prevotellaceae | Bacteroidales | Bacteroidia | Bacteroidetes | Bacteria |
| ASV4119 | 427 | 1 | 1 | 426 | 99.77 | NR_027596.1 Mitsuokella multacida strain DSM 20544 16S ribosomal RNA, partial sequence | NR_027596.1 | 1511 | 346 | 771 | 28.19 | 640.1 | 0 | 399/426 | 93 | 0/426 | 0 | Plus | Plus | Bacteria; Firmicutes; Negativicutes; Selenomonadales; Selenomonadaceae; Mitsuokella; Mitsuokella multacida | Mitsuokella multacida | Mitsuokella | Selenomonadaceae | Selenomonadales | Negativicutes | Firmicutes | Bacteria |
| ASV4120 | 422 | 1 | 1 | 422 | 100 | NR_040877.1 Prevotella copri DSM 18205 strain C87 16S ribosomal RNA, partial sequence | NR_040877.1 | 1494 | 358 | 779 | 28.25 | 660.4 | 0 | 401/423 | 94 | 2/423 | 0 | Plus | Plus | Bacteria; Bacteroidetes; Bacteroidia; Bacteroidales; Prevotellaceae; Prevotella; Prevotella copri | Prevotella copri | Prevotella | Prevotellaceae | Bacteroidales | Bacteroidia | Bacteroidetes | Bacteria |
| ASV4121 | 427 | 1 | 1 | 427 | 100 | NR_029348.1 Treponema brennaborense strain DD5/3 16S ribosomal RNA, partial sequence | NR_029348.1 | 1497 | 305 | 732 | 28.59 | 514.5 | 2E-145 | 379/429 | 88 | 3/429 | 0 | Plus | Plus | Bacteria; Spirochaetes; Spirochaetia; Spirochaetales; Spirochaetaceae; Treponema; Treponema brennaborense | Treponema brennaborense | Treponema | Spirochaetaceae | Spirochaetales | Spirochaetia | Spirochaetes | Bacteria |
| ASV4122 | 405 | 1 | 1 | 405 | 100 | NR_159227.1 Monoglobus pectinilyticus strain 14 16S ribosomal RNA, partial sequence | NR_159227.1 | 1493 | 368 | 769 | 26.93 | 551.4 | 1E-156 | 370/405 | 91 | 3/405 | 0 | Plus | Plus | Bacteria; Firmicutes; Clostridia; Eubacteriales; Oscillospiraceae; Monoglobus; Monoglobus pectinilyticus | Monoglobus pectinilyticus | Monoglobus | Oscillospiraceae | Eubacteriales | Clostridia | Firmicutes | Bacteria |
| ASV4126 | 405 | 1 | 4 | 405 | 99.26 | NR_112900.1 Christensenella minuta strain YIT 12065 16S ribosomal RNA, partial sequence | NR_112900.1 | 1497 | 361 | 762 | 26.85 | 477.6 | 2E-134 | 354/402 | 88 | 0/402 | 0 | Plus | Plus | Bacteria; Firmicutes; Clostridia; Eubacteriales; Christensenellaceae; Christensenella; Christensenella minuta | Christensenella minuta | Christensenella | Christensenellaceae | Eubacteriales | Clostridia | Firmicutes | Bacteria |
| ASV4127 | 423 | 1 | 1 | 423 | 100 | NR_041364.1 Prevotella stercorea DSM 18206 strain CB35 16S ribosomal RNA, partial sequence | NR_041364.1 | 1488 | 354 | 775 | 28.36 | 636.4 | 0 | 400/426 | 93 | 7/426 | 1 | Plus | Plus | Bacteria; Bacteroidetes; Bacteroidia; Bacteroidales; Prevotellaceae; Prevotella; Prevotella stercorea | Prevotella stercorea | Prevotella | Prevotellaceae | Bacteroidales | Bacteroidia | Bacteroidetes | Bacteria |
| ASV4128 | 405 | 1 | 1 | 405 | 100 | NR_144743.1 Christensenella timonensis strain Marseille-P2437 16S ribosomal RNA, partial sequence | NR_144743.1 | 1498 | 369 | 773 | 27.04 | 460.9 | 2E-129 | 355/407 | 87 | 4/407 | 0 | Plus | Plus | Bacteria; Firmicutes; Clostridia; Eubacteriales; Christensenellaceae; Christensenella; Christensenella timonensis | Christensenella timonensis | Christensenella | Christensenellaceae | Eubacteriales | Clostridia | Firmicutes | Bacteria |
| ASV4129 | 404 | 2 | 3 | 404 | 99.5 | NR_144696.1 Clostridium saudiense strain JCC 16S ribosomal RNA, partial sequence | NR_144696.1 | 1478 | 352 | 751 | 27.06 | 592 | 7E-169 | 375/402 | 93 | 2/402 | 0 | Plus | Plus | Bacteria; Firmicutes; Clostridia; Eubacteriales; Clostridiaceae; Clostridium; Clostridium saudiense | Clostridium saudiense | Clostridium | Clostridiaceae | Eubacteriales | Clostridia | Firmicutes | Bacteria |
| ASV4131 | 402 | 3 | 1 | 402 | 100 | NR_156081.1 Kineothrix alysoides strain KNHs209 16S ribosomal RNA, partial sequence | NR_156081.1 | 1411 | 353 | 754 | 28.49 | 610.5 | 2E-174 | 378/402 | 94 | 0/402 | 0 | Plus | Plus | Bacteria; Firmicutes; Clostridia; Eubacteriales; Lachnospiraceae; Kineothrix; Kineothrix alysoides | Kineothrix alysoides | Kineothrix | Lachnospiraceae | Eubacteriales | Clostridia | Firmicutes | Bacteria |
| ASV4133 | 405 | 1 | 1 | 405 | 100 | NR_114789.1 [Clostridium] leptum strain DSM 753 16S ribosomal RNA, partial sequence | NR_114789.1 | 1505 | 364 | 765 | 26.71 | 534.8 | 1E-151 | 367/405 | 90 | 3/405 | 0 | Plus | Plus | Bacteria; Firmicutes; Clostridia; Eubacteriales; Oscillospiraceae; ; [Clostridium] leptum | [Clostridium] leptum | | Oscillospiraceae | Eubacteriales | Clostridia | Firmicutes | Bacteria |
| ASV4134 | 405 | 2 | 1 | 405 | 100 | NG_041947.1 Ruminiclostridium cellulolyticum H10 16S ribosomal RNA gene, complete sequence | NG_041947.1 | 1641 | 486 | 890 | 24.68 | 538.5 | 9E-153 | 367/405 | 90 | 0/405 | 0 | Plus | Plus | Bacteria; Firmicutes; Clostridia; Eubacteriales; Oscillospiraceae; Ruminiclostridium; Ruminiclostridium cellulolyticum | Ruminiclostridium cellulolyticum | Ruminiclostridium | Oscillospiraceae | Eubacteriales | Clostridia | Firmicutes | Bacteria |
| ASV4135 | 405 | 1 | 1 | 405 | 100 | NR_119067.1 Cellulosilyticum lentocellum DSM 5427 16S ribosomal RNA, partial sequence | NR_119067.1 | 1458 | 314 | 718 | 27.78 | 449.9 | 5E-126 | 353/407 | 86 | 4/407 | 0 | Plus | Plus | Bacteria; Firmicutes; Clostridia; Eubacteriales; Lachnospiraceae; Cellulosilyticum; Cellulosilyticum lentocellum | Cellulosilyticum lentocellum | Cellulosilyticum | Lachnospiraceae | Eubacteriales | Clostridia | Firmicutes | Bacteria |
| ASV4138 | 422 | 1 | 1 | 422 | 100 | NR_113411.1 Prevotella copri DSM 18205 strain JCM 13464 16S ribosomal RNA, partial sequence | NR_113411.1 | 1493 | 358 | 779 | 28.27 | 619.7 | 3E-177 | 393/422 | 93 | 0/422 | 0 | Plus | Plus | Bacteria; Bacteroidetes; Bacteroidia; Bacteroidales; Prevotellaceae; Prevotella; Prevotella copri | Prevotella copri | Prevotella | Prevotellaceae | Bacteroidales | Bacteroidia | Bacteroidetes | Bacteria |
| ASV4139 | 421 | 3 | 1 | 421 | 100 | NR_102980.1 Megasphaera elsdenii DSM 20460 16S ribosomal RNA, partial sequence | NR_102980.1 | 1552 | 369 | 795 | 27.51 | 590.2 | 3E-168 | 394/429 | 91 | 10/429 | 2 | Plus | Plus | Bacteria; Firmicutes; Negativicutes; Veillonellales; Veillonellaceae; Megasphaera; Megasphaera elsdenii | Megasphaera elsdenii | Megasphaera | Veillonellaceae | Veillonellales | Negativicutes | Firmicutes | Bacteria |
| ASV4140 | 427 | 1 | 1 | 427 | 100 | NR_157643.1 Treponema rectale strain CHPA 16S ribosomal RNA, partial sequence | NR_157643.1 | 1309 | 259 | 685 | 32.62 | 590.2 | 3E-168 | 393/429 | 91 | 4/429 | 0 | Plus | Plus | Bacteria; Spirochaetes; Spirochaetia; Spirochaetales; Spirochaetaceae; Treponema; Treponema rectale | Treponema rectale | Treponema | Spirochaetaceae | Spirochaetales | Spirochaetia | Spirochaetes | Bacteria |
| ASV4141 | 402 | 4 | 1 | 402 | 100 | NR_042802.1 Lactobacillus ultunensis strain Kx146C1 16S ribosomal RNA, partial sequence | NR_042802.1 | 1550 | 370 | 796 | 27.55 | 549.6 | 4E-156 | 388/427 | 90 | 25/427 | 5 | Plus | Plus | Bacteria; Firmicutes; Bacilli; Lactobacillales; Lactobacillaceae; Lactobacillus; Lactobacillus ultunensis | Lactobacillus ultunensis | Lactobacillus | Lactobacillaceae | Lactobacillales | Bacilli | Firmicutes | Bacteria |
| ASV4142 | 422 | 1 | 1 | 422 | 100 | NR_113411.1 Prevotella copri DSM 18205 strain JCM 13464 16S ribosomal RNA, partial sequence | NR_113411.1 | 1493 | 358 | 779 | 28.27 | 653 | 0 | 399/422 | 94 | 0/422 | 0 | Plus | Plus | Bacteria; Bacteroidetes; Bacteroidia; Bacteroidales; Prevotellaceae; Prevotella; Prevotella copri | Prevotella copri | Prevotella | Prevotellaceae | Bacteroidales | Bacteroidia | Bacteroidetes | Bacteria |
| ASV4143 | 427 | 1 | 1 | 427 | 100 | NR_028840.1 Mitsuokella jalaludinii strain M9 16S ribosomal RNA, partial sequence | NR_028840.1 | 1543 | 362 | 788 | 27.67 | 701 | 0 | 413/429 | 96 | 4/429 | 0 | Plus | Plus | Bacteria; Firmicutes; Negativicutes; Selenomonadales; Selenomonadaceae; Mitsuokella; Mitsuokella jalaludinii | Mitsuokella jalaludinii | Mitsuokella | Selenomonadaceae | Selenomonadales | Negativicutes | Firmicutes | Bacteria |
| ASV4145 | 405 | 1 | 1 | 405 | 100 | NR_118156.1 Oscillibacter ruminantium GH1 16S ribosomal RNA, partial sequence | NR_118156.1 | 1409 | 321 | 724 | 28.67 | 575.4 | 7E-164 | 376/407 | 92 | 5/407 | 1 | Plus | Plus | Bacteria; Firmicutes; Clostridia; Eubacteriales; Oscillospiraceae; Oscillibacter; Oscillibacter ruminantium | Oscillibacter ruminantium | Oscillibacter | Oscillospiraceae | Eubacteriales | Clostridia | Firmicutes | Bacteria |
| ASV4146 | 402 | 3 | 3 | 402 | 99.5 | NR_114728.1 Clostridium tertium strain ATCC 14573 16S ribosomal RNA, partial sequence | NR_114728.1 | 1415 | 306 | 705 | 28.27 | 518.2 | 1E-146 | 362/402 | 90 | 4/402 | 0 | Plus | Plus | Bacteria; Firmicutes; Clostridia; Eubacteriales; Clostridiaceae; Clostridium; Clostridium tertium | Clostridium tertium | Clostridium | Clostridiaceae | Eubacteriales | Clostridia | Firmicutes | Bacteria |
| ASV4151 | 427 | 1 | 3 | 426 | 99.3 | NR_027596.1 Mitsuokella multacida strain DSM 20544 16S ribosomal RNA, partial sequence | NR_027596.1 | 1511 | 348 | 771 | 28.06 | 630.8 | 2E-180 | 397/425 | 93 | 2/425 | 0 | Plus | Plus | Bacteria; Firmicutes; Negativicutes; Selenomonadales; Selenomonadaceae; Mitsuokella; Mitsuokella multacida | Mitsuokella multacida | Mitsuokella | Selenomonadaceae | Selenomonadales | Negativicutes | Firmicutes | Bacteria |
| ASV4152 | 402 | 1 | 1 | 402 | 100 | NR_159261.1 Bifidobacterium callitrichidarum strain TRI 5 16S ribosomal RNA, partial sequence | NR_159261.1 | 1374 | 279 | 686 | 29.69 | 560.7 | 2E-159 | 374/408 | 91 | 6/408 | 1 | Plus | Plus | Bacteria; Actinobacteria; Actinomycetia; Bifidobacteriales; Bifidobacteriaceae; Bifidobacterium; Bifidobacterium callitrichidarum | Bifidobacterium callitrichidarum | Bifidobacterium | Bifidobacteriaceae | Bifidobacteriales | Actinomycetia | Actinobacteria | Bacteria |
| ASV4154 | 427 | 1 | 1 | 426 | 99.77 | NR_112902.1 Phascolarctobacterium succinatutens strain YIT 12067 16S ribosomal RNA, partial sequence | NR_112902.1 | 1610 | 444 | 869 | 26.46 | 660.4 | 0 | 403/426 | 94 | 0/426 | 0 | Plus | Plus | Bacteria; Firmicutes; Negativicutes; Acidaminococcales; Acidaminococcaceae; Phascolarctobacterium; Phascolarctobacterium succinatutens | Phascolarctobacterium succinatutens | Phascolarctobacterium | Acidaminococcaceae | Acidaminococcales | Negativicutes | Firmicutes | Bacteria |
| ASV4156 | 405 | 1 | 1 | 405 | 100 | NR_118554.1 Intestinimonas butyriciproducens strain SRB-521-5-I 16S ribosomal RNA, partial sequence | NR_118554.1 | 1475 | 346 | 750 | 27.46 | 533 | 4E-151 | 367/406 | 90 | 2/406 | 0 | Plus | Plus | Bacteria; Firmicutes; Clostridia; Eubacteriales; ; Intestinimonas; Intestinimonas butyriciproducens | Intestinimonas butyriciproducens | Intestinimonas | | Eubacteriales | Clostridia | Firmicutes | Bacteria |
| ASV4157 | 405 | 1 | 3 | 405 | 99.51 | NR_044054.1 Blautia wexlerae DSM 19850 16S ribosomal RNA, partial sequence | NR_044054.1 | 1438 | 316 | 715 | 27.82 | 564.3 | 2E-160 | 371/403 | 92 | 3/403 | 0 | Plus | Plus | Bacteria; Firmicutes; Clostridia; Eubacteriales; Lachnospiraceae; Blautia; Blautia wexlerae | Blautia wexlerae | Blautia | Lachnospiraceae | Eubacteriales | Clostridia | Firmicutes | Bacteria |
| ASV4159 | 402 | 1 | 3 | 402 | 99.5 | NR_026476.1 Succinivibrio dextrinosolvens strain 0554 16S ribosomal RNA, partial sequence | NR_026476.1 | 1463 | 333 | 732 | 27.34 | 529.3 | 6E-150 | 363/401 | 90 | 2/401 | 0 | Plus | Plus | Bacteria; Proteobacteria; Gammaproteobacteria; Aeromonadales; Succinivibrionaceae; Succinivibrio; Succinivibrio dextrinosolvens | Succinivibrio dextrinosolvens | Succinivibrio | Succinivibrionaceae | Aeromonadales | Gammaproteobacteria | Proteobacteria | Bacteria |
| ASV4161 | 402 | 1 | 5 | 402 | 99 | NR_170495.1 Blautia faecicola strain KGMB01111 16S ribosomal RNA, complete sequence | NR_170495.1 | 1534 | 376 | 773 | 25.95 | 564.3 | 2E-160 | 367/398 | 92 | 0/398 | 0 | Plus | Plus | Bacteria; Firmicutes; Clostridia; Eubacteriales; Lachnospiraceae; Blautia; Blautia faecicola | Blautia faecicola | Blautia | Lachnospiraceae | Eubacteriales | Clostridia | Firmicutes | Bacteria |
| ASV4164 | 402 | 2 | 1 | 402 | 100 | NR_152688.1 Falcatimonas natans strain WN011 16S ribosomal RNA, partial sequence | NR_152688.1 | 1484 | 365 | 766 | 27.09 | 444.3 | 2E-124 | 349/403 | 86 | 2/403 | 0 | Plus | Plus | Bacteria; Firmicutes; Clostridia; Eubacteriales; Clostridiaceae; Falcatimonas; Falcatimonas natans | Falcatimonas natans | Falcatimonas | Clostridiaceae | Eubacteriales | Clostridia | Firmicutes | Bacteria |
| ASV4165 | 401 | 1 | 1 | 392 | 97.76 | NR_118676.1 Eubacterium xylanophilum strain ATCC 35991 16S ribosomal RNA, partial sequence | NR_118676.1 | 1494 | 387 | 779 | 26.31 | 407.4 | 3E-113 | 335/394 | 85 | 3/394 | 0 | Plus | Plus | Bacteria; Firmicutes; Clostridia; Eubacteriales; Eubacteriaceae; Eubacterium; Eubacterium xylanophilum | Eubacterium xylanophilum | Eubacterium | Eubacteriaceae | Eubacteriales | Clostridia | Firmicutes | Bacteria |
| ASV4167 | 428 | 4 | 3 | 428 | 99.53 | NR_042802.1 Lactobacillus ultunensis strain Kx146C1 16S ribosomal RNA, partial sequence | NR_042802.1 | 1550 | 372 | 796 | 27.42 | 569.9 | 4E-162 | 389/428 | 90 | 5/428 | 1 | Plus | Plus | Bacteria; Firmicutes; Bacilli; Lactobacillales; Lactobacillaceae; Lactobacillus; Lactobacillus ultunensis | Lactobacillus ultunensis | Lactobacillus | Lactobacillaceae | Lactobacillales | Bacilli | Firmicutes | Bacteria |
| ASV4168 | 405 | 2 | 1 | 405 | 100 | NR_144727.1 Anaeromassilibacillus senegalensis strain mt9 16S ribosomal RNA, partial sequence | NR_144727.1 | 1478 | 352 | 753 | 27.2 | 540.3 | 3E-153 | 370/407 | 90 | 7/407 | 1 | Plus | Plus | Bacteria; Firmicutes; Clostridia; Eubacteriales; Oscillospiraceae; Anaeromassilibacillus; Anaeromassilibacillus senegalensis | Anaeromassilibacillus senegalensis | Anaeromassilibacillus | Oscillospiraceae | Eubacteriales | Clostridia | Firmicutes | Bacteria |
| ASV4169 | 404 | 2 | 1 | 404 | 100 | NR_159296.1 Aminipila butyrica strain FH042 16S ribosomal RNA, partial sequence | NR_159296.1 | 1460 | 352 | 755 | 27.67 | 503.4 | 3E-142 | 360/404 | 89 | 0/404 | 0 | Plus | Plus | Bacteria; Firmicutes; Clostridia; Eubacteriales; Clostridiales Family XIII. Incertae Sedis; Aminipila; Aminipila butyrica | Aminipila butyrica | Aminipila | Clostridiales Family XIII. Incertae Sedis | Eubacteriales | Clostridia | Firmicutes | Bacteria |
| ASV4170 | 401 | 1 | 1 | 401 | 100 | NR_137408.1 Terrisporobacter petrolearius strain LAM0A37 16S ribosomal RNA, partial sequence | NR_137408.1 | 1405 | 307 | 707 | 28.54 | 603.1 | 3E-172 | 376/401 | 93 | 0/401 | 0 | Plus | Plus | Bacteria; Firmicutes; Clostridia; Eubacteriales; Peptostreptococcaceae; Terrisporobacter; Terrisporobacter petrolearius | Terrisporobacter petrolearius | Terrisporobacter | Peptostreptococcaceae | Eubacteriales | Clostridia | Firmicutes | Bacteria |
| ASV4173 | 427 | 1 | 3 | 427 | 99.53 | NR_153710.1 Peptococcus simiae strain M108 16S ribosomal RNA, partial sequence | NR_153710.1 | 1488 | 356 | 780 | 28.56 | 592 | 8E-169 | 394/429 | 91 | 8/429 | 1 | Plus | Plus | Bacteria; Firmicutes; Clostridia; Eubacteriales; Peptococcaceae; Peptococcus; Peptococcus simiae | Peptococcus simiae | Peptococcus | Peptococcaceae | Eubacteriales | Clostridia | Firmicutes | Bacteria |
| ASV4174 | 402 | 1 | 1 | 402 | 100 | NR_074652.1 Lachnoclostridium phytofermentans strain ISDg 16S ribosomal RNA, partial sequence | NR_074652.1 | 1523 | 364 | 765 | 26.4 | 533 | 4E-151 | 364/402 | 90 | 0/402 | 0 | Plus | Plus | Bacteria; Firmicutes; Clostridia; Eubacteriales; Lachnospiraceae; Lachnoclostridium; Lachnoclostridium phytofermentans | Lachnoclostridium phytofermentans | Lachnoclostridium | Lachnospiraceae | Eubacteriales | Clostridia | Firmicutes | Bacteria |
| ASV4175 | 402 | 2 | 1 | 402 | 100 | NR_144696.1 Clostridium saudiense strain JCC 16S ribosomal RNA, partial sequence | NR_144696.1 | 1478 | 350 | 751 | 27.2 | 577.3 | 2E-164 | 372/402 | 92 | 0/402 | 0 | Plus | Plus | Bacteria; Firmicutes; Clostridia; Eubacteriales; Clostridiaceae; Clostridium; Clostridium saudiense | Clostridium saudiense | Clostridium | Clostridiaceae | Eubacteriales | Clostridia | Firmicutes | Bacteria |
| ASV4179 | 405 | 1 | 1 | 405 | 100 | NR_145929.1 Caproiciproducens galactitolivorans strain BS-1 16S ribosomal RNA, partial sequence | NR_145929.1 | 1445 | 336 | 741 | 28.1 | 499.7 | 4E-141 | 363/408 | 88 | 5/408 | 1 | Plus | Plus | Bacteria; Firmicutes; Clostridia; Eubacteriales; Oscillospiraceae; Caproiciproducens; Caproiciproducens galactitolivorans | Caproiciproducens galactitolivorans | Caproiciproducens | Oscillospiraceae | Eubacteriales | Clostridia | Firmicutes | Bacteria |
| ASV4180 | 402 | 2 | 1 | 402 | 100 | NR_151894.1 Anaerotaenia torta strain FH052 16S ribosomal RNA, partial sequence | NR_151894.1 | 1479 | 352 | 753 | 27.18 | 610.5 | 2E-174 | 378/402 | 94 | 0/402 | 0 | Plus | Plus | Bacteria; Firmicutes; Clostridia; Eubacteriales; Lachnospiraceae; Anaerotaenia; Anaerotaenia torta | Anaerotaenia torta | Anaerotaenia | Lachnospiraceae | Eubacteriales | Clostridia | Firmicutes | Bacteria |
| ASV4182 | 401 | 1 | 1 | 401 | 100 | NR_042825.1 Paraeggerthella hongkongensis JCM 14552 strain HKU10 16S ribosomal RNA, partial sequence | NR_042825.1 | 1427 | 328 | 729 | 28.17 | 603.1 | 3E-172 | 379/404 | 93 | 5/404 | 1 | Plus | Plus | Bacteria; Actinobacteria; Coriobacteriia; Eggerthellales; Eggerthellaceae; Paraeggerthella; Paraeggerthella hongkongensis | Paraeggerthella hongkongensis | Paraeggerthella | Eggerthellaceae | Eggerthellales | Coriobacteriia | Actinobacteria | Bacteria |
| ASV4183 | 422 | 1 | 1 | 422 | 100 | NR_144704.1 Bacteroides timonensis AP1 16S ribosomal RNA, partial sequence | NR_144704.1 | 1315 | 268 | 689 | 32.09 | 481.2 | 2E-135 | 372/426 | 87 | 8/426 | 1 | Plus | Plus | Bacteria; Bacteroidetes; Bacteroidia; Bacteroidales; Bacteroidaceae; Bacteroides; Bacteroides timonensis | Bacteroides timonensis | Bacteroides | Bacteroidaceae | Bacteroidales | Bacteroidia | Bacteroidetes | Bacteria |
| ASV4184 | 402 | 1 | 1 | 402 | 100 | NR_026112.1 Clostridium thermopalmarium strain BVP 16S ribosomal RNA, partial sequence | NR_026112.1 | 1503 | 346 | 747 | 26.75 | 512.6 | 6E-145 | 360/402 | 89 | 0/402 | 0 | Plus | Plus | Bacteria; Firmicutes; Clostridia; Eubacteriales; Clostridiaceae; Clostridium; Clostridium thermopalmarium | Clostridium thermopalmarium | Clostridium | Clostridiaceae | Eubacteriales | Clostridia | Firmicutes | Bacteria |
| ASV4185 | 403 | 1 | 1 | 403 | 100 | NR_151900.1 Ruthenibacterium lactatiformans strain 585-1 16S ribosomal RNA, partial sequence | NR_151900.1 | 1519 | 368 | 769 | 26.46 | 516.3 | 4E-146 | 365/406 | 89 | 7/406 | 1 | Plus | Plus | Bacteria; Firmicutes; Clostridia; Eubacteriales; Oscillospiraceae; Ruthenibacterium; Ruthenibacterium lactatiformans | Ruthenibacterium lactatiformans | Ruthenibacterium | Oscillospiraceae | Eubacteriales | Clostridia | Firmicutes | Bacteria |
| ASV4186 | 405 | 1 | 1 | 405 | 100 | NR_118667.1 Lachnospira pectinoschiza strain 150-1 16S ribosomal RNA, partial sequence | NR_118667.1 | 1465 | 372 | 773 | 27.44 | 542.2 | 7E-154 | 366/405 | 90 | 3/405 | 0 | Plus | Plus | Bacteria; Firmicutes; Clostridia; Eubacteriales; Lachnospiraceae; Lachnospira; Lachnospira pectinoschiza | Lachnospira pectinoschiza | Lachnospira | Lachnospiraceae | Eubacteriales | Clostridia | Firmicutes | Bacteria |
| ASV4188 | 405 | 2 | 1 | 405 | 100 | NR_117110.1 Desulfovibrio simplex strain DSM 4141 16S ribosomal RNA, partial sequence | NR_117110.1 | 1539 | 354 | 780 | 27.75 | 436.9 | 4E-122 | 367/427 | 85 | 22/427 | 5 | Plus | Plus | Bacteria; Proteobacteria; Deltaproteobacteria; Desulfovibrionales; Desulfovibrionaceae; Desulfovibrio; Desulfovibrio simplex | Desulfovibrio simplex | Desulfovibrio | Desulfovibrionaceae | Desulfovibrionales | Deltaproteobacteria | Proteobacteria | Bacteria |
| ASV4189 | 405 | 1 | 1 | 405 | 100 | NR_159227.1 Monoglobus pectinilyticus strain 14 16S ribosomal RNA, partial sequence | NR_159227.1 | 1493 | 368 | 769 | 26.93 | 523.7 | 3E-148 | 367/407 | 90 | 7/407 | 1 | Plus | Plus | Bacteria; Firmicutes; Clostridia; Eubacteriales; Oscillospiraceae; Monoglobus; Monoglobus pectinilyticus | Monoglobus pectinilyticus | Monoglobus | Oscillospiraceae | Eubacteriales | Clostridia | Firmicutes | Bacteria |
| ASV4190 | 427 | 5 | 1 | 427 | 100 | NR_042111.1 Lactobacillus gallinarum strain ATCC 33199 16S ribosomal RNA, partial sequence | NR_042111.1 | 1546 | 362 | 788 | 27.62 | 712.1 | 0 | 414/428 | 96 | 2/428 | 0 | Plus | Plus | Bacteria; Firmicutes; Bacilli; Lactobacillales; Lactobacillaceae; Lactobacillus; Lactobacillus gallinarum | Lactobacillus gallinarum | Lactobacillus | Lactobacillaceae | Lactobacillales | Bacilli | Firmicutes | Bacteria |
| ASV4191 | 405 | 2 | 1 | 405 | 100 | NG_041947.1 Ruminiclostridium cellulolyticum H10 16S ribosomal RNA gene, complete sequence | NG_041947.1 | 1641 | 486 | 890 | 24.68 | 527.4 | 2E-149 | 366/406 | 90 | 2/406 | 0 | Plus | Plus | Bacteria; Firmicutes; Clostridia; Eubacteriales; Oscillospiraceae; Ruminiclostridium; Ruminiclostridium cellulolyticum | Ruminiclostridium cellulolyticum | Ruminiclostridium | Oscillospiraceae | Eubacteriales | Clostridia | Firmicutes | Bacteria |
| ASV4192 | 405 | 1 | 1 | 405 | 100 | NR_159227.1 Monoglobus pectinilyticus strain 14 16S ribosomal RNA, partial sequence | NR_159227.1 | 1493 | 368 | 769 | 26.93 | 507.1 | 3E-143 | 365/408 | 89 | 9/408 | 2 | Plus | Plus | Bacteria; Firmicutes; Clostridia; Eubacteriales; Oscillospiraceae; Monoglobus; Monoglobus pectinilyticus | Monoglobus pectinilyticus | Monoglobus | Oscillospiraceae | Eubacteriales | Clostridia | Firmicutes | Bacteria |
| ASV4193 | 427 | 1 | 1 | 427 | 100 | NR_042797.1 Treponema berlinense strain 7CPL208 16S ribosomal RNA, partial sequence | NR_042797.1 | 1440 | 275 | 701 | 29.65 | 662.2 | 0 | 404/427 | 94 | 0/427 | 0 | Plus | Plus | Bacteria; Spirochaetes; Spirochaetia; Spirochaetales; Spirochaetaceae; Treponema; Treponema berlinense | Treponema berlinense | Treponema | Spirochaetaceae | Spirochaetales | Spirochaetia | Spirochaetes | Bacteria |
| ASV4194 | 405 | 1 | 1 | 405 | 100 | NR_025025.1 Papillibacter cinnamivorans strain CIN1 16S ribosomal RNA, partial sequence | NR_025025.1 | 1491 | 349 | 751 | 27.03 | 481.2 | 2E-135 | 358/407 | 87 | 6/407 | 1 | Plus | Plus | Bacteria; Firmicutes; Clostridia; Eubacteriales; Oscillospiraceae; Papillibacter; Papillibacter cinnamivorans | Papillibacter cinnamivorans | Papillibacter | Oscillospiraceae | Eubacteriales | Clostridia | Firmicutes | Bacteria |
| ASV4198 | 422 | 1 | 1 | 422 | 100 | NR_144750.1 Prevotellamassilia timonensis strain Marseille-P2831 16S ribosomal RNA, partial sequence | NR_144750.1 | 1493 | 358 | 779 | 28.27 | 553.3 | 4E-157 | 381/422 | 90 | 0/422 | 0 | Plus | Plus | Bacteria; Bacteroidetes; Bacteroidia; Bacteroidales; Prevotellaceae; Prevotellamassilia; Prevotellamassilia timonensis | Prevotellamassilia timonensis | Prevotellamassilia | Prevotellaceae | Bacteroidales | Bacteroidia | Bacteroidetes | Bacteria |
| ASV4199 | 427 | 5 | 15 | 427 | 96.72 | NR_103946.1 Spiroplasma syrphidicola EA-1 16S ribosomal RNA, partial sequence | NR_103946.1 | 1508 | 355 | 766 | 27.32 | 424 | 3E-118 | 353/414 | 85 | 3/414 | 0 | Plus | Plus | Bacteria; Tenericutes; Mollicutes; Entomoplasmatales; Spiroplasmataceae; Spiroplasma; Spiroplasma syrphidicola | Spiroplasma syrphidicola | Spiroplasma | Spiroplasmataceae | Entomoplasmatales | Mollicutes | Tenericutes | Bacteria |
| ASV4201 | 402 | 1 | 1 | 401 | 99.75 | NR_041236.1 Lutispora thermophila DSM 19022 strain EBR46 16S ribosomal RNA, partial sequence | NR_041236.1 | 1438 | 338 | 739 | 27.96 | 497.9 | 2E-140 | 360/404 | 89 | 5/404 | 1 | Plus | Plus | Bacteria; Firmicutes; Clostridia; Eubacteriales; Clostridiaceae; Lutispora; Lutispora thermophila | Lutispora thermophila | Lutispora | Clostridiaceae | Eubacteriales | Clostridia | Firmicutes | Bacteria |
| ASV4202 | 405 | 1 | 1 | 405 | 100 | NR_144611.1 Flintibacter butyricus strain BLS21 16S ribosomal RNA, partial sequence | NR_144611.1 | 808 | 299 | 703 | 50.12 | 505.3 | 1E-142 | 362/406 | 89 | 2/406 | 0 | Plus | Plus | Bacteria; Firmicutes; Clostridia; Eubacteriales; ; Flintibacter; Flintibacter butyricus | Flintibacter butyricus | Flintibacter | | Eubacteriales | Clostridia | Firmicutes | Bacteria |
| ASV4203 | 422 | 1 | 1 | 422 | 100 | NR_041342.1 Parabacteroides distasonis strain JCM 5825 16S ribosomal RNA, partial sequence | NR_041342.1 | 1488 | 353 | 774 | 28.36 | 586.5 | 3E-167 | 387/422 | 91 | 0/422 | 0 | Plus | Plus | Bacteria; Bacteroidetes; Bacteroidia; Bacteroidales; Tannerellaceae; Parabacteroides; Parabacteroides distasonis | Parabacteroides distasonis | Parabacteroides | Tannerellaceae | Bacteroidales | Bacteroidia | Bacteroidetes | Bacteria |
| ASV4204 | 405 | 4 | 1 | 405 | 100 | NR_074399.1 Ruminococcus albus 7 = DSM 20455 16S ribosomal RNA, partial sequence | NR_074399.1 | 1500 | 350 | 751 | 26.8 | 606.8 | 3E-173 | 380/405 | 93 | 3/405 | 0 | Plus | Plus | Bacteria; Firmicutes; Clostridia; Eubacteriales; Oscillospiraceae; Ruminococcus; Ruminococcus albus | Ruminococcus albus | Ruminococcus | Oscillospiraceae | Eubacteriales | Clostridia | Firmicutes | Bacteria |
| ASV4207 | 402 | 1 | 1 | 402 | 100 | NR_104783.1 Acetivibrio ethanolgignens strain 77-6 16S ribosomal RNA, partial sequence | NR_104783.1 | 1529 | 370 | 771 | 26.29 | 411.1 | 2E-114 | 344/404 | 85 | 4/404 | 0 | Plus | Plus | Bacteria; Firmicutes; Clostridia; Eubacteriales; Oscillospiraceae; Acetivibrio; Acetivibrio ethanolgignens | Acetivibrio ethanolgignens | Acetivibrio | Oscillospiraceae | Eubacteriales | Clostridia | Firmicutes | Bacteria |
| ASV4208 | 421 | 2 | 1 | 421 | 100 | NR_113077.1 Paraprevotella clara strain JCM 14859 16S ribosomal RNA, partial sequence | NR_113077.1 | 1480 | 346 | 766 | 28.45 | 490.5 | 3E-138 | 369/421 | 87 | 0/421 | 0 | Plus | Plus | Bacteria; Bacteroidetes; Bacteroidia; Bacteroidales; Prevotellaceae; Paraprevotella; Paraprevotella clara | Paraprevotella clara | Paraprevotella | Prevotellaceae | Bacteroidales | Bacteroidia | Bacteroidetes | Bacteria |
| ASV4209 | 404 | 1 | 3 | 404 | 99.5 | NR_028961.1 Faecalibacterium prausnitzii strain ATCC 27768 16S ribosomal RNA, partial sequence | NR_028961.1 | 1462 | 346 | 745 | 27.36 | 503.4 | 3E-142 | 363/406 | 89 | 10/406 | 2 | Plus | Plus | Bacteria; Firmicutes; Clostridia; Eubacteriales; Oscillospiraceae; Faecalibacterium; Faecalibacterium prausnitzii | Faecalibacterium prausnitzii | Faecalibacterium | Oscillospiraceae | Eubacteriales | Clostridia | Firmicutes | Bacteria |
| ASV4211 | 405 | 2 | 1 | 405 | 100 | NR_147396.1 Lachnoclostridium pacaense strain Marseille-P3100 16S ribosomal RNA, partial sequence | NR_147396.1 | 1453 | 345 | 746 | 27.67 | 557 | 3E-158 | 372/406 | 91 | 5/406 | 1 | Plus | Plus | Bacteria; Firmicutes; Clostridia; Eubacteriales; Lachnospiraceae; Lachnoclostridium; Lachnoclostridium pacaense | Lachnoclostridium pacaense | Lachnoclostridium | Lachnospiraceae | Eubacteriales | Clostridia | Firmicutes | Bacteria |
| ASV4212 | 405 | 1 | 1 | 405 | 100 | NR_028961.1 Faecalibacterium prausnitzii strain ATCC 27768 16S ribosomal RNA, partial sequence | NR_028961.1 | 1462 | 344 | 745 | 27.5 | 606.8 | 3E-173 | 380/405 | 93 | 3/405 | 0 | Plus | Plus | Bacteria; Firmicutes; Clostridia; Eubacteriales; Oscillospiraceae; Faecalibacterium; Faecalibacterium prausnitzii | Faecalibacterium prausnitzii | Faecalibacterium | Oscillospiraceae | Eubacteriales | Clostridia | Firmicutes | Bacteria |
| ASV4217 | 403 | 5 | 1 | 403 | 100 | NR_115693.1 Gracilibacter thermotolerans JW/YJL-S1 16S ribosomal RNA, partial sequence | NR_115693.1 | 1526 | 459 | 861 | 26.41 | 518.2 | 1E-146 | 362/403 | 89 | 0/403 | 0 | Plus | Plus | Bacteria; Firmicutes; Clostridia; Eubacteriales; Gracilibacteraceae; Gracilibacter; Gracilibacter thermotolerans | Gracilibacter thermotolerans | Gracilibacter | Gracilibacteraceae | Eubacteriales | Clostridia | Firmicutes | Bacteria |
| ASV4218 | 425 | 1 | 1 | 425 | 100 | NR_029325.1 Schwartzia succinivorans strain S1-1 16S ribosomal RNA, partial sequence | NR_029325.1 | 1489 | 344 | 769 | 28.61 | 464.6 | 2E-130 | 372/430 | 86 | 9/430 | 2 | Plus | Plus | Bacteria; Firmicutes; Negativicutes; Selenomonadales; Selenomonadaceae; Schwartzia; Schwartzia succinivorans | Schwartzia succinivorans | Schwartzia | Selenomonadaceae | Selenomonadales | Negativicutes | Firmicutes | Bacteria |
| ASV4220 | 422 | 1 | 1 | 422 | 100 | NR_147368.1 Millionella massiliensis strain Marseille-P3215 16S ribosomal RNA, partial sequence | NR_147368.1 | 1492 | 355 | 776 | 28.28 | 472 | 1E-132 | 367/423 | 86 | 2/423 | 0 | Plus | Plus | Bacteria; Bacteroidetes; Bacteroidia; Bacteroidales; Rikenellaceae; Millionella; Millionella massiliensis | Millionella massiliensis | Millionella | Rikenellaceae | Bacteroidales | Bacteroidia | Bacteroidetes | Bacteria |

| | | | | | | | | | | | | | | | | | | | | | | |
|---|---|---|---|---|---|---|---|---|---|---|---|---|---|---|---|---|---|---|---|---|---|---|
| ASV4221 | 427 | 1 | 1 | 427 | 100 | NR_104990.1 | Desulfovibrio desulfuricans strain Essex 6 16S ribosomal RNA, partial sequence | NR_104990.1 | 1542 | 357 | 783 | 27.69 | 595.7 | 6E-170 | 394/429 | 91 | 4/429 | 0 | Plus | Plus | Bacteria; Proteobacteria; Deltaproteobacteria; Desulfovibrionales; Desulfovibrionaceae; Desulfovibrio; Desulfovibrio desulfuricans | Desulfovibrio desulfuricans |
| ASV4222 | 402 | 1 | 1 | 402 | 100 | NR_114789.1 | [Clostridium] leptum strain DSM 753 16S ribosomal RNA, partial sequence | NR_114789.1 | 1505 | 364 | 765 | 26.71 | 560.7 | 2E-159 | 369/402 | 91 | 0/402 | 0 | Plus | Plus | Bacteria; Firmicutes; Clostridia; Eubacteriales; Oscillospiraceae; ; [Clostridium] leptum | [Clostridium] leptum |
| ASV4224 | 422 | 1 | 1 | 422 | 100 | NR_117198.1 | Alkaliflexus imshenetskii strain Z-7010 16S ribosomal RNA, partial sequence | NR_117198.1 | 1515 | 350 | 772 | 27.92 | 444.3 | 2E-124 | 365/426 | 85 | 7/426 | 1 | Plus | Plus | Bacteria; Bacteroidetes; Bacteroidia; Marinilabiliales; Marinilabiliaceae; Alkaliflexus; Alkaliflexus imshenetskii | Alkaliflexus imshenetskii |
| ASV4225 | 401 | 1 | 1 | 401 | 100 | NR_158111.1 | Paludicola psychrotolerans strain NC1253 16S ribosomal RNA, partial sequence | NR_158111.1 | 1490 | 366 | 766 | 26.91 | 641.9 | 0 | 383/401 | 95 | 0/401 | 0 | Plus | Plus | Bacteria; Firmicutes; Clostridia; Eubacteriales; Oscillospiraceae; Paludicola; Paludicola psychrotolerans | Paludicola psychrotolerans |
| ASV4226 | 421 | 5 | 1 | 421 | 100 | NR_116726.1 | Prevotella scopos JCM 17725 strain W2052 16S ribosomal RNA, partial sequence | NR_116726.1 | 1453 | 338 | 759 | 29.04 | 440.6 | 3E-123 | 362/423 | 85 | 3/423 | 0 | Plus | Plus | Bacteria; Bacteroidetes; Bacteroidia; Bacteroidales; Prevotellaceae; Prevotella; Prevotella scopos | Prevotella scopos |
| ASV4227 | 427 | 1 | 1 | 427 | 100 | NR_137408.1 | Terrisporobacter petrolearius strain LAM0A37 16S ribosomal RNA, partial sequence | NR_137408.1 | 1405 | 307 | 707 | 28.54 | 494.2 | 2E-139 | 378/427 | 88 | 26/427 | 6 | Plus | Plus | Bacteria; Firmicutes; Clostridia; Eubacteriales; Peptostreptococcaceae; Terrisporobacter; Terrisporobacter petrolearius | Terrisporobacter petrolearius |
| ASV4228 | 421 | 1 | 1 | 421 | 100 | NR_144616.1 | Muribaculum intestinale strain YL27 16S ribosomal RNA, partial sequence | NR_144616.1 | 1460 | 297 | 718 | 28.9 | 490.5 | 3E-138 | 371/423 | 87 | 3/423 | 0 | Plus | Plus | Bacteria; Bacteroidetes; Bacteroidia; Bacteroidales; Muribaculaceae; Muribaculum; Muribaculum intestinale | Muribaculum intestinale |
| ASV4229 | 401 | 1 | 1 | 399 | 99.5 | NR_104737.1 | Eubacterium oxidoreducens strain G41 16S ribosomal RNA, partial sequence | NR_104737.1 | 1521 | 363 | 762 | 26.3 | 444.3 | 2E-124 | 349/402 | 86 | 5/402 | 1 | Plus | Plus | Bacteria; Firmicutes; Clostridia; Eubacteriales; Eubacteriaceae; Eubacterium; Eubacterium oxidoreducens | Eubacterium oxidoreducens |
| ASV4230 | 401 | 1 | 1 | 401 | 100 | NR_152059.1 | Harryflintia acetispora strain V20-281a 16S ribosomal RNA, partial sequence | NR_152059.1 | 1363 | 289 | 692 | 29.64 | 605 | 9E-173 | 379/404 | 93 | 3/404 | 0 | Plus | Plus | Bacteria; Firmicutes; Clostridia; Eubacteriales; Oscillospiraceae; Harryflintia; Harryflintia acetispora | Harryflintia acetispora |
| ASV4232 | 427 | 1 | 1 | 427 | 100 | NR_041666.1 | Dialister succinatiphilus YIT 11850 16S ribosomal RNA, partial sequence | NR_041666.1 | 1521 | 353 | 779 | 28.07 | 629 | 6E-180 | 400/429 | 93 | 4/429 | 0 | Plus | Plus | Bacteria; Firmicutes; Negativicutes; Veillonellales; Veillonellaceae; Dialister; Dialister succinatiphilus | Dialister succinatiphilus |
| ASV4233 | 427 | 1 | 1 | 422 | 98.83 | NR_028840.1 | Mitsuokella jalaludinii strain M9 16S ribosomal RNA, partial sequence | NR_028840.1 | 1543 | 362 | 783 | 27.35 | 686.2 | 0 | 405/422 | 96 | 0/422 | 0 | Plus | Plus | Bacteria; Firmicutes; Negativicutes; Selenomonadales; Selenomonadaceae; Mitsuokella; Mitsuokella jalaludinii | Mitsuokella jalaludinii |
| ASV4235 | 401 | 1 | 1 | 401 | 100 | NR_119085.1 | [Clostridium] polysaccharolyticum strain DSM 1801 16S ribosomal RNA, partial sequence | NR_119085.1 | 1503 | 364 | 763 | 26.61 | 418.5 | 1E-116 | 345/403 | 85 | 5/403 | 1 | Plus | Plus | Bacteria; Firmicutes; Clostridia; Eubacteriales; Lachnospiraceae; Lachnoclostridium; [Clostridium] polysaccharolyticum | [Clostridium] polysaccharolyticum |
| ASV4236 | 402 | 4 | 1 | 402 | 100 | NR_156080.1 | Kineothrix alysoides strain KNHs209 16S ribosomal RNA, partial sequence | NR_156080.1 | 1410 | 353 | 754 | 28.51 | 460.9 | 2E-129 | 351/402 | 87 | 0/402 | 0 | Plus | Plus | Bacteria; Firmicutes; Clostridia; Eubacteriales; Lachnospiraceae; Kineothrix; Kineothrix alysoides | Kineothrix alysoides |
| ASV4237 | 421 | 1 | 1 | 421 | 100 | NR_144616.1 | Muribaculum intestinale strain YL27 16S ribosomal RNA, partial sequence | NR_144616.1 | 1460 | 297 | 718 | 28.9 | 507.1 | 3E-143 | 379/428 | 88 | 13/428 | 3 | Plus | Plus | Bacteria; Bacteroidetes; Bacteroidia; Bacteroidales; Muribaculaceae; Muribaculum; Muribaculum intestinale | Muribaculum intestinale |
| ASV4238 | 402 | 1 | 3 | 402 | 99.5 | NR_144611.1 | Flintibacter butyricus strain BLS21 16S ribosomal RNA, partial sequence | NR_144611.1 | 808 | 301 | 703 | 49.88 | 464.6 | 2E-130 | 353/403 | 87 | 3/403 | 0 | Plus | Plus | Bacteria; Firmicutes; Clostridia; Eubacteriales; ; Flintibacter; Flintibacter butyricus | Flintibacter butyricus |
| ASV4239 | 405 | 2 | 1 | 405 | 100 | NR_148620.1 | Lachnotalea glycerini strain : DLD10 16S ribosomal RNA, partial sequence | NR_148620.1 | 1207 | 212 | 613 | 33.31 | 496 | 6E-140 | 364/409 | 88 | 11/409 | 2 | Plus | Plus | Bacteria; Firmicutes; Clostridia; Eubacteriales; Lachnospiraceae; Lachnotalea; Lachnotalea glycerini | Lachnotalea glycerini |
| ASV4240 | 427 | 3 | 1 | 425 | 99.53 | NR_041781.1 | Streptococcus alactolyticus strain ATCC 43077 16S ribosomal RNA, partial sequence | NR_041781.1 | 1437 | 346 | 770 | 29.58 | 708.4 | 0 | 412/426 | 96 | 2/426 | 0 | Plus | Plus | Bacteria; Firmicutes; Bacilli; Lactobacillales; Streptococcaceae; Streptococcus; Streptococcus alactolyticus | Streptococcus alactolyticus |
| ASV4241 | 422 | 3 | 1 | 422 | 100 | NR_113110.1 | Prevotella maculosa strain JCM 15638 16S ribosomal RNA, partial sequence | NR_113110.1 | 1405 | 269 | 690 | 30.04 | 658.5 | 0 | 400/422 | 94 | 0/422 | 0 | Plus | Plus | Bacteria; Bacteroidetes; Bacteroidia; Bacteroidales; Prevotellaceae; Prevotella; Prevotella maculosa | Prevotella maculosa |
| ASV4242 | 427 | 3 | 1 | 425 | 99.53 | NR_041781.1 | Streptococcus alactolyticus strain ATCC 43077 16S ribosomal RNA, partial sequence | NR_041781.1 | 1437 | 346 | 770 | 29.58 | 736.1 | 0 | 416/425 | 97 | 0/425 | 0 | Plus | Plus | Bacteria; Firmicutes; Bacilli; Lactobacillales; Streptococcaceae; Streptococcus; Streptococcus alactolyticus | Streptococcus alactolyticus |
| ASV4244 | 401 | 1 | 1 | 401 | 100 | NR_137408.1 | Terrisporobacter petrolearius strain LAM0A37 16S ribosomal RNA, partial sequence | NR_137408.1 | 1405 | 307 | 707 | 28.54 | 592 | 7E-169 | 374/401 | 93 | 0/401 | 0 | Plus | Plus | Bacteria; Firmicutes; Clostridia; Eubacteriales; Peptostreptococcaceae; Terrisporobacter; Terrisporobacter petrolearius | Terrisporobacter petrolearius |
| ASV4245 | 405 | 1 | 1 | 405 | 100 | NR_159227.1 | Monoglobus pectinilyticus strain 14 16S ribosomal RNA, partial sequence | NR_159227.1 | 1493 | 368 | 769 | 26.93 | 507.1 | 3E-143 | 364/407 | 89 | 7/407 | 1 | Plus | Plus | Bacteria; Firmicutes; Clostridia; Eubacteriales; Oscillospiraceae; Monoglobus; Monoglobus pectinilyticus | Monoglobus pectinilyticus |
| ASV4246 | 409 | 2 | 1 | 408 | 99.76 | NR_134688.1 | Actinomadura rayongensis strain RY35-68 16S ribosomal RNA, partial sequence | NR_134688.1 | 1418 | 316 | 721 | 28.63 | 442.5 | 8E-124 | 357/413 | 86 | 12/413 | 2 | Plus | Plus | Bacteria; Actinobacteria; Actinomycetia; Streptosporangiales; Thermomonosporaceae; Actinomadura; Actinomadura rayongensis | Actinomadura rayongensis |
| ASV4247 | 422 | 1 | 1 | 422 | 100 | NR_144611.1 | Flintibacter butyricus strain BLS21 16S ribosomal RNA, partial sequence | NR_144611.1 | 808 | 299 | 703 | 50.12 | 420.3 | 4E-117 | 360/422 | 85 | 17/422 | 4 | Plus | Plus | Bacteria; Firmicutes; Clostridia; Eubacteriales; ; Flintibacter; Flintibacter butyricus | Flintibacter butyricus |
| ASV4249 | 404 | 1 | 1 | 404 | 100 | NR_113173.1 | Bifidobacterium saguini DSM 23967 strain AF823-1 16S ribosomal RNA, partial sequence | NR_113173.1 | 1433 | 341 | 748 | 28.47 | 592 | 7E-169 | 380/409 | 92 | 6/409 | 1 | Plus | Plus | Bacteria; Actinobacteria; Actinomycetia; Bifidobacteriales; Bifidobacteriaceae; Bifidobacterium; Bifidobacterium saguini | Bifidobacterium saguini |
| ASV4250 | 427 | 1 | 1 | 426 | 99.77 | NR_044111.1 | Selenomonas bovis strain WG 16S ribosomal RNA, partial sequence | NR_044111.1 | 1525 | 370 | 795 | 27.93 | 732.4 | 0 | 416/426 | 97 | 0/426 | 0 | Plus | Plus | Bacteria; Firmicutes; Negativicutes; Selenomonadales; Selenomonadaceae; Selenomonas; Selenomonas bovis | Selenomonas bovis |
| ASV4253 | 404 | 1 | 3 | 404 | 99.5 | NR_029097.1 | Oribacterium sinus strain AIP 354.02 16S ribosomal RNA, partial sequence | NR_029097.1 | 1374 | 310 | 709 | 29.11 | 514.5 | 2E-145 | 361/402 | 89 | 2/402 | 0 | Plus | Plus | Bacteria; Firmicutes; Clostridia; Eubacteriales; Lachnospiraceae; Oribacterium; Oribacterium sinus | Oribacterium sinus |
| ASV4254 | 422 | 1 | 1 | 422 | 100 | NR_113411.1 | Prevotella copri DSM 18205 strain JCM 13464 16S ribosomal RNA, partial sequence | NR_113411.1 | 1493 | 358 | 779 | 28.27 | 658.5 | 0 | 401/423 | 94 | 2/423 | 0 | Plus | Plus | Bacteria; Bacteroidetes; Bacteroidia; Bacteroidales; Prevotellaceae; Prevotella; Prevotella copri | Prevotella copri |
| ASV4255 | 422 | 1 | 1 | 422 | 100 | NR_113411.1 | Prevotella copri DSM 18205 strain JCM 13464 16S ribosomal RNA, partial sequence | NR_113411.1 | 1493 | 358 | 779 | 28.27 | 636.4 | 0 | 397/423 | 93 | 2/423 | 0 | Plus | Plus | Bacteria; Bacteroidetes; Bacteroidia; Bacteroidales; Prevotellaceae; Prevotella; Prevotella copri | Prevotella copri |
| ASV4256 | 422 | 1 | 1 | 422 | 100 | NR_113411.1 | Prevotella copri DSM 18205 strain JCM 13464 16S ribosomal RNA, partial sequence | NR_113411.1 | 1493 | 358 | 779 | 28.27 | 675.1 | 0 | 404/423 | 95 | 2/423 | 0 | Plus | Plus | Bacteria; Bacteroidetes; Bacteroidia; Bacteroidales; Prevotellaceae; Prevotella; Prevotella copri | Prevotella copri |
| ASV4259 | 427 | 1 | 1 | 412 | 96.49 | NR_044111.1 | Selenomonas bovis strain WG 16S ribosomal RNA, partial sequence | NR_044111.1 | 1525 | 370 | 781 | 27.02 | 557 | 3E-158 | 375/412 | 91 | 0/412 | 0 | Plus | Plus | Bacteria; Firmicutes; Negativicutes; Selenomonadales; Selenomonadaceae; Selenomonas; Selenomonas bovis | Selenomonas bovis |
| ASV4260 | 402 | 1 | 1 | 402 | 100 | NR_118675.1 | [Eubacterium] siraeum strain ATCC 29066 16S ribosomal RNA, partial sequence | NR_118675.1 | 1453 | 356 | 757 | 27.67 | 557 | 3E-158 | 367/402 | 91 | 0/402 | 0 | Plus | Plus | Bacteria; Firmicutes; Clostridia; Eubacteriales; Oscillospiraceae; ; [Eubacterium] siraeum | [Eubacterium] siraeum |
| ASV4264 | 403 | 1 | 1 | 402 | 99.75 | NR_042834.1 | Alkalibacter saccharofermentans strain Z-79820 16S ribosomal RNA, partial sequence | NR_042834.1 | 1465 | 338 | 740 | 27.51 | 483.1 | 4E-136 | 358/405 | 88 | 5/405 | 1 | Plus | Plus | Bacteria; Firmicutes; Clostridia; Eubacteriales; Eubacteriaceae; Alkalibacter; Alkalibacter saccharofermentans | Alkalibacter saccharofermentans |
| ASV4266 | 402 | 1 | 1 | 402 | 100 | NR_144617.1 | Muricomes intestini strain 2PG-424-CC-1 16S ribosomal RNA, partial sequence | NR_144617.1 | 1431 | 357 | 758 | 28.09 | 732.4 | 0 | 400/402 | 99 | 0/402 | 0 | Plus | Plus | Bacteria; Firmicutes; Clostridia; Eubacteriales; Lachnospiraceae; Muricomes; Muricomes intestini | Muricomes intestini |
| ASV4267 | 427 | 2 | 1 | 427 | 100 | NR_112903.1 | Parasutterella secunda strain YIT 12071 16S ribosomal RNA, partial sequence | NR_112903.1 | 1485 | 329 | 755 | 28.75 | 673.3 | 0 | 408/429 | 95 | 4/429 | 0 | Plus | Plus | Bacteria; Proteobacteria; Betaproteobacteria; Burkholderiales; Sutterellaceae; Parasutterella; Parasutterella secunda | Parasutterella secunda |
| ASV4268 | 405 | 1 | 1 | 405 | 100 | NR_159227.1 | Monoglobus pectinilyticus strain 14 16S ribosomal RNA, partial sequence | NR_159227.1 | 1493 | 368 | 769 | 26.93 | 568 | 1E-161 | 375/407 | 92 | 7/407 | 1 | Plus | Plus | Bacteria; Firmicutes; Clostridia; Eubacteriales; Oscillospiraceae; Monoglobus; Monoglobus pectinilyticus | Monoglobus pectinilyticus |
| ASV4269 | 402 | 2 | 1 | 402 | 100 | NR_029239.3 | Clostridium chartatabidum strain 163 16S ribosomal RNA, partial sequence | NR_029239.3 | 1460 | 323 | 724 | 27.53 | 527.4 | 2E-149 | 364/403 | 90 | 2/403 | 0 | Plus | Plus | Bacteria; Firmicutes; Clostridia; Eubacteriales; Clostridiaceae; Clostridium; Clostridium chartatabidum | Clostridium chartatabidum |
| ASV4273 | 404 | 1 | 1 | 404 | 100 | NR_029239.3 | Clostridium chartatabidum strain 163 16S ribosomal RNA, partial sequence | NR_029239.3 | 1460 | 323 | 724 | 27.53 | 590.2 | 3E-168 | 378/406 | 93 | 6/406 | 1 | Plus | Plus | Bacteria; Firmicutes; Clostridia; Eubacteriales; Clostridiaceae; Clostridium; Clostridium chartatabidum | Clostridium chartatabidum |
| ASV4274 | 401 | 1 | 3 | 401 | 99.5 | NR_029167.1 | Anaeroplasma abactoclasticum strain 6-1 16S ribosomal RNA, partial sequence | NR_029167.1 | 1446 | 346 | 751 | 28.08 | 473.9 | 3E-133 | 360/409 | 88 | 13/409 | 3 | Plus | Plus | Bacteria; Tenericutes; Mollicutes; Anaeroplasmatales; Anaeroplasmataceae; Anaeroplasma; Anaeroplasma abactoclasticum | Anaeroplasma abactoclasticum |
| ASV4275 | 402 | 1 | 1 | 402 | 100 | NR_044048.1 | Coprococcus comes ATCC 27758 16S ribosomal RNA, partial sequence | NR_044048.1 | 1453 | 345 | 746 | 27.67 | 560.7 | 2E-159 | 370/403 | 91 | 2/403 | 0 | Plus | Plus | Bacteria; Firmicutes; Clostridia; Eubacteriales; Lachnospiraceae; Coprococcus; Coprococcus comes | Coprococcus comes |
| ASV4276 | 405 | 1 | 3 | 405 | 99.51 | NR_026476.1 | Succinivibrio dextrinosolvens strain 0554 16S ribosomal RNA, partial sequence | NR_026476.1 | 1463 | 333 | 732 | 27.34 | 531.1 | 2E-150 | 365/403 | 90 | 3/403 | 0 | Plus | Plus | Bacteria; Proteobacteria; Gammaproteobacteria; Aeromonadales; Succinivibrionaceae; Succinivibrio; Succinivibrio dextrinosolvens | Succinivibrio dextrinosolvens |
| ASV4277 | 427 | 1 | 4 | 427 | 99.3 | NR_146687.1 | Breznakia pachnodae strain Pei061 16S ribosomal RNA, partial sequence | NR_146687.1 | 1499 | 370 | 792 | 28.22 | 444.3 | 2E-124 | 367/428 | 85 | 9/428 | 2 | Plus | Plus | Bacteria; Firmicutes; Erysipelotrichia; Erysipelotrichales; Erysipelotrichaceae; Breznakia; Breznakia pachnodae | Breznakia pachnodae |
| ASV4278 | 423 | 2 | 1 | 423 | 100 | NR_113411.1 | Prevotella copri DSM 18205 strain JCM 13464 16S ribosomal RNA, partial sequence | NR_113411.1 | 1493 | 358 | 779 | 28.27 | 636.4 | 0 | 399/425 | 93 | 5/425 | 1 | Plus | Plus | Bacteria; Bacteroidetes; Bacteroidia; Bacteroidales; Prevotellaceae; Prevotella; Prevotella copri | Prevotella copri |
| ASV4279 | 421 | 2 | 1 | 421 | 100 | NR_113077.1 | Paraprevotella clara strain JCM 14859 16S ribosomal RNA, partial sequence | NR_113077.1 | 1480 | 346 | 766 | 28.45 | 473.9 | 3E-133 | 367/422 | 86 | 2/422 | 0 | Plus | Plus | Bacteria; Bacteroidetes; Bacteroidia; Bacteroidales; Prevotellaceae; Paraprevotella; Paraprevotella clara | Paraprevotella clara |
| ASV4281 | 401 | 1 | 1 | 401 | 100 | NR_144749.1 | Ihubacter massiliensis strain Marseille-P2843 16S ribosomal RNA, partial sequence | NR_144749.1 | 1479 | 364 | 766 | 27.25 | 521.9 | 9E-148 | 365/405 | 90 | 6/405 | 1 | Plus | Plus | Bacteria; Firmicutes; Clostridia; Eubacteriales; Clostridiales Family XIII. Incertae Sedis; Ihubacter; Ihubacter massiliensis | Ihubacter massiliensis |
| ASV4283 | 405 | 2 | 4 | 405 | 99.26 | NR_024948.1 | Campylobacter hyointestinalis subsp. lawsonii strain CHY5 16S ribosomal RNA, partial sequence | NR_024948.1 | 1419 | 321 | 720 | 28.19 | 542.2 | 7E-154 | 371/407 | 91 | 12/407 | 2 | Plus | Plus | Bacteria; Proteobacteria; Epsilonproteobacteria; Campylobacterales; Campylobacteraceae; Campylobacter; Campylobacter hyointestinalis | Campylobacter hyointestinalis |
| ASV4284 | 402 | 1 | 3 | 402 | 99.5 | NR_026476.1 | Succinivibrio dextrinosolvens strain 0554 16S ribosomal RNA, partial sequence | NR_026476.1 | 1463 | 333 | 732 | 27.34 | 540.3 | 3E-153 | 366/402 | 91 | 4/402 | 0 | Plus | Plus | Bacteria; Proteobacteria; Gammaproteobacteria; Aeromonadales; Succinivibrionaceae; Succinivibrio; Succinivibrio dextrinosolvens | Succinivibrio dextrinosolvens |
| ASV4288 | 422 | 1 | 1 | 422 | 100 | NR_113411.1 | Prevotella copri DSM 18205 strain JCM 13464 16S ribosomal RNA, partial sequence | NR_113411.1 | 1493 | 358 | 779 | 28.27 | 636.4 | 0 | 397/423 | 93 | 2/423 | 0 | Plus | Plus | Bacteria; Bacteroidetes; Bacteroidia; Bacteroidales; Prevotellaceae; Prevotella; Prevotella copri | Prevotella copri |
| ASV4290 | 402 | 1 | 3 | 402 | 99.5 | NR_026476.1 | Succinivibrio dextrinosolvens strain 0554 16S ribosomal RNA, partial sequence | NR_026476.1 | 1463 | 333 | 732 | 27.34 | 507.1 | 3E-143 | 360/402 | 89 | 4/402 | 0 | Plus | Plus | Bacteria; Proteobacteria; Gammaproteobacteria; Aeromonadales; Succinivibrionaceae; Succinivibrio; Succinivibrio dextrinosolvens | Succinivibrio dextrinosolvens |
| ASV4295 | 404 | 1 | 1 | 404 | 100 | NR_109636.1 | Cloacibacillus porcorum strain CL-84 16S ribosomal RNA, partial sequence | NR_109636.1 | 1447 | 320 | 721 | 27.78 | 512.6 | 6E-145 | 362/404 | 89 | 2/404 | 0 | Plus | Plus | Bacteria; Synergistetes; Synergistia; Synergistales; Synergistaceae; Cloacibacillus; Cloacibacillus porcorum | Cloacibacillus porcorum |
| ASV4296 | 423 | 1 | 1 | 423 | 100 | NR_144750.1 | Prevotellamassilia timonensis strain Marseille-P2831 16S ribosomal RNA, partial sequence | NR_144750.1 | 1493 | 358 | 779 | 28.27 | 542.2 | 8E-154 | 380/423 | 89 | 1/423 | 0 | Plus | Plus | Bacteria; Bacteroidetes; Bacteroidia; Bacteroidales; Prevotellaceae; Prevotellamassilia; Prevotellamassilia timonensis | Prevotellamassilia timonensis |
| ASV4298 | 406 | 1 | 3 | 406 | 99.51 | NR_026102.1 | Ruminiclostridium papyrosolvens DSM 2782 16S ribosomal RNA, partial sequence | NR_026102.1 | 1491 | 332 | 733 | 26.96 | 490.5 | 3E-138 | 361/407 | 88 | 8/407 | 1 | Plus | Plus | Bacteria; Firmicutes; Clostridia; Eubacteriales; Oscillospiraceae; Ruminiclostridium; Ruminiclostridium papyrosolvens | Ruminiclostridium papyrosolvens |
| ASV4300 | 404 | 1 | 1 | 404 | 100 | NR_028911.1 | Anaerovorax odorimutans strain NorPut 16S ribosomal RNA, partial sequence | NR_028911.1 | 1488 | 352 | 755 | 27.15 | 542.2 | 7E-154 | 368/405 | 90 | 2/405 | 0 | Plus | Plus | Bacteria; Firmicutes; Clostridia; Eubacteriales; Clostridiales Family XIII. Incertae Sedis; Anaerovorax; Anaerovorax odorimutans | Anaerovorax odorimutans |
| ASV4301 | 403 | 1 | 1 | 394 | 97.77 | NR_104907.1 | Eubacterium coprostanoligenes strain HL 16S ribosomal RNA, partial sequence | NR_104907.1 | 1446 | 332 | 725 | 27.25 | 451.7 | 1E-126 | 345/395 | 87 | 2/395 | 0 | Plus | Plus | Bacteria; Firmicutes; Clostridia; Eubacteriales; Eubacteriaceae; Eubacterium; Eubacterium coprostanoligenes | Eubacterium coprostanoligenes |
| ASV4302 | 402 | 1 | 1 | 402 | 100 | NR_117867.1 | Blautia stercoris strain GAM6-1 16S ribosomal RNA, partial sequence | NR_117867.1 | 1372 | 317 | 718 | 29.3 | 538.5 | 9E-153 | 365/402 | 90 | 0/402 | 0 | Plus | Plus | Bacteria; Firmicutes; Clostridia; Eubacteriales; Lachnospiraceae; Blautia; Blautia stercoris | Blautia stercoris |
| ASV4305 | 402 | 2 | 1 | 401 | 99.75 | NR_152095.1 | Herbinix luporum strain SD1D 16S ribosomal RNA, partial sequence | NR_152095.1 | 1521 | 365 | 765 | 26.36 | 525.6 | 7E-149 | 364/403 | 90 | 4/403 | 0 | Plus | Plus | Bacteria; Firmicutes; Clostridia; Eubacteriales; Lachnospiraceae; Herbinix; Herbinix luporum | Herbinix luporum |
| ASV4307 | 427 | 1 | 1 | 427 | 100 | NR_028862.1 | Allisonella histaminiformans strain MR2 16S ribosomal RNA, partial sequence | NR_028862.1 | 1537 | 376 | 802 | 27.78 | 745.3 | 0 | 419/427 | 98 | 0/427 | 0 | Plus | Plus | Bacteria; Firmicutes; Negativicutes; Veillonellales; Veillonellaceae; Allisonella; Allisonella histaminiformans | Allisonella histaminiformans |
| ASV4310 | 426 | 1 | 3 | 426 | 99.53 | NR_041304.1 | Sharpea azabuensis strain ST18 16S ribosomal RNA, partial sequence | NR_041304.1 | 1460 | 327 | 750 | 29.04 | 595.7 | 6E-170 | 390/424 | 91 | 0/424 | 0 | Plus | Plus | Bacteria; Firmicutes; Erysipelotrichia; Erysipelotrichales; Coprobacillaceae; Sharpea; Sharpea azabuensis | Sharpea azabuensis |
| ASV4312 | 404 | 1 | 1 | 404 | 100 | NR_118156.1 | Oscillibacter ruminantium GH1 16S ribosomal RNA, partial sequence | NR_118156.1 | 1409 | 321 | 724 | 28.67 | 641.9 | 0 | 385/404 | 95 | 0/404 | 0 | Plus | Plus | Bacteria; Firmicutes; Clostridia; Eubacteriales; Oscillospiraceae; Oscillibacter; Oscillibacter ruminantium | Oscillibacter ruminantium |
| ASV4313 | 407 | 1 | 1 | 407 | 100 | NR_025310.1 | Rothia nasimurium strain CCUG 35957 16S ribosomal RNA, partial sequence | NR_025310.1 | 1481 | 332 | 738 | 27.48 | 752.7 | 0 | 407/407 | 100 | 0/407 | 0 | Plus | Plus | Bacteria; Actinobacteria; Actinomycetia; Micrococcales; Micrococcaceae; Rothia; Rothia nasimurium | Rothia nasimurium |
| ASV4317 | 402 | 1 | 1 | 402 | 100 | NR_025931.1 | Ruminococcus flavefaciens strain C94 16S ribosomal RNA, partial sequence | NR_025931.1 | 1388 | 325 | 726 | 28.96 | 538.5 | 9E-153 | 365/402 | 90 | 0/402 | 0 | Plus | Plus | Bacteria; Firmicutes; Clostridia; Eubacteriales; Oscillospiraceae; Ruminococcus; Ruminococcus flavefaciens | Ruminococcus flavefaciens |
| ASV4323 | 421 | 1 | 1 | 421 | 100 | NR_146018.1 | Paludibacter jiangxiensis strain NM7 16S ribosomal RNA, partial sequence | NR_146018.1 | 1469 | 340 | 761 | 28.73 | 429.5 | 6E-120 | 361/424 | 85 | 5/424 | 1 | Plus | Plus | Bacteria; Bacteroidetes; Bacteroidia; Bacteroidales; Paludibacteraceae; Paludibacter; Paludibacter jiangxiensis | Paludibacter jiangxiensis |
| ASV4326 | 427 | 4 | 1 | 427 | 100 | NR_163661.1 | Herbaspirillum robiniae strain HZ10 16S ribosomal RNA, partial sequence | NR_163661.1 | 1405 | 296 | 722 | 30.39 | 634.5 | 0 | 399/427 | 93 | 0/427 | 0 | Plus | Plus | Bacteria; Proteobacteria; Betaproteobacteria; Burkholderiales; Oxalobacteraceae; Herbaspirillum; Herbaspirillum robiniae | Herbaspirillum robiniae |
| ASV4327 | 403 | 1 | 1 | 403 | 100 | NR_102987.1 | Hungateiclostridium clariflavum DSM 19732 16S ribosomal RNA, partial sequence | NR_102987.1 | 1634 | 481 | 882 | 24.6 | 621.6 | 9E-178 | 381/403 | 94 | 1/403 | 0 | Plus | Plus | Bacteria; Firmicutes; Clostridia; Eubacteriales; Oscillospiraceae; Acetivibrio; Acetivibrio clariflavus | Acetivibrio clariflavus |
| ASV4328 | 421 | 1 | 1 | 421 | 100 | NR_144616.1 | Muribaculum intestinale strain YL27 16S ribosomal RNA, partial sequence | NR_144616.1 | 1460 | 297 | 718 | 28.9 | 473.9 | 3E-133 | 372/427 | 87 | 11/427 | 2 | Plus | Plus | Bacteria; Bacteroidetes; Bacteroidia; Bacteroidales; Muribaculaceae; Muribaculum; Muribaculum intestinale | Muribaculum intestinale |
| ASV4331 | 403 | 1 | 1 | 403 | 100 | NR_118156.1 | Oscillibacter ruminantium GH1 16S ribosomal RNA, partial sequence | NR_118156.1 | 1409 | 321 | 724 | 28.67 | 501.6 | 1E-141 | 361/405 | 89 | 3/405 | 0 | Plus | Plus | Bacteria; Firmicutes; Clostridia; Eubacteriales; Oscillospiraceae; Oscillibacter; Oscillibacter ruminantium | Oscillibacter ruminantium |
| ASV4332 | 422 | 1 | 1 | 407 | 96.45 | NR_113411.1 | Prevotella copri DSM 18205 strain JCM 13464 16S ribosomal RNA, partial sequence | NR_113411.1 | 1493 | 358 | 764 | 27.26 | 630.8 | 2E-180 | 386/408 | 94 | 2/408 | 0 | Plus | Plus | Bacteria; Bacteroidetes; Bacteroidia; Bacteroidales; Prevotellaceae; Prevotella; Prevotella copri | Prevotella copri |
| ASV4336 | 422 | 1 | 1 | 422 | 100 | NR_024816.1 | Prevotella salivae strain EPSA11 16S ribosomal RNA, partial sequence | NR_024816.1 | 1493 | 356 | 777 | 28.27 | 503.4 | 4E-142 | 374/424 | 88 | 4/424 | 0 | Plus | Plus | Bacteria; Bacteroidetes; Bacteroidia; Bacteroidales; Prevotellaceae; Prevotella; Prevotella salivae | Prevotella salivae |
| ASV4338 | 403 | 4 | 1 | 403 | 100 | NR_074399.1 | Ruminococcus albus 7 = DSM 20455 16S ribosomal RNA, partial sequence | NR_074399.1 | 1500 | 350 | 751 | 26.8 | 460.9 | 2E-129 | 353/404 | 87 | 3/404 | 0 | Plus | Plus | Bacteria; Firmicutes; Clostridia; Eubacteriales; Oscillospiraceae; Ruminococcus; Ruminococcus albus | Ruminococcus albus |
| ASV4339 | 402 | 1 | 1 | 402 | 100 | NR_151894.1 | Anaerotaenia torta strain FH052 16S ribosomal RNA, partial sequence | NR_151894.1 | 1479 | 352 | 753 | 27.18 | 693.6 | 0 | 393/402 | 97 | 0/402 | 0 | Plus | Plus | Bacteria; Firmicutes; Clostridia; Eubacteriales; Lachnospiraceae; Anaerotaenia; Anaerotaenia torta | Anaerotaenia torta |
| ASV4340 | 401 | 1 | 1 | 386 | 96.26 | NR_134091.1 | Mobilitalea sibirica strain P3M-3 16S ribosomal RNA, partial sequence | NR_134091.1 | 1444 | 352 | 738 | 26.8 | 398.1 | 2E-110 | 331/388 | 85 | 3/388 | 0 | Plus | Plus | Bacteria; Firmicutes; Clostridia; Eubacteriales; Lachnospiraceae; Mobilitalea; Mobilitalea sibirica | Mobilitalea sibirica |
| ASV4342 | 421 | 5 | 1 | 421 | 100 | NR_119109.1 | Pseudescherichia vulneris strain ATCC 33821 16S ribosomal RNA, partial sequence | NR_119109.1 | 1443 | 344 | 770 | 29.59 | 551.4 | 1E-156 | 388/430 | 90 | 12/430 | 2 | Plus | Plus | Bacteria; Proteobacteria; Gammaproteobacteria; Enterobacterales; Enterobacteriaceae; Pseudescherichia; Pseudescherichia vulneris | Pseudescherichia vulneris |
| ASV4343 | 404 | 1 | 1 | 403 | 99.75 | NR_025670.1 | Pseudoflavonifractor capillosus ATCC 29799 16S ribosomal RNA, partial sequence | NR_025670.1 | 1483 | 346 | 749 | 27.24 | 534.8 | 1E-151 | 367/405 | 90 | 3/405 | 0 | Plus | Plus | Bacteria; Firmicutes; Clostridia; Eubacteriales; Oscillospiraceae; Pseudoflavonifractor; Pseudoflavonifractor capillosus | Pseudoflavonifractor capillosus |
| ASV4344 | 422 | 2 | 1 | 422 | 100 | NR_029356.1 | Flavonifractor plautii strain 265 16S ribosomal RNA, partial sequence | NR_029356.1 | 1550 | 353 | 757 | 26.13 | 436.9 | 4E-122 | 363/422 | 86 | 17/422 | 4 | Plus | Plus | Bacteria; Firmicutes; Clostridia; Eubacteriales; Oscillospiraceae; Flavonifractor; Flavonifractor plautii | Flavonifractor plautii |
| ASV4346 | 424 | 1 | 1 | 424 | 100 | NR_144750.1 | Prevotellamassilia timonensis strain Marseille-P2831 16S ribosomal RNA, partial sequence | NR_144750.1 | 1493 | 358 | 779 | 28.27 | 599.4 | 4E-171 | 394/427 | 92 | 8/427 | 1 | Plus | Plus | Bacteria; Bacteroidetes; Bacteroidia; Bacteroidales; Prevotellaceae; Prevotellamassilia; Prevotellamassilia timonensis | Prevotellamassilia timonensis |
| ASV4348 | 402 | 1 | 1 | 402 | 100 | NR_029239.3 | Clostridium chartatabidum strain 163 16S ribosomal RNA, partial sequence | NR_029239.3 | 1460 | 323 | 724 | 27.53 | 516.3 | 4E-146 | 363/404 | 89 | 4/404 | 0 | Plus | Plus | Bacteria; Firmicutes; Clostridia; Eubacteriales; Clostridiaceae; Clostridium; Clostridium chartatabidum | Clostridium chartatabidum |
| ASV4349 | 403 | 1 | 3 | 403 | 99.5 | NR_117133.2 | Clostridium oceanicum strain DSM 1290 16S ribosomal RNA, partial sequence | NR_117133.2 | 1504 | 349 | 748 | 26.6 | 446.2 | 6E-125 | 354/407 | 86 | 13/407 | 3 | Plus | Plus | Bacteria; Firmicutes; Clostridia; Eubacteriales; Clostridiaceae; Clostridium; Clostridium oceanicum | Clostridium oceanicum |
| ASV4350 | 401 | 1 | 1 | 401 | 100 | NR_117374.1 | Parvibacter caecicola strain NR06 16S ribosomal RNA, partial sequence | NR_117374.1 | 1442 | 336 | 736 | 27.81 | 592 | 7E-169 | 374/401 | 93 | 0/401 | 0 | Plus | Plus | Bacteria; Actinobacteria; Coriobacteriia; Coriobacteriales; Coriobacteriaceae; Parvibacter; Parvibacter caecicola | Parvibacter caecicola |
| ASV4352 | 405 | 5 | 1 | 405 | 100 | NR_115693.1 | Gracilibacter thermotolerans JW/YJL-S1 16S ribosomal RNA, partial sequence | NR_115693.1 | 1526 | 459 | 861 | 26.41 | 558.8 | 7E-159 | 372/406 | 91 | 4/406 | 0 | Plus | Plus | Bacteria; Firmicutes; Clostridia; Eubacteriales; Gracilibacteraceae; Gracilibacter; Gracilibacter thermotolerans | Gracilibacter thermotolerans |
| ASV4353 | 405 | 1 | 1 | 405 | 100 | NR_044616.1 | Synergistes jonesii strain 78-1 16S ribosomal RNA, partial sequence | NR_044616.1 | 1355 | 345 | 749 | 29.89 | 715.8 | 0 | 398/405 | 98 | 0/405 | 0 | Plus | Plus | Bacteria; Synergistetes; Synergistia; Synergistales; Synergistaceae; Synergistes; Synergistes jonesii | Synergistes jonesii |
| ASV4354 | 402 | 2 | 1 | 402 | 100 | NR_133762.1 | Hungatella effluvii strain UB-B.2 16S ribosomal RNA, partial sequence | NR_133762.1 | 1409 | 318 | 719 | 28.53 | 549.6 | 4E-156 | 368/403 | 91 | 2/403 | 0 | Plus | Plus | Bacteria; Firmicutes; Clostridia; Eubacteriales; Clostridiaceae; Hungatella; Hungatella effluvii | Hungatella effluvii |
| ASV4357 | 422 | 1 | 1 | 413 | 97.87 | NR_041364.1 | Prevotella stercorea DSM 18206 strain CB35 16S ribosomal RNA, partial sequence | NR_041364.1 | 1488 | 354 | 766 | 27.76 | 592 | 7E-169 | 383/414 | 92 | 2/414 | 0 | Plus | Plus | Bacteria; Bacteroidetes; Bacteroidia; Bacteroidales; Prevotellaceae; Prevotella; Prevotella stercorea | Prevotella stercorea |
| ASV4361 | 405 | 1 | 1 | 405 | 100 | NR_159261.1 | Bifidobacterium callitrichidarum strain TRI 5 16S ribosomal RNA, partial sequence | NR_159261.1 | 1374 | 279 | 686 | 29.69 | 579.1 | 6E-165 | 377/408 | 92 | 3/408 | 0 | Plus | Plus | Bacteria; Actinobacteria; Actinomycetia; Bifidobacteriales; Bifidobacteriaceae; Bifidobacterium; Bifidobacterium callitrichidarum | Bifidobacterium callitrichidarum |
| ASV4362 | 405 | 1 | 3 | 405 | 99.51 | NR_026476.1 | Succinivibrio dextrinosolvens strain 0554 16S ribosomal RNA, partial sequence | NR_026476.1 | 1463 | 333 | 732 | 27.34 | 420.3 | 4E-117 | 345/403 | 85 | 3/403 | 0 | Plus | Plus | Bacteria; Proteobacteria; Gammaproteobacteria; Aeromonadales; Succinivibrionaceae; Succinivibrio; Succinivibrio dextrinosolvens | Succinivibrio dextrinosolvens |
| ASV4363 | 402 | 3 | 5 | 402 | 99 | NR_114728.1 | Clostridium tertium strain ATCC 14573 16S ribosomal RNA, partial sequence | NR_114728.1 | 1415 | 308 | 705 | 28.13 | 564.3 | 2E-160 | 368/399 | 92 | 2/399 | 0 | Plus | Plus | Bacteria; Firmicutes; Clostridia; Eubacteriales; Clostridiaceae; Clostridium; Clostridium tertium | Clostridium tertium |
| ASV4365 | 402 | 3 | 5 | 402 | 99 | NR_114728.1 | Clostridium tertium strain ATCC 14573 16S ribosomal RNA, partial sequence | NR_114728.1 | 1415 | 308 | 705 | 28.13 | 553.3 | 3E-157 | 366/399 | 91 | 2/399 | 0 | Plus | Plus | Bacteria; Firmicutes; Clostridia; Eubacteriales; Clostridiaceae; Clostridium; Clostridium tertium | Clostridium tertium |
| ASV4366 | 405 | 1 | 3 | 405 | 99.51 | NR_028816.1 | Turicibacter sanguinis strain MOL361 16S ribosomal RNA, partial sequence | NR_028816.1 | 1508 | 329 | 753 | 28.18 | 538.5 | 9E-153 | 384/425 | 90 | 22/425 | 5 | Plus | Plus | Bacteria; Firmicutes; Erysipelotrichia; Erysipelotrichales; Turicibacteraceae; Turicibacter; Turicibacter sanguinis | Turicibacter sanguinis |
| ASV4371 | 427 | 1 | 1 | 427 | 100 | NR_042797.1 | Treponema berlinense strain 7CPL208 16S ribosomal RNA, partial sequence | NR_042797.1 | 1440 | 275 | 701 | 29.65 | 629 | 6E-180 | 400/429 | 93 | 4/429 | 0 | Plus | Plus | Bacteria; Spirochaetes; Spirochaetia; Spirochaetales; Spirochaetaceae; Treponema; Treponema berlinense | Treponema berlinense |
| ASV4374 | 403 | 1 | 1 | 403 | 100 | NR_144737.1 | Emergencia timonensis strain SN18 16S ribosomal RNA, partial sequence | NR_144737.1 | 1480 | 364 | 767 | 27.3 | 507.1 | 3E-143 | 362/405 | 89 | 3/405 | 0 | Plus | Plus | Bacteria; Firmicutes; Clostridia; Eubacteriales; Clostridiales Family XIII. Incertae Sedis; Emergencia; Emergencia timonensis | Emergencia timonensis |
| ASV4375 | 403 | 3 | 1 | 403 | 100 | NR_117139.2 | Anaerostipes hadrus strain DSM 3319 16S ribosomal RNA, partial sequence | NR_117139.2 | 1521 | 363 | 764 | 26.43 | 555.1 | 9E-158 | 369/403 | 91 | 1/403 | 0 | Plus | Plus | Bacteria; Firmicutes; Clostridia; Eubacteriales; Lachnospiraceae; Anaerostipes; Anaerostipes hadrus | Anaerostipes hadrus |
| ASV4376 | 427 | 1 | 3 | 427 | 99.53 | NR_156094.1 | Desulfitispora elongata strain Acr1 16S ribosomal RNA, partial sequence | NR_156094.1 | 1491 | 394 | 819 | 28.57 | 444.3 | 2E-124 | 370/432 | 85 | 13/432 | 3 | Plus | Plus | Bacteria; Firmicutes; Clostridia; Eubacteriales; Peptococcaceae; Desulfitispora; Desulfitispora elongata | Desulfitispora elongata |
| ASV4377 | 405 | 1 | 3 | 405 | 99.51 | NR_026476.1 | Succinivibrio dextrinosolvens strain 0554 16S ribosomal RNA, partial sequence | NR_026476.1 | 1463 | 333 | 732 | 27.34 | 486.8 | 3E-137 | 358/404 | 88 | 5/404 | 1 | Plus | Plus | Bacteria; Proteobacteria; Gammaproteobacteria; Aeromonadales; Succinivibrionaceae; Succinivibrio; Succinivibrio dextrinosolvens | Succinivibrio dextrinosolvens |
| ASV4379 | 405 | 1 | 1 | 405 | 100 | NR_044048.1 | Coprococcus comes ATCC 27758 16S ribosomal RNA, partial sequence | NR_044048.1 | 1453 | 345 | 746 | 27.67 | 617.9 | 1E-176 | 382/405 | 94 | 3/405 | 0 | Plus | Plus | Bacteria; Firmicutes; Clostridia; Eubacteriales; Lachnospiraceae; Coprococcus; Coprococcus comes | Coprococcus comes |
| ASV4380 | 404 | 1 | 1 | 404 | 100 | NR_134714.1 | Clostridium oryzae strain KC3 16S ribosomal RNA, partial sequence | NR_134714.1 | 1458 | 320 | 721 | 27.57 | 529.3 | 6E-150 | 367/406 | 90 | 6/406 | 1 | Plus | Plus | Bacteria; Firmicutes; Clostridia; Eubacteriales; Clostridiaceae; Clostridium; Clostridium oryzae | Clostridium oryzae |
| ASV4381 | 403 | 1 | 1 | 394 | 97.77 | NR_104907.1 | Eubacterium coprostanoligenes strain HL 16S ribosomal RNA, partial sequence | NR_104907.1 | 1446 | 332 | 725 | 27.25 | 606.8 | 3E-173 | 373/395 | 94 | 2/395 | 0 | Plus | Plus | Bacteria; Firmicutes; Clostridia; Eubacteriales; Eubacteriaceae; Eubacterium; Eubacterium coprostanoligenes | Eubacterium coprostanoligenes |
| ASV4382 | 427 | 1 | 1 | 427 | 100 | NR_041805.1 | Selenomonas dianae strain ATCC 43527 16S ribosomal RNA, partial sequence | NR_041805.1 | 1494 | 378 | 804 | 28.58 | 610.5 | 2E-174 | 394/427 | 92 | 0/427 | 0 | Plus | Plus | Bacteria; Firmicutes; Negativicutes; Selenomonadales; Selenomonadaceae; Selenomonas; Selenomonas dianae | Selenomonas dianae |
| ASV4383 | 423 | 1 | 1 | 423 | 100 | NR_042842.1 | Prevotella denticola strain ATCC 35308 16S ribosomal RNA, partial sequence | NR_042842.1 | 1506 | 338 | 759 | 28.02 | 538.5 | 1E-152 | 381/425 | 89 | 5/425 | 1 | Plus | Plus | Bacteria; Bacteroidetes; Bacteroidia; Bacteroidales; Prevotellaceae; Prevotella; Prevotella denticola | Prevotella denticola |
| ASV4386 | 402 | 3 | 1 | 402 | 100 | NR_151917.1 | Helicobacter apri strain A19 16S ribosomal RNA, partial sequence | NR_151917.1 | 1099 | 36 | 437 | 36.58 | 688.1 | 0 | 392/402 | 97 | 0/402 | 0 | Plus | Plus | Bacteria; Proteobacteria; Epsilonproteobacteria; Campylobacterales; Helicobacteraceae; Helicobacter; Helicobacter apri | Helicobacter apri |
| ASV4389 | 422 | 3 | 1 | 422 | 100 | NR_102895.1 | Prevotella melaninogenica ATCC 25845 16S ribosomal RNA, partial sequence | NR_102895.1 | 1531 | 366 | 787 | 27.56 | 780.4 | 0 | 422/422 | 100 | 0/422 | 0 | Plus | Plus | Bacteria; Bacteroidetes; Bacteroidia; Bacteroidales; Prevotellaceae; Prevotella; Prevotella melaninogenica | Prevotella melaninogenica |
| ASV4390 | 422 | 1 | 1 | 422 | 100 | NR_113411.1 | Prevotella copri DSM 18205 strain JCM 13464 16S ribosomal RNA, partial sequence | NR_113411.1 | 1493 | 358 | 779 | 28.27 | 641.9 | 0 | 397/422 | 94 | 0/422 | 0 | Plus | Plus | Bacteria; Bacteroidetes; Bacteroidia; Bacteroidales; Prevotellaceae; Prevotella; Prevotella copri | Prevotella copri |
| ASV4391 | 422 | 1 | 1 | 407 | 96.45 | NR_113411.1 | Prevotella copri DSM 18205 strain JCM 13464 16S ribosomal RNA, partial sequence | NR_113411.1 | 1493 | 358 | 764 | 27.26 | 547.7 | 2E-155 | 371/408 | 90 | 2/408 | 0 | Plus | Plus | Bacteria; Bacteroidetes; Bacteroidia; Bacteroidales; Prevotellaceae; Prevotella; Prevotella copri | Prevotella copri |
| ASV4392 | 422 | 1 | 1 | 422 | 100 | NR_041364.1 | Prevotella stercorea DSM 18206 strain CB35 16S ribosomal RNA, partial sequence | NR_041364.1 | 1488 | 354 | 775 | 28.36 | 680.7 | 0 | 404/422 | 95 | 0/422 | 0 | Plus | Plus | Bacteria; Bacteroidetes; Bacteroidia; Bacteroidales; Prevotellaceae; Prevotella; Prevotella stercorea | Prevotella stercorea |
| ASV4393 | 402 | 4 | 1 | 402 | 100 | NR_042802.1 | Lactobacillus ultunensis strain Kx146C1 16S ribosomal RNA, partial sequence | NR_042802.1 | 1550 | 370 | 796 | 27.55 | 560.7 | 2E-159 | 390/427 | 91 | 25/427 | 5 | Plus | Plus | Bacteria; Firmicutes; Bacilli; Lactobacillales; Lactobacillaceae; Lactobacillus; Lactobacillus ultunensis | Lactobacillus ultunensis |
| ASV4394 | 405 | 1 | 1 | 405 | 100 | NR_026061.1 | Desulfohalotomaculum halophilum strain SEBR 3139 16S ribosomal RNA, partial sequence | NR_026061.1 | 1526 | 365 | 768 | 26.47 | 486.8 | 3E-137 | 358/405 | 88 | 1/405 | 0 | Plus | Plus | Bacteria; Firmicutes; Clostridia; Eubacteriales; Peptococcaceae; Desulfohalotomaculum; Desulfohalotomaculum halophilum | Desulfohalotomaculum halophilum |
| ASV4395 | 401 | 3 | 1 | 401 | 100 | NR_158111.1 | Paludicola psychrotolerans strain NC1253 16S ribosomal RNA, partial sequence | NR_158111.1 | 1490 | 366 | 766 | 26.91 | 592 | 7E-169 | 374/401 | 93 | 0/401 | 0 | Plus | Plus | Bacteria; Firmicutes; Clostridia; Eubacteriales; Oscillospiraceae; Paludicola; Paludicola psychrotolerans | Paludicola psychrotolerans |
| ASV4396 | 402 | 1 | 7 | 402 | 98.51 | NR_104846.1 | Gemmiger formicilis strain X2-56 16S ribosomal RNA, partial sequence | NR_104846.1 | 1426 | 311 | 706 | 27.77 | 582.8 | 4E-166 | 372/399 | 93 | 6/399 | 1 | Plus | Plus | Bacteria; Firmicutes; Clostridia; Eubacteriales; ; Gemmiger; Gemmiger formicilis | Gemmiger formicilis |
| ASV4399 | 427 | 3 | 1 | 427 | 100 | NR_169348.1 | Sutterella faecalis strain KGMB03119 16S ribosomal RNA, partial sequence | NR_169348.1 | 1367 | 336 | 762 | 31.24 | 612.4 | 6E-175 | 396/428 | 92 | 2/428 | 0 | Plus | Plus | Bacteria; Proteobacteria; Betaproteobacteria; Burkholderiales; Sutterellaceae; Sutterella; Sutterella faecalis | Sutterella faecalis |
| ASV4403 | 402 | 1 | 1 | 402 | 100 | NR_134005.1 | Clostridium bornimense strain M2/40 16S ribosomal RNA, partial sequence | NR_134005.1 | 1363 | 284 | 685 | 29.49 | 494.2 | 2E-139 | 358/403 | 88 | 2/403 | 0 | Plus | Plus | Bacteria; Firmicutes; Clostridia; Eubacteriales; Clostridiaceae; Clostridium; Clostridium bornimense | Clostridium bornimense |
| ASV4404 | 427 | 5 | 1 | 425 | 99.53 | NR_042052.1 | Streptococcus equinus ATCC 9812 16S ribosomal RNA, partial sequence | NR_042052.1 | 1433 | 260 | 684 | 29.66 | 508.9 | 8E-144 | 376/426 | 88 | 2/426 | 0 | Plus | Plus | Bacteria; Firmicutes; Bacilli; Lactobacillales; Streptococcaceae; Streptococcus; Streptococcus equinus | Streptococcus equinus |
| ASV4406 | 402 | 3 | 1 | 402 | 100 | NR_156081.1 | Kineothrix alysoides strain KNHs209 16S ribosomal RNA, partial sequence | NR_156081.1 | 1411 | 353 | 754 | 28.49 | 577.3 | 2E-164 | 372/402 | 92 | 0/402 | 0 | Plus | Plus | Bacteria; Firmicutes; Clostridia; Eubacteriales; Lachnospiraceae; Kineothrix; Kineothrix alysoides | Kineothrix alysoides |
| ASV4410 | 403 | 3 | 3 | 403 | 99.5 | NR_041781.1 | Streptococcus alactolyticus strain ATCC 43077 16S ribosomal RNA, partial sequence | NR_041781.1 | 1437 | 348 | 772 | 29.58 | 525.6 | 7E-149 | 383/426 | 89 | 26/426 | 6 | Plus | Plus | Bacteria; Firmicutes; Bacilli; Lactobacillales; Streptococcaceae; Streptococcus; Streptococcus alactolyticus | Streptococcus alactolyticus |
| ASV4411 | 404 | 1 | 3 | 404 | 99.5 | NR_112900.1 | Christensenella minuta strain YIT 12065 16S ribosomal RNA, partial sequence | NR_112900.1 | 1497 | 360 | 762 | 26.92 | 411.1 | 2E-114 | 348/408 | 85 | 11/408 | 2 | Plus | Plus | Bacteria; Firmicutes; Clostridia; Eubacteriales; Christensenellaceae; Christensenella; Christensenella minuta | Christensenella minuta |
| ASV4412 | 405 | 1 | 1 | 405 | 100 | NR_114789.1 | [Clostridium] leptum strain DSM 753 16S ribosomal RNA, partial sequence | NR_114789.1 | 1505 | 364 | 765 | 26.71 | 468.3 | 1E-131 | 355/405 | 87 | 3/405 | 0 | Plus | Plus | Bacteria; Firmicutes; Clostridia; Eubacteriales; Oscillospiraceae; ; [Clostridium] leptum | [Clostridium] leptum |
| ASV4414 | 404 | 1 | 3 | 404 | 99.5 | NR_118156.1 | Oscillibacter ruminantium GH1 16S ribosomal RNA, partial sequence | NR_118156.1 | 1409 | 323 | 724 | 28.53 | 621.6 | 9E-178 | 380/402 | 94 | 0/402 | 0 | Plus | Plus | Bacteria; Firmicutes; Clostridia; Eubacteriales; Oscillospiraceae; Oscillibacter; Oscillibacter ruminantium | Oscillibacter ruminantium |
| ASV4415 | 404 | 1 | 3 | 404 | 99.5 | NR_144737.1 | Emergencia timonensis strain SN18 16S ribosomal RNA, partial sequence | NR_144737.1 | 1480 | 366 | 767 | 27.16 | 505.3 | 1E-142 | 360/403 | 89 | 2/403 | 0 | Plus | Plus | Bacteria; Firmicutes; Clostridia; Eubacteriales; Clostridiales Family XIII. Incertae Sedis; Emergencia; Emergencia timonensis | Emergencia timonensis |
| ASV4417 | 427 | 1 | 1 | 379 | 88.76 | NR_042942.1 | Treponema porcinum strain 14V28 16S ribosomal RNA, partial sequence | NR_042942.1 | 1426 | 258 | 637 | 26.65 | 557 | 3E-158 | 355/381 | 93 | 3/381 | 0 | Plus | Plus | Bacteria; Spirochaetes; Spirochaetia; Spirochaetales; Spirochaetaceae; Treponema; Treponema porcinum | Treponema porcinum |
| ASV4418 | 357 | 1 | 40 | 357 | 89.08 | NR_042942.1 | Treponema porcinum strain 14V28 16S ribosomal RNA, partial sequence | NR_042942.1 | 1426 | 368 | 684 | 22.23 | 575.4 | 6E-164 | 316/318 | 99 | 1/318 | 0 | Plus | Plus | Bacteria; Spirochaetes; Spirochaetia; Spirochaetales; Spirochaetaceae; Treponema; Treponema porcinum | Treponema porcinum |
| ASV4421 | 403 | 1 | 3 | 403 | 99.5 | NR_125677.1 | Vallitalea pronyensis strain FatNI3 16S ribosomal RNA, partial sequence | NR_125677.1 | 1480 | 365 | 764 | 27.03 | 479.4 | 6E-135 | 356/403 | 88 | 5/403 | 1 | Plus | Plus | Bacteria; Firmicutes; Clostridia; Eubacteriales; Vallitaleaceae; Vallitalea; Vallitalea pronyensis | Vallitalea pronyensis |
| ASV4422 | 402 | 1 | 1 | 402 | 100 | NR_041960.1 | Blautia luti strain BInIX 16S ribosomal RNA, partial sequence | NR_041960.1 | 1308 | 239 | 641 | 30.81 | 605 | 9E-173 | 378/403 | 93 | 1/403 | 0 | Plus | Plus | Bacteria; Firmicutes; Clostridia; Eubacteriales; Lachnospiraceae; Blautia; Blautia luti | Blautia luti |
| ASV4423 | 420 | 2 | 1 | 420 | 100 | NR_029335.1 | Holdemania filiformis strain J1-31B-1 16S ribosomal RNA, partial sequence | NR_029335.1 | 1391 | 267 | 692 | 30.63 | 455.4 | 1E-127 | 367/426 | 86 | 6/426 | 1 | Plus | Plus | Bacteria; Firmicutes; Erysipelotrichia; Erysipelotrichales; Erysipelotrichaceae; Holdemania; Holdemania filiformis | Holdemania filiformis |
| ASV4426 | 404 | 1 | 1 | 404 | 100 | NR_116936.1 | Olsenella umbonata strain lac31 16S ribosomal RNA, partial sequence | NR_116936.1 | 1428 | 328 | 735 | 28.57 | 595.7 | 5E-170 | 382/410 | 93 | 8/410 | 1 | Plus | Plus | Bacteria; Actinobacteria; Coriobacteriia; Coriobacteriales; Atopobiaceae; Olsenella; Olsenella umbonata | Olsenella umbonata |
| ASV4428 | 423 | 1 | 1 | 423 | 100 | NR_113411.1 | Prevotella copri DSM 18205 strain JCM 13464 16S ribosomal RNA, partial sequence | NR_113411.1 | 1493 | 358 | 779 | 28.27 | 575.4 | 7E-164 | 390/427 | 91 | 9/427 | 2 | Plus | Plus | Bacteria; Bacteroidetes; Bacteroidia; Bacteroidales; Prevotellaceae; Prevotella; Prevotella copri | Prevotella copri |
| ASV4429 | 403 | 1 | 3 | 403 | 99.5 | NR_026476.1 | Succinivibrio dextrinosolvens strain 0554 16S ribosomal RNA, partial sequence | NR_026476.1 | 1463 | 333 | 732 | 27.34 | 435.1 | 1E-121 | 347/402 | 86 | 3/402 | 0 | Plus | Plus | Bacteria; Proteobacteria; Gammaproteobacteria; Aeromonadales; Succinivibrionaceae; Succinivibrio; Succinivibrio dextrinosolvens | Succinivibrio dextrinosolvens |
| ASV4430 | 428 | 4 | 3 | 428 | 99.53 | NR_042802.1 | Lactobacillus ultunensis strain Kx146C1 16S ribosomal RNA, partial sequence | NR_042802.1 | 1550 | 372 | 796 | 27.42 | 597.6 | 2E-170 | 393/427 | 92 | 3/427 | 0 | Plus | Plus | Bacteria; Firmicutes; Bacilli; Lactobacillales; Lactobacillaceae; Lactobacillus; Lactobacillus ultunensis | Lactobacillus ultunensis |
| ASV4433 | 402 | 1 | 1 | 402 | 100 | NR_134005.1 | Clostridium bornimense strain M2/40 16S ribosomal RNA, partial sequence | NR_134005.1 | 1363 | 284 | 685 | 29.49 | 616.1 | 4E-176 | 379/402 | 94 | 0/402 | 0 | Plus | Plus | Bacteria; Firmicutes; Clostridia; Eubacteriales; Clostridiaceae; Clostridium; Clostridium bornimense | Clostridium bornimense |
| ASV4435 | 405 | 1 | 1 | 405 | 100 | NR_044972.1 | Sporobacter termitidis strain SYR 16S ribosomal RNA, partial sequence | NR_044972.1 | 1506 | 348 | 752 | 26.89 | 536.6 | 3E-152 | 366/405 | 90 | 0/405 | 0 | Plus | Plus | Bacteria; Firmicutes; Clostridia; Eubacteriales; Oscillospiraceae; Sporobacter; Sporobacter termitidis | Sporobacter termitidis |
| ASV4436 | 401 | 1 | 3 | 401 | 99.5 | NR_118156.1 | Oscillibacter ruminantium GH1 16S ribosomal RNA, partial sequence | NR_118156.1 | 1409 | 323 | 724 | 28.53 | 529.3 | 6E-150 | 366/404 | 90 | 7/404 | 1 | Plus | Plus | Bacteria; Firmicutes; Clostridia; Eubacteriales; Oscillospiraceae; Oscillibacter; Oscillibacter ruminantium | Oscillibacter ruminantium |
| ASV4438 | 422 | 1 | 1 | 422 | 100 | NR_145844.1 | Bacteroides caecigallinarum strain C13EG111 16S ribosomal RNA, partial sequence | NR_145844.1 | 1489 | 356 | 777 | 28.34 | 531.1 | 2E-150 | 377/422 | 89 | 0/422 | 0 | Plus | Plus | Bacteria; Bacteroidetes; Bacteroidia; Bacteroidales; Bacteroidaceae; Bacteroides; Bacteroides caecigallinarum | Bacteroides caecigallinarum |
| ASV4439 | 403 | 1 | 3 | 403 | 99.5 | NR_074809.1 | Roseburia hominis A2-183 16S ribosomal RNA, partial sequence | NR_074809.1 | 1535 | 369 | 769 | 26.12 | 508.9 | 7E-144 | 359/401 | 90 | 0/401 | 0 | Plus | Plus | Bacteria; Firmicutes; Clostridia; Eubacteriales; Lachnospiraceae; Roseburia; Roseburia hominis | Roseburia hominis |
| ASV4440 | 427 | 1 | 15 | 427 | 96.72 | NR_104750.1 | Spiroplasma clarkii strain CN-5 16S ribosomal RNA, partial sequence | NR_104750.1 | 1507 | 351 | 762 | 27.34 | 429.5 | 6E-120 | 354/414 | 85 | 3/414 | 0 | Plus | Plus | Bacteria; Tenericutes; Mollicutes; Entomoplasmatales; Spiroplasmataceae; Spiroplasma; Spiroplasma clarkii | Spiroplasma clarkii |
| ASV4441 | 402 | 1 | 1 | 402 | 100 | NR_114789.1 | [Clostridium] leptum strain DSM 753 16S ribosomal RNA, partial sequence | NR_114789.1 | 1505 | 364 | 765 | 26.71 | 566.2 | 4E-161 | 370/402 | 92 | 0/402 | 0 | Plus | Plus | Bacteria; Firmicutes; Clostridia; Eubacteriales; Oscillospiraceae; ; [Clostridium] leptum | [Clostridium] leptum |
| ASV4442 | 426 | 5 | 1 | 426 | 100 | NR_146813.2 | Erysipelothrix larvae strain LV19 16S ribosomal RNA, complete sequence | NR_146813.2 | 1543 | 367 | 792 | 27.61 | 472 | 1E-132 | 370/427 | 87 | 2/427 | 0 | Plus | Plus | Bacteria; Firmicutes; Erysipelotrichia; Erysipelotrichales; Erysipelotrichaceae; Erysipelothrix; Erysipelothrix larvae | Erysipelothrix larvae |
| ASV4444 | 401 | 1 | 1 | 401 | 100 | NR_114326.1 | Fusicatenibacter saccharivorans strain HT03-11 16S ribosomal RNA, partial sequence | NR_114326.1 | 1423 | 351 | 752 | 28.25 | 448 | 2E-125 | 351/404 | 86 | 5/404 | 1 | Plus | Plus | Bacteria; Firmicutes; Clostridia; Eubacteriales; Lachnospiraceae; Fusicatenibacter; Fusicatenibacter saccharivorans | Fusicatenibacter saccharivorans |
| ASV4445 | 422 | 2 | 1 | 422 | 100 | NR_113119.1 | Prevotella oris strain JCM 8540 16S ribosomal RNA, partial sequence | NR_113119.1 | 1481 | 345 | 766 | 28.49 | 581 | 2E-165 | 386/422 | 91 | 0/422 | 0 | Plus | Plus | Bacteria; Bacteroidetes; Bacteroidia; Bacteroidales; Prevotellaceae; Prevotella; Prevotella oris | Prevotella oris |
| ASV4450 | 426 | 1 | 1 | 426 | 100 | NR_041611.1 | Lactobacillus ruminis strain NBRC 102161 16S ribosomal RNA, partial sequence | NR_041611.1 | 1488 | 352 | 778 | 28.7 | 643.8 | 0 | 402/428 | 93 | 3/428 | 0 | Plus | Plus | Bacteria; Firmicutes; Bacilli; Lactobacillales; Lactobacillaceae; Ligilactobacillus; Ligilactobacillus ruminis | Ligilactobacillus ruminis |
| ASV4451 | 405 | 4 | 3 | 405 | 99.51 | NR_118511.1 | Campylobacter coli strain ATCC 33559 16S ribosomal RNA, partial sequence | NR_118511.1 | 1435 | 331 | 731 | 27.94 | 599.4 | 4E-171 | 377/403 | 93 | 2/403 | 0 | Plus | Plus | Bacteria; Proteobacteria; Epsilonproteobacteria; Campylobacterales; Campylobacteraceae; Campylobacter; Campylobacter coli | Campylobacter coli |
| ASV4453 | 401 | 1 | 1 | 399 | 99.5 | NR_118667.1 | Lachnospira pectinoschiza strain 150-1 16S ribosomal RNA, partial sequence | NR_118667.1 | 1465 | 372 | 771 | 27.3 | 468.3 | 1E-131 | 350/401 | 87 | 3/401 | 0 | Plus | Plus | Bacteria; Firmicutes; Clostridia; Eubacteriales; Lachnospiraceae; Lachnospira; Lachnospira pectinoschiza | Lachnospira pectinoschiza |
| ASV4454 | 403 | 2 | 3 | 403 | 99.5 | NR_104892.1 | Clostridium moniliforme strain 2055 16S ribosomal RNA, partial sequence | NR_104892.1 | 1488 | 332 | 731 | 26.88 | 518.2 | 1E-146 | 363/403 | 90 | 5/403 | 1 | Plus | Plus | Bacteria; Firmicutes; Clostridia; Eubacteriales; Clostridiaceae; Clostridium; Clostridium moniliforme | Clostridium moniliforme |
| ASV4457 | 427 | 2 | 1 | 426 | 99.77 | NR_037018.1 | Acidaminococcus fermentans strain VR4 16S ribosomal RNA gene, partial sequence | NR_037018.1 | 1516 | 349 | 774 | 28.1 | 710.2 | 0 | 412/426 | 96 | 0/426 | 0 | Plus | Plus | Bacteria; Firmicutes; Negativicutes; Acidaminococcales; Acidaminococcaceae; Acidaminococcus; Acidaminococcus fermentans | Acidaminococcus fermentans |
| ASV4459 | 404 | 1 | 1 | 404 | 100 | NR_159261.1 | Bifidobacterium callitrichidarum strain TRI 5 16S ribosomal RNA, partial sequence | NR_159261.1 | 1374 | 279 | 686 | 29.69 | 529.3 | 6E-150 | 368/408 | 90 | 4/408 | 0 | Plus | Plus | Bacteria; Actinobacteria; Actinomycetia; Bifidobacteriales; Bifidobacteriaceae; Bifidobacterium; Bifidobacterium callitrichidarum | Bifidobacterium callitrichidarum |
| ASV4460 | 403 | 2 | 1 | 403 | 100 | NR_074629.1 | Hungateiclostridium thermocellum strain ATCC 27405 16S ribosomal RNA, partial sequence | NR_074629.1 | 1521 | 358 | 762 | 26.63 | 503.4 | 3E-142 | 361/405 | 89 | 2/405 | 0 | Plus | Plus | Bacteria; Firmicutes; Clostridia; Eubacteriales; Oscillospiraceae; Acetivibrio; Acetivibrio thermocellus | Acetivibrio thermocellus |
| ASV4464 | 427 | 1 | 1 | 427 | 100 | NR_025131.1 | Treponema parvum strain OMZ 833 16S ribosomal RNA, partial sequence | NR_025131.1 | 1485 | 341 | 767 | 28.75 | 529.3 | 6E-150 | 385/432 | 89 | 10/432 | 2 | Plus | Plus | Bacteria; Spirochaetes; Spirochaetia; Spirochaetales; Spirochaetaceae; Treponema; Treponema parvum | Treponema parvum |
| ASV4465 | 404 | 1 | 1 | 404 | 100 | NR_112900.1 | Christensenella minuta strain YIT 12065 16S ribosomal RNA, partial sequence | NR_112900.1 | 1497 | 358 | 762 | 27.05 | 586.5 | 3E-167 | 377/406 | 92 | 3/406 | 0 | Plus | Plus | Bacteria; Firmicutes; Clostridia; Eubacteriales; Christensenellaceae; Christensenella; Christensenella minuta | Christensenella minuta |
| ASV4466 | 427 | 1 | 1 | 427 | 100 | NR_028816.1 | Turicibacter sanguinis strain MOL361 16S ribosomal RNA, partial sequence | NR_028816.1 | 1508 | 327 | 753 | 28.32 | 473.9 | 3E-133 | 371/428 | 86 | 2/428 | 0 | Plus | Plus | Bacteria; Firmicutes; Erysipelotrichia; Erysipelotrichales; Turicibacteraceae; Turicibacter; Turicibacter sanguinis | Turicibacter sanguinis |
| ASV4467 | 404 | 1 | 1 | 404 | 100 | NR_117867.1 | Blautia stercoris strain GAM6-1 16S ribosomal RNA, partial sequence | NR_117867.1 | 1372 | 317 | 718 | 29.3 | 468.3 | 1E-131 | 355/405 | 87 | 4/405 | 0 | Plus | Plus | Bacteria; Firmicutes; Clostridia; Eubacteriales; Lachnospiraceae; Blautia; Blautia stercoris | Blautia stercoris |
| ASV4468 | 402 | 1 | 1 | 402 | 100 | NR_144727.1 | Anaeromassilibacillus senegalensis strain mt9 16S ribosomal RNA, partial sequence | NR_144727.1 | 1478 | 352 | 753 | 27.2 | 577.3 | 2E-164 | 373/403 | 92 | 2/403 | 0 | Plus | Plus | Bacteria; Firmicutes; Clostridia; Eubacteriales; Oscillospiraceae; Anaeromassilibacillus; Anaeromassilibacillus senegalensis | Anaeromassilibacillus senegalensis |
| ASV4469 | 404 | 3 | 1 | 404 | 100 | NR_041781.1 | Streptococcus alactolyticus strain ATCC 43077 16S ribosomal RNA, partial sequence | NR_041781.1 | 1437 | 346 | 772 | 29.71 | 523.7 | 3E-148 | 383/427 | 89 | 23/427 | 5 | Plus | Plus | Bacteria; Firmicutes; Bacilli; Lactobacillales; Streptococcaceae; Streptococcus; Streptococcus alactolyticus | Streptococcus alactolyticus |
| ASV4471 | 401 | 1 | 3 | 401 | 99.5 | NR_074652.1 | Lachnoclostridium phytofermentans strain ISDg 16S ribosomal RNA, partial sequence | NR_074652.1 | 1523 | 366 | 765 | 26.26 | 494.2 | 2E-139 | 359/403 | 89 | 7/403 | 1 | Plus | Plus | Bacteria; Firmicutes; Clostridia; Eubacteriales; Lachnospiraceae; Lachnoclostridium; Lachnoclostridium phytofermentans | Lachnoclostridium phytofermentans |
| ASV4472 | 402 | 5 | 1 | 402 | 100 | NR_115693.1 | Gracilibacter thermotolerans JW/YJL-S1 16S ribosomal RNA, partial sequence | NR_115693.1 | 1526 | 459 | 861 | 26.41 | 533 | 4E-151 | 366/404 | 90 | 3/404 | 0 | Plus | Plus | Bacteria; Firmicutes; Clostridia; Eubacteriales; Gracilibacteraceae; Gracilibacter; Gracilibacter thermotolerans | Gracilibacter thermotolerans |
| ASV4475 | 422 | 1 | 1 | 422 | 100 | NR_113411.1 | Prevotella copri DSM 18205 strain JCM 13464 16S ribosomal RNA, partial sequence | NR_113411.1 | 1493 | 358 | 779 | 28.27 | 664.1 | 0 | 401/422 | 95 | 0/422 | 0 | Plus | Plus | Bacteria; Bacteroidetes; Bacteroidia; Bacteroidales; Prevotellaceae; Prevotella; Prevotella copri | Prevotella copri |
| ASV4476 | 422 | 1 | 1 | 422 | 100 | NR_113411.1 | Prevotella copri DSM 18205 strain JCM 13464 16S ribosomal RNA, partial sequence | NR_113411.1 | 1493 | 358 | 779 | 28.27 | 625.3 | 7E-179 | 394/422 | 93 | 0/422 | 0 | Plus | Plus | Bacteria; Bacteroidetes; Bacteroidia; Bacteroidales; Prevotellaceae; Prevotella; Prevotella copri | Prevotella copri |
| ASV4477 | 427 | 1 | 1 | 427 | 100 | NR_041778.1 | Desulfovibrio piger strain ATCC 29098 16S ribosomal RNA, partial sequence | NR_041778.1 | 1542 | 357 | 783 | 27.69 | 667.8 | 0 | 405/427 | 94 | 0/427 | 0 | Plus | Plus | Bacteria; Proteobacteria; Deltaproteobacteria; Desulfovibrionales; Desulfovibrionaceae; Desulfovibrio; Desulfovibrio piger | Desulfovibrio piger |
| ASV4478 | 407 | 1 | 1 | 407 | 100 | NR_028961.1 | Faecalibacterium prausnitzii strain ATCC 27768 16S ribosomal RNA, partial sequence | NR_028961.1 | 1462 | 344 | 745 | 27.5 | 558.8 | 7E-159 | 374/408 | 91 | 7/408 | 1 | Plus | Plus | Bacteria; Firmicutes; Clostridia; Eubacteriales; Oscillospiraceae; Faecalibacterium; Faecalibacterium prausnitzii | Faecalibacterium prausnitzii |
| ASV4479 | 403 | 2 | 1 | 403 | 100 | NR_029160.1 | Ruminococcus callidus strain ATCC 27760 16S ribosomal RNA, partial sequence | NR_029160.1 | 1371 | 310 | 711 | 29.32 | 549.6 | 4E-156 | 371/406 | 91 | 7/406 | 1 | Plus | Plus | Bacteria; Firmicutes; Clostridia; Eubacteriales; Oscillospiraceae; Ruminococcus; Ruminococcus callidus | Ruminococcus callidus |
| ASV4480 | 401 | 4 | 1 | 399 | 99.5 | NR_144617.1 | Muricomes intestini strain 2PG-424-CC-1 16S ribosomal RNA, partial sequence | NR_144617.1 | 1431 | 357 | 756 | 27.95 | 455.4 | 1E-127 | 353/404 | 87 | 9/404 | 2 | Plus | Plus | Bacteria; Firmicutes; Clostridia; Eubacteriales; Lachnospiraceae; Muricomes; Muricomes intestini | Muricomes intestini |
| ASV4481 | 405 | 1 | 1 | 405 | 100 | NR_159227.1 | Monoglobus pectinilyticus strain 14 16S ribosomal RNA, partial sequence | NR_159227.1 | 1493 | 368 | 769 | 26.93 | 518.2 | 1E-146 | 366/407 | 89 | 7/407 | 1 | Plus | Plus | Bacteria; Firmicutes; Clostridia; Eubacteriales; Oscillospiraceae; Monoglobus; Monoglobus pectinilyticus | Monoglobus pectinilyticus |
| ASV4482 | 401 | 1 | 1 | 401 | 100 | NR_119085.1 | [Clostridium] polysaccharolyticum strain DSM 1801 16S ribosomal RNA, partial sequence | NR_119085.1 | 1503 | 364 | 763 | 26.61 | 424 | 3E-118 | 345/402 | 85 | 3/402 | 0 | Plus | Plus | Bacteria; Firmicutes; Clostridia; Eubacteriales; Lachnospiraceae; Lachnoclostridium; [Clostridium] polysaccharolyticum | [Clostridium] polysaccharolyticum |
| ASV4483 | 405 | 1 | 3 | 405 | 99.51 | NR_042832.1 | Roseburia faecis strain M72/1 16S ribosomal RNA, partial sequence | NR_042832.1 | 1456 | 349 | 749 | 27.54 | 638.2 | 0 | 384/403 | 95 | 2/403 | 0 | Plus | Plus | Bacteria; Firmicutes; Clostridia; Eubacteriales; Lachnospiraceae; Roseburia; Roseburia faecis | Roseburia faecis |
| ASV4484 | 405 | 3 | 3 | 405 | 99.51 | NR_042144.1 | Clostridium butyricum strain VPI3266 16S ribosomal RNA, partial sequence | NR_042144.1 | 1425 | 340 | 739 | 28.07 | 581 | 2E-165 | 374/403 | 92 | 3/403 | 0 | Plus | Plus | Bacteria; Firmicutes; Clostridia; Eubacteriales; Clostridiaceae; Clostridium; Clostridium butyricum | Clostridium butyricum |
| ASV4487 | 422 | 2 | 1 | 422 | 100 | NR_074376.1 | Parabacteroides distasonis ATCC 8503 16S ribosomal RNA, partial sequence | NR_074376.1 | 1497 | 368 | 789 | 28.19 | 497.9 | 2E-140 | 371/422 | 87 | 0/422 | 0 | Plus | Plus | Bacteria; Bacteroidetes; Bacteroidia; Bacteroidales; Tannerellaceae; Parabacteroides; Parabacteroides distasonis | Parabacteroides distasonis |
| ASV4488 | 403 | 2 | 2 | 403 | 99.75 | NR_027579.1 | Ruminococcus lactaris ATCC 29176 16S ribosomal RNA, partial sequence | NR_027579.1 | 1402 | 334 | 734 | 28.6 | 571.7 | 9E-163 | 371/402 | 92 | 1/402 | 0 | Plus | Plus | Bacteria; Firmicutes; Clostridia; Eubacteriales; Lachnospiraceae; Mediterraneibacter; [Ruminococcus] lactaris | [Ruminococcus] lactaris |
| ASV4490 | 402 | 1 | 1 | 402 | 100 | NR_104846.1 | Gemmiger formicilis strain X2-56 16S ribosomal RNA, partial sequence | NR_104846.1 | 1426 | 305 | 706 | 28.19 | 632.7 | 0 | 384/404 | 95 | 4/404 | 0 | Plus | Plus | Bacteria; Firmicutes; Clostridia; Eubacteriales; ; Gemmiger; Gemmiger formicilis | Gemmiger formicilis |
| ASV4491 | 427 | 4 | 1 | 427 | 100 | NR_042802.1 | Lactobacillus ultunensis strain Kx146C1 16S ribosomal RNA, partial sequence | NR_042802.1 | 1550 | 370 | 796 | 27.55 | 684.4 | 0 | 411/430 | 95 | 6/430 | 1 | Plus | Plus | Bacteria; Firmicutes; Bacilli; Lactobacillales; Lactobacillaceae; Lactobacillus; Lactobacillus ultunensis | Lactobacillus ultunensis |
| ASV4493 | 405 | 3 | 1 | 405 | 100 | NR_074629.1 | Hungateiclostridium thermocellum strain ATCC 27405 16S ribosomal RNA, partial sequence | NR_074629.1 | 1521 | 358 | 762 | 26.63 | 472 | 1E-132 | 355/405 | 87 | 0/405 | 0 | Plus | Plus | Bacteria; Firmicutes; Clostridia; Eubacteriales; Oscillospiraceae; Acetivibrio; Acetivibrio thermocellus | Acetivibrio thermocellus |
| ASV4494 | 403 | 1 | 3 | 403 | 99.5 | NR_125464.1 | Anaerobacterium chartisolvens strain T-1-35 16S ribosomal RNA, partial sequence | NR_125464.1 | 1451 | 344 | 745 | 27.71 | 486.8 | 3E-137 | 356/402 | 88 | 1/402 | 0 | Plus | Plus | Bacteria; Firmicutes; Clostridia; Eubacteriales; Oscillospiraceae; Anaerobacterium; Anaerobacterium chartisolvens | Anaerobacterium chartisolvens |
| ASV4496 | 403 | 1 | 1 | 403 | 100 | NR_137408.1 | Terrisporobacter petrolearius strain LAM0A37 16S ribosomal RNA, partial sequence | NR_137408.1 | 1405 | 307 | 707 | 28.54 | 527.4 | 2E-149 | 367/406 | 90 | 8/406 | 1 | Plus | Plus | Bacteria; Firmicutes; Clostridia; Eubacteriales; Peptostreptococcaceae; Terrisporobacter; Terrisporobacter petrolearius | Terrisporobacter petrolearius |
| ASV4497 | 401 | 1 | 1 | 399 | 99.5 | NR_024945.1 | Clostridium gasigenes strain DSM 12272 16S ribosomal RNA, partial sequence | NR_024945.1 | 1483 | 348 | 747 | 26.97 | 416.6 | 5E-116 | 344/402 | 85 | 5/402 | 1 | Plus | Plus | Bacteria; Firmicutes; Clostridia; Eubacteriales; Clostridiaceae; Clostridium; Clostridium gasigenes | Clostridium gasigenes |
| ASV4498 | 427 | 1 | 1 | 427 | 100 | NR_102980.1 | Megasphaera elsdenii DSM 20460 16S ribosomal RNA, partial sequence | NR_102980.1 | 1552 | 369 | 795 | 27.51 | 634.5 | 0 | 399/427 | 93 | 0/427 | 0 | Plus | Plus | Bacteria; Firmicutes; Negativicutes; Veillonellales; Veillonellaceae; Megasphaera; Megasphaera elsdenii | Megasphaera elsdenii |
| ASV4501 | 428 | 5 | 1 | 427 | 99.77 | NR_042052.1 | Streptococcus equinus ATCC 9812 16S ribosomal RNA, partial sequence | NR_042052.1 | 1433 | 260 | 685 | 29.73 | 472 | 1E-132 | 371/428 | 86 | 3/428 | 0 | Plus | Plus | Bacteria; Firmicutes; Bacilli; Lactobacillales; Streptococcaceae; Streptococcus; Streptococcus equinus | Streptococcus equinus |
| ASV4503 | 402 | 1 | 1 | 393 | 97.76 | NR_170495.1 | Blautia faecicola strain KGMB01111 16S ribosomal RNA, complete sequence | NR_170495.1 | 1534 | 372 | 764 | 25.62 | 494.2 | 2E-139 | 352/394 | 89 | 2/394 | 0 | Plus | Plus | Bacteria; Firmicutes; Clostridia; Eubacteriales; Lachnospiraceae; Blautia; Blautia faecicola | Blautia faecicola |
| ASV4504 | 405 | 2 | 1 | 405 | 100 | NR_074629.1 | Hungateiclostridium thermocellum strain ATCC 27405 16S ribosomal RNA, partial sequence | NR_074629.1 | 1521 | 358 | 762 | 26.63 | 466.5 | 4E-131 | 354/405 | 87 | 0/405 | 0 | Plus | Plus | Bacteria; Firmicutes; Clostridia; Eubacteriales; Oscillospiraceae; Acetivibrio; Acetivibrio thermocellus | Acetivibrio thermocellus |
| ASV4505 | 405 | 1 | 1 | 405 | 100 | NR_165727.1 | Anaerotignum faecicola strain KGMB03357 16S ribosomal RNA, complete sequence | NR_165727.1 | 1535 | 375 | 780 | 26.45 | 599.4 | 4E-171 | 379/406 | 93 | 1/406 | 0 | Plus | Plus | Bacteria; Firmicutes; Clostridia; Eubacteriales; Lachnospiraceae; Anaerotignum; Anaerotignum faecicola | Anaerotignum faecicola |
| ASV4507 | 404 | 1 | 1 | 404 | 100 | NR_027562.1 | Anaerofustis stercorihominis strain WAL 14563 16S ribosomal RNA, partial sequence | NR_027562.1 | 1462 | 335 | 736 | 27.5 | 496 | 6E-140 | 360/405 | 88 | 4/405 | 0 | Plus | Plus | Bacteria; Firmicutes; Clostridia; Eubacteriales; Eubacteriaceae; Anaerofustis; Anaerofustis stercorihominis | Anaerofustis stercorihominis |
| ASV4508 | 403 | 1 | 1 | 403 | 100 | NR_025930.1 | Ruminococcus bromii strain ATCC 27255 16S ribosomal RNA, partial sequence | NR_025930.1 | 1380 | 313 | 716 | 29.28 | 520 | 3E-147 | 363/404 | 89 | 1/404 | 0 | Plus | Plus | Bacteria; Firmicutes; Clostridia; Eubacteriales; Oscillospiraceae; Ruminococcus; Ruminococcus bromii | Ruminococcus bromii |
| ASV4510 | 404 | 1 | 3 | 404 | 99.5 | NR_044490.1 | Butyricicoccus pullicaecorum strain 25-3 16S ribosomal RNA, partial sequence | NR_044490.1 | 1482 | 368 | 770 | 27.19 | 455.4 | 1E-127 | 351/403 | 87 | 1/403 | 0 | Plus | Plus | Bacteria; Firmicutes; Clostridia; Eubacteriales; Clostridiaceae; Butyricicoccus; Butyricicoccus pullicaecorum | Butyricicoccus pullicaecorum |
| ASV4512 | 407 | 3 | 1 | 407 | 100 | NR_036856.1 | Bifidobacterium pullorum strain B 145 16S ribosomal RNA, partial sequence | NR_036856.1 | 1517 | 347 | 753 | 26.83 | 564.3 | 2E-160 | 373/407 | 91 | 0/407 | 0 | Plus | Plus | Bacteria; Actinobacteria; Actinomycetia; Bifidobacteriales; Bifidobacteriaceae; Bifidobacterium; Bifidobacterium pullorum | Bifidobacterium pullorum |
| ASV4513 | 422 | 1 | 1 | 422 | 100 | NR_144750.1 | Prevotellamassilia timonensis strain Marseille-P2831 16S ribosomal RNA, partial sequence | NR_144750.1 | 1493 | 358 | 779 | 28.27 | 525.6 | 8E-149 | 382/428 | 89 | 12/428 | 2 | Plus | Plus | Bacteria; Bacteroidetes; Bacteroidia; Bacteroidales; Prevotellaceae; Prevotellamassilia; Prevotellamassilia timonensis | Prevotellamassilia timonensis |
| ASV4514 | 425 | 1 | 1 | 425 | 100 | NR_042074.1 | Eubacterium pyruvativorans strain I-6 16S ribosomal RNA, partial sequence | NR_042074.1 | 1296 | 264 | 667 | 31.17 | 483.1 | 5E-136 | 375/426 | 88 | 23/426 | 5 | Plus | Plus | Bacteria; Firmicutes; Clostridia; Eubacteriales; Eubacteriaceae; Eubacterium; Eubacterium pyruvativorans | Eubacterium pyruvativorans |
| ASV4515 | 426 | 1 | 1 | 426 | 100 | NR_145570.1 | Vibrio mexicanus strain CAIM 1540 16S ribosomal RNA, partial sequence | NR_145570.1 | 1436 | 324 | 750 | 29.74 | 479.4 | 6E-135 | 373/429 | 86 | 5/429 | 1 | Plus | Plus | Bacteria; Proteobacteria; Gammaproteobacteria; Vibrionales; Vibrionaceae; Vibrio; Vibrio mexicanus | Vibrio mexicanus |
| ASV4517 | 423 | 1 | 1 | 423 | 100 | NR_024815.1 | Prevotella shahii strain EHS11 16S ribosomal RNA, partial sequence | NR_024815.1 | 1490 | 356 | 777 | 28.32 | 431.4 | 2E-120 | 362/425 | 85 | 5/425 | 1 | Plus | Plus | Bacteria; Bacteroidetes; Bacteroidia; Bacteroidales; Prevotellaceae; Prevotella; Prevotella shahii | Prevotella shahii |
| ASV4518 | 402 | 3 | 1 | 402 | 100 | NR_114879.1 | Clostridium bowmanii strain DSM 14206 16S ribosomal RNA, partial sequence | NR_114879.1 | 1474 | 346 | 747 | 27.27 | 510.8 | 2E-144 | 362/404 | 89 | 4/404 | 0 | Plus | Plus | Bacteria; Firmicutes; Clostridia; Eubacteriales; Clostridiaceae; Clostridium; Clostridium bowmanii | Clostridium bowmanii |
| ASV4520 | 403 | 3 | 1 | 403 | 100 | NR_041781.1 | Streptococcus alactolyticus strain ATCC 43077 16S ribosomal RNA, partial sequence | NR_041781.1 | 1437 | 346 | 772 | 29.71 | 557 | 3E-158 | 389/427 | 91 | 24/427 | 5 | Plus | Plus | Bacteria; Firmicutes; Bacilli; Lactobacillales; Streptococcaceae; Streptococcus; Streptococcus alactolyticus | Streptococcus alactolyticus |
| ASV4523 | 405 | 2 | 1 | 405 | 100 | NG_041947.1 | Ruminiclostridium cellulolyticum H10 16S ribosomal RNA gene, complete sequence | NG_041947.1 | 1641 | 486 | 890 | 24.68 | 460.9 | 2E-129 | 353/405 | 87 | 0/405 | 0 | Plus | Plus | Bacteria; Firmicutes; Clostridia; Eubacteriales; Oscillospiraceae; Ruminiclostridium; Ruminiclostridium cellulolyticum | Ruminiclostridium cellulolyticum |
| ASV4524 | 402 | 1 | 1 | 402 | 100 | NR_042007.1 | Roseburia inulinivorans DSM 16841 strain A2-194 16S ribosomal RNA, partial sequence | NR_042007.1 | 1454 | 360 | 763 | 27.79 | 484.9 | 1E-136 | 358/405 | 88 | 4/405 | 0 | Plus | Plus | Bacteria; Firmicutes; Clostridia; Eubacteriales; Lachnospiraceae; Roseburia; Roseburia inulinivorans | Roseburia inulinivorans |
| ASV4527 | 405 | 1 | 1 | 405 | 100 | NR_028961.1 | Faecalibacterium prausnitzii strain ATCC 27768 16S ribosomal RNA, partial sequence | NR_028961.1 | 1462 | 344 | 745 | 27.5 | 573.6 | 3E-163 | 376/407 | 92 | 7/407 | 1 | Plus | Plus | Bacteria; Firmicutes; Clostridia; Eubacteriales; Oscillospiraceae; Faecalibacterium; Faecalibacterium prausnitzii | Faecalibacterium prausnitzii |
| ASV4528 | 427 | 4 | 1 | 427 | 100 | NR_041781.1 | Streptococcus alactolyticus strain ATCC 43077 16S ribosomal RNA, partial sequence | NR_041781.1 | 1437 | 346 | 772 | 29.71 | 617.9 | 1E-176 | 400/431 | 92 | 8/431 | 1 | Plus | Plus | Bacteria; Firmicutes; Bacilli; Lactobacillales; Streptococcaceae; Streptococcus; Streptococcus alactolyticus | Streptococcus alactolyticus |
| ASV4533 | 406 | 1 | 1 | 406 | 100 | NR_165727.1 | Anaerotignum faecicola strain KGMB03357 16S ribosomal RNA, complete sequence | NR_165727.1 | 1535 | 375 | 780 | 26.45 | 645.6 | 0 | 388/407 | 95 | 2/407 | 0 | Plus | Plus | Bacteria; Firmicutes; Clostridia; Eubacteriales; Lachnospiraceae; Anaerotignum; Anaerotignum faecicola | Anaerotignum faecicola |
| ASV4534 | 422 | 1 | 1 | 407 | 96.45 | NR_040877.1 | Prevotella copri DSM 18205 strain CB7 16S ribosomal RNA, partial sequence | NR_040877.1 | 1494 | 358 | 764 | 27.24 | 660.4 | 0 | 391/408 | 95 | 2/408 | 0 | Plus | Plus | Bacteria; Bacteroidetes; Bacteroidia; Bacteroidales; Prevotellaceae; Prevotella; Prevotella copri | Prevotella copri |
| ASV4535 | 427 | 2 | 1 | 427 | 100 | NR_133868.1 | Streptococcus phocae subsp. salmonis strain C-4 16S ribosomal RNA, partial sequence | NR_133868.1 | 1410 | 291 | 717 | 30.28 | 551.4 | 1E-156 | 387/430 | 90 | 6/430 | 1 | Plus | Plus | Bacteria; Firmicutes; Bacilli; Lactobacillales; Streptococcaceae; Streptococcus; Streptococcus phocae | Streptococcus phocae |
| ASV4536 | 427 | 2 | 1 | 427 | 100 | NR_025516.1 | Streptococcus ratti strain ATCC 19645 16S ribosomal RNA, partial sequence | NR_025516.1 | 1444 | 347 | 773 | 29.57 | 789.6 | 0 | 427/427 | 100 | 0/427 | 0 | Plus | Plus | Bacteria; Firmicutes; Bacilli; Lactobacillales; Streptococcaceae; Streptococcus; Streptococcus ratti | Streptococcus ratti |
| ASV4537 | 401 | 3 | 1 | 401 | 100 | NR_114338.1 | Parvimonas micra strain JCM 12970 16S ribosomal RNA, partial sequence | NR_114338.1 | 1487 | 364 | 764 | 26.97 | 719.5 | 0 | 397/401 | 99 | 0/401 | 0 | Plus | Plus | Bacteria; Firmicutes; Tissierellia; Tissierellales; Peptoniphilaceae; Parvimonas; Parvimonas micra | Parvimonas micra |
| ASV4538 | 427 | 3 | 1 | 427 | 100 | NR_074906.1 | [Mannheimia] succiniciproducens strain MBEL55E 16S ribosomal RNA, partial sequence | NR_074906.1 | 1543 | 358 | 784 | 27.67 | 712.1 | 0 | 413/427 | 96 | 0/427 | 0 | Plus | Plus | Bacteria; Proteobacteria; Gammaproteobacteria; Pasteurellales; Pasteurellaceae; Basfia; [Mannheimia] succiniciproducens | [Mannheimia] succiniciproducens |
| ASV4539 | 427 | 1 | 1 | 427 | 100 | NR_145928.1 | Pelistega suis strain 3340-03 16S ribosomal RNA, partial sequence | NR_145928.1 | 1424 | 320 | 746 | 29.99 | 784.1 | 0 | 426/427 | 99 | 0/427 | 0 | Plus | Plus | Bacteria; Proteobacteria; Betaproteobacteria; Burkholderiales; Alcaligenaceae; Pelistega; Pelistega suis | Pelistega suis |
| ASV4540 | 427 | 4 | 1 | 427 | 100 | NR_132590.1 | Staphylococcus petrasii subsp. croceilyticus strain MCC10046 16S ribosomal RNA, partial sequence | NR_132590.1 | 1546 | 361 | 787 | 27.62 | 789.6 | 0 | 427/427 | 100 | 0/427 | 0 | Plus | Plus | Bacteria; Firmicutes; Bacilli; Bacillales; Staphylococcaceae; Staphylococcus; Staphylococcus croceilyticus | Staphylococcus croceilyticus |
| ASV4541 | 422 | 1 | 1 | 422 | 100 | NR_113411.1 | Prevotella copri DSM 18205 strain JCM 13464 16S ribosomal RNA, partial sequence | NR_113411.1 | 1493 | 358 | 779 | 28.27 | 581 | 2E-165 | 387/423 | 91 | 2/423 | 0 | Plus | Plus | Bacteria; Bacteroidetes; Bacteroidia; Bacteroidales; Prevotellaceae; Prevotella; Prevotella copri | Prevotella copri |
| ASV4543 | 402 | 1 | 1 | 402 | 100 | NR_144727.1 | Anaeromassilibacillus senegalensis strain mt9 16S ribosomal RNA, partial sequence | NR_144727.1 | 1478 | 352 | 753 | 27.2 | 621.6 | 9E-178 | 382/404 | 94 | 4/404 | 0 | Plus | Plus | Bacteria; Firmicutes; Clostridia; Eubacteriales; Oscillospiraceae; Anaeromassilibacillus; Anaeromassilibacillus senegalensis | Anaeromassilibacillus senegalensis |
| ASV4544 | 424 | 2 | 1 | 424 | 100 | NR_113411.1 | Prevotella copri DSM 18205 strain JCM 13464 16S ribosomal RNA, partial sequence | NR_113411.1 | 1493 | 358 | 779 | 28.27 | 610.5 | 2E-174 | 393/424 | 92 | 2/424 | 0 | Plus | Plus | Bacteria; Bacteroidetes; Bacteroidia; Bacteroidales; Prevotellaceae; Prevotella; Prevotella copri | Prevotella copri |
| ASV4545 | 428 | 4 | 3 | 428 | 99.53 | NR_042802.1 | Lactobacillus ultunensis strain Kx146C1 16S ribosomal RNA, partial sequence | NR_042802.1 | 1550 | 372 | 796 | 27.42 | 569.9 | 4E-162 | 390/429 | 90 | 7/429 | 1 | Plus | Plus | Bacteria; Firmicutes; Bacilli; Lactobacillales; Lactobacillaceae; Lactobacillus; Lactobacillus ultunensis | Lactobacillus ultunensis |
| ASV4546 | 421 | 3 | 1 | 412 | 97.86 | NR_144616.1 | Muribaculum intestinale strain YL27 16S ribosomal RNA, partial sequence | NR_144616.1 | 1460 | 297 | 709 | 28.29 | 429.5 | 6E-120 | 358/418 | 85 | 11/418 | 2 | Plus | Plus | Bacteria; Bacteroidetes; Bacteroidia; Bacteroidales; Muribaculaceae; Muribaculum; Muribaculum intestinale | Muribaculum intestinale |
| ASV4547 | 422 | 2 | 1 | 422 | 100 | NR_113411.1 | Prevotella copri DSM 18205 strain JCM 13464 16S ribosomal RNA, partial sequence | NR_113411.1 | 1493 | 358 | 779 | 28.27 | 608.7 | 7E-174 | 391/422 | 92 | 0/422 | 0 | Plus | Plus | Bacteria; Bacteroidetes; Bacteroidia; Bacteroidales; Prevotellaceae; Prevotella; Prevotella copri | Prevotella copri |
| ASV4549 | 401 | 1 | 1 | 399 | 99.5 | NR_026248.1 | Catonella morbi ATCC 51271 16S ribosomal RNA, partial sequence | NR_026248.1 | 1467 | 345 | 744 | 27.27 | 451.7 | 1E-126 | 348/400 | 87 | 1/400 | 0 | Plus | Plus | Bacteria; Firmicutes; Clostridia; Eubacteriales; Lachnospiraceae; Catonella; Catonella morbi | Catonella morbi |
| ASV4551 | 402 | 3 | 3 | 402 | 99.5 | NR_114728.1 | Clostridium tertium strain ATCC 14573 16S ribosomal RNA, partial sequence | NR_114728.1 | 1415 | 306 | 705 | 28.27 | 545.9 | 6E-155 | 366/401 | 91 | 2/401 | 0 | Plus | Plus | Bacteria; Firmicutes; Clostridia; Eubacteriales; Clostridiaceae; Clostridium; Clostridium tertium | Clostridium tertium |
| ASV4552 | 402 | 1 | 1 | 402 | 100 | NR_029325.1 | Schwartzia succinivorans strain S1-1 16S ribosomal RNA, partial sequence | NR_029325.1 | 1489 | 344 | 769 | 28.61 | 449.9 | 4E-126 | 372/429 | 86 | 30/429 | 6 | Plus | Plus | Bacteria; Firmicutes; Negativicutes; Selenomonadales; Selenomonadaceae; Schwartzia; Schwartzia succinivorans | Schwartzia succinivorans |
| ASV4553 | 402 | 1 | 5 | 402 | 99 | NR_134005.1 | Clostridium bornimense strain M2/40 16S ribosomal RNA, partial sequence | NR_134005.1 | 1363 | 288 | 685 | 29.2 | 564.3 | 2E-160 | 368/399 | 92 | 2/399 | 0 | Plus | Plus | Bacteria; Firmicutes; Clostridia; Eubacteriales; Clostridiaceae; Clostridium; Clostridium bornimense | Clostridium bornimense |
| ASV4554 | 421 | 2 | 1 | 421 | 100 | NR_126195.1 | Butyricimonas paravirosa strain 214-4 16S ribosomal RNA, partial sequence | NR_126195.1 | 1486 | 352 | 772 | 28.33 | 562.5 | 6E-160 | 384/423 | 90 | 4/423 | 0 | Plus | Plus | Bacteria; Bacteroidetes; Bacteroidia; Bacteroidales; Odoribacteraceae; Butyricimonas; Butyricimonas paravirosa | Butyricimonas paravirosa |
| ASV4555 | 419 | 1 | 1 | 419 | 100 | NR_116001.1 | Cellulosilyticum ruminicola JCM 14822 strain H1 16S ribosomal RNA, partial sequence | NR_116001.1 | 1547 | 389 | 792 | 26.12 | 566.2 | 4E-161 | 385/420 | 91 | 17/420 | 4 | Plus | Plus | Bacteria; Firmicutes; Clostridia; Eubacteriales; Lachnospiraceae; Cellulosilyticum; Cellulosilyticum ruminicola | Cellulosilyticum ruminicola |
| ASV4559 | 405 | 1 | 3 | 405 | 99.51 | NR_026476.1 | Succinivibrio dextrinosolvens strain 0554 16S ribosomal RNA, partial sequence | NR_026476.1 | 1463 | 333 | 732 | 27.34 | 464.6 | 2E-130 | 354/404 | 87 | 5/404 | 1 | Plus | Plus | Bacteria; Proteobacteria; Gammaproteobacteria; Aeromonadales; Succinivibrionaceae; Succinivibrio; Succinivibrio dextrinosolvens | Succinivibrio dextrinosolvens |
| ASV4560 | 404 | 2 | 1 | 404 | 100 | NR_113185.1 | Pyramidobacter piscolens strain W5455 16S ribosomal RNA, partial sequence | NR_113185.1 | 1484 | 345 | 748 | 27.22 | 542.2 | 7E-154 | 371/408 | 90 | 8/408 | 1 | Plus | Plus | Bacteria; Synergistetes; Synergistia; Synergistales; Synergistaceae; Pyramidobacter; Pyramidobacter piscolens | Pyramidobacter piscolens |
| ASV4563 | 402 | 1 | 1 | 402 | 100 | NR_074634.1 | [Eubacterium] rectale ATCC 33656 16S ribosomal RNA, partial sequence | NR_074634.1 | 1521 | 367 | 768 | 26.43 | 566.2 | 4E-161 | 370/402 | 92 | 0/402 | 0 | Plus | Plus | Bacteria; Firmicutes; Clostridia; Eubacteriales; Lachnospiraceae; ; [Eubacterium] rectale | [Eubacterium] rectale |
| ASV4564 | 427 | 4 | 5 | 427 | 99.06 | NR_042802.1 | Lactobacillus ultunensis strain Kx146C1 16S ribosomal RNA, partial sequence | NR_042802.1 | 1550 | 374 | 796 | 27.29 | 510.8 | 2E-144 | 379/428 | 88 | 10/428 | 2 | Plus | Plus | Bacteria; Firmicutes; Bacilli; Lactobacillales; Lactobacillaceae; Lactobacillus; Lactobacillus ultunensis | Lactobacillus ultunensis |
| ASV4565 | 402 | 3 | 1 | 402 | 100 | NR_117139.2 | Anaerostipes hadrus strain DSM 3319 16S ribosomal RNA, partial sequence | NR_117139.2 | 1521 | 363 | 764 | 26.43 | 616.1 | 4E-176 | 380/403 | 94 | 2/403 | 0 | Plus | Plus | Bacteria; Firmicutes; Clostridia; Eubacteriales; Lachnospiraceae; Anaerostipes; Anaerostipes hadrus | Anaerostipes hadrus |
| ASV4566 | 402 | 3 | 3 | 402 | 99.5 | NR_117131.1 | Faecalicatena orotica strain DSM 1287 16S ribosomal RNA, partial sequence | NR_117131.1 | 1526 | 367 | 766 | 26.21 | 518.2 | 1E-146 | 360/400 | 90 | 0/400 | 0 | Plus | Plus | Bacteria; Firmicutes; Clostridia; Eubacteriales; Lachnospiraceae; Faecalicatena; Faecalicatena orotica | Faecalicatena orotica |
| ASV4567 | 427 | 1 | 4 | 427 | 99.3 | NR_146687.1 | Breznakia pachnodae strain Pei061 16S ribosomal RNA, partial sequence | NR_146687.1 | 1499 | 370 | 792 | 28.22 | 455.4 | 1E-127 | 367/426 | 86 | 5/426 | 1 | Plus | Plus | Bacteria; Firmicutes; Erysipelotrichia; Erysipelotrichales; Erysipelotrichaceae; Breznakia; Breznakia pachnodae | Breznakia pachnodae |
| ASV4568 | 404 | 1 | 1 | 404 | 100 | NR_116001.1 | Cellulosilyticum ruminicola JCM 14822 strain H1 16S ribosomal RNA, partial sequence | NR_116001.1 | 1547 | 389 | 792 | 26.12 | 575.4 | 7E-164 | 374/405 | 92 | 2/405 | 0 | Plus | Plus | Bacteria; Firmicutes; Clostridia; Eubacteriales; Lachnospiraceae; Cellulosilyticum; Cellulosilyticum ruminicola | Cellulosilyticum ruminicola |
| ASV4569 | 405 | 2 | 1 | 405 | 100 | NR_156911.1 | Fournierella massiliensis strain AT2 16S ribosomal RNA, partial sequence | NR_156911.1 | 1491 | 368 | 769 | 26.96 | 518.2 | 1E-146 | 364/405 | 89 | 3/405 | 0 | Plus | Plus | Bacteria; Firmicutes; Clostridia; Eubacteriales; Oscillospiraceae; Fournierella; Fournierella massiliensis | Fournierella massiliensis |
| ASV4574 | 402 | 2 | 1 | 402 | 100 | NR_148837.1 | Abyssivirga alkaniphila strain L81 16S ribosomal RNA, partial sequence | NR_148837.1 | 1520 | 364 | 765 | 26.45 | 472 | 1E-132 | 357/406 | 87 | 8/406 | 1 | Plus | Plus | Bacteria; Firmicutes; Clostridia; Eubacteriales; Lachnospiraceae; Abyssivirga; Abyssivirga alkaniphila | Abyssivirga alkaniphila |
| ASV4575 | 405 | 1 | 1 | 405 | 100 | NR_044425.1 | Hydrogenoanaerobacterium saccharovorans strain SW512 16S ribosomal RNA, partial sequence | NR_044425.1 | 1510 | 363 | 764 | 26.62 | 490.5 | 3E-138 | 361/407 | 88 | 7/407 | 1 | Plus | Plus | Bacteria; Firmicutes; Clostridia; Eubacteriales; Oscillospiraceae; Hydrogenoanaerobacterium; Hydrogenoanaerobacterium saccharovorans | [illegible] |
| ASV4577 | 402 | 1 | 1 | 382 | 95.02 | NR_118674.1 | Eubacterium ramulus ATCC 29099 16S ribosomal RNA, partial sequence | NR_118674.1 | 1474 | 373 | 754 | 25.92 | 436.9 | 3E-122 | 333/384 | 86 | 4/384 | 1 | Plus | Plus | Bacteria; Firmicutes; Clostridia; Eubacteriales; Eubacteriaceae; Eubacterium; Eubacterium ramulus | Eubacterium ramulus |
| ASV4578 | 426 | 1 | 1 | 425 | 99.77 | NR_029325.1 | Schwartzia succinivorans strain S1-1 16S ribosomal RNA, partial sequence | NR_029325.1 | 1489 | 344 | 768 | 28.54 | 769.3 | 0 | 422/425 | 99 | 0/425 | 0 | Plus | Plus | Bacteria; Firmicutes; Negativicutes; Selenomonadales; Selenomonadaceae; Schwartzia; Schwartzia succinivorans | Schwartzia succinivorans |
| ASV4581 | 422 | 2 | 1 | 422 | 100 | NR_025300.1 | Prevotella albensis strain M384 16S ribosomal RNA, partial sequence | NR_025300.1 | 1495 | 358 | 779 | 28.23 | 614.2 | 2E-175 | 393/423 | 92 | 2/423 | 0 | Plus | Plus | Bacteria; Bacteroidetes; Bacteroidia; Bacteroidales; Prevotellaceae; Prevotella; Prevotella albensis | Prevotella albensis |
| ASV4582 | 402 | 1 | 1 | 402 | 100 | NR_029355.1 | [Clostridium] methylpentosum strain R2 16S ribosomal RNA, partial sequence | NR_029355.1 | 1435 | 349 | 750 | 28.01 | 560.7 | 2E-159 | 369/402 | 91 | 0/402 | 0 | Plus | Plus | Bacteria; Firmicutes; Clostridia; Eubacteriales; Oscillospiraceae; ; [Clostridium] methylpentosum | [Clostridium] methylpentosum |
| ASV4584 | 405 | 2 | 1 | 405 | 100 | NR_074629.1 | Hungateiclostridium thermocellum strain ATCC 27405 16S ribosomal RNA, partial sequence | NR_074629.1 | 1521 | 358 | 762 | 26.63 | 549.6 | 4E-156 | 369/405 | 91 | 0/405 | 0 | Plus | Plus | Bacteria; Firmicutes; Clostridia; Eubacteriales; Oscillospiraceae; Acetivibrio; Acetivibrio thermocellus | Acetivibrio thermocellus |
| ASV4589 | 426 | 2 | 1 | 426 | 100 | NR_113306.1 | Megasphaera elsdenii DSM 20460 16S ribosomal RNA, partial sequence | NR_113306.1 | 1174 | 232 | 658 | 36.37 | 551.4 | 1E-156 | 385/428 | 89 | 3/428 | 0 | Plus | Plus | Bacteria; Firmicutes; Negativicutes; Veillonellales; Veillonellaceae; Megasphaera; Megasphaera elsdenii | Megasphaera elsdenii |
| ASV4592 | 427 | 1 | 1 | 426 | 99.77 | NR_028840.1 | Mitsuokella jalaludinii strain M9 16S ribosomal RNA, partial sequence | NR_028840.1 | 1543 | 362 | 787 | 27.61 | 765.6 | 0 | 422/426 | 99 | 0/426 | 0 | Plus | Plus | Bacteria; Firmicutes; Negativicutes; Selenomonadales; Selenomonadaceae; Mitsuokella; Mitsuokella jalaludinii | Mitsuokella jalaludinii |
| ASV4593 | 405 | 1 | 1 | 405 | 100 | NR_144742.1 | Christensenella massiliensis strain Marseille-P2438 16S ribosomal RNA, partial sequence | NR_144742.1 | 1499 | 369 | 773 | 27.02 | 416.6 | 5E-116 | 346/406 | 85 | 2/406 | 0 | Plus | Plus | Bacteria; Firmicutes; Clostridia; Eubacteriales; Christensenellaceae; Christensenella; Christensenella massiliensis | Christensenella massiliensis |
| ASV4594 | 422 | 1 | 1 | 422 | 100 | NR_041342.1 | Parabacteroides distasonis strain JCM 5825 16S ribosomal RNA, partial sequence | NR_041342.1 | 1488 | 353 | 774 | 28.36 | 553.3 | 4E-157 | 383/424 | 90 | 4/424 | 0 | Plus | Plus | Bacteria; Bacteroidetes; Bacteroidia; Bacteroidales; Tannerellaceae; Parabacteroides; Parabacteroides distasonis | Parabacteroides distasonis |
| ASV4595 | 427 | 1 | 1 | 427 | 100 | NR_028840.1 | Mitsuokella jalaludinii strain M9 16S ribosomal RNA, partial sequence | NR_028840.1 | 1543 | 362 | 788 | 27.67 | 745.3 | 0 | 419/427 | 98 | 0/427 | 0 | Plus | Plus | Bacteria; Firmicutes; Negativicutes; Selenomonadales; Selenomonadaceae; Mitsuokella; Mitsuokella jalaludinii | Mitsuokella jalaludinii |
| ASV4596 | 404 | 1 | 1 | 404 | 100 | NR_114789.1 | [Clostridium] leptum strain DSM 753 16S ribosomal RNA, partial sequence | NR_114789.1 | 1505 | 364 | 765 | 26.71 | 601.3 | 1E-171 | 378/404 | 93 | 2/404 | 0 | Plus | Plus | Bacteria; Firmicutes; Clostridia; Eubacteriales; Oscillospiraceae; ; [Clostridium] leptum | [Clostridium] leptum |
| ASV4597 | 405 | 2 | 1 | 405 | 100 | NR_118554.1 | Intestinimonas butyriciproducens strain SRB-521-5-I 16S ribosomal RNA, partial sequence | NR_118554.1 | 1475 | 346 | 750 | 27.46 | 582.8 | 4E-166 | 375/405 | 92 | 0/405 | 0 | Plus | Plus | Bacteria; Firmicutes; Clostridia; Eubacteriales; ; Intestinimonas; Intestinimonas butyriciproducens | Intestinimonas butyriciproducens |
| ASV4599 | 427 | 1 | 1 | 426 | 99.77 | NR_112902.1 | Phascolarctobacterium succinatutens strain YIT 12067 16S ribosomal RNA, partial sequence | NR_112902.1 | 1610 | 444 | 869 | 26.46 | 599.4 | 4E-171 | 392/426 | 92 | 0/426 | 0 | Plus | Plus | Bacteria; Firmicutes; Negativicutes; Acidaminococcales; Acidaminococcaceae; Phascolarctobacterium; Phascolarctobacterium succinatutens | Phascolarctobacterium succinatutens |
| ASV4600 | 427 | 1 | 1 | 427 | 100 | NR_147381.1 | Duodenibacillus massiliensis strain Marseille-P2968 16S ribosomal RNA, partial sequence | NR_147381.1 | 1497 | 349 | 775 | 28.52 | 678.8 | 0 | 410/430 | 95 | 6/430 | 1 | Plus | Plus | Bacteria; Proteobacteria; Betaproteobacteria; Burkholderiales; Sutterellaceae; Duodenibacillus; Duodenibacillus massiliensis | Duodenibacillus massiliensis |
| ASV4605 | 405 | 1 | 1 | 405 | 100 | NR_144611.1 | Flintibacter butyricus strain BLS21 16S ribosomal RNA, partial sequence | NR_144611.1 | 808 | 299 | 703 | 50.12 | 671.5 | 0 | 391/405 | 96 | 0/405 | 0 | Plus | Plus | Bacteria; Firmicutes; Clostridia; Eubacteriales; ; Flintibacter; Flintibacter butyricus | Flintibacter butyricus |
| ASV4606 | 427 | 1 | 1 | 426 | 99.77 | NR_102980.1 | Megasphaera elsdenii DSM 20460 16S ribosomal RNA, partial sequence | NR_102980.1 | 1552 | 369 | 794 | 27.45 | 660.4 | 0 | 403/426 | 94 | 0/426 | 0 | Plus | Plus | Bacteria; Firmicutes; Negativicutes; Veillonellales; Veillonellaceae; Megasphaera; Megasphaera elsdenii | Megasphaera elsdenii |
| ASV4609 | 421 | 1 | 1 | 421 | 100 | NR_144616.1 | Muribaculum intestinale strain YL27 16S ribosomal RNA, partial sequence | NR_144616.1 | 1460 | 297 | 718 | 28.9 | 479.4 | 6E-135 | 369/423 | 87 | 3/423 | 0 | Plus | Plus | Bacteria; Bacteroidetes; Bacteroidia; Bacteroidales; Muribaculaceae; Muribaculum; Muribaculum intestinale | Muribaculum intestinale |
| ASV4611 | 421 | 1 | 1 | 420 | 99.76 | NR_171489.1 | Coraliomargarita sinensis strain WN38 16S ribosomal RNA, partial sequence | NR_171489.1 | 1542 | 367 | 787 | 27.3 | 438.8 | 1E-122 | 364/425 | 85 | 9/425 | 2 | Plus | Plus | Bacteria; Verrucomicrobia; Opitutae; Puniceicoccales; Puniceicoccaceae; Coraliomargarita; Coraliomargarita sinensis | Coraliomargarita sinensis |
| ASV4612 | 402 | 1 | 1 | 402 | 100 | NR_044386.1 | Clostridium amylolyticum strain SW408 16S ribosomal RNA, partial sequence | NR_044386.1 | 1505 | 348 | 749 | 26.71 | 516.3 | 4E-146 | 361/402 | 89 | 0/402 | 0 | Plus | Plus | Bacteria; Firmicutes; Clostridia; Eubacteriales; Clostridiaceae; Clostridium; Clostridium amylolyticum | Clostridium amylolyticum |
| ASV4613 | 402 | 2 | 1 | 402 | 100 | NR_133762.1 | Hungatella effluvii strain UB-B.2 16S ribosomal RNA, partial sequence | NR_133762.1 | 1409 | 318 | 719 | 28.53 | 560.7 | 2E-159 | 371/404 | 91 | 4/404 | 0 | Plus | Plus | Bacteria; Firmicutes; Clostridia; Eubacteriales; Clostridiaceae; Hungatella; Hungatella effluvii | Hungatella effluvii |
| ASV4614 | 404 | 2 | 1 | 404 | 100 | NR_113306.1 | Megasphaera elsdenii DSM 20460 16S ribosomal RNA, partial sequence | NR_113306.1 | 1174 | 232 | 658 | 36.37 | 551.4 | 1E-156 | 389/428 | 90 | 25/428 | 5 | Plus | Plus | Bacteria; Firmicutes; Negativicutes; Veillonellales; Veillonellaceae; Megasphaera; Megasphaera elsdenii | Megasphaera elsdenii |
| ASV4615 | 427 | 1 | 3 | 427 | 99.53 | NR_147405.1 | Mailhella massiliensis strain Marseille-P3199 16S ribosomal RNA, partial sequence | NR_147405.1 | 1511 | 357 | 781 | 28.13 | 503.4 | 4E-142 | 376/427 | 88 | 4/427 | 0 | Plus | Plus | Bacteria; Proteobacteria; Deltaproteobacteria; Desulfovibrionales; Desulfovibrionaceae; Mailhella; Mailhella massiliensis | Mailhella massiliensis |
| ASV4616 | 427 | 1 | 1 | 427 | 100 | NR_112902.1 | Phascolarctobacterium succinatutens strain YIT 12067 16S ribosomal RNA, partial sequence | NR_112902.1 | 1610 | 444 | 870 | 26.52 | 562.5 | 6E-160 | 388/429 | 90 | 4/429 | 0 | Plus | Plus | Bacteria; Firmicutes; Negativicutes; Acidaminococcales; Acidaminococcaceae; Phascolarctobacterium; Phascolarctobacterium succinatutens | Phascolarctobacterium succinatutens |
| ASV4617 | 404 | 1 | 1 | 404 | 100 | NR_115340.1 | Saccharofermentans acetigenes strain P6 16S ribosomal RNA, partial sequence | NR_115340.1 | 1510 | 353 | 757 | 26.82 | 409.2 | 8E-114 | 347/408 | 85 | 7/408 | 1 | Plus | Plus | Bacteria; Firmicutes; Clostridia; Eubacteriales; Oscillospiraceae; Saccharofermentans; Saccharofermentans acetigenes | Saccharofermentans acetigenes |
| ASV4618 | 422 | 1 | 1 | 422 | 100 | NR_113411.1 | Prevotella copri DSM 18205 strain JCM 13464 16S ribosomal RNA, partial sequence | NR_113411.1 | 1493 | 358 | 779 | 28.27 | 592 | 7E-169 | 389/423 | 91 | 2/423 | 0 | Plus | Plus | Bacteria; Bacteroidetes; Bacteroidia; Bacteroidales; Prevotellaceae; Prevotella; Prevotella copri | Prevotella copri |
| ASV4622 | 422 | 1 | 1 | 422 | 100 | NR_113411.1 | Prevotella copri DSM 18205 strain JCM 13464 16S ribosomal RNA, partial sequence | NR_113411.1 | 1493 | 358 | 779 | 28.27 | 658.5 | 0 | 401/423 | 94 | 2/423 | 0 | Plus | Plus | Bacteria; Bacteroidetes; Bacteroidia; Bacteroidales; Prevotellaceae; Prevotella; Prevotella copri | Prevotella copri |
| ASV4624 | 402 | 1 | 1 | 402 | 100 | NR_144749.1 | Ihubacter massiliensis strain Marseille-P2843 16S ribosomal RNA, partial sequence | NR_144749.1 | 1479 | 364 | 766 | 27.25 | 516.3 | 4E-146 | 362/403 | 89 | 1/403 | 0 | Plus | Plus | Bacteria; Firmicutes; Clostridia; Eubacteriales; Clostridiales Family XIII. Incertae Sedis; Ihubacter; Ihubacter massiliensis | Ihubacter massiliensis |
| ASV4625 | 421 | 1 | 1 | 421 | 100 | NR_144750.1 | Prevotellamassilia timonensis strain Marseille-P2831 16S ribosomal RNA, partial sequence | NR_144750.1 | 1493 | 358 | 779 | 28.27 | 446.2 | 6E-125 | 366/426 | 85 | 9/426 | 2 | Plus | Plus | Bacteria; Bacteroidetes; Bacteroidia; Bacteroidales; Prevotellaceae; Prevotellamassilia; Prevotellamassilia timonensis | Prevotellamassilia timonensis |
| ASV4626 | 427 | 1 | 1 | 427 | 100 | NR_112902.1 | Phascolarctobacterium succinatutens strain YIT 12067 16S ribosomal RNA, partial sequence | NR_112902.1 | 1610 | 444 | 870 | 26.52 | 573.6 | 3E-163 | 390/429 | 90 | 4/429 | 0 | Plus | Plus | Bacteria; Firmicutes; Negativicutes; Acidaminococcales; Acidaminococcaceae; Phascolarctobacterium; Phascolarctobacterium succinatutens | Phascolarctobacterium succinatutens |
| ASV4627 | 405 | 1 | 1 | 405 | 100 | NR_025670.1 | Pseudoflavonifractor capillosus ATCC 29799 16S ribosomal RNA, partial sequence | NR_025670.1 | 1483 | 346 | 750 | 27.31 | 521.9 | 9E-148 | 364/405 | 89 | 0/405 | 0 | Plus | Plus | Bacteria; Firmicutes; Clostridia; Eubacteriales; Oscillospiraceae; Pseudoflavonifractor; Pseudoflavonifractor capillosus | Pseudoflavonifractor capillosus |

| | | | | | | | | | | | | | | | | | | | | | | | | | | | | |
|---|---|---|---|---|---|---|---|---|---|---|---|---|---|---|---|---|---|---|---|---|---|---|---|---|---|---|---|---|
| ASV4628 | 421 | 1 | 1 | 421 | 100 | NR_041342.1 | Parabacteroides distasonis strain JCM 5825 16S ribosomal RNA, partial sequence | NR_041342.1 | 1488 | 353 | 774 | 28.36 | 523.7 | 3E-148 | 378/424 | 89 | 5/424 | 1 | Plus | Plus | Bacteria; Bacteroidetes; Bacteroidia; Bacteroidales; Tannerellaceae; Parabacteroides; Parabacteroides distasonis | Parabacteroides distasonis | Parabacteroides | Tannerellaceae | Bacteroidales | Bacteroidia | Bacteroidetes | Bacteria |
| ASV4631 | 421 | 2 | 1 | 421 | 100 | NR_113076.1 | Parabacteroides goldsteinii DSM 19448 = WAL 12034 strain JCM 13446 16S ribosomal RNA, partial sequence | NR_113076.1 | 1476 | 341 | 762 | 28.59 | 512.6 | 6E-145 | 375/423 | 88 | 3/423 | 0 | Plus | Plus | Bacteria; Bacteroidetes; Bacteroidia; Bacteroidales; Tannerellaceae; Parabacteroides; Parabacteroides goldsteinii | Parabacteroides goldsteinii | Parabacteroides | Tannerellaceae | Bacteroidales | Bacteroidia | Bacteroidetes | Bacteria |
| ASV4632 | 402 | 1 | 1 | 402 | 100 | NR_029355.1 | [Clostridium] methylpentosum strain R2 16S ribosomal RNA, partial sequence | NR_029355.1 | 1435 | 349 | 750 | 28.01 | 494.2 | 2E-139 | 358/403 | 88 | 2/403 | 0 | Plus | Plus | Bacteria; Firmicutes; Clostridia; Eubacteriales; Oscillospiraceae; ; [Clostridium] methylpentosum | [Clostridium] methylpentosum | | Oscillospiraceae | Eubacteriales | Clostridia | Firmicutes | Bacteria |
| ASV4633 | 403 | 1 | 3 | 403 | 99.5 | NR_125677.1 | Vallitalea pronyensis strain FatNI3 16S ribosomal RNA, partial sequence | NR_125677.1 | 1480 | 365 | 764 | 27.03 | 457.2 | 3E-128 | 351/402 | 87 | 3/402 | 0 | Plus | Plus | Bacteria; Firmicutes; Clostridia; Eubacteriales; Vallitaleaceae; Vallitalea; Vallitalea pronyensis | Vallitalea pronyensis | Vallitalea | Vallitaleaceae | Eubacteriales | Clostridia | Firmicutes | Bacteria |
| ASV4634 | 426 | 1 | 3 | 426 | 99.53 | NR_041304.1 | Sharpea azabuensis strain ST18 16S ribosomal RNA, partial sequence | NR_041304.1 | 1460 | 327 | 750 | 29.04 | 507.1 | 3E-143 | 378/428 | 88 | 8/428 | 1 | Plus | Plus | Bacteria; Firmicutes; Erysipelotrichia; Erysipelotrichales; Coprobacillaceae; Sharpea; Sharpea azabuensis | Sharpea azabuensis | Sharpea | Coprobacillaceae | Erysipelotrichales | Erysipelotrichia | Firmicutes | Bacteria |
| ASV4635 | 402 | 1 | 1 | 402 | 100 | NR_137216.1 | Brassicibacter thermophilus strain Cel2f 16S ribosomal RNA, partial sequence | NR_137216.1 | 1408 | 321 | 721 | 28.48 | 520 | 3E-147 | 362/402 | 90 | 1/402 | 0 | Plus | Plus | Bacteria; Firmicutes; Clostridia; Eubacteriales; Clostridiaceae; Brassicibacter; Brassicibacter thermophilus | Brassicibacter thermophilus | Brassicibacter | Clostridiaceae | Eubacteriales | Clostridia | Firmicutes | Bacteria |
| ASV4638 | 402 | 1 | 1 | 402 | 100 | NR_118554.1 | Intestinimonas butyriciproducens strain SRB-521-5-I 16S ribosomal RNA, partial sequence | NR_118554.1 | 1475 | 346 | 750 | 27.46 | 595.7 | 5E-170 | 381/408 | 93 | 9/408 | 2 | Plus | Plus | Bacteria; Firmicutes; Clostridia; Eubacteriales; ; Intestinimonas; Intestinimonas butyriciproducens | Intestinimonas butyriciproducens | Intestinimonas | | Eubacteriales | Clostridia | Firmicutes | Bacteria |
| ASV4640 | 403 | 1 | 1 | 403 | 100 | NR_104907.1 | Eubacterium coprostanoligenes strain HL 16S ribosomal RNA, partial sequence | NR_104907.1 | 1446 | 332 | 734 | 27.87 | 623.4 | 3E-178 | 382/404 | 94 | 2/404 | 0 | Plus | Plus | Bacteria; Firmicutes; Clostridia; Eubacteriales; Eubacteriaceae; Eubacterium; Eubacterium coprostanoligenes | Eubacterium coprostanoligenes | Eubacterium | Eubacteriaceae | Eubacteriales | Clostridia | Firmicutes | Bacteria |
| ASV4642 | 427 | 1 | 4 | 427 | 99.3 | NR_146687.1 | Breznakia pachnodae strain Pei061 16S ribosomal RNA, partial sequence | NR_146687.1 | 1499 | 370 | 792 | 28.22 | 433.2 | 5E-121 | 364/427 | 85 | 7/427 | 1 | Plus | Plus | Bacteria; Firmicutes; Erysipelotrichia; Erysipelotrichales; Erysipelotrichaceae; Breznakia; Breznakia pachnodae | Breznakia pachnodae | Breznakia | Erysipelotrichaceae | Erysipelotrichales | Erysipelotrichia | Firmicutes | Bacteria |
| ASV4645 | 405 | 2 | 1 | 405 | 100 | NR_024948.1 | Campylobacter hyointestinalis subsp. lawsonii strain CHY5 16S ribosomal RNA, partial sequence | NR_024948.1 | 1419 | 318 | 720 | 28.4 | 608.7 | 7E-174 | 380/405 | 93 | 2/405 | 0 | Plus | Plus | Bacteria; Proteobacteria; Epsilonproteobacteria; Campylobacterales; Campylobacteraceae; Campylobacter; Campylobacter hyointestinalis | Campylobacter hyointestinalis | Campylobacter | Campylobacteraceae | Campylobacterales | Epsilonproteobacteria | Proteobacteria | Bacteria |
| ASV4646 | 405 | 1 | 1 | 405 | 100 | NR_118554.1 | Intestinimonas butyriciproducens strain SRB-521-5-I 16S ribosomal RNA, partial sequence | NR_118554.1 | 1475 | 346 | 750 | 27.46 | 483.1 | 4E-136 | 360/408 | 88 | 6/408 | 1 | Plus | Plus | Bacteria; Firmicutes; Clostridia; Eubacteriales; ; Intestinimonas; Intestinimonas butyriciproducens | Intestinimonas butyriciproducens | Intestinimonas | | Eubacteriales | Clostridia | Firmicutes | Bacteria |
| ASV4647 | 426 | 1 | 1 | 426 | 100 | NR_114696.1 | Anaerovibrio lipolyticus DSM 3074 16S ribosomal RNA, partial sequence | NR_114696.1 | 1526 | 372 | 797 | 27.92 | 523.7 | 3E-148 | 380/429 | 88 | 6/429 | 1 | Plus | Plus | Bacteria; Firmicutes; Negativicutes; Selenomonadales; Selenomonadaceae; Anaerovibrio; Anaerovibrio lipolyticus | Anaerovibrio lipolyticus | Anaerovibrio | Selenomonadaceae | Selenomonadales | Negativicutes | Firmicutes | Bacteria |
| ASV4648 | 403 | 1 | 1 | 403 | 100 | NR_026315.1 | Pseudobutyrivibrio ruminis strain DSM 9787 16S ribosomal RNA, partial sequence | NR_026315.1 | 1462 | 334 | 735 | 27.5 | 549.6 | 4E-156 | 370/405 | 91 | 5/405 | 1 | Plus | Plus | Bacteria; Firmicutes; Clostridia; Eubacteriales; Lachnospiraceae; Pseudobutyrivibrio; Pseudobutyrivibrio ruminis | Pseudobutyrivibrio ruminis | Pseudobutyrivibrio | Lachnospiraceae | Eubacteriales | Clostridia | Firmicutes | Bacteria |
| ASV4649 | 402 | 2 | 1 | 402 | 100 | NR_027557.1 | Roseburia intestinalis L1-82 16S ribosomal RNA, partial sequence | NR_027557.1 | 1482 | 360 | 763 | 27.26 | 612.4 | 5E-175 | 381/405 | 94 | 4/405 | 0 | Plus | Plus | Bacteria; Firmicutes; Clostridia; Eubacteriales; Lachnospiraceae; Roseburia; Roseburia intestinalis | Roseburia intestinalis | Roseburia | Lachnospiraceae | Eubacteriales | Clostridia | Firmicutes | Bacteria |
| ASV4650 | 422 | 1 | 1 | 422 | 100 | NR_041342.1 | Parabacteroides distasonis strain JCM 5825 16S ribosomal RNA, partial sequence | NR_041342.1 | 1488 | 353 | 774 | 28.36 | 558.8 | 8E-159 | 382/422 | 90 | 0/422 | 0 | Plus | Plus | Bacteria; Bacteroidetes; Bacteroidia; Bacteroidales; Tannerellaceae; Parabacteroides; Parabacteroides distasonis | Parabacteroides distasonis | Parabacteroides | Tannerellaceae | Bacteroidales | Bacteroidia | Bacteroidetes | Bacteria |
| ASV4651 | 404 | 2 | 1 | 404 | 100 | NR_113185.1 | Pyramidobacter piscolens strain W5455 16S ribosomal RNA, partial sequence | NR_113185.1 | 1484 | 345 | 748 | 27.22 | 536.6 | 3E-152 | 370/408 | 90 | 8/408 | 1 | Plus | Plus | Bacteria; Synergistetes; Synergistia; Synergistales; Synergistaceae; Pyramidobacter; Pyramidobacter piscolens | Pyramidobacter piscolens | Pyramidobacter | Synergistaceae | Synergistales | Synergistia | Synergistetes | Bacteria |
| ASV4652 | 402 | 2 | 1 | 402 | 100 | NR_156080.1 | Kineothrix alysoides strain KNHs209 16S ribosomal RNA, partial sequence | NR_156080.1 | 1410 | 353 | 754 | 28.51 | 693.6 | 0 | 393/402 | 97 | 0/402 | 0 | Plus | Plus | Bacteria; Firmicutes; Clostridia; Eubacteriales; Lachnospiraceae; Kineothrix; Kineothrix alysoides | Kineothrix alysoides | Kineothrix | Lachnospiraceae | Eubacteriales | Clostridia | Firmicutes | Bacteria |
| ASV4654 | 405 | 3 | 1 | 405 | 100 | NR_152095.1 | Herbinix luporum strain SD1D 16S ribosomal RNA, partial sequence | NR_152095.1 | 1521 | 365 | 766 | 26.43 | 523.7 | 3E-148 | 365/405 | 90 | 3/405 | 0 | Plus | Plus | Bacteria; Firmicutes; Clostridia; Eubacteriales; Lachnospiraceae; Herbinix; Herbinix luporum | Herbinix luporum | Herbinix | Lachnospiraceae | Eubacteriales | Clostridia | Firmicutes | Bacteria |
| ASV4656 | 401 | 1 | 1 | 401 | 100 | NR_144740.1 | Romboutsia timonensis strain DR1 16S ribosomal RNA, partial sequence | NR_144740.1 | 1466 | 338 | 738 | 27.35 | 603.1 | 3E-172 | 376/401 | 93 | 0/401 | 0 | Plus | Plus | Bacteria; Firmicutes; Clostridia; Eubacteriales; Peptostreptococcaceae; Romboutsia; Romboutsia timonensis | Romboutsia timonensis | Romboutsia | Peptostreptococcaceae | Eubacteriales | Clostridia | Firmicutes | Bacteria |
| ASV4657 | 405 | 1 | 1 | 405 | 100 | NR_025931.1 | Ruminococcus flavefaciens strain C94 16S ribosomal RNA, partial sequence | NR_025931.1 | 1388 | 325 | 726 | 28.96 | 601.3 | 1E-171 | 379/405 | 93 | 3/405 | 0 | Plus | Plus | Bacteria; Firmicutes; Clostridia; Eubacteriales; Oscillospiraceae; Ruminococcus; Ruminococcus flavefaciens | Ruminococcus flavefaciens | Ruminococcus | Oscillospiraceae | Eubacteriales | Clostridia | Firmicutes | Bacteria |
| ASV4660 | 422 | 1 | 1 | 422 | 100 | NR_113153.1 | Alistipes shahii strain JCM 16773 16S ribosomal RNA, partial sequence | NR_113153.1 | 1486 | 351 | 772 | 28.4 | 774.9 | 0 | 421/422 | 99 | 0/422 | 0 | Plus | Plus | Bacteria; Bacteroidetes; Bacteroidia; Bacteroidales; Rikenellaceae; Alistipes; Alistipes shahii | Alistipes shahii | Alistipes | Rikenellaceae | Bacteroidales | Bacteroidia | Bacteroidetes | Bacteria |
| ASV4661 | 408 | 1 | 1 | 408 | 100 | NR_029167.1 | Anaeroplasma abactoclasticum strain 6-1 16S ribosomal RNA, partial sequence | NR_029167.1 | 1446 | 344 | 751 | 28.22 | 579.1 | 6E-165 | 379/411 | 92 | 6/411 | 1 | Plus | Plus | Bacteria; Tenericutes; Mollicutes; Anaeroplasmatales; Anaeroplasmataceae; Anaeroplasma; Anaeroplasma abactoclasticum | Anaeroplasma abactoclasticum | Anaeroplasma | Anaeroplasmataceae | Anaeroplasmatales | Mollicutes | Tenericutes | Bacteria |
| ASV4662 | 401 | 1 | 1 | 400 | 99.75 | NR_158111.1 | Paludicola psychrotolerans strain NC1253 16S ribosomal RNA, partial sequence | NR_158111.1 | 1490 | 366 | 765 | 26.85 | 606.8 | 3E-173 | 377/401 | 94 | 2/401 | 0 | Plus | Plus | Bacteria; Firmicutes; Clostridia; Eubacteriales; Oscillospiraceae; Paludicola; Paludicola psychrotolerans | Paludicola psychrotolerans | Paludicola | Oscillospiraceae | Eubacteriales | Clostridia | Firmicutes | Bacteria |
| ASV4665 | 401 | 1 | 1 | 399 | 99.5 | NR_036928.1 | Hungatella hathewayi strain 1313 16S ribosomal RNA, partial sequence | NR_036928.1 | 1431 | 350 | 749 | 27.95 | 460.9 | 2E-129 | 351/401 | 87 | 3/401 | 0 | Plus | Plus | Bacteria; Firmicutes; Clostridia; Eubacteriales; Clostridiaceae; Hungatella; Hungatella hathewayi | Hungatella hathewayi | Hungatella | Clostridiaceae | Eubacteriales | Clostridia | Firmicutes | Bacteria |
| ASV4666 | 402 | 1 | 1 | 402 | 100 | NR_118667.1 | Lachnospira pectinoschiza strain 150-1 16S ribosomal RNA, partial sequence | NR_118667.1 | 1465 | 372 | 773 | 27.44 | 640.1 | 0 | 381/402 | 94 | 0/402 | 0 | Plus | Plus | Bacteria; Firmicutes; Clostridia; Eubacteriales; Lachnospiraceae; Lachnospira; Lachnospira pectinoschiza | Lachnospira pectinoschiza | Lachnospira | Lachnospiraceae | Eubacteriales | Clostridia | Firmicutes | Bacteria |
| ASV4667 | 404 | 1 | 1 | 404 | 100 | NR_074634.1 | [Eubacterium] rectale ATCC 33656 16S ribosomal RNA, partial sequence | NR_074634.1 | 1521 | 367 | 768 | 26.43 | 606.8 | 3E-173 | 379/404 | 93 | 2/404 | 0 | Plus | Plus | Bacteria; Firmicutes; Clostridia; Eubacteriales; Lachnospiraceae; ; [Eubacterium] rectale | [Eubacterium] rectale | | Lachnospiraceae | Eubacteriales | Clostridia | Firmicutes | Bacteria |
| ASV4670 | 405 | 1 | 1 | 405 | 100 | NR_026476.1 | Succinivibrio dextrinosolvens strain 0554 16S ribosomal RNA, partial sequence | NR_026476.1 | 1463 | 331 | 732 | 27.48 | 584.7 | 1E-166 | 376/405 | 92 | 3/405 | 0 | Plus | Plus | Bacteria; Proteobacteria; Gammaproteobacteria; Aeromonadales; Succinivibrionaceae; Succinivibrio; Succinivibrio dextrinosolvens | Succinivibrio dextrinosolvens | Succinivibrio | Succinivibrionaceae | Aeromonadales | Gammaproteobacteria | Proteobacteria | Bacteria |
| ASV4672 | 401 | 2 | 1 | 401 | 100 | NR_144737.1 | Emergencia timonensis strain SN18 16S ribosomal RNA, partial sequence | NR_144737.1 | 1480 | 364 | 767 | 27.3 | 538.5 | 9E-153 | 369/406 | 90 | 7/406 | 1 | Plus | Plus | Bacteria; Firmicutes; Clostridia; Eubacteriales; Clostridiales Family XIII. Incertae Sedis; Emergencia; Emergencia timonensis | Emergencia timonensis | Emergencia | Clostridiales Family XIII. Incertae Sedis | Eubacteriales | Clostridia | Firmicutes | Bacteria |
| ASV4674 | 421 | 1 | 1 | 421 | 100 | NR_144616.1 | Muribaculum intestinale strain YL27 16S ribosomal RNA, partial sequence | NR_144616.1 | 1460 | 297 | 718 | 28.9 | 484.9 | 1E-136 | 374/427 | 87 | 11/427 | 2 | Plus | Plus | Bacteria; Bacteroidetes; Bacteroidia; Bacteroidales; Muribaculaceae; Muribaculum; Muribaculum intestinale | Muribaculum intestinale | Muribaculum | Muribaculaceae | Bacteroidales | Bacteroidia | Bacteroidetes | Bacteria |
| ASV4675 | 268 | 1 | 1 | 260 | 97.01 | NR_029335.1 | Holdemania filiformis strain J1-31B-1 16S ribosomal RNA, partial sequence | NR_029335.1 | 1391 | 267 | 526 | 18.69 | 326.1 | 5E-89 | 232/260 | 89 | 0/260 | 0 | Plus | Plus | Bacteria; Firmicutes; Erysipelotrichia; Erysipelotrichales; Erysipelotrichaceae; Holdemania; Holdemania filiformis | Holdemania filiformis | Holdemania | Erysipelotrichaceae | Erysipelotrichales | Erysipelotrichia | Firmicutes | Bacteria |
| ASV4676 | 427 | 1 | 5 | 427 | 99.06 | NR_024883.1 | Kluyvera georgiana strain ATCC 51603 16S ribosomal RNA, partial sequence | NR_024883.1 | 1414 | 327 | 749 | 29.92 | 536.6 | 4E-152 | 379/425 | 89 | 4/425 | 0 | Plus | Plus | Bacteria; Proteobacteria; Gammaproteobacteria; Enterobacterales; Enterobacteriaceae; Kluyvera; Kluyvera georgiana | Kluyvera georgiana | Kluyvera | Enterobacteriaceae | Enterobacterales | Gammaproteobacteria | Proteobacteria | Bacteria |
| ASV4677 | 404 | 1 | 1 | 404 | 100 | NR_041236.1 | Lutispora thermophila DSM 19022 strain EBR46 16S ribosomal RNA, partial sequence | NR_041236.1 | 1438 | 338 | 740 | 28.03 | 435.1 | 1E-121 | 349/405 | 86 | 3/405 | 0 | Plus | Plus | Bacteria; Firmicutes; Clostridia; Eubacteriales; Clostridiaceae; Lutispora; Lutispora thermophila | Lutispora thermophila | Lutispora | Clostridiaceae | Eubacteriales | Clostridia | Firmicutes | Bacteria |
| ASV4678 | 403 | 1 | 1 | 401 | 99.5 | NR_147375.1 | Colidextribacter massiliensis strain Marseille-P3083 16S ribosomal RNA, partial sequence | NR_147375.1 | 1487 | 364 | 763 | 26.9 | 446.2 | 6E-125 | 354/407 | 86 | 13/407 | 3 | Plus | Plus | Bacteria; Firmicutes; Clostridia; Eubacteriales; ; Colidextribacter; Colidextribacter massiliensis | Colidextribacter massiliensis | Colidextribacter | | Eubacteriales | Clostridia | Firmicutes | Bacteria |
| ASV4679 | 402 | 1 | 1 | 402 | 100 | NR_118554.1 | Intestinimonas butyriciproducens strain SRB-521-5-I 16S ribosomal RNA, partial sequence | NR_118554.1 | 1475 | 346 | 750 | 27.46 | 540.3 | 3E-153 | 368/405 | 90 | 3/405 | 0 | Plus | Plus | Bacteria; Firmicutes; Clostridia; Eubacteriales; ; Intestinimonas; Intestinimonas butyriciproducens | Intestinimonas butyriciproducens | Intestinimonas | | Eubacteriales | Clostridia | Firmicutes | Bacteria |
| ASV4681 | 402 | 5 | 1 | 402 | 100 | NR_029355.1 | [Clostridium] methylpentosum strain R2 16S ribosomal RNA, partial sequence | NR_029355.1 | 1435 | 349 | 750 | 28.01 | 466.5 | 4E-131 | 353/403 | 87 | 2/403 | 0 | Plus | Plus | Bacteria; Firmicutes; Clostridia; Eubacteriales; Oscillospiraceae; ; [Clostridium] methylpentosum | [Clostridium] methylpentosum | | Oscillospiraceae | Eubacteriales | Clostridia | Firmicutes | Bacteria |
| ASV4685 | 422 | 1 | 1 | 422 | 100 | NR_041364.1 | Prevotella stercorea DSM 18206 strain CB35 16S ribosomal RNA, partial sequence | NR_041364.1 | 1488 | 354 | 775 | 28.36 | 664.1 | 0 | 401/422 | 95 | 0/422 | 0 | Plus | Plus | Bacteria; Bacteroidetes; Bacteroidia; Bacteroidales; Prevotellaceae; Prevotella; Prevotella stercorea | Prevotella stercorea | Prevotella | Prevotellaceae | Bacteroidales | Bacteroidia | Bacteroidetes | Bacteria |
| ASV4686 | 409 | 1 | 1 | 409 | 100 | NR_102980.1 | Megasphaera elsdenii DSM 20460 16S ribosomal RNA, partial sequence | NR_102980.1 | 1552 | 369 | 795 | 27.51 | 534.8 | 1E-151 | 384/427 | 89 | 18/427 | 4 | Plus | Plus | Bacteria; Firmicutes; Negativicutes; Veillonellales; Veillonellaceae; Megasphaera; Megasphaera elsdenii | Megasphaera elsdenii | Megasphaera | Veillonellaceae | Veillonellales | Negativicutes | Firmicutes | Bacteria |
| ASV4687 | 421 | 2 | 1 | 374 | 88.84 | NR_113039.1 | Solobacterium moorei strain JCM 10645 16S ribosomal RNA, partial sequence | NR_113039.1 | 1486 | 352 | 725 | 25.17 | 508.9 | 8E-144 | 342/375 | 91 | 2/375 | 0 | Plus | Plus | Bacteria; Firmicutes; Erysipelotrichia; Erysipelotrichales; Erysipelotrichaceae; Solobacterium; Solobacterium moorei | Solobacterium moorei | Solobacterium | Erysipelotrichaceae | Erysipelotrichales | Erysipelotrichia | Firmicutes | Bacteria |
| ASV4690 | 402 | 1 | 1 | 402 | 100 | NR_029239.3 | Clostridium chartatabidum strain 163 16S ribosomal RNA, partial sequence | NR_029239.3 | 1460 | 323 | 724 | 27.53 | 588.4 | 9E-168 | 375/403 | 93 | 2/403 | 0 | Plus | Plus | Bacteria; Firmicutes; Clostridia; Eubacteriales; Clostridiaceae; Clostridium; Clostridium chartatabidum | Clostridium chartatabidum | Clostridium | Clostridiaceae | Eubacteriales | Clostridia | Firmicutes | Bacteria |
| ASV4693 | 403 | 1 | 1 | 403 | 100 | NR_044425.1 | Hydrogenoanaerobacterium saccharovorans strain SW512 16S ribosomal RNA, partial sequence | NR_044425.1 | 1510 | 363 | 764 | 26.62 | 599.4 | 4E-171 | 378/404 | 93 | 3/404 | 0 | Plus | Plus | Bacteria; Firmicutes; Clostridia; Eubacteriales; Oscillospiraceae; Hydrogenoanaerobacterium; Hydrogenoanaerobacterium saccharovorans | Hydrogenoanaerobacterium saccharovorans | Hydrogenoanaerobacterium | Oscillospiraceae | Eubacteriales | Clostridia | Firmicutes | Bacteria |
| ASV4694 | 427 | 4 | 15 | 427 | 96.72 | NR_041781.1 | Streptococcus alactolyticus strain ATCC 43077 16S ribosomal RNA, partial sequence | NR_041781.1 | 1437 | 360 | 772 | 28.74 | 547.7 | 2E-155 | 378/417 | 90 | 8/417 | 1 | Plus | Plus | Bacteria; Firmicutes; Bacilli; Lactobacillales; Streptococcaceae; Streptococcus; Streptococcus alactolyticus | Streptococcus alactolyticus | Streptococcus | Streptococcaceae | Lactobacillales | Bacilli | Firmicutes | Bacteria |
| ASV4695 | 405 | 3 | 1 | 405 | 100 | NR_114728.1 | Clostridium tertium strain ATCC 14573 16S ribosomal RNA, partial sequence | NR_114728.1 | 1415 | 304 | 705 | 28.41 | 579.1 | 6E-165 | 375/405 | 92 | 3/405 | 0 | Plus | Plus | Bacteria; Firmicutes; Clostridia; Eubacteriales; Clostridiaceae; Clostridium; Clostridium tertium | Clostridium tertium | Clostridium | Clostridiaceae | Eubacteriales | Clostridia | Firmicutes | Bacteria |
| ASV4696 | 401 | 1 | 3 | 401 | 99.5 | NR_158111.1 | Paludicola psychrotolerans strain NC1253 16S ribosomal RNA, partial sequence | NR_158111.1 | 1490 | 368 | 766 | 26.78 | 449.9 | 4E-126 | 350/402 | 87 | 6/402 | 1 | Plus | Plus | Bacteria; Firmicutes; Clostridia; Eubacteriales; Oscillospiraceae; Paludicola; Paludicola psychrotolerans | Paludicola psychrotolerans | Paludicola | Oscillospiraceae | Eubacteriales | Clostridia | Firmicutes | Bacteria |
| ASV4699 | 404 | 1 | 3 | 404 | 99.5 | NR_102884.1 | Ruminococcus champanellensis 18P13 = JCM 17042 16S ribosomal RNA, partial sequence | NR_102884.1 | 1494 | 350 | 749 | 26.77 | 520 | 3E-147 | 362/402 | 90 | 2/402 | 0 | Plus | Plus | Bacteria; Firmicutes; Clostridia; Eubacteriales; Oscillospiraceae; Ruminococcus; Ruminococcus champanellensis | Ruminococcus champanellensis | Ruminococcus | Oscillospiraceae | Eubacteriales | Clostridia | Firmicutes | Bacteria |
| ASV4700 | 427 | 1 | 5 | 427 | 99.06 | NR_041778.1 | Desulfovibrio piger strain ATCC 29098 16S ribosomal RNA, partial sequence | NR_041778.1 | 1542 | 361 | 783 | 27.43 | 544 | 2E-154 | 385/428 | 89 | 10/428 | 2 | Plus | Plus | Bacteria; Proteobacteria; Deltaproteobacteria; Desulfovibrionales; Desulfovibrionaceae; Desulfovibrio; Desulfovibrio piger | Desulfovibrio piger | Desulfovibrio | Desulfovibrionaceae | Desulfovibrionales | Deltaproteobacteria | Proteobacteria | Bacteria |
| ASV4701 | 405 | 3 | 1 | 405 | 100 | NR_115307.1 | Ethanoligenens harbinense YUAN-3 16S ribosomal RNA, partial sequence | NR_115307.1 | 1497 | 340 | 743 | 26.99 | 547.7 | 2E-155 | 371/407 | 91 | 5/407 | 1 | Plus | Plus | Bacteria; Firmicutes; Clostridia; Eubacteriales; Oscillospiraceae; Ethanoligenens; Ethanoligenens harbinense | Ethanoligenens harbinense | Ethanoligenens | Oscillospiraceae | Eubacteriales | Clostridia | Firmicutes | Bacteria |
| ASV4702 | 405 | 4 | 5 | 405 | 99.01 | NR_118511.1 | Campylobacter coli strain ATCC 33559 16S ribosomal RNA, partial sequence | NR_118511.1 | 1435 | 333 | 731 | 27.8 | 545.9 | 6E-155 | 366/401 | 91 | 2/401 | 0 | Plus | Plus | Bacteria; Proteobacteria; Epsilonproteobacteria; Campylobacterales; Campylobacteraceae; Campylobacter; Campylobacter coli | Campylobacter coli | Campylobacter | Campylobacteraceae | Campylobacterales | Epsilonproteobacteria | Proteobacteria | Bacteria |
| ASV4704 | 404 | 1 | 3 | 404 | 99.5 | NR_026476.1 | Succinivibrio dextrinosolvens strain 0554 16S ribosomal RNA, partial sequence | NR_026476.1 | 1463 | 333 | 732 | 27.34 | 459.1 | 8E-129 | 352/403 | 87 | 4/403 | 0 | Plus | Plus | Bacteria; Proteobacteria; Gammaproteobacteria; Aeromonadales; Succinivibrionaceae; Succinivibrio; Succinivibrio dextrinosolvens | Succinivibrio dextrinosolvens | Succinivibrio | Succinivibrionaceae | Aeromonadales | Gammaproteobacteria | Proteobacteria | Bacteria |
| ASV4706 | 421 | 1 | 1 | 421 | 100 | NR_118334.1 | Alloprevotella rava strain 81/4-12 16S ribosomal RNA, partial sequence | NR_118334.1 | 1455 | 338 | 759 | 29 | 584.7 | 1E-166 | 387/422 | 91 | 1/422 | 0 | Plus | Plus | Bacteria; Bacteroidetes; Bacteroidia; Bacteroidales; Prevotellaceae; Alloprevotella; Alloprevotella rava | Alloprevotella rava | Alloprevotella | Prevotellaceae | Bacteroidales | Bacteroidia | Bacteroidetes | Bacteria |
| ASV4707 | 402 | 5 | 1 | 402 | 100 | NR_113140.1 | Paeniclostridium sordellii strain JCM 3814 16S ribosomal RNA, partial sequence | NR_113140.1 | 1463 | 336 | 736 | 27.41 | 508.9 | 7E-144 | 365/407 | 89 | 11/407 | 2 | Plus | Plus | Bacteria; Firmicutes; Clostridia; Eubacteriales; Peptostreptococcaceae; Paeniclostridium; Paeniclostridium sordellii | Paeniclostridium sordellii | Paeniclostridium | Peptostreptococcaceae | Eubacteriales | Clostridia | Firmicutes | Bacteria |
| ASV4710 | 405 | 1 | 1 | 405 | 100 | NR_044490.1 | Butyricicoccus pullicaecorum strain 25-3 16S ribosomal RNA, partial sequence | NR_044490.1 | 1482 | 366 | 770 | 27.33 | 582.8 | 4E-166 | 376/406 | 90 | 2/406 | 0 | Plus | Plus | Bacteria; Firmicutes; Clostridia; Eubacteriales; Clostridiaceae; Butyricicoccus; Butyricicoccus pullicaecorum | Butyricicoccus pullicaecorum | Butyricicoccus | Clostridiaceae | Eubacteriales | Clostridia | Firmicutes | Bacteria |
| ASV4711 | 426 | 1 | 1 | 426 | 100 | NR_146686.1 | Breznakia blatticola strain ErySL 16S ribosomal RNA, partial sequence | NR_146686.1 | 1429 | 335 | 760 | 29.81 | 433.2 | 5E-121 | 367/431 | 85 | 10/431 | 2 | Plus | Plus | Bacteria; Firmicutes; Erysipelotrichia; Erysipelotrichales; Erysipelotrichaceae; Breznakia; Breznakia blatticola | Breznakia blatticola | Breznakia | Erysipelotrichaceae | Erysipelotrichales | Erysipelotrichia | Firmicutes | Bacteria |
| ASV4712 | 403 | 1 | 1 | 403 | 100 | NR_147375.1 | Colidextribacter massiliensis strain Marseille-P3083 16S ribosomal RNA, partial sequence | NR_147375.1 | 1487 | 364 | 765 | 27.03 | 499.7 | 4E-141 | 359/403 | 89 | 1/403 | 0 | Plus | Plus | Bacteria; Firmicutes; Clostridia; Eubacteriales; ; Colidextribacter; Colidextribacter massiliensis | Colidextribacter massiliensis | Colidextribacter | | Eubacteriales | Clostridia | Firmicutes | Bacteria |
| ASV4714 | 403 | 1 | 1 | 403 | 100 | NR_044054.1 | Blautia wexlerae DSM 19850 16S ribosomal RNA, partial sequence | NR_044054.1 | 1438 | 314 | 715 | 27.96 | 593.9 | 2E-169 | 376/403 | 93 | 1/403 | 0 | Plus | Plus | Bacteria; Firmicutes; Clostridia; Eubacteriales; Lachnospiraceae; Blautia; Blautia wexlerae | Blautia wexlerae | Blautia | Lachnospiraceae | Eubacteriales | Clostridia | Firmicutes | Bacteria |
| ASV4717 | 402 | 1 | 1 | 422 | 100 | NR_132609.1 | Marinilabilia nitratireducens strain AK2 16S ribosomal RNA, partial sequence | NR_132609.1 | 1403 | 336 | 757 | 30.08 | 503.4 | 4E-142 | 372/422 | 88 | 0/422 | 0 | Plus | Plus | Bacteria; Bacteroidetes; Bacteroidia; Marinilabiliales; Marinilabiliaceae; Marinilabilia; Marinilabilia nitratireducens | Marinilabilia nitratireducens | Marinilabilia | Marinilabiliaceae | Marinilabiliales | Bacteroidia | Bacteroidetes | Bacteria |
| ASV4718 | 422 | 1 | 1 | 422 | 100 | NR_113411.1 | Prevotella copri DSM 18205 strain JCM 13464 16S ribosomal RNA, partial sequence | NR_113411.1 | 1493 | 358 | 779 | 28.27 | 619.7 | 3E-177 | 394/423 | 93 | 2/423 | 0 | Plus | Plus | Bacteria; Bacteroidetes; Bacteroidia; Bacteroidales; Prevotellaceae; Prevotella; Prevotella copri | Prevotella copri | Prevotella | Prevotellaceae | Bacteroidales | Bacteroidia | Bacteroidetes | Bacteria |
| ASV4719 | 402 | 1 | 1 | 402 | 100 | NR_074613.1 | [Eubacterium] eligens ATCC 27750 16S ribosomal RNA, partial sequence | NR_074613.1 | 1519 | 367 | 768 | 26.46 | 411.1 | 2E-114 | 343/403 | 85 | 2/403 | 0 | Plus | Plus | Bacteria; Firmicutes; Clostridia; Eubacteriales; Lachnospiraceae; Lachnospira; Lachnospira eligens | Lachnospira eligens | Lachnospira | Lachnospiraceae | Eubacteriales | Clostridia | Firmicutes | Bacteria |
| ASV4720 | 422 | 2 | 1 | 422 | 100 | NR_113117.1 | Prevotella oralis ATCC 33269 strain JCM 12251 16S ribosomal RNA, partial sequence | NR_113117.1 | 1496 | 361 | 782 | 28.21 | 592 | 7E-169 | 390/424 | 91 | 4/424 | 0 | Plus | Plus | Bacteria; Bacteroidetes; Bacteroidia; Bacteroidales; Prevotellaceae; Prevotella; Prevotella oralis | Prevotella oralis | Prevotella | Prevotellaceae | Bacteroidales | Bacteroidia | Bacteroidetes | Bacteria |
| ASV4724 | 422 | 1 | 1 | 422 | 100 | NR_113411.1 | Prevotella copri DSM 18205 strain JCM 13464 16S ribosomal RNA, partial sequence | NR_113411.1 | 1493 | 358 | 779 | 28.27 | 725 | 0 | 412/422 | 97 | 0/422 | 0 | Plus | Plus | Bacteria; Bacteroidetes; Bacteroidia; Bacteroidales; Prevotellaceae; Prevotella; Prevotella copri | Prevotella copri | Prevotella | Prevotellaceae | Bacteroidales | Bacteroidia | Bacteroidetes | Bacteria |
| ASV4725 | 402 | 1 | 1 | 402 | 100 | NR_159261.1 | Bifidobacterium callitrichidarum strain TRI 5 16S ribosomal RNA, partial sequence | NR_159261.1 | 1374 | 279 | 686 | 29.69 | 538.5 | 9E-153 | 372/410 | 90 | 10/410 | 2 | Plus | Plus | Bacteria; Actinobacteria; Actinomycetia; Bifidobacteriales; Bifidobacteriaceae; Bifidobacterium; Bifidobacterium callitrichidarum | Bifidobacterium callitrichidarum | Bifidobacterium | Bifidobacteriaceae | Bifidobacteriales | Actinomycetia | Actinobacteria | Bacteria |
| ASV4726 | 426 | 1 | 1 | 426 | 100 | NR_043438.1 | Bifidobacterium animalis strain JCM 1190 16S ribosomal RNA, partial sequence | NR_043438.1 | 1529 | 348 | 767 | 27.47 | 549.6 | 5E-156 | 389/431 | 90 | 16/431 | 3 | Plus | Plus | Bacteria; Actinobacteria; Actinomycetia; Bifidobacteriales; Bifidobacteriaceae; Bifidobacterium; Bifidobacterium animalis | Bifidobacterium animalis | Bifidobacterium | Bifidobacteriaceae | Bifidobacteriales | Actinomycetia | Actinobacteria | Bacteria |
| ASV4729 | 428 | 1 | 1 | 428 | 100 | NR_118156.1 | Oscillibacter ruminantium GH1 16S ribosomal RNA, partial sequence | NR_118156.1 | 1409 | 321 | 724 | 28.67 | 453.5 | 4E-127 | 373/430 | 86 | 28/430 | 6 | Plus | Plus | Bacteria; Firmicutes; Clostridia; Eubacteriales; Oscillospiraceae; Oscillibacter; Oscillibacter ruminantium | Oscillibacter ruminantium | Oscillibacter | Oscillospiraceae | Eubacteriales | Clostridia | Firmicutes | Bacteria |
| ASV4730 | 409 | 1 | 1 | 409 | 100 | NR_029097.1 | Oribacterium sinus strain AIP 354.02 16S ribosomal RNA, partial sequence | NR_029097.1 | 1374 | 308 | 709 | 29.26 | 521.9 | 1E-147 | 368/409 | 89 | 7/409 | 1 | Plus | Plus | Bacteria; Firmicutes; Clostridia; Eubacteriales; Lachnospiraceae; Oribacterium; Oribacterium sinus | Oribacterium sinus | Oribacterium | Lachnospiraceae | Eubacteriales | Clostridia | Firmicutes | Bacteria |
| ASV4731 | 404 | 1 | 3 | 404 | 99.5 | NR_026476.1 | Succinivibrio dextrinosolvens strain 0554 16S ribosomal RNA, partial sequence | NR_026476.1 | 1463 | 333 | 732 | 27.34 | 492.3 | 7E-139 | 358/403 | 88 | 4/403 | 0 | Plus | Plus | Bacteria; Proteobacteria; Gammaproteobacteria; Aeromonadales; Succinivibrionaceae; Succinivibrio; Succinivibrio dextrinosolvens | Succinivibrio dextrinosolvens | Succinivibrio | Succinivibrionaceae | Aeromonadales | Gammaproteobacteria | Proteobacteria | Bacteria |
| ASV4732 | 403 | 1 | 1 | 403 | 100 | NR_152688.1 | Falcatimonas natans strain WN011 16S ribosomal RNA, partial sequence | NR_152688.1 | 1484 | 365 | 766 | 27.09 | 405.5 | 1E-112 | 347/408 | 85 | 11/408 | 2 | Plus | Plus | Bacteria; Firmicutes; Clostridia; Eubacteriales; Clostridiaceae; Falcatimonas; Falcatimonas natans | Falcatimonas natans | Falcatimonas | Clostridiaceae | Eubacteriales | Clostridia | Firmicutes | Bacteria |
| ASV4733 | 401 | 1 | 3 | 401 | 99.5 | NR_137408.1 | Terrisporobacter petrolearius strain LAM0A37 16S ribosomal RNA, partial sequence | NR_137408.1 | 1405 | 309 | 707 | 28.4 | 605 | 9E-173 | 375/399 | 93 | 0/399 | 0 | Plus | Plus | Bacteria; Firmicutes; Clostridia; Eubacteriales; Peptostreptococcaceae; Terrisporobacter; Terrisporobacter petrolearius | Terrisporobacter petrolearius | Terrisporobacter | Peptostreptococcaceae | Eubacteriales | Clostridia | Firmicutes | Bacteria |
| ASV4737 | 426 | 1 | 4 | 426 | 99.3 | NR_125628.1 | Holdemania massiliensis AP2 16S ribosomal RNA, partial sequence | NR_125628.1 | 1499 | 356 | 778 | 28.22 | 444.3 | 2E-124 | 365/426 | 85 | 6/426 | 1 | Plus | Plus | Bacteria; Firmicutes; Erysipelotrichia; Erysipelotrichales; Erysipelotrichaceae; Holdemania; Holdemania massiliensis | Holdemania massiliensis | Holdemania | Erysipelotrichaceae | Erysipelotrichales | Erysipelotrichia | Firmicutes | Bacteria |
| ASV4738 | 404 | 1 | 3 | 404 | 99.5 | NR_125464.1 | Anaerobacterium chartisolvens strain T-1-35 16S ribosomal RNA, partial sequence | NR_125464.1 | 1451 | 344 | 745 | 27.71 | 411.1 | 2E-114 | 345/405 | 85 | 6/405 | 1 | Plus | Plus | Bacteria; Firmicutes; Clostridia; Eubacteriales; Oscillospiraceae; Anaerobacterium; Anaerobacterium chartisolvens | Anaerobacterium chartisolvens | Anaerobacterium | Oscillospiraceae | Eubacteriales | Clostridia | Firmicutes | Bacteria |
| ASV4741 | 403 | 1 | 1 | 403 | 100 | NR_134694.1 | Aminicella lysinilytica strain WN037 16S ribosomal RNA, partial sequence | NR_134694.1 | 1465 | 353 | 756 | 27.58 | 479.4 | 6E-135 | 360/408 | 88 | 9/408 | 2 | Plus | Plus | Bacteria; Firmicutes; Clostridia; Eubacteriales; Eubacteriaceae; Aminicella; Aminicella lysinilytica | Aminicella lysinilytica | Aminicella | Eubacteriaceae | Eubacteriales | Clostridia | Firmicutes | Bacteria |
| ASV4743 | 406 | 3 | 1 | 391 | 96.31 | NR_116939.1 | Olsenella profusa DSM 13989 strain CCUG 45371 16S ribosomal RNA, partial sequence | NR_116939.1 | 1428 | 328 | 720 | 27.52 | 575.4 | 7E-164 | 368/395 | 93 | 6/395 | 1 | Plus | Plus | Bacteria; Actinobacteria; Coriobacteriia; Coriobacteriales; Atopobiaceae; Olsenella; Olsenella profusa | Olsenella profusa | Olsenella | Atopobiaceae | Coriobacteriales | Coriobacteriia | Actinobacteria | Bacteria |
| ASV4744 | 422 | 1 | 1 | 422 | 100 | NR_040783.1 | Bifidobacterium breve DSM 20213 = JCM 1192 16S ribosomal RNA, partial sequence | NR_040783.1 | 1520 | 345 | 756 | 27.11 | 483.1 | 5E-136 | 370/422 | 87 | 10/422 | 2 | Plus | Plus | Bacteria; Actinobacteria; Actinomycetia; Bifidobacteriales; Bifidobacteriaceae; Bifidobacterium; Bifidobacterium breve | Bifidobacterium breve | Bifidobacterium | Bifidobacteriaceae | Bifidobacteriales | Actinomycetia | Actinobacteria | Bacteria |
| ASV4746 | 405 | 1 | 1 | 405 | 100 | NR_044972.1 | Sporobacter termitidis strain SYR 16S ribosomal RNA, partial sequence | NR_044972.1 | 1506 | 348 | 752 | 26.89 | 542.2 | 7E-154 | 368/406 | 90 | 2/406 | 0 | Plus | Plus | Bacteria; Firmicutes; Clostridia; Eubacteriales; Oscillospiraceae; Sporobacter; Sporobacter termitidis | Sporobacter termitidis | Sporobacter | Oscillospiraceae | Eubacteriales | Clostridia | Firmicutes | Bacteria |
| ASV4747 | 423 | 1 | 1 | 423 | 100 | NR_144750.1 | Prevotellamassilia timonensis strain Marseille-P2831 16S ribosomal RNA, partial sequence | NR_144750.1 | 1493 | 358 | 779 | 28.27 | 547.7 | 2E-155 | 384/426 | 90 | 7/426 | 1 | Plus | Plus | Bacteria; Bacteroidetes; Bacteroidia; Bacteroidales; Prevotellaceae; Prevotellamassilia; Prevotellamassilia timonensis | Prevotellamassilia timonensis | Prevotellamassilia | Prevotellaceae | Bacteroidales | Bacteroidia | Bacteroidetes | Bacteria |
| ASV4748 | 422 | 1 | 1 | 408 | 96.68 | NR_024815.1 | Prevotella shahii strain EHS11 16S ribosomal RNA, partial sequence | NR_024815.1 | 1490 | 356 | 763 | 27.38 | 499.7 | 5E-141 | 365/411 | 88 | 6/411 | 1 | Plus | Plus | Bacteria; Bacteroidetes; Bacteroidia; Bacteroidales; Prevotellaceae; Prevotella; Prevotella shahii | Prevotella shahii | Prevotella | Prevotellaceae | Bacteroidales | Bacteroidia | Bacteroidetes | Bacteria |
| ASV4750 | 402 | 1 | 1 | 402 | 100 | NR_118651.1 | [Clostridium] aminophilum strain F 16S ribosomal RNA, partial sequence | NR_118651.1 | 1477 | 372 | 773 | 27.22 | 505.3 | 9E-143 | 359/403 | 89 | 2/403 | 0 | Plus | Plus | Bacteria; Firmicutes; Clostridia; Eubacteriales; Lachnospiraceae; Lachnoclostridium; [Clostridium] aminophilum | [Clostridium] aminophilum | Lachnoclostridium | Lachnospiraceae | Eubacteriales | Clostridia | Firmicutes | Bacteria |
| ASV4751 | 421 | 1 | 1 | 412 | 97.86 | NR_144616.1 | Muribaculum intestinale strain YL27 16S ribosomal RNA, partial sequence | NR_144616.1 | 1460 | 297 | 709 | 28.29 | 429.5 | 6E-120 | 355/415 | 85 | 5/415 | 1 | Plus | Plus | Bacteria; Bacteroidetes; Bacteroidia; Bacteroidales; Muribaculaceae; Muribaculum; Muribaculum intestinale | Muribaculum intestinale | Muribaculum | Muribaculaceae | Bacteroidales | Bacteroidia | Bacteroidetes | Bacteria |
| ASV4753 | 405 | 5 | 1 | 405 | 100 | NR_115693.1 | Gracilibacter thermotolerans JW/YJL-S1 16S ribosomal RNA, partial sequence | NR_115693.1 | 1526 | 459 | 861 | 26.41 | 481.2 | 2E-135 | 357/405 | 88 | 2/405 | 0 | Plus | Plus | Bacteria; Firmicutes; Clostridia; Eubacteriales; Gracilibacteraceae; Gracilibacter; Gracilibacter thermotolerans | Gracilibacter thermotolerans | Gracilibacter | Gracilibacteraceae | Eubacteriales | Clostridia | Firmicutes | Bacteria |
| ASV4756 | 402 | 1 | 1 | 402 | 100 | NR_024945.1 | Clostridium gasigenes strain DSM 12272 16S ribosomal RNA, partial sequence | NR_024945.1 | 1483 | 348 | 749 | 27.11 | 510.8 | 2E-144 | 361/403 | 89 | 2/403 | 0 | Plus | Plus | Bacteria; Firmicutes; Clostridia; Eubacteriales; Clostridiaceae; Clostridium; Clostridium gasigenes | Clostridium gasigenes | Clostridium | Clostridiaceae | Eubacteriales | Clostridia | Firmicutes | Bacteria |
| ASV4757 | 427 | 2 | 1 | 427 | 100 | NR_028797.1 | Selenomonas infelix strain VPI D 75 B-30 16S ribosomal RNA, partial sequence | NR_028797.1 | 1524 | 373 | 799 | 28.02 | 601.3 | 1E-171 | 393/427 | 92 | 0/427 | 0 | Plus | Plus | Bacteria; Firmicutes; Negativicutes; Selenomonadales; Selenomonadaceae; Selenomonas; Selenomonas infelix | Selenomonas infelix | Selenomonas | Selenomonadaceae | Selenomonadales | Negativicutes | Firmicutes | Bacteria |
| ASV4758 | 402 | 1 | 3 | 402 | 99.5 | NR_104846.1 | Gemmiger formicilis strain X2-56 16S ribosomal RNA, partial sequence | NR_104846.1 | 1426 | 307 | 706 | 28.05 | 562.5 | 6E-160 | 371/403 | 92 | 6/403 | 1 | Plus | Plus | Bacteria; Firmicutes; Clostridia; Eubacteriales; ; Gemmiger; Gemmiger formicilis | Gemmiger formicilis | Gemmiger | | Eubacteriales | Clostridia | Firmicutes | Bacteria |
| ASV4759 | 405 | 3 | 3 | 405 | 99.51 | NR_074629.1 | Hungateiclostridium thermocellum strain ATCC 27405 16S ribosomal RNA, partial sequence | NR_074629.1 | 1521 | 360 | 762 | 26.5 | 462.8 | 6E-130 | 352/403 | 87 | 0/403 | 0 | Plus | Plus | Bacteria; Firmicutes; Clostridia; Eubacteriales; Oscillospiraceae; Acetivibrio; Acetivibrio thermocellus | Acetivibrio thermocellus | Acetivibrio | Oscillospiraceae | Eubacteriales | Clostridia | Firmicutes | Bacteria |
| ASV4761 | 422 | 4 | 1 | 422 | 100 | NR_113119.1 | Prevotella oris strain JCM 8540 16S ribosomal RNA, partial sequence | NR_113119.1 | 1481 | 345 | 766 | 28.49 | 531.1 | 2E-150 | 379/424 | 89 | 4/424 | 0 | Plus | Plus | Bacteria; Bacteroidetes; Bacteroidia; Bacteroidales; Prevotellaceae; Prevotella; Prevotella oris | Prevotella oris | Prevotella | Prevotellaceae | Bacteroidales | Bacteroidia | Bacteroidetes | Bacteria |
| ASV4763 | 422 | 1 | 1 | 422 | 100 | NR_144704.1 | Bacteroides timonensis AP1 16S ribosomal RNA, partial sequence | NR_144704.1 | 1315 | 268 | 689 | 32.09 | 630.8 | 2E-180 | 397/424 | 93 | 4/424 | 0 | Plus | Plus | Bacteria; Bacteroidetes; Bacteroidia; Bacteroidales; Bacteroidaceae; Bacteroides; Bacteroides timonensis | Bacteroides timonensis | Bacteroides | Bacteroidaceae | Bacteroidales | Bacteroidia | Bacteroidetes | Bacteria |
| ASV4764 | 405 | 4 | 1 | 405 | 100 | NR_042802.1 | Lactobacillus ultunensis strain Kx146C1 16S ribosomal RNA, partial sequence | NR_042802.1 | 1550 | 370 | 796 | 27.55 | 564.3 | 2E-160 | 390/427 | 91 | 22/427 | 5 | Plus | Plus | Bacteria; Firmicutes; Bacilli; Lactobacillales; Lactobacillaceae; Lactobacillus; Lactobacillus ultunensis | Lactobacillus ultunensis | Lactobacillus | Lactobacillaceae | Lactobacillales | Bacilli | Firmicutes | Bacteria |
| ASV4765 | 403 | 1 | 3 | 403 | 99.5 | NR_144749.1 | Ihubacter massiliensis strain Marseille-P2843 16S ribosomal RNA, partial sequence | NR_144749.1 | 1479 | 366 | 766 | 27.11 | 475.7 | 7E-134 | 355/403 | 88 | 4/403 | 0 | Plus | Plus | Bacteria; Firmicutes; Clostridia; Eubacteriales; Clostridiales Family XIII. Incertae Sedis; Ihubacter; Ihubacter massiliensis | Ihubacter massiliensis | Ihubacter | Clostridiales Family XIII. Incertae Sedis | Eubacteriales | Clostridia | Firmicutes | Bacteria |
| ASV4771 | 427 | 2 | 1 | 427 | 100 | NR_113039.1 | Solobacterium moorei strain JCM 10645 16S ribosomal RNA, partial sequence | NR_113039.1 | 1486 | 352 | 772 | 28.33 | 440.6 | 3E-123 | 367/429 | 85 | 10/429 | 2 | Plus | Plus | Bacteria; Firmicutes; Erysipelotrichia; Erysipelotrichales; Erysipelotrichaceae; Solobacterium; Solobacterium moorei | Solobacterium moorei | Solobacterium | Erysipelotrichaceae | Erysipelotrichales | Erysipelotrichia | Firmicutes | Bacteria |
| ASV4772 | 421 | 4 | 1 | 421 | 100 | NR_042802.1 | Lactobacillus ultunensis strain Kx146C1 16S ribosomal RNA, partial sequence | NR_042802.1 | 1550 | 370 | 796 | 27.55 | 545.9 | 6E-155 | 384/427 | 89 | 6/427 | 1 | Plus | Plus | Bacteria; Firmicutes; Bacilli; Lactobacillales; Lactobacillaceae; Lactobacillus; Lactobacillus ultunensis | Lactobacillus ultunensis | Lactobacillus | Lactobacillaceae | Lactobacillales | Bacilli | Firmicutes | Bacteria |
| ASV4777 | 402 | 1 | 3 | 402 | 99.5 | NR_117133.2 | Clostridium oceanicum strain DSM 1290 16S ribosomal RNA, partial sequence | NR_117133.2 | 1504 | 349 | 748 | 26.6 | 501.6 | 1E-141 | 363/406 | 89 | 12/406 | 2 | Plus | Plus | Bacteria; Firmicutes; Clostridia; Eubacteriales; Clostridiaceae; Clostridium; Clostridium oceanicum | Clostridium oceanicum | Clostridium | Clostridiaceae | Eubacteriales | Clostridia | Firmicutes | Bacteria |
| ASV4778 | 402 | 1 | 1 | 402 | 100 | NR_104907.1 | Eubacterium coprostanoligenes strain HL 16S ribosomal RNA, partial sequence | NR_104907.1 | 1446 | 332 | 734 | 27.87 | 533 | 4E-151 | 365/403 | 90 | 1/403 | 0 | Plus | Plus | Bacteria; Firmicutes; Clostridia; Eubacteriales; Eubacteriaceae; Eubacterium; Eubacterium coprostanoligenes | Eubacterium coprostanoligenes | Eubacterium | Eubacteriaceae | Eubacteriales | Clostridia | Firmicutes | Bacteria |
| ASV4779 | 405 | 1 | 1 | 405 | 100 | NR_102884.1 | Ruminococcus champanellensis 18P13 = JCM 17042 16S ribosomal RNA, partial sequence | NR_102884.1 | 1494 | 348 | 749 | 26.91 | 573.6 | 3E-163 | 374/405 | 92 | 3/405 | 0 | Plus | Plus | Bacteria; Firmicutes; Clostridia; Eubacteriales; Oscillospiraceae; Ruminococcus; Ruminococcus champanellensis | Ruminococcus champanellensis | Ruminococcus | Oscillospiraceae | Eubacteriales | Clostridia | Firmicutes | Bacteria |
| ASV4780 | 422 | 1 | 1 | 422 | 100 | NR_113153.1 | Alistipes shahii strain JCM 16773 16S ribosomal RNA, partial sequence | NR_113153.1 | 1486 | 351 | 772 | 28.4 | 725 | 0 | 413/423 | 97 | 2/423 | 0 | Plus | Plus | Bacteria; Bacteroidetes; Bacteroidia; Bacteroidales; Rikenellaceae; Alistipes; Alistipes shahii | Alistipes shahii | Alistipes | Rikenellaceae | Bacteroidales | Bacteroidia | Bacteroidetes | Bacteria |
| ASV4781 | 403 | 1 | 3 | 403 | 99.5 | NR_137408.1 | Terrisporobacter petrolearius strain LAM0A37 16S ribosomal RNA, partial sequence | NR_137408.1 | 1405 | 309 | 707 | 28.4 | 579.1 | 6E-165 | 372/401 | 92 | 2/401 | 0 | Plus | Plus | Bacteria; Firmicutes; Clostridia; Eubacteriales; Peptostreptococcaceae; Terrisporobacter; Terrisporobacter petrolearius | Terrisporobacter petrolearius | Terrisporobacter | Peptostreptococcaceae | Eubacteriales | Clostridia | Firmicutes | Bacteria |
| ASV4783 | 427 | 2 | 1 | 427 | 100 | NR_113306.1 | Megasphaera elsdenii DSM 20460 16S ribosomal RNA, partial sequence | NR_113306.1 | 1174 | 232 | 658 | 36.37 | 634.5 | 0 | 399/427 | 93 | 0/427 | 0 | Plus | Plus | Bacteria; Firmicutes; Negativicutes; Veillonellales; Veillonellaceae; Megasphaera; Megasphaera elsdenii | Megasphaera elsdenii | Megasphaera | Veillonellaceae | Veillonellales | Negativicutes | Firmicutes | Bacteria |
| ASV4784 | 422 | 4 | 1 | 422 | 100 | NR_044864.1 | Hallella seregens ATCC 51272 16S ribosomal RNA, partial sequence | NR_044864.1 | 1463 | 335 | 756 | 28.84 | 453.5 | 4E-127 | 367/426 | 86 | 8/426 | 1 | Plus | Plus | Bacteria; Bacteroidetes; Bacteroidia; Bacteroidales; Prevotellaceae; Prevotella; Hallella seregens | Hallella seregens | Prevotella | Prevotellaceae | Bacteroidales | Bacteroidia | Bacteroidetes | Bacteria |
| ASV4785 | 422 | 1 | 1 | 422 | 100 | NR_152671.1 | Marinifilum albidiflavum strain FB208 16S ribosomal RNA, partial sequence | NR_152671.1 | 1451 | 332 | 753 | 29.08 | 442.5 | 8E-124 | 361/422 | 85 | 0/422 | 0 | Plus | Plus | Bacteria; Bacteroidetes; Bacteroidia; Marinilabiliales; Marinifilaceae; Marinifilum; Marinifilum albidiflavum | Marinifilum albidiflavum | Marinifilum | Marinifilaceae | Marinilabiliales | Bacteroidia | Bacteroidetes | Bacteria |
| ASV4787 | 401 | 1 | 1 | 400 | 99.75 | NR_144737.1 | Emergencia timonensis strain SN18 16S ribosomal RNA, partial sequence | NR_144737.1 | 1480 | 364 | 766 | 27.23 | 569.9 | 3E-162 | 372/403 | 92 | 3/403 | 0 | Plus | Plus | Bacteria; Firmicutes; Clostridia; Eubacteriales; Clostridiales Family XIII. Incertae Sedis; Emergencia; Emergencia timonensis | Emergencia timonensis | Emergencia | Clostridiales Family XIII. Incertae Sedis | Eubacteriales | Clostridia | Firmicutes | Bacteria |
| ASV4788 | 402 | 1 | 3 | 402 | 99.5 | NR_029097.1 | Oribacterium sinus strain AIP 354.02 16S ribosomal RNA, partial sequence | NR_029097.1 | 1374 | 310 | 709 | 29.11 | 529.3 | 6E-150 | 362/400 | 90 | 0/400 | 0 | Plus | Plus | Bacteria; Firmicutes; Clostridia; Eubacteriales; Lachnospiraceae; Oribacterium; Oribacterium sinus | Oribacterium sinus | Oribacterium | Lachnospiraceae | Eubacteriales | Clostridia | Firmicutes | Bacteria |
| ASV4790 | 422 | 2 | 1 | 407 | 96.45 | NR_113119.1 | Prevotella oris strain JCM 8540 16S ribosomal RNA, partial sequence | NR_113119.1 | 1481 | 345 | 751 | 27.48 | 414.8 | 2E-115 | 351/412 | 85 | 10/412 | 2 | Plus | Plus | Bacteria; Bacteroidetes; Bacteroidia; Bacteroidales; Prevotellaceae; Prevotella; Prevotella oris | Prevotella oris | Prevotella | Prevotellaceae | Bacteroidales | Bacteroidia | Bacteroidetes | Bacteria |
| ASV4791 | 422 | 1 | 1 | 422 | 100 | NR_041342.1 | Parabacteroides distasonis strain JCM 5825 16S ribosomal RNA, partial sequence | NR_041342.1 | 1488 | 353 | 774 | 28.36 | 569.9 | 3E-162 | 384/422 | 90 | 0/422 | 0 | Plus | Plus | Bacteria; Bacteroidetes; Bacteroidia; Bacteroidales; Tannerellaceae; Parabacteroides; Parabacteroides distasonis | Parabacteroides distasonis | Parabacteroides | Tannerellaceae | Bacteroidales | Bacteroidia | Bacteroidetes | Bacteria |
| ASV4794 | 402 | 1 | 1 | 401 | 99.75 | NR_118675.1 | [Eubacterium] siraeum strain ATCC 29066 16S ribosomal RNA, partial sequence | NR_118675.1 | 1453 | 356 | 756 | 27.6 | 649.3 | 0 | 383/401 | 95 | 0/401 | 0 | Plus | Plus | Bacteria; Firmicutes; Clostridia; Eubacteriales; Oscillospiraceae; ; [Eubacterium] siraeum | [Eubacterium] siraeum | | Oscillospiraceae | Eubacteriales | Clostridia | Firmicutes | Bacteria |
| ASV4795 | 401 | 1 | 1 | 401 | 100 | NR_144737.1 | Emergencia timonensis strain SN18 16S ribosomal RNA, partial sequence | NR_144737.1 | 1480 | 364 | 767 | 27.3 | 521.9 | 9E-148 | 366/406 | 90 | 7/406 | 1 | Plus | Plus | Bacteria; Firmicutes; Clostridia; Eubacteriales; Clostridiales Family XIII. Incertae Sedis; Emergencia; Emergencia timonensis | Emergencia timonensis | Emergencia | Clostridiales Family XIII. Incertae Sedis | Eubacteriales | Clostridia | Firmicutes | Bacteria |
| ASV4797 | 408 | 2 | 1 | 408 | 100 | NR_037018.1 | Acidaminococcus fermentans strain VR4 16S ribosomal RNA gene, partial sequence | NR_037018.1 | 1516 | 349 | 775 | 28.17 | 582.8 | 4E-166 | 393/427 | 92 | 19/427 | 4 | Plus | Plus | Bacteria; Firmicutes; Negativicutes; Acidaminococcales; Acidaminococcaceae; Acidaminococcus; Acidaminococcus fermentans | Acidaminococcus fermentans | Acidaminococcus | Acidaminococcaceae | Acidaminococcales | Negativicutes | Firmicutes | Bacteria |
| ASV4798 | 422 | 2 | 1 | 422 | 100 | NR_040866.1 | Bacteroides uniformis strain JCM 5828 16S ribosomal RNA, partial sequence | NR_040866.1 | 1425 | 351 | 772 | 29.61 | 442.5 | 8E-124 | 363/424 | 85 | 4/424 | 0 | Plus | Plus | Bacteria; Bacteroidetes; Bacteroidia; Bacteroidales; Bacteroidaceae; Bacteroides; Bacteroides uniformis | Bacteroides uniformis | Bacteroides | Bacteroidaceae | Bacteroidales | Bacteroidia | Bacteroidetes | Bacteria |
| ASV4799 | 403 | 1 | 1 | 403 | 100 | NR_144609.1 | Enterorhabdus muris strain WCA-131-CoC-2 16S ribosomal RNA, partial sequence | NR_144609.1 | 1399 | 336 | 738 | 28.81 | 629 | 5E-180 | 382/403 | 94 | 0/403 | 0 | Plus | Plus | Bacteria; Actinobacteria; Coriobacteriia; Eggerthellales; Eggerthellaceae; Adlercreutzia; Adlercreutzia muris | Adlercreutzia muris | Adlercreutzia | Eggerthellaceae | Eggerthellales | Coriobacteriia | Actinobacteria | Bacteria |
| ASV4800 | 426 | 1 | 1 | 425 | 99.77 | NR_156976.1 | Desulfosporosinus fructosivorans strain 63.6F 16S ribosomal RNA, partial sequence | NR_156976.1 | 1529 | 350 | 776 | 27.93 | 510.8 | 2E-144 | 380/430 | 88 | 8/430 | 1 | Plus | Plus | Bacteria; Firmicutes; Clostridia; Eubacteriales; Peptococcaceae; Desulfosporosinus; Desulfosporosinus fructosivorans | Desulfosporosinus fructosivorans | Desulfosporosinus | Peptococcaceae | Eubacteriales | Clostridia | Firmicutes | Bacteria |
| ASV4801 | 428 | 1 | 3 | 427 | 99.3 | NR_044111.1 | Selenomonas bovis strain WG 16S ribosomal RNA, partial sequence | NR_044111.1 | 1525 | 372 | 795 | 27.8 | 468.3 | 1E-131 | 369/426 | 86 | 3/426 | 0 | Plus | Plus | Bacteria; Firmicutes; Negativicutes; Selenomonadales; Selenomonadaceae; Selenomonas; Selenomonas bovis | Selenomonas bovis | Selenomonas | Selenomonadaceae | Selenomonadales | Negativicutes | Firmicutes | Bacteria |
| ASV4803 | 402 | 2 | 1 | 402 | 100 | NR_114789.1 | [Clostridium] leptum strain DSM 753 16S ribosomal RNA, partial sequence | NR_114789.1 | 1505 | 364 | 765 | 26.71 | 627.1 | 2E-179 | 381/402 | 94 | 0/402 | 0 | Plus | Plus | Bacteria; Firmicutes; Clostridia; Eubacteriales; Oscillospiraceae; ; [Clostridium] leptum | [Clostridium] leptum | | Oscillospiraceae | Eubacteriales | Clostridia | Firmicutes | Bacteria |
| ASV4806 | 405 | 4 | 1 | 405 | 100 | NR_042802.1 | Lactobacillus ultunensis strain Kx146C1 16S ribosomal RNA, partial sequence | NR_042802.1 | 1550 | 370 | 796 | 27.55 | 558.8 | 7E-159 | 389/427 | 91 | 22/427 | 5 | Plus | Plus | Bacteria; Firmicutes; Bacilli; Lactobacillales; Lactobacillaceae; Lactobacillus; Lactobacillus ultunensis | Lactobacillus ultunensis | Lactobacillus | Lactobacillaceae | Lactobacillales | Bacilli | Firmicutes | Bacteria |
| ASV4807 | 422 | 1 | 1 | 422 | 100 | NR_144611.1 | Flintibacter butyricus strain BLS21 16S ribosomal RNA, partial sequence | NR_144611.1 | 808 | 299 | 703 | 50.12 | 448 | 2E-125 | 365/422 | 86 | 17/422 | 4 | Plus | Plus | Bacteria; Firmicutes; Clostridia; Eubacteriales; ; Flintibacter; Flintibacter butyricus | Flintibacter butyricus | Flintibacter | | Eubacteriales | Clostridia | Firmicutes | Bacteria |
| ASV4808 | 402 | 1 | 1 | 402 | 100 | NR_159227.1 | Monoglobus pectinilyticus strain 14 16S ribosomal RNA, partial sequence | NR_159227.1 | 1493 | 368 | 769 | 26.93 | 516.3 | 4E-146 | 361/402 | 89 | 0/402 | 0 | Plus | Plus | Bacteria; Firmicutes; Clostridia; Eubacteriales; Oscillospiraceae; Monoglobus; Monoglobus pectinilyticus | Monoglobus pectinilyticus | Monoglobus | Oscillospiraceae | Eubacteriales | Clostridia | Firmicutes | Bacteria |
| ASV4810 | 428 | 4 | 1 | 428 | 100 | NR_042802.1 | Lactobacillus ultunensis strain Kx146C1 16S ribosomal RNA, partial sequence | NR_042802.1 | 1550 | 370 | 796 | 27.55 | 612.4 | 6E-175 | 399/431 | 92 | 7/431 | 1 | Plus | Plus | Bacteria; Firmicutes; Bacilli; Lactobacillales; Lactobacillaceae; Lactobacillus; Lactobacillus ultunensis | Lactobacillus ultunensis | Lactobacillus | Lactobacillaceae | Lactobacillales | Bacilli | Firmicutes | Bacteria |
| ASV4811 | 402 | 1 | 1 | 402 | 100 | NR_147375.1 | Colidextribacter massiliensis strain Marseille-P3083 16S ribosomal RNA, partial sequence | NR_147375.1 | 1487 | 364 | 765 | 27.03 | 599.4 | 4E-171 | 377/403 | 93 | 2/403 | 0 | Plus | Plus | Bacteria; Firmicutes; Clostridia; Eubacteriales; ; Colidextribacter; Colidextribacter massiliensis | Colidextribacter massiliensis | Colidextribacter | | Eubacteriales | Clostridia | Firmicutes | Bacteria |
| ASV4813 | 403 | 1 | 1 | 403 | 100 | NR_144611.1 | Flintibacter butyricus strain BLS21 16S ribosomal RNA, partial sequence | NR_144611.1 | 808 | 299 | 703 | 50.12 | 503.4 | 3E-142 | 364/408 | 89 | 8/408 | 1 | Plus | Plus | Bacteria; Firmicutes; Clostridia; Eubacteriales; ; Flintibacter; Flintibacter butyricus | Flintibacter butyricus | Flintibacter | | Eubacteriales | Clostridia | Firmicutes | Bacteria |
| ASV4814 | 402 | 1 | 1 | 402 | 100 | NR_118673.1 | [Eubacterium] hallii strain ATCC 27751 16S ribosomal RNA, partial sequence | NR_118673.1 | 1479 | 371 | 772 | 27.18 | 673.3 | 0 | 388/402 | 96 | 0/402 | 0 | Plus | Plus | Bacteria; Firmicutes; Clostridia; Eubacteriales; Lachnospiraceae; Anaerobutyricum; Anaerobutyricum hallii | Anaerobutyricum hallii | Anaerobutyricum | Lachnospiraceae | Eubacteriales | Clostridia | Firmicutes | Bacteria |
| ASV4817 | 405 | 1 | 1 | 405 | 100 | NR_144742.1 | Christensenella massiliensis strain Marseille-P2438 16S ribosomal RNA, partial sequence | NR_144742.1 | 1499 | 369 | 773 | 27.02 | 477.6 | 2E-134 | 356/405 | 87 | 0/405 | 0 | Plus | Plus | Bacteria; Firmicutes; Clostridia; Eubacteriales; Christensenellaceae; Christensenella; Christensenella massiliensis | Christensenella massiliensis | Christensenella | Christensenellaceae | Eubacteriales | Clostridia | Firmicutes | Bacteria |
| ASV4819 | 421 | 3 | 1 | 421 | 100 | NR_041781.1 | Streptococcus alactolyticus strain ATCC 43077 16S ribosomal RNA, partial sequence | NR_041781.1 | 1437 | 346 | 772 | 29.71 | 629 | 6E-180 | 399/427 | 93 | 6/427 | 1 | Plus | Plus | Bacteria; Firmicutes; Bacilli; Lactobacillales; Streptococcaceae; Streptococcus; Streptococcus alactolyticus | Streptococcus alactolyticus | Streptococcus | Streptococcaceae | Lactobacillales | Bacilli | Firmicutes | Bacteria |
| ASV4822 | 402 | 1 | 1 | 402 | 100 | NR_041236.1 | Lutispora thermophila DSM 19022 strain EBR46 16S ribosomal RNA, partial sequence | NR_041236.1 | 1438 | 338 | 740 | 28.03 | 516.3 | 4E-146 | 362/403 | 89 | 1/403 | 0 | Plus | Plus | Bacteria; Firmicutes; Clostridia; Eubacteriales; Clostridiaceae; Lutispora; Lutispora thermophila | Lutispora thermophila | Lutispora | Clostridiaceae | Eubacteriales | Clostridia | Firmicutes | Bacteria |
| ASV4823 | 405 | 1 | 1 | 405 | 100 | NR_156897.1 | Anaeromicrobium sediminis strain DY2726D 16S ribosomal RNA, partial sequence | NR_156897.1 | 1414 | 335 | 736 | 28.43 | 446.2 | 6E-125 | 353/407 | 86 | 7/407 | 1 | Plus | Plus | Bacteria; Firmicutes; Clostridia; Eubacteriales; Clostridiaceae; Anaeromicrobium; Anaeromicrobium sediminis | Anaeromicrobium sediminis | Anaeromicrobium | Clostridiaceae | Eubacteriales | Clostridia | Firmicutes | Bacteria |
| ASV4826 | 405 | 1 | 1 | 405 | 100 | NR_026104.1 | Ruminiclostridium cellobioparum DSM 1351 = ATCC 15832 16S ribosomal RNA, partial sequence | NR_026104.1 | 1487 | 329 | 733 | 27.24 | 472 | 1E-132 | 355/405 | 87 | 0/405 | 0 | Plus | Plus | Bacteria; Firmicutes; Clostridia; Eubacteriales; Oscillospiraceae; Ruminiclostridium; Ruminiclostridium cellobioparum | Ruminiclostridium cellobioparum | Ruminiclostridium | Oscillospiraceae | Eubacteriales | Clostridia | Firmicutes | Bacteria |
| ASV4829 | 405 | 1 | 1 | 405 | 100 | NR_109014.1 | Blautia faecis strain M25 16S ribosomal RNA, partial sequence | NR_109014.1 | 1262 | 212 | 613 | 31.85 | 557 | 3E-158 | 373/407 | 91 | 7/407 | 1 | Plus | Plus | Bacteria; Firmicutes; Clostridia; Eubacteriales; Lachnospiraceae; Blautia; Blautia faecis | Blautia faecis | Blautia | Lachnospiraceae | Eubacteriales | Clostridia | Firmicutes | Bacteria |
| ASV4831 | 404 | 2 | 1 | 404 | 100 | NR_074793.2 | Oscillibacter valericigenes strain Sjm18-20 16S ribosomal RNA, complete sequence | NR_074793.2 | 1530 | 372 | 775 | 26.41 | 669.6 | 0 | 390/404 | 96 | 0/404 | 0 | Plus | Plus | Bacteria; Firmicutes; Clostridia; Eubacteriales; Oscillospiraceae; Oscillibacter; Oscillibacter valericigenes | Oscillibacter valericigenes | Oscillibacter | Oscillospiraceae | Eubacteriales | Clostridia | Firmicutes | Bacteria |
| ASV4832 | 405 | 1 | 1 | 388 | 95.8 | NR_147375.1 | Colidextribacter massiliensis strain Marseille-P3083 16S ribosomal RNA, partial sequence | NR_147375.1 | 1487 | 364 | 748 | 25.89 | 514.5 | 2E-145 | 353/389 | 90 | 5/389 | 1 | Plus | Plus | Bacteria; Firmicutes; Clostridia; Eubacteriales; ; Colidextribacter; Colidextribacter massiliensis | Colidextribacter massiliensis | Colidextribacter | | Eubacteriales | Clostridia | Firmicutes | Bacteria |
| ASV4833 | 404 | 3 | 1 | 404 | 100 | NR_029356.1 | Flavonifractor plautii strain 265 16S ribosomal RNA, partial sequence | NR_029356.1 | 1550 | 353 | 757 | 26.13 | 497.9 | 2E-140 | 363/408 | 88 | 7/408 | 1 | Plus | Plus | Bacteria; Firmicutes; Clostridia; Eubacteriales; Oscillospiraceae; Flavonifractor; Flavonifractor plautii | Flavonifractor plautii | Flavonifractor | Oscillospiraceae | Eubacteriales | Clostridia | Firmicutes | Bacteria |
| ASV4835 | 404 | 2 | 1 | 404 | 100 | NR_133762.1 | Hungatella effluvii strain UB-B.2 16S ribosomal RNA, partial sequence | NR_133762.1 | 1409 | 318 | 719 | 28.53 | 557 | 3E-158 | 370/404 | 91 | 2/404 | 0 | Plus | Plus | Bacteria; Firmicutes; Clostridia; Eubacteriales; Clostridiaceae; Hungatella; Hungatella effluvii | Hungatella effluvii | Hungatella | Clostridiaceae | Eubacteriales | Clostridia | Firmicutes | Bacteria |
| ASV4836 | 402 | 2 | 1 | 402 | 100 | NR_115549.1 | Rhodospirillum rubrum ATCC 11170 16S ribosomal RNA, partial sequence | NR_115549.1 | 1406 | 299 | 700 | 28.59 | 416.6 | 5E-116 | 345/404 | 85 | 4/404 | 0 | Plus | Plus | Bacteria; Proteobacteria; Alphaproteobacteria; Rhodospirillales; Rhodospirillaceae; Rhodospirillum; Rhodospirillum rubrum | Rhodospirillum rubrum | Rhodospirillum | Rhodospirillaceae | Rhodospirillales | Alphaproteobacteria | Proteobacteria | Bacteria |
| ASV4839 | 402 | 2 | 5 | 402 | 99 | NR_024917.1 | [Clostridium] methoxybenzovorans SR3 16S ribosomal RNA, partial sequence | NR_024917.1 | 1514 | 360 | 757 | 26.29 | 558.8 | 7E-159 | 368/400 | 92 | 4/400 | 1 | Plus | Plus | Bacteria; Firmicutes; Clostridia; Eubacteriales; Lachnospiraceae; Lachnoclostridium; [Clostridium] methoxybenzovorans | [Clostridium] methoxybenzovorans | Lachnoclostridium | Lachnospiraceae | Eubacteriales | Clostridia | Firmicutes | Bacteria |
| ASV4841 | 405 | 1 | 1 | 405 | 100 | NR_170495.1 | Blautia faecicola strain KGMB01111 16S ribosomal RNA, complete sequence | NR_170495.1 | 1534 | 372 | 773 | 26.21 | 584.7 | 1E-166 | 376/405 | 92 | 3/405 | 0 | Plus | Plus | Bacteria; Firmicutes; Clostridia; Eubacteriales; Lachnospiraceae; Blautia; Blautia faecicola | Blautia faecicola | Blautia | Lachnospiraceae | Eubacteriales | Clostridia | Firmicutes | Bacteria |
| ASV4842 | 404 | 3 | 1 | 404 | 100 | NR_147396.1 | Lachnoclostridium pacaense strain Marseille-P3100 16S ribosomal RNA, partial sequence | NR_147396.1 | 1453 | 345 | 746 | 27.67 | 518.2 | 1E-146 | 364/405 | 89 | 4/405 | 0 | Plus | Plus | Bacteria; Firmicutes; Clostridia; Eubacteriales; Lachnospiraceae; Lachnoclostridium; Lachnoclostridium pacaense | Lachnoclostridium pacaense | Lachnoclostridium | Lachnospiraceae | Eubacteriales | Clostridia | Firmicutes | Bacteria |
| ASV4843 | 402 | 1 | 1 | 402 | 100 | NR_152635.1 | Kiloniella majae strain M56.1 16S ribosomal RNA, partial sequence | NR_152635.1 | 1484 | 324 | 725 | 27.09 | 477.6 | 2E-134 | 361/409 | 88 | 14/409 | 3 | Plus | Plus | Bacteria; Proteobacteria; Alphaproteobacteria; Kiloniellales; Kiloniellaceae; Kiloniella; Kiloniella majae | Kiloniella majae | Kiloniella | Kiloniellaceae | Kiloniellales | Alphaproteobacteria | Proteobacteria | Bacteria |
| ASV4846 | 421 | 1 | 1 | 421 | 100 | NR_102980.1 | Megasphaera elsdenii DSM 20460 16S ribosomal RNA, partial sequence | NR_102980.1 | 1552 | 369 | 795 | 27.51 | 545.9 | 6E-155 | 386/429 | 89 | 10/429 | 2 | Plus | Plus | Bacteria; Firmicutes; Negativicutes; Veillonellales; Veillonellaceae; Megasphaera; Megasphaera elsdenii | Megasphaera elsdenii | Megasphaera | Veillonellaceae | Veillonellales | Negativicutes | Firmicutes | Bacteria |
| ASV4847 | 404 | 1 | 1 | 404 | 100 | NR_148619.1 | Agathobacter ruminis strain JK623 16S ribosomal RNA, partial sequence | NR_148619.1 | 1434 | 318 | 721 | 28.17 | 597.6 | 2E-170 | 378/405 | 93 | 2/405 | 0 | Plus | Plus | Bacteria; Firmicutes; Clostridia; Eubacteriales; Lachnospiraceae; Agathobacter; Agathobacter ruminis | Agathobacter ruminis | Agathobacter | Lachnospiraceae | Eubacteriales | Clostridia | Firmicutes | Bacteria |
| ASV4848 | 405 | 5 | 3 | 405 | 99.51 | NR_115693.1 | Gracilibacter thermotolerans JW/YJL-S1 16S ribosomal RNA, partial sequence | NR_115693.1 | 1526 | 461 | 861 | 26.28 | 411.1 | 2E-114 | 343/403 | 85 | 2/403 | 0 | Plus | Plus | Bacteria; Firmicutes; Clostridia; Eubacteriales; Gracilibacteraceae; Gracilibacter; Gracilibacter thermotolerans | Gracilibacter thermotolerans | Gracilibacter | Gracilibacteraceae | Eubacteriales | Clostridia | Firmicutes | Bacteria |
| ASV4849 | 405 | 2 | 1 | 405 | 100 | NR_144742.1 | Christensenella massiliensis strain Marseille-P2438 16S ribosomal RNA, partial sequence | NR_144742.1 | 1499 | 369 | 773 | 27.02 | 560.7 | 2E-159 | 371/405 | 91 | 0/405 | 0 | Plus | Plus | Bacteria; Firmicutes; Clostridia; Eubacteriales; Christensenellaceae; Christensenella; Christensenella massiliensis | Christensenella massiliensis | Christensenella | Christensenellaceae | Eubacteriales | Clostridia | Firmicutes | Bacteria |
| ASV4850 | 409 | 1 | 1 | 409 | 100 | NR_147752.1 | Egibacter rhizosphaerae strain EGI 80759 16S ribosomal RNA, partial sequence | NR_147752.1 | 1516 | 349 | 755 | 26.85 | 438.8 | 1E-122 | 357/414 | 86 | 12/414 | 2 | Plus | Plus | Bacteria; Actinobacteria; Nitriliruptoria; Egibacterales; Egibacteraceae; Egibacter; Egibacter rhizosphaerae | Egibacter rhizosphaerae | Egibacter | Egibacteraceae | Egibacterales | Nitriliruptoria | Actinobacteria | Bacteria |
| ASV4851 | 427 | 3 | 3 | 427 | 99.53 | NR_116939.1 | Olsenella profusa DSM 13989 strain CCUG 45371 16S ribosomal RNA, partial sequence | NR_116939.1 | 1428 | 330 | 735 | 28.43 | 507.1 | 3E-143 | 378/425 | 88 | 19/425 | 4 | Plus | Plus | Bacteria; Actinobacteria; Coriobacteriia; Coriobacteriales; Atopobiaceae; Olsenella; Olsenella profusa | Olsenella profusa | Olsenella | Atopobiaceae | Coriobacteriales | Coriobacteriia | Actinobacteria | Bacteria |
| ASV4854 | 402 | 3 | 3 | 402 | 99.5 | NR_074793.2 | Oscillibacter valericigenes strain Sjm18-20 16S ribosomal RNA, complete sequence | NR_074793.2 | 1530 | 374 | 775 | 26.27 | 492.3 | 7E-139 | 361/406 | 88 | 10/406 | 2 | Plus | Plus | Bacteria; Firmicutes; Clostridia; Eubacteriales; Oscillospiraceae; Oscillibacter; Oscillibacter valericigenes | Oscillibacter valericigenes | Oscillibacter | Oscillospiraceae | Eubacteriales | Clostridia | Firmicutes | Bacteria |
| ASV4855 | 427 | 4 | 3 | 427 | 99.53 | NR_041781.1 | Streptococcus alactolyticus strain ATCC 43077 16S ribosomal RNA, partial sequence | NR_041781.1 | 1437 | 348 | 772 | 29.58 | 564.3 | 2E-160 | 389/429 | 90 | 8/429 | 1 | Plus | Plus | Bacteria; Firmicutes; Bacilli; Lactobacillales; Streptococcaceae; Streptococcus; Streptococcus alactolyticus | Streptococcus alactolyticus | Streptococcus | Streptococcaceae | Lactobacillales | Bacilli | Firmicutes | Bacteria |
| ASV4856 | 402 | 1 | 3 | 402 | 99.5 | NR_134005.1 | Clostridium bornimense strain M2/40 16S ribosomal RNA, partial sequence | NR_134005.1 | 1363 | 286 | 685 | 29.35 | 584.7 | 1E-166 | 376/404 | 93 | 8/404 | 1 | Plus | Plus | Bacteria; Firmicutes; Clostridia; Eubacteriales; Clostridiaceae; Clostridium; Clostridium bornimense | Clostridium bornimense | Clostridium | Clostridiaceae | Eubacteriales | Clostridia | Firmicutes | Bacteria |
| ASV4857 | 403 | 1 | 1 | 403 | 100 | NR_117912.1 | Defluviitalea saccharophila strain LIND6LT2 16S ribosomal RNA, partial sequence | NR_117912.1 | 1495 | 342 | 746 | 27.09 | 558.8 | 7E-159 | 372/406 | 91 | 4/406 | 0 | Plus | Plus | Bacteria; Firmicutes; Clostridia; Eubacteriales; Defluviitaleaceae; Defluviitalea; Defluviitalea saccharophila | Defluviitalea saccharophila | Defluviitalea | Defluviitaleaceae | Eubacteriales | Clostridia | Firmicutes | Bacteria |
| ASV4859 | 427 | 1 | 1 | 427 | 100 | NR_042896.1 | Mucispirillum schaedleri strain HRI I17 16S ribosomal RNA, partial sequence | NR_042896.1 | 1471 | 333 | 759 | 29.03 | 678.8 | 0 | 407/427 | 95 | 0/427 | 0 | Plus | Plus | Bacteria; Deferribacteres; Deferribacteres; Deferribacterales; Deferribacteraceae; Mucispirillum; Mucispirillum schaedleri | Mucispirillum schaedleri | Mucispirillum | Deferribacteraceae | Deferribacterales | Deferribacteres | Deferribacteres | Bacteria |
| ASV4866 | 402 | 1 | 1 | 402 | 100 | NR_029160.1 | Ruminococcus callidus strain ATCC 27760 16S ribosomal RNA, partial sequence | NR_029160.1 | 1371 | 310 | 711 | 29.32 | 582.8 | 4E-166 | 373/402 | 92 | 0/402 | 0 | Plus | Plus | Bacteria; Firmicutes; Clostridia; Eubacteriales; Oscillospiraceae; Ruminococcus; Ruminococcus callidus | Ruminococcus callidus | Ruminococcus | Oscillospiraceae | Eubacteriales | Clostridia | Firmicutes | Bacteria |
| ASV4870 | 404 | 4 | 3 | 404 | 99.5 | NR_118511.1 | Campylobacter coli strain ATCC 33559 16S ribosomal RNA, partial sequence | NR_118511.1 | 1435 | 331 | 731 | 27.94 | 592 | 7E-169 | 377/404 | 93 | 5/404 | 1 | Plus | Plus | Bacteria; Proteobacteria; Epsilonproteobacteria; Campylobacterales; Campylobacteraceae; Campylobacter; Campylobacter coli | Campylobacter coli | Campylobacter | Campylobacteraceae | Campylobacterales | Epsilonproteobacteria | Proteobacteria | Bacteria |
| ASV4871 | 402 | 2 | 3 | 402 | 99.5 | NR_042144.1 | Clostridium butyricum strain VPI3266 16S ribosomal RNA, partial sequence | NR_042144.1 | 1425 | 340 | 739 | 28.07 | 601.3 | 1E-171 | 375/400 | 93 | 0/400 | 0 | Plus | Plus | Bacteria; Firmicutes; Clostridia; Eubacteriales; Clostridiaceae; Clostridium; Clostridium butyricum | Clostridium butyricum | Clostridium | Clostridiaceae | Eubacteriales | Clostridia | Firmicutes | Bacteria |
| ASV4873 | 402 | 1 | 1 | 402 | 100 | NR_044289.1 | [Clostridium] lavalense strain CCRI-9842 16S ribosomal RNA, partial sequence | NR_044289.1 | 1454 | 345 | 746 | 27.65 | 444.3 | 2E-124 | 350/404 | 86 | 4/404 | 0 | Plus | Plus | Bacteria; Firmicutes; Clostridia; Eubacteriales; Lachnospiraceae; Enterocloster; Enterocloster lavalensis | Enterocloster lavalensis | Enterocloster | Lachnospiraceae | Eubacteriales | Clostridia | Firmicutes | Bacteria |
| ASV4875 | 428 | 1 | 1 | 427 | 99.77 | NR_102980.1 | Megasphaera elsdenii DSM 20460 16S ribosomal RNA, partial sequence | NR_102980.1 | 1552 | 369 | 794 | 27.45 | 555.1 | 1E-157 | 388/430 | 90 | 7/430 | 1 | Plus | Plus | Bacteria; Firmicutes; Negativicutes; Veillonellales; Veillonellaceae; Megasphaera; Megasphaera elsdenii | Megasphaera elsdenii | Megasphaera | Veillonellaceae | Veillonellales | Negativicutes | Firmicutes | Bacteria |
| ASV4880 | 401 | 1 | 1 | 401 | 100 | NR_116936.1 | Olsenella umbonata strain lac31 16S ribosomal RNA, partial sequence | NR_116936.1 | 1428 | 328 | 735 | 28.57 | 558.8 | 7E-159 | 374/408 | 91 | 7/408 | 1 | Plus | Plus | Bacteria; Actinobacteria; Coriobacteriia; Coriobacteriales; Atopobiaceae; Olsenella; Olsenella umbonata | Olsenella umbonata | Olsenella | Atopobiaceae | Coriobacteriales | Coriobacteriia | Actinobacteria | Bacteria |
| ASV4883 | 402 | 1 | 1 | 402 | 100 | NR_102884.1 | Ruminococcus champanellensis 18P13 = JCM 17042 16S ribosomal RNA, partial sequence | NR_102884.1 | 1494 | 348 | 749 | 26.91 | 560.7 | 2E-159 | 370/403 | 91 | 2/403 | 0 | Plus | Plus | Bacteria; Firmicutes; Clostridia; Eubacteriales; Oscillospiraceae; Ruminococcus; Ruminococcus champanellensis | Ruminococcus champanellensis | Ruminococcus | Oscillospiraceae | Eubacteriales | Clostridia | Firmicutes | Bacteria |
| ASV4886 | 402 | 5 | 3 | 402 | 99.5 | NR_114728.1 | Clostridium tertium strain ATCC 14573 16S ribosomal RNA, partial sequence | NR_114728.1 | 1415 | 306 | 705 | 28.27 | 518.2 | 1E-146 | 361/401 | 90 | 2/401 | 0 | Plus | Plus | Bacteria; Firmicutes; Clostridia; Eubacteriales; Clostridiaceae; Clostridium; Clostridium tertium | Clostridium tertium | Clostridium | Clostridiaceae | Eubacteriales | Clostridia | Firmicutes | Bacteria |
| ASV4887 | 422 | 1 | 1 | 422 | 100 | NR_026204.1 | [Clostridium] viride strain T2-7 16S ribosomal RNA, partial sequence | NR_026204.1 | 1482 | 345 | 749 | 27.33 | 416.6 | 5E-116 | 362/425 | 85 | 23/425 | 5 | Plus | Plus | Bacteria; Firmicutes; Clostridia; Eubacteriales; Oscillospiraceae; ; [Clostridium] viride | [Clostridium] viride | | Oscillospiraceae | Eubacteriales | Clostridia | Firmicutes | Bacteria |
| ASV4888 | 403 | 2 | 1 | 403 | 100 | NR_118690.1 | [Ruminococcus] gnavus ATCC 29149 16S ribosomal RNA, partial sequence | NR_118690.1 | 1453 | 355 | 756 | 28.25 | 605 | 9E-173 | 378/403 | 93 | 1/403 | 0 | Plus | Plus | Bacteria; Firmicutes; Clostridia; Eubacteriales; Lachnospiraceae; Mediterraneibacter; [Ruminococcus] gnavus | [Ruminococcus] gnavus | Mediterraneibacter | Lachnospiraceae | Eubacteriales | Clostridia | Firmicutes | Bacteria |
| ASV4889 | 428 | 1 | 1 | 427 | 99.77 | NR_115726.1 | Desulfovibrio arcticus strain B15 16S ribosomal RNA, partial sequence | NR_115726.1 | 1425 | 316 | 742 | 30.04 | 529.3 | 6E-150 | 385/432 | 89 | 10/432 | 2 | Plus | Plus | Bacteria; Proteobacteria; Deltaproteobacteria; Desulfovibrionales; Desulfovibrionaceae; Desulfovibrio; Desulfovibrio arcticus | Desulfovibrio arcticus | Desulfovibrio | Desulfovibrionaceae | Desulfovibrionales | Deltaproteobacteria | Proteobacteria | Bacteria |
| ASV4890 | 420 | 2 | 1 | 420 | 100 | NR_113073.1 | Barnesiella intestinihominis strain JCM 15079 16S ribosomal RNA, partial sequence | NR_113073.1 | 1481 | 346 | 767 | 28.49 | 507.1 | 3E-143 | 375/424 | 88 | 6/424 | 1 | Plus | Plus | Bacteria; Bacteroidetes; Bacteroidia; Bacteroidales; Barnesiellaceae; Barnesiella; Barnesiella intestinihominis | Barnesiella intestinihominis | Barnesiella | Barnesiellaceae | Bacteroidales | Bacteroidia | Bacteroidetes | Bacteria |
| ASV4891 | 402 | 1 | 3 | 402 | 99.5 | NR_104899.1 | Bacteroides xylanolyticus strain X5-1 16S ribosomal RNA, partial sequence | NR_104899.1 | 1480 | 347 | 746 | 27.03 | 529.3 | 6E-150 | 366/404 | 90 | 8/404 | 1 | Plus | Plus | Bacteria; Firmicutes; Clostridia; Eubacteriales; Clostridiaceae; Hungatella; Hungatella xylanolytica | Hungatella xylanolytica | Hungatella | Clostridiaceae | Eubacteriales | Clostridia | Firmicutes | Bacteria |
| ASV4892 | 404 | 1 | 1 | 404 | 100 | NR_148619.1 | Agathobacter ruminis strain JK623 16S ribosomal RNA, partial sequence | NR_148619.1 | 1434 | 318 | 721 | 28.17 | 564.3 | 2E-160 | 375/408 | 91 | 8/408 | 1 | Plus | Plus | Bacteria; Firmicutes; Clostridia; Eubacteriales; Lachnospiraceae; Agathobacter; Agathobacter ruminis | Agathobacter ruminis | Agathobacter | Lachnospiraceae | Eubacteriales | Clostridia | Firmicutes | Bacteria |
| ASV4893 | 405 | 1 | 3 | 405 | 99.51 | NR_144611.1 | Flintibacter butyricus strain BLS21 16S ribosomal RNA, partial sequence | NR_144611.1 | 808 | 301 | 703 | 49.88 | 490.5 | 3E-138 | 358/404 | 88 | 2/404 | 0 | Plus | Plus | Bacteria; Firmicutes; Clostridia; Eubacteriales; ; Flintibacter; Flintibacter butyricus | Flintibacter butyricus | Flintibacter | | Eubacteriales | Clostridia | Firmicutes | Bacteria |
| ASV4896 | 427 | 1 | 1 | 427 | 100 | NR_028816.1 | Turicibacter sanguinis strain MOL361 16S ribosomal RNA, partial sequence | NR_028816.1 | 1508 | 327 | 753 | 28.32 | 457.2 | 3E-128 | 367/427 | 85 | 0/427 | 0 | Plus | Plus | Bacteria; Firmicutes; Erysipelotrichia; Erysipelotrichales; Turicibacteraceae; Turicibacter; Turicibacter sanguinis | Turicibacter sanguinis | Turicibacter | Turicibacteraceae | Erysipelotrichales | Erysipelotrichia | Firmicutes | Bacteria |
| ASV4902 | 421 | 2 | 1 | 421 | 100 | NR_113073.1 | Barnesiella intestinihominis strain JCM 15079 16S ribosomal RNA, partial sequence | NR_113073.1 | 1481 | 346 | 767 | 28.49 | 457.2 | 3E-128 | 365/423 | 86 | 3/423 | 0 | Plus | Plus | Bacteria; Bacteroidetes; Bacteroidia; Bacteroidales; Barnesiellaceae; Barnesiella; Barnesiella intestinihominis | Barnesiella intestinihominis | Barnesiella | Barnesiellaceae | Bacteroidales | Bacteroidia | Bacteroidetes | Bacteria |
| ASV4903 | 427 | 1 | 1 | 427 | 100 | NR_029335.1 | Holdemania filiformis strain J1-31B-1 16S ribosomal RNA, partial sequence | NR_029335.1 | 1391 | 267 | 692 | 30.63 | 555.1 | 1E-157 | 387/429 | 90 | 5/429 | 1 | Plus | Plus | Bacteria; Firmicutes; Erysipelotrichia; Erysipelotrichales; Erysipelotrichaceae; Holdemania; Holdemania filiformis | Holdemania filiformis | Holdemania | Erysipelotrichaceae | Erysipelotrichales | Erysipelotrichia | Firmicutes | Bacteria |
| ASV4904 | 405 | 1 | 3 | 405 | 99.51 | NR_104907.1 | Eubacterium coprostanoligenes strain HL 16S ribosomal RNA, partial sequence | NR_104907.1 | 1446 | 334 | 734 | 27.73 | 449.9 | 5E-126 | 352/405 | 86 | 6/405 | 1 | Plus | Plus | Bacteria; Firmicutes; Clostridia; Eubacteriales; Eubacteriaceae; Eubacterium; Eubacterium coprostanoligenes | Eubacterium coprostanoligenes | Eubacterium | Eubacteriaceae | Eubacteriales | Clostridia | Firmicutes | Bacteria |
| ASV4905 | 402 | 2 | 1 | 402 | 100 | NR_113231.1 | Blautia glucerasea strain JCM 17039 16S ribosomal RNA, partial sequence | NR_113231.1 | 1491 | 364 | 765 | 26.96 | 521.9 | 9E-148 | 363/403 | 90 | 2/403 | 0 | Plus | Plus | Bacteria; Firmicutes; Clostridia; Eubacteriales; Lachnospiraceae; Blautia; Blautia glucerasea | Blautia glucerasea | Blautia | Lachnospiraceae | Eubacteriales | Clostridia | Firmicutes | Bacteria |
| ASV4910 | 401 | 1 | 1 | 401 | 100 | NR_137408.1 | Terrisporobacter petrolearius strain LAM0A37 16S ribosomal RNA, partial sequence | NR_137408.1 | 1405 | 307 | 707 | 28.54 | 608.7 | 7E-174 | 377/401 | 94 | 0/401 | 0 | Plus | Plus | Bacteria; Firmicutes; Clostridia; Eubacteriales; Peptostreptococcaceae; Terrisporobacter; Terrisporobacter petrolearius | Terrisporobacter petrolearius | Terrisporobacter | Peptostreptococcaceae | Eubacteriales | Clostridia | Firmicutes | Bacteria |
| ASV4911 | 422 | 5 | 1 | 422 | 100 | NR_044864.1 | Hallella seregens ATCC 51272 16S ribosomal RNA, partial sequence | NR_044864.1 | 1463 | 335 | 756 | 28.84 | 553.3 | 4E-157 | 381/422 | 90 | 0/422 | 0 | Plus | Plus | Bacteria; Bacteroidetes; Bacteroidia; Bacteroidales; Prevotellaceae; Prevotella; Hallella seregens | Hallella seregens | Prevotella | Prevotellaceae | Bacteroidales | Bacteroidia | Bacteroidetes | Bacteria |
| ASV4914 | 422 | 2 | 1 | 422 | 100 | NR_025300.1 | Prevotella albensis strain M384 16S ribosomal RNA, partial sequence | NR_025300.1 | 1495 | 358 | 779 | 28.23 | 569.9 | 3E-162 | 384/422 | 90 | 0/422 | 0 | Plus | Plus | Bacteria; Bacteroidetes; Bacteroidia; Bacteroidales; Prevotellaceae; Prevotella; Prevotella albensis | Prevotella albensis | Prevotella | Prevotellaceae | Bacteroidales | Bacteroidia | Bacteroidetes | Bacteria |
| ASV4916 | 403 | 3 | 1 | 403 | 100 | NR_041781.1 | Streptococcus alactolyticus strain ATCC 43077 16S ribosomal RNA, partial sequence | NR_041781.1 | 1437 | 346 | 772 | 29.71 | 534.8 | 1E-151 | 385/427 | 90 | 24/427 | 5 | Plus | Plus | Bacteria; Firmicutes; Bacilli; Lactobacillales; Streptococcaceae; Streptococcus; Streptococcus alactolyticus | Streptococcus alactolyticus | Streptococcus | Streptococcaceae | Lactobacillales | Bacilli | Firmicutes | Bacteria |
| ASV4918 | 425 | 1 | 1 | 425 | 100 | NR_042896.1 | Mucispirillum schaedleri strain HRI I17 16S ribosomal RNA, partial sequence | NR_042896.1 | 1471 | 333 | 759 | 29.03 | 715.8 | 0 | 414/427 | 97 | 2/427 | 0 | Plus | Plus | Bacteria; Deferribacteres; Deferribacteres; Deferribacterales; Deferribacteraceae; Mucispirillum; Mucispirillum schaedleri | Mucispirillum schaedleri | Mucispirillum | Deferribacteraceae | Deferribacterales | Deferribacteres | Deferribacteres | Bacteria |
| ASV4919 | 422 | 2 | 1 | 422 | 100 | NR_074376.1 | Parabacteroides distasonis ATCC 8503 16S ribosomal RNA, partial sequence | NR_074376.1 | 1497 | 368 | 789 | 28.19 | 481.2 | 2E-135 | 368/422 | 87 | 0/422 | 0 | Plus | Plus | Bacteria; Bacteroidetes; Bacteroidia; Bacteroidales; Tannerellaceae; Parabacteroides; Parabacteroides distasonis | Parabacteroides distasonis | Parabacteroides | Tannerellaceae | Bacteroidales | Bacteroidia | Bacteroidetes | Bacteria |
| ASV4920 | 402 | 4 | 1 | 402 | 100 | NR_125677.1 | Vallitalea pronyensis strain FatNI3 16S ribosomal RNA, partial sequence | NR_125677.1 | 1480 | 363 | 764 | 27.16 | 477.6 | 2E-134 | 355/403 | 88 | 2/403 | 0 | Plus | Plus | Bacteria; Firmicutes; Clostridia; Eubacteriales; Vallitaleaceae; Vallitalea; Vallitalea pronyensis | Vallitalea pronyensis | Vallitalea | Vallitaleaceae | Eubacteriales | Clostridia | Firmicutes | Bacteria |
| ASV4921 | 422 | 2 | 1 | 422 | 100 | NR_113411.1 | Prevotella copri DSM 18205 strain JCM 13464 16S ribosomal RNA, partial sequence | NR_113411.1 | 1493 | 358 | 779 | 28.27 | 614.2 | 2E-175 | 393/423 | 93 | 2/423 | 0 | Plus | Plus | Bacteria; Bacteroidetes; Bacteroidia; Bacteroidales; Prevotellaceae; Prevotella; Prevotella copri | Prevotella copri | Prevotella | Prevotellaceae | Bacteroidales | Bacteroidia | Bacteroidetes | Bacteria |
| ASV4924 | 427 | 5 | 3 | 427 | 99.53 | NR_133868.1 | Streptococcus phocae subsp. salmonis strain C-4 16S ribosomal RNA, partial sequence | NR_133868.1 | 1410 | 293 | 717 | 30.14 | 547.7 | 2E-155 | 386/429 | 90 | 8/429 | 1 | Plus | Plus | Bacteria; Firmicutes; Bacilli; Lactobacillales; Streptococcaceae; Streptococcus; Streptococcus phocae | Streptococcus phocae | Streptococcus | Streptococcaceae | Lactobacillales | Bacilli | Firmicutes | Bacteria |
| ASV4925 | 402 | 1 | 1 | 401 | 99.75 | NR_151895.1 | Anaerocolumna cellulosilytica strain SN021 16S ribosomal RNA, partial sequence | NR_151895.1 | 1478 | 353 | 753 | 27.13 | 531.1 | 2E-150 | 363/401 | 90 | 0/401 | 0 | Plus | Plus | Bacteria; Firmicutes; Clostridia; Eubacteriales; Lachnospiraceae; Anaerocolumna; Anaerocolumna cellulosilytica | Anaerocolumna cellulosilytica | Anaerocolumna | Lachnospiraceae | Eubacteriales | Clostridia | Firmicutes | Bacteria |
| ASV4926 | 404 | 1 | 1 | 404 | 100 | NR_044054.1 | Blautia wexlerae DSM 19850 16S ribosomal RNA, partial sequence | NR_044054.1 | 1438 | 314 | 715 | 27.96 | 595.7 | 5E-170 | 380/407 | 93 | 8/407 | 1 | Plus | Plus | Bacteria; Firmicutes; Clostridia; Eubacteriales; Lachnospiraceae; Blautia; Blautia wexlerae | Blautia wexlerae | Blautia | Lachnospiraceae | Eubacteriales | Clostridia | Firmicutes | Bacteria |
| ASV4931 | 408 | 1 | 1 | 408 | 100 | NR_116936.1 | Olsenella umbonata strain lac31 16S ribosomal RNA, partial sequence | NR_116936.1 | 1428 | 328 | 735 | 28.57 | 682.5 | 0 | 395/408 | 96 | 0/408 | 0 | Plus | Plus | Bacteria; Actinobacteria; Coriobacteriia; Coriobacteriales; Atopobiaceae; Olsenella; Olsenella umbonata | Olsenella umbonata | Olsenella | Atopobiaceae | Coriobacteriales | Coriobacteriia | Actinobacteria | Bacteria |
| ASV4934 | 427 | 3 | 1 | 427 | 100 | NR_159156.1 | Neisseria dumasiana strain 93087 16S ribosomal RNA, partial sequence | NR_159156.1 | 1463 | 331 | 757 | 29.19 | 701 | 0 | 412/428 | 96 | 2/428 | 0 | Plus | Plus | Bacteria; Proteobacteria; Betaproteobacteria; Neisseriales; Neisseriaceae; Neisseria; Neisseria dumasiana | Neisseria dumasiana | Neisseria | Neisseriaceae | Neisseriales | Betaproteobacteria | Proteobacteria | Bacteria |
| ASV4938 | 406 | 1 | 1 | 406 | 100 | NR_144740.1 | Romboutsia timonensis strain DR1 16S ribosomal RNA, partial sequence | NR_144740.1 | 1466 | 338 | 738 | 27.35 | 629 | 5E-180 | 386/407 | 94 | 7/407 | 1 | Plus | Plus | Bacteria; Firmicutes; Clostridia; Eubacteriales; Peptostreptococcaceae; Romboutsia; Romboutsia timonensis | Romboutsia timonensis | Romboutsia | Peptostreptococcaceae | Eubacteriales | Clostridia | Firmicutes | Bacteria |
| ASV4940 | 405 | 1 | 1 | 405 | 100 | NR_029097.1 | Oribacterium sinus strain AIP 354.02 16S ribosomal RNA, partial sequence | NR_029097.1 | 1374 | 308 | 709 | 29.26 | 507.1 | 3E-143 | 362/405 | 89 | 3/405 | 0 | Plus | Plus | Bacteria; Firmicutes; Clostridia; Eubacteriales; Lachnospiraceae; Oribacterium; Oribacterium sinus | Oribacterium sinus | Oribacterium | Lachnospiraceae | Eubacteriales | Clostridia | Firmicutes | Bacteria |
| ASV4942 | 404 | 1 | 1 | 404 | 100 | NR_117506.1 | Bifidobacterium longum strain KCTC 3128 16S ribosomal RNA, partial sequence | NR_117506.1 | 1477 | 328 | 734 | 27.56 | 582.8 | 4E-166 | 377/407 | 92 | 3/407 | 0 | Plus | Plus | Bacteria; Actinobacteria; Actinomycetia; Bifidobacteriales; Bifidobacteriaceae; Bifidobacterium; Bifidobacterium longum | Bifidobacterium longum | Bifidobacterium | Bifidobacteriaceae | Bifidobacteriales | Actinomycetia | Actinobacteria | Bacteria |
| ASV4944 | 405 | 1 | 1 | 405 | 100 | NR_133738.1 | Desulfohalotomaculum tongense strain TGB60-1 16S ribosomal RNA, partial sequence | NR_133738.1 | 1447 | 352 | 756 | 27.99 | 438.8 | 1E-122 | 351/407 | 86 | 4/407 | 0 | Plus | Plus | Bacteria; Firmicutes; Clostridia; Eubacteriales; Peptococcaceae; Desulfohalotomaculum; Desulfohalotomaculum tongense | Desulfohalotomaculum tongense | Desulfohalotomaculum | Peptococcaceae | Eubacteriales | Clostridia | Firmicutes | Bacteria |
| ASV4947 | 406 | 1 | 3 | 406 | 99.51 | NR_145929.1 | Caproiciproducens galactitolivorans strain BS-1 16S ribosomal RNA, partial sequence | NR_145929.1 | 1445 | 338 | 741 | 27.96 | 497.9 | 2E-140 | 362/407 | 88 | 6/407 | 1 | Plus | Plus | Bacteria; Firmicutes; Clostridia; Eubacteriales; Oscillospiraceae; Caproiciproducens; Caproiciproducens galactitolivorans | Caproiciproducens galactitolivorans | Caproiciproducens | Oscillospiraceae | Eubacteriales | Clostridia | Firmicutes | Bacteria |
| ASV4951 | 402 | 5 | 1 | 402 | 100 | NR_113388.1 | Clostridium beijerinckii strain JCM 1390 16S ribosomal RNA, partial sequence | NR_113388.1 | 1473 | 348 | 749 | 27.29 | 433.2 | 5E-121 | 348/404 | 86 | 4/404 | 0 | Plus | Plus | Bacteria; Firmicutes; Clostridia; Eubacteriales; Clostridiaceae; Clostridium; Clostridium beijerinckii | Clostridium beijerinckii | Clostridium | Clostridiaceae | Eubacteriales | Clostridia | Firmicutes | Bacteria |
| ASV4952 | 370 | 3 | 41 | 370 | 89.19 | NR_121773.2 | Barnesiella viscericola strain C46 16S ribosomal RNA, complete sequence | NR_121773.2 | 1532 | 456 | 786 | 21.61 | 405.5 | 9E-113 | 294/331 | 88 | 1/331 | 0 | Plus | Plus | Bacteria; Bacteroidetes; Bacteroidia; Bacteroidales; Barnesiellaceae; Barnesiella; Barnesiella viscericola | Barnesiella viscericola | Barnesiella | Barnesiellaceae | Bacteroidales | Bacteroidia | Bacteroidetes | Bacteria |
| ASV4955 | 402 | 1 | 1 | 402 | 100 | NR_044289.1 | [Clostridium] lavalense strain CCRI-9842 16S ribosomal RNA, partial sequence | NR_044289.1 | 1454 | 345 | 746 | 27.65 | 483.1 | 4E-136 | 356/403 | 88 | 2/403 | 0 | Plus | Plus | Bacteria; Firmicutes; Clostridia; Eubacteriales; Lachnospiraceae; Enterocloster; Enterocloster lavalensis | Enterocloster lavalensis | Enterocloster | Lachnospiraceae | Eubacteriales | Clostridia | Firmicutes | Bacteria |
| ASV4956 | 422 | 1 | 1 | 422 | 100 | NR_041342.1 | Parabacteroides distasonis strain JCM 5825 16S ribosomal RNA, partial sequence | NR_041342.1 | 1488 | 353 | 774 | 28.36 | 536.6 | 4E-152 | 380/424 | 89 | 4/424 | 0 | Plus | Plus | Bacteria; Bacteroidetes; Bacteroidia; Bacteroidales; Tannerellaceae; Parabacteroides; Parabacteroides distasonis | Parabacteroides distasonis | Parabacteroides | Tannerellaceae | Bacteroidales | Bacteroidia | Bacteroidetes | Bacteria |
| ASV4959 | 402 | 1 | 1 | 402 | 100 | NR_029263.1 | Clostridium intestinale strain Catt39 16S ribosomal RNA, partial sequence | NR_029263.1 | 1385 | 304 | 705 | 29.03 | 510.8 | 2E-144 | 360/402 | 89 | 0/402 | 0 | Plus | Plus | Bacteria; Firmicutes; Clostridia; Eubacteriales; Clostridiaceae; Clostridium; Clostridium intestinale | Clostridium intestinale | Clostridium | Clostridiaceae | Eubacteriales | Clostridia | Firmicutes | Bacteria |
| ASV4960 | 404 | 1 | 1 | 404 | 100 | NR_041236.1 | Lutispora thermophila DSM 19022 strain EBR46 16S ribosomal RNA, partial sequence | NR_041236.1 | 1438 | 338 | 740 | 28.03 | 418.5 | 1E-116 | 348/407 | 85 | 7/407 | 1 | Plus | Plus | Bacteria; Firmicutes; Clostridia; Eubacteriales; Clostridiaceae; Lutispora; Lutispora thermophila | Lutispora thermophila | Lutispora | Clostridiaceae | Eubacteriales | Clostridia | Firmicutes | Bacteria |
| ASV4963 | 402 | 1 | 3 | 402 | 99.5 | NR_044054.1 | Blautia wexlerae DSM 19850 16S ribosomal RNA, partial sequence | NR_044054.1 | 1438 | 316 | 715 | 27.82 | 595.7 | 5E-170 | 374/400 | 93 | 0/400 | 0 | Plus | Plus | Bacteria; Firmicutes; Clostridia; Eubacteriales; Lachnospiraceae; Blautia; Blautia wexlerae | Blautia wexlerae | Blautia | Lachnospiraceae | Eubacteriales | Clostridia | Firmicutes | Bacteria |
| ASV4966 | 410 | 1 | 1 | 410 | 100 | NR_116936.1 | Olsenella umbonata strain lac31 16S ribosomal RNA, partial sequence | NR_116936.1 | 1428 | 328 | 735 | 28.57 | 601.3 | 1E-171 | 382/410 | 93 | 2/410 | 0 | Plus | Plus | Bacteria; Actinobacteria; Coriobacteriia; Coriobacteriales; Atopobiaceae; Olsenella; Olsenella umbonata | Olsenella umbonata | Olsenella | Atopobiaceae | Coriobacteriales | Coriobacteriia | Actinobacteria | Bacteria |
| ASV4968 | 405 | 5 | 4 | 405 | 99.26 | NR_115693.1 | Gracilibacter thermotolerans JW/YJL-S1 16S ribosomal RNA, partial sequence | NR_115693.1 | 1526 | 462 | 861 | 26.21 | 442.5 | 8E-124 | 349/403 | 86 | 4/403 | 0 | Plus | Plus | Bacteria; Firmicutes; Clostridia; Eubacteriales; Gracilibacteraceae; Gracilibacter; Gracilibacter thermotolerans | Gracilibacter thermotolerans | Gracilibacter | Gracilibacteraceae | Eubacteriales | Clostridia | Firmicutes | Bacteria |
| ASV4970 | 422 | 1 | 1 | 422 | 100 | NR_041343.1 | Parabacteroides merdae strain JCM 9497 16S ribosomal RNA, partial sequence | NR_041343.1 | 1492 | 357 | 778 | 28.28 | 619.7 | 3E-177 | 395/424 | 93 | 4/424 | 0 | Plus | Plus | Bacteria; Bacteroidetes; Bacteroidia; Bacteroidales; Tannerellaceae; Parabacteroides; Parabacteroides merdae | Parabacteroides merdae | Parabacteroides | Tannerellaceae | Bacteroidales | Bacteroidia | Bacteroidetes | Bacteria |
| ASV4973 | 402 | 1 | 1 | 402 | 100 | NR_159261.1 | Bifidobacterium callitrichidarum strain TRI 5 16S ribosomal RNA, partial sequence | NR_159261.1 | 1374 | 279 | 686 | 29.69 | 538.5 | 9E-153 | 370/408 | 90 | 6/408 | 1 | Plus | Plus | Bacteria; Actinobacteria; Actinomycetia; Bifidobacteriales; Bifidobacteriaceae; Bifidobacterium; Bifidobacterium callitrichidarum | Bifidobacterium callitrichidarum | Bifidobacterium | Bifidobacteriaceae | Bifidobacteriales | Actinomycetia | Actinobacteria | Bacteria |
| ASV4977 | 422 | 1 | 1 | 395 | 93.6 | NR_113411.1 | Prevotella copri DSM 18205 strain JCM 13464 16S ribosomal RNA, partial sequence | NR_113411.1 | 1493 | 358 | 752 | 26.46 | 581 | 2E-165 | 370/397 | 93 | 4/397 | 1 | Plus | Plus | Bacteria; Bacteroidetes; Bacteroidia; Bacteroidales; Prevotellaceae; Prevotella; Prevotella copri | Prevotella copri | Prevotella | Prevotellaceae | Bacteroidales | Bacteroidia | Bacteroidetes | Bacteria |
| ASV4978 | 403 | 1 | 1 | 403 | 100 | NR_117912.1 | Defluviitalea saccharophila strain LIND6LT2 16S ribosomal RNA, partial sequence | NR_117912.1 | 1495 | 342 | 746 | 27.09 | 553.3 | 3E-157 | 371/406 | 91 | 4/406 | 0 | Plus | Plus | Bacteria; Firmicutes; Clostridia; Eubacteriales; Defluviitaleaceae; Defluviitalea; Defluviitalea saccharophila | Defluviitalea saccharophila | Defluviitalea | Defluviitaleaceae | Eubacteriales | Clostridia | Firmicutes | Bacteria |
| ASV4979 | 402 | 1 | 3 | 402 | 99.5 | NR_025930.1 | Ruminococcus bromii strain ATCC 27255 16S ribosomal RNA, partial sequence | NR_025930.1 | 1380 | 315 | 716 | 29.13 | 586.5 | 3E-167 | 374/402 | 93 | 2/402 | 0 | Plus | Plus | Bacteria; Firmicutes; Clostridia; Eubacteriales; Oscillospiraceae; Ruminococcus; Ruminococcus bromii | Ruminococcus bromii | Ruminococcus | Oscillospiraceae | Eubacteriales | Clostridia | Firmicutes | Bacteria |
| ASV4981 | 402 | 2 | 1 | 402 | 100 | NR_024995.1 | Clostridium putrefaciens strain DSM 1291 16S ribosomal RNA, partial sequence | NR_024995.1 | 1487 | 348 | 749 | 27.03 | 499.7 | 4E-141 | 359/403 | 89 | 2/403 | 0 | Plus | Plus | Bacteria; Firmicutes; Clostridia; Eubacteriales; Clostridiaceae; Clostridium; Clostridium putrefaciens | Clostridium putrefaciens | Clostridium | Clostridiaceae | Eubacteriales | Clostridia | Firmicutes | Bacteria |
| ASV4982 | 421 | 2 | 1 | 412 | 97.86 | NR_113073.1 | Barnesiella intestinihominis strain JCM 15079 16S ribosomal RNA, partial sequence | NR_113073.1 | 1481 | 346 | 758 | 27.89 | 468.3 | 1E-131 | 362/415 | 87 | 5/415 | 1 | Plus | Plus | Bacteria; Bacteroidetes; Bacteroidia; Bacteroidales; Barnesiellaceae; Barnesiella; Barnesiella intestinihominis | Barnesiella intestinihominis | Barnesiella | Barnesiellaceae | Bacteroidales | Bacteroidia | Bacteroidetes | Bacteria |
| ASV4983 | 402 | 1 | 1 | 402 | 100 | NR_036822.1 | Clostridium frigoris strain D-1/D-an/II 16S ribosomal RNA, partial sequence | NR_036822.1 | 1477 | 348 | 749 | 27.22 | 521.9 | 9E-148 | 362/402 | 90 | 0/402 | 0 | Plus | Plus | Bacteria; Firmicutes; Clostridia; Eubacteriales; Clostridiaceae; Clostridium; Clostridium frigoris | Clostridium frigoris | Clostridium | Clostridiaceae | Eubacteriales | Clostridia | Firmicutes | Bacteria |
| ASV4986 | 402 | 1 | 3 | 402 | 99.5 | NR_025930.1 | Ruminococcus bromii strain ATCC 27255 16S ribosomal RNA, partial sequence | NR_025930.1 | 1380 | 315 | 716 | 29.13 | 592 | 7E-169 | 375/402 | 93 | 2/402 | 0 | Plus | Plus | Bacteria; Firmicutes; Clostridia; Eubacteriales; Oscillospiraceae; Ruminococcus; Ruminococcus bromii | Ruminococcus bromii | Ruminococcus | Oscillospiraceae | Eubacteriales | Clostridia | Firmicutes | Bacteria |
| ASV4987 | 401 | 1 | 1 | 401 | 100 | NR_152095.1 | Herbinix luporum strain SD1D 16S ribosomal RNA, partial sequence | NR_152095.1 | 1521 | 365 | 766 | 26.43 | 508.9 | 7E-144 | 361/403 | 89 | 3/403 | 0 | Plus | Plus | Bacteria; Firmicutes; Clostridia; Eubacteriales; Lachnospiraceae; Herbinix; Herbinix luporum | Herbinix luporum | Herbinix | Lachnospiraceae | Eubacteriales | Clostridia | Firmicutes | Bacteria |
| ASV4989 | 422 | 1 | 1 | 422 | 100 | NR_171489.1 | Coraliomargarita sinensis strain WN38 16S ribosomal RNA, partial sequence | NR_171489.1 | 1542 | 367 | 788 | 27.37 | 464.6 | 2E-130 | 366/423 | 86 | 2/423 | 0 | Plus | Plus | Bacteria; Verrucomicrobia; Opitutae; Puniceicoccales; Puniceicoccaceae; Coraliomargarita; Coraliomargarita sinensis | Coraliomargarita sinensis | Coraliomargarita | Puniceicoccaceae | Puniceicoccales | Opitutae | Verrucomicrobia | Bacteria |
| ASV4995 | 402 | 1 | 1 | 402 | 100 | NR_137405.1 | Anaerobium acetethylicum strain GluBS11 16S ribosomal RNA, partial sequence | NR_137405.1 | 1402 | 324 | 725 | 28.67 | 483.1 | 4E-136 | 355/402 | 88 | 0/402 | 0 | Plus | Plus | Bacteria; Firmicutes; Clostridia; Eubacteriales; Lachnospiraceae; Anaerobium; Anaerobium acetethylicum | Anaerobium acetethylicum | Anaerobium | Lachnospiraceae | Eubacteriales | Clostridia | Firmicutes | Bacteria |
| ASV4997 | 422 | 1 | 1 | 422 | 100 | NR_116999.1 | Cronobacter condimenti 1330 16S ribosomal RNA, partial sequence | NR_116999.1 | 1413 | 263 | 689 | 30.22 | 529.3 | 6E-150 | 385/431 | 89 | 13/431 | 3 | Plus | Plus | Bacteria; Proteobacteria; Gammaproteobacteria; Enterobacterales; Enterobacteriaceae; Cronobacter; Cronobacter condimenti | Cronobacter condimenti | Cronobacter | Enterobacteriaceae | Enterobacterales | Gammaproteobacteria | Proteobacteria | Bacteria |
| ASV5000 | 422 | 1 | 1 | 422 | 100 | NR_041343.1 | Parabacteroides merdae strain JCM 9497 16S ribosomal RNA, partial sequence | NR_041343.1 | 1492 | 357 | 778 | 28.28 | 603.1 | 3E-172 | 393/425 | 92 | 6/425 | 1 | Plus | Plus | Bacteria; Bacteroidetes; Bacteroidia; Bacteroidales; Tannerellaceae; Parabacteroides; Parabacteroides merdae | Parabacteroides merdae | Parabacteroides | Tannerellaceae | Bacteroidales | Bacteroidia | Bacteroidetes | Bacteria |
| ASV5003 | 402 | 1 | 1 | 402 | 100 | NR_144612.1 | Frisingicoccus caecimuris strain PG-426-CC-1 16S ribosomal RNA, partial sequence | NR_144612.1 | 1448 | 367 | 768 | 27.76 | 577.3 | 2E-164 | 374/404 | 92 | 4/404 | 0 | Plus | Plus | Bacteria; Firmicutes; Clostridia; Eubacteriales; Lachnospiraceae; Frisingicoccus; Frisingicoccus caecimuris | Frisingicoccus caecimuris | Frisingicoccus | Lachnospiraceae | Eubacteriales | Clostridia | Firmicutes | Bacteria |
| ASV5004 | 428 | 1 | 5 | 428 | 99.07 | NR_025131.1 | Treponema parvum strain OMZ 833 16S ribosomal RNA, partial sequence | NR_025131.1 | 1485 | 345 | 767 | 28.48 | 466.5 | 5E-131 | 370/427 | 86 | 7/427 | 1 | Plus | Plus | Bacteria; Spirochaetes; Spirochaetia; Spirochaetales; Spirochaetaceae; Treponema; Treponema parvum | Treponema parvum | Treponema | Spirochaetaceae | Spirochaetales | Spirochaetia | Spirochaetes | Bacteria |
| ASV5006 | 426 | 1 | 5 | 425 | 98.83 | NR_027526.1 | Catenibacterium mitsuokai strain DSM 15897 16S ribosomal RNA, partial sequence | NR_027526.1 | 1485 | 349 | 767 | 28.22 | 577.3 | 2E-164 | 387/423 | 91 | 6/423 | 1 | Plus | Plus | Bacteria; Firmicutes; Erysipelotrichia; Erysipelotrichales; Coprobacillaceae; Catenibacterium; Catenibacterium mitsuokai | Catenibacterium mitsuokai | Catenibacterium | Coprobacillaceae | Erysipelotrichales | Erysipelotrichia | Firmicutes | Bacteria |
| ASV5008 | 426 | 2 | 1 | 425 | 99.77 | NR_027596.1 | Mitsuokella multacida strain DSM 20544 16S ribosomal RNA, partial sequence | NR_027596.1 | 1511 | 346 | 771 | 28.19 | 582.8 | 5E-166 | 392/429 | 91 | 7/429 | 1 | Plus | Plus | Bacteria; Firmicutes; Negativicutes; Selenomonadales; Selenomonadaceae; Mitsuokella; Mitsuokella multacida | Mitsuokella multacida | Mitsuokella | Selenomonadaceae | Selenomonadales | Negativicutes | Firmicutes | Bacteria |
| ASV5009 | 424 | 1 | 1 | 409 | 96.46 | NR_118334.1 | Alloprevotella rava strain 81/4-12 16S ribosomal RNA, partial sequence | NR_118334.1 | 1455 | 338 | 744 | 27.97 | 516.3 | 5E-146 | 368/411 | 89 | 6/411 | 1 | Plus | Plus | Bacteria; Bacteroidetes; Bacteroidia; Bacteroidales; Prevotellaceae; Alloprevotella; Alloprevotella rava | Alloprevotella rava | Alloprevotella | Prevotellaceae | Bacteroidales | Bacteroidia | Bacteroidetes | Bacteria |
| ASV5010 | 426 | 1 | 1 | 426 | 100 | NR_027596.1 | Mitsuokella multacida strain DSM 20544 16S ribosomal RNA, partial sequence | NR_027596.1 | 1511 | 346 | 772 | 28.26 | 568 | 1E-161 | 390/430 | 90 | 7/430 | 1 | Plus | Plus | Bacteria; Firmicutes; Negativicutes; Selenomonadales; Selenomonadaceae; Mitsuokella; Mitsuokella multacida | Mitsuokella multacida | Mitsuokella | Selenomonadaceae | Selenomonadales | Negativicutes | Firmicutes | Bacteria |
| ASV5015 | 407 | 1 | 1 | 407 | 100 | NR_028883.1 | Dorea longicatena strain 111-35 16S ribosomal RNA, partial sequence | NR_028883.1 | 1316 | 248 | 645 | 30.62 | 605 | 9E-173 | 383/409 | 93 | 8/409 | 1 | Plus | Plus | Bacteria; Firmicutes; Clostridia; Eubacteriales; Lachnospiraceae; Dorea; Dorea longicatena | Dorea longicatena | Dorea | Lachnospiraceae | Eubacteriales | Clostridia | Firmicutes | Bacteria |
| ASV5017 | 427 | 2 | 1 | 375 | 87.82 | NR_037018.1 | Acidaminococcus fermentans strain VR4 16S ribosomal RNA gene, partial sequence | NR_037018.1 | 1516 | 349 | 723 | 24.74 | 654.8 | 0 | 368/375 | 98 | 0/375 | 0 | Plus | Plus | Bacteria; Firmicutes; Negativicutes; Acidaminococcales; Acidaminococcaceae; Acidaminococcus; Acidaminococcus fermentans | Acidaminococcus fermentans | Acidaminococcus | Acidaminococcaceae | Acidaminococcales | Negativicutes | Firmicutes | Bacteria |
| ASV5018 | 405 | 2 | 1 | 405 | 100 | NR_113119.1 | Prevotella oris strain JCM 8540 16S ribosomal RNA, partial sequence | NR_113119.1 | 1481 | 345 | 766 | 28.49 | 420.3 | 4E-117 | 361/423 | 85 | 19/423 | 4 | Plus | Plus | Bacteria; Bacteroidetes; Bacteroidia; Bacteroidales; Prevotellaceae; Prevotella; Prevotella oris | Prevotella oris | Prevotella | Prevotellaceae | Bacteroidales | Bacteroidia | Bacteroidetes | Bacteria |
| ASV5021 | 426 | 2 | 1 | 426 | 100 | NR_029352.1 | Lactobacillus amylolyticus strain DSM 11664 strain LA 5 16S ribosomal RNA, partial sequence | NR_029352.1 | 1544 | 361 | 787 | 27.66 | 444.3 | 2E-124 | 368/430 | 85 | 7/430 | 1 | Plus | Plus | Bacteria; Firmicutes; Bacilli; Lactobacillales; Lactobacillaceae; Lactobacillus; Lactobacillus amylolyticus | Lactobacillus amylolyticus | Lactobacillus | Lactobacillaceae | Lactobacillales | Bacilli | Firmicutes | Bacteria |
| ASV5023 | 402 | 1 | 3 | 393 | 97.26 | NR_121686.1 | [Bacteroides] pectinophilus strain N3 16S ribosomal RNA, partial sequence | NR_121686.1 | 1521 | 367 | 757 | 25.71 | 440.6 | 3E-123 | 341/392 | 86 | 2/392 | 0 | Plus | Plus | Bacteria; Firmicutes; Clostridia; Eubacteriales; ; ; [Bacteroides] pectinophilus | [Bacteroides] pectinophilus | | | Eubacteriales | Clostridia | Firmicutes | Bacteria |

| | | | | | | | | | | | | | | | | | | | | | | | | | | | |
|---|---|---|---|---|---|---|---|---|---|---|---|---|---|---|---|---|---|---|---|---|---|---|---|---|---|---|---|
| ASV5105 | 422 | 1 | 1 | 422 | 100 | NR_041446.1 Bacteroides barnesiae strain BL2 16S ribosomal RNA, partial sequence | NR_041446.1 | 1456 | 340 | 761 | 28.98 | 547.7 | 2E-155 | 387/429 | 90 | 14/429 | 3 | Plus | Plus | Bacteria; Bacteroidetes; Bacteroidia; Bacteroidales; ; Phocaeicola; Phocaeicola barnesiae | Phocaeicola barnesiae | Phocaeicola | | Bacteroidales | Bacteroidia | Bacteroidetes | Bacteria |
| ASV5106 | 421 | 2 | 1 | 406 | 96.44 | NR_113104.1 Prevotella enoeca strain JCM 12259 16S ribosomal RNA, partial sequence | NR_113104.1 | 1480 | 345 | 751 | 27.5 | 446.2 | 6E-125 | 357/412 | 86 | 11/412 | 2 | Plus | Plus | Bacteria; Bacteroidetes; Bacteroidia; Bacteroidales; Prevotellaceae; Prevotella; Prevotella enoeca | Prevotella enoeca | Prevotella | Prevotellaceae | Bacteroidales | Bacteroidia | Bacteroidetes | Bacteria |
| ASV5110 | 402 | 2 | 3 | 402 | 99.5 | NR_118690.1 [Ruminococcus] gnavus ATCC 29149 16S ribosomal RNA, partial sequence | NR_118690.1 | 1423 | 357 | 756 | 28.11 | 606.8 | 3E-173 | 376/400 | 94 | 0/400 | 0 | Plus | Plus | Bacteria; Firmicutes; Clostridia; Eubacteriales; Lachnospiraceae; Mediterraneibacter; [Ruminococcus] gnavus | [Ruminococcus] gnavus | Mediterraneibacter | Lachnospiraceae | Eubacteriales | Clostridia | Firmicutes | Bacteria |
| ASV5113 | 405 | 2 | 1 | 405 | 100 | NR_074629.1 Hungateiclostridium thermocellum strain ATCC 27405 16S ribosomal RNA, partial sequence | NR_074629.1 | 1521 | 358 | 762 | 26.63 | 488.6 | 1E-137 | 358/405 | 88 | 0/405 | 0 | Plus | Plus | Bacteria; Firmicutes; Clostridia; Eubacteriales; Oscillospiraceae; Acetivibrio; Acetivibrio thermocellus | Acetivibrio thermocellus | Acetivibrio | Oscillospiraceae | Eubacteriales | Clostridia | Firmicutes | Bacteria |
| ASV5114 | 427 | 1 | 4 | 425 | 98.83 | NR_125593.1 Dielma fastidiosa strain JC13 16S ribosomal RNA, partial sequence | NR_125593.1 | 1499 | 358 | 778 | 28.09 | 473.9 | 3E-133 | 370/425 | 87 | 7/425 | 1 | Plus | Plus | Bacteria; Firmicutes; Erysipelotrichia; Erysipelotrichales; Erysipelotrichaceae; Dielma; Dielma fastidiosa | Dielma fastidiosa | Dielma | Erysipelotrichaceae | Erysipelotrichales | Erysipelotrichia | Firmicutes | Bacteria |
| ASV5116 | 405 | 1 | 1 | 405 | 100 | NR_102884.1 Ruminococcus champanellensis 18P13 = JCM 17042 16S ribosomal RNA, partial sequence | NR_102884.1 | 1494 | 348 | 749 | 26.91 | 457.2 | 3E-128 | 355/407 | 87 | 7/407 | 1 | Plus | Plus | Bacteria; Firmicutes; Clostridia; Eubacteriales; Oscillospiraceae; Ruminococcus; Ruminococcus champanellensis | Ruminococcus champanellensis | Ruminococcus | Oscillospiraceae | Eubacteriales | Clostridia | Firmicutes | Bacteria |
| ASV5117 | 402 | 2 | 1 | 402 | 100 | NR_042144.1 Clostridium butyricum strain VPI3266 16S ribosomal RNA, partial sequence | NR_042144.1 | 1425 | 338 | 739 | 28.21 | 616.1 | 4E-176 | 380/403 | 94 | 2/403 | 0 | Plus | Plus | Bacteria; Firmicutes; Clostridia; Eubacteriales; Clostridiaceae; Clostridium; Clostridium butyricum | Clostridium butyricum | Clostridium | Clostridiaceae | Eubacteriales | Clostridia | Firmicutes | Bacteria |
| ASV5118 | 428 | 1 | 1 | 413 | 96.5 | NR_102980.1 Megasphaera elsdenii DSM 20460 16S ribosomal RNA, partial sequence | NR_102980.1 | 1552 | 369 | 780 | 26.55 | 462.8 | 6E-130 | 362/416 | 87 | 7/416 | 1 | Plus | Plus | Bacteria; Firmicutes; Negativicutes; Veillonellales; Veillonellaceae; Megasphaera; Megasphaera elsdenii | Megasphaera elsdenii | Megasphaera | Veillonellaceae | Veillonellales | Negativicutes | Firmicutes | Bacteria |
| ASV5120 | 402 | 3 | 5 | 402 | 99 | NR_117139.2 Anaerostipes hadrus strain DSM 3319 16S ribosomal RNA, partial sequence | NR_117139.2 | 1521 | 367 | 764 | 26.17 | 508.9 | 7E-144 | 358/399 | 89 | 2/399 | 0 | Plus | Plus | Bacteria; Firmicutes; Clostridia; Eubacteriales; Lachnospiraceae; Anaerostipes; Anaerostipes hadrus | Anaerostipes hadrus | Anaerostipes | Lachnospiraceae | Eubacteriales | Clostridia | Firmicutes | Bacteria |
| ASV5122 | 401 | 4 | 1 | 401 | 100 | NR_119068.1 [Clostridium] aerotolerans strain DSM 5434 16S ribosomal RNA, partial sequence | NR_119068.1 | 1470 | 332 | 732 | 27.28 | 409.2 | 8E-114 | 343/403 | 85 | 4/403 | 0 | Plus | Plus | Bacteria; Firmicutes; Clostridia; Eubacteriales; Lachnospiraceae; Lacrimispora; Lacrimispora aerotolerans | Lacrimispora aerotolerans | Lacrimispora | Lachnospiraceae | Eubacteriales | Clostridia | Firmicutes | Bacteria |
| ASV5123 | 404 | 3 | 1 | 404 | 100 | NR_156081.1 Kineothrix alysoides strain KNHs209 16S ribosomal RNA, partial sequence | NR_156081.1 | 1411 | 353 | 754 | 28.49 | 551.4 | 1E-156 | 370/405 | 91 | 4/405 | 0 | Plus | Plus | Bacteria; Firmicutes; Clostridia; Eubacteriales; Lachnospiraceae; Kineothrix; Kineothrix alysoides | Kineothrix alysoides | Kineothrix | Lachnospiraceae | Eubacteriales | Clostridia | Firmicutes | Bacteria |
| ASV5124 | 405 | 3 | 1 | 405 | 100 | NR_148619.1 Agathobacter ruminis strain JK623 16S ribosomal RNA, partial sequence | NR_148619.1 | 1434 | 318 | 721 | 28.17 | 603.1 | 3E-172 | 380/406 | 93 | 3/406 | 0 | Plus | Plus | Bacteria; Firmicutes; Clostridia; Eubacteriales; Lachnospiraceae; Agathobacter; Agathobacter ruminis | Agathobacter ruminis | Agathobacter | Lachnospiraceae | Eubacteriales | Clostridia | Firmicutes | Bacteria |
| ASV5125 | 405 | 2 | 1 | 405 | 100 | NR_029160.1 Ruminococcus callidus strain ATCC 27760 16S ribosomal RNA, partial sequence | NR_029160.1 | 1371 | 310 | 711 | 29.32 | 545.9 | 6E-155 | 371/407 | 91 | 7/407 | 1 | Plus | Plus | Bacteria; Firmicutes; Clostridia; Eubacteriales; Oscillospiraceae; Ruminococcus; Ruminococcus callidus | Ruminococcus callidus | Ruminococcus | Oscillospiraceae | Eubacteriales | Clostridia | Firmicutes | Bacteria |
| ASV5126 | 403 | 4 | 1 | 403 | 100 | NR_074399.1 Ruminococcus albus 7 = DSM 20455 16S ribosomal RNA, partial sequence | NR_074399.1 | 1500 | 350 | 751 | 26.8 | 449.9 | 5E-126 | 350/403 | 86 | 1/403 | 0 | Plus | Plus | Bacteria; Firmicutes; Clostridia; Eubacteriales; Oscillospiraceae; Ruminococcus; Ruminococcus albus | Ruminococcus albus | Ruminococcus | Oscillospiraceae | Eubacteriales | Clostridia | Firmicutes | Bacteria |
| ASV5129 | 406 | 1 | 4 | 406 | 99.26 | NR_026476.1 Succinivibrio dextrinosolvens strain 0554 16S ribosomal RNA, partial sequence | NR_026476.1 | 1463 | 334 | 732 | 27.27 | 464.6 | 2E-130 | 353/403 | 87 | 4/403 | 0 | Plus | Plus | Bacteria; Proteobacteria; Gammaproteobacteria; Aeromonadales; Succinivibrionaceae; Succinivibrio; Succinivibrio dextrinosolvens | Succinivibrio dextrinosolvens | Succinivibrio | Succinivibrionaceae | Aeromonadales | Gammaproteobacteria | Proteobacteria | Bacteria |
| ASV5132 | 427 | 1 | 5 | 427 | 99.06 | NR_024883.1 Kluyvera georgiana strain ATCC 51603 16S ribosomal RNA, partial sequence | NR_024883.1 | 1414 | 327 | 749 | 29.92 | 553.3 | 4E-157 | 382/425 | 89 | 4/425 | 0 | Plus | Plus | Bacteria; Proteobacteria; Gammaproteobacteria; Enterobacterales; Enterobacteriaceae; Kluyvera; Kluyvera georgiana | Kluyvera georgiana | Kluyvera | Enterobacteriaceae | Enterobacterales | Gammaproteobacteria | Proteobacteria | Bacteria |
| ASV5133 | 402 | 1 | 1 | 402 | 100 | NR_118730.1 [Clostridium] symbiosum strain ATCC 14940 16S ribosomal RNA, partial sequence | NR_118730.1 | 1478 | 372 | 773 | 27.2 | 710.2 | 0 | 394/402 | 98 | 0/402 | 0 | Plus | Plus | Bacteria; Firmicutes; Clostridia; Eubacteriales; Lachnospiraceae; Lachnoclostridium; [Clostridium] symbiosum | [Clostridium] symbiosum | Lachnoclostridium | Lachnospiraceae | Eubacteriales | Clostridia | Firmicutes | Bacteria |
| ASV5134 | 428 | 1 | 1 | 428 | 100 | NR_026497.1 Desulfonispora thiosulfatigenes strain GKNTAUT 16S ribosomal RNA, partial sequence | NR_026497.1 | 1507 | 347 | 774 | 28.4 | 497.9 | 2E-140 | 378/431 | 87 | 6/431 | 1 | Plus | Plus | Bacteria; Firmicutes; Clostridia; Eubacteriales; Peptococcaceae; Desulfonispora; Desulfonispora thiosulfatigenes | Desulfonispora thiosulfatigenes | Desulfonispora | Peptococcaceae | Eubacteriales | Clostridia | Firmicutes | Bacteria |
| ASV5135 | 401 | 2 | 1 | 401 | 100 | NR_148837.1 Abyssivirga alkaniphila strain L81 16S ribosomal RNA, partial sequence | NR_148837.1 | 1520 | 364 | 765 | 26.45 | 520 | 3E-147 | 362/402 | 90 | 1/402 | 0 | Plus | Plus | Bacteria; Firmicutes; Clostridia; Eubacteriales; Lachnospiraceae; Abyssivirga; Abyssivirga alkaniphila | Abyssivirga alkaniphila | Abyssivirga | Lachnospiraceae | Eubacteriales | Clostridia | Firmicutes | Bacteria |
| ASV5137 | 402 | 1 | 1 | 402 | 100 | NR_029355.1 [Clostridium] methylpentosum strain R2 16S ribosomal RNA, partial sequence | NR_029355.1 | 1435 | 349 | 750 | 28.01 | 488.6 | 1E-137 | 358/404 | 88 | 4/404 | 0 | Plus | Plus | Bacteria; Firmicutes; Clostridia; Eubacteriales; Oscillospiraceae; ; [Clostridium] methylpentosum | [Clostridium] methylpentosum | | Oscillospiraceae | Eubacteriales | Clostridia | Firmicutes | Bacteria |
| ASV5138 | 405 | 1 | 1 | 405 | 100 | NR_118554.1 Intestinimonas butyriciproducens strain SRB-521-5-I 16S ribosomal RNA, partial sequence | NR_118554.1 | 1475 | 346 | 750 | 27.46 | 505.3 | 1E-142 | 362/406 | 89 | 2/406 | 0 | Plus | Plus | Bacteria; Firmicutes; Clostridia; Eubacteriales; ; Intestinimonas; Intestinimonas butyriciproducens | Intestinimonas butyriciproducens | Intestinimonas | | Eubacteriales | Clostridia | Firmicutes | Bacteria |
| ASV5140 | 402 | 1 | 1 | 402 | 100 | NR_044616.1 Synergistes jonesii strain 78-1 16S ribosomal RNA, partial sequence | NR_044616.1 | 1355 | 345 | 749 | 29.89 | 573.6 | 3E-163 | 374/406 | 92 | 5/406 | 1 | Plus | Plus | Bacteria; Synergistetes; Synergistia; Synergistales; Synergistaceae; Synergistes; Synergistes jonesii | Synergistes jonesii | Synergistes | Synergistaceae | Synergistales | Synergistia | Synergistetes | Bacteria |
| ASV5142 | 401 | 4 | 1 | 401 | 100 | NR_074399.1 Ruminococcus albus 7 = DSM 20455 16S ribosomal RNA, partial sequence | NR_074399.1 | 1500 | 350 | 751 | 26.8 | 508.9 | 7E-144 | 360/402 | 89 | 1/402 | 0 | Plus | Plus | Bacteria; Firmicutes; Clostridia; Eubacteriales; Oscillospiraceae; Ruminococcus; Ruminococcus albus | Ruminococcus albus | Ruminococcus | Oscillospiraceae | Eubacteriales | Clostridia | Firmicutes | Bacteria |
| ASV5145 | 427 | 5 | 1 | 427 | 100 | NR_042052.1 Streptococcus equinus ATCC 9812 16S ribosomal RNA, partial sequence | NR_042052.1 | 1433 | 260 | 686 | 29.8 | 451.7 | 1E-126 | 372/433 | 85 | 12/433 | 2 | Plus | Plus | Bacteria; Firmicutes; Bacilli; Lactobacillales; Streptococcaceae; Streptococcus; Streptococcus equinus | Streptococcus equinus | Streptococcus | Streptococcaceae | Lactobacillales | Bacilli | Firmicutes | Bacteria |
| ASV5146 | 404 | 3 | 1 | 404 | 100 | NR_041781.1 Streptococcus alactolyticus strain ATCC 43077 16S ribosomal RNA, partial sequence | NR_041781.1 | 1437 | 346 | 772 | 29.71 | 490.5 | 3E-138 | 377/427 | 88 | 23/427 | 5 | Plus | Plus | Bacteria; Firmicutes; Bacilli; Lactobacillales; Streptococcaceae; Streptococcus; Streptococcus alactolyticus | Streptococcus alactolyticus | Streptococcus | Streptococcaceae | Lactobacillales | Bacilli | Firmicutes | Bacteria |
| ASV5147 | 427 | 1 | 1 | 426 | 99.77 | NR_044111.1 Selenomonas bovis strain WG 16S ribosomal RNA, partial sequence | NR_044111.1 | 1525 | 370 | 795 | 27.93 | 438.8 | 1E-122 | 364/427 | 85 | 2/427 | 0 | Plus | Plus | Bacteria; Firmicutes; Negativicutes; Selenomonadales; Selenomonadaceae; Selenomonas; Selenomonas bovis | Selenomonas bovis | Selenomonas | Selenomonadaceae | Selenomonadales | Negativicutes | Firmicutes | Bacteria |
| ASV5148 | 423 | 1 | 1 | 423 | 100 | NR_144750.1 Prevotellamassilia timonensis strain Marseille-P2831 16S ribosomal RNA, partial sequence | NR_144750.1 | 1493 | 358 | 779 | 28.27 | 547.7 | 2E-155 | 383/425 | 90 | 5/425 | 1 | Plus | Plus | Bacteria; Bacteroidetes; Bacteroidia; Bacteroidales; Prevotellaceae; Prevotellamassilia; Prevotellamassilia timonensis | Prevotellamassilia timonensis | Prevotellamassilia | Prevotellaceae | Bacteroidales | Bacteroidia | Bacteroidetes | Bacteria |
| ASV5150 | 402 | 1 | 1 | 402 | 100 | NR_029160.1 Ruminococcus callidus strain ATCC 27760 16S ribosomal RNA, partial sequence | NR_029160.1 | 1371 | 310 | 711 | 29.32 | 494.2 | 2E-139 | 359/404 | 88 | 4/404 | 0 | Plus | Plus | Bacteria; Firmicutes; Clostridia; Eubacteriales; Oscillospiraceae; Ruminococcus; Ruminococcus callidus | Ruminococcus callidus | Ruminococcus | Oscillospiraceae | Eubacteriales | Clostridia | Firmicutes | Bacteria |
| ASV5152 | 428 | 1 | 1 | 427 | 99.77 | NR_028839.1 Veillonella montpellierensis strain ADV 281.99 16S ribosomal RNA, partial sequence | NR_028839.1 | 1409 | 306 | 731 | 30.23 | 499.7 | 5E-141 | 377/429 | 87 | 5/429 | 1 | Plus | Plus | Bacteria; Firmicutes; Negativicutes; Veillonellales; Veillonellaceae; Veillonella; Veillonella montpellierensis | Veillonella montpellierensis | Veillonella | Veillonellaceae | Veillonellales | Negativicutes | Firmicutes | Bacteria |
| ASV5153 | 404 | 3 | 1 | 404 | 100 | NR_102980.1 Megasphaera elsdenii DSM 20460 16S ribosomal RNA, partial sequence | NR_102980.1 | 1552 | 369 | 795 | 27.51 | 590.2 | 3E-168 | 395/427 | 92 | 23/427 | 5 | Plus | Plus | Bacteria; Firmicutes; Negativicutes; Veillonellales; Veillonellaceae; Megasphaera; Megasphaera elsdenii | Megasphaera elsdenii | Megasphaera | Veillonellaceae | Veillonellales | Negativicutes | Firmicutes | Bacteria |
| ASV5154 | 401 | 1 | 1 | 401 | 100 | NR_104758.1 Lachnospira multipara ATCC 19207 strain D32 16S ribosomal RNA, partial sequence | NR_104758.1 | 1518 | 362 | 763 | 26.48 | 508.9 | 7E-144 | 360/402 | 89 | 1/402 | 0 | Plus | Plus | Bacteria; Firmicutes; Clostridia; Eubacteriales; Lachnospiraceae; Lachnospira; Lachnospira multipara | Lachnospira multipara | Lachnospira | Lachnospiraceae | Eubacteriales | Clostridia | Firmicutes | Bacteria |
| ASV5155 | 422 | 1 | 1 | 422 | 100 | NR_144616.1 Muribaculum intestinale strain YL27 16S ribosomal RNA, partial sequence | NR_144616.1 | 1460 | 297 | 718 | 28.9 | 453.5 | 4E-127 | 369/428 | 86 | 12/428 | 2 | Plus | Plus | Bacteria; Bacteroidetes; Bacteroidia; Bacteroidales; Muribaculaceae; Muribaculum; Muribaculum intestinale | Muribaculum intestinale | Muribaculum | Muribaculaceae | Bacteroidales | Bacteroidia | Bacteroidetes | Bacteria |
| ASV5160 | 404 | 1 | 1 | 402 | 99.5 | NR_112900.1 Christensenella minuta strain YIT 12065 16S ribosomal RNA, partial sequence | NR_112900.1 | 1497 | 358 | 760 | 26.92 | 416.6 | 5E-116 | 347/406 | 85 | 7/406 | 1 | Plus | Plus | Bacteria; Firmicutes; Clostridia; Eubacteriales; Christensenellaceae; Christensenella; Christensenella minuta | Christensenella minuta | Christensenella | Christensenellaceae | Eubacteriales | Clostridia | Firmicutes | Bacteria |
| ASV5161 | 404 | 1 | 1 | 404 | 100 | NR_159227.1 Monoglobus pectinilyticus strain 14 16S ribosomal RNA, partial sequence | NR_159227.1 | 1493 | 368 | 769 | 26.93 | 523.7 | 3E-148 | 368/408 | 90 | 10/408 | 2 | Plus | Plus | Bacteria; Firmicutes; Clostridia; Eubacteriales; Oscillospiraceae; Monoglobus; Monoglobus pectinilyticus | Monoglobus pectinilyticus | Monoglobus | Oscillospiraceae | Eubacteriales | Clostridia | Firmicutes | Bacteria |
| ASV5165 | 424 | 1 | 1 | 424 | 100 | NR_118334.1 Alloprevotella rava strain 81/4-12 16S ribosomal RNA, partial sequence | NR_118334.1 | 1455 | 338 | 759 | 29 | 588.4 | 1E-167 | 391/426 | 91 | 6/426 | 1 | Plus | Plus | Bacteria; Bacteroidetes; Bacteroidia; Bacteroidales; Prevotellaceae; Alloprevotella; Alloprevotella rava | Alloprevotella rava | Alloprevotella | Prevotellaceae | Bacteroidales | Bacteroidia | Bacteroidetes | Bacteria |
| ASV5168 | 405 | 1 | 1 | 405 | 100 | NR_144742.1 Christensenella massiliensis strain Marseille-P2438 16S ribosomal RNA, partial sequence | NR_144742.1 | 1499 | 369 | 773 | 27.02 | 499.7 | 5E-141 | 360/405 | 88 | 0/405 | 0 | Plus | Plus | Bacteria; Firmicutes; Clostridia; Eubacteriales; Christensenellaceae; Christensenella; Christensenella massiliensis | Christensenella massiliensis | Christensenella | Christensenellaceae | Eubacteriales | Clostridia | Firmicutes | Bacteria |
| ASV5169 | 360 | 1 | 52 | 360 | 85.83 | NR_117112.1 Cytophaga xylanolytica strain DSM 6779 16S ribosomal RNA, partial sequence | NR_117112.1 | 1507 | 456 | 764 | 20.5 | 344.6 | 2E-94 | 269/310 | 86 | 2/310 | 0 | Plus | Plus | Bacteria; Bacteroidetes; Bacteroidia; Marinilabiliales; Marinilabiliaceae; Breznakibacter; Breznakibacter xylanolyticus | Breznakibacter xylanolyticus | Breznakibacter | Marinilabiliaceae | Marinilabiliales | Bacteroidia | Bacteroidetes | Bacteria |
| ASV5172 | 405 | 1 | 4 | 405 | 99.26 | NR_144742.1 Christensenella massiliensis strain Marseille-P2438 16S ribosomal RNA, partial sequence | NR_144742.1 | 1499 | 372 | 773 | 26.82 | 422.2 | 1E-117 | 345/403 | 85 | 2/403 | 0 | Plus | Plus | Bacteria; Firmicutes; Clostridia; Eubacteriales; Christensenellaceae; Christensenella; Christensenella massiliensis | Christensenella massiliensis | Christensenella | Christensenellaceae | Eubacteriales | Clostridia | Firmicutes | Bacteria |
| ASV5174 | 423 | 1 | 1 | 423 | 100 | NR_041364.1 Prevotella stercorea DSM 18206 strain CB35 16S ribosomal RNA, partial sequence | NR_041364.1 | 1488 | 354 | 775 | 28.36 | 558.8 | 8E-159 | 384/424 | 90 | 3/424 | 0 | Plus | Plus | Bacteria; Bacteroidetes; Bacteroidia; Bacteroidales; Prevotellaceae; Prevotella; Prevotella stercorea | Prevotella stercorea | Prevotella | Prevotellaceae | Bacteroidales | Bacteroidia | Bacteroidetes | Bacteria |
| ASV5175 | 402 | 1 | 1 | 402 | 100 | NR_026347.1 Clostridium isatidis strain WV6 16S ribosomal RNA gene, partial sequence | NR_026347.1 | 1332 | 317 | 718 | 30.18 | 422.2 | 1E-117 | 347/405 | 85 | 6/405 | 1 | Plus | Plus | Bacteria; Firmicutes; Clostridia; Eubacteriales; Clostridiaceae; Clostridium; Clostridium isatidis | Clostridium isatidis | Clostridium | Clostridiaceae | Eubacteriales | Clostridia | Firmicutes | Bacteria |
| ASV5176 | 402 | 2 | 1 | 402 | 100 | NR_102987.1 Hungateiclostridium clariflavum DSM 19732 16S ribosomal RNA, partial sequence | NR_102987.1 | 1634 | 481 | 882 | 24.6 | 533 | 4E-151 | 365/403 | 90 | 2/403 | 0 | Plus | Plus | Bacteria; Firmicutes; Clostridia; Eubacteriales; Oscillospiraceae; Acetivibrio; Acetivibrio clariflavus | Acetivibrio clariflavus | Acetivibrio | Oscillospiraceae | Eubacteriales | Clostridia | Firmicutes | Bacteria |
| ASV5178 | 404 | 1 | 1 | 404 | 100 | NR_026330.1 Eubacterium callanderi strain DSM 3662 16S ribosomal RNA, partial sequence | NR_026330.1 | 1474 | 342 | 745 | 27.41 | 736.1 | 0 | 402/404 | 99 | 0/404 | 0 | Plus | Plus | Bacteria; Firmicutes; Clostridia; Eubacteriales; Eubacteriaceae; Eubacterium; Eubacterium callanderi | Eubacterium callanderi | Eubacterium | Eubacteriaceae | Eubacteriales | Clostridia | Firmicutes | Bacteria |
| ASV5182 | 422 | 1 | 1 | 413 | 97.87 | NR_133025.1 Alistipes obesi strain ph8 16S ribosomal RNA, partial sequence | NR_133025.1 | 1388 | 292 | 704 | 29.76 | 431.4 | 2E-120 | 358/418 | 85 | 10/418 | 2 | Plus | Plus | Bacteria; Bacteroidetes; Bacteroidia; Bacteroidales; Rikenellaceae; Alistipes; Alistipes communis | Alistipes communis | Alistipes | Rikenellaceae | Bacteroidales | Bacteroidia | Bacteroidetes | Bacteria |
| ASV5184 | 404 | 1 | 1 | 404 | 100 | NR_024836.1 Helicobacter ganmani strain CMRI H02 16S ribosomal RNA, partial sequence | NR_024836.1 | 1418 | 313 | 714 | 28.35 | 562.5 | 6E-160 | 371/404 | 91 | 2/404 | 0 | Plus | Plus | Bacteria; Proteobacteria; Epsilonproteobacteria; Campylobacterales; Helicobacteraceae; Helicobacter; Helicobacter ganmani | Helicobacter ganmani | Helicobacter | Helicobacteraceae | Campylobacterales | Epsilonproteobacteria | Proteobacteria | Bacteria |
| ASV5185 | 402 | 1 | 3 | 402 | 99.5 | NR_026476.1 Succinivibrio dextrinosolvens strain 0554 16S ribosomal RNA, partial sequence | NR_026476.1 | 1463 | 333 | 732 | 27.34 | 540.3 | 3E-153 | 364/400 | 91 | 0/400 | 0 | Plus | Plus | Bacteria; Proteobacteria; Gammaproteobacteria; Aeromonadales; Succinivibrionaceae; Succinivibrio; Succinivibrio dextrinosolvens | Succinivibrio dextrinosolvens | Succinivibrio | Succinivibrionaceae | Aeromonadales | Gammaproteobacteria | Proteobacteria | Bacteria |
| ASV5187 | 427 | 5 | 1 | 426 | 99.77 | NR_119109.1 Pseudescherichia vulneris strain ATCC 33821 16S ribosomal RNA, partial sequence | NR_119109.1 | 1443 | 344 | 769 | 29.52 | 605 | 1E-172 | 394/427 | 92 | 2/427 | 0 | Plus | Plus | Bacteria; Proteobacteria; Gammaproteobacteria; Enterobacterales; Enterobacteriaceae; Pseudescherichia; Pseudescherichia vulneris | Pseudescherichia vulneris | Pseudescherichia | Enterobacteriaceae | Enterobacterales | Gammaproteobacteria | Proteobacteria | Bacteria |
| ASV5190 | 401 | 1 | 1 | 401 | 100 | NR_044734.1 Selenomonas ruminantium strain GA192 16S ribosomal RNA, partial sequence | NR_044734.1 | 1486 | 375 | 801 | 28.73 | 483.1 | 4E-136 | 376/427 | 88 | 26/427 | 6 | Plus | Plus | Bacteria; Firmicutes; Negativicutes; Selenomonadales; Selenomonadaceae; Selenomonas; Selenomonas ruminantium | Selenomonas ruminantium | Selenomonas | Selenomonadaceae | Selenomonadales | Negativicutes | Firmicutes | Bacteria |
| ASV5191 | 402 | 1 | 1 | 402 | 100 | NR_044425.1 Hydrogenoanaerobacterium saccharovorans strain SW512 16S ribosomal RNA, partial sequence | NR_044425.1 | 1510 | 363 | 764 | 26.62 | 455.4 | 1E-127 | 354/406 | 87 | 8/406 | 1 | Plus | Plus | Bacteria; Firmicutes; Clostridia; Eubacteriales; Oscillospiraceae; Hydrogenoanaerobacterium; Hydrogenoanaerobacterium saccharovorans | Hydrogenoanaerobacterium saccharovorans | Hydrogenoanaerobacterium | Oscillospiraceae | Eubacteriales | Clostridia | Firmicutes | Bacteria |
| ASV5192 | 422 | 1 | 1 | 422 | 100 | NR_042842.1 Prevotella denticola strain ATCC 35308 16S ribosomal RNA, partial sequence | NR_042842.1 | 1506 | 338 | 759 | 28.02 | 610.5 | 2E-174 | 391/422 | 92 | 0/422 | 0 | Plus | Plus | Bacteria; Bacteroidetes; Bacteroidia; Bacteroidales; Prevotellaceae; Prevotella; Prevotella denticola | Prevotella denticola | Prevotella | Prevotellaceae | Bacteroidales | Bacteroidia | Bacteroidetes | Bacteria |
| ASV5193 | 422 | 3 | 1 | 422 | 100 | NR_113411.1 Prevotella copri DSM 18205 strain JCM 13464 16S ribosomal RNA, partial sequence | NR_113411.1 | 1493 | 358 | 779 | 28.27 | 536.6 | 4E-152 | 379/423 | 89 | 2/423 | 0 | Plus | Plus | Bacteria; Bacteroidetes; Bacteroidia; Bacteroidales; Prevotellaceae; Prevotella; Prevotella copri | Prevotella copri | Prevotella | Prevotellaceae | Bacteroidales | Bacteroidia | Bacteroidetes | Bacteria |
| ASV5195 | 427 | 1 | 1 | 420 | 98.36 | NR_102980.1 Megasphaera elsdenii DSM 20460 16S ribosomal RNA, partial sequence | NR_102980.1 | 1552 | 369 | 788 | 27.06 | 444.3 | 2E-124 | 364/424 | 85 | 8/424 | 1 | Plus | Plus | Bacteria; Firmicutes; Negativicutes; Veillonellales; Veillonellaceae; Megasphaera; Megasphaera elsdenii | Megasphaera elsdenii | Megasphaera | Veillonellaceae | Veillonellales | Negativicutes | Firmicutes | Bacteria |
| ASV5197 | 403 | 1 | 1 | 403 | 100 | NR_147371.1 Intestinibacillus massiliensis strain Marseille-P3216 16S ribosomal RNA, partial sequence | NR_147371.1 | 1424 | 294 | 696 | 28.3 | 518.2 | 1E-146 | 364/405 | 89 | 4/405 | 0 | Plus | Plus | Bacteria; Firmicutes; Clostridia; Eubacteriales; Eubacteriaceae; Intestinibacillus; Intestinibacillus massiliensis | Intestinibacillus massiliensis | Intestinibacillus | Eubacteriaceae | Eubacteriales | Clostridia | Firmicutes | Bacteria |
| ASV5202 | 402 | 1 | 1 | 402 | 100 | NR_104846.1 Gemmiger formicilis strain X2-56 16S ribosomal RNA, partial sequence | NR_104846.1 | 1426 | 305 | 706 | 28.19 | 527.4 | 2E-149 | 363/402 | 90 | 0/402 | 0 | Plus | Plus | Bacteria; Firmicutes; Clostridia; Eubacteriales; ; Gemmiger; Gemmiger formicilis | Gemmiger formicilis | Gemmiger | | Eubacteriales | Clostridia | Firmicutes | Bacteria |
| ASV5203 | 404 | 2 | 3 | 404 | 99.5 | NR_144605.1 Acutalibacter muris strain KB18 16S ribosomal RNA, partial sequence | NR_144605.1 | 1510 | 387 | 785 | 26.42 | 440.6 | 3E-123 | 349/403 | 86 | 5/403 | 1 | Plus | Plus | Bacteria; Firmicutes; Clostridia; Eubacteriales; Oscillospiraceae; Acutalibacter; Acutalibacter muris | Acutalibacter muris | Acutalibacter | Oscillospiraceae | Eubacteriales | Clostridia | Firmicutes | Bacteria |
| ASV5205 | 422 | 1 | 1 | 422 | 100 | NR_041364.1 Prevotella stercorea DSM 18206 strain CB35 16S ribosomal RNA, partial sequence | NR_041364.1 | 1488 | 354 | 775 | 28.36 | 553.3 | 4E-157 | 381/422 | 90 | 0/422 | 0 | Plus | Plus | Bacteria; Bacteroidetes; Bacteroidia; Bacteroidales; Prevotellaceae; Prevotella; Prevotella stercorea | Prevotella stercorea | Prevotella | Prevotellaceae | Bacteroidales | Bacteroidia | Bacteroidetes | Bacteria |
| ASV5206 | 422 | 2 | 1 | 422 | 100 | NR_113119.1 Prevotella oris strain JCM 8540 16S ribosomal RNA, partial sequence | NR_113119.1 | 1481 | 345 | 766 | 28.49 | 531.1 | 2E-150 | 381/426 | 89 | 8/426 | 1 | Plus | Plus | Bacteria; Bacteroidetes; Bacteroidia; Bacteroidales; Prevotellaceae; Prevotella; Prevotella oris | Prevotella oris | Prevotella | Prevotellaceae | Bacteroidales | Bacteroidia | Bacteroidetes | Bacteria |
| ASV5207 | 422 | 1 | 1 | 420 | 99.53 | NR_125593.1 Dielma fastidiosa strain JC13 16S ribosomal RNA, partial sequence | NR_125593.1 | 1499 | 355 | 778 | 28.29 | 470.2 | 4E-132 | 371/427 | 86 | 10/427 | 2 | Plus | Plus | Bacteria; Firmicutes; Erysipelotrichia; Erysipelotrichales; Erysipelotrichaceae; Dielma; Dielma fastidiosa | Dielma fastidiosa | Dielma | Erysipelotrichaceae | Erysipelotrichales | Erysipelotrichia | Firmicutes | Bacteria |
| ASV5209 | 405 | 1 | 1 | 405 | 100 | NR_044054.1 Blautia wexlerae DSM 19850 16S ribosomal RNA, partial sequence | NR_044054.1 | 1438 | 314 | 715 | 27.96 | 601.3 | 1E-171 | 380/406 | 93 | 5/406 | 1 | Plus | Plus | Bacteria; Firmicutes; Clostridia; Eubacteriales; Lachnospiraceae; Blautia; Blautia wexlerae | Blautia wexlerae | Blautia | Lachnospiraceae | Eubacteriales | Clostridia | Firmicutes | Bacteria |
| ASV5214 | 427 | 1 | 1 | 427 | 100 | NR_026032.1 [Pasteurella] aerogenes strain P-172-71 16S ribosomal RNA, partial sequence | NR_026032.1 | 1405 | 353 | 779 | 30.39 | 557 | 3E-158 | 387/429 | 90 | 4/429 | 0 | Plus | Plus | Bacteria; Proteobacteria; Gammaproteobacteria; Pasteurellales; Pasteurellaceae; ; [Pasteurella] aerogenes | [Pasteurella] aerogenes | | Pasteurellaceae | Pasteurellales | Gammaproteobacteria | Proteobacteria | Bacteria |
| ASV5218 | 422 | 1 | 1 | 422 | 100 | NR_113411.1 Prevotella copri DSM 18205 strain JCM 13464 16S ribosomal RNA, partial sequence | NR_113411.1 | 1493 | 358 | 779 | 28.27 | 619.7 | 3E-177 | 394/423 | 93 | 2/423 | 0 | Plus | Plus | Bacteria; Bacteroidetes; Bacteroidia; Bacteroidales; Prevotellaceae; Prevotella; Prevotella copri | Prevotella copri | Prevotella | Prevotellaceae | Bacteroidales | Bacteroidia | Bacteroidetes | Bacteria |
| ASV5220 | 405 | 1 | 1 | 405 | 100 | NR_044490.1 Butyricicoccus pullicaecorum strain 25-3 16S ribosomal RNA, partial sequence | NR_044490.1 | 1482 | 366 | 770 | 27.33 | 533 | 4E-151 | 369/408 | 90 | 6/408 | 1 | Plus | Plus | Bacteria; Firmicutes; Clostridia; Eubacteriales; Clostridiaceae; Butyricicoccus; Butyricicoccus pullicaecorum | Butyricicoccus pullicaecorum | Butyricicoccus | Clostridiaceae | Eubacteriales | Clostridia | Firmicutes | Bacteria |
| ASV5221 | 403 | 1 | 1 | 403 | 100 | NR_042832.1 Roseburia faecis strain M72/1 16S ribosomal RNA, partial sequence | NR_042832.1 | 1456 | 347 | 749 | 27.68 | 584.7 | 1E-166 | 375/404 | 92 | 2/404 | 0 | Plus | Plus | Bacteria; Firmicutes; Clostridia; Eubacteriales; Lachnospiraceae; Roseburia; Roseburia faecis | Roseburia faecis | Roseburia | Lachnospiraceae | Eubacteriales | Clostridia | Firmicutes | Bacteria |
| ASV5222 | 402 | 4 | 1 | 402 | 100 | NR_042802.1 Lactobacillus ultunensis strain Kx146C1 16S ribosomal RNA, partial sequence | NR_042802.1 | 1550 | 370 | 796 | 27.55 | 521.9 | 9E-148 | 385/429 | 89 | 29/429 | 6 | Plus | Plus | Bacteria; Firmicutes; Bacilli; Lactobacillales; Lactobacillaceae; Lactobacillus; Lactobacillus ultunensis | Lactobacillus ultunensis | Lactobacillus | Lactobacillaceae | Lactobacillales | Bacilli | Firmicutes | Bacteria |
| ASV5224 | 403 | 1 | 1 | 394 | 97.77 | NR_104907.1 Eubacterium coprostanoligenes strain HL 16S ribosomal RNA, partial sequence | NR_104907.1 | 1446 | 332 | 725 | 27.25 | 462.8 | 6E-130 | 347/395 | 87 | 2/395 | 0 | Plus | Plus | Bacteria; Firmicutes; Clostridia; Eubacteriales; Eubacteriaceae; Eubacterium; Eubacterium coprostanoligenes | Eubacterium coprostanoligenes | Eubacterium | Eubacteriaceae | Eubacteriales | Clostridia | Firmicutes | Bacteria |
| ASV5227 | 403 | 2 | 1 | 403 | 100 | NR_152060.1 Butyricicoccus faecihominis strain KS-2 16S ribosomal RNA, partial sequence | NR_152060.1 | 1418 | 353 | 757 | 28.56 | 536.6 | 4E-152 | 367/405 | 90 | 2/405 | 0 | Plus | Plus | Bacteria; Firmicutes; Clostridia; Eubacteriales; Clostridiaceae; Butyricicoccus; Butyricicoccus faecihominis | Butyricicoccus faecihominis | Butyricicoccus | Clostridiaceae | Eubacteriales | Clostridia | Firmicutes | Bacteria |
| ASV5233 | 402 | 5 | 1 | 402 | 100 | NR_114789.1 [Clostridium] leptum strain DSM 753 16S ribosomal RNA, partial sequence | NR_114789.1 | 1505 | 364 | 765 | 27.16 | 516.3 | 4E-146 | 361/402 | 89 | 0/402 | 0 | Plus | Plus | Bacteria; Firmicutes; Clostridia; Eubacteriales; Oscillospiraceae; ; [Clostridium] leptum | [Clostridium] leptum | | Oscillospiraceae | Eubacteriales | Clostridia | Firmicutes | Bacteria |
| ASV5234 | 401 | 1 | 1 | 401 | 100 | NR_158111.1 Paludicola psychrotolerans strain NC1253 16S ribosomal RNA, partial sequence | NR_158111.1 | 1490 | 366 | 766 | 26.91 | 558.8 | 7E-159 | 369/402 | 91 | 2/402 | 0 | Plus | Plus | Bacteria; Firmicutes; Clostridia; Eubacteriales; Oscillospiraceae; Paludicola; Paludicola psychrotolerans | Paludicola psychrotolerans | Paludicola | Oscillospiraceae | Eubacteriales | Clostridia | Firmicutes | Bacteria |
| ASV5239 | 405 | 1 | 1 | 405 | 100 | NR_115340.1 Saccharofermentans acetigenes strain P6 16S ribosomal RNA, partial sequence | NR_115340.1 | 1510 | 353 | 757 | 26.82 | 538.5 | 9E-153 | 370/408 | 90 | 6/408 | 1 | Plus | Plus | Bacteria; Firmicutes; Clostridia; Eubacteriales; Oscillospiraceae; Saccharofermentans; Saccharofermentans acetigenes | Saccharofermentans acetigenes | Saccharofermentans | Oscillospiraceae | Eubacteriales | Clostridia | Firmicutes | Bacteria |
| ASV5241 | 405 | 2 | 1 | 405 | 100 | NR_104899.1 Bacteroides xylanolyticus strain X5-1 16S ribosomal RNA, partial sequence | NR_104899.1 | 1480 | 345 | 746 | 27.16 | 573.6 | 3E-163 | 374/405 | 92 | 3/405 | 0 | Plus | Plus | Bacteria; Firmicutes; Clostridia; Eubacteriales; Clostridiaceae; Hungatella; Hungatella xylanolytica | Hungatella xylanolytica | Hungatella | Clostridiaceae | Eubacteriales | Clostridia | Firmicutes | Bacteria |
| ASV5244 | 401 | 1 | 1 | 398 | 99.25 | NR_119087.1 Peptoclostridium litorale strain DSM 5388 16S ribosomal RNA, partial sequence | NR_119087.1 | 1501 | 365 | 766 | 26.52 | 409.2 | 8E-114 | 343/402 | 85 | 8/402 | 1 | Plus | Plus | Bacteria; Firmicutes; Clostridia; Eubacteriales; Peptostreptococcaceae; Peptoclostridium; Peptoclostridium litorale | Peptoclostridium litorale | Peptoclostridium | Peptostreptococcaceae | Eubacteriales | Clostridia | Firmicutes | Bacteria |
| ASV5245 | 402 | 2 | 1 | 402 | 100 | NR_113231.1 Blautia glucerasea strain JCM 17039 16S ribosomal RNA, partial sequence | NR_113231.1 | 1491 | 364 | 765 | 26.96 | 610.5 | 2E-174 | 378/402 | 94 | 0/402 | 0 | Plus | Plus | Bacteria; Firmicutes; Clostridia; Eubacteriales; Lachnospiraceae; Blautia; Blautia glucerasea | Blautia glucerasea | Blautia | Lachnospiraceae | Eubacteriales | Clostridia | Firmicutes | Bacteria |
| ASV5247 | 403 | 1 | 3 | 403 | 99.5 | NR_137408.1 Terrisporobacter petrolearius strain LAM0A37 16S ribosomal RNA, partial sequence | NR_137408.1 | 1405 | 309 | 707 | 28.4 | 617.9 | 1E-176 | 381/403 | 94 | 6/403 | 1 | Plus | Plus | Bacteria; Firmicutes; Clostridia; Eubacteriales; Peptostreptococcaceae; Terrisporobacter; Terrisporobacter petrolearius | Terrisporobacter petrolearius | Terrisporobacter | Peptostreptococcaceae | Eubacteriales | Clostridia | Firmicutes | Bacteria |
| ASV5248 | 402 | 1 | 1 | 402 | 100 | NR_044624.1 [Clostridium] cellulosi strain AS 1.1777 16S ribosomal RNA, partial sequence | NR_044624.1 | 1500 | 344 | 748 | 27 | 529.3 | 6E-150 | 368/407 | 90 | 7/407 | 1 | Plus | Plus | Bacteria; Firmicutes; Clostridia; Eubacteriales; Oscillospiraceae; ; [Clostridium] cellulosi | [Clostridium] cellulosi | | Oscillospiraceae | Eubacteriales | Clostridia | Firmicutes | Bacteria |
| ASV5249 | 404 | 1 | 1 | 387 | 95.79 | NR_026315.1 Pseudobutyrivibrio ruminis strain DSM 9787 16S ribosomal RNA, partial sequence | NR_026315.1 | 1462 | 334 | 718 | 26.33 | 564.3 | 2E-160 | 360/387 | 93 | 2/387 | 0 | Plus | Plus | Bacteria; Firmicutes; Clostridia; Eubacteriales; Lachnospiraceae; Pseudobutyrivibrio; Pseudobutyrivibrio ruminis | Pseudobutyrivibrio ruminis | Pseudobutyrivibrio | Lachnospiraceae | Eubacteriales | Clostridia | Firmicutes | Bacteria |
| ASV5251 | 422 | 1 | 1 | 422 | 100 | NR_040877.1 Prevotella copri DSM 18205 strain CB7 16S ribosomal RNA, partial sequence | NR_040877.1 | 1494 | 358 | 779 | 28.25 | 521.9 | 1E-147 | 378/425 | 88 | 6/425 | 1 | Plus | Plus | Bacteria; Bacteroidetes; Bacteroidia; Bacteroidales; Prevotellaceae; Prevotella; Prevotella copri | Prevotella copri | Prevotella | Prevotellaceae | Bacteroidales | Bacteroidia | Bacteroidetes | Bacteria |
| ASV5254 | 422 | 1 | 1 | 422 | 100 | NR_113411.1 Prevotella copri DSM 18205 strain JCM 13464 16S ribosomal RNA, partial sequence | NR_113411.1 | 1493 | 358 | 779 | 28.27 | 608.7 | 7E-174 | 392/423 | 92 | 2/423 | 0 | Plus | Plus | Bacteria; Bacteroidetes; Bacteroidia; Bacteroidales; Prevotellaceae; Prevotella; Prevotella copri | Prevotella copri | Prevotella | Prevotellaceae | Bacteroidales | Bacteroidia | Bacteroidetes | Bacteria |
| ASV5257 | 426 | 1 | 1 | 424 | 99.53 | NR_042797.1 Treponema berlinense strain 7CPL208 16S ribosomal RNA, partial sequence | NR_042797.1 | 1440 | 275 | 699 | 29.51 | 479.4 | 6E-135 | 372/427 | 87 | 5/427 | 1 | Plus | Plus | Bacteria; Spirochaetes; Spirochaetia; Spirochaetales; Spirochaetaceae; Treponema; Treponema berlinense | Treponema berlinense | Treponema | Spirochaetaceae | Spirochaetales | Spirochaetia | Spirochaetes | Bacteria |
| ASV5260 | 402 | 5 | 5 | 402 | 99 | NR_148837.1 Abyssivirga alkaniphila strain L81 16S ribosomal RNA, partial sequence | NR_148837.1 | 1520 | 368 | 765 | 26.18 | 508.9 | 7E-144 | 359/400 | 89 | 4/400 | 1 | Plus | Plus | Bacteria; Firmicutes; Clostridia; Eubacteriales; Lachnospiraceae; Abyssivirga; Abyssivirga alkaniphila | Abyssivirga alkaniphila | Abyssivirga | Lachnospiraceae | Eubacteriales | Clostridia | Firmicutes | Bacteria |
| ASV5261 | 406 | 5 | 1 | 406 | 100 | NR_074414.1 Olsenella uli DSM 7084 16S ribosomal RNA, partial sequence | NR_074414.1 | 1503 | 348 | 755 | 27.15 | 592 | 7E-169 | 379/408 | 92 | 2/408 | 0 | Plus | Plus | Bacteria; Actinobacteria; Coriobacteriia; Coriobacteriales; Atopobiaceae; Olsenella; Olsenella uli | Olsenella uli | Olsenella | Atopobiaceae | Coriobacteriales | Coriobacteriia | Actinobacteria | Bacteria |
| ASV5262 | 404 | 1 | 1 | 404 | 100 | NR_041691.1 Butyricimonas virosa strain MT12 16S ribosomal RNA, partial sequence | NR_041691.1 | 1486 | 352 | 772 | 28.33 | 529.3 | 6E-150 | 380/422 | 90 | 19/422 | 4 | Plus | Plus | Bacteria; Bacteroidetes; Bacteroidia; Bacteroidales; Odoribacteraceae; Butyricimonas; Butyricimonas virosa | Butyricimonas virosa | Butyricimonas | Odoribacteraceae | Bacteroidales | Bacteroidia | Bacteroidetes | Bacteria |
| ASV5263 | 404 | 2 | 1 | 404 | 100 | NR_024661.1 Eubacterium ruminantium strain GA195 16S ribosomal RNA, partial sequence | NR_024661.1 | 1508 | 378 | 779 | 26.66 | 496 | 6E-140 | 359/404 | 88 | 2/404 | 0 | Plus | Plus | Bacteria; Firmicutes; Clostridia; Eubacteriales; Eubacteriaceae; Eubacterium; Eubacterium ruminantium | Eubacterium ruminantium | Eubacterium | Eubacteriaceae | Eubacteriales | Clostridia | Firmicutes | Bacteria |
| ASV5265 | 422 | 2 | 1 | 412 | 97.63 | NR_113119.1 Prevotella oris strain JCM 8540 16S ribosomal RNA, partial sequence | NR_113119.1 | 1481 | 345 | 757 | 27.89 | 518.2 | 1E-146 | 371/415 | 89 | 5/415 | 1 | Plus | Plus | Bacteria; Bacteroidetes; Bacteroidia; Bacteroidales; Prevotellaceae; Prevotella; Prevotella oris | Prevotella oris | Prevotella | Prevotellaceae | Bacteroidales | Bacteroidia | Bacteroidetes | Bacteria |
| ASV5266 | 402 | 1 | 1 | 402 | 100 | NR_025206.1 Hespellia porcina strain PC80 16S ribosomal RNA, partial sequence | NR_025206.1 | 1449 | 331 | 732 | 27.74 | 422.2 | 1E-117 | 348/406 | 85 | 8/406 | 1 | Plus | Plus | Bacteria; Firmicutes; Clostridia; Eubacteriales; Lachnospiraceae; Hespellia; Hespellia porcina | Hespellia porcina | Hespellia | Lachnospiraceae | Eubacteriales | Clostridia | Firmicutes | Bacteria |
| ASV5267 | 405 | 2 | 1 | 405 | 100 | NR_113070.1 Bacteroides oleiciplenus YIT 12058 strain JCM 16102 16S ribosomal RNA, partial sequence | NR_113070.1 | 1485 | 351 | 772 | 28.42 | 414.8 | 2E-115 | 361/424 | 85 | 21/424 | 4 | Plus | Plus | Bacteria; Bacteroidetes; Bacteroidia; Bacteroidales; Bacteroidaceae; Bacteroides; Bacteroides oleiciplenus | Bacteroides oleiciplenus | Bacteroides | Bacteroidaceae | Bacteroidales | Bacteroidia | Bacteroidetes | Bacteria |
| ASV5268 | 402 | 1 | 1 | 402 | 100 | NR_042832.1 Roseburia faecis strain M72/1 16S ribosomal RNA, partial sequence | NR_042832.1 | 1456 | 347 | 749 | 27.68 | 671.5 | 0 | 390/403 | 96 | 1/403 | 0 | Plus | Plus | Bacteria; Firmicutes; Clostridia; Eubacteriales; Lachnospiraceae; Roseburia; Roseburia faecis | Roseburia faecis | Roseburia | Lachnospiraceae | Eubacteriales | Clostridia | Firmicutes | Bacteria |
| ASV5272 | 422 | 2 | 1 | 422 | 100 | NR_041364.1 Prevotella stercorea DSM 18206 strain CB35 16S ribosomal RNA, partial sequence | NR_041364.1 | 1488 | 354 | 775 | 28.36 | 486.8 | 4E-137 | 369/422 | 87 | 0/422 | 0 | Plus | Plus | Bacteria; Bacteroidetes; Bacteroidia; Bacteroidales; Prevotellaceae; Prevotella; Prevotella stercorea | Prevotella stercorea | Prevotella | Prevotellaceae | Bacteroidales | Bacteroidia | Bacteroidetes | Bacteria |
| ASV5273 | 402 | 1 | 1 | 402 | 100 | NR_044386.1 Clostridium amylolyticum strain SW408 16S ribosomal RNA, partial sequence | NR_044386.1 | 1505 | 348 | 749 | 26.71 | 427.7 | 2E-119 | 349/406 | 85 | 8/406 | 1 | Plus | Plus | Bacteria; Firmicutes; Clostridia; Eubacteriales; Clostridiaceae; Clostridium; Clostridium amylolyticum | Clostridium amylolyticum | Clostridium | Clostridiaceae | Eubacteriales | Clostridia | Firmicutes | Bacteria |
| ASV5274 | 404 | 1 | 1 | 404 | 100 | NR_102884.1 Ruminococcus champanellensis 18P13 = JCM 17042 16S ribosomal RNA, partial sequence | NR_102884.1 | 1494 | 348 | 749 | 26.91 | 545.9 | 6E-155 | 368/404 | 91 | 2/404 | 0 | Plus | Plus | Bacteria; Firmicutes; Clostridia; Eubacteriales; Oscillospiraceae; Ruminococcus; Ruminococcus champanellensis | Ruminococcus champanellensis | Ruminococcus | Oscillospiraceae | Eubacteriales | Clostridia | Firmicutes | Bacteria |
| ASV5277 | 402 | 1 | 1 | 402 | 100 | NR_114326.1 Fusicatenibacter saccharivorans strain HT03-11 16S ribosomal RNA, partial sequence | NR_114326.1 | 1423 | 351 | 752 | 28.25 | 632.7 | 0 | 382/402 | 95 | 0/402 | 0 | Plus | Plus | Bacteria; Firmicutes; Clostridia; Eubacteriales; Lachnospiraceae; Fusicatenibacter; Fusicatenibacter saccharivorans | Fusicatenibacter saccharivorans | Fusicatenibacter | Lachnospiraceae | Eubacteriales | Clostridia | Firmicutes | Bacteria |
| ASV5279 | 421 | 1 | 15 | 421 | 96.67 | NR_113122.1 Prevotella paludivivens strain JCM 13650 16S ribosomal RNA, partial sequence | NR_113122.1 | 1491 | 370 | 777 | 27.36 | 481.2 | 2E-135 | 359/408 | 87 | 1/408 | 0 | Plus | Plus | Bacteria; Bacteroidetes; Bacteroidia; Bacteroidales; Prevotellaceae; Prevotella; Prevotella paludivivens | Prevotella paludivivens | Prevotella | Prevotellaceae | Bacteroidales | Bacteroidia | Bacteroidetes | Bacteria |
| ASV5281 | 403 | 1 | 3 | 403 | 99.5 | NR_115345.1 Hungateiclostridium alkalicellulosi strain Z-7026 16S ribosomal RNA, partial sequence | NR_115345.1 | 1489 | 357 | 758 | 27 | 508.9 | 7E-144 | 360/402 | 89 | 1/402 | 0 | Plus | Plus | Bacteria; Firmicutes; Clostridia; Eubacteriales; Oscillospiraceae; Acetivibrio; Acetivibrio alkalicellulosi | Acetivibrio alkalicellulosi | Acetivibrio | Oscillospiraceae | Eubacteriales | Clostridia | Firmicutes | Bacteria |
| ASV5285 | 423 | 2 | 1 | 423 | 100 | NR_113119.1 Prevotella oris strain JCM 8540 16S ribosomal RNA, partial sequence | NR_113119.1 | 1481 | 345 | 766 | 28.49 | 531.1 | 2E-150 | 381/426 | 89 | 7/426 | 1 | Plus | Plus | Bacteria; Bacteroidetes; Bacteroidia; Bacteroidales; Prevotellaceae; Prevotella; Prevotella oris | Prevotella oris | Prevotella | Prevotellaceae | Bacteroidales | Bacteroidia | Bacteroidetes | Bacteria |
| ASV5290 | 405 | 1 | 1 | 405 | 100 | NR_144611.1 Flintibacter butyricus strain BLS21 16S ribosomal RNA, partial sequence | NR_144611.1 | 808 | 299 | 703 | 50.12 | 533 | 4E-151 | 366/405 | 90 | 0/405 | 0 | Plus | Plus | Bacteria; Firmicutes; Clostridia; Eubacteriales; ; Flintibacter; Flintibacter butyricus | Flintibacter butyricus | Flintibacter | | Eubacteriales | Clostridia | Firmicutes | Bacteria |
| ASV5295 | 405 | 1 | 1 | 405 | 100 | NR_112900.1 Christensenella minuta strain YIT 12065 16S ribosomal RNA, partial sequence | NR_112900.1 | 1497 | 358 | 762 | 27.05 | 483.1 | 4E-136 | 359/407 | 88 | 4/407 | 0 | Plus | Plus | Bacteria; Firmicutes; Clostridia; Eubacteriales; Christensenellaceae; Christensenella; Christensenella minuta | Christensenella minuta | Christensenella | Christensenellaceae | Eubacteriales | Clostridia | Firmicutes | Bacteria |
| ASV5298 | 404 | 2 | 1 | 389 | 96.29 | NR_118554.1 Intestinimonas butyriciproducens strain SRB-521-5-I 16S ribosomal RNA, partial sequence | NR_118554.1 | 1475 | 346 | 735 | 26.44 | 481.2 | 2E-135 | 348/391 | 89 | 3/391 | 0 | Plus | Plus | Bacteria; Firmicutes; Clostridia; Eubacteriales; ; Intestinimonas; Intestinimonas butyriciproducens | Intestinimonas butyriciproducens | Intestinimonas | | Eubacteriales | Clostridia | Firmicutes | Bacteria |
| ASV5299 | 403 | 2 | 1 | 403 | 100 | NR_152059.1 Harryflintia acetispora strain V20-281a 16S ribosomal RNA, partial sequence | NR_152059.1 | 1363 | 289 | 692 | 29.64 | 457.2 | 3E-128 | 355/407 | 87 | 7/407 | 1 | Plus | Plus | Bacteria; Firmicutes; Clostridia; Eubacteriales; Oscillospiraceae; Harryflintia; Harryflintia acetispora | Harryflintia acetispora | Harryflintia | Oscillospiraceae | Eubacteriales | Clostridia | Firmicutes | Bacteria |
| ASV5301 | 427 | 5 | 3 | 427 | 99.53 | NR_025448.1 Lactobacillus hamsteri strain DSM 5661 16S ribosomal RNA, partial sequence | NR_025448.1 | 1510 | 356 | 780 | 28.15 | 470.2 | 4E-132 | 370/427 | 86 | 4/427 | 0 | Plus | Plus | Bacteria; Firmicutes; Bacilli; Lactobacillales; Lactobacillaceae; Lactobacillus; Lactobacillus hamsteri | Lactobacillus hamsteri | Lactobacillus | Lactobacillaceae | Lactobacillales | Bacilli | Firmicutes | Bacteria |
| ASV5302 | 402 | 1 | 1 | 402 | 100 | NR_028961.1 Faecalibacterium prausnitzii strain ATCC 27768 16S ribosomal RNA, partial sequence | NR_028961.1 | 1462 | 344 | 745 | 27.5 | 671.5 | 0 | 390/403 | 96 | 2/403 | 0 | Plus | Plus | Bacteria; Firmicutes; Clostridia; Eubacteriales; Oscillospiraceae; Faecalibacterium; Faecalibacterium prausnitzii | Faecalibacterium prausnitzii | Faecalibacterium | Oscillospiraceae | Eubacteriales | Clostridia | Firmicutes | Bacteria |
| ASV5303 | 422 | 3 | 1 | 421 | 99.76 | NR_113110.1 Prevotella maculosa strain JCM 15638 16S ribosomal RNA, partial sequence | NR_113110.1 | 1405 | 269 | 689 | 29.96 | 545.9 | 6E-155 | 380/422 | 90 | 2/422 | 0 | Plus | Plus | Bacteria; Bacteroidetes; Bacteroidia; Bacteroidales; Prevotellaceae; Prevotella; Prevotella maculosa | Prevotella maculosa | Prevotella | Prevotellaceae | Bacteroidales | Bacteroidia | Bacteroidetes | Bacteria |
| ASV5305 | 426 | 2 | 1 | 426 | 100 | NR_042260.1 Sphaerochaeta coccoides strain SPN1 16S ribosomal RNA, partial sequence | NR_042260.1 | 1500 | 320 | 746 | 28.47 | 505.3 | 1E-142 | 378/429 | 88 | 5/429 | 1 | Plus | Plus | Bacteria; Spirochaetes; Spirochaetia; Spirochaetales; Spirochaetaceae; Sphaerochaeta; Sphaerochaeta coccoides | Sphaerochaeta coccoides | Sphaerochaeta | Spirochaetaceae | Spirochaetales | Spirochaetia | Spirochaetes | Bacteria |
| ASV5307 | 421 | 1 | 1 | 421 | 100 | NR_113411.1 Prevotella copri DSM 18205 strain JCM 13464 16S ribosomal RNA, partial sequence | NR_113411.1 | 1493 | 358 | 779 | 28.27 | 545.9 | 6E-155 | 380/422 | 90 | 1/422 | 0 | Plus | Plus | Bacteria; Bacteroidetes; Bacteroidia; Bacteroidales; Prevotellaceae; Prevotella; Prevotella copri | Prevotella copri | Prevotella | Prevotellaceae | Bacteroidales | Bacteroidia | Bacteroidetes | Bacteria |
| ASV5313 | 427 | 1 | 1 | 427 | 100 | NR_102980.1 Megasphaera elsdenii DSM 20460 16S ribosomal RNA, partial sequence | NR_102980.1 | 1552 | 369 | 795 | 27.51 | 573.6 | 3E-163 | 389/428 | 90 | 2/428 | 0 | Plus | Plus | Bacteria; Firmicutes; Negativicutes; Veillonellales; Veillonellaceae; Megasphaera; Megasphaera elsdenii | Megasphaera elsdenii | Megasphaera | Veillonellaceae | Veillonellales | Negativicutes | Firmicutes | Bacteria |
| ASV5314 | 402 | 1 | 1 | 395 | 98.26 | NR_118692.1 Blautia obeum ATCC 29174 16S ribosomal RNA, partial sequence | NR_118692.1 | 1398 | 330 | 724 | 28.25 | 588.4 | 9E-168 | 370/396 | 93 | 2/396 | 0 | Plus | Plus | Bacteria; Firmicutes; Clostridia; Eubacteriales; Lachnospiraceae; Blautia; Blautia obeum | Blautia obeum | Blautia | Lachnospiraceae | Eubacteriales | Clostridia | Firmicutes | Bacteria |
| ASV5315 | 402 | 1 | 1 | 401 | 99.75 | NR_041236.1 Lutispora thermophila DSM 19022 strain EBR46 16S ribosomal RNA, partial sequence | NR_041236.1 | 1438 | 338 | 739 | 27.96 | 481.2 | 2E-135 | 356/403 | 88 | 3/403 | 0 | Plus | Plus | Bacteria; Firmicutes; Clostridia; Eubacteriales; Clostridiaceae; Lutispora; Lutispora thermophila | Lutispora thermophila | Lutispora | Clostridiaceae | Eubacteriales | Clostridia | Firmicutes | Bacteria |
| ASV5318 | 422 | 1 | 1 | 422 | 100 | NR_113411.1 Prevotella copri DSM 18205 strain JCM 13464 16S ribosomal RNA, partial sequence | NR_113411.1 | 1493 | 358 | 779 | 28.27 | 614.2 | 2E-175 | 396/426 | 92 | 8/426 | 1 | Plus | Plus | Bacteria; Bacteroidetes; Bacteroidia; Bacteroidales; Prevotellaceae; Prevotella; Prevotella copri | Prevotella copri | Prevotella | Prevotellaceae | Bacteroidales | Bacteroidia | Bacteroidetes | Bacteria |
| ASV5319 | 404 | 1 | 4 | 403 | 99.26 | NR_159296.1 Aminipila butyrica strain FH042 16S ribosomal RNA, partial sequence | NR_159296.1 | 1460 | 354 | 755 | 27.53 | 508.9 | 7E-144 | 360/402 | 89 | 2/402 | 0 | Plus | Plus | Bacteria; Firmicutes; Clostridia; Eubacteriales; Clostridiales Family XIII. Incertae Sedis; Aminipila; Aminipila butyrica | Aminipila butyrica | Aminipila | Clostridiales Family XIII. Incertae Sedis | Eubacteriales | Clostridia | Firmicutes | Bacteria |
| ASV5320 | 402 | 1 | 1 | 402 | 100 | NR_144727.1 Anaeromassilibacillus senegalensis strain mt9 16S ribosomal RNA, partial sequence | NR_144727.1 | 1478 | 352 | 753 | 27.2 | 494.2 | 2E-139 | 357/402 | 88 | 0/402 | 0 | Plus | Plus | Bacteria; Firmicutes; Clostridia; Eubacteriales; Oscillospiraceae; Anaeromassilibacillus; Anaeromassilibacillus senegalensis | Anaeromassilibacillus senegalensis | Anaeromassilibacillus | Oscillospiraceae | Eubacteriales | Clostridia | Firmicutes | Bacteria |
| ASV5322 | 405 | 1 | 1 | 405 | 100 | NR_112900.1 Christensenella minuta strain YIT 12065 16S ribosomal RNA, partial sequence | NR_112900.1 | 1497 | 358 | 762 | 27.05 | 593.9 | 2E-169 | 377/405 | 93 | 0/405 | 0 | Plus | Plus | Bacteria; Firmicutes; Clostridia; Eubacteriales; Christensenellaceae; Christensenella; Christensenella minuta | Christensenella minuta | Christensenella | Christensenellaceae | Eubacteriales | Clostridia | Firmicutes | Bacteria |
| ASV5324 | 401 | 1 | 1 | 400 | 99.75 | NR_026103.1 [Clostridium] populeti strain 743A 16S ribosomal RNA, partial sequence | NR_026103.1 | 1489 | 333 | 733 | 26.93 | 457.2 | 3E-128 | 352/403 | 87 | 5/403 | 1 | Plus | Plus | Bacteria; Firmicutes; Clostridia; Eubacteriales; Lachnospiraceae; Lachnoclostridium; [Clostridium] populeti | [Clostridium] populeti | Lachnoclostridium | Lachnospiraceae | Eubacteriales | Clostridia | Firmicutes | Bacteria |
| ASV5327 | 402 | 1 | 3 | 402 | 99.5 | NR_024661.1 Eubacterium ruminantium strain GA195 16S ribosomal RNA, partial sequence | NR_024661.1 | 1508 | 380 | 779 | 26.53 | 534.8 | 1E-151 | 364/401 | 90 | 2/401 | 0 | Plus | Plus | Bacteria; Firmicutes; Clostridia; Eubacteriales; Eubacteriaceae; Eubacterium; Eubacterium ruminantium | Eubacterium ruminantium | Eubacterium | Eubacteriaceae | Eubacteriales | Clostridia | Firmicutes | Bacteria |
| ASV5330 | 423 | 1 | 1 | 423 | 100 | NR_041954.1 Prevotella brevis strain GA33 16S ribosomal RNA, partial sequence | NR_041954.1 | 1493 | 356 | 777 | 28.27 | 451.7 | 1E-126 | 367/427 | 85 | 9/427 | 2 | Plus | Plus | Bacteria; Bacteroidetes; Bacteroidia; Bacteroidales; Prevotellaceae; Prevotella; Prevotella brevis | Prevotella brevis | Prevotella | Prevotellaceae | Bacteroidales | Bacteroidia | Bacteroidetes | Bacteria |
| ASV5332 | 404 | 1 | 1 | 404 | 100 | NR_044054.1 Blautia wexlerae DSM 19850 16S ribosomal RNA, partial sequence | NR_044054.1 | 1438 | 314 | 715 | 27.96 | 534.8 | 1E-151 | 366/404 | 90 | 2/404 | 0 | Plus | Plus | Bacteria; Firmicutes; Clostridia; Eubacteriales; Lachnospiraceae; Blautia; Blautia wexlerae | Blautia wexlerae | Blautia | Lachnospiraceae | Eubacteriales | Clostridia | Firmicutes | Bacteria |
| ASV5333 | 402 | 1 | 1 | 402 | 100 | NR_125677.1 Vallitalea pronyensis strain FatNI3 16S ribosomal RNA, partial sequence | NR_125677.1 | 1480 | 363 | 764 | 27.16 | 488.6 | 1E-137 | 360/406 | 88 | 8/406 | 1 | Plus | Plus | Bacteria; Firmicutes; Clostridia; Eubacteriales; Vallitaleaceae; Vallitalea; Vallitalea pronyensis | Vallitalea pronyensis | Vallitalea | Vallitaleaceae | Eubacteriales | Clostridia | Firmicutes | Bacteria |
| ASV5338 | 402 | 1 | 1 | 402 | 100 | NR_029239.3 Clostridium chartatabidum strain 163 16S ribosomal RNA, partial sequence | NR_029239.3 | 1460 | 323 | 724 | 27.53 | 605 | 9E-173 | 379/404 | 93 | 4/404 | 0 | Plus | Plus | Bacteria; Firmicutes; Clostridia; Eubacteriales; Clostridiaceae; Clostridium; Clostridium chartatabidum | Clostridium chartatabidum | Clostridium | Clostridiaceae | Eubacteriales | Clostridia | Firmicutes | Bacteria |
| ASV5339 | 427 | 2 | 15 | 427 | 96.72 | NR_125516.1 Spiroplasma cantharicola strain CC-1 16S ribosomal RNA, partial sequence | NR_125516.1 | 1505 | 347 | 758 | 27.38 | 418.5 | 1E-116 | 354/416 | 85 | 7/416 | 1 | Plus | Plus | Bacteria; Tenericutes; Mollicutes; Entomoplasmatales; Spiroplasmataceae; Spiroplasma; Spiroplasma cantharicola | Spiroplasma cantharicola | Spiroplasma | Spiroplasmataceae | Entomoplasmatales | Mollicutes | Tenericutes | Bacteria |
| ASV5341 | 426 | 1 | 1 | 374 | 87.79 | NR_044731.2 Holdemanella biformis strain DSM 3989 16S ribosomal RNA, partial sequence | NR_044731.2 | 1487 | 364 | 737 | 25.15 | 665.9 | 0 | 369/374 | 98 | 0/374 | 0 | Plus | Plus | Bacteria; Firmicutes; Erysipelotrichia; Erysipelotrichales; Erysipelotrichaceae; Holdemanella; Holdemanella biformis | Holdemanella biformis | Holdemanella | Erysipelotrichaceae | Erysipelotrichales | Erysipelotrichia | Firmicutes | Bacteria |
| ASV5342 | 401 | 1 | 1 | 401 | 100 | NR_134694.1 Aminicella lysinilytica strain WN037 16S ribosomal RNA, partial sequence | NR_134694.1 | 1465 | 353 | 756 | 27.58 | 477.6 | 2E-134 | 356/404 | 88 | 3/404 | 0 | Plus | Plus | Bacteria; Firmicutes; Clostridia; Eubacteriales; Eubacteriaceae; Aminicella; Aminicella lysinilytica | Aminicella lysinilytica | Aminicella | Eubacteriaceae | Eubacteriales | Clostridia | Firmicutes | Bacteria |
| ASV5343 | 426 | 1 | 3 | 426 | 99.53 | NR_025697.1 Spiroplasma alleghenense strain PLHS-1 16S ribosomal RNA, partial sequence | NR_025697.1 | 1516 | 347 | 770 | 27.97 | 436.9 | 4E-122 | 362/425 | 85 | 2/425 | 0 | Plus | Plus | Bacteria; Tenericutes; Mollicutes; Entomoplasmatales; Spiroplasmataceae; Spiroplasma; Spiroplasma alleghenense | Spiroplasma alleghenense | Spiroplasma | Spiroplasmataceae | Entomoplasmatales | Mollicutes | Tenericutes | Bacteria |
| ASV5347 | 421 | 2 | 1 | 421 | 100 | NR_074535.1 Odoribacter splanchnicus strain DSM 220712 16S ribosomal RNA, partial sequence | NR_074535.1 | 1512 | 351 | 771 | 27.84 | 601.3 | 1E-171 | 393/425 | 92 | 8/425 | 1 | Plus | Plus | Bacteria; Bacteroidetes; Bacteroidia; Bacteroidales; Odoribacteraceae; Odoribacter; Odoribacter splanchnicus | Odoribacter splanchnicus | Odoribacter | Odoribacteraceae | Bacteroidales | Bacteroidia | Bacteroidetes | Bacteria |
| ASV5348 | 405 | 1 | 1 | 403 | 99.51 | NR_026104.1 Ruminiclostridium cellobioparum DSM 1351 = ATCC 15832 16S ribosomal RNA, partial sequence | NR_026104.1 | 1487 | 329 | 731 | 27.1 | 451.7 | 1E-126 | 350/403 | 86 | 0/403 | 0 | Plus | Plus | Bacteria; Firmicutes; Clostridia; Eubacteriales; Oscillospiraceae; Ruminiclostridium; Ruminiclostridium cellobioparum | Ruminiclostridium cellobioparum | Ruminiclostridium | Oscillospiraceae | Eubacteriales | Clostridia | Firmicutes | Bacteria |
| ASV5350 | 406 | 1 | 3 | 406 | 99.51 | NR_159296.1 Aminipila butyrica strain FH042 16S ribosomal RNA, partial sequence | NR_159296.1 | 1460 | 354 | 755 | 27.53 | 490.5 | 3E-138 | 358/404 | 88 | 2/404 | 0 | Plus | Plus | Bacteria; Firmicutes; Clostridia; Eubacteriales; Clostridiales Family XIII. Incertae Sedis; Aminipila; Aminipila butyrica | Aminipila butyrica | Aminipila | Clostridiales Family XIII. Incertae Sedis | Eubacteriales | Clostridia | Firmicutes | Bacteria |
| ASV5351 | 402 | 1 | 1 | 400 | 99.5 | NR_102884.1 Ruminococcus champanellensis 18P13 = JCM 17042 16S ribosomal RNA, partial sequence | NR_102884.1 | 1494 | 348 | 747 | 26.77 | 468.3 | 1E-131 | 352/401 | 87 | 2/401 | 0 | Plus | Plus | Bacteria; Firmicutes; Clostridia; Eubacteriales; Oscillospiraceae; Ruminococcus; Ruminococcus champanellensis | Ruminococcus champanellensis | Ruminococcus | Oscillospiraceae | Eubacteriales | Clostridia | Firmicutes | Bacteria |
| ASV5353 | 428 | 1 | 1 | 427 | 99.77 | NR_102980.1 Megasphaera elsdenii DSM 20460 16S ribosomal RNA, partial sequence | NR_102980.1 | 1552 | 369 | 794 | 27.45 | 549.6 | 5E-156 | 386/429 | 89 | 5/429 | 1 | Plus | Plus | Bacteria; Firmicutes; Negativicutes; Veillonellales; Veillonellaceae; Megasphaera; Megasphaera elsdenii | Megasphaera elsdenii | Megasphaera | Veillonellaceae | Veillonellales | Negativicutes | Firmicutes | Bacteria |
| ASV5355 | 403 | 1 | 3 | 403 | 99.5 | NR_026315.1 Pseudobutyrivibrio ruminis strain DSM 9787 16S ribosomal RNA, partial sequence | NR_026315.1 | 1462 | 336 | 735 | 27.36 | 479.4 | 6E-135 | 357/404 | 88 | 7/404 | 1 | Plus | Plus | Bacteria; Firmicutes; Clostridia; Eubacteriales; Lachnospiraceae; Pseudobutyrivibrio; Pseudobutyrivibrio ruminis | Pseudobutyrivibrio ruminis | Pseudobutyrivibrio | Lachnospiraceae | Eubacteriales | Clostridia | Firmicutes | Bacteria |
| ASV5358 | 402 | 2 | 1 | 385 | 95.77 | NR_074809.1 Roseburia hominis A2-183 16S ribosomal RNA, partial sequence | NR_074809.1 | 1535 | 367 | 752 | 25.15 | 551.4 | 1E-156 | 358/387 | 92 | 3/387 | 0 | Plus | Plus | Bacteria; Firmicutes; Clostridia; Eubacteriales; Lachnospiraceae; Roseburia; Roseburia hominis | Roseburia hominis | Roseburia | Lachnospiraceae | Eubacteriales | Clostridia | Firmicutes | Bacteria |
| ASV5359 | 426 | 1 | 3 | 426 | 99.53 | NR_102980.1 Megasphaera elsdenii DSM 20460 16S ribosomal RNA, partial sequence | NR_102980.1 | 1552 | 371 | 795 | 27.38 | 601.3 | 1E-171 | 394/427 | 92 | 5/427 | 1 | Plus | Plus | Bacteria; Firmicutes; Negativicutes; Veillonellales; Veillonellaceae; Megasphaera; Megasphaera elsdenii | Megasphaera elsdenii | Megasphaera | Veillonellaceae | Veillonellales | Negativicutes | Firmicutes | Bacteria |
| ASV5362 | 428 | 1 | 1 | 428 | 100 | NR_104781.1 Treponema bryantii strain RUS-1 16S ribosomal RNA, partial sequence | NR_104781.1 | 1535 | 351 | 778 | 27.88 | 514.5 | 2E-145 | 379/429 | 88 | 2/429 | 0 | Plus | Plus | Bacteria; Spirochaetes; Spirochaetia; Spirochaetales; Spirochaetaceae; Treponema; Treponema bryantii | Treponema bryantii | Treponema | Spirochaetaceae | Spirochaetales | Spirochaetia | Spirochaetes | Bacteria |
| ASV5364 | 422 | 1 | 1 | 422 | 100 | NR_113411.1 Prevotella copri DSM 18205 strain JCM 13464 16S ribosomal RNA, partial sequence | NR_113411.1 | 1493 | 358 | 779 | 28.27 | 669.6 | 0 | 402/422 | 95 | 0/422 | 0 | Plus | Plus | Bacteria; Bacteroidetes; Bacteroidia; Bacteroidales; Prevotellaceae; Prevotella; Prevotella copri | Prevotella copri | Prevotella | Prevotellaceae | Bacteroidales | Bacteroidia | Bacteroidetes | Bacteria |
| ASV5366 | 404 | 1 | 1 | 402 | 99.5 | NR_112900.1 Christensenella minuta strain YIT 12065 16S ribosomal RNA, partial sequence | NR_112900.1 | 1497 | 358 | 760 | 26.92 | 444.3 | 2E-124 | 349/403 | 86 | 1/403 | 0 | Plus | Plus | Bacteria; Firmicutes; Clostridia; Eubacteriales; Christensenellaceae; Christensenella; Christensenella minuta | Christensenella minuta | Christensenella | Christensenellaceae | Eubacteriales | Clostridia | Firmicutes | Bacteria |
| ASV5367 | 401 | 1 | 3 | 401 | 99.5 | NR_144749.1 Ihubacter massiliensis strain Marseille-P2843 16S ribosomal RNA, partial sequence | NR_144749.1 | 1479 | 366 | 766 | 27.11 | 579.1 | 5E-165 | 372/401 | 92 | 2/401 | 0 | Plus | Plus | Bacteria; Firmicutes; Clostridia; Eubacteriales; Clostridiales Family XIII. Incertae Sedis; Ihubacter; Ihubacter massiliensis | Ihubacter massiliensis | Ihubacter | Clostridiales Family XIII. Incertae Sedis | Eubacteriales | Clostridia | Firmicutes | Bacteria |
| ASV5368 | 403 | 1 | 1 | 403 | 100 | NR_134005.1 Clostridium bornimense strain M2/40 16S ribosomal RNA, partial sequence | NR_134005.1 | 1463 | 284 | 685 | 29.49 | 643.8 | 0 | 385/403 | 95 | 1/403 | 0 | Plus | Plus | Bacteria; Firmicutes; Clostridia; Eubacteriales; Clostridiaceae; Clostridium; Clostridium bornimense | Clostridium bornimense | Clostridium | Clostridiaceae | Eubacteriales | Clostridia | Firmicutes | Bacteria |
| ASV5371 | 422 | 1 | 1 | 422 | 100 | NR_041342.1 Parabacteroides distasonis strain JCM 5825 16S ribosomal RNA, partial sequence | NR_041342.1 | 1488 | 353 | 774 | 28.36 | 547.7 | 2E-155 | 380/422 | 90 | 0/422 | 0 | Plus | Plus | Bacteria; Bacteroidetes; Bacteroidia; Bacteroidales; Tannerellaceae; Parabacteroides; Parabacteroides distasonis | Parabacteroides distasonis | Parabacteroides | Tannerellaceae | Bacteroidales | Bacteroidia | Bacteroidetes | Bacteria |
| ASV5372 | 402 | 1 | 1 | 402 | 100 | NR_029160.1 Ruminococcus callidus strain ATCC 27760 16S ribosomal RNA, partial sequence | NR_029160.1 | 1371 | 310 | 711 | 29.32 | 560.7 | 2E-159 | 372/405 | 91 | 6/405 | 1 | Plus | Plus | Bacteria; Firmicutes; Clostridia; Eubacteriales; Oscillospiraceae; Ruminococcus; Ruminococcus callidus | Ruminococcus callidus | Ruminococcus | Oscillospiraceae | Eubacteriales | Clostridia | Firmicutes | Bacteria |
| ASV5376 | 404 | 1 | 1 | 402 | 99.5 | NR_028683.1 Acidaminobacter hydrogenoformans strain glu 65 16S ribosomal RNA, partial sequence | NR_028683.1 | 1468 | 345 | 745 | 27.32 | 420.3 | 4E-117 | 345/403 | 85 | 3/403 | 0 | Plus | Plus | Bacteria; Firmicutes; Clostridia; Eubacteriales; Clostridiales Family XII. Incertae Sedis; Acidaminobacter; Acidaminobacter hydrogenoformans | Acidaminobacter hydrogenoformans | Acidaminobacter | Clostridiales Family XII. Incertae Sedis | Eubacteriales | Clostridia | Firmicutes | Bacteria |
| ASV5378 | 405 | 4 | 3 | 405 | 99.51 | NR_042802.1 Lactobacillus ultunensis strain Kx146C1 16S ribosomal RNA, partial sequence | NR_042802.1 | 1550 | 372 | 796 | 27.42 | 499.7 | 4E-141 | 377/425 | 88 | 22/425 | 5 | Plus | Plus | Bacteria; Firmicutes; Bacilli; Lactobacillales; Lactobacillaceae; Lactobacillus; Lactobacillus ultunensis | Lactobacillus ultunensis | Lactobacillus | Lactobacillaceae | Lactobacillales | Bacilli | Firmicutes | Bacteria |
| ASV5379 | 428 | 1 | 1 | 428 | 100 | NR_074742.1 Treponema brennaborense strain DD5/3 16S ribosomal RNA, partial sequence | NR_074742.1 | 1530 | 351 | 776 | 27.84 | 429.5 | 6E-120 | 370/435 | 85 | 16/435 | 3 | Plus | Plus | Bacteria; Spirochaetes; Spirochaetia; Spirochaetales; Spirochaetaceae; Treponema; Treponema brennaborense | Treponema brennaborense | Treponema | Spirochaetaceae | Spirochaetales | Spirochaetia | Spirochaetes | Bacteria |
| ASV5381 | 405 | 1 | 1 | 405 | 100 | NR_144611.1 Flintibacter butyricus strain BLS21 16S ribosomal RNA, partial sequence | NR_144611.1 | 808 | 299 | 703 | 50.12 | 538.5 | 9E-153 | 367/405 | 90 | 0/405 | 0 | Plus | Plus | Bacteria; Firmicutes; Clostridia; Eubacteriales; ; Flintibacter; Flintibacter butyricus | Flintibacter butyricus | Flintibacter | | Eubacteriales | Clostridia | Firmicutes | Bacteria |
| ASV5384 | 403 | 1 | 1 | 403 | 100 | NR_074809.1 Roseburia hominis A2-183 16S ribosomal RNA, partial sequence | NR_074809.1 | 1535 | 367 | 769 | 26.25 | 623.4 | 3E-178 | 382/404 | 94 | 2/404 | 0 | Plus | Plus | Bacteria; Firmicutes; Clostridia; Eubacteriales; Lachnospiraceae; Roseburia; Roseburia hominis | Roseburia hominis | Roseburia | Lachnospiraceae | Eubacteriales | Clostridia | Firmicutes | Bacteria |
| ASV5387 | 404 | 1 | 1 | 402 | 99.5 | NR_028683.1 Acidaminobacter hydrogenoformans strain glu 65 16S ribosomal RNA, partial sequence | NR_028683.1 | 1468 | 345 | 745 | 27.32 | 481.2 | 2E-135 | 356/403 | 88 | 3/403 | 0 | Plus | Plus | Bacteria; Firmicutes; Clostridia; Eubacteriales; Clostridiales Family XII. Incertae Sedis; Acidaminobacter; Acidaminobacter hydrogenoformans | Acidaminobacter hydrogenoformans | Acidaminobacter | Clostridiales Family XII. Incertae Sedis | Eubacteriales | Clostridia | Firmicutes | Bacteria |
| ASV5388 | 426 | 3 | 4 | 426 | 99.3 | NR_041781.1 Streptococcus alactolyticus strain ATCC 43077 16S ribosomal RNA, partial sequence | NR_041781.1 | 1437 | 349 | 772 | 29.51 | 605 | 1E-172 | 393/425 | 92 | 3/425 | 0 | Plus | Plus | Bacteria; Firmicutes; Bacilli; Lactobacillales; Streptococcaceae; Streptococcus; Streptococcus alactolyticus | Streptococcus alactolyticus | Streptococcus | Streptococcaceae | Lactobacillales | Bacilli | Firmicutes | Bacteria |
| ASV5389 | 403 | 1 | 1 | 403 | 100 | NR_118673.1 [Eubacterium] hallii strain ATCC 27751 16S ribosomal RNA, partial sequence | NR_118673.1 | 1479 | 371 | 771 | 27.18 | 590.2 | 3E-168 | 375/404 | 92 | 3/404 | 0 | Plus | Plus | Bacteria; Firmicutes; Clostridia; Eubacteriales; Lachnospiraceae; Anaerobutyricum; Anaerobutyricum hallii | Anaerobutyricum hallii | Anaerobutyricum | Lachnospiraceae | Eubacteriales | Clostridia | Firmicutes | Bacteria |
| ASV5390 | 402 | 1 | 3 | 402 | 99.5 | NR_026476.1 Succinivibrio dextrinosolvens strain 0554 16S ribosomal RNA, partial sequence | NR_026476.1 | 1463 | 333 | 732 | 27.34 | 523.7 | 3E-148 | 362/401 | 90 | 2/401 | 0 | Plus | Plus | Bacteria; Proteobacteria; Gammaproteobacteria; Aeromonadales; Succinivibrionaceae; Succinivibrio; Succinivibrio dextrinosolvens | Succinivibrio dextrinosolvens | Succinivibrio | Succinivibrionaceae | Aeromonadales | Gammaproteobacteria | Proteobacteria | Bacteria |
| ASV5391 | 402 | 1 | 1 | 385 | 95.77 | NR_117792.1 Stomatobaculum longum strain ACC2 16S ribosomal RNA, partial sequence | NR_117792.1 | 1377 | 267 | 651 | 27.96 | 390.8 | 3E-108 | 329/387 | 85 | 4/387 | 1 | Plus | Plus | Bacteria; Firmicutes; Clostridia; Eubacteriales; Lachnospiraceae; Stomatobaculum; Stomatobaculum longum | Stomatobaculum longum | Stomatobaculum | Lachnospiraceae | Eubacteriales | Clostridia | Firmicutes | Bacteria |
| ASV5393 | 402 | 2 | 1 | 402 | 100 | NR_156080.1 Kineothrix alysoides strain KNHs209 16S ribosomal RNA, partial sequence | NR_156080.1 | 1410 | 353 | 754 | 28.51 | 538.5 | 9E-153 | 365/402 | 90 | 0/402 | 0 | Plus | Plus | Bacteria; Firmicutes; Clostridia; Eubacteriales; Lachnospiraceae; Kineothrix; Kineothrix alysoides | Kineothrix alysoides | Kineothrix | Lachnospiraceae | Eubacteriales | Clostridia | Firmicutes | Bacteria |
| ASV5394 | 426 | 3 | 1 | 426 | 100 | NR_041781.1 Streptococcus alactolyticus strain ATCC 43077 16S ribosomal RNA, partial sequence | NR_041781.1 | 1437 | 346 | 772 | 29.71 | 533 | 4E-151 | 387/433 | 89 | 13/433 | 3 | Plus | Plus | Bacteria; Firmicutes; Bacilli; Lactobacillales; Streptococcaceae; Streptococcus; Streptococcus alactolyticus | Streptococcus alactolyticus | Streptococcus | Streptococcaceae | Lactobacillales | Bacilli | Firmicutes | Bacteria |
| ASV5395 | 402 | 1 | 1 | 402 | 100 | NR_029097.1 Oribacterium sinus strain AIP 354.02 16S ribosomal RNA, partial sequence | NR_029097.1 | 1374 | 308 | 709 | 29.26 | 433.2 | 5E-121 | 347/403 | 86 | 2/403 | 0 | Plus | Plus | Bacteria; Firmicutes; Clostridia; Eubacteriales; Lachnospiraceae; Oribacterium; Oribacterium sinus | Oribacterium sinus | Oribacterium | Lachnospiraceae | Eubacteriales | Clostridia | Firmicutes | Bacteria |
| ASV5398 | 405 | 1 | 1 | 405 | 100 | NR_159227.1 Monoglobus pectinilyticus strain 14 16S ribosomal RNA, partial sequence | NR_159227.1 | 1493 | 368 | 769 | 26.93 | 518.2 | 1E-146 | 366/407 | 89 | 7/407 | 1 | Plus | Plus | Bacteria; Firmicutes; Clostridia; Eubacteriales; Oscillospiraceae; Monoglobus; Monoglobus pectinilyticus | Monoglobus pectinilyticus | Monoglobus | Oscillospiraceae | Eubacteriales | Clostridia | Firmicutes | Bacteria |
| ASV5399 | 403 | 1 | 1 | 403 | 100 | NR_026104.1 Ruminiclostridium cellobioparum DSM 1351 = ATCC 15832 16S ribosomal RNA, partial sequence | NR_026104.1 | 1487 | 329 | 733 | 27.24 | 486.8 | 4E-137 | 358/405 | 88 | 2/405 | 0 | Plus | Plus | Bacteria; Firmicutes; Clostridia; Eubacteriales; Oscillospiraceae; Ruminiclostridium; Ruminiclostridium cellobioparum | Ruminiclostridium cellobioparum | Ruminiclostridium | Oscillospiraceae | Eubacteriales | Clostridia | Firmicutes | Bacteria |
| ASV5405 | 422 | 1 | 1 | 422 | 100 | NR_041342.1 Parabacteroides distasonis strain JCM 5825 16S ribosomal RNA, partial sequence | NR_041342.1 | 1488 | 353 | 774 | 28.36 | 497.9 | 2E-140 | 374/425 | 88 | 6/425 | 1 | Plus | Plus | Bacteria; Bacteroidetes; Bacteroidia; Bacteroidales; Tannerellaceae; Parabacteroides; Parabacteroides distasonis | Parabacteroides distasonis | Parabacteroides | Tannerellaceae | Bacteroidales | Bacteroidia | Bacteroidetes | Bacteria |
| ASV5406 | 405 | 2 | 1 | 405 | 100 | NG_041947.1 Ruminiclostridium cellulolyticum H10 16S ribosomal RNA gene, complete sequence | NG_041947.1 | 1641 | 486 | 890 | 24.68 | 510.8 | 2E-144 | 362/405 | 89 | 0/405 | 0 | Plus | Plus | Bacteria; Firmicutes; Clostridia; Eubacteriales; Oscillospiraceae; Ruminiclostridium; Ruminiclostridium cellulolyticum | Ruminiclostridium cellulolyticum | Ruminiclostridium | Oscillospiraceae | Eubacteriales | Clostridia | Firmicutes | Bacteria |
| ASV5407 | 409 | 4 | 1 | 409 | 100 | NR_118630.1 Clostridium sporogenes strain DSM 795 16S ribosomal RNA, partial sequence | NR_118630.1 | 1081 | 269 | 670 | 37.19 | 477.6 | 2E-134 | 361/410 | 88 | 9/410 | 2 | Plus | Plus | Bacteria; Firmicutes; Clostridia; Eubacteriales; Clostridiaceae; Clostridium; Clostridium sporogenes | Clostridium sporogenes | Clostridium | Clostridiaceae | Eubacteriales | Clostridia | Firmicutes | Bacteria |
| ASV5409 | 427 | 3 | 3 | 427 | 99.53 | NR_125811.1 Lactobacillus psittaci 16S ribosomal RNA, partial sequence | NR_125811.1 | 1421 | 346 | 770 | 29.91 | 569.9 | 4E-162 | 386/425 | 90 | 0/425 | 0 | Plus | Plus | Bacteria; Firmicutes; Bacilli; Lactobacillales; Lactobacillaceae; Lactobacillus; Lactobacillus psittaci | Lactobacillus psittaci | Lactobacillus | Lactobacillaceae | Lactobacillales | Bacilli | Firmicutes | Bacteria |
| ASV5410 | 405 | 2 | 1 | 405 | 100 | NR_118554.1 Intestinimonas butyriciproducens strain SRB-521-5-I 16S ribosomal RNA, partial sequence | NR_118554.1 | 1475 | 346 | 750 | 27.46 | 505.3 | 1E-142 | 361/405 | 89 | 0/405 | 0 | Plus | Plus | Bacteria; Firmicutes; Clostridia; Eubacteriales; ; Intestinimonas; Intestinimonas butyriciproducens | Intestinimonas butyriciproducens | Intestinimonas | | Eubacteriales | Clostridia | Firmicutes | Bacteria |
| ASV5411 | 402 | 5 | 1 | 402 | 100 | NR_113388.1 Clostridium beijerinckii strain JCM 1390 16S ribosomal RNA, partial sequence | NR_113388.1 | 1473 | 348 | 749 | 27.29 | 416.6 | 5E-116 | 345/404 | 85 | 4/404 | 0 | Plus | Plus | Bacteria; Firmicutes; Clostridia; Eubacteriales; Clostridiaceae; Clostridium; Clostridium beijerinckii | Clostridium beijerinckii | Clostridium | Clostridiaceae | Eubacteriales | Clostridia | Firmicutes | Bacteria |
| ASV5412 | 404 | 1 | 1 | 404 | 100 | NR_144611.1 Flintibacter butyricus strain BLS21 16S ribosomal RNA, partial sequence | NR_144611.1 | 808 | 299 | 703 | 50.12 | 592 | 7E-169 | 377/405 | 93 | 1/405 | 0 | Plus | Plus | Bacteria; Firmicutes; Clostridia; Eubacteriales; ; Flintibacter; Flintibacter butyricus | Flintibacter butyricus | Flintibacter | | Eubacteriales | Clostridia | Firmicutes | Bacteria |
| ASV5414 | 427 | 1 | 5 | 427 | 99.06 | NR_042942.1 Treponema porcinum strain 14V28 16S ribosomal RNA, partial sequence | NR_042942.1 | 1426 | 262 | 684 | 29.66 | 533 | 5E-151 | 381/426 | 89 | 6/426 | 1 | Plus | Plus | Bacteria; Spirochaetes; Spirochaetia; Spirochaetales; Spirochaetaceae; Treponema; Treponema porcinum | Treponema porcinum | Treponema | Spirochaetaceae | Spirochaetales | Spirochaetia | Spirochaetes | Bacteria |
| ASV5416 | 402 | 1 | 3 | 402 | 99.5 | NR_109625.1 Oceanirhabdus sediminicola strain NH-JN4 16S ribosomal RNA, partial sequence | NR_109625.1 | 1484 | 352 | 751 | 27.16 | 507.1 | 3E-143 | 358/400 | 89 | 0/400 | 0 | Plus | Plus | Bacteria; Firmicutes; Clostridia; Eubacteriales; Clostridiaceae; Oceanirhabdus; Oceanirhabdus sediminicola | Oceanirhabdus sediminicola | Oceanirhabdus | Clostridiaceae | Eubacteriales | Clostridia | Firmicutes | Bacteria |
| ASV5421 | 403 | 2 | 1 | 403 | 100 | NR_134138.1 Propionispira arcuata strain WK011 16S ribosomal RNA, partial sequence | NR_134138.1 | 1429 | 279 | 705 | 29.88 | 507.1 | 3E-143 | 380/427 | 88 | 24/427 | 5 | Plus | Plus | Bacteria; Firmicutes; Negativicutes; Selenomonadales; Selenomonadaceae; Propionispira; Propionispira arcuata | Propionispira arcuata | Propionispira | Selenomonadaceae | Selenomonadales | Negativicutes | Firmicutes | Bacteria |
| ASV5423 | 407 | 1 | 1 | 407 | 100 | NR_029239.3 Clostridium chartatabidum strain 163 16S ribosomal RNA, partial sequence | NR_029239.3 | 1460 | 323 | 724 | 27.53 | 564.3 | 2E-160 | 374/407 | 91 | 5/407 | 1 | Plus | Plus | Bacteria; Firmicutes; Clostridia; Eubacteriales; Clostridiaceae; Clostridium; Clostridium chartatabidum | Clostridium chartatabidum | Clostridium | Clostridiaceae | Eubacteriales | Clostridia | Firmicutes | Bacteria |
| ASV5424 | 427 | 2 | 1 | 425 | 99.53 | NR_037018.1 Acidaminococcus fermentans strain VR4 16S ribosomal RNA gene, partial sequence | NR_037018.1 | 1516 | 349 | 773 | 28.03 | 669.6 | 0 | 404/425 | 95 | 0/425 | 0 | Plus | Plus | Bacteria; Firmicutes; Negativicutes; Acidaminococcales; Acidaminococcaceae; Acidaminococcus; Acidaminococcus fermentans | Acidaminococcus fermentans | Acidaminococcus | Acidaminococcaceae | Acidaminococcales | Negativicutes | Firmicutes | Bacteria |
| ASV5425 | 402 | 1 | 1 | 402 | 100 | NR_028961.1 Faecalibacterium prausnitzii strain ATCC 27768 16S ribosomal RNA, partial sequence | NR_028961.1 | 1462 | 344 | 745 | 27.5 | 577.3 | 2E-164 | 372/402 | 92 | 0/402 | 0 | Plus | Plus | Bacteria; Firmicutes; Clostridia; Eubacteriales; Oscillospiraceae; Faecalibacterium; Faecalibacterium prausnitzii | Faecalibacterium prausnitzii | Faecalibacterium | Oscillospiraceae | Eubacteriales | Clostridia | Firmicutes | Bacteria |
| ASV5426 | 405 | 1 | 5 | 405 | 99.01 | NR_118554.1 Intestinimonas butyriciproducens strain SRB-521-5-I 16S ribosomal RNA, partial sequence | NR_118554.1 | 1475 | 350 | 750 | 27.19 | 486.8 | 3E-137 | 355/401 | 88 | 0/401 | 0 | Plus | Plus | Bacteria; Firmicutes; Clostridia; Eubacteriales; ; Intestinimonas; Intestinimonas butyriciproducens | Intestinimonas butyriciproducens | Intestinimonas | | Eubacteriales | Clostridia | Firmicutes | Bacteria |
| ASV5428 | 422 | 1 | 1 | 422 | 100 | NR_113411.1 Prevotella copri DSM 18205 strain JCM 13464 16S ribosomal RNA, partial sequence | NR_113411.1 | 1493 | 358 | 779 | 28.27 | 603.1 | 3E-172 | 391/423 | 92 | 2/423 | 0 | Plus | Plus | Bacteria; Bacteroidetes; Bacteroidia; Bacteroidales; Prevotellaceae; Prevotella; Prevotella copri | Prevotella copri | Prevotella | Prevotellaceae | Bacteroidales | Bacteroidia | Bacteroidetes | Bacteria |
| ASV5429 | 403 | 1 | 3 | 403 | 99.5 | NR_025930.1 Ruminococcus bromii strain ATCC 27255 16S ribosomal RNA, partial sequence | NR_025930.1 | 1380 | 315 | 716 | 29.13 | 427.7 | 2E-119 | 345/402 | 85 | 1/402 | 0 | Plus | Plus | Bacteria; Firmicutes; Clostridia; Eubacteriales; Oscillospiraceae; Ruminococcus; Ruminococcus bromii | Ruminococcus bromii | Ruminococcus | Oscillospiraceae | Eubacteriales | Clostridia | Firmicutes | Bacteria |
| ASV5435 | 402 | 1 | 4 | 402 | 99.25 | NR_044503.1 Thermotalea metallivorans strain B2-1 16S ribosomal RNA, partial sequence | NR_044503.1 | 1627 | 470 | 868 | 24.52 | 444.3 | 2E-124 | 350/403 | 86 | 8/403 | 1 | Plus | Plus | Bacteria; Firmicutes; Clostridia; Eubacteriales; Clostridiaceae; Thermotalea; Thermotalea metallivorans | Thermotalea metallivorans | Thermotalea | Clostridiaceae | Eubacteriales | Clostridia | Firmicutes | Bacteria |
| ASV5436 | 405 | 2 | 3 | 405 | 99.51 | NR_147396.1 Lachnoclostridium pacaense strain Marseille-P3100 16S ribosomal RNA, partial sequence | NR_147396.1 | 1453 | 347 | 746 | 27.53 | 503.4 | 3E-142 | 360/403 | 89 | 3/403 | 0 | Plus | Plus | Bacteria; Firmicutes; Clostridia; Eubacteriales; Lachnospiraceae; Lachnoclostridium; Lachnoclostridium pacaense | Lachnoclostridium pacaense | Lachnoclostridium | Lachnospiraceae | Eubacteriales | Clostridia | Firmicutes | Bacteria |
| ASV5438 | 402 | 1 | 14 | 402 | 96.77 | NR_159227.1 Monoglobus pectinilyticus strain 14 16S ribosomal RNA, partial sequence | NR_159227.1 | 1493 | 381 | 769 | 26.05 | 514.5 | 2E-145 | 354/391 | 90 | 4/391 | 1 | Plus | Plus | Bacteria; Firmicutes; Clostridia; Eubacteriales; Oscillospiraceae; Monoglobus; Monoglobus pectinilyticus | Monoglobus pectinilyticus | Monoglobus | Oscillospiraceae | Eubacteriales | Clostridia | Firmicutes | Bacteria |
| ASV5440 | 405 | 3 | 3 | 405 | 99.51 | NR_144617.1 Muricomes intestini strain 2PG-424-CC-1 16S ribosomal RNA, partial sequence | NR_144617.1 | 1431 | 359 | 758 | 27.95 | 542.2 | 7E-154 | 369/405 | 91 | 7/405 | 1 | Plus | Plus | Bacteria; Firmicutes; Clostridia; Eubacteriales; Lachnospiraceae; Muricomes; Muricomes intestini | Muricomes intestini | Muricomes | Lachnospiraceae | Eubacteriales | Clostridia | Firmicutes | Bacteria |
| ASV5442 | 411 | 1 | 1 | 411 | 100 | NR_113411.1 Prevotella copri DSM 18205 strain JCM 13464 16S ribosomal RNA, partial sequence | NR_113411.1 | 1493 | 358 | 779 | 28.27 | 459.1 | 8E-129 | 366/422 | 86 | 11/422 | 2 | Plus | Plus | Bacteria; Bacteroidetes; Bacteroidia; Bacteroidales; Prevotellaceae; Prevotella; Prevotella copri | Prevotella copri | Prevotella | Prevotellaceae | Bacteroidales | Bacteroidia | Bacteroidetes | Bacteria |
| ASV5445 | 404 | 1 | 1 | 404 | 100 | NR_028961.1 Faecalibacterium prausnitzii strain ATCC 27768 16S ribosomal RNA, partial sequence | NR_028961.1 | 1462 | 344 | 745 | 27.5 | 568 | 1E-161 | 374/406 | 92 | 6/406 | 1 | Plus | Plus | Bacteria; Firmicutes; Clostridia; Eubacteriales; Oscillospiraceae; Faecalibacterium; Faecalibacterium prausnitzii | Faecalibacterium prausnitzii | Faecalibacterium | Oscillospiraceae | Eubacteriales | Clostridia | Firmicutes | Bacteria |
| ASV5446 | 404 | 2 | 3 | 404 | 99.5 | NR_074634.1 [Eubacterium] rectale ATCC 33656 16S ribosomal RNA, partial sequence | NR_074634.1 | 1521 | 369 | 768 | 26.3 | 558.8 | 7E-159 | 370/403 | 91 | 4/403 | 0 | Plus | Plus | Bacteria; Firmicutes; Clostridia; Eubacteriales; Lachnospiraceae; ; [Eubacterium] rectale | [Eubacterium] rectale | | Lachnospiraceae | Eubacteriales | Clostridia | Firmicutes | Bacteria |
| ASV5448 | 404 | 1 | 14 | 404 | 96.78 | NR_025930.1 Ruminococcus bromii strain ATCC 27255 16S ribosomal RNA, partial sequence | NR_025930.1 | 1380 | 326 | 716 | 28.33 | 562.5 | 6E-160 | 363/392 | 92 | 2/392 | 0 | Plus | Plus | Bacteria; Firmicutes; Clostridia; Eubacteriales; Oscillospiraceae; Ruminococcus; Ruminococcus bromii | Ruminococcus bromii | Ruminococcus | Oscillospiraceae | Eubacteriales | Clostridia | Firmicutes | Bacteria |
| ASV5449 | 426 | 1 | 1 | 426 | 100 | NR_044111.1 Selenomonas bovis strain WG 16S ribosomal RNA, partial sequence | NR_044111.1 | 1525 | 370 | 796 | 28 | 499.7 | 5E-141 | 383/435 | 88 | 17/435 | 3 | Plus | Plus | Bacteria; Firmicutes; Negativicutes; Selenomonadales; Selenomonadaceae; Selenomonas; Selenomonas bovis | Selenomonas bovis | Selenomonas | Selenomonadaceae | Selenomonadales | Negativicutes | Firmicutes | Bacteria |
| ASV5453 | 402 | 1 | 1 | 402 | 100 | NR_025931.1 Ruminococcus flavefaciens strain C94 16S ribosomal RNA, partial sequence | NR_025931.1 | 1388 | 325 | 726 | 28.96 | 494.2 | 2E-139 | 357/402 | 88 | 0/402 | 0 | Plus | Plus | Bacteria; Firmicutes; Clostridia; Eubacteriales; Oscillospiraceae; Ruminococcus; Ruminococcus flavefaciens | Ruminococcus flavefaciens | Ruminococcus | Oscillospiraceae | Eubacteriales | Clostridia | Firmicutes | Bacteria |
| ASV5454 | 404 | 3 | 3 | 404 | 99.5 | NR_074793.2 Oscillibacter valericigenes strain Sjm18-20 16S ribosomal RNA, complete sequence | NR_074793.2 | 1530 | 374 | 775 | 26.27 | 460.9 | 2E-129 | 352/403 | 87 | 2/403 | 0 | Plus | Plus | Bacteria; Firmicutes; Clostridia; Eubacteriales; Oscillospiraceae; Oscillibacter; Oscillibacter valericigenes | Oscillibacter valericigenes | Oscillibacter | Oscillospiraceae | Eubacteriales | Clostridia | Firmicutes | Bacteria |
| ASV5455 | 402 | 4 | 1 | 402 | 100 | NR_074399.1 Ruminococcus albus 7 = DSM 20455 16S ribosomal RNA, partial sequence | NR_074399.1 | 1500 | 350 | 751 | 26.8 | 483.1 | 4E-136 | 356/403 | 88 | 2/403 | 0 | Plus | Plus | Bacteria; Firmicutes; Clostridia; Eubacteriales; Oscillospiraceae; Ruminococcus; Ruminococcus albus | Ruminococcus albus | Ruminococcus | Oscillospiraceae | Eubacteriales | Clostridia | Firmicutes | Bacteria |
| ASV5456 | 403 | 1 | 1 | 403 | 100 | NR_144749.1 Ihubacter massiliensis strain Marseille-P2843 16S ribosomal RNA, partial sequence | NR_144749.1 | 1479 | 364 | 766 | 27.25 | 512.6 | 6E-145 | 364/406 | 89 | 6/406 | 1 | Plus | Plus | Bacteria; Firmicutes; Clostridia; Eubacteriales; Clostridiales Family XIII. Incertae Sedis; Ihubacter; Ihubacter massiliensis | Ihubacter massiliensis | Ihubacter | Clostridiales Family XIII. Incertae Sedis | Eubacteriales | Clostridia | Firmicutes | Bacteria |
| ASV5457 | 405 | 1 | 3 | 405 | 99.51 | NR_026476.1 Succinivibrio dextrinosolvens strain 0554 16S ribosomal RNA, partial sequence | NR_026476.1 | 1463 | 333 | 732 | 27.34 | 436.9 | 4E-122 | 349/404 | 86 | 5/404 | 1 | Plus | Plus | Bacteria; Proteobacteria; Gammaproteobacteria; Aeromonadales; Succinivibrionaceae; Succinivibrio; Succinivibrio dextrinosolvens | Succinivibrio dextrinosolvens | Succinivibrio | Succinivibrionaceae | Aeromonadales | Gammaproteobacteria | Proteobacteria | Bacteria |
| ASV5458 | 403 | 1 | 1 | 403 | 100 | NR_144749.1 Ihubacter massiliensis strain Marseille-P2843 16S ribosomal RNA, partial sequence | NR_144749.1 | 1479 | 364 | 766 | 27.25 | 484.9 | 1E-136 | 359/406 | 88 | 6/406 | 1 | Plus | Plus | Bacteria; Firmicutes; Clostridia; Eubacteriales; Clostridiales Family XIII. Incertae Sedis; Ihubacter; Ihubacter massiliensis | Ihubacter massiliensis | Ihubacter | Clostridiales Family XIII. Incertae Sedis | Eubacteriales | Clostridia | Firmicutes | Bacteria |
| ASV5462 | 422 | 2 | 3 | 422 | 99.53 | NR_074793.2 Oscillibacter valericigenes strain Sjm18-20 16S ribosomal RNA, complete sequence | NR_074793.2 | 1530 | 374 | 775 | 26.27 | 455.4 | 1E-127 | 366/421 | 86 | 20/421 | 4 | Plus | Plus | Bacteria; Firmicutes; Clostridia; Eubacteriales; Oscillospiraceae; Oscillibacter; Oscillibacter valericigenes | Oscillibacter valericigenes | Oscillibacter | Oscillospiraceae | Eubacteriales | Clostridia | Firmicutes | Bacteria |
| ASV5463 | 401 | 1 | 1 | 399 | 99.5 | NR_028961.1 Faecalibacterium prausnitzii strain ATCC 27768 16S ribosomal RNA, partial sequence | NR_028961.1 | 1462 | 344 | 743 | 27.36 | 449.9 | 4E-126 | 351/403 | 87 | 7/403 | 1 | Plus | Plus | Bacteria; Firmicutes; Clostridia; Eubacteriales; Oscillospiraceae; Faecalibacterium; Faecalibacterium prausnitzii | Faecalibacterium prausnitzii | Faecalibacterium | Oscillospiraceae | Eubacteriales | Clostridia | Firmicutes | Bacteria |
| ASV5464 | 405 | 1 | 1 | 405 | 100 | NR_170495.1 Blautia faecicola strain KGMB01111 16S ribosomal RNA, complete sequence | NR_170495.1 | 1534 | 372 | 773 | 26.21 | 601.3 | 1E-171 | 379/405 | 93 | 3/405 | 0 | Plus | Plus | Bacteria; Firmicutes; Clostridia; Eubacteriales; Lachnospiraceae; Blautia; Blautia faecicola | Blautia faecicola | Blautia | Lachnospiraceae | Eubacteriales | Clostridia | Firmicutes | Bacteria |
| ASV5465 | 402 | 1 | 3 | 402 | 99.5 | NR_156975.1 Marinisporobacter balticus strain 59.4M 16S ribosomal RNA, partial sequence | NR_156975.1 | 1435 | 336 | 735 | 27.87 | 484.9 | 1E-136 | 356/402 | 88 | 4/402 | 0 | Plus | Plus | Bacteria; Firmicutes; Clostridia; Eubacteriales; Clostridiaceae; Marinisporobacter; Marinisporobacter balticus | Marinisporobacter balticus | Marinisporobacter | Clostridiaceae | Eubacteriales | Clostridia | Firmicutes | Bacteria |
| ASV5467 | 402 | 1 | 1 | 402 | 100 | NR_027573.1 Intestinibacter bartlettii strain WAL 16138 16S ribosomal RNA, partial sequence | NR_027573.1 | 1429 | 320 | 720 | 28.06 | 564.3 | 2E-160 | 370/402 | 92 | 1/402 | 0 | Plus | Plus | Bacteria; Firmicutes; Clostridia; Eubacteriales; Peptostreptococcaceae; Intestinibacter; Intestinibacter bartlettii | Intestinibacter bartlettii | Intestinibacter | Peptostreptococcaceae | Eubacteriales | Clostridia | Firmicutes | Bacteria |
| ASV5468 | 408 | 1 | 3 | 408 | 99.51 | NR_027203.1 Mogibacterium neglectum strain P9a-h 16S ribosomal RNA, partial sequence | NR_027203.1 | 1478 | 364 | 765 | 27.2 | 503.4 | 3E-142 | 364/408 | 89 | 8/408 | 1 | Plus | Plus | Bacteria; Firmicutes; Clostridia; Eubacteriales; Clostridiales Family XIII. Incertae Sedis; Mogibacterium; Mogibacterium neglectum | Mogibacterium neglectum | Mogibacterium | Clostridiales Family XIII. Incertae Sedis | Eubacteriales | Clostridia | Firmicutes | Bacteria |
| ASV5469 | 405 | 1 | 3 | 405 | 99.51 | NR_025127.1 Lachnobacterium bovis strain LRC 5382 16S ribosomal RNA, partial sequence | NR_025127.1 | 1453 | 364 | 765 | 27.67 | 488.6 | 1E-137 | 357/403 | 88 | 1/403 | 0 | Plus | Plus | Bacteria; Firmicutes; Clostridia; Eubacteriales; Lachnospiraceae; Lachnobacterium; Lachnobacterium bovis | Lachnobacterium bovis | Lachnobacterium | Lachnospiraceae | Eubacteriales | Clostridia | Firmicutes | Bacteria |
| ASV5471 | 427 | 5 | 1 | 427 | 100 | NR_042111.1 Lactobacillus gallinarum strain ATCC 33199 16S ribosomal RNA, partial sequence | NR_042111.1 | 1546 | 362 | 788 | 27.62 | 534.8 | 1E-151 | 382/428 | 89 | 2/428 | 0 | Plus | Plus | Bacteria; Firmicutes; Bacilli; Lactobacillales; Lactobacillaceae; Lactobacillus; Lactobacillus gallinarum | Lactobacillus gallinarum | Lactobacillus | Lactobacillaceae | Lactobacillales | Bacilli | Firmicutes | Bacteria |
| ASV5473 | 401 | 1 | 3 | 399 | 99 | NR_036928.1 Hungatella hathewayi strain 1313 16S ribosomal RNA, partial sequence | NR_036928.1 | 1431 | 352 | 749 | 27.81 | 429.5 | 6E-120 | 346/401 | 86 | 7/401 | 1 | Plus | Plus | Bacteria; Firmicutes; Clostridia; Eubacteriales; Clostridiaceae; Hungatella; Hungatella hathewayi | Hungatella hathewayi | Hungatella | Clostridiaceae | Eubacteriales | Clostridia | Firmicutes | Bacteria |
| ASV5477 | 402 | 1 | 3 | 401 | 99.5 | NR_144737.1 Emergencia timonensis strain SN18 16S ribosomal RNA, partial sequence | NR_144737.1 | 1480 | 366 | 767 | 27.16 | 429.5 | 6E-120 | 348/404 | 86 | 7/404 | 1 | Plus | Plus | Bacteria; Firmicutes; Clostridia; Eubacteriales; Clostridiales Family XIII. Incertae Sedis; Emergencia; Emergencia timonensis | Emergencia timonensis | Emergencia | Clostridiales Family XIII. Incertae Sedis | Eubacteriales | Clostridia | Firmicutes | Bacteria |
| ASV5478 | 426 | 1 | 1 | 421 | 98.83 | NR_044111.1 Selenomonas bovis strain WG 16S ribosomal RNA, partial sequence | NR_044111.1 | 1525 | 370 | 791 | 27.67 | 590.2 | 3E-168 | 389/423 | 91 | 3/423 | 0 | Plus | Plus | Bacteria; Firmicutes; Negativicutes; Selenomonadales; Selenomonadaceae; Selenomonas; Selenomonas bovis | Selenomonas bovis | Selenomonas | Selenomonadaceae | Selenomonadales | Negativicutes | Firmicutes | Bacteria |
| ASV5479 | 401 | 1 | 1 | 401 | 100 | NR_144749.1 Ihubacter massiliensis strain Marseille-P2843 16S ribosomal RNA, partial sequence | NR_144749.1 | 1479 | 364 | 766 | 27.25 | 566.2 | 4E-161 | 371/403 | 92 | 2/403 | 0 | Plus | Plus | Bacteria; Firmicutes; Clostridia; Eubacteriales; Clostridiales Family XIII. Incertae Sedis; Ihubacter; Ihubacter massiliensis | Ihubacter massiliensis | Ihubacter | Clostridiales Family XIII. Incertae Sedis | Eubacteriales | Clostridia | Firmicutes | Bacteria |
| ASV5480 | 428 | 1 | 5 | 428 | 99.07 | NR_042942.1 Treponema porcinum strain 14V28 16S ribosomal RNA, partial sequence | NR_042942.1 | 1426 | 262 | 684 | 29.66 | 505.3 | 1E-142 | 380/430 | 88 | 13/430 | 3 | Plus | Plus | Bacteria; Spirochaetes; Spirochaetia; Spirochaetales; Spirochaetaceae; Treponema; Treponema porcinum | Treponema porcinum | Treponema | Spirochaetaceae | Spirochaetales | Spirochaetia | Spirochaetes | Bacteria |
| ASV5482 | 428 | 4 | 3 | 428 | 99.53 | NR_042802.1 Lactobacillus ultunensis strain Kx146C1 16S ribosomal RNA, partial sequence | NR_042802.1 | 1550 | 372 | 796 | 27.42 | 558.8 | 8E-159 | 387/428 | 90 | 5/428 | 1 | Plus | Plus | Bacteria; Firmicutes; Bacilli; Lactobacillales; Lactobacillaceae; Lactobacillus; Lactobacillus ultunensis | Lactobacillus ultunensis | Lactobacillus | Lactobacillaceae | Lactobacillales | Bacilli | Firmicutes | Bacteria |
| ASV5483 | 402 | 1 | 3 | 402 | 99.5 | NR_026074.1 Helicobacter rodentium strain MIT 95-1707 16S ribosomal RNA, partial sequence | NR_026074.1 | 1423 | 319 | 718 | 28.11 | 579.1 | 5E-165 | 375/404 | 92 | 8/404 | 1 | Plus | Plus | Bacteria; Proteobacteria; Epsilonproteobacteria; Campylobacterales; Helicobacteraceae; Helicobacter; Helicobacter rodentium | Helicobacter rodentium | Helicobacter | Helicobacteraceae | Campylobacterales | Epsilonproteobacteria | Proteobacteria | Bacteria |
| ASV5487 | 402 | 1 | 3 | 402 | 99.5 | NR_028961.1 Faecalibacterium prausnitzii strain ATCC 27768 16S ribosomal RNA, partial sequence | NR_028961.1 | 1462 | 346 | 745 | 27.36 | 529.3 | 6E-150 | 365/403 | 90 | 6/403 | 1 | Plus | Plus | Bacteria; Firmicutes; Clostridia; Eubacteriales; Oscillospiraceae; Faecalibacterium; Faecalibacterium prausnitzii | Faecalibacterium prausnitzii | Faecalibacterium | Oscillospiraceae | Eubacteriales | Clostridia | Firmicutes | Bacteria |
| ASV5491 | 402 | 3 | 3 | 402 | 99.5 | NR_171444.1 Blautia argi strain N6H1-15 16S ribosomal RNA, partial sequence | NR_171444.1 | 1409 | 320 | 719 | 28.39 | 446.2 | 6E-125 | 350/403 | 86 | 6/403 | 1 | Plus | Plus | Bacteria; Firmicutes; Clostridia; Eubacteriales; Lachnospiraceae; Blautia; Blautia argi | Blautia argi | Blautia | Lachnospiraceae | Eubacteriales | Clostridia | Firmicutes | Bacteria |
| ASV5492 | 428 | 3 | 1 | 428 | 100 | NR_041781.1 Streptococcus alactolyticus strain ATCC 43077 16S ribosomal RNA, partial sequence | NR_041781.1 | 1437 | 346 | 772 | 29.71 | 551.4 | 1E-156 | 387/430 | 90 | 5/430 | 1 | Plus | Plus | Bacteria; Firmicutes; Bacilli; Lactobacillales; Streptococcaceae; Streptococcus; Streptococcus alactolyticus | Streptococcus alactolyticus | Streptococcus | Streptococcaceae | Lactobacillales | Bacilli | Firmicutes | Bacteria |
| ASV5493 | 426 | 1 | 3 | 425 | 99.3 | NR_027526.1 Catenibacterium mitsuokai strain DSM 15897 16S ribosomal RNA, partial sequence | NR_027526.1 | 1522 | 348 | 767 | 28.35 | 558.8 | 8E-159 | 384/424 | 90 | 4/424 | 0 | Plus | Plus | Bacteria; Firmicutes; Erysipelotrichia; Erysipelotrichales; Coprobacillaceae; Catenibacterium; Catenibacterium mitsuokai | Catenibacterium mitsuokai | Catenibacterium | Coprobacillaceae | Erysipelotrichales | Erysipelotrichia | Firmicutes | Bacteria |
| ASV5496 | 402 | 1 | 3 | 402 | 99.5 | NR_108636.1 Natranaerovirga pectinivora strain AP3 16S ribosomal RNA, partial sequence | NR_108636.1 | 1497 | 367 | 766 | 26.72 | 529.3 | 6E-150 | 362/400 | 90 | 0/400 | 0 | Plus | Plus | Bacteria; Firmicutes; Clostridia; Eubacteriales; ; Natranaerovirga; Natranaerovirga pectinivora | Natranaerovirga pectinivora | Natranaerovirga | | Eubacteriales | Clostridia | Firmicutes | Bacteria |
| ASV5497 | 427 | 1 | 1 | 427 | 100 | NR_028816.1 Turicibacter sanguinis strain MOL361 16S ribosomal RNA, partial sequence | NR_028816.1 | 1508 | 327 | 753 | 28.32 | 462.8 | 6E-130 | 369/428 | 86 | 2/428 | 0 | Plus | Plus | Bacteria; Firmicutes; Erysipelotrichia; Erysipelotrichales; Turicibacteraceae; Turicibacter; Turicibacter sanguinis | Turicibacter sanguinis | Turicibacter | Turicibacteraceae | Erysipelotrichales | Erysipelotrichia | Firmicutes | Bacteria |
| ASV5498 | 401 | 1 | 3 | 399 | 99.5 | NR_027573.1 Intestinibacter bartlettii strain WAL 16138 16S ribosomal RNA, partial sequence | NR_027573.1 | 1429 | 320 | 718 | 27.92 | 488.6 | 9E-138 | 355/400 | 88 | 2/400 | 0 | Plus | Plus | Bacteria; Firmicutes; Clostridia; Eubacteriales; Peptostreptococcaceae; Intestinibacter; Intestinibacter bartlettii | Intestinibacter bartlettii | Intestinibacter | Peptostreptococcaceae | Eubacteriales | Clostridia | Firmicutes | Bacteria |
| ASV5499 | 421 | 1 | 3 | 421 | 99.52 | NR_044111.1 Selenomonas bovis strain WG 16S ribosomal RNA, partial sequence | NR_044111.1 | 1525 | 372 | 796 | 27.87 | 575.4 | 7E-164 | 392/429 | 91 | 14/429 | 3 | Plus | Plus | Bacteria; Firmicutes; Negativicutes; Selenomonadales; Selenomonadaceae; Selenomonas; Selenomonas bovis | Selenomonas bovis | Selenomonas | Selenomonadaceae | Selenomonadales | Negativicutes | Firmicutes | Bacteria |
| ASV5501 | 428 | 4 | 3 | 428 | 99.53 | NR_042802.1 Lactobacillus ultunensis strain Kx146C1 16S ribosomal RNA, partial sequence | NR_042802.1 | 1550 | 372 | 796 | 27.42 | 586.5 | 4E-167 | 392/428 | 91 | 5/428 | 1 | Plus | Plus | Bacteria; Firmicutes; Bacilli; Lactobacillales; Lactobacillaceae; Lactobacillus; Lactobacillus ultunensis | Lactobacillus ultunensis | Lactobacillus | Lactobacillaceae | Lactobacillales | Bacilli | Firmicutes | Bacteria |
| ASV5502 | 402 | 1 | 1 | 402 | 100 | NR_134772.1 Murimonas intestini strain SRB-530-5-H 16S ribosomal RNA, partial sequence | NR_134772.1 | 1433 | 314 | 715 | 28.05 | 488.6 | 1E-137 | 357/403 | 88 | 2/403 | 0 | Plus | Plus | Bacteria; Firmicutes; Clostridia; Eubacteriales; Lachnospiraceae; Murimonas; Murimonas intestini | Murimonas intestini | Murimonas | Lachnospiraceae | Eubacteriales | Clostridia | Firmicutes | Bacteria |
| ASV5503 | 428 | 1 | 1 | 427 | 99.77 | NR_102980.1 Megasphaera elsdenii DSM 20460 16S ribosomal RNA, partial sequence | NR_102980.1 | 1552 | 369 | 794 | 27.45 | 483.1 | 5E-136 | 376/431 | 87 | 9/431 | 2 | Plus | Plus | Bacteria; Firmicutes; Negativicutes; Veillonellales; Veillonellaceae; Megasphaera; Megasphaera elsdenii | Megasphaera elsdenii | Megasphaera | Veillonellaceae | Veillonellales | Negativicutes | Firmicutes | Bacteria |
| ASV5508 | 427 | 5 | 4 | 427 | 99.3 | NR_041781.1 Streptococcus alactolyticus strain ATCC 43077 16S ribosomal RNA, partial sequence | NR_041781.1 | 1437 | 349 | 772 | 29.51 | 562.5 | 6E-160 | 389/429 | 90 | 10/429 | 2 | Plus | Plus | Bacteria; Firmicutes; Bacilli; Lactobacillales; Streptococcaceae; Streptococcus; Streptococcus alactolyticus | Streptococcus alactolyticus | Streptococcus | Streptococcaceae | Lactobacillales | Bacilli | Firmicutes | Bacteria |
| ASV5509 | 401 | 1 | 1 | 401 | 100 | NR_137408.1 Terrisporobacter petrolearius strain LAM0A37 16S ribosomal RNA, partial sequence | NR_137408.1 | 1405 | 307 | 707 | 28.54 | 581 | 2E-165 | 372/401 | 92 | 0/401 | 0 | Plus | Plus | Bacteria; Firmicutes; Clostridia; Eubacteriales; Peptostreptococcaceae; Terrisporobacter; Terrisporobacter petrolearius | Terrisporobacter petrolearius | Terrisporobacter | Peptostreptococcaceae | Eubacteriales | Clostridia | Firmicutes | Bacteria |
| ASV5511 | 427 | 5 | 4 | 427 | 99.3 | NR_041781.1 Streptococcus alactolyticus strain ATCC 43077 16S ribosomal RNA, partial sequence | NR_041781.1 | 1437 | 349 | 772 | 29.51 | 501.6 | 1E-141 | 378/429 | 88 | 10/429 | 2 | Plus | Plus | Bacteria; Firmicutes; Bacilli; Lactobacillales; Streptococcaceae; Streptococcus; Streptococcus alactolyticus | Streptococcus alactolyticus | Streptococcus | Streptococcaceae | Lactobacillales | Bacilli | Firmicutes | Bacteria |
| ASV5512 | 427 | 3 | 4 | 427 | 99.3 | NR_041781.1 Streptococcus alactolyticus strain ATCC 43077 16S ribosomal RNA, partial sequence | NR_041781.1 | 1437 | 349 | 772 | 29.51 | 529.3 | 6E-150 | 383/429 | 89 | 10/429 | 2 | Plus | Plus | Bacteria; Firmicutes; Bacilli; Lactobacillales; Streptococcaceae; Streptococcus; Streptococcus alactolyticus | Streptococcus alactolyticus | Streptococcus | Streptococcaceae | Lactobacillales | Bacilli | Firmicutes | Bacteria |
| ASV5515 | 401 | 1 | 1 | 399 | 99.5 | NR_115712.1 Clostridium hydrogeniformans DSM 21757 strain BL-20 16S ribosomal RNA, partial sequence | NR_115712.1 | 1447 | 311 | 710 | 27.64 | 449.9 | 4E-126 | 351/403 | 87 | 7/403 | 1 | Plus | Plus | Bacteria; Firmicutes; Clostridia; Eubacteriales; Clostridiaceae; Clostridium; Clostridium hydrogeniformans | Clostridium hydrogeniformans | Clostridium | Clostridiaceae | Eubacteriales | Clostridia | Firmicutes | Bacteria |
| ASV5517 | 405 | 1 | 1 | 405 | 100 | NR_025025.1 Papillibacter cinnamivorans strain CIN1 16S ribosomal RNA, partial sequence | NR_025025.1 | 1491 | 349 | 751 | 27.03 | 497.9 | 2E-140 | 359/405 | 88 | 2/405 | 0 | Plus | Plus | Bacteria; Firmicutes; Clostridia; Eubacteriales; Oscillospiraceae; Papillibacter; Papillibacter cinnamivorans | Papillibacter cinnamivorans | Papillibacter | Oscillospiraceae | Eubacteriales | Clostridia | Firmicutes | Bacteria |
| ASV5519 | 401 | 1 | 1 | 401 | 100 | NR_144749.1 Ihubacter massiliensis strain Marseille-P2843 16S ribosomal RNA, partial sequence | NR_144749.1 | 1479 | 364 | 766 | 27.25 | 544 | 2E-154 | 367/403 | 91 | 2/403 | 0 | Plus | Plus | Bacteria; Firmicutes; Clostridia; Eubacteriales; Clostridiales Family XIII. Incertae Sedis; Ihubacter; Ihubacter massiliensis | Ihubacter massiliensis | Ihubacter | Clostridiales Family XIII. Incertae Sedis | Eubacteriales | Clostridia | Firmicutes | Bacteria |
| ASV5522 | 427 | 3 | 1 | 427 | 100 | NR_112903.1 Parasutterella secunda strain YIT 12071 16S ribosomal RNA, partial sequence | NR_112903.1 | 1485 | 329 | 755 | 28.75 | 584.7 | 1E-166 | 391/428 | 91 | 2/428 | 0 | Plus | Plus | Bacteria; Proteobacteria; Betaproteobacteria; Burkholderiales; Sutterellaceae; Parasutterella; Parasutterella secunda | Parasutterella secunda | Parasutterella | Sutterellaceae | Burkholderiales | Betaproteobacteria | Proteobacteria | Bacteria |
| ASV5523 | 404 | 1 | 1 | 404 | 100 | NR_041236.1 Lutispora thermophila DSM 19022 strain EBR46 16S ribosomal RNA, partial sequence | NR_041236.1 | 1438 | 338 | 740 | 28.03 | 484.9 | 1E-136 | 360/407 | 88 | 7/407 | 1 | Plus | Plus | Bacteria; Firmicutes; Clostridia; Eubacteriales; Clostridiaceae; Lutispora; Lutispora thermophila | Lutispora thermophila | Lutispora | Clostridiaceae | Eubacteriales | Clostridia | Firmicutes | Bacteria |
| ASV5525 | 427 | 5 | 5 | 427 | 99.06 | NR_119109.1 Pseudescherichia vulneris strain ATCC 33821 16S ribosomal RNA, partial sequence | NR_119109.1 | 1443 | 348 | 770 | 29.31 | 677 | 0 | 404/423 | 95 | 0/423 | 0 | Plus | Plus | Bacteria; Proteobacteria; Gammaproteobacteria; Enterobacterales; Enterobacteriaceae; Pseudescherichia; Pseudescherichia vulneris | Pseudescherichia vulneris | Pseudescherichia | Enterobacteriaceae | Enterobacterales | Gammaproteobacteria | Proteobacteria | Bacteria |
| ASV5526 | 403 | 1 | 4 | 403 | 99.26 | NR_044716.2 Clostridium carnis strain ATCC 25777 16S ribosomal RNA, partial sequence | NR_044716.2 | 1455 | 360 | 758 | 27.42 | 481.2 | 2E-135 | 355/402 | 88 | 5/402 | 1 | Plus | Plus | Bacteria; Firmicutes; Clostridia; Eubacteriales; Clostridiaceae; Clostridium; Clostridium carnis | Clostridium carnis | Clostridium | Clostridiaceae | Eubacteriales | Clostridia | Firmicutes | Bacteria |
| ASV5527 | 402 | 1 | 1 | 402 | 100 | NR_027562.1 Anaerofustis stercorihominis strain WAL 14563 16S ribosomal RNA, partial sequence | NR_027562.1 | 1462 | 335 | 736 | 27.5 | 643.8 | 0 | 384/402 | 95 | 0/402 | 0 | Plus | Plus | Bacteria; Firmicutes; Clostridia; Eubacteriales; Eubacteriaceae; Anaerofustis; Anaerofustis stercorihominis | Anaerofustis stercorihominis | Anaerofustis | Eubacteriaceae | Eubacteriales | Clostridia | Firmicutes | Bacteria |
| ASV5529 | 402 | 1 | 3 | 402 | 99.5 | NR_029355.1 [Clostridium] methylpentosum strain R2 16S ribosomal RNA, partial sequence | NR_029355.1 | 1435 | 351 | 750 | 27.87 | 496 | 6E-140 | 357/401 | 89 | 2/401 | 0 | Plus | Plus | Bacteria; Firmicutes; Clostridia; Eubacteriales; Oscillospiraceae; ; [Clostridium] methylpentosum | [Clostridium] methylpentosum | | Oscillospiraceae | Eubacteriales | Clostridia | Firmicutes | Bacteria |
| ASV5535 | 422 | 1 | 1 | 422 | 100 | NR_113411.1 Prevotella copri DSM 18205 strain JCM 13464 16S ribosomal RNA, partial sequence | NR_113411.1 | 1493 | 358 | 779 | 28.27 | 592 | 7E-169 | 388/422 | 91 | 0/422 | 0 | Plus | Plus | Bacteria; Bacteroidetes; Bacteroidia; Bacteroidales; Prevotellaceae; Prevotella; Prevotella copri | Prevotella copri | Prevotella | Prevotellaceae | Bacteroidales | Bacteroidia | Bacteroidetes | Bacteria |
| ASV5537 | 422 | 1 | 1 | 422 | 100 | NR_113411.1 Prevotella copri DSM 18205 strain JCM 13464 16S ribosomal RNA, partial sequence | NR_113411.1 | 1493 | 358 | 779 | 28.27 | 630.8 | 2E-180 | 395/422 | 93 | 0/422 | 0 | Plus | Plus | Bacteria; Bacteroidetes; Bacteroidia; Bacteroidales; Prevotellaceae; Prevotella; Prevotella copri | Prevotella copri | Prevotella | Prevotellaceae | Bacteroidales | Bacteroidia | Bacteroidetes | Bacteria |
| ASV5538 | 402 | 2 | 3 | 402 | 99.5 | NR_147371.1 Intestinibacillus massiliensis strain Marseille-P3216 16S ribosomal RNA, partial sequence | NR_147371.1 | 1424 | 296 | 696 | 28.16 | 473.9 | 3E-133 | 353/401 | 88 | 1/401 | 0 | Plus | Plus | Bacteria; Firmicutes; Clostridia; Eubacteriales; Eubacteriaceae; Intestinibacillus; Intestinibacillus massiliensis | Intestinibacillus massiliensis | Intestinibacillus | Eubacteriaceae | Eubacteriales | Clostridia | Firmicutes | Bacteria |
| ASV5544 | 428 | 1 | 3 | 428 | 99.53 | NR_136805.1 Marinithermofilum abyssi strain SCSIO 11157 16S ribosomal RNA, partial sequence | NR_136805.1 | 1523 | 372 | 797 | 27.97 | 460.9 | 2E-129 | 372/431 | 86 | 10/431 | 2 | Plus | Plus | Bacteria; Firmicutes; Bacilli; Bacillales; Thermoactinomycetaceae; Marinithermofilum; Marinithermofilum abyssi | Marinithermofilum abyssi | Marinithermofilum | Thermoactinomycetaceae | Bacillales | Bacilli | Firmicutes | Bacteria |
| ASV5545 | 422 | 3 | 1 | 407 | 96.45 | NR_113110.1 Prevotella maculosa strain JCM 15638 16S ribosomal RNA, partial sequence | NR_113110.1 | 1405 | 269 | 675 | 28.97 | 481.2 | 2E-135 | 360/409 | 88 | 4/409 | 0 | Plus | Plus | Bacteria; Bacteroidetes; Bacteroidia; Bacteroidales; Prevotellaceae; Prevotella; Prevotella maculosa | Prevotella maculosa | Prevotella | Prevotellaceae | Bacteroidales | Bacteroidia | Bacteroidetes | Bacteria |
| ASV5546 | 401 | 2 | 3 | 401 | 99.5 | NR_144737.1 Emergencia timonensis strain SN18 16S ribosomal RNA, partial sequence | NR_144737.1 | 1480 | 366 | 767 | 27.16 | 540.3 | 3E-153 | 366/402 | 91 | 3/402 | 0 | Plus | Plus | Bacteria; Firmicutes; Clostridia; Eubacteriales; Clostridiales Family XIII. Incertae Sedis; Emergencia; Emergencia timonensis | Emergencia timonensis | Emergencia | Clostridiales Family XIII. Incertae Sedis | Eubacteriales | Clostridia | Firmicutes | Bacteria |
| ASV5547 | 427 | 1 | 3 | 427 | 99.53 | NR_102980.1 Megasphaera elsdenii DSM 20460 16S ribosomal RNA, partial sequence | NR_102980.1 | 1552 | 371 | 795 | 27.38 | 547.7 | 2E-155 | 385/428 | 89 | 6/428 | 1 | Plus | Plus | Bacteria; Firmicutes; Negativicutes; Veillonellales; Veillonellaceae; Megasphaera; Megasphaera elsdenii | Megasphaera elsdenii | Megasphaera | Veillonellaceae | Veillonellales | Negativicutes | Firmicutes | Bacteria |
| ASV5549 | 428 | 1 | 3 | 428 | 99.53 | NR_041666.1 Dialister succinatiphilus YIT 11850 16S ribosomal RNA, partial sequence | NR_041666.1 | 1521 | 355 | 779 | 27.94 | 464.6 | 2E-130 | 369/427 | 86 | 3/427 | 0 | Plus | Plus | Bacteria; Firmicutes; Negativicutes; Veillonellales; Veillonellaceae; Dialister; Dialister succinatiphilus | Dialister succinatiphilus | Dialister | Veillonellaceae | Veillonellales | Negativicutes | Firmicutes | Bacteria |
| ASV5550 | 402 | 3 | 1 | 402 | 100 | NR_114728.1 Clostridium tertium strain ATCC 14573 16S ribosomal RNA, partial sequence | NR_114728.1 | 1415 | 304 | 705 | 28.41 | 566.2 | 4E-161 | 372/404 | 92 | 4/404 | 0 | Plus | Plus | Bacteria; Firmicutes; Clostridia; Eubacteriales; Clostridiaceae; Clostridium; Clostridium tertium | Clostridium tertium | Clostridium | Clostridiaceae | Eubacteriales | Clostridia | Firmicutes | Bacteria |
| ASV5551 | 422 | 2 | 1 | 422 | 100 | NR_113076.1 Parabacteroides goldsteinii DSM 19448 = WAL 12034 strain JCM 13446 16S ribosomal RNA, partial sequence | NR_113076.1 | 1476 | 341 | 762 | 28.59 | 569.9 | 3E-162 | 385/423 | 91 | 2/423 | 0 | Plus | Plus | Bacteria; Bacteroidetes; Bacteroidia; Bacteroidales; Tannerellaceae; Parabacteroides; Parabacteroides goldsteinii | Parabacteroides goldsteinii | Parabacteroides | Tannerellaceae | Bacteroidales | Bacteroidia | Bacteroidetes | Bacteria |
| ASV5552 | 404 | 1 | 1 | 404 | 100 | NR_144611.1 Flintibacter butyricus strain BLS21 16S ribosomal RNA, partial sequence | NR_144611.1 | 808 | 299 | 703 | 50.12 | 586.5 | 3E-167 | 376/405 | 92 | 1/405 | 0 | Plus | Plus | Bacteria; Firmicutes; Clostridia; Eubacteriales; ; Flintibacter; Flintibacter butyricus | Flintibacter butyricus | Flintibacter | | Eubacteriales | Clostridia | Firmicutes | Bacteria |
| ASV5553 | 405 | 4 | 1 | 405 | 100 | NR_042802.1 Lactobacillus ultunensis strain Kx146C1 16S ribosomal RNA, partial sequence | NR_042802.1 | 1550 | 370 | 796 | 27.55 | 547.7 | 2E-155 | 387/427 | 90 | 22/427 | 5 | Plus | Plus | Bacteria; Firmicutes; Bacilli; Lactobacillales; Lactobacillaceae; Lactobacillus; Lactobacillus ultunensis | Lactobacillus ultunensis | Lactobacillus | Lactobacillaceae | Lactobacillales | Bacilli | Firmicutes | Bacteria |
| ASV5554 | 401 | 1 | 1 | 401 | 100 | NR_144737.1 Emergencia timonensis strain SN18 16S ribosomal RNA, partial sequence | NR_144737.1 | 1480 | 364 | 767 | 27.3 | 510.8 | 2E-144 | 362/404 | 89 | 3/404 | 0 | Plus | Plus | Bacteria; Firmicutes; Clostridia; Eubacteriales; Clostridiales Family XIII. Incertae Sedis; Emergencia; Emergencia timonensis | Emergencia timonensis | Emergencia | Clostridiales Family XIII. Incertae Sedis | Eubacteriales | Clostridia | Firmicutes | Bacteria |
| ASV5559 | 401 | 1 | 1 | 401 | 100 | NR_029355.1 [Clostridium] methylpentosum strain R2 16S ribosomal RNA, partial sequence | NR_029355.1 | 1435 | 349 | 750 | 28.01 | 542.2 | 7E-154 | 366/402 | 91 | 1/402 | 0 | Plus | Plus | Bacteria; Firmicutes; Clostridia; Eubacteriales; Oscillospiraceae; ; [Clostridium] methylpentosum | [Clostridium] methylpentosum | | Oscillospiraceae | Eubacteriales | Clostridia | Firmicutes | Bacteria |
| ASV5560 | 409 | 1 | 1 | 409 | 100 | NR_147375.1 Colidextribacter massiliensis strain Marseille-P3083 16S ribosomal RNA, partial sequence | NR_147375.1 | 1487 | 364 | 765 | 27.03 | 505.3 | 1E-142 | 365/409 | 89 | 7/409 | 1 | Plus | Plus | Bacteria; Firmicutes; Clostridia; Eubacteriales; ; Colidextribacter; Colidextribacter massiliensis | Colidextribacter massiliensis | Colidextribacter | | Eubacteriales | Clostridia | Firmicutes | Bacteria |
| ASV5562 | 402 | 1 | 3 | 401 | 99.25 | NR_117867.1 Blautia stercoris strain GAM6-1 16S ribosomal RNA, partial sequence | NR_117867.1 | 1372 | 319 | 717 | 29.08 | 466.5 | 4E-131 | 350/399 | 87 | 0/399 | 0 | Plus | Plus | Bacteria; Firmicutes; Clostridia; Eubacteriales; Lachnospiraceae; Blautia; Blautia stercoris | Blautia stercoris | Blautia | Lachnospiraceae | Eubacteriales | Clostridia | Firmicutes | Bacteria |
| ASV5563 | 402 | 1 | 1 | 402 | 100 | NR_144736.1 Neglecta timonensis strain SN17 16S ribosomal RNA, partial sequence | NR_144736.1 | 1503 | 377 | 778 | 26.75 | 544 | 2E-154 | 366/402 | 91 | 0/402 | 0 | Plus | Plus | Bacteria; Firmicutes; Clostridia; Eubacteriales; ; Neglecta; Neglecta timonensis | Neglecta timonensis | Neglecta | | Eubacteriales | Clostridia | Firmicutes | Bacteria |
| ASV5565 | 404 | 1 | 1 | 404 | 100 | NR_112097.1 Pseudoramibacter alactolyticus ATCC 23263 16S ribosomal RNA, partial sequence | NR_112097.1 | 1480 | 376 | 779 | 27.3 | 614.2 | 2E-175 | 381/405 | 94 | 2/405 | 0 | Plus | Plus | Bacteria; Firmicutes; Clostridia; Eubacteriales; Eubacteriaceae; Pseudoramibacter; Pseudoramibacter alactolyticus | Pseudoramibacter alactolyticus | Pseudoramibacter | Eubacteriaceae | Eubacteriales | Clostridia | Firmicutes | Bacteria |
| ASV5568 | 405 | 1 | 3 | 405 | 99.51 | NR_144742.1 Christensenella massiliensis strain Marseille-P2438 16S ribosomal RNA, partial sequence | NR_144742.1 | 1499 | 371 | 773 | 26.88 | 479.4 | 6E-135 | 355/403 | 88 | 0/403 | 0 | Plus | Plus | Bacteria; Firmicutes; Clostridia; Eubacteriales; Christensenellaceae; Christensenella; Christensenella massiliensis | Christensenella massiliensis | Christensenella | Christensenellaceae | Eubacteriales | Clostridia | Firmicutes | Bacteria |
| ASV5569 | 421 | 3 | 1 | 421 | 100 | NR_121773.2 Barnesiella viscericola strain C46 16S ribosomal RNA, complete sequence | NR_121773.2 | 1532 | 365 | 786 | 27.55 | 479.4 | 6E-135 | 370/424 | 87 | 5/424 | 1 | Plus | Plus | Bacteria; Bacteroidetes; Bacteroidia; Bacteroidales; Barnesiellaceae; Barnesiella; Barnesiella viscericola | Barnesiella viscericola | Barnesiella | Barnesiellaceae | Bacteroidales | Bacteroidia | Bacteroidetes | Bacteria |
| ASV5576 | 428 | 1 | 1 | 428 | 100 | NR_156911.1 Fournierella massiliensis strain AT2 16S ribosomal RNA, partial sequence | NR_156911.1 | 1491 | 368 | 769 | 26.96 | 479.4 | 6E-135 | 377/429 | 87 | 28/429 | 6 | Plus | Plus | Bacteria; Firmicutes; Clostridia; Eubacteriales; Oscillospiraceae; Fournierella; Fournierella massiliensis | Fournierella massiliensis | Fournierella | Oscillospiraceae | Eubacteriales | Clostridia | Firmicutes | Bacteria |
| ASV5577 | 422 | 2 | 1 | 422 | 100 | NR_113411.1 Prevotella copri DSM 18205 strain JCM 13464 16S ribosomal RNA, partial sequence | NR_113411.1 | 1493 | 358 | 779 | 28.27 | 636.4 | 0 | 396/422 | 93 | 0/422 | 0 | Plus | Plus | Bacteria; Bacteroidetes; Bacteroidia; Bacteroidales; Prevotellaceae; Prevotella; Prevotella copri | Prevotella copri | Prevotella | Prevotellaceae | Bacteroidales | Bacteroidia | Bacteroidetes | Bacteria |
| ASV5578 | 427 | 2 | 3 | 427 | 99.53 | NR_113306.1 Megasphaera elsdenii strain JCM 1772 16S ribosomal RNA, partial sequence | NR_113306.1 | 1174 | 234 | 658 | 36.2 | 577.3 | 2E-164 | 388/426 | 91 | 2/426 | 0 | Plus | Plus | Bacteria; Firmicutes; Negativicutes; Veillonellales; Veillonellaceae; Megasphaera; Megasphaera elsdenii | Megasphaera elsdenii | Megasphaera | Veillonellaceae | Veillonellales | Negativicutes | Firmicutes | Bacteria |
| ASV5580 | 402 | 1 | 3 | 402 | 99.5 | NR_113316.1 Collinsella aerofaciens strain JCM 10188 16S ribosomal RNA, partial sequence | NR_113316.1 | 1467 | 346 | 746 | 27.33 | 573.6 | 3E-163 | 371/401 | 92 | 1/401 | 0 | Plus | Plus | Bacteria; Actinobacteria; Coriobacteriia; Coriobacteriales; Coriobacteriaceae; Collinsella; Collinsella aerofaciens | Collinsella aerofaciens | Collinsella | Coriobacteriaceae | Coriobacteriales | Coriobacteriia | Actinobacteria | Bacteria |
| ASV5581 | 427 | 1 | 1 | 427 | 100 | NR_025981.1 Butyrivibrio fibrisolvens strain ATCC 19171 16S ribosomal RNA, partial sequence | NR_025981.1 | 1477 | 336 | 753 | 28.3 | 464.6 | 2E-130 | 370/427 | 86 | 9/427 | 2 | Plus | Plus | Bacteria; Firmicutes; Clostridia; Eubacteriales; Lachnospiraceae; Butyrivibrio; Butyrivibrio fibrisolvens | Butyrivibrio fibrisolvens | Butyrivibrio | Lachnospiraceae | Eubacteriales | Clostridia | Firmicutes | Bacteria |
| ASV5583 | 426 | 1 | 1 | 426 | 100 | NR_147371.1 Intestinibacillus massiliensis strain Marseille-P3216 16S ribosomal RNA, partial sequence | NR_147371.1 | 1424 | 294 | 696 | 28.3 | 422.2 | 1E-117 | 364/426 | 85 | 23/426 | 5 | Plus | Plus | Bacteria; Firmicutes; Clostridia; Eubacteriales; Eubacteriaceae; Intestinibacillus; Intestinibacillus massiliensis | Intestinibacillus massiliensis | Intestinibacillus | Eubacteriaceae | Eubacteriales | Clostridia | Firmicutes | Bacteria |
| ASV5587 | 428 | 5 | 1 | 428 | 100 | NR_042052.1 Streptococcus equinus ATCC 9812 16S ribosomal RNA, partial sequence | NR_042052.1 | 1433 | 260 | 686 | 29.8 | 468.3 | 1E-131 | 370/428 | 86 | 1/428 | 0 | Plus | Plus | Bacteria; Firmicutes; Bacilli; Lactobacillales; Streptococcaceae; Streptococcus; Streptococcus equinus | Streptococcus equinus | Streptococcus | Streptococcaceae | Lactobacillales | Bacilli | Firmicutes | Bacteria |
| ASV5588 | 426 | 2 | 1 | 426 | 100 | NR_116012.1 Streptococcus equi subsp. ruminatorum CECT 5772 strain CCUG 47520 16S ribosomal RNA, partial sequence | NR_116012.1 | 1470 | 337 | 763 | 29.05 | 632.7 | 0 | 401/429 | 93 | 5/429 | 1 | Plus | Plus | Bacteria; Firmicutes; Bacilli; Lactobacillales; Streptococcaceae; Streptococcus; Streptococcus equi | Streptococcus equi | Streptococcus | Streptococcaceae | Lactobacillales | Bacilli | Firmicutes | Bacteria |

| | | | | | | | | | | | | | | | | | | | | |
|---|---|---|---|---|---|---|---|---|---|---|---|---|---|---|---|---|---|---|---|---|
| ASV5595 | 404 | 1 | 1 | 404 | 100 | NR_115340.1 Saccharofermentans acetigenes strain P6 16S ribosomal RNA, partial sequence | NR_115340.1 | 1510 | 353 | 757 | 26.82 | 464.6 | 2E-130 | 354/405 | 87 | 1/405 | 0 | Plus | Plus | [illegible] |
| ASV5596 | 421 | 1 | 1 | 421 | 100 | NR_144616.1 Muribaculum intestinale strain YL27 16S ribosomal RNA, partial sequence | NR_144616.1 | 1460 | 297 | 718 | 28.9 | 462.8 | 6E-130 | 370/427 | 86 | 11/427 | 2 | Plus | Plus | [illegible] |
| ASV5597 | 422 | 1 | 1 | 407 | 96.45 | NR_041364.1 Prevotella stercorea DSM 18206 strain CB35 16S ribosomal RNA, partial sequence | NR_041364.1 | 1488 | 354 | 760 | 27.35 | 575.4 | 7E-164 | 376/408 | 92 | 2/408 | 0 | Plus | Plus | [illegible] |
| ASV5599 | 402 | 1 | 1 | 402 | 100 | NR_144749.1 Ihubacter massiliensis strain Marseille-P2843 16S ribosomal RNA, partial sequence | NR_144749.1 | 1479 | 364 | 766 | 27.25 | 527.4 | 2E-149 | 366/405 | 90 | 5/405 | 1 | Plus | Plus | [illegible] |
| ASV5602 | 402 | 1 | 1 | 402 | 100 | NR_109625.1 Oceanirhabdus sediminicola strain NH-JN4 16S ribosomal RNA, partial sequence | NR_109625.1 | 1473 | 350 | 751 | 27.29 | 499.7 | 4E-141 | 358/402 | 89 | 0/402 | 0 | Plus | Plus | [illegible] |
| ASV5603 | 405 | 2 | 1 | 405 | 100 | NR_074440.1 Leptotrichia buccalis C-1013-b strain DSM 1135 16S ribosomal RNA, partial sequence | NR_074440.1 | 1505 | 346 | 750 | 26.91 | 715.8 | 0 | 399/405 | 98 | 0/405 | 0 | Plus | Plus | [illegible] |
| ASV5604 | 409 | 1 | 1 | 409 | 100 | NR_042832.1 Roseburia faecis strain M72/1 16S ribosomal RNA, partial sequence | NR_042832.1 | 1456 | 347 | 749 | 27.68 | 568 | 1E-161 | 376/409 | 91 | 6/409 | 1 | Plus | Plus | [illegible] |
| ASV5605 | 405 | 1 | 3 | 405 | 99.51 | NR_028816.1 Turicibacter sanguinis strain MOL361 16S ribosomal RNA, partial sequence | NR_028816.1 | 1508 | 329 | 753 | 28.18 | 560.7 | 2E-159 | 388/425 | 91 | 22/425 | 5 | Plus | Plus | [illegible] |
| ASV5607 | 403 | 1 | 3 | 403 | 99.5 | NR_025930.1 Ruminococcus bromii strain ATCC 27255 16S ribosomal RNA, partial sequence | NR_025930.1 | 1380 | 315 | 716 | 29.13 | 448 | 2E-125 | 351/404 | 86 | 5/404 | 1 | Plus | Plus | [illegible] |
| ASV5608 | 402 | 1 | 1 | 400 | 99.5 | NR_028961.1 Faecalibacterium prausnitzii strain ATCC 27768 16S ribosomal RNA, partial sequence | NR_028961.1 | 1462 | 344 | 743 | 27.36 | 601.3 | 1E-171 | 375/400 | 93 | 0/400 | 0 | Plus | Plus | [illegible] |
| ASV5609 | 402 | 2 | 1 | 402 | 100 | NR_029355.1 [Clostridium] methylpentosum strain R2 16S ribosomal RNA, partial sequence | NR_029355.1 | 1435 | 349 | 750 | 28.01 | 483.1 | 4E-136 | 358/405 | 88 | 6/405 | 1 | Plus | Plus | [illegible] |
| ASV5613 | 401 | 1 | 1 | 401 | 100 | NR_036928.1 Hungatella hathewayi strain 1313 16S ribosomal RNA, partial sequence | NR_036928.1 | 1431 | 350 | 751 | 28.09 | 508.9 | 7E-144 | 360/402 | 89 | 1/402 | 0 | Plus | Plus | [illegible] |
| ASV5614 | 405 | 4 | 1 | 405 | 100 | NR_042802.1 Lactobacillus ultunensis strain Kx146C1 16S ribosomal RNA, partial sequence | NR_042802.1 | 1550 | 370 | 796 | 27.55 | 508.9 | 7E-144 | 380/427 | 88 | 22/427 | 5 | Plus | Plus | [illegible] |
| ASV5615 | 404 | 1 | 3 | 404 | 99.5 | NR_118673.1 [Eubacterium] hallii strain ATCC 27751 16S ribosomal RNA, partial sequence | NR_118673.1 | 1479 | 373 | 772 | 27.05 | 577.3 | 2E-164 | 372/403 | 92 | 4/403 | 0 | Plus | Plus | [illegible] |
| ASV5616 | 405 | 1 | 1 | 405 | 100 | NR_026476.1 Succinivibrio dextrinosolvens strain 0554 16S ribosomal RNA, partial sequence | NR_026476.1 | 1463 | 331 | 732 | 27.48 | 507.1 | 3E-143 | 366/409 | 89 | 11/409 | 2 | Plus | Plus | [illegible] |
| ASV5617 | 405 | 3 | 1 | 404 | 99.75 | NR_113360.1 Ruminiclostridium cellobioparum DSM 1351 = ATCC 15832 strain JCM 1422 16S ribosomal RNA, partial sequence | NR_113360.1 | 1484 | 356 | 759 | 27.22 | 453.5 | 3E-127 | 354/407 | 86 | 6/407 | 1 | Plus | Plus | [illegible] |
| ASV5620 | 425 | 3 | 15 | 424 | 96.47 | NR_102980.1 Megasphaera elsdenii DSM 20460 16S ribosomal RNA, partial sequence | NR_102980.1 | 1552 | 383 | 794 | 26.55 | 449.9 | 5E-126 | 359/415 | 86 | 8/415 | 1 | Plus | Plus | [illegible] |
| ASV5626 | 402 | 1 | 1 | 402 | 100 | NR_159227.1 Monoglobus pectinilyticus strain 14 16S ribosomal RNA, partial sequence | NR_159227.1 | 1493 | 368 | 769 | 26.93 | 499.7 | 4E-141 | 358/402 | 89 | 0/402 | 0 | Plus | Plus | [illegible] |
| ASV5628 | 427 | 2 | 1 | 427 | 100 | NR_042144.1 Clostridium butyricum strain VPI3266 16S ribosomal RNA, partial sequence | NR_042144.1 | 1425 | 338 | 739 | 28.21 | 560.7 | 2E-159 | 390/427 | 91 | 25/427 | 5 | Plus | Plus | [illegible] |
| ASV5629 | 402 | 1 | 1 | 402 | 100 | NR_156911.1 Fournierella massiliensis strain AT2 16S ribosomal RNA, partial sequence | NR_156911.1 | 1491 | 368 | 769 | 26.96 | 538.5 | 9E-153 | 365/402 | 90 | 0/402 | 0 | Plus | Plus | [illegible] |
| ASV5630 | 404 | 1 | 3 | 404 | 99.5 | NR_125711.1 Arthrospira platensis strain PCC 7345 16S ribosomal RNA, partial sequence | NR_125711.1 | 1460 | 299 | 700 | 27.53 | 414.8 | 2E-115 | 347/407 | 85 | 10/407 | 2 | Plus | Plus | [illegible] |
| ASV5631 | 402 | 1 | 1 | 402 | 100 | NR_044425.1 Hydrogenoanaerobacterium saccharovorans strain SW512 16S ribosomal RNA, partial sequence | NR_044425.1 | 1510 | 363 | 764 | 26.62 | 499.7 | 4E-141 | 362/406 | 89 | 8/406 | 1 | Plus | Plus | [illegible] |
| ASV5632 | 426 | 1 | 1 | 426 | 100 | NR_028840.1 Mitsuokella jalaludinii strain M9 16S ribosomal RNA, partial sequence | NR_028840.1 | 1543 | 362 | 788 | 27.67 | 549.6 | 5E-156 | 385/428 | 89 | 3/428 | 0 | Plus | Plus | [illegible] |
| ASV5633 | 405 | 1 | 1 | 405 | 100 | NR_156911.1 Fournierella massiliensis strain AT2 16S ribosomal RNA, partial sequence | NR_156911.1 | 1491 | 368 | 769 | 26.96 | 501.6 | 1E-141 | 362/406 | 89 | 5/406 | 1 | Plus | Plus | [illegible] |
| ASV5638 | 403 | 1 | 3 | 403 | 99.5 | NR_144607.1 Blautia caecimuris strain SJ18 16S ribosomal RNA, partial sequence | NR_144607.1 | 1378 | 301 | 700 | 29.03 | 496 | 6E-140 | 357/401 | 89 | 1/401 | 0 | Plus | Plus | [illegible] |
| ASV5640 | 420 | 2 | 3 | 420 | 99.52 | NR_113306.1 Megasphaera elsdenii DSM 20460 16S ribosomal RNA, partial sequence | NR_113306.1 | 1174 | 234 | 658 | 36.2 | 595.7 | 6E-170 | 392/425 | 92 | 7/425 | 1 | Plus | Plus | [illegible] |
| ASV5641 | 422 | 1 | 1 | 422 | 100 | NR_118554.1 Intestinimonas butyriciproducens strain SRB-521-5-I 16S ribosomal RNA, partial sequence | NR_118554.1 | 1475 | 346 | 750 | 27.46 | 459.1 | 8E-129 | 368/423 | 86 | 19/423 | 4 | Plus | Plus | [illegible] |
| ASV5644 | 417 | 1 | 1 | 415 | 99.52 | NR_025525.1 Butyrivibrio hungatei strain JK 615 16S ribosomal RNA, partial sequence | NR_025525.1 | 1475 | 335 | 747 | 28 | 449.9 | 5E-126 | 360/417 | 86 | 6/417 | 1 | Plus | Plus | [illegible] |
| ASV5646 | 404 | 1 | 1 | 404 | 100 | NR_025930.1 Ruminococcus bromii strain ATCC 27255 16S ribosomal RNA, partial sequence | NR_025930.1 | 1380 | 313 | 716 | 29.28 | 525.6 | 7E-149 | 365/405 | 90 | 2/405 | 0 | Plus | Plus | [illegible] |
| ASV5647 | 422 | 1 | 1 | 422 | 100 | NR_041342.1 Parabacteroides distasonis strain JCM 5825 16S ribosomal RNA, partial sequence | NR_041342.1 | 1488 | 353 | 774 | 28.36 | 492.3 | 8E-139 | 370/422 | 87 | 0/422 | 0 | Plus | Plus | [illegible] |
| ASV5648 | 404 | 1 | 1 | 404 | 100 | NR_025930.1 Ruminococcus bromii strain ATCC 27255 16S ribosomal RNA, partial sequence | NR_025930.1 | 1380 | 313 | 716 | 29.28 | 569.9 | 3E-162 | 373/405 | 92 | 2/405 | 0 | Plus | Plus | [illegible] |
| ASV5649 | 402 | 3 | 1 | 393 | 97.76 | NR_144696.1 Clostridium saudiense strain JCC 16S ribosomal RNA, partial sequence | NR_144696.1 | 1478 | 350 | 742 | 26.59 | 416.6 | 5E-116 | 341/397 | 85 | 8/397 | 2 | Plus | Plus | [illegible] |
| ASV5651 | 403 | 3 | 3 | 403 | 99.5 | NR_114728.1 Clostridium tertium strain ATCC 14573 16S ribosomal RNA, partial sequence | NR_114728.1 | 1415 | 306 | 705 | 28.27 | 540.3 | 3E-153 | 367/403 | 91 | 5/403 | 1 | Plus | Plus | [illegible] |
| ASV5652 | 428 | 1 | 3 | 427 | 99.3 | NR_112902.1 Phascolarctobacterium succinatutens strain YIT 12067 16S ribosomal RNA, partial sequence | NR_112902.1 | 1610 | 446 | 869 | 26.34 | 473.9 | 3E-133 | 371/427 | 86 | 5/427 | 1 | Plus | Plus | [illegible] |
| ASV5654 | 402 | 1 | 1 | 402 | 100 | NR_119085.1 [Clostridium] polysaccharolyticum strain DSM 1801 16S ribosomal RNA, partial sequence | NR_119085.1 | 1503 | 364 | 763 | 26.61 | 503.4 | 3E-142 | 360/403 | 89 | 4/403 | 0 | Plus | Plus | [illegible] |
| ASV5655 | 427 | 4 | 3 | 427 | 99.53 | NR_041781.1 Streptococcus alactolyticus strain ATCC 43077 16S ribosomal RNA, partial sequence | NR_041781.1 | 1437 | 348 | 772 | 29.58 | 575.4 | 8E-164 | 391/429 | 91 | 8/429 | 1 | Plus | Plus | [illegible] |
| ASV5656 | 422 | 1 | 1 | 422 | 100 | NR_112835.1 Parabacteroides gordonii strain MS-1 16S ribosomal RNA, partial sequence | NR_112835.1 | 1485 | 353 | 773 | 28.35 | 490.5 | 3E-138 | 370/422 | 87 | 1/422 | 0 | Plus | Plus | [illegible] |
| ASV5657 | 421 | 2 | 1 | 421 | 100 | NR_116726.1 Prevotella scopos JCM 17725 strain W2052 16S ribosomal RNA, partial sequence | NR_116726.1 | 1453 | 338 | 759 | 29.04 | 457.2 | 3E-128 | 365/423 | 86 | 3/423 | 0 | Plus | Plus | [illegible] |
| ASV5660 | 401 | 1 | 1 | 401 | 100 | NR_144736.1 Neglecta timonensis strain SN17 16S ribosomal RNA, partial sequence | NR_144736.1 | 1503 | 377 | 778 | 26.75 | 475.7 | 7E-134 | 356/404 | 88 | 5/404 | 1 | Plus | Plus | [illegible] |
| ASV5661 | 403 | 2 | 3 | 403 | 99.5 | NR_156907.1 Faecalimonas umbilicata strain EGH7 16S ribosomal RNA, partial sequence | NR_156907.1 | 1492 | 367 | 766 | 26.81 | 507.1 | 3E-143 | 360/402 | 89 | 3/402 | 0 | Plus | Plus | [illegible] |
| ASV5662 | 401 | 1 | 1 | 401 | 100 | NR_025498.1 Sedimentibacter saalensis strain ZF2 16S ribosomal RNA, partial sequence | NR_025498.1 | 1434 | 325 | 728 | 28.17 | 643.8 | 0 | 387/405 | 95 | 5/405 | 1 | Plus | Plus | [illegible] |
| ASV5663 | 428 | 1 | 1 | 427 | 99.77 | NR_152054.1 Streptococcus dentiloxodontae strain NUM2404 16S ribosomal RNA, partial sequence | NR_152054.1 | 1550 | 365 | 790 | 27.48 | 505.3 | 1E-142 | 380/431 | 88 | 9/431 | 2 | Plus | Plus | [illegible] |
| ASV5664 | 422 | 1 | 4 | 422 | 99.29 | NR_113193.1 Aureibacter tunicatorum strain A5Q-118 16S ribosomal RNA, partial sequence | NR_113193.1 | 1485 | 354 | 771 | 28.15 | 468.3 | 1E-131 | 369/424 | 87 | 11/424 | 2 | Plus | Plus | [illegible] |
| ASV5669 | 422 | 1 | 1 | 422 | 100 | NR_113569.1 Portibacter lacus strain YM8-076 16S ribosomal RNA, partial sequence | NR_113569.1 | 1478 | 349 | 770 | 28.55 | 536.6 | 4E-152 | 381/425 | 89 | 6/425 | 1 | Plus | Plus | [illegible] |
| ASV5670 | 405 | 1 | 3 | 405 | 99.51 | NR_114326.1 Fusicatenibacter saccharivorans strain HT03-11 16S ribosomal RNA, partial sequence | NR_114326.1 | 1423 | 353 | 752 | 28.11 | 592 | 7E-169 | 376/403 | 93 | 3/403 | 0 | Plus | Plus | [illegible] |
| ASV5671 | 402 | 1 | 1 | 402 | 100 | NR_134781.1 Olsenella scatoligenes strain SK9K4 16S ribosomal RNA, partial sequence | NR_134781.1 | 1469 | 348 | 755 | 27.77 | 560.7 | 2E-159 | 377/411 | 91 | 12/411 | 2 | Plus | Plus | [illegible] |
| ASV5672 | 422 | 2 | 1 | 422 | 100 | NR_112931.1 Bacteroides acidifaciens strain JCM 10556 16S ribosomal RNA, partial sequence | NR_112931.1 | 1490 | 357 | 778 | 28.32 | 459.1 | 8E-129 | 367/425 | 86 | 6/425 | 1 | Plus | Plus | [illegible] |
| ASV5674 | 401 | 3 | 1 | 386 | 96.26 | NR_148815.1 Paraclostridium benzoelyticum strain JC272 16S ribosomal RNA, partial sequence | NR_148815.1 | 1490 | 336 | 721 | 25.91 | 564.3 | 2E-160 | 359/386 | 93 | 0/386 | 0 | Plus | Plus | [illegible] |
| ASV5677 | 403 | 2 | 1 | 398 | 98.76 | NR_113408.1 Anaerotignum propionicum strain JCM 1430 16S ribosomal RNA, partial sequence | NR_113408.1 | 1495 | 368 | 767 | 26.76 | 422.2 | 1E-117 | 345/402 | 85 | 6/402 | 1 | Plus | Plus | [illegible] |
| ASV5678 | 404 | 1 | 1 | 404 | 100 | NR_042074.1 Eubacterium pyruvativorans strain I-6 16S ribosomal RNA, partial sequence | NR_042074.1 | 1296 | 264 | 667 | 31.17 | 586.5 | 3E-167 | 375/404 | 92 | 0/404 | 0 | Plus | Plus | [illegible] |
| ASV5679 | 422 | 1 | 1 | 422 | 100 | NR_117373.1 Bacteroides sartorii JCM 17136 = DSM 21941 strain A-C2-0 16S ribosomal RNA, partial sequence | NR_117373.1 | 1327 | 306 | 727 | 31.8 | 592 | 7E-169 | 388/422 | 91 | 0/422 | 0 | Plus | Plus | [illegible] |
| ASV5680 | 407 | 1 | 1 | 407 | 100 | NR_108845.1 Nocardioides alpinus strain Cr7-14 16S ribosomal RNA, partial sequence | NR_108845.1 | 1507 | 344 | 750 | 27.01 | 708.4 | 0 | 399/407 | 98 | 0/407 | 0 | Plus | Plus | [illegible] |
| ASV5681 | 404 | 1 | 3 | 404 | 99.5 | NR_044111.1 Selenomonas bovis strain WG 16S ribosomal RNA, partial sequence | NR_044111.1 | 1525 | 372 | 796 | 27.87 | 508.9 | 7E-144 | 380/426 | 89 | 25/426 | 5 | Plus | Plus | [illegible] |
| ASV5682 | 426 | 1 | 1 | 426 | 100 | NR_115225.1 Chthoniobacter flavus Ellin428 16S ribosomal RNA, partial sequence | NR_115225.1 | 1360 | 341 | 766 | 31.32 | 544 | 2E-154 | 385/429 | 89 | 6/429 | 1 | Plus | Plus | [illegible] |
| ASV5683 | 402 | 2 | 3 | 402 | 99.5 | NR_074793.2 Oscillibacter valericigenes strain Sjm18-20 16S ribosomal RNA, complete sequence | NR_074793.2 | 1530 | 374 | 775 | 26.27 | 475.7 | 7E-134 | 359/407 | 88 | 12/407 | 2 | Plus | Plus | [illegible] |
| ASV5684 | 402 | 2 | 1 | 402 | 100 | NR_156080.1 Kineothrix alysoides strain KNHs209 16S ribosomal RNA, partial sequence | NR_156080.1 | 1410 | 353 | 754 | 28.51 | 521.9 | 9E-148 | 362/402 | 90 | 0/402 | 0 | Plus | Plus | [illegible] |
| ASV5685 | 421 | 2 | 1 | 421 | 100 | NR_113077.1 Paraprevotella clara strain JCM 14859 16S ribosomal RNA, partial sequence | NR_113077.1 | 1480 | 346 | 766 | 28.45 | 601.3 | 1E-171 | 390/422 | 92 | 2/422 | 0 | Plus | Plus | [illegible] |
| ASV5686 | 421 | 2 | 1 | 420 | 99.76 | NR_113306.1 Megasphaera elsdenii DSM 20460 16S ribosomal RNA, partial sequence | NR_113306.1 | 1174 | 232 | 657 | 36.29 | 549.6 | 5E-156 | 384/426 | 90 | 6/426 | 1 | Plus | Plus | [illegible] |
| ASV5687 | 422 | 1 | 1 | 413 | 97.87 | NR_041364.1 Prevotella stercorea DSM 18206 strain CB35 16S ribosomal RNA, partial sequence | NR_041364.1 | 1488 | 354 | 766 | 27.76 | 503.4 | 4E-142 | 369/416 | 88 | 6/416 | 1 | Plus | Plus | [illegible] |
| ASV5690 | 421 | 1 | 1 | 421 | 100 | NR_042842.1 Prevotella denticola strain ATCC 35308 16S ribosomal RNA, partial sequence | NR_042842.1 | 1506 | 338 | 759 | 28.02 | 520 | 4E-147 | 376/423 | 88 | 3/423 | 0 | Plus | Plus | [illegible] |
| ASV5691 | 422 | 1 | 1 | 413 | 97.87 | NR_041364.1 Prevotella stercorea DSM 18206 strain CB35 16S ribosomal RNA, partial sequence | NR_041364.1 | 1488 | 354 | 766 | 27.76 | 508.9 | 8E-144 | 370/416 | 88 | 6/416 | 1 | Plus | Plus | [illegible] |
| ASV5694 | 420 | 1 | 1 | 420 | 100 | NR_144616.1 Muribaculum intestinale strain YL27 16S ribosomal RNA, partial sequence | NR_144616.1 | 1460 | 297 | 718 | 28.9 | 490.5 | 3E-138 | 371/423 | 87 | 4/423 | 0 | Plus | Plus | [illegible] |
| ASV5696 | 403 | 1 | 14 | 403 | 96.77 | NR_144749.1 Ihubacter massiliensis strain Marseille-P2843 16S ribosomal RNA, partial sequence | NR_144749.1 | 1479 | 377 | 766 | 26.37 | 488.6 | 1E-137 | 348/390 | 89 | 0/390 | 0 | Plus | Plus | [illegible] |
| ASV5697 | 403 | 1 | 3 | 403 | 99.5 | NR_026104.1 Ruminiclostridium cellobioparum DSM 1351 = ATCC 15832 16S ribosomal RNA, partial sequence | NR_026104.1 | 1487 | 331 | 733 | 27.1 | 521.9 | 9E-148 | 363/403 | 90 | 2/403 | 0 | Plus | Plus | [illegible] |
| ASV5700 | 402 | 2 | 1 | 402 | 100 | NR_158111.1 Paludicola psychrotolerans strain NC1253 16S ribosomal RNA, partial sequence | NR_158111.1 | 1490 | 366 | 766 | 26.91 | 542.2 | 7E-154 | 368/404 | 91 | 5/404 | 1 | Plus | Plus | [illegible] |
| ASV5703 | 402 | 5 | 1 | 402 | 100 | NR_144617.1 Muricomes intestini strain 2PG-424-CC-1 16S ribosomal RNA, partial sequence | NR_144617.1 | 1431 | 357 | 758 | 28.09 | 538.5 | 9E-153 | 367/404 | 90 | 4/404 | 0 | Plus | Plus | [illegible] |
| ASV5708 | 422 | 1 | 1 | 422 | 100 | NR_144616.1 Muribaculum intestinale strain YL27 16S ribosomal RNA, partial sequence | NR_144616.1 | 1460 | 297 | 718 | 28.9 | 436.9 | 4E-122 | 366/428 | 85 | 12/428 | 2 | Plus | Plus | [illegible] |
| ASV5709 | 402 | 1 | 1 | 402 | 100 | NR_147375.1 Colidextribacter massiliensis strain Marseille-P3083 16S ribosomal RNA, partial sequence | NR_147375.1 | 1487 | 364 | 765 | 27.03 | 477.6 | 2E-134 | 355/403 | 88 | 2/403 | 0 | Plus | Plus | [illegible] |
| ASV5710 | 405 | 1 | 1 | 405 | 100 | NR_044972.1 Sporobacter termitidis strain SYR 16S ribosomal RNA, partial sequence | NR_044972.1 | 1506 | 348 | 752 | 26.89 | 520 | 3E-147 | 364/406 | 89 | 2/406 | 0 | Plus | Plus | [illegible] |
| ASV5711 | 403 | 1 | 1 | 403 | 100 | NR_116936.1 Olsenella umbonata strain lac31 16S ribosomal RNA, partial sequence | NR_116936.1 | 1428 | 328 | 735 | 28.57 | 571.7 | 9E-163 | 376/408 | 92 | 5/408 | 1 | Plus | Plus | [illegible] |
| ASV5712 | 425 | 1 | 1 | 425 | 100 | NR_029325.1 Schwartzia succinivorans strain S1-1 16S ribosomal RNA, partial sequence | NR_029325.1 | 1489 | 344 | 769 | 28.61 | 475.7 | 8E-134 | 373/429 | 86 | 7/429 | 1 | Plus | Plus | [illegible] |
| ASV5713 | 403 | 2 | 1 | 403 | 100 | NR_148837.1 Abyssivirga alkaniphila strain L81 16S ribosomal RNA, partial sequence | NR_148837.1 | 1520 | 364 | 765 | 26.45 | 466.5 | 4E-131 | 356/406 | 87 | 7/406 | 1 | Plus | Plus | [illegible] |
| ASV5720 | 422 | 3 | 1 | 422 | 100 | NR_074793.2 Oscillibacter valericigenes strain Sjm18-20 16S ribosomal RNA, complete sequence | NR_074793.2 | 1530 | 372 | 775 | 26.41 | 436.9 | 4E-122 | 364/423 | 86 | 20/423 | 4 | Plus | Plus | [illegible] |
| ASV5723 | 405 | 1 | 1 | 405 | 100 | NR_115269.1 Catabacter hongkongensis strain HKU16 16S ribosomal RNA, partial sequence | NR_115269.1 | 1453 | 349 | 753 | 27.87 | 533 | 4E-151 | 367/406 | 90 | 2/406 | 0 | Plus | Plus | [illegible] |
| ASV5724 | 422 | 1 | 1 | 413 | 97.87 | NR_113411.1 Prevotella copri DSM 18205 strain JCM 13464 16S ribosomal RNA, partial sequence | NR_113411.1 | 1493 | 358 | 770 | 27.66 | 625.3 | 7E-179 | 388/413 | 93 | 0/413 | 0 | Plus | Plus | [illegible] |
| ASV5726 | 422 | 1 | 1 | 413 | 97.87 | NR_113411.1 Prevotella copri DSM 18205 strain JCM 13464 16S ribosomal RNA, partial sequence | NR_113411.1 | 1493 | 358 | 770 | 27.66 | 625.3 | 7E-179 | 388/413 | 93 | 0/413 | 0 | Plus | Plus | [illegible] |
| ASV5730 | 403 | 1 | 3 | 403 | 99.5 | NR_137408.1 Terrisporobacter petrolearius strain LAM0A37 16S ribosomal RNA, partial sequence | NR_137408.1 | 1405 | 309 | 707 | 28.4 | 568 | 1E-161 | 371/402 | 92 | 4/402 | 0 | Plus | Plus | [illegible] |
| ASV5731 | 403 | 4 | 3 | 403 | 99.5 | NR_134044.1 Gordonibacter urolithinfaciens strain CEBAS 1/15P 16S ribosomal RNA, partial sequence | NR_134044.1 | 1477 | 337 | 735 | 27.01 | 468.3 | 1E-131 | 354/403 | 87 | 6/403 | 1 | Plus | Plus | [illegible] |
| ASV5733 | 405 | 1 | 1 | 405 | 100 | NR_042074.1 Eubacterium pyruvativorans strain I-6 16S ribosomal RNA, partial sequence | NR_042074.1 | 1296 | 264 | 667 | 31.17 | 453.5 | 3E-127 | 354/407 | 86 | 5/407 | 1 | Plus | Plus | [illegible] |
| ASV5734 | 427 | 1 | 1 | 427 | 100 | NR_027596.1 Mitsuokella multacida strain DSM 20544 16S ribosomal RNA, partial sequence | NR_027596.1 | 1511 | 346 | 772 | 28.26 | 603.1 | 3E-172 | 393/427 | 92 | 0/427 | 0 | Plus | Plus | [illegible] |
| ASV5736 | 427 | 1 | 1 | 426 | 99.77 | NR_044111.1 Selenomonas bovis strain WG 16S ribosomal RNA, partial sequence | NR_044111.1 | 1525 | 370 | 795 | 27.93 | 610.5 | 2E-174 | 396/428 | 92 | 4/428 | 0 | Plus | Plus | [illegible] |
| ASV5743 | 428 | 5 | 1 | 413 | 96.5 | NR_042052.1 Streptococcus equinus ATCC 9812 16S ribosomal RNA, partial sequence | NR_042052.1 | 1433 | 260 | 671 | 28.75 | 518.2 | 1E-146 | 369/413 | 89 | 1/413 | 0 | Plus | Plus | [illegible] |
| ASV5744 | 402 | 2 | 1 | 401 | 99.75 | NR_114807.1 Marvinbryantia formatexigens strain I-52 16S ribosomal RNA, partial sequence | NR_114807.1 | 1456 | 336 | 736 | 27.54 | 592 | 7E-169 | 374/401 | 93 | 0/401 | 0 | Plus | Plus | [illegible] |
| ASV5747 | 403 | 1 | 1 | 403 | 100 | NR_044054.1 Blautia wexlerae DSM 19850 16S ribosomal RNA, partial sequence | NR_044054.1 | 1438 | 314 | 715 | 27.96 | 599.4 | 4E-171 | 377/403 | 93 | 1/403 | 0 | Plus | Plus | [illegible] |
| ASV5754 | 403 | 3 | 1 | 403 | 100 | NR_102980.1 Megasphaera elsdenii DSM 20460 16S ribosomal RNA, partial sequence | NR_102980.1 | 1552 | 369 | 795 | 27.51 | 579.1 | 6E-165 | 394/428 | 92 | 26/428 | 6 | Plus | Plus | [illegible] |
| ASV5755 | 427 | 2 | 4 | 427 | 99.3 | NR_113306.1 Megasphaera elsdenii DSM 20460 16S ribosomal RNA, partial sequence | NR_113306.1 | 1174 | 235 | 658 | 36.12 | 540.3 | 3E-153 | 381/425 | 89 | 2/425 | 0 | Plus | Plus | [illegible] |
| ASV5756 | 428 | 1 | 3 | 428 | 99.53 | NR_126243.1 Lactobacillus delbrueckii subsp. jakobsenii strain ZN7a-9 16S ribosomal RNA, partial sequence | NR_126243.1 | 1382 | 271 | 696 | 30.82 | 494.2 | 2E-139 | 376/429 | 87 | 6/429 | 1 | Plus | Plus | [illegible] |
| ASV5758 | 402 | 1 | 1 | 402 | 100 | NR_144740.1 Romboutsia timonensis strain DR1 16S ribosomal RNA, partial sequence | NR_144740.1 | 1466 | 338 | 738 | 27.35 | 475.7 | 7E-134 | 355/403 | 88 | 3/403 | 0 | Plus | Plus | [illegible] |
| ASV5759 | 427 | 5 | 1 | 427 | 100 | NR_042111.1 Lactobacillus gallinarum strain ATCC 33199 16S ribosomal RNA, partial sequence | NR_042111.1 | 1546 | 362 | 788 | 27.62 | 501.6 | 1E-141 | 378/430 | 87 | 6/430 | 1 | Plus | Plus | [illegible] |
| ASV5762 | 404 | 1 | 1 | 389 | 96.29 | NR_147371.1 Intestinibacillus massiliensis strain Marseille-P3216 16S ribosomal RNA, partial sequence | NR_147371.1 | 1424 | 294 | 681 | 27.25 | 446.2 | 6E-125 | 343/392 | 87 | 7/392 | 1 | Plus | Plus | [illegible] |
| ASV5764 | 404 | 1 | 15 | 404 | 96.53 | NR_104846.1 Gemmiger formicilis strain X2-56 16S ribosomal RNA, partial sequence | NR_104846.1 | 1426 | 319 | 706 | 27.21 | 581 | 2E-165 | 368/393 | 93 | 8/393 | 2 | Plus | Plus | [illegible] |
| ASV5765 | 403 | 1 | 3 | 403 | 99.5 | NR_144749.1 Ihubacter massiliensis strain Marseille-P2843 16S ribosomal RNA, partial sequence | NR_144749.1 | 1479 | 366 | 766 | 27.11 | 492.3 | 7E-139 | 356/401 | 88 | 0/401 | 0 | Plus | Plus | [illegible] |
| ASV5766 | 402 | 1 | 3 | 402 | 99.5 | NR_044490.1 Butyricicoccus pullicaecorum strain 25-3 16S ribosomal RNA, partial sequence | NR_044490.1 | 1482 | 368 | 770 | 27.19 | 481.2 | 2E-135 | 360/407 | 88 | 11/407 | 2 | Plus | Plus | [illegible] |
| ASV5769 | 402 | 2 | 1 | 402 | 100 | NR_074793.2 Oscillibacter valericigenes strain Sjm18-20 16S ribosomal RNA, complete sequence | NR_074793.2 | 1530 | 372 | 775 | 26.41 | 507.1 | 3E-143 | 362/405 | 89 | 4/405 | 0 | Plus | Plus | [illegible] |
| ASV5770 | 404 | 1 | 1 | 404 | 100 | NR_026312.1 Blautia schinkii strain B 16S ribosomal RNA, partial sequence | NR_026312.1 | 1482 | 344 | 745 | 27.13 | 564.3 | 2E-160 | 372/405 | 91 | 4/405 | 0 | Plus | Plus | [illegible] |
| ASV5771 | 427 | 1 | 1 | 420 | 98.36 | NR_044731.2 Holdemanella biformis strain DSM 3989 16S ribosomal RNA, partial sequence | NR_044731.2 | 1487 | 364 | 782 | 28.18 | 516.3 | 5E-146 | 373/420 | 88 | 1/420 | 0 | Plus | Plus | [illegible] |
| ASV5772 | 422 | 2 | 1 | 422 | 100 | NR_113065.1 Bacteroides clarus YIT 12056 strain JCM 16067 16S ribosomal RNA, partial sequence | NR_113065.1 | 1488 | 355 | 776 | 28.36 | 459.1 | 8E-129 | 368/426 | 86 | 8/426 | 1 | Plus | Plus | [illegible] |
| ASV5775 | 426 | 1 | 1 | 426 | 100 | NR_029335.1 Holdemania filiformis strain J1-31B-1 16S ribosomal RNA, partial sequence | NR_029335.1 | 1391 | 267 | 692 | 30.63 | 455.4 | 1E-127 | 367/427 | 85 | 2/427 | 0 | Plus | Plus | [illegible] |
| ASV5781 | 402 | 1 | 3 | 402 | 99.5 | NR_144611.1 Flintibacter butyricus strain BLS21 16S ribosomal RNA, partial sequence | NR_144611.1 | 808 | 301 | 703 | 49.88 | 536.6 | 3E-152 | 367/404 | 90 | 5/404 | 1 | Plus | Plus | [illegible] |
| ASV5785 | 403 | 1 | 3 | 403 | 99.5 | NR_117506.1 Bifidobacterium longum strain KCTC 3128 16S ribosomal RNA, partial sequence | NR_117506.1 | 1477 | 330 | 734 | 27.42 | 568 | 1E-161 | 373/405 | 92 | 4/405 | 0 | Plus | Plus | [illegible] |
| ASV5787 | 402 | 5 | 1 | 402 | 100 | NR_171498.1 Clostridium fermenticellae strain JN500901 16S ribosomal RNA, partial sequence | NR_171498.1 | 1382 | 302 | 703 | 29.09 | 505.3 | 9E-143 | 361/404 | 89 | 4/404 | 0 | Plus | Plus | [illegible] |
| ASV5788 | 404 | 1 | 3 | 399 | 98.27 | NR_027526.1 Catenibacterium mitsuokai strain DSM 15897 16S ribosomal RNA, partial sequence | NR_027526.1 | 1485 | 347 | 763 | 28.08 | 448 | 2E-125 | 364/419 | 86 | 24/419 | 5 | Plus | Plus | [illegible] |
| ASV5791 | 422 | 1 | 1 | 422 | 100 | NR_126243.1 Lactobacillus delbrueckii subsp. jakobsenii strain ZN7a-9 16S ribosomal RNA, partial sequence | NR_126243.1 | 1382 | 269 | 696 | 30.97 | 581 | 2E-165 | 392/429 | 91 | 8/429 | 1 | Plus | Plus | [illegible] |
| ASV5794 | 402 | 3 | 1 | 402 | 100 | NR_114326.1 Fusicatenibacter saccharivorans strain HT03-11 16S ribosomal RNA, partial sequence | NR_114326.1 | 1423 | 351 | 752 | 28.25 | 505.3 | 9E-143 | 359/402 | 89 | 0/402 | 0 | Plus | Plus | [illegible] |
| ASV5795 | 404 | 1 | 1 | 404 | 100 | NR_118156.1 Oscillibacter ruminantium GH1 16S ribosomal RNA, partial sequence | NR_118156.1 | 1409 | 321 | 724 | 28.67 | 508.9 | 7E-144 | 365/408 | 89 | 8/408 | 1 | Plus | Plus | [illegible] |
| ASV5797 | 402 | 1 | 1 | 402 | 100 | NR_028961.1 Faecalibacterium prausnitzii strain ATCC 27768 16S ribosomal RNA, partial sequence | NR_028961.1 | 1462 | 344 | 745 | 27.5 | 621.6 | 9E-178 | 380/402 | 94 | 0/402 | 0 | Plus | Plus | [illegible] |
| ASV5798 | 420 | 3 | 3 | 420 | 99.52 | NR_113355.1 Dialister invisus strain JCM 17566 16S ribosomal RNA, partial sequence | NR_113355.1 | 1530 | 375 | 799 | 27.78 | 523.7 | 3E-148 | 380/426 | 89 | 9/426 | 2 | Plus | Plus | [illegible] |
| ASV5802 | 423 | 1 | 1 | 423 | 100 | NR_144750.1 Prevotellamassilia timonensis strain Marseille-P2831 16S ribosomal RNA, partial sequence | NR_144750.1 | 1493 | 358 | 779 | 28.27 | 459.1 | 8E-129 | 368/426 | 86 | 7/426 | 1 | Plus | Plus | [illegible] |
| ASV5803 | 427 | 3 | 1 | 425 | 99.53 | NR_041781.1 Streptococcus alactolyticus strain ATCC 43077 16S ribosomal RNA, partial sequence | NR_041781.1 | 1437 | 346 | 770 | 29.58 | 719.5 | 0 | 414/426 | 97 | 2/426 | 0 | Plus | Plus | [illegible] |
| ASV5806 | 405 | 2 | 1 | 405 | 100 | NG_041947.1 Ruminiclostridium cellulolyticum H10 16S ribosomal RNA gene, complete sequence | NG_041947.1 | 1641 | 486 | 890 | 24.68 | 521.9 | 9E-148 | 364/405 | 89 | 0/405 | 0 | Plus | Plus | [illegible] |
| ASV5809 | 426 | 1 | 5 | 426 | 99.06 | NR_029325.1 Schwartzia succinivorans strain S1-1 16S ribosomal RNA, partial sequence | NR_029325.1 | 1489 | 348 | 769 | 28.34 | 470.2 | 4E-132 | 375/431 | 87 | 18/431 | 4 | Plus | Plus | [illegible] |
| ASV5815 | 428 | 1 | 1 | 428 | 100 | NR_028833.1 Streptococcus sinensis strain HKU4 16S ribosomal RNA, partial sequence | NR_028833.1 | 1512 | 364 | 790 | 28.24 | 435.1 | 1E-121 | 369/433 | 85 | 11/433 | 2 | Plus | Plus | [illegible] |
| ASV5817 | 402 | 1 | 1 | 402 | 100 | NR_044503.1 Thermotalea metallivorans strain B2-1 16S ribosomal RNA, partial sequence | NR_044503.1 | 1627 | 467 | 868 | 24.71 | 477.6 | 2E-134 | 358/406 | 88 | 8/406 | 1 | Plus | Plus | [illegible] |
| ASV5825 | 422 | 1 | 1 | 422 | 100 | NR_113411.1 Prevotella copri DSM 18205 strain JCM 13464 16S ribosomal RNA, partial sequence | NR_113411.1 | 1493 | 358 | 779 | 28.27 | 675.1 | 0 | 404/423 | 95 | 2/423 | 0 | Plus | Plus | [illegible] |
| ASV5826 | 409 | 1 | 1 | 409 | 100 | NR_042832.1 Roseburia faecis strain M72/1 16S ribosomal RNA, partial sequence | NR_042832.1 | 1456 | 347 | 749 | 27.68 | 584.7 | 1E-166 | 379/409 | 92 | 6/409 | 1 | Plus | Plus | [illegible] |
| ASV5829 | 402 | 1 | 3 | 402 | 99.5 | NR_044490.1 Butyricicoccus pullicaecorum strain 25-3 16S ribosomal RNA, partial sequence | NR_044490.1 | 1482 | 368 | 770 | 27.19 | 514.5 | 2E-145 | 363/404 | 89 | 5/404 | 1 | Plus | Plus | [illegible] |
| ASV5831 | 403 | 1 | 3 | 403 | 99.5 | NR_125464.1 Anaerobacterium chartisolvens strain T-1-35 16S ribosomal RNA, partial sequence | NR_125464.1 | 1451 | 344 | 745 | 27.71 | 459.1 | 7E-129 | 351/402 | 87 | 1/402 | 0 | Plus | Plus | [illegible] |
| ASV5835 | 402 | 1 | 1 | 400 | 99.5 | NR_116747.1 Ruminococcus faecis JCM 15917 strain Eg2 16S ribosomal RNA, partial sequence | NR_116747.1 | 1404 | 318 | 717 | 28.49 | 584.7 | 1E-166 | 372/400 | 93 | 0/400 | 0 | Plus | Plus | [illegible] |
| ASV5836 | 427 | 4 | 1 | 426 | 99.77 | NR_041781.1 Streptococcus alactolyticus strain ATCC 43077 16S ribosomal RNA, partial sequence | NR_041781.1 | 1437 | 346 | 771 | 29.65 | 516.3 | 5E-146 | 381/430 | 88 | 8/430 | 1 | Plus | Plus | [illegible] |
| ASV5842 | 402 | 2 | 1 | 402 | 100 | NR_113231.1 Blautia glucerasea strain JCM 17039 16S ribosomal RNA, partial sequence | NR_113231.1 | 1491 | 364 | 765 | 26.96 | 538.5 | 9E-153 | 366/403 | 90 | 2/403 | 0 | Plus | Plus | [illegible] |
| ASV5845 | 422 | 1 | 1 | 422 | 100 | NR_144747.1 Mediterranea massiliensis strain Marseille-P2645 16S ribosomal RNA, partial sequence | NR_144747.1 | 1491 | 354 | 775 | 28.3 | 702.8 | 0 | 408/422 | 96 | 0/422 | 0 | Plus | Plus | [illegible] |
| ASV5849 | 403 | 1 | 3 | 403 | 99.5 | NR_026104.1 Ruminiclostridium cellobioparum DSM 1351 = ATCC 15832 16S ribosomal RNA, partial sequence | NR_026104.1 | 1487 | 331 | 733 | 27.1 | 460.9 | 2E-129 | 353/404 | 87 | 4/404 | 0 | Plus | Plus | [illegible] |
| ASV5852 | 422 | 5 | 1 | 422 | 100 | NR_044864.1 Hallella seregens ATCC 51272 16S ribosomal RNA, partial sequence | NR_044864.1 | 1463 | 335 | 756 | 28.84 | 453.5 | 4E-127 | 368/427 | 86 | 10/427 | 2 | Plus | Plus | [illegible] |
| ASV5853 | 422 | 1 | 1 | 413 | 97.87 | NR_040877.1 Prevotella copri DSM 18205 strain CB7 16S ribosomal RNA, partial sequence | NR_040877.1 | 1494 | 358 | 770 | 27.64 | 582.8 | 4E-166 | 380/413 | 92 | 0/413 | 0 | Plus | Plus | [illegible] |
| ASV5854 | 422 | 1 | 1 | 413 | 97.87 | NR_113411.1 Prevotella copri DSM 18205 strain JCM 13464 16S ribosomal RNA, partial sequence | NR_113411.1 | 1493 | 358 | 770 | 27.66 | 636.4 | 0 | 390/413 | 94 | 0/413 | 0 | Plus | Plus | [illegible] |
| ASV5855 | 403 | 5 | 3 | 403 | 99.5 | NR_074399.1 Ruminococcus albus 7 = DSM 20455 16S ribosomal RNA, partial sequence | NR_074399.1 | 1500 | 352 | 751 | 26.67 | 490.5 | 3E-138 | 356/401 | 88 | 1/401 | 0 | Plus | Plus | [illegible] |
| ASV5856 | 405 | 1 | 4 | 405 | 99.26 | NR_026476.1 Succinivibrio dextrinosolvens strain 0554 16S ribosomal RNA, partial sequence | NR_026476.1 | 1463 | 334 | 732 | 27.27 | 501.6 | 1E-141 | 360/403 | 89 | 5/403 | 1 | Plus | Plus | [illegible] |
| ASV5857 | 402 | 2 | 1 | 402 | 100 | NR_024919.1 Clostridium frigidicarnis strain SPL77A 16S ribosomal RNA, partial sequence | NR_024919.1 | 1481 | 346 | 747 | 27.14 | 510.8 | 2E-144 | 361/403 | 89 | 2/403 | 0 | Plus | Plus | [illegible] |
| ASV5859 | 426 | 4 | 29 | 426 | 93.43 | NR_042802.1 Lactobacillus ultunensis strain Kx146C1 16S ribosomal RNA, partial sequence | NR_042802.1 | 1550 | 398 | 796 | 25.74 | 514.5 | 2E-145 | 361/401 | 90 | 5/401 | 1 | Plus | Plus | [illegible] |
| ASV5860 | 403 | 3 | 1 | 403 | 100 | NR_029106.1 Lactobacillus delbrueckii subsp. indicus strain NCC725 16S ribosomal RNA, partial sequence | NR_029106.1 | 1515 | 348 | 774 | 28.18 | 545.9 | 6E-155 | 390/430 | 90 | 30/430 | 6 | Plus | Plus | [illegible] |
| ASV5861 | 402 | 1 | 3 | 402 | 99.5 | NR_042007.1 Roseburia inulinivorans DSM 16841 strain A2-194 16S ribosomal RNA, partial sequence | NR_042007.1 | 1454 | 362 | 763 | 27.65 | 464.6 | 2E-130 | 352/402 | 87 | 2/402 | 0 | Plus | Plus | [illegible] |
| ASV5862 | 419 | 4 | 1 | 419 | 100 | NR_118511.1 Campylobacter coli strain ATCC 33559 16S ribosomal RNA, partial sequence | NR_118511.1 | 1435 | 329 | 731 | 28.08 | 533 | 5E-151 | 379/420 | 90 | 18/420 | 4 | Plus | Plus | [illegible] |
| ASV5863 | 422 | 1 | 1 | 422 | 100 | NR_027196.1 Bacteroides stercoris ATCC 43183 16S ribosomal RNA, partial sequence | NR_027196.1 | 1342 | 334 | 755 | 31.45 | 588.4 | 1E-167 | 387/422 | 91 | 0/422 | 0 | Plus | Plus | [illegible] |
| ASV5864 | 402 | 1 | 3 | 402 | 99.5 | NR_133689.1 Aestuariispira insulae strain AH-MY2 16S ribosomal RNA, partial sequence | NR_133689.1 | 1417 | 311 | 710 | 28.23 | 496 | 6E-140 | 360/404 | 89 | 8/404 | 1 | Plus | Plus | [illegible] |
| ASV5874 | 403 | 2 | 1 | 403 | 100 | NR_144696.1 Clostridium saudiense strain JCC 16S ribosomal RNA, partial sequence | NR_144696.1 | 1478 | 350 | 751 | 27.2 | 588.4 | 9E-168 | 377/405 | 93 | 5/405 | 1 | Plus | Plus | [illegible] |
| ASV5875 | 404 | 1 | 1 | 404 | 100 | NR_027573.1 Intestinibacter bartlettii strain WAL 16138 16S ribosomal RNA, partial sequence | NR_027573.1 | 1429 | 320 | 720 | 28.06 | 544 | 2E-154 | 368/404 | 91 | 3/404 | 0 | Plus | Plus | [illegible] |
| ASV5876 | 404 | 1 | 1 | 404 | 100 | NR_025931.1 Ruminococcus flavefaciens strain C94 16S ribosomal RNA, partial sequence | NR_025931.1 | 1388 | 325 | 726 | 28.96 | 606.8 | 3E-173 | 379/404 | 93 | 2/404 | 0 | Plus | Plus | [illegible] |
| ASV5877 | 405 | 1 | 1 | 404 | 99.75 | NR_118554.1 Intestinimonas butyriciproducens strain SRB-521-5-I 16S ribosomal RNA, partial sequence | NR_118554.1 | 1475 | 346 | 749 | 27.39 | 514.5 | 2E-145 | 362/404 | 89 | 0/404 | 0 | Plus | Plus | [illegible] |
| ASV5881 | 404 | 2 | 1 | 404 | 100 | NR_074793.2 Oscillibacter valericigenes strain Sjm18-20 16S ribosomal RNA, complete sequence | NR_074793.2 | 1530 | 372 | 775 | 26.41 | 542.2 | 7E-154 | 369/406 | 90 | 4/406 | 0 | Plus | Plus | [illegible] |
| ASV5882 | 421 | 2 | 1 | 421 | 100 | NR_113077.1 Paraprevotella clara strain JCM 14859 16S ribosomal RNA, partial sequence | NR_113077.1 | 1480 | 346 | 766 | 28.45 | 473.9 | 3E-133 | 367/422 | 86 | 2/422 | 0 | Plus | Plus | [illegible] |
| ASV5889 | 426 | 1 | 1 | 426 | 100 | NR_114696.1 Anaerovibrio lipolyticus DSM 3074 16S ribosomal RNA, partial sequence | NR_114696.1 | 1526 | 372 | 797 | 27.92 | 520 | 4E-147 | 377/426 | 88 | 0/426 | 0 | Plus | Plus | [illegible] |
| ASV5890 | 422 | 1 | 1 | 422 | 100 | NR_136443.1 Porphyromonas pogonae strain MI 10-1288 16S ribosomal RNA, partial sequence | NR_136443.1 | 1429 | 317 | 739 | 29.6 | 459.1 | 8E-129 | 369/427 | 86 | 9/427 | 2 | Plus | Plus | [illegible] |
| ASV5893 | 405 | 1 | 1 | 405 | 100 | NR_041778.1 Desulfovibrio piger strain ATCC 29098 16S ribosomal RNA, partial sequence | NR_041778.1 | 1542 | 357 | 783 | 27.69 | 525.6 | 7E-149 | 383/427 | 89 | 22/427 | 5 | Plus | Plus | [illegible] |
| ASV5894 | 402 | 1 | 1 | 385 | 95.77 | NR_165739.1 Lachnoanaerobaculum gingivalis strain ChDC B114 16S ribosomal RNA, partial sequence | NR_165739.1 | 1454 | 345 | 729 | 26.48 | 523.7 | 3E-148 | 352/386 | 91 | 2/386 | 0 | Plus | Plus | [illegible] |
| ASV5896 | 403 | 1 | 1 | 403 | 100 | NR_104907.1 Eubacterium coprostanoligenes strain HL 16S ribosomal RNA, partial sequence | NR_104907.1 | 1446 | 332 | 734 | 27.87 | 573.6 | 3E-163 | 373/404 | 92 | 2/404 | 0 | Plus | Plus | [illegible] |
| ASV5905 | 427 | 1 | 1 | 427 | 100 | NR_044362.1 Haloplasma contractile SSD-17B 16S ribosomal RNA, partial sequence | NR_044362.1 | 1345 | 322 | 748 | 31.75 | 496 | 6E-140 | 375/428 | 87 | 2/428 | 0 | Plus | Plus | [illegible] |
| ASV5908 | 404 | 1 | 1 | 404 | 100 | NR_025670.1 Pseudoflavonifractor capillosus ATCC 29799 16S ribosomal RNA, partial sequence | NR_025670.1 | 1483 | 346 | 750 | 27.31 | 525.6 | 7E-149 | 365/405 | 90 | 1/405 | 0 | Plus | Plus | [illegible] |
| ASV5909 | 405 | 2 | 1 | 404 | 99.75 | NR_144743.1 Christensenella timonensis strain Marseille-P2437 16S ribosomal RNA, partial sequence | NR_144743.1 | 1498 | 369 | 772 | 26.97 | 481.2 | 2E-135 | 356/404 | 88 | 0/404 | 0 | Plus | Plus | [illegible] |
| ASV5912 | 422 | 1 | 1 | 422 | 100 | NR_113411.1 Prevotella copri DSM 18205 strain JCM 13464 16S ribosomal RNA, partial sequence | NR_113411.1 | 1493 | 358 | 779 | 28.27 | 592 | 7E-169 | 388/422 | 91 | 0/422 | 0 | Plus | Plus | [illegible] |
| ASV5917 | 405 | 1 | 1 | 405 | 100 | NR_044054.1 Blautia wexlerae DSM 19850 16S ribosomal RNA, partial sequence | NR_044054.1 | 1438 | 314 | 715 | 27.96 | 617.9 | 1E-176 | 383/406 | 94 | 5/406 | 1 | Plus | Plus | [illegible] |
| ASV5918 | 406 | 1 | 1 | 406 | 100 | NR_117133.2 Clostridium oceanicum strain DSM 1290 16S ribosomal RNA, partial sequence | NR_117133.2 | 1504 | 347 | 748 | 26.73 | 414.8 | 2E-115 | 350/410 | 85 | 12/410 | 2 | Plus | Plus | [illegible] |
| ASV5920 | 402 | 1 | 1 | 402 | 100 | NR_029239.3 Clostridium chartatabidum strain 163 16S ribosomal RNA, partial sequence | NR_029239.3 | 1460 | 323 | 724 | 27.53 | 599.4 | 4E-171 | 376/402 | 93 | 0/402 | 0 | Plus | Plus | [illegible] |
| ASV5922 | 402 | 2 | 1 | 402 | 100 | NR_156911.1 Fournierella massiliensis strain AT2 16S ribosomal RNA, partial sequence | NR_156911.1 | 1491 | 368 | 769 | 26.96 | 494.2 | 2E-139 | 361/406 | 88 | 8/406 | 1 | Plus | Plus | [illegible] |
| ASV5924 | 405 | 5 | 3 | 405 | 99.51 | NR_117768.1 Lactobacillus delbrueckii strain DSM 20074 16S ribosomal RNA, partial sequence | NR_117768.1 | 1365 | 330 | 754 | 31.14 | 527.4 | 2E-149 | 382/425 | 89 | 22/425 | 5 | Plus | Plus | [illegible] |
| ASV5926 | 422 | 2 | 1 | 422 | 100 | NR_116726.1 Prevotella scopos JCM 17725 strain W2052 16S ribosomal RNA, partial sequence | NR_116726.1 | 1453 | 338 | 759 | 29.04 | 575.4 | 7E-164 | 385/422 | 91 | 0/422 | 0 | Plus | Plus | [illegible] |
| ASV5930 | 404 | 1 | 3 | 404 | 99.5 | NR_118156.1 Oscillibacter ruminantium GH1 16S ribosomal RNA, partial sequence | NR_118156.1 | 1409 | 323 | 724 | 28.53 | 560.7 | 2E-159 | 371/404 | 91 | 4/404 | 0 | Plus | Plus | [illegible] |
| ASV5938 | 426 | 2 | 5 | 426 | 99.06 | NR_029207.1 Megasphaera elsdenii strain LC 1 16S ribosomal RNA, partial sequence | NR_029207.1 | 1426 | 378 | 792 | 29.59 | 486.8 | 4E-137 | 374/427 | 87 | 10/427 | 2 | Plus | Plus | [illegible] |
| ASV5940 | 403 | 2 | 3 | 403 | 99.5 | NR_024948.1 Campylobacter hyointestinalis subsp. lawsonii strain CHY5 16S ribosomal RNA, partial sequence | NR_024948.1 | 1419 | 320 | 720 | 28.26 | 608.7 | 7E-174 | 377/401 | 94 | 0/401 | 0 | Plus | Plus | [illegible] |
| ASV5950 | 405 | 1 | 1 | 404 | 99.75 | NR_041236.1 Lutispora thermophila DSM 19022 strain EBR46 16S ribosomal RNA, partial sequence | NR_041236.1 | 1438 | 338 | 739 | 27.96 | 446.2 | 6E-125 | 352/406 | 86 | 6/406 | 1 | Plus | Plus | [illegible] |
| ASV5952 | 422 | 2 | 1 | 422 | 100 | NR_113073.1 Barnesiella intestinihominis strain JCM 15079 16S ribosomal RNA, partial sequence | NR_113073.1 | 1481 | 346 | 767 | 28.49 | 497.9 | 2E-140 | 375/426 | 88 | 8/426 | 1 | Plus | Plus | [illegible] |
| ASV5953 | 421 | 3 | 1 | 421 | 100 | NR_113123.1 Prevotella timonensis 4401737 = DSM 22865 = JCM 15640 16S ribosomal RNA, partial sequence | NR_113123.1 | 1493 | 356 | 777 | 28.27 | 440.6 | 3E-123 | 366/427 | 85 | 11/427 | 2 | Plus | Plus | [illegible] |
| ASV5954 | 422 | 1 | 1 | 422 | 100 | NR_113411.1 Prevotella copri DSM 18205 strain JCM 13464 16S ribosomal RNA, partial sequence | NR_113411.1 | 1493 | 358 | 779 | 28.27 | 520 | 4E-147 | 376/423 | 88 | 2/423 | 0 | Plus | Plus | [illegible] |
| ASV5959 | 423 | 1 | 1 | 423 | 100 | NR_144750.1 Prevotellamassilia timonensis strain Marseille-P2831 16S ribosomal RNA, partial sequence | NR_144750.1 | 1493 | 358 | 779 | 28.27 | 564.3 | 2E-160 | 385/424 | 90 | 3/424 | 0 | Plus | Plus | [illegible] |
| ASV5961 | 422 | 1 | 1 | 422 | 100 | NR_145844.1 Bacteroides caecigallinarum strain C13EG111 16S ribosomal RNA, partial sequence | NR_145844.1 | 1489 | 356 | 777 | 28.34 | 453.5 | 4E-127 | 364/423 | 86 | 2/423 | 0 | Plus | Plus | [illegible] |
| ASV5970 | 405 | 3 | 1 | 405 | 100 | NR_115307.1 Ethanoligenens harbinense YUAN-3 16S ribosomal RNA, partial sequence | NR_115307.1 | 1497 | 340 | 743 | 26.99 | 492.3 | 7E-139 | 361/407 | 88 | 5/407 | 1 | Plus | Plus | [illegible] |
| ASV5974 | 422 | 5 | 1 | 422 | 100 | NR_118334.1 Alloprevotella rava strain 81/4-12 16S ribosomal RNA, partial sequence | NR_118334.1 | 1455 | 338 | 759 | 29 | 492.3 | 8E-139 | 376/428 | 87 | 12/428 | 2 | Plus | Plus | [illegible] |
| ASV5976 | 402 | 1 | 1 | 402 | 100 | NR_042832.1 Roseburia faecis strain M72/1 16S ribosomal RNA, partial sequence | NR_042832.1 | 1456 | 347 | 749 | 27.68 | 593.9 | 2E-169 | 378/405 | 93 | 5/405 | 1 | Plus | Plus | [illegible] |
| ASV5978 | 403 | 1 | 1 | 403 | 100 | NR_144737.1 Emergencia timonensis strain SN18 16S ribosomal RNA, partial sequence | NR_144737.1 | 1480 | 364 | 767 | 27.3 | 512.6 | 6E-145 | 362/404 | 89 | 1/404 | 0 | Plus | Plus | [illegible] |
| ASV5980 | 421 | 1 | 1 | 413 | 97.87 | NR_113411.1 Prevotella copri DSM 18205 strain JCM 13464 16S ribosomal RNA, partial sequence | NR_113411.1 | 1493 | 358 | 779 | 28.27 | 518.2 | 1E-146 | 378/425 | 88 | 7/425 | 1 | Plus | Plus | [illegible] |
| ASV5981 | 404 | 1 | 1 | 404 | 100 | NR_074577.1 Paludibacter propionicigenes WB4 16S ribosomal RNA, partial sequence | NR_074577.1 | 1516 | 353 | 774 | 27.84 | 414.8 | 2E-115 | 359/422 | 85 | 18/422 | 4 | Plus | Plus | [illegible] |
| ASV5985 | 402 | 5 | 1 | 402 | 100 | NR_156081.1 Kineothrix alysoides strain KNHs209 16S ribosomal RNA, partial sequence | NR_156081.1 | 1411 | 353 | 754 | 28.49 | 527.4 | 2E-149 | 366/405 | 90 | 6/405 | 1 | Plus | Plus | [illegible] |
| ASV5986 | 401 | 5 | 1 | 401 | 100 | NR_119066.1 Paraclostridium bifermentans strain ATCC 638 16S ribosomal RNA, partial sequence | NR_119066.1 | 1451 | 329 | 727 | 27.5 | 520 | 3E-147 | 362/402 | 90 | 4/402 | 0 | Plus | Plus | [illegible] |
| ASV5987 | 405 | 1 | 1 | 405 | 100 | NR_042150.1 Fibrobacter intestinalis strain NR9 16S ribosomal RNA, partial sequence | NR_042150.1 | 1436 | 325 | 743 | 29.18 | 518.2 | 1E-146 | 376/420 | 89 | 16/420 | 3 | Plus | Plus | [illegible] |
| ASV5993 | 426 | 1 | 1 | 426 | 100 | NR_102980.1 Megasphaera elsdenii DSM 20460 16S ribosomal RNA, partial sequence | NR_102980.1 | 1552 | 369 | 795 | 27.51 | 605 | 1E-172 | 399/432 | 92 | 11/432 | 2 | Plus | Plus | [illegible] |
| ASV5995 | 402 | 1 | 5 | 402 | 99 | NR_115269.1 Catabacter hongkongensis strain HKU16 16S ribosomal RNA, partial sequence | NR_115269.1 | 1453 | 353 | 753 | 27.6 | 405.5 | 1E-112 | 346/406 | 85 | 13/406 | 3 | Plus | Plus | [illegible] |
| ASV5997 | 421 | 1 | 1 | 421 | 100 | NR_041342.1 Parabacteroides distasonis strain JCM 5825 16S ribosomal RNA, partial sequence | NR_041342.1 | 1488 | 353 | 774 | 28.36 | 606.8 | 3E-173 | 393/424 | 92 | 5/424 | 1 | Plus | Plus | [illegible] |
| ASV5998 | 405 | 1 | 1 | 404 | 99.75 | NR_118554.1 Intestinimonas butyriciproducens strain SRB-521-5-I 16S ribosomal RNA, partial sequence | NR_118554.1 | 1475 | 346 | 749 | 27.39 | 459.1 | 8E-129 | 353/405 | 87 | 2/405 | 0 | Plus | Plus | [illegible] |
| ASV6002 | 422 | 2 | 1 | 422 | 100 | NR_113067.1 Bacteroides faecis MAJ27 16S ribosomal RNA, partial sequence | NR_113067.1 | 1475 | 342 | 763 | 28.61 | 558.8 | 8E-159 | 387/427 | 90 | 10/427 | 2 | Plus | Plus | [illegible] |
| ASV6004 | 422 | 5 | 1 | 422 | 100 | NR_113119.1 Prevotella oris strain JCM 8540 16S ribosomal RNA, partial sequence | NR_113119.1 | 1481 | 345 | 766 | 28.49 | 520 | 4E-147 | 375/422 | 88 | 0/422 | 0 | Plus | Plus | [illegible] |
| ASV6005 | 402 | 1 | 1 | 402 | 100 | NR_148620.1 Lachnotalea glycerini strain : DLD10 16S ribosomal RNA, partial sequence | NR_148620.1 | 1207 | 212 | 613 | 33.31 | 505.3 | 9E-143 | 360/403 | 89 | 2/403 | 0 | Plus | Plus | [illegible] |
| ASV6006 | 402 | 1 | 3 | 402 | 99.5 | NR_147375.1 Colidextribacter massiliensis strain Marseille-P3083 16S ribosomal RNA, partial sequence | NR_147375.1 | 1487 | 366 | 765 | 26.9 | 612.4 | 5E-175 | 377/400 | 94 | 0/400 | 0 | Plus | Plus | [illegible] |
| ASV6008 | 427 | 1 | 1 | 427 | 100 | NR_044972.1 Sporobacter termitidis strain SYR 16S ribosomal RNA, partial sequence | NR_044972.1 | 1506 | 348 | 752 | 26.89 | 457.2 | 3E-128 | 370/427 | 86 | 22/427 | 5 | Plus | Plus | [illegible] |
| ASV6009 | 421 | 1 | 3 | 421 | 99.52 | NR_028840.1 Mitsuokella jalaludinii strain M9 16S ribosomal RNA, partial sequence | NR_028840.1 | 1543 | 364 | 788 | 27.54 | 603.1 | 3E-172 | 393/425 | 92 | 6/425 | 1 | Plus | Plus | [illegible] |
| ASV6010 | 402 | 1 | 1 | 402 | 100 | NR_144613.1 Irregularibacter muris strain 2PG-426-CC-4.2 16S ribosomal RNA, partial sequence | NR_144613.1 | 1431 | 347 | 751 | 28.3 | 457.2 | 3E-128 | 356/408 | 87 | 9/408 | 2 | Plus | Plus | [illegible] |
| ASV6022 | 421 | 1 | 1 | 421 | 100 | NR_041364.1 Prevotella stercorea DSM 18206 strain CB35 16S ribosomal RNA, partial sequence | NR_041364.1 | 1488 | 354 | 775 | 28.36 | 479.4 | 6E-135 | 371/425 | 87 | 7/425 | 1 | Plus | Plus | [illegible] |
| ASV6023 | 428 | 1 | 3 | 428 | 99.53 | NR_044362.1 Haloplasma contractile SSD-17B 16S ribosomal RNA, partial sequence | NR_044362.1 | 1345 | 324 | 748 | 31.6 | 492.3 | 8E-139 | 379/432 | 87 | 13/432 | 3 | Plus | Plus | [illegible] |
| ASV6025 | 427 | 1 | 1 | 427 | 100 | NR_041304.1 Sharpea azabuensis strain ST18 16S ribosomal RNA, partial sequence | NR_041304.1 | 1460 | 325 | 750 | 29.18 | 616.1 | 4E-176 | 399/430 | 92 | 7/430 | 1 | Plus | Plus | [illegible] |
| ASV6026 | 403 | 3 | 3 | 403 | 99.5 | NR_156081.1 Kineothrix alysoides strain KNHs209 16S ribosomal RNA, partial sequence | NR_156081.1 | 1411 | 355 | 754 | 28.35 | 545.9 | 6E-155 | 369/404 | 91 | 7/404 | 1 | Plus | Plus | [illegible] |
| ASV6031 | 405 | 1 | 1 | 405 | 100 | NR_144611.1 Flintibacter butyricus strain BLS21 16S ribosomal RNA, partial sequence | NR_144611.1 | 808 | 299 | 703 | 50.12 | 555.1 | 9E-158 | 370/405 | 91 | 0/405 | 0 | Plus | Plus | [illegible] |
| ASV6033 | 405 | 2 | 1 | 405 | 100 | NR_029356.1 Flavonifractor plautii strain 265 16S ribosomal RNA, partial sequence | NR_029356.1 | 1550 | 353 | 757 | 26.13 | 516.3 | 4E-146 | 370/412 | 89 | 14/412 | 3 | Plus | Plus | [illegible] |
| ASV6034 | 405 | 1 | 1 | 405 | 100 | NR_147370.1 Pseudoflavonifractor phocaeensis strain Marseille-P3064 16S ribosomal RNA, partial sequence | NR_147370.1 | 1495 | 366 | 770 | 27.09 | 571.7 | 9E-163 | 373/405 | 92 | 0/405 | 0 | Plus | Plus | [illegible] |
| ASV6035 | 422 | 2 | 1 | 422 | 100 | NR_113119.1 Prevotella oris strain JCM 8540 16S ribosomal RNA, partial sequence | NR_113119.1 | 1481 | 345 | 766 | 28.49 | 448 | 2E-125 | 365/425 | 85 | 6/425 | 1 | Plus | Plus | [illegible] |
| ASV6044 | 421 | 1 | 15 | 421 | 96.67 | NR_044111.1 Selenomonas bovis strain WG 16S ribosomal RNA, partial sequence | NR_044111.1 | 1525 | 384 | 796 | 27.08 | 475.7 | 8E-134 | 367/418 | 87 | 16/418 | 3 | Plus | Plus | [illegible] |
| ASV6048 | 422 | 4 | 1 | 422 | 100 | NR_043016.1 Bacteroides salyersiae strain WAL 10018 16S ribosomal RNA, partial sequence | NR_043016.1 | 1419 | 333 | 754 | 29.74 | 503.4 | 4E-142 | 374/424 | 88 | 4/424 | 0 | Plus | Plus | [illegible] |
| ASV6049 | 428 | 1 | 3 | 428 | 99.53 | NR_117574.1 Lactobacillus johnsonii strain CIP 103620 16S ribosomal RNA, partial sequence | NR_117574.1 | 1543 | 368 | 792 | 27.54 | 536.6 | 4E-152 | 385/430 | 89 | 9/430 | 2 | Plus | Plus | [illegible] |
| ASV6052 | 422 | 1 | 1 | 422 | 100 | NR_074546.1 Bacteroides helcogenes strain P 36-108 16S ribosomal RNA, partial sequence | NR_074546.1 | 1513 | 353 | 774 | 27.89 | 508.9 | 8E-144 | 374/423 | 88 | 2/423 | 0 | Plus | Plus | [illegible] |
| ASV6054 | 404 | 1 | 1 | 404 | 100 | NR_144616.1 Muribaculum intestinale strain YL27 16S ribosomal RNA, partial sequence | NR_144616.1 | 1460 | 297 | 718 | 28.9 | 448 | 2E-125 | 367/424 | 86 | 22/424 | 5 | Plus | Plus | [illegible] |
| ASV6058 | 403 | 1 | 1 | 403 | 100 | NR_134005.1 Clostridium bornimense strain M2/40 16S ribosomal RNA, partial sequence | NR_134005.1 | 1363 | 284 | 685 | 29.49 | 555.1 | 9E-158 | 369/403 | 91 | 1/403 | 0 | Plus | Plus | [illegible] |
| ASV6062 | 402 | 1 | 1 | 387 | 96.27 | NR_134772.1 Murimonas intestini strain SRB-530-5-H 16S ribosomal RNA, partial sequence | NR_134772.1 | 1433 | 314 | 700 | 27.01 | 577.3 | 2E-164 | 362/387 | 93 | 0/387 | 0 | Plus | Plus | [illegible] |
| ASV6065 | 422 | 2 | 1 | 422 | 100 | NR_040866.1 Bacteroides uniformis strain JCM 5828 16S ribosomal RNA, partial sequence | NR_040866.1 | 1425 | 351 | 772 | 29.61 | 514.5 | 2E-145 | 375/423 | 88 | 2/423 | 0 | Plus | Plus | [illegible] |
| ASV6066 | 428 | 4 | 1 | 428 | 100 | NR_042802.1 Lactobacillus ultunensis strain Kx146C1 16S ribosomal RNA, partial sequence | NR_042802.1 | 1550 | 370 | 796 | 27.55 | 590.2 | 3E-168 | 392/428 | 91 | 1/428 | 0 | Plus | Plus | [illegible] |
| ASV6074 | 422 | 1 | 1 | 422 | 100 | NR_118219.1 Alistipes senegalensis JC50 16S ribosomal RNA, partial sequence | NR_118219.1 | 1480 | 349 | 770 | 28.51 | 503.4 | 4E-142 | 374/424 | 88 | 4/424 | 0 | Plus | Plus | [illegible] |
| ASV6077 | 405 | 1 | 1 | 405 | 100 | NR_115340.1 Saccharofermentans acetigenes strain P6 16S ribosomal RNA, partial sequence | NR_115340.1 | 1510 | 353 | 757 | 26.82 | 422.2 | 1E-117 | 350/409 | 85 | 8/409 | 1 | Plus | Plus | [illegible] |
| ASV6078 | 402 | 1 | 1 | 402 | 100 | NR_108636.1 Natranaerovirga pectinivora strain AP3 16S ribosomal RNA, partial sequence | NR_108636.1 | 1497 | 365 | 766 | 26.85 | 577.3 | 2E-164 | 373/403 | 92 | 2/403 | 0 | Plus | Plus | [illegible] |
| ASV6079 | 428 | 2 | 15 | 428 | 96.73 | NR_113306.1 Megasphaera elsdenii DSM 20460 16S ribosomal RNA, partial sequence | NR_113306.1 | 1174 | 246 | 658 | 35.18 | 569.9 | 4E-162 | 379/414 | 91 | 1/414 | 0 | Plus | Plus | [illegible] |
| ASV6081 | 404 | 1 | 1 | 404 | 100 | NR_026204.1 [Clostridium] viride strain T2-7 16S ribosomal RNA, partial sequence | NR_026204.1 | 1482 | 345 | 749 | 27.33 | 477.6 | 2E-134 | 356/405 | 87 | 1/405 | 0 | Plus | Plus | [illegible] |
| ASV6088 | 405 | 3 | 1 | 405 | 100 | NR_117131.1 Faecalicatena orotica strain DSM 1287 16S ribosomal RNA, partial sequence | NR_117131.1 | 1526 | 365 | 766 | 26.34 | 479.4 | 6E-135 | 357/405 | 88 | 3/405 | 0 | Plus | Plus | [illegible] |
| ASV6092 | 402 | 1 | 1 | 402 | 100 | NR_041236.1 Lutispora thermophila DSM 19022 strain EBR46 16S ribosomal RNA, partial sequence | NR_041236.1 | 1438 | 338 | 740 | 28.03 | 505.3 | 9E-143 | 362/405 | 89 | 5/405 | 1 | Plus | Plus | [illegible] |
| ASV6095 | 404 | 1 | 1 | 403 | 99.75 | NR_125464.1 Anaerobacterium chartisolvens strain T-1-35 16S ribosomal RNA, partial sequence | NR_125464.1 | 1451 | 342 | 744 | 27.77 | 468.3 | 1E-131 | 356/406 | 87 | 6/406 | 1 | Plus | Plus | [illegible] |
| ASV6096 | 402 | 2 | 1 | 402 | 100 | NR_113360.1 Ruminiclostridium cellobioparum DSM 1351 = ATCC 15832 strain JCM 1422 16S ribosomal RNA, partial sequence | NR_113360.1 | 1484 | 356 | 760 | 27.29 | 501.6 | 1E-141 | 362/406 | 89 | 5/406 | 1 | Plus | Plus | [illegible] |
| ASV6098 | 421 | 2 | 1 | 421 | 100 | NR_113073.1 Barnesiella intestinihominis strain JCM 15079 16S ribosomal RNA, partial sequence | NR_113073.1 | 1481 | 346 | 767 | 28.49 | 457.2 | 3E-128 | 372/430 | 86 | 17/430 | 3 | Plus | Plus | [illegible] |
| ASV6100 | 403 | 3 | 1 | 403 | 100 | NR_102980.1 Megasphaera elsdenii DSM 20460 16S ribosomal RNA, partial sequence | NR_102980.1 | 1552 | 369 | 795 | 27.51 | 545.9 | 6E-155 | 387/427 | 90 | 24/427 | 5 | Plus | Plus | [illegible] |
| ASV6104 | 403 | 1 | 3 | 403 | 99.5 | NR_026102.1 Ruminiclostridium papyrosolvens DSM 2782 16S ribosomal RNA, partial sequence | NR_026102.1 | 1491 | 332 | 733 | 26.96 | 414.8 | 2E-115 | 350/409 | 85 | 15/409 | 3 | Plus | Plus | [illegible] |
| ASV6105 | 404 | 3 | 3 | 404 | 99.5 | NR_156081.1 Kineothrix alysoides strain KNHs209 16S ribosomal RNA, partial sequence | NR_156081.1 | 1411 | 355 | 754 | 28.35 | 569.9 | 3E-162 | 371/402 | 92 | 2/402 | 0 | Plus | Plus | [illegible] |
| ASV6106 | 427 | 1 | 1 | 426 | 99.77 | NR_157635.1 Megasphaera hexanoica strain MH 16S ribosomal RNA, partial sequence | NR_157635.1 | 1561 | 372 | 797 | 27.29 | 660.4 | 0 | 403/426 | 94 | 0/426 | 0 | Plus | Plus | [illegible] |
| ASV6107 | 420 | 3 | 1 | 420 | 100 | NR_102980.1 Megasphaera elsdenii DSM 20460 16S ribosomal RNA, partial sequence | NR_102980.1 | 1552 | 369 | 795 | 27.51 | 538.5 | 1E-152 | 387/431 | 89 | 15/431 | 3 | Plus | Plus | [illegible] |
| ASV6108 | 421 | 3 | 1 | 421 | 100 | NR_144616.1 Muribaculum intestinale strain YL27 16S ribosomal RNA, partial sequence | NR_144616.1 | 1460 | 297 | 718 | 28.9 | 435.1 | 1E-121 | 364/426 | 85 | 9/426 | 2 | Plus | Plus | [illegible] |
| ASV6111 | 403 | 1 | 3 | 403 | 99.5 | NR_028883.1 Dorea longicatena strain 111-35 16S ribosomal RNA, partial sequence | NR_028883.1 | 1316 | 245 | 645 | 30.47 | 558.8 | 7E-159 | 371/404 | 91 | 6/404 | 1 | Plus | Plus | [illegible] |
| ASV6118 | 426 | 1 | 1 | 426 | 100 | NR_044111.1 Selenomonas bovis strain WG 16S ribosomal RNA, partial sequence | NR_044111.1 | 1525 | 370 | 796 | 28 | 538.5 | 1E-152 | 383/428 | 89 | 3/428 | 0 | Plus | Plus | [illegible] |
| ASV6125 | 405 | 1 | 1 | 405 | 100 | NR_118554.1 Intestinimonas butyriciproducens strain SRB-521-5-I 16S ribosomal RNA, partial sequence | NR_118554.1 | 1475 | 346 | 750 | 27.46 | 483.1 | 4E-136 | 358/406 | 88 | 2/406 | 0 | Plus | Plus | [illegible] |
| ASV6127 | 426 | 3 | 1 | 426 | 100 | NR_119170.1 Veillonella atypica strain KON 16S ribosomal RNA, partial sequence | NR_119170.1 | 1513 | 348 | 774 | 28.22 | 446.2 | 6E-125 | 367/429 | 85 | 5/429 | 1 | Plus | Plus | [illegible] |
| ASV6131 | 402 | 2 | 1 | 402 | 100 | NR_117867.1 Blautia stercoris strain GAM6-1 16S ribosomal RNA, partial sequence | NR_117867.1 | 1372 | 317 | 718 | 29.3 | 538.5 | 9E-153 | 366/403 | 90 | 2/403 | 0 | Plus | Plus | [illegible] |
| ASV6132 | 402 | 1 | 1 | 402 | 100 | NR_147375.1 Colidextribacter massiliensis strain Marseille-P3083 16S ribosomal RNA, partial sequence | NR_147375.1 | 1487 | 364 | 765 | 27.03 | 555.1 | 9E-158 | 368/402 | 91 | 0/402 | 0 | Plus | Plus | [illegible] |
| ASV6133 | 404 | 4 | 4 | 404 | 99.26 | NR_040821.1 Streptococcus agalactiae ATCC 13813 strain JCM 5671 16S ribosomal RNA, partial sequence | NR_040821.1 | 1501 | 340 | 763 | 28.25 | 473.9 | 3E-133 | 372/424 | 87 | 23/424 | 5 | Plus | Plus | [illegible] |
| ASV6134 | 404 | 2 | 1 | 404 | 100 | NR_118352.1 Victivallis vadensis ATCC BAA-548 strain Cello 16S ribosomal RNA, partial sequence | NR_118352.1 | 1360 | 338 | 739 | 29.56 | 512.6 | 6E-145 | 362/404 | 89 | 2/404 | 0 | Plus | Plus | [illegible] |
| ASV6137 | 402 | 1 | 1 | 402 | 100 | NR_041236.1 Lutispora thermophila DSM 19022 strain EBR46 16S ribosomal RNA, partial sequence | NR_041236.1 | 1438 | 338 | 740 | 28.03 | 499.7 | 4E-141 | 361/405 | 89 | 5/405 | 1 | Plus | Plus | [illegible] |
| ASV6141 | 403 | 3 | 1 | 402 | 99.75 | NR_147375.1 Colidextribacter massiliensis strain Marseille-P3083 16S ribosomal RNA, partial sequence | NR_147375.1 | 1487 | 364 | 764 | 26.97 | 481.2 | 2E-135 | 358/405 | 88 | 7/405 | 1 | Plus | Plus | [illegible] |
| ASV6143 | 404 | 2 | 1 | 402 | 99.5 | NR_144742.1 Christensenella massiliensis strain Marseille-P2438 16S ribosomal RNA, partial sequence | NR_144742.1 | 1499 | 369 | 771 | 26.88 | 483.1 | 4E-136 | 357/404 | 88 | 3/404 | 0 | Plus | Plus | [illegible] |
| ASV6145 | 404 | 1 | 1 | 402 | 99.5 | NR_025670.1 Pseudoflavonifractor capillosus ATCC 29799 16S ribosomal RNA, partial sequence | NR_025670.1 | 1483 | 346 | 748 | 27.17 | 438.8 | 1E-122 | 352/407 | 86 | 9/407 | 2 | Plus | Plus | [illegible] |
| ASV6146 | 402 | 1 | 1 | 402 | 100 | NR_134091.1 Mobilitalea sibirica strain P3M-3 16S ribosomal RNA, partial sequence | NR_134091.1 | 1444 | 352 | 753 | 27.84 | 422.2 | 1E-117 | 344/402 | 85 | 0/402 | 0 | Plus | Plus | [illegible] |
| ASV6148 | 427 | 1 | 1 | 427 | 100 | NR_041304.1 Sharpea azabuensis strain ST18 16S ribosomal RNA, partial sequence | NR_041304.1 | 1460 | 325 | 750 | 29.18 | 593.9 | 2E-169 | 394/429 | 91 | 5/429 | 1 | Plus | Plus | [illegible] |
| ASV6155 | 403 | 1 | 1 | 403 | 100 | NR_134005.1 Clostridium bornimense strain M2/40 16S ribosomal RNA, partial sequence | NR_134005.1 | 1363 | 284 | 685 | 29.49 | 593.9 | 2E-169 | 376/403 | 93 | 1/403 | 0 | Plus | Plus | [illegible] |
| ASV6156 | 404 | 1 | 1 | 403 | 99.75 | NR_144742.1 Christensenella massiliensis strain Marseille-P2438 16S ribosomal RNA, partial sequence | NR_144742.1 | 1499 | 369 | 772 | 26.95 | 518.2 | 1E-146 | 363/404 | 89 | 1/404 | 0 | Plus | Plus | [illegible] |
| ASV6158 | 402 | 1 | 1 | 402 | 100 | NR_025930.1 Ruminococcus bromii strain ATCC 27255 16S ribosomal RNA, partial sequence | NR_025930.1 | 1380 | 313 | 716 | 29.28 | 606.8 | 3E-173 | 381/406 | 93 | 6/406 | 1 | Plus | Plus | [illegible] |
| ASV6161 | 402 | 5 | 1 | 402 | 100 | NR_102852.1 [Clostridium] saccharolyticum strain WM1 16S ribosomal RNA, partial sequence | NR_102852.1 | 1519 | 365 | 766 | 26.46 | 505.3 | 9E-143 | 360/403 | 89 | 2/403 | 0 | Plus | Plus | [illegible] |
| ASV6164 | 423 | 1 | 1 | 423 | 100 | NR_144750.1 Prevotellamassilia timonensis strain Marseille-P2831 16S ribosomal RNA, partial sequence | NR_144750.1 | 1493 | 358 | 779 | 28.27 | 508.9 | 8E-144 | 376/425 | 88 | 5/425 | 1 | Plus | Plus | [illegible] |
| ASV6166 | 401 | 1 | 3 | 401 | 99.5 | NR_027558.1 Anaerotruncus colihominis strain WAL 14565 16S ribosomal RNA, partial sequence | NR_027558.1 | 1365 | 311 | 709 | 29.23 | 455.4 | 1E-127 | 350/401 | 87 | 4/401 | 0 | Plus | Plus | [illegible] |
| ASV6167 | 402 | 1 | 1 | 402 | 100 | NR_025930.1 Ruminococcus bromii strain ATCC 27255 16S ribosomal RNA, partial sequence | NR_025930.1 | 1380 | 313 | 716 | 29.28 | 507.1 | 3E-143 | 361/404 | 89 | 2/404 | 0 | Plus | Plus | [illegible] |
| ASV6171 | 403 | 2 | 1 | 398 | 98.76 | NR_156975.1 Marinisporobacter balticus strain 59.4M 16S ribosomal RNA, partial sequence | NR_156975.1 | 1435 | 334 | 730 | 27.67 | 446.2 | 6E-125 | 350/402 | 87 | 9/402 | 2 | Plus | Plus | [illegible] |
| ASV6173 | 402 | 1 | 1 | 402 | 100 | NR_122051.1 Clostridium saccharobutylicum strain NCP 262 16S ribosomal RNA, partial sequence | NR_122051.1 | 1501 | 348 | 749 | 26.78 | 593.9 | 2E-169 | 375/402 | 93 | 0/402 | 0 | Plus | Plus | [illegible] |
| ASV6179 | 402 | 1 | 1 | 402 | 100 | NR_118554.1 Intestinimonas butyriciproducens strain SRB-521-5-I 16S ribosomal RNA, partial sequence | NR_118554.1 | 1475 | 346 | 750 | 27.46 | 484.9 | 1E-136 | 359/406 | 88 | 5/406 | 1 | Plus | Plus | [illegible] |
| ASV6183 | 405 | 1 | 1 | 405 | 100 | NR_104907.1 Eubacterium coprostanoligenes strain HL 16S ribosomal RNA, partial sequence | NR_104907.1 | 1446 | 332 | 734 | 27.87 | 569.9 | 3E-162 | 373/405 | 92 | 2/405 | 0 | Plus | Plus | [illegible] |
| ASV6185 | 427 | 2 | 1 | 427 | 100 | NR_026030.1 Actinobacillus porcinus strain NM319 16S ribosomal RNA, partial sequence | NR_026030.1 | 1466 | 335 | 761 | 29.13 | 656.7 | 0 | 404/428 | 94 | 2/428 | 0 | Plus | Plus | [illegible] |
| ASV6186 | 405 | 1 | 1 | 405 | 100 | NR_028997.1 Subdoligranulum variabile strain BI 114 16S ribosomal RNA, partial sequence | NR_028997.1 | 1428 | 320 | 722 | 28.22 | 508.9 | 7E-144 | 363/406 | 89 | 4/406 | 0 | Plus | Plus | [illegible] |
| ASV6187 | 422 | 2 | 1 | 422 | 100 | NR_113119.1 Prevotella oris strain JCM 8540 16S ribosomal RNA, partial sequence | NR_113119.1 | 1481 | 345 | 766 | 28.49 | 497.9 | 2E-140 | 372/423 | 87 | 2/423 | 0 | Plus | Plus | [illegible] |
| ASV6188 | 405 | 4 | 3 | 405 | 99.51 | NR_171444.1 Blautia argi strain N6H1-15 16S ribosomal RNA, partial sequence | NR_171444.1 | 1409 | 320 | 719 | 28.39 | 520 | 3E-147 | 364/404 | 90 | 5/404 | 1 | Plus | Plus | [illegible] |

| | | | | | | | | | | | | | | | | | | | | | | | | | | | | |
|---|---|---|---|---|---|---|---|---|---|---|---|---|---|---|---|---|---|---|---|---|---|---|---|---|---|---|---|---|
| ASV6191 | 402 | 4 | 4 | 402 | 99.25 | NR_102852.1 [Clostridium] saccharolyticum strain WM1 16S ribosomal RNA, partial sequence | NR_102852.1 | 1519 | 368 | 766 | 26.27 | 510.8 | 2E-144 | 359/400 | 89 | 2/400 | 0 | Plus | Plus | Bacteria; Firmicutes; Clostridia; Eubacteriales; Lachnospiraceae; Lacrimispora; Lacrimispora saccharolytica | Lacrimispora saccharolytica | Lacrimispora | Lachnospiraceae | Eubacteriales | Clostridia | Firmicutes | Bacteria |
| ASV6192 | 422 | 1 | 1 | 413 | 97.87 | NR_041364.1 Prevotella stercorea DSM 18206 strain CB35 16S ribosomal RNA, partial sequence | NR_041364.1 | 1488 | 354 | 766 | 27.76 | 503.4 | 4E-142 | 366/413 | 88 | 0/413 | 0 | Plus | Plus | Bacteria; Bacteroidetes; Bacteroidia; Bacteroidales; Prevotellaceae; Prevotella; Prevotella stercorea | Prevotella stercorea | Prevotella | Prevotellaceae | Bacteroidales | Bacteroidia | Bacteroidetes | Bacteria |
| ASV6193 | 402 | 2 | 1 | 402 | 100 | NR_156911.1 Fournierella massiliensis strain AT2 16S ribosomal RNA, partial sequence | NR_156911.1 | 1491 | 368 | 769 | 26.96 | 499.7 | 4E-141 | 359/403 | 89 | 2/403 | 0 | Plus | Plus | Bacteria; Firmicutes; Clostridia; Eubacteriales; Oscillospiraceae; Fournierella; Fournierella massiliensis | Fournierella massiliensis | Fournierella | Oscillospiraceae | Eubacteriales | Clostridia | Firmicutes | Bacteria |
| ASV6194 | 402 | 2 | 1 | 402 | 100 | NR_156080.1 Kineothrix alysoides strain KNHs209 16S ribosomal RNA, partial sequence | NR_156080.1 | 1410 | 353 | 754 | 28.51 | 599.4 | 4E-171 | 377/403 | 93 | 2/403 | 0 | Plus | Plus | Bacteria; Firmicutes; Clostridia; Eubacteriales; Lachnospiraceae; Kineothrix; Kineothrix alysoides | Kineothrix alysoides | Kineothrix | Lachnospiraceae | Eubacteriales | Clostridia | Firmicutes | Bacteria |
| ASV6195 | 401 | 1 | 1 | 401 | 100 | NR_152059.1 Harryflintia acetispora strain V20-281a 16S ribosomal RNA, partial sequence | NR_152059.1 | 1363 | 289 | 692 | 29.64 | 571.7 | 9E-163 | 373/404 | 92 | 3/404 | 0 | Plus | Plus | Bacteria; Firmicutes; Clostridia; Eubacteriales; Oscillospiraceae; Harryflintia; Harryflintia acetispora | Harryflintia acetispora | Harryflintia | Oscillospiraceae | Eubacteriales | Clostridia | Firmicutes | Bacteria |
| ASV6196 | 406 | 1 | 1 | 406 | 100 | NR_148620.1 Lachnotalea glycerini strain : DLD10 16S ribosomal RNA, partial sequence | NR_148620.1 | 1207 | 212 | 613 | 33.31 | 470.2 | 3E-132 | 357/407 | 87 | 6/407 | 1 | Plus | Plus | Bacteria; Firmicutes; Clostridia; Eubacteriales; Lachnospiraceae; Lachnotalea; Lachnotalea glycerini | Lachnotalea glycerini | Lachnotalea | Lachnospiraceae | Eubacteriales | Clostridia | Firmicutes | Bacteria |
| ASV6201 | 406 | 1 | 1 | 406 | 100 | NR_025025.1 Papillibacter cinnamivorans strain CIN1 16S ribosomal RNA, partial sequence | NR_025025.1 | 1491 | 349 | 751 | 27.03 | 481.2 | 2E-135 | 359/408 | 87 | 7/408 | 1 | Plus | Plus | Bacteria; Firmicutes; Clostridia; Eubacteriales; Oscillospiraceae; Papillibacter; Papillibacter cinnamivorans | Papillibacter cinnamivorans | Papillibacter | Oscillospiraceae | Eubacteriales | Clostridia | Firmicutes | Bacteria |
| ASV6205 | 402 | 1 | 1 | 402 | 100 | NR_118667.1 Lachnospira pectinoschiza strain 150-1 16S ribosomal RNA, partial sequence | NR_118667.1 | 1465 | 372 | 773 | 27.44 | 590.2 | 3E-168 | 372/402 | 92 | 0/402 | 0 | Plus | Plus | Bacteria; Firmicutes; Clostridia; Eubacteriales; Lachnospiraceae; Lachnospira; Lachnospira pectinoschiza | Lachnospira pectinoschiza | Lachnospira | Lachnospiraceae | Eubacteriales | Clostridia | Firmicutes | Bacteria |
| ASV6206 | 427 | 1 | 1 | 427 | 100 | NR_112902.1 Phascolarctobacterium succinatutens strain YIT 12067 16S ribosomal RNA, partial sequence | NR_112902.1 | 1610 | 444 | 870 | 26.52 | 584.7 | 1E-166 | 392/429 | 91 | 4/429 | 0 | Plus | Plus | Bacteria; Firmicutes; Negativicutes; Acidaminococcales; Acidaminococcaceae; Phascolarctobacterium; Phascolarctobacterium succinatutens | Phascolarctobacterium succinatutens | Phascolarctobacterium | Acidaminococcaceae | Acidaminococcales | Negativicutes | Firmicutes | Bacteria |
| ASV6215 | 405 | 1 | 3 | 405 | 99.51 | NR_042200.1 [Clostridium] asparagiforme strain N6 16S ribosomal RNA, partial sequence | NR_042200.1 | 1468 | 337 | 736 | 27.25 | 514.5 | 2E-145 | 363/404 | 89 | 5/404 | 1 | Plus | Plus | Bacteria; Firmicutes; Clostridia; Eubacteriales; Lachnospiraceae; Enterocloster; Enterocloster asparagiformis | Enterocloster asparagiformis | Enterocloster | Lachnospiraceae | Eubacteriales | Clostridia | Firmicutes | Bacteria |
| ASV6216 | 402 | 1 | 1 | 402 | 100 | NR_074634.1 [Eubacterium] rectale ATCC 33656 16S ribosomal RNA, partial sequence | NR_074634.1 | 1521 | 367 | 768 | 26.43 | 527.4 | 2E-149 | 363/402 | 90 | 0/402 | 0 | Plus | Plus | Bacteria; Firmicutes; Clostridia; Eubacteriales; Lachnospiraceae; ; [Eubacterium] rectale | [Eubacterium] rectale | | Lachnospiraceae | Eubacteriales | Clostridia | Firmicutes | Bacteria |
| ASV6217 | 421 | 1 | 1 | 421 | 100 | NR_041342.1 Parabacteroides distasonis strain JCM 5825 16S ribosomal RNA, partial sequence | NR_041342.1 | 1488 | 353 | 774 | 28.36 | 507.1 | 3E-143 | 373/422 | 88 | 1/422 | 0 | Plus | Plus | Bacteria; Bacteroidetes; Bacteroidia; Bacteroidales; Tannerellaceae; Parabacteroides; Parabacteroides distasonis | Parabacteroides distasonis | Parabacteroides | Tannerellaceae | Bacteroidales | Bacteroidia | Bacteroidetes | Bacteria |
| ASV6219 | 427 | 1 | 3 | 427 | 99.53 | NR_042184.1 Effusibacillus pohliae strain MP4 16S ribosomal RNA, partial sequence | NR_042184.1 | 1505 | 335 | 759 | 28.24 | 431.4 | 2E-120 | 366/430 | 85 | 10/430 | 2 | Plus | Plus | Bacteria; Firmicutes; Bacilli; Bacillales; Alicyclobacillaceae; Effusibacillus; Effusibacillus pohliae | Effusibacillus pohliae | Effusibacillus | Alicyclobacillaceae | Bacillales | Bacilli | Firmicutes | Bacteria |
| ASV6220 | 404 | 1 | 1 | 404 | 100 | NR_144742.1 Christensenella massiliensis strain Marseille-P2438 16S ribosomal RNA, partial sequence | NR_144742.1 | 1499 | 369 | 773 | 27.02 | 508.9 | 7E-144 | 363/406 | 89 | 3/406 | 0 | Plus | Plus | Bacteria; Firmicutes; Clostridia; Eubacteriales; Christensenellaceae; Christensenella; Christensenella massiliensis | Christensenella massiliensis | Christensenella | Christensenellaceae | Eubacteriales | Clostridia | Firmicutes | Bacteria |
| ASV6223 | 404 | 1 | 1 | 404 | 100 | NR_159227.1 Monoglobus pectinilyticus strain 14 16S ribosomal RNA, partial sequence | NR_159227.1 | 1493 | 368 | 769 | 26.93 | 479.4 | 6E-135 | 359/407 | 88 | 8/407 | 1 | Plus | Plus | Bacteria; Firmicutes; Clostridia; Eubacteriales; Oscillospiraceae; Monoglobus; Monoglobus pectinilyticus | Monoglobus pectinilyticus | Monoglobus | Oscillospiraceae | Eubacteriales | Clostridia | Firmicutes | Bacteria |
| ASV6228 | 422 | 1 | 1 | 422 | 100 | NR_074577.1 Paludibacter propionicigenes WB4 16S ribosomal RNA, partial sequence | NR_074577.1 | 1516 | 353 | 774 | 27.84 | 531.1 | 2E-150 | 378/423 | 89 | 2/423 | 0 | Plus | Plus | Bacteria; Bacteroidetes; Bacteroidia; Bacteroidales; Paludibacteraceae; Paludibacter; Paludibacter propionicigenes | Paludibacter propionicigenes | Paludibacter | Paludibacteraceae | Bacteroidales | Bacteroidia | Bacteroidetes | Bacteria |
| ASV6232 | 402 | 5 | 1 | 393 | 97.76 | NR_115693.1 Gracilibacter thermotolerans JW/YJL-S1 16S ribosomal RNA, partial sequence | NR_115693.1 | 1526 | 459 | 852 | 25.82 | 433.2 | 5E-121 | 343/396 | 86 | 5/396 | 1 | Plus | Plus | Bacteria; Firmicutes; Clostridia; Eubacteriales; Gracilibacteraceae; Gracilibacter; Gracilibacter thermotolerans | Gracilibacter thermotolerans | Gracilibacter | Gracilibacteraceae | Eubacteriales | Clostridia | Firmicutes | Bacteria |
| ASV6234 | 403 | 1 | 1 | 403 | 100 | NR_028997.1 Subdoligranulum variabile strain BI 114 16S ribosomal RNA, partial sequence | NR_028997.1 | 1428 | 320 | 722 | 28.22 | 557 | 3E-158 | 369/403 | 91 | 0/403 | 0 | Plus | Plus | Bacteria; Firmicutes; Clostridia; Eubacteriales; Oscillospiraceae; Subdoligranulum; Subdoligranulum variabile | Subdoligranulum variabile | Subdoligranulum | Oscillospiraceae | Eubacteriales | Clostridia | Firmicutes | Bacteria |
| ASV6237 | 401 | 1 | 1 | 399 | 99.5 | NR_044289.1 [Clostridium] lavalense strain CCRI-9842 16S ribosomal RNA, partial sequence | NR_044289.1 | 1454 | 345 | 744 | 27.51 | 438.8 | 1E-122 | 348/402 | 86 | 5/402 | 1 | Plus | Plus | Bacteria; Firmicutes; Clostridia; Eubacteriales; Lachnospiraceae; Enterocloster; Enterocloster lavalensis | Enterocloster lavalensis | Enterocloster | Lachnospiraceae | Eubacteriales | Clostridia | Firmicutes | Bacteria |
| ASV6240 | 402 | 2 | 1 | 402 | 100 | NR_026496.1 Clostridium polyendosporum strain PS-1 16S ribosomal RNA, partial sequence | NR_026496.1 | 1456 | 338 | 739 | 27.61 | 649.3 | 0 | 385/402 | 95 | 0/402 | 0 | Plus | Plus | Bacteria; Firmicutes; Clostridia; Eubacteriales; Clostridiaceae; Clostridium; Clostridium polyendosporum | Clostridium polyendosporum | Clostridium | Clostridiaceae | Eubacteriales | Clostridia | Firmicutes | Bacteria |
| ASV6242 | 403 | 1 | 1 | 403 | 100 | NR_118156.1 Oscillibacter ruminantium GH1 16S ribosomal RNA, partial sequence | NR_118156.1 | 1409 | 321 | 724 | 28.67 | 523.7 | 3E-148 | 365/405 | 90 | 3/405 | 0 | Plus | Plus | Bacteria; Firmicutes; Clostridia; Eubacteriales; Oscillospiraceae; Oscillibacter; Oscillibacter ruminantium | Oscillibacter ruminantium | Oscillibacter | Oscillospiraceae | Eubacteriales | Clostridia | Firmicutes | Bacteria |
| ASV6250 | 404 | 1 | 1 | 404 | 100 | NR_044111.1 Selenomonas bovis strain WG 16S ribosomal RNA, partial sequence | NR_044111.1 | 1525 | 370 | 796 | 28 | 490.5 | 3E-138 | 377/427 | 88 | 23/427 | 5 | Plus | Plus | Bacteria; Firmicutes; Negativicutes; Selenomonadales; Selenomonadaceae; Selenomonas; Selenomonas bovis | Selenomonas bovis | Selenomonas | Selenomonadaceae | Selenomonadales | Negativicutes | Firmicutes | Bacteria |
| ASV6252 | 402 | 2 | 1 | 402 | 100 | NR_159227.1 Monoglobus pectinilyticus strain 14 16S ribosomal RNA, partial sequence | NR_159227.1 | 1493 | 368 | 769 | 26.93 | 533 | 4E-151 | 364/402 | 90 | 0/402 | 0 | Plus | Plus | Bacteria; Firmicutes; Clostridia; Eubacteriales; Oscillospiraceae; Monoglobus; Monoglobus pectinilyticus | Monoglobus pectinilyticus | Monoglobus | Oscillospiraceae | Eubacteriales | Clostridia | Firmicutes | Bacteria |
| ASV6254 | 405 | 1 | 5 | 405 | 99.01 | NR_118554.1 Intestinimonas butyriciproducens strain SRB-521-5-I 16S ribosomal RNA, partial sequence | NR_118554.1 | 1475 | 350 | 750 | 27.19 | 486.8 | 3E-137 | 357/403 | 88 | 4/403 | 0 | Plus | Plus | Bacteria; Firmicutes; Clostridia; Eubacteriales; ; Intestinimonas; Intestinimonas butyriciproducens | Intestinimonas butyriciproducens | Intestinimonas | | Eubacteriales | Clostridia | Firmicutes | Bacteria |
| ASV6256 | 402 | 5 | 5 | 402 | 99 | NR_102884.1 Ruminococcus champanellensis 18P13 = JCM 17042 16S ribosomal RNA, partial sequence | NR_102884.1 | 1494 | 352 | 749 | 26.64 | 475.7 | 7E-134 | 352/399 | 88 | 2/399 | 0 | Plus | Plus | Bacteria; Firmicutes; Clostridia; Eubacteriales; Oscillospiraceae; Ruminococcus; Ruminococcus champanellensis | Ruminococcus champanellensis | Ruminococcus | Oscillospiraceae | Eubacteriales | Clostridia | Firmicutes | Bacteria |
| ASV6257 | 426 | 1 | 1 | 426 | 100 | NR_152059.1 Harryflintia acetispora strain V20-281a 16S ribosomal RNA, partial sequence | NR_152059.1 | 1363 | 289 | 692 | 29.64 | 451.7 | 1E-126 | 369/426 | 86 | 22/426 | 5 | Plus | Plus | Bacteria; Firmicutes; Clostridia; Eubacteriales; Oscillospiraceae; Harryflintia; Harryflintia acetispora | Harryflintia acetispora | Harryflintia | Oscillospiraceae | Eubacteriales | Clostridia | Firmicutes | Bacteria |
| ASV6258 | 402 | 1 | 1 | 402 | 100 | NR_151895.1 Anaerocolumna cellulosilytica strain SN021 16S ribosomal RNA, partial sequence | NR_151895.1 | 1478 | 353 | 754 | 27.2 | 571.7 | 9E-163 | 373/404 | 92 | 4/404 | 0 | Plus | Plus | Bacteria; Firmicutes; Clostridia; Eubacteriales; Lachnospiraceae; Anaerocolumna; Anaerocolumna cellulosilytica | Anaerocolumna cellulosilytica | Anaerocolumna | Lachnospiraceae | Eubacteriales | Clostridia | Firmicutes | Bacteria |
| ASV6259 | 406 | 1 | 1 | 406 | 100 | NR_025670.1 Pseudoflavonifractor capillosus ATCC 29799 16S ribosomal RNA, partial sequence | NR_025670.1 | 1483 | 346 | 750 | 27.31 | 488.6 | 1E-137 | 359/406 | 88 | 1/406 | 0 | Plus | Plus | Bacteria; Firmicutes; Clostridia; Eubacteriales; Oscillospiraceae; Pseudoflavonifractor; Pseudoflavonifractor capillosus | Pseudoflavonifractor capillosus | Pseudoflavonifractor | Oscillospiraceae | Eubacteriales | Clostridia | Firmicutes | Bacteria |
| ASV6260 | 427 | 1 | 1 | 375 | 87.82 | NR_041304.1 Sharpea azabuensis strain ST18 16S ribosomal RNA, partial sequence | NR_041304.1 | 1460 | 325 | 698 | 25.62 | 569.9 | 4E-162 | 353/375 | 94 | 1/375 | 0 | Plus | Plus | Bacteria; Firmicutes; Erysipelotrichia; Erysipelotrichales; Coprobacillaceae; Sharpea; Sharpea azabuensis | Sharpea azabuensis | Sharpea | Coprobacillaceae | Erysipelotrichales | Erysipelotrichia | Firmicutes | Bacteria |
| ASV6261 | 401 | 4 | 1 | 386 | 96.26 | NR_114807.1 Marvinbryantia formatexigens strain I-52 16S ribosomal RNA, partial sequence | NR_114807.1 | 1456 | 336 | 722 | 26.58 | 398.1 | 2E-110 | 333/390 | 85 | 7/390 | 1 | Plus | Plus | Bacteria; Firmicutes; Clostridia; Eubacteriales; Lachnospiraceae; Marvinbryantia; Marvinbryantia formatexigens | Marvinbryantia formatexigens | Marvinbryantia | Lachnospiraceae | Eubacteriales | Clostridia | Firmicutes | Bacteria |
| ASV6262 | 401 | 1 | 1 | 401 | 100 | NR_147370.1 Pseudoflavonifractor phocaeensis strain Marseille-P3064 16S ribosomal RNA, partial sequence | NR_147370.1 | 1495 | 366 | 770 | 27.09 | 435.1 | 1E-121 | 349/405 | 86 | 4/405 | 0 | Plus | Plus | Bacteria; Firmicutes; Clostridia; Eubacteriales; Oscillospiraceae; Pseudoflavonifractor; Pseudoflavonifractor phocaeensis | Pseudoflavonifractor phocaeensis | Pseudoflavonifractor | Oscillospiraceae | Eubacteriales | Clostridia | Firmicutes | Bacteria |
| ASV6263 | 405 | 1 | 1 | 405 | 100 | NR_118554.1 Intestinimonas butyriciproducens strain SRB-521-5-I 16S ribosomal RNA, partial sequence | NR_118554.1 | 1475 | 346 | 750 | 27.46 | 488.6 | 1E-137 | 359/406 | 88 | 2/406 | 0 | Plus | Plus | Bacteria; Firmicutes; Clostridia; Eubacteriales; ; Intestinimonas; Intestinimonas butyriciproducens | Intestinimonas butyriciproducens | Intestinimonas | | Eubacteriales | Clostridia | Firmicutes | Bacteria |
| [illegible] | [illegible] | [illegible] | [illegible] | [illegible] | [illegible] | [illegible] | [illegible] | [illegible] | [illegible] | [illegible] | [illegible] | [illegible] | [illegible] | [illegible] | [illegible] | [illegible] | [illegible] | [illegible] | [illegible] | [illegible] | [illegible] | [illegible] | [illegible] | [illegible] | [illegible] | [illegible] | [illegible] |

| | | | | | | | | | | | | | | | | |
|---|---|---|---|---|---|---|---|---|---|---|---|---|---|---|---|---|
| ASV6828 | 427 | 2 | 1 | 427 | 100 | NR_037018.1 Acidaminococcus fermentans strain VR4 16S ribosomal RNA gene, partial sequence | NR_037018.1 | 1516 | 349 | 775 | 28.17 | 612.4 | 6E-175 | 400/432 | 92 | [illegible] |
| ASV6831 | 403 | 1 | 3 | 403 | 99.5 | NR_042832.1 Roseburia faecis strain M72/1 16S ribosomal RNA, partial sequence | NR_042832.1 | 1456 | 349 | 749 | 27.54 | 536.6 | 3E-152 | 367/404 | 90 | [illegible] |
| ASV6842 | 402 | 1 | 1 | 402 | 100 | NR_042832.1 Roseburia faecis strain M72/1 16S ribosomal RNA, partial sequence | NR_042832.1 | 1456 | 347 | 749 | 27.68 | 593.9 | 2E-169 | 377/404 | 93 | [illegible] |
| ASV6845 | 405 | 4 | 3 | 405 | 99.51 | NR_117165.1 Ruminiclostridium hungatei strain DSM 14427 16S ribosomal RNA, partial sequence | NR_117165.1 | 1654 | 495 | 896 | 24.3 | 494.2 | 2E-139 | 358/403 | 88 | [illegible] |
| ASV6850 | 403 | 2 | 1 | 403 | 100 | NR_112089.1 [Clostridium] hylemonae DSM 15053 strain TN-271 16S ribosomal RNA, partial sequence | NR_112089.1 | 1377 | 353 | 754 | 29.19 | 483.1 | 4E-136 | 357/404 | 88 | [illegible] |
| ASV6851 | 401 | 1 | 1 | 401 | 100 | NR_169458.1 Tepidibaculum saccharolyticum strain STR9 16S ribosomal RNA, partial sequence | NR_169458.1 | 1477 | 362 | 763 | 27.22 | 497.9 | 2E-140 | 359/403 | 89 | [illegible] |
| ASV6855 | 427 | 1 | 3 | 427 | 99.53 | NR_156840.1 Desulfuribacillus stibiiarsenatis strain MLFW-2 16S ribosomal RNA, partial sequence | NR_156840.1 | 1446 | 332 | 756 | 29.39 | 436.9 | 4E-122 | 366/429 | 85 | [illegible] |
| ASV6858 | 406 | 1 | 1 | 406 | 100 | NR_152059.1 Harryflintia acetispora strain V20-281a 16S ribosomal RNA, partial sequence | NR_152059.1 | 1363 | 289 | 692 | 29.64 | 560.7 | 2E-159 | 372/406 | 91 | [illegible] |
| ASV6859 | 424 | 3 | 1 | 424 | 100 | NR_116726.1 Prevotella scopos JCM 17725 strain W2052 16S ribosomal RNA, partial sequence | NR_116726.1 | 1453 | 338 | 759 | 29.04 | 455.4 | 1E-127 | 367/426 | 86 | [illegible] |
| ASV6860 | 405 | 1 | 1 | 404 | 99.75 | NR_118554.1 Intestinimonas butyriciproducens strain SRB-521-5-I 16S ribosomal RNA, partial sequence | NR_118554.1 | 1475 | 346 | 749 | 27.39 | 542.2 | 7E-154 | 367/404 | 90 | [illegible] |
| ASV6864 | 402 | 1 | 1 | 402 | 100 | NR_025930.1 Ruminococcus bromii strain ATCC 27255 16S ribosomal RNA, partial sequence | NR_025930.1 | 1380 | 313 | 716 | 29.28 | 534.8 | 1E-151 | 366/404 | 90 | [illegible] |
| ASV6871 | 404 | 1 | 1 | 404 | 100 | NR_028961.1 Faecalibacterium prausnitzii strain ATCC 27768 16S ribosomal RNA, partial sequence | NR_028961.1 | 1462 | 344 | 745 | 27.5 | 545.9 | 6E-155 | 369/405 | 91 | [illegible] |
| ASV6873 | 403 | 1 | 1 | 398 | 98.76 | NR_026446.1 Tindallia magadiensis strain Z-7934 16S ribosomal RNA, partial sequence | NR_026446.1 | 1482 | 345 | 742 | 26.86 | 425.9 | 8E-119 | 348/404 | 86 | [illegible] |
| ASV6876 | 404 | 1 | 1 | 404 | 100 | NR_118156.1 Oscillibacter ruminantium GH1 16S ribosomal RNA, partial sequence | NR_118156.1 | 1409 | 321 | 724 | 28.67 | 542.2 | 7E-154 | 371/408 | 90 | [illegible] |
| ASV6879 | 427 | 5 | 3 | 425 | 99.06 | NR_042052.1 Streptococcus equinus ATCC 9812 16S ribosomal RNA, partial sequence | NR_042052.1 | 1433 | 262 | 684 | 29.52 | 460.9 | 2E-129 | 365/423 | 86 | [illegible] |
| ASV6880 | 425 | 1 | 3 | 425 | 99.53 | NR_028840.1 Mitsuokella jalaludinii strain M9 16S ribosomal RNA, partial sequence | NR_028840.1 | 1543 | 364 | 788 | 27.54 | 568 | 1E-161 | 390/429 | 90 | [illegible] |
| ASV6881 | 401 | 4 | 1 | 399 | 99.5 | NR_134772.1 Murimonas intestini strain SRB-530-5-H 16S ribosomal RNA, partial sequence | NR_134772.1 | 1433 | 314 | 713 | 27.91 | 477.6 | 2E-134 | 355/402 | 88 | [illegible] |
| ASV6882 | 422 | 1 | 1 | 422 | 100 | NR_113411.1 Prevotella copri DSM 18205 strain JCM 13464 16S ribosomal RNA, partial sequence | NR_113411.1 | 1493 | 358 | 779 | 28.27 | 520 | 4E-147 | 375/422 | 88 | [illegible] |
| ASV6884 | 405 | 1 | 3 | 405 | 99.51 | NR_159150.1 Anoxynatronum buryatiense strain Su22 16S ribosomal RNA, partial sequence | NR_159150.1 | 1507 | 369 | 770 | 26.68 | 460.9 | 2E-129 | 353/404 | 87 | [illegible] |
| ASV6885 | 405 | 1 | 1 | 405 | 100 | NR_044048.1 Coprococcus comes ATCC 27758 16S ribosomal RNA, partial sequence | NR_044048.1 | 1453 | 345 | 746 | 27.67 | 623.4 | 3E-178 | 383/405 | 94 | [illegible] |
| ASV6886 | 403 | 1 | 1 | 403 | 100 | NR_025930.1 Ruminococcus bromii strain ATCC 27255 16S ribosomal RNA, partial sequence | NR_025930.1 | 1380 | 313 | 716 | 29.28 | 545.9 | 6E-155 | 368/404 | 91 | [illegible] |
| ASV6889 | 426 | 1 | 1 | 425 | 99.77 | NR_027596.1 Mitsuokella multacida strain DSM 20544 16S ribosomal RNA, partial sequence | NR_027596.1 | 1511 | 346 | 771 | 28.19 | 555.1 | 1E-157 | 387/429 | 90 | [illegible] |
| ASV6894 | 427 | 4 | 1 | 426 | 99.77 | NR_042802.1 Lactobacillus ultunensis strain Kx146C1 16S ribosomal RNA, partial sequence | NR_042802.1 | 1550 | 370 | 795 | 27.48 | 499.7 | 5E-141 | 377/429 | 87 | [illegible] |
| ASV6896 | 421 | 1 | 15 | 421 | 96.67 | NR_044111.1 Selenomonas bovis strain WG 16S ribosomal RNA, partial sequence | NR_044111.1 | 1525 | 384 | 796 | 27.08 | 464.6 | 2E-130 | 362/415 | 87 | [illegible] |
| ASV6903 | 404 | 1 | 1 | 404 | 100 | NR_112097.1 Pseudoramibacter alactolyticus ATCC 23263 16S ribosomal RNA, partial sequence | NR_112097.1 | 1480 | 376 | 779 | 27.3 | 608.7 | 7E-174 | 379/404 | 93 | [illegible] |
| ASV6904 | 427 | 1 | 3 | 410 | 95.55 | NR_102980.1 Megasphaera elsdenii DSM 20460 16S ribosomal RNA, partial sequence | NR_102980.1 | 1552 | 371 | 778 | 26.29 | 566.2 | 5E-161 | 374/408 | 91 | [illegible] |
| ASV6908 | 422 | 2 | 1 | 422 | 100 | NR_113089.1 Porphyromonas levii DSM 23370 strain JCM 13866 16S ribosomal RNA, partial sequence | NR_113089.1 | 1495 | 356 | 777 | 28.23 | 431.4 | 2E-120 | 365/428 | 85 | [illegible] |
| ASV6909 | 404 | 4 | 1 | 404 | 100 | NR_042802.1 Lactobacillus ultunensis strain Kx146C1 16S ribosomal RNA, partial sequence | NR_042802.1 | 1550 | 370 | 796 | 27.55 | 529.3 | 6E-150 | 384/427 | 89 | [illegible] |
| ASV6910 | 422 | 3 | 1 | 422 | 100 | NR_113094.1 Prevotella baroniae strain JCM 13447 16S ribosomal RNA, partial sequence | NR_113094.1 | 1494 | 358 | 779 | 28.25 | 453.5 | 4E-127 | 367/426 | 86 | [illegible] |
| ASV6911 | 426 | 1 | 1 | 426 | 100 | NR_144737.1 Emergencia timonensis strain SN18 16S ribosomal RNA, partial sequence | NR_144737.1 | 1480 | 364 | 767 | 27.3 | 457.2 | 3E-128 | 371/427 | 86 | [illegible] |
| ASV6912 | 402 | 1 | 3 | 402 | 99.5 | NR_159261.1 Bifidobacterium callitrichidarum strain TRI 5 16S ribosomal RNA, partial sequence | NR_159261.1 | 1374 | 281 | 686 | 29.55 | 534.8 | 1E-151 | 368/406 | 90 | [illegible] |
| ASV6916 | 407 | 1 | 1 | 407 | 100 | NR_112652.1 Lactobacillus equicursoris DSM 19284 = JCM 14600 = CIP 110162 strain DI70 16S ribosomal RNA, partial sequence | NR_112652.1 | 1486 | 351 | 777 | 28.73 | 521.9 | 1E-147 | 383/428 | 89 | [illegible] |
| ASV6918 | 425 | 3 | 1 | 425 | 100 | NR_102980.1 Megasphaera elsdenii DSM 20460 16S ribosomal RNA, partial sequence | NR_102980.1 | 1552 | 369 | 795 | 27.51 | 582.8 | 5E-166 | 395/432 | 91 | [illegible] |
| ASV6930 | 422 | 1 | 1 | 407 | 96.45 | NR_113411.1 Prevotella copri DSM 18205 strain JCM 13464 16S ribosomal RNA, partial sequence | NR_113411.1 | 1493 | 358 | 764 | 27.26 | 614.2 | 2E-175 | 383/408 | 93 | [illegible] |
| ASV6935 | 422 | 1 | 1 | 422 | 100 | NR_144704.1 Bacteroides timonensis AP1 16S ribosomal RNA, partial sequence | NR_144704.1 | 1315 | 268 | 689 | 32.09 | 453.5 | 4E-127 | 367/426 | 86 | [illegible] |
| ASV6944 | 405 | 2 | 1 | 405 | 100 | NR_118690.1 [Ruminococcus] gnavus ATCC 29149 16S ribosomal RNA, partial sequence | NR_118690.1 | 1423 | 355 | 756 | 28.25 | 507.1 | 3E-143 | 363/406 | 89 | [illegible] |
| ASV6951 | 401 | 1 | 5 | 399 | 98.5 | NR_027573.1 Intestinibacter bartlettii strain WAL 16138 16S ribosomal RNA, partial sequence | NR_027573.1 | 1429 | 324 | 718 | 27.64 | 508.9 | 7E-144 | 358/398 | 89 | [illegible] |
| ASV6956 | 427 | 3 | 3 | 427 | 99.53 | NR_041781.1 Streptococcus alactolyticus strain ATCC 43077 16S ribosomal RNA, partial sequence | NR_041781.1 | 1437 | 348 | 772 | 29.58 | 553.3 | 4E-157 | 386/428 | 90 | [illegible] |
| ASV6958 | 402 | 1 | 3 | 402 | 99.5 | NR_042074.1 Eubacterium pyruvativorans strain I-6 16S ribosomal RNA, partial sequence | NR_042074.1 | 1296 | 266 | 667 | 31.02 | 586.5 | 3E-167 | 374/402 | 93 | [illegible] |
| ASV6964 | 408 | 1 | 1 | 408 | 100 | NR_137408.1 Terrisporobacter petrolearius strain LAM0A37 16S ribosomal RNA, partial sequence | NR_137408.1 | 1405 | 307 | 707 | 28.54 | 569.9 | 3E-162 | 376/408 | 92 | [illegible] |
| ASV6966 | 402 | 1 | 1 | 402 | 100 | NR_134005.1 Clostridium bornimense strain M2/40 16S ribosomal RNA, partial sequence | NR_134005.1 | 1363 | 284 | 685 | 29.49 | 488.6 | 1E-137 | 357/403 | 88 | [illegible] |
| ASV6968 | 403 | 3 | 3 | 403 | 99.5 | NR_041781.1 Streptococcus alactolyticus strain ATCC 43077 16S ribosomal RNA, partial sequence | NR_041781.1 | 1437 | 348 | 772 | 29.58 | 503.4 | 3E-142 | 378/425 | 88 | [illegible] |
| ASV6973 | 402 | 3 | 1 | 402 | 100 | NR_026409.1 [Desulfotomaculum] guttoideum strain DSM 4024 16S ribosomal RNA, partial sequence | NR_026409.1 | 1495 | 358 | 759 | 26.89 | 582.8 | 4E-166 | 373/402 | 92 | [illegible] |
| ASV6974 | 428 | 2 | 1 | 428 | 100 | NR_118156.1 Oscillibacter ruminantium GH1 16S ribosomal RNA, partial sequence | NR_118156.1 | 1409 | 321 | 724 | 28.67 | 470.2 | 4E-132 | 375/429 | 87 | [illegible] |
| ASV6976 | 401 | 1 | 1 | 401 | 100 | NR_152059.1 Harryflintia acetispora strain V20-281a 16S ribosomal RNA, partial sequence | NR_152059.1 | 1363 | 289 | 692 | 29.64 | 499.7 | 4E-141 | 360/404 | 89 | [illegible] |
| ASV6977 | 422 | 1 | 1 | 422 | 100 | NR_041342.1 Parabacteroides distasonis strain JCM 5825 16S ribosomal RNA, partial sequence | NR_041342.1 | 1488 | 353 | 774 | 28.36 | 481.2 | 2E-135 | 371/425 | 87 | [illegible] |
| ASV6978 | 403 | 1 | 1 | 403 | 100 | NR_043681.1 [Clostridium] citroniae strain RMA 16102 16S ribosomal RNA, partial sequence | NR_043681.1 | 1420 | 330 | 731 | 28.31 | 477.6 | 2E-134 | 357/405 | 88 | [illegible] |
| ASV6983 | 427 | 1 | 1 | 427 | 100 | NR_029239.3 Clostridium chartatabidum strain 163 16S ribosomal RNA, partial sequence | NR_029239.3 | 1460 | 323 | 724 | 27.53 | 538.5 | 1E-152 | 387/428 | 90 | [illegible] |
| ASV6985 | 408 | 1 | 1 | 403 | 98.77 | NR_117757.1 Atopobium vaginae DSM 15829 16S ribosomal RNA, partial sequence | NR_117757.1 | 1331 | 322 | 724 | 30.28 | 462.8 | 6E-130 | 354/405 | 87 | [illegible] |
| ASV6989 | 406 | 1 | 1 | 406 | 100 | NR_144611.1 Flintibacter butyricus strain BLS21 16S ribosomal RNA, partial sequence | NR_144611.1 | 808 | 299 | 703 | 50.12 | 472 | 1E-132 | 357/407 | 87 | [illegible] |
| ASV6991 | 403 | 3 | 3 | 403 | 99.5 | NR_102980.1 Megasphaera elsdenii DSM 20460 16S ribosomal RNA, partial sequence | NR_102980.1 | 1552 | 371 | 795 | 27.38 | 531.1 | 2E-150 | 385/427 | 90 | [illegible] |
| ASV6992 | 405 | 2 | 1 | 405 | 100 | NR_026336.1 Clostridium vincentii strain DSM 10228 16S ribosomal RNA, partial sequence | NR_026336.1 | 1464 | 328 | 729 | 27.46 | 534.8 | 1E-151 | 368/406 | 90 | [illegible] |
| ASV6994 | 428 | 2 | 1 | 427 | 99.77 | NR_037018.1 Acidaminococcus fermentans strain VR4 16S ribosomal RNA gene, partial sequence | NR_037018.1 | 1516 | 349 | 774 | 28.1 | 516.3 | 5E-146 | 379/428 | 88 | [illegible] |
| ASV6997 | 402 | 1 | 1 | 402 | 100 | NR_114789.1 [Clostridium] leptum strain DSM 753 16S ribosomal RNA, partial sequence | NR_114789.1 | 1505 | 364 | 765 | 26.71 | 499.7 | 4E-141 | 358/402 | 89 | [illegible] |
| ASV6998 | 427 | 1 | 1 | 426 | 99.77 | NR_029348.1 Treponema brennaborense strain DD5/3 16S ribosomal RNA, partial sequence | NR_029348.1 | 1497 | 305 | 731 | 28.52 | 534.8 | 1E-151 | 381/427 | 89 | [illegible] |
| ASV7001 | 402 | 1 | 1 | 402 | 100 | NR_028785.1 [Clostridium] scindens strain ATCC 35704 16S ribosomal RNA, partial sequence | NR_028785.1 | 1529 | 368 | 769 | 26.29 | 477.6 | 2E-134 | 356/404 | 88 | [illegible] |
| ASV7003 | 405 | 1 | 1 | 405 | 100 | NR_118692.1 Blautia obeum ATCC 29174 16S ribosomal RNA, partial sequence | NR_118692.1 | 1398 | 330 | 731 | 28.76 | 558.8 | 7E-159 | 374/408 | 91 | [illegible] |
| ASV7006 | 409 | 1 | 1 | 409 | 100 | NR_147375.1 Colidextribacter massiliensis strain Marseille-P3083 16S ribosomal RNA, partial sequence | NR_147375.1 | 1487 | 364 | 765 | 27.03 | 488.6 | 1E-137 | 364/411 | 88 | [illegible] |
| ASV7010 | 404 | 1 | 3 | 404 | 99.5 | NR_025930.1 Ruminococcus bromii strain ATCC 27255 16S ribosomal RNA, partial sequence | NR_025930.1 | 1380 | 315 | 716 | 29.13 | 449.9 | 5E-126 | 351/404 | 86 | [illegible] |
| ASV7012 | 422 | 1 | 1 | 422 | 100 | NR_144750.1 Prevotellamassilia timonensis strain Marseille-P2831 16S ribosomal RNA, partial sequence | NR_144750.1 | 1493 | 358 | 779 | 28.27 | 442.5 | 8E-124 | 364/425 | 85 | [illegible] |
| ASV7015 | 404 | 1 | 1 | 402 | 99.5 | NR_115340.1 Saccharofermentans acetigenes strain P6 16S ribosomal RNA, partial sequence | NR_115340.1 | 1510 | 353 | 755 | 26.69 | 438.8 | 1E-122 | 351/406 | 86 | [illegible] |
| ASV7018 | 422 | 1 | 1 | 422 | 100 | NR_144750.1 Prevotellamassilia timonensis strain Marseille-P2831 16S ribosomal RNA, partial sequence | NR_144750.1 | 1493 | 358 | 779 | 28.27 | 431.4 | 2E-120 | 364/427 | 85 | [illegible] |
| ASV7019 | 404 | 1 | 1 | 403 | 99.75 | NR_144749.1 Ihubacter massiliensis strain Marseille-P2843 16S ribosomal RNA, partial sequence | NR_144749.1 | 1479 | 364 | 765 | 27.18 | 499.7 | 4E-141 | 361/405 | 89 | [illegible] |
| ASV7020 | 402 | 5 | 1 | 402 | 100 | NR_115693.1 Gracilibacter thermotolerans JW/YJL-S1 16S ribosomal RNA, partial sequence | NR_115693.1 | 1526 | 459 | 861 | 26.41 | 438.8 | 1E-122 | 350/405 | 86 | [illegible] |
| ASV7022 | 402 | 1 | 1 | 402 | 100 | NR_147375.1 Colidextribacter massiliensis strain Marseille-P3083 16S ribosomal RNA, partial sequence | NR_147375.1 | 1487 | 364 | 765 | 27.03 | 433.2 | 5E-121 | 346/402 | 86 | [illegible] |
| ASV7023 | 402 | 1 | 1 | 402 | 100 | NR_134781.1 Olsenella scatoligenes strain SK9K4 16S ribosomal RNA, partial sequence | NR_134781.1 | 1469 | 348 | 755 | 27.77 | 521.9 | 9E-148 | 367/408 | 89 | [illegible] |
| ASV7026 | 428 | 1 | 1 | 428 | 100 | NR_028740.1 Anaerocolumna xylanovorans strain HESP1 16S ribosomal RNA, partial sequence | NR_028740.1 | 1533 | 356 | 777 | 27.53 | 436.9 | 4E-122 | 368/431 | 85 | [illegible] |
| ASV7032 | 402 | 1 | 36 | 401 | 91.04 | NR_144736.1 Neglecta timonensis strain SN17 16S ribosomal RNA, partial sequence | NR_144736.1 | 1503 | 412 | 777 | 24.35 | 527.4 | 2E-149 | 339/366 | 92 | [illegible] |
| ASV7033 | 402 | 5 | 1 | 402 | 100 | NR_026490.1 Clostridium sartagoforme strain DSM 1292 16S ribosomal RNA, partial sequence | NR_026490.1 | 1470 | 337 | 738 | 27.35 | 466.5 | 4E-131 | 352/402 | 87 | [illegible] |
| ASV7036 | 405 | 2 | 1 | 405 | 100 | NR_074634.1 [Eubacterium] rectale ATCC 33656 16S ribosomal RNA, partial sequence | NR_074634.1 | 1521 | 367 | 768 | 26.43 | 435.1 | 1E-121 | 349/405 | 86 | [illegible] |
| ASV7040 | 421 | 2 | 1 | 421 | 100 | NR_144616.1 Muribaculum intestinale strain YL27 16S ribosomal RNA, partial sequence | NR_144616.1 | 1460 | 297 | 718 | 28.9 | 440.6 | 3E-123 | 362/423 | 85 | [illegible] |
| ASV7041 | 426 | 2 | 1 | 426 | 100 | NR_029352.1 Lactobacillus amylolyticus DSM 11664 strain LA 5 16S ribosomal RNA, partial sequence | NR_029352.1 | 1544 | 361 | 787 | 27.66 | 527.4 | 2E-149 | 380/427 | 88 | [illegible] |
| ASV7042 | 427 | 3 | 10 | 427 | 97.89 | NR_036835.1 Chlamydia muridarum strain MoPn/Wiess-Nigg 16S ribosomal RNA, partial sequence | NR_036835.1 | 1549 | 373 | 789 | 26.92 | 438.8 | 1E-122 | 363/423 | 85 | [illegible] |
| ASV7043 | 421 | 1 | 1 | 421 | 100 | NR_041342.1 Parabacteroides distasonis strain JCM 5825 16S ribosomal RNA, partial sequence | NR_041342.1 | 1488 | 353 | 774 | 28.36 | 468.3 | 1E-131 | 367/423 | 86 | [illegible] |
| ASV7045 | 401 | 1 | 1 | 401 | 100 | NR_137408.1 Terrisporobacter petrolearius strain LAM0A37 16S ribosomal RNA, partial sequence | NR_137408.1 | 1405 | 307 | 707 | 28.54 | 558.8 | 7E-159 | 370/403 | 91 | [illegible] |
| ASV7048 | 421 | 1 | 1 | 421 | 100 | NR_041342.1 Parabacteroides distasonis strain JCM 5825 16S ribosomal RNA, partial sequence | NR_041342.1 | 1488 | 353 | 774 | 28.36 | 473.9 | 3E-133 | 368/423 | 86 | [illegible] |
| ASV7049 | 405 | 1 | 1 | 405 | 100 | NR_102884.1 Ruminococcus champanellensis 18P13 = JCM 17042 16S ribosomal RNA, partial sequence | NR_102884.1 | 1494 | 348 | 749 | 26.91 | 512.6 | 6E-145 | 363/405 | 89 | [illegible] |
| ASV7050 | 404 | 2 | 1 | 395 | 97.77 | NR_147370.1 Pseudoflavonifractor phocaeensis strain Marseille-P3064 16S ribosomal RNA, partial sequence | NR_147370.1 | 1495 | 366 | 761 | 26.49 | 403.7 | 4E-112 | 340/399 | 85 | [illegible] |
| ASV7051 | 405 | 1 | 4 | 405 | 99.26 | NR_029239.3 Clostridium chartatabidum strain 163 16S ribosomal RNA, partial sequence | NR_029239.3 | 1460 | 326 | 724 | 27.33 | 579.1 | 6E-165 | 373/402 | 92 | [illegible] |
| ASV7052 | 402 | 4 | 3 | 402 | 99.5 | NR_170495.1 Blautia faecicola strain KGMB01111 16S ribosomal RNA, complete sequence | NR_170495.1 | 1534 | 374 | 773 | 26.08 | 507.1 | 3E-143 | 358/400 | 89 | [illegible] |
| ASV7056 | 401 | 2 | 1 | 399 | 99.5 | NR_134091.1 Mobilitalea sibirica strain P3M-3 16S ribosomal RNA, partial sequence | NR_134091.1 | 1444 | 352 | 751 | 27.7 | 460.9 | 2E-129 | 352/402 | 87 | [illegible] |
| ASV7058 | 426 | 2 | 1 | 426 | 100 | NR_029352.1 Lactobacillus amylolyticus DSM 11664 strain LA 5 16S ribosomal RNA, partial sequence | NR_029352.1 | 1544 | 361 | 787 | 27.66 | 510.8 | 2E-144 | 377/427 | 88 | [illegible] |
| ASV7060 | 421 | 2 | 1 | 421 | 100 | NR_113073.1 Barnesiella intestinihominis strain JCM 15079 16S ribosomal RNA, partial sequence | NR_113073.1 | 1481 | 346 | 767 | 28.49 | 496 | 6E-140 | 374/425 | 88 | [illegible] |
| ASV7063 | 428 | 1 | 3 | 428 | 99.53 | NR_152054.1 Streptococcus dentiloxodontae strain NUM2404 16S ribosomal RNA, partial sequence | NR_152054.1 | 1550 | 367 | 791 | 27.42 | 448 | 2E-125 | 369/430 | 85 | [illegible] |
| ASV7064 | 402 | 1 | 1 | 402 | 100 | NR_028964.1 Clostridium colicanis strain 3WC2 16S ribosomal RNA, partial sequence | NR_028964.1 | 1435 | 330 | 731 | 28.01 | 555.1 | 9E-158 | 369/403 | 91 | [illegible] |
| ASV7065 | 402 | 1 | 3 | 402 | 99.5 | NR_104846.1 Gemmiger formicilis strain X2-56 16S ribosomal RNA, partial sequence | NR_104846.1 | 1426 | 307 | 706 | 28.05 | 568 | 1E-161 | 372/403 | 92 | [illegible] |
| ASV7069 | 428 | 1 | 1 | 427 | 99.77 | NR_102980.1 Megasphaera elsdenii DSM 20460 16S ribosomal RNA, partial sequence | NR_102980.1 | 1552 | 369 | 794 | 27.45 | 472 | 1E-132 | 375/432 | 86 | [illegible] |
| ASV7075 | 422 | 1 | 1 | 422 | 100 | NR_144747.1 Mediterranea massiliensis strain Marseille-P2645 16S ribosomal RNA, partial sequence | NR_144747.1 | 1491 | 354 | 775 | 28.3 | 564.3 | 2E-160 | 383/422 | 90 | [illegible] |
| ASV7077 | 403 | 1 | 3 | 403 | 99.5 | NR_144611.1 Flintibacter butyricus strain BLS21 16S ribosomal RNA, partial sequence | NR_144611.1 | 808 | 301 | 703 | 49.88 | 494.2 | 2E-139 | 358/403 | 88 | [illegible] |
| ASV7078 | 402 | 1 | 1 | 401 | 99.75 | NR_102884.1 Ruminococcus champanellensis 18P13 = JCM 17042 16S ribosomal RNA, partial sequence | NR_102884.1 | 1494 | 348 | 748 | 26.84 | 525.6 | 7E-149 | 362/401 | 90 | [illegible] |
| ASV7079 | 404 | 2 | 1 | 404 | 100 | NR_146698.1 Mageeibacillus indolicus strain 0009-5 16S ribosomal RNA, partial sequence | NR_146698.1 | 1480 | 340 | 744 | 27.36 | 431.4 | 2E-120 | 351/408 | 86 | [illegible] |
| ASV7081 | 428 | 1 | 1 | 427 | 99.77 | NR_157635.1 Megasphaera hexanoica strain MH 16S ribosomal RNA, partial sequence | NR_157635.1 | 1561 | 372 | 797 | 27.29 | 433.2 | 5E-121 | 365/429 | 85 | [illegible] |
| ASV7086 | 403 | 1 | 3 | 403 | 99.5 | NR_137408.1 Terrisporobacter petrolearius strain LAM0A37 16S ribosomal RNA, partial sequence | NR_137408.1 | 1405 | 309 | 707 | 28.4 | 573.6 | 3E-163 | 371/401 | 92 | [illegible] |
| ASV7087 | 422 | 1 | 1 | 422 | 100 | NR_145950.1 Streptococcus panodentis strain TKU50 16S ribosomal RNA, partial sequence | NR_145950.1 | 1471 | 337 | 763 | 29.03 | 501.6 | 1E-141 | 379/430 | 88 | [illegible] |
| ASV7089 | 402 | 2 | 1 | 401 | 99.75 | NR_125464.1 Anaerobacterium chartisolvens strain T-1-35 16S ribosomal RNA, partial sequence | NR_125464.1 | 1451 | 342 | 744 | 27.77 | 477.6 | 2E-134 | 357/405 | 88 | [illegible] |
| ASV7091 | 403 | 1 | 3 | 403 | 99.5 | NR_074613.1 [Eubacterium] eligens ATCC 27750 16S ribosomal RNA, partial sequence | NR_074613.1 | 1519 | 369 | 768 | 26.33 | 496 | 6E-140 | 361/405 | 89 | [illegible] |
| ASV7092 | 403 | 5 | 5 | 403 | 99.01 | NR_115693.1 Gracilibacter thermotolerans JW/YJL-S1 16S ribosomal RNA, partial sequence | NR_115693.1 | 1526 | 463 | 861 | 26.15 | 460.9 | 2E-129 | 350/400 | 87 | [illegible] |
| ASV7099 | 405 | 1 | 1 | 405 | 100 | NR_042074.1 Eubacterium pyruvativorans strain I-6 16S ribosomal RNA, partial sequence | NR_042074.1 | 1296 | 264 | 667 | 31.17 | 497.9 | 2E-140 | 362/407 | 88 | [illegible] |
| ASV7103 | 427 | 1 | 1 | 427 | 100 | NR_044362.1 Haloplasma contractile SSD-17B 16S ribosomal RNA, partial sequence | NR_044362.1 | 1345 | 322 | 748 | 31.75 | 462.8 | 6E-130 | 370/429 | 86 | [illegible] |
| ASV7105 | 402 | 1 | 3 | 402 | 99.5 | NR_148620.1 Lachnotalea glycerini strain : DLD10 16S ribosomal RNA, partial sequence | NR_148620.1 | 1207 | 214 | 613 | 33.14 | 512.6 | 6E-145 | 361/402 | 89 | [illegible] |
| ASV7106 | 404 | 1 | 5 | 404 | 99.01 | NR_144749.1 Ihubacter massiliensis strain Marseille-P2843 16S ribosomal RNA, partial sequence | NR_144749.1 | 1479 | 368 | 766 | 26.98 | 555.1 | 9E-158 | 367/400 | 91 | [illegible] |
| ASV7107 | 426 | 1 | 1 | 421 | 98.83 | NR_027526.1 Catenibacterium mitsuokai strain DSM 15897 16S ribosomal RNA, partial sequence | NR_027526.1 | 1485 | 345 | 763 | 28.22 | 605 | 1E-172 | 392/423 | 92 | [illegible] |
| ASV7108 | 422 | 4 | 1 | 422 | 100 | NR_144612.1 Frisingicoccus caecimuris strain PG-426-CC-1 16S ribosomal RNA, partial sequence | NR_144612.1 | 1448 | 367 | 768 | 27.76 | 440.6 | 3E-123 | 365/423 | 86 | [illegible] |
| ASV7109 | 401 | 1 | 3 | 401 | 99.5 | NR_044111.1 Selenomonas bovis strain WG 16S ribosomal RNA, partial sequence | NR_044111.1 | 1525 | 372 | 796 | 27.87 | 484.9 | 1E-136 | 376/426 | 88 | [illegible] |
| ASV7110 | 405 | 1 | 1 | 405 | 100 | NR_134005.1 Clostridium bornimense strain M2/40 16S ribosomal RNA, partial sequence | NR_134005.1 | 1363 | 284 | 685 | 29.49 | 595.7 | 5E-170 | 378/405 | 93 | [illegible] |
| ASV7112 | 428 | 2 | 1 | 428 | 100 | NR_113411.1 Prevotella copri DSM 18205 strain JCM 13464 16S ribosomal RNA, partial sequence | NR_113411.1 | 1493 | 358 | 779 | 28.27 | 464.6 | 2E-130 | 374/432 | 86 | [illegible] |
| ASV7115 | 402 | 1 | 1 | 402 | 100 | NR_108636.1 Natranaerovirga pectinivora strain AP3 16S ribosomal RNA, partial sequence | NR_108636.1 | 1497 | 365 | 766 | 26.85 | 499.7 | 4E-141 | 359/403 | 89 | [illegible] |
| ASV7119 | 403 | 3 | 3 | 403 | 99.5 | NR_151894.1 Anaerotaenia torta strain FH052 16S ribosomal RNA, partial sequence | NR_151894.1 | 1479 | 354 | 753 | 27.05 | 584.7 | 1E-166 | 373/401 | 93 | [illegible] |
| ASV7120 | 403 | 1 | 1 | 403 | 100 | NR_029160.1 Ruminococcus callidus strain ATCC 27760 16S ribosomal RNA, partial sequence | NR_029160.1 | 1371 | 310 | 711 | 29.32 | 533 | 4E-151 | 368/406 | 90 | [illegible] |
| ASV7121 | 427 | 5 | 1 | 427 | 100 | NR_125811.1 Lactobacillus psittaci 16S ribosomal RNA, partial sequence | NR_125811.1 | 1421 | 344 | 770 | 30.05 | 484.9 | 1E-136 | 375/430 | 87 | [illegible] |
| ASV7128 | 401 | 1 | 1 | 401 | 100 | NR_134781.1 Olsenella scatoligenes strain SK9K4 16S ribosomal RNA, partial sequence | NR_134781.1 | 1469 | 348 | 755 | 27.77 | 547.7 | 2E-155 | 374/410 | 91 | [illegible] |
| ASV7129 | 409 | 1 | 1 | 409 | 100 | NR_125677.1 Vallitalea pronyensis strain FatNI3 16S ribosomal RNA, partial sequence | NR_125677.1 | 1480 | 363 | 764 | 27.16 | 449.9 | 5E-126 | 358/412 | 86 | [illegible] |
| ASV7133 | 402 | 2 | 1 | 402 | 100 | NR_026336.1 Clostridium vincentii strain DSM 10228 16S ribosomal RNA, partial sequence | NR_026336.1 | 1464 | 328 | 729 | 27.46 | 616.1 | 4E-176 | 379/402 | 94 | [illegible] |
| ASV7134 | 405 | 1 | 5 | 405 | 99.01 | NR_118554.1 Intestinimonas butyriciproducens strain SRB-521-5-I 16S ribosomal RNA, partial sequence | NR_118554.1 | 1475 | 350 | 750 | 27.19 | 492.3 | 7E-139 | 356/401 | 88 | [illegible] |
| ASV7135 | 404 | 1 | 3 | 404 | 99.5 | NR_126170.1 Thermoclostridium caenicola strain EBR596 16S ribosomal RNA, partial sequence | NR_126170.1 | 1565 | 458 | 858 | 25.62 | 442.5 | 8E-124 | 350/404 | 86 | [illegible] |
| ASV7136 | 405 | 2 | 3 | 405 | 99.51 | NR_074629.1 Hungateiclostridium thermocellum strain ATCC 27405 16S ribosomal RNA, partial sequence | NR_074629.1 | 1521 | 360 | 762 | 26.5 | 468.3 | 1E-131 | 355/405 | 87 | [illegible] |
| ASV7137 | 402 | 2 | 3 | 402 | 99.5 | NR_042200.1 [Clostridium] asparagiforme strain N6 16S ribosomal RNA, partial sequence | NR_042200.1 | 1468 | 337 | 736 | 27.25 | 507.1 | 3E-143 | 360/402 | 89 | [illegible] |
| ASV7138 | 404 | 1 | 1 | 404 | 100 | NR_144749.1 Ihubacter massiliensis strain Marseille-P2843 16S ribosomal RNA, partial sequence | NR_144749.1 | 1479 | 364 | 766 | 27.25 | 496 | 6E-140 | 361/406 | 88 | [illegible] |
| ASV7143 | 426 | 3 | 3 | 426 | 99.53 | NR_041781.1 Streptococcus alactolyticus strain ATCC 43077 16S ribosomal RNA, partial sequence | NR_041781.1 | 1437 | 348 | 772 | 29.58 | 595.7 | 6E-170 | 392/426 | 92 | [illegible] |
| ASV7144 | 427 | 1 | 1 | 427 | 100 | NR_147375.1 Colidextribacter massiliensis strain Marseille-P3083 16S ribosomal RNA, partial sequence | NR_147375.1 | 1487 | 364 | 765 | 27.03 | 477.6 | 2E-134 | 376/428 | 87 | [illegible] |
| ASV7150 | 405 | 1 | 1 | 405 | 100 | NR_118554.1 Intestinimonas butyriciproducens strain SRB-521-5-I 16S ribosomal RNA, partial sequence | NR_118554.1 | 1475 | 346 | 750 | 27.46 | 488.6 | 1E-137 | 359/406 | 88 | [illegible] |
| ASV7151 | 405 | 1 | 1 | 405 | 100 | NR_025127.1 Lachnobacterium bovis strain LRC 5382 16S ribosomal RNA, partial sequence | NR_025127.1 | 1453 | 362 | 765 | 27.8 | 497.9 | 2E-140 | 360/405 | 88 | [illegible] |
| ASV7152 | 405 | 1 | 1 | 405 | 100 | NR_144611.1 Flintibacter butyricus strain BLS21 16S ribosomal RNA, partial sequence | NR_144611.1 | 808 | 299 | 703 | 50.12 | 499.7 | 4E-141 | 362/407 | 88 | [illegible] |
| ASV7153 | 404 | 1 | 1 | 404 | 100 | NR_147370.1 Pseudoflavonifractor phocaeensis strain Marseille-P3064 16S ribosomal RNA, partial sequence | NR_147370.1 | 1495 | 366 | 770 | 27.09 | 564.3 | 2E-160 | 372/405 | 91 | [illegible] |
| ASV7155 | 427 | 3 | 1 | 427 | 100 | NR_117597.1 Selenomonas lacticifex strain DSM 20757 16S ribosomal RNA, partial sequence | NR_117597.1 | 1549 | 364 | 790 | 27.57 | 545.9 | 6E-155 | 388/432 | 89 | [illegible] |
| ASV7159 | 427 | 2 | 1 | 427 | 100 | NR_113772.1 Brochothrix campestris strain NBRC 15547 16S ribosomal RNA, partial sequence | NR_113772.1 | 1473 | 338 | 764 | 28.99 | 473.9 | 3E-133 | 373/430 | 86 | [illegible] |
| ASV7164 | 403 | 1 | 1 | 403 | 100 | NR_024994.1 Lactobacillus mucosae strain S32 16S ribosomal RNA, partial sequence | NR_024994.1 | 1568 | 384 | 810 | 27.23 | 540.3 | 3E-153 | 386/427 | 90 | [illegible] |
| ASV7166 | 427 | 2 | 3 | 427 | 99.53 | NR_037018.1 Acidaminococcus fermentans strain VR4 16S ribosomal RNA gene, partial sequence | NR_037018.1 | 1516 | 351 | 775 | 28.03 | 603.1 | 3E-172 | 395/428 | 92 | [illegible] |
| ASV7167 | 426 | 1 | 1 | 426 | 100 | NR_102980.1 Megasphaera elsdenii DSM 20460 16S ribosomal RNA, partial sequence | NR_102980.1 | 1552 | 369 | 795 | 27.51 | 505.3 | 1E-142 | 380/431 | 88 | [illegible] |
| ASV7169 | 428 | 4 | 3 | 428 | 99.53 | NR_042802.1 Lactobacillus ultunensis strain Kx146C1 16S ribosomal RNA, partial sequence | NR_042802.1 | 1550 | 372 | 796 | 27.42 | 547.7 | 2E-155 | 385/428 | 89 | [illegible] |
| ASV7171 | 427 | 2 | 1 | 427 | 100 | NR_113393.1 Peptococcus niger strain JCM 6506 16S ribosomal RNA, partial sequence | NR_113393.1 | 1528 | 374 | 800 | 27.95 | 496 | 6E-140 | 375/428 | 87 | [illegible] |
| ASV7173 | 421 | 2 | 1 | 421 | 100 | NR_041464.1 Parabacteroides johnsonii strain M-165 16S ribosomal RNA, partial sequence | NR_041464.1 | 1492 | 357 | 778 | 28.28 | 512.6 | 6E-145 | 374/422 | 88 | [illegible] |
| ASV7182 | 405 | 2 | 1 | 405 | 100 | NR_144742.1 Christensenella massiliensis strain Marseille-P2438 16S ribosomal RNA, partial sequence | NR_144742.1 | 1499 | 369 | 773 | 27.02 | 472 | 1E-132 | 356/406 | 87 | [illegible] |
| ASV7185 | 427 | 1 | 1 | 427 | 100 | NR_137408.1 Terrisporobacter petrolearius strain LAM0A37 16S ribosomal RNA, partial sequence | NR_137408.1 | 1405 | 307 | 707 | 28.54 | 538.5 | 1E-152 | 386/427 | 90 | [illegible] |
| ASV7188 | 402 | 4 | 1 | 402 | 100 | NR_118511.1 Campylobacter coli strain ATCC 33559 16S ribosomal RNA, partial sequence | NR_118511.1 | 1435 | 329 | 731 | 28.08 | 533 | 4E-151 | 367/405 | 90 | [illegible] |
| ASV7190 | 405 | 4 | 1 | 405 | 100 | NR_074399.1 Ruminococcus albus 7 = DSM 20455 16S ribosomal RNA, partial sequence | NR_074399.1 | 1500 | 350 | 751 | 26.8 | 529.3 | 6E-150 | 366/405 | 90 | [illegible] |
| ASV7195 | 427 | 1 | 1 | 427 | 100 | NR_025600.1 Sutterella stercoricanis strain 5BAC4 16S ribosomal RNA, partial sequence | NR_025600.1 | 1455 | 319 | 745 | 29.35 | 507.1 | 3E-143 | 377/428 | 88 | [illegible] |
| ASV7200 | 426 | 1 | 4 | 426 | 99.3 | NR_102980.1 Megasphaera elsdenii DSM 20460 16S ribosomal RNA, partial sequence | NR_102980.1 | 1552 | 372 | 795 | 27.32 | 560.7 | 2E-159 | 388/428 | 90 | [illegible] |
| ASV7202 | 405 | 1 | 5 | 405 | 99.01 | NR_152688.1 Falcatimonas natans strain WN011 16S ribosomal RNA, partial sequence | NR_152688.1 | 1484 | 369 | 766 | 26.82 | 527.4 | 2E-149 | 363/401 | 90 | [illegible] |
| ASV7206 | 402 | 1 | 1 | 402 | 100 | NR_044624.1 [Clostridium] cellulosi strain AS 1.1777 16S ribosomal RNA, partial sequence | NR_044624.1 | 1500 | 344 | 748 | 27 | 512.6 | 6E-145 | 367/409 | 89 | [illegible] |
| ASV7207 | 404 | 1 | 1 | 404 | 100 | NR_114306.1 Aerosakkonema funiforme strain Lao26 16S ribosomal RNA, partial sequence | NR_114306.1 | 1338 | 223 | 627 | 30.27 | 425.9 | 8E-119 | 348/406 | 85 | [illegible] |
| ASV7209 | 403 | 2 | 1 | 403 | 100 | NR_148620.1 Lachnotalea glycerini strain : DLD10 16S ribosomal RNA, partial sequence | NR_148620.1 | 1207 | 212 | 613 | 33.31 | 538.5 | 9E-153 | 367/404 | 90 | [illegible] |
| ASV7210 | 427 | 1 | 1 | 426 | 99.77 | NR_102980.1 Megasphaera elsdenii DSM 20460 16S ribosomal RNA, partial sequence | NR_102980.1 | 1552 | 369 | 794 | 27.45 | 483.1 | 5E-136 | 373/428 | 87 | [illegible] |
| ASV7212 | 427 | 1 | 1 | 427 | 100 | NR_028840.1 Mitsuokella jalaludinii strain M9 16S ribosomal RNA, partial sequence | NR_028840.1 | 1543 | 362 | 788 | 27.67 | 678.8 | 0 | 407/427 | 95 | [illegible] |
| ASV7213 | 402 | 1 | 3 | 402 | 99.5 | NR_134772.1 Murimonas intestini strain SRB-530-5-H 16S ribosomal RNA, partial sequence | NR_134772.1 | 1433 | 316 | 715 | 27.91 | 518.2 | 1E-146 | 362/402 | 90 | [illegible] |
| ASV7215 | 409 | 1 | 15 | 409 | 96.58 | NR_144605.1 Acutalibacter muris strain KB18 16S ribosomal RNA, partial sequence | NR_144605.1 | 1510 | 398 | 785 | 25.7 | 484.9 | 1E-136 | 353/396 | 89 | [illegible] |
| ASV7218 | 402 | 1 | 1 | 402 | 100 | NR_074809.1 Roseburia hominis A2-183 16S ribosomal RNA, partial sequence | NR_074809.1 | 1535 | 367 | 769 | 26.25 | 555.1 | 9E-158 | 371/405 | 91 | [illegible] |
| ASV7221 | 422 | 1 | 1 | 413 | 97.87 | NR_041364.1 Prevotella stercorea DSM 18206 strain CB35 16S ribosomal RNA, partial sequence | NR_041364.1 | 1488 | 354 | 766 | 27.76 | 614.2 | 2E-175 | 387/414 | 93 | [illegible] |
| ASV7223 | 428 | 1 | 5 | 428 | 99.07 | NR_029196.1 Chlamydia suis strain S45 16S ribosomal RNA, partial sequence | NR_029196.1 | 1545 | 362 | 784 | 27.38 | 521.9 | 1E-147 | 381/428 | 89 | [illegible] |
| ASV7227 | 426 | 1 | 3 | 425 | 99.3 | NR_102980.1 Megasphaera elsdenii DSM 20460 16S ribosomal RNA, partial sequence | NR_102980.1 | 1552 | 371 | 794 | 27.32 | 549.6 | 5E-156 | 384/426 | 90 | [illegible] |
| ASV7228 | 422 | 4 | 1 | 422 | 100 | NR_044864.1 Hallella seregens ATCC 51272 16S ribosomal RNA, partial sequence | NR_044864.1 | 1463 | 335 | 756 | 28.84 | 492.3 | 8E-139 | 372/424 | 87 | [illegible] |
| ASV7230 | 411 | 2 | 3 | 411 | 99.51 | NR_144696.1 Clostridium saudiense strain JCC 16S ribosomal RNA, partial sequence | NR_144696.1 | 1478 | 352 | 751 | 27.06 | 475.7 | 8E-134 | 360/409 | 88 | [illegible] |
| ASV7232 | 404 | 1 | 38 | 404 | 90.84 | NR_025930.1 Ruminococcus bromii strain ATCC 27255 16S ribosomal RNA, partial sequence | NR_025930.1 | 1380 | 349 | 716 | 26.67 | 534.8 | 1E-151 | 343/369 | 92 | [illegible] |
| ASV7233 | 402 | 3 | 5 | 402 | 99 | NR_144724.1 Desnuesiella massiliensis strain mt10 16S ribosomal RNA, partial sequence | NR_144724.1 | 1465 | 344 | 741 | 27.17 | 508.9 | 7E-144 | 357/398 | 89 | [illegible] |
| ASV7239 | 427 | 2 | 3 | 427 | 99.53 | NR_029352.1 Lactobacillus amylolyticus DSM 11664 strain LA 5 16S ribosomal RNA, partial sequence | NR_029352.1 | 1544 | 363 | 787 | 27.53 | 497.9 | 2E-140 | 375/427 | 87 | [illegible] |
| ASV7240 | 405 | 4 | 29 | 405 | 93.09 | NR_118156.1 Oscillibacter ruminantium GH1 16S ribosomal RNA, partial sequence | NR_118156.1 | 1409 | 349 | 724 | 26.69 | 473.9 | 3E-133 | 337/377 | 89 | [illegible] |
| ASV7243 | 427 | 1 | 15 | 427 | 96.72 | NR_041342.1 Parabacteroides distasonis strain JCM 5825 16S ribosomal RNA, partial sequence | NR_041342.1 | 1488 | 367 | 774 | 27.42 | 414.8 | 2E-115 | 354/416 | 85 | [illegible] |
| ASV7244 | 405 | 1 | 4 | 405 | 99.26 | NR_144742.1 Christensenella massiliensis strain Marseille-P2438 16S ribosomal RNA, partial sequence | NR_144742.1 | 1499 | 372 | 773 | 26.82 | 466.5 | 4E-131 | 352/402 | 87 | [illegible] |
| ASV7246 | 428 | 1 | 1 | 428 | 100 | NR_028816.1 Turicibacter sanguinis strain MOL361 16S ribosomal RNA, partial sequence | NR_028816.1 | 1508 | 327 | 753 | 28.32 | 640.1 | 0 | 403/430 | 93 | [illegible] |
| ASV7250 | 405 | 2 | 1 | 388 | 95.8 | NR_074629.1 Hungateiclostridium thermocellum strain ATCC 27405 16S ribosomal RNA, partial sequence | NR_074629.1 | 1521 | 358 | 745 | 25.51 | 429.5 | 6E-120 | 337/389 | 86 | [illegible] |
| ASV7251 | 421 | 2 | 1 | 421 | 100 | NR_113073.1 Barnesiella intestinihominis strain JCM 15079 16S ribosomal RNA, partial sequence | NR_113073.1 | 1481 | 346 | 767 | 28.49 | 484.9 | 1E-136 | 373/426 | 87 | [illegible] |
| ASV7255 | 426 | 5 | 1 | 426 | 100 | NR_041781.1 Streptococcus alactolyticus strain ATCC 43077 16S ribosomal RNA, partial sequence | NR_041781.1 | 1437 | 346 | 772 | 29.71 | 505.3 | 1E-142 | 379/430 | 88 | [illegible] |
| ASV7256 | 402 | 1 | 3 | 402 | 99.5 | NR_136763.1 Herbinix hemicellulosilytica strain T3/55 16S ribosomal RNA, partial sequence | NR_136763.1 | 1523 | 377 | 776 | 26.26 | 499.7 | 4E-141 | 357/401 | 89 | [illegible] |
| ASV7257 | 402 | 1 | 1 | 387 | 96.27 | NR_025931.1 Ruminococcus flavefaciens strain C94 16S ribosomal RNA, partial sequence | NR_025931.1 | 1388 | 325 | 711 | 27.88 | 433.2 | 5E-121 | 338/389 | 86 | [illegible] |
| ASV7258 | 403 | 1 | 1 | 402 | 99.75 | NR_144611.1 Flintibacter butyricus strain BLS21 16S ribosomal RNA, partial sequence | NR_144611.1 | 808 | 299 | 702 | 50 | 490.5 | 3E-138 | 359/405 | 88 | [illegible] |
| ASV7261 | 401 | 1 | 3 | 399 | 99 | NR_044503.1 Thermotalea metallivorans strain B2-1 16S ribosomal RNA, partial sequence | NR_044503.1 | 1627 | 469 | 866 | 24.46 | 451.7 | 1E-126 | 348/399 | 87 | [illegible] |
| ASV7263 | 423 | 1 | 1 | 423 | 100 | NR_113411.1 Prevotella copri DSM 18205 strain JCM 13464 16S ribosomal RNA, partial sequence | NR_113411.1 | 1493 | 358 | 779 | 28.27 | 636.4 | 0 | 399/425 | 93 | [illegible] |
| ASV7264 | 404 | 1 | 3 | 404 | 99.5 | NR_028883.1 Dorea longicatena strain 111-35 16S ribosomal RNA, partial sequence | NR_028883.1 | 1316 | 245 | 645 | 30.47 | 569.9 | 3E-162 | 374/405 | 92 | [illegible] |
| ASV7266 | 403 | 1 | 3 | 403 | 99.5 | NR_026476.1 Succinivibrio dextrinosolvens strain 0554 16S ribosomal RNA, partial sequence | NR_026476.1 | 1463 | 333 | 732 | 27.34 | 468.3 | 1E-131 | 353/402 | 87 | [illegible] |
| ASV7267 | 404 | 1 | 15 | 404 | 96.53 | NR_029167.1 Anaeroplasma abactoclasticum strain 6-1 16S ribosomal RNA, partial sequence | NR_029167.1 | 1446 | 358 | 751 | 27.25 | 427.7 | 2E-119 | 341/395 | 86 | [illegible] |
| ASV7270 | 428 | 1 | 3 | 428 | 99.53 | NR_132294.1 Texcoconibacillus texcoconensis strain 13CC 16S ribosomal RNA, partial sequence | NR_132294.1 | 1374 | 300 | 725 | 31 | 477.6 | 2E-134 | 372/428 | 86 | [illegible] |
| ASV7271 | 403 | 1 | 3 | 403 | 99.5 | NR_147375.1 Colidextribacter massiliensis strain Marseille-P3083 16S ribosomal RNA, partial sequence | NR_147375.1 | 1487 | 366 | 765 | 26.9 | 518.2 | 1E-146 | 362/402 | 90 | [illegible] |
| ASV7274 | 405 | 1 | 1 | 405 | 100 | NR_024919.1 Clostridium frigidicarnis strain SPL77A 16S ribosomal RNA, partial sequence | NR_024919.1 | 1481 | 346 | 747 | 27.14 | 529.3 | 6E-150 | 368/407 | 90 | [illegible] |
| ASV7277 | 402 | 1 | 1 | 402 | 100 | NR_118156.1 Oscillibacter ruminantium GH1 16S ribosomal RNA, partial sequence | NR_118156.1 | 1409 | 321 | 724 | 28.67 | 512.6 | 6E-145 | 363/405 | 89 | [illegible] |
| ASV7278 | 427 | 2 | 3 | 427 | 99.53 | NR_117574.1 Lactobacillus johnsonii strain CIP 103620 16S ribosomal RNA, partial sequence | NR_117574.1 | 1543 | 368 | 792 | 27.54 | 608.7 | 7E-174 | 395/427 | 92 | [illegible] |
| ASV7283 | 404 | 1 | 1 | 404 | 100 | NR_147375.1 Colidextribacter massiliensis strain Marseille-P3083 16S ribosomal RNA, partial sequence | NR_147375.1 | 1487 | 364 | 765 | 27.03 | 501.6 | 1E-141 | 361/405 | 89 | [illegible] |
| ASV7284 | 422 | 2 | 1 | 422 | 100 | NR_028997.1 Subdoligranulum variabile strain BI 114 16S ribosomal RNA, partial sequence | NR_028997.1 | 1428 | 320 | 722 | 28.22 | 457.2 | 3E-128 | 369/424 | 87 | [illegible] |
| ASV7285 | 402 | 1 | 3 | 402 | 99.5 | NR_118156.1 Oscillibacter ruminantium GH1 16S ribosomal RNA, partial sequence | NR_118156.1 | 1409 | 323 | 724 | 28.53 | 492.3 | 7E-139 | 358/403 | 88 | [illegible] |
| ASV7286 | 427 | 3 | 1 | 427 | 100 | NR_144696.1 Clostridium saudiense strain JCC 16S ribosomal RNA, partial sequence | NR_144696.1 | 1478 | 350 | 751 | 27.2 | 521.9 | 1E-147 | 384/428 | 89 | [illegible] |
| ASV7289 | 404 | 1 | 1 | 404 | 100 | NR_125464.1 Anaerobacterium chartisolvens strain T-1-35 16S ribosomal RNA, partial sequence | NR_125464.1 | 1451 | 342 | 745 | 27.84 | 475.7 | 7E-134 | 355/404 | 87 | [illegible] |
| ASV7290 | 427 | 3 | 4 | 427 | 99.3 | NR_041781.1 Streptococcus alactolyticus strain ATCC 43077 16S ribosomal RNA, partial sequence | NR_041781.1 | 1437 | 349 | 772 | 29.51 | 595.7 | 6E-170 | 393/427 | 92 | [illegible] |
| ASV7294 | 422 | 2 | 1 | 422 | 100 | NR_040866.1 Bacteroides uniformis strain JCM 5828 16S ribosomal RNA, partial sequence | NR_040866.1 | 1425 | 351 | 772 | 29.61 | 453.5 | 4E-127 | 367/426 | 86 | [illegible] |
| ASV7295 | 404 | 1 | 1 | 404 | 100 | NR_104907.1 Eubacterium coprostanoligenes strain HL 16S ribosomal RNA, partial sequence | NR_104907.1 | 1446 | 332 | 734 | 27.87 | 507.1 | 3E-143 | 363/406 | 89 | [illegible] |
| ASV7300 | 403 | 1 | 3 | 403 | 99.5 | NR_026312.1 Blautia schinkii strain B 16S ribosomal RNA, partial sequence | NR_026312.1 | 1482 | 346 | 745 | 26.99 | 497.9 | 2E-140 | 358/402 | 89 | [illegible] |
| ASV7307 | 403 | 1 | 1 | 403 | 100 | NR_118554.1 Intestinimonas butyriciproducens strain SRB-521-5-I 16S ribosomal RNA, partial sequence | NR_118554.1 | 1475 | 346 | 750 | 27.46 | 492.3 | 7E-139 | 360/406 | 88 | [illegible] |
| ASV7308 | 402 | 1 | 3 | 402 | 99.5 | NR_144737.1 Emergencia timonensis strain SN18 16S ribosomal RNA, partial sequence | NR_144737.1 | 1480 | 366 | 767 | 27.16 | 536.6 | 3E-152 | 365/402 | 90 | [illegible] |
| ASV7309 | 405 | 1 | 3 | 405 | 99.51 | NR_029097.1 Oribacterium sinus strain AIP 354.02 16S ribosomal RNA, partial sequence | NR_029097.1 | 1374 | 310 | 709 | 29.11 | 486.8 | 3E-137 | 359/405 | 88 | [illegible] |
| ASV7311 | 420 | 1 | 1 | 420 | 100 | NR_144737.1 Emergencia timonensis strain SN18 16S ribosomal RNA, partial sequence | NR_144737.1 | 1480 | 364 | 767 | 27.3 | 446.2 | 6E-125 | 365/422 | 86 | [illegible] |
| ASV7312 | 403 | 2 | 1 | 403 | 100 | NR_119074.1 Terrisporobacter glycolicus ATCC 14880 = DSM 1288 16S ribosomal RNA, partial sequence | NR_119074.1 | 1461 | 314 | 714 | 27.45 | 444.3 | 2E-124 | 349/403 | 86 | [illegible] |
| ASV7316 | 404 | 2 | 3 | 404 | 99.5 | NR_118554.1 Intestinimonas butyriciproducens strain SRB-521-5-I 16S ribosomal RNA, partial sequence | NR_118554.1 | 1475 | 348 | 750 | 27.32 | 555.1 | 9E-158 | 369/403 | 91 | [illegible] |
| ASV7317 | 427 | 5 | 1 | 427 | 100 | NR_134772.1 Murimonas intestini strain SRB-530-5-H 16S ribosomal RNA, partial sequence | NR_134772.1 | 1433 | 314 | 715 | 28.05 | 466.5 | 5E-131 | 373/427 | 87 | [illegible] |
| ASV7319 | 403 | 4 | 3 | 403 | 99.5 | NR_118511.1 Campylobacter coli strain ATCC 33559 16S ribosomal RNA, partial sequence | NR_118511.1 | 1435 | 331 | 731 | 27.94 | 630.8 | 1E-180 | 381/401 | 95 | [illegible] |
| ASV7324 | 404 | 2 | 1 | 404 | 100 | NR_074793.2 Oscillibacter valericigenes strain Sjm18-20 16S ribosomal RNA, complete sequence | NR_074793.2 | 1530 | 372 | 775 | 26.41 | 608.7 | 7E-174 | 379/404 | 93 | [illegible] |
| ASV7326 | 405 | 1 | 3 | 405 | 99.51 | NR_144743.1 Christensenella timonensis strain Marseille-P2437 16S ribosomal RNA, partial sequence | NR_144743.1 | 1498 | 371 | 773 | 26.9 | 468.3 | 1E-131 | 354/404 | 87 | [illegible] |
| ASV7330 | 428 | 1 | 1 | 428 | 100 | NR_025670.1 Pseudoflavonifractor capillosus ATCC 29799 16S ribosomal RNA, partial sequence | NR_025670.1 | 1483 | 346 | 750 | 27.31 | 464.6 | 2E-130 | 373/428 | 87 | [illegible] |
| ASV7332 | 404 | 1 | 1 | 404 | 100 | NR_108636.1 Natranaerovirga pectinivora strain AP3 16S ribosomal RNA, partial sequence | NR_108636.1 | 1497 | 365 | 766 | 26.85 | 529.3 | 6E-150 | 365/404 | 90 | [illegible] |
| ASV7337 | 404 | 1 | 1 | 404 | 100 | NR_134005.1 Clostridium bornimense strain M2/40 16S ribosomal RNA, partial sequence | NR_134005.1 | 1363 | 284 | 685 | 29.49 | 551.4 | 1E-156 | 370/405 | 91 | [illegible] |
| ASV7341 | 403 | 1 | 1 | 403 | 100 | NR_118156.1 Oscillibacter ruminantium GH1 16S ribosomal RNA, partial sequence | NR_118156.1 | 1409 | 321 | 724 | 28.67 | 462.8 | 6E-130 | 353/404 | 87 | [illegible] |
| ASV7345 | 402 | 1 | 5 | 402 | 99 | NR_026476.1 Succinivibrio dextrinosolvens strain 0554 16S ribosomal RNA, partial sequence | NR_026476.1 | 1463 | 335 | 732 | 27.2 | 569.9 | 3E-162 | 368/398 | 92 | [illegible] |
| ASV7351 | 402 | 2 | 1 | 402 | 100 | NR_144697.1 Clostridium jeddahense strain JCD 16S ribosomal RNA, partial sequence | NR_144697.1 | 1387 | 266 | 667 | 28.98 | 455.4 | 1E-127 | 352/404 | 87 | [illegible] |
| ASV7354 | 404 | 1 | 1 | 404 | 100 | NR_144737.1 Emergencia timonensis strain SN18 16S ribosomal RNA, partial sequence | NR_144737.1 | 1480 | 364 | 767 | 27.3 | 514.5 | 2E-145 | 364/406 | 89 | [illegible] |
| ASV7355 | 402 | 1 | 1 | 393 | 97.76 | NR_118673.1 [Eubacterium] hallii strain ATCC 27751 16S ribosomal RNA, partial sequence | NR_118673.1 | 1479 | 371 | 763 | 26.57 | 409.2 | 8E-114 | 338/396 | 85 | [illegible] |
| ASV7356 | 422 | 1 | 1 | 420 | 99.53 | NR_113208.1 Parabacteroides chinchillae strain JCM 17104 16S ribosomal RNA, partial sequence | NR_113208.1 | 1489 | 353 | 772 | 28.21 | 566.2 | 5E-161 | 383/421 | 90 | [illegible] |
| ASV7363 | 403 | 3 | 3 | 403 | 99.5 | NR_074629.1 Hungateiclostridium thermocellum strain ATCC 27405 16S ribosomal RNA, partial sequence | NR_074629.1 | 1521 | 360 | 762 | 26.5 | 483.1 | 4E-136 | 356/403 | 88 | [illegible] |
| ASV7364 | 427 | 2 | 3 | 427 | 99.53 | NR_133029.1 Bacillus massiliogorillae strain G2 16S ribosomal RNA, partial sequence | NR_133029.1 | 1507 | 359 | 783 | 28.2 | 520 | 4E-147 | 381/429 | 88 | [illegible] |
| ASV7366 | 401 | 1 | 3 | 399 | 99 | NR_044265.1 Ruminococcus gauvreauii strain CCRI-16110 16S ribosomal RNA, partial sequence | NR_044265.1 | 1456 | 349 | 746 | 27.34 | 451.7 | 1E-126 | 347/398 | 87 | [illegible] |
| ASV7367 | 402 | 1 | 1 | 402 | 100 | NR_137408.1 Terrisporobacter petrolearius strain LAM0A37 16S ribosomal RNA, partial sequence | NR_137408.1 | 1405 | 307 | 707 | 28.54 | 508.9 | 7E-144 | 362/404 | 89 | [illegible] |
| ASV7370 | 402 | 1 | 1 | 402 | 100 | NR_041236.1 Lutispora thermophila DSM 19022 strain EBR46 16S ribosomal RNA, partial sequence | NR_041236.1 | 1438 | 338 | 740 | 28.03 | 494.2 | 2E-139 | 360/405 | 88 | [illegible] |
| ASV7371 | 401 | 1 | 1 | 401 | 100 | NR_119085.1 [Clostridium] polysaccharolyticum strain DSM 1801 16S ribosomal RNA, partial sequence | NR_119085.1 | 1503 | 364 | 763 | 26.61 | 457.2 | 3E-128 | 353/404 | 87 | [illegible] |
| ASV7372 | 427 | 1 | 1 | 427 | 100 | NR_041611.1 Lactobacillus ruminis strain NBRC 102161 16S ribosomal RNA, partial sequence | NR_041611.1 | 1488 | 352 | 778 | 28.7 | 501.6 | 1E-141 | 376/428 | 87 | [illegible] |
| ASV7373 | 405 | 2 | 3 | 405 | 99.51 | NR_114585.1 Helicobacter muridarum strain ATCC 49282 16S ribosomal RNA, partial sequence | NR_114585.1 | 1440 | 315 | 714 | 27.78 | 586.5 | 3E-167 | 375/403 | 93 | [illegible] |
| ASV7375 | 405 | 1 | 1 | 404 | 99.75 | NR_152684.1 Hungateiclostridium saccincola strain GGR1 16S ribosomal RNA, partial sequence | NR_152684.1 | 1421 | 306 | 707 | 28.29 | 462.8 | 6E-130 | 356/407 | 87 | [illegible] |
| ASV7376 | 401 | 1 | 3 | 400 | 99.25 | NR_041236.1 Lutispora thermophila DSM 19022 strain EBR46 16S ribosomal RNA, partial sequence | NR_041236.1 | 1438 | 340 | 739 | 27.82 | 444.3 | 2E-124 | 347/400 | 86 | [illegible] |
| ASV7377 | 402 | 2 | 1 | 402 | 100 | NR_029160.1 Ruminococcus callidus strain ATCC 27760 16S ribosomal RNA, partial sequence | NR_029160.1 | 1371 | 310 | 711 | 29.32 | 533 | 4E-151 | 366/404 | 90 | [illegible] |
| ASV7378 | 405 | 1 | 3 | 405 | 99.51 | NR_144611.1 Flintibacter butyricus strain BLS21 16S ribosomal RNA, partial sequence | NR_144611.1 | 808 | 301 | 703 | 49.88 | 507.1 | 3E-143 | 362/405 | 89 | [illegible] |
| ASV7381 | 405 | 1 | 3 | 405 | 99.51 | NR_148619.1 Agathobacter ruminis strain JK623 16S ribosomal RNA, partial sequence | NR_148619.1 | 1434 | 320 | 721 | 28.03 | 566.2 | 4E-161 | 373/405 | 92 | [illegible] |
| ASV7383 | 427 | 4 | 1 | 427 | 100 | NR_042802.1 Lactobacillus ultunensis strain Kx146C1 16S ribosomal RNA, partial sequence | NR_042802.1 | 1550 | 370 | 796 | 27.55 | 540.3 | 3E-153 | 384/429 | 89 | [illegible] |
| ASV7384 | 404 | 1 | 1 | 404 | 100 | NR_042200.1 [Clostridium] asparagiforme strain N6 16S ribosomal RNA, partial sequence | NR_042200.1 | 1468 | 335 | 736 | 27.38 | 440.6 | 3E-123 | 350/405 | 86 | [illegible] |
| ASV7385 | 427 | 4 | 1 | 427 | 100 | NR_043231.1 Dialister propionicifaciens strain ADV 1053.03 16S ribosomal RNA, partial sequence | NR_043231.1 | 1396 | 300 | 726 | 30.59 | 484.9 | 1E-136 | 376/431 | 87 | [illegible] |
| ASV7386 | 428 | 3 | 1 | 428 | 100 | NR_144696.1 Clostridium saudiense strain JCC 16S ribosomal RNA, partial sequence | NR_144696.1 | 1478 | 350 | 751 | 27.2 | 523.7 | 3E-148 | 384/428 | 89 | [illegible] |
| ASV7389 | 420 | 1 | 1 | 420 | 100 | NR_102884.1 Ruminococcus champanellensis 18P13 = JCM 17042 16S ribosomal RNA, partial sequence | NR_102884.1 | 1494 | 348 | 749 | 26.91 | 483.1 | 5E-136 | 370/420 | 88 | [illegible] |
| ASV7395 | 404 | 1 | 1 | 404 | 100 | NR_144737.1 Emergencia timonensis strain SN18 16S ribosomal RNA, partial sequence | NR_144737.1 | 1480 | 364 | 767 | 27.3 | 575.4 | 7E-164 | 373/404 | 92 | [illegible] |
| ASV7400 | 427 | 1 | 3 | 427 | 99.53 | NR_044111.1 Selenomonas bovis strain WG 16S ribosomal RNA, partial sequence | NR_044111.1 | 1525 | 372 | 796 | 27.87 | 608.7 | 7E-174 | 394/426 | 92 | [illegible] |
| ASV7403 | 427 | 1 | 1 | 427 | 100 | NR_041666.1 Dialister succinatiphilus YIT 11850 16S ribosomal RNA, partial sequence | NR_041666.1 | 1521 | 353 | 779 | 28.07 | 557 | 3E-158 | 388/430 | 90 | [illegible] |
| ASV7405 | 418 | 2 | 1 | 418 | 100 | NR_119275.1 Lactobacillus nagelii strain LuE10 16S ribosomal RNA, partial sequence | NR_119275.1 | 1100 | 358 | 784 | 38.82 | 608.7 | 7E-174 | 398/429 | 92 | [illegible] |
| ASV7407 | 427 | 4 | 1 | 427 | 100 | NR_074414.1 Olsenella uli DSM 7084 16S ribosomal RNA, partial sequence | NR_074414.1 | 1503 | 348 | 755 | 27.15 | 494.2 | 2E-139 | 378/428 | 88 | [illegible] |
| ASV7409 | 405 | 3 | 1 | 405 | 100 | NR_043437.1 Bifidobacterium longum subsp. infantis strain ATCC 15697 16S ribosomal RNA, partial sequence | NR_043437.1 | 1515 | 345 | 751 | 26.86 | 579.1 | 6E-165 | 376/407 | 92 | [illegible] |
| ASV7410 | 426 | 2 | 3 | 426 | 99.53 | NR_113306.1 Megasphaera elsdenii DSM 20460 16S ribosomal RNA, partial sequence | NR_113306.1 | 1174 | 234 | 658 | 36.2 | 529.3 | 6E-150 | 381/427 | 89 | [illegible] |
| ASV7412 | 427 | 1 | 1 | 426 | 99.77 | NR_028840.1 Mitsuokella jalaludinii strain M9 16S ribosomal RNA, partial sequence | NR_028840.1 | 1543 | 362 | 787 | 27.61 | 660.4 | 0 | 404/427 | 94 | [illegible] |
| ASV7413 | 428 | 1 | 3 | 428 | 99.53 | NR_117574.1 Lactobacillus johnsonii strain CIP 103620 16S ribosomal RNA, partial sequence | NR_117574.1 | 1543 | 368 | 792 | 27.54 | 553.3 | 4E-157 | 387/429 | 90 | [illegible] |
| ASV7415 | 417 | 1 | 1 | 417 | 100 | NR_102893.1 Butyrivibrio proteoclasticus strain B316 16S ribosomal RNA, partial sequence | NR_102893.1 | 1537 | 367 | 782 | 27.07 | 436.9 | 4E-122 | 359/419 | 85 | [illegible] |
| ASV7418 | 405 | 1 | 1 | 405 | 100 | NR_144611.1 Flintibacter butyricus strain BLS21 16S ribosomal RNA, partial sequence | NR_144611.1 | 808 | 299 | 703 | 50.12 | 521.9 | 9E-148 | 364/405 | 89 | [illegible] |
| ASV7419 | 402 | 1 | 1 | 402 | 100 | NR_028773.1 Bulleidia extructa strain W1219 16S ribosomal RNA, partial sequence | NR_028773.1 | 1479 | 331 | 751 | 28.47 | 411.1 | 2E-114 | 359/422 | 85 | [illegible] |
| ASV7421 | 402 | 1 | 1 | 402 | 100 | NR_028961.1 Faecalibacterium prausnitzii strain ATCC 27768 16S ribosomal RNA, partial sequence | NR_028961.1 | 1462 | 344 | 745 | 27.5 | 610.5 | 2E-174 | 379/403 | 94 | [illegible] |
| ASV7424 | 403 | 1 | 3 | 403 | 99.5 | NR_044490.1 Butyricicoccus pullicaecorum strain 25-3 16S ribosomal RNA, partial sequence | NR_044490.1 | 1482 | 368 | 770 | 27.19 | 521.9 | 9E-148 | 363/403 | 90 | [illegible] |
| ASV7427 | 402 | 2 | 1 | 402 | 100 | NR_102987.1 Hungateiclostridium clariflavum DSM 19732 16S ribosomal RNA, partial sequence | NR_102987.1 | 1634 | 481 | 882 | 24.6 | 505.3 | 9E-143 | 359/402 | 89 | [illegible] |
| ASV7430 | 421 | 4 | 3 | 421 | 99.52 | NR_042802.1 Lactobacillus ultunensis strain Kx146C1 16S ribosomal RNA, partial sequence | NR_042802.1 | 1550 | 372 | 796 | 27.42 | 553.3 | 3E-157 | 385/426 | 90 | [illegible] |
| ASV7431 | 405 | 2 | 3 | 405 | 99.51 | NR_074629.1 Hungateiclostridium thermocellum strain ATCC 27405 16S ribosomal RNA, partial sequence | NR_074629.1 | 1521 | 360 | 762 | 26.5 | 490.5 | 3E-138 | 357/403 | 88 | [illegible] |
| ASV7433 | 422 | 1 | 1 | 422 | 100 | NR_041778.1 Desulfovibrio piger strain ATCC 29098 16S ribosomal RNA, partial sequence | NR_041778.1 | 1542 | 357 | 783 | 27.69 | 540.3 | 3E-153 | 384/428 | 89 | [illegible] |
| ASV7434 | 402 | 1 | 3 | 402 | 99.5 | NR_119034.1 Asaccharospora irregularis DSM 2635 16S ribosomal RNA, partial sequence | NR_119034.1 | 1489 | 339 | 736 | 26.73 | 416.6 | 5E-116 | 347/405 | 85 | [illegible] |
| ASV7436 | 402 | 3 | 3 | 402 | 99.5 | NR_074793.2 Oscillibacter valericigenes strain Sjm18-20 16S ribosomal RNA, complete sequence | NR_074793.2 | 1530 | 374 | 775 | 26.27 | 514.5 | 2E-145 | 362/403 | 89 | [illegible] |
| ASV7437 | 422 | 1 | 15 | 421 | 96.45 | NR_112037.1 Ruminiclostridium josui strain FERM P-9684 16S ribosomal RNA, partial sequence | NR_112037.1 | 1565 | 472 | 861 | 24.92 | 431.4 | 2E-120 | 352/407 | 86 | [illegible] |
| ASV7438 | 427 | 1 | 1 | 427 | 100 | NR_028816.1 Turicibacter sanguinis strain MOL361 16S ribosomal RNA, partial sequence | NR_028816.1 | 1508 | 327 | 753 | 28.32 | 606.8 | 3E-173 | 395/428 | 92 | [illegible] |
| ASV7440 | 401 | 2 | 1 | 399 | 99.5 | NR_134800.1 Romboutsia sedimentorum strain LAM201 16S ribosomal RNA, partial sequence | NR_134800.1 | 1339 | 272 | 670 | 29.8 | 472 | 1E-132 | 351/399 | 87 | [illegible] |
| ASV7441 | 404 | 1 | 1 | 402 | 99.5 | NR_144743.1 Christensenella timonensis strain Marseille-P2437 16S ribosomal RNA, partial sequence | NR_144743.1 | 1498 | 369 | 771 | 26.9 | 433.2 | 5E-121 | 348/404 | 86 | [illegible] |
| ASV7442 | 427 | 1 | 1 | 427 | 100 | NR_029160.1 Ruminococcus callidus strain ATCC 27760 16S ribosomal RNA, partial sequence | NR_029160.1 | 1371 | 310 | 711 | 29.32 | 483.1 | 5E-136 | 376/427 | 88 | [illegible] |

| | | | | | | | | | | | | | | | | | | | | | | | | | | | |
|---|---|---|---|---|---|---|---|---|---|---|---|---|---|---|---|---|---|---|---|---|---|---|---|---|---|---|---|
| ASV7445 | 428 | 1 | 1 | 427 | 99.77 | NR_112902.1 Phascolarctobacterium succinatutens strain YIT 12067 16S ribosomal RNA, partial sequence | NR_112902.1 | 1610 | 444 | 869 | 26.46 | 483.1 | 5E-136 | 372/427 | 87 | 1/427 | 0 | Plus | Plus | Bacteria; Firmicutes; Negativicutes; Acidaminococcales; Acidaminococcaceae; Phascolarctobacterium; Phascolarctobacterium succinatutens | Phascolarctobacterium succinatutens | Phascolarctobacterium | Acidaminococcaceae | Acidaminococcales | Negativicutes | Firmicutes | Bacteria |
| ASV7447 | 427 | 5 | 1 | 427 | 100 | NR_042052.1 Streptococcus equinus ATCC 9812 16S ribosomal RNA, partial sequence | NR_042052.1 | 1433 | 260 | 686 | 29.8 | 462.8 | 6E-130 | 371/430 | 86 | 6/430 | 1 | Plus | Plus | Bacteria; Firmicutes; Bacilli; Lactobacillales; Streptococcaceae; Streptococcus; Streptococcus equinus | Streptococcus equinus | Streptococcus | Streptococcaceae | Lactobacillales | Bacilli | Firmicutes | Bacteria |
| ASV7448 | 405 | 1 | 1 | 405 | 100 | NR_134005.1 Clostridium bornimense strain M2/40 16S ribosomal RNA, partial sequence | NR_134005.1 | 1363 | 284 | 685 | 29.49 | 523.7 | 3E-148 | 365/405 | 90 | 3/405 | 0 | Plus | Plus | Bacteria; Firmicutes; Clostridia; Eubacteriales; Clostridiaceae; Clostridium; Clostridium bornimense | Clostridium bornimense | Clostridium | Clostridiaceae | Eubacteriales | Clostridia | Firmicutes | Bacteria |
| ASV7450 | 422 | 1 | 1 | 422 | 100 | NR_144611.1 Flintibacter butyricus strain BLS21 16S ribosomal RNA, partial sequence | NR_144611.1 | 808 | 299 | 703 | 50.12 | 420.3 | 4E-117 | 360/422 | 85 | 17/422 | 4 | Plus | Plus | Bacteria; Firmicutes; Clostridia; Eubacteriales; ; Flintibacter; Flintibacter butyricus | Flintibacter butyricus | Flintibacter | | Eubacteriales | Clostridia | Firmicutes | Bacteria |
| ASV7452 | 427 | 1 | 1 | 427 | 100 | NR_104863.1 Selenomonas lacticifex strain VB4b 16S ribosomal RNA, partial sequence | NR_104863.1 | 1549 | 364 | 790 | 27.57 | 540.3 | 3E-153 | 382/427 | 89 | 0/427 | 0 | Plus | Plus | Bacteria; Firmicutes; Negativicutes; Selenomonadales; Selenomonadaceae; Selenomonas; Selenomonas lacticifex | Selenomonas lacticifex | Selenomonas | Selenomonadaceae | Selenomonadales | Negativicutes | Firmicutes | Bacteria |
| ASV7453 | 403 | 1 | 4 | 402 | 99.01 | NR_044054.1 Blautia wexlerae DSM 19850 16S ribosomal RNA, partial sequence | NR_044054.1 | 1438 | 316 | 714 | 27.75 | 505.3 | 9E-143 | 360/402 | 89 | 6/402 | 1 | Plus | Plus | Bacteria; Firmicutes; Clostridia; Eubacteriales; Lachnospiraceae; Blautia; Blautia wexlerae | Blautia wexlerae | Blautia | Lachnospiraceae | Eubacteriales | Clostridia | Firmicutes | Bacteria |
| ASV7458 | 403 | 2 | 1 | 403 | 100 | NR_113360.1 Ruminiclostridium cellobioparum DSM 1351 = ATCC 15832 strain JCM 1422 16S ribosomal RNA, partial sequence | NR_113360.1 | 1484 | 356 | 760 | 27.29 | 453.5 | 3E-127 | 355/408 | 87 | 8/408 | 1 | Plus | Plus | Bacteria; Firmicutes; Clostridia; Eubacteriales; Oscillospiraceae; Ruminiclostridium; Ruminiclostridium cellobioparum | Ruminiclostridium cellobioparum | Ruminiclostridium | Oscillospiraceae | Eubacteriales | Clostridia | Firmicutes | Bacteria |
| ASV7459 | 427 | 2 | 5 | 427 | 99.06 | NR_036835.1 Chlamydia muridarum strain MoPn/Wiess-Nigg 16S ribosomal RNA, partial sequence | NR_036835.1 | 1549 | 367 | 789 | 27.31 | 449.9 | 5E-126 | 366/426 | 85 | 6/426 | 1 | Plus | Plus | Bacteria; Chlamydiae; Chlamydiia; Chlamydiales; Chlamydiaceae; Chlamydia; Chlamydia muridarum | Chlamydia muridarum | Chlamydia | Chlamydiaceae | Chlamydiales | Chlamydiia | Chlamydiae | Bacteria |
| ASV7460 | 403 | 1 | 1 | 403 | 100 | NR_118675.1 [Eubacterium] siraeum strain ATCC 29066 16S ribosomal RNA, partial sequence | NR_118675.1 | 1453 | 356 | 757 | 27.67 | 468.3 | 1E-131 | 355/406 | 87 | 7/406 | 1 | Plus | Plus | Bacteria; Firmicutes; Clostridia; Eubacteriales; Oscillospiraceae; ; [Eubacterium] siraeum | [Eubacterium] siraeum | | Oscillospiraceae | Eubacteriales | Clostridia | Firmicutes | Bacteria |
| ASV7461 | 428 | 2 | 1 | 373 | 87.15 | NR_117375.1 Streptococcus danieliae strain ERD01G 16S ribosomal RNA, partial sequence | NR_117375.1 | 1419 | 314 | 685 | 26.22 | 455.4 | 1E-127 | 331/373 | 88 | 1/373 | 0 | Plus | Plus | Bacteria; Firmicutes; Bacilli; Lactobacillales; Streptococcaceae; Streptococcus; Streptococcus danieliae | Streptococcus danieliae | Streptococcus | Streptococcaceae | Lactobacillales | Bacilli | Firmicutes | Bacteria |
| ASV7464 | 403 | 1 | 3 | 403 | 99.5 | NR_028961.1 Faecalibacterium prausnitzii strain ATCC 27768 16S ribosomal RNA, partial sequence | NR_028961.1 | 1462 | 346 | 745 | 27.36 | 595.7 | 5E-170 | 375/401 | 93 | 1/401 | 0 | Plus | Plus | Bacteria; Firmicutes; Clostridia; Eubacteriales; Oscillospiraceae; Faecalibacterium; Faecalibacterium prausnitzii | Faecalibacterium prausnitzii | Faecalibacterium | Oscillospiraceae | Eubacteriales | Clostridia | Firmicutes | Bacteria |
| ASV7467 | 402 | 4 | 4 | 402 | 99.25 | NR_118511.1 Campylobacter coli strain ATCC 33559 16S ribosomal RNA, partial sequence | NR_118511.1 | 1435 | 332 | 731 | 27.87 | 593.9 | 2E-169 | 375/401 | 93 | 3/401 | 0 | Plus | Plus | Bacteria; Proteobacteria; Epsilonproteobacteria; Campylobacterales; Campylobacteraceae; Campylobacter; Campylobacter coli | Campylobacter coli | Campylobacter | Campylobacteraceae | Campylobacterales | Epsilonproteobacteria | Proteobacteria | Bacteria |
| ASV7468 | 427 | 3 | 3 | 427 | 99.53 | NR_041781.1 Streptococcus alactolyticus strain ATCC 43077 16S ribosomal RNA, partial sequence | NR_041781.1 | 1437 | 348 | 772 | 29.58 | 575.4 | 8E-164 | 391/429 | 91 | 8/429 | 1 | Plus | Plus | Bacteria; Firmicutes; Bacilli; Lactobacillales; Streptococcaceae; Streptococcus; Streptococcus alactolyticus | Streptococcus alactolyticus | Streptococcus | Streptococcaceae | Lactobacillales | Bacilli | Firmicutes | Bacteria |
| ASV7470 | 426 | 2 | 3 | 426 | 99.53 | NR_113355.1 Dialister invisus strain JCM 17566 16S ribosomal RNA, partial sequence | NR_113355.1 | 1530 | 375 | 799 | 27.78 | 529.3 | 6E-150 | 380/426 | 89 | 3/426 | 0 | Plus | Plus | Bacteria; Firmicutes; Negativicutes; Veillonellales; Veillonellaceae; Dialister; Dialister invisus | Dialister invisus | Dialister | Veillonellaceae | Veillonellales | Negativicutes | Firmicutes | Bacteria |
| ASV7471 | 379 | 2 | 50 | 379 | 87.07 | NR_102893.1 Butyrivibrio proteoclasticus strain B316 16S ribosomal RNA, partial sequence | NR_102893.1 | 1537 | 453 | 782 | 21.47 | 460.9 | 2E-129 | 305/332 | 91 | 4/332 | 1 | Plus | Plus | Bacteria; Firmicutes; Clostridia; Eubacteriales; Lachnospiraceae; Butyrivibrio; Butyrivibrio proteoclasticus | Butyrivibrio proteoclasticus | Butyrivibrio | Lachnospiraceae | Eubacteriales | Clostridia | Firmicutes | Bacteria |
| ASV7472 | 402 | 2 | 3 | 402 | 99.5 | NR_104892.1 Clostridium moniliforme strain 2055 16S ribosomal RNA, partial sequence | NR_104892.1 | 1488 | 332 | 731 | 26.88 | 557 | 3E-158 | 368/401 | 91 | 2/401 | 0 | Plus | Plus | Bacteria; Firmicutes; Clostridia; Eubacteriales; Clostridiaceae; Clostridium; Clostridium moniliforme | Clostridium moniliforme | Clostridium | Clostridiaceae | Eubacteriales | Clostridia | Firmicutes | Bacteria |
| ASV7474 | 404 | 1 | 1 | 404 | 100 | NR_112097.1 Pseudoramibacter alactolyticus ATCC 23263 16S ribosomal RNA, partial sequence | NR_112097.1 | 1480 | 376 | 779 | 27.3 | 581 | 2E-165 | 376/406 | 92 | 4/406 | 0 | Plus | Plus | Bacteria; Firmicutes; Clostridia; Eubacteriales; Eubacteriaceae; Pseudoramibacter; Pseudoramibacter alactolyticus | Pseudoramibacter alactolyticus | Pseudoramibacter | Eubacteriaceae | Eubacteriales | Clostridia | Firmicutes | Bacteria |
| ASV7480 | 403 | 2 | 1 | 398 | 98.76 | NR_134044.1 Gordonibacter urolithinfaciens strain CEBAS 1/15P 16S ribosomal RNA, partial sequence | NR_134044.1 | 1477 | 335 | 730 | 26.81 | 412.9 | 6E-115 | 341/399 | 85 | 4/399 | 1 | Plus | Plus | Bacteria; Actinobacteria; Coriobacteriia; Eggerthellales; Eggerthellaceae; Gordonibacter; Gordonibacter urolithinfaciens | Gordonibacter urolithinfaciens | Gordonibacter | Eggerthellaceae | Eggerthellales | Coriobacteriia | Actinobacteria | Bacteria |
| ASV7481 | 404 | 1 | 1 | 404 | 100 | NR_144611.1 Flintibacter butyricus strain BLS21 16S ribosomal RNA, partial sequence | NR_144611.1 | 808 | 299 | 703 | 50.12 | 531.1 | 2E-150 | 366/405 | 90 | 1/405 | 0 | Plus | Plus | Bacteria; Firmicutes; Clostridia; Eubacteriales; ; Flintibacter; Flintibacter butyricus | Flintibacter butyricus | Flintibacter | | Eubacteriales | Clostridia | Firmicutes | Bacteria |
| ASV7484 | 402 | 1 | 5 | 402 | 99 | NR_025917.1 Hungateiclostridium cellulolyticum strain CD2 16S ribosomal RNA, partial sequence | NR_025917.1 | 1464 | 330 | 726 | 27.12 | 540.3 | 3E-153 | 365/400 | 91 | 5/400 | 1 | Plus | Plus | Bacteria; Firmicutes; Clostridia; Eubacteriales; Oscillospiraceae; Acetivibrio; Acetivibrio cellulolyticus | Acetivibrio cellulolyticus | Acetivibrio | Oscillospiraceae | Eubacteriales | Clostridia | Firmicutes | Bacteria |
| ASV7486 | 402 | 3 | 1 | 402 | 100 | NR_041781.1 Streptococcus alactolyticus strain ATCC 43077 16S ribosomal RNA, partial sequence | NR_041781.1 | 1437 | 346 | 772 | 29.71 | 516.3 | 4E-146 | 383/428 | 89 | 27/428 | 6 | Plus | Plus | Bacteria; Firmicutes; Bacilli; Lactobacillales; Streptococcaceae; Streptococcus; Streptococcus alactolyticus | Streptococcus alactolyticus | Streptococcus | Streptococcaceae | Lactobacillales | Bacilli | Firmicutes | Bacteria |
| ASV7489 | 404 | 1 | 1 | 403 | 99.75 | NR_115345.1 Hungateiclostridium alkalicellulosi strain Z-7026 16S ribosomal RNA, partial sequence | NR_115345.1 | 1489 | 355 | 757 | 27.07 | 484.9 | 1E-136 | 361/408 | 88 | 10/408 | 2 | Plus | Plus | Bacteria; Firmicutes; Clostridia; Eubacteriales; Oscillospiraceae; Acetivibrio; Acetivibrio alkalicellulosi | Acetivibrio alkalicellulosi | Acetivibrio | Oscillospiraceae | Eubacteriales | Clostridia | Firmicutes | Bacteria |
| ASV7492 | 427 | 3 | 1 | 426 | 99.77 | NR_119168.1 Veillonella parvula DSM 2008 16S ribosomal RNA, partial sequence | NR_119168.1 | 1513 | 348 | 773 | 28.16 | 654.8 | 0 | 404/428 | 94 | 4/428 | 0 | Plus | Plus | Bacteria; Firmicutes; Negativicutes; Veillonellales; Veillonellaceae; Veillonella; Veillonella parvula | Veillonella parvula | Veillonella | Veillonellaceae | Veillonellales | Negativicutes | Firmicutes | Bacteria |
| ASV7495 | 427 | 1 | 5 | 427 | 99.06 | NR_042942.1 Treponema porcinum strain 14V28 16S ribosomal RNA, partial sequence | NR_042942.1 | 1426 | 262 | 684 | 29.66 | 483.1 | 5E-136 | 379/433 | 87 | 20/433 | 4 | Plus | Plus | Bacteria; Spirochaetes; Spirochaetia; Spirochaetales; Spirochaetaceae; Treponema; Treponema porcinum | Treponema porcinum | Treponema | Spirochaetaceae | Spirochaetales | Spirochaetia | Spirochaetes | Bacteria |
| ASV7497 | 402 | 1 | 3 | 402 | 99.5 | NR_118692.1 Blautia obeum ATCC 29174 16S ribosomal RNA, partial sequence | NR_118692.1 | 1398 | 332 | 731 | 28.61 | 542.2 | 7E-154 | 365/401 | 91 | 2/401 | 0 | Plus | Plus | Bacteria; Firmicutes; Clostridia; Eubacteriales; Lachnospiraceae; Blautia; Blautia obeum | Blautia obeum | Blautia | Lachnospiraceae | Eubacteriales | Clostridia | Firmicutes | Bacteria |
| ASV7502 | 403 | 1 | 5 | 403 | 99.01 | NR_026312.1 Blautia schinkii strain B 16S ribosomal RNA, partial sequence | NR_026312.1 | 1482 | 348 | 745 | 26.86 | 438.8 | 1E-122 | 345/399 | 86 | 1/399 | 0 | Plus | Plus | Bacteria; Firmicutes; Clostridia; Eubacteriales; Lachnospiraceae; Blautia; Blautia schinkii | Blautia schinkii | Blautia | Lachnospiraceae | Eubacteriales | Clostridia | Firmicutes | Bacteria |
| ASV7504 | 405 | 1 | 1 | 405 | 100 | NR_044645.2 Dorea formicigenerans strain ATCC 27755 16S ribosomal RNA, partial sequence | NR_044645.2 | 1479 | 372 | 773 | 27.18 | 581 | 2E-165 | 375/406 | 92 | 5/406 | 1 | Plus | Plus | Bacteria; Firmicutes; Clostridia; Eubacteriales; Lachnospiraceae; Dorea; Dorea formicigenerans | Dorea formicigenerans | Dorea | Lachnospiraceae | Eubacteriales | Clostridia | Firmicutes | Bacteria |
| ASV7505 | 405 | 1 | 3 | 405 | 99.51 | NR_114326.1 Fusicatenibacter saccharivorans strain HT03-11 16S ribosomal RNA, partial sequence | NR_114326.1 | 1423 | 353 | 752 | 28.11 | 575.4 | 7E-164 | 373/403 | 92 | 3/403 | 0 | Plus | Plus | Bacteria; Firmicutes; Clostridia; Eubacteriales; Lachnospiraceae; Fusicatenibacter; Fusicatenibacter saccharivorans | Fusicatenibacter saccharivorans | Fusicatenibacter | Lachnospiraceae | Eubacteriales | Clostridia | Firmicutes | Bacteria |
| ASV7508 | 401 | 1 | 3 | 399 | 99 | NR_025044.1 Caloramator viterbiensis strain JW/MS-VS5 16S ribosomal RNA, partial sequence | NR_025044.1 | 1480 | 326 | 723 | 26.89 | 412.9 | 6E-115 | 342/400 | 85 | 5/400 | 1 | Plus | Plus | Bacteria; Firmicutes; Clostridia; Eubacteriales; Clostridiaceae; Caloramator; Caloramator viterbiensis | Caloramator viterbiensis | Caloramator | Clostridiaceae | Eubacteriales | Clostridia | Firmicutes | Bacteria |
| ASV7509 | 402 | 1 | 1 | 402 | 100 | NR_125464.1 Anaerobacterium chartisolvens strain T-1-35 16S ribosomal RNA, partial sequence | NR_125464.1 | 1451 | 342 | 745 | 27.84 | 523.7 | 3E-148 | 364/404 | 90 | 2/404 | 0 | Plus | Plus | Bacteria; Firmicutes; Clostridia; Eubacteriales; Oscillospiraceae; Anaerobacterium; Anaerobacterium chartisolvens | Anaerobacterium chartisolvens | Anaerobacterium | Oscillospiraceae | Eubacteriales | Clostridia | Firmicutes | Bacteria |
| ASV7514 | 402 | 1 | 1 | 402 | 100 | NR_147375.1 Colidextribacter massiliensis strain Marseille-P3083 16S ribosomal RNA, partial sequence | NR_147375.1 | 1487 | 364 | 765 | 27.03 | 505.3 | 9E-143 | 360/403 | 89 | 2/403 | 0 | Plus | Plus | Bacteria; Firmicutes; Clostridia; Eubacteriales; ; Colidextribacter; Colidextribacter massiliensis | Colidextribacter massiliensis | Colidextribacter | | Eubacteriales | Clostridia | Firmicutes | Bacteria |
| ASV7516 | 409 | 5 | 3 | 409 | 99.51 | NR_118511.1 Campylobacter coli strain ATCC 33559 16S ribosomal RNA, partial sequence | NR_118511.1 | 1435 | 331 | 731 | 27.94 | 514.5 | 2E-145 | 365/407 | 89 | 6/407 | 1 | Plus | Plus | Bacteria; Proteobacteria; Epsilonproteobacteria; Campylobacterales; Campylobacteraceae; Campylobacter; Campylobacter coli | Campylobacter coli | Campylobacter | Campylobacteraceae | Campylobacterales | Epsilonproteobacteria | Proteobacteria | Bacteria |

[illegible]

| | | | | | | | | | | | | | | | | | | | | | | | | | | | |
|---|---|---|---|---|---|---|---|---|---|---|---|---|---|---|---|---|---|---|---|---|---|---|---|---|---|---|---|
| ASV8036 | 403 | 1 | 1 | 403 | 100 | NR_134005.1 Clostridium bornimense strain M2/40 16S ribosomal RNA, partial sequence | NR_134005.1 | 1363 | 284 | 685 | 29.49 | 593.9 | 2E-169 | 378/405 | 93 | 5/405 | 1 | Plus | Plus | Bacteria; Firmicutes; Clostridia; Eubacteriales; Clostridiaceae; Clostridium; Clostridium bornimense | Clostridium bornimense | Clostridium | Clostridiaceae | Eubacteriales | Clostridia | Firmicutes | Bacteria |
| ASV8038 | 426 | 1 | 1 | 426 | 100 | NR_125628.1 Holdemania massiliensis AP2 16S ribosomal RNA, partial sequence | NR_125628.1 | 1499 | 353 | 778 | 28.42 | 444.3 | 2E-124 | 367/429 | 85 | 6/429 | 1 | Plus | Plus | Bacteria; Firmicutes; Erysipelotrichia; Erysipelotrichales; Erysipelotrichaceae; Holdemania; Holdemania massiliensis | Holdemania massiliensis | Holdemania | Erysipelotrichaceae | Erysipelotrichales | Erysipelotrichia | Firmicutes | Bacteria |
| ASV8039 | 402 | 1 | 3 | 402 | 99.5 | NR_144737.1 Emergencia timonensis strain SN18 16S ribosomal RNA, partial sequence | NR_144737.1 | 1480 | 366 | 767 | 27.16 | 470.2 | 3E-132 | 353/402 | 87 | 2/402 | 0 | Plus | Plus | Bacteria; Firmicutes; Clostridia; Eubacteriales; Clostridiales Family XIII. Incertae Sedis; Emergencia; Emergencia timonensis | Emergencia timonensis | Emergencia | Clostridiales Family XIII. Incertae Sedis | Eubacteriales | Clostridia | Firmicutes | Bacteria |
| ASV8040 | 422 | 1 | 1 | 422 | 100 | NR_113411.1 Prevotella copri DSM 18205 strain JCM 13464 16S ribosomal RNA, partial sequence | NR_113411.1 | 1493 | 358 | 779 | 28.27 | 547.7 | 2E-155 | 380/422 | 90 | 0/422 | 0 | Plus | Plus | Bacteria; Bacteroidetes; Bacteroidia; Bacteroidales; Prevotellaceae; Prevotella; Prevotella copri | Prevotella copri | Prevotella | Prevotellaceae | Bacteroidales | Bacteroidia | Bacteroidetes | Bacteria |
| ASV8041 | 402 | 3 | 1 | 402 | 100 | NR_117139.2 Anaerostipes hadrus strain DSM 3319 16S ribosomal RNA, partial sequence | NR_117139.2 | 1521 | 363 | 764 | 26.43 | 510.8 | 2E-144 | 363/405 | 89 | 6/405 | 1 | Plus | Plus | Bacteria; Firmicutes; Clostridia; Eubacteriales; Lachnospiraceae; Anaerostipes; Anaerostipes hadrus | Anaerostipes hadrus | Anaerostipes | Lachnospiraceae | Eubacteriales | Clostridia | Firmicutes | Bacteria |
| ASV8043 | 428 | 1 | 3 | 428 | 99.53 | NR_026476.1 Succinivibrio dextrinosolvens strain 0554 16S ribosomal RNA, partial sequence | NR_026476.1 | 1463 | 333 | 732 | 27.34 | 459.1 | 8E-129 | 372/427 | 87 | 28/427 | 6 | Plus | Plus | Bacteria; Proteobacteria; Gammaproteobacteria; Aeromonadales; Succinivibrionaceae; Succinivibrio; Succinivibrio dextrinosolvens | Succinivibrio dextrinosolvens | Succinivibrio | Succinivibrionaceae | Aeromonadales | Gammaproteobacteria | Proteobacteria | Bacteria |
| ASV8048 | 422 | 1 | 1 | 422 | 100 | NR_113411.1 Prevotella copri DSM 18205 strain JCM 13464 16S ribosomal RNA, partial sequence | NR_113411.1 | 1493 | 358 | 779 | 28.27 | 503.4 | 4E-142 | 373/423 | 88 | 2/423 | 0 | Plus | Plus | Bacteria; Bacteroidetes; Bacteroidia; Bacteroidales; Prevotellaceae; Prevotella; Prevotella copri | Prevotella copri | Prevotella | Prevotellaceae | Bacteroidales | Bacteroidia | Bacteroidetes | Bacteria |
| ASV8049 | 422 | 1 | 1 | 407 | 96.45 | NR_113227.1 Prevotella oryzae DSM 17970 strain JCM 13648 16S ribosomal RNA, partial sequence | NR_113227.1 | 1490 | 356 | 762 | 27.32 | 425.9 | 8E-119 | 351/410 | 85 | 6/410 | 1 | Plus | Plus | Bacteria; Bacteroidetes; Bacteroidia; Bacteroidales; Prevotellaceae; Prevotella; Prevotella oryzae | Prevotella oryzae | Prevotella | Prevotellaceae | Bacteroidales | Bacteroidia | Bacteroidetes | Bacteria |
| ASV8050 | 401 | 2 | 1 | 400 | 99.75 | NR_102987.1 Hungateiclostridium clariflavum DSM 19732 16S ribosomal RNA, partial sequence | NR_102987.1 | 1634 | 481 | 881 | 24.54 | 484.9 | 1E-136 | 358/404 | 88 | 7/404 | 1 | Plus | Plus | Bacteria; Firmicutes; Clostridia; Eubacteriales; Oscillospiraceae; Acetivibrio; Acetivibrio clariflavus | Acetivibrio clariflavus | Acetivibrio | Oscillospiraceae | Eubacteriales | Clostridia | Firmicutes | Bacteria |
| ASV8051 | 401 | 1 | 1 | 401 | 100 | NR_102987.1 Hungateiclostridium clariflavum DSM 19732 16S ribosomal RNA, partial sequence | NR_102987.1 | 1634 | 481 | 882 | 24.6 | 492.3 | 7E-139 | 360/405 | 88 | 7/405 | 1 | Plus | Plus | Bacteria; Firmicutes; Clostridia; Eubacteriales; Oscillospiraceae; Acetivibrio; Acetivibrio clariflavus | Acetivibrio clariflavus | Acetivibrio | Oscillospiraceae | Eubacteriales | Clostridia | Firmicutes | Bacteria |
| ASV8055 | 428 | 1 | 1 | 428 | 100 | NR_118554.1 Intestinimonas butyriciproducens strain SRB-521-5-I 16S ribosomal RNA, partial sequence | NR_118554.1 | 1475 | 346 | 750 | 27.46 | 470.2 | 4E-132 | 376/430 | 87 | 27/430 | 6 | Plus | Plus | Bacteria; Firmicutes; Clostridia; Eubacteriales; ; Intestinimonas; Intestinimonas butyriciproducens | Intestinimonas butyriciproducens | Intestinimonas | | Eubacteriales | Clostridia | Firmicutes | Bacteria |
| ASV8057 | 401 | 3 | 1 | 401 | 100 | NR_102980.1 Megasphaera elsdenii DSM 20460 16S ribosomal RNA, partial sequence | NR_102980.1 | 1552 | 369 | 795 | 27.51 | 533 | 4E-151 | 386/428 | 90 | 28/428 | 6 | Plus | Plus | Bacteria; Firmicutes; Negativicutes; Veillonellales; Veillonellaceae; Megasphaera; Megasphaera elsdenii | Megasphaera elsdenii | Megasphaera | Veillonellaceae | Veillonellales | Negativicutes | Firmicutes | Bacteria |
| ASV8058 | 402 | 1 | 1 | 402 | 100 | NR_104783.1 Acetivibrio ethanolgignens strain 77-6 16S ribosomal RNA, partial sequence | NR_104783.1 | 1529 | 370 | 771 | 26.29 | 588.4 | 9E-168 | 374/402 | 93 | 0/402 | 0 | Plus | Plus | Bacteria; Firmicutes; Clostridia; Eubacteriales; Oscillospiraceae; Acetivibrio; Acetivibrio ethanolgignens | Acetivibrio ethanolgignens | Acetivibrio | Oscillospiraceae | Eubacteriales | Clostridia | Firmicutes | Bacteria |
| ASV8060 | 402 | 1 | 1 | 387 | 96.27 | NR_118675.1 [Eubacterium] siraeum strain ATCC 29066 16S ribosomal RNA, partial sequence | NR_118675.1 | 1453 | 356 | 742 | 26.63 | 457.2 | 3E-128 | 339/387 | 87 | 0/387 | 0 | Plus | Plus | Bacteria; Firmicutes; Clostridia; Eubacteriales; Oscillospiraceae; ; [Eubacterium] siraeum | [Eubacterium] siraeum | | Oscillospiraceae | Eubacteriales | Clostridia | Firmicutes | Bacteria |
| ASV8061 | 426 | 1 | 3 | 426 | 99.53 | NR_027596.1 Mitsuokella multacida strain DSM 20544 16S ribosomal RNA, partial sequence | NR_027596.1 | 1511 | 348 | 772 | 28.13 | 542.2 | 8E-154 | 383/427 | 89 | 5/427 | 1 | Plus | Plus | Bacteria; Firmicutes; Negativicutes; Selenomonadales; Selenomonadaceae; Mitsuokella; Mitsuokella multacida | Mitsuokella multacida | Mitsuokella | Selenomonadaceae | Selenomonadales | Negativicutes | Firmicutes | Bacteria |
| ASV8063 | 403 | 2 | 1 | 403 | 100 | NR_037018.1 Acidaminococcus fermentans strain VR4 16S ribosomal RNA gene, partial sequence | NR_037018.1 | 1516 | 349 | 775 | 28.17 | 418.5 | 1E-116 | 364/427 | 85 | 24/427 | 5 | Plus | Plus | Bacteria; Firmicutes; Negativicutes; Acidaminococcales; Acidaminococcaceae; Acidaminococcus; Acidaminococcus fermentans | Acidaminococcus fermentans | Acidaminococcus | Acidaminococcaceae | Acidaminococcales | Negativicutes | Firmicutes | Bacteria |
| ASV8065 | 422 | 1 | 1 | 422 | 100 | NR_104901.1 Shigella boydii strain P288 16S ribosomal RNA, partial sequence | NR_104901.1 | 1515 | 331 | 757 | 28.18 | 473.9 | 3E-133 | 376/432 | 87 | 15/432 | 3 | Plus | Plus | Bacteria; Proteobacteria; Gammaproteobacteria; Enterobacterales; Enterobacteriaceae; Shigella; Shigella boydii | Shigella boydii | Shigella | Enterobacteriaceae | Enterobacterales | Gammaproteobacteria | Proteobacteria | Bacteria |
| ASV8066 | 402 | 1 | 5 | 402 | 99 | NR_026476.1 Succinivibrio dextrinosolvens strain 0554 16S ribosomal RNA, partial sequence | NR_026476.1 | 1463 | 335 | 732 | 27.2 | 448 | 2E-125 | 347/399 | 86 | 2/399 | 0 | Plus | Plus | Bacteria; Proteobacteria; Gammaproteobacteria; Aeromonadales; Succinivibrionaceae; Succinivibrio; Succinivibrio dextrinosolvens | Succinivibrio dextrinosolvens | Succinivibrio | Succinivibrionaceae | Aeromonadales | Gammaproteobacteria | Proteobacteria | Bacteria |
| ASV8077 | 426 | 1 | 3 | 426 | 99.53 | NR_044111.1 Selenomonas bovis strain WG 16S ribosomal RNA, partial sequence | NR_044111.1 | 1525 | 372 | 796 | 27.87 | 540.3 | 3E-153 | 383/427 | 89 | 5/427 | 1 | Plus | Plus | Bacteria; Firmicutes; Negativicutes; Selenomonadales; Selenomonadaceae; Selenomonas; Selenomonas bovis | Selenomonas bovis | Selenomonas | Selenomonadaceae | Selenomonadales | Negativicutes | Firmicutes | Bacteria |
| ASV8078 | 402 | 1 | 1 | 402 | 100 | NR_029097.1 Oribacterium sinus strain AIP 354.02 16S ribosomal RNA, partial sequence | NR_029097.1 | 1374 | 308 | 709 | 29.26 | 577.3 | 2E-164 | 372/402 | 92 | 0/402 | 0 | Plus | Plus | Bacteria; Firmicutes; Clostridia; Eubacteriales; Lachnospiraceae; Oribacterium; Oribacterium sinus | Oribacterium sinus | Oribacterium | Lachnospiraceae | Eubacteriales | Clostridia | Firmicutes | Bacteria |
| ASV8082 | 409 | 1 | 1 | 409 | 100 | NR_125677.1 Vallitalea pronyensis strain FatNI3 16S ribosomal RNA, partial sequence | NR_125677.1 | 1480 | 363 | 764 | 27.16 | 444.3 | 2E-124 | 355/410 | 86 | 9/410 | 2 | Plus | Plus | Bacteria; Firmicutes; Clostridia; Eubacteriales; Vallitaleaceae; Vallitalea; Vallitalea pronyensis | Vallitalea pronyensis | Vallitalea | Vallitaleaceae | Eubacteriales | Clostridia | Firmicutes | Bacteria |
| ASV8088 | 403 | 1 | 3 | 403 | 99.5 | NR_147396.1 Lachnoclostridium pacaense strain Marseille-P3100 16S ribosomal RNA, partial sequence | NR_147396.1 | 1453 | 347 | 746 | 27.53 | 573.6 | 3E-163 | 374/404 | 92 | 7/404 | 1 | Plus | Plus | Bacteria; Firmicutes; Clostridia; Eubacteriales; Lachnospiraceae; Lachnoclostridium; Lachnoclostridium pacaense | Lachnoclostridium pacaense | Lachnoclostridium | Lachnospiraceae | Eubacteriales | Clostridia | Firmicutes | Bacteria |
| ASV8093 | 405 | 2 | 1 | 405 | 100 | NG_041947.1 Ruminiclostridium cellulolyticum H10 16S ribosomal RNA gene, complete sequence | NG_041947.1 | 1641 | 486 | 890 | 24.68 | 416.6 | 5E-116 | 349/409 | 85 | 8/409 | 1 | Plus | Plus | Bacteria; Firmicutes; Clostridia; Eubacteriales; Oscillospiraceae; Ruminiclostridium; Ruminiclostridium cellulolyticum | Ruminiclostridium cellulolyticum | Ruminiclostridium | Oscillospiraceae | Eubacteriales | Clostridia | Firmicutes | Bacteria |
| ASV8094 | 428 | 5 | 5 | 428 | 99.07 | NR_102895.1 Prevotella melaninogenica ATCC 25845 16S ribosomal RNA, partial sequence | NR_102895.1 | 1531 | 370 | 787 | 27.3 | 429.5 | 6E-120 | 361/424 | 85 | 6/424 | 1 | Plus | Plus | Bacteria; Bacteroidetes; Bacteroidia; Bacteroidales; Prevotellaceae; Prevotella; Prevotella melaninogenica | Prevotella melaninogenica | Prevotella | Prevotellaceae | Bacteroidales | Bacteroidia | Bacteroidetes | Bacteria |
| ASV8095 | 405 | 1 | 3 | 405 | 99.51 | NR_025025.1 Papillibacter cinnamivorans strain CIN1 16S ribosomal RNA, partial sequence | NR_025025.1 | 1491 | 351 | 751 | 26.89 | 466.5 | 4E-131 | 353/404 | 87 | 4/404 | 0 | Plus | Plus | Bacteria; Firmicutes; Clostridia; Eubacteriales; Oscillospiraceae; Papillibacter; Papillibacter cinnamivorans | Papillibacter cinnamivorans | Papillibacter | Oscillospiraceae | Eubacteriales | Clostridia | Firmicutes | Bacteria |
| ASV8096 | 404 | 1 | 1 | 404 | 100 | NR_118692.1 Blautia obeum ATCC 29174 16S ribosomal RNA, partial sequence | NR_118692.1 | 1398 | 330 | 731 | 28.76 | 564.3 | 2E-160 | 373/406 | 91 | 6/406 | 1 | Plus | Plus | Bacteria; Firmicutes; Clostridia; Eubacteriales; Lachnospiraceae; Blautia; Blautia obeum | Blautia obeum | Blautia | Lachnospiraceae | Eubacteriales | Clostridia | Firmicutes | Bacteria |
| ASV8099 | 428 | 2 | 3 | 428 | 99.53 | NR_044734.1 Selenomonas ruminantium strain GA192 16S ribosomal RNA, partial sequence | NR_044734.1 | 1486 | 377 | 801 | 28.6 | 433.2 | 5E-121 | 364/428 | 85 | 5/428 | 1 | Plus | Plus | Bacteria; Firmicutes; Negativicutes; Selenomonadales; Selenomonadaceae; Selenomonas; Selenomonas ruminantium | Selenomonas ruminantium | Selenomonas | Selenomonadaceae | Selenomonadales | Negativicutes | Firmicutes | Bacteria |
| ASV8100 | 422 | 1 | 1 | 422 | 100 | NR_113411.1 Prevotella copri DSM 18205 strain JCM 13464 16S ribosomal RNA, partial sequence | NR_113411.1 | 1493 | 358 | 779 | 28.27 | 525.6 | 8E-149 | 380/426 | 89 | 8/426 | 1 | Plus | Plus | Bacteria; Bacteroidetes; Bacteroidia; Bacteroidales; Prevotellaceae; Prevotella; Prevotella copri | Prevotella copri | Prevotella | Prevotellaceae | Bacteroidales | Bacteroidia | Bacteroidetes | Bacteria |
| ASV8103 | 427 | 1 | 4 | 426 | 99.06 | NR_102980.1 Megasphaera elsdenii DSM 20460 16S ribosomal RNA, partial sequence | NR_102980.1 | 1552 | 372 | 794 | 27.26 | 510.8 | 2E-144 | 377/426 | 88 | 6/426 | 1 | Plus | Plus | Bacteria; Firmicutes; Negativicutes; Veillonellales; Veillonellaceae; Megasphaera; Megasphaera elsdenii | Megasphaera elsdenii | Megasphaera | Veillonellaceae | Veillonellales | Negativicutes | Firmicutes | Bacteria |
| ASV8105 | 427 | 4 | 1 | 427 | 100 | NR_042802.1 Lactobacillus ultunensis strain Kx146C1 16S ribosomal RNA, partial sequence | NR_042802.1 | 1550 | 370 | 796 | 27.55 | 612.4 | 6E-175 | 396/428 | 92 | 2/428 | 0 | Plus | Plus | Bacteria; Firmicutes; Bacilli; Lactobacillales; Lactobacillaceae; Lactobacillus; Lactobacillus ultunensis | Lactobacillus ultunensis | Lactobacillus | Lactobacillaceae | Lactobacillales | Bacilli | Firmicutes | Bacteria |
| ASV8106 | 422 | 1 | 1 | 422 | 100 | NR_113411.1 Prevotella copri DSM 18205 strain JCM 13464 16S ribosomal RNA, partial sequence | NR_113411.1 | 1493 | 358 | 779 | 28.27 | 564.3 | 2E-160 | 387/426 | 90 | 8/426 | 1 | Plus | Plus | Bacteria; Bacteroidetes; Bacteroidia; Bacteroidales; Prevotellaceae; Prevotella; Prevotella copri | Prevotella copri | Prevotella | Prevotellaceae | Bacteroidales | Bacteroidia | Bacteroidetes | Bacteria |
| ASV8108 | 422 | 1 | 1 | 422 | 100 | NR_041342.1 Parabacteroides distasonis strain JCM 5825 16S ribosomal RNA, partial sequence | NR_041342.1 | 1488 | 353 | 774 | 28.36 | 448 | 2E-125 | 367/427 | 85 | 10/427 | 2 | Plus | Plus | Bacteria; Bacteroidetes; Bacteroidia; Bacteroidales; Tannerellaceae; Parabacteroides; Parabacteroides distasonis | Parabacteroides distasonis | Parabacteroides | Tannerellaceae | Bacteroidales | Bacteroidia | Bacteroidetes | Bacteria |
| [illegible] | [illegible] | [illegible] | [illegible] | [illegible] | [illegible] | [illegible] | [illegible] | [illegible] | [illegible] | [illegible] | [illegible] | [illegible] | [illegible] | [illegible] | [illegible] | [illegible] | [illegible] | [illegible] | [illegible] | [illegible] | [illegible] | [illegible] | [illegible] | [illegible] | [illegible] | [illegible] | [illegible] |

| | | | | | | | | | | | | | | | | | | | | | | | | | | | |
|---|---|---|---|---|---|---|---|---|---|---|---|---|---|---|---|---|---|---|---|---|---|---|---|---|---|---|---|
| ASV8696 | 422 | 2 | 1 | 422 | 100 | NR_113119.1 Prevotella oris strain JCM 8540 16S ribosomal RNA, partial sequence | NR_113119.1 | 1481 | 345 | 766 | 28.49 | 597.6 | 2E-170 | 391/424 | 92 | 4/424 | 0 | Plus | Plus | Bacteria; Bacteroidetes; Bacteroidia; Bacteroidales; Prevotellaceae; Prevotella; Prevotella oris | Prevotella oris | Prevotella | Prevotellaceae | Bacteroidales | Bacteroidia | Bacteroidetes | Bacteria |
| ASV8705 | 422 | 1 | 1 | 422 | 100 | NR_144750.1 Prevotellamassilia timonensis strain Marseille-P2831 16S ribosomal RNA, partial sequence | NR_144750.1 | 1493 | 358 | 779 | 28.27 | 492.3 | 8E-139 | 373/425 | 87 | 6/425 | 1 | Plus | Plus | Bacteria; Bacteroidetes; Bacteroidia; Bacteroidales; Prevotellaceae; Prevotellamassilia; Prevotellamassilia timonensis | Prevotellamassilia timonensis | Prevotellamassilia | Prevotellaceae | Bacteroidales | Bacteroidia | Bacteroidetes | Bacteria |
| ASV8706 | 423 | 1 | 1 | 423 | 100 | NR_144750.1 Prevotellamassilia timonensis strain Marseille-P2831 16S ribosomal RNA, partial sequence | NR_144750.1 | 1493 | 358 | 779 | 28.27 | 475.7 | 8E-134 | 372/427 | 87 | 9/427 | 2 | Plus | Plus | Bacteria; Bacteroidetes; Bacteroidia; Bacteroidales; Prevotellaceae; Prevotellamassilia; Prevotellamassilia timonensis | Prevotellamassilia timonensis | Prevotellamassilia | Prevotellaceae | Bacteroidales | Bacteroidia | Bacteroidetes | Bacteria |
| ASV8707 | 423 | 1 | 1 | 423 | 100 | NR_144750.1 Prevotellamassilia timonensis strain Marseille-P2831 16S ribosomal RNA, partial sequence | NR_144750.1 | 1493 | 358 | 779 | 28.27 | 475.7 | 8E-134 | 371/426 | 87 | 7/426 | 1 | Plus | Plus | Bacteria; Bacteroidetes; Bacteroidia; Bacteroidales; Prevotellaceae; Prevotellamassilia; Prevotellamassilia timonensis | Prevotellamassilia timonensis | Prevotellamassilia | Prevotellaceae | Bacteroidales | Bacteroidia | Bacteroidetes | Bacteria |
| ASV8708 | 423 | 1 | 1 | 423 | 100 | NR_144750.1 Prevotellamassilia timonensis strain Marseille-P2831 16S ribosomal RNA, partial sequence | NR_144750.1 | 1493 | 358 | 779 | 28.27 | 508.9 | 8E-144 | 376/425 | 88 | 5/425 | 1 | Plus | Plus | Bacteria; Bacteroidetes; Bacteroidia; Bacteroidales; Prevotellaceae; Prevotellamassilia; Prevotellamassilia timonensis | Prevotellamassilia timonensis | Prevotellamassilia | Prevotellaceae | Bacteroidales | Bacteroidia | Bacteroidetes | Bacteria |
| ASV8710 | 428 | 1 | 1 | 428 | 100 | NR_102980.1 Megasphaera elsdenii DSM 20460 16S ribosomal RNA, partial sequence | NR_102980.1 | 1552 | 369 | 795 | 27.51 | 540.3 | 3E-153 | 386/431 | 89 | 7/431 | 1 | Plus | Plus | Bacteria; Firmicutes; Negativicutes; Veillonellales; Veillonellaceae; Megasphaera; Megasphaera elsdenii | Megasphaera elsdenii | Megasphaera | Veillonellaceae | Veillonellales | Negativicutes | Firmicutes | Bacteria |
| ASV8712 | 427 | 5 | 3 | 427 | 99.53 | NR_113719.1 Lactobacillus helveticus strain NBRC 15019 16S ribosomal RNA, partial sequence | NR_113719.1 | 1487 | 354 | 778 | 28.58 | 508.9 | 8E-144 | 376/426 | 88 | 2/426 | 0 | Plus | Plus | Bacteria; Firmicutes; Bacilli; Lactobacillales; Lactobacillaceae; Lactobacillus; Lactobacillus helveticus | Lactobacillus helveticus | Lactobacillus | Lactobacillaceae | Lactobacillales | Bacilli | Firmicutes | Bacteria |
| ASV8714 | 405 | 1 | 1 | 405 | 100 | NR_024829.1 Hungateiclostridium straminisolvens strain CSK1 16S ribosomal RNA, partial sequence | NR_024829.1 | 1447 | 338 | 742 | 27.99 | 521.9 | 9E-148 | 365/406 | 89 | 2/406 | 0 | Plus | Plus | Bacteria; Firmicutes; Clostridia; Eubacteriales; Oscillospiraceae; Acetivibrio; Acetivibrio straminisolvens | Acetivibrio straminisolvens | Acetivibrio | Oscillospiraceae | Eubacteriales | Clostridia | Firmicutes | Bacteria |
| ASV8718 | 405 | 1 | 1 | 405 | 100 | NR_041611.1 Lactobacillus ruminis strain NBRC 102161 16S ribosomal RNA, partial sequence | NR_041611.1 | 1488 | 352 | 778 | 28.7 | 553.3 | 3E-157 | 388/427 | 90 | 22/427 | 5 | Plus | Plus | Bacteria; Firmicutes; Bacilli; Lactobacillales; Lactobacillaceae; Ligilactobacillus; Ligilactobacillus ruminis | Ligilactobacillus ruminis | Ligilactobacillus | Lactobacillaceae | Lactobacillales | Bacilli | Firmicutes | Bacteria |
| ASV8725 | 422 | 4 | 1 | 422 | 100 | NR_044864.1 Hallella seregens ATCC 51272 16S ribosomal RNA, partial sequence | NR_044864.1 | 1463 | 335 | 756 | 28.84 | 514.5 | 2E-145 | 376/424 | 88 | 4/424 | 0 | Plus | Plus | Bacteria; Bacteroidetes; Bacteroidia; Bacteroidales; Prevotellaceae; Prevotella; Hallella seregens | Hallella seregens | Prevotella | Prevotellaceae | Bacteroidales | Bacteroidia | Bacteroidetes | Bacteria |
| ASV8726 | 422 | 1 | 1 | 422 | 100 | NR_118554.1 Intestinimonas butyriciproducens strain SRB-521-5-I 16S ribosomal RNA, partial sequence | NR_118554.1 | 1475 | 346 | 750 | 27.46 | 448 | 2E-125 | 366/423 | 86 | 19/423 | 4 | Plus | Plus | Bacteria; Firmicutes; Clostridia; Eubacteriales; ; Intestinimonas; Intestinimonas butyriciproducens | Intestinimonas butyriciproducens | Intestinimonas | | Eubacteriales | Clostridia | Firmicutes | Bacteria |
| ASV8727 | 422 | 2 | 1 | 422 | 100 | NR_113117.1 Prevotella oralis ATCC 33269 strain JCM 12251 16S ribosomal RNA, partial sequence | NR_113117.1 | 1496 | 361 | 782 | 28.21 | 542.2 | 8E-154 | 381/424 | 89 | 4/424 | 0 | Plus | Plus | Bacteria; Bacteroidetes; Bacteroidia; Bacteroidales; Prevotellaceae; Prevotella; Prevotella oralis | Prevotella oralis | Prevotella | Prevotellaceae | Bacteroidales | Bacteroidia | Bacteroidetes | Bacteria |
| ASV8729 | 422 | 3 | 1 | 422 | 100 | NR_113109.1 Prevotella loescheii strain JCM 8530 16S ribosomal RNA, partial sequence | NR_113109.1 | 1476 | 342 | 763 | 28.59 | 547.7 | 2E-155 | 384/426 | 90 | 8/426 | 1 | Plus | Plus | Bacteria; Bacteroidetes; Bacteroidia; Bacteroidales; Prevotellaceae; Prevotella; Prevotella loescheii | Prevotella loescheii | Prevotella | Prevotellaceae | Bacteroidales | Bacteroidia | Bacteroidetes | Bacteria |
| ASV8731 | 404 | 2 | 3 | 404 | 99.5 | NR_027557.1 Roseburia intestinalis L1-82 16S ribosomal RNA, partial sequence | NR_027557.1 | 1482 | 362 | 763 | 27.13 | 588.4 | 9E-168 | 376/404 | 93 | 4/404 | 0 | Plus | Plus | Bacteria; Firmicutes; Clostridia; Eubacteriales; Lachnospiraceae; Roseburia; Roseburia intestinalis | Roseburia intestinalis | Roseburia | Lachnospiraceae | Eubacteriales | Clostridia | Firmicutes | Bacteria |
| ASV8741 | 407 | 1 | 1 | 407 | 100 | NR_134005.1 Clostridium bornimense strain M2/40 16S ribosomal RNA, partial sequence | NR_134005.1 | 1363 | 284 | 685 | 29.49 | 569.9 | 3E-162 | 375/407 | 92 | 5/407 | 1 | Plus | Plus | Bacteria; Firmicutes; Clostridia; Eubacteriales; Clostridiaceae; Clostridium; Clostridium bornimense | Clostridium bornimense | Clostridium | Clostridiaceae | Eubacteriales | Clostridia | Firmicutes | Bacteria |
| ASV8744 | 404 | 1 | 1 | 404 | 100 | NR_134005.1 Clostridium bornimense strain M2/40 16S ribosomal RNA, partial sequence | NR_134005.1 | 1363 | 284 | 685 | 29.49 | 545.9 | 6E-155 | 372/408 | 91 | 10/408 | 2 | Plus | Plus | Bacteria; Firmicutes; Clostridia; Eubacteriales; Clostridiaceae; Clostridium; Clostridium bornimense | Clostridium bornimense | Clostridium | Clostridiaceae | Eubacteriales | Clostridia | Firmicutes | Bacteria |
| ASV8750 | 421 | 4 | 1 | 421 | 100 | NR_113048.1 Bacteroides pyogenes strain DSM 20611 16S ribosomal RNA, partial sequence | NR_113048.1 | 1476 | 343 | 765 | 28.66 | 464.6 | 2E-130 | 369/426 | 86 | 8/426 | 1 | Plus | Plus | Bacteria; Bacteroidetes; Bacteroidia; Bacteroidales; Bacteroidaceae; Bacteroides; Bacteroides pyogenes | Bacteroides pyogenes | Bacteroides | Bacteroidaceae | Bacteroidales | Bacteroidia | Bacteroidetes | Bacteria |
| ASV8751 | 427 | 5 | 1 | 427 | 100 | NR_042111.1 Lactobacillus gallinarum strain ATCC 33199 16S ribosomal RNA, partial sequence | NR_042111.1 | 1546 | 362 | 788 | 27.62 | 507.1 | 3E-143 | 377/428 | 88 | 2/428 | 0 | Plus | Plus | Bacteria; Firmicutes; Bacilli; Lactobacillales; Lactobacillaceae; Lactobacillus; Lactobacillus gallinarum | Lactobacillus gallinarum | Lactobacillus | Lactobacillaceae | Lactobacillales | Bacilli | Firmicutes | Bacteria |
| ASV8758 | 427 | 1 | 1 | 427 | 100 | NR_025569.1 Escherichia albertii strain Albert 19982 16S ribosomal RNA, partial sequence | NR_025569.1 | 1494 | 331 | 757 | 28.58 | 484.9 | 1E-136 | 376/431 | 87 | 8/431 | 1 | Plus | Plus | Bacteria; Proteobacteria; Gammaproteobacteria; Enterobacterales; Enterobacteriaceae; Escherichia; Escherichia albertii | Escherichia albertii | Escherichia | Enterobacteriaceae | Enterobacterales | Gammaproteobacteria | Proteobacteria | Bacteria |
| ASV8760 | 427 | 1 | 1 | 427 | 100 | NR_025569.1 Escherichia albertii strain Albert 19982 16S ribosomal RNA, partial sequence | NR_025569.1 | 1494 | 331 | 757 | 28.58 | 507.1 | 3E-143 | 378/429 | 88 | 4/429 | 0 | Plus | Plus | Bacteria; Proteobacteria; Gammaproteobacteria; Enterobacterales; Enterobacteriaceae; Escherichia; Escherichia albertii | Escherichia albertii | Escherichia | Enterobacteriaceae | Enterobacterales | Gammaproteobacteria | Proteobacteria | Bacteria |
| ASV8761 | 402 | 3 | 1 | 393 | 97.76 | NR_144696.1 Clostridium saudiense strain JCC 16S ribosomal RNA, partial sequence | NR_144696.1 | 1478 | 350 | 742 | 26.59 | 632.7 | 0 | 377/394 | 95 | 2/394 | 0 | Plus | Plus | Bacteria; Firmicutes; Clostridia; Eubacteriales; Clostridiaceae; Clostridium; Clostridium saudiense | Clostridium saudiense | Clostridium | Clostridiaceae | Eubacteriales | Clostridia | Firmicutes | Bacteria |
| ASV8768 | 422 | 1 | 1 | 422 | 100 | NR_041364.1 Prevotella stercorea DSM 18206 strain CB35 16S ribosomal RNA, partial sequence | NR_041364.1 | 1488 | 354 | 775 | 28.36 | 625.3 | 7E-179 | 395/423 | 93 | 2/423 | 0 | Plus | Plus | Bacteria; Bacteroidetes; Bacteroidia; Bacteroidales; Prevotellaceae; Prevotella; Prevotella stercorea | Prevotella stercorea | Prevotella | Prevotellaceae | Bacteroidales | Bacteroidia | Bacteroidetes | Bacteria |
| ASV8769 | 422 | 1 | 1 | 422 | 100 | NR_041364.1 Prevotella stercorea DSM 18206 strain CB35 16S ribosomal RNA, partial sequence | NR_041364.1 | 1488 | 354 | 775 | 28.36 | 508.9 | 8E-144 | 374/423 | 88 | 2/423 | 0 | Plus | Plus | Bacteria; Bacteroidetes; Bacteroidia; Bacteroidales; Prevotellaceae; Prevotella; Prevotella stercorea | Prevotella stercorea | Prevotella | Prevotellaceae | Bacteroidales | Bacteroidia | Bacteroidetes | Bacteria |
| ASV8774 | 422 | 1 | 1 | 422 | 100 | NR_113096.1 Prevotella bivia strain JCM 6331 16S ribosomal RNA, partial sequence | NR_113096.1 | 1490 | 356 | 777 | 28.32 | 564.3 | 2E-160 | 385/424 | 90 | 4/424 | 0 | Plus | Plus | Bacteria; Bacteroidetes; Bacteroidia; Bacteroidales; Prevotellaceae; Prevotella; Prevotella bivia | Prevotella bivia | Prevotella | Prevotellaceae | Bacteroidales | Bacteroidia | Bacteroidetes | Bacteria |
| ASV8779 | 422 | 1 | 1 | 422 | 100 | NR_113122.1 Prevotella paludivivens strain JCM 13650 16S ribosomal RNA, partial sequence | NR_113122.1 | 1491 | 356 | 777 | 28.3 | 553.3 | 4E-157 | 381/422 | 90 | 0/422 | 0 | Plus | Plus | Bacteria; Bacteroidetes; Bacteroidia; Bacteroidales; Prevotellaceae; Prevotella; Prevotella paludivivens | Prevotella paludivivens | Prevotella | Prevotellaceae | Bacteroidales | Bacteroidia | Bacteroidetes | Bacteria |
| ASV8784 | 402 | 1 | 1 | 387 | 96.27 | NR_074613.1 [Eubacterium] eligens ATCC 27750 16S ribosomal RNA, partial sequence | NR_074613.1 | 1519 | 367 | 753 | 25.48 | 616.1 | 4E-176 | 369/387 | 95 | 0/387 | 0 | Plus | Plus | Bacteria; Firmicutes; Clostridia; Eubacteriales; Lachnospiraceae; Lachnospira; Lachnospira eligens | Lachnospira eligens | Lachnospira | Lachnospiraceae | Eubacteriales | Clostridia | Firmicutes | Bacteria |
| ASV8787 | 428 | 1 | 1 | 428 | 100 | NR_118554.1 Intestinimonas butyriciproducens strain SRB-521-5-I 16S ribosomal RNA, partial sequence | NR_118554.1 | 1475 | 346 | 750 | 27.46 | 442.5 | 8E-124 | 369/428 | 86 | 23/428 | 5 | Plus | Plus | Bacteria; Firmicutes; Clostridia; Eubacteriales; ; Intestinimonas; Intestinimonas butyriciproducens | Intestinimonas butyriciproducens | Intestinimonas | | Eubacteriales | Clostridia | Firmicutes | Bacteria |
| ASV8789 | 422 | 2 | 1 | 422 | 100 | NR_113073.1 Barnesiella intestinihominis strain JCM 15079 16S ribosomal RNA, partial sequence | NR_113073.1 | 1481 | 346 | 767 | 28.49 | 459.1 | 8E-129 | 366/424 | 86 | 4/424 | 0 | Plus | Plus | Bacteria; Bacteroidetes; Bacteroidia; Bacteroidales; Barnesiellaceae; Barnesiella; Barnesiella intestinihominis | Barnesiella intestinihominis | Barnesiella | Barnesiellaceae | Bacteroidales | Bacteroidia | Bacteroidetes | Bacteria |
| ASV8793 | 403 | 1 | 4 | 403 | 99.26 | NR_144749.1 Ihubacter massiliensis strain Marseille-P2843 16S ribosomal RNA, partial sequence | NR_144749.1 | 1479 | 367 | 766 | 27.05 | 507.1 | 3E-143 | 358/400 | 89 | 0/400 | 0 | Plus | Plus | Bacteria; Firmicutes; Clostridia; Eubacteriales; Clostridiales Family XIII. Incertae Sedis; Ihubacter; Ihubacter massiliensis | Ihubacter massiliensis | Ihubacter | Clostridiales Family XIII. Incertae Sedis | Eubacteriales | Clostridia | Firmicutes | Bacteria |
| ASV8794 | 428 | 4 | 4 | 428 | 99.3 | NR_042802.1 Lactobacillus ultunensis strain Kx146C1 16S ribosomal RNA, partial sequence | NR_042802.1 | 1550 | 373 | 796 | 27.35 | 562.5 | 6E-160 | 388/428 | 90 | 7/428 | 1 | Plus | Plus | Bacteria; Firmicutes; Bacilli; Lactobacillales; Lactobacillaceae; Lactobacillus; Lactobacillus ultunensis | Lactobacillus ultunensis | Lactobacillus | Lactobacillaceae | Lactobacillales | Bacilli | Firmicutes | Bacteria |

**Table S2** The 16 phyla and 336 genera identified by high-throughput 16s rRNA metagenomics sequencing in CON and RPS-fed groups at 20 dpf

| No. | Taxonomy | RPS10 | | | | | RPS5 | | | | | CON | | | | | | |
|---|---|---|---|---|---|---|---|---|---|---|---|---|---|---|---|---|---|---|
| | | 1 | 2 | 3 | 4 | Mean | 1 | 2 | 3 | 4 | Mean | 1 | 3 | 4 | 5 | 6 | 7 | Mean |
| | **Phylum (No. of reads (%))** | | | | | | | | | | | | | | | | | |
| 1 | Firmicutes | 11862 (61.35 | 14080 (73.41 | 12854 (68.53 | 14065 (75.25 | 13215 (69.59) | 14253 (75.85 | 11297 (61.82 | 15069 (79.78 | 8775 (47.51 | 12349 (66.37 | 15898 (84.39 | 13100 (69.68 | 12384 (65.56) | 12652 (67.32 | 9132 (48.68) | 15206 (81.34 | 13062 (69.49) |
| 2 | Bacteroidetes | 1147 (5.93) | 4239 (22.10 | 4062 (21.65) | 4076 (21.81) | 3381 (17.80) | 4200 (22.35) | 5416 (29.64) | 3452 (18.28) | 3268 (17.69 | 4084 (21.95) | 2496 (13.25) | 3790 (20.16) | 5599 (29.64) | 5175 (27.54) | 5655 (30.14) | 2332 (12.47) | 4175 (22.21) |
| 3 | Spirochaetes | 6019 (31.13 | 28 (0.15) | 4 (0.02) | 425 (2.27) | 1619 (8.52) | 162 (0.86) | 393 (2.15) | 254 (1.34) | 5713 (30.93 | 1631 (8.76) | 169 (0.90) | 1716 (9.13) | 613 (3.25) | 672 (3.58) | 3582 (19.09) | 593 (3.17) | 1224 (6.51) |
| 4 | Actinobacteria | 254 (1.31) | 611 (3.19) | 1633 (8.71) | 50 (0.27) | 637 (3.35) | 62 (0.33) | 154 (0.84) | 35 (0.19) | 48 (0.26) | 75 (0.40) | 123 (0.65) | 16 (0.09) | 26 (0.14) | 123 (0.65) | 25 (0.13) | 94 (0.50) | 68 (0.36) |
| 5 | Proteobacteria | 36 (0.19) | 70 (0.36) | 192 (1.02) | 65 (0.35) | 91 (0.48) | 71 (0.38) | 958 (5.24) | 53 (0.28) | 511 (2.77) | 398 (2.14) | 143 (0.76) | 127 (0.68) | 244 (1.29) | 156 (0.83) | 298 (1.59) | 261 (1.40) | 205 (1.09) |
| 6 | Tenericutes | 0 (0.00) | 136 (0.71) | 0 (0.00) | 3 (0.02) | 35 (0.18) | 7 (0.04) | 0 (0.00) | 12 (0.06) | 0 (0.00) | 5 (0.03) | 6 (0.03) | 42 (0.22) | 10 (0.05) | 11 (0.06) | 16 (0.09) | 15 (0.08) | 17 (0.09) |
| 7 | Chlamydiae | 17 (0.09) | 13 (0.07) | 13 (0.07) | 0 (0.00) | 11 (0.06) | 7 (0.04) | 15 (0.08) | 0 (0.00) | 128 (0.69) | 38 (0.20) | 0 (0.00) | 0 (0.00) | 0 (0.00) | 2 (0.01) | 9 (0.05) | 179 (0.96) | 32 (0.17) |
| 8 | Candidatus Melainabacteria | 0 (0.00) | 3 (0.02) | 0 (0.00) | 6 (0.03) | 2 (0.01) | 26 (0.14) | 38 (0.21) | 10 (0.05) | 0 (0.00) | 19 (0.10) | 0 (0.00) | 0 (0.00) | 4 (0.02) | 2 (0.01) | 0 (0.00) | 4 (0.02) | 2 (0.01) |
| 9 | Fibrobacteres | 0 (0.00) | 0 (0.00) | 0 (0.00) | 2 (0.01) | 1 (0.00) | 0 (0.00) | 0 (0.00) | 0 (0.00) | 2 (0.01) | 1 (0.00) | 0 (0.00) | 1 (0.01) | 4 (0.02) | 0 (0.00) | 43 (0.23) | 4 (0.02) | 9 (0.05) |
| 10 | Cyanobacteria | 0 (0.00) | 0 (0.00) | 0 (0.00) | 0 (0.00) | 0 (0.00) | 0 (0.00) | 0 (0.00) | 0 (0.00) | 0 (0.00) | 0 (0.00) | 2 (0.01) | 0 (0.00) | 0 (0.00) | 0 (0.00) | 0 (0.00) | 0 (0.00) | 0 (0.00) |
| 11 | Deferribacteres | 0 (0.00) | 0 (0.00) | 0 (0.00) | 0 (0.00) | 0 (0.00) | 2 (0.01) | 0 (0.00) | 0 (0.00) | 26 (0.14) | 7 (0.04) | 2 (0.01) | 8 (0.04) | 6 (0.03) | 0 (0.00) | 0 (0.00) | 7 (0.04) | 4 (0.02) |
| 12 | Elusimicrobia | 0 (0.00) | 0 (0.00) | 0 (0.00) | 0 (0.00) | 0 (0.00) | 0 (0.00) | 0 (0.00) | 0 (0.00) | 0 (0.00) | 0 (0.00) | 0 (0.00) | 0 (0.00) | 0 (0.00) | 0 (0.00) | 0 (0.00) | 0 (0.00) | 0 (0.00) |
| 13 | Fusobacteria | 0 (0.00) | 0 (0.00) | 0 (0.00) | 0 (0.00) | 0 (0.00) | 0 (0.00) | 0 (0.00) | 3 (0.02) | 0 (0.00) | 1 (0.00) | 0 (0.00) | 0 (0.00) | 0 (0.00) | 0 (0.00) | 0 (0.00) | 0 (0.00) | 0 (0.00) |
| 14 | Lentisphaerae | 0 (0.00) | 0 (0.00) | 0 (0.00) | 0 (0.00) | 0 (0.00) | 0 (0.00) | 0 (0.00) | 0 (0.00) | 0 (0.00) | 0 (0.00) | 0 (0.00) | 0 (0.00) | 0 (0.00) | 0 (0.00) | 0 (0.00) | 0 (0.00) | 0 (0.00) |
| 15 | Synergistetes | 0 (0.00) | 0 (0.00) | 0 (0.00) | 0 (0.00) | 0 (0.00) | 0 (0.00) | 4 (0.02) | 0 (0.00) | 0 (0.00) | 1 (0.01) | 0 (0.00) | 0 (0.00) | 0 (0.00) | 0 (0.00) | 0 (0.00) | 0 (0.00) | 0 (0.00) |
| 16 | Verrucomicrobia | 0 (0.00) | 0 (0.00) | 0 (0.00) | 0 (0.00) | 0 (0.00) | 0 (0.00) | 0 (0.00) | 0 (0.00) | 0 (0.00) | 0 (0.00) | 0 (0.00) | 0 (0.00) | 0 (0.00) | 0 (0.00) | 0 (0.00) | 0 (0.00) | 0 (0.00) |
| | **Genus (No. of reads (%))** | | | | | | | | | | | | | | | | | |
| 1 | *Streptococcus* | 322 (1.67) | 36 (0.19) | 1060 (5.65) | 7747 (41.45) | 2291 (12.06) | 3700 (19.69) | 2000 (10.94) | 1178 (6.24) | 1540 (8.34) | 2105 (11.31) | 2084 (11.06) | 5880 (31.28) | 2725 (14.43) | 783 (4.17) | 472 (2.52) | 5825 (31.16) | 2962 (15.76) |
| 2 | *Prevotella* | 116 (0.60) | 619 (3.23) | 3895 (20.76) | 2351 (12.58) | 1745 (9.19) | 3191 (16.98) | 4630 (25.34) | 1056 (5.59) | 2435 (13.18 | 2828 (15.20) | 1178 (6.25) | 1914 (10.18) | 3423 (18.12) | 2385 (12.69) | 3068 (16.35) | 831 (4.45) | 2133 (11.35) |
| 3 | *Treponema* | 6019 (31.13 | 28 (0.15) | 4 (0.02) | 425 (2.27) | 1619 (8.52) | 162 (0.86) | 393 (2.15) | 254 (1.34) | 5689 (30.80 | 1625 (8.73) | 169 (0.90) | 1714 (9.12) | 604 (3.20) | 672 (3.58) | 3582 (19.09) | 577 (3.09) | 1220 (6.49) |
| 4 | *Lactobacillus* | 411 (2.13) | 3247 (16.93 | 1223 (6.52) | 826 (4.42) | 1427 (7.51) | 235 (1.25) | 580 (3.17) | 1926 (10.20) | 1961 (10.62 | 1176 (6.32) | 1136 (6.03) | 118 (0.63) | 485 (2.57) | 2472 (13.15) | 563 (3.00) | 190 (1.02) | 827 (4.40) |
| 5 | *Ruminococcus* | 4963 (25.67 | 159 (0.83) | 270 (1.44) | 126 (0.67) | 1380 (7.26) | 201 (1.07) | 387 (2.12) | 364 (1.93) | 252 (1.36) | 301 (1.62) | 112 (0.59) | 169 (0.90) | 295 (1.56) | 308 (1.64) | 181 (0.96) | 116 (0.62) | 197 (1.05) |
| 6 | *Megasphaera* | 881 (4.56) | 1360 (7.09) | 1651 (8.80) | 931 (4.98) | 1206 (6.35) | 2637 (14.03) | 601 (3.29) | 98 (0.52) | 712 (3.85) | 1012 (5.44) | 2508 (13.31) | 172 (0.91) | 1128 (5.97) | 165 (0.88) | 18 (0.10) | 1158 (6.19) | 858 (4.57) |
| 7 | *Eubacterium* | 303 (1.57) | 2040 (10.64 | 1463 (7.80) | 320 (1.71) | 1032 (5.43) | 218 (1.16) | 1010 (5.53) | 692 (3.66) | 104 (0.56) | 506 (2.72) | 386 (2.05) | 101 (0.54) | 257 (1.36) | 184 (0.98) | 91 (0.49) | 383 (2.05) | 234 (1.24) |
| 8 | *Clostridium* | 2067 (10.69 | 347 (1.81) | 52 (0.28) | 341 (1.82) | 702 (3.70) | 738 (3.93) | 619 (3.39) | 944 (5.00) | 446 (2.41) | 687 (3.69) | 1500 (7.96) | 1996 (10.62) | 1412 (7.47) | 2444 (13.00) | 3289 (17.53) | 967 (5.17) | 1935 (10.29) |
| 9 | *Olsenella* | 124 (0.64) | 529 (2.76) | 1556 (8.30) | 22 (0.12) | 558 (2.94) | 18 (0.10) | 45 (0.25) | 10 (0.05) | 43 (0.23) | 29 (0.16) | 55 (0.29) | 8 (0.04) | 21 (0.11) | 6 (0.03) | 1 (0.01) | 74 (0.40) | 28 (0.15) |
| 10 | *Duncaniella* | 8 (0.04) | 2039 (10.63 | 0 (0.00) | 69 (0.37) | 529 (2.79) | 191 (1.02) | 66 (0.36) | 24 (0.13) | 3 (0.02) | 71 (0.38) | 30 (0.16) | 0 (0.00) | 0 (0.00) | 6 (0.03) | 10 (0.05) | 145 (0.78) | 32 (0.17) |
| 11 | *Oribacterium* | 13 (0.07) | 682 (3.56) | 951 (5.07) | 49 (0.26) | 424 (2.23) | 377 (2.01) | 501 (2.74) | 50 (0.26) | 56 (0.30) | 246 (1.32) | 298 (1.58) | 32 (0.17) | 362 (1.92) | 60 (0.32) | 16 (0.09) | 195 (1.04) | 161 (0.85) |
| 12 | *Parabacteroides* | 901 (4.66) | 9 (0.05) | 0 (0.00) | 769 (4.11) | 420 (2.21) | 42 (0.22) | 73 (0.40) | 47 (0.25) | 436 (2.36) | 150 (0.80) | 37 (0.20) | 431 (2.29) | 59 (0.31) | 46 (0.24) | 1071 (5.71) | 330 (1.77) | 329 (1.75) |
| 13 | *Dialister* | 15 (0.08) | 377 (1.97) | 1038 (5.53) | 61 (0.33) | 373 (1.96) | 158 (0.84) | 217 (1.19) | 136 (0.72) | 7 (0.04) | 130 (0.70) | 238 (1.26) | 66 (0.35) | 40 (0.21) | 2 (0.01) | 0 (0.00) | 288 (1.54) | 106 (0.56) |
| 14 | *Muribaculum* | 3 (0.02) | 1047 (5.46) | 26 (0.14) | 280 (1.50) | 339 (1.79) | 49 (0.26) | 300 (1.64) | 0 (0.00) | 107 (0.58) | 114 (0.61) | 22 (0.12) | 11 (0.06) | 41 (0.22) | 184 (0.98) | 101 (0.54) | 37 (0.20) | 66 (0.35) |
| 15 | *Oscillibacter* | 96 (0.50) | 526 (2.74) | 186 (0.99) | 454 (2.43) | 316 (1.66) | 336 (1.79) | 350 (1.92) | 170 (0.90) | 434 (2.35) | 323 (1.73) | 292 (1.55) | 435 (2.31) | 461 (2.44) | 586 (3.12) | 604 (3.22) | 607 (3.25) | 498 (2.65) |
| 16 | *Flintibacter* | 11 (0.06) | 734 (3.83) | 80 (0.43) | 212 (1.13) | 259 (1.37) | 273 (1.45) | 160 (0.88) | 242 (1.28) | 78 (0.42) | 188 (1.01) | 197 (1.05) | 257 (1.37) | 197 (1.04) | 220 (1.17) | 182 (0.97) | 695 (3.72) | 291 (1.55) |
| 17 | *Terrisporobacter* | 788 (4.08) | 135 (0.70) | 0 (0.00) | 27 (0.14) | 238 (1.25) | 135 (0.72) | 225 (1.23) | 613 (3.25) | 224 (1.21) | 299 (1.61) | 399 (2.12) | 323 (1.72) | 375 (1.99) | 536 (2.85) | 436 (2.32) | 310 (1.66) | 397 (2.11) |
| 18 | *Catenibacterium* | 217 (1.12) | 149 (0.78) | 537 (2.86) | 25 (0.13) | 232 (1.22) | 99 (0.53) | 0 (0.00) | 19 (0.10) | 60 (0.32) | 45 (0.24) | 51 (0.27) | 36 (0.19) | 10 (0.05) | 0 (0.00) | 19 (0.10) | 37 (0.20) | 26 (0.14) |
| 19 | *Butyrivibrio* | 10 (0.05) | 24 (0.13) | 841 (4.48) | 4 (0.02) | 220 (1.16) | 9 (0.05) | 27 (0.15) | 9 (0.05) | 0 (0.00) | 11 (0.06) | 13 (0.07) | 0 (0.00) | 8 (0.04) | 0 (0.00) | 0 (0.00) | 54 (0.29) | 13 (0.07) |
| 20 | *Anaerobacterium* | 3 (0.02) | 301 (1.57) | 439 (2.34) | 123 (0.66) | 217 (1.14) | 326 (1.73) | 226 (1.24) | 305 (1.61) | 79 (0.43) | 234 (1.26) | 284 (1.51) | 34 (0.18) | 174 (0.92) | 205 (1.09) | 11 (0.06) | 130 (0.70) | 140 (0.74) |
| 21 | *Acetanaerobacterium* | 0 (0.00) | 787 (4.10) | 35 (0.19) | 37 (0.20) | 215 (1.13) | 21 (0.11) | 13 (0.07) | 0 (0.00) | 52 (0.28) | 22 (0.12) | 84 (0.45) | 14 (0.07) | 150 (0.79) | 0 (0.00) | 18 (0.10) | 4 (0.02) | 45 (0.24) |
| 22 | *Barnesiella* | 115 (0.59) | 416 (2.17) | 60 (0.32) | 262 (1.40) | 213 (1.12) | 401 (2.13) | 159 (0.87) | 710 (3.76) | 92 (0.50) | 341 (1.83) | 1010 (5.36) | 1210 (6.44) | 1623 (8.59) | 2078 (11.06) | 1135 (6.05) | 513 (2.74) | 1262 (6.71) |
| 23 | *Blautia* | 333 (1.72) | 321 (1.67) | 61 (0.33) | 129 (0.69) | 211 (1.11) | 626 (3.33) | 352 (1.93) | 512 (2.71) | 140 (0.76) | 408 (2.19) | 216 (1.15) | 103 (0.55) | 141 (0.75) | 198 (1.05) | 62 (0.33) | 159 (0.85) | 147 (0.78) |
| 24 | *Mitsuokella* | 0 (0.00) | 11 (0.06) | 499 (2.66) | 36 (0.19) | 137 (0.72) | 214 (1.14) | 377 (2.06) | 81 (0.43) | 9 (0.05) | 170 (0.92) | 233 (1.24) | 69 (0.37) | 141 (0.75) | 63 (0.34) | 111 (0.59) | 415 (2.22) | 172 (0.92) |
| 25 | *Butyricicoccus* | 137 (0.71) | 96 (0.50) | 0 (0.00) | 261 (1.40) | 124 (0.65) | 241 (1.28) | 129 (0.71) | 73 (0.39) | 270 (1.46) | 178 (0.96) | 75 (0.40) | 80 (0.43) | 115 (0.61) | 200 (1.06) | 39 (0.21) | 8 (0.04) | 86 (0.46) |
| 26 | *Gemmiger* | 65 (0.34) | 135 (0.70) | 166 (0.88) | 46 (0.25) | 103 (0.54) | 305 (1.62) | 54 (0.30) | 1438 (7.61) | 78 (0.42) | 469 (2.52) | 429 (2.28) | 82 (0.44) | 70 (0.37) | 221 (1.18) | 18 (0.10) | 139 (0.74) | 160 (0.85) |
| 27 | *Holdemania* | 0 (0.00) | 61 (0.32) | 266 (1.42) | 81 (0.43) | 102 (0.54) | 18 (0.10) | 32 (0.18) | 32 (0.17) | 5 (0.03) | 22 (0.12) | 54 (0.29) | 11 (0.06) | 40 (0.21) | 70 (0.37) | 39 (0.21) | 57 (0.30) | 45 (0.24) |
| 28 | *Intestinimonas* | 43 (0.22) | 166 (0.87) | 123 (0.66) | 74 (0.40) | 102 (0.53) | 112 (0.60) | 238 (1.30) | 167 (0.88) | 214 (1.16) | 183 (0.98) | 289 (1.53) | 272 (1.45) | 318 (1.68) | 338 (1.80) | 312 (1.66) | 604 (3.23) | 356 (1.89) |
| 29 | *Acidaminococcus* | 23 (0.12) | 15 (0.08) | 331 (1.76) | 34 (0.18) | 101 (0.53) | 58 (0.31) | 33 (0.18) | 0 (0.00) | 10 (0.05) | 25 (0.14) | 18 (0.10) | 11 (0.06) | 8 (0.04) | 2 (0.01) | 0 (0.00) | 52 (0.28) | 15 (0.08) |
| 30 | *Sporobacter* | 0 (0.00) | 282 (1.47) | 11 (0.06) | 109 (0.58) | 101 (0.53) | 26 (0.14) | 136 (0.74) | 0 (0.00) | 92 (0.50) | 64 (0.34) | 70 (0.37) | 64 (0.34) | 34 (0.18) | 274 (1.46) | 78 (0.42) | 181 (0.97) | 117 (0.62) |
| 31 | *Selenomonas* | 0 (0.00) | 92 (0.48) | 235 (1.25) | 38 (0.20) | 91 (0.48) | 328 (1.75) | 1051 (5.75) | 84 (0.44) | 23 (0.12) | 372 (2.00) | 594 (3.15) | 91 (0.48) | 277 (1.47) | 0 (0.00) | 6 (0.03) | 490 (2.62) | 243 (1.29) |
| 32 | *Roseburia* | 29 (0.15) | 112 (0.58) | 0 (0.00) | 153 (0.82) | 74 (0.39) | 665 (3.54) | 574 (3.14) | 202 (1.07) | 148 (0.80) | 397 (2.14) | 227 (1.20) | 16 (0.09) | 146 (0.77) | 110 (0.59) | 5 (0.03) | 102 (0.55) | 101 (0.54) |
| 33 | *Limosilactobacillus* | 11 (0.06) | 0 (0.00) | 124 (0.66) | 73 (0.39) | 52 (0.27) | 46 (0.24) | 69 (0.38) | 357 (1.89) | 33 (0.18) | 126 (0.68) | 166 (0.88) | 4 (0.02) | 65 (0.34) | 5 (0.03) | 0 (0.00) | 3 (0.02) | 41 (0.22) |
| 34 | *Mediterraneibacter* | 16 (0.08) | 15 (0.08) | 150 (0.80) | 3 (0.02) | 46 (0.24) | 0 (0.00) | 0 (0.00) | 0 (0.00) | 32 (0.17) | 8 (0.04) | 32 (0.17) | 158 (0.84) | 100 (0.53) | 157 (0.84) | 386 (2.06) | 37 (0.20) | 145 (0.77) |
| 35 | *Vallitalea* | 0 (0.00) | 55 (0.29) | 0 (0.00) | 121 (0.65) | 44 (0.23) | 115 (0.61) | 60 (0.33) | 192 (1.02) | 70 (0.38) | 109 (0.59) | 278 (1.48) | 125 (0.66) | 269 (1.42) | 126 (0.67) | 128 (0.68) | 207 (1.11) | 189 (1.00) |
| 36 | *Faecalibacterium* | 0 (0.00) | 33 (0.17) | 0 (0.00) | 124 (0.66) | 39 (0.21) | 279 (1.48) | 179 (0.98) | 2408 (12.75) | 159 (0.86) | 756 (4.06) | 464 (2.46) | 66 (0.35) | 121 (0.64) | 72 (0.38) | 34 (0.18) | 40 (0.21) | 133 (0.71) |
| 37 | *Prevotellamassilia* | 0 (0.00) | 2 (0.01) | 0 (0.00) | 121 (0.65) | 31 (0.16) | 78 (0.42) | 28 (0.15) | 1494 (7.91) | 0 (0.00) | 400 (2.15) | 75 (0.40) | 11 (0.06) | 31 (0.16) | 69 (0.37) | 15 (0.08) | 37 (0.20) | 40 (0.21) |
| 38 | *Anaerovibrio* | 0 (0.00) | 9 (0.05) | 18 (0.10) | 41 (0.22) | 17 (0.09) | 113 (0.60) | 40 (0.22) | 48 (0.25) | 6 (0.03) | 52 (0.28) | 120 (0.64) | 17 (0.09) | 33 (0.17) | 65 (0.35) | 4 (0.02) | 37 (0.20) | 46 (0.24) |
| 39 | *Lacrimispora* | 0 (0.00) | 39 (0.20) | 0 (0.00) | 25 (0.13) | 16 (0.08) | 32 (0.17) | 81 (0.44) | 21 (0.11) | 13 (0.07) | 37 (0.20) | 629 (3.34) | 0 (0.00) | 27 (0.14) | 41 (0.22) | 291 (1.55) | 12 (0.06) | 167 (0.89) |
| 40 | *Pseudobutyrivibrio* | 19 (0.10) | 26 (0.14) | 7 (0.04) | 8 (0.04) | 15 (0.08) | 37 (0.20) | 0 (0.00) | 79 (0.42) | 14 (0.08) | 33 (0.17) | 43 (0.23) | 602 (3.20) | 601 (3.18) | 15 (0.08) | 8 (0.04) | 34 (0.18) | 217 (1.16) |
| 41 | *Leminorella* | 0 (0.00) | 4 (0.02) | 30 (0.16) | 0 (0.00) | 9 (0.04) | 0 (0.00) | 344 (1.88) | 0 (0.00) | 87 (0.47) | 108 (0.58) | 7 (0.04) | 19 (0.10) | 4 (0.02) | 0 (0.00) | 0 (0.00) | 3 (0.02) | 6 (0.03) |
| 42 | *Kineothrix* | 0 (0.00) | 2 (0.01) | 0 (0.00) | 24 (0.13) | 7 (0.03) | 57 (0.30) | 42 (0.23) | 0 (0.00) | 368 (1.99) | 117 (0.63) | 207 (1.10) | 304 (1.62) | 122 (0.65) | 127 (0.68) | 185 (0.99) | 288 (1.54) | 206 (1.09) |
| 43 | *Abyssivirga* | 2 (0.01) | 6 (0.03) | 0 (0.00) | 17 (0.09) | 6 (0.03) | 33 (0.18) | 95 (0.52) | 522 (2.76) | 18 (0.10) | 167 (0.90) | 45 (0.24) | 55 (0.29) | 148 (0.78) | 37 (0.20) | 0 (0.00) | 0 (0.00) | 48 (0.25) |
| 44 | *Paraprevotella* | 2 (0.01) | 0 (0.00) | 0 (0.00) | 11 (0.06) | 3 (0.02) | 53 (0.28) | 62 (0.34) | 44 (0.23) | 40 (0.22) | 50 (0.27) | 27 (0.14) | 81 (0.43) | 223 (1.18) | 274 (1.46) | 93 (0.50) | 99 (0.53) | 133 (0.71) |
| 45 | *Campylobacter* | 0 (0.00) | 3 (0.02) | 0 (0.00) | 9 (0.05) | 3 (0.02) | 0 (0.00) | 190 (1.04) | 13 (0.07) | 190 (1.03) | 98 (0.53) | 11 (0.06) | 48 (0.26) | 129 (0.68) | 1 (0.01) | 73 (0.39) | 144 (0.77) | 68 (0.36) |
| 46 | *Christensenella* | 0 (0.00) | 223 (1.16) | 17 (0.09) | 70 (0.37) | 78 (0.41) | 56 (0.30) | 32 (0.18) | 12 (0.06) | 83 (0.45) | 46 (0.25) | 35 (0.19) | 107 (0.57) | 30 (0.16) | 105 (0.56) | 159 (0.85) | 52 (0.28) | 81 (0.43) |
| 47 | *Alloprevotella* | 2 (0.01) | 84 (0.44) | 29 (0.15) | 141 (0.75) | 64 (0.34) | 121 (0.64) | 41 (0.22) | 31 (0.16) | 79 (0.43) | 68 (0.37) | 115 (0.61) | 37 (0.20) | 166 (0.88) | 42 (0.22) | 5 (0.03) | 74 (0.40) | 73 (0.39) |
| 48 | *Turicibacter* | 178 (0.92) | 41 (0.21) | 0 (0.00) | 10 (0.05) | 57 (0.30) | 65 (0.35) | 13 (0.07) | 205 (1.09) | 68 (0.37) | 88 (0.47) | 70 (0.37) | 76 (0.40) | 91 (0.48) | 68 (0.36) | 115 (0.61) | 36 (0.19) | 76 (0.40) |
| 49 | *Solobacterium* | 84 (0.43) | 72 (0.38) | 34 (0.18) | 33 (0.18) | 56 (0.29) | 66 (0.35) | 0 (0.00) | 68 (0.36) | 10 (0.05) | 36 (0.19) | 26 (0.14) | 22 (0.12) | 25 (0.13) | 5 (0.03) | 0 (0.00) | 7 (0.04) | 14 (0.08) |
| 50 | *Bifidobacterium* | 118 (0.61) | 8 (0.04) | 59 (0.31) | 16 (0.09) | 50 (0.26) | 34 (0.18) | 107 (0.59) | 12 (0.06) | 4 (0.02) | 39 (0.21) | 51 (0.27) | 8 (0.04) | 5 (0.03) | 100 (0.53) | 14 (0.07) | 0 (0.00) | 30 (0.16) |
| 51 | *Ihubacter* | 32 (0.17) | 60 (0.31) | 58 (0.31) | 47 (0.25) | 49 (0.26) | 28 (0.15) | 13 (0.07) | 35 (0.19) | 43 (0.23) | 30 (0.16) | 19 (0.10) | 37 (0.20) | 21 (0.11) | 125 (0.67) | 27 (0.14) | 57 (0.30) | 48 (0.25) |
| 52 | *Pseudoramibacter* | 8 (0.04) | 110 (0.57) | 73 (0.39) | 2 (0.01) | 48 (0.25) | 34 (0.18) | 35 (0.19) | 10 (0.05) | 30 (0.16) | 27 (0.15) | 0 (0.00) | 18 (0.10) | 25 (0.13) | 0 (0.00) | 0 (0.00) | 52 (0.28) | 16 (0.08) |
| 53 | *Lachnospira* | 19 (0.10) | 51 (0.27) | 10 (0.05) | 90 (0.48) | 43 (0.22) | 29 (0.15) | 92 (0.50) | 228 (1.21) | 5 (0.03) | 89 (0.48) | 178 (0.94) | 46 (0.24) | 160 (0.85) | 35 (0.19) | 0 (0.00) | 39 (0.21) | 76 (0.41) |
| 54 | *Anaeromassilibacillus* | 0 (0.00) | 67 (0.35) | 49 (0.26) | 52 (0.28) | 42 (0.22) | 36 (0.19) | 17 (0.09) | 13 (0.07) | 12 (0.06) | 20 (0.10) | 21 (0.11) | 29 (0.15) | 43 (0.23) | 20 (0.11) | 23 (0.12) | 26 (0.14) | 27 (0.14) |
| 55 | *Emergencia* | 50 (0.26) | 59 (0.31) | 0 (0.00) | 55 (0.29) | 41 (0.22) | 14 (0.07) | 26 (0.14) | 61 (0.32) | 26 (0.14) | 32 (0.17) | 64 (0.34) | 13 (0.07) | 0 (0.00) | 84 (0.45) | 48 (0.26) | 47 (0.25) | 43 (0.23) |
| 56 | *Gracilibacter* | 0 (0.00) | 42 (0.22) | 29 (0.15) | 92 (0.49) | 41 (0.21) | 0 (0.00) | 10 (0.05) | 0 (0.00) | 102 (0.55) | 28 (0.15) | 6 (0.03) | 17 (0.09) | 40 (0.21) | 319 (1.70) | 65 (0.35) | 15 (0.08) | 77 (0.41) |
| 57 | *Anaerocolumna* | 0 (0.00) | 0 (0.00) | 0 (0.00) | 163 (0.87) | 41 (0.21) | 15 (0.08) | 0 (0.00) | 0 (0.00) | 0 (0.00) | 4 (0.02) | 0 (0.00) | 0 (0.00) | 8 (0.04) | 0 (0.00) | 0 (0.00) | 41 (0.22) | 8 (0.04) |
| 58 | *Coprococcus* | 56 (0.29) | 57 (0.30) | 31 (0.17) | 16 (0.09) | 40 (0.21) | 46 (0.24) | 56 (0.31) | 32 (0.17) | 65 (0.35) | 50 (0.27) | 25 (0.13) | 44 (0.23) | 37 (0.20) | 40 (0.21) | 0 (0.00) | 37 (0.20) | 31 (0.16) |
| 59 | *Intestinibacter* | 18 (0.09) | 87 (0.45) | 3 (0.02) | 52 (0.28) | 40 (0.21) | 81 (0.43) | 0 (0.00) | 31 (0.16) | 14 (0.08) | 32 (0.17) | 83 (0.44) | 0 (0.00) | 19 (0.10) | 29 (0.15) | 8 (0.04) | 15 (0.08) | 26 (0.14) |
| 60 | *Desulfovibrio* | 0 (0.00) | 48 (0.25) | 65 (0.35) | 45 (0.24) | 40 (0.21) | 24 (0.13) | 24 (0.13) | 5 (0.03) | 18 (0.10) | 18 (0.10) | 48 (0.25) | 30 (0.16) | 46 (0.24) | 112 (0.60) | 70 (0.37) | 69 (0.37) | 63 (0.33) |
| 61 | *Faecalitalea* | 17 (0.09) | 16 (0.08) | 106 (0.57) | 16 (0.09) | 39 (0.20) | 0 (0.00) | 7 (0.04) | 0 (0.00) | 9 (0.05) | 4 (0.02) | 0 (0.00) | 18 (0.10) | 6 (0.03) | 0 (0.00) | 0 (0.00) | 5 (0.03) | 5 (0.03) |
| 62 | *Intestinibaculum* | 73 (0.38) | 0 (0.00) | 78 (0.42) | 0 (0.00) | 38 (0.20) | 5 (0.03) | 7 (0.04) | 0 (0.00) | 0 (0.00) | 3 (0.02) | 0 (0.00) | 29 (0.15) | 0 (0.00) | 0 (0.00) | 0 (0.00) | 0 (0.00) | 5 (0.03) |
| 63 | *Syntrophococcus* | 0 (0.00) | 0 (0.00) | 146 (0.78) | 0 (0.00) | 37 (0.19) | 0 (0.00) | 11 (0.06) | 0 (0.00) | 0 (0.00) | 3 (0.01) | 0 (0.00) | 0 (0.00) | 0 (0.00) | 0 (0.00) | 0 (0.00) | 43 (0.23) | 7 (0.04) |
| 64 | *Anaerobutyricum* | 92 (0.48) | 0 (0.00) | 0 (0.00) | 48 (0.26) | 35 (0.18) | 42 (0.22) | 29 (0.16) | 39 (0.21) | 66 (0.36) | 44 (0.24) | 78 (0.41) | 21 (0.11) | 18 (0.10) | 112 (0.60) | 22 (0.12) | 4 (0.02) | 43 (0.23) |
| 65 | *Spiroplasma* | 0 (0.00) | 136 (0.71) | 0 (0.00) | 3 (0.02) | 35 (0.18) | 7 (0.04) | 0 (0.00) | 7 (0.04) | 0 (0.00) | 4 (0.02) | 0 (0.00) | 7 (0.04) | 6 (0.03) | 11 (0.06) | 0 (0.00) | 9 (0.05) | 6 (0.03) |
| 66 | *Faecalicoccus* | 53 (0.27) | 51 (0.27) | 0 (0.00) | 30 (0.16) | 34 (0.18) | 0 (0.00) | 0 (0.00) | 0 (0.00) | 20 (0.11) | 5 (0.03) | 36 (0.19) | 23 (0.12) | 4 (0.02) | 51 (0.27) | 13 (0.07) | 0 (0.00) | 21 (0.11) |
| 67 | *Ligilactobacillus* | 0 (0.00) | 0 (0.00) | 93 (0.50) | 28 (0.15) | 30 (0.16) | 44 (0.23) | 8 (0.04) | 0 (0.00) | 0 (0.00) | 13 (0.07) | 100 (0.53) | 0 (0.00) | 3 (0.02) | 0 (0.00) | 0 (0.00) | 1 (0.01) | 17 (0.09) |
| 68 | *Fusicatenibacter* | 76 (0.39) | 22 (0.11) | 9 (0.05) | 11 (0.06) | 30 (0.16) | 63 (0.34) | 0 (0.00) | 62 (0.33) | 29 (0.16) | 39 (0.21) | 9 (0.05) | 19 (0.10) | 47 (0.25) | 22 (0.12) | 56 (0.30) | 9 (0.05) | 27 (0.14) |
| 69 | *Anaerotruncus* | 0 (0.00) | 7 (0.04) | 52 (0.28) | 42 (0.22) | 25 (0.13) | 5 (0.03) | 0 (0.00) | 2 (0.01) | 14 (0.08) | 5 (0.03) | 51 (0.27) | 64 (0.34) | 26 (0.14) | 111 (0.59) | 27 (0.14) | 28 (0.15) | 51 (0.27) |
| 70 | *Holdemanella* | 46 (0.24) | 6 (0.03) | 22 (0.12) | 22 (0.12) | 24 (0.13) | 36 (0.19) | 14 (0.08) | 28 (0.15) | 14 (0.08) | 23 (0.12) | 34 (0.18) | 10 (0.05) | 12 (0.06) | 9 (0.05) | 2 (0.01) | 27 (0.14) | 16 (0.08) |
| 71 | *Collinsella* | 4 (0.02) | 56 (0.29) | 14 (0.07) | 10 (0.05) | 21 (0.11) | 9 (0.05) | 0 (0.00) | 7 (0.04) | 1 (0.01) | 4 (0.02) | 4 (0.02) | 0 (0.00) | 0 (0.00) | 11 (0.06) | 7 (0.04) | 20 (0.11) | 7 (0.04) |
| 72 | *Mogibacterium* | 17 (0.09) | 10 (0.05) | 27 (0.14) | 28 (0.15) | 21 (0.11) | 4 (0.02) | 5 (0.03) | 0 (0.00) | 25 (0.14) | 9 (0.05) | 14 (0.07) | 8 (0.04) | 10 (0.05) | 12 (0.06) | 7 (0.04) | 10 (0.05) | 10 (0.05) |
| 73 | *Succinivibrio* | 22 (0.11) | 15 (0.08) | 27 (0.14) | 11 (0.06) | 19 (0.10) | 34 (0.18) | 67 (0.37) | 0 (0.00) | 37 (0.20) | 35 (0.19) | 50 (0.27) | 14 (0.07) | 42 (0.22) | 3 (0.02) | 142 (0.76) | 12 (0.06) | 44 (0.23) |
| 74 | *Monoglobus* | 0 (0.00) | 17 (0.09) | 0 (0.00) | 57 (0.30) | 19 (0.10) | 0 (0.00) | 6 (0.03) | 33 (0.17) | 0 (0.00) | 10 (0.05) | 28 (0.15) | 20 (0.11) | 9 (0.05) | 10 (0.05) | 23 (0.12) | 21 (0.11) | 19 (0.10) |
| 75 | *Erysipelothrix* | 0 (0.00) | 22 (0.11) | 0 (0.00) | 49 (0.26) | 18 (0.09) | 19 (0.10) | 14 (0.08) | 0 (0.00) | 63 (0.34) | 24 (0.13) | 140 (0.74) | 35 (0.19) | 56 (0.30) | 137 (0.73) | 37 (0.20) | 42 (0.22) | 75 (0.40) |
| 76 | *Ethanoligenens* | 0 (0.00) | 63 (0.33) | 0 (0.00) | 5 (0.03) | 17 (0.09) | 22 (0.12) | 11 (0.06) | 36 (0.19) | 5 (0.03) | 19 (0.10) | 77 (0.41) | 9 (0.05) | 12 (0.06) | 26 (0.14) | 11 (0.06) | 24 (0.13) | 27 (0.14) |
| 77 | *Sharpea* | 68 (0.35) | 0 (0.00) | 0 (0.00) | 0 (0.00) | 17 (0.09) | 0 (0.00) | 26 (0.14) | 0 (0.00) | 0 (0.00) | 7 (0.03) | 0 (0.00) | 0 (0.00) | 0 (0.00) | 2 (0.01) | 0 (0.00) | 12 (0.06) | 2 (0.01) |
| 78 | Unidentified (1) | 11 (0.06) | 2 (0.01) | 55 (0.29) | 0 (0.00) | 17 (0.09) | 0 (0.00) | 0 (0.00) | 0 (0.00) | 0 (0.00) | 0 (0.00) | 0 (0.00) | 0 (0.00) | 0 (0.00) | 0 (0.00) | 0 (0.00) | 0 (0.00) | 0 (0.00) |
| [illegible] | [illegible] | [illegible] | [illegible] | [illegible] | [illegible] | [illegible] | [illegible] | [illegible] | [illegible] | [illegible] | [illegible] | [illegible] | [illegible] | [illegible] | [illegible] | [illegible] | [illegible] | [illegible] |

| | | | | | | | | | | | | | | | | | | |
|---|---|---|---|---|---|---|---|---|---|---|---|---|---|---|---|---|---|---|
| 195 | *Mobilitalea* | 0 (0.00) | 0 (0.00) | 0 (0.00) | 0 (0.00) | 0 (0.00) | 0 (0.00) | 0 (0.00) | 0 (0.00) | 0 (0.00) | 0 (0.00) | 0 (0.00) | 0 (0.00) | 0 (0.00) | 0 (0.00) | 0 (0.00) | 0 (0.00) | 0 (0.00) |
| 196 | *Actinobacillus* | 0 (0.00) | 0 (0.00) | 0 (0.00) | 0 (0.00) | 0 (0.00) | 0 (0.00) | 0 (0.00) | 0 (0.00) | 0 (0.00) | 0 (0.00) | 0 (0.00) | 0 (0.00) | 0 (0.00) | 0 (0.00) | 0 (0.00) | 0 (0.00) | 0 (0.00) |
| 197 | *Mailhella* | 0 (0.00) | 0 (0.00) | 0 (0.00) | 0 (0.00) | 0 (0.00) | 0 (0.00) | 0 (0.00) | 0 (0.00) | 0 (0.00) | 0 (0.00) | 0 (0.00) | 0 (0.00) | 0 (0.00) | 0 (0.00) | 0 (0.00) | 0 (0.00) | 0 (0.00) |
| 198 | *Massiliprevotella* | 0 (0.00) | 0 (0.00) | 0 (0.00) | 0 (0.00) | 0 (0.00) | 0 (0.00) | 0 (0.00) | 0 (0.00) | 0 (0.00) | 0 (0.00) | 0 (0.00) | 0 (0.00) | 0 (0.00) | 0 (0.00) | 0 (0.00) | 0 (0.00) | 0 (0.00) |
| 199 | *Duodenibacillus* | 0 (0.00) | 0 (0.00) | 0 (0.00) | 0 (0.00) | 0 (0.00) | 0 (0.00) | 0 (0.00) | 0 (0.00) | 0 (0.00) | 0 (0.00) | 0 (0.00) | 0 (0.00) | 0 (0.00) | 0 (0.00) | 0 (0.00) | 0 (0.00) | 0 (0.00) |
| 200 | Unidentified (5) | 0 (0.00) | 0 (0.00) | 0 (0.00) | 0 (0.00) | 0 (0.00) | 0 (0.00) | 0 (0.00) | 0 (0.00) | 0 (0.00) | 0 (0.00) | 0 (0.00) | 0 (0.00) | 0 (0.00) | 0 (0.00) | 28 (0.15) | 0 (0.00) | 5 (0.02) |
| 201 | *Muricomes* | 0 (0.00) | 0 (0.00) | 0 (0.00) | 0 (0.00) | 0 (0.00) | 0 (0.00) | 0 (0.00) | 0 (0.00) | 0 (0.00) | 0 (0.00) | 0 (0.00) | 0 (0.00) | 0 (0.00) | 0 (0.00) | 0 (0.00) | 0 (0.00) | 0 (0.00) |
| 202 | *Asteroleplasma* | 0 (0.00) | 0 (0.00) | 0 (0.00) | 0 (0.00) | 0 (0.00) | 0 (0.00) | 0 (0.00) | 0 (0.00) | 0 (0.00) | 0 (0.00) | 0 (0.00) | 0 (0.00) | 0 (0.00) | 0 (0.00) | 0 (0.00) | 0 (0.00) | 0 (0.00) |
| 203 | *Cloacibacillus* | 0 (0.00) | 0 (0.00) | 0 (0.00) | 0 (0.00) | 0 (0.00) | 0 (0.00) | 0 (0.00) | 0 (0.00) | 0 (0.00) | 0 (0.00) | 0 (0.00) | 0 (0.00) | 0 (0.00) | 0 (0.00) | 0 (0.00) | 0 (0.00) | 0 (0.00) |
| 204 | *Desulfohalotomaculum* | 0 (0.00) | 0 (0.00) | 0 (0.00) | 0 (0.00) | 0 (0.00) | 0 (0.00) | 0 (0.00) | 0 (0.00) | 0 (0.00) | 0 (0.00) | 0 (0.00) | 0 (0.00) | 0 (0.00) | 0 (0.00) | 0 (0.00) | 0 (0.00) | 0 (0.00) |
| 205 | *Leptotrichia* | 0 (0.00) | 0 (0.00) | 0 (0.00) | 0 (0.00) | 0 (0.00) | 0 (0.00) | 0 (0.00) | 0 (0.00) | 0 (0.00) | 0 (0.00) | 0 (0.00) | 0 (0.00) | 0 (0.00) | 0 (0.00) | 0 (0.00) | 0 (0.00) | 0 (0.00) |
| 206 | *Lacibacterium* | 0 (0.00) | 0 (0.00) | 0 (0.00) | 0 (0.00) | 0 (0.00) | 0 (0.00) | 0 (0.00) | 0 (0.00) | 0 (0.00) | 0 (0.00) | 0 (0.00) | 0 (0.00) | 0 (0.00) | 0 (0.00) | 0 (0.00) | 0 (0.00) | 0 (0.00) |
| 207 | *Gordonibacter* | 0 (0.00) | 0 (0.00) | 0 (0.00) | 0 (0.00) | 0 (0.00) | 0 (0.00) | 0 (0.00) | 0 (0.00) | 0 (0.00) | 0 (0.00) | 0 (0.00) | 0 (0.00) | 0 (0.00) | 0 (0.00) | 0 (0.00) | 0 (0.00) | 0 (0.00) |
| 208 | *Oceanirhabdus* | 0 (0.00) | 0 (0.00) | 0 (0.00) | 0 (0.00) | 0 (0.00) | 0 (0.00) | 0 (0.00) | 0 (0.00) | 0 (0.00) | 0 (0.00) | 0 (0.00) | 0 (0.00) | 0 (0.00) | 0 (0.00) | 0 (0.00) | 12 (0.06) | 2 (0.01) |
| 209 | *Sutterella* | 0 (0.00) | 0 (0.00) | 0 (0.00) | 0 (0.00) | 0 (0.00) | 0 (0.00) | 0 (0.00) | 0 (0.00) | 0 (0.00) | 0 (0.00) | 0 (0.00) | 0 (0.00) | 0 (0.00) | 0 (0.00) | 0 (0.00) | 0 (0.00) | 0 (0.00) |
| 210 | *Sphaerochaeta* | 0 (0.00) | 0 (0.00) | 0 (0.00) | 0 (0.00) | 0 (0.00) | 0 (0.00) | 0 (0.00) | 0 (0.00) | 0 (0.00) | 0 (0.00) | 0 (0.00) | 0 (0.00) | 0 (0.00) | 3 (0.02) | 0 (0.00) | 0 (0.00) | 1 (0.00) |
| 211 | *Caloramator* | 0 (0.00) | 0 (0.00) | 0 (0.00) | 0 (0.00) | 0 (0.00) | 0 (0.00) | 0 (0.00) | 0 (0.00) | 0 (0.00) | 0 (0.00) | 0 (0.00) | 0 (0.00) | 0 (0.00) | 0 (0.00) | 0 (0.00) | 0 (0.00) | 0 (0.00) |
| 212 | *Asaccharospora* | 0 (0.00) | 0 (0.00) | 0 (0.00) | 0 (0.00) | 0 (0.00) | 0 (0.00) | 0 (0.00) | 0 (0.00) | 0 (0.00) | 0 (0.00) | 0 (0.00) | 0 (0.00) | 0 (0.00) | 0 (0.00) | 0 (0.00) | 0 (0.00) | 0 (0.00) |
| 213 | *Fannyhessea* | 0 (0.00) | 0 (0.00) | 0 (0.00) | 0 (0.00) | 0 (0.00) | 0 (0.00) | 0 (0.00) | 0 (0.00) | 0 (0.00) | 0 (0.00) | 2 (0.01) | 0 (0.00) | 0 (0.00) | 0 (0.00) | 0 (0.00) | 0 (0.00) | 0 (0.00) |
| 214 | *Trueperella* | 0 (0.00) | 0 (0.00) | 0 (0.00) | 0 (0.00) | 0 (0.00) | 0 (0.00) | 0 (0.00) | 0 (0.00) | 0 (0.00) | 0 (0.00) | 0 (0.00) | 0 (0.00) | 0 (0.00) | 0 (0.00) | 0 (0.00) | 0 (0.00) | 0 (0.00) |
| 215 | *Pseudoscardovia* | 0 (0.00) | 0 (0.00) | 0 (0.00) | 0 (0.00) | 0 (0.00) | 0 (0.00) | 0 (0.00) | 0 (0.00) | 0 (0.00) | 0 (0.00) | 0 (0.00) | 0 (0.00) | 0 (0.00) | 0 (0.00) | 0 (0.00) | 0 (0.00) | 0 (0.00) |
| 216 | *Corynebacterium* | 0 (0.00) | 0 (0.00) | 0 (0.00) | 0 (0.00) | 0 (0.00) | 0 (0.00) | 0 (0.00) | 0 (0.00) | 0 (0.00) | 0 (0.00) | 0 (0.00) | 0 (0.00) | 0 (0.00) | 0 (0.00) | 0 (0.00) | 0 (0.00) | 0 (0.00) |
| 217 | *Nesterenkonia* | 0 (0.00) | 0 (0.00) | 0 (0.00) | 0 (0.00) | 0 (0.00) | 0 (0.00) | 0 (0.00) | 0 (0.00) | 0 (0.00) | 0 (0.00) | 0 (0.00) | 0 (0.00) | 0 (0.00) | 0 (0.00) | 0 (0.00) | 0 (0.00) | 0 (0.00) |
| 218 | *Rothia* | 0 (0.00) | 0 (0.00) | 0 (0.00) | 0 (0.00) | 0 (0.00) | 0 (0.00) | 0 (0.00) | 0 (0.00) | 0 (0.00) | 0 (0.00) | 0 (0.00) | 0 (0.00) | 0 (0.00) | 0 (0.00) | 0 (0.00) | 0 (0.00) | 0 (0.00) |
| 219 | *Nocardioides* | 0 (0.00) | 0 (0.00) | 0 (0.00) | 0 (0.00) | 0 (0.00) | 0 (0.00) | 0 (0.00) | 0 (0.00) | 0 (0.00) | 0 (0.00) | 0 (0.00) | 0 (0.00) | 0 (0.00) | 0 (0.00) | 0 (0.00) | 0 (0.00) | 0 (0.00) |
| 220 | *Actinomadura* | 0 (0.00) | 0 (0.00) | 0 (0.00) | 0 (0.00) | 0 (0.00) | 0 (0.00) | 0 (0.00) | 0 (0.00) | 0 (0.00) | 0 (0.00) | 0 (0.00) | 0 (0.00) | 0 (0.00) | 0 (0.00) | 0 (0.00) | 2 (0.01) | 0 (0.00) |
| 221 | *Parvibacter* | 0 (0.00) | 0 (0.00) | 0 (0.00) | 0 (0.00) | 0 (0.00) | 0 (0.00) | 0 (0.00) | 0 (0.00) | 0 (0.00) | 0 (0.00) | 0 (0.00) | 6 (0.03) | 0 (0.00) | 0 (0.00) | 0 (0.00) | 0 (0.00) | 1 (0.01) |
| 222 | *Eggerthella* | 0 (0.00) | 0 (0.00) | 0 (0.00) | 0 (0.00) | 0 (0.00) | 0 (0.00) | 0 (0.00) | 0 (0.00) | 0 (0.00) | 0 (0.00) | 0 (0.00) | 0 (0.00) | 0 (0.00) | 0 (0.00) | 0 (0.00) | 0 (0.00) | 0 (0.00) |
| 223 | *Raoultibacter* | 0 (0.00) | 0 (0.00) | 0 (0.00) | 0 (0.00) | 0 (0.00) | 0 (0.00) | 0 (0.00) | 0 (0.00) | 0 (0.00) | 0 (0.00) | 0 (0.00) | 0 (0.00) | 1 (0.01) | 0 (0.00) | 0 (0.00) | 0 (0.00) | 0 (0.00) |
| 224 | *Phocaeicola* | 0 (0.00) | 0 (0.00) | 0 (0.00) | 0 (0.00) | 0 (0.00) | 0 (0.00) | 0 (0.00) | 0 (0.00) | 0 (0.00) | 0 (0.00) | 0 (0.00) | 0 (0.00) | 0 (0.00) | 0 (0.00) | 0 (0.00) | 0 (0.00) | 0 (0.00) |
| 225 | *Coprobacter* | 0 (0.00) | 0 (0.00) | 0 (0.00) | 0 (0.00) | 0 (0.00) | 0 (0.00) | 0 (0.00) | 0 (0.00) | 0 (0.00) | 0 (0.00) | 0 (0.00) | 0 (0.00) | 0 (0.00) | 0 (0.00) | 0 (0.00) | 0 (0.00) | 0 (0.00) |
| 226 | *Lentimicrobium* | 0 (0.00) | 0 (0.00) | 0 (0.00) | 0 (0.00) | 0 (0.00) | 0 (0.00) | 0 (0.00) | 0 (0.00) | 0 (0.00) | 0 (0.00) | 0 (0.00) | 0 (0.00) | 0 (0.00) | 0 (0.00) | 0 (0.00) | 2 (0.01) | 0 (0.00) |
| 227 | *Culturomica* | 0 (0.00) | 0 (0.00) | 0 (0.00) | 0 (0.00) | 0 (0.00) | 0 (0.00) | 0 (0.00) | 0 (0.00) | 0 (0.00) | 0 (0.00) | 0 (0.00) | 0 (0.00) | 0 (0.00) | 0 (0.00) | 0 (0.00) | 0 (0.00) | 0 (0.00) |
| 228 | *Odoribacter* | 0 (0.00) | 0 (0.00) | 0 (0.00) | 0 (0.00) | 0 (0.00) | 0 (0.00) | 0 (0.00) | 0 (0.00) | 0 (0.00) | 0 (0.00) | 0 (0.00) | 0 (0.00) | 0 (0.00) | 0 (0.00) | 0 (0.00) | 0 (0.00) | 0 (0.00) |
| 229 | *Gabonibacter* | 0 (0.00) | 0 (0.00) | 0 (0.00) | 0 (0.00) | 0 (0.00) | 0 (0.00) | 0 (0.00) | 0 (0.00) | 0 (0.00) | 0 (0.00) | 0 (0.00) | 0 (0.00) | 4 (0.02) | 0 (0.00) | 0 (0.00) | 0 (0.00) | 1 (0.00) |
| 230 | *Macellibacteroides* | 0 (0.00) | 0 (0.00) | 0 (0.00) | 0 (0.00) | 0 (0.00) | 0 (0.00) | 0 (0.00) | 0 (0.00) | 0 (0.00) | 0 (0.00) | 0 (0.00) | 0 (0.00) | 0 (0.00) | 0 (0.00) | 0 (0.00) | 0 (0.00) | 0 (0.00) |
| 231 | *Porphyromonas* | 0 (0.00) | 0 (0.00) | 0 (0.00) | 0 (0.00) | 0 (0.00) | 0 (0.00) | 0 (0.00) | 0 (0.00) | 0 (0.00) | 0 (0.00) | 0 (0.00) | 0 (0.00) | 0 (0.00) | 0 (0.00) | 0 (0.00) | 0 (0.00) | 0 (0.00) |
| 232 | *Marinifilum* | 0 (0.00) | 0 (0.00) | 0 (0.00) | 0 (0.00) | 0 (0.00) | 0 (0.00) | 0 (0.00) | 0 (0.00) | 0 (0.00) | 0 (0.00) | 0 (0.00) | 1 (0.01) | 0 (0.00) | 0 (0.00) | 0 (0.00) | 0 (0.00) | 0 (0.00) |
| 233 | *Alkaliflexus* | 0 (0.00) | 0 (0.00) | 0 (0.00) | 0 (0.00) | 0 (0.00) | 0 (0.00) | 0 (0.00) | 0 (0.00) | 0 (0.00) | 0 (0.00) | 0 (0.00) | 0 (0.00) | 0 (0.00) | 0 (0.00) | 0 (0.00) | 0 (0.00) | 0 (0.00) |
| 234 | *Breznakibacter* | 0 (0.00) | 0 (0.00) | 0 (0.00) | 0 (0.00) | 0 (0.00) | 0 (0.00) | 0 (0.00) | 0 (0.00) | 0 (0.00) | 0 (0.00) | 0 (0.00) | 0 (0.00) | 0 (0.00) | 0 (0.00) | 0 (0.00) | 0 (0.00) | 0 (0.00) |
| 235 | *Geofilum* | 0 (0.00) | 0 (0.00) | 0 (0.00) | 0 (0.00) | 0 (0.00) | 0 (0.00) | 0 (0.00) | 0 (0.00) | 0 (0.00) | 0 (0.00) | 0 (0.00) | 0 (0.00) | 0 (0.00) | 0 (0.00) | 0 (0.00) | 0 (0.00) | 0 (0.00) |
| 236 | *Mangroviflexus* | 0 (0.00) | 0 (0.00) | 0 (0.00) | 0 (0.00) | 0 (0.00) | 0 (0.00) | 0 (0.00) | 0 (0.00) | 0 (0.00) | 0 (0.00) | 0 (0.00) | 0 (0.00) | 0 (0.00) | 0 (0.00) | 0 (0.00) | 0 (0.00) | 0 (0.00) |
| 237 | *Marinilabilia* | 0 (0.00) | 0 (0.00) | 0 (0.00) | 0 (0.00) | 0 (0.00) | 0 (0.00) | 0 (0.00) | 0 (0.00) | 0 (0.00) | 0 (0.00) | 0 (0.00) | 0 (0.00) | 0 (0.00) | 19 (0.10) | 0 (0.00) | 12 (0.06) | 5 (0.03) |
| 238 | *Aureibacter* | 0 (0.00) | 0 (0.00) | 0 (0.00) | 0 (0.00) | 0 (0.00) | 0 (0.00) | 0 (0.00) | 0 (0.00) | 0 (0.00) | 0 (0.00) | 0 (0.00) | 0 (0.00) | 0 (0.00) | 0 (0.00) | 0 (0.00) | 0 (0.00) | 0 (0.00) |
| 239 | *Portibacter* | 0 (0.00) | 0 (0.00) | 0 (0.00) | 0 (0.00) | 0 (0.00) | 0 (0.00) | 0 (0.00) | 0 (0.00) | 0 (0.00) | 0 (0.00) | 0 (0.00) | 0 (0.00) | 0 (0.00) | 0 (0.00) | 2 (0.01) | 0 (0.00) | 0 (0.00) |
| 240 | *Olivibacter* | 0 (0.00) | 0 (0.00) | 0 (0.00) | 0 (0.00) | 0 (0.00) | 0 (0.00) | 0 (0.00) | 0 (0.00) | 0 (0.00) | 0 (0.00) | 11 (0.06) | 0 (0.00) | 0 (0.00) | 0 (0.00) | 0 (0.00) | 0 (0.00) | 2 (0.01) |
| 241 | *Sphingobacterium* | 0 (0.00) | 0 (0.00) | 0 (0.00) | 0 (0.00) | 0 (0.00) | 0 (0.00) | 0 (0.00) | 0 (0.00) | 0 (0.00) | 0 (0.00) | 0 (0.00) | 0 (0.00) | 0 (0.00) | 0 (0.00) | 0 (0.00) | 0 (0.00) | 0 (0.00) |
| 242 | *Aerosakkonema* | 0 (0.00) | 0 (0.00) | 0 (0.00) | 0 (0.00) | 0 (0.00) | 0 (0.00) | 0 (0.00) | 0 (0.00) | 0 (0.00) | 0 (0.00) | 0 (0.00) | 0 (0.00) | 0 (0.00) | 0 (0.00) | 0 (0.00) | 0 (0.00) | 0 (0.00) |
| 243 | *Elusimicrobium* | 0 (0.00) | 0 (0.00) | 0 (0.00) | 0 (0.00) | 0 (0.00) | 0 (0.00) | 0 (0.00) | 0 (0.00) | 0 (0.00) | 0 (0.00) | 0 (0.00) | 0 (0.00) | 0 (0.00) | 0 (0.00) | 0 (0.00) | 0 (0.00) | 0 (0.00) |
| 244 | *Hydrogenispora* | 0 (0.00) | 0 (0.00) | 0 (0.00) | 0 (0.00) | 0 (0.00) | 0 (0.00) | 0 (0.00) | 0 (0.00) | 0 (0.00) | 0 (0.00) | 0 (0.00) | 0 (0.00) | 0 (0.00) | 4 (0.02) | 3 (0.02) | 0 (0.00) | 1 (0.01) |
| 245 | *Desulfuribacillus* | 0 (0.00) | 0 (0.00) | 0 (0.00) | 0 (0.00) | 0 (0.00) | 0 (0.00) | 0 (0.00) | 0 (0.00) | 0 (0.00) | 0 (0.00) | 0 (0.00) | 0 (0.00) | 0 (0.00) | 0 (0.00) | 0 (0.00) | 0 (0.00) | 0 (0.00) |
| 246 | *Gemella* | 0 (0.00) | 0 (0.00) | 0 (0.00) | 0 (0.00) | 0 (0.00) | 0 (0.00) | 0 (0.00) | 0 (0.00) | 0 (0.00) | 0 (0.00) | 0 (0.00) | 0 (0.00) | 0 (0.00) | 37 (0.20) | 0 (0.00) | 8 (0.04) | 8 (0.04) |
| 247 | *Effusibacillus* | 0 (0.00) | 0 (0.00) | 0 (0.00) | 0 (0.00) | 0 (0.00) | 0 (0.00) | 0 (0.00) | 0 (0.00) | 0 (0.00) | 0 (0.00) | 0 (0.00) | 0 (0.00) | 0 (0.00) | 0 (0.00) | 0 (0.00) | 0 (0.00) | 0 (0.00) |
| 248 | *Rossellomorea* | 0 (0.00) | 0 (0.00) | 0 (0.00) | 0 (0.00) | 0 (0.00) | 0 (0.00) | 0 (0.00) | 0 (0.00) | 0 (0.00) | 0 (0.00) | 0 (0.00) | 0 (0.00) | 0 (0.00) | 17 (0.09) | 20 (0.11) | 0 (0.00) | 6 (0.03) |
| 249 | *Brochothrix* | 0 (0.00) | 0 (0.00) | 0 (0.00) | 0 (0.00) | 0 (0.00) | 0 (0.00) | 0 (0.00) | 0 (0.00) | 0 (0.00) | 0 (0.00) | 0 (0.00) | 0 (0.00) | 2 (0.01) | 0 (0.00) | 0 (0.00) | 0 (0.00) | 0 (0.00) |
| 250 | *Staphylococcus* | 0 (0.00) | 0 (0.00) | 0 (0.00) | 0 (0.00) | 0 (0.00) | 0 (0.00) | 0 (0.00) | 0 (0.00) | 0 (0.00) | 0 (0.00) | 0 (0.00) | 0 (0.00) | 6 (0.03) | 9 (0.05) | 0 (0.00) | 0 (0.00) | 3 (0.01) |
| 251 | *Howardella* | 0 (0.00) | 0 (0.00) | 0 (0.00) | 0 (0.00) | 0 (0.00) | 0 (0.00) | 0 (0.00) | 0 (0.00) | 0 (0.00) | 0 (0.00) | 0 (0.00) | 0 (0.00) | 0 (0.00) | 0 (0.00) | 0 (0.00) | 0 (0.00) | 0 (0.00) |
| 252 | *Caldicoprobacter* | 0 (0.00) | 0 (0.00) | 0 (0.00) | 0 (0.00) | 0 (0.00) | 0 (0.00) | 0 (0.00) | 0 (0.00) | 0 (0.00) | 0 (0.00) | 12 (0.06) | 0 (0.00) | 0 (0.00) | 0 (0.00) | 0 (0.00) | 0 (0.00) | 2 (0.01) |
| 253 | *Anoxynatronum* | 0 (0.00) | 0 (0.00) | 0 (0.00) | 0 (0.00) | 0 (0.00) | 0 (0.00) | 0 (0.00) | 0 (0.00) | 0 (0.00) | 0 (0.00) | 0 (0.00) | 0 (0.00) | 0 (0.00) | 0 (0.00) | 0 (0.00) | 0 (0.00) | 0 (0.00) |
| 254 | *Brassicibacter* | 0 (0.00) | 0 (0.00) | 0 (0.00) | 0 (0.00) | 0 (0.00) | 0 (0.00) | 0 (0.00) | 0 (0.00) | 0 (0.00) | 0 (0.00) | 0 (0.00) | 0 (0.00) | 0 (0.00) | 0 (0.00) | 0 (0.00) | 0 (0.00) | 0 (0.00) |
| 255 | *Lactonifactor* | 0 (0.00) | 0 (0.00) | 0 (0.00) | 0 (0.00) | 0 (0.00) | 0 (0.00) | 0 (0.00) | 0 (0.00) | 0 (0.00) | 0 (0.00) | 0 (0.00) | 0 (0.00) | 0 (0.00) | 0 (0.00) | 0 (0.00) | 0 (0.00) | 0 (0.00) |
| 256 | *Marinisporobacter* | 0 (0.00) | 0 (0.00) | 0 (0.00) | 0 (0.00) | 0 (0.00) | 0 (0.00) | 0 (0.00) | 0 (0.00) | 0 (0.00) | 0 (0.00) | 0 (0.00) | 0 (0.00) | 0 (0.00) | 0 (0.00) | 0 (0.00) | 0 (0.00) | 0 (0.00) |
| 257 | *Tindallia* | 0 (0.00) | 0 (0.00) | 0 (0.00) | 0 (0.00) | 0 (0.00) | 0 (0.00) | 0 (0.00) | 0 (0.00) | 0 (0.00) | 0 (0.00) | 0 (0.00) | 0 (0.00) | 0 (0.00) | 0 (0.00) | 0 (0.00) | 0 (0.00) | 0 (0.00) |
| 258 | *Acidaminobacter* | 0 (0.00) | 0 (0.00) | 0 (0.00) | 0 (0.00) | 0 (0.00) | 0 (0.00) | 0 (0.00) | 0 (0.00) | 0 (0.00) | 0 (0.00) | 2 (0.01) | 0 (0.00) | 0 (0.00) | 0 (0.00) | 0 (0.00) | 0 (0.00) | 0 (0.00) |
| 259 | *Aminicella* | 0 (0.00) | 0 (0.00) | 0 (0.00) | 0 (0.00) | 0 (0.00) | 0 (0.00) | 0 (0.00) | 0 (0.00) | 0 (0.00) | 0 (0.00) | 0 (0.00) | 0 (0.00) | 0 (0.00) | 0 (0.00) | 0 (0.00) | 0 (0.00) | 0 (0.00) |
| 260 | *Anaerosporobacter* | 0 (0.00) | 0 (0.00) | 0 (0.00) | 0 (0.00) | 0 (0.00) | 0 (0.00) | 0 (0.00) | 0 (0.00) | 0 (0.00) | 0 (0.00) | 10 (0.05) | 42 (0.22) | 0 (0.00) | 3 (0.02) | 197 (1.05) | 2 (0.01) | 42 (0.23) |
| 261 | *Caecibacterium* | 0 (0.00) | 0 (0.00) | 0 (0.00) | 0 (0.00) | 0 (0.00) | 0 (0.00) | 0 (0.00) | 0 (0.00) | 0 (0.00) | 0 (0.00) | 0 (0.00) | 0 (0.00) | 0 (0.00) | 0 (0.00) | 0 (0.00) | 0 (0.00) | 0 (0.00) |
| 262 | *Catonella* | 0 (0.00) | 0 (0.00) | 0 (0.00) | 0 (0.00) | 0 (0.00) | 0 (0.00) | 0 (0.00) | 0 (0.00) | 0 (0.00) | 0 (0.00) | 0 (0.00) | 0 (0.00) | 0 (0.00) | 0 (0.00) | 0 (0.00) | 0 (0.00) | 0 (0.00) |
| 263 | *Cuneatibacter* | 0 (0.00) | 0 (0.00) | 0 (0.00) | 0 (0.00) | 0 (0.00) | 0 (0.00) | 0 (0.00) | 0 (0.00) | 0 (0.00) | 0 (0.00) | 0 (0.00) | 0 (0.00) | 0 (0.00) | 2 (0.01) | 0 (0.00) | 0 (0.00) | 0 (0.00) |
| 264 | *Faecalimonas* | 0 (0.00) | 0 (0.00) | 0 (0.00) | 0 (0.00) | 0 (0.00) | 0 (0.00) | 0 (0.00) | 0 (0.00) | 0 (0.00) | 0 (0.00) | 0 (0.00) | 0 (0.00) | 0 (0.00) | 0 (0.00) | 0 (0.00) | 0 (0.00) | 0 (0.00) |
| 265 | *Lachnoanaerobaculum* | 0 (0.00) | 0 (0.00) | 0 (0.00) | 0 (0.00) | 0 (0.00) | 0 (0.00) | 0 (0.00) | 0 (0.00) | 0 (0.00) | 0 (0.00) | 0 (0.00) | 0 (0.00) | 5 (0.03) | 0 (0.00) | 0 (0.00) | 0 (0.00) | 1 (0.00) |
| 266 | *Marvinbryantia* | 0 (0.00) | 0 (0.00) | 0 (0.00) | 0 (0.00) | 0 (0.00) | 0 (0.00) | 0 (0.00) | 0 (0.00) | 0 (0.00) | 0 (0.00) | 0 (0.00) | 0 (0.00) | 0 (0.00) | 0 (0.00) | 0 (0.00) | 0 (0.00) | 0 (0.00) |
| 267 | *Robinsoniella* | 0 (0.00) | 0 (0.00) | 0 (0.00) | 0 (0.00) | 0 (0.00) | 0 (0.00) | 0 (0.00) | 0 (0.00) | 0 (0.00) | 0 (0.00) | 0 (0.00) | 0 (0.00) | 1 (0.01) | 0 (0.00) | 0 (0.00) | 0 (0.00) | 0 (0.00) |
| 268 | *Sellimonas* | 0 (0.00) | 0 (0.00) | 0 (0.00) | 0 (0.00) | 0 (0.00) | 0 (0.00) | 0 (0.00) | 0 (0.00) | 0 (0.00) | 0 (0.00) | 0 (0.00) | 0 (0.00) | 0 (0.00) | 0 (0.00) | 0 (0.00) | 0 (0.00) | 0 (0.00) |
| 269 | *Stomatobaculum* | 0 (0.00) | 0 (0.00) | 0 (0.00) | 0 (0.00) | 0 (0.00) | 0 (0.00) | 0 (0.00) | 0 (0.00) | 0 (0.00) | 0 (0.00) | 0 (0.00) | 0 (0.00) | 0 (0.00) | 0 (0.00) | 0 (0.00) | 0 (0.00) | 0 (0.00) |
| 270 | *Tyzzerella* | 0 (0.00) | 0 (0.00) | 0 (0.00) | 0 (0.00) | 0 (0.00) | 0 (0.00) | 0 (0.00) | 0 (0.00) | 0 (0.00) | 0 (0.00) | 0 (0.00) | 0 (0.00) | 0 (0.00) | 0 (0.00) | 0 (0.00) | 0 (0.00) | 0 (0.00) |
| 271 | *Mageeibacillus* | 0 (0.00) | 0 (0.00) | 0 (0.00) | 0 (0.00) | 0 (0.00) | 0 (0.00) | 0 (0.00) | 0 (0.00) | 0 (0.00) | 0 (0.00) | 0 (0.00) | 0 (0.00) | 0 (0.00) | 0 (0.00) | 0 (0.00) | 0 (0.00) | 0 (0.00) |
| 272 | *Pseudobacteroides* | 0 (0.00) | 0 (0.00) | 0 (0.00) | 0 (0.00) | 0 (0.00) | 0 (0.00) | 0 (0.00) | 0 (0.00) | 0 (0.00) | 0 (0.00) | 0 (0.00) | 0 (0.00) | 0 (0.00) | 0 (0.00) | 0 (0.00) | 0 (0.00) | 0 (0.00) |
| 273 | *Pseudoclostridium* | 0 (0.00) | 0 (0.00) | 0 (0.00) | 0 (0.00) | 0 (0.00) | 0 (0.00) | 0 (0.00) | 0 (0.00) | 0 (0.00) | 0 (0.00) | 0 (0.00) | 0 (0.00) | 0 (0.00) | 0 (0.00) | 0 (0.00) | 0 (0.00) | 0 (0.00) |
| 274 | *Thermoclostridium* | 0 (0.00) | 0 (0.00) | 0 (0.00) | 0 (0.00) | 0 (0.00) | 0 (0.00) | 0 (0.00) | 0 (0.00) | 0 (0.00) | 0 (0.00) | 0 (0.00) | 0 (0.00) | 0 (0.00) | 0 (0.00) | 0 (0.00) | 0 (0.00) | 0 (0.00) |
| 275 | *Desulfitobacterium* | 0 (0.00) | 0 (0.00) | 0 (0.00) | 0 (0.00) | 0 (0.00) | 0 (0.00) | 0 (0.00) | 0 (0.00) | 0 (0.00) | 0 (0.00) | 0 (0.00) | 0 (0.00) | 0 (0.00) | 0 (0.00) | 0 (0.00) | 0 (0.00) | 0 (0.00) |
| 276 | *Desulfonispora* | 0 (0.00) | 0 (0.00) | 0 (0.00) | 0 (0.00) | 0 (0.00) | 0 (0.00) | 0 (0.00) | 0 (0.00) | 0 (0.00) | 0 (0.00) | 0 (0.00) | 0 (0.00) | 0 (0.00) | 0 (0.00) | 0 (0.00) | 0 (0.00) | 0 (0.00) |
| 277 | *Acetoanaerobium* | 0 (0.00) | 0 (0.00) | 0 (0.00) | 0 (0.00) | 0 (0.00) | 0 (0.00) | 0 (0.00) | 0 (0.00) | 0 (0.00) | 0 (0.00) | 0 (0.00) | 0 (0.00) | 0 (0.00) | 0 (0.00) | 0 (0.00) | 0 (0.00) | 0 (0.00) |
| 278 | *Paeniclostridium* | 0 (0.00) | 0 (0.00) | 0 (0.00) | 0 (0.00) | 0 (0.00) | 0 (0.00) | 0 (0.00) | 0 (0.00) | 0 (0.00) | 0 (0.00) | 0 (0.00) | 0 (0.00) | 0 (0.00) | 0 (0.00) | 0 (0.00) | 0 (0.00) | 0 (0.00) |
| 279 | *Paraclostridium* | 0 (0.00) | 0 (0.00) | 0 (0.00) | 0 (0.00) | 0 (0.00) | 0 (0.00) | 0 (0.00) | 0 (0.00) | 0 (0.00) | 0 (0.00) | 0 (0.00) | 0 (0.00) | 0 (0.00) | 0 (0.00) | 0 (0.00) | 0 (0.00) | 0 (0.00) |
| 280 | *Peptacetobacter* | 0 (0.00) | 0 (0.00) | 0 (0.00) | 0 (0.00) | 0 (0.00) | 0 (0.00) | 0 (0.00) | 0 (0.00) | 0 (0.00) | 0 (0.00) | 0 (0.00) | 0 (0.00) | 0 (0.00) | 0 (0.00) | 0 (0.00) | 0 (0.00) | 0 (0.00) |
| 281 | *Peptoclostridium* | 0 (0.00) | 0 (0.00) | 0 (0.00) | 0 (0.00) | 0 (0.00) | 0 (0.00) | 0 (0.00) | 0 (0.00) | 0 (0.00) | 0 (0.00) | 0 (0.00) | 0 (0.00) | 0 (0.00) | 0 (0.00) | 0 (0.00) | 0 (0.00) | 0 (0.00) |
| 282 | *Peptostreptococcus* | 0 (0.00) | 0 (0.00) | 0 (0.00) | 0 (0.00) | 0 (0.00) | 0 (0.00) | 0 (0.00) | 0 (0.00) | 0 (0.00) | 0 (0.00) | 0 (0.00) | 0 (0.00) | 0 (0.00) | 0 (0.00) | 0 (0.00) | 0 (0.00) | 0 (0.00) |
| 283 | *Petrocella* | 0 (0.00) | 0 (0.00) | 0 (0.00) | 0 (0.00) | 0 (0.00) | 0 (0.00) | 0 (0.00) | 0 (0.00) | 0 (0.00) | 0 (0.00) | 0 (0.00) | 0 (0.00) | 0 (0.00) | 0 (0.00) | 0 (0.00) | 0 (0.00) | 0 (0.00) |
| 284 | *Moorella* | 0 (0.00) | 0 (0.00) | 0 (0.00) | 0 (0.00) | 0 (0.00) | 0 (0.00) | 0 (0.00) | 0 (0.00) | 0 (0.00) | 0 (0.00) | 0 (0.00) | 0 (0.00) | 0 (0.00) | 0 (0.00) | 0 (0.00) | 0 (0.00) | 0 (0.00) |
| 285 | *Dielma* | 0 (0.00) | 0 (0.00) | 0 (0.00) | 0 (0.00) | 0 (0.00) | 0 (0.00) | 0 (0.00) | 0 (0.00) | 0 (0.00) | 0 (0.00) | 0 (0.00) | 0 (0.00) | 0 (0.00) | 0 (0.00) | 0 (0.00) | 0 (0.00) | 0 (0.00) |
| 286 | *Erysipelatoclostridium* | 0 (0.00) | 0 (0.00) | 0 (0.00) | 0 (0.00) | 0 (0.00) | 0 (0.00) | 0 (0.00) | 0 (0.00) | 0 (0.00) | 0 (0.00) | 0 (0.00) | 0 (0.00) | 0 (0.00) | 0 (0.00) | 0 (0.00) | 0 (0.00) | 0 (0.00) |
| 287 | *Traorella* | 0 (0.00) | 0 (0.00) | 0 (0.00) | 0 (0.00) | 0 (0.00) | 0 (0.00) | 0 (0.00) | 0 (0.00) | 0 (0.00) | 0 (0.00) | 0 (0.00) | 0 (0.00) | 0 (0.00) | 0 (0.00) | 0 (0.00) | 0 (0.00) | 0 (0.00) |
| 288 | *Propionispira* | 0 (0.00) | 0 (0.00) | 0 (0.00) | 0 (0.00) | 0 (0.00) | 0 (0.00) | 0 (0.00) | 0 (0.00) | 0 (0.00) | 0 (0.00) | 0 (0.00) | 0 (0.00) | 0 (0.00) | 0 (0.00) | 0 (0.00) | 0 (0.00) | 0 (0.00) |
| 289 | *Pelosinus* | 0 (0.00) | 0 (0.00) | 0 (0.00) | 0 (0.00) | 0 (0.00) | 0 (0.00) | 0 (0.00) | 0 (0.00) | 0 (0.00) | 0 (0.00) | 0 (0.00) | 0 (0.00) | 0 (0.00) | 0 (0.00) | 0 (0.00) | 0 (0.00) | 0 (0.00) |
| 290 | *Sedimentibacter* | 0 (0.00) | 0 (0.00) | 0 (0.00) | 0 (0.00) | 0 (0.00) | 0 (0.00) | 0 (0.00) | 0 (0.00) | 0 (0.00) | 0 (0.00) | 0 (0.00) | 0 (0.00) | 0 (0.00) | 0 (0.00) | 0 (0.00) | 0 (0.00) | 0 (0.00) |
| 291 | *Anaerococcus* | 0 (0.00) | 0 (0.00) | 0 (0.00) | 0 (0.00) | 0 (0.00) | 0 (0.00) | 0 (0.00) | 0 (0.00) | 0 (0.00) | 0 (0.00) | 0 (0.00) | 0 (0.00) | 0 (0.00) | 0 (0.00) | 0 (0.00) | 0 (0.00) | 0 (0.00) |
| 292 | *Finegoldia* | 0 (0.00) | 0 (0.00) | 0 (0.00) | 0 (0.00) | 0 (0.00) | 0 (0.00) | 0 (0.00) | 0 (0.00) | 0 (0.00) | 0 (0.00) | 0 (0.00) | 0 (0.00) | 0 (0.00) | 0 (0.00) | 0 (0.00) | 0 (0.00) | 0 (0.00) |
| 293 | *Helcococcus* | 0 (0.00) | 0 (0.00) | 0 (0.00) | 0 (0.00) | 0 (0.00) | 0 (0.00) | 0 (0.00) | 0 (0.00) | 0 (0.00) | 0 (0.00) | 0 (0.00) | 0 (0.00) | 0 (0.00) | 0 (0.00) | 0 (0.00) | 2 (0.01) | 0 (0.00) |
| 294 | *Murdochiella* | 0 (0.00) | 0 (0.00) | 0 (0.00) | 0 (0.00) | 0 (0.00) | 0 (0.00) | 0 (0.00) | 0 (0.00) | 0 (0.00) | 0 (0.00) | 4 (0.02) | 0 (0.00) | 0 (0.00) | 0 (0.00) | 0 (0.00) | 0 (0.00) | 1 (0.00) |
| 295 | *Parvimonas* | 0 (0.00) | 0 (0.00) | 0 (0.00) | 0 (0.00) | 0 (0.00) | 0 (0.00) | 0 (0.00) | 0 (0.00) | 0 (0.00) | 0 (0.00) | 0 (0.00) | 0 (0.00) | 0 (0.00) | 0 (0.00) | 0 (0.00) | 0 (0.00) | 0 (0.00) |
| 296 | *Peptoniphilus* | 0 (0.00) | 0 (0.00) | 0 (0.00) | 0 (0.00) | 0 (0.00) | 0 (0.00) | 0 (0.00) | 0 (0.00) | 0 (0.00) | 0 (0.00) | 0 (0.00) | 0 (0.00) | 0 (0.00) | 0 (0.00) | 4 (0.02) | 0 (0.00) | 1 (0.00) |
| 297 | *Fusobacterium* | 0 (0.00) | 0 (0.00) | 0 (0.00) | 0 (0.00) | 0 (0.00) | 0 (0.00) | 0 (0.00) | 0 (0.00) | 0 (0.00) | 0 (0.00) | 0 (0.00) | 0 (0.00) | 0 (0.00) | 0 (0.00) | 0 (0.00) | 0 (0.00) | 0 (0.00) |
| 298 | *Victivallis* | 0 (0.00) | 0 (0.00) | 0 (0.00) | 0 (0.00) | 0 (0.00) | 0 (0.00) | 0 (0.00) | 0 (0.00) | 0 (0.00) | 0 (0.00) | 0 (0.00) | 0 (0.00) | 0 (0.00) | 0 (0.00) | 0 (0.00) | 0 (0.00) | 0 (0.00) |
| 299 | *Oligosphaera* | 0 (0.00) | 0 (0.00) | 0 (0.00) | 0 (0.00) | 0 (0.00) | 0 (0.00) | 0 (0.00) | 0 (0.00) | 0 (0.00) | 0 (0.00) | 0 (0.00) | 0 (0.00) | 0 (0.00) | 0 (0.00) | 0 (0.00) | 0 (0.00) | 0 (0.00) |
| 300 | *Devosia* | 0 (0.00) | 0 (0.00) | 0 (0.00) | 0 (0.00) | 0 (0.00) | 0 (0.00) | 0 (0.00) | 0 (0.00) | 0 (0.00) | 0 (0.00) | 0 (0.00) | 0 (0.00) | 0 (0.00) | 0 (0.00) | 0 (0.00) | 0 (0.00) | 0 (0.00) |
| 301 | *Dongia* | 0 (0.00) | 0 (0.00) | 0 (0.00) | 0 (0.00) | 0 (0.00) | 0 (0.00) | 0 (0.00) | 0 (0.00) | 0 (0.00) | 0 (0.00) | 0 (0.00) | 0 (0.00) | 0 (0.00) | 0 (0.00) | 0 (0.00) | 0 (0.00) | 0 (0.00) |
| 302 | *Novosphingobium* | 0 (0.00) | 0 (0.00) | 0 (0.00) | 0 (0.00) | 0 (0.00) | 0 (0.00) | 0 (0.00) | 0 (0.00) | 0 (0.00) | 0 (0.00) | 0 (0.00) | 0 (0.00) | 0 (0.00) | 0 (0.00) | 0 (0.00) | 0 (0.00) | 0 (0.00) |
| 303 | *Mitsuaria* | 0 (0.00) | 0 (0.00) | 0 (0.00) | 0 (0.00) | 0 (0.00) | 0 (0.00) | 0 (0.00) | 0 (0.00) | 0 (0.00) | 0 (0.00) | 0 (0.00) | 0 (0.00) | 0 (0.00) | 0 (0.00) | 0 (0.00) | 0 (0.00) | 0 (0.00) |
| 304 | *Pelistega* | 0 (0.00) | 0 (0.00) | 0 (0.00) | 0 (0.00) | 0 (0.00) | 0 (0.00) | 0 (0.00) | 0 (0.00) | 0 (0.00) | 0 (0.00) | 0 (0.00) | 0 (0.00) | 0 (0.00) | 1 (0.01) | 0 (0.00) | 0 (0.00) | 0 (0.00) |
| 305 | *Pandoraea* | 0 (0.00) | 0 (0.00) | 0 (0.00) | 0 (0.00) | 0 (0.00) | 0 (0.00) | 0 (0.00) | 0 (0.00) | 0 (0.00) | 0 (0.00) | 0 (0.00) | 0 (0.00) | 0 (0.00) | 0 (0.00) | 0 (0.00) | 0 (0.00) | 0 (0.00) |
| 306 | *Herbaspirillum* | 0 (0.00) | 0 (0.00) | 0 (0.00) | 0 (0.00) | 0 (0.00) | 0 (0.00) | 0 (0.00) | 0 (0.00) | 0 (0.00) | 0 (0.00) | 0 (0.00) | 0 (0.00) | 0 (0.00) | 0 (0.00) | 0 (0.00) | 0 (0.00) | 0 (0.00) |
| 307 | *Noviherbaspirillum* | 0 (0.00) | 0 (0.00) | 0 (0.00) | 0 (0.00) | 0 (0.00) | 0 (0.00) | 0 (0.00) | 0 (0.00) | 0 (0.00) | 0 (0.00) | 0 (0.00) | 0 (0.00) | 0 (0.00) | 0 (0.00) | 0 (0.00) | 0 (0.00) | 0 (0.00) |
| 308 | *Oxalobacter* | 0 (0.00) | 0 (0.00) | 0 (0.00) | 0 (0.00) | 0 (0.00) | 0 (0.00) | 0 (0.00) | 0 (0.00) | 0 (0.00) | 0 (0.00) | 0 (0.00) | 0 (0.00) | 0 (0.00) | 0 (0.00) | 0 (0.00) | 0 (0.00) | 0 (0.00) |
| 309 | *Aquaspirillum* | 0 (0.00) | 0 (0.00) | 0 (0.00) | 0 (0.00) | 0 (0.00) | 0 (0.00) | 0 (0.00) | 0 (0.00) | 0 (0.00) | 0 (0.00) | 17 (0.09) | 0 (0.00) | 0 (0.00) | 0 (0.00) | 0 (0.00) | 0 (0.00) | 3 (0.02) |
| 310 | *Neisseria* | 0 (0.00) | 0 (0.00) | 0 (0.00) | 0 (0.00) | 0 (0.00) | 0 (0.00) | 0 (0.00) | 0 (0.00) | 0 (0.00) | 0 (0.00) | 0 (0.00) | 0 (0.00) | 0 (0.00) | 0 (0.00) | 0 (0.00) | 0 (0.00) | 0 (0.00) |
| 311 | *Annwoodia* | 0 (0.00) | 0 (0.00) | 0 (0.00) | 0 (0.00) | 0 (0.00) | 0 (0.00) | 0 (0.00) | 0 (0.00) | 0 (0.00) | 0 (0.00) | 0 (0.00) | 0 (0.00) | 0 (0.00) | 0 (0.00) | 0 (0.00) | 0 (0.00) | 0 (0.00) |
| 312 | *Azoarcus* | 0 (0.00) | 0 (0.00) | 0 (0.00) | 0 (0.00) | 0 (0.00) | 0 (0.00) | 0 (0.00) | 0 (0.00) | 0 (0.00) | 0 (0.00) | 0 (0.00) | 0 (0.00) | 0 (0.00) | 0 (0.00) | 3 (0.02) | 18 (0.10) | 4 (0.02) |
| 313 | *Cupidesulfovibrio* | 0 (0.00) | 0 (0.00) | 0 (0.00) | 0 (0.00) | 0 (0.00) | 0 (0.00) | 0 (0.00) | 0 (0.00) | 0 (0.00) | 0 (0.00) | 0 (0.00) | 0 (0.00) | 0 (0.00) | 0 (0.00) | 0 (0.00) | 0 (0.00) | 0 (0.00) |
| 314 | *Geobacter* | 0 (0.00) | 0 (0.00) | 0 (0.00) | 0 (0.00) | 0 (0.00) | 0 (0.00) | 0 (0.00) | 0 (0.00) | 0 (0.00) | 0 (0.00) | 0 (0.00) | 0 (0.00) | 0 (0.00) | 0 (0.00) | 0 (0.00) | 0 (0.00) | 0 (0.00) |
| 315 | *Tolumonas* | 0 (0.00) | 0 (0.00) | 0 (0.00) | 0 (0.00) | 0 (0.00) | 0 (0.00) | 0 (0.00) | 0 (0.00) | 0 (0.00) | 0 (0.00) | 0 (0.00) | 0 (0.00) | 0 (0.00) | 0 (0.00) | 0 (0.00) | 0 (0.00) | 0 (0.00) |
| 316 | *Anaerobiospirillum* | 0 (0.00) | 0 (0.00) | 0 (0.00) | 0 (0.00) | 0 (0.00) | 0 (0.00) | 0 (0.00) | 0 (0.00) | 0 (0.00) | 0 (0.00) | 0 (0.00) | 0 (0.00) | 0 (0.00) | 0 (0.00) | 0 (0.00) | 0 (0.00) | 0 (0.00) |
| 317 | *Ruminobacter* | 0 (0.00) | 0 (0.00) | 0 (0.00) | 0 (0.00) | 0 (0.00) | 0 (0.00) | 0 (0.00) | 0 (0.00) | 0 (0.00) | 0 (0.00) | 0 (0.00) | 0 (0.00) | 0 (0.00) | 0 (0.00) | 0 (0.00) | 0 (0.00) | 0 (0.00) |
| 318 | *Thiohalobacter* | 0 (0.00) | 0 (0.00) | 0 (0.00) | 0 (0.00) | 0 (0.00) | 0 (0.00) | 0 (0.00) | 0 (0.00) | 0 (0.00) | 0 (0.00) | 0 (0.00) | 0 (0.00) | 0 (0.00) | 0 (0.00) | 0 (0.00) | 0 (0.00) | 0 (0.00) |
| 319 | *Cronobacter* | 0 (0.00) | 0 (0.00) | 0 (0.00) | 0 (0.00) | 0 (0.00) | 0 (0.00) | 0 (0.00) | 0 (0.00) | 0 (0.00) | 0 (0.00) | 0 (0.00) | 0 (0.00) | 0 (0.00) | 0 (0.00) | 0 (0.00) | 0 (0.00) | 0 (0.00) |
| 320 | *Escherichia* | 0 (0.00) | 0 (0.00) | 0 (0.00) | 0 (0.00) | 0 (0.00) | 0 (0.00) | 0 (0.00) | 0 (0.00) | 0 (0.00) | 0 (0.00) | 0 (0.00) | 0 (0.00) | 0 (0.00) | 0 (0.00) | 0 (0.00) | 0 (0.00) | 0 (0.00) |
| 321 | *Kluyvera* | 0 (0.00) | 0 (0.00) | 0 (0.00) | 0 (0.00) | 0 (0.00) | 0 (0.00) | 0 (0.00) | 0 (0.00) | 0 (0.00) | 0 (0.00) | 0 (0.00) | 0 (0.00) | 0 (0.00) | 0 (0.00) | 0 (0.00) | 0 (0.00) | 0 (0.00) |
| 322 | *Raoultella* | 0 (0.00) | 0 (0.00) | 0 (0.00) | 0 (0.00) | 0 (0.00) | 0 (0.00) | 0 (0.00) | 0 (0.00) | 0 (0.00) | 0 (0.00) | 0 (0.00) | 0 (0.00) | 0 (0.00) | 0 (0.00) | 0 (0.00) | 0 (0.00) | 0 (0.00) |
| 323 | *Shigella* | 0 (0.00) | 0 (0.00) | 0 (0.00) | 0 (0.00) | 0 (0.00) | 0 (0.00) | 0 (0.00) | 0 (0.00) | 0 (0.00) | 0 (0.00) | 0 (0.00) | 0 (0.00) | 0 (0.00) | 0 (0.00) | 0 (0.00) | 0 (0.00) | 0 (0.00) |
| 324 | *Aggregatibacter* | 0 (0.00) | 0 (0.00) | 0 (0.00) | 0 (0.00) | 0 (0.00) | 0 (0.00) | 0 (0.00) | 0 (0.00) | 0 (0.00) | 0 (0.00) | 0 (0.00) | 0 (0.00) | 0 (0.00) | 0 (0.00) | 0 (0.00) | 0 (0.00) | 0 (0.00) |
| 325 | *Basfia* | 0 (0.00) | 0 (0.00) | 0 (0.00) | 0 (0.00) | 0 (0.00) | 0 (0.00) | 0 (0.00) | 0 (0.00) | 0 (0.00) | 0 (0.00) | 0 (0.00) | 0 (0.00) | 0 (0.00) | 0 (0.00) | 0 (0.00) | 0 (0.00) | 0 (0.00) |
| 326 | *Glaesserella* | 0 (0.00) | 0 (0.00) | 0 (0.00) | 0 (0.00) | 0 (0.00) | 0 (0.00) | 0 (0.00) | 0 (0.00) | 0 (0.00) | 0 (0.00) | 0 (0.00) | 0 (0.00) | 0 (0.00) | 0 (0.00) | 0 (0.00) | 0 (0.00) | 0 (0.00) |
| 327 | *Haemophilus* | 0 (0.00) | 0 (0.00) | 0 (0.00) | 0 (0.00) | 0 (0.00) | 0 (0.00) | 0 (0.00) | 0 (0.00) | 0 (0.00) | 0 (0.00) | 0 (0.00) | 0 (0.00) | 0 (0.00) | 0 (0.00) | 0 (0.00) | 0 (0.00) | 0 (0.00) |
| 328 | *Pyramidobacter* | 0 (0.00) | 0 (0.00) | 0 (0.00) | 0 (0.00) | 0 (0.00) | 0 (0.00) | 0 (0.00) | 0 (0.00) | 0 (0.00) | 0 (0.00) | 0 (0.00) | 0 (0.00) | 0 (0.00) | 0 (0.00) | 0 (0.00) | 0 (0.00) | 0 (0.00) |
| 329 | *Rarimicrobium* | 0 (0.00) | 0 (0.00) | 0 (0.00) | 0 (0.00) | 0 (0.00) | 0 (0.00) | 0 (0.00) | 0 (0.00) | 0 (0.00) | 0 (0.00) | 0 (0.00) | 0 (0.00) | 0 (0.00) | 0 (0.00) | 0 (0.00) | 0 (0.00) | 0 (0.00) |
| 330 | *Synergistes* | 0 (0.00) | 0 (0.00) | 0 (0.00) | 0 (0.00) | 0 (0.00) | 0 (0.00) | 0 (0.00) | 0 (0.00) | 0 (0.00) | 0 (0.00) | 4 (0.02) | 35 (0.19) | 4 (0.02) | 0 (0.00) | 16 (0.09) | 6 (0.03) | 11 (0.06) |
| 331 | *Acholeplasma* | 0 (0.00) | 0 (0.00) | 0 (0.00) | 0 (0.00) | 0 (0.00) | 0 (0.00) | 0 (0.00) | 0 (0.00) | 0 (0.00) | 0 (0.00) | 2 (0.01) | 0 (0.00) | 0 (0.00) | 0 (0.00) | 0 (0.00) | 0 (0.00) | 0 (0.00) |
| 332 | *Haloplasma* | 0 (0.00) | 0 (0.00) | 0 (0.00) | 0 (0.00) | 0 (0.00) | 0 (0.00) | 0 (0.00) | 0 (0.00) | 0 (0.00) | 0 (0.00) | 0 (0.00) | 0 (0.00) | 0 (0.00) | 0 (0.00) | 0 (0.00) | 0 (0.00) | 0 (0.00) |
| 333 | *Coraliomargarita* | 0 (0.00) | 0 (0.00) | 0 (0.00) | 0 (0.00) | 0 (0.00) | 0 (0.00) | 0 (0.00) | 0 (0.00) | 0 (0.00) | 0 (0.00) | 0 (0.00) | 0 (0.00) | 0 (0.00) | 0 (0.00) | 0 (0.00) | 0 (0.00) | 0 (0.00) |
| 334 | *Ruficoccus* | 0 (0.00) | 0 (0.00) | 0 (0.00) | 0 (0.00) | 0 (0.00) | 0 (0.00) | 0 (0.00) | 0 (0.00) | 0 (0.00) | 0 (0.00) | 0 (0.00) | 0 (0.00) | 0 (0.00) | 0 (0.00) | 0 (0.00) | 0 (0.00) | 0 (0.00) |
| 335 | *Chthoniobacter* | 0 (0.00) | 0 (0.00) | 0 (0.00) | 0 (0.00) | 0 (0.00) | 0 (0.00) | 0 (0.00) | 0 (0.00) | 0 (0.00) | 0 (0.00) | 0 (0.00) | 0 (0.00) | 0 (0.00) | 0 (0.00) | 0 (0.00) | 0 (0.00) | 0 (0.00) |
| 336 | *Akkermansia* | 0 (0.00) | 0 (0.00) | 0 (0.00) | 0 (0.00) | 0 (0.00) | 0 (0.00) | 0 (0.00) | 0 (0.00) | 0 (0.00) | 0 (0.00) | 0 (0.00) | 0 (0.00) | 0 (0.00) | 0 (0.00) | 0 (0.00) | 0 (0.00) | 0 (0.00) |
